# Traits and ecological space availability predict avian densities at the country scale of the Czech Republic

**DOI:** 10.1002/ece3.9119

**Published:** 2022-07-17

**Authors:** David Hořák, Javier Rivas‐Salvador, Jan Farkač, Jiří Reif

**Affiliations:** ^1^ Department of Ecology, Faculty of Science Charles University Prague Czech Republic; ^2^ Institute of Environmental Sciences, Faculty of Science Charles University Prague Czech Republic; ^3^ Department of Zoology, Faculty of Science Palacký University Olomouc Czech Republic; ^4^ Czech Society for Ornithology Prague Czech Republic

**Keywords:** abundance, birds, ecological space, ecological traits, specialization

## Abstract

Species' geographical distributions and abundances are a central focus of current ecological research. Although multiple studies have been conducted on their elucidation, some important information is still missing. One of them is the knowledge of ecological traits of species responsible for the population density variations across geographical (i.e., total physical area) and ecological spaces (i.e., suitable habitat area). This is crucial for understanding how ecological specialization shapes the geographical distribution of species, and provides key knowledge about the sensitivity of species to current environmental challenges. Here, we precisely describe habitat availability for individual species using fine‐scale field data collected across the entire Czech Republic. In the next step, we used this information to test the relationships between bird traits and country‐scale estimates of population densities assessed in both geographical and ecological spaces. We did not find any effect of habitat specialization on avian density in geographical space. But when we recalculated densities for ecological space available, we found a positive correlation with habitat specialization. Specialists occur at higher densities in suitable habitats. Moreover, birds with arboreal and hole‐nesting strategies showed higher densities in both geographical and ecological spaces. However, we found no significant effects of morphological (body mass and structural body size) and reproductive (position along the slow–fast life‐history continuum) traits on avian densities in either geographical or ecological space. Our findings suggest that ecological space availability is a strong determinant of avian abundance and highlight the importance of precise knowledge of species‐specific habitat requirements. Revival of this classical but challenging ecological topic of habitat‐specific densities is needed for both proper understanding of pure ecological issues and practical steps in the conservation of nature.

## Graphical abstract

We test the relationships between traits of birds and country scale estimates of population densities assessed in both geographical and ecological space. We did not find any effect of the habitat specialization on avian densities in geographical space, but the effect turned positive once we recalculated the densities for ecological space available. Moreover, birds with arboreal and hole nesting strategies showed higher densities in both geographical and ecological space. However, we found no significant effects of the morphological and reproductive traits on avian densities.

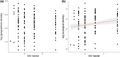

## 1 INTRODUCTION

The geographical distribution of species and individuals is a central focus area of ecology as it directly influences species richness patterns (MacArthur, 1984). Not surprisingly, it has attracted a lot of attention to date (Mertes et al., 2020; Rahbek et al., 2019; Stephens et al., 2019). Yet, there are still significant gaps in our understanding, such as fine‐scale information about spatial distributions of species or proper knowledge of factors determining local densities and consequently population sizes. However, these are crucial for the ultimate resolution of mechanisms behind spatial patterns of biodiversity because limits on the number of individuals in communities, and within populations of individual species, are core parameters in biodiversity theories (e.g., the more individuals’ hypothesis, Storch et al., 2018).

Local population densities are affected by intrinsic and extrinsic factors. Firstly, intrinsic factors include the qualities and requirements of individual species, such as body mass, territory size, and life span. Body mass is a very informative trait, as it is strongly correlated with many other morphological, ecological, and behavioral traits (Sæther & Saether, 1987) and is therefore frequently tested. In summary, higher vertebrate population densities are usually found in smaller species (Blackburn & Gaston, 1996; Greenwood et al., 1996), species with smaller propagules (Blackburn et al., 1996; Böhning‐Gaese & Oberrath, 2001), species at lower positions on the trophic chain (Arita, 1993; Carbone & Gittleman, 2002; Peters & Wassenberg, 1983), species with higher dispersal ability (Gaston & Kunin, 1997), generalist species (Brown, 1984), and species specialized in exploiting an abundant resource (Gregory & Gaston, 2000). Extrinsic factors include the availability of geographical and ecological spaces, such as environmental productivity (Carbone & Gittleman, 2002; Coe et al., 1976; Pettorelli et al., 2009). The relationship between geographical and ecological space is at the heart of niche theory (Grinnell, 1917) and fundamental ecological processes such as mapping geographical distributions of species and generating species diversity patterns.

From a broad perspective, the role of geographical versus ecological space in shaping the community structure is clearly visible under island conditions, where physical space evidently restricts ecological space. A low immigration rate and high extinction probability result in a lower number of species on an island compared to similar areas on the mainland, which allows the present populations to reach high numbers, which is also known as the density compensation hypothesis (Andrews, 1979; Blondel, 2000; Buckley & Jetz, 2007; Djomo Nana et al., 2014; MacArthur et al., 1972; Reif et al., 2006; Rodda & Dean‐Bradley, 2002). However, more effort is needed to properly understand the link between geographical and ecological space on the mainland due to the interaction with various confounding factors.

It has been shown in the early stages of ecological research that population densities are highest at a habitat optimum (Brown, 1984; Whittaker, 1960, 1965) with potentially high levels of suitable resources. Moreover, the population density of a focal species is also affected by other members of the community, depending on the amount of space and resources required by an individual (Buckley & Jetz, 2007). Therefore, the spatial distribution of a species is strongly determined by the distribution of adequate and free ecological space, which suggests a strong connection between geographical and ecological space. Evidently, not all geographical locations are suitable for the survival and reproduction of a species (Lack, 1933). Thus, correctly estimating species density within its ecological limitations (i.e., ecological density) reveals different information compared to the number of individuals per unit of geographical area (i.e., geographical density). Although the concept of “ecological density” (Gaston et al., 2001) has been known since the early 20th century when reported by Elton as “economic density” (Elton, 1932, 1933), it remains seriously understudied until now. At the same time, ecological density is a very important measure, as it truly informs how a species performs within *its habitat*.

The concept of ecological density is crucial for estimating ecological specializations, which are frequently discussed in the context of nature conservation (Barnagaud et al., 2011; Devictor et al., 2010; Rivas‐Salvador et al., 2019) and responses of species to undergoing environmental and climate changes (Jiguet et al., 2007; Julliard et al., 2004). Specialized species with narrow habitat niches have been revealed as endangered because of significant population declines (Clavel et al., 2010; Gregory et al., 2004; Heldbjerg et al., 2017), and they show sensitive responses to environmental changes (Keinath et al., 2017; Manne & Pimm, 2001; Matthews et al., 2014; Purvis et al., 2000). However, the sensitivity and abundance of specialists strongly depend on their traits as well as on the availability and quality of the ecological space to which they are adapted.

In this study, we focused on birds because they are ideal models for testing the above‐mentioned ecological questions, as reliable data on their densities and population sizes are available. Moreover, detailed and complete bird trait datasets already exist (Storchová & Hořák, 2018), and due to a long history of avian research, we understand the main ecological principles behind avian trait variation (Tobias et al., 2020). We estimated avian densities at a local scale of a few hundred meters, which properly reflects the relationship between birds and their habitats. We then analyzed the data at the country scale, which brought geographical distribution into play. This approach allowed us to test the importance of both intrinsic and extrinsic factors on observed avian densities. We postulated that traits related to dispersal, reproductive rates, and niche breadth would influence bird population densities. Moreover, we assumed that these effects would be different for densities estimated in the geographical space and within suitable habitats (ecological space), as specialists can afford higher population densities within habitats. Specifically, we investigated (i) how different sets of ecological traits of birds are related to their densities, (ii) whether the level of ecological specialization has significant effects on avian densities, and (iii) whether the amount of species‐specific ecological space reflects the species densities at the country scale.

## 2 MATERIALS AND METHODS

### 2.1 Bird and habitat data

We used data on avian densities from the Common Bird Monitoring Program in the Czech Republic (JPSP—abbreviation in Czech). The community structures were estimated at the census points (radius 100 m) located along transects, each containing 20 points. Neighboring points were separated by 300–500 m. The birds were censused for 5 min at each point, and each point was visited twice during the breeding season (April–June) to register the maximum abundance of early and late breeding birds. We used JPSP data collected in 2009, which offered the best spatial coverage: information for 2580 census points along 129 transects was available. The JPSP has been a common bird monitoring scheme performed annually since 1982, but the locations and number of census transects were not stable between years. Our primary objective was to include all the spatial heterogeneity within the Czech Republic by covering the largest area possible, and the JPSP transects are widely distributed providing a representative sample of bird populations across the whole country. Thus, we used the population estimates for 2009, which seem to best represent the geographical and ecological space of the Czech Republic given the available data. In addition, we compared the density estimates from JPSP 2009 with estimates based on the breeding bird mapping atlas covering the period shortly preceding the focal year (Šťastný et al., 2006), and found them to be correlated (Pearson correlation 0.7). Thus, we believe that these values are adequately reflected in our data. Unfortunately, the data we used did not allow for a reasonable estimate of the detection probability because the critical assumption of repeated sampling of demographically closed populations (Royle, 2004a; Royle, 2004b) was not the case in our data. In JPSP, citizen scientists visit the points twice per breeding season, which theoretically meets the minimum of repeated sampling required. However, this is not met in reality; repeated visits are performed because bird populations change over the entire breeding season. The bird communities differ at the beginning and end of the breeding season, not only because of the presence/absence of migrants that arrive after the first visit is completed but also because of changes in the abundance of non‐migrating species. Thus, combining both controls is the best approach for estimating the maximum local population. In addition, the program does not include distance sampling (Buckland et al., 2001; Norvell et al., 2003)—an alternative approach to account for the unequal detectability of the species. Effort, time of day, and weather were standardized in the methodology—all the points were censused under the same conditions. In addition, the effectiveness of adjusting bird count data has been disputed. For instance, Johnson (2008) stated that no method for adjusting bird count data appears to be effective for large‐scale, multi‐species monitoring surveys.

The JPSP data also contain information about the surrounding biotopes, within a 100 m radius from each census point. Biotopes were classified into 12 categories: coniferous forest, deciduous forest, mixed forest, shrubs, meadows, fields, alpine, rocks, water bodies, marshes, urban, and suburban. For this analysis, we aggregated the 12 habitat categories into seven: deciduous forest, coniferous forest, mixed forest, shrub, open habitats (i.e., meadows, fields, alpine, and rocks), water habitats (water bodies and marshes), and urban habitats (suburban and urban categories).

### 2.2 Response variables

We divided the abundance information by the geographical area covered by the surveys to calculate the “geographical density” of the species (number of individuals per unit of physical space [ha]). In addition, we also calculated the “ecological density,” which reflects the habitat preferences of birds and is estimated as the number of individuals per unit of habitat suitable for a given species. Based on the species‐specific habitat preferences, we calculated the area of suitable habitat available for each species at each point, in which it was present. We estimated the habitat preferences of species at the European scale. The information was extracted from Storchová and Hořák (2018), and to adjust their habitat classification to our study, we merged some classes as follows: rock, savanna, tundra, grassland, mountain, and deserts were considered as open biotopes; reeds, swamps, freshwater, and marshes were considered as water biotopes; and suburban and urban environments were considered as urban biotopes. The remaining habitat categories remained unchanged.

### 2.3 Predictors

Bird densities are influenced mostly by dispersal ability, reproductive rates, and niche width (Böhning‐Gaese & Oberrath, 2001; Brown, 1984, 1995; Gaston & Kunin, 1997). Therefore, we focused on three types of traits: (i) morphological traits that reflect the use of space by birds, (ii) reproductive traits that reflect the life histories of birds, and (iii) diet and habitat specializations, in which the two crucial axes of avian niches are mirrored. Due to the complex effects of morphological traits on avian use of space, we characterized the size and structure of birds using all available morphological traits, similar to the approach for reproductive traits. To reduce the number of variables in our models and facilitate their interpretation, we reduced both morphological and reproductive traits from Storchová and Hořák (2018) by running two separate principal component analyses (PCAs). We decided on PCA as it combines variation in traits which seems to be the best option in our case because (i) the dataset is large, and we benefited from the reduced number of variables, and (ii) we wanted to characterize the overall species quality using available traits. The first PC axis included all morphological traits including body length, wing length, tail length, bill length, and *tarsometatarsus* length. For all morphological measurements, we averaged trait values across females and males. The first PC axis described 91.97% of the variance and could be interpreted as the structural size principal component (ssPC, Figure S1a). The second PC axis included all reproductive traits: clutch size, number of broods per year, egg mass, and life span. The first PC axis describes 98.01% of the variance and can be understood as a slow–fast continuum principal component (sfPC, Figure S1b). Body mass is the trait most frequently tested in the context of abundance, and it is a special trait that contains a lot of ecological information (Brown, 1995). Thus, it is correlated with many other traits, including reproduction and morphology (Sæther & Saether, 1987). Therefore, we considered body mass (calculated as the mean of sex‐specific values) as a separate predictor.

Furthermore, we calculated the species specialization index (SSI; SSI = (*H*/*h* − 1)^1/2^ for species present in *h* habitat classes among *H* possible habitat classes, Julliard et al., 2006) separately for habitat and diet (SSI habitat and SSI diet) following Reif et al. (2016). We determined breeding habitats to estimate habitat specialization of species. We determined whether each habitat was occupied by a given species, and classified as “presence” (quantified as 1) when a species occupied a given habitat and as “absence” (quantified as 0) when a habitat was not occupied by that species. Thus, for each species, we obtained a vector of presences and absences across the habitats. From the presence–absence data, we calculated the species habitat specialization index as a coefficient of variation of a given species occurrence across habitats (Julliard et al., 2006). Specialists thus have high values for this specialization index, while generalists have low values. Diet specialization was expressed in a manner similar to habitat specialization. Habitat and dietary preferences were again obtained from Storchová and Hořák (2018). We distinguished between 15 habitat categories (coniferous forest, deciduous forest, woodland, shrub, savanna, tundra, grassland, reed, mountain meadows, swamp, desert, freshwater, marine, rocks, and human settlement) and 9 diet categories (folivore, frugivore, granivore, insectivore, other invertebrates, piscivores, other vertebrates, scavengers, and omnivores). Please note that using the habitat categories from Storchová and Hořák (2018) for SSI habitat calculation was necessary to ensure the independence of this measure from the measure of habitat availability based on the field JPSP data.

Finally, we used forest dependency, area of breeding range, and nest type as additional possible predictors of avian density. Forest dependency and breeding range were obtained from the BirdLife International database (BirdLife International, 2014). Forest dependency distinguishes between not dependent, low‐dependent, medium‐dependent, and forest‐dependent species. The nest type was obtained from Storchová and Hořák (2018) and classified according to their placement and structure (i.e., ground—used as a reference factor level in the analyses, hole, open arboreal, closed arboreal, closed ground). However, owing to the lack of representation of species in closed arboreal nests, we merged this class with open arboreal nests.

### 2.4 Phylogenetic data

We extracted 1000 phylogenetic trees from BirdTree.org (Jetz et al., 2012) to include information on phylogenetic correlations among the study species. This is the largest and most complete source of bird phylogenetic data, and the consensus tree was built using the R‐package “phytools” (Revell, 2012). The consensus tree was used to build a Brownian correlation matrix using the R‐package “ape” (Paradis & Schliep, 2019) for posterior inclusion in our models.

### 2.5 Statistical analysis

Statistical analyses were performed using the R software version 4.1.1 (R Core Team, 2021). We used phylogenetic generalized least squares models (pgls, R‐package “nlme”; Pinheiro et al., 2020) to include the Brownian correlation structure of our models. In our first round of analyses, we tested the influence of body mass, SSI habitat, and SSI diet in separate (i.e., single predictor) pgls models on both response variables (i.e., geographical and ecological densities).

Second, to offer a full insight into how ecological traits affect avian densities, we ran two more sets of pgls models for geographical and ecological densities. For both separate sets of models, we included all available traits except for the body mass because of its correlation with both principal components (ssPC: *r* = −0.564, sfPC: *r* = −0.592). Moreover, we analyzed the PCs (i.e., ssPC and sfPC) separately because of their high correlation (*r* = 0.85). In each set of models, we first constructed a full model containing all the predictors mentioned above, and then we used a model‐dredging approach (R‐package MuMIn, Barton, 2020) to assess models containing all possible combinations of predictors. Based on this assessment using the Akaike information criterion (AIC), only models with ΔAIC < 2 were used for inference (Burnham & Anderson, 1998). Additionally, to ensure that the consensus tree did not bring additional uncertainty into our analyses, we ran the model dredging for each of the full models with a random sample of 1000 phylogenetic trees (results not shown). As these results were consistent with those obtained based on the consensus tree, we only present the consensus tree results in the manuscript. The consistency of the results was most likely due to the fact that the quality of phylogenetic trees for European birds is quite high in the BirdTree database, thus there was no distinction among particular types of trees.

## 3 RESULTS

The JPSP data for 2009 contained information on the abundance of 153 species, which account for approximately 75% of breeding birds in the Czech Republic. The calculated densities ranged between 0.0001 and 0.658 individuals per hectare for geographical density and between 0.0003 and 3.182 individuals/ha for ecological density.

### 3.1 Body mass–density relationships

The pgls models relating the body mass of the species to both geographical and ecological densities did not show any significant effects (Table 1).

**TABLE 1:** Result for the pgls models relating geographical density (left column) and ecological density (right column) with the body mass, species habitat (SSI habitat), and dietary (SSI diet) specialization. Statistically significant results are provided in bold

	Geographical density				Ecological density			
	Estimate	*SE*	*t*‐value	Pr(>|*t*|)	Estimate	*SE*	*t*‐value	Pr(>|*t*|)
Intercept	0.045	0.108	0.413	0.680	0.345	0.646	0.535	0.594
Log(body mass)	0.009	0.009	−1.038	0.301	−0.036	0.054	−0.672	0.503
Intercept	−0.019	0.099	−0.188	0.852	−0.458	0.572	−0.800	0.425
SSI habitat	0.004	0.011	0.110	0.724	0.215	0.060	3.561	**<0.001**
Intercept	0.023	0.098	0.236	0.814	0.326	0.584	0.558	0.578
SSI diet	−0.016	0.012	−1.317	0.190	−0.092	0.071	−1.301	0.195

### 3.2 SSI–Density relationships

**FIGURE 1:**
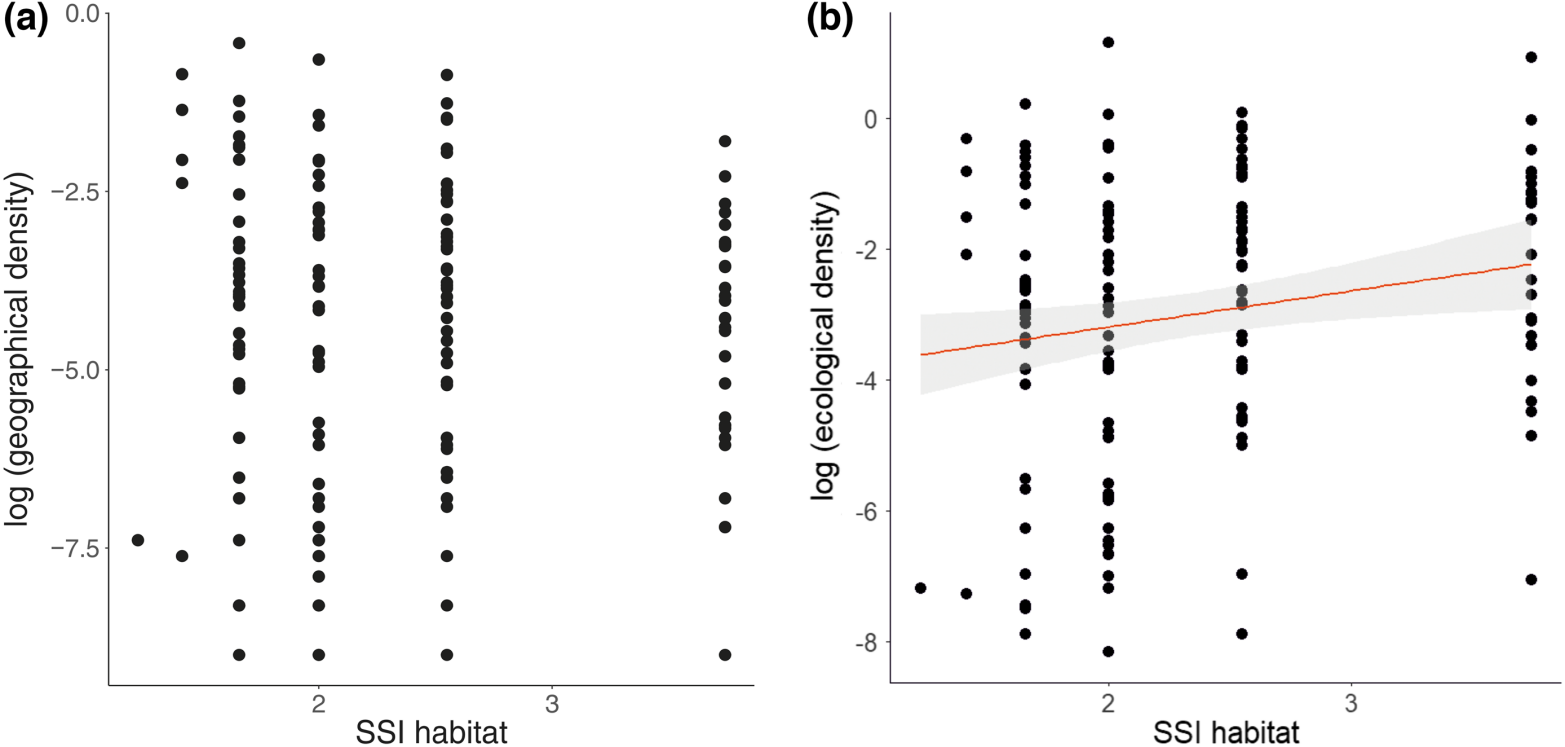
Relationship between the log‐transformed geographical densities (a) and ecological densities (b), and the habitat species specialization index (SSI habitat), the red line represents the significant linear relationship among the variables with confidence intervals (*p* < .001). Please note that raw data were used in these plots

The SSI habitat was, on average, 2.382 ± 0.748 (*SD*), median = 2, and range = 1.225–3.742. The SSI diet was on average 1.935 ± 0.714 (*SD*), median = 1.871, and range = 0–2.828. The pgls models relating the geographical density to the specialization indices did not show any significant effect for either the SSI habitat or the SSI diet (Table 2a; Figure 1a). In the case of the model relating ecological density to the specialization indices, we detected a significant positive effect (Table 2b; Figure 1b) for the SSI habitat, but not for the SSI diet. These results indicate that habitat specialists are more abundant in their preferred habitats than generalists (Tables 1, 2b). We provided estimates of the ecological densities for all species under study (Table S1), which might help in making future conservation decisions.

**TABLE 2:** Detailed view of the averaged model resulted from the model dredging for both geographical (a) and ecological densities (b). The upper panel showed the average model using the ssPC as a predictor and the lower one uses the sfPC. Statistically significant results are provided in bold

	Estimate	*SE*	Adjusted *SE*	*z*‐value	Pr(>|*z*|)
(a)					
(Intercept)	0.008	0.091	0.091	0.092	0.927
Type of Nest (GC)	0.006	0.030	0.030	0.199	0.842
Type of Nest (H)	0.067	0.025	0.025	2.695	**0.007**
Type of Nest (OA)	0.058	0.028	0.028	2.089	**0.037**
ssPC	1.383E‐05	4.390E‐05	4.412E‐05	0.313	0.754
SSI diet	−0.001	0.006	0.006	0.257	0.797
Breeding range	1.079E‐11	1.087E‐10	1.096E‐10	0.098	0.922
SSI habitat	−0.0002	0.004	0.004	0.050	0.960
(Intercept)	0.013	0.091	0.092	0.146	0.884
Type of Nest (GC)	0.004	0.029	0.029	0.146	0.884
Type of Nest (H)	0.058	0.032	0.032	1.772	0.076
Type of Nest (OA)	0.049	0.033	0.033	1.505	0.132
sfPC	9.098E‐05	1.737E‐04	1.742E‐04	0.522	0.602
SSI diet	−0.001	0.005	0.005	0.235	0.815
Breeding range	9.114E‐12	9.999E‐11	1.008E‐10	0.090	0.928
SSI habitat	−0.0002	0.003	0.003	0.046	0.964
(b)					
(Intercept)	−0.502	0.569	0.574	0.875	0.382
Type of Nest (GC)	0.139	0.185	0.186	0.745	0.456
Type of Nest (H)	0.470	0.154	0.155	3.025	**0.002**
Type of Nest (OA)	0.266	0.171	0.173	1.538	0.124
SSI habitat	0.177	0.062	0.062	2.848	**0.004**
ssPC	−8.9300E‐05	2.7720E‐04	2.7870E‐04	0.320	0.749
Breeding range	2.3150E‐10	9.0320E‐10	9.0860E‐10	0.255	0.799
SSI diet	−0.006	0.032	0.032	0.193	0.847
(Intercept)	−0.494	0.568	0.573	0.862	0.389
Type of Nest (GC)	0.133	0.183	0.185	0.718	0.473
Type of Nest (H)	0.465	0.154	0.155	3.006	**0.003**
Type of Nest (OA)	0.261	0.171	0.173	1.511	0.131
SSI habitat	0.176	0.061	0.062	2.839	**0.005**
Breeding range	2.401E‐10	9.186E‐10	9.242E‐10	0.260	0.795
sfPC	−0.0001	0.001	0.001	0.239	0.811
SSI diet	−0.006	0.033	0.033	0.196	0.844

### 3.3 Effect of ecological traits on densities

**FIGURE 2:**
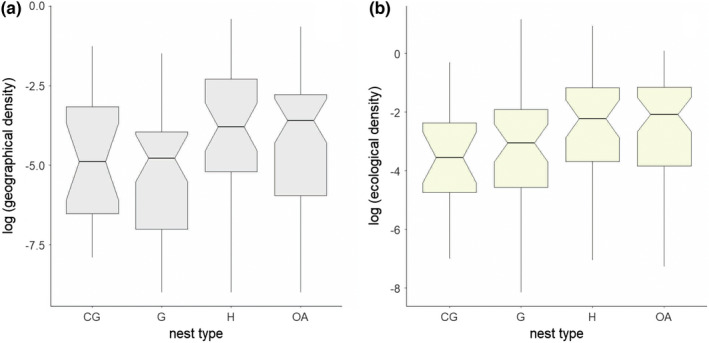
The effect of nest type on log‐transformed geographical densities (a) and ecological densities (b). For statistical results, see Table 2. The figure shows differences among closed ground (GC), ground (G), hole (H), and open arboreal nests (OA). Boxes show median, the notches give approximately 95% confidence interval for comparing medians (extend 1.58 × the inter‐quartile range/sqrt[*n*]). Please note that raw data were used in these plots

The variables selected in both subsets of the best models (ΔAIC < 2) for geographical density were consistent. Four of the five best models that included ssPC as a predictor also included nest type, and the effect increased with nesting height (Table S2a; Figure 2). The sfPC was present in only two of five models, whereas the ssPC was considered only in one of the models, similar to the SSI habitat, SSI diet, and breeding range (Table S2a).

In the case of ecological density, both sets of models, using ssPC or sfPC as predictors, had a subset of four models with ΔAIC < 2, all of which considered the type of nest and SSI habitat as predictors of species density (Table S2b). Thus, habitat specialists and those breeding higher above the ground level have higher ecological densities (Figures 1b and 2b). Furthermore, ssPC and sfPC were considered in one of four models (Table S2b), with a negative effect on ecological density. Similarly, the breeding range and SSI diet were considered in one of the four models with positive and negative effects, respectively (Table S2b). Results from the analyses using a random subset were consistent with those obtained using the consensus tree (see Tables [Link], [Link], [Link], [Link]).

## 4 DISCUSSION

We tested the relationships between estimates of avian population densities and morphological, reproductive, and other ecological traits at the country scale in the Czech Republic. In the analysis, we distinguished between population densities estimated in geographical (number of individuals per unit area) and ecological spaces (number of individuals per unit area of suitable habitat). Such a distinction has proven to be important for the strength of the observed relationships in our analyses. Specifically, we did not find any significant relationships between habitat specialization and population density estimated in geographical space, indicating no difference in densities between habitat specialists and generalists. However, we found strong evidence for a positive effect of habitat specialization on the population density of ecological space, indicating that densities of birds are determined mostly by ecological space availability and that abundances of habitat specialists and generalists differ once their habitat requirements are considered. Furthermore, we observed an effect of nest type on avian density, where geographical and ecological densities were higher in arboreal and hole‐nesting species.

Habitat specialists and generalists have similar densities in geographical space. This finding partly contrasts with the abundance‐range size relationship (Brown, 1984), which suggests that generalists are widely distributed and common locally. This relationship has several explanations (Borregaard & Rahbek, 2010) as well as exceptions (Ferenc et al., 2016; Reif et al., 2006); however, it is frequently mechanistically attributed to the larger physical area occupied by generalists due to preferences for more biotope types. Therefore, given the equal distribution of biotopes, generalists should have higher population densities per unit area than that of the specialists. However, a wider geographical distribution across more biotope types is not necessarily accompanied by higher local densities (Kouki & Hayrinen, 1991), and our results support this hypothesis. In addition, after controlling for suitable habitats, we found that specialists had even higher densities than generalists. This might suggest better adaptations of specialists to their habitats (Pulliam, 1985; Reif et al., 2016) or selection for higher densities, given that specialists have a spatial restriction of preferred environments (density compensation hypothesis; MacArthur et al., 1972; Ferenc et al., 2016). Geographical distributions of species‐specific habitats thus likely explain much of the variation in abundance observed in physical space at the country scale (cf. Ricklefs, 2013). Specialists and generalists are frequently contrasted if changes in contemporary biotope (Hahn et al., 2011; Hanzelka & Reif, 2015) or population declines are tested (Clavel et al., 2010; Gregory et al., 2007). Specialists are generally reported to be more sensitive to environmental changes. This might be caused by their restricted geographical distribution, as relatively high proportions of their habitats are potentially negatively affected.

We did not find any strong evidence for the effect of diet specialization on variation in avian density. In theory, local specialization enables the species to utilize resources more effectively (Pulliam, 1985), which might make diet specialists more successful, even in the habitats they prefer. However, in the context of previous studies on birds, our findings are not surprising. Dietary specialization has been found to either correlate negatively with estimates of abundance (De Almeida‐Rocha et al., 2019; Herrera, 1978) or not at all (Brändle et al., 2002; Symonds & Johnson, 2006). Combining our results regarding habitat and diet specializations, we suggest that bird densities are mostly determined by the area of available habitats. Habitat selection is a strong determinant of avian geographical distributions (Grinnell, 1917; Ricklefs, 2013; Tellería et al., 2009), and there is presumably not enough potential for diet specializations to affect avian population densities. In addition, both avian diet and abundance can vary and respond to the current food supply (Korpimäki, 1992; Marone et al., 2017). Such flexibility could confound the influence of diet specialization on population density.

There is a huge body of evidence regarding the negative relationship between body mass and population density (Blackburn & Lawton, 1994; Cotgreave, 1993, 1995), although it is sometimes reported to be non‐significant (Blackburn & Lawton, 1994; Cotgreave, 1993). However, body mass seems to be a moderate predictor of population density (Gregory & Blackburn, 1995). The weak relationship between body mass and avian population density is not well understood (Hatton et al., 2019). This may be because the strength of the body mass–density relationship depends on the body mass variance in the dataset. Large birds have lower densities than small birds because each individual needs a larger home range to fulfill its energy requirements (Jetz et al., 2004). However, with restricted body mass variation, such as within taxonomical subsets or groups of similar members (such as Passerine families), we might predict relatively weak body mass–population density relationships (Symonds & Johnson, 2006) because small inter‐specific differences are overridden by species idiosyncrasies in ecological requirements. This may be the case for our dataset because the data came from the bird monitoring program designed to survey small territorial species, and being not particularly suitable for larger species (Voříšek et al., 2008).

We analyzed morphological and reproductive traits separately from body mass and did not find any significant effect of morphology on population densities in either ecological or geographical space. Similarly, we found no significant effects of reproductive traits on either estimate of population density. Interestingly, the effects of other ecological traits on avian population densities have not been tested explicitly (but see Blackburn et al., 1996; Symonds & Johnson, 2006). In contrast to our results, available evidence suggests that traits associated with offspring production are related to avian abundance. In addition, there is much more information on the relationship between geographic distribution and traits (Cofre et al., 2007; Laube et al., 2013). These studies observed that rarity can be linked to low reproductive investment, sedentary migratory mode, low dispersal ability, and habitat specialization (Cofre et al., 2007; Laube et al., 2013). Although the link between abundance and geographical distribution is not straightforward, it is likely that reproductive effort, dispersal ability, and habitat specialization strongly determine both. Our inability to detect a significant relationship between morphological and reproductive traits can be attributed to a relatively phylogenetically restricted dataset of birds. In addition, our dataset covers a small country with limited ecological variation owing to the absence of pronounced environmental gradients. Such conditions limit the variation in avian ecological strategies, which might hamper trend detection. More information on population densities in a wider spectrum of birds is needed to provide a comprehensive picture.

Surprisingly, we found that nest type had a significant effect on avian density. In both geographical and ecological spaces, birds breeding in holes and trees had higher densities. We are not aware of any previous study explicitly linking avian densities and nest type, and thus, we can only speculate about the mechanisms. First, both types of nests are considered quite safe when it comes to nest predation (Alerstam & Högstedt, 1981; Huhta et al., 1998; Nice, 1957), the hole nesters have been reported to have even higher adult survival (Martin, 1993). Arboreal nests are usually well concealed, located high above the ground, and are less accessible to predators (Burhans et al., 2002; Söderström, 1999; Yahner & Cypher, 1987). The same holds for hole nests, in which predators cannot enter easily (Lack, 1954). Therefore, limited predation pressure is a likely explanation for the relatively high densities observed in these species. Secondly, explanations that are not mutually exclusive are based on the link between nest type and habitat preference. Evidently, both arboreal and cavity nesters are closely confined to trees, and are thus forest specialists. Although bird populations have declined rapidly in Europe (Inger et al., 2015), forest species have performed well (Schulze et al., 2019). The forest biotopes are less affected by current human activities when compared to open wetland (Lehikoinen et al., 2016) or agricultural landscapes (Reif & Hanzelka, 2020) and forest birds seem to be more tolerant to loss of habitat area (Desrochers et al., 2011; Hořák et al., 2010). Consequently, forest species, typically with arboreal and hole‐nesting strategies, may generally perform better and thus live at higher densities.

Our study was based on population densities collected in a single breeding season, although considering that more years may buffer the annual density fluctuations driven by temporal environmental variability (for instance, a species may have higher or lower abundance each year due to more or less favorable weather conditions, e.g., Pearce‐Higgins et al., 2015), extending the temporal window would compromise the spatial coverage of monitoring data used in the analysis. The year 2009 was characterized by the highest number of monitoring transects and including previous or subsequent years would reduce the sample size considerably. Since we focused on interspecific differences that are unlikely to vary over time, we are convinced that selecting the year with the maximum spatial coverage is the best solution, given the available data.

We confirmed that the quality and quantity of species‐specific habitats are crucial determinants of population density in birds, as they reflect the amount of suitable food resources as well as the available area. Currently, human impacts on natural landscapes are vastly changing the distribution and quality of indigenous biotopes, which negatively affects bird populations. Even though altered biotopes might objectively offer high‐quality environments and sufficient food resources, the ability of birds to adapt to new situations can be limited. This limitation potentially resides in the species‐specific perspectives of birds on habitat requirements (Lack, 1933). However, these are still poorly understood in detail, as it is difficult to observe them directly. Thus, further research is needed to find new ways for how to quantify the individual ecological space requirements of birds and effectively apply this knowledge to protect avian habitats.

## AUTHOR CONTRIBUTIONS

**David Hořák:** Conceptualization (lead); formal analysis (supporting); funding acquisition (lead); investigation (lead); methodology (lead); project administration (lead); resources (lead); supervision (lead); validation (equal); visualization (supporting); writing – original draft (lead); writing – review and editing (lead). **Javier Rivas‐Salvador:** Formal analysis (lead); methodology (equal); validation (equal); visualization (lead); writing – original draft (equal); writing – review and editing (equal). **Jan Farkač:** Data curation (supporting); formal analysis (supporting); investigation (supporting); writing – original draft (supporting). **Jiří Reif:** Data curation (lead); formal analysis (supporting); writing – original draft (supporting); writing – review and editing (equal).

## CONFLICT OF INTEREST

The authors declare no conflict of interest.

## Supporting information


Figure S1

Table S1

Table S2

Table S3

Table S4

Table S5

Table S6

## Data Availability

Data used in the analyses are available from Dryad depository 10.5061/dryad.hhmgqnkk0.
