# Supplementary material for: Traits and ecological space availability predict avian densities at the country scale of the Czech Republic
Source: Ecol Evol. 2022 Jul 17;12(7):e9119. doi: 10.1002/ece3.9119 (PMC9289119; doi:10.1002/ece3.9119)
Supplement: Supplementary file 2 — Table S1 [file ECE3-12-e9119-s001.docx]

| (Intercept) | Range | Forest dependency | Nest type | ssPC | SSI diet | SSI habitat | df | logLik | AIC | delta | weight |
| --- | --- | --- | --- | --- | --- | --- | --- | --- | --- | --- | --- |
| -0.59 | NA | NA | + | -8.05E-04 | -9.36E-02 | 2.22E-01 | 8 | -154.563 | 325.126 | 0 | 0.203 |
| -0.78 | NA | NA | + | -7.02E-04 | NA | 2.18E-01 | 7 | -155.57 | 325.141 | 0.015 | 0.202 |
| -0.638 | NA | NA | + | NA | NA | 2.06E-01 | 6 | -156.805 | 325.61 | 0.484 | 0.16 |
| -0.833 | 1.76E-09 | NA | + | -7.17E-04 | NA | 2.27E-01 | 8 | -155.084 | 326.168 | 1.042 | 0.121 |
| -0.646 | 1.61E-09 | NA | + | -8.14E-04 | -8.99E-02 | 2.29E-01 | 9 | -154.154 | 326.308 | 1.182 | 0.113 |
| -0.47 | NA | NA | + | NA | -7.46E-02 | 2.08E-01 | 7 | -156.16 | 326.32 | 1.195 | 0.112 |
| -0.686 | 1.66E-09 | NA | + | NA | NA | 2.14E-01 | 7 | -156.377 | 326.755 | 1.629 | 0.09 |
| -0.329 | NA | NA | + | NA | NA | 1.32E-01 | 6 | -149.411 | 310.823 | 0 | 0.268 |
| -0.28 | NA | NA | NA | NA | NA | 1.68E-01 | 3 | -152.593 | 311.187 | 0.364 | 0.223 |
| -0.184 | NA | NA | NA | 4.84E-04 | NA | 1.44E-01 | 4 | -151.733 | 311.465 | 0.643 | 0.194 |
| -0.355 | 7.55E-10 | NA | + | NA | NA | 1.38E-01 | 7 | -149.28 | 312.559 | 1.737 | 0.112 |
| -0.384 | NA | NA | + | NA | 2.54E-02 | 1.31E-01 | 7 | -149.351 | 312.702 | 1.879 | 0.105 |
| -0.322 | NA | NA | + | 2.92E-05 | NA | 1.32E-01 | 7 | -149.409 | 312.819 | 1.996 | 0.099 |
| -0.53 | NA | NA | + | NA | NA | 1.86E-01 | 6 | -156.812 | 325.625 | 0 | 0.39 |
| -0.637 | NA | NA | + | -4.99E-04 | NA | 1.94E-01 | 7 | -156.25 | 326.499 | 0.874 | 0.252 |
| -0.57 | 1.40E-09 | NA | + | NA | NA | 1.92E-01 | 7 | -156.522 | 327.044 | 1.42 | 0.192 |
| -0.446 | NA | NA | + | NA | -3.83E-02 | 1.86E-01 | 7 | -156.657 | 327.314 | 1.689 | 0.167 |
| -0.479 | NA | NA | + | NA | NA | 1.73E-01 | 6 | -159.511 | 331.022 | 0 | 0.433 |
| -0.515 | 1.25E-09 | NA | + | NA | NA | 1.79E-01 | 7 | -159.264 | 332.527 | 1.506 | 0.204 |
| -0.54 | NA | NA | + | -2.75E-04 | NA | 1.78E-01 | 7 | -159.343 | 332.686 | 1.664 | 0.188 |
| -0.412 | NA | NA | + | NA | -3.07E-02 | 1.74E-01 | 7 | -159.417 | 332.835 | 1.813 | 0.175 |
| -0.602 | NA | NA | + | NA | NA | 1.98E-01 | 6 | -168.814 | 349.628 | 0 | 0.403 |
| -0.704 | NA | NA | + | -5.00E-04 | NA | 2.05E-01 | 7 | -168.281 | 350.561 | 0.933 | 0.253 |
| -0.642 | 1.16E-09 | NA | + | NA | NA | 2.06E-01 | 7 | -168.551 | 351.101 | 1.474 | 0.193 |
| -0.568 | NA | NA | + | NA | -1.59E-02 | 1.98E-01 | 7 | -168.791 | 351.583 | 1.955 | 0.152 |
| -0.503 | NA | NA | + | NA | NA | 1.80E-01 | 6 | -147.089 | 306.177 | 0 | 0.273 |
| -0.622 | NA | NA | + | -5.44E-04 | NA | 1.91E-01 | 7 | -146.324 | 306.648 | 0.47 | 0.216 |
| -0.382 | NA | NA | + | NA | -5.59E-02 | 1.82E-01 | 7 | -146.727 | 307.453 | 1.276 | 0.144 |
| -0.49 | NA | NA | + | -6.01E-04 | -6.72E-02 | 1.94E-01 | 8 | -145.804 | 307.609 | 1.431 | 0.133 |
| -0.539 | 1.34E-09 | NA | + | NA | NA | 1.86E-01 | 7 | -146.836 | 307.672 | 1.495 | 0.129 |
| -0.663 | 1.41E-09 | NA | + | -5.54E-04 | NA | 1.97E-01 | 8 | -146.041 | 308.082 | 1.904 | 0.105 |
| -0.495 | NA | NA | + | NA | NA | 1.76E-01 | 6 | -147.614 | 307.229 | 0 | 0.385 |
| -0.592 | NA | NA | + | -4.31E-04 | NA | 1.83E-01 | 7 | -147.154 | 308.308 | 1.079 | 0.224 |
| -0.372 | NA | NA | + | NA | -5.50E-02 | 1.78E-01 | 7 | -147.249 | 308.499 | 1.27 | 0.204 |
| -0.532 | 1.40E-09 | NA | + | NA | NA | 1.81E-01 | 7 | -147.333 | 308.665 | 1.436 | 0.188 |
| -0.419 | NA | NA | + | NA | NA | 1.57E-01 | 6 | -152.483 | 316.966 | 0 | 0.372 |
| -0.453 | 1.26E-09 | NA | + | NA | NA | 1.62E-01 | 7 | -152.252 | 318.504 | 1.538 | 0.172 |
| -0.335 | NA | NA | NA | NA | NA | 1.95E-01 | 3 | -156.296 | 318.591 | 1.626 | 0.165 |
| -0.474 | NA | NA | + | -2.32E-04 | NA | 1.61E-01 | 7 | -152.366 | 318.731 | 1.766 | 0.154 |
| -0.399 | NA | NA | + | NA | -9.02E-03 | 1.57E-01 | 7 | -152.475 | 318.949 | 1.984 | 0.138 |
| -0.469 | NA | NA | + | NA | NA | 1.67E-01 | 6 | -149.44 | 310.881 | 0 | 0.429 |
| -0.539 | NA | NA | + | -3.15E-04 | NA | 1.74E-01 | 7 | -149.207 | 312.414 | 1.533 | 0.199 |
| -0.501 | 9.41E-10 | NA | + | NA | NA | 1.74E-01 | 7 | -149.224 | 312.448 | 1.567 | 0.196 |
| -0.4 | NA | NA | + | NA | -3.12E-02 | 1.68E-01 | 7 | -149.334 | 312.668 | 1.787 | 0.176 |
| -0.584 | NA | NA | + | NA | NA | 2.00E-01 | 6 | -153.382 | 318.764 | 0 | 0.239 |
| -0.714 | NA | NA | + | -6.34E-04 | NA | 2.12E-01 | 7 | -152.389 | 318.777 | 0.013 | 0.237 |
| -0.575 | NA | NA | + | -6.96E-04 | -7.09E-02 | 2.15E-01 | 8 | -151.837 | 319.674 | 0.909 | 0.151 |
| -0.761 | 1.59E-09 | NA | + | -6.54E-04 | NA | 2.19E-01 | 8 | -152.013 | 320.025 | 1.261 | 0.127 |
| -0.462 | NA | NA | + | NA | -5.74E-02 | 2.02E-01 | 7 | -153.02 | 320.04 | 1.275 | 0.126 |
| -0.624 | 1.45E-09 | NA | + | NA | NA | 2.07E-01 | 7 | -153.073 | 320.145 | 1.381 | 0.12 |
| -0.471 | NA | NA | + | NA | NA | 1.75E-01 | 6 | -143.739 | 299.477 | 0 | 0.28 |
| -0.585 | NA | NA | + | -5.21E-04 | NA | 1.82E-01 | 7 | -143.016 | 300.032 | 0.555 | 0.212 |
| -0.368 | NA | NA | + | NA | -4.95E-02 | 1.78E-01 | 7 | -143.438 | 300.876 | 1.399 | 0.139 |
| -0.503 | 1.03E-09 | NA | + | NA | NA | 1.80E-01 | 7 | -143.484 | 300.968 | 1.49 | 0.133 |
| -0.467 | NA | NA | + | -5.88E-04 | -6.30E-02 | 1.87E-01 | 8 | -142.537 | 301.073 | 1.596 | 0.126 |
| -0.627 | 1.18E-09 | NA | + | -5.51E-04 | NA | 1.89E-01 | 8 | -142.678 | 301.357 | 1.88 | 0.109 |
| -0.601 | NA | NA | + | NA | NA | 2.03E-01 | 6 | -168.225 | 348.45 | 0 | 0.331 |
| -0.714 | NA | NA | + | -5.58E-04 | NA | 2.14E-01 | 7 | -167.579 | 349.157 | 0.708 | 0.233 |
| -0.643 | 1.57E-09 | NA | + | NA | NA | 2.10E-01 | 7 | -167.916 | 349.832 | 1.382 | 0.166 |
| -0.508 | NA | NA | + | NA | -4.12E-02 | 2.03E-01 | 7 | -168.052 | 350.104 | 1.654 | 0.145 |
| -0.767 | 1.74E-09 | NA | + | -5.88E-04 | NA | 2.22E-01 | 8 | -167.199 | 350.398 | 1.949 | 0.125 |
| -0.424 | NA | NA | + | NA | NA | 1.59E-01 | 6 | -150.545 | 313.09 | 0 | 0.424 |
| -0.503 | NA | NA | + | -3.55E-04 | NA | 1.66E-01 | 7 | -150.252 | 314.503 | 1.414 | 0.209 |
| -0.457 | 1.17E-09 | NA | + | NA | NA | 1.64E-01 | 7 | -150.31 | 314.621 | 1.531 | 0.197 |
| -0.362 | NA | NA | + | NA | -2.90E-02 | 1.60E-01 | 7 | -150.455 | 314.91 | 1.82 | 0.171 |
| -0.498 | NA | NA | + | NA | NA | 1.70E-01 | 6 | -152.537 | 317.074 | 0 | 0.425 |
| -0.575 | NA | NA | + | -3.64E-04 | NA | 1.76E-01 | 7 | -152.228 | 318.456 | 1.381 | 0.213 |
| -0.53 | 1.19E-09 | NA | + | NA | NA | 1.75E-01 | 7 | -152.322 | 318.643 | 1.569 | 0.194 |
| -0.439 | NA | NA | + | NA | -2.67E-02 | 1.71E-01 | 7 | -152.465 | 318.93 | 1.856 | 0.168 |
| -0.49 | NA | NA | + | NA | NA | 1.75E-01 | 6 | -151.014 | 314.029 | 0 | 0.391 |
| -0.587 | NA | NA | + | -4.45E-04 | NA | 1.83E-01 | 7 | -150.536 | 315.072 | 1.043 | 0.232 |
| -0.527 | 1.24E-09 | NA | + | NA | NA | 1.81E-01 | 7 | -150.735 | 315.471 | 1.442 | 0.19 |
| -0.381 | NA | NA | + | NA | -4.92E-02 | 1.75E-01 | 7 | -150.746 | 315.493 | 1.464 | 0.188 |
| -0.462 | NA | NA | + | NA | NA | 1.71E-01 | 6 | -152.708 | 317.416 | 0 | 0.422 |
| -0.554 | NA | NA | + | -4.04E-04 | NA | 1.80E-01 | 7 | -152.331 | 318.662 | 1.247 | 0.226 |
| -0.494 | 1.24E-09 | NA | + | NA | NA | 1.76E-01 | 7 | -152.499 | 318.998 | 1.582 | 0.191 |
| -0.427 | NA | NA | + | NA | -1.64E-02 | 1.72E-01 | 7 | -152.68 | 319.36 | 1.944 | 0.16 |
| -0.721 | NA | NA | + | -6.46E-04 | NA | 2.07E-01 | 7 | -158.075 | 330.151 | 0 | 0.21 |
| -0.592 | NA | NA | + | NA | NA | 1.98E-01 | 6 | -159.091 | 330.182 | 0.031 | 0.207 |
| -0.567 | NA | NA | + | -7.22E-04 | -7.83E-02 | 2.12E-01 | 8 | -157.397 | 330.795 | 0.644 | 0.152 |
| -0.771 | 1.63E-09 | NA | + | -6.73E-04 | NA | 2.15E-01 | 8 | -157.631 | 331.262 | 1.112 | 0.121 |
| -0.456 | NA | NA | + | NA | -6.32E-02 | 2.01E-01 | 7 | -158.647 | 331.294 | 1.143 | 0.119 |
| -0.633 | 1.47E-09 | NA | + | NA | NA | 2.05E-01 | 7 | -158.735 | 331.469 | 1.319 | 0.109 |
| -0.622 | 1.50E-09 | NA | + | -7.43E-04 | -7.43E-02 | 2.19E-01 | 9 | -157.019 | 332.038 | 1.887 | 0.082 |
| -0.494 | NA | NA | + | NA | NA | 1.83E-01 | 6 | -148.186 | 308.371 | 0 | 0.283 |
| -0.607 | NA | NA | + | -5.32E-04 | NA | 1.93E-01 | 7 | -147.49 | 308.98 | 0.609 | 0.209 |
| -0.384 | NA | NA | + | NA | -5.07E-02 | 1.83E-01 | 7 | -147.886 | 309.772 | 1.401 | 0.141 |
| -0.53 | 1.33E-09 | NA | + | NA | NA | 1.88E-01 | 7 | -147.904 | 309.808 | 1.437 | 0.138 |
| -0.485 | NA | NA | + | -5.84E-04 | -6.10E-02 | 1.95E-01 | 8 | -147.058 | 310.116 | 1.745 | 0.118 |
| -0.654 | 1.50E-09 | NA | + | -5.62E-04 | NA | 2.01E-01 | 8 | -147.13 | 310.26 | 1.889 | 0.11 |
| -0.486 | NA | NA | + | NA | NA | 1.73E-01 | 6 | -157.086 | 326.172 | 0 | 0.43 |
| -0.523 | 1.37E-09 | NA | + | NA | NA | 1.78E-01 | 7 | -156.816 | 327.633 | 1.46 | 0.207 |
| -0.544 | NA | NA | + | -2.67E-04 | NA | 1.78E-01 | 7 | -156.931 | 327.862 | 1.689 | 0.185 |
| -0.41 | NA | NA | + | NA | -3.45E-02 | 1.74E-01 | 7 | -156.966 | 327.931 | 1.759 | 0.178 |
| -0.41 | NA | NA | + | NA | NA | 1.56E-01 | 6 | -150.137 | 312.274 | 0 | 0.369 |
| -0.332 | NA | NA | NA | NA | NA | 1.91E-01 | 3 | -153.857 | 313.715 | 1.441 | 0.18 |
| -0.44 | 1.16E-09 | NA | + | NA | NA | 1.61E-01 | 7 | -149.935 | 313.87 | 1.596 | 0.166 |
| -0.352 | NA | NA | + | NA | -2.69E-02 | 1.58E-01 | 7 | -150.065 | 314.13 | 1.856 | 0.146 |
| -0.432 | NA | NA | + | -9.34E-05 | NA | 1.58E-01 | 7 | -150.117 | 314.234 | 1.96 | 0.139 |
| -0.717 | NA | NA | + | -6.30E-04 | NA | 2.10E-01 | 7 | -151.683 | 317.367 | 0 | 0.191 |
| -0.589 | NA | NA | + | NA | NA | 1.99E-01 | 6 | -152.698 | 317.397 | 0.03 | 0.188 |
| -0.531 | NA | NA | + | -7.23E-04 | -8.96E-02 | 2.10E-01 | 8 | -150.735 | 317.47 | 0.103 | 0.182 |
| -0.422 | NA | NA | + | NA | -7.35E-02 | 1.98E-01 | 7 | -152.055 | 318.11 | 0.743 | 0.132 |
| -0.767 | 1.75E-09 | NA | + | -6.51E-04 | NA | 2.17E-01 | 8 | -151.233 | 318.467 | 1.1 | 0.11 |
| -0.632 | 1.62E-09 | NA | + | NA | NA | 2.06E-01 | 7 | -152.32 | 318.64 | 1.273 | 0.101 |
| -0.585 | 1.55E-09 | NA | + | -7.37E-04 | -8.51E-02 | 2.17E-01 | 9 | -150.38 | 318.76 | 1.393 | 0.095 |
| -0.543 | NA | NA | + | NA | NA | 1.84E-01 | 6 | -150.227 | 312.455 | 0 | 0.213 |
| -0.686 | NA | NA | + | -6.16E-04 | NA | 1.98E-01 | 7 | -149.262 | 312.524 | 0.069 | 0.206 |
| -0.526 | NA | NA | + | -6.72E-04 | -7.72E-02 | 2.01E-01 | 8 | -148.543 | 313.085 | 0.63 | 0.155 |
| -0.393 | NA | NA | + | NA | -6.70E-02 | 1.85E-01 | 7 | -149.688 | 313.375 | 0.92 | 0.134 |
| -0.738 | 1.33E-09 | NA | + | -6.59E-04 | NA | 2.08E-01 | 8 | -148.875 | 313.751 | 1.296 | 0.111 |
| -0.577 | 1.08E-09 | NA | + | NA | NA | 1.91E-01 | 7 | -149.973 | 313.946 | 1.491 | 0.101 |
| -0.581 | 1.20E-09 | NA | + | -7.08E-04 | -7.34E-02 | 2.09E-01 | 9 | -148.226 | 314.451 | 1.997 | 0.079 |
| -0.545 | NA | NA | + | NA | NA | 1.95E-01 | 6 | -153.623 | 319.246 | 0 | 0.378 |
| -0.404 | NA | NA | + | NA | -6.20E-02 | 1.94E-01 | 7 | -153.171 | 320.342 | 1.097 | 0.219 |
| -0.591 | 1.71E-09 | NA | + | NA | NA | 2.02E-01 | 7 | -153.228 | 320.455 | 1.209 | 0.207 |
| -0.628 | NA | NA | + | -3.79E-04 | NA | 2.03E-01 | 7 | -153.277 | 320.554 | 1.308 | 0.197 |
| -0.506 | NA | NA | + | -7.00E-04 | -1.01E-01 | 2.17E-01 | 8 | -149.403 | 314.806 | 0 | 0.21 |
| -0.714 | NA | NA | + | -6.09E-04 | NA | 2.15E-01 | 7 | -150.654 | 315.308 | 0.503 | 0.163 |
| -0.596 | NA | NA | + | NA | NA | 2.05E-01 | 6 | -151.681 | 315.363 | 0.557 | 0.159 |
| -0.402 | NA | NA | + | NA | -8.64E-02 | 2.05E-01 | 7 | -150.756 | 315.511 | 0.706 | 0.148 |
| -0.568 | 1.35E-09 | NA | + | -7.58E-04 | -9.83E-02 | 2.28E-01 | 9 | -148.887 | 315.774 | 0.968 | 0.13 |
| -0.773 | 1.42E-09 | NA | + | -6.72E-04 | NA | 2.27E-01 | 8 | -150.086 | 316.172 | 1.367 | 0.106 |
| -0.633 | 1.13E-09 | NA | + | NA | NA | 2.13E-01 | 7 | -151.322 | 316.644 | 1.838 | 0.084 |
| -0.363 | NA | NA | + | NA | NA | 1.42E-01 | 6 | -139.912 | 291.823 | 0 | 0.369 |
| -0.281 | NA | NA | NA | NA | NA | 1.72E-01 | 3 | -143.579 | 293.157 | 1.334 | 0.189 |
| -0.387 | 7.81E-10 | NA | + | NA | NA | 1.46E-01 | 7 | -139.77 | 293.539 | 1.716 | 0.156 |
| -0.411 | NA | NA | + | -1.99E-04 | NA | 1.45E-01 | 7 | -139.817 | 293.633 | 1.81 | 0.149 |
| -0.381 | NA | NA | + | NA | 8.58E-03 | 1.41E-01 | 7 | -139.904 | 293.808 | 1.984 | 0.137 |
| -0.515 | NA | NA | + | NA | NA | 1.78E-01 | 6 | -159.117 | 330.235 | 0 | 0.406 |
| -0.603 | NA | NA | + | -3.91E-04 | NA | 1.87E-01 | 7 | -158.782 | 331.564 | 1.329 | 0.209 |
| -0.557 | 1.53E-09 | NA | + | NA | NA | 1.84E-01 | 7 | -158.797 | 331.595 | 1.36 | 0.206 |
| -0.42 | NA | NA | + | NA | -4.23E-02 | 1.78E-01 | 7 | -158.936 | 331.872 | 1.637 | 0.179 |
| -0.374 | NA | NA | + | NA | NA | 1.52E-01 | 6 | -137.207 | 286.414 | 0 | 0.299 |
| -0.298 | NA | NA | NA | NA | NA | 1.80E-01 | 3 | -140.536 | 287.072 | 0.658 | 0.215 |
| -0.401 | 1.00E-09 | NA | + | NA | NA | 1.56E-01 | 7 | -137.036 | 288.071 | 1.657 | 0.131 |
| -0.243 | NA | NA | NA | 3.22E-04 | NA | 1.65E-01 | 4 | -140.103 | 288.206 | 1.791 | 0.122 |
| -0.413 | NA | NA | + | -1.65E-04 | NA | 1.56E-01 | 7 | -137.137 | 288.275 | 1.86 | 0.118 |
| -0.334 | NA | NA | + | NA | -1.84E-02 | 1.53E-01 | 7 | -137.169 | 288.338 | 1.923 | 0.114 |
| -0.679 | NA | NA | + | NA | NA | 2.16E-01 | 6 | -168.227 | 348.455 | 0 | 0.266 |
| -0.791 | NA | NA | + | -5.70E-04 | NA | 2.25E-01 | 7 | -167.539 | 349.077 | 0.622 | 0.195 |
| -0.732 | 1.87E-09 | NA | + | NA | NA | 2.24E-01 | 7 | -167.762 | 349.524 | 1.069 | 0.156 |
| -0.547 | NA | NA | + | NA | -5.86E-02 | 2.17E-01 | 7 | -167.866 | 349.733 | 1.278 | 0.14 |
| -0.644 | NA | NA | + | -6.41E-04 | -7.14E-02 | 2.27E-01 | 8 | -167.009 | 350.017 | 1.562 | 0.122 |
| -0.852 | 1.98E-09 | NA | + | -5.93E-04 | NA | 2.34E-01 | 8 | -167.012 | 350.024 | 1.569 | 0.121 |
| -0.723 | NA | NA | + | -7.18E-04 | NA | 2.06E-01 | 7 | -152.034 | 318.068 | 0 | 0.234 |
| -0.579 | NA | NA | + | NA | NA | 1.95E-01 | 6 | -153.287 | 318.573 | 0.506 | 0.181 |
| -0.572 | NA | NA | + | -7.95E-04 | -7.52E-02 | 2.08E-01 | 8 | -151.384 | 318.768 | 0.701 | 0.165 |
| -0.774 | 1.81E-09 | NA | + | -7.32E-04 | NA | 2.14E-01 | 8 | -151.583 | 319.166 | 1.098 | 0.135 |
| -0.625 | 1.71E-09 | NA | + | NA | NA | 2.02E-01 | 7 | -152.891 | 319.781 | 1.714 | 0.099 |
| -0.45 | NA | NA | + | NA | -5.80E-02 | 1.96E-01 | 7 | -152.898 | 319.797 | 1.729 | 0.098 |
| -0.628 | 1.64E-09 | NA | + | -8.03E-04 | -7.05E-02 | 2.15E-01 | 9 | -151.013 | 320.026 | 1.958 | 0.088 |
| -0.499 | NA | NA | + | NA | NA | 1.83E-01 | 6 | -159.64 | 331.281 | 0 | 0.427 |
| -0.538 | 1.50E-09 | NA | + | NA | NA | 1.89E-01 | 7 | -159.346 | 332.692 | 1.411 | 0.211 |
| -0.413 | NA | NA | + | NA | -3.82E-02 | 1.83E-01 | 7 | -159.484 | 332.968 | 1.687 | 0.184 |
| -0.552 | NA | NA | + | -2.41E-04 | NA | 1.88E-01 | 7 | -159.512 | 333.024 | 1.744 | 0.179 |
| -0.649 | NA | NA | + | -9.30E-04 | -1.05E-01 | 2.38E-01 | 8 | -153.066 | 322.132 | 0 | 0.339 |
| -0.729 | 1.76E-09 | NA | + | -9.78E-04 | -9.88E-02 | 2.50E-01 | 9 | -152.412 | 322.824 | 0.693 | 0.24 |
| -0.861 | NA | NA | + | -8.06E-04 | NA | 2.35E-01 | 7 | -154.452 | 322.904 | 0.772 | 0.23 |
| -0.937 | 1.97E-09 | NA | + | -8.67E-04 | NA | 2.48E-01 | 8 | -153.641 | 323.282 | 1.151 | 0.191 |
| -0.439 | NA | NA | + | NA | NA | 1.55E-01 | 6 | -150.582 | 313.164 | 0 | 0.433 |
| -0.472 | 1.24E-09 | NA | + | NA | NA | 1.60E-01 | 7 | -150.368 | 314.736 | 1.573 | 0.197 |
| -0.351 | NA | NA | + | NA | -3.89E-02 | 1.57E-01 | 7 | -150.42 | 314.839 | 1.675 | 0.187 |
| -0.495 | NA | NA | + | -2.41E-04 | NA | 1.60E-01 | 7 | -150.449 | 314.897 | 1.733 | 0.182 |
| -0.485 | NA | NA | + | NA | NA | 1.69E-01 | 6 | -145.662 | 303.325 | 0 | 0.273 |
| -0.604 | NA | NA | + | -5.19E-04 | NA | 1.80E-01 | 7 | -144.947 | 303.894 | 0.569 | 0.205 |
| -0.351 | NA | NA | + | NA | -6.03E-02 | 1.71E-01 | 7 | -145.226 | 304.453 | 1.128 | 0.155 |
| -0.461 | NA | NA | + | -5.73E-04 | -7.00E-02 | 1.83E-01 | 8 | -144.36 | 304.72 | 1.395 | 0.136 |
| -0.516 | 1.09E-09 | NA | + | NA | NA | 1.75E-01 | 7 | -145.435 | 304.87 | 1.545 | 0.126 |
| -0.648 | 1.29E-09 | NA | + | -5.53E-04 | NA | 1.87E-01 | 8 | -144.63 | 305.259 | 1.934 | 0.104 |
| -0.671 | NA | NA | + | -7.80E-04 | -9.66E-02 | 2.55E-01 | 8 | -169.146 | 354.293 | 0 | 0.166 |
| -0.75 | NA | NA | + | NA | NA | 2.47E-01 | 6 | -171.18 | 354.361 | 0.068 | 0.161 |
| -0.873 | NA | NA | + | -6.68E-04 | NA | 2.55E-01 | 7 | -170.181 | 354.361 | 0.069 | 0.161 |
| -0.57 | NA | NA | + | NA | -7.84E-02 | 2.45E-01 | 7 | -170.49 | 354.981 | 0.688 | 0.118 |
| -0.943 | 2.44E-09 | NA | + | -6.94E-04 | NA | 2.66E-01 | 8 | -169.505 | 355.01 | 0.717 | 0.116 |
| -0.811 | 2.29E-09 | NA | + | NA | NA | 2.56E-01 | 7 | -170.593 | 355.186 | 0.893 | 0.106 |
| -0.748 | 2.14E-09 | NA | + | -7.95E-04 | -8.91E-02 | 2.64E-01 | 9 | -168.629 | 355.257 | 0.965 | 0.103 |
| -0.641 | 2.03E-09 | NA | + | NA | -7.10E-02 | 2.53E-01 | 8 | -170.031 | 356.063 | 1.77 | 0.069 |
| -0.198 | NA | NA | NA | 5.34E-04 | NA | 1.48E-01 | 4 | -159.891 | 327.783 | 0 | 0.23 |
| -0.309 | NA | NA | NA | NA | NA | 1.78E-01 | 3 | -160.923 | 327.845 | 0.062 | 0.223 |
| -0.365 | NA | NA | + | NA | NA | 1.41E-01 | 6 | -158.108 | 328.215 | 0.432 | 0.185 |
| -0.225 | 1.10E-09 | NA | NA | 5.28E-04 | NA | 1.52E-01 | 5 | -159.761 | 329.521 | 1.738 | 0.096 |
| -0.338 | 1.19E-09 | NA | NA | NA | NA | 1.81E-01 | 4 | -160.773 | 329.546 | 1.763 | 0.095 |
| -0.217 | NA | NA | NA | 5.49E-04 | 1.08E-02 | 1.47E-01 | 5 | -159.881 | 329.762 | 1.979 | 0.085 |
| -0.271 | NA | NA | NA | NA | -1.87E-02 | 1.77E-01 | 4 | -160.889 | 329.778 | 1.995 | 0.085 |
| -0.656 | NA | NA | + | NA | NA | 2.14E-01 | 6 | -161.667 | 335.335 | 0 | 0.261 |
| -0.768 | NA | NA | + | -5.65E-04 | NA | 2.23E-01 | 7 | -160.93 | 335.86 | 0.526 | 0.201 |
| -0.703 | 1.75E-09 | NA | + | NA | NA | 2.22E-01 | 7 | -161.271 | 336.541 | 1.207 | 0.143 |
| -0.523 | NA | NA | + | NA | -5.76E-02 | 2.13E-01 | 7 | -161.282 | 336.564 | 1.23 | 0.141 |
| -0.617 | NA | NA | + | -6.52E-04 | -7.29E-02 | 2.22E-01 | 8 | -160.323 | 336.646 | 1.311 | 0.136 |
| -0.824 | 1.88E-09 | NA | + | -5.89E-04 | NA | 2.32E-01 | 8 | -160.468 | 336.935 | 1.601 | 0.117 |
| -0.699 | NA | NA | + | -6.70E-04 | NA | 2.08E-01 | 7 | -151.69 | 317.381 | 0 | 0.249 |
| -0.565 | NA | NA | + | NA | NA | 1.97E-01 | 6 | -152.813 | 317.626 | 0.245 | 0.22 |
| -0.554 | NA | NA | + | -7.43E-04 | -7.41E-02 | 2.12E-01 | 8 | -151.059 | 318.118 | 0.738 | 0.172 |
| -0.741 | 1.52E-09 | NA | + | -6.82E-04 | NA | 2.14E-01 | 8 | -151.348 | 318.696 | 1.316 | 0.129 |
| -0.44 | NA | NA | + | NA | -5.82E-02 | 2.00E-01 | 7 | -152.421 | 318.843 | 1.462 | 0.12 |
| -0.603 | 1.42E-09 | NA | + | NA | NA | 2.03E-01 | 7 | -152.518 | 319.035 | 1.655 | 0.109 |
| -0.454 | NA | NA | + | NA | NA | 1.67E-01 | 6 | -145.034 | 302.067 | 0 | 0.408 |
| -0.547 | NA | NA | + | -4.17E-04 | NA | 1.74E-01 | 7 | -144.611 | 303.221 | 1.154 | 0.229 |
| -0.486 | 1.18E-09 | NA | + | NA | NA | 1.72E-01 | 7 | -144.804 | 303.608 | 1.54 | 0.189 |
| -0.379 | NA | NA | + | NA | -3.51E-02 | 1.69E-01 | 7 | -144.893 | 303.786 | 1.718 | 0.173 |
| -0.427 | NA | NA | + | NA | NA | 1.61E-01 | 6 | -150.937 | 313.874 | 0 | 0.436 |
| -0.495 | NA | NA | + | -2.90E-04 | NA | 1.68E-01 | 7 | -150.745 | 315.49 | 1.616 | 0.195 |
| -0.457 | 1.05E-09 | NA | + | NA | NA | 1.66E-01 | 7 | -150.752 | 315.505 | 1.631 | 0.193 |
| -0.365 | NA | NA | + | NA | -2.96E-02 | 1.63E-01 | 7 | -150.846 | 315.691 | 1.817 | 0.176 |
| -0.517 | NA | NA | + | NA | NA | 1.84E-01 | 6 | -155.584 | 323.167 | 0 | 0.416 |
| -0.554 | 1.22E-09 | NA | + | NA | NA | 1.92E-01 | 7 | -155.31 | 324.619 | 1.452 | 0.201 |
| -0.586 | NA | NA | + | -3.24E-04 | NA | 1.91E-01 | 7 | -155.342 | 324.684 | 1.517 | 0.195 |
| -0.42 | NA | NA | + | NA | -4.32E-02 | 1.85E-01 | 7 | -155.381 | 324.762 | 1.595 | 0.187 |
| -0.459 | NA | NA | + | NA | NA | 1.68E-01 | 6 | -146.183 | 304.365 | 0 | 0.404 |
| -0.542 | NA | NA | + | -3.79E-04 | NA | 1.75E-01 | 7 | -145.814 | 305.628 | 1.262 | 0.215 |
| -0.493 | 9.63E-10 | NA | + | NA | NA | 1.76E-01 | 7 | -145.89 | 305.779 | 1.414 | 0.199 |
| -0.368 | NA | NA | + | NA | -4.26E-02 | 1.71E-01 | 7 | -145.981 | 305.961 | 1.596 | 0.182 |
| -0.498 | NA | NA | + | NA | NA | 1.78E-01 | 6 | -159.36 | 330.719 | 0 | 0.445 |
| -0.534 | 1.20E-09 | NA | + | NA | NA | 1.85E-01 | 7 | -159.12 | 332.239 | 1.52 | 0.208 |
| -0.443 | NA | NA | + | NA | -2.44E-02 | 1.79E-01 | 7 | -159.298 | 332.597 | 1.877 | 0.174 |
| -0.535 | NA | NA | + | -1.63E-04 | NA | 1.82E-01 | 7 | -159.302 | 332.604 | 1.885 | 0.173 |
| -0.547 | NA | NA | + | NA | NA | 1.94E-01 | 6 | -161.817 | 335.635 | 0 | 0.43 |
| -0.586 | 1.51E-09 | NA | + | NA | NA | 2.00E-01 | 7 | -161.55 | 337.099 | 1.464 | 0.207 |
| -0.612 | NA | NA | + | -3.03E-04 | NA | 2.00E-01 | 7 | -161.622 | 337.244 | 1.609 | 0.192 |
| -0.488 | NA | NA | + | NA | -2.69E-02 | 1.95E-01 | 7 | -161.746 | 337.491 | 1.856 | 0.17 |
| -0.486 | NA | NA | + | NA | NA | 1.76E-01 | 6 | -157.133 | 326.265 | 0 | 0.439 |
| -0.521 | 1.04E-09 | NA | + | NA | NA | 1.84E-01 | 7 | -156.89 | 327.78 | 1.514 | 0.206 |
| -0.54 | NA | NA | + | -2.55E-04 | NA | 1.81E-01 | 7 | -156.991 | 327.981 | 1.716 | 0.186 |
| -0.44 | NA | NA | + | NA | -2.15E-02 | 1.77E-01 | 7 | -157.087 | 328.175 | 1.909 | 0.169 |
| -0.209 | NA | NA | NA | NA | NA | 1.50E-01 | 3 | -149.022 | 304.044 | 0 | 0.309 |
| -0.131 | NA | NA | NA | 4.43E-04 | NA | 1.29E-01 | 4 | -148.256 | 304.512 | 0.469 | 0.245 |
| -0.296 | NA | NA | + | NA | NA | 1.25E-01 | 6 | -146.429 | 304.858 | 0.814 | 0.206 |
| -0.231 | 6.67E-10 | NA | NA | NA | NA | 1.54E-01 | 4 | -148.916 | 305.833 | 1.789 | 0.126 |
| -0.204 | NA | NA | NA | NA | -2.94E-03 | 1.50E-01 | 4 | -149.021 | 306.042 | 1.998 | 0.114 |
| -0.44 | NA | NA | + | NA | NA | 1.62E-01 | 6 | -143.045 | 298.09 | 0 | 0.43 |
| -0.514 | NA | NA | + | -3.12E-04 | NA | 1.68E-01 | 7 | -142.803 | 299.607 | 1.517 | 0.202 |
| -0.472 | 1.06E-09 | NA | + | NA | NA | 1.67E-01 | 7 | -142.815 | 299.629 | 1.539 | 0.199 |
| -0.385 | NA | NA | + | NA | -2.44E-02 | 1.63E-01 | 7 | -142.981 | 299.961 | 1.871 | 0.169 |
| -0.412 | NA | NA | + | NA | NA | 1.52E-01 | 6 | -150.605 | 313.21 | 0 | 0.439 |
| -0.481 | NA | NA | + | -3.01E-04 | NA | 1.58E-01 | 7 | -150.396 | 314.793 | 1.583 | 0.199 |
| -0.444 | 9.69E-10 | NA | + | NA | NA | 1.58E-01 | 7 | -150.403 | 314.807 | 1.597 | 0.198 |
| -0.388 | NA | NA | + | NA | -1.07E-02 | 1.53E-01 | 7 | -150.593 | 315.187 | 1.977 | 0.164 |
| -0.48 | NA | NA | + | NA | NA | 1.70E-01 | 6 | -157.388 | 326.777 | 0 | 0.44 |
| -0.516 | 1.14E-09 | NA | + | NA | NA | 1.76E-01 | 7 | -157.16 | 328.32 | 1.543 | 0.204 |
| -0.531 | NA | NA | + | -2.33E-04 | NA | 1.74E-01 | 7 | -157.27 | 328.54 | 1.764 | 0.182 |
| -0.423 | NA | NA | + | NA | -2.59E-02 | 1.70E-01 | 7 | -157.319 | 328.639 | 1.862 | 0.174 |
| -0.398 | NA | NA | + | NA | NA | 1.52E-01 | 6 | -141.643 | 295.287 | 0 | 0.436 |
| -0.468 | NA | NA | + | -2.89E-04 | NA | 1.58E-01 | 7 | -141.437 | 296.875 | 1.588 | 0.197 |
| -0.426 | 1.01E-09 | NA | + | NA | NA | 1.57E-01 | 7 | -141.472 | 296.944 | 1.657 | 0.19 |
| -0.336 | NA | NA | + | NA | -2.85E-02 | 1.54E-01 | 7 | -141.55 | 297.1 | 1.813 | 0.176 |
| -0.677 | NA | NA | + | -9.73E-04 | -1.21E-01 | 2.63E-01 | 8 | -163.139 | 342.277 | 0 | 0.497 |
| -0.748 | 1.97E-09 | NA | + | -9.85E-04 | -1.15E-01 | 2.72E-01 | 9 | -162.647 | 343.294 | 1.016 | 0.299 |
| -0.925 | NA | NA | + | -8.09E-04 | NA | 2.64E-01 | 7 | -165.027 | 344.053 | 1.776 | 0.204 |
| -0.576 | NA | NA | + | -8.67E-04 | -1.10E-01 | 2.32E-01 | 8 | -151.376 | 318.752 | 0 | 0.306 |
| -0.801 | NA | NA | + | -7.45E-04 | NA | 2.32E-01 | 7 | -152.893 | 319.786 | 1.034 | 0.183 |
| -0.636 | 1.69E-09 | NA | + | -8.80E-04 | -1.04E-01 | 2.39E-01 | 9 | -150.995 | 319.989 | 1.237 | 0.165 |
| -0.44 | NA | NA | + | NA | -8.96E-02 | 2.17E-01 | 7 | -153.339 | 320.678 | 1.925 | 0.117 |
| -0.645 | NA | NA | + | NA | NA | 2.19E-01 | 6 | -154.357 | 320.714 | 1.962 | 0.115 |
| -0.859 | 2.01E-09 | NA | + | -7.68E-04 | NA | 2.40E-01 | 8 | -152.36 | 320.719 | 1.967 | 0.115 |
| -0.468 | NA | NA | + | NA | NA | 1.67E-01 | 6 | -146.311 | 304.622 | 0 | 0.426 |
| -0.541 | NA | NA | + | -3.29E-04 | NA | 1.73E-01 | 7 | -146.042 | 306.084 | 1.462 | 0.205 |
| -0.499 | 1.00E-09 | NA | + | NA | NA | 1.73E-01 | 7 | -146.092 | 306.184 | 1.562 | 0.195 |
| -0.398 | NA | NA | + | NA | -3.18E-02 | 1.68E-01 | 7 | -146.202 | 306.403 | 1.781 | 0.175 |
| -0.409 | NA | NA | + | NA | NA | 1.57E-01 | 6 | -150.237 | 312.474 | 0 | 0.392 |
| -0.438 | 9.99E-10 | NA | + | NA | NA | 1.62E-01 | 7 | -150.055 | 314.11 | 1.636 | 0.173 |
| -0.322 | NA | NA | NA | NA | NA | 1.91E-01 | 3 | -154.216 | 314.432 | 1.958 | 0.147 |
| -0.416 | NA | NA | + | -3.23E-05 | NA | 1.58E-01 | 7 | -150.235 | 314.47 | 1.995 | 0.144 |
| -0.409 | NA | NA | + | NA | 4.27E-04 | 1.57E-01 | 7 | -150.237 | 314.474 | 2 | 0.144 |
| -0.526 | NA | NA | + | NA | NA | 1.80E-01 | 6 | -151.542 | 315.084 | 0 | 0.281 |
| -0.655 | NA | NA | + | -5.86E-04 | NA | 1.93E-01 | 7 | -150.708 | 315.416 | 0.332 | 0.238 |
| -0.563 | 1.26E-09 | NA | + | NA | NA | 1.86E-01 | 7 | -151.295 | 316.591 | 1.507 | 0.132 |
| -0.446 | NA | NA | + | NA | -3.64E-02 | 1.81E-01 | 7 | -151.398 | 316.797 | 1.713 | 0.119 |
| -0.699 | 1.38E-09 | NA | + | -6.04E-04 | NA | 1.99E-01 | 8 | -150.409 | 316.818 | 1.735 | 0.118 |
| -0.558 | NA | NA | + | -6.23E-04 | -4.74E-02 | 1.95E-01 | 8 | -150.466 | 316.932 | 1.848 | 0.112 |
| -0.39 | NA | NA | + | NA | NA | 1.52E-01 | 6 | -146.183 | 304.366 | 0 | 0.379 |
| -0.418 | 9.79E-10 | NA | + | NA | NA | 1.57E-01 | 7 | -146.009 | 306.017 | 1.651 | 0.166 |
| -0.323 | NA | NA | NA | NA | NA | 1.89E-01 | 3 | -150.065 | 306.13 | 1.763 | 0.157 |
| -0.434 | NA | NA | + | -1.75E-04 | NA | 1.56E-01 | 7 | -146.111 | 306.223 | 1.857 | 0.15 |
| -0.333 | NA | NA | + | NA | -2.58E-02 | 1.53E-01 | 7 | -146.113 | 306.226 | 1.86 | 0.149 |
| -0.699 | NA | NA | + | NA | NA | 2.33E-01 | 6 | -162.056 | 336.112 | 0 | 0.172 |
| -0.624 | NA | NA | + | -7.42E-04 | -9.30E-02 | 2.42E-01 | 8 | -160.063 | 336.126 | 0.014 | 0.17 |
| -0.813 | NA | NA | + | -6.27E-04 | NA | 2.42E-01 | 7 | -161.098 | 336.195 | 0.083 | 0.165 |
| -0.53 | NA | NA | + | NA | -7.46E-02 | 2.32E-01 | 7 | -161.379 | 336.759 | 0.646 | 0.124 |
| -0.873 | 2.09E-09 | NA | + | -6.61E-04 | NA | 2.51E-01 | 8 | -160.53 | 337.06 | 0.948 | 0.107 |
| -0.747 | 1.89E-09 | NA | + | NA | NA | 2.40E-01 | 7 | -161.598 | 337.196 | 1.083 | 0.1 |
| -0.688 | 1.84E-09 | NA | + | -7.65E-04 | -8.72E-02 | 2.50E-01 | 9 | -159.622 | 337.243 | 1.131 | 0.097 |
| -0.586 | 1.67E-09 | NA | + | NA | -6.89E-02 | 2.39E-01 | 8 | -161.024 | 338.048 | 1.936 | 0.065 |
| -0.516 | NA | NA | + | NA | NA | 1.82E-01 | 6 | -156.802 | 325.605 | 0 | 0.423 |
| -0.591 | NA | NA | + | -3.63E-04 | NA | 1.87E-01 | 7 | -156.503 | 327.005 | 1.4 | 0.21 |
| -0.548 | 1.12E-09 | NA | + | NA | NA | 1.88E-01 | 7 | -156.574 | 327.148 | 1.543 | 0.196 |
| -0.452 | NA | NA | + | NA | -2.94E-02 | 1.83E-01 | 7 | -156.71 | 327.421 | 1.816 | 0.171 |
| -0.538 | NA | NA | + | NA | NA | 1.83E-01 | 6 | -172.9 | 357.799 | 0 | 0.451 |
| -0.577 | 1.41E-09 | NA | + | NA | NA | 1.89E-01 | 7 | -172.674 | 359.348 | 1.549 | 0.208 |
| -0.576 | NA | NA | + | -1.72E-04 | NA | 1.87E-01 | 7 | -172.844 | 359.687 | 1.888 | 0.175 |
| -0.522 | NA | NA | + | NA | -7.28E-03 | 1.84E-01 | 7 | -172.895 | 359.79 | 1.991 | 0.167 |
| -0.724 | NA | NA | + | -6.56E-04 | NA | 2.09E-01 | 7 | -155.089 | 324.179 | 0 | 0.241 |
| -0.592 | NA | NA | + | NA | NA | 1.99E-01 | 6 | -156.127 | 324.254 | 0.075 | 0.233 |
| -0.587 | NA | NA | + | -7.16E-04 | -6.61E-02 | 2.11E-01 | 8 | -154.598 | 325.197 | 1.018 | 0.145 |
| -0.778 | 1.65E-09 | NA | + | -6.86E-04 | NA | 2.18E-01 | 8 | -154.626 | 325.251 | 1.073 | 0.141 |
| -0.635 | 1.47E-09 | NA | + | NA | NA | 2.07E-01 | 7 | -155.761 | 325.522 | 1.343 | 0.123 |
| -0.475 | NA | NA | + | NA | -5.21E-02 | 2.00E-01 | 7 | -155.821 | 325.642 | 1.463 | 0.116 |
| -0.493 | NA | NA | + | NA | NA | 1.69E-01 | 6 | -149.04 | 310.08 | 0 | 0.391 |
| -0.359 | NA | NA | + | NA | -5.77E-02 | 1.69E-01 | 7 | -148.644 | 311.288 | 1.208 | 0.214 |
| -0.578 | NA | NA | + | -3.68E-04 | NA | 1.75E-01 | 7 | -148.717 | 311.434 | 1.354 | 0.199 |
| -0.533 | 1.36E-09 | NA | + | NA | NA | 1.75E-01 | 7 | -148.725 | 311.45 | 1.37 | 0.197 |
| -0.442 | NA | NA | + | NA | NA | 1.63E-01 | 6 | -146.718 | 305.435 | 0 | 0.442 |
| -0.51 | NA | NA | + | -2.91E-04 | NA | 1.70E-01 | 7 | -146.507 | 307.013 | 1.578 | 0.201 |
| -0.47 | 9.77E-10 | NA | + | NA | NA | 1.68E-01 | 7 | -146.565 | 307.131 | 1.695 | 0.189 |
| -0.405 | NA | NA | + | NA | -1.68E-02 | 1.64E-01 | 7 | -146.687 | 307.375 | 1.94 | 0.168 |
| -0.359 | NA | NA | + | NA | NA | 1.40E-01 | 6 | -137.823 | 287.646 | 0 | 0.442 |
| -0.434 | NA | NA | + | -3.02E-04 | NA | 1.46E-01 | 7 | -137.589 | 289.178 | 1.531 | 0.206 |
| -0.384 | 7.42E-10 | NA | + | NA | NA | 1.45E-01 | 7 | -137.682 | 289.364 | 1.717 | 0.187 |
| -0.332 | NA | NA | + | NA | -1.25E-02 | 1.41E-01 | 7 | -137.806 | 289.613 | 1.966 | 0.165 |
| -0.715 | NA | NA | + | -6.68E-04 | NA | 2.13E-01 | 7 | -152.571 | 319.142 | 0 | 0.215 |
| -0.576 | NA | NA | + | NA | NA | 2.01E-01 | 6 | -153.64 | 319.28 | 0.137 | 0.2 |
| -0.564 | NA | NA | + | -7.47E-04 | -7.57E-02 | 2.16E-01 | 8 | -151.93 | 319.859 | 0.717 | 0.15 |
| -0.773 | 1.68E-09 | NA | + | -7.13E-04 | NA | 2.23E-01 | 8 | -152.06 | 320.119 | 0.977 | 0.132 |
| -0.445 | NA | NA | + | NA | -5.91E-02 | 2.01E-01 | 7 | -153.245 | 320.491 | 1.348 | 0.109 |
| -0.618 | 1.43E-09 | NA | + | NA | NA | 2.08E-01 | 7 | -153.271 | 320.543 | 1.4 | 0.107 |
| -0.625 | 1.58E-09 | NA | + | -7.86E-04 | -7.24E-02 | 2.24E-01 | 9 | -151.47 | 320.94 | 1.798 | 0.087 |
| -0.518 | NA | NA | + | -8.24E-04 | -9.84E-02 | 2.12E-01 | 8 | -140.669 | 297.338 | 0 | 0.287 |
| -0.713 | NA | NA | + | -7.39E-04 | NA | 2.08E-01 | 7 | -141.951 | 297.902 | 0.563 | 0.217 |
| -0.565 | 1.28E-09 | NA | + | -8.41E-04 | -9.48E-02 | 2.18E-01 | 9 | -140.363 | 298.726 | 1.388 | 0.144 |
| -0.552 | NA | NA | + | NA | NA | 1.94E-01 | 6 | -143.525 | 299.05 | 1.712 | 0.122 |
| -0.759 | 1.47E-09 | NA | + | -7.62E-04 | NA | 2.15E-01 | 8 | -141.551 | 299.103 | 1.764 | 0.119 |
| -0.373 | NA | NA | + | NA | -8.29E-02 | 1.95E-01 | 7 | -142.622 | 299.245 | 1.907 | 0.111 |
| -0.556 | NA | NA | + | NA | NA | 1.97E-01 | 6 | -164.688 | 341.377 | 0 | 0.429 |
| -0.596 | 1.32E-09 | NA | + | NA | NA | 2.04E-01 | 7 | -164.4 | 342.799 | 1.423 | 0.211 |
| -0.471 | NA | NA | + | NA | -3.72E-02 | 1.96E-01 | 7 | -164.552 | 343.103 | 1.727 | 0.181 |
| -0.609 | NA | NA | + | -2.40E-04 | NA | 2.02E-01 | 7 | -164.566 | 343.133 | 1.756 | 0.178 |
| -0.574 | NA | NA | + | NA | NA | 1.99E-01 | 6 | -162.864 | 337.727 | 0 | 0.379 |
| -0.668 | NA | NA | + | -4.66E-04 | NA | 2.07E-01 | 7 | -162.369 | 338.739 | 1.012 | 0.229 |
| -0.442 | NA | NA | + | NA | -5.83E-02 | 1.99E-01 | 7 | -162.507 | 339.015 | 1.287 | 0.199 |
| -0.616 | 1.40E-09 | NA | + | NA | NA | 2.08E-01 | 7 | -162.543 | 339.086 | 1.359 | 0.192 |
| -0.521 | NA | NA | + | NA | NA | 1.80E-01 | 6 | -166.672 | 345.344 | 0 | 0.455 |
| -0.555 | 1.14E-09 | NA | + | NA | NA | 1.86E-01 | 7 | -166.474 | 346.948 | 1.604 | 0.204 |
| -0.551 | NA | NA | + | -1.35E-04 | NA | 1.83E-01 | 7 | -166.636 | 347.271 | 1.927 | 0.174 |
| -0.512 | NA | NA | + | NA | -4.12E-03 | 1.80E-01 | 7 | -166.671 | 347.341 | 1.997 | 0.168 |
| -0.505 | NA | NA | + | NA | NA | 1.78E-01 | 6 | -155.365 | 322.731 | 0 | 0.382 |
| -0.61 | NA | NA | + | -4.66E-04 | NA | 1.88E-01 | 7 | -154.861 | 323.723 | 0.992 | 0.233 |
| -0.384 | NA | NA | + | NA | -5.39E-02 | 1.78E-01 | 7 | -155.043 | 324.086 | 1.355 | 0.194 |
| -0.546 | 1.41E-09 | NA | + | NA | NA | 1.84E-01 | 7 | -155.06 | 324.12 | 1.389 | 0.191 |
| -0.535 | NA | NA | + | NA | NA | 1.87E-01 | 6 | -152.632 | 317.265 | 0 | 0.262 |
| -0.645 | NA | NA | + | -5.53E-04 | NA | 1.96E-01 | 7 | -151.864 | 317.729 | 0.464 | 0.208 |
| -0.41 | NA | NA | + | NA | -5.74E-02 | 1.88E-01 | 7 | -152.255 | 318.51 | 1.246 | 0.141 |
| -0.575 | 1.15E-09 | NA | + | NA | NA | 1.96E-01 | 7 | -152.306 | 318.612 | 1.348 | 0.134 |
| -0.506 | NA | NA | + | -6.18E-04 | -7.00E-02 | 1.99E-01 | 8 | -151.308 | 318.616 | 1.352 | 0.133 |
| -0.702 | 1.37E-09 | NA | + | -6.02E-04 | NA | 2.07E-01 | 8 | -151.401 | 318.802 | 1.537 | 0.122 |
| -0.496 | NA | NA | + | NA | NA | 1.73E-01 | 6 | -162.117 | 336.234 | 0 | 0.432 |
| -0.533 | 1.36E-09 | NA | + | NA | NA | 1.79E-01 | 7 | -161.894 | 337.789 | 1.555 | 0.198 |
| -0.569 | NA | NA | + | -3.14E-04 | NA | 1.80E-01 | 7 | -161.907 | 337.814 | 1.58 | 0.196 |
| -0.43 | NA | NA | + | NA | -3.01E-02 | 1.74E-01 | 7 | -162.025 | 338.049 | 1.815 | 0.174 |
| -0.517 | NA | NA | + | -9.85E-04 | -1.28E-01 | 2.31E-01 | 8 | -141.426 | 298.853 | 0 | 0.622 |
| -0.577 | 1.50E-09 | NA | + | -1.00E-03 | -1.24E-01 | 2.40E-01 | 9 | -140.926 | 299.851 | 0.998 | 0.378 |
| -0.621 | NA | NA | + | NA | NA | 2.06E-01 | 6 | -159.55 | 331.1 | 0 | 0.217 |
| -0.751 | NA | NA | + | -6.28E-04 | NA | 2.16E-01 | 7 | -158.629 | 331.257 | 0.158 | 0.2 |
| -0.597 | NA | NA | + | -7.09E-04 | -7.47E-02 | 2.18E-01 | 8 | -157.995 | 331.989 | 0.89 | 0.139 |
| -0.811 | 1.71E-09 | NA | + | -6.67E-04 | NA | 2.26E-01 | 8 | -158.105 | 332.21 | 1.111 | 0.124 |
| -0.668 | 1.51E-09 | NA | + | NA | NA | 2.15E-01 | 7 | -159.143 | 332.286 | 1.187 | 0.12 |
| -0.486 | NA | NA | + | NA | -5.92E-02 | 2.07E-01 | 7 | -159.147 | 332.295 | 1.195 | 0.119 |
| -0.661 | 1.60E-09 | NA | + | -7.41E-04 | -7.08E-02 | 2.28E-01 | 9 | -157.534 | 333.069 | 1.969 | 0.081 |
| -0.406 | NA | NA | + | NA | NA | 1.55E-01 | 6 | -154.092 | 320.183 | 0 | 0.387 |
| -0.436 | 9.66E-10 | NA | + | NA | NA | 1.60E-01 | 7 | -153.907 | 321.815 | 1.632 | 0.171 |
| -0.436 | NA | NA | + | -1.27E-04 | NA | 1.58E-01 | 7 | -154.056 | 322.112 | 1.929 | 0.148 |
| -0.345 | NA | NA | NA | NA | NA | 1.94E-01 | 3 | -158.058 | 322.116 | 1.933 | 0.147 |
| -0.37 | NA | NA | + | NA | -1.62E-02 | 1.55E-01 | 7 | -154.066 | 322.132 | 1.949 | 0.146 |
| -0.46 | NA | NA | + | NA | NA | 1.68E-01 | 6 | -150.375 | 312.75 | 0 | 0.418 |
| -0.54 | NA | NA | + | -3.69E-04 | NA | 1.75E-01 | 7 | -150.064 | 314.128 | 1.378 | 0.21 |
| -0.497 | 1.18E-09 | NA | + | NA | NA | 1.75E-01 | 7 | -150.1 | 314.2 | 1.45 | 0.203 |
| -0.396 | NA | NA | + | NA | -2.92E-02 | 1.69E-01 | 7 | -150.283 | 314.566 | 1.816 | 0.169 |
| -0.659 | NA | NA | + | -9.55E-04 | -1.04E-01 | 2.55E-01 | 8 | -156.259 | 328.517 | 0 | 0.342 |
| -0.87 | NA | NA | + | -8.26E-04 | NA | 2.56E-01 | 7 | -157.603 | 329.206 | 0.689 | 0.242 |
| -0.73 | 1.78E-09 | NA | + | -9.93E-04 | -9.92E-02 | 2.65E-01 | 9 | -155.655 | 329.311 | 0.794 | 0.23 |
| -0.936 | 1.98E-09 | NA | + | -8.75E-04 | NA | 2.66E-01 | 8 | -156.871 | 329.743 | 1.226 | 0.185 |
| -0.382 | NA | NA | + | NA | NA | 1.47E-01 | 6 | -146.678 | 305.355 | 0 | 0.377 |
| -0.413 | 7.64E-10 | NA | + | NA | NA | 1.54E-01 | 7 | -146.429 | 306.858 | 1.502 | 0.178 |
| -0.302 | NA | NA | NA | NA | NA | 1.79E-01 | 3 | -150.575 | 307.15 | 1.795 | 0.154 |
| -0.415 | NA | NA | + | -1.57E-04 | NA | 1.48E-01 | 7 | -146.619 | 307.238 | 1.883 | 0.147 |
| -0.339 | NA | NA | + | NA | -2.01E-02 | 1.48E-01 | 7 | -146.635 | 307.269 | 1.914 | 0.145 |
| -0.535 | NA | NA | + | NA | NA | 1.82E-01 | 6 | -152.617 | 317.235 | 0 | 0.244 |
| -0.676 | NA | NA | + | -6.20E-04 | NA | 1.96E-01 | 7 | -151.666 | 317.331 | 0.097 | 0.232 |
| -0.531 | NA | NA | + | -6.83E-04 | -7.12E-02 | 1.98E-01 | 8 | -151.088 | 318.176 | 0.942 | 0.152 |
| -0.405 | NA | NA | + | NA | -5.81E-02 | 1.83E-01 | 7 | -152.232 | 318.464 | 1.229 | 0.132 |
| -0.723 | 1.67E-09 | NA | + | -6.40E-04 | NA | 2.03E-01 | 8 | -151.32 | 318.64 | 1.405 | 0.121 |
| -0.573 | 1.51E-09 | NA | + | NA | NA | 1.88E-01 | 7 | -152.335 | 318.671 | 1.436 | 0.119 |
| -0.47 | NA | NA | + | NA | NA | 1.67E-01 | 6 | -152.887 | 317.774 | 0 | 0.431 |
| -0.549 | NA | NA | + | -3.35E-04 | NA | 1.74E-01 | 7 | -152.637 | 319.274 | 1.499 | 0.204 |
| -0.505 | 1.37E-09 | NA | + | NA | NA | 1.71E-01 | 7 | -152.675 | 319.35 | 1.576 | 0.196 |
| -0.415 | NA | NA | + | NA | -2.50E-02 | 1.68E-01 | 7 | -152.822 | 319.643 | 1.869 | 0.169 |
| -0.597 | NA | NA | + | -8.33E-04 | -1.16E-01 | 2.43E-01 | 8 | -159.968 | 335.936 | 0 | 0.283 |
| -0.669 | 1.92E-09 | NA | + | -8.54E-04 | -1.09E-01 | 2.52E-01 | 9 | -159.433 | 336.865 | 0.929 | 0.178 |
| -0.488 | NA | NA | + | NA | -9.66E-02 | 2.31E-01 | 7 | -161.609 | 337.217 | 1.281 | 0.149 |
| -0.837 | NA | NA | + | -6.92E-04 | NA | 2.43E-01 | 7 | -161.615 | 337.231 | 1.294 | 0.148 |
| -0.71 | NA | NA | + | NA | NA | 2.33E-01 | 6 | -162.759 | 337.518 | 1.581 | 0.128 |
| -0.905 | 2.24E-09 | NA | + | -7.26E-04 | NA | 2.54E-01 | 8 | -160.89 | 337.781 | 1.844 | 0.113 |
| -0.518 | NA | NA | + | NA | NA | 1.87E-01 | 6 | -150.988 | 313.976 | 0 | 0.388 |
| -0.607 | NA | NA | + | -4.24E-04 | NA | 1.95E-01 | 7 | -150.552 | 315.104 | 1.128 | 0.221 |
| -0.557 | 1.47E-09 | NA | + | NA | NA | 1.93E-01 | 7 | -150.665 | 315.331 | 1.354 | 0.197 |
| -0.409 | NA | NA | + | NA | -5.15E-02 | 1.90E-01 | 7 | -150.687 | 315.375 | 1.398 | 0.193 |
| -0.507 | NA | NA | + | NA | NA | 1.80E-01 | 6 | -154.082 | 320.163 | 0 | 0.331 |
| -0.634 | NA | NA | + | -5.52E-04 | NA | 1.92E-01 | 7 | -153.356 | 320.712 | 0.549 | 0.251 |
| -0.539 | 1.11E-09 | NA | + | NA | NA | 1.86E-01 | 7 | -153.859 | 321.718 | 1.555 | 0.152 |
| -0.425 | NA | NA | + | NA | -3.88E-02 | 1.82E-01 | 7 | -153.926 | 321.852 | 1.689 | 0.142 |
| -0.678 | 1.26E-09 | NA | + | -5.78E-04 | NA | 1.99E-01 | 8 | -153.065 | 322.13 | 1.967 | 0.124 |
| -0.514 | NA | NA | + | NA | NA | 1.84E-01 | 6 | -161.864 | 335.728 | 0 | 0.436 |
| -0.549 | 1.16E-09 | NA | + | NA | NA | 1.90E-01 | 7 | -161.601 | 337.202 | 1.473 | 0.209 |
| -0.561 | NA | NA | + | -2.26E-04 | NA | 1.87E-01 | 7 | -161.755 | 337.51 | 1.782 | 0.179 |
| -0.449 | NA | NA | + | NA | -3.04E-02 | 1.85E-01 | 7 | -161.77 | 337.54 | 1.812 | 0.176 |
| -0.502 | NA | NA | + | NA | NA | 1.76E-01 | 6 | -153.514 | 319.027 | 0 | 0.388 |
| -0.612 | NA | NA | + | -4.74E-04 | NA | 1.86E-01 | 7 | -152.999 | 319.999 | 0.971 | 0.239 |
| -0.392 | NA | NA | + | NA | -4.93E-02 | 1.77E-01 | 7 | -153.245 | 320.49 | 1.463 | 0.187 |
| -0.541 | 1.34E-09 | NA | + | NA | NA | 1.82E-01 | 7 | -153.251 | 320.502 | 1.474 | 0.186 |
| -0.528 | NA | NA | + | NA | NA | 1.84E-01 | 6 | -163.614 | 339.228 | 0 | 0.433 |
| -0.6 | NA | NA | + | -3.47E-04 | NA | 1.89E-01 | 7 | -163.365 | 340.73 | 1.501 | 0.205 |
| -0.563 | 1.12E-09 | NA | + | NA | NA | 1.91E-01 | 7 | -163.386 | 340.771 | 1.543 | 0.2 |
| -0.5 | NA | NA | + | NA | -1.25E-02 | 1.84E-01 | 7 | -163.599 | 341.199 | 1.97 | 0.162 |
| -0.33 | NA | NA | + | NA | NA | 1.36E-01 | 6 | -144.26 | 300.52 | 0 | 0.247 |
| -0.283 | NA | NA | NA | NA | NA | 1.71E-01 | 3 | -147.387 | 300.775 | 0.255 | 0.218 |
| -0.202 | NA | NA | NA | 4.30E-04 | NA | 1.50E-01 | 4 | -146.7 | 301.399 | 0.88 | 0.159 |
| -0.353 | 8.10E-10 | NA | + | NA | NA | 1.40E-01 | 7 | -144.141 | 302.282 | 1.762 | 0.102 |
| -0.308 | 8.71E-10 | NA | NA | NA | NA | 1.75E-01 | 4 | -147.255 | 302.51 | 1.991 | 0.091 |
| -0.337 | NA | NA | + | -2.88E-05 | NA | 1.37E-01 | 7 | -144.258 | 302.516 | 1.996 | 0.091 |
| -0.339 | NA | NA | + | NA | 3.87E-03 | 1.36E-01 | 7 | -144.258 | 302.517 | 1.997 | 0.091 |
| -0.365 | NA | NA | + | NA | NA | 1.43E-01 | 6 | -142.832 | 297.664 | 0 | 0.377 |
| -0.445 | NA | NA | + | -3.24E-04 | NA | 1.51E-01 | 7 | -142.577 | 299.155 | 1.491 | 0.179 |
| -0.39 | 8.55E-10 | NA | + | NA | NA | 1.48E-01 | 7 | -142.687 | 299.374 | 1.71 | 0.16 |
| -0.33 | NA | NA | + | NA | -1.62E-02 | 1.44E-01 | 7 | -142.804 | 299.608 | 1.945 | 0.143 |
| -0.303 | NA | NA | NA | NA | NA | 1.81E-01 | 3 | -146.812 | 299.625 | 1.961 | 0.141 |
| -0.433 | NA | NA | + | NA | NA | 1.57E-01 | 6 | -152.421 | 316.842 | 0 | 0.447 |
| -0.465 | 1.08E-09 | NA | + | NA | NA | 1.62E-01 | 7 | -152.24 | 318.479 | 1.637 | 0.197 |
| -0.493 | NA | NA | + | -2.59E-04 | NA | 1.62E-01 | 7 | -152.274 | 318.547 | 1.705 | 0.191 |
| -0.414 | NA | NA | + | NA | -8.86E-03 | 1.57E-01 | 7 | -152.413 | 318.827 | 1.984 | 0.166 |
| -0.425 | NA | NA | + | NA | NA | 1.58E-01 | 6 | -137.925 | 287.849 | 0 | 0.318 |
| -0.535 | NA | NA | + | -4.80E-04 | NA | 1.67E-01 | 7 | -137.3 | 288.601 | 0.752 | 0.218 |
| -0.307 | NA | NA | + | NA | -5.50E-02 | 1.60E-01 | 7 | -137.544 | 289.088 | 1.239 | 0.171 |
| -0.459 | 1.20E-09 | NA | + | NA | NA | 1.63E-01 | 7 | -137.631 | 289.263 | 1.413 | 0.157 |
| -0.409 | NA | NA | + | -5.34E-04 | -6.44E-02 | 1.71E-01 | 8 | -136.78 | 289.561 | 1.712 | 0.135 |
| -0.498 | NA | NA | + | NA | NA | 1.72E-01 | 6 | -150.805 | 313.61 | 0 | 0.328 |
| -0.617 | NA | NA | + | -5.24E-04 | NA | 1.84E-01 | 7 | -150.139 | 314.279 | 0.669 | 0.235 |
| -0.388 | NA | NA | + | NA | -4.91E-02 | 1.73E-01 | 7 | -150.549 | 315.098 | 1.488 | 0.156 |
| -0.533 | 1.22E-09 | NA | + | NA | NA | 1.78E-01 | 7 | -150.55 | 315.101 | 1.49 | 0.156 |
| -0.494 | NA | NA | + | -5.69E-04 | -5.90E-02 | 1.86E-01 | 8 | -149.772 | 315.543 | 1.933 | 0.125 |
| -0.399 | NA | NA | + | NA | NA | 1.51E-01 | 6 | -162.568 | 337.137 | 0 | 0.306 |
| -0.323 | NA | NA | NA | NA | NA | 1.88E-01 | 3 | -166.099 | 338.199 | 1.062 | 0.18 |
| -0.229 | NA | NA | NA | 5.19E-04 | NA | 1.63E-01 | 4 | -165.251 | 338.502 | 1.366 | 0.154 |
| -0.429 | 1.04E-09 | NA | + | NA | NA | 1.56E-01 | 7 | -162.412 | 338.824 | 1.688 | 0.132 |
| -0.433 | NA | NA | + | NA | 1.53E-02 | 1.51E-01 | 7 | -162.548 | 339.096 | 1.959 | 0.115 |
| -0.384 | NA | NA | + | 6.71E-05 | NA | 1.50E-01 | 7 | -162.559 | 339.119 | 1.982 | 0.114 |
| -0.378 | NA | NA | + | NA | NA | 1.47E-01 | 6 | -148.327 | 308.655 | 0 | 0.297 |
| -0.32 | NA | NA | NA | NA | NA | 1.86E-01 | 3 | -151.675 | 309.35 | 0.695 | 0.21 |
| -0.242 | NA | NA | NA | 4.05E-04 | NA | 1.65E-01 | 4 | -151.063 | 310.127 | 1.472 | 0.142 |
| -0.405 | 9.73E-10 | NA | + | NA | NA | 1.52E-01 | 7 | -148.165 | 310.33 | 1.675 | 0.128 |
| -0.409 | NA | NA | + | -1.32E-04 | NA | 1.50E-01 | 7 | -148.29 | 310.579 | 1.924 | 0.113 |
| -0.383 | NA | NA | + | NA | 2.47E-03 | 1.47E-01 | 7 | -148.327 | 310.654 | 1.999 | 0.109 |
| -0.34 | NA | NA | + | NA | NA | 1.35E-01 | 6 | -139.859 | 291.717 | 0 | 0.278 |
| -0.268 | NA | NA | NA | NA | NA | 1.66E-01 | 3 | -143.021 | 292.041 | 0.324 | 0.236 |
| -0.192 | NA | NA | NA | 4.01E-04 | NA | 1.47E-01 | 4 | -142.391 | 292.781 | 1.064 | 0.163 |
| -0.364 | 6.89E-10 | NA | + | NA | NA | 1.40E-01 | 7 | -139.723 | 293.446 | 1.729 | 0.117 |
| -0.352 | NA | NA | + | -4.72E-05 | NA | 1.37E-01 | 7 | -139.853 | 293.707 | 1.989 | 0.103 |
| -0.346 | NA | NA | + | NA | 2.91E-03 | 1.35E-01 | 7 | -139.858 | 293.716 | 1.998 | 0.102 |
| -0.506 | NA | NA | + | NA | NA | 1.78E-01 | 6 | -154.842 | 321.685 | 0 | 0.39 |
| -0.618 | NA | NA | + | -5.24E-04 | NA | 1.87E-01 | 7 | -154.201 | 322.402 | 0.717 | 0.272 |
| -0.54 | 1.11E-09 | NA | + | NA | NA | 1.84E-01 | 7 | -154.625 | 323.249 | 1.565 | 0.178 |
| -0.434 | NA | NA | + | NA | -3.23E-02 | 1.78E-01 | 7 | -154.732 | 323.464 | 1.78 | 0.16 |
| -0.497 | NA | NA | + | NA | NA | 1.75E-01 | 6 | -148.466 | 308.932 | 0 | 0.395 |
| -0.594 | NA | NA | + | -4.51E-04 | NA | 1.84E-01 | 7 | -147.95 | 309.901 | 0.969 | 0.243 |
| -0.531 | 9.76E-10 | NA | + | NA | NA | 1.82E-01 | 7 | -148.217 | 310.434 | 1.502 | 0.186 |
| -0.409 | NA | NA | + | NA | -4.18E-02 | 1.78E-01 | 7 | -148.276 | 310.551 | 1.619 | 0.176 |
| -0.772 | NA | NA | + | -7.61E-04 | NA | 2.19E-01 | 7 | -148.219 | 310.438 | 0 | 0.238 |
| -0.593 | NA | NA | + | -8.57E-04 | -8.90E-02 | 2.21E-01 | 8 | -147.255 | 310.509 | 0.071 | 0.23 |
| -0.832 | 1.76E-09 | NA | + | -7.90E-04 | NA | 2.29E-01 | 8 | -147.64 | 311.281 | 0.843 | 0.156 |
| -0.622 | NA | NA | + | NA | NA | 2.08E-01 | 6 | -149.732 | 311.464 | 1.026 | 0.143 |
| -0.656 | 1.61E-09 | NA | + | -8.79E-04 | -8.46E-02 | 2.29E-01 | 9 | -146.767 | 311.534 | 1.096 | 0.138 |
| -0.467 | NA | NA | + | NA | -6.92E-02 | 2.08E-01 | 7 | -149.147 | 312.294 | 1.856 | 0.094 |
| -0.56 | NA | NA | + | NA | NA | 1.95E-01 | 6 | -160.341 | 332.681 | 0 | 0.391 |
| -0.644 | NA | NA | + | -4.13E-04 | NA | 2.02E-01 | 7 | -159.964 | 333.927 | 1.246 | 0.21 |
| -0.605 | 1.66E-09 | NA | + | NA | NA | 2.02E-01 | 7 | -159.968 | 333.935 | 1.254 | 0.209 |
| -0.448 | NA | NA | + | NA | -5.10E-02 | 1.96E-01 | 7 | -160.059 | 334.118 | 1.437 | 0.191 |
| -0.397 | NA | NA | + | NA | NA | 1.47E-01 | 6 | -156.777 | 325.554 | 0 | 0.309 |
| -0.309 | NA | NA | NA | NA | NA | 1.83E-01 | 3 | -160.274 | 326.548 | 0.993 | 0.188 |
| -0.224 | NA | NA | NA | 4.68E-04 | NA | 1.60E-01 | 4 | -159.565 | 327.13 | 1.575 | 0.141 |
| -0.425 | 9.53E-10 | NA | + | NA | NA | 1.52E-01 | 7 | -156.63 | 327.26 | 1.706 | 0.132 |
| -0.431 | NA | NA | + | NA | 1.51E-02 | 1.46E-01 | 7 | -156.757 | 327.513 | 1.959 | 0.116 |
| -0.399 | NA | NA | + | -8.93E-06 | NA | 1.47E-01 | 7 | -156.777 | 327.554 | 2 | 0.114 |
| -0.407 | NA | NA | + | NA | NA | 1.57E-01 | 6 | -141.477 | 294.953 | 0 | 0.396 |
| -0.521 | NA | NA | + | -4.80E-04 | NA | 1.66E-01 | 7 | -140.885 | 295.771 | 0.818 | 0.263 |
| -0.437 | 1.07E-09 | NA | + | NA | NA | 1.61E-01 | 7 | -141.274 | 296.548 | 1.595 | 0.178 |
| -0.339 | NA | NA | + | NA | -3.15E-02 | 1.58E-01 | 7 | -141.365 | 296.729 | 1.776 | 0.163 |
| -0.583 | NA | NA | + | -8.56E-04 | -1.05E-01 | 2.41E-01 | 8 | -154.295 | 324.589 | 0 | 0.278 |
| -0.796 | NA | NA | + | -7.25E-04 | NA | 2.40E-01 | 7 | -155.669 | 325.338 | 0.748 | 0.192 |
| -0.64 | 1.75E-09 | NA | + | -8.65E-04 | -1.00E-01 | 2.48E-01 | 9 | -153.887 | 325.775 | 1.185 | 0.154 |
| -0.648 | NA | NA | + | NA | NA | 2.26E-01 | 6 | -157.01 | 326.019 | 1.43 | 0.136 |
| -0.458 | NA | NA | + | NA | -8.32E-02 | 2.24E-01 | 7 | -156.132 | 326.264 | 1.675 | 0.12 |
| -0.851 | 2.00E-09 | NA | + | -7.42E-04 | NA | 2.48E-01 | 8 | -155.14 | 326.28 | 1.69 | 0.12 |
| -0.841 | NA | NA | + | -6.74E-04 | NA | 2.41E-01 | 7 | -163.609 | 341.217 | 0 | 0.183 |
| -0.656 | NA | NA | + | -7.81E-04 | -9.21E-02 | 2.43E-01 | 8 | -162.62 | 341.24 | 0.022 | 0.181 |
| -0.711 | NA | NA | + | NA | NA | 2.32E-01 | 6 | -164.655 | 341.31 | 0.093 | 0.175 |
| -0.545 | NA | NA | + | NA | -7.44E-02 | 2.33E-01 | 7 | -164.003 | 342.006 | 0.788 | 0.124 |
| -0.906 | 2.06E-09 | NA | + | -7.10E-04 | NA | 2.51E-01 | 8 | -163.003 | 342.006 | 0.789 | 0.124 |
| -0.726 | 1.83E-09 | NA | + | -8.06E-04 | -8.60E-02 | 2.52E-01 | 9 | -162.144 | 342.287 | 1.07 | 0.107 |
| -0.763 | 1.86E-09 | NA | + | NA | NA | 2.41E-01 | 7 | -164.167 | 342.334 | 1.117 | 0.105 |
| -0.562 | NA | NA | + | NA | NA | 1.87E-01 | 6 | -159.35 | 330.699 | 0 | 0.427 |
| -0.639 | NA | NA | + | -3.50E-04 | NA | 1.94E-01 | 7 | -159.083 | 332.166 | 1.467 | 0.205 |
| -0.599 | 1.12E-09 | NA | + | NA | NA | 1.94E-01 | 7 | -159.096 | 332.192 | 1.492 | 0.202 |
| -0.508 | NA | NA | + | NA | -2.41E-02 | 1.88E-01 | 7 | -159.293 | 332.586 | 1.887 | 0.166 |
| -0.43 | NA | NA | + | NA | NA | 1.68E-01 | 6 | -150.159 | 312.318 | 0 | 0.368 |
| -0.374 | NA | NA | NA | NA | NA | 2.02E-01 | 3 | -153.893 | 313.786 | 1.468 | 0.177 |
| -0.463 | 1.22E-09 | NA | + | NA | NA | 1.73E-01 | 7 | -149.933 | 313.866 | 1.548 | 0.17 |
| -0.469 | NA | NA | + | -1.82E-04 | NA | 1.71E-01 | 7 | -150.085 | 314.171 | 1.853 | 0.146 |
| -0.397 | NA | NA | + | NA | -1.60E-02 | 1.69E-01 | 7 | -150.132 | 314.264 | 1.946 | 0.139 |
| -0.633 | NA | NA | + | -1.10E-03 | -1.23E-01 | 2.46E-01 | 8 | -155.631 | 327.263 | 0 | 0.487 |
| -0.701 | 1.69E-09 | NA | + | -1.14E-03 | -1.18E-01 | 2.56E-01 | 9 | -155.027 | 328.054 | 0.791 | 0.328 |
| -0.878 | NA | NA | + | -9.52E-04 | NA | 2.43E-01 | 7 | -157.594 | 329.187 | 1.924 | 0.186 |
| -0.351 | NA | NA | + | NA | NA | 1.45E-01 | 6 | -133.593 | 279.185 | 0 | 0.358 |
| -0.303 | NA | NA | NA | NA | NA | 1.78E-01 | 3 | -137.249 | 280.499 | 1.313 | 0.186 |
| -0.42 | NA | NA | + | -2.88E-04 | NA | 1.51E-01 | 7 | -133.375 | 280.751 | 1.565 | 0.164 |
| -0.372 | 7.50E-10 | NA | + | NA | NA | 1.49E-01 | 7 | -133.469 | 280.938 | 1.752 | 0.149 |
| -0.297 | NA | NA | + | NA | -2.57E-02 | 1.47E-01 | 7 | -133.515 | 281.03 | 1.844 | 0.143 |
| -0.673 | NA | NA | + | NA | NA | 2.17E-01 | 6 | -164.88 | 341.761 | 0 | 0.252 |
| -0.803 | NA | NA | + | -6.45E-04 | NA | 2.27E-01 | 7 | -163.945 | 341.891 | 0.13 | 0.236 |
| -0.86 | 1.70E-09 | NA | + | -6.75E-04 | NA | 2.37E-01 | 8 | -163.509 | 343.017 | 1.257 | 0.134 |
| -0.719 | 1.52E-09 | NA | + | NA | NA | 2.25E-01 | 7 | -164.535 | 343.07 | 1.31 | 0.131 |
| -0.669 | NA | NA | + | -7.08E-04 | -6.40E-02 | 2.28E-01 | 8 | -163.542 | 343.083 | 1.323 | 0.13 |
| -0.563 | NA | NA | + | NA | -4.83E-02 | 2.17E-01 | 7 | -164.648 | 343.296 | 1.536 | 0.117 |
| -0.779 | NA | NA | + | -6.93E-04 | NA | 2.32E-01 | 7 | -151.882 | 317.763 | 0 | 0.21 |
| -0.603 | NA | NA | + | -7.87E-04 | -8.75E-02 | 2.34E-01 | 8 | -150.939 | 317.878 | 0.115 | 0.198 |
| -0.643 | NA | NA | + | NA | NA | 2.20E-01 | 6 | -153.152 | 318.305 | 0.541 | 0.16 |
| -0.835 | 1.61E-09 | NA | + | -7.26E-04 | NA | 2.42E-01 | 8 | -151.381 | 318.763 | 0.999 | 0.128 |
| -0.662 | 1.46E-09 | NA | + | -8.13E-04 | -8.34E-02 | 2.43E-01 | 9 | -150.525 | 319.05 | 1.287 | 0.11 |
| -0.489 | NA | NA | + | NA | -6.96E-02 | 2.20E-01 | 7 | -152.553 | 319.106 | 1.342 | 0.107 |
| -0.687 | 1.40E-09 | NA | + | NA | NA | 2.28E-01 | 7 | -152.776 | 319.553 | 1.789 | 0.086 |
| -0.544 | NA | NA | + | NA | NA | 1.92E-01 | 6 | -156.87 | 325.739 | 0 | 0.392 |
| -0.631 | NA | NA | + | -4.17E-04 | NA | 2.00E-01 | 7 | -156.466 | 326.932 | 1.192 | 0.216 |
| -0.423 | NA | NA | + | NA | -5.40E-02 | 1.92E-01 | 7 | -156.551 | 327.101 | 1.362 | 0.198 |
| -0.583 | 1.25E-09 | NA | + | NA | NA | 1.99E-01 | 7 | -156.569 | 327.138 | 1.399 | 0.195 |
| -0.727 | NA | NA | + | NA | NA | 2.34E-01 | 6 | -166.115 | 344.23 | 0 | 0.179 |
| -0.852 | NA | NA | + | -6.26E-04 | NA | 2.45E-01 | 7 | -165.187 | 344.375 | 0.145 | 0.166 |
| -0.653 | NA | NA | + | -7.39E-04 | -9.32E-02 | 2.44E-01 | 8 | -164.22 | 344.44 | 0.21 | 0.161 |
| -0.549 | NA | NA | + | NA | -7.45E-02 | 2.32E-01 | 7 | -165.486 | 344.972 | 0.742 | 0.123 |
| -0.782 | 1.99E-09 | NA | + | NA | NA | 2.43E-01 | 7 | -165.628 | 345.256 | 1.026 | 0.107 |
| -0.915 | 2.11E-09 | NA | + | -6.47E-04 | NA | 2.55E-01 | 8 | -164.633 | 345.267 | 1.037 | 0.106 |
| -0.721 | 1.86E-09 | NA | + | -7.50E-04 | -8.72E-02 | 2.53E-01 | 9 | -163.789 | 345.578 | 1.348 | 0.091 |
| -0.613 | 1.77E-09 | NA | + | NA | -6.85E-02 | 2.40E-01 | 8 | -165.099 | 346.198 | 1.968 | 0.067 |
| -0.461 | NA | NA | + | NA | NA | 1.66E-01 | 6 | -161.758 | 335.517 | 0 | 0.445 |
| -0.496 | 1.22E-09 | NA | + | NA | NA | 1.71E-01 | 7 | -161.556 | 337.112 | 1.595 | 0.2 |
| -0.512 | NA | NA | + | -2.14E-04 | NA | 1.71E-01 | 7 | -161.664 | 337.329 | 1.812 | 0.18 |
| -0.399 | NA | NA | + | NA | -2.78E-02 | 1.66E-01 | 7 | -161.687 | 337.374 | 1.857 | 0.176 |
| -0.552 | NA | NA | + | NA | NA | 1.86E-01 | 6 | -162.52 | 337.039 | 0 | 0.412 |
| -0.645 | NA | NA | + | -4.58E-04 | NA | 1.93E-01 | 7 | -162.072 | 338.144 | 1.105 | 0.237 |
| -0.591 | 1.38E-09 | NA | + | NA | NA | 1.93E-01 | 7 | -162.257 | 338.514 | 1.475 | 0.197 |
| -0.518 | NA | NA | + | NA | -1.51E-02 | 1.86E-01 | 7 | -162.498 | 338.996 | 1.957 | 0.155 |
| -0.364 | NA | NA | + | NA | NA | 1.44E-01 | 6 | -142.444 | 296.889 | 0 | 0.373 |
| -0.291 | NA | NA | NA | NA | NA | 1.76E-01 | 3 | -146.226 | 298.452 | 1.564 | 0.171 |
| -0.423 | NA | NA | + | -2.54E-04 | NA | 1.49E-01 | 7 | -142.288 | 298.575 | 1.686 | 0.16 |
| -0.387 | 7.66E-10 | NA | + | NA | NA | 1.48E-01 | 7 | -142.31 | 298.62 | 1.732 | 0.157 |
| -0.34 | NA | NA | + | NA | -1.12E-02 | 1.45E-01 | 7 | -142.431 | 298.863 | 1.974 | 0.139 |
| -0.376 | NA | NA | + | NA | NA | 1.44E-01 | 6 | -150.657 | 313.314 | 0 | 0.31 |
| -0.294 | NA | NA | NA | NA | NA | 1.78E-01 | 3 | -154.197 | 314.394 | 1.08 | 0.181 |
| -0.206 | NA | NA | NA | 4.61E-04 | NA | 1.54E-01 | 4 | -153.412 | 314.825 | 1.511 | 0.146 |
| -0.405 | 9.92E-10 | NA | + | NA | NA | 1.49E-01 | 7 | -150.496 | 314.992 | 1.678 | 0.134 |
| -0.384 | NA | NA | + | NA | 3.54E-03 | 1.44E-01 | 7 | -150.656 | 315.311 | 1.998 | 0.114 |
| -0.38 | NA | NA | + | -1.63E-05 | NA | 1.44E-01 | 7 | -150.656 | 315.312 | 1.999 | 0.114 |
| -0.51 | NA | NA | + | NA | NA | 1.79E-01 | 6 | -152.66 | 317.32 | 0 | 0.413 |
| -0.597 | NA | NA | + | -3.98E-04 | NA | 1.86E-01 | 7 | -152.288 | 318.576 | 1.257 | 0.22 |
| -0.547 | 1.14E-09 | NA | + | NA | NA | 1.86E-01 | 7 | -152.386 | 318.772 | 1.453 | 0.2 |
| -0.443 | NA | NA | + | NA | -3.07E-02 | 1.80E-01 | 7 | -152.561 | 319.122 | 1.803 | 0.168 |
| -0.512 | NA | NA | + | NA | NA | 1.79E-01 | 6 | -144.937 | 301.875 | 0 | 0.252 |
| -0.642 | NA | NA | + | -6.04E-04 | NA | 1.90E-01 | 7 | -144.006 | 302.012 | 0.137 | 0.235 |
| -0.52 | NA | NA | + | -6.53E-04 | -6.21E-02 | 1.93E-01 | 8 | -143.557 | 303.113 | 1.239 | 0.136 |
| -0.551 | 1.51E-09 | NA | + | NA | NA | 1.85E-01 | 7 | -144.619 | 303.237 | 1.362 | 0.128 |
| -0.403 | NA | NA | + | NA | -5.07E-02 | 1.81E-01 | 7 | -144.637 | 303.275 | 1.4 | 0.125 |
| -0.686 | 1.59E-09 | NA | + | -6.14E-04 | NA | 1.96E-01 | 8 | -143.65 | 303.301 | 1.426 | 0.124 |
| -0.599 | NA | NA | + | NA | NA | 2.11E-01 | 6 | -160.554 | 333.109 | 0 | 0.262 |
| -0.701 | NA | NA | + | -5.04E-04 | NA | 2.19E-01 | 7 | -159.949 | 333.898 | 0.789 | 0.176 |
| -0.451 | NA | NA | + | NA | -6.82E-02 | 2.12E-01 | 7 | -159.986 | 333.972 | 0.863 | 0.17 |
| -0.541 | NA | NA | + | -6.03E-04 | -8.24E-02 | 2.21E-01 | 8 | -159.134 | 334.269 | 1.16 | 0.147 |
| -0.643 | 1.64E-09 | NA | + | NA | NA | 2.18E-01 | 7 | -160.165 | 334.33 | 1.221 | 0.142 |
| -0.754 | 1.78E-09 | NA | + | -5.31E-04 | NA | 2.27E-01 | 8 | -159.491 | 334.981 | 1.872 | 0.103 |
| -0.413 | NA | NA | + | NA | NA | 1.57E-01 | 6 | -142.305 | 296.609 | 0 | 0.419 |
| -0.497 | NA | NA | + | -3.57E-04 | NA | 1.64E-01 | 7 | -141.989 | 297.977 | 1.368 | 0.212 |
| -0.442 | 1.12E-09 | NA | + | NA | NA | 1.61E-01 | 7 | -142.102 | 298.205 | 1.595 | 0.189 |
| -0.335 | NA | NA | + | NA | -3.69E-02 | 1.59E-01 | 7 | -142.148 | 298.295 | 1.686 | 0.18 |
| -0.746 | NA | NA | + | -6.75E-04 | NA | 2.16E-01 | 7 | -159.95 | 333.901 | 0 | 0.203 |
| -0.604 | NA | NA | + | NA | NA | 2.04E-01 | 6 | -161.007 | 334.014 | 0.113 | 0.191 |
| -0.572 | NA | NA | + | -7.57E-04 | -8.53E-02 | 2.19E-01 | 8 | -159.146 | 334.292 | 0.392 | 0.167 |
| -0.448 | NA | NA | + | NA | -7.02E-02 | 2.05E-01 | 7 | -160.46 | 334.92 | 1.019 | 0.122 |
| -0.803 | 1.90E-09 | NA | + | -6.98E-04 | NA | 2.25E-01 | 8 | -159.465 | 334.93 | 1.029 | 0.121 |
| -0.653 | 1.75E-09 | NA | + | NA | NA | 2.12E-01 | 7 | -160.598 | 335.196 | 1.295 | 0.106 |
| -0.634 | 1.70E-09 | NA | + | -7.73E-04 | -8.01E-02 | 2.26E-01 | 9 | -158.756 | 335.511 | 1.611 | 0.091 |
| -0.403 | NA | NA | + | NA | NA | 1.53E-01 | 6 | -156.496 | 324.992 | 0 | 0.313 |
| -0.335 | NA | NA | NA | NA | NA | 1.91E-01 | 3 | -159.972 | 325.944 | 0.951 | 0.195 |
| -0.429 | 9.90E-10 | NA | + | NA | NA | 1.57E-01 | 7 | -156.382 | 326.763 | 1.771 | 0.129 |
| -0.459 | NA | NA | + | NA | 2.66E-02 | 1.52E-01 | 7 | -156.434 | 326.869 | 1.876 | 0.123 |
| -0.259 | NA | NA | NA | 4.07E-04 | NA | 1.71E-01 | 4 | -159.439 | 326.878 | 1.886 | 0.122 |
| -0.428 | NA | NA | + | -1.07E-04 | NA | 1.56E-01 | 7 | -156.472 | 326.945 | 1.952 | 0.118 |
| -0.402 | NA | NA | + | NA | NA | 1.57E-01 | 6 | -150.333 | 312.666 | 0 | 0.365 |
| -0.336 | NA | NA | NA | NA | NA | 1.95E-01 | 3 | -153.934 | 313.869 | 1.202 | 0.2 |
| -0.434 | 1.25E-09 | NA | + | NA | NA | 1.61E-01 | 7 | -150.144 | 314.288 | 1.622 | 0.162 |
| -0.433 | NA | NA | + | -1.30E-04 | NA | 1.60E-01 | 7 | -150.297 | 314.594 | 1.927 | 0.139 |
| -0.407 | NA | NA | + | NA | 2.29E-03 | 1.57E-01 | 7 | -150.333 | 314.665 | 1.999 | 0.134 |
| -0.45 | NA | NA | + | NA | NA | 1.65E-01 | 6 | -143.998 | 299.996 | 0 | 0.414 |
| -0.544 | NA | NA | + | -4.17E-04 | NA | 1.72E-01 | 7 | -143.566 | 301.131 | 1.135 | 0.235 |
| -0.481 | 9.64E-10 | NA | + | NA | NA | 1.71E-01 | 7 | -143.774 | 301.549 | 1.552 | 0.191 |
| -0.403 | NA | NA | + | NA | -2.13E-02 | 1.66E-01 | 7 | -143.948 | 301.897 | 1.9 | 0.16 |
| -0.477 | NA | NA | + | NA | NA | 1.68E-01 | 6 | -146.327 | 304.654 | 0 | 0.391 |
| -0.59 | NA | NA | + | -4.91E-04 | NA | 1.78E-01 | 7 | -145.72 | 305.439 | 0.785 | 0.264 |
| -0.51 | 1.20E-09 | NA | + | NA | NA | 1.73E-01 | 7 | -146.087 | 306.173 | 1.519 | 0.183 |
| -0.404 | NA | NA | + | NA | -3.27E-02 | 1.69E-01 | 7 | -146.213 | 306.426 | 1.772 | 0.161 |
| -0.436 | NA | NA | + | NA | NA | 1.57E-01 | 6 | -159.716 | 331.431 | 0 | 0.46 |
| -0.465 | 1.07E-09 | NA | + | NA | NA | 1.61E-01 | 7 | -159.565 | 333.13 | 1.698 | 0.197 |
| -0.46 | NA | NA | + | -9.73E-05 | NA | 1.59E-01 | 7 | -159.696 | 333.392 | 1.961 | 0.173 |
| -0.454 | NA | NA | + | NA | 7.89E-03 | 1.56E-01 | 7 | -159.71 | 333.42 | 1.989 | 0.17 |
| -0.458 | NA | NA | + | NA | NA | 1.62E-01 | 6 | -165.359 | 342.718 | 0 | 0.388 |
| -0.488 | 1.12E-09 | NA | + | NA | NA | 1.67E-01 | 7 | -165.227 | 344.455 | 1.737 | 0.163 |
| -0.377 | NA | NA | NA | NA | NA | 2.05E-01 | 3 | -169.231 | 344.462 | 1.744 | 0.162 |
| -0.467 | NA | NA | + | -3.72E-05 | NA | 1.63E-01 | 7 | -165.356 | 344.713 | 1.995 | 0.143 |
| -0.449 | NA | NA | + | NA | -3.97E-03 | 1.63E-01 | 7 | -165.358 | 344.715 | 1.997 | 0.143 |
| -0.392 | NA | NA | + | NA | NA | 1.52E-01 | 6 | -147.31 | 306.62 | 0 | 0.364 |
| -0.331 | NA | NA | NA | NA | NA | 1.89E-01 | 3 | -150.907 | 307.813 | 1.194 | 0.201 |
| -0.422 | 9.61E-10 | NA | + | NA | NA | 1.57E-01 | 7 | -147.128 | 308.257 | 1.637 | 0.161 |
| -0.36 | NA | NA | + | NA | -1.53E-02 | 1.52E-01 | 7 | -147.286 | 308.571 | 1.952 | 0.137 |
| -0.416 | NA | NA | + | -1.00E-04 | NA | 1.54E-01 | 7 | -147.287 | 308.573 | 1.954 | 0.137 |
| -0.5 | NA | NA | + | NA | NA | 1.75E-01 | 6 | -155.423 | 322.845 | 0 | 0.417 |
| -0.574 | NA | NA | + | -3.40E-04 | NA | 1.81E-01 | 7 | -155.159 | 324.317 | 1.472 | 0.2 |
| -0.536 | 1.35E-09 | NA | + | NA | NA | 1.80E-01 | 7 | -155.179 | 324.357 | 1.512 | 0.196 |
| -0.404 | NA | NA | + | NA | -4.27E-02 | 1.75E-01 | 7 | -155.227 | 324.453 | 1.608 | 0.187 |
| -0.429 | NA | NA | + | NA | NA | 1.56E-01 | 6 | -148.739 | 309.478 | 0 | 0.431 |
| -0.464 | 1.22E-09 | NA | + | NA | NA | 1.61E-01 | 7 | -148.49 | 310.98 | 1.502 | 0.203 |
| -0.492 | NA | NA | + | -2.72E-04 | NA | 1.61E-01 | 7 | -148.565 | 311.129 | 1.651 | 0.189 |
| -0.359 | NA | NA | + | NA | -3.14E-02 | 1.57E-01 | 7 | -148.632 | 311.264 | 1.786 | 0.177 |
| -0.582 | NA | NA | + | -9.61E-04 | -1.17E-01 | 2.41E-01 | 8 | -152.74 | 321.481 | 0 | 0.474 |
| -0.652 | 1.87E-09 | NA | + | -9.91E-04 | -1.12E-01 | 2.50E-01 | 9 | -152.149 | 322.298 | 0.817 | 0.315 |
| -0.815 | NA | NA | + | -8.24E-04 | NA | 2.39E-01 | 7 | -154.548 | 323.097 | 1.616 | 0.211 |
| -0.417 | NA | NA | + | NA | NA | 1.54E-01 | 6 | -150.46 | 312.92 | 0 | 0.451 |
| -0.447 | 9.30E-10 | NA | + | NA | NA | 1.60E-01 | 7 | -150.268 | 314.536 | 1.616 | 0.201 |
| -0.462 | NA | NA | + | -1.99E-04 | NA | 1.57E-01 | 7 | -150.373 | 314.745 | 1.825 | 0.181 |
| -0.43 | NA | NA | + | NA | 6.03E-03 | 1.53E-01 | 7 | -150.457 | 314.913 | 1.993 | 0.167 |
| -0.479 | NA | NA | + | NA | NA | 1.69E-01 | 6 | -153.125 | 318.25 | 0 | 0.391 |
| -0.592 | NA | NA | + | -5.11E-04 | NA | 1.78E-01 | 7 | -152.514 | 319.028 | 0.778 | 0.265 |
| -0.512 | 1.11E-09 | NA | + | NA | NA | 1.74E-01 | 7 | -152.898 | 319.796 | 1.546 | 0.181 |
| -0.406 | NA | NA | + | NA | -3.49E-02 | 1.71E-01 | 7 | -152.998 | 319.997 | 1.746 | 0.163 |
| -0.634 | NA | NA | + | NA | NA | 2.11E-01 | 6 | -167.772 | 347.545 | 0 | 0.409 |
| -0.68 | 1.55E-09 | NA | + | NA | NA | 2.19E-01 | 7 | -167.444 | 348.887 | 1.342 | 0.209 |
| -0.707 | NA | NA | + | -3.43E-04 | NA | 2.17E-01 | 7 | -167.521 | 349.043 | 1.498 | 0.193 |
| -0.524 | NA | NA | + | NA | -4.74E-02 | 2.10E-01 | 7 | -167.544 | 349.089 | 1.544 | 0.189 |
| -0.487 | NA | NA | + | NA | NA | 1.75E-01 | 6 | -154.16 | 320.319 | 0 | 0.413 |
| -0.523 | 1.19E-09 | NA | + | NA | NA | 1.82E-01 | 7 | -153.889 | 321.778 | 1.459 | 0.199 |
| -0.562 | NA | NA | + | -3.38E-04 | NA | 1.82E-01 | 7 | -153.896 | 321.793 | 1.473 | 0.198 |
| -0.386 | NA | NA | + | NA | -4.62E-02 | 1.77E-01 | 7 | -153.935 | 321.87 | 1.551 | 0.19 |
| -0.434 | NA | NA | + | NA | NA | 1.63E-01 | 6 | -144.843 | 301.687 | 0 | 0.425 |
| -0.518 | NA | NA | + | -3.48E-04 | NA | 1.72E-01 | 7 | -144.549 | 303.098 | 1.411 | 0.21 |
| -0.464 | 1.11E-09 | NA | + | NA | NA | 1.68E-01 | 7 | -144.648 | 303.296 | 1.609 | 0.19 |
| -0.365 | NA | NA | + | NA | -3.12E-02 | 1.64E-01 | 7 | -144.735 | 303.47 | 1.783 | 0.174 |
| -0.416 | NA | NA | + | NA | NA | 1.54E-01 | 6 | -149.89 | 311.779 | 0 | 0.442 |
| -0.445 | 9.99E-10 | NA | + | NA | NA | 1.60E-01 | 7 | -149.699 | 313.397 | 1.618 | 0.197 |
| -0.467 | NA | NA | + | -2.21E-04 | NA | 1.59E-01 | 7 | -149.779 | 313.558 | 1.779 | 0.182 |
| -0.347 | NA | NA | + | NA | -3.09E-02 | 1.55E-01 | 7 | -149.79 | 313.581 | 1.801 | 0.18 |
| -0.477 | NA | NA | + | NA | NA | 1.69E-01 | 6 | -145.099 | 302.198 | 0 | 0.265 |
| -0.592 | NA | NA | + | -5.45E-04 | NA | 1.77E-01 | 7 | -144.332 | 302.663 | 0.465 | 0.21 |
| -0.358 | NA | NA | + | NA | -5.51E-02 | 1.72E-01 | 7 | -144.727 | 303.455 | 1.257 | 0.142 |
| -0.516 | 1.41E-09 | NA | + | NA | NA | 1.75E-01 | 7 | -144.769 | 303.538 | 1.34 | 0.136 |
| -0.46 | NA | NA | + | -6.07E-04 | -6.69E-02 | 1.82E-01 | 8 | -143.788 | 303.576 | 1.379 | 0.133 |
| -0.638 | 1.52E-09 | NA | + | -5.65E-04 | NA | 1.83E-01 | 8 | -143.944 | 303.888 | 1.691 | 0.114 |
| -0.509 | NA | NA | + | -7.58E-04 | -9.08E-02 | 2.07E-01 | 8 | -136.233 | 288.466 | 0 | 0.209 |
| -0.694 | NA | NA | + | -6.59E-04 | NA | 2.06E-01 | 7 | -137.313 | 288.625 | 0.159 | 0.193 |
| -0.555 | NA | NA | + | NA | NA | 1.95E-01 | 6 | -138.622 | 289.245 | 0.778 | 0.142 |
| -0.576 | 1.39E-09 | NA | + | -8.12E-04 | -8.77E-02 | 2.19E-01 | 9 | -135.653 | 289.307 | 0.841 | 0.138 |
| -0.758 | 1.48E-09 | NA | + | -7.20E-04 | NA | 2.19E-01 | 8 | -136.667 | 289.333 | 0.867 | 0.136 |
| -0.391 | NA | NA | + | NA | -7.20E-02 | 1.94E-01 | 7 | -137.938 | 289.877 | 1.411 | 0.103 |
| -0.596 | 1.18E-09 | NA | + | NA | NA | 2.04E-01 | 7 | -138.214 | 290.427 | 1.961 | 0.079 |
| -0.618 | NA | NA | + | -7.98E-04 | -1.04E-01 | 2.43E-01 | 8 | -158.959 | 333.919 | 0 | 0.226 |
| -0.832 | NA | NA | + | -6.75E-04 | NA | 2.42E-01 | 7 | -160.251 | 334.501 | 0.582 | 0.169 |
| -0.7 | NA | NA | + | NA | NA | 2.31E-01 | 6 | -161.37 | 334.741 | 0.822 | 0.15 |
| -0.505 | NA | NA | + | NA | -8.49E-02 | 2.30E-01 | 7 | -160.502 | 335.004 | 1.085 | 0.131 |
| -0.683 | 1.76E-09 | NA | + | -8.25E-04 | -9.85E-02 | 2.52E-01 | 9 | -158.524 | 335.048 | 1.129 | 0.128 |
| -0.894 | 2.03E-09 | NA | + | -7.14E-04 | NA | 2.52E-01 | 8 | -159.676 | 335.352 | 1.434 | 0.11 |
| -0.748 | 1.79E-09 | NA | + | NA | NA | 2.39E-01 | 7 | -160.929 | 335.859 | 1.94 | 0.086 |
| -0.576 | NA | NA | + | NA | NA | 1.94E-01 | 6 | -162.23 | 336.46 | 0 | 0.419 |
| -0.618 | 1.21E-09 | NA | + | NA | NA | 2.03E-01 | 7 | -161.931 | 337.863 | 1.403 | 0.208 |
| -0.646 | NA | NA | + | -3.52E-04 | NA | 2.00E-01 | 7 | -161.964 | 337.928 | 1.468 | 0.201 |
| -0.502 | NA | NA | + | NA | -3.38E-02 | 1.95E-01 | 7 | -162.113 | 338.225 | 1.766 | 0.173 |
| -0.638 | NA | NA | + | NA | NA | 2.16E-01 | 6 | -176.911 | 365.822 | 0 | 0.393 |
| -0.687 | 1.75E-09 | NA | + | NA | NA | 2.24E-01 | 7 | -176.512 | 367.024 | 1.202 | 0.216 |
| -0.507 | NA | NA | + | NA | -5.98E-02 | 2.17E-01 | 7 | -176.532 | 367.063 | 1.241 | 0.211 |
| -0.702 | NA | NA | + | -3.28E-04 | NA | 2.21E-01 | 7 | -176.692 | 367.385 | 1.563 | 0.18 |
| -0.535 | NA | NA | + | NA | NA | 1.88E-01 | 6 | -164.072 | 340.143 | 0 | 0.435 |
| -0.571 | 1.31E-09 | NA | + | NA | NA | 1.94E-01 | 7 | -163.837 | 341.673 | 1.53 | 0.202 |
| -0.594 | NA | NA | + | -2.80E-04 | NA | 1.94E-01 | 7 | -163.907 | 341.813 | 1.67 | 0.189 |
| -0.47 | NA | NA | + | NA | -2.95E-02 | 1.89E-01 | 7 | -163.988 | 341.976 | 1.833 | 0.174 |
| -0.599 | NA | NA | + | NA | NA | 2.05E-01 | 6 | -159.661 | 331.322 | 0 | 0.27 |
| -0.712 | NA | NA | + | -5.31E-04 | NA | 2.16E-01 | 7 | -159.015 | 332.03 | 0.708 | 0.189 |
| -0.646 | 1.72E-09 | NA | + | NA | NA | 2.13E-01 | 7 | -159.245 | 332.49 | 1.168 | 0.15 |
| -0.462 | NA | NA | + | NA | -6.07E-02 | 2.05E-01 | 7 | -159.251 | 332.502 | 1.18 | 0.15 |
| -0.563 | NA | NA | + | -6.05E-04 | -7.31E-02 | 2.18E-01 | 8 | -158.426 | 332.851 | 1.529 | 0.126 |
| -0.772 | 1.88E-09 | NA | + | -5.66E-04 | NA | 2.25E-01 | 8 | -158.512 | 333.024 | 1.702 | 0.115 |
| -0.39 | NA | NA | + | NA | NA | 1.53E-01 | 6 | -152.529 | 317.058 | 0 | 0.385 |
| -0.319 | NA | NA | NA | NA | NA | 1.86E-01 | 3 | -156.367 | 318.735 | 1.677 | 0.166 |
| -0.413 | 8.81E-10 | NA | + | NA | NA | 1.56E-01 | 7 | -152.413 | 318.826 | 1.769 | 0.159 |
| -0.422 | NA | NA | + | -1.36E-04 | NA | 1.56E-01 | 7 | -152.487 | 318.974 | 1.916 | 0.148 |
| -0.398 | NA | NA | + | NA | 3.96E-03 | 1.53E-01 | 7 | -152.527 | 319.055 | 1.997 | 0.142 |
| -0.716 | NA | NA | + | -7.48E-04 | NA | 2.08E-01 | 7 | -145.407 | 304.814 | 0 | 0.365 |
| -0.562 | NA | NA | + | NA | NA | 1.95E-01 | 6 | -146.884 | 305.769 | 0.955 | 0.227 |
| -0.592 | NA | NA | + | -8.03E-04 | -6.45E-02 | 2.12E-01 | 8 | -144.932 | 305.864 | 1.05 | 0.216 |
| -0.761 | 1.34E-09 | NA | + | -7.74E-04 | NA | 2.15E-01 | 8 | -145.051 | 306.102 | 1.288 | 0.192 |
| -0.417 | NA | NA | + | NA | NA | 1.62E-01 | 6 | -138.278 | 288.557 | 0 | 0.329 |
| -0.527 | NA | NA | + | -4.87E-04 | NA | 1.73E-01 | 7 | -137.638 | 289.276 | 0.719 | 0.23 |
| -0.314 | NA | NA | + | NA | -4.75E-02 | 1.64E-01 | 7 | -138.003 | 290.006 | 1.449 | 0.16 |
| -0.449 | 9.66E-10 | NA | + | NA | NA | 1.69E-01 | 7 | -138.032 | 290.063 | 1.507 | 0.155 |
| -0.412 | NA | NA | + | -5.37E-04 | -5.77E-02 | 1.76E-01 | 8 | -137.234 | 290.468 | 1.912 | 0.127 |
| -0.398 | NA | NA | + | NA | NA | 1.49E-01 | 6 | -149.598 | 311.195 | 0 | 0.32 |
| -0.334 | NA | NA | NA | NA | NA | 1.90E-01 | 3 | -153.184 | 312.367 | 1.172 | 0.178 |
| -0.425 | 9.76E-10 | NA | + | NA | NA | 1.54E-01 | 7 | -149.443 | 312.885 | 1.69 | 0.137 |
| -0.251 | NA | NA | NA | 4.08E-04 | NA | 1.67E-01 | 4 | -152.538 | 313.077 | 1.882 | 0.125 |
| -0.431 | NA | NA | + | -1.37E-04 | NA | 1.52E-01 | 7 | -149.555 | 313.11 | 1.915 | 0.123 |
| -0.403 | NA | NA | + | NA | 2.21E-03 | 1.49E-01 | 7 | -149.597 | 313.194 | 1.999 | 0.118 |
| -0.475 | NA | NA | + | NA | NA | 1.68E-01 | 6 | -143.274 | 298.549 | 0 | 0.242 |
| -0.607 | NA | NA | + | -6.15E-04 | NA | 1.77E-01 | 7 | -142.287 | 298.574 | 0.025 | 0.239 |
| -0.479 | NA | NA | + | -6.74E-04 | -6.44E-02 | 1.80E-01 | 8 | -141.788 | 299.575 | 1.026 | 0.145 |
| -0.657 | 1.29E-09 | NA | + | -6.53E-04 | NA | 1.85E-01 | 8 | -141.873 | 299.747 | 1.198 | 0.133 |
| -0.363 | NA | NA | + | NA | -5.12E-02 | 1.70E-01 | 7 | -142.958 | 299.915 | 1.367 | 0.122 |
| -0.51 | 1.09E-09 | NA | + | NA | NA | 1.75E-01 | 7 | -142.982 | 299.964 | 1.416 | 0.119 |
| -0.69 | NA | NA | + | NA | NA | 2.31E-01 | 6 | -166.012 | 344.023 | 0 | 0.278 |
| -0.787 | NA | NA | + | -5.02E-04 | NA | 2.40E-01 | 7 | -165.439 | 344.879 | 0.855 | 0.181 |
| -0.739 | 1.73E-09 | NA | + | NA | NA | 2.39E-01 | 7 | -165.593 | 345.186 | 1.163 | 0.155 |
| -0.549 | NA | NA | + | NA | -6.05E-02 | 2.29E-01 | 7 | -165.609 | 345.218 | 1.194 | 0.153 |
| -0.631 | NA | NA | + | -5.84E-04 | -7.40E-02 | 2.39E-01 | 8 | -164.848 | 345.696 | 1.672 | 0.121 |
| -0.85 | 1.92E-09 | NA | + | -5.42E-04 | NA | 2.50E-01 | 8 | -164.926 | 345.851 | 1.828 | 0.111 |
| -0.551 | NA | NA | + | NA | NA | 1.94E-01 | 6 | -153.183 | 318.367 | 0 | 0.4 |
| -0.643 | NA | NA | + | -4.69E-04 | NA | 2.01E-01 | 7 | -152.654 | 319.308 | 0.941 | 0.25 |
| -0.589 | 9.75E-10 | NA | + | NA | NA | 2.03E-01 | 7 | -152.889 | 319.777 | 1.411 | 0.198 |
| -0.511 | NA | NA | + | NA | -1.83E-02 | 1.94E-01 | 7 | -153.147 | 320.295 | 1.928 | 0.153 |
| -0.434 | NA | NA | + | NA | NA | 1.59E-01 | 6 | -157.071 | 326.143 | 0 | 0.454 |
| -0.463 | 1.09E-09 | NA | + | NA | NA | 1.63E-01 | 7 | -156.903 | 327.806 | 1.663 | 0.198 |
| -0.48 | NA | NA | + | -1.95E-04 | NA | 1.63E-01 | 7 | -156.99 | 327.98 | 1.837 | 0.181 |
| -0.433 | NA | NA | + | NA | -8.07E-04 | 1.59E-01 | 7 | -157.071 | 328.143 | 2 | 0.167 |
| -0.499 | NA | NA | + | NA | NA | 1.74E-01 | 6 | -149.353 | 310.705 | 0 | 0.409 |
| -0.584 | NA | NA | + | -3.71E-04 | NA | 1.82E-01 | 7 | -149.023 | 312.047 | 1.341 | 0.209 |
| -0.532 | 1.03E-09 | NA | + | NA | NA | 1.81E-01 | 7 | -149.108 | 312.217 | 1.511 | 0.192 |
| -0.393 | NA | NA | + | NA | -4.68E-02 | 1.75E-01 | 7 | -149.121 | 312.242 | 1.537 | 0.19 |
| -0.49 | NA | NA | + | NA | NA | 1.73E-01 | 6 | -152.288 | 316.576 | 0 | 0.407 |
| -0.573 | NA | NA | + | -3.79E-04 | NA | 1.80E-01 | 7 | -151.953 | 317.906 | 1.33 | 0.209 |
| -0.526 | 1.27E-09 | NA | + | NA | NA | 1.80E-01 | 7 | -152.002 | 318.003 | 1.428 | 0.199 |
| -0.394 | NA | NA | + | NA | -4.34E-02 | 1.74E-01 | 7 | -152.074 | 318.148 | 1.572 | 0.185 |
| -0.48 | NA | NA | + | NA | NA | 1.71E-01 | 6 | -156.736 | 325.473 | 0 | 0.444 |
| -0.518 | 8.84E-10 | NA | + | NA | NA | 1.81E-01 | 7 | -156.457 | 326.913 | 1.44 | 0.216 |
| -0.509 | NA | NA | + | -1.39E-04 | NA | 1.73E-01 | 7 | -156.693 | 327.385 | 1.913 | 0.171 |
| -0.441 | NA | NA | + | NA | -1.72E-02 | 1.72E-01 | 7 | -156.707 | 327.414 | 1.941 | 0.168 |
| -0.317 | NA | NA | + | NA | NA | 1.30E-01 | 6 | -131.002 | 274.004 | 0 | 0.307 |
| -0.254 | NA | NA | NA | NA | NA | 1.60E-01 | 3 | -134.453 | 274.906 | 0.902 | 0.195 |
| -0.189 | NA | NA | NA | 3.55E-04 | NA | 1.43E-01 | 4 | -133.844 | 275.687 | 1.684 | 0.132 |
| -0.339 | 6.45E-10 | NA | + | NA | NA | 1.34E-01 | 7 | -130.872 | 275.745 | 1.741 | 0.129 |
| -0.361 | NA | NA | + | -1.83E-04 | NA | 1.33E-01 | 7 | -130.916 | 275.832 | 1.828 | 0.123 |
| -0.3 | NA | NA | + | NA | -7.88E-03 | 1.31E-01 | 7 | -130.995 | 275.989 | 1.986 | 0.114 |
| -0.663 | NA | NA | + | NA | NA | 2.22E-01 | 6 | -172.801 | 357.602 | 0 | 0.375 |
| -0.763 | NA | NA | + | -4.87E-04 | NA | 2.30E-01 | 7 | -172.303 | 358.607 | 1.004 | 0.227 |
| -0.72 | 2.23E-09 | NA | + | NA | NA | 2.29E-01 | 7 | -172.348 | 358.696 | 1.094 | 0.217 |
| -0.547 | NA | NA | + | NA | -5.18E-02 | 2.23E-01 | 7 | -172.523 | 359.045 | 1.443 | 0.182 |
| -0.42 | NA | NA | + | NA | NA | 1.55E-01 | 6 | -144.163 | 300.326 | 0 | 0.444 |
| -0.483 | NA | NA | + | -2.73E-04 | NA | 1.60E-01 | 7 | -143.974 | 301.948 | 1.622 | 0.197 |
| -0.446 | 8.05E-10 | NA | + | NA | NA | 1.61E-01 | 7 | -144 | 301.999 | 1.673 | 0.192 |
| -0.389 | NA | NA | + | NA | -1.44E-02 | 1.56E-01 | 7 | -144.141 | 302.282 | 1.956 | 0.167 |
| -0.522 | NA | NA | + | NA | NA | 1.85E-01 | 6 | -154.092 | 320.184 | 0 | 0.397 |
| -0.605 | NA | NA | + | -3.93E-04 | NA | 1.92E-01 | 7 | -153.731 | 321.463 | 1.279 | 0.21 |
| -0.563 | 1.37E-09 | NA | + | NA | NA | 1.92E-01 | 7 | -153.763 | 321.526 | 1.342 | 0.203 |
| -0.411 | NA | NA | + | NA | -4.91E-02 | 1.85E-01 | 7 | -153.831 | 321.662 | 1.478 | 0.19 |
| -0.377 | NA | NA | + | NA | NA | 1.47E-01 | 6 | -145.58 | 303.161 | 0 | 0.308 |
| -0.301 | NA | NA | NA | NA | NA | 1.79E-01 | 3 | -148.987 | 303.974 | 0.813 | 0.205 |
| -0.401 | 8.80E-10 | NA | + | NA | NA | 1.51E-01 | 7 | -145.453 | 304.905 | 1.745 | 0.129 |
| -0.235 | NA | NA | NA | 3.72E-04 | NA | 1.62E-01 | 4 | -148.472 | 304.943 | 1.783 | 0.126 |
| -0.409 | NA | NA | + | -1.38E-04 | NA | 1.50E-01 | 7 | -145.536 | 305.072 | 1.912 | 0.118 |
| -0.378 | NA | NA | + | NA | 2.30E-04 | 1.47E-01 | 7 | -145.58 | 305.161 | 2 | 0.113 |
| -0.416 | NA | NA | + | NA | NA | 1.56E-01 | 6 | -163.363 | 338.727 | 0 | 0.286 |
| -0.333 | NA | NA | NA | NA | NA | 1.87E-01 | 3 | -166.709 | 339.419 | 0.692 | 0.203 |
| -0.247 | NA | NA | NA | 5.16E-04 | NA | 1.66E-01 | 4 | -165.895 | 339.789 | 1.062 | 0.168 |
| -0.448 | 1.24E-09 | NA | + | NA | NA | 1.60E-01 | 7 | -163.185 | 340.371 | 1.644 | 0.126 |
| -0.469 | NA | NA | + | NA | 2.53E-02 | 1.54E-01 | 7 | -163.308 | 340.617 | 1.89 | 0.111 |
| -0.405 | NA | NA | + | 4.91E-05 | NA | 1.55E-01 | 7 | -163.359 | 340.717 | 1.991 | 0.106 |
| -0.482 | NA | NA | + | NA | NA | 1.74E-01 | 6 | -154.558 | 321.116 | 0 | 0.416 |
| -0.519 | 1.32E-09 | NA | + | NA | NA | 1.81E-01 | 7 | -154.268 | 322.535 | 1.419 | 0.205 |
| -0.559 | NA | NA | + | -3.54E-04 | NA | 1.82E-01 | 7 | -154.272 | 322.544 | 1.428 | 0.204 |
| -0.401 | NA | NA | + | NA | -3.60E-02 | 1.74E-01 | 7 | -154.418 | 322.837 | 1.72 | 0.176 |
| -0.406 | NA | NA | + | NA | NA | 1.55E-01 | 6 | -144.67 | 301.341 | 0 | 0.441 |
| -0.471 | NA | NA | + | -2.90E-04 | NA | 1.60E-01 | 7 | -144.465 | 302.93 | 1.589 | 0.199 |
| -0.432 | 9.95E-10 | NA | + | NA | NA | 1.60E-01 | 7 | -144.488 | 302.976 | 1.636 | 0.195 |
| -0.377 | NA | NA | + | NA | -1.37E-02 | 1.56E-01 | 7 | -144.651 | 303.301 | 1.96 | 0.165 |
| -0.499 | NA | NA | + | NA | NA | 1.79E-01 | 6 | -148.279 | 308.557 | 0 | 0.391 |
| -0.593 | NA | NA | + | -4.24E-04 | NA | 1.88E-01 | 7 | -147.818 | 309.637 | 1.079 | 0.228 |
| -0.385 | NA | NA | + | NA | -5.12E-02 | 1.79E-01 | 7 | -147.981 | 309.962 | 1.405 | 0.193 |
| -0.535 | 1.31E-09 | NA | + | NA | NA | 1.85E-01 | 7 | -148.008 | 310.015 | 1.458 | 0.188 |
| -0.607 | NA | NA | + | NA | NA | 2.04E-01 | 6 | -157.224 | 326.449 | 0 | 0.217 |
| -0.721 | NA | NA | + | -5.48E-04 | NA | 2.14E-01 | 7 | -156.496 | 326.991 | 0.543 | 0.165 |
| -0.54 | NA | NA | + | -6.48E-04 | -9.01E-02 | 2.17E-01 | 8 | -155.54 | 327.08 | 0.631 | 0.158 |
| -0.438 | NA | NA | + | NA | -7.56E-02 | 2.05E-01 | 7 | -156.543 | 327.086 | 0.637 | 0.158 |
| -0.653 | 1.61E-09 | NA | + | NA | NA | 2.12E-01 | 7 | -156.829 | 327.659 | 1.21 | 0.118 |
| -0.778 | 1.78E-09 | NA | + | -5.80E-04 | NA | 2.23E-01 | 8 | -156.014 | 328.027 | 1.579 | 0.098 |
| -0.6 | 1.59E-09 | NA | + | -6.71E-04 | -8.57E-02 | 2.25E-01 | 9 | -155.15 | 328.3 | 1.852 | 0.086 |
| -0.396 | NA | NA | + | NA | NA | 1.52E-01 | 6 | -142.312 | 296.624 | 0 | 0.442 |
| -0.463 | NA | NA | + | -2.84E-04 | NA | 1.58E-01 | 7 | -142.117 | 298.235 | 1.611 | 0.198 |
| -0.423 | 9.78E-10 | NA | + | NA | NA | 1.56E-01 | 7 | -142.157 | 298.314 | 1.69 | 0.19 |
| -0.354 | NA | NA | + | NA | -2.01E-02 | 1.53E-01 | 7 | -142.267 | 298.535 | 1.911 | 0.17 |
| -0.45 | NA | NA | + | NA | NA | 1.65E-01 | 6 | -139.017 | 290.034 | 0 | 0.268 |
| -0.572 | NA | NA | + | -5.36E-04 | NA | 1.75E-01 | 7 | -138.214 | 290.427 | 0.393 | 0.22 |
| -0.337 | NA | NA | + | NA | -5.22E-02 | 1.68E-01 | 7 | -138.692 | 291.384 | 1.35 | 0.136 |
| -0.447 | NA | NA | + | -5.84E-04 | -6.26E-02 | 1.80E-01 | 8 | -137.747 | 291.493 | 1.459 | 0.129 |
| -0.48 | 9.04E-10 | NA | + | NA | NA | 1.72E-01 | 7 | -138.767 | 291.533 | 1.499 | 0.126 |
| -0.621 | 1.15E-09 | NA | + | -5.89E-04 | NA | 1.84E-01 | 8 | -137.814 | 291.628 | 1.593 | 0.121 |
| -0.592 | NA | NA | + | NA | NA | 2.03E-01 | 6 | -161.888 | 335.776 | 0 | 0.186 |
| -0.524 | NA | NA | + | -6.81E-04 | -9.22E-02 | 2.14E-01 | 8 | -160.096 | 336.191 | 0.415 | 0.152 |
| -0.707 | NA | NA | + | -5.71E-04 | NA | 2.11E-01 | 7 | -161.122 | 336.245 | 0.469 | 0.148 |
| -0.42 | NA | NA | + | NA | -7.67E-02 | 2.04E-01 | 7 | -161.166 | 336.333 | 0.556 | 0.141 |
| -0.64 | 1.65E-09 | NA | + | NA | NA | 2.12E-01 | 7 | -161.419 | 336.837 | 1.061 | 0.11 |
| -0.769 | 1.83E-09 | NA | + | -6.12E-04 | NA | 2.21E-01 | 8 | -160.54 | 337.079 | 1.303 | 0.097 |
| -0.59 | 1.66E-09 | NA | + | -7.12E-04 | -8.75E-02 | 2.23E-01 | 9 | -159.613 | 337.227 | 1.451 | 0.09 |
| -0.475 | 1.48E-09 | NA | + | NA | -7.19E-02 | 2.12E-01 | 8 | -160.786 | 337.572 | 1.796 | 0.076 |
| -0.418 | NA | NA | + | NA | NA | 1.57E-01 | 6 | -146.367 | 304.734 | 0 | 0.445 |
| -0.451 | 1.11E-09 | NA | + | NA | NA | 1.62E-01 | 7 | -146.142 | 306.285 | 1.551 | 0.205 |
| -0.466 | NA | NA | + | -2.13E-04 | NA | 1.61E-01 | 7 | -146.262 | 306.524 | 1.791 | 0.182 |
| -0.381 | NA | NA | + | NA | -1.71E-02 | 1.58E-01 | 7 | -146.337 | 306.673 | 1.94 | 0.169 |
| -0.548 | NA | NA | + | NA | NA | 1.87E-01 | 6 | -152.114 | 316.227 | 0 | 0.268 |
| -0.668 | NA | NA | + | -5.40E-04 | NA | 1.97E-01 | 7 | -151.386 | 316.772 | 0.545 | 0.204 |
| -0.407 | NA | NA | + | NA | -6.45E-02 | 1.89E-01 | 7 | -151.646 | 317.291 | 1.064 | 0.158 |
| -0.519 | NA | NA | + | -5.89E-04 | -7.32E-02 | 2.00E-01 | 8 | -150.783 | 317.566 | 1.339 | 0.137 |
| -0.583 | 1.19E-09 | NA | + | NA | NA | 1.93E-01 | 7 | -151.856 | 317.712 | 1.485 | 0.128 |
| -0.714 | 1.35E-09 | NA | + | -5.69E-04 | NA | 2.05E-01 | 8 | -151.051 | 318.101 | 1.874 | 0.105 |
| -0.629 | NA | NA | + | -6.41E-04 | NA | 1.88E-01 | 7 | -146.025 | 306.049 | 0 | 0.211 |
| -0.489 | NA | NA | + | NA | NA | 1.76E-01 | 6 | -147.102 | 306.204 | 0.155 | 0.196 |
| -0.472 | NA | NA | + | -7.14E-04 | -7.94E-02 | 1.91E-01 | 8 | -145.249 | 306.499 | 0.45 | 0.169 |
| -0.347 | NA | NA | + | NA | -6.55E-02 | 1.77E-01 | 7 | -146.574 | 307.147 | 1.098 | 0.122 |
| -0.678 | 1.36E-09 | NA | + | -6.76E-04 | NA | 1.96E-01 | 8 | -145.62 | 307.24 | 1.191 | 0.117 |
| -0.525 | 1.15E-09 | NA | + | NA | NA | 1.82E-01 | 7 | -146.812 | 307.623 | 1.574 | 0.096 |
| -0.523 | 1.28E-09 | NA | + | -7.45E-04 | -7.71E-02 | 1.98E-01 | 9 | -144.886 | 307.772 | 1.723 | 0.089 |
| -0.456 | NA | NA | + | NA | NA | 1.69E-01 | 6 | -151.928 | 315.857 | 0 | 0.425 |
| -0.489 | 9.76E-10 | NA | + | NA | NA | 1.76E-01 | 7 | -151.68 | 317.36 | 1.503 | 0.201 |
| -0.352 | NA | NA | + | NA | -4.70E-02 | 1.70E-01 | 7 | -151.688 | 317.376 | 1.52 | 0.199 |
| -0.505 | NA | NA | + | -2.20E-04 | NA | 1.74E-01 | 7 | -151.815 | 317.629 | 1.773 | 0.175 |
| -0.473 | NA | NA | + | NA | NA | 1.74E-01 | 6 | -147.364 | 306.728 | 0 | 0.402 |
| -0.558 | NA | NA | + | -4.01E-04 | NA | 1.81E-01 | 7 | -146.966 | 307.931 | 1.203 | 0.22 |
| -0.508 | 9.80E-10 | NA | + | NA | NA | 1.82E-01 | 7 | -147.092 | 308.184 | 1.456 | 0.194 |
| -0.378 | NA | NA | + | NA | -4.30E-02 | 1.74E-01 | 7 | -147.153 | 308.306 | 1.577 | 0.183 |
| -0.419 | NA | NA | + | NA | NA | 1.55E-01 | 6 | -148.368 | 308.736 | 0 | 0.417 |
| -0.502 | NA | NA | + | -3.51E-04 | NA | 1.63E-01 | 7 | -148.084 | 310.167 | 1.431 | 0.204 |
| -0.451 | 1.01E-09 | NA | + | NA | NA | 1.61E-01 | 7 | -148.153 | 310.305 | 1.569 | 0.19 |
| -0.325 | NA | NA | + | NA | -4.27E-02 | 1.57E-01 | 7 | -148.162 | 310.324 | 1.588 | 0.189 |
| -0.681 | NA | NA | + | NA | NA | 2.30E-01 | 6 | -167.662 | 347.324 | 0 | 0.212 |
| -0.81 | NA | NA | + | -6.57E-04 | NA | 2.41E-01 | 7 | -166.686 | 347.371 | 0.048 | 0.207 |
| -0.655 | NA | NA | + | -7.50E-04 | -7.80E-02 | 2.42E-01 | 8 | -166.043 | 348.087 | 0.763 | 0.145 |
| -0.871 | 1.88E-09 | NA | + | -6.84E-04 | NA | 2.51E-01 | 8 | -166.189 | 348.378 | 1.054 | 0.125 |
| -0.732 | 1.72E-09 | NA | + | NA | NA | 2.38E-01 | 7 | -167.252 | 348.503 | 1.18 | 0.117 |
| -0.549 | NA | NA | + | NA | -5.90E-02 | 2.29E-01 | 7 | -167.288 | 348.576 | 1.252 | 0.113 |
| -0.721 | 1.72E-09 | NA | + | -7.70E-04 | -7.29E-02 | 2.51E-01 | 9 | -165.627 | 349.254 | 1.93 | 0.081 |
| -0.359 | NA | NA | + | NA | NA | 1.44E-01 | 6 | -140.361 | 292.721 | 0 | 0.364 |
| -0.3 | NA | NA | NA | NA | NA | 1.83E-01 | 3 | -143.997 | 293.994 | 1.273 | 0.193 |
| -0.384 | 7.90E-10 | NA | + | NA | NA | 1.50E-01 | 7 | -140.206 | 294.412 | 1.69 | 0.156 |
| -0.299 | NA | NA | + | NA | -2.79E-02 | 1.46E-01 | 7 | -140.273 | 294.546 | 1.825 | 0.146 |
| -0.394 | NA | NA | + | -1.44E-04 | NA | 1.48E-01 | 7 | -140.308 | 294.616 | 1.895 | 0.141 |
| -0.474 | NA | NA | + | NA | NA | 1.68E-01 | 6 | -159.532 | 331.063 | 0 | 0.453 |
| -0.506 | 1.13E-09 | NA | + | NA | NA | 1.73E-01 | 7 | -159.336 | 332.671 | 1.608 | 0.203 |
| -0.508 | NA | NA | + | -1.45E-04 | NA | 1.71E-01 | 7 | -159.486 | 332.973 | 1.909 | 0.175 |
| -0.448 | NA | NA | + | NA | -1.20E-02 | 1.68E-01 | 7 | -159.518 | 333.036 | 1.973 | 0.169 |
| -0.336 | NA | NA | + | NA | NA | 1.40E-01 | 6 | -137.432 | 286.864 | 0 | 0.228 |
| -0.272 | NA | NA | NA | NA | NA | 1.68E-01 | 3 | -140.531 | 287.062 | 0.198 | 0.207 |
| -0.21 | NA | NA | NA | 3.31E-04 | NA | 1.52E-01 | 4 | -140.035 | 288.07 | 1.206 | 0.125 |
| -0.358 | 7.27E-10 | NA | + | NA | NA | 1.44E-01 | 7 | -137.309 | 288.618 | 1.754 | 0.095 |
| -0.371 | NA | NA | + | -1.39E-04 | NA | 1.43E-01 | 7 | -137.383 | 288.765 | 1.901 | 0.088 |
| -0.294 | 7.56E-10 | NA | NA | NA | NA | 1.72E-01 | 4 | -140.403 | 288.805 | 1.941 | 0.086 |
| -0.208 | NA | NA | NA | NA | -3.32E-02 | 1.68E-01 | 4 | -140.405 | 288.81 | 1.946 | 0.086 |
| -0.34 | NA | NA | + | NA | 1.84E-03 | 1.40E-01 | 7 | -137.432 | 288.863 | 1.999 | 0.084 |
| -0.497 | NA | NA | + | NA | NA | 1.78E-01 | 6 | -149.1 | 310.201 | 0 | 0.413 |
| -0.533 | 1.18E-09 | NA | + | NA | NA | 1.84E-01 | 7 | -148.824 | 311.648 | 1.448 | 0.2 |
| -0.569 | NA | NA | + | -3.34E-04 | NA | 1.84E-01 | 7 | -148.831 | 311.661 | 1.461 | 0.199 |
| -0.403 | NA | NA | + | NA | -4.29E-02 | 1.80E-01 | 7 | -148.885 | 311.77 | 1.57 | 0.188 |
| -0.425 | NA | NA | + | NA | NA | 1.56E-01 | 6 | -147.356 | 306.711 | 0 | 0.409 |
| -0.529 | NA | NA | + | -4.62E-04 | NA | 1.66E-01 | 7 | -146.845 | 307.69 | 0.979 | 0.251 |
| -0.455 | 1.04E-09 | NA | + | NA | NA | 1.61E-01 | 7 | -147.176 | 308.352 | 1.641 | 0.18 |
| -0.371 | NA | NA | + | NA | -2.46E-02 | 1.57E-01 | 7 | -147.292 | 308.585 | 1.874 | 0.16 |
| -0.493 | NA | NA | + | NA | NA | 1.81E-01 | 6 | -142.233 | 296.467 | 0 | 0.196 |
| -0.317 | NA | NA | + | NA | -7.79E-02 | 1.82E-01 | 7 | -141.418 | 296.836 | 0.369 | 0.163 |
| -0.415 | NA | NA | + | -5.68E-04 | -8.92E-02 | 1.93E-01 | 8 | -140.513 | 297.025 | 0.558 | 0.148 |
| -0.598 | NA | NA | + | -4.83E-04 | NA | 1.90E-01 | 7 | -141.573 | 297.146 | 0.679 | 0.139 |
| -0.529 | 1.05E-09 | NA | + | NA | NA | 1.90E-01 | 7 | -141.895 | 297.79 | 1.323 | 0.101 |
| -0.477 | 1.23E-09 | NA | + | -6.31E-04 | -8.65E-02 | 2.03E-01 | 9 | -140.045 | 298.091 | 1.624 | 0.087 |
| -0.658 | 1.32E-09 | NA | + | -5.53E-04 | NA | 2.01E-01 | 8 | -141.048 | 298.096 | 1.63 | 0.087 |
| -0.356 | 9.45E-10 | NA | + | NA | -7.49E-02 | 1.90E-01 | 8 | -141.141 | 298.283 | 1.816 | 0.079 |
| -0.589 | NA | NA | + | NA | NA | 2.01E-01 | 6 | -157.275 | 326.55 | 0 | 0.21 |
| -0.71 | NA | NA | + | -6.15E-04 | NA | 2.09E-01 | 7 | -156.34 | 326.681 | 0.131 | 0.197 |
| -0.555 | NA | NA | + | -6.85E-04 | -7.67E-02 | 2.12E-01 | 8 | -155.695 | 327.39 | 0.84 | 0.138 |
| -0.777 | 1.57E-09 | NA | + | -6.81E-04 | NA | 2.20E-01 | 8 | -155.737 | 327.473 | 0.924 | 0.132 |
| -0.451 | NA | NA | + | NA | -6.28E-02 | 2.02E-01 | 7 | -156.84 | 327.68 | 1.13 | 0.119 |
| -0.632 | 1.28E-09 | NA | + | NA | NA | 2.09E-01 | 7 | -156.869 | 327.737 | 1.187 | 0.116 |
| -0.626 | 1.48E-09 | NA | + | -7.44E-04 | -7.29E-02 | 2.23E-01 | 9 | -155.151 | 328.301 | 1.752 | 0.087 |
| -0.449 | NA | NA | + | NA | NA | 1.68E-01 | 6 | -142.246 | 296.491 | 0 | 0.245 |
| -0.587 | NA | NA | + | -5.97E-04 | NA | 1.79E-01 | 7 | -141.296 | 296.593 | 0.101 | 0.233 |
| -0.452 | NA | NA | + | -6.54E-04 | -6.74E-02 | 1.82E-01 | 8 | -140.768 | 297.535 | 1.044 | 0.145 |
| -0.329 | NA | NA | + | NA | -5.49E-02 | 1.68E-01 | 7 | -141.894 | 297.788 | 1.297 | 0.128 |
| -0.634 | 1.44E-09 | NA | + | -6.25E-04 | NA | 1.86E-01 | 8 | -140.907 | 297.814 | 1.323 | 0.127 |
| -0.485 | 1.27E-09 | NA | + | NA | NA | 1.73E-01 | 7 | -141.947 | 297.895 | 1.403 | 0.122 |
| -0.399 | NA | NA | + | NA | NA | 1.45E-01 | 6 | -142.007 | 296.014 | 0 | 0.433 |
| -0.429 | 7.63E-10 | NA | + | NA | NA | 1.52E-01 | 7 | -141.792 | 297.584 | 1.57 | 0.198 |
| -0.463 | NA | NA | + | -2.74E-04 | NA | 1.49E-01 | 7 | -141.813 | 297.626 | 1.612 | 0.194 |
| -0.332 | NA | NA | + | NA | -2.96E-02 | 1.45E-01 | 7 | -141.912 | 297.824 | 1.809 | 0.175 |
| -0.397 | NA | NA | + | NA | NA | 1.53E-01 | 6 | -144.693 | 301.386 | 0 | 0.449 |
| -0.425 | 9.34E-10 | NA | + | NA | NA | 1.58E-01 | 7 | -144.511 | 303.023 | 1.637 | 0.198 |
| -0.339 | NA | NA | + | NA | -2.66E-02 | 1.53E-01 | 7 | -144.618 | 303.236 | 1.85 | 0.178 |
| -0.434 | NA | NA | + | -1.54E-04 | NA | 1.56E-01 | 7 | -144.636 | 303.272 | 1.886 | 0.175 |
| -0.586 | NA | NA | + | NA | NA | 1.97E-01 | 6 | -164.462 | 340.924 | 0 | 0.422 |
| -0.627 | 1.65E-09 | NA | + | NA | NA | 2.03E-01 | 7 | -164.166 | 342.332 | 1.408 | 0.209 |
| -0.663 | NA | NA | + | -3.71E-04 | NA | 2.03E-01 | 7 | -164.175 | 342.349 | 1.425 | 0.207 |
| -0.538 | NA | NA | + | NA | -2.16E-02 | 1.98E-01 | 7 | -164.416 | 342.831 | 1.907 | 0.163 |
| -0.5 | NA | NA | + | NA | NA | 1.82E-01 | 6 | -159.393 | 330.786 | 0 | 0.408 |
| -0.543 | 1.58E-09 | NA | + | NA | NA | 1.89E-01 | 7 | -159.046 | 332.093 | 1.307 | 0.212 |
| -0.386 | NA | NA | + | NA | -5.38E-02 | 1.84E-01 | 7 | -159.057 | 332.113 | 1.327 | 0.21 |
| -0.553 | NA | NA | + | -2.39E-04 | NA | 1.87E-01 | 7 | -159.269 | 332.539 | 1.753 | 0.17 |
| -0.757 | NA | NA | + | NA | NA | 2.45E-01 | 6 | -178.053 | 368.107 | 0 | 0.197 |
| -0.571 | NA | NA | + | NA | -7.99E-02 | 2.42E-01 | 7 | -177.334 | 368.667 | 0.561 | 0.149 |
| -0.656 | NA | NA | + | -6.84E-04 | -9.96E-02 | 2.52E-01 | 8 | -176.349 | 368.698 | 0.591 | 0.146 |
| -0.859 | NA | NA | + | -5.35E-04 | NA | 2.53E-01 | 7 | -177.43 | 368.861 | 0.754 | 0.135 |
| -0.811 | 1.96E-09 | NA | + | NA | NA | 2.53E-01 | 7 | -177.565 | 369.13 | 1.023 | 0.118 |
| -0.925 | 2.13E-09 | NA | + | -5.72E-04 | NA | 2.62E-01 | 8 | -176.853 | 369.705 | 1.599 | 0.088 |
| -0.726 | 1.91E-09 | NA | + | -7.09E-04 | -9.43E-02 | 2.61E-01 | 9 | -175.882 | 369.765 | 1.658 | 0.086 |
| -0.633 | 1.76E-09 | NA | + | NA | -7.44E-02 | 2.50E-01 | 8 | -176.943 | 369.886 | 1.779 | 0.081 |
| -0.448 | NA | NA | + | NA | NA | 1.66E-01 | 6 | -147.766 | 307.532 | 0 | 0.417 |
| -0.523 | NA | NA | + | -3.33E-04 | NA | 1.73E-01 | 7 | -147.489 | 308.977 | 1.445 | 0.202 |
| -0.481 | 1.02E-09 | NA | + | NA | NA | 1.72E-01 | 7 | -147.524 | 309.048 | 1.516 | 0.195 |
| -0.359 | NA | NA | + | NA | -4.08E-02 | 1.67E-01 | 7 | -147.575 | 309.149 | 1.617 | 0.186 |
| -0.723 | NA | NA | + | -7.01E-04 | NA | 2.21E-01 | 7 | -144.43 | 302.861 | 0 | 0.297 |
| -0.575 | NA | NA | + | -7.70E-04 | -7.39E-02 | 2.22E-01 | 8 | -143.751 | 303.503 | 0.642 | 0.215 |
| -0.584 | NA | NA | + | NA | NA | 2.08E-01 | 6 | -145.781 | 303.563 | 0.702 | 0.209 |
| -0.77 | 1.56E-09 | NA | + | -7.21E-04 | NA | 2.28E-01 | 8 | -144.039 | 304.077 | 1.216 | 0.162 |
| -0.457 | NA | NA | + | NA | -5.81E-02 | 2.08E-01 | 7 | -145.362 | 304.725 | 1.864 | 0.117 |
| -0.521 | NA | NA | + | NA | NA | 1.78E-01 | 6 | -151.692 | 315.384 | 0 | 0.391 |
| -0.625 | NA | NA | + | -4.84E-04 | NA | 1.87E-01 | 7 | -151.137 | 316.274 | 0.89 | 0.25 |
| -0.557 | 1.24E-09 | NA | + | NA | NA | 1.85E-01 | 7 | -151.436 | 316.872 | 1.487 | 0.186 |
| -0.426 | NA | NA | + | NA | -4.18E-02 | 1.78E-01 | 7 | -151.503 | 317.007 | 1.622 | 0.174 |
| -0.471 | NA | NA | + | NA | NA | 1.66E-01 | 6 | -147.919 | 307.839 | 0 | 0.406 |
| -0.35 | NA | NA | + | NA | -5.37E-02 | 1.67E-01 | 7 | -147.598 | 309.197 | 1.358 | 0.206 |
| -0.507 | 1.34E-09 | NA | + | NA | NA | 1.71E-01 | 7 | -147.643 | 309.286 | 1.447 | 0.197 |
| -0.546 | NA | NA | + | -3.15E-04 | NA | 1.73E-01 | 7 | -147.678 | 309.356 | 1.518 | 0.19 |
| -0.667 | NA | NA | + | -1.11E-03 | -1.21E-01 | 2.57E-01 | 8 | -154.147 | 324.294 | 0 | 0.489 |
| -0.738 | 1.85E-09 | NA | + | -1.14E-03 | -1.15E-01 | 2.67E-01 | 9 | -153.608 | 325.215 | 0.921 | 0.308 |
| -0.919 | NA | NA | + | -9.69E-04 | NA | 2.59E-01 | 7 | -156.026 | 326.051 | 1.757 | 0.203 |
| -0.508 | NA | NA | + | NA | NA | 1.83E-01 | 6 | -151.877 | 315.754 | 0 | 0.405 |
| -0.379 | NA | NA | + | NA | -5.77E-02 | 1.83E-01 | 7 | -151.491 | 316.981 | 1.228 | 0.219 |
| -0.543 | 1.31E-09 | NA | + | NA | NA | 1.88E-01 | 7 | -151.623 | 317.247 | 1.493 | 0.192 |
| -0.573 | NA | NA | + | -2.96E-04 | NA | 1.89E-01 | 7 | -151.667 | 317.333 | 1.58 | 0.184 |
| -0.414 | NA | NA | + | NA | NA | 1.51E-01 | 6 | -145.13 | 302.26 | 0 | 0.423 |
| -0.5 | NA | NA | + | -3.63E-04 | NA | 1.59E-01 | 7 | -144.805 | 303.611 | 1.351 | 0.215 |
| -0.442 | 9.86E-10 | NA | + | NA | NA | 1.56E-01 | 7 | -144.956 | 303.912 | 1.653 | 0.185 |
| -0.339 | NA | NA | + | NA | -3.45E-02 | 1.53E-01 | 7 | -145.002 | 304.003 | 1.744 | 0.177 |
| -0.445 | NA | NA | + | NA | NA | 1.67E-01 | 6 | -139.679 | 291.358 | 0 | 0.219 |
| -0.571 | NA | NA | + | -5.37E-04 | NA | 1.79E-01 | 7 | -138.879 | 291.759 | 0.401 | 0.179 |
| -0.406 | NA | NA | + | -6.12E-04 | -8.08E-02 | 1.80E-01 | 8 | -138.026 | 292.052 | 0.694 | 0.155 |
| -0.29 | NA | NA | + | NA | -6.89E-02 | 1.67E-01 | 7 | -139.054 | 292.107 | 0.749 | 0.151 |
| -0.482 | 1.18E-09 | NA | + | NA | NA | 1.74E-01 | 7 | -139.362 | 292.724 | 1.367 | 0.111 |
| -0.624 | 1.40E-09 | NA | + | -5.81E-04 | NA | 1.88E-01 | 8 | -138.435 | 292.87 | 1.512 | 0.103 |
| -0.462 | 1.29E-09 | NA | + | -6.48E-04 | -7.75E-02 | 1.88E-01 | 9 | -137.649 | 293.298 | 1.94 | 0.083 |
| -0.831 | NA | NA | + | -7.30E-04 | NA | 2.42E-01 | 7 | -156.287 | 326.574 | 0 | 0.224 |
| -0.654 | NA | NA | + | -8.27E-04 | -8.68E-02 | 2.44E-01 | 8 | -155.419 | 326.839 | 0.265 | 0.196 |
| -0.686 | NA | NA | + | NA | NA | 2.28E-01 | 6 | -157.63 | 327.26 | 0.686 | 0.159 |
| -0.886 | 1.72E-09 | NA | + | -7.61E-04 | NA | 2.52E-01 | 8 | -155.812 | 327.625 | 1.051 | 0.132 |
| -0.712 | 1.56E-09 | NA | + | -8.50E-04 | -8.27E-02 | 2.53E-01 | 9 | -155.024 | 328.048 | 1.474 | 0.107 |
| -0.535 | NA | NA | + | NA | -6.70E-02 | 2.28E-01 | 7 | -157.11 | 328.22 | 1.646 | 0.098 |
| -0.728 | 1.50E-09 | NA | + | NA | NA | 2.36E-01 | 7 | -157.272 | 328.543 | 1.969 | 0.084 |
| -0.469 | NA | NA | + | NA | NA | 1.72E-01 | 6 | -157.2 | 326.4 | 0 | 0.391 |
| -0.58 | NA | NA | + | -5.27E-04 | NA | 1.80E-01 | 7 | -156.57 | 327.14 | 0.74 | 0.27 |
| -0.505 | 1.27E-09 | NA | + | NA | NA | 1.78E-01 | 7 | -156.945 | 327.89 | 1.49 | 0.186 |
| -0.416 | NA | NA | + | NA | -2.50E-02 | 1.74E-01 | 7 | -157.134 | 328.269 | 1.868 | 0.154 |
| -0.526 | NA | NA | + | -8.87E-04 | -1.19E-01 | 2.26E-01 | 8 | -149.996 | 315.992 | 0 | 0.492 |
| -0.585 | 1.62E-09 | NA | + | -9.09E-04 | -1.15E-01 | 2.34E-01 | 9 | -149.549 | 317.098 | 1.106 | 0.283 |
| -0.767 | NA | NA | + | -7.43E-04 | NA | 2.25E-01 | 7 | -151.778 | 317.556 | 1.564 | 0.225 |
| -0.422 | NA | NA | + | NA | NA | 1.62E-01 | 6 | -144.16 | 300.319 | 0 | 0.437 |
| -0.493 | NA | NA | + | -3.02E-04 | NA | 1.68E-01 | 7 | -143.937 | 301.874 | 1.555 | 0.201 |
| -0.454 | 1.16E-09 | NA | + | NA | NA | 1.66E-01 | 7 | -143.951 | 301.902 | 1.583 | 0.198 |
| -0.39 | NA | NA | + | NA | -1.52E-02 | 1.63E-01 | 7 | -144.135 | 302.269 | 1.95 | 0.165 |
| -0.505 | NA | NA | + | NA | NA | 1.87E-01 | 6 | -158.558 | 329.115 | 0 | 0.449 |
| -0.54 | 1.40E-09 | NA | + | NA | NA | 1.92E-01 | 7 | -158.336 | 330.671 | 1.556 | 0.206 |
| -0.54 | NA | NA | + | -1.64E-04 | NA | 1.90E-01 | 7 | -158.5 | 330.999 | 1.884 | 0.175 |
| -0.469 | NA | NA | + | NA | -1.67E-02 | 1.87E-01 | 7 | -158.53 | 331.06 | 1.944 | 0.17 |
| -0.637 | NA | NA | + | NA | NA | 2.05E-01 | 6 | -158.118 | 328.237 | 0 | 0.199 |
| -0.769 | NA | NA | + | -6.33E-04 | NA | 2.16E-01 | 7 | -157.158 | 328.315 | 0.079 | 0.191 |
| -0.578 | NA | NA | + | -7.26E-04 | -8.87E-02 | 2.17E-01 | 8 | -156.282 | 328.563 | 0.326 | 0.169 |
| -0.466 | NA | NA | + | NA | -7.25E-02 | 2.04E-01 | 7 | -157.528 | 329.055 | 0.819 | 0.132 |
| -0.823 | 1.68E-09 | NA | + | -6.55E-04 | NA | 2.25E-01 | 8 | -156.709 | 329.419 | 1.182 | 0.11 |
| -0.683 | 1.55E-09 | NA | + | NA | NA | 2.12E-01 | 7 | -157.741 | 329.482 | 1.246 | 0.107 |
| -0.636 | 1.54E-09 | NA | + | -7.42E-04 | -8.50E-02 | 2.25E-01 | 9 | -155.903 | 329.807 | 1.57 | 0.091 |
| -0.379 | NA | NA | + | NA | NA | 1.42E-01 | 6 | -146.78 | 305.56 | 0 | 0.37 |
| -0.302 | NA | NA | NA | NA | NA | 1.79E-01 | 3 | -150.434 | 306.869 | 1.309 | 0.192 |
| -0.408 | 9.89E-10 | NA | + | NA | NA | 1.47E-01 | 7 | -146.597 | 307.194 | 1.634 | 0.163 |
| -0.398 | NA | NA | + | -8.47E-05 | NA | 1.44E-01 | 7 | -146.764 | 307.528 | 1.968 | 0.138 |
| -0.38 | NA | NA | + | NA | 4.72E-04 | 1.42E-01 | 7 | -146.78 | 307.56 | 2 | 0.136 |
| -0.363 | NA | NA | + | NA | NA | 1.41E-01 | 6 | -149.689 | 311.378 | 0 | 0.289 |
| -0.299 | NA | NA | NA | NA | NA | 1.78E-01 | 3 | -152.996 | 311.991 | 0.613 | 0.213 |
| -0.213 | NA | NA | NA | 4.35E-04 | NA | 1.55E-01 | 4 | -152.329 | 312.659 | 1.28 | 0.152 |
| -0.393 | 9.21E-10 | NA | + | NA | NA | 1.47E-01 | 7 | -149.513 | 313.026 | 1.648 | 0.127 |
| -0.31 | NA | NA | + | NA | -2.48E-02 | 1.42E-01 | 7 | -149.627 | 313.254 | 1.875 | 0.113 |
| -0.369 | NA | NA | + | -2.39E-05 | NA | 1.42E-01 | 7 | -149.688 | 313.376 | 1.997 | 0.106 |
| -0.549 | NA | NA | + | NA | NA | 1.88E-01 | 6 | -156.142 | 324.285 | 0 | 0.29 |
| -0.677 | NA | NA | + | -5.79E-04 | NA | 1.99E-01 | 7 | -155.375 | 324.749 | 0.465 | 0.23 |
| -0.584 | 1.34E-09 | NA | + | NA | NA | 1.93E-01 | 7 | -155.921 | 325.843 | 1.558 | 0.133 |
| -0.461 | NA | NA | + | NA | -3.92E-02 | 1.88E-01 | 7 | -155.985 | 325.971 | 1.686 | 0.125 |
| -0.722 | 1.52E-09 | NA | + | -6.03E-04 | NA | 2.05E-01 | 8 | -155.09 | 326.181 | 1.896 | 0.113 |
| -0.574 | NA | NA | + | -6.15E-04 | -4.92E-02 | 2.00E-01 | 8 | -155.128 | 326.255 | 1.971 | 0.108 |
| -0.401 | NA | NA | + | NA | NA | 1.55E-01 | 6 | -158.109 | 328.219 | 0 | 0.289 |
| -0.353 | NA | NA | NA | NA | NA | 1.94E-01 | 3 | -161.514 | 329.027 | 0.809 | 0.193 |
| -0.253 | NA | NA | NA | 5.13E-04 | NA | 1.69E-01 | 4 | -160.614 | 329.229 | 1.01 | 0.175 |
| -0.431 | 9.57E-10 | NA | + | NA | NA | 1.61E-01 | 7 | -157.922 | 329.843 | 1.625 | 0.128 |
| -0.424 | NA | NA | + | NA | 1.13E-02 | 1.54E-01 | 7 | -158.097 | 330.195 | 1.976 | 0.108 |
| -0.392 | NA | NA | + | 3.72E-05 | NA | 1.54E-01 | 7 | -158.106 | 330.213 | 1.994 | 0.107 |
| -0.423 | NA | NA | + | NA | NA | 1.53E-01 | 6 | -149.285 | 310.57 | 0 | 0.444 |
| -0.491 | NA | NA | + | -2.98E-04 | NA | 1.59E-01 | 7 | -149.086 | 312.172 | 1.602 | 0.199 |
| -0.452 | 8.72E-10 | NA | + | NA | NA | 1.58E-01 | 7 | -149.121 | 312.242 | 1.673 | 0.192 |
| -0.445 | NA | NA | + | NA | 9.93E-03 | 1.53E-01 | 7 | -149.275 | 312.551 | 1.981 | 0.165 |
| -0.673 | NA | NA | + | -6.44E-04 | NA | 2.04E-01 | 7 | -142.127 | 298.253 | 0 | 0.259 |
| -0.534 | NA | NA | + | NA | NA | 1.92E-01 | 6 | -143.241 | 298.482 | 0.229 | 0.231 |
| -0.549 | NA | NA | + | -7.00E-04 | -6.27E-02 | 2.07E-01 | 8 | -141.648 | 299.295 | 1.042 | 0.154 |
| -0.715 | 1.50E-09 | NA | + | -6.56E-04 | NA | 2.11E-01 | 8 | -141.809 | 299.618 | 1.365 | 0.131 |
| -0.427 | NA | NA | + | NA | -4.93E-02 | 1.93E-01 | 7 | -142.946 | 299.891 | 1.638 | 0.114 |
| -0.57 | 1.40E-09 | NA | + | NA | NA | 1.97E-01 | 7 | -142.968 | 299.935 | 1.682 | 0.112 |
| -0.278 | NA | NA | + | NA | NA | 1.23E-01 | 6 | -135.447 | 282.894 | 0 | 0.215 |
| -0.249 | NA | NA | NA | NA | NA | 1.59E-01 | 3 | -138.473 | 282.946 | 0.052 | 0.209 |
| -0.17 | NA | NA | NA | 3.41E-04 | NA | 1.37E-01 | 4 | -137.965 | 283.93 | 1.036 | 0.128 |
| -0.149 | NA | NA | NA | NA | -4.74E-02 | 1.56E-01 | 4 | -138.206 | 284.413 | 1.519 | 0.101 |
| -0.303 | 8.68E-10 | NA | + | NA | NA | 1.27E-01 | 7 | -135.299 | 284.598 | 1.704 | 0.092 |
| -0.275 | 9.17E-10 | NA | NA | NA | NA | 1.63E-01 | 4 | -138.314 | 284.628 | 1.734 | 0.09 |
| -0.228 | NA | NA | + | NA | -2.33E-02 | 1.24E-01 | 7 | -135.385 | 284.771 | 1.877 | 0.084 |
| -0.304 | NA | NA | + | -9.87E-05 | NA | 1.26E-01 | 7 | -135.423 | 284.845 | 1.951 | 0.081 |
| -0.452 | NA | NA | + | NA | NA | 1.72E-01 | 6 | -146.854 | 305.708 | 0 | 0.425 |
| -0.485 | 1.12E-09 | NA | + | NA | NA | 1.78E-01 | 7 | -146.585 | 307.169 | 1.462 | 0.205 |
| -0.514 | NA | NA | + | -2.73E-04 | NA | 1.77E-01 | 7 | -146.669 | 307.339 | 1.631 | 0.188 |
| -0.369 | NA | NA | + | NA | -3.80E-02 | 1.73E-01 | 7 | -146.699 | 307.398 | 1.69 | 0.183 |
| -0.411 | NA | NA | + | NA | NA | 1.49E-01 | 6 | -151.6 | 315.2 | 0 | 0.449 |
| -0.44 | 1.10E-09 | NA | + | NA | NA | 1.53E-01 | 7 | -151.43 | 316.861 | 1.661 | 0.196 |
| -0.468 | NA | NA | + | -2.45E-04 | NA | 1.54E-01 | 7 | -151.469 | 316.937 | 1.737 | 0.189 |
| -0.395 | NA | NA | + | NA | -7.17E-03 | 1.49E-01 | 7 | -151.595 | 317.19 | 1.99 | 0.166 |
| -0.453 | NA | NA | + | NA | NA | 1.68E-01 | 6 | -146.566 | 305.133 | 0 | 0.414 |
| -0.542 | NA | NA | + | -3.86E-04 | NA | 1.76E-01 | 7 | -146.196 | 306.393 | 1.26 | 0.22 |
| -0.483 | 9.74E-10 | NA | + | NA | NA | 1.73E-01 | 7 | -146.36 | 306.72 | 1.587 | 0.187 |
| -0.37 | NA | NA | + | NA | -3.77E-02 | 1.69E-01 | 7 | -146.407 | 306.814 | 1.682 | 0.179 |
| -0.559 | NA | NA | + | NA | NA | 1.91E-01 | 6 | -155.265 | 322.53 | 0 | 0.275 |
| -0.667 | NA | NA | + | -5.35E-04 | NA | 1.99E-01 | 7 | -154.556 | 323.111 | 0.581 | 0.206 |
| -0.598 | 1.23E-09 | NA | + | NA | NA | 1.99E-01 | 7 | -154.918 | 323.835 | 1.305 | 0.143 |
| -0.446 | NA | NA | + | NA | -5.18E-02 | 1.93E-01 | 7 | -154.966 | 323.933 | 1.402 | 0.136 |
| -0.723 | 1.44E-09 | NA | + | -5.84E-04 | NA | 2.09E-01 | 8 | -154.078 | 324.155 | 1.625 | 0.122 |
| -0.541 | NA | NA | + | -5.90E-04 | -6.31E-02 | 2.03E-01 | 8 | -154.115 | 324.229 | 1.699 | 0.118 |
| -0.362 | NA | NA | + | NA | NA | 1.41E-01 | 6 | -147.308 | 306.616 | 0 | 0.374 |
| -0.308 | NA | NA | NA | NA | NA | 1.79E-01 | 3 | -150.995 | 307.99 | 1.373 | 0.188 |
| -0.386 | 9.31E-10 | NA | + | NA | NA | 1.45E-01 | 7 | -147.184 | 308.369 | 1.753 | 0.156 |
| -0.392 | NA | NA | + | -1.25E-04 | NA | 1.44E-01 | 7 | -147.273 | 308.546 | 1.93 | 0.142 |
| -0.395 | NA | NA | + | NA | 1.55E-02 | 1.41E-01 | 7 | -147.285 | 308.571 | 1.955 | 0.141 |
| -0.558 | NA | NA | + | NA | NA | 1.92E-01 | 6 | -154.648 | 321.297 | 0 | 0.333 |
| -0.671 | NA | NA | + | -5.21E-04 | NA | 2.02E-01 | 7 | -153.997 | 321.994 | 0.698 | 0.235 |
| -0.593 | 1.05E-09 | NA | + | NA | NA | 1.99E-01 | 7 | -154.4 | 322.799 | 1.503 | 0.157 |
| -0.463 | NA | NA | + | NA | -4.34E-02 | 1.93E-01 | 7 | -154.45 | 322.9 | 1.604 | 0.149 |
| -0.724 | 1.28E-09 | NA | + | -5.69E-04 | NA | 2.12E-01 | 8 | -153.63 | 323.261 | 1.964 | 0.125 |
| -0.709 | NA | NA | + | -8.03E-04 | NA | 1.98E-01 | 7 | -140.871 | 295.742 | 0 | 0.389 |
| -0.758 | 1.60E-09 | NA | + | -8.31E-04 | NA | 2.06E-01 | 8 | -140.425 | 296.851 | 1.109 | 0.223 |
| -0.597 | NA | NA | + | -8.54E-04 | -5.65E-02 | 2.02E-01 | 8 | -140.492 | 296.983 | 1.241 | 0.209 |
| -0.544 | NA | NA | + | NA | NA | 1.87E-01 | 6 | -142.647 | 297.293 | 1.551 | 0.179 |
| -0.5 | NA | NA | + | NA | NA | 1.76E-01 | 6 | -149.338 | 310.676 | 0 | 0.319 |
| -0.612 | NA | NA | + | -5.24E-04 | NA | 1.87E-01 | 7 | -148.65 | 311.301 | 0.625 | 0.234 |
| -0.385 | NA | NA | + | NA | -5.31E-02 | 1.78E-01 | 7 | -149.016 | 312.032 | 1.356 | 0.162 |
| -0.537 | 1.24E-09 | NA | + | NA | NA | 1.83E-01 | 7 | -149.083 | 312.167 | 1.491 | 0.152 |
| -0.486 | NA | NA | + | -5.67E-04 | -6.17E-02 | 1.89E-01 | 8 | -148.215 | 312.431 | 1.755 | 0.133 |
| -0.522 | NA | NA | + | -8.19E-04 | -1.05E-01 | 2.18E-01 | 8 | -146.964 | 309.928 | 0 | 0.271 |
| -0.731 | NA | NA | + | -7.13E-04 | NA | 2.16E-01 | 7 | -148.34 | 310.68 | 0.752 | 0.186 |
| -0.586 | 1.48E-09 | NA | + | -8.68E-04 | -1.02E-01 | 2.28E-01 | 9 | -146.434 | 310.868 | 0.94 | 0.169 |
| -0.589 | NA | NA | + | NA | NA | 2.05E-01 | 6 | -149.717 | 311.433 | 1.506 | 0.128 |
| -0.793 | 1.60E-09 | NA | + | -7.70E-04 | NA | 2.27E-01 | 8 | -147.733 | 311.466 | 1.538 | 0.125 |
| -0.398 | NA | NA | + | NA | -8.70E-02 | 2.05E-01 | 7 | -148.768 | 311.536 | 1.608 | 0.121 |
| -0.483 | NA | NA | + | -7.77E-04 | -9.66E-02 | 2.01E-01 | 8 | -142.271 | 300.541 | 0 | 0.245 |
| -0.671 | NA | NA | + | -6.66E-04 | NA | 1.96E-01 | 7 | -143.429 | 300.859 | 0.318 | 0.209 |
| -0.524 | NA | NA | + | NA | NA | 1.85E-01 | 6 | -144.661 | 301.323 | 0.782 | 0.166 |
| -0.531 | 1.36E-09 | NA | + | -7.94E-04 | -9.42E-02 | 2.07E-01 | 9 | -141.889 | 301.778 | 1.237 | 0.132 |
| -0.355 | NA | NA | + | NA | -7.68E-02 | 1.87E-01 | 7 | -143.92 | 301.84 | 1.299 | 0.128 |
| -0.718 | 1.46E-09 | NA | + | -6.87E-04 | NA | 2.03E-01 | 8 | -142.996 | 301.991 | 1.45 | 0.119 |
| -0.424 | NA | NA | + | NA | NA | 1.56E-01 | 6 | -156.572 | 325.144 | 0 | 0.454 |
| -0.452 | 9.16E-10 | NA | + | NA | NA | 1.60E-01 | 7 | -156.425 | 326.851 | 1.707 | 0.193 |
| -0.47 | NA | NA | + | -1.93E-04 | NA | 1.60E-01 | 7 | -156.491 | 326.982 | 1.839 | 0.181 |
| -0.385 | NA | NA | + | NA | -1.78E-02 | 1.56E-01 | 7 | -156.542 | 327.083 | 1.94 | 0.172 |
| -0.539 | NA | NA | + | NA | NA | 1.91E-01 | 6 | -146.192 | 304.385 | 0 | 0.24 |
| -0.658 | NA | NA | + | -5.49E-04 | NA | 2.03E-01 | 7 | -145.398 | 304.795 | 0.411 | 0.196 |
| -0.488 | NA | NA | + | -6.27E-04 | -8.26E-02 | 2.05E-01 | 8 | -144.565 | 305.131 | 0.746 | 0.165 |
| -0.381 | NA | NA | + | NA | -6.99E-02 | 1.91E-01 | 7 | -145.592 | 305.184 | 0.799 | 0.161 |
| -0.579 | 1.33E-09 | NA | + | NA | NA | 1.99E-01 | 7 | -145.842 | 305.684 | 1.3 | 0.125 |
| -0.71 | 1.49E-09 | NA | + | -5.79E-04 | NA | 2.12E-01 | 8 | -144.957 | 305.914 | 1.529 | 0.112 |
| -0.777 | NA | NA | + | -7.14E-04 | NA | 2.29E-01 | 7 | -158.745 | 331.49 | 0 | 0.223 |
| -0.634 | NA | NA | + | NA | NA | 2.16E-01 | 6 | -159.97 | 331.94 | 0.45 | 0.178 |
| -0.612 | NA | NA | + | -8.08E-04 | -8.14E-02 | 2.30E-01 | 8 | -157.992 | 331.983 | 0.493 | 0.174 |
| -0.833 | 1.85E-09 | NA | + | -7.40E-04 | NA | 2.38E-01 | 8 | -158.267 | 332.534 | 1.044 | 0.132 |
| -0.494 | NA | NA | + | NA | -6.21E-02 | 2.15E-01 | 7 | -159.527 | 333.053 | 1.563 | 0.102 |
| -0.68 | 1.67E-09 | NA | + | NA | NA | 2.24E-01 | 7 | -159.585 | 333.171 | 1.681 | 0.096 |
| -0.672 | 1.69E-09 | NA | + | -8.26E-04 | -7.71E-02 | 2.38E-01 | 9 | -157.591 | 333.182 | 1.692 | 0.096 |
| -0.471 | NA | NA | + | NA | NA | 1.70E-01 | 6 | -162.671 | 337.342 | 0 | 0.446 |
| -0.505 | 1.15E-09 | NA | + | NA | NA | 1.76E-01 | 7 | -162.445 | 338.889 | 1.548 | 0.206 |
| -0.41 | NA | NA | + | NA | -2.74E-02 | 1.71E-01 | 7 | -162.6 | 339.199 | 1.858 | 0.176 |
| -0.501 | NA | NA | + | -1.40E-04 | NA | 1.73E-01 | 7 | -162.629 | 339.258 | 1.917 | 0.171 |
| -0.609 | NA | NA | + | -8.71E-04 | -1.13E-01 | 2.45E-01 | 8 | -162.002 | 340.005 | 0 | 0.288 |
| -0.682 | 1.73E-09 | NA | + | -9.08E-04 | -1.08E-01 | 2.56E-01 | 9 | -161.478 | 340.956 | 0.951 | 0.179 |
| -0.844 | NA | NA | + | -7.36E-04 | NA | 2.46E-01 | 7 | -163.552 | 341.105 | 1.1 | 0.166 |
| -0.482 | NA | NA | + | NA | -9.22E-02 | 2.30E-01 | 7 | -163.825 | 341.651 | 1.646 | 0.127 |
| -0.696 | NA | NA | + | NA | NA | 2.32E-01 | 6 | -164.87 | 341.74 | 1.736 | 0.121 |
| -0.914 | 1.95E-09 | NA | + | -7.85E-04 | NA | 2.58E-01 | 8 | -162.893 | 341.786 | 1.781 | 0.118 |
| -0.478 | NA | NA | + | NA | NA | 1.68E-01 | 6 | -150.973 | 313.946 | 0 | 0.418 |
| -0.518 | 1.35E-09 | NA | + | NA | NA | 1.75E-01 | 7 | -150.682 | 315.363 | 1.417 | 0.206 |
| -0.552 | NA | NA | + | -3.23E-04 | NA | 1.75E-01 | 7 | -150.728 | 315.456 | 1.51 | 0.196 |
| -0.39 | NA | NA | + | NA | -3.84E-02 | 1.68E-01 | 7 | -150.812 | 315.624 | 1.677 | 0.18 |
| -0.481 | NA | NA | + | NA | NA | 1.72E-01 | 6 | -155.489 | 322.978 | 0 | 0.441 |
| -0.514 | 1.09E-09 | NA | + | NA | NA | 1.79E-01 | 7 | -155.256 | 324.511 | 1.533 | 0.205 |
| -0.53 | NA | NA | + | -2.30E-04 | NA | 1.76E-01 | 7 | -155.37 | 324.741 | 1.763 | 0.183 |
| -0.431 | NA | NA | + | NA | -2.23E-02 | 1.72E-01 | 7 | -155.439 | 324.877 | 1.899 | 0.171 |
| -0.367 | NA | NA | + | NA | NA | 1.46E-01 | 6 | -146.195 | 304.39 | 0 | 0.296 |
| -0.311 | NA | NA | NA | NA | NA | 1.76E-01 | 3 | -149.557 | 305.114 | 0.724 | 0.206 |
| -0.235 | NA | NA | NA | 4.09E-04 | NA | 1.57E-01 | 4 | -148.894 | 305.787 | 1.397 | 0.147 |
| -0.394 | 8.96E-10 | NA | + | NA | NA | 1.51E-01 | 7 | -146.024 | 306.048 | 1.658 | 0.129 |
| -0.39 | NA | NA | + | -9.84E-05 | NA | 1.48E-01 | 7 | -146.173 | 306.346 | 1.956 | 0.111 |
| -0.358 | NA | NA | + | NA | -4.45E-03 | 1.47E-01 | 7 | -146.193 | 306.386 | 1.996 | 0.109 |
| -0.496 | NA | NA | + | NA | NA | 1.83E-01 | 6 | -141.278 | 294.556 | 0 | 0.385 |
| -0.592 | NA | NA | + | -4.67E-04 | NA | 1.91E-01 | 7 | -140.7 | 295.401 | 0.845 | 0.252 |
| -0.529 | 1.11E-09 | NA | + | NA | NA | 1.89E-01 | 7 | -141 | 295.999 | 1.443 | 0.187 |
| -0.406 | NA | NA | + | NA | -4.25E-02 | 1.85E-01 | 7 | -141.067 | 296.133 | 1.577 | 0.175 |
| -0.56 | NA | NA | + | NA | NA | 1.91E-01 | 6 | -156.982 | 325.964 | 0 | 0.325 |
| -0.657 | NA | NA | + | -4.76E-04 | NA | 1.99E-01 | 7 | -156.454 | 326.909 | 0.945 | 0.202 |
| -0.425 | NA | NA | + | NA | -6.03E-02 | 1.91E-01 | 7 | -156.582 | 327.164 | 1.2 | 0.178 |
| -0.601 | 1.31E-09 | NA | + | NA | NA | 1.99E-01 | 7 | -156.652 | 327.304 | 1.341 | 0.166 |
| -0.513 | NA | NA | + | -5.40E-04 | -7.06E-02 | 2.01E-01 | 8 | -155.911 | 327.821 | 1.858 | 0.128 |
| -0.671 | NA | NA | + | -6.89E-04 | NA | 1.95E-01 | 7 | -143.716 | 301.433 | 0 | 0.236 |
| -0.524 | NA | NA | + | NA | NA | 1.82E-01 | 6 | -144.989 | 301.979 | 0.546 | 0.18 |
| -0.518 | NA | NA | + | -7.49E-04 | -7.64E-02 | 1.99E-01 | 8 | -143.031 | 302.063 | 0.63 | 0.172 |
| -0.718 | 1.40E-09 | NA | + | -7.17E-04 | NA | 2.03E-01 | 8 | -143.318 | 302.636 | 1.203 | 0.129 |
| -0.388 | NA | NA | + | NA | -6.30E-02 | 1.85E-01 | 7 | -144.525 | 303.051 | 1.618 | 0.105 |
| -0.568 | 1.30E-09 | NA | + | -7.72E-04 | -7.33E-02 | 2.06E-01 | 9 | -142.686 | 303.372 | 1.939 | 0.089 |
| -0.56 | 1.21E-09 | NA | + | NA | NA | 1.89E-01 | 7 | -144.693 | 303.386 | 1.953 | 0.089 |
| -0.414 | NA | NA | + | NA | NA | 1.51E-01 | 6 | -141.563 | 295.126 | 0 | 0.418 |
| -0.502 | NA | NA | + | -3.75E-04 | NA | 1.59E-01 | 7 | -141.216 | 296.432 | 1.305 | 0.218 |
| -0.447 | 1.06E-09 | NA | + | NA | NA | 1.57E-01 | 7 | -141.328 | 296.656 | 1.529 | 0.194 |
| -0.349 | NA | NA | + | NA | -3.01E-02 | 1.53E-01 | 7 | -141.462 | 296.924 | 1.797 | 0.17 |
| -0.473 | NA | NA | + | NA | NA | 1.71E-01 | 6 | -144.532 | 301.064 | 0 | 0.396 |
| -0.579 | NA | NA | + | -4.69E-04 | NA | 1.81E-01 | 7 | -143.968 | 301.937 | 0.872 | 0.256 |
| -0.504 | 1.03E-09 | NA | + | NA | NA | 1.77E-01 | 7 | -144.319 | 302.638 | 1.574 | 0.18 |
| -0.392 | NA | NA | + | NA | -3.65E-02 | 1.72E-01 | 7 | -144.385 | 302.769 | 1.705 | 0.169 |
| -0.413 | NA | NA | + | NA | NA | 1.60E-01 | 6 | -146.235 | 304.47 | 0 | 0.414 |
| -0.498 | NA | NA | + | -3.73E-04 | NA | 1.68E-01 | 7 | -145.895 | 305.791 | 1.321 | 0.214 |
| -0.443 | 9.94E-10 | NA | + | NA | NA | 1.65E-01 | 7 | -146.018 | 306.035 | 1.566 | 0.189 |
| -0.327 | NA | NA | + | NA | -4.06E-02 | 1.62E-01 | 7 | -146.048 | 306.096 | 1.626 | 0.183 |
| -0.528 | NA | NA | + | -8.05E-04 | -1.02E-01 | 2.18E-01 | 8 | -149.21 | 314.419 | 0 | 0.25 |
| -0.737 | NA | NA | + | -7.00E-04 | NA | 2.17E-01 | 7 | -150.509 | 315.017 | 0.598 | 0.186 |
| -0.599 | 1.50E-09 | NA | + | -8.66E-04 | -9.88E-02 | 2.30E-01 | 9 | -148.565 | 315.131 | 0.712 | 0.175 |
| -0.805 | 1.58E-09 | NA | + | -7.68E-04 | NA | 2.30E-01 | 8 | -149.798 | 315.596 | 1.177 | 0.139 |
| -0.603 | NA | NA | + | NA | NA | 2.06E-01 | 6 | -151.847 | 315.694 | 1.275 | 0.132 |
| -0.414 | NA | NA | + | NA | -8.39E-02 | 2.06E-01 | 7 | -150.961 | 315.922 | 1.503 | 0.118 |
| -0.476 | NA | NA | + | NA | NA | 1.70E-01 | 6 | -154.4 | 320.799 | 0 | 0.396 |
| -0.591 | NA | NA | + | -5.25E-04 | NA | 1.78E-01 | 7 | -153.758 | 321.516 | 0.716 | 0.277 |
| -0.508 | 9.55E-10 | NA | + | NA | NA | 1.76E-01 | 7 | -154.205 | 322.41 | 1.611 | 0.177 |
| -0.443 | NA | NA | + | NA | -1.55E-02 | 1.70E-01 | 7 | -154.375 | 322.751 | 1.951 | 0.149 |
| -0.356 | NA | NA | + | NA | NA | 1.40E-01 | 6 | -143.697 | 299.395 | 0 | 0.27 |
| -0.281 | NA | NA | NA | NA | NA | 1.74E-01 | 3 | -146.834 | 299.669 | 0.274 | 0.236 |
| -0.196 | NA | NA | NA | 4.31E-04 | NA | 1.51E-01 | 4 | -146.103 | 300.205 | 0.81 | 0.18 |
| -0.379 | 7.86E-10 | NA | + | NA | NA | 1.44E-01 | 7 | -143.578 | 301.157 | 1.762 | 0.112 |
| -0.386 | NA | NA | + | NA | 1.41E-02 | 1.39E-01 | 7 | -143.677 | 301.355 | 1.96 | 0.102 |
| -0.361 | NA | NA | + | -2.25E-05 | NA | 1.40E-01 | 7 | -143.696 | 301.393 | 1.998 | 0.1 |
| -0.474 | NA | NA | + | NA | NA | 1.67E-01 | 6 | -156.021 | 324.043 | 0 | 0.434 |
| -0.551 | NA | NA | + | -3.51E-04 | NA | 1.73E-01 | 7 | -155.749 | 325.499 | 1.456 | 0.21 |
| -0.507 | 1.10E-09 | NA | + | NA | NA | 1.73E-01 | 7 | -155.818 | 325.636 | 1.593 | 0.196 |
| -0.459 | NA | NA | + | NA | -6.98E-03 | 1.68E-01 | 7 | -156.017 | 326.034 | 1.991 | 0.16 |
| -0.593 | NA | NA | + | NA | NA | 2.02E-01 | 6 | -159.353 | 330.706 | 0 | 0.197 |
| -0.408 | NA | NA | + | NA | -8.14E-02 | 2.02E-01 | 7 | -158.56 | 331.121 | 0.415 | 0.16 |
| -0.512 | NA | NA | + | -6.13E-04 | -9.45E-02 | 2.15E-01 | 8 | -157.68 | 331.359 | 0.653 | 0.142 |
| -0.704 | NA | NA | + | -5.11E-04 | NA | 2.13E-01 | 7 | -158.734 | 331.468 | 0.762 | 0.135 |
| -0.645 | 1.89E-09 | NA | + | NA | NA | 2.09E-01 | 7 | -158.875 | 331.751 | 1.045 | 0.117 |
| -0.467 | 1.68E-09 | NA | + | NA | -7.60E-02 | 2.09E-01 | 8 | -158.187 | 332.373 | 1.667 | 0.086 |
| -0.764 | 2.00E-09 | NA | + | -5.33E-04 | NA | 2.21E-01 | 8 | -158.2 | 332.4 | 1.694 | 0.084 |
| -0.575 | 1.76E-09 | NA | + | -6.26E-04 | -8.92E-02 | 2.22E-01 | 9 | -157.263 | 332.527 | 1.821 | 0.079 |
| -0.426 | NA | NA | + | NA | NA | 1.58E-01 | 6 | -136.555 | 285.11 | 0 | 0.32 |
| -0.556 | NA | NA | + | -5.53E-04 | NA | 1.65E-01 | 7 | -135.754 | 285.507 | 0.397 | 0.263 |
| -0.455 | 1.01E-09 | NA | + | NA | NA | 1.61E-01 | 7 | -136.368 | 286.736 | 1.626 | 0.142 |
| -0.345 | NA | NA | + | NA | -3.82E-02 | 1.62E-01 | 7 | -136.376 | 286.752 | 1.642 | 0.141 |
| -0.459 | NA | NA | + | -6.09E-04 | -5.23E-02 | 1.71E-01 | 8 | -135.423 | 286.846 | 1.736 | 0.134 |
| -0.517 | NA | NA | + | NA | NA | 1.83E-01 | 6 | -158.616 | 329.232 | 0 | 0.417 |
| -0.602 | NA | NA | + | -3.89E-04 | NA | 1.90E-01 | 7 | -158.267 | 330.535 | 1.303 | 0.218 |
| -0.552 | 1.22E-09 | NA | + | NA | NA | 1.90E-01 | 7 | -158.376 | 330.752 | 1.521 | 0.195 |
| -0.448 | NA | NA | + | NA | -3.22E-02 | 1.85E-01 | 7 | -158.513 | 331.026 | 1.794 | 0.17 |
| -0.534 | NA | NA | + | -8.45E-04 | -1.11E-01 | 2.17E-01 | 8 | -150.694 | 317.388 | 0 | 0.283 |
| -0.608 | 1.50E-09 | NA | + | -9.06E-04 | -1.08E-01 | 2.30E-01 | 9 | -150.024 | 318.047 | 0.659 | 0.204 |
| -0.764 | NA | NA | + | -7.30E-04 | NA | 2.15E-01 | 7 | -152.245 | 318.489 | 1.101 | 0.163 |
| -0.836 | 1.59E-09 | NA | + | -7.99E-04 | NA | 2.29E-01 | 8 | -151.503 | 319.006 | 1.618 | 0.126 |
| -0.401 | NA | NA | + | NA | -9.29E-02 | 2.03E-01 | 7 | -152.581 | 319.161 | 1.773 | 0.117 |
| -0.613 | NA | NA | + | NA | NA | 2.03E-01 | 6 | -153.662 | 319.325 | 1.936 | 0.107 |
| -0.544 | NA | NA | + | -7.81E-04 | -9.41E-02 | 2.20E-01 | 8 | -156.198 | 328.395 | 0 | 0.195 |
| -0.73 | NA | NA | + | -6.80E-04 | NA | 2.17E-01 | 7 | -157.233 | 328.466 | 0.071 | 0.188 |
| -0.595 | NA | NA | + | NA | NA | 2.07E-01 | 6 | -158.348 | 328.697 | 0.302 | 0.168 |
| -0.427 | NA | NA | + | NA | -7.70E-02 | 2.08E-01 | 7 | -157.651 | 329.301 | 0.906 | 0.124 |
| -0.788 | 1.82E-09 | NA | + | -7.16E-04 | NA | 2.27E-01 | 8 | -156.691 | 329.383 | 0.988 | 0.119 |
| -0.606 | 1.63E-09 | NA | + | -8.08E-04 | -8.93E-02 | 2.28E-01 | 9 | -155.76 | 329.52 | 1.125 | 0.111 |
| -0.64 | 1.61E-09 | NA | + | NA | NA | 2.15E-01 | 7 | -157.929 | 329.858 | 1.463 | 0.094 |
| -0.586 | NA | NA | + | NA | NA | 1.97E-01 | 6 | -166.483 | 344.965 | 0 | 0.404 |
| -0.679 | NA | NA | + | -4.42E-04 | NA | 2.06E-01 | 7 | -166.066 | 346.132 | 1.166 | 0.226 |
| -0.622 | 1.32E-09 | NA | + | NA | NA | 2.03E-01 | 7 | -166.229 | 346.458 | 1.492 | 0.192 |
| -0.491 | NA | NA | + | NA | -4.32E-02 | 1.98E-01 | 7 | -166.3 | 346.6 | 1.635 | 0.178 |
| -0.497 | NA | NA | + | NA | NA | 1.78E-01 | 6 | -156.305 | 324.61 | 0 | 0.437 |
| -0.531 | 1.14E-09 | NA | + | NA | NA | 1.85E-01 | 7 | -156.073 | 326.145 | 1.535 | 0.203 |
| -0.564 | NA | NA | + | -2.91E-04 | NA | 1.85E-01 | 7 | -156.114 | 326.228 | 1.619 | 0.194 |
| -0.458 | NA | NA | + | NA | -1.82E-02 | 1.79E-01 | 7 | -156.272 | 326.544 | 1.934 | 0.166 |
| -0.566 | NA | NA | + | NA | NA | 1.93E-01 | 6 | -157.315 | 326.63 | 0 | 0.378 |
| -0.667 | NA | NA | + | -4.77E-04 | NA | 2.01E-01 | 7 | -156.795 | 327.59 | 0.96 | 0.234 |
| -0.608 | 1.49E-09 | NA | + | NA | NA | 2.00E-01 | 7 | -156.973 | 327.946 | 1.316 | 0.196 |
| -0.446 | NA | NA | + | NA | -5.37E-02 | 1.94E-01 | 7 | -156.989 | 327.978 | 1.348 | 0.193 |
| -0.426 | NA | NA | + | NA | NA | 1.58E-01 | 6 | -153.266 | 318.532 | 0 | 0.447 |
| -0.458 | 1.08E-09 | NA | + | NA | NA | 1.64E-01 | 7 | -153.063 | 320.126 | 1.594 | 0.201 |
| -0.476 | NA | NA | + | -2.19E-04 | NA | 1.63E-01 | 7 | -153.16 | 320.321 | 1.789 | 0.183 |
| -0.388 | NA | NA | + | NA | -1.71E-02 | 1.59E-01 | 7 | -153.237 | 320.475 | 1.942 | 0.169 |
| -0.582 | NA | NA | + | NA | NA | 2.04E-01 | 6 | -158.093 | 328.185 | 0 | 0.38 |
| -0.685 | NA | NA | + | -4.98E-04 | NA | 2.12E-01 | 7 | -157.518 | 329.035 | 0.85 | 0.249 |
| -0.626 | 1.56E-09 | NA | + | NA | NA | 2.11E-01 | 7 | -157.742 | 329.485 | 1.299 | 0.199 |
| -0.487 | NA | NA | + | NA | -4.43E-02 | 2.05E-01 | 7 | -157.882 | 329.764 | 1.579 | 0.173 |
| -0.256 | NA | NA | NA | NA | NA | 1.67E-01 | 3 | -154.4 | 314.8 | 0 | 0.291 |
| -0.314 | NA | NA | + | NA | NA | 1.31E-01 | 6 | -151.555 | 315.11 | 0.311 | 0.249 |
| -0.16 | NA | NA | NA | 4.68E-04 | NA | 1.41E-01 | 4 | -153.618 | 315.236 | 0.437 | 0.234 |
| -0.278 | 8.24E-10 | NA | NA | NA | NA | 1.70E-01 | 4 | -154.305 | 316.609 | 1.81 | 0.118 |
| -0.259 | NA | NA | NA | NA | 1.64E-03 | 1.67E-01 | 4 | -154.399 | 316.799 | 1.999 | 0.107 |
| -0.363 | NA | NA | + | NA | NA | 1.42E-01 | 6 | -141.368 | 294.736 | 0 | 0.311 |
| -0.288 | NA | NA | NA | NA | NA | 1.71E-01 | 3 | -144.839 | 295.678 | 0.942 | 0.194 |
| -0.389 | 9.03E-10 | NA | + | NA | NA | 1.46E-01 | 7 | -141.221 | 296.443 | 1.707 | 0.132 |
| -0.217 | NA | NA | NA | 3.87E-04 | NA | 1.53E-01 | 4 | -144.26 | 296.521 | 1.784 | 0.127 |
| -0.393 | NA | NA | + | -1.30E-04 | NA | 1.44E-01 | 7 | -141.328 | 296.656 | 1.92 | 0.119 |
| -0.338 | NA | NA | + | NA | -1.17E-02 | 1.42E-01 | 7 | -141.354 | 296.707 | 1.971 | 0.116 |
| -0.436 | NA | NA | + | NA | NA | 1.58E-01 | 6 | -142.886 | 297.772 | 0 | 0.428 |
| -0.471 | 1.33E-09 | NA | + | NA | NA | 1.64E-01 | 7 | -142.633 | 299.266 | 1.494 | 0.203 |
| -0.496 | NA | NA | + | -2.64E-04 | NA | 1.64E-01 | 7 | -142.714 | 299.427 | 1.655 | 0.187 |
| -0.359 | NA | NA | + | NA | -3.52E-02 | 1.60E-01 | 7 | -142.741 | 299.481 | 1.709 | 0.182 |
| -0.379 | NA | NA | + | NA | NA | 1.45E-01 | 6 | -137.615 | 287.229 | 0 | 0.401 |
| -0.468 | NA | NA | + | -3.73E-04 | NA | 1.53E-01 | 7 | -137.241 | 288.483 | 1.253 | 0.215 |
| -0.265 | NA | NA | + | NA | -5.30E-02 | 1.47E-01 | 7 | -137.279 | 288.558 | 1.329 | 0.207 |
| -0.406 | 8.95E-10 | NA | + | NA | NA | 1.50E-01 | 7 | -137.431 | 288.862 | 1.633 | 0.177 |
| -0.585 | NA | NA | + | NA | NA | 2.00E-01 | 6 | -158.998 | 329.996 | 0 | 0.276 |
| -0.701 | NA | NA | + | -5.70E-04 | NA | 2.09E-01 | 7 | -158.239 | 330.477 | 0.481 | 0.217 |
| -0.624 | 1.38E-09 | NA | + | NA | NA | 2.07E-01 | 7 | -158.698 | 331.396 | 1.4 | 0.137 |
| -0.475 | NA | NA | + | NA | -4.91E-02 | 2.01E-01 | 7 | -158.728 | 331.456 | 1.46 | 0.133 |
| -0.575 | NA | NA | + | -6.31E-04 | -6.19E-02 | 2.11E-01 | 8 | -157.813 | 331.626 | 1.63 | 0.122 |
| -0.75 | 1.53E-09 | NA | + | -5.95E-04 | NA | 2.17E-01 | 8 | -157.869 | 331.739 | 1.743 | 0.115 |
| -0.478 | NA | NA | + | NA | NA | 1.69E-01 | 6 | -151.338 | 314.676 | 0 | 0.409 |
| -0.561 | NA | NA | + | -3.85E-04 | NA | 1.76E-01 | 7 | -150.972 | 315.944 | 1.268 | 0.217 |
| -0.511 | 8.38E-10 | NA | + | NA | NA | 1.77E-01 | 7 | -151.103 | 316.205 | 1.529 | 0.19 |
| -0.385 | NA | NA | + | NA | -4.31E-02 | 1.71E-01 | 7 | -151.138 | 316.276 | 1.599 | 0.184 |
| -0.386 | NA | NA | + | NA | NA | 1.47E-01 | 6 | -152.748 | 317.496 | 0 | 0.305 |
| -0.297 | NA | NA | NA | NA | NA | 1.78E-01 | 3 | -156.27 | 318.541 | 1.044 | 0.181 |
| -0.211 | NA | NA | NA | 4.90E-04 | NA | 1.56E-01 | 4 | -155.42 | 318.84 | 1.344 | 0.156 |
| -0.414 | 8.82E-10 | NA | + | NA | NA | 1.52E-01 | 7 | -152.583 | 319.165 | 1.669 | 0.132 |
| -0.356 | NA | NA | + | NA | -1.40E-02 | 1.48E-01 | 7 | -152.729 | 319.458 | 1.962 | 0.114 |
| -0.383 | NA | NA | + | 1.45E-05 | NA | 1.47E-01 | 7 | -152.748 | 319.495 | 1.999 | 0.112 |
| -0.508 | NA | NA | + | NA | NA | 1.87E-01 | 6 | -169.329 | 350.658 | 0 | 0.449 |
| -0.545 | 1.36E-09 | NA | + | NA | NA | 1.93E-01 | 7 | -169.092 | 352.184 | 1.525 | 0.21 |
| -0.541 | NA | NA | + | -1.51E-04 | NA | 1.90E-01 | 7 | -169.284 | 352.569 | 1.911 | 0.173 |
| -0.479 | NA | NA | + | NA | -1.37E-02 | 1.88E-01 | 7 | -169.312 | 352.624 | 1.965 | 0.168 |
| -0.589 | NA | NA | + | -7.67E-04 | -9.69E-02 | 2.28E-01 | 8 | -159.064 | 334.127 | 0 | 0.175 |
| -0.792 | NA | NA | + | -6.57E-04 | NA | 2.27E-01 | 7 | -160.164 | 334.328 | 0.2 | 0.158 |
| -0.66 | NA | NA | + | NA | NA | 2.16E-01 | 6 | -161.169 | 334.339 | 0.211 | 0.157 |
| -0.475 | NA | NA | + | NA | -7.97E-02 | 2.15E-01 | 7 | -160.416 | 334.833 | 0.705 | 0.123 |
| -0.858 | 2.23E-09 | NA | + | -6.82E-04 | NA | 2.37E-01 | 8 | -159.52 | 335.04 | 0.913 | 0.111 |
| -0.661 | 1.95E-09 | NA | + | -7.82E-04 | -9.02E-02 | 2.37E-01 | 9 | -158.569 | 335.137 | 1.01 | 0.105 |
| -0.717 | 2.09E-09 | NA | + | NA | NA | 2.25E-01 | 7 | -160.609 | 335.218 | 1.091 | 0.101 |
| -0.541 | 1.85E-09 | NA | + | NA | -7.31E-02 | 2.23E-01 | 8 | -159.979 | 335.958 | 1.831 | 0.07 |
| -0.438 | NA | NA | + | NA | NA | 1.61E-01 | 6 | -155.168 | 322.336 | 0 | 0.45 |
| -0.471 | 9.57E-10 | NA | + | NA | NA | 1.67E-01 | 7 | -154.956 | 323.913 | 1.576 | 0.205 |
| -0.481 | NA | NA | + | -1.91E-04 | NA | 1.64E-01 | 7 | -155.089 | 324.178 | 1.842 | 0.179 |
| -0.447 | NA | NA | + | NA | 4.20E-03 | 1.61E-01 | 7 | -155.166 | 324.333 | 1.997 | 0.166 |
| -0.56 | NA | NA | + | NA | NA | 1.90E-01 | 6 | -180.003 | 372.005 | 0 | 0.453 |
| -0.602 | 1.59E-09 | NA | + | NA | NA | 1.96E-01 | 7 | -179.754 | 373.508 | 1.502 | 0.214 |
| -0.552 | NA | NA | + | NA | -3.29E-03 | 1.90E-01 | 7 | -180.002 | 374.003 | 1.998 | 0.167 |
| -0.557 | NA | NA | + | 1.46E-05 | NA | 1.89E-01 | 7 | -180.002 | 374.004 | 1.999 | 0.167 |
| -0.519 | NA | NA | + | NA | NA | 1.80E-01 | 6 | -152.994 | 317.988 | 0 | 0.405 |
| -0.61 | NA | NA | + | -4.04E-04 | NA | 1.90E-01 | 7 | -152.603 | 319.205 | 1.218 | 0.22 |
| -0.411 | NA | NA | + | NA | -4.86E-02 | 1.81E-01 | 7 | -152.742 | 319.485 | 1.497 | 0.192 |
| -0.551 | 1.13E-09 | NA | + | NA | NA | 1.86E-01 | 7 | -152.788 | 319.575 | 1.588 | 0.183 |
| -0.62 | NA | NA | + | -9.09E-04 | -1.09E-01 | 2.41E-01 | 8 | -153.48 | 322.96 | 0 | 0.369 |
| -0.691 | 1.63E-09 | NA | + | -9.53E-04 | -1.04E-01 | 2.52E-01 | 9 | -152.917 | 323.833 | 0.874 | 0.238 |
| -0.846 | NA | NA | + | -7.82E-04 | NA | 2.40E-01 | 7 | -154.97 | 323.939 | 0.98 | 0.226 |
| -0.913 | 1.82E-09 | NA | + | -8.38E-04 | NA | 2.53E-01 | 8 | -154.276 | 324.552 | 1.593 | 0.166 |
| -0.437 | NA | NA | + | NA | NA | 1.61E-01 | 6 | -154.965 | 321.93 | 0 | 0.444 |
| -0.469 | 9.44E-10 | NA | + | NA | NA | 1.67E-01 | 7 | -154.767 | 323.533 | 1.603 | 0.199 |
| -0.5 | NA | NA | + | -2.79E-04 | NA | 1.66E-01 | 7 | -154.796 | 323.592 | 1.662 | 0.193 |
| -0.451 | NA | NA | + | NA | 6.60E-03 | 1.61E-01 | 7 | -154.961 | 323.921 | 1.992 | 0.164 |
| -0.488 | NA | NA | + | -8.52E-04 | -1.00E-01 | 2.02E-01 | 8 | -140.785 | 297.571 | 0 | 0.28 |
| -0.688 | NA | NA | + | -7.53E-04 | NA | 2.00E-01 | 7 | -142.063 | 298.125 | 0.554 | 0.212 |
| -0.543 | 1.46E-09 | NA | + | -8.67E-04 | -9.61E-02 | 2.09E-01 | 9 | -140.381 | 298.762 | 1.191 | 0.155 |
| -0.74 | 1.64E-09 | NA | + | -7.75E-04 | NA | 2.07E-01 | 8 | -141.56 | 299.12 | 1.549 | 0.129 |
| -0.53 | NA | NA | + | NA | NA | 1.86E-01 | 6 | -143.634 | 299.268 | 1.697 | 0.12 |
| -0.349 | NA | NA | + | NA | -8.18E-02 | 1.87E-01 | 7 | -142.782 | 299.564 | 1.993 | 0.103 |
| -0.627 | NA | NA | + | NA | NA | 2.13E-01 | 6 | -161.563 | 335.126 | 0 | 0.251 |
| -0.747 | NA | NA | + | -5.97E-04 | NA | 2.23E-01 | 7 | -160.733 | 335.466 | 0.339 | 0.212 |
| -0.603 | NA | NA | + | -6.72E-04 | -7.19E-02 | 2.25E-01 | 8 | -160.156 | 336.311 | 1.185 | 0.139 |
| -0.676 | 1.76E-09 | NA | + | NA | NA | 2.21E-01 | 7 | -161.161 | 336.322 | 1.196 | 0.138 |
| -0.5 | NA | NA | + | NA | -5.74E-02 | 2.13E-01 | 7 | -161.191 | 336.381 | 1.255 | 0.134 |
| -0.805 | 1.91E-09 | NA | + | -6.23E-04 | NA | 2.33E-01 | 8 | -160.256 | 336.512 | 1.386 | 0.126 |
| -0.485 | NA | NA | + | NA | NA | 1.75E-01 | 6 | -148.485 | 308.97 | 0 | 0.403 |
| -0.584 | NA | NA | + | -4.54E-04 | NA | 1.82E-01 | 7 | -147.986 | 309.972 | 1.002 | 0.244 |
| -0.52 | 1.26E-09 | NA | + | NA | NA | 1.80E-01 | 7 | -148.209 | 310.418 | 1.448 | 0.195 |
| -0.433 | NA | NA | + | NA | -2.42E-02 | 1.76E-01 | 7 | -148.423 | 310.845 | 1.876 | 0.158 |
| -0.336 | NA | NA | + | NA | NA | 1.37E-01 | 6 | -147.651 | 307.301 | 0 | 0.246 |
| -0.289 | NA | NA | NA | NA | NA | 1.72E-01 | 3 | -150.823 | 307.646 | 0.345 | 0.207 |
| -0.211 | NA | NA | NA | 4.36E-04 | NA | 1.52E-01 | 4 | -150.123 | 308.246 | 0.945 | 0.154 |
| -0.368 | 6.88E-10 | NA | + | NA | NA | 1.45E-01 | 7 | -147.446 | 308.892 | 1.59 | 0.111 |
| -0.326 | 7.96E-10 | NA | NA | NA | NA | 1.81E-01 | 4 | -150.559 | 309.119 | 1.818 | 0.099 |
| -0.317 | NA | NA | + | NA | -8.95E-03 | 1.37E-01 | 7 | -147.643 | 309.286 | 1.984 | 0.091 |
| -0.335 | NA | NA | + | 5.76E-06 | NA | 1.36E-01 | 7 | -147.65 | 309.301 | 2 | 0.091 |
| -0.496 | NA | NA | + | NA | NA | 1.77E-01 | 6 | -147.728 | 307.455 | 0 | 0.405 |
| -0.591 | NA | NA | + | -4.29E-04 | NA | 1.85E-01 | 7 | -147.27 | 308.54 | 1.085 | 0.236 |
| -0.53 | 1.18E-09 | NA | + | NA | NA | 1.83E-01 | 7 | -147.483 | 308.966 | 1.511 | 0.19 |
| -0.424 | NA | NA | + | NA | -3.32E-02 | 1.78E-01 | 7 | -147.605 | 309.21 | 1.754 | 0.169 |
| -0.564 | NA | NA | + | NA | NA | 1.98E-01 | 6 | -162.103 | 336.205 | 0 | 0.422 |
| -0.605 | 1.40E-09 | NA | + | NA | NA | 2.05E-01 | 7 | -161.789 | 337.578 | 1.373 | 0.213 |
| -0.627 | NA | NA | + | -3.01E-04 | NA | 2.03E-01 | 7 | -161.904 | 337.808 | 1.603 | 0.189 |
| -0.487 | NA | NA | + | NA | -3.45E-02 | 1.98E-01 | 7 | -161.979 | 337.958 | 1.752 | 0.176 |
| -0.181 | NA | NA | NA | 5.76E-04 | NA | 1.46E-01 | 4 | -165.136 | 338.272 | 0 | 0.226 |
| -0.29 | NA | NA | NA | NA | NA | 1.75E-01 | 3 | -166.194 | 338.387 | 0.115 | 0.214 |
| -0.36 | NA | NA | + | NA | NA | 1.40E-01 | 6 | -163.243 | 338.487 | 0.214 | 0.203 |
| -0.208 | 8.63E-10 | NA | NA | 5.63E-04 | NA | 1.50E-01 | 5 | -165.028 | 340.056 | 1.784 | 0.093 |
| -0.32 | 1.06E-09 | NA | NA | NA | NA | 1.80E-01 | 4 | -166.033 | 340.066 | 1.794 | 0.092 |
| -0.389 | 1.01E-09 | NA | + | NA | NA | 1.45E-01 | 7 | -163.092 | 340.184 | 1.911 | 0.087 |
| -0.197 | NA | NA | NA | 5.89E-04 | 9.31E-03 | 1.45E-01 | 5 | -165.129 | 340.257 | 1.985 | 0.084 |
| -0.534 | NA | NA | + | NA | NA | 1.85E-01 | 6 | -152.744 | 317.487 | 0 | 0.296 |
| -0.373 | NA | NA | + | NA | -7.22E-02 | 1.87E-01 | 7 | -152.088 | 318.176 | 0.689 | 0.21 |
| -0.63 | NA | NA | + | -4.50E-04 | NA | 1.92E-01 | 7 | -152.232 | 318.463 | 0.976 | 0.182 |
| -0.461 | NA | NA | + | -5.44E-04 | -8.44E-02 | 1.96E-01 | 8 | -151.35 | 318.701 | 1.213 | 0.162 |
| -0.573 | 1.26E-09 | NA | + | NA | NA | 1.92E-01 | 7 | -152.426 | 318.852 | 1.364 | 0.15 |
| -0.529 | NA | NA | + | NA | NA | 1.85E-01 | 6 | -160.505 | 333.01 | 0 | 0.436 |
| -0.567 | 1.21E-09 | NA | + | NA | NA | 1.92E-01 | 7 | -160.251 | 334.502 | 1.492 | 0.207 |
| -0.583 | NA | NA | + | -2.43E-04 | NA | 1.90E-01 | 7 | -160.38 | 334.759 | 1.75 | 0.182 |
| -0.463 | NA | NA | + | NA | -2.92E-02 | 1.85E-01 | 7 | -160.419 | 334.838 | 1.829 | 0.175 |
| -0.522 | NA | NA | + | NA | NA | 1.85E-01 | 6 | -156.485 | 324.97 | 0 | 0.398 |
| -0.628 | NA | NA | + | -5.05E-04 | NA | 1.93E-01 | 7 | -155.905 | 325.81 | 0.84 | 0.262 |
| -0.558 | 1.27E-09 | NA | + | NA | NA | 1.91E-01 | 7 | -156.232 | 326.463 | 1.493 | 0.189 |
| -0.483 | NA | NA | + | NA | -1.84E-02 | 1.86E-01 | 7 | -156.45 | 326.901 | 1.931 | 0.152 |
| -0.464 | NA | NA | + | NA | NA | 1.69E-01 | 6 | -149.185 | 310.37 | 0 | 0.419 |
| -0.5 | 1.10E-09 | NA | + | NA | NA | 1.76E-01 | 7 | -148.885 | 311.77 | 1.401 | 0.208 |
| -0.53 | NA | NA | + | -3.02E-04 | NA | 1.74E-01 | 7 | -148.966 | 311.931 | 1.562 | 0.192 |
| -0.382 | NA | NA | + | NA | -3.75E-02 | 1.70E-01 | 7 | -149.028 | 312.056 | 1.686 | 0.18 |
| -0.447 | NA | NA | + | NA | NA | 1.60E-01 | 6 | -147.813 | 307.626 | 0 | 0.425 |
| -0.479 | 9.56E-10 | NA | + | NA | NA | 1.67E-01 | 7 | -147.58 | 309.159 | 1.533 | 0.198 |
| -0.351 | NA | NA | + | NA | -4.34E-02 | 1.62E-01 | 7 | -147.605 | 309.21 | 1.584 | 0.193 |
| -0.507 | NA | NA | + | -2.59E-04 | NA | 1.65E-01 | 7 | -147.65 | 309.3 | 1.674 | 0.184 |
| -0.527 | NA | NA | + | -7.05E-04 | -1.02E-01 | 2.23E-01 | 8 | -154.562 | 325.123 | 0 | 0.176 |
| -0.612 | NA | NA | + | NA | NA | 2.10E-01 | 6 | -156.643 | 325.286 | 0.163 | 0.162 |
| -0.735 | NA | NA | + | -5.98E-04 | NA | 2.21E-01 | 7 | -155.758 | 325.516 | 0.393 | 0.144 |
| -0.418 | NA | NA | + | NA | -8.61E-02 | 2.09E-01 | 7 | -155.78 | 325.56 | 0.437 | 0.141 |
| -0.593 | 1.58E-09 | NA | + | -7.47E-04 | -9.85E-02 | 2.33E-01 | 9 | -154.031 | 326.061 | 0.938 | 0.11 |
| -0.798 | 1.70E-09 | NA | + | -6.47E-04 | NA | 2.32E-01 | 8 | -155.155 | 326.309 | 1.186 | 0.097 |
| -0.658 | 1.47E-09 | NA | + | NA | NA | 2.18E-01 | 7 | -156.188 | 326.376 | 1.253 | 0.094 |
| -0.469 | 1.35E-09 | NA | + | NA | -8.24E-02 | 2.17E-01 | 8 | -155.397 | 326.793 | 1.67 | 0.076 |
| -0.529 | NA | NA | + | NA | NA | 1.82E-01 | 6 | -153.03 | 318.06 | 0 | 0.324 |
| -0.646 | NA | NA | + | -5.65E-04 | NA | 1.91E-01 | 7 | -152.294 | 318.587 | 0.527 | 0.249 |
| -0.569 | 1.37E-09 | NA | + | NA | NA | 1.88E-01 | 7 | -152.733 | 319.466 | 1.406 | 0.161 |
| -0.456 | NA | NA | + | NA | -3.31E-02 | 1.82E-01 | 7 | -152.912 | 319.824 | 1.764 | 0.134 |
| -0.695 | 1.51E-09 | NA | + | -5.91E-04 | NA | 1.99E-01 | 8 | -151.928 | 319.855 | 1.795 | 0.132 |
| -0.552 | NA | NA | + | NA | NA | 1.83E-01 | 6 | -157.651 | 327.301 | 0 | 0.266 |
| -0.69 | NA | NA | + | -6.43E-04 | NA | 1.96E-01 | 7 | -156.693 | 327.386 | 0.085 | 0.255 |
| -0.733 | 1.55E-09 | NA | + | -6.58E-04 | NA | 2.01E-01 | 8 | -156.403 | 328.807 | 1.506 | 0.125 |
| -0.588 | 1.42E-09 | NA | + | NA | NA | 1.88E-01 | 7 | -157.408 | 328.817 | 1.515 | 0.125 |
| -0.594 | NA | NA | + | -6.74E-04 | -4.70E-02 | 1.98E-01 | 8 | -156.471 | 328.943 | 1.641 | 0.117 |
| -0.472 | NA | NA | + | NA | -3.67E-02 | 1.85E-01 | 7 | -157.515 | 329.031 | 1.73 | 0.112 |
| -0.447 | NA | NA | + | NA | NA | 1.57E-01 | 6 | -160.836 | 333.672 | 0 | 0.44 |
| -0.522 | NA | NA | + | -3.26E-04 | NA | 1.64E-01 | 7 | -160.613 | 335.225 | 1.553 | 0.202 |
| -0.477 | 9.95E-10 | NA | + | NA | NA | 1.62E-01 | 7 | -160.666 | 335.332 | 1.66 | 0.192 |
| -0.409 | NA | NA | + | NA | -1.71E-02 | 1.57E-01 | 7 | -160.81 | 335.62 | 1.947 | 0.166 |
| -0.547 | NA | NA | + | NA | NA | 1.87E-01 | 6 | -150.329 | 312.658 | 0 | 0.279 |
| -0.663 | NA | NA | + | -5.21E-04 | NA | 1.99E-01 | 7 | -149.67 | 313.341 | 0.683 | 0.198 |
| -0.414 | NA | NA | + | NA | -5.95E-02 | 1.89E-01 | 7 | -149.915 | 313.83 | 1.172 | 0.155 |
| -0.586 | 1.30E-09 | NA | + | NA | NA | 1.94E-01 | 7 | -150.035 | 314.07 | 1.412 | 0.138 |
| -0.521 | NA | NA | + | -5.76E-04 | -6.91E-02 | 2.01E-01 | 8 | -149.115 | 314.231 | 1.573 | 0.127 |
| -0.711 | 1.43E-09 | NA | + | -5.45E-04 | NA | 2.06E-01 | 8 | -149.315 | 314.631 | 1.973 | 0.104 |
| -0.507 | NA | NA | + | NA | NA | 1.73E-01 | 6 | -161.402 | 334.804 | 0 | 0.454 |
| -0.538 | 1.09E-09 | NA | + | NA | NA | 1.78E-01 | 7 | -161.241 | 336.481 | 1.677 | 0.196 |
| -0.553 | NA | NA | + | -2.08E-04 | NA | 1.78E-01 | 7 | -161.314 | 336.629 | 1.825 | 0.182 |
| -0.515 | NA | NA | + | NA | 3.50E-03 | 1.73E-01 | 7 | -161.401 | 336.802 | 1.998 | 0.167 |
| -0.443 | NA | NA | + | NA | NA | 1.61E-01 | 6 | -136.844 | 285.688 | 0 | 0.386 |
| -0.531 | NA | NA | + | -3.95E-04 | NA | 1.69E-01 | 7 | -136.416 | 286.832 | 1.144 | 0.218 |
| -0.322 | NA | NA | + | NA | -5.60E-02 | 1.64E-01 | 7 | -136.441 | 286.883 | 1.195 | 0.212 |
| -0.474 | 9.45E-10 | NA | + | NA | NA | 1.68E-01 | 7 | -136.583 | 287.166 | 1.479 | 0.184 |
| -0.478 | NA | NA | + | NA | NA | 1.63E-01 | 6 | -153.56 | 319.121 | 0 | 0.434 |
| -0.553 | NA | NA | + | -3.39E-04 | NA | 1.70E-01 | 7 | -153.294 | 320.588 | 1.467 | 0.208 |
| -0.508 | 9.15E-10 | NA | + | NA | NA | 1.70E-01 | 7 | -153.372 | 320.744 | 1.624 | 0.193 |
| -0.438 | NA | NA | + | NA | -1.75E-02 | 1.63E-01 | 7 | -153.529 | 321.059 | 1.938 | 0.165 |
| -0.543 | NA | NA | + | NA | NA | 1.86E-01 | 6 | -158.453 | 328.906 | 0 | 0.426 |
| -0.62 | NA | NA | + | -3.51E-04 | NA | 1.93E-01 | 7 | -158.175 | 330.35 | 1.444 | 0.207 |
| -0.581 | 1.33E-09 | NA | + | NA | NA | 1.93E-01 | 7 | -158.188 | 330.377 | 1.471 | 0.204 |
| -0.497 | NA | NA | + | NA | -2.03E-02 | 1.86E-01 | 7 | -158.413 | 330.825 | 1.919 | 0.163 |
| -0.475 | NA | NA | + | NA | NA | 1.71E-01 | 6 | -156.471 | 324.942 | 0 | 0.439 |
| -0.51 | 1.15E-09 | NA | + | NA | NA | 1.77E-01 | 7 | -156.227 | 326.454 | 1.512 | 0.206 |
| -0.538 | NA | NA | + | -2.88E-04 | NA | 1.75E-01 | 7 | -156.292 | 326.584 | 1.642 | 0.193 |
| -0.47 | NA | NA | + | NA | -2.07E-03 | 1.71E-01 | 7 | -156.471 | 326.941 | 1.999 | 0.162 |
| -0.418 | NA | NA | + | NA | NA | 1.61E-01 | 6 | -148.566 | 309.131 | 0 | 0.382 |
| -0.445 | 1.04E-09 | NA | + | NA | NA | 1.65E-01 | 7 | -148.409 | 310.817 | 1.686 | 0.164 |
| -0.466 | NA | NA | + | -2.19E-04 | NA | 1.65E-01 | 7 | -148.456 | 310.911 | 1.78 | 0.157 |
| -0.353 | NA | NA | NA | NA | NA | 1.99E-01 | 3 | -152.458 | 310.916 | 1.785 | 0.156 |
| -0.41 | NA | NA | + | NA | -3.58E-03 | 1.61E-01 | 7 | -148.564 | 311.129 | 1.997 | 0.141 |
| -0.486 | NA | NA | + | NA | NA | 1.70E-01 | 6 | -156.436 | 324.872 | 0 | 0.425 |
| -0.573 | NA | NA | + | -3.93E-04 | NA | 1.76E-01 | 7 | -156.095 | 326.189 | 1.318 | 0.22 |
| -0.52 | 9.94E-10 | NA | + | NA | NA | 1.76E-01 | 7 | -156.205 | 326.41 | 1.538 | 0.197 |
| -0.46 | NA | NA | + | NA | -1.22E-02 | 1.70E-01 | 7 | -156.422 | 326.843 | 1.971 | 0.159 |
| -0.57 | NA | NA | + | NA | NA | 1.98E-01 | 6 | -164.348 | 340.697 | 0 | 0.398 |
| -0.613 | 1.32E-09 | NA | + | NA | NA | 2.06E-01 | 7 | -163.987 | 341.974 | 1.277 | 0.21 |
| -0.443 | NA | NA | + | NA | -5.76E-02 | 1.99E-01 | 7 | -163.987 | 341.974 | 1.277 | 0.21 |
| -0.633 | NA | NA | + | -3.15E-04 | NA | 2.03E-01 | 7 | -164.131 | 342.261 | 1.565 | 0.182 |
| -0.658 | NA | NA | + | -8.55E-04 | -1.04E-01 | 2.56E-01 | 8 | -164.235 | 344.47 | 0 | 0.254 |
| -0.878 | NA | NA | + | -7.29E-04 | NA | 2.58E-01 | 7 | -165.519 | 345.039 | 0.568 | 0.191 |
| -0.729 | 1.90E-09 | NA | + | -8.80E-04 | -9.81E-02 | 2.66E-01 | 9 | -163.761 | 345.521 | 1.051 | 0.15 |
| -0.741 | NA | NA | + | NA | NA | 2.46E-01 | 6 | -166.775 | 345.55 | 1.08 | 0.148 |
| -0.945 | 2.19E-09 | NA | + | -7.67E-04 | NA | 2.69E-01 | 8 | -164.891 | 345.782 | 1.312 | 0.132 |
| -0.545 | NA | NA | + | NA | -8.40E-02 | 2.43E-01 | 7 | -165.936 | 345.873 | 1.402 | 0.126 |
| -0.424 | NA | NA | + | NA | NA | 1.57E-01 | 6 | -142.622 | 297.245 | 0 | 0.402 |
| -0.531 | NA | NA | + | -4.41E-04 | NA | 1.68E-01 | 7 | -142.128 | 298.256 | 1.011 | 0.243 |
| -0.333 | NA | NA | + | NA | -4.14E-02 | 1.58E-01 | 7 | -142.43 | 298.86 | 1.615 | 0.179 |
| -0.453 | 1.08E-09 | NA | + | NA | NA | 1.62E-01 | 7 | -142.452 | 298.905 | 1.66 | 0.175 |
| -0.561 | NA | NA | + | NA | NA | 1.97E-01 | 6 | -148.412 | 308.824 | 0 | 0.264 |
| -0.679 | NA | NA | + | -5.62E-04 | NA | 2.06E-01 | 7 | -147.597 | 309.195 | 0.37 | 0.22 |
| -0.599 | 1.39E-09 | NA | + | NA | NA | 2.03E-01 | 7 | -148.086 | 310.172 | 1.348 | 0.135 |
| -0.449 | NA | NA | + | NA | -5.07E-02 | 1.97E-01 | 7 | -148.118 | 310.235 | 1.411 | 0.131 |
| -0.55 | NA | NA | + | -6.28E-04 | -6.49E-02 | 2.08E-01 | 8 | -147.119 | 310.238 | 1.414 | 0.13 |
| -0.727 | 1.54E-09 | NA | + | -5.88E-04 | NA | 2.13E-01 | 8 | -147.196 | 310.391 | 1.567 | 0.121 |
| -0.594 | NA | NA | + | -6.41E-04 | NA | 1.71E-01 | 7 | -135.527 | 285.054 | 0 | 0.256 |
| -0.443 | NA | NA | + | NA | NA | 1.58E-01 | 6 | -136.652 | 285.303 | 0.249 | 0.226 |
| -0.46 | NA | NA | + | -6.86E-04 | -6.64E-02 | 1.76E-01 | 8 | -134.987 | 285.974 | 0.92 | 0.162 |
| -0.635 | 1.27E-09 | NA | + | -6.63E-04 | NA | 1.78E-01 | 8 | -135.206 | 286.412 | 1.358 | 0.13 |
| -0.322 | NA | NA | + | NA | -5.59E-02 | 1.60E-01 | 7 | -136.271 | 286.542 | 1.488 | 0.122 |
| -0.475 | 1.11E-09 | NA | + | NA | NA | 1.63E-01 | 7 | -136.411 | 286.822 | 1.768 | 0.106 |
| -0.575 | NA | NA | + | NA | NA | 1.94E-01 | 6 | -161.3 | 334.6 | 0 | 0.399 |
| -0.666 | NA | NA | + | -4.22E-04 | NA | 2.02E-01 | 7 | -160.91 | 335.821 | 1.22 | 0.217 |
| -0.616 | 1.45E-09 | NA | + | NA | NA | 2.01E-01 | 7 | -161.011 | 336.021 | 1.421 | 0.196 |
| -0.466 | NA | NA | + | NA | -4.87E-02 | 1.94E-01 | 7 | -161.05 | 336.1 | 1.5 | 0.188 |
| -0.49 | NA | NA | + | NA | NA | 1.76E-01 | 6 | -156.231 | 324.462 | 0 | 0.434 |
| -0.524 | 1.13E-09 | NA | + | NA | NA | 1.82E-01 | 7 | -156.018 | 326.035 | 1.573 | 0.197 |
| -0.557 | NA | NA | + | -3.02E-04 | NA | 1.82E-01 | 7 | -156.031 | 326.062 | 1.6 | 0.195 |
| -0.428 | NA | NA | + | NA | -2.94E-02 | 1.77E-01 | 7 | -156.143 | 326.286 | 1.824 | 0.174 |
| -0.597 | NA | NA | + | NA | NA | 2.02E-01 | 6 | -153.55 | 319.1 | 0 | 0.211 |
| -0.718 | NA | NA | + | -5.82E-04 | NA | 2.13E-01 | 7 | -152.704 | 319.407 | 0.307 | 0.181 |
| -0.54 | NA | NA | + | -6.77E-04 | -8.72E-02 | 2.15E-01 | 8 | -151.822 | 319.644 | 0.544 | 0.161 |
| -0.434 | NA | NA | + | NA | -7.15E-02 | 2.02E-01 | 7 | -152.95 | 319.899 | 0.799 | 0.142 |
| -0.644 | 1.64E-09 | NA | + | NA | NA | 2.10E-01 | 7 | -153.156 | 320.311 | 1.211 | 0.115 |
| -0.772 | 1.73E-09 | NA | + | -5.97E-04 | NA | 2.22E-01 | 8 | -152.26 | 320.519 | 1.419 | 0.104 |
| -0.596 | 1.55E-09 | NA | + | -6.86E-04 | -8.29E-02 | 2.22E-01 | 9 | -151.464 | 320.927 | 1.827 | 0.085 |
| -0.617 | NA | NA | + | -9.56E-04 | -1.37E-01 | 2.61E-01 | 8 | -157.692 | 331.385 | 0 | 0.575 |
| -0.701 | 2.08E-09 | NA | + | -9.86E-04 | -1.29E-01 | 2.72E-01 | 9 | -156.995 | 331.99 | 0.605 | 0.425 |
| -0.672 | NA | NA | + | NA | NA | 2.24E-01 | 6 | -160.464 | 332.928 | 0 | 0.206 |
| -0.791 | NA | NA | + | -6.02E-04 | NA | 2.35E-01 | 7 | -159.614 | 333.229 | 0.301 | 0.177 |
| -0.6 | NA | NA | + | -6.96E-04 | -9.00E-02 | 2.34E-01 | 8 | -158.711 | 333.422 | 0.494 | 0.161 |
| -0.497 | NA | NA | + | NA | -7.49E-02 | 2.22E-01 | 7 | -159.831 | 333.663 | 0.735 | 0.142 |
| -0.722 | 1.81E-09 | NA | + | NA | NA | 2.32E-01 | 7 | -160.032 | 334.065 | 1.137 | 0.116 |
| -0.85 | 1.96E-09 | NA | + | -6.29E-04 | NA | 2.44E-01 | 8 | -159.103 | 334.206 | 1.278 | 0.109 |
| -0.663 | 1.76E-09 | NA | + | -7.14E-04 | -8.50E-02 | 2.42E-01 | 9 | -158.297 | 334.594 | 1.666 | 0.089 |
| -0.456 | NA | NA | + | NA | NA | 1.60E-01 | 6 | -156.483 | 324.966 | 0 | 0.435 |
| -0.491 | 1.17E-09 | NA | + | NA | NA | 1.66E-01 | 7 | -156.246 | 326.491 | 1.525 | 0.203 |
| -0.523 | NA | NA | + | -3.14E-04 | NA | 1.66E-01 | 7 | -156.264 | 326.529 | 1.563 | 0.199 |
| -0.425 | NA | NA | + | NA | -1.42E-02 | 1.60E-01 | 7 | -156.463 | 326.926 | 1.961 | 0.163 |
| -0.395 | NA | NA | + | NA | NA | 1.50E-01 | 6 | -146.816 | 305.632 | 0 | 0.435 |
| -0.428 | 1.07E-09 | NA | + | NA | NA | 1.56E-01 | 7 | -146.589 | 307.178 | 1.546 | 0.201 |
| -0.313 | NA | NA | + | NA | -3.74E-02 | 1.51E-01 | 7 | -146.663 | 307.325 | 1.693 | 0.187 |
| -0.447 | NA | NA | + | -2.06E-04 | NA | 1.55E-01 | 7 | -146.714 | 307.428 | 1.796 | 0.177 |
| -0.56 | NA | NA | + | -1.06E-03 | -1.35E-01 | 2.44E-01 | 8 | -150.205 | 316.41 | 0 | 0.574 |
| -0.631 | 1.83E-09 | NA | + | -1.08E-03 | -1.30E-01 | 2.55E-01 | 9 | -149.504 | 317.009 | 0.599 | 0.426 |
| -0.514 | NA | NA | + | NA | NA | 1.84E-01 | 6 | -146.19 | 304.379 | 0 | 0.265 |
| -0.632 | NA | NA | + | -5.22E-04 | NA | 1.94E-01 | 7 | -145.503 | 305.006 | 0.627 | 0.194 |
| -0.373 | NA | NA | + | NA | -6.29E-02 | 1.84E-01 | 7 | -145.726 | 305.452 | 1.073 | 0.155 |
| -0.556 | 1.54E-09 | NA | + | NA | NA | 1.90E-01 | 7 | -145.83 | 305.659 | 1.28 | 0.14 |
| -0.48 | NA | NA | + | -5.95E-04 | -7.52E-02 | 1.96E-01 | 8 | -144.847 | 305.695 | 1.315 | 0.137 |
| -0.683 | 1.66E-09 | NA | + | -5.46E-04 | NA | 2.02E-01 | 8 | -145.078 | 306.155 | 1.776 | 0.109 |
| -0.501 | NA | NA | + | NA | NA | 1.77E-01 | 6 | -147.706 | 307.412 | 0 | 0.33 |
| -0.6 | NA | NA | + | -4.28E-04 | NA | 1.86E-01 | 7 | -147.254 | 308.509 | 1.097 | 0.191 |
| -0.366 | NA | NA | + | NA | -6.12E-02 | 1.79E-01 | 7 | -147.254 | 308.509 | 1.097 | 0.191 |
| -0.539 | 1.49E-09 | NA | + | NA | NA | 1.83E-01 | 7 | -147.41 | 308.82 | 1.408 | 0.163 |
| -0.459 | NA | NA | + | -4.90E-04 | -7.02E-02 | 1.89E-01 | 8 | -146.667 | 309.334 | 1.922 | 0.126 |
| -0.702 | NA | NA | + | -6.71E-04 | NA | 2.08E-01 | 7 | -148.589 | 311.177 | 0 | 0.251 |
| -0.563 | NA | NA | + | NA | NA | 1.98E-01 | 6 | -149.76 | 311.519 | 0.342 | 0.212 |
| -0.559 | NA | NA | + | -7.46E-04 | -7.24E-02 | 2.11E-01 | 8 | -147.969 | 311.937 | 0.76 | 0.172 |
| -0.751 | 1.54E-09 | NA | + | -6.94E-04 | NA | 2.17E-01 | 8 | -148.155 | 312.31 | 1.133 | 0.143 |
| -0.441 | NA | NA | + | NA | -5.56E-02 | 1.99E-01 | 7 | -149.391 | 312.782 | 1.605 | 0.113 |
| -0.603 | 1.39E-09 | NA | + | NA | NA | 2.05E-01 | 7 | -149.41 | 312.82 | 1.643 | 0.11 |
| -0.518 | NA | NA | + | NA | NA | 1.78E-01 | 6 | -157.009 | 326.017 | 0 | 0.406 |
| -0.609 | NA | NA | + | -4.43E-04 | NA | 1.84E-01 | 7 | -156.57 | 327.141 | 1.123 | 0.231 |
| -0.556 | 1.36E-09 | NA | + | NA | NA | 1.84E-01 | 7 | -156.707 | 327.413 | 1.396 | 0.202 |
| -0.458 | NA | NA | + | NA | -2.76E-02 | 1.79E-01 | 7 | -156.93 | 327.861 | 1.843 | 0.161 |
| -0.459 | NA | NA | + | NA | NA | 1.68E-01 | 6 | -148.974 | 309.948 | 0 | 0.4 |
| -0.56 | NA | NA | + | -4.65E-04 | NA | 1.75E-01 | 7 | -148.453 | 310.906 | 0.958 | 0.248 |
| -0.493 | 1.33E-09 | NA | + | NA | NA | 1.72E-01 | 7 | -148.731 | 311.463 | 1.515 | 0.188 |
| -0.393 | NA | NA | + | NA | -3.16E-02 | 1.70E-01 | 7 | -148.863 | 311.725 | 1.777 | 0.165 |
| -0.336 | NA | NA | + | NA | NA | 1.41E-01 | 6 | -136.651 | 285.302 | 0 | 0.359 |
| -0.314 | NA | NA | NA | NA | NA | 1.80E-01 | 3 | -140.24 | 286.481 | 1.179 | 0.199 |
| -0.36 | 9.67E-10 | NA | + | NA | NA | 1.45E-01 | 7 | -136.498 | 286.997 | 1.695 | 0.154 |
| -0.391 | NA | NA | + | -2.27E-04 | NA | 1.46E-01 | 7 | -136.521 | 287.041 | 1.739 | 0.15 |
| -0.293 | NA | NA | + | NA | -2.03E-02 | 1.42E-01 | 7 | -136.604 | 287.207 | 1.905 | 0.138 |
| -0.504 | NA | NA | + | NA | NA | 1.73E-01 | 6 | -156.741 | 325.481 | 0 | 0.423 |
| -0.541 | 1.14E-09 | NA | + | NA | NA | 1.80E-01 | 7 | -156.466 | 326.932 | 1.451 | 0.205 |
| -0.582 | NA | NA | + | -3.47E-04 | NA | 1.79E-01 | 7 | -156.471 | 326.943 | 1.462 | 0.204 |
| -0.441 | NA | NA | + | NA | -2.86E-02 | 1.74E-01 | 7 | -156.659 | 327.318 | 1.837 | 0.169 |
| -0.502 | NA | NA | + | NA | NA | 1.75E-01 | 6 | -159.624 | 331.247 | 0 | 0.437 |
| -0.542 | 1.37E-09 | NA | + | NA | NA | 1.81E-01 | 7 | -159.37 | 332.74 | 1.492 | 0.207 |
| -0.426 | NA | NA | + | NA | -3.43E-02 | 1.76E-01 | 7 | -159.501 | 333.003 | 1.755 | 0.182 |
| -0.544 | NA | NA | + | -1.85E-04 | NA | 1.78E-01 | 7 | -159.55 | 333.099 | 1.852 | 0.173 |
| -0.441 | NA | NA | + | NA | NA | 1.62E-01 | 6 | -139.915 | 291.831 | 0 | 0.388 |
| -0.534 | NA | NA | + | -4.08E-04 | NA | 1.70E-01 | 7 | -139.466 | 292.933 | 1.102 | 0.224 |
| -0.317 | NA | NA | + | NA | -5.51E-02 | 1.64E-01 | 7 | -139.545 | 293.091 | 1.26 | 0.207 |
| -0.472 | 1.00E-09 | NA | + | NA | NA | 1.68E-01 | 7 | -139.671 | 293.342 | 1.512 | 0.182 |
| -0.587 | NA | NA | + | NA | NA | 1.97E-01 | 6 | -154.18 | 320.359 | 0 | 0.248 |
| -0.709 | NA | NA | + | -6.04E-04 | NA | 2.06E-01 | 7 | -153.289 | 320.578 | 0.219 | 0.222 |
| -0.56 | NA | NA | + | -6.72E-04 | -7.44E-02 | 2.09E-01 | 8 | -152.672 | 321.343 | 0.984 | 0.152 |
| -0.453 | NA | NA | + | NA | -6.10E-02 | 1.99E-01 | 7 | -153.762 | 321.525 | 1.165 | 0.138 |
| -0.622 | 1.23E-09 | NA | + | NA | NA | 2.03E-01 | 7 | -153.898 | 321.795 | 1.436 | 0.121 |
| -0.756 | 1.41E-09 | NA | + | -6.35E-04 | NA | 2.14E-01 | 8 | -152.915 | 321.831 | 1.471 | 0.119 |
| -0.562 | NA | NA | + | -7.54E-04 | -1.04E-01 | 2.29E-01 | 8 | -151.585 | 319.169 | 0 | 0.252 |
| -0.778 | NA | NA | + | -6.48E-04 | NA | 2.27E-01 | 7 | -152.931 | 319.862 | 0.693 | 0.178 |
| -0.626 | 1.51E-09 | NA | + | -7.93E-04 | -1.00E-01 | 2.39E-01 | 9 | -151.078 | 320.156 | 0.987 | 0.154 |
| -0.658 | NA | NA | + | NA | NA | 2.18E-01 | 6 | -154.09 | 320.18 | 1.011 | 0.152 |
| -0.46 | NA | NA | + | NA | -8.75E-02 | 2.18E-01 | 7 | -153.139 | 320.278 | 1.109 | 0.145 |
| -0.839 | 1.67E-09 | NA | + | -6.96E-04 | NA | 2.38E-01 | 8 | -152.319 | 320.638 | 1.468 | 0.121 |
| -0.386 | NA | NA | + | NA | NA | 1.51E-01 | 6 | -146.513 | 305.027 | 0 | 0.308 |
| -0.325 | NA | NA | NA | NA | NA | 1.87E-01 | 3 | -149.889 | 305.777 | 0.751 | 0.211 |
| -0.41 | 8.49E-10 | NA | + | NA | NA | 1.54E-01 | 7 | -146.401 | 306.801 | 1.775 | 0.127 |
| -0.254 | NA | NA | NA | 3.66E-04 | NA | 1.70E-01 | 4 | -149.421 | 306.842 | 1.815 | 0.124 |
| -0.415 | NA | NA | + | -1.21E-04 | NA | 1.53E-01 | 7 | -146.48 | 306.959 | 1.933 | 0.117 |
| -0.378 | NA | NA | + | NA | -3.68E-03 | 1.51E-01 | 7 | -146.512 | 307.024 | 1.997 | 0.113 |
| -0.412 | NA | NA | + | NA | NA | 1.53E-01 | 6 | -167.246 | 346.492 | 0 | 0.296 |
| -0.34 | NA | NA | NA | NA | NA | 1.98E-01 | 3 | -170.756 | 347.512 | 1.02 | 0.178 |
| -0.224 | NA | NA | NA | 5.73E-04 | NA | 1.67E-01 | 4 | -169.759 | 347.518 | 1.026 | 0.177 |
| -0.441 | 1.07E-09 | NA | + | NA | NA | 1.58E-01 | 7 | -167.105 | 348.21 | 1.718 | 0.125 |
| -0.454 | NA | NA | + | NA | 1.86E-02 | 1.52E-01 | 7 | -167.217 | 348.434 | 1.942 | 0.112 |
| -0.388 | NA | NA | + | 9.79E-05 | NA | 1.51E-01 | 7 | -167.228 | 348.456 | 1.964 | 0.111 |
| -0.657 | NA | NA | + | NA | NA | 2.21E-01 | 6 | -159.362 | 330.724 | 0 | 0.221 |
| -0.773 | NA | NA | + | -5.68E-04 | NA | 2.32E-01 | 7 | -158.582 | 331.164 | 0.44 | 0.177 |
| -0.6 | NA | NA | + | -6.68E-04 | -8.38E-02 | 2.33E-01 | 8 | -157.78 | 331.561 | 0.837 | 0.145 |
| -0.501 | NA | NA | + | NA | -6.73E-02 | 2.20E-01 | 7 | -158.835 | 331.67 | 0.947 | 0.138 |
| -0.705 | 1.64E-09 | NA | + | NA | NA | 2.30E-01 | 7 | -158.933 | 331.865 | 1.141 | 0.125 |
| -0.833 | 1.81E-09 | NA | + | -6.03E-04 | NA | 2.43E-01 | 8 | -158.055 | 332.11 | 1.386 | 0.11 |
| -0.664 | 1.66E-09 | NA | + | -6.94E-04 | -7.93E-02 | 2.43E-01 | 9 | -157.335 | 332.67 | 1.946 | 0.083 |
| -0.56 | NA | NA | + | NA | NA | 1.93E-01 | 6 | -160.76 | 333.52 | 0 | 0.403 |
| -0.6 | 1.30E-09 | NA | + | NA | NA | 2.00E-01 | 7 | -160.454 | 334.907 | 1.387 | 0.201 |
| -0.442 | NA | NA | + | NA | -5.19E-02 | 1.92E-01 | 7 | -160.47 | 334.94 | 1.42 | 0.198 |
| -0.639 | NA | NA | + | -3.57E-04 | NA | 2.00E-01 | 7 | -160.471 | 334.941 | 1.421 | 0.198 |
| -0.778 | NA | NA | + | -6.45E-04 | NA | 2.31E-01 | 7 | -154.856 | 323.711 | 0 | 0.237 |
| -0.646 | NA | NA | + | NA | NA | 2.20E-01 | 6 | -155.864 | 323.728 | 0.017 | 0.235 |
| -0.839 | 1.69E-09 | NA | + | -6.89E-04 | NA | 2.42E-01 | 8 | -154.357 | 324.714 | 1.003 | 0.144 |
| -0.64 | NA | NA | + | -7.16E-04 | -6.72E-02 | 2.31E-01 | 8 | -154.358 | 324.716 | 1.005 | 0.144 |
| -0.69 | 1.45E-09 | NA | + | NA | NA | 2.28E-01 | 7 | -155.502 | 325.004 | 1.292 | 0.124 |
| -0.53 | NA | NA | + | NA | -5.09E-02 | 2.19E-01 | 7 | -155.576 | 325.152 | 1.441 | 0.115 |
| -0.617 | NA | NA | + | NA | NA | 2.10E-01 | 6 | -156.784 | 325.568 | 0 | 0.328 |
| -0.708 | NA | NA | + | -4.43E-04 | NA | 2.18E-01 | 7 | -156.318 | 326.637 | 1.068 | 0.192 |
| -0.483 | NA | NA | + | NA | -5.94E-02 | 2.11E-01 | 7 | -156.364 | 326.727 | 1.159 | 0.184 |
| -0.66 | 1.61E-09 | NA | + | NA | NA | 2.17E-01 | 7 | -156.439 | 326.878 | 1.31 | 0.17 |
| -0.565 | NA | NA | + | -5.20E-04 | -7.06E-02 | 2.20E-01 | 8 | -155.735 | 327.47 | 1.901 | 0.127 |
| -0.237 | NA | NA | NA | NA | NA | 1.57E-01 | 3 | -140.003 | 286.007 | 0 | 0.257 |
| -0.306 | NA | NA | + | NA | NA | 1.27E-01 | 6 | -137.088 | 286.177 | 0.17 | 0.236 |
| -0.158 | NA | NA | NA | 4.11E-04 | NA | 1.35E-01 | 4 | -139.278 | 286.557 | 0.55 | 0.195 |
| -0.163 | NA | NA | NA | NA | -3.56E-02 | 1.56E-01 | 4 | -139.853 | 287.706 | 1.699 | 0.11 |
| -0.26 | 8.27E-10 | NA | NA | NA | NA | 1.60E-01 | 4 | -139.887 | 287.774 | 1.767 | 0.106 |
| -0.327 | 7.61E-10 | NA | + | NA | NA | 1.30E-01 | 7 | -136.986 | 287.973 | 1.966 | 0.096 |
| -0.489 | NA | NA | + | NA | NA | 1.73E-01 | 6 | -163.451 | 338.903 | 0 | 0.441 |
| -0.526 | 1.34E-09 | NA | + | NA | NA | 1.79E-01 | 7 | -163.223 | 340.447 | 1.544 | 0.204 |
| -0.542 | NA | NA | + | -2.51E-04 | NA | 1.78E-01 | 7 | -163.32 | 340.64 | 1.737 | 0.185 |
| -0.441 | NA | NA | + | NA | -2.19E-02 | 1.73E-01 | 7 | -163.406 | 340.811 | 1.908 | 0.17 |
| -0.501 | NA | NA | + | NA | NA | 1.78E-01 | 6 | -148.774 | 309.547 | 0 | 0.281 |
| -0.617 | NA | NA | + | -5.50E-04 | NA | 1.87E-01 | 7 | -147.998 | 309.995 | 0.448 | 0.225 |
| -0.538 | 9.93E-10 | NA | + | NA | NA | 1.86E-01 | 7 | -148.489 | 310.979 | 1.432 | 0.138 |
| -0.672 | 1.22E-09 | NA | + | -6.01E-04 | NA | 1.98E-01 | 8 | -147.574 | 311.147 | 1.6 | 0.126 |
| -0.416 | NA | NA | + | NA | -3.89E-02 | 1.78E-01 | 7 | -148.611 | 311.222 | 1.675 | 0.122 |
| -0.515 | NA | NA | + | -5.89E-04 | -4.99E-02 | 1.89E-01 | 8 | -147.731 | 311.461 | 1.914 | 0.108 |
| -0.491 | NA | NA | + | NA | NA | 1.80E-01 | 6 | -155.747 | 323.494 | 0 | 0.429 |
| -0.529 | 1.20E-09 | NA | + | NA | NA | 1.87E-01 | 7 | -155.477 | 324.954 | 1.46 | 0.207 |
| -0.557 | NA | NA | + | -3.05E-04 | NA | 1.85E-01 | 7 | -155.538 | 325.076 | 1.582 | 0.194 |
| -0.432 | NA | NA | + | NA | -2.68E-02 | 1.80E-01 | 7 | -155.673 | 325.346 | 1.852 | 0.17 |
| -0.458 | NA | NA | + | NA | NA | 1.72E-01 | 6 | -146.581 | 305.163 | 0 | 0.332 |
| -0.559 | NA | NA | + | -4.65E-04 | NA | 1.81E-01 | 7 | -146.021 | 306.042 | 0.879 | 0.214 |
| -0.342 | NA | NA | + | NA | -5.44E-02 | 1.74E-01 | 7 | -146.245 | 306.49 | 1.327 | 0.171 |
| -0.492 | 9.84E-10 | NA | + | NA | NA | 1.79E-01 | 7 | -146.313 | 306.625 | 1.462 | 0.16 |
| -0.434 | NA | NA | + | -5.11E-04 | -6.30E-02 | 1.83E-01 | 8 | -145.571 | 307.142 | 1.98 | 0.123 |
| -0.436 | NA | NA | + | NA | NA | 1.64E-01 | 6 | -150.045 | 312.091 | 0 | 0.394 |
| -0.546 | NA | NA | + | -4.67E-04 | NA | 1.73E-01 | 7 | -149.525 | 313.05 | 0.959 | 0.244 |
| -0.337 | NA | NA | + | NA | -4.52E-02 | 1.64E-01 | 7 | -149.822 | 313.643 | 1.552 | 0.181 |
| -0.467 | 1.24E-09 | NA | + | NA | NA | 1.68E-01 | 7 | -149.826 | 313.652 | 1.561 | 0.181 |
| -0.463 | NA | NA | + | NA | NA | 1.62E-01 | 6 | -151.994 | 315.988 | 0 | 0.41 |
| -0.563 | NA | NA | + | -4.38E-04 | NA | 1.71E-01 | 7 | -151.552 | 317.103 | 1.115 | 0.235 |
| -0.496 | 1.26E-09 | NA | + | NA | NA | 1.67E-01 | 7 | -151.766 | 317.533 | 1.545 | 0.189 |
| -0.394 | NA | NA | + | NA | -3.09E-02 | 1.63E-01 | 7 | -151.896 | 317.792 | 1.804 | 0.166 |
| -0.657 | NA | NA | + | -1.08E-03 | -1.19E-01 | 2.39E-01 | 8 | -160.805 | 337.611 | 0 | 0.382 |
| -0.731 | 1.74E-09 | NA | + | -1.13E-03 | -1.15E-01 | 2.51E-01 | 9 | -160.097 | 338.193 | 0.583 | 0.286 |
| -0.898 | NA | NA | + | -9.29E-04 | NA | 2.37E-01 | 7 | -162.551 | 339.102 | 1.492 | 0.181 |
| -0.969 | 1.88E-09 | NA | + | -9.87E-04 | NA | 2.50E-01 | 8 | -161.737 | 339.473 | 1.863 | 0.151 |
| -0.826 | NA | NA | + | -6.87E-04 | NA | 2.34E-01 | 7 | -164.835 | 343.669 | 0 | 0.205 |
| -0.69 | NA | NA | + | NA | NA | 2.23E-01 | 6 | -165.935 | 343.869 | 0.2 | 0.186 |
| -0.648 | NA | NA | + | -7.80E-04 | -8.61E-02 | 2.37E-01 | 8 | -164.058 | 344.116 | 0.446 | 0.164 |
| -0.884 | 2.01E-09 | NA | + | -6.99E-04 | NA | 2.43E-01 | 8 | -164.301 | 344.602 | 0.933 | 0.129 |
| -0.744 | 1.94E-09 | NA | + | NA | NA | 2.32E-01 | 7 | -165.445 | 344.89 | 1.221 | 0.111 |
| -0.536 | NA | NA | + | NA | -6.76E-02 | 2.24E-01 | 7 | -165.45 | 344.9 | 1.231 | 0.111 |
| -0.712 | 1.82E-09 | NA | + | -7.85E-04 | -8.08E-02 | 2.45E-01 | 9 | -163.617 | 345.235 | 1.565 | 0.094 |
| -0.437 | NA | NA | + | NA | NA | 1.64E-01 | 6 | -149.214 | 310.428 | 0 | 0.43 |
| -0.519 | NA | NA | + | -3.74E-04 | NA | 1.71E-01 | 7 | -148.887 | 311.773 | 1.345 | 0.219 |
| -0.467 | 1.11E-09 | NA | + | NA | NA | 1.69E-01 | 7 | -149.017 | 312.034 | 1.606 | 0.193 |
| -0.43 | NA | NA | + | NA | -3.42E-03 | 1.64E-01 | 7 | -149.213 | 312.426 | 1.998 | 0.158 |
| -0.814 | NA | NA | + | -7.52E-04 | NA | 2.29E-01 | 7 | -162.205 | 338.41 | 0 | 0.267 |
| -0.671 | NA | NA | + | NA | NA | 2.19E-01 | 6 | -163.526 | 339.052 | 0.642 | 0.194 |
| -0.66 | NA | NA | + | -8.24E-04 | -7.40E-02 | 2.30E-01 | 8 | -161.62 | 339.239 | 0.829 | 0.176 |
| -0.87 | 2.03E-09 | NA | + | -7.72E-04 | NA | 2.37E-01 | 8 | -161.738 | 339.475 | 1.065 | 0.157 |
| -0.72 | 1.87E-09 | NA | + | NA | NA | 2.26E-01 | 7 | -163.134 | 340.268 | 1.858 | 0.105 |
| -0.543 | NA | NA | + | NA | -5.67E-02 | 2.19E-01 | 7 | -163.182 | 340.365 | 1.955 | 0.1 |
| -0.488 | NA | NA | + | NA | NA | 1.73E-01 | 6 | -148.044 | 308.089 | 0 | 0.319 |
| -0.601 | NA | NA | + | -5.03E-04 | NA | 1.83E-01 | 7 | -147.414 | 308.828 | 0.739 | 0.22 |
| -0.362 | NA | NA | + | NA | -5.68E-02 | 1.74E-01 | 7 | -147.678 | 309.356 | 1.268 | 0.169 |
| -0.525 | 1.34E-09 | NA | + | NA | NA | 1.78E-01 | 7 | -147.761 | 309.523 | 1.434 | 0.156 |
| -0.464 | NA | NA | + | -5.64E-04 | -6.79E-02 | 1.85E-01 | 8 | -146.895 | 309.79 | 1.701 | 0.136 |
| -0.458 | NA | NA | + | NA | NA | 1.65E-01 | 6 | -146.815 | 305.629 | 0 | 0.422 |
| -0.546 | NA | NA | + | -3.81E-04 | NA | 1.73E-01 | 7 | -146.462 | 306.925 | 1.296 | 0.221 |
| -0.489 | 1.24E-09 | NA | + | NA | NA | 1.70E-01 | 7 | -146.616 | 307.232 | 1.602 | 0.189 |
| -0.401 | NA | NA | + | NA | -2.65E-02 | 1.67E-01 | 7 | -146.739 | 307.479 | 1.849 | 0.167 |
| -0.711 | NA | NA | + | -1.18E-03 | -1.55E-01 | 2.94E-01 | 8 | -161.744 | 339.487 | 0 | 0.589 |
| -0.798 | 2.45E-09 | NA | + | -1.18E-03 | -1.45E-01 | 3.04E-01 | 9 | -161.102 | 340.203 | 0.716 | 0.411 |
| -0.738 | NA | NA | + | -6.58E-04 | NA | 2.11E-01 | 7 | -151.896 | 317.791 | 0 | 0.205 |
| -0.597 | NA | NA | + | NA | NA | 1.98E-01 | 6 | -152.96 | 317.919 | 0.128 | 0.193 |
| -0.566 | NA | NA | + | -7.46E-04 | -8.25E-02 | 2.11E-01 | 8 | -151.092 | 318.184 | 0.392 | 0.169 |
| -0.793 | 1.72E-09 | NA | + | -6.84E-04 | NA | 2.19E-01 | 8 | -151.435 | 318.87 | 1.079 | 0.12 |
| -0.444 | NA | NA | + | NA | -6.61E-02 | 1.97E-01 | 7 | -152.44 | 318.881 | 1.09 | 0.119 |
| -0.641 | 1.55E-09 | NA | + | NA | NA | 2.05E-01 | 7 | -152.589 | 319.178 | 1.386 | 0.103 |
| -0.624 | 1.57E-09 | NA | + | -7.66E-04 | -7.86E-02 | 2.19E-01 | 9 | -150.706 | 319.412 | 1.621 | 0.091 |
| -0.355 | NA | NA | + | NA | NA | 1.36E-01 | 6 | -146.027 | 304.054 | 0 | 0.385 |
| -0.379 | 7.49E-10 | NA | + | NA | NA | 1.40E-01 | 7 | -145.898 | 305.796 | 1.742 | 0.161 |
| -0.277 | NA | NA | NA | NA | NA | 1.70E-01 | 3 | -149.908 | 305.816 | 1.761 | 0.159 |
| -0.394 | NA | NA | + | -1.67E-04 | NA | 1.39E-01 | 7 | -145.962 | 305.923 | 1.869 | 0.151 |
| -0.328 | NA | NA | + | NA | -1.24E-02 | 1.37E-01 | 7 | -146.011 | 306.023 | 1.968 | 0.144 |
| -0.474 | NA | NA | + | NA | NA | 1.68E-01 | 6 | -162.734 | 337.469 | 0 | 0.452 |
| -0.508 | 1.25E-09 | NA | + | NA | NA | 1.74E-01 | 7 | -162.549 | 339.097 | 1.628 | 0.2 |
| -0.518 | NA | NA | + | -1.91E-04 | NA | 1.73E-01 | 7 | -162.659 | 339.318 | 1.85 | 0.179 |
| -0.454 | NA | NA | + | NA | -9.19E-03 | 1.69E-01 | 7 | -162.726 | 339.453 | 1.984 | 0.168 |
| -0.668 | NA | NA | + | -6.31E-04 | NA | 1.95E-01 | 7 | -147.71 | 309.42 | 0 | 0.217 |
| -0.541 | NA | NA | + | NA | NA | 1.86E-01 | 6 | -148.741 | 309.482 | 0.061 | 0.211 |
| -0.739 | 1.32E-09 | NA | + | -7.20E-04 | NA | 2.09E-01 | 8 | -147.056 | 310.113 | 0.693 | 0.154 |
| -0.549 | NA | NA | + | -6.83E-04 | -6.02E-02 | 1.99E-01 | 8 | -147.291 | 310.582 | 1.162 | 0.121 |
| -0.58 | 9.91E-10 | NA | + | NA | NA | 1.95E-01 | 7 | -148.367 | 310.733 | 1.313 | 0.113 |
| -0.44 | NA | NA | + | NA | -4.71E-02 | 1.88E-01 | 7 | -148.484 | 310.968 | 1.548 | 0.1 |
| -0.622 | 1.30E-09 | NA | + | -7.70E-04 | -5.87E-02 | 2.12E-01 | 9 | -146.654 | 311.308 | 1.888 | 0.084 |
| -0.662 | NA | NA | + | NA | NA | 2.19E-01 | 6 | -163.402 | 338.805 | 0 | 0.251 |
| -0.783 | NA | NA | + | -5.86E-04 | NA | 2.31E-01 | 7 | -162.627 | 339.253 | 0.448 | 0.201 |
| -0.511 | NA | NA | + | NA | -6.54E-02 | 2.18E-01 | 7 | -162.914 | 339.827 | 1.022 | 0.151 |
| -0.618 | NA | NA | + | -6.66E-04 | -7.87E-02 | 2.31E-01 | 8 | -161.925 | 339.85 | 1.045 | 0.149 |
| -0.713 | 1.89E-09 | NA | + | NA | NA | 2.28E-01 | 7 | -163.017 | 340.034 | 1.229 | 0.136 |
| -0.84 | 1.99E-09 | NA | + | -6.01E-04 | NA | 2.39E-01 | 8 | -162.197 | 340.394 | 1.59 | 0.113 |
| -0.706 | NA | NA | + | -6.73E-04 | NA | 2.09E-01 | 7 | -152.358 | 318.716 | 0 | 0.228 |
| -0.559 | NA | NA | + | NA | NA | 1.97E-01 | 6 | -153.52 | 319.039 | 0.323 | 0.194 |
| -0.56 | NA | NA | + | -7.53E-04 | -7.34E-02 | 2.12E-01 | 8 | -151.747 | 319.494 | 0.778 | 0.154 |
| -0.762 | 1.63E-09 | NA | + | -7.05E-04 | NA | 2.18E-01 | 8 | -151.881 | 319.761 | 1.046 | 0.135 |
| -0.602 | 1.43E-09 | NA | + | NA | NA | 2.05E-01 | 7 | -153.158 | 320.316 | 1.601 | 0.102 |
| -0.437 | NA | NA | + | NA | -5.50E-02 | 1.98E-01 | 7 | -153.173 | 320.347 | 1.631 | 0.101 |
| -0.619 | 1.54E-09 | NA | + | -7.80E-04 | -7.00E-02 | 2.20E-01 | 9 | -151.323 | 320.646 | 1.93 | 0.087 |
| -0.288 | NA | NA | NA | NA | NA | 1.74E-01 | 3 | -154.263 | 314.527 | 0 | 0.253 |
| -0.359 | NA | NA | + | NA | NA | 1.46E-01 | 6 | -151.288 | 314.575 | 0.049 | 0.247 |
| -0.206 | NA | NA | NA | 4.52E-04 | NA | 1.54E-01 | 4 | -153.546 | 315.092 | 0.565 | 0.191 |
| -0.312 | 8.56E-10 | NA | NA | NA | NA | 1.78E-01 | 4 | -154.127 | 316.253 | 1.727 | 0.107 |
| -0.382 | 8.26E-10 | NA | + | NA | NA | 1.50E-01 | 7 | -151.156 | 316.312 | 1.786 | 0.104 |
| -0.246 | NA | NA | NA | NA | -2.12E-02 | 1.74E-01 | 4 | -154.218 | 316.436 | 1.909 | 0.098 |
| -0.279 | NA | NA | NA | NA | NA | 1.73E-01 | 3 | -162.114 | 330.228 | 0 | 0.239 |
| -0.178 | NA | NA | NA | 5.47E-04 | NA | 1.46E-01 | 4 | -161.17 | 330.34 | 0.112 | 0.226 |
| -0.357 | NA | NA | + | NA | NA | 1.39E-01 | 6 | -159.443 | 330.887 | 0.659 | 0.172 |
| -0.302 | 8.41E-10 | NA | NA | NA | NA | 1.77E-01 | 4 | -162.021 | 332.042 | 1.814 | 0.096 |
| -0.236 | NA | NA | NA | 5.90E-04 | 3.27E-02 | 1.43E-01 | 5 | -161.078 | 332.157 | 1.929 | 0.091 |
| -0.199 | 7.10E-10 | NA | NA | 5.40E-04 | NA | 1.49E-01 | 5 | -161.103 | 332.205 | 1.977 | 0.089 |
| -0.291 | NA | NA | NA | NA | 6.14E-03 | 1.73E-01 | 4 | -162.111 | 332.221 | 1.993 | 0.088 |
| -0.384 | NA | NA | + | NA | NA | 1.50E-01 | 6 | -158.509 | 329.017 | 0 | 0.31 |
| -0.324 | NA | NA | NA | NA | NA | 1.89E-01 | 3 | -161.973 | 329.946 | 0.928 | 0.195 |
| -0.235 | NA | NA | NA | 4.58E-04 | NA | 1.65E-01 | 4 | -161.308 | 330.616 | 1.598 | 0.139 |
| -0.409 | 9.33E-10 | NA | + | NA | NA | 1.54E-01 | 7 | -158.393 | 330.785 | 1.768 | 0.128 |
| -0.397 | NA | NA | + | NA | 6.00E-03 | 1.49E-01 | 7 | -158.505 | 331.011 | 1.993 | 0.114 |
| -0.389 | NA | NA | + | -2.29E-05 | NA | 1.50E-01 | 7 | -158.508 | 331.015 | 1.998 | 0.114 |
| -0.585 | NA | NA | + | NA | NA | 2.01E-01 | 6 | -160.671 | 333.342 | 0 | 0.385 |
| -0.689 | NA | NA | + | -5.21E-04 | NA | 2.09E-01 | 7 | -160.053 | 334.105 | 0.763 | 0.263 |
| -0.625 | 1.41E-09 | NA | + | NA | NA | 2.07E-01 | 7 | -160.371 | 334.743 | 1.4 | 0.191 |
| -0.504 | NA | NA | + | NA | -3.54E-02 | 2.01E-01 | 7 | -160.549 | 335.099 | 1.756 | 0.16 |
| -0.435 | NA | NA | + | NA | NA | 1.60E-01 | 6 | -152.022 | 316.044 | 0 | 0.449 |
| -0.465 | 1.07E-09 | NA | + | NA | NA | 1.65E-01 | 7 | -151.841 | 317.682 | 1.638 | 0.198 |
| -0.484 | NA | NA | + | -2.01E-04 | NA | 1.64E-01 | 7 | -151.929 | 317.858 | 1.815 | 0.181 |
| -0.392 | NA | NA | + | NA | -1.93E-02 | 1.61E-01 | 7 | -151.984 | 317.968 | 1.924 | 0.172 |
| -0.424 | NA | NA | + | NA | NA | 1.59E-01 | 6 | -158.353 | 328.706 | 0 | 0.392 |
| -0.451 | 9.75E-10 | NA | + | NA | NA | 1.63E-01 | 7 | -158.211 | 330.421 | 1.715 | 0.166 |
| -0.353 | NA | NA | NA | NA | NA | 1.96E-01 | 3 | -162.305 | 330.61 | 1.903 | 0.151 |
| -0.451 | NA | NA | + | NA | 1.24E-02 | 1.58E-01 | 7 | -158.339 | 330.679 | 1.972 | 0.146 |
| -0.436 | NA | NA | + | -5.11E-05 | NA | 1.60E-01 | 7 | -158.348 | 330.696 | 1.989 | 0.145 |
| -0.537 | NA | NA | + | -8.71E-04 | -1.07E-01 | 2.21E-01 | 8 | -150.22 | 316.44 | 0 | 0.293 |
| -0.754 | NA | NA | + | -7.49E-04 | NA | 2.19E-01 | 7 | -151.688 | 317.376 | 0.937 | 0.183 |
| -0.598 | 1.65E-09 | NA | + | -8.87E-04 | -1.02E-01 | 2.29E-01 | 9 | -149.763 | 317.527 | 1.087 | 0.17 |
| -0.812 | 1.89E-09 | NA | + | -7.73E-04 | NA | 2.28E-01 | 8 | -151.101 | 318.201 | 1.762 | 0.121 |
| -0.6 | NA | NA | + | NA | NA | 2.05E-01 | 6 | -153.135 | 318.27 | 1.83 | 0.117 |
| -0.404 | NA | NA | + | NA | -8.74E-02 | 2.06E-01 | 7 | -152.156 | 318.313 | 1.873 | 0.115 |
| -0.472 | NA | NA | + | NA | NA | 1.76E-01 | 6 | -156.365 | 324.731 | 0 | 0.447 |
| -0.511 | 1.50E-09 | NA | + | NA | NA | 1.82E-01 | 7 | -156.09 | 326.179 | 1.448 | 0.217 |
| -0.505 | NA | NA | + | -1.40E-04 | NA | 1.79E-01 | 7 | -156.323 | 326.647 | 1.916 | 0.172 |
| -0.475 | NA | NA | + | NA | 1.21E-03 | 1.76E-01 | 7 | -156.365 | 326.731 | 2 | 0.165 |
| -0.443 | NA | NA | + | NA | NA | 1.71E-01 | 6 | -142.921 | 297.841 | 0 | 0.414 |
| -0.515 | NA | NA | + | -3.26E-04 | NA | 1.78E-01 | 7 | -142.652 | 299.305 | 1.464 | 0.199 |
| -0.343 | NA | NA | + | NA | -4.68E-02 | 1.73E-01 | 7 | -142.656 | 299.312 | 1.471 | 0.198 |
| -0.473 | 9.79E-10 | NA | + | NA | NA | 1.78E-01 | 7 | -142.705 | 299.409 | 1.568 | 0.189 |
| -0.482 | NA | NA | + | NA | NA | 1.70E-01 | 6 | -140.18 | 292.36 | 0 | 0.211 |
| -0.621 | NA | NA | + | -5.99E-04 | NA | 1.80E-01 | 7 | -139.239 | 292.478 | 0.118 | 0.199 |
| -0.468 | NA | NA | + | -6.80E-04 | -7.75E-02 | 1.84E-01 | 8 | -138.458 | 292.916 | 0.556 | 0.16 |
| -0.341 | NA | NA | + | NA | -6.33E-02 | 1.71E-01 | 7 | -139.655 | 293.31 | 0.949 | 0.132 |
| -0.669 | 1.40E-09 | NA | + | -6.25E-04 | NA | 1.88E-01 | 8 | -138.843 | 293.685 | 1.325 | 0.109 |
| -0.52 | 1.25E-09 | NA | + | NA | NA | 1.76E-01 | 7 | -139.867 | 293.734 | 1.374 | 0.106 |
| -0.519 | 1.28E-09 | NA | + | -7.00E-04 | -7.42E-02 | 1.91E-01 | 9 | -138.128 | 294.255 | 1.895 | 0.082 |
| -0.407 | NA | NA | + | NA | NA | 1.55E-01 | 6 | -152.186 | 316.372 | 0 | 0.373 |
| -0.341 | NA | NA | NA | NA | NA | 1.91E-01 | 3 | -155.913 | 317.826 | 1.453 | 0.18 |
| -0.437 | 1.07E-09 | NA | + | NA | NA | 1.60E-01 | 7 | -152.006 | 318.013 | 1.64 | 0.164 |
| -0.368 | NA | NA | + | NA | -1.83E-02 | 1.56E-01 | 7 | -152.153 | 318.305 | 1.933 | 0.142 |
| -0.431 | NA | NA | + | -1.04E-04 | NA | 1.57E-01 | 7 | -152.162 | 318.324 | 1.952 | 0.141 |
| -0.369 | NA | NA | + | NA | NA | 1.50E-01 | 6 | -137.482 | 286.964 | 0 | 0.365 |
| -0.329 | NA | NA | NA | NA | NA | 1.86E-01 | 3 | -141.213 | 288.425 | 1.462 | 0.176 |
| -0.397 | 1.07E-09 | NA | + | NA | NA | 1.54E-01 | 7 | -137.306 | 288.612 | 1.648 | 0.16 |
| -0.425 | NA | NA | + | -2.38E-04 | NA | 1.55E-01 | 7 | -137.339 | 288.679 | 1.715 | 0.155 |
| -0.319 | NA | NA | + | NA | -2.43E-02 | 1.51E-01 | 7 | -137.411 | 288.823 | 1.859 | 0.144 |
| -0.584 | NA | NA | + | NA | NA | 2.02E-01 | 6 | -160.239 | 332.478 | 0 | 0.381 |
| -0.444 | NA | NA | + | NA | -6.23E-02 | 2.02E-01 | 7 | -159.819 | 333.638 | 1.16 | 0.213 |
| -0.629 | 1.32E-09 | NA | + | NA | NA | 2.12E-01 | 7 | -159.857 | 333.713 | 1.235 | 0.205 |
| -0.667 | NA | NA | + | -3.91E-04 | NA | 2.10E-01 | 7 | -159.882 | 333.764 | 1.286 | 0.2 |
| -0.378 | NA | NA | + | NA | NA | 1.45E-01 | 6 | -148.577 | 309.154 | 0 | 0.31 |
| -0.212 | NA | NA | NA | 5.12E-04 | NA | 1.55E-01 | 4 | -151.215 | 310.43 | 1.276 | 0.164 |
| -0.319 | NA | NA | NA | NA | NA | 1.83E-01 | 3 | -152.237 | 310.474 | 1.32 | 0.16 |
| -0.406 | 9.36E-10 | NA | + | NA | NA | 1.50E-01 | 7 | -148.413 | 310.827 | 1.672 | 0.134 |
| -0.343 | NA | NA | + | NA | -1.60E-02 | 1.46E-01 | 7 | -148.551 | 311.102 | 1.947 | 0.117 |
| -0.367 | NA | NA | + | 4.14E-05 | NA | 1.44E-01 | 7 | -148.573 | 311.146 | 1.992 | 0.115 |
| -0.489 | NA | NA | + | NA | NA | 1.74E-01 | 6 | -145.376 | 302.752 | 0 | 0.291 |
| -0.605 | NA | NA | + | -5.37E-04 | NA | 1.83E-01 | 7 | -144.644 | 303.287 | 0.535 | 0.223 |
| -0.521 | 1.20E-09 | NA | + | NA | NA | 1.79E-01 | 7 | -145.121 | 304.243 | 1.491 | 0.138 |
| -0.407 | NA | NA | + | NA | -3.90E-02 | 1.76E-01 | 7 | -145.204 | 304.408 | 1.656 | 0.127 |
| -0.646 | 1.33E-09 | NA | + | -5.57E-04 | NA | 1.89E-01 | 8 | -144.331 | 304.661 | 1.909 | 0.112 |
| -0.51 | NA | NA | + | -5.75E-04 | -4.90E-02 | 1.87E-01 | 8 | -144.373 | 304.746 | 1.994 | 0.108 |
| -0.424 | NA | NA | + | NA | NA | 1.58E-01 | 6 | -141.147 | 294.294 | 0 | 0.39 |
| -0.535 | NA | NA | + | -4.91E-04 | NA | 1.69E-01 | 7 | -140.52 | 295.039 | 0.745 | 0.269 |
| -0.453 | 1.13E-09 | NA | + | NA | NA | 1.63E-01 | 7 | -140.943 | 295.885 | 1.591 | 0.176 |
| -0.35 | NA | NA | + | NA | -3.46E-02 | 1.60E-01 | 7 | -141.01 | 296.02 | 1.726 | 0.165 |
| -0.408 | NA | NA | + | NA | NA | 1.51E-01 | 6 | -160.49 | 332.98 | 0 | 0.274 |
| -0.333 | NA | NA | NA | NA | NA | 1.90E-01 | 3 | -163.761 | 333.523 | 0.542 | 0.209 |
| -0.229 | NA | NA | NA | 5.50E-04 | NA | 1.63E-01 | 4 | -162.839 | 333.678 | 0.698 | 0.194 |
| -0.437 | 1.03E-09 | NA | + | NA | NA | 1.56E-01 | 7 | -160.349 | 334.697 | 1.717 | 0.116 |
| -0.444 | NA | NA | + | NA | 1.62E-02 | 1.50E-01 | 7 | -160.467 | 334.934 | 1.953 | 0.103 |
| -0.384 | NA | NA | + | 9.98E-05 | NA | 1.49E-01 | 7 | -160.471 | 334.942 | 1.961 | 0.103 |
| -0.439 | NA | NA | + | NA | NA | 1.60E-01 | 6 | -148.934 | 309.868 | 0 | 0.434 |
| -0.517 | NA | NA | + | -3.38E-04 | NA | 1.66E-01 | 7 | -148.668 | 311.336 | 1.468 | 0.208 |
| -0.468 | 1.04E-09 | NA | + | NA | NA | 1.64E-01 | 7 | -148.767 | 311.533 | 1.665 | 0.189 |
| -0.39 | NA | NA | + | NA | -2.23E-02 | 1.61E-01 | 7 | -148.883 | 311.766 | 1.898 | 0.168 |
| -0.641 | NA | NA | + | NA | NA | 2.18E-01 | 6 | -165.214 | 342.428 | 0 | 0.386 |
| -0.688 | 1.67E-09 | NA | + | NA | NA | 2.27E-01 | 7 | -164.819 | 343.637 | 1.209 | 0.211 |
| -0.726 | NA | NA | + | -4.14E-04 | NA | 2.26E-01 | 7 | -164.837 | 343.673 | 1.245 | 0.207 |
| -0.516 | NA | NA | + | NA | -5.48E-02 | 2.18E-01 | 7 | -164.889 | 343.778 | 1.35 | 0.196 |
| -0.495 | NA | NA | + | NA | NA | 1.75E-01 | 6 | -153.347 | 318.694 | 0 | 0.431 |
| -0.529 | 1.22E-09 | NA | + | NA | NA | 1.80E-01 | 7 | -153.104 | 320.208 | 1.514 | 0.202 |
| -0.566 | NA | NA | + | -3.14E-04 | NA | 1.81E-01 | 7 | -153.123 | 320.246 | 1.552 | 0.198 |
| -0.439 | NA | NA | + | NA | -2.56E-02 | 1.76E-01 | 7 | -153.281 | 320.562 | 1.868 | 0.169 |
| -0.431 | NA | NA | + | NA | NA | 1.62E-01 | 6 | -143.965 | 299.93 | 0 | 0.427 |
| -0.506 | NA | NA | + | -3.27E-04 | NA | 1.69E-01 | 7 | -143.702 | 301.403 | 1.474 | 0.204 |
| -0.46 | 9.89E-10 | NA | + | NA | NA | 1.67E-01 | 7 | -143.762 | 301.523 | 1.593 | 0.192 |
| -0.363 | NA | NA | + | NA | -3.24E-02 | 1.64E-01 | 7 | -143.846 | 301.692 | 1.763 | 0.177 |
| -0.476 | NA | NA | + | NA | NA | 1.71E-01 | 6 | -155.731 | 323.463 | 0 | 0.434 |
| -0.509 | 1.10E-09 | NA | + | NA | NA | 1.76E-01 | 7 | -155.507 | 325.014 | 1.552 | 0.2 |
| -0.537 | NA | NA | + | -2.78E-04 | NA | 1.75E-01 | 7 | -155.555 | 325.11 | 1.647 | 0.191 |
| -0.414 | NA | NA | + | NA | -2.96E-02 | 1.72E-01 | 7 | -155.64 | 325.281 | 1.818 | 0.175 |
| -0.148 | NA | NA | NA | 5.29E-04 | NA | 1.33E-01 | 4 | -154.807 | 317.614 | 0 | 0.229 |
| -0.247 | NA | NA | NA | NA | NA | 1.60E-01 | 3 | -155.807 | 317.614 | 0 | 0.229 |
| -0.322 | NA | NA | + | NA | NA | 1.27E-01 | 6 | -153.028 | 318.055 | 0.442 | 0.184 |
| -0.271 | 7.62E-10 | NA | NA | NA | NA | 1.65E-01 | 4 | -155.688 | 319.376 | 1.763 | 0.095 |
| -0.169 | 5.75E-10 | NA | NA | 5.17E-04 | NA | 1.38E-01 | 5 | -154.739 | 319.478 | 1.864 | 0.09 |
| -0.175 | NA | NA | NA | 5.49E-04 | 1.48E-02 | 1.33E-01 | 5 | -154.787 | 319.574 | 1.96 | 0.086 |
| -0.22 | NA | NA | NA | NA | -1.30E-02 | 1.60E-01 | 4 | -155.791 | 319.581 | 1.968 | 0.086 |
| -0.384 | NA | NA | + | NA | NA | 1.44E-01 | 6 | -154.468 | 320.937 | 0 | 0.371 |
| -0.313 | NA | NA | NA | NA | NA | 1.86E-01 | 3 | -158.128 | 322.256 | 1.319 | 0.192 |
| -0.41 | 8.15E-10 | NA | + | NA | NA | 1.50E-01 | 7 | -154.332 | 322.663 | 1.726 | 0.156 |
| -0.346 | NA | NA | + | NA | -1.75E-02 | 1.45E-01 | 7 | -154.438 | 322.877 | 1.94 | 0.141 |
| -0.409 | NA | NA | + | -1.07E-04 | NA | 1.47E-01 | 7 | -154.443 | 322.886 | 1.949 | 0.14 |
| -0.426 | NA | NA | + | NA | NA | 1.51E-01 | 6 | -142.425 | 296.85 | 0 | 0.406 |
| -0.468 | 7.47E-10 | NA | + | NA | NA | 1.64E-01 | 7 | -142.072 | 298.144 | 1.294 | 0.213 |
| -0.496 | NA | NA | + | -3.13E-04 | NA | 1.56E-01 | 7 | -142.154 | 298.308 | 1.458 | 0.196 |
| -0.327 | NA | NA | + | NA | -4.45E-02 | 1.53E-01 | 7 | -142.207 | 298.413 | 1.564 | 0.186 |
| -0.429 | NA | NA | + | NA | NA | 1.58E-01 | 6 | -149.472 | 310.944 | 0 | 0.44 |
| -0.498 | NA | NA | + | -2.86E-04 | NA | 1.64E-01 | 7 | -149.282 | 312.563 | 1.619 | 0.196 |
| -0.459 | 1.13E-09 | NA | + | NA | NA | 1.62E-01 | 7 | -149.301 | 312.603 | 1.659 | 0.192 |
| -0.378 | NA | NA | + | NA | -2.34E-02 | 1.59E-01 | 7 | -149.415 | 312.829 | 1.885 | 0.172 |
| -0.57 | NA | NA | + | -8.06E-04 | -1.05E-01 | 2.35E-01 | 8 | -156.017 | 328.034 | 0 | 0.248 |
| -0.788 | NA | NA | + | -6.87E-04 | NA | 2.34E-01 | 7 | -157.357 | 328.715 | 0.681 | 0.177 |
| -0.643 | 1.68E-09 | NA | + | -8.54E-04 | -1.01E-01 | 2.46E-01 | 9 | -155.424 | 328.848 | 0.814 | 0.165 |
| -0.649 | NA | NA | + | NA | NA | 2.21E-01 | 6 | -158.552 | 329.104 | 1.07 | 0.145 |
| -0.451 | NA | NA | + | NA | -8.65E-02 | 2.19E-01 | 7 | -157.643 | 329.287 | 1.253 | 0.133 |
| -0.858 | 1.84E-09 | NA | + | -7.45E-04 | NA | 2.47E-01 | 8 | -156.651 | 329.303 | 1.269 | 0.132 |
| -0.421 | NA | NA | + | NA | NA | 1.52E-01 | 6 | -145.408 | 302.816 | 0 | 0.415 |
| -0.526 | NA | NA | + | -4.41E-04 | NA | 1.62E-01 | 7 | -144.929 | 303.858 | 1.041 | 0.246 |
| -0.445 | 7.42E-10 | NA | + | NA | NA | 1.57E-01 | 7 | -145.277 | 304.553 | 1.737 | 0.174 |
| -0.363 | NA | NA | + | NA | -2.68E-02 | 1.54E-01 | 7 | -145.332 | 304.664 | 1.848 | 0.165 |
| -0.377 | NA | NA | + | NA | NA | 1.55E-01 | 6 | -141.302 | 294.604 | 0 | 0.432 |
| -0.439 | NA | NA | + | -2.72E-04 | NA | 1.61E-01 | 7 | -141.112 | 296.225 | 1.62 | 0.192 |
| -0.404 | 1.08E-09 | NA | + | NA | NA | 1.59E-01 | 7 | -141.112 | 296.225 | 1.62 | 0.192 |
| -0.299 | NA | NA | + | NA | -3.63E-02 | 1.56E-01 | 7 | -141.153 | 296.307 | 1.702 | 0.184 |
| -0.404 | NA | NA | + | NA | NA | 1.47E-01 | 6 | -159.673 | 331.345 | 0 | 0.323 |
| -0.328 | NA | NA | NA | NA | NA | 1.86E-01 | 3 | -163.289 | 332.578 | 1.232 | 0.174 |
| -0.432 | 8.91E-10 | NA | + | NA | NA | 1.52E-01 | 7 | -159.532 | 333.064 | 1.719 | 0.137 |
| -0.238 | NA | NA | NA | 4.61E-04 | NA | 1.62E-01 | 4 | -162.597 | 333.194 | 1.848 | 0.128 |
| -0.418 | NA | NA | + | NA | 6.55E-03 | 1.46E-01 | 7 | -159.669 | 333.338 | 1.993 | 0.119 |
| -0.409 | NA | NA | + | -1.89E-05 | NA | 1.47E-01 | 7 | -159.672 | 333.344 | 1.999 | 0.119 |
| -0.464 | NA | NA | + | NA | NA | 1.65E-01 | 6 | -153.583 | 319.167 | 0 | 0.446 |
| -0.536 | NA | NA | + | -2.92E-04 | NA | 1.73E-01 | 7 | -153.392 | 320.784 | 1.617 | 0.199 |
| -0.492 | 1.03E-09 | NA | + | NA | NA | 1.69E-01 | 7 | -153.436 | 320.873 | 1.706 | 0.19 |
| -0.44 | NA | NA | + | NA | -1.08E-02 | 1.65E-01 | 7 | -153.572 | 321.144 | 1.977 | 0.166 |
| -0.459 | NA | NA | + | -6.79E-04 | -8.81E-02 | 1.94E-01 | 8 | -137.358 | 290.716 | 0 | 0.188 |
| -0.637 | NA | NA | + | -6.03E-04 | NA | 1.89E-01 | 7 | -138.367 | 290.734 | 0.018 | 0.186 |
| -0.511 | NA | NA | + | NA | NA | 1.79E-01 | 6 | -139.393 | 290.787 | 0.07 | 0.181 |
| -0.345 | NA | NA | + | NA | -7.53E-02 | 1.82E-01 | 7 | -138.656 | 291.311 | 0.595 | 0.14 |
| -0.689 | 1.27E-09 | NA | + | -6.45E-04 | NA | 1.99E-01 | 8 | -137.905 | 291.81 | 1.093 | 0.109 |
| -0.512 | 1.19E-09 | NA | + | -7.17E-04 | -8.58E-02 | 2.02E-01 | 9 | -136.944 | 291.889 | 1.172 | 0.105 |
| -0.546 | 1.06E-09 | NA | + | NA | NA | 1.86E-01 | 7 | -139.073 | 292.146 | 1.43 | 0.092 |
| -0.834 | NA | NA | + | -7.25E-04 | NA | 2.28E-01 | 7 | -165.136 | 344.271 | 0 | 0.211 |
| -0.651 | NA | NA | + | -8.12E-04 | -8.80E-02 | 2.31E-01 | 8 | -164.306 | 344.612 | 0.34 | 0.178 |
| -0.695 | NA | NA | + | NA | NA | 2.17E-01 | 6 | -166.376 | 344.751 | 0.48 | 0.166 |
| -0.898 | 1.73E-09 | NA | + | -7.69E-04 | NA | 2.40E-01 | 8 | -164.561 | 345.122 | 0.851 | 0.138 |
| -0.718 | 1.61E-09 | NA | + | -8.50E-04 | -8.40E-02 | 2.42E-01 | 9 | -163.803 | 345.607 | 1.335 | 0.108 |
| -0.535 | NA | NA | + | NA | -7.04E-02 | 2.19E-01 | 7 | -165.844 | 345.688 | 1.416 | 0.104 |
| -0.742 | 1.49E-09 | NA | + | NA | NA | 2.27E-01 | 7 | -165.955 | 345.91 | 1.639 | 0.093 |
| -0.477 | NA | NA | + | NA | NA | 1.65E-01 | 6 | -160.436 | 332.872 | 0 | 0.447 |
| -0.511 | 1.26E-09 | NA | + | NA | NA | 1.70E-01 | 7 | -160.237 | 334.475 | 1.603 | 0.201 |
| -0.534 | NA | NA | + | -2.54E-04 | NA | 1.70E-01 | 7 | -160.303 | 334.606 | 1.734 | 0.188 |
| -0.468 | NA | NA | + | NA | -3.98E-03 | 1.65E-01 | 7 | -160.434 | 334.869 | 1.997 | 0.165 |
| -0.599 | NA | NA | + | NA | NA | 2.03E-01 | 6 | -156.92 | 325.839 | 0 | 0.326 |
| -0.703 | NA | NA | + | -4.88E-04 | NA | 2.12E-01 | 7 | -156.36 | 326.721 | 0.881 | 0.21 |
| -0.473 | NA | NA | + | NA | -5.54E-02 | 2.04E-01 | 7 | -156.568 | 327.136 | 1.297 | 0.171 |
| -0.641 | 1.58E-09 | NA | + | NA | NA | 2.10E-01 | 7 | -156.594 | 327.188 | 1.349 | 0.166 |
| -0.566 | NA | NA | + | -5.53E-04 | -6.64E-02 | 2.14E-01 | 8 | -155.861 | 327.722 | 1.883 | 0.127 |
| -0.411 | NA | NA | + | NA | NA | 1.54E-01 | 6 | -156.475 | 324.949 | 0 | 0.375 |
| -0.356 | NA | NA | NA | NA | NA | 1.96E-01 | 3 | -160.271 | 326.542 | 1.593 | 0.169 |
| -0.438 | 9.00E-10 | NA | + | NA | NA | 1.59E-01 | 7 | -156.319 | 326.638 | 1.688 | 0.161 |
| -0.478 | NA | NA | + | NA | 3.15E-02 | 1.52E-01 | 7 | -156.387 | 326.773 | 1.824 | 0.151 |
| -0.447 | NA | NA | + | -1.53E-04 | NA | 1.56E-01 | 7 | -156.426 | 326.853 | 1.903 | 0.145 |
| -0.426 | NA | NA | + | NA | NA | 1.57E-01 | 6 | -151.217 | 314.433 | 0 | 0.452 |
| -0.454 | 1.02E-09 | NA | + | NA | NA | 1.61E-01 | 7 | -151.067 | 316.135 | 1.702 | 0.193 |
| -0.482 | NA | NA | + | -2.39E-04 | NA | 1.63E-01 | 7 | -151.09 | 316.18 | 1.747 | 0.189 |
| -0.424 | NA | NA | + | NA | -1.26E-03 | 1.57E-01 | 7 | -151.216 | 316.433 | 2 | 0.166 |
| -0.458 | NA | NA | + | NA | NA | 1.65E-01 | 6 | -149.31 | 310.62 | 0 | 0.438 |
| -0.488 | 1.06E-09 | NA | + | NA | NA | 1.70E-01 | 7 | -149.125 | 312.25 | 1.63 | 0.194 |
| -0.517 | NA | NA | + | -2.54E-04 | NA | 1.71E-01 | 7 | -149.159 | 312.318 | 1.699 | 0.187 |
| -0.382 | NA | NA | + | NA | -3.38E-02 | 1.65E-01 | 7 | -149.193 | 312.386 | 1.766 | 0.181 |
| -0.626 | NA | NA | + | -9.29E-04 | -9.57E-02 | 2.36E-01 | 8 | -153.994 | 323.988 | 0 | 0.283 |
| -0.82 | NA | NA | + | -8.22E-04 | NA | 2.36E-01 | 7 | -155.059 | 324.119 | 0.131 | 0.265 |
| -0.877 | 2.05E-09 | NA | + | -8.30E-04 | NA | 2.45E-01 | 8 | -154.531 | 325.061 | 1.074 | 0.166 |
| -0.687 | 1.78E-09 | NA | + | -9.30E-04 | -9.00E-02 | 2.44E-01 | 9 | -153.591 | 325.183 | 1.195 | 0.156 |
| -0.653 | NA | NA | + | NA | NA | 2.22E-01 | 6 | -156.778 | 325.555 | 1.568 | 0.129 |
| -0.339 | NA | NA | + | NA | NA | 1.34E-01 | 6 | -146.852 | 305.704 | 0 | 0.369 |
| -0.293 | NA | NA | NA | NA | NA | 1.75E-01 | 3 | -150.471 | 306.943 | 1.239 | 0.199 |
| -0.361 | 8.59E-10 | NA | + | NA | NA | 1.38E-01 | 7 | -146.744 | 307.487 | 1.783 | 0.151 |
| -0.381 | NA | NA | + | -1.70E-04 | NA | 1.38E-01 | 7 | -146.787 | 307.574 | 1.87 | 0.145 |
| -0.337 | NA | NA | + | NA | -1.07E-03 | 1.34E-01 | 7 | -146.852 | 307.704 | 2 | 0.136 |
| -0.364 | NA | NA | + | NA | NA | 1.42E-01 | 6 | -148.179 | 308.357 | 0 | 0.32 |
| -0.309 | NA | NA | NA | NA | NA | 1.84E-01 | 3 | -151.761 | 309.522 | 1.165 | 0.179 |
| -0.392 | 1.04E-09 | NA | + | NA | NA | 1.47E-01 | 7 | -148.014 | 310.028 | 1.671 | 0.139 |
| -0.224 | NA | NA | NA | 4.01E-04 | NA | 1.60E-01 | 4 | -151.145 | 310.291 | 1.934 | 0.122 |
| -0.39 | NA | NA | + | -1.03E-04 | NA | 1.45E-01 | 7 | -148.154 | 310.308 | 1.951 | 0.121 |
| -0.332 | NA | NA | + | NA | -1.48E-02 | 1.43E-01 | 7 | -148.156 | 310.313 | 1.956 | 0.12 |
| -0.89 | NA | NA | + | -6.75E-04 | NA | 2.59E-01 | 7 | -170.382 | 354.763 | 0 | 0.19 |
| -0.766 | NA | NA | + | NA | NA | 2.49E-01 | 6 | -171.428 | 354.855 | 0.092 | 0.181 |
| -0.694 | NA | NA | + | -7.75E-04 | -9.19E-02 | 2.58E-01 | 8 | -169.45 | 354.899 | 0.136 | 0.177 |
| -0.592 | NA | NA | + | NA | -7.46E-02 | 2.46E-01 | 7 | -170.809 | 355.618 | 0.854 | 0.124 |
| -0.954 | 2.11E-09 | NA | + | -7.06E-04 | NA | 2.70E-01 | 8 | -169.823 | 355.645 | 0.882 | 0.122 |
| -0.819 | 1.92E-09 | NA | + | NA | NA | 2.58E-01 | 7 | -170.969 | 355.939 | 1.175 | 0.106 |
| -0.764 | 1.83E-09 | NA | + | -7.95E-04 | -8.51E-02 | 2.67E-01 | 9 | -169.028 | 356.056 | 1.293 | 0.1 |
| -0.632 | NA | NA | + | NA | NA | 2.12E-01 | 6 | -162.109 | 336.219 | 0 | 0.281 |
| -0.754 | NA | NA | + | -5.97E-04 | NA | 2.24E-01 | 7 | -161.319 | 336.638 | 0.42 | 0.227 |
| -0.677 | 1.68E-09 | NA | + | NA | NA | 2.19E-01 | 7 | -161.778 | 337.555 | 1.337 | 0.144 |
| -0.807 | 1.82E-09 | NA | + | -6.18E-04 | NA | 2.32E-01 | 8 | -160.928 | 337.855 | 1.637 | 0.124 |
| -0.55 | NA | NA | + | NA | -3.71E-02 | 2.13E-01 | 7 | -161.974 | 337.949 | 1.73 | 0.118 |
| -0.653 | NA | NA | + | -6.40E-04 | -4.97E-02 | 2.26E-01 | 8 | -161.079 | 338.157 | 1.939 | 0.106 |
| -0.371 | NA | NA | + | NA | NA | 1.46E-01 | 6 | -133.762 | 279.523 | 0 | 0.407 |
| -0.456 | NA | NA | + | -3.60E-04 | NA | 1.55E-01 | 7 | -133.4 | 280.801 | 1.278 | 0.215 |
| -0.269 | NA | NA | + | NA | -4.71E-02 | 1.48E-01 | 7 | -133.489 | 280.978 | 1.455 | 0.197 |
| -0.398 | 7.65E-10 | NA | + | NA | NA | 1.52E-01 | 7 | -133.566 | 281.132 | 1.609 | 0.182 |
| -0.473 | NA | NA | + | NA | NA | 1.65E-01 | 6 | -147.591 | 307.181 | 0 | 0.399 |
| -0.569 | NA | NA | + | -4.33E-04 | NA | 1.73E-01 | 7 | -147.124 | 308.247 | 1.066 | 0.234 |
| -0.507 | 9.08E-10 | NA | + | NA | NA | 1.73E-01 | 7 | -147.336 | 308.673 | 1.492 | 0.189 |
| -0.384 | NA | NA | + | NA | -4.07E-02 | 1.67E-01 | 7 | -147.406 | 308.813 | 1.632 | 0.177 |
| -0.744 | NA | NA | + | -7.74E-04 | NA | 2.08E-01 | 7 | -150.486 | 314.972 | 0 | 0.309 |
| -0.598 | NA | NA | + | -8.46E-04 | -7.37E-02 | 2.12E-01 | 8 | -149.887 | 315.774 | 0.801 | 0.207 |
| -0.582 | NA | NA | + | NA | NA | 1.96E-01 | 6 | -151.989 | 315.978 | 1.005 | 0.187 |
| -0.797 | 1.64E-09 | NA | + | -8.00E-04 | NA | 2.16E-01 | 8 | -150.015 | 316.03 | 1.058 | 0.182 |
| -0.654 | 1.54E-09 | NA | + | -8.67E-04 | -7.04E-02 | 2.20E-01 | 9 | -149.467 | 316.933 | 1.961 | 0.116 |
| -0.623 | NA | NA | + | -7.84E-04 | -1.01E-01 | 2.44E-01 | 8 | -163.84 | 343.681 | 0 | 0.205 |
| -0.832 | NA | NA | + | -6.73E-04 | NA | 2.44E-01 | 7 | -165.003 | 344.006 | 0.325 | 0.175 |
| -0.692 | NA | NA | + | NA | NA | 2.31E-01 | 6 | -166.061 | 344.122 | 0.442 | 0.165 |
| -0.499 | NA | NA | + | NA | -8.38E-02 | 2.29E-01 | 7 | -165.257 | 344.514 | 0.833 | 0.135 |
| -0.69 | 1.83E-09 | NA | + | -7.98E-04 | -9.46E-02 | 2.52E-01 | 9 | -163.443 | 344.886 | 1.205 | 0.112 |
| -0.895 | 2.15E-09 | NA | + | -6.98E-04 | NA | 2.54E-01 | 8 | -164.455 | 344.91 | 1.229 | 0.111 |
| -0.745 | 1.99E-09 | NA | + | NA | NA | 2.40E-01 | 7 | -165.597 | 345.194 | 1.513 | 0.096 |
| -0.525 | NA | NA | + | NA | NA | 1.84E-01 | 6 | -159.368 | 330.736 | 0 | 0.383 |
| -0.625 | NA | NA | + | -4.73E-04 | NA | 1.93E-01 | 7 | -158.867 | 331.733 | 0.997 | 0.233 |
| -0.404 | NA | NA | + | NA | -5.45E-02 | 1.84E-01 | 7 | -159.046 | 332.092 | 1.356 | 0.194 |
| -0.563 | 1.37E-09 | NA | + | NA | NA | 1.91E-01 | 7 | -159.069 | 332.138 | 1.402 | 0.19 |
| -0.493 | NA | NA | + | NA | NA | 1.73E-01 | 6 | -162.492 | 336.985 | 0 | 0.436 |
| -0.529 | 1.30E-09 | NA | + | NA | NA | 1.78E-01 | 7 | -162.272 | 338.545 | 1.56 | 0.2 |
| -0.56 | NA | NA | + | -3.04E-04 | NA | 1.78E-01 | 7 | -162.298 | 338.595 | 1.611 | 0.195 |
| -0.444 | NA | NA | + | NA | -2.32E-02 | 1.74E-01 | 7 | -162.439 | 338.878 | 1.894 | 0.169 |
| -0.541 | NA | NA | + | -7.55E-04 | -9.31E-02 | 2.19E-01 | 8 | -149.637 | 315.274 | 0 | 0.201 |
| -0.727 | NA | NA | + | -6.59E-04 | NA | 2.17E-01 | 7 | -150.704 | 315.408 | 0.134 | 0.188 |
| -0.586 | NA | NA | + | NA | NA | 2.05E-01 | 6 | -151.828 | 315.657 | 0.382 | 0.166 |
| -0.415 | NA | NA | + | NA | -7.68E-02 | 2.05E-01 | 7 | -151.099 | 316.197 | 0.923 | 0.127 |
| -0.782 | 1.85E-09 | NA | + | -6.77E-04 | NA | 2.26E-01 | 8 | -150.205 | 316.41 | 1.135 | 0.114 |
| -0.599 | 1.62E-09 | NA | + | -7.66E-04 | -8.82E-02 | 2.27E-01 | 9 | -149.25 | 316.5 | 1.226 | 0.109 |
| -0.634 | 1.73E-09 | NA | + | NA | NA | 2.12E-01 | 7 | -151.397 | 316.794 | 1.519 | 0.094 |
| -0.562 | NA | NA | + | -8.37E-04 | -9.52E-02 | 2.21E-01 | 8 | -151.506 | 319.012 | 0 | 0.235 |
| -0.757 | NA | NA | + | -7.45E-04 | NA | 2.19E-01 | 7 | -152.595 | 319.189 | 0.177 | 0.215 |
| -0.632 | 1.58E-09 | NA | + | -8.81E-04 | -9.12E-02 | 2.31E-01 | 9 | -150.961 | 319.922 | 0.91 | 0.149 |
| -0.824 | 1.71E-09 | NA | + | -7.97E-04 | NA | 2.31E-01 | 8 | -151.964 | 319.928 | 0.916 | 0.149 |
| -0.598 | NA | NA | + | NA | NA | 2.05E-01 | 6 | -154.006 | 320.011 | 0.999 | 0.143 |
| -0.422 | NA | NA | + | NA | -7.83E-02 | 2.05E-01 | 7 | -153.271 | 320.543 | 1.53 | 0.109 |
| -0.752 | NA | NA | + | -6.48E-04 | NA | 2.15E-01 | 7 | -155.171 | 324.341 | 0 | 0.205 |
| -0.616 | NA | NA | + | NA | NA | 2.04E-01 | 6 | -156.185 | 324.369 | 0.028 | 0.202 |
| -0.584 | NA | NA | + | -7.25E-04 | -8.07E-02 | 2.17E-01 | 8 | -154.409 | 324.818 | 0.476 | 0.161 |
| -0.464 | NA | NA | + | NA | -6.64E-02 | 2.04E-01 | 7 | -155.666 | 325.332 | 0.991 | 0.125 |
| -0.805 | 1.74E-09 | NA | + | -6.73E-04 | NA | 2.23E-01 | 8 | -154.733 | 325.466 | 1.125 | 0.117 |
| -0.659 | 1.58E-09 | NA | + | NA | NA | 2.11E-01 | 7 | -155.83 | 325.66 | 1.319 | 0.106 |
| -0.641 | 1.59E-09 | NA | + | -7.45E-04 | -7.68E-02 | 2.24E-01 | 9 | -154.043 | 326.086 | 1.745 | 0.085 |
| -0.517 | NA | NA | + | NA | NA | 1.78E-01 | 6 | -160.597 | 333.194 | 0 | 0.439 |
| -0.553 | 1.15E-09 | NA | + | NA | NA | 1.85E-01 | 7 | -160.347 | 334.694 | 1.5 | 0.207 |
| -0.566 | NA | NA | + | -2.31E-04 | NA | 1.83E-01 | 7 | -160.483 | 334.966 | 1.772 | 0.181 |
| -0.462 | NA | NA | + | NA | -2.46E-02 | 1.79E-01 | 7 | -160.535 | 335.069 | 1.875 | 0.172 |
| -0.597 | NA | NA | + | NA | NA | 2.02E-01 | 6 | -162.85 | 337.7 | 0 | 0.331 |
| -0.712 | NA | NA | + | -5.48E-04 | NA | 2.12E-01 | 7 | -162.186 | 338.372 | 0.673 | 0.236 |
| -0.64 | 1.38E-09 | NA | + | NA | NA | 2.10E-01 | 7 | -162.535 | 339.071 | 1.371 | 0.167 |
| -0.521 | NA | NA | + | NA | -3.40E-02 | 2.02E-01 | 7 | -162.733 | 339.467 | 1.767 | 0.137 |
| -0.767 | 1.55E-09 | NA | + | -5.84E-04 | NA | 2.22E-01 | 8 | -161.785 | 339.569 | 1.869 | 0.13 |
| -0.442 | NA | NA | + | NA | NA | 1.58E-01 | 6 | -144.89 | 301.779 | 0 | 0.41 |
| -0.54 | NA | NA | + | -4.21E-04 | NA | 1.67E-01 | 7 | -144.449 | 302.899 | 1.12 | 0.234 |
| -0.47 | 1.01E-09 | NA | + | NA | NA | 1.62E-01 | 7 | -144.711 | 303.422 | 1.643 | 0.18 |
| -0.359 | NA | NA | + | NA | -3.81E-02 | 1.60E-01 | 7 | -144.733 | 303.465 | 1.686 | 0.176 |
| -0.417 | NA | NA | + | NA | NA | 1.52E-01 | 6 | -142.924 | 297.848 | 0 | 0.4 |
| -0.524 | NA | NA | + | -4.58E-04 | NA | 1.61E-01 | 7 | -142.402 | 298.804 | 0.956 | 0.248 |
| -0.446 | 1.02E-09 | NA | + | NA | NA | 1.57E-01 | 7 | -142.722 | 299.445 | 1.597 | 0.18 |
| -0.337 | NA | NA | + | NA | -3.68E-02 | 1.54E-01 | 7 | -142.775 | 299.549 | 1.702 | 0.171 |
| -0.425 | NA | NA | + | NA | NA | 1.60E-01 | 6 | -138.886 | 289.772 | 0 | 0.41 |
| -0.524 | NA | NA | + | -4.01E-04 | NA | 1.69E-01 | 7 | -138.457 | 290.915 | 1.143 | 0.232 |
| -0.454 | 8.82E-10 | NA | + | NA | NA | 1.65E-01 | 7 | -138.689 | 291.378 | 1.606 | 0.184 |
| -0.345 | NA | NA | + | NA | -3.57E-02 | 1.61E-01 | 7 | -138.742 | 291.483 | 1.712 | 0.174 |
| -0.475 | NA | NA | + | NA | NA | 1.68E-01 | 6 | -143.404 | 298.808 | 0 | 0.318 |
| -0.582 | NA | NA | + | -4.61E-04 | NA | 1.78E-01 | 7 | -142.85 | 299.7 | 0.892 | 0.204 |
| -0.336 | NA | NA | + | NA | -6.17E-02 | 1.69E-01 | 7 | -142.943 | 299.886 | 1.078 | 0.186 |
| -0.51 | 1.16E-09 | NA | + | NA | NA | 1.74E-01 | 7 | -143.148 | 300.295 | 1.487 | 0.151 |
| -0.434 | NA | NA | + | -5.33E-04 | -7.31E-02 | 1.81E-01 | 8 | -142.213 | 300.426 | 1.618 | 0.142 |
| -0.536 | NA | NA | + | NA | NA | 1.83E-01 | 6 | -153.081 | 318.162 | 0 | 0.244 |
| -0.679 | NA | NA | + | -6.47E-04 | NA | 1.94E-01 | 7 | -152.081 | 318.162 | 0 | 0.244 |
| -0.552 | NA | NA | + | -7.03E-04 | -6.39E-02 | 1.96E-01 | 8 | -151.641 | 319.281 | 1.12 | 0.14 |
| -0.729 | 1.44E-09 | NA | + | -6.75E-04 | NA | 2.02E-01 | 8 | -151.697 | 319.394 | 1.232 | 0.132 |
| -0.575 | 1.27E-09 | NA | + | NA | NA | 1.90E-01 | 7 | -152.785 | 319.571 | 1.409 | 0.121 |
| -0.426 | NA | NA | + | NA | -5.04E-02 | 1.85E-01 | 7 | -152.806 | 319.611 | 1.45 | 0.118 |
| -0.574 | NA | NA | + | -8.44E-04 | -1.22E-01 | 2.45E-01 | 8 | -160.179 | 336.357 | 0 | 0.409 |
| -0.642 | 1.84E-09 | NA | + | -8.54E-04 | -1.15E-01 | 2.54E-01 | 9 | -159.727 | 337.454 | 1.097 | 0.236 |
| -0.452 | NA | NA | + | NA | -1.03E-01 | 2.30E-01 | 7 | -161.929 | 337.858 | 1.501 | 0.193 |
| -0.825 | NA | NA | + | -6.98E-04 | NA | 2.47E-01 | 7 | -162.103 | 338.206 | 1.848 | 0.162 |
| -0.462 | NA | NA | + | NA | NA | 1.66E-01 | 6 | -156.354 | 324.707 | 0 | 0.441 |
| -0.496 | 1.15E-09 | NA | + | NA | NA | 1.71E-01 | 7 | -156.149 | 326.298 | 1.591 | 0.199 |
| -0.385 | NA | NA | + | NA | -3.47E-02 | 1.66E-01 | 7 | -156.232 | 326.463 | 1.756 | 0.183 |
| -0.509 | NA | NA | + | -2.01E-04 | NA | 1.71E-01 | 7 | -156.264 | 326.527 | 1.82 | 0.177 |
| -0.453 | NA | NA | + | NA | NA | 1.63E-01 | 6 | -148.621 | 309.241 | 0 | 0.426 |
| -0.535 | NA | NA | + | -3.54E-04 | NA | 1.70E-01 | 7 | -148.318 | 310.636 | 1.395 | 0.212 |
| -0.485 | 1.09E-09 | NA | + | NA | NA | 1.68E-01 | 7 | -148.412 | 310.824 | 1.583 | 0.193 |
| -0.397 | NA | NA | + | NA | -2.62E-02 | 1.64E-01 | 7 | -148.547 | 311.094 | 1.853 | 0.169 |
| -0.43 | NA | NA | + | NA | NA | 1.64E-01 | 6 | -147.187 | 306.373 | 0 | 0.424 |
| -0.519 | NA | NA | + | -3.82E-04 | NA | 1.72E-01 | 7 | -146.841 | 307.682 | 1.309 | 0.22 |
| -0.459 | 1.14E-09 | NA | + | NA | NA | 1.68E-01 | 7 | -147.004 | 308.009 | 1.636 | 0.187 |
| -0.372 | NA | NA | + | NA | -2.65E-02 | 1.64E-01 | 7 | -147.111 | 308.222 | 1.849 | 0.168 |
| -0.56 | NA | NA | + | NA | NA | 2.05E-01 | 6 | -155.881 | 323.762 | 0 | 0.324 |
| -0.415 | NA | NA | + | NA | -6.50E-02 | 2.05E-01 | 7 | -155.396 | 324.792 | 1.03 | 0.194 |
| -0.654 | NA | NA | + | -4.30E-04 | NA | 2.14E-01 | 7 | -155.444 | 324.889 | 1.127 | 0.185 |
| -0.602 | 1.54E-09 | NA | + | NA | NA | 2.11E-01 | 7 | -155.542 | 325.083 | 1.321 | 0.167 |
| -0.503 | NA | NA | + | -5.09E-04 | -7.58E-02 | 2.16E-01 | 8 | -154.794 | 325.588 | 1.826 | 0.13 |
| -0.484 | NA | NA | + | NA | NA | 1.73E-01 | 6 | -161.706 | 335.412 | 0 | 0.447 |
| -0.516 | 1.04E-09 | NA | + | NA | NA | 1.79E-01 | 7 | -161.521 | 337.042 | 1.629 | 0.198 |
| -0.423 | NA | NA | + | NA | -2.82E-02 | 1.74E-01 | 7 | -161.628 | 337.256 | 1.844 | 0.178 |
| -0.526 | NA | NA | + | -1.80E-04 | NA | 1.77E-01 | 7 | -161.637 | 337.273 | 1.861 | 0.176 |
| -0.413 | NA | NA | + | NA | NA | 1.56E-01 | 6 | -168.381 | 348.762 | 0 | 0.276 |
| -0.348 | NA | NA | NA | NA | NA | 1.94E-01 | 3 | -171.681 | 349.363 | 0.6 | 0.204 |
| -0.24 | NA | NA | NA | 5.73E-04 | NA | 1.66E-01 | 4 | -170.754 | 349.508 | 0.745 | 0.19 |
| -0.441 | 8.73E-10 | NA | + | NA | NA | 1.62E-01 | 7 | -168.24 | 350.48 | 1.718 | 0.117 |
| -0.372 | NA | NA | + | 1.76E-04 | NA | 1.52E-01 | 7 | -168.322 | 350.644 | 1.881 | 0.108 |
| -0.459 | NA | NA | + | NA | 2.17E-02 | 1.55E-01 | 7 | -168.343 | 350.685 | 1.923 | 0.105 |
| -0.376 | NA | NA | + | NA | NA | 1.47E-01 | 6 | -148.497 | 308.995 | 0 | 0.388 |
| -0.404 | 1.06E-09 | NA | + | NA | NA | 1.52E-01 | 7 | -148.32 | 310.64 | 1.645 | 0.17 |
| -0.408 | NA | NA | + | -1.33E-04 | NA | 1.50E-01 | 7 | -148.456 | 310.913 | 1.918 | 0.149 |
| -0.306 | NA | NA | NA | NA | NA | 1.86E-01 | 3 | -152.464 | 310.928 | 1.934 | 0.147 |
| -0.341 | NA | NA | + | NA | -1.58E-02 | 1.48E-01 | 7 | -148.473 | 310.946 | 1.951 | 0.146 |
| -0.772 | NA | NA | + | -7.01E-04 | NA | 2.27E-01 | 7 | -157.023 | 328.047 | 0 | 0.225 |
| -0.625 | NA | NA | + | NA | NA | 2.14E-01 | 6 | -158.194 | 328.388 | 0.341 | 0.19 |
| -0.614 | NA | NA | + | -7.81E-04 | -7.86E-02 | 2.28E-01 | 8 | -156.326 | 328.652 | 0.605 | 0.166 |
| -0.824 | 1.97E-09 | NA | + | -7.08E-04 | NA | 2.34E-01 | 8 | -156.608 | 329.215 | 1.168 | 0.125 |
| -0.487 | NA | NA | + | NA | -6.21E-02 | 2.14E-01 | 7 | -157.756 | 329.512 | 1.465 | 0.108 |
| -0.675 | 1.92E-09 | NA | + | NA | NA | 2.21E-01 | 7 | -157.806 | 329.611 | 1.564 | 0.103 |
| -0.671 | 1.70E-09 | NA | + | -7.80E-04 | -7.26E-02 | 2.35E-01 | 9 | -156.017 | 330.035 | 1.988 | 0.083 |
| -0.588 | NA | NA | + | NA | NA | 2.00E-01 | 6 | -169.134 | 350.269 | 0 | 0.419 |
| -0.633 | 1.63E-09 | NA | + | NA | NA | 2.07E-01 | 7 | -168.808 | 351.615 | 1.346 | 0.214 |
| -0.665 | NA | NA | + | -3.70E-04 | NA | 2.06E-01 | 7 | -168.856 | 351.711 | 1.442 | 0.204 |
| -0.533 | NA | NA | + | NA | -2.54E-02 | 2.00E-01 | 7 | -169.075 | 352.151 | 1.882 | 0.164 |
| -0.47 | NA | NA | + | NA | NA | 1.70E-01 | 6 | -146.154 | 304.308 | 0 | 0.402 |
| -0.558 | NA | NA | + | -4.15E-04 | NA | 1.78E-01 | 7 | -145.73 | 305.461 | 1.153 | 0.226 |
| -0.507 | 1.25E-09 | NA | + | NA | NA | 1.76E-01 | 7 | -145.87 | 305.74 | 1.432 | 0.197 |
| -0.385 | NA | NA | + | NA | -3.82E-02 | 1.71E-01 | 7 | -145.985 | 305.971 | 1.663 | 0.175 |
| -0.581 | NA | NA | + | NA | NA | 1.95E-01 | 6 | -159.357 | 330.713 | 0 | 0.29 |
| -0.699 | NA | NA | + | -5.50E-04 | NA | 2.05E-01 | 7 | -158.639 | 331.279 | 0.566 | 0.218 |
| -0.618 | 1.21E-09 | NA | + | NA | NA | 2.02E-01 | 7 | -159.078 | 332.157 | 1.444 | 0.141 |
| -0.489 | NA | NA | + | NA | -4.02E-02 | 1.95E-01 | 7 | -159.186 | 332.372 | 1.659 | 0.126 |
| -0.75 | 1.40E-09 | NA | + | -5.87E-04 | NA | 2.14E-01 | 8 | -158.265 | 332.529 | 1.816 | 0.117 |
| -0.587 | NA | NA | + | -6.02E-04 | -5.35E-02 | 2.06E-01 | 8 | -158.341 | 332.683 | 1.97 | 0.108 |
| -0.489 | NA | NA | + | NA | NA | 1.74E-01 | 6 | -149.103 | 310.206 | 0 | 0.392 |
| -0.582 | NA | NA | + | -4.35E-04 | NA | 1.81E-01 | 7 | -148.618 | 311.237 | 1.031 | 0.234 |
| -0.385 | NA | NA | + | NA | -4.84E-02 | 1.77E-01 | 7 | -148.831 | 311.661 | 1.455 | 0.189 |
| -0.52 | 9.98E-10 | NA | + | NA | NA | 1.80E-01 | 7 | -148.856 | 311.712 | 1.506 | 0.185 |
| -0.633 | NA | NA | + | NA | NA | 2.05E-01 | 6 | -161.118 | 334.237 | 0 | 0.265 |
| -0.733 | NA | NA | + | -5.26E-04 | NA | 2.13E-01 | 7 | -160.466 | 334.932 | 0.695 | 0.187 |
| -0.478 | NA | NA | + | NA | -6.70E-02 | 2.04E-01 | 7 | -160.624 | 335.248 | 1.011 | 0.16 |
| -0.678 | 1.50E-09 | NA | + | NA | NA | 2.13E-01 | 7 | -160.751 | 335.501 | 1.264 | 0.141 |
| -0.563 | NA | NA | + | -6.07E-04 | -8.01E-02 | 2.13E-01 | 8 | -159.767 | 335.535 | 1.298 | 0.139 |
| -0.787 | 1.64E-09 | NA | + | -5.55E-04 | NA | 2.23E-01 | 8 | -160.023 | 336.045 | 1.809 | 0.107 |
| -0.495 | NA | NA | + | NA | NA | 1.75E-01 | 6 | -157.792 | 327.585 | 0 | 0.428 |
| -0.535 | 1.43E-09 | NA | + | NA | NA | 1.81E-01 | 7 | -157.506 | 329.013 | 1.428 | 0.21 |
| -0.409 | NA | NA | + | NA | -3.78E-02 | 1.75E-01 | 7 | -157.646 | 329.292 | 1.707 | 0.182 |
| -0.552 | NA | NA | + | -2.45E-04 | NA | 1.80E-01 | 7 | -157.663 | 329.325 | 1.741 | 0.179 |
| -0.429 | NA | NA | + | NA | NA | 1.62E-01 | 6 | -158.281 | 328.562 | 0 | 0.38 |
| -0.367 | NA | NA | NA | NA | NA | 1.99E-01 | 3 | -162.072 | 330.144 | 1.582 | 0.172 |
| -0.459 | 1.10E-09 | NA | + | NA | NA | 1.67E-01 | 7 | -158.136 | 330.272 | 1.71 | 0.162 |
| -0.458 | NA | NA | + | -1.29E-04 | NA | 1.65E-01 | 7 | -158.246 | 330.492 | 1.93 | 0.145 |
| -0.454 | NA | NA | + | NA | 1.17E-02 | 1.62E-01 | 7 | -158.269 | 330.537 | 1.976 | 0.141 |
| -0.576 | NA | NA | + | NA | NA | 1.96E-01 | 6 | -150.586 | 313.172 | 0 | 0.244 |
| -0.696 | NA | NA | + | -5.57E-04 | NA | 2.06E-01 | 7 | -149.811 | 313.622 | 0.45 | 0.195 |
| -0.532 | NA | NA | + | -6.41E-04 | -8.22E-02 | 2.11E-01 | 8 | -148.997 | 313.995 | 0.823 | 0.162 |
| -0.424 | NA | NA | + | NA | -6.88E-02 | 1.99E-01 | 7 | -150.01 | 314.021 | 0.849 | 0.16 |
| -0.617 | 1.30E-09 | NA | + | NA | NA | 2.04E-01 | 7 | -150.239 | 314.477 | 1.305 | 0.127 |
| -0.75 | 1.49E-09 | NA | + | -5.95E-04 | NA | 2.15E-01 | 8 | -149.356 | 314.713 | 1.541 | 0.113 |
| -0.666 | NA | NA | + | -9.94E-04 | -1.43E-01 | 2.76E-01 | 8 | -158.862 | 333.723 | 0 | 0.612 |
| -0.738 | 1.96E-09 | NA | + | -1.01E-03 | -1.36E-01 | 2.86E-01 | 9 | -158.319 | 334.638 | 0.914 | 0.388 |
| -0.594 | NA | NA | + | NA | NA | 2.01E-01 | 6 | -160.356 | 332.713 | 0 | 0.244 |
| -0.721 | NA | NA | + | -5.94E-04 | NA | 2.13E-01 | 7 | -159.513 | 333.026 | 0.314 | 0.208 |
| -0.552 | NA | NA | + | -6.61E-04 | -8.03E-02 | 2.14E-01 | 8 | -158.804 | 333.609 | 0.896 | 0.156 |
| -0.438 | NA | NA | + | NA | -6.84E-02 | 2.00E-01 | 7 | -159.84 | 333.681 | 0.968 | 0.15 |
| -0.636 | 1.57E-09 | NA | + | NA | NA | 2.08E-01 | 7 | -160.013 | 334.027 | 1.314 | 0.126 |
| -0.772 | 1.71E-09 | NA | + | -6.18E-04 | NA | 2.21E-01 | 8 | -159.1 | 334.2 | 1.487 | 0.116 |
| -0.469 | NA | NA | + | NA | NA | 1.70E-01 | 6 | -156.527 | 325.054 | 0 | 0.442 |
| -0.503 | 9.77E-10 | NA | + | NA | NA | 1.77E-01 | 7 | -156.281 | 326.562 | 1.508 | 0.208 |
| -0.515 | NA | NA | + | -2.19E-04 | NA | 1.74E-01 | 7 | -156.419 | 326.838 | 1.784 | 0.181 |
| -0.426 | NA | NA | + | NA | -2.02E-02 | 1.71E-01 | 7 | -156.486 | 326.971 | 1.917 | 0.169 |
| -0.42 | NA | NA | + | NA | NA | 1.57E-01 | 6 | -164.024 | 340.048 | 0 | 0.333 |
| -0.345 | NA | NA | NA | NA | NA | 1.98E-01 | 3 | -167.823 | 341.645 | 1.597 | 0.15 |
| -0.448 | 9.87E-10 | NA | + | NA | NA | 1.62E-01 | 7 | -163.875 | 341.751 | 1.703 | 0.142 |
| -0.247 | NA | NA | NA | 5.23E-04 | NA | 1.73E-01 | 4 | -166.973 | 341.947 | 1.899 | 0.129 |
| -0.451 | NA | NA | + | NA | 1.38E-02 | 1.57E-01 | 7 | -164.007 | 342.015 | 1.967 | 0.124 |
| -0.413 | NA | NA | + | 3.09E-05 | NA | 1.57E-01 | 7 | -164.022 | 342.044 | 1.996 | 0.123 |
| -0.484 | NA | NA | + | NA | NA | 1.68E-01 | 6 | -148.369 | 308.738 | 0 | 0.389 |
| -0.591 | NA | NA | + | -5.17E-04 | NA | 1.76E-01 | 7 | -147.73 | 309.461 | 0.723 | 0.271 |
| -0.518 | 1.19E-09 | NA | + | NA | NA | 1.74E-01 | 7 | -148.111 | 310.223 | 1.485 | 0.185 |
| -0.426 | NA | NA | + | NA | -2.60E-02 | 1.69E-01 | 7 | -148.296 | 310.593 | 1.855 | 0.154 |
| -0.35 | NA | NA | + | NA | NA | 1.41E-01 | 6 | -149.922 | 311.844 | 0 | 0.235 |
| -0.309 | NA | NA | NA | NA | NA | 1.77E-01 | 3 | -153 | 311.999 | 0.155 | 0.217 |
| -0.213 | NA | NA | NA | 4.92E-04 | NA | 1.53E-01 | 4 | -152.188 | 312.375 | 0.531 | 0.18 |
| -0.377 | 8.74E-10 | NA | + | NA | NA | 1.46E-01 | 7 | -149.779 | 313.558 | 1.714 | 0.1 |
| -0.338 | 9.43E-10 | NA | NA | NA | NA | 1.82E-01 | 4 | -152.839 | 313.678 | 1.834 | 0.094 |
| -0.331 | NA | NA | + | 8.00E-05 | NA | 1.39E-01 | 7 | -149.908 | 313.817 | 1.973 | 0.088 |
| -0.362 | NA | NA | + | NA | 5.65E-03 | 1.40E-01 | 7 | -149.919 | 313.838 | 1.994 | 0.087 |
| -0.626 | NA | NA | + | NA | NA | 2.06E-01 | 6 | -163.305 | 338.61 | 0 | 0.324 |
| -0.476 | NA | NA | + | NA | -6.70E-02 | 2.07E-01 | 7 | -162.821 | 339.642 | 1.032 | 0.193 |
| -0.715 | NA | NA | + | -4.47E-04 | NA | 2.14E-01 | 7 | -162.85 | 339.7 | 1.09 | 0.188 |
| -0.666 | 1.30E-09 | NA | + | NA | NA | 2.14E-01 | 7 | -162.978 | 339.955 | 1.345 | 0.165 |
| -0.558 | NA | NA | + | -5.13E-04 | -7.65E-02 | 2.17E-01 | 8 | -162.227 | 340.453 | 1.843 | 0.129 |
| -0.427 | NA | NA | + | NA | NA | 1.64E-01 | 6 | -143.25 | 298.5 | 0 | 0.445 |
| -0.455 | 1.01E-09 | NA | + | NA | NA | 1.68E-01 | 7 | -143.062 | 300.124 | 1.624 | 0.198 |
| -0.36 | NA | NA | + | NA | -3.14E-02 | 1.66E-01 | 7 | -143.141 | 300.283 | 1.783 | 0.183 |
| -0.463 | NA | NA | + | -1.60E-04 | NA | 1.67E-01 | 7 | -143.186 | 300.372 | 1.872 | 0.175 |
| -0.726 | NA | NA | + | -6.52E-04 | NA | 2.12E-01 | 7 | -152.522 | 319.043 | 0 | 0.196 |
| -0.588 | NA | NA | + | NA | NA | 2.00E-01 | 6 | -153.585 | 319.171 | 0.128 | 0.184 |
| -0.546 | NA | NA | + | -7.46E-04 | -8.98E-02 | 2.15E-01 | 8 | -151.592 | 319.185 | 0.142 | 0.182 |
| -0.426 | NA | NA | + | NA | -7.30E-02 | 2.01E-01 | 7 | -152.966 | 319.933 | 0.89 | 0.126 |
| -0.776 | 1.93E-09 | NA | + | -6.61E-04 | NA | 2.19E-01 | 8 | -152.065 | 320.129 | 1.086 | 0.114 |
| -0.636 | 1.86E-09 | NA | + | NA | NA | 2.06E-01 | 7 | -153.165 | 320.329 | 1.286 | 0.103 |
| -0.6 | 1.69E-09 | NA | + | -7.49E-04 | -8.47E-02 | 2.21E-01 | 9 | -151.24 | 320.48 | 1.437 | 0.095 |
| -0.379 | NA | NA | + | NA | NA | 1.49E-01 | 6 | -157.038 | 326.076 | 0 | 0.276 |
| -0.332 | NA | NA | NA | NA | NA | 1.88E-01 | 3 | -160.272 | 326.545 | 0.469 | 0.219 |
| -0.234 | NA | NA | NA | 4.86E-04 | NA | 1.62E-01 | 4 | -159.473 | 326.946 | 0.87 | 0.179 |
| -0.406 | 9.34E-10 | NA | + | NA | NA | 1.53E-01 | 7 | -156.895 | 327.791 | 1.715 | 0.117 |
| -0.432 | NA | NA | + | NA | 2.46E-02 | 1.48E-01 | 7 | -156.983 | 327.965 | 1.89 | 0.107 |
| -0.371 | NA | NA | + | 3.53E-05 | NA | 1.48E-01 | 7 | -157.035 | 328.071 | 1.995 | 0.102 |
| -0.563 | NA | NA | + | NA | NA | 1.97E-01 | 6 | -162.724 | 337.449 | 0 | 0.41 |
| -0.649 | NA | NA | + | -4.00E-04 | NA | 2.05E-01 | 7 | -162.364 | 338.729 | 1.28 | 0.216 |
| -0.602 | 1.41E-09 | NA | + | NA | NA | 2.04E-01 | 7 | -162.449 | 338.897 | 1.448 | 0.199 |
| -0.479 | NA | NA | + | NA | -3.73E-02 | 1.97E-01 | 7 | -162.58 | 339.159 | 1.711 | 0.174 |
| -0.407 | NA | NA | + | NA | NA | 1.48E-01 | 6 | -155.935 | 323.87 | 0 | 0.437 |
| -0.485 | NA | NA | + | -3.26E-04 | NA | 1.55E-01 | 7 | -155.705 | 325.41 | 1.54 | 0.203 |
| -0.435 | 1.02E-09 | NA | + | NA | NA | 1.53E-01 | 7 | -155.785 | 325.569 | 1.699 | 0.187 |
| -0.351 | NA | NA | + | NA | -2.69E-02 | 1.50E-01 | 7 | -155.862 | 325.724 | 1.854 | 0.173 |
| -0.521 | NA | NA | + | NA | NA | 1.83E-01 | 6 | -159.174 | 330.348 | 0 | 0.408 |
| -0.619 | NA | NA | + | -4.58E-04 | NA | 1.90E-01 | 7 | -158.712 | 331.425 | 1.077 | 0.238 |
| -0.559 | 1.37E-09 | NA | + | NA | NA | 1.88E-01 | 7 | -158.933 | 331.865 | 1.517 | 0.191 |
| -0.459 | NA | NA | + | NA | -2.87E-02 | 1.84E-01 | 7 | -159.092 | 332.185 | 1.837 | 0.163 |
| -0.507 | NA | NA | + | NA | NA | 1.76E-01 | 6 | -158.364 | 328.728 | 0 | 0.441 |
| -0.543 | 1.38E-09 | NA | + | NA | NA | 1.81E-01 | 7 | -158.121 | 330.242 | 1.513 | 0.207 |
| -0.554 | NA | NA | + | -2.26E-04 | NA | 1.80E-01 | 7 | -158.253 | 330.506 | 1.778 | 0.181 |
| -0.46 | NA | NA | + | NA | -2.17E-02 | 1.77E-01 | 7 | -158.317 | 330.633 | 1.905 | 0.17 |
| -0.428 | NA | NA | + | NA | NA | 1.64E-01 | 6 | -141.135 | 294.269 | 0 | 0.394 |
| -0.309 | NA | NA | + | NA | -5.46E-02 | 1.65E-01 | 7 | -140.771 | 295.541 | 1.272 | 0.208 |
| -0.511 | NA | NA | + | -3.65E-04 | NA | 1.71E-01 | 7 | -140.791 | 295.583 | 1.314 | 0.204 |
| -0.461 | 1.05E-09 | NA | + | NA | NA | 1.70E-01 | 7 | -140.845 | 295.689 | 1.42 | 0.194 |
| -0.366 | NA | NA | + | NA | NA | 1.49E-01 | 6 | -151.162 | 314.324 | 0 | 0.234 |
| -0.309 | NA | NA | NA | NA | NA | 1.82E-01 | 3 | -154.296 | 314.592 | 0.268 | 0.205 |
| -0.211 | NA | NA | NA | 5.18E-04 | NA | 1.57E-01 | 4 | -153.333 | 314.665 | 0.342 | 0.197 |
| -0.393 | 7.85E-10 | NA | + | NA | NA | 1.54E-01 | 7 | -151.01 | 316.019 | 1.696 | 0.1 |
| -0.338 | 8.59E-10 | NA | NA | NA | NA | 1.88E-01 | 4 | -154.121 | 316.241 | 1.918 | 0.09 |
| -0.342 | NA | NA | + | 1.04E-04 | NA | 1.47E-01 | 7 | -151.139 | 316.277 | 1.953 | 0.088 |
| -0.377 | NA | NA | + | NA | 5.09E-03 | 1.49E-01 | 7 | -151.159 | 316.319 | 1.995 | 0.086 |
| -0.518 | NA | NA | + | NA | NA | 1.79E-01 | 6 | -158.524 | 329.048 | 0 | 0.404 |
| -0.615 | NA | NA | + | -4.55E-04 | NA | 1.86E-01 | 7 | -158.058 | 330.117 | 1.069 | 0.237 |
| -0.554 | 1.31E-09 | NA | + | NA | NA | 1.85E-01 | 7 | -158.26 | 330.52 | 1.472 | 0.193 |
| -0.446 | NA | NA | + | NA | -3.31E-02 | 1.81E-01 | 7 | -158.41 | 330.819 | 1.771 | 0.166 |
| -0.415 | NA | NA | + | NA | NA | 1.63E-01 | 6 | -146.633 | 305.267 | 0 | 0.441 |
| -0.443 | 8.77E-10 | NA | + | NA | NA | 1.69E-01 | 7 | -146.424 | 306.848 | 1.581 | 0.2 |
| -0.467 | NA | NA | + | -2.41E-04 | NA | 1.67E-01 | 7 | -146.492 | 306.984 | 1.718 | 0.187 |
| -0.37 | NA | NA | + | NA | -2.21E-02 | 1.65E-01 | 7 | -146.58 | 307.159 | 1.893 | 0.171 |
| -0.476 | NA | NA | + | NA | NA | 1.75E-01 | 6 | -156.116 | 324.232 | 0 | 0.441 |
| -0.508 | 1.22E-09 | NA | + | NA | NA | 1.80E-01 | 7 | -155.898 | 325.797 | 1.565 | 0.202 |
| -0.533 | NA | NA | + | -2.47E-04 | NA | 1.81E-01 | 7 | -155.981 | 325.963 | 1.731 | 0.185 |
| -0.421 | NA | NA | + | NA | -2.44E-02 | 1.74E-01 | 7 | -156.056 | 326.112 | 1.88 | 0.172 |
| -0.552 | NA | NA | + | NA | NA | 1.91E-01 | 6 | -157.599 | 327.198 | 0 | 0.258 |
| -0.668 | NA | NA | + | -5.68E-04 | NA | 1.99E-01 | 7 | -156.837 | 327.675 | 0.477 | 0.203 |
| -0.414 | NA | NA | + | NA | -6.29E-02 | 1.92E-01 | 7 | -157.146 | 328.292 | 1.094 | 0.149 |
| -0.518 | NA | NA | + | -6.31E-04 | -7.40E-02 | 2.02E-01 | 8 | -156.212 | 328.424 | 1.226 | 0.14 |
| -0.591 | 1.14E-09 | NA | + | NA | NA | 2.00E-01 | 7 | -157.282 | 328.564 | 1.367 | 0.13 |
| -0.728 | 1.40E-09 | NA | + | -6.28E-04 | NA | 2.11E-01 | 8 | -156.362 | 328.724 | 1.527 | 0.12 |
| -0.401 | NA | NA | + | NA | NA | 1.55E-01 | 6 | -161.985 | 335.97 | 0 | 0.299 |
| -0.338 | NA | NA | NA | NA | NA | 1.94E-01 | 3 | -165.358 | 336.717 | 0.747 | 0.206 |
| -0.254 | NA | NA | NA | 4.40E-04 | NA | 1.72E-01 | 4 | -164.746 | 337.491 | 1.522 | 0.14 |
| -0.429 | 1.04E-09 | NA | + | NA | NA | 1.59E-01 | 7 | -161.835 | 337.669 | 1.7 | 0.128 |
| -0.454 | NA | NA | + | NA | 2.45E-02 | 1.53E-01 | 7 | -161.934 | 337.868 | 1.898 | 0.116 |
| -0.413 | NA | NA | + | -4.91E-05 | NA | 1.56E-01 | 7 | -161.98 | 337.96 | 1.99 | 0.111 |
| -0.488 | NA | NA | + | NA | NA | 1.77E-01 | 6 | -144.055 | 300.109 | 0 | 0.27 |
| -0.602 | NA | NA | + | -5.02E-04 | NA | 1.87E-01 | 7 | -143.382 | 300.764 | 0.655 | 0.195 |
| -0.353 | NA | NA | + | NA | -6.25E-02 | 1.79E-01 | 7 | -143.575 | 301.15 | 1.041 | 0.16 |
| -0.457 | NA | NA | + | -5.69E-04 | -7.38E-02 | 1.91E-01 | 8 | -142.719 | 301.438 | 1.329 | 0.139 |
| -0.523 | 1.30E-09 | NA | + | NA | NA | 1.82E-01 | 7 | -143.761 | 301.522 | 1.413 | 0.133 |
| -0.646 | 1.43E-09 | NA | + | -5.26E-04 | NA | 1.94E-01 | 8 | -143.023 | 302.045 | 1.936 | 0.103 |
| -0.747 | NA | NA | + | -6.48E-04 | NA | 2.16E-01 | 7 | -152.267 | 318.533 | 0 | 0.198 |
| -0.611 | NA | NA | + | NA | NA | 2.06E-01 | 6 | -153.358 | 318.715 | 0.182 | 0.181 |
| -0.571 | NA | NA | + | -7.51E-04 | -8.65E-02 | 2.19E-01 | 8 | -151.366 | 318.732 | 0.198 | 0.18 |
| -0.804 | 1.59E-09 | NA | + | -6.94E-04 | NA | 2.26E-01 | 8 | -151.745 | 319.49 | 0.957 | 0.123 |
| -0.458 | NA | NA | + | NA | -6.76E-02 | 2.07E-01 | 7 | -152.8 | 319.6 | 1.067 | 0.116 |
| -0.631 | 1.49E-09 | NA | + | -7.90E-04 | -8.33E-02 | 2.28E-01 | 9 | -150.906 | 319.813 | 1.279 | 0.105 |
| -0.651 | 1.34E-09 | NA | + | NA | NA | 2.14E-01 | 7 | -152.988 | 319.975 | 1.442 | 0.097 |
| -0.405 | NA | NA | + | NA | NA | 1.57E-01 | 6 | -155.805 | 323.61 | 0 | 0.314 |
| -0.351 | NA | NA | NA | NA | NA | 1.98E-01 | 3 | -159.321 | 324.642 | 1.031 | 0.188 |
| -0.264 | NA | NA | NA | 4.42E-04 | NA | 1.74E-01 | 4 | -158.66 | 325.32 | 1.71 | 0.134 |
| -0.432 | 9.93E-10 | NA | + | NA | NA | 1.60E-01 | 7 | -155.68 | 325.36 | 1.749 | 0.131 |
| -0.43 | NA | NA | + | NA | 1.15E-02 | 1.56E-01 | 7 | -155.793 | 325.586 | 1.975 | 0.117 |
| -0.42 | NA | NA | + | -6.29E-05 | NA | 1.58E-01 | 7 | -155.797 | 325.594 | 1.983 | 0.117 |
| -0.442 | NA | NA | + | NA | NA | 1.63E-01 | 6 | -141.952 | 295.903 | 0 | 0.409 |
| -0.527 | NA | NA | + | -3.64E-04 | NA | 1.71E-01 | 7 | -141.618 | 297.236 | 1.332 | 0.21 |
| -0.342 | NA | NA | + | NA | -4.66E-02 | 1.66E-01 | 7 | -141.688 | 297.376 | 1.472 | 0.196 |
| -0.473 | 1.18E-09 | NA | + | NA | NA | 1.68E-01 | 7 | -141.749 | 297.497 | 1.594 | 0.184 |
| -0.529 | NA | NA | + | NA | NA | 1.85E-01 | 6 | -153.097 | 318.194 | 0 | 0.285 |
| -0.647 | NA | NA | + | -5.31E-04 | NA | 1.95E-01 | 7 | -152.427 | 318.854 | 0.659 | 0.205 |
| -0.569 | 1.43E-09 | NA | + | NA | NA | 1.91E-01 | 7 | -152.794 | 319.587 | 1.393 | 0.142 |
| -0.415 | NA | NA | + | NA | -5.21E-02 | 1.87E-01 | 7 | -152.801 | 319.602 | 1.408 | 0.141 |
| -0.518 | NA | NA | + | -6.05E-04 | -6.68E-02 | 1.99E-01 | 8 | -151.95 | 319.899 | 1.705 | 0.121 |
| -0.694 | 1.54E-09 | NA | + | -5.49E-04 | NA | 2.02E-01 | 8 | -152.075 | 320.15 | 1.956 | 0.107 |
| -0.387 | NA | NA | + | NA | NA | 1.48E-01 | 6 | -142.536 | 297.072 | 0 | 0.436 |
| -0.456 | NA | NA | + | -3.07E-04 | NA | 1.54E-01 | 7 | -142.307 | 298.613 | 1.541 | 0.202 |
| -0.417 | 1.06E-09 | NA | + | NA | NA | 1.53E-01 | 7 | -142.348 | 298.696 | 1.624 | 0.193 |
| -0.341 | NA | NA | + | NA | -2.21E-02 | 1.50E-01 | 7 | -142.482 | 298.964 | 1.892 | 0.169 |
| -0.409 | NA | NA | + | NA | NA | 1.54E-01 | 6 | -144.307 | 300.614 | 0 | 0.43 |
| -0.486 | NA | NA | + | -3.19E-04 | NA | 1.61E-01 | 7 | -144.056 | 302.112 | 1.498 | 0.203 |
| -0.441 | 1.14E-09 | NA | + | NA | NA | 1.59E-01 | 7 | -144.099 | 302.199 | 1.584 | 0.195 |
| -0.349 | NA | NA | + | NA | -2.76E-02 | 1.55E-01 | 7 | -144.225 | 302.45 | 1.836 | 0.172 |
| -0.762 | NA | NA | + | -8.11E-04 | NA | 2.15E-01 | 7 | -151.818 | 317.637 | 0 | 0.28 |
| -0.589 | NA | NA | + | -9.00E-04 | -8.65E-02 | 2.17E-01 | 8 | -150.929 | 317.858 | 0.221 | 0.251 |
| -0.819 | 1.78E-09 | NA | + | -8.40E-04 | NA | 2.24E-01 | 8 | -151.261 | 318.522 | 0.885 | 0.18 |
| -0.649 | 1.63E-09 | NA | + | -9.22E-04 | -8.22E-02 | 2.25E-01 | 9 | -150.457 | 318.914 | 1.278 | 0.148 |
| -0.613 | NA | NA | + | NA | NA | 2.05E-01 | 6 | -153.503 | 319.006 | 1.369 | 0.141 |
| -0.648 | NA | NA | + | -9.30E-04 | -1.00E-01 | 2.40E-01 | 8 | -156.85 | 329.701 | 0 | 0.299 |
| -0.857 | NA | NA | + | -8.19E-04 | NA | 2.40E-01 | 7 | -158.036 | 330.073 | 0.372 | 0.248 |
| -0.712 | 1.72E-09 | NA | + | -9.51E-04 | -9.51E-02 | 2.48E-01 | 9 | -156.42 | 330.841 | 1.14 | 0.169 |
| -0.918 | 1.95E-09 | NA | + | -8.49E-04 | NA | 2.49E-01 | 8 | -157.486 | 330.972 | 1.271 | 0.158 |
| -0.686 | NA | NA | + | NA | NA | 2.29E-01 | 6 | -159.722 | 331.445 | 1.744 | 0.125 |
| -0.534 | NA | NA | + | NA | NA | 1.83E-01 | 6 | -162.349 | 336.698 | 0 | 0.41 |
| -0.635 | NA | NA | + | -4.54E-04 | NA | 1.92E-01 | 7 | -161.895 | 337.79 | 1.092 | 0.237 |
| -0.568 | 1.27E-09 | NA | + | NA | NA | 1.88E-01 | 7 | -162.137 | 338.274 | 1.576 | 0.186 |
| -0.465 | NA | NA | + | NA | -3.12E-02 | 1.83E-01 | 7 | -162.253 | 338.506 | 1.808 | 0.166 |
| -0.837 | NA | NA | + | -7.80E-04 | NA | 2.30E-01 | 7 | -164.258 | 342.516 | 0 | 0.211 |
| -0.645 | NA | NA | + | -8.80E-04 | -9.37E-02 | 2.32E-01 | 8 | -163.288 | 342.577 | 0.061 | 0.205 |
| -0.681 | NA | NA | + | NA | NA | 2.18E-01 | 6 | -165.664 | 343.327 | 0.811 | 0.141 |
| -0.899 | 1.89E-09 | NA | + | -8.13E-04 | NA | 2.40E-01 | 8 | -163.668 | 343.336 | 0.819 | 0.14 |
| -0.712 | 1.73E-09 | NA | + | -9.05E-04 | -8.90E-02 | 2.42E-01 | 9 | -162.791 | 343.582 | 1.066 | 0.124 |
| -0.514 | NA | NA | + | NA | -7.41E-02 | 2.18E-01 | 7 | -165.055 | 344.109 | 1.593 | 0.095 |
| -0.731 | 1.69E-09 | NA | + | NA | NA | 2.26E-01 | 7 | -165.2 | 344.4 | 1.884 | 0.082 |
| -0.498 | NA | NA | + | NA | NA | 1.77E-01 | 6 | -168.559 | 349.117 | 0 | 0.454 |
| -0.536 | 1.42E-09 | NA | + | NA | NA | 1.83E-01 | 7 | -168.335 | 350.669 | 1.552 | 0.209 |
| -0.519 | NA | NA | + | -9.67E-05 | NA | 1.79E-01 | 7 | -168.541 | 351.081 | 1.964 | 0.17 |
| -0.495 | NA | NA | + | NA | -1.20E-03 | 1.77E-01 | 7 | -168.559 | 351.117 | 2 | 0.167 |
| -0.453 | NA | NA | + | NA | NA | 1.70E-01 | 6 | -155.775 | 323.551 | 0 | 0.436 |
| -0.489 | 9.02E-10 | NA | + | NA | NA | 1.79E-01 | 7 | -155.512 | 325.023 | 1.473 | 0.209 |
| -0.503 | NA | NA | + | -2.35E-04 | NA | 1.74E-01 | 7 | -155.647 | 325.294 | 1.743 | 0.183 |
| -0.397 | NA | NA | + | NA | -2.61E-02 | 1.71E-01 | 7 | -155.705 | 325.411 | 1.86 | 0.172 |
| -0.334 | NA | NA | + | NA | NA | 1.31E-01 | 6 | -143.439 | 298.877 | 0 | 0.372 |
| -0.277 | NA | NA | NA | NA | NA | 1.68E-01 | 3 | -147.149 | 300.299 | 1.421 | 0.183 |
| -0.359 | 7.04E-10 | NA | + | NA | NA | 1.36E-01 | 7 | -143.297 | 300.593 | 1.716 | 0.158 |
| -0.374 | NA | NA | + | -1.69E-04 | NA | 1.34E-01 | 7 | -143.371 | 300.743 | 1.865 | 0.146 |
| -0.369 | NA | NA | + | NA | 1.61E-02 | 1.30E-01 | 7 | -143.413 | 300.826 | 1.949 | 0.141 |
| -0.669 | NA | NA | + | NA | NA | 2.18E-01 | 6 | -169.955 | 351.909 | 0 | 0.381 |
| -0.77 | NA | NA | + | -4.93E-04 | NA | 2.27E-01 | 7 | -169.437 | 352.873 | 0.964 | 0.235 |
| -0.719 | 1.69E-09 | NA | + | NA | NA | 2.27E-01 | 7 | -169.567 | 353.134 | 1.225 | 0.206 |
| -0.555 | NA | NA | + | NA | -4.92E-02 | 2.18E-01 | 7 | -169.713 | 353.426 | 1.516 | 0.178 |
| -0.371 | NA | NA | + | NA | NA | 1.50E-01 | 6 | -136.119 | 284.238 | 0 | 0.364 |
| -0.402 | 9.87E-10 | NA | + | NA | NA | 1.56E-01 | 7 | -135.895 | 285.789 | 1.551 | 0.168 |
| -0.288 | NA | NA | + | NA | -3.83E-02 | 1.51E-01 | 7 | -135.942 | 285.883 | 1.646 | 0.16 |
| -0.43 | NA | NA | + | -2.46E-04 | NA | 1.56E-01 | 7 | -135.966 | 285.932 | 1.695 | 0.156 |
| -0.325 | NA | NA | NA | NA | NA | 1.87E-01 | 3 | -139.994 | 285.989 | 1.751 | 0.152 |
| -0.431 | NA | NA | + | NA | NA | 1.63E-01 | 6 | -149.932 | 311.863 | 0 | 0.441 |
| -0.462 | 9.98E-10 | NA | + | NA | NA | 1.68E-01 | 7 | -149.724 | 313.447 | 1.584 | 0.2 |
| -0.494 | NA | NA | + | -2.86E-04 | NA | 1.68E-01 | 7 | -149.743 | 313.486 | 1.622 | 0.196 |
| -0.448 | NA | NA | + | NA | 8.04E-03 | 1.62E-01 | 7 | -149.925 | 313.851 | 1.987 | 0.163 |
| -0.492 | NA | NA | + | NA | NA | 1.75E-01 | 6 | -156.484 | 324.967 | 0 | 0.419 |
| -0.571 | NA | NA | + | -3.72E-04 | NA | 1.81E-01 | 7 | -156.172 | 326.344 | 1.377 | 0.21 |
| -0.528 | 1.20E-09 | NA | + | NA | NA | 1.81E-01 | 7 | -156.232 | 326.464 | 1.497 | 0.198 |
| -0.418 | NA | NA | + | NA | -3.36E-02 | 1.76E-01 | 7 | -156.369 | 326.738 | 1.771 | 0.173 |
| -0.617 | NA | NA | + | -8.35E-04 | -9.82E-02 | 2.42E-01 | 8 | -156.718 | 329.436 | 0 | 0.217 |
| -0.815 | NA | NA | + | -7.09E-04 | NA | 2.41E-01 | 7 | -157.821 | 329.641 | 0.206 | 0.196 |
| -0.672 | NA | NA | + | NA | NA | 2.29E-01 | 6 | -159.086 | 330.173 | 0.737 | 0.15 |
| -0.873 | 2.15E-09 | NA | + | -7.24E-04 | NA | 2.49E-01 | 8 | -157.292 | 330.583 | 1.148 | 0.122 |
| -0.679 | 1.87E-09 | NA | + | -8.40E-04 | -9.25E-02 | 2.49E-01 | 9 | -156.317 | 330.634 | 1.199 | 0.119 |
| -0.502 | NA | NA | + | NA | -7.52E-02 | 2.28E-01 | 7 | -158.429 | 330.858 | 1.422 | 0.107 |
| -0.725 | 2.04E-09 | NA | + | NA | NA | 2.36E-01 | 7 | -158.617 | 331.233 | 1.798 | 0.088 |
| -0.473 | NA | NA | + | NA | NA | 1.71E-01 | 6 | -150.623 | 313.245 | 0 | 0.416 |
| -0.511 | 1.38E-09 | NA | + | NA | NA | 1.77E-01 | 7 | -150.337 | 314.674 | 1.429 | 0.204 |
| -0.542 | NA | NA | + | -3.06E-04 | NA | 1.77E-01 | 7 | -150.404 | 314.807 | 1.562 | 0.19 |
| -0.375 | NA | NA | + | NA | -4.47E-02 | 1.73E-01 | 7 | -150.406 | 314.812 | 1.567 | 0.19 |
| -0.648 | NA | NA | + | -9.03E-04 | -1.22E-01 | 2.60E-01 | 8 | -166.317 | 348.634 | 0 | 0.352 |
| -0.726 | 2.23E-09 | NA | + | -9.14E-04 | -1.14E-01 | 2.69E-01 | 9 | -165.775 | 349.551 | 0.917 | 0.223 |
| -0.903 | NA | NA | + | -7.50E-04 | NA | 2.62E-01 | 7 | -168.083 | 350.165 | 1.531 | 0.164 |
| -0.53 | NA | NA | + | NA | -9.88E-02 | 2.47E-01 | 7 | -168.306 | 350.613 | 1.979 | 0.131 |
| -0.976 | 2.66E-09 | NA | + | -7.75E-04 | NA | 2.72E-01 | 8 | -167.317 | 350.633 | 1.999 | 0.13 |
| -0.397 | NA | NA | + | NA | NA | 1.58E-01 | 6 | -145.022 | 302.044 | 0 | 0.367 |
| -0.348 | NA | NA | NA | NA | NA | 1.94E-01 | 3 | -148.617 | 303.234 | 1.191 | 0.202 |
| -0.422 | 8.17E-10 | NA | + | NA | NA | 1.62E-01 | 7 | -144.882 | 303.765 | 1.721 | 0.155 |
| -0.426 | NA | NA | + | -1.23E-04 | NA | 1.60E-01 | 7 | -144.986 | 303.972 | 1.928 | 0.14 |
| -0.396 | NA | NA | + | NA | -6.84E-04 | 1.58E-01 | 7 | -145.022 | 304.044 | 2 | 0.135 |
| -0.533 | NA | NA | + | NA | NA | 1.87E-01 | 6 | -159.113 | 330.226 | 0 | 0.39 |
| -0.643 | NA | NA | + | -5.11E-04 | NA | 1.95E-01 | 7 | -158.529 | 331.058 | 0.832 | 0.257 |
| -0.571 | 1.35E-09 | NA | + | NA | NA | 1.93E-01 | 7 | -158.829 | 331.657 | 1.431 | 0.191 |
| -0.459 | NA | NA | + | NA | -3.35E-02 | 1.88E-01 | 7 | -158.996 | 331.992 | 1.765 | 0.161 |
| -0.521 | NA | NA | + | NA | NA | 1.83E-01 | 6 | -146.225 | 304.45 | 0 | 0.249 |
| -0.652 | NA | NA | + | -6.03E-04 | NA | 1.94E-01 | 7 | -145.286 | 304.571 | 0.121 | 0.234 |
| -0.518 | NA | NA | + | -6.61E-04 | -6.70E-02 | 1.97E-01 | 8 | -144.764 | 305.527 | 1.077 | 0.145 |
| -0.401 | NA | NA | + | NA | -5.45E-02 | 1.84E-01 | 7 | -145.879 | 305.757 | 1.307 | 0.13 |
| -0.697 | 1.43E-09 | NA | + | -6.29E-04 | NA | 2.01E-01 | 8 | -144.938 | 305.877 | 1.427 | 0.122 |
| -0.555 | 1.26E-09 | NA | + | NA | NA | 1.89E-01 | 7 | -145.958 | 305.915 | 1.465 | 0.12 |
| -0.428 | NA | NA | + | NA | NA | 1.61E-01 | 6 | -143.552 | 299.104 | 0 | 0.405 |
| -0.516 | NA | NA | + | -3.86E-04 | NA | 1.69E-01 | 7 | -143.178 | 300.356 | 1.252 | 0.217 |
| -0.326 | NA | NA | + | NA | -4.60E-02 | 1.62E-01 | 7 | -143.31 | 300.621 | 1.517 | 0.19 |
| -0.46 | 1.01E-09 | NA | + | NA | NA | 1.67E-01 | 7 | -143.321 | 300.643 | 1.539 | 0.188 |
| -0.453 | NA | NA | + | NA | NA | 1.64E-01 | 6 | -153.35 | 318.7 | 0 | 0.424 |
| -0.489 | 1.31E-09 | NA | + | NA | NA | 1.69E-01 | 7 | -153.11 | 320.22 | 1.52 | 0.198 |
| -0.352 | NA | NA | + | NA | -4.59E-02 | 1.64E-01 | 7 | -153.12 | 320.239 | 1.539 | 0.196 |
| -0.515 | NA | NA | + | -2.59E-04 | NA | 1.69E-01 | 7 | -153.199 | 320.399 | 1.698 | 0.181 |
| -0.459 | NA | NA | + | NA | NA | 1.67E-01 | 6 | -148.246 | 308.493 | 0 | 0.424 |
| -0.494 | 1.09E-09 | NA | + | NA | NA | 1.74E-01 | 7 | -148.007 | 310.015 | 1.522 | 0.198 |
| -0.531 | NA | NA | + | -3.02E-04 | NA | 1.74E-01 | 7 | -148.031 | 310.063 | 1.57 | 0.193 |
| -0.371 | NA | NA | + | NA | -3.90E-02 | 1.68E-01 | 7 | -148.079 | 310.157 | 1.665 | 0.184 |
| -0.389 | NA | NA | + | NA | NA | 1.52E-01 | 6 | -145.054 | 302.108 | 0 | 0.372 |
| -0.32 | NA | NA | NA | NA | NA | 1.88E-01 | 3 | -148.807 | 303.614 | 1.506 | 0.175 |
| -0.414 | 9.40E-10 | NA | + | NA | NA | 1.56E-01 | 7 | -144.912 | 303.824 | 1.715 | 0.158 |
| -0.445 | NA | NA | + | -2.43E-04 | NA | 1.57E-01 | 7 | -144.916 | 303.832 | 1.723 | 0.157 |
| -0.377 | NA | NA | + | NA | -5.57E-03 | 1.53E-01 | 7 | -145.051 | 304.102 | 1.994 | 0.137 |
| -0.528 | NA | NA | + | -7.70E-04 | -9.04E-02 | 2.08E-01 | 8 | -149.988 | 315.976 | 0 | 0.202 |
| -0.713 | NA | NA | + | -6.72E-04 | NA | 2.07E-01 | 7 | -150.998 | 315.997 | 0.021 | 0.2 |
| -0.563 | NA | NA | + | NA | NA | 1.94E-01 | 6 | -152.186 | 316.372 | 0.396 | 0.166 |
| -0.398 | NA | NA | + | NA | -7.28E-02 | 1.93E-01 | 7 | -151.526 | 317.052 | 1.076 | 0.118 |
| -0.766 | 1.64E-09 | NA | + | -7.04E-04 | NA | 2.15E-01 | 8 | -150.53 | 317.06 | 1.084 | 0.118 |
| -0.585 | 1.46E-09 | NA | + | -7.94E-04 | -8.61E-02 | 2.15E-01 | 9 | -149.615 | 317.23 | 1.254 | 0.108 |
| -0.604 | 1.43E-09 | NA | + | NA | NA | 2.01E-01 | 7 | -151.834 | 317.668 | 1.692 | 0.087 |
| -0.434 | NA | NA | + | NA | NA | 1.61E-01 | 6 | -143.327 | 298.654 | 0 | 0.415 |
| -0.526 | NA | NA | + | -3.87E-04 | NA | 1.70E-01 | 7 | -142.945 | 299.889 | 1.236 | 0.224 |
| -0.463 | 1.12E-09 | NA | + | NA | NA | 1.65E-01 | 7 | -143.156 | 300.312 | 1.659 | 0.181 |
| -0.349 | NA | NA | + | NA | -3.82E-02 | 1.62E-01 | 7 | -143.156 | 300.313 | 1.659 | 0.181 |
| -0.576 | NA | NA | + | NA | NA | 1.95E-01 | 6 | -153.042 | 318.084 | 0 | 0.191 |
| -0.701 | NA | NA | + | -6.02E-04 | NA | 2.07E-01 | 7 | -152.07 | 318.141 | 0.057 | 0.185 |
| -0.528 | NA | NA | + | -6.77E-04 | -8.74E-02 | 2.10E-01 | 8 | -151.127 | 318.253 | 0.169 | 0.175 |
| -0.414 | NA | NA | + | NA | -7.48E-02 | 1.97E-01 | 7 | -152.35 | 318.7 | 0.616 | 0.14 |
| -0.759 | 1.36E-09 | NA | + | -6.53E-04 | NA | 2.18E-01 | 8 | -151.574 | 319.148 | 1.065 | 0.112 |
| -0.588 | 1.25E-09 | NA | + | -7.21E-04 | -8.39E-02 | 2.21E-01 | 9 | -150.703 | 319.406 | 1.322 | 0.098 |
| -0.614 | 1.11E-09 | NA | + | NA | NA | 2.04E-01 | 7 | -152.708 | 319.416 | 1.332 | 0.098 |
| -0.393 | NA | NA | + | NA | NA | 1.49E-01 | 6 | -155.825 | 323.649 | 0 | 0.374 |
| -0.352 | NA | NA | NA | NA | NA | 1.94E-01 | 3 | -159.578 | 325.155 | 1.506 | 0.176 |
| -0.424 | 1.11E-09 | NA | + | NA | NA | 1.54E-01 | 7 | -155.645 | 325.29 | 1.641 | 0.165 |
| -0.342 | NA | NA | + | NA | -2.29E-02 | 1.49E-01 | 7 | -155.773 | 325.546 | 1.897 | 0.145 |
| -0.417 | NA | NA | + | -9.68E-05 | NA | 1.51E-01 | 7 | -155.805 | 325.61 | 1.961 | 0.14 |
| -0.509 | NA | NA | + | NA | NA | 1.69E-01 | 6 | -157.954 | 327.909 | 0 | 0.431 |
| -0.583 | NA | NA | + | -3.24E-04 | NA | 1.76E-01 | 7 | -157.719 | 329.439 | 1.53 | 0.201 |
| -0.543 | 1.25E-09 | NA | + | NA | NA | 1.75E-01 | 7 | -157.749 | 329.498 | 1.589 | 0.195 |
| -0.441 | NA | NA | + | NA | -2.98E-02 | 1.70E-01 | 7 | -157.865 | 329.731 | 1.822 | 0.173 |
| -0.495 | NA | NA | + | NA | NA | 1.77E-01 | 6 | -159.013 | 330.027 | 0 | 0.427 |
| -0.583 | NA | NA | + | -3.98E-04 | NA | 1.85E-01 | 7 | -158.671 | 331.342 | 1.316 | 0.221 |
| -0.529 | 1.28E-09 | NA | + | NA | NA | 1.82E-01 | 7 | -158.818 | 331.635 | 1.608 | 0.191 |
| -0.463 | NA | NA | + | NA | -1.45E-02 | 1.77E-01 | 7 | -158.993 | 331.987 | 1.96 | 0.16 |
| -0.465 | NA | NA | + | NA | NA | 1.73E-01 | 6 | -146.536 | 305.071 | 0 | 0.418 |
| -0.54 | NA | NA | + | -3.40E-04 | NA | 1.79E-01 | 7 | -146.255 | 306.511 | 1.439 | 0.204 |
| -0.499 | 1.27E-09 | NA | + | NA | NA | 1.78E-01 | 7 | -146.282 | 306.563 | 1.492 | 0.198 |
| -0.383 | NA | NA | + | NA | -3.78E-02 | 1.74E-01 | 7 | -146.376 | 306.752 | 1.68 | 0.18 |
| -0.544 | NA | NA | + | NA | NA | 1.89E-01 | 6 | -165.155 | 342.311 | 0 | 0.427 |
| -0.583 | 1.34E-09 | NA | + | NA | NA | 1.96E-01 | 7 | -164.91 | 343.82 | 1.509 | 0.201 |
| -0.618 | NA | NA | + | -3.40E-04 | NA | 1.96E-01 | 7 | -164.912 | 343.824 | 1.513 | 0.201 |
| -0.476 | NA | NA | + | NA | -3.02E-02 | 1.89E-01 | 7 | -165.069 | 344.137 | 1.827 | 0.171 |
| -0.366 | NA | NA | + | NA | NA | 1.46E-01 | 6 | -140.147 | 292.295 | 0 | 0.377 |
| -0.308 | NA | NA | NA | NA | NA | 1.81E-01 | 3 | -143.945 | 293.889 | 1.595 | 0.17 |
| -0.392 | 7.53E-10 | NA | + | NA | NA | 1.51E-01 | 7 | -139.995 | 293.989 | 1.695 | 0.162 |
| -0.413 | NA | NA | + | -1.94E-04 | NA | 1.50E-01 | 7 | -140.054 | 294.107 | 1.813 | 0.152 |
| -0.373 | NA | NA | + | NA | 3.39E-03 | 1.46E-01 | 7 | -140.146 | 294.292 | 1.998 | 0.139 |
| -0.57 | NA | NA | + | NA | NA | 1.98E-01 | 6 | -155.889 | 323.778 | 0 | 0.276 |
| -0.679 | NA | NA | + | -5.09E-04 | NA | 2.07E-01 | 7 | -155.26 | 324.519 | 0.741 | 0.19 |
| -0.612 | 1.24E-09 | NA | + | NA | NA | 2.07E-01 | 7 | -155.498 | 324.996 | 1.218 | 0.15 |
| -0.442 | NA | NA | + | NA | -5.68E-02 | 1.99E-01 | 7 | -155.534 | 325.068 | 1.29 | 0.145 |
| -0.744 | 1.47E-09 | NA | + | -5.72E-04 | NA | 2.19E-01 | 8 | -154.712 | 325.425 | 1.646 | 0.121 |
| -0.539 | NA | NA | + | -5.76E-04 | -6.91E-02 | 2.09E-01 | 8 | -154.74 | 325.479 | 1.701 | 0.118 |
| -0.359 | NA | NA | + | NA | NA | 1.39E-01 | 6 | -151.348 | 314.697 | 0 | 0.319 |
| -0.299 | NA | NA | NA | NA | NA | 1.80E-01 | 3 | -154.959 | 315.919 | 1.222 | 0.173 |
| -0.209 | NA | NA | NA | 4.67E-04 | NA | 1.56E-01 | 4 | -154.185 | 316.369 | 1.673 | 0.138 |
| -0.387 | 8.92E-10 | NA | + | NA | NA | 1.44E-01 | 7 | -151.208 | 316.416 | 1.719 | 0.135 |
| -0.373 | NA | NA | + | -5.64E-05 | NA | 1.40E-01 | 7 | -151.341 | 316.683 | 1.986 | 0.118 |
| -0.356 | NA | NA | + | NA | -1.34E-03 | 1.39E-01 | 7 | -151.348 | 316.696 | 2 | 0.117 |
| -0.563 | NA | NA | + | NA | NA | 1.84E-01 | 6 | -168.896 | 349.791 | 0 | 0.422 |
| -0.642 | NA | NA | + | -3.67E-04 | NA | 1.91E-01 | 7 | -168.622 | 351.245 | 1.453 | 0.204 |
| -0.603 | 1.49E-09 | NA | + | NA | NA | 1.90E-01 | 7 | -168.641 | 351.281 | 1.49 | 0.2 |
| -0.482 | NA | NA | + | NA | -3.56E-02 | 1.84E-01 | 7 | -168.779 | 351.558 | 1.766 | 0.174 |
| -0.491 | NA | NA | + | NA | NA | 1.75E-01 | 6 | -152.896 | 317.793 | 0 | 0.426 |
| -0.528 | 1.33E-09 | NA | + | NA | NA | 1.81E-01 | 7 | -152.626 | 319.251 | 1.458 | 0.206 |
| -0.562 | NA | NA | + | -3.11E-04 | NA | 1.81E-01 | 7 | -152.668 | 319.335 | 1.542 | 0.197 |
| -0.426 | NA | NA | + | NA | -2.91E-02 | 1.76E-01 | 7 | -152.81 | 319.62 | 1.827 | 0.171 |
| -0.451 | NA | NA | + | NA | NA | 1.60E-01 | 6 | -149.959 | 311.919 | 0 | 0.425 |
| -0.482 | 1.09E-09 | NA | + | NA | NA | 1.65E-01 | 7 | -149.742 | 313.484 | 1.565 | 0.194 |
| -0.356 | NA | NA | + | NA | -4.36E-02 | 1.62E-01 | 7 | -149.761 | 313.521 | 1.602 | 0.191 |
| -0.518 | NA | NA | + | -2.85E-04 | NA | 1.65E-01 | 7 | -149.765 | 313.53 | 1.611 | 0.19 |
| -0.535 | NA | NA | + | NA | NA | 1.86E-01 | 6 | -156.111 | 324.223 | 0 | 0.404 |
| -0.629 | NA | NA | + | -4.30E-04 | NA | 1.95E-01 | 7 | -155.687 | 325.373 | 1.15 | 0.227 |
| -0.572 | 1.43E-09 | NA | + | NA | NA | 1.91E-01 | 7 | -155.862 | 325.725 | 1.502 | 0.19 |
| -0.44 | NA | NA | + | NA | -4.17E-02 | 1.86E-01 | 7 | -155.926 | 325.851 | 1.628 | 0.179 |
| -0.391 | NA | NA | + | NA | NA | 1.45E-01 | 6 | -159.172 | 330.343 | 0 | 0.316 |
| -0.323 | NA | NA | NA | NA | NA | 1.84E-01 | 3 | -162.734 | 331.469 | 1.125 | 0.18 |
| -0.42 | 9.39E-10 | NA | + | NA | NA | 1.50E-01 | 7 | -159.016 | 332.033 | 1.689 | 0.136 |
| -0.234 | NA | NA | NA | 4.70E-04 | NA | 1.61E-01 | 4 | -162.042 | 332.084 | 1.74 | 0.132 |
| -0.433 | NA | NA | + | NA | 1.90E-02 | 1.44E-01 | 7 | -159.141 | 332.282 | 1.938 | 0.12 |
| -0.404 | NA | NA | + | -5.48E-05 | NA | 1.46E-01 | 7 | -159.166 | 332.332 | 1.988 | 0.117 |
| -0.672 | NA | NA | + | -6.74E-04 | NA | 1.96E-01 | 7 | -142.506 | 299.012 | 0 | 0.229 |
| -0.532 | NA | NA | + | NA | NA | 1.85E-01 | 6 | -143.729 | 299.459 | 0.446 | 0.184 |
| -0.524 | NA | NA | + | -7.41E-04 | -7.58E-02 | 2.00E-01 | 8 | -141.814 | 299.627 | 0.615 | 0.169 |
| -0.72 | 1.33E-09 | NA | + | -7.06E-04 | NA | 2.04E-01 | 8 | -142.082 | 300.163 | 1.151 | 0.129 |
| -0.4 | NA | NA | + | NA | -6.14E-02 | 1.88E-01 | 7 | -143.275 | 300.551 | 1.538 | 0.106 |
| -0.567 | 1.14E-09 | NA | + | NA | NA | 1.91E-01 | 7 | -143.42 | 300.841 | 1.828 | 0.092 |
| -0.574 | 1.26E-09 | NA | + | -7.69E-04 | -7.34E-02 | 2.08E-01 | 9 | -141.431 | 300.861 | 1.849 | 0.091 |
| -0.504 | NA | NA | + | NA | NA | 1.77E-01 | 6 | -148.278 | 308.555 | 0 | 0.406 |
| -0.582 | NA | NA | + | -3.70E-04 | NA | 1.84E-01 | 7 | -147.946 | 309.893 | 1.338 | 0.208 |
| -0.54 | 1.24E-09 | NA | + | NA | NA | 1.83E-01 | 7 | -148.014 | 310.027 | 1.472 | 0.194 |
| -0.399 | NA | NA | + | NA | -4.73E-02 | 1.77E-01 | 7 | -148.024 | 310.047 | 1.492 | 0.192 |
| -0.499 | NA | NA | + | NA | NA | 1.75E-01 | 6 | -148.069 | 308.138 | 0 | 0.317 |
| -0.602 | NA | NA | + | -4.59E-04 | NA | 1.83E-01 | 7 | -147.524 | 309.048 | 0.909 | 0.201 |
| -0.36 | NA | NA | + | NA | -6.13E-02 | 1.76E-01 | 7 | -147.61 | 309.22 | 1.082 | 0.185 |
| -0.536 | 1.19E-09 | NA | + | NA | NA | 1.83E-01 | 7 | -147.766 | 309.532 | 1.394 | 0.158 |
| -0.453 | NA | NA | + | -5.30E-04 | -7.23E-02 | 1.85E-01 | 8 | -146.893 | 309.786 | 1.648 | 0.139 |
| -0.711 | NA | NA | + | -6.39E-04 | NA | 2.10E-01 | 7 | -152.087 | 318.174 | 0 | 0.244 |
| -0.579 | NA | NA | + | NA | NA | 1.98E-01 | 6 | -153.1 | 318.2 | 0.026 | 0.241 |
| -0.579 | NA | NA | + | -7.01E-04 | -6.65E-02 | 2.13E-01 | 8 | -151.611 | 319.222 | 1.048 | 0.145 |
| -0.76 | 1.50E-09 | NA | + | -6.64E-04 | NA | 2.18E-01 | 8 | -151.703 | 319.406 | 1.232 | 0.132 |
| -0.618 | 1.34E-09 | NA | + | NA | NA | 2.05E-01 | 7 | -152.798 | 319.596 | 1.422 | 0.12 |
| -0.467 | NA | NA | + | NA | -5.14E-02 | 1.99E-01 | 7 | -152.814 | 319.628 | 1.454 | 0.118 |
| -0.494 | NA | NA | + | NA | NA | 1.84E-01 | 6 | -157.134 | 326.267 | 0 | 0.431 |
| -0.528 | 1.13E-09 | NA | + | NA | NA | 1.90E-01 | 7 | -156.89 | 327.78 | 1.513 | 0.202 |
| -0.41 | NA | NA | + | NA | -3.83E-02 | 1.85E-01 | 7 | -156.978 | 327.957 | 1.69 | 0.185 |
| -0.549 | NA | NA | + | -2.40E-04 | NA | 1.89E-01 | 7 | -157.004 | 328.008 | 1.741 | 0.181 |
| -0.341 | NA | NA | + | NA | NA | 1.36E-01 | 6 | -150.634 | 313.268 | 0 | 0.275 |
| -0.287 | NA | NA | NA | NA | NA | 1.73E-01 | 3 | -153.951 | 313.902 | 0.634 | 0.201 |
| -0.188 | NA | NA | NA | 5.13E-04 | NA | 1.47E-01 | 4 | -152.956 | 313.912 | 0.644 | 0.2 |
| -0.369 | 8.83E-10 | NA | + | NA | NA | 1.42E-01 | 7 | -150.46 | 314.919 | 1.651 | 0.121 |
| -0.325 | NA | NA | + | 6.75E-05 | NA | 1.35E-01 | 7 | -150.624 | 315.248 | 1.98 | 0.102 |
| -0.328 | NA | NA | + | NA | -5.77E-03 | 1.37E-01 | 7 | -150.631 | 315.261 | 1.993 | 0.102 |
| -0.412 | NA | NA | + | NA | NA | 1.56E-01 | 6 | -142.248 | 296.496 | 0 | 0.371 |
| -0.317 | NA | NA | + | NA | -4.39E-02 | 1.58E-01 | 7 | -142.028 | 298.056 | 1.56 | 0.17 |
| -0.441 | 1.01E-09 | NA | + | NA | NA | 1.61E-01 | 7 | -142.064 | 298.128 | 1.633 | 0.164 |
| -0.465 | NA | NA | + | -2.08E-04 | NA | 1.61E-01 | 7 | -142.138 | 298.276 | 1.78 | 0.152 |
| -0.332 | NA | NA | NA | NA | NA | 1.90E-01 | 3 | -146.211 | 298.422 | 1.926 | 0.142 |
| -0.58 | NA | NA | + | NA | NA | 1.98E-01 | 6 | -161.273 | 334.546 | 0 | 0.394 |
| -0.622 | 1.40E-09 | NA | + | NA | NA | 2.06E-01 | 7 | -160.915 | 335.83 | 1.284 | 0.208 |
| -0.659 | NA | NA | + | -3.90E-04 | NA | 2.04E-01 | 7 | -160.926 | 335.853 | 1.307 | 0.205 |
| -0.466 | NA | NA | + | NA | -5.01E-02 | 1.98E-01 | 7 | -160.988 | 335.977 | 1.431 | 0.193 |
| -0.488 | NA | NA | + | NA | NA | 1.69E-01 | 6 | -147.873 | 307.747 | 0 | 0.282 |
| -0.617 | NA | NA | + | -5.55E-04 | NA | 1.82E-01 | 7 | -147.079 | 308.158 | 0.411 | 0.23 |
| -0.38 | NA | NA | + | NA | -4.76E-02 | 1.70E-01 | 7 | -147.629 | 309.257 | 1.51 | 0.132 |
| -0.516 | 1.04E-09 | NA | + | NA | NA | 1.74E-01 | 7 | -147.682 | 309.363 | 1.617 | 0.126 |
| -0.496 | NA | NA | + | -5.98E-04 | -5.80E-02 | 1.83E-01 | 8 | -146.715 | 309.431 | 1.684 | 0.121 |
| -0.656 | 1.20E-09 | NA | + | -5.78E-04 | NA | 1.88E-01 | 8 | -146.822 | 309.645 | 1.898 | 0.109 |
| -0.499 | NA | NA | + | -6.87E-04 | -9.06E-02 | 2.11E-01 | 8 | -143.935 | 303.869 | 0 | 0.175 |
| -0.557 | NA | NA | + | NA | NA | 1.96E-01 | 6 | -145.944 | 303.888 | 0.019 | 0.173 |
| -0.68 | NA | NA | + | -5.95E-04 | NA | 2.07E-01 | 7 | -144.986 | 303.973 | 0.103 | 0.166 |
| -0.389 | NA | NA | + | NA | -7.59E-02 | 1.98E-01 | 7 | -145.2 | 304.399 | 0.53 | 0.134 |
| -0.733 | 1.52E-09 | NA | + | -6.36E-04 | NA | 2.16E-01 | 8 | -144.505 | 305.011 | 1.142 | 0.099 |
| -0.554 | 1.40E-09 | NA | + | -7.21E-04 | -8.72E-02 | 2.18E-01 | 9 | -143.528 | 305.055 | 1.186 | 0.097 |
| -0.595 | 1.30E-09 | NA | + | NA | NA | 2.03E-01 | 7 | -145.595 | 305.189 | 1.32 | 0.09 |
| -0.431 | 1.17E-09 | NA | + | NA | -7.25E-02 | 2.04E-01 | 8 | -144.916 | 305.832 | 1.963 | 0.066 |
| -0.576 | NA | NA | + | NA | NA | 1.97E-01 | 6 | -161.304 | 334.609 | 0 | 0.401 |
| -0.658 | NA | NA | + | -3.95E-04 | NA | 2.04E-01 | 7 | -160.948 | 335.897 | 1.288 | 0.211 |
| -0.614 | 1.31E-09 | NA | + | NA | NA | 2.04E-01 | 7 | -161.013 | 336.026 | 1.417 | 0.198 |
| -0.465 | NA | NA | + | NA | -4.95E-02 | 1.98E-01 | 7 | -161.048 | 336.097 | 1.488 | 0.191 |
| -0.472 | NA | NA | + | NA | NA | 1.66E-01 | 6 | -153.6 | 319.2 | 0 | 0.435 |
| -0.549 | NA | NA | + | -3.27E-04 | NA | 1.74E-01 | 7 | -153.364 | 320.727 | 1.528 | 0.202 |
| -0.505 | 1.22E-09 | NA | + | NA | NA | 1.71E-01 | 7 | -153.415 | 320.831 | 1.631 | 0.192 |
| -0.413 | NA | NA | + | NA | -2.58E-02 | 1.67E-01 | 7 | -153.534 | 321.068 | 1.869 | 0.171 |
| -0.494 | NA | NA | + | NA | NA | 1.76E-01 | 6 | -146.051 | 304.101 | 0 | 0.33 |
| -0.596 | NA | NA | + | -4.92E-04 | NA | 1.84E-01 | 7 | -145.427 | 304.853 | 0.752 | 0.226 |
| -0.53 | 9.95E-10 | NA | + | NA | NA | 1.85E-01 | 7 | -145.727 | 305.454 | 1.353 | 0.168 |
| -0.419 | NA | NA | + | NA | -3.42E-02 | 1.77E-01 | 7 | -145.915 | 305.829 | 1.728 | 0.139 |
| -0.656 | 1.25E-09 | NA | + | -5.64E-04 | NA | 1.96E-01 | 8 | -144.925 | 305.85 | 1.749 | 0.138 |
| -0.433 | NA | NA | + | NA | NA | 1.63E-01 | 6 | -145.412 | 302.824 | 0 | 0.422 |
| -0.511 | NA | NA | + | -3.28E-04 | NA | 1.71E-01 | 7 | -145.147 | 304.294 | 1.47 | 0.202 |
| -0.464 | 1.01E-09 | NA | + | NA | NA | 1.69E-01 | 7 | -145.204 | 304.408 | 1.584 | 0.191 |
| -0.343 | NA | NA | + | NA | -3.97E-02 | 1.64E-01 | 7 | -145.239 | 304.478 | 1.654 | 0.185 |
| -0.649 | NA | NA | + | NA | NA | 2.21E-01 | 6 | -165.304 | 342.609 | 0 | 0.172 |
| -0.574 | NA | NA | + | -7.18E-04 | -9.83E-02 | 2.32E-01 | 8 | -163.361 | 342.721 | 0.112 | 0.162 |
| -0.766 | NA | NA | + | -5.97E-04 | NA | 2.29E-01 | 7 | -164.478 | 342.955 | 0.346 | 0.144 |
| -0.472 | NA | NA | + | NA | -8.09E-02 | 2.21E-01 | 7 | -164.534 | 343.068 | 0.459 | 0.136 |
| -0.705 | 2.10E-09 | NA | + | NA | NA | 2.29E-01 | 7 | -164.742 | 343.485 | 0.876 | 0.111 |
| -0.829 | 2.21E-09 | NA | + | -6.18E-04 | NA | 2.38E-01 | 8 | -163.851 | 343.702 | 1.093 | 0.099 |
| -0.642 | 1.95E-09 | NA | + | -7.29E-04 | -9.21E-02 | 2.39E-01 | 9 | -162.871 | 343.742 | 1.133 | 0.097 |
| -0.535 | 1.88E-09 | NA | + | NA | -7.48E-02 | 2.28E-01 | 8 | -164.087 | 344.174 | 1.565 | 0.078 |
| -0.397 | NA | NA | + | NA | NA | 1.40E-01 | 6 | -146.863 | 305.726 | 0 | 0.442 |
| -0.43 | 1.17E-09 | NA | + | NA | NA | 1.45E-01 | 7 | -146.668 | 307.336 | 1.61 | 0.198 |
| -0.465 | NA | NA | + | -2.85E-04 | NA | 1.46E-01 | 7 | -146.68 | 307.359 | 1.633 | 0.195 |
| -0.371 | NA | NA | + | NA | -1.15E-02 | 1.41E-01 | 7 | -146.849 | 307.699 | 1.973 | 0.165 |
| -0.486 | NA | NA | + | NA | NA | 1.73E-01 | 6 | -168.429 | 348.858 | 0 | 0.454 |
| -0.522 | 1.08E-09 | NA | + | NA | NA | 1.81E-01 | 7 | -168.189 | 350.377 | 1.52 | 0.212 |
| -0.475 | NA | NA | + | NA | -4.74E-03 | 1.74E-01 | 7 | -168.427 | 350.854 | 1.996 | 0.167 |
| -0.492 | NA | NA | + | -2.80E-05 | NA | 1.74E-01 | 7 | -168.427 | 350.855 | 1.997 | 0.167 |
| -0.487 | NA | NA | + | NA | NA | 1.77E-01 | 6 | -147.563 | 307.126 | 0 | 0.381 |
| -0.598 | NA | NA | + | -4.92E-04 | NA | 1.86E-01 | 7 | -146.975 | 307.951 | 0.825 | 0.252 |
| -0.524 | 1.41E-09 | NA | + | NA | NA | 1.82E-01 | 7 | -147.289 | 308.579 | 1.453 | 0.184 |
| -0.383 | NA | NA | + | NA | -4.84E-02 | 1.80E-01 | 7 | -147.292 | 308.584 | 1.458 | 0.184 |
| -0.441 | NA | NA | + | NA | NA | 1.63E-01 | 6 | -140.543 | 293.086 | 0 | 0.411 |
| -0.524 | NA | NA | + | -3.81E-04 | NA | 1.69E-01 | 7 | -140.151 | 294.303 | 1.217 | 0.224 |
| -0.473 | 7.93E-10 | NA | + | NA | NA | 1.71E-01 | 7 | -140.281 | 294.562 | 1.476 | 0.197 |
| -0.374 | NA | NA | + | NA | -3.08E-02 | 1.64E-01 | 7 | -140.436 | 294.871 | 1.786 | 0.168 |
| -0.476 | NA | NA | + | NA | NA | 1.70E-01 | 6 | -151.52 | 315.039 | 0 | 0.403 |
| -0.578 | NA | NA | + | -4.63E-04 | NA | 1.77E-01 | 7 | -151.008 | 316.016 | 0.977 | 0.247 |
| -0.51 | 1.11E-09 | NA | + | NA | NA | 1.75E-01 | 7 | -151.257 | 316.514 | 1.475 | 0.193 |
| -0.422 | NA | NA | + | NA | -2.48E-02 | 1.71E-01 | 7 | -151.455 | 316.911 | 1.872 | 0.158 |
| -0.413 | NA | NA | + | NA | NA | 1.55E-01 | 6 | -149.505 | 311.011 | 0 | 0.451 |
| -0.443 | 8.11E-10 | NA | + | NA | NA | 1.62E-01 | 7 | -149.308 | 312.615 | 1.605 | 0.202 |
| -0.449 | NA | NA | + | -1.66E-04 | NA | 1.58E-01 | 7 | -149.441 | 312.883 | 1.872 | 0.177 |
| -0.377 | NA | NA | + | NA | -1.72E-02 | 1.56E-01 | 7 | -149.475 | 312.95 | 1.939 | 0.171 |
| -0.496 | NA | NA | + | NA | NA | 1.75E-01 | 6 | -157.898 | 327.795 | 0 | 0.401 |
| -0.373 | NA | NA | + | NA | -5.63E-02 | 1.77E-01 | 7 | -157.549 | 329.098 | 1.303 | 0.209 |
| -0.577 | NA | NA | + | -3.59E-04 | NA | 1.82E-01 | 7 | -157.608 | 329.215 | 1.42 | 0.197 |
| -0.534 | 1.45E-09 | NA | + | NA | NA | 1.81E-01 | 7 | -157.626 | 329.253 | 1.458 | 0.193 |
| -0.299 | NA | NA | NA | NA | NA | 1.76E-01 | 3 | -149.722 | 305.444 | 0 | 0.285 |
| -0.212 | NA | NA | NA | 4.69E-04 | NA | 1.54E-01 | 4 | -148.89 | 305.78 | 0.336 | 0.241 |
| -0.351 | NA | NA | + | NA | NA | 1.44E-01 | 6 | -146.894 | 305.788 | 0.344 | 0.24 |
| -0.328 | 8.02E-10 | NA | NA | NA | NA | 1.82E-01 | 4 | -149.534 | 307.067 | 1.623 | 0.127 |
| -0.27 | NA | NA | NA | NA | -1.40E-02 | 1.76E-01 | 4 | -149.702 | 307.404 | 1.96 | 0.107 |
| -0.686 | NA | NA | + | -1.12E-03 | -1.29E-01 | 2.74E-01 | 8 | -156.437 | 328.875 | 0 | 0.583 |
| -0.765 | 2.04E-09 | NA | + | -1.15E-03 | -1.22E-01 | 2.85E-01 | 9 | -155.773 | 329.546 | 0.671 | 0.417 |
| -0.431 | NA | NA | + | NA | NA | 1.63E-01 | 6 | -142.114 | 296.228 | 0 | 0.394 |
| -0.539 | NA | NA | + | -5.00E-04 | NA | 1.70E-01 | 7 | -141.486 | 296.973 | 0.745 | 0.272 |
| -0.463 | 1.03E-09 | NA | + | NA | NA | 1.68E-01 | 7 | -141.888 | 297.776 | 1.548 | 0.182 |
| -0.388 | NA | NA | + | NA | -2.08E-02 | 1.64E-01 | 7 | -142.065 | 298.131 | 1.903 | 0.152 |
| -0.52 | NA | NA | + | NA | NA | 1.82E-01 | 6 | -151.181 | 314.363 | 0 | 0.243 |
| -0.652 | NA | NA | + | -5.90E-04 | NA | 1.93E-01 | 7 | -150.299 | 314.599 | 0.236 | 0.216 |
| -0.494 | NA | NA | + | -6.65E-04 | -7.82E-02 | 1.96E-01 | 8 | -149.604 | 315.208 | 0.845 | 0.159 |
| -0.376 | NA | NA | + | NA | -6.41E-02 | 1.83E-01 | 7 | -150.711 | 315.422 | 1.059 | 0.143 |
| -0.558 | 1.46E-09 | NA | + | NA | NA | 1.88E-01 | 7 | -150.872 | 315.744 | 1.381 | 0.122 |
| -0.7 | 1.61E-09 | NA | + | -6.12E-04 | NA | 2.00E-01 | 8 | -149.92 | 315.84 | 1.477 | 0.116 |
| -0.386 | NA | NA | + | NA | NA | 1.47E-01 | 6 | -142.539 | 297.077 | 0 | 0.437 |
| -0.462 | NA | NA | + | -3.16E-04 | NA | 1.53E-01 | 7 | -142.305 | 298.611 | 1.533 | 0.203 |
| -0.414 | 9.31E-10 | NA | + | NA | NA | 1.52E-01 | 7 | -142.359 | 298.718 | 1.64 | 0.192 |
| -0.341 | NA | NA | + | NA | -2.07E-02 | 1.48E-01 | 7 | -142.492 | 298.983 | 1.906 | 0.168 |
| -0.33 | NA | NA | + | NA | NA | 1.37E-01 | 6 | -130.467 | 272.933 | 0 | 0.367 |
| -0.279 | NA | NA | NA | NA | NA | 1.72E-01 | 3 | -134.24 | 274.48 | 1.547 | 0.169 |
| -0.354 | 7.85E-10 | NA | + | NA | NA | 1.41E-01 | 7 | -130.304 | 274.607 | 1.674 | 0.159 |
| -0.381 | NA | NA | + | -2.13E-04 | NA | 1.41E-01 | 7 | -130.341 | 274.681 | 1.748 | 0.153 |
| -0.262 | NA | NA | + | NA | -3.20E-02 | 1.39E-01 | 7 | -130.343 | 274.686 | 1.753 | 0.153 |
| -0.527 | NA | NA | + | NA | NA | 1.86E-01 | 6 | -156.937 | 325.874 | 0 | 0.413 |
| -0.624 | NA | NA | + | -4.34E-04 | NA | 1.94E-01 | 7 | -156.504 | 327.008 | 1.133 | 0.235 |
| -0.564 | 1.37E-09 | NA | + | NA | NA | 1.91E-01 | 7 | -156.692 | 327.383 | 1.509 | 0.194 |
| -0.484 | NA | NA | + | NA | -1.93E-02 | 1.87E-01 | 7 | -156.9 | 327.8 | 1.926 | 0.158 |
| -0.588 | NA | NA | + | NA | NA | 2.07E-01 | 6 | -153.712 | 319.425 | 0 | 0.26 |
| -0.71 | NA | NA | + | -5.95E-04 | NA | 2.18E-01 | 7 | -152.844 | 319.687 | 0.262 | 0.228 |
| -0.628 | 1.51E-09 | NA | + | NA | NA | 2.13E-01 | 7 | -153.392 | 320.784 | 1.359 | 0.132 |
| -0.58 | NA | NA | + | -6.53E-04 | -6.25E-02 | 2.18E-01 | 8 | -152.405 | 320.81 | 1.385 | 0.13 |
| -0.476 | NA | NA | + | NA | -4.92E-02 | 2.06E-01 | 7 | -153.439 | 320.878 | 1.453 | 0.126 |
| -0.758 | 1.64E-09 | NA | + | -6.15E-04 | NA | 2.25E-01 | 8 | -152.462 | 320.923 | 1.499 | 0.123 |
| -0.213 | NA | NA | NA | NA | NA | 1.46E-01 | 3 | -142.919 | 291.837 | 0 | 0.322 |
| -0.119 | NA | NA | NA | 4.46E-04 | NA | 1.21E-01 | 4 | -142.064 | 292.129 | 0.291 | 0.278 |
| -0.253 | NA | NA | + | NA | NA | 1.18E-01 | 6 | -140.688 | 293.376 | 1.539 | 0.149 |
| -0.235 | 6.71E-10 | NA | NA | NA | NA | 1.50E-01 | 4 | -142.8 | 293.6 | 1.762 | 0.133 |
| -0.205 | NA | NA | NA | NA | -3.72E-03 | 1.46E-01 | 4 | -142.917 | 293.835 | 1.997 | 0.118 |
| -0.6 | NA | NA | + | NA | NA | 2.06E-01 | 6 | -167.472 | 346.943 | 0 | 0.421 |
| -0.641 | 1.35E-09 | NA | + | NA | NA | 2.14E-01 | 7 | -167.198 | 348.396 | 1.453 | 0.203 |
| -0.67 | NA | NA | + | -3.31E-04 | NA | 2.13E-01 | 7 | -167.243 | 348.486 | 1.542 | 0.195 |
| -0.509 | NA | NA | + | NA | -3.98E-02 | 2.05E-01 | 7 | -167.314 | 348.628 | 1.684 | 0.181 |
| -0.487 | NA | NA | + | NA | NA | 1.74E-01 | 6 | -147.875 | 307.75 | 0 | 0.421 |
| -0.56 | NA | NA | + | -3.27E-04 | NA | 1.80E-01 | 7 | -147.613 | 309.227 | 1.477 | 0.201 |
| -0.519 | 1.16E-09 | NA | + | NA | NA | 1.79E-01 | 7 | -147.639 | 309.279 | 1.529 | 0.196 |
| -0.403 | NA | NA | + | NA | -3.77E-02 | 1.76E-01 | 7 | -147.715 | 309.43 | 1.68 | 0.182 |
| -0.438 | NA | NA | + | NA | NA | 1.68E-01 | 6 | -151.757 | 315.513 | 0 | 0.446 |
| -0.468 | 1.22E-09 | NA | + | NA | NA | 1.73E-01 | 7 | -151.553 | 317.106 | 1.593 | 0.201 |
| -0.481 | NA | NA | + | -1.92E-04 | NA | 1.72E-01 | 7 | -151.672 | 317.344 | 1.831 | 0.179 |
| -0.387 | NA | NA | + | NA | -2.38E-02 | 1.69E-01 | 7 | -151.698 | 317.397 | 1.884 | 0.174 |
| -0.503 | NA | NA | + | NA | NA | 1.81E-01 | 6 | -147.276 | 306.551 | 0 | 0.247 |
| -0.624 | NA | NA | + | -5.39E-04 | NA | 1.93E-01 | 7 | -146.527 | 307.053 | 0.502 | 0.192 |
| -0.349 | NA | NA | + | NA | -7.01E-02 | 1.82E-01 | 7 | -146.667 | 307.333 | 0.782 | 0.167 |
| -0.461 | NA | NA | + | -6.21E-04 | -8.29E-02 | 1.97E-01 | 8 | -145.683 | 307.365 | 0.814 | 0.164 |
| -0.542 | 1.37E-09 | NA | + | NA | NA | 1.88E-01 | 7 | -146.957 | 307.914 | 1.363 | 0.125 |
| -0.674 | 1.52E-09 | NA | + | -5.66E-04 | NA | 2.01E-01 | 8 | -146.131 | 308.261 | 1.71 | 0.105 |
| -0.482 | NA | NA | + | NA | NA | 1.68E-01 | 6 | -162.534 | 337.068 | 0 | 0.45 |
| -0.541 | NA | NA | + | -2.79E-04 | NA | 1.73E-01 | 7 | -162.372 | 338.744 | 1.676 | 0.194 |
| -0.51 | 9.99E-10 | NA | + | NA | NA | 1.73E-01 | 7 | -162.392 | 338.784 | 1.717 | 0.191 |
| -0.481 | NA | NA | + | NA | -2.17E-04 | 1.68E-01 | 7 | -162.534 | 339.068 | 2 | 0.165 |
| -0.762 | NA | NA | + | -6.68E-04 | NA | 2.21E-01 | 7 | -156.012 | 326.024 | 0 | 0.202 |
| -0.64 | NA | NA | + | NA | NA | 2.11E-01 | 6 | -157.134 | 326.268 | 0.244 | 0.179 |
| -0.59 | NA | NA | + | -7.65E-04 | -8.52E-02 | 2.24E-01 | 8 | -155.162 | 326.323 | 0.299 | 0.174 |
| -0.819 | 1.60E-09 | NA | + | -7.07E-04 | NA | 2.30E-01 | 8 | -155.463 | 326.927 | 0.903 | 0.129 |
| -0.491 | NA | NA | + | NA | -6.69E-02 | 2.13E-01 | 7 | -156.603 | 327.207 | 1.183 | 0.112 |
| -0.649 | 1.52E-09 | NA | + | -7.98E-04 | -8.25E-02 | 2.33E-01 | 9 | -154.661 | 327.322 | 1.298 | 0.106 |
| -0.683 | 1.39E-09 | NA | + | NA | NA | 2.19E-01 | 7 | -156.718 | 327.437 | 1.413 | 0.1 |
| -0.47 | NA | NA | + | NA | NA | 1.67E-01 | 6 | -144.379 | 300.758 | 0 | 0.395 |
| -0.563 | NA | NA | + | -4.03E-04 | NA | 1.76E-01 | 7 | -143.963 | 301.926 | 1.167 | 0.22 |
| -0.353 | NA | NA | + | NA | -5.29E-02 | 1.69E-01 | 7 | -144.057 | 302.114 | 1.356 | 0.201 |
| -0.505 | 1.21E-09 | NA | + | NA | NA | 1.73E-01 | 7 | -144.146 | 302.291 | 1.533 | 0.184 |
| -0.302 | NA | NA | + | NA | NA | 1.26E-01 | 6 | -138.269 | 288.538 | 0 | 0.277 |
| -0.248 | NA | NA | NA | NA | NA | 1.59E-01 | 3 | -141.424 | 288.847 | 0.309 | 0.237 |
| -0.17 | NA | NA | NA | 3.81E-04 | NA | 1.38E-01 | 4 | -140.796 | 289.592 | 1.054 | 0.163 |
| -0.326 | 9.23E-10 | NA | + | NA | NA | 1.30E-01 | 7 | -138.138 | 290.277 | 1.739 | 0.116 |
| -0.331 | NA | NA | + | -1.16E-04 | NA | 1.29E-01 | 7 | -138.237 | 290.474 | 1.936 | 0.105 |
| -0.301 | NA | NA | + | NA | -6.18E-04 | 1.26E-01 | 7 | -138.269 | 290.538 | 2 | 0.102 |
| -0.452 | NA | NA | + | NA | NA | 1.59E-01 | 6 | -154.743 | 321.487 | 0 | 0.439 |
| -0.487 | 7.66E-10 | NA | + | NA | NA | 1.68E-01 | 7 | -154.521 | 323.042 | 1.555 | 0.202 |
| -0.515 | NA | NA | + | -2.73E-04 | NA | 1.64E-01 | 7 | -154.572 | 323.144 | 1.657 | 0.192 |
| -0.412 | NA | NA | + | NA | -1.83E-02 | 1.59E-01 | 7 | -154.71 | 323.42 | 1.933 | 0.167 |
| -0.375 | NA | NA | + | NA | NA | 1.50E-01 | 6 | -145.46 | 302.919 | 0 | 0.307 |
| -0.301 | NA | NA | NA | NA | NA | 1.78E-01 | 3 | -148.892 | 303.784 | 0.864 | 0.199 |
| -0.401 | 1.01E-09 | NA | + | NA | NA | 1.54E-01 | 7 | -145.298 | 304.596 | 1.677 | 0.133 |
| -0.236 | NA | NA | NA | 3.67E-04 | NA | 1.61E-01 | 4 | -148.326 | 304.652 | 1.733 | 0.129 |
| -0.411 | NA | NA | + | -1.54E-04 | NA | 1.53E-01 | 7 | -145.406 | 304.812 | 1.892 | 0.119 |
| -0.381 | NA | NA | + | NA | 2.82E-03 | 1.50E-01 | 7 | -145.459 | 304.918 | 1.998 | 0.113 |
| -0.534 | NA | NA | + | NA | NA | 1.88E-01 | 6 | -149.6 | 311.201 | 0 | 0.271 |
| -0.64 | NA | NA | + | -5.27E-04 | NA | 1.97E-01 | 7 | -148.885 | 311.771 | 0.57 | 0.204 |
| -0.407 | NA | NA | + | NA | -5.80E-02 | 1.90E-01 | 7 | -149.203 | 312.406 | 1.206 | 0.149 |
| -0.573 | 1.46E-09 | NA | + | NA | NA | 1.93E-01 | 7 | -149.286 | 312.573 | 1.372 | 0.137 |
| -0.499 | NA | NA | + | -5.97E-04 | -7.09E-02 | 2.00E-01 | 8 | -148.3 | 312.599 | 1.398 | 0.135 |
| -0.681 | 1.49E-09 | NA | + | -5.32E-04 | NA | 2.03E-01 | 8 | -148.556 | 313.111 | 1.91 | 0.104 |
| -0.608 | NA | NA | + | NA | NA | 2.02E-01 | 6 | -167.494 | 346.987 | 0 | 0.378 |
| -0.693 | NA | NA | + | -4.32E-04 | NA | 2.08E-01 | 7 | -167.075 | 348.149 | 1.162 | 0.211 |
| -0.472 | NA | NA | + | NA | -6.17E-02 | 2.04E-01 | 7 | -167.084 | 348.167 | 1.18 | 0.209 |
| -0.654 | 1.65E-09 | NA | + | NA | NA | 2.09E-01 | 7 | -167.123 | 348.245 | 1.258 | 0.201 |
| -0.699 | NA | NA | + | -1.15E-03 | -1.40E-01 | 2.78E-01 | 8 | -157.1 | 330.199 | 0 | 0.609 |
| -0.77 | 1.92E-09 | NA | + | -1.16E-03 | -1.33E-01 | 2.87E-01 | 9 | -156.541 | 331.081 | 0.882 | 0.391 |
| -0.625 | NA | NA | + | NA | NA | 2.08E-01 | 6 | -165.335 | 342.671 | 0 | 0.264 |
| -0.738 | NA | NA | + | -5.61E-04 | NA | 2.16E-01 | 7 | -164.645 | 343.291 | 0.62 | 0.193 |
| -0.479 | NA | NA | + | NA | -6.46E-02 | 2.09E-01 | 7 | -164.876 | 343.751 | 1.08 | 0.154 |
| -0.669 | 1.58E-09 | NA | + | NA | NA | 2.15E-01 | 7 | -164.967 | 343.933 | 1.262 | 0.14 |
| -0.579 | NA | NA | + | -6.41E-04 | -7.77E-02 | 2.19E-01 | 8 | -163.987 | 343.974 | 1.304 | 0.137 |
| -0.793 | 1.74E-09 | NA | + | -5.92E-04 | NA | 2.25E-01 | 8 | -164.198 | 344.396 | 1.725 | 0.111 |
| -0.336 | NA | NA | + | NA | NA | 1.28E-01 | 6 | -142.569 | 297.138 | 0 | 0.376 |
| -0.265 | NA | NA | NA | NA | NA | 1.67E-01 | 3 | -146.346 | 298.693 | 1.555 | 0.173 |
| -0.358 | 7.06E-10 | NA | + | NA | NA | 1.32E-01 | 7 | -142.457 | 298.914 | 1.777 | 0.155 |
| -0.381 | NA | NA | + | -1.78E-04 | NA | 1.33E-01 | 7 | -142.492 | 298.985 | 1.847 | 0.149 |
| -0.286 | NA | NA | + | NA | -2.34E-02 | 1.30E-01 | 7 | -142.511 | 299.022 | 1.884 | 0.147 |
| -0.532 | NA | NA | + | NA | NA | 1.82E-01 | 6 | -165.414 | 342.827 | 0 | 0.428 |
| -0.574 | 1.41E-09 | NA | + | NA | NA | 1.89E-01 | 7 | -165.134 | 344.269 | 1.442 | 0.208 |
| -0.61 | NA | NA | + | -3.62E-04 | NA | 1.88E-01 | 7 | -165.143 | 344.285 | 1.458 | 0.206 |
| -0.518 | NA | NA | + | NA | -6.48E-03 | 1.82E-01 | 7 | -165.41 | 344.82 | 1.992 | 0.158 |
| -0.509 | NA | NA | + | NA | NA | 1.79E-01 | 6 | -165.889 | 343.779 | 0 | 0.439 |
| -0.556 | 1.65E-09 | NA | + | NA | NA | 1.86E-01 | 7 | -165.574 | 345.148 | 1.37 | 0.221 |
| -0.448 | NA | NA | + | NA | -2.68E-02 | 1.79E-01 | 7 | -165.82 | 345.639 | 1.861 | 0.173 |
| -0.538 | NA | NA | + | -1.29E-04 | NA | 1.82E-01 | 7 | -165.857 | 345.713 | 1.935 | 0.167 |
| -0.536 | NA | NA | + | NA | NA | 1.80E-01 | 6 | -156.634 | 325.267 | 0 | 0.432 |
| -0.618 | NA | NA | + | -3.88E-04 | NA | 1.87E-01 | 7 | -156.291 | 326.582 | 1.315 | 0.224 |
| -0.564 | 9.34E-10 | NA | + | NA | NA | 1.86E-01 | 7 | -156.477 | 326.953 | 1.686 | 0.186 |
| -0.541 | NA | NA | + | NA | 2.18E-03 | 1.80E-01 | 7 | -156.633 | 327.267 | 1.999 | 0.159 |
| -0.444 | NA | NA | + | NA | NA | 1.64E-01 | 6 | -150.36 | 312.721 | 0 | 0.378 |
| -0.476 | 1.11E-09 | NA | + | NA | NA | 1.70E-01 | 7 | -150.132 | 314.263 | 1.542 | 0.175 |
| -0.374 | NA | NA | + | NA | -3.19E-02 | 1.65E-01 | 7 | -150.252 | 314.503 | 1.782 | 0.155 |
| -0.484 | NA | NA | + | -1.74E-04 | NA | 1.68E-01 | 7 | -150.29 | 314.579 | 1.858 | 0.149 |
| -0.368 | NA | NA | NA | NA | NA | 2.04E-01 | 3 | -154.329 | 314.659 | 1.938 | 0.143 |
| -0.739 | NA | NA | + | -6.44E-04 | NA | 2.21E-01 | 7 | -152.496 | 318.993 | 0 | 0.195 |
| -0.605 | NA | NA | + | NA | NA | 2.09E-01 | 6 | -153.533 | 319.066 | 0.073 | 0.188 |
| -0.564 | NA | NA | + | -7.25E-04 | -8.61E-02 | 2.24E-01 | 8 | -151.614 | 319.229 | 0.236 | 0.174 |
| -0.446 | NA | NA | + | NA | -7.19E-02 | 2.11E-01 | 7 | -152.916 | 319.832 | 0.839 | 0.128 |
| -0.794 | 1.78E-09 | NA | + | -6.70E-04 | NA | 2.30E-01 | 8 | -152.005 | 320.009 | 1.016 | 0.118 |
| -0.651 | 1.62E-09 | NA | + | NA | NA | 2.17E-01 | 7 | -153.13 | 320.26 | 1.267 | 0.104 |
| -0.625 | 1.55E-09 | NA | + | -7.42E-04 | -8.03E-02 | 2.32E-01 | 9 | -151.241 | 320.482 | 1.489 | 0.093 |
| -0.583 | NA | NA | + | -1.04E-03 | -1.13E-01 | 2.26E-01 | 8 | -142.448 | 300.896 | 0 | 0.47 |
| -0.646 | 1.55E-09 | NA | + | -1.06E-03 | -1.09E-01 | 2.36E-01 | 9 | -141.902 | 301.804 | 0.909 | 0.298 |
| -0.808 | NA | NA | + | -9.21E-04 | NA | 2.23E-01 | 7 | -144.152 | 302.304 | 1.409 | 0.232 |
| -0.446 | NA | NA | + | NA | NA | 1.64E-01 | 6 | -150.813 | 313.626 | 0 | 0.428 |
| -0.479 | 9.53E-10 | NA | + | NA | NA | 1.71E-01 | 7 | -150.592 | 315.183 | 1.558 | 0.197 |
| -0.347 | NA | NA | + | NA | -4.44E-02 | 1.65E-01 | 7 | -150.601 | 315.202 | 1.576 | 0.195 |
| -0.499 | NA | NA | + | -2.35E-04 | NA | 1.69E-01 | 7 | -150.679 | 315.358 | 1.732 | 0.18 |
| -0.419 | NA | NA | + | NA | NA | 1.47E-01 | 6 | -138.71 | 289.42 | 0 | 0.258 |
| -0.549 | NA | NA | + | -5.65E-04 | NA | 1.57E-01 | 7 | -137.829 | 289.658 | 0.239 | 0.229 |
| -0.421 | NA | NA | + | -6.14E-04 | -6.33E-02 | 1.61E-01 | 8 | -137.36 | 290.72 | 1.3 | 0.135 |
| -0.304 | NA | NA | + | NA | -5.22E-02 | 1.49E-01 | 7 | -138.39 | 290.781 | 1.361 | 0.131 |
| -0.6 | 1.13E-09 | NA | + | -6.11E-04 | NA | 1.66E-01 | 8 | -137.434 | 290.867 | 1.448 | 0.125 |
| -0.452 | 9.17E-10 | NA | + | NA | NA | 1.54E-01 | 7 | -138.45 | 290.9 | 1.481 | 0.123 |
| -0.442 | NA | NA | + | NA | NA | 1.71E-01 | 6 | -143.127 | 298.253 | 0 | 0.434 |
| -0.479 | 1.27E-09 | NA | + | NA | NA | 1.76E-01 | 7 | -142.853 | 299.707 | 1.453 | 0.21 |
| -0.505 | NA | NA | + | -2.58E-04 | NA | 1.77E-01 | 7 | -142.962 | 299.924 | 1.671 | 0.188 |
| -0.393 | NA | NA | + | NA | -2.23E-02 | 1.71E-01 | 7 | -143.07 | 300.141 | 1.888 | 0.169 |
| -0.462 | NA | NA | + | NA | NA | 1.65E-01 | 6 | -153.229 | 318.458 | 0 | 0.44 |
| -0.495 | 1.03E-09 | NA | + | NA | NA | 1.71E-01 | 7 | -153.002 | 320.004 | 1.546 | 0.203 |
| -0.523 | NA | NA | + | -2.75E-04 | NA | 1.69E-01 | 7 | -153.059 | 320.118 | 1.66 | 0.192 |
| -0.436 | NA | NA | + | NA | -1.20E-02 | 1.65E-01 | 7 | -153.214 | 320.429 | 1.971 | 0.164 |
| -0.537 | NA | NA | + | NA | NA | 1.75E-01 | 6 | -164.469 | 340.937 | 0 | 0.435 |
| -0.572 | 9.55E-10 | NA | + | NA | NA | 1.83E-01 | 7 | -164.234 | 342.469 | 1.532 | 0.202 |
| -0.6 | NA | NA | + | -3.02E-04 | NA | 1.80E-01 | 7 | -164.276 | 342.551 | 1.614 | 0.194 |
| -0.482 | NA | NA | + | NA | -2.45E-02 | 1.76E-01 | 7 | -164.413 | 342.825 | 1.888 | 0.169 |
| -0.435 | NA | NA | + | NA | NA | 1.60E-01 | 6 | -150.193 | 312.386 | 0 | 0.441 |
| -0.507 | NA | NA | + | -3.07E-04 | NA | 1.66E-01 | 7 | -149.979 | 313.958 | 1.572 | 0.201 |
| -0.465 | 1.09E-09 | NA | + | NA | NA | 1.65E-01 | 7 | -150.014 | 314.028 | 1.642 | 0.194 |
| -0.421 | NA | NA | + | NA | -6.67E-03 | 1.60E-01 | 7 | -150.189 | 314.377 | 1.991 | 0.163 |
| -0.476 | NA | NA | + | NA | NA | 1.71E-01 | 6 | -144.553 | 301.105 | 0 | 0.385 |
| -0.572 | NA | NA | + | -4.37E-04 | NA | 1.79E-01 | 7 | -144.093 | 302.186 | 1.081 | 0.224 |
| -0.358 | NA | NA | + | NA | -5.28E-02 | 1.71E-01 | 7 | -144.213 | 302.425 | 1.32 | 0.199 |
| -0.516 | 1.38E-09 | NA | + | NA | NA | 1.77E-01 | 7 | -144.244 | 302.489 | 1.383 | 0.193 |
| -0.458 | NA | NA | + | NA | NA | 1.71E-01 | 6 | -145.972 | 303.945 | 0 | 0.38 |
| -0.581 | NA | NA | + | -5.36E-04 | NA | 1.82E-01 | 7 | -145.243 | 304.485 | 0.54 | 0.29 |
| -0.491 | 1.23E-09 | NA | + | NA | NA | 1.76E-01 | 7 | -145.759 | 305.517 | 1.573 | 0.173 |
| -0.389 | NA | NA | + | NA | -3.21E-02 | 1.72E-01 | 7 | -145.858 | 305.716 | 1.772 | 0.157 |
| -0.507 | NA | NA | + | NA | NA | 1.77E-01 | 6 | -157.671 | 327.342 | 0 | 0.4 |
| -0.379 | NA | NA | + | NA | -5.71E-02 | 1.79E-01 | 7 | -157.321 | 328.641 | 1.299 | 0.209 |
| -0.547 | 1.44E-09 | NA | + | NA | NA | 1.84E-01 | 7 | -157.389 | 328.777 | 1.436 | 0.195 |
| -0.589 | NA | NA | + | -3.52E-04 | NA | 1.86E-01 | 7 | -157.391 | 328.781 | 1.439 | 0.195 |
| -0.537 | NA | NA | + | NA | NA | 1.84E-01 | 6 | -160.895 | 333.791 | 0 | 0.382 |
| -0.386 | NA | NA | + | NA | -6.73E-02 | 1.85E-01 | 7 | -160.397 | 334.795 | 1.004 | 0.231 |
| -0.578 | 1.26E-09 | NA | + | NA | NA | 1.92E-01 | 7 | -160.555 | 335.109 | 1.318 | 0.198 |
| -0.614 | NA | NA | + | -3.66E-04 | NA | 1.90E-01 | 7 | -160.599 | 335.197 | 1.406 | 0.189 |
| -0.564 | NA | NA | + | NA | NA | 1.90E-01 | 6 | -156.307 | 324.614 | 0 | 0.221 |
| -0.681 | NA | NA | + | -5.75E-04 | NA | 2.00E-01 | 7 | -155.514 | 325.027 | 0.413 | 0.18 |
| -0.508 | NA | NA | + | -6.59E-04 | -8.41E-02 | 2.01E-01 | 8 | -154.695 | 325.39 | 0.776 | 0.15 |
| -0.404 | NA | NA | + | NA | -7.07E-02 | 1.90E-01 | 7 | -155.725 | 325.449 | 0.835 | 0.145 |
| -0.608 | 1.38E-09 | NA | + | NA | NA | 1.99E-01 | 7 | -155.935 | 325.87 | 1.255 | 0.118 |
| -0.737 | 1.53E-09 | NA | + | -6.06E-04 | NA | 2.09E-01 | 8 | -155.054 | 326.108 | 1.493 | 0.105 |
| -0.567 | 1.41E-09 | NA | + | -6.83E-04 | -8.05E-02 | 2.10E-01 | 9 | -154.303 | 326.607 | 1.992 | 0.082 |
| -0.515 | NA | NA | + | NA | NA | 1.82E-01 | 6 | -147.559 | 307.119 | 0 | 0.206 |
| -0.647 | NA | NA | + | -5.86E-04 | NA | 1.95E-01 | 7 | -146.665 | 307.329 | 0.211 | 0.186 |
| -0.467 | NA | NA | + | -6.65E-04 | -8.75E-02 | 1.97E-01 | 8 | -145.747 | 307.495 | 0.376 | 0.171 |
| -0.346 | NA | NA | + | NA | -7.46E-02 | 1.82E-01 | 7 | -146.891 | 307.782 | 0.663 | 0.148 |
| -0.553 | 1.42E-09 | NA | + | NA | NA | 1.88E-01 | 7 | -147.25 | 308.5 | 1.382 | 0.103 |
| -0.695 | 1.56E-09 | NA | + | -6.08E-04 | NA | 2.02E-01 | 8 | -146.286 | 308.572 | 1.453 | 0.1 |
| -0.516 | 1.43E-09 | NA | + | -6.83E-04 | -8.45E-02 | 2.03E-01 | 9 | -145.43 | 308.859 | 1.74 | 0.086 |
| -0.56 | NA | NA | + | NA | NA | 1.86E-01 | 6 | -158.156 | 328.311 | 0 | 0.38 |
| -0.669 | NA | NA | + | -5.22E-04 | NA | 1.94E-01 | 7 | -157.533 | 329.065 | 0.754 | 0.261 |
| -0.597 | 1.21E-09 | NA | + | NA | NA | 1.92E-01 | 7 | -157.871 | 329.742 | 1.43 | 0.186 |
| -0.46 | NA | NA | + | NA | -4.47E-02 | 1.87E-01 | 7 | -157.943 | 329.885 | 1.574 | 0.173 |
| -0.672 | NA | NA | + | -6.37E-04 | NA | 2.02E-01 | 7 | -144.611 | 303.221 | 0 | 0.243 |
| -0.537 | NA | NA | + | NA | NA | 1.90E-01 | 6 | -145.711 | 303.421 | 0.2 | 0.22 |
| -0.525 | NA | NA | + | -7.06E-04 | -7.50E-02 | 2.06E-01 | 8 | -143.953 | 303.907 | 0.686 | 0.172 |
| -0.72 | 1.46E-09 | NA | + | -6.61E-04 | NA | 2.10E-01 | 8 | -144.22 | 304.441 | 1.22 | 0.132 |
| -0.408 | NA | NA | + | NA | -6.00E-02 | 1.92E-01 | 7 | -145.288 | 304.576 | 1.355 | 0.123 |
| -0.576 | 1.30E-09 | NA | + | NA | NA | 1.97E-01 | 7 | -145.406 | 304.812 | 1.591 | 0.11 |
| -0.497 | NA | NA | + | NA | NA | 1.71E-01 | 6 | -153.181 | 318.363 | 0 | 0.269 |
| -0.616 | NA | NA | + | -5.31E-04 | NA | 1.83E-01 | 7 | -152.485 | 318.97 | 0.607 | 0.199 |
| -0.364 | NA | NA | + | NA | -6.07E-02 | 1.73E-01 | 7 | -152.742 | 319.484 | 1.122 | 0.154 |
| -0.534 | 1.14E-09 | NA | + | NA | NA | 1.79E-01 | 7 | -152.887 | 319.775 | 1.412 | 0.133 |
| -0.474 | NA | NA | + | -5.90E-04 | -7.09E-02 | 1.86E-01 | 8 | -151.889 | 319.778 | 1.416 | 0.133 |
| -0.673 | 1.39E-09 | NA | + | -5.85E-04 | NA | 1.93E-01 | 8 | -152.053 | 320.106 | 1.743 | 0.113 |
| -0.613 | NA | NA | + | -1.03E-03 | -1.20E-01 | 2.43E-01 | 8 | -153.621 | 323.242 | 0 | 0.485 |
| -0.678 | 1.93E-09 | NA | + | -1.05E-03 | -1.15E-01 | 2.50E-01 | 9 | -153.109 | 324.219 | 0.977 | 0.298 |
| -0.852 | NA | NA | + | -8.94E-04 | NA | 2.40E-01 | 7 | -155.425 | 324.849 | 1.607 | 0.217 |
| -0.605 | NA | NA | + | NA | NA | 2.01E-01 | 6 | -158.672 | 329.345 | 0 | 0.206 |
| -0.736 | NA | NA | + | -6.42E-04 | NA | 2.11E-01 | 7 | -157.692 | 329.384 | 0.039 | 0.202 |
| -0.569 | NA | NA | + | -7.22E-04 | -8.14E-02 | 2.12E-01 | 8 | -156.945 | 329.891 | 0.546 | 0.157 |
| -0.456 | NA | NA | + | NA | -6.65E-02 | 2.01E-01 | 7 | -158.17 | 330.34 | 0.995 | 0.125 |
| -0.79 | 1.88E-09 | NA | + | -6.68E-04 | NA | 2.19E-01 | 8 | -157.237 | 330.475 | 1.13 | 0.117 |
| -0.65 | 1.71E-09 | NA | + | NA | NA | 2.08E-01 | 7 | -158.301 | 330.602 | 1.257 | 0.11 |
| -0.628 | 1.70E-09 | NA | + | -7.41E-04 | -7.68E-02 | 2.19E-01 | 9 | -156.572 | 331.144 | 1.799 | 0.084 |
| -0.588 | NA | NA | + | NA | NA | 1.98E-01 | 6 | -156.452 | 324.905 | 0 | 0.258 |
| -0.706 | NA | NA | + | -5.66E-04 | NA | 2.08E-01 | 7 | -155.676 | 325.351 | 0.446 | 0.206 |
| -0.445 | NA | NA | + | NA | -6.30E-02 | 1.98E-01 | 7 | -156.008 | 326.017 | 1.112 | 0.148 |
| -0.548 | NA | NA | + | -6.38E-04 | -7.62E-02 | 2.10E-01 | 8 | -155.032 | 326.063 | 1.159 | 0.144 |
| -0.627 | 1.37E-09 | NA | + | NA | NA | 2.05E-01 | 7 | -156.136 | 326.271 | 1.366 | 0.13 |
| -0.757 | 1.55E-09 | NA | + | -5.99E-04 | NA | 2.17E-01 | 8 | -155.266 | 326.532 | 1.627 | 0.114 |
| -0.457 | NA | NA | + | NA | NA | 1.70E-01 | 6 | -152.549 | 317.099 | 0 | 0.431 |
| -0.53 | NA | NA | + | -3.28E-04 | NA | 1.77E-01 | 7 | -152.301 | 318.603 | 1.504 | 0.203 |
| -0.49 | 1.03E-09 | NA | + | NA | NA | 1.77E-01 | 7 | -152.312 | 318.623 | 1.524 | 0.201 |
| -0.412 | NA | NA | + | NA | -2.04E-02 | 1.71E-01 | 7 | -152.508 | 319.016 | 1.917 | 0.165 |
| -0.523 | NA | NA | + | NA | NA | 1.84E-01 | 6 | -151.737 | 315.475 | 0 | 0.329 |
| -0.645 | NA | NA | + | -5.41E-04 | NA | 1.95E-01 | 7 | -151.038 | 316.075 | 0.6 | 0.244 |
| -0.559 | 1.29E-09 | NA | + | NA | NA | 1.90E-01 | 7 | -151.496 | 316.991 | 1.517 | 0.154 |
| -0.422 | NA | NA | + | NA | -4.48E-02 | 1.85E-01 | 7 | -151.521 | 317.043 | 1.568 | 0.15 |
| -0.532 | NA | NA | + | -5.82E-04 | -5.46E-02 | 1.97E-01 | 8 | -150.717 | 317.435 | 1.96 | 0.123 |
| -0.55 | NA | NA | + | NA | NA | 1.92E-01 | 6 | -155.248 | 322.496 | 0 | 0.38 |
| -0.644 | NA | NA | + | -4.41E-04 | NA | 2.01E-01 | 7 | -154.789 | 323.578 | 1.082 | 0.221 |
| -0.422 | NA | NA | + | NA | -5.72E-02 | 1.92E-01 | 7 | -154.863 | 323.726 | 1.23 | 0.205 |
| -0.591 | 1.23E-09 | NA | + | NA | NA | 2.00E-01 | 7 | -154.921 | 323.842 | 1.346 | 0.194 |
| -0.569 | NA | NA | + | NA | NA | 1.93E-01 | 6 | -159.722 | 331.444 | 0 | 0.4 |
| -0.439 | NA | NA | + | NA | -5.59E-02 | 1.92E-01 | 7 | -159.376 | 332.753 | 1.309 | 0.208 |
| -0.646 | NA | NA | + | -3.64E-04 | NA | 2.00E-01 | 7 | -159.417 | 332.833 | 1.389 | 0.2 |
| -0.608 | 1.42E-09 | NA | + | NA | NA | 2.00E-01 | 7 | -159.453 | 332.905 | 1.461 | 0.193 |
| -0.545 | NA | NA | + | NA | NA | 1.88E-01 | 6 | -152.167 | 316.335 | 0 | 0.304 |
| -0.384 | NA | NA | + | NA | -7.10E-02 | 1.88E-01 | 7 | -151.577 | 317.154 | 0.819 | 0.202 |
| -0.649 | NA | NA | + | -4.68E-04 | NA | 1.98E-01 | 7 | -151.632 | 317.265 | 0.93 | 0.191 |
| -0.481 | NA | NA | + | -5.46E-04 | -8.19E-02 | 2.00E-01 | 8 | -150.856 | 317.712 | 1.377 | 0.153 |
| -0.582 | 1.29E-09 | NA | + | NA | NA | 1.95E-01 | 7 | -151.864 | 317.729 | 1.394 | 0.151 |
| -0.703 | NA | NA | + | -6.59E-04 | NA | 2.10E-01 | 7 | -150.126 | 314.252 | 0 | 0.215 |
| -0.576 | NA | NA | + | NA | NA | 2.00E-01 | 6 | -151.286 | 314.573 | 0.321 | 0.183 |
| -0.552 | NA | NA | + | -7.29E-04 | -7.67E-02 | 2.13E-01 | 8 | -149.424 | 314.848 | 0.596 | 0.16 |
| -0.765 | 1.44E-09 | NA | + | -7.20E-04 | NA | 2.22E-01 | 8 | -149.557 | 315.114 | 0.862 | 0.14 |
| -0.443 | NA | NA | + | NA | -6.17E-02 | 2.02E-01 | 7 | -150.831 | 315.662 | 1.411 | 0.106 |
| -0.617 | 1.37E-09 | NA | + | -7.85E-04 | -7.37E-02 | 2.25E-01 | 9 | -148.906 | 315.811 | 1.559 | 0.099 |
| -0.616 | 1.15E-09 | NA | + | NA | NA | 2.09E-01 | 7 | -150.927 | 315.854 | 1.603 | 0.097 |
| -0.64 | NA | NA | + | NA | NA | 2.11E-01 | 6 | -169.309 | 350.618 | 0 | 0.38 |
| -0.745 | NA | NA | + | -5.19E-04 | NA | 2.20E-01 | 7 | -168.744 | 351.487 | 0.869 | 0.246 |
| -0.687 | 1.78E-09 | NA | + | NA | NA | 2.18E-01 | 7 | -168.963 | 351.926 | 1.307 | 0.197 |
| -0.533 | NA | NA | + | NA | -4.80E-02 | 2.11E-01 | 7 | -169.073 | 352.146 | 1.527 | 0.177 |
| -0.437 | NA | NA | + | NA | NA | 1.64E-01 | 6 | -139.95 | 291.901 | 0 | 0.392 |
| -0.544 | NA | NA | + | -4.64E-04 | NA | 1.72E-01 | 7 | -139.38 | 292.76 | 0.859 | 0.255 |
| -0.349 | NA | NA | + | NA | -4.10E-02 | 1.66E-01 | 7 | -139.747 | 293.493 | 1.592 | 0.177 |
| -0.467 | 1.10E-09 | NA | + | NA | NA | 1.68E-01 | 7 | -139.752 | 293.504 | 1.603 | 0.176 |
| -0.58 | NA | NA | + | NA | NA | 1.95E-01 | 6 | -151.67 | 315.339 | 0 | 0.25 |
| -0.705 | NA | NA | + | -5.99E-04 | NA | 2.04E-01 | 7 | -150.768 | 315.537 | 0.197 | 0.227 |
| -0.571 | NA | NA | + | -6.55E-04 | -6.50E-02 | 2.07E-01 | 8 | -150.289 | 316.579 | 1.24 | 0.135 |
| -0.758 | 1.59E-09 | NA | + | -6.36E-04 | NA | 2.13E-01 | 8 | -150.309 | 316.618 | 1.279 | 0.132 |
| -0.619 | 1.38E-09 | NA | + | NA | NA | 2.02E-01 | 7 | -151.323 | 316.645 | 1.306 | 0.13 |
| -0.462 | NA | NA | + | NA | -5.26E-02 | 1.97E-01 | 7 | -151.356 | 316.712 | 1.372 | 0.126 |
| -0.281 | NA | NA | NA | NA | NA | 1.72E-01 | 3 | -147.069 | 300.139 | 0 | 0.253 |
| -0.35 | NA | NA | + | NA | NA | 1.46E-01 | 6 | -144.083 | 300.167 | 0.028 | 0.25 |
| -0.21 | NA | NA | NA | 3.93E-04 | NA | 1.54E-01 | 4 | -146.467 | 300.934 | 0.795 | 0.17 |
| -0.306 | 9.03E-10 | NA | NA | NA | NA | 1.76E-01 | 4 | -146.909 | 301.818 | 1.68 | 0.109 |
| -0.208 | NA | NA | NA | NA | -3.71E-02 | 1.72E-01 | 4 | -146.918 | 301.835 | 1.697 | 0.109 |
| -0.375 | 8.96E-10 | NA | + | NA | NA | 1.50E-01 | 7 | -143.92 | 301.841 | 1.702 | 0.108 |
| -0.416 | NA | NA | + | NA | NA | 1.54E-01 | 6 | -147.332 | 306.664 | 0 | 0.445 |
| -0.487 | NA | NA | + | -3.15E-04 | NA | 1.60E-01 | 7 | -147.098 | 308.195 | 1.531 | 0.207 |
| -0.439 | 7.96E-10 | NA | + | NA | NA | 1.58E-01 | 7 | -147.212 | 308.424 | 1.76 | 0.184 |
| -0.422 | NA | NA | + | NA | 2.94E-03 | 1.54E-01 | 7 | -147.331 | 308.662 | 1.998 | 0.164 |
| -0.585 | NA | NA | + | NA | NA | 2.01E-01 | 6 | -158.8 | 329.599 | 0 | 0.379 |
| -0.69 | NA | NA | + | -5.04E-04 | NA | 2.09E-01 | 7 | -158.224 | 330.448 | 0.848 | 0.248 |
| -0.627 | 1.55E-09 | NA | + | NA | NA | 2.08E-01 | 7 | -158.462 | 330.924 | 1.324 | 0.195 |
| -0.479 | NA | NA | + | NA | -4.75E-02 | 2.02E-01 | 7 | -158.553 | 331.106 | 1.506 | 0.178 |
| -0.424 | NA | NA | + | NA | NA | 1.56E-01 | 6 | -159.829 | 331.658 | 0 | 0.39 |
| -0.453 | 1.07E-09 | NA | + | NA | NA | 1.61E-01 | 7 | -159.685 | 333.369 | 1.712 | 0.166 |
| -0.465 | NA | NA | + | -1.77E-04 | NA | 1.60E-01 | 7 | -159.765 | 333.53 | 1.872 | 0.153 |
| -0.351 | NA | NA | NA | NA | NA | 1.95E-01 | 3 | -163.793 | 333.585 | 1.927 | 0.149 |
| -0.434 | NA | NA | + | NA | 4.79E-03 | 1.56E-01 | 7 | -159.827 | 333.654 | 1.996 | 0.144 |
| -0.481 | NA | NA | + | NA | NA | 1.73E-01 | 6 | -155.882 | 323.763 | 0 | 0.441 |
| -0.516 | 1.31E-09 | NA | + | NA | NA | 1.79E-01 | 7 | -155.623 | 325.247 | 1.484 | 0.21 |
| -0.532 | NA | NA | + | -2.26E-04 | NA | 1.78E-01 | 7 | -155.769 | 325.538 | 1.775 | 0.182 |
| -0.446 | NA | NA | + | NA | -1.62E-02 | 1.74E-01 | 7 | -155.855 | 325.711 | 1.948 | 0.167 |
| -0.102 | NA | NA | NA | 6.00E-04 | NA | 1.23E-01 | 4 | -157.066 | 322.131 | 0 | 0.279 |
| -0.228 | NA | NA | NA | NA | NA | 1.56E-01 | 3 | -158.27 | 322.541 | 0.41 | 0.228 |
| 0.246 | NA | NA | NA | 8.26E-04 | NA | NA | 3 | -158.737 | 323.475 | 1.344 | 0.143 |
| -0.279 | NA | NA | + | NA | NA | 1.22E-01 | 6 | -155.886 | 323.773 | 1.642 | 0.123 |
| -0.176 | NA | NA | NA | 6.49E-04 | 4.07E-02 | 1.21E-01 | 5 | -156.921 | 323.842 | 1.711 | 0.119 |
| -0.122 | 5.45E-10 | NA | NA | 5.87E-04 | NA | 1.26E-01 | 5 | -157.011 | 324.022 | 1.891 | 0.109 |
| -0.573 | NA | NA | + | NA | NA | 1.87E-01 | 6 | -153.492 | 318.985 | 0 | 0.261 |
| -0.713 | NA | NA | + | -6.35E-04 | NA | 2.00E-01 | 7 | -152.52 | 319.039 | 0.054 | 0.254 |
| -0.759 | 1.30E-09 | NA | + | -6.63E-04 | NA | 2.07E-01 | 8 | -152.211 | 320.421 | 1.436 | 0.127 |
| -0.601 | NA | NA | + | -6.72E-04 | -5.23E-02 | 2.01E-01 | 8 | -152.233 | 320.466 | 1.481 | 0.124 |
| -0.607 | 1.11E-09 | NA | + | NA | NA | 1.93E-01 | 7 | -153.268 | 320.536 | 1.551 | 0.12 |
| -0.478 | NA | NA | + | NA | -4.14E-02 | 1.87E-01 | 7 | -153.313 | 320.626 | 1.641 | 0.115 |
| -0.412 | NA | NA | + | NA | NA | 1.58E-01 | 6 | -141.254 | 294.507 | 0 | 0.437 |
| -0.445 | 8.99E-10 | NA | + | NA | NA | 1.65E-01 | 7 | -141.005 | 296.011 | 1.504 | 0.206 |
| -0.465 | NA | NA | + | -2.25E-04 | NA | 1.62E-01 | 7 | -141.126 | 296.253 | 1.746 | 0.183 |
| -0.355 | NA | NA | + | NA | -2.61E-02 | 1.59E-01 | 7 | -141.174 | 296.347 | 1.84 | 0.174 |
| -0.364 | NA | NA | + | NA | NA | 1.44E-01 | 6 | -153.852 | 319.704 | 0 | 0.238 |
| -0.316 | NA | NA | NA | NA | NA | 1.79E-01 | 3 | -156.965 | 319.929 | 0.225 | 0.213 |
| -0.218 | NA | NA | NA | 4.77E-04 | NA | 1.54E-01 | 4 | -156.156 | 320.311 | 0.607 | 0.176 |
| -0.391 | 8.25E-10 | NA | + | NA | NA | 1.50E-01 | 7 | -153.696 | 321.392 | 1.688 | 0.103 |
| -0.346 | 9.00E-10 | NA | NA | NA | NA | 1.85E-01 | 4 | -156.786 | 321.572 | 1.868 | 0.094 |
| -0.383 | NA | NA | + | NA | 9.33E-03 | 1.44E-01 | 7 | -153.844 | 321.688 | 1.984 | 0.088 |
| -0.357 | NA | NA | + | 2.84E-05 | NA | 1.44E-01 | 7 | -153.85 | 321.701 | 1.997 | 0.088 |
| -0.465 | NA | NA | + | NA | NA | 1.74E-01 | 6 | -151.103 | 314.206 | 0 | 0.421 |
| -0.499 | 1.12E-09 | NA | + | NA | NA | 1.80E-01 | 7 | -150.859 | 315.718 | 1.512 | 0.198 |
| -0.534 | NA | NA | + | -3.14E-04 | NA | 1.80E-01 | 7 | -150.867 | 315.735 | 1.529 | 0.196 |
| -0.375 | NA | NA | + | NA | -4.01E-02 | 1.73E-01 | 7 | -150.927 | 315.853 | 1.647 | 0.185 |
| -0.497 | NA | NA | + | NA | NA | 1.72E-01 | 6 | -173.617 | 359.234 | 0 | 0.456 |
| -0.532 | 1.16E-09 | NA | + | NA | NA | 1.79E-01 | 7 | -173.416 | 360.833 | 1.599 | 0.205 |
| -0.527 | NA | NA | + | NA | 1.35E-02 | 1.72E-01 | 7 | -173.602 | 361.203 | 1.97 | 0.17 |
| -0.508 | NA | NA | + | -5.20E-05 | NA | 1.73E-01 | 7 | -173.612 | 361.224 | 1.99 | 0.169 |
| -0.471 | NA | NA | + | NA | NA | 1.67E-01 | 6 | -147.339 | 306.677 | 0 | 0.384 |
| -0.587 | NA | NA | + | -5.21E-04 | NA | 1.77E-01 | 7 | -146.653 | 307.305 | 0.628 | 0.281 |
| -0.5 | 9.59E-10 | NA | + | NA | NA | 1.72E-01 | 7 | -147.16 | 308.321 | 1.643 | 0.169 |
| -0.385 | NA | NA | + | NA | -3.90E-02 | 1.68E-01 | 7 | -147.173 | 308.346 | 1.669 | 0.167 |
| -0.574 | NA | NA | + | -8.73E-04 | -1.25E-01 | 2.45E-01 | 8 | -159.269 | 334.538 | 0 | 0.459 |
| -0.655 | 1.75E-09 | NA | + | -9.23E-04 | -1.21E-01 | 2.58E-01 | 9 | -158.532 | 335.063 | 0.525 | 0.353 |
| -0.467 | NA | NA | + | NA | -1.02E-01 | 2.32E-01 | 7 | -161.167 | 336.334 | 1.795 | 0.187 |
| -0.396 | NA | NA | + | NA | NA | 1.52E-01 | 6 | -150.547 | 313.095 | 0 | 0.318 |
| -0.328 | NA | NA | NA | NA | NA | 1.87E-01 | 3 | -154.184 | 314.368 | 1.273 | 0.168 |
| -0.233 | NA | NA | NA | 4.89E-04 | NA | 1.62E-01 | 4 | -153.355 | 314.709 | 1.614 | 0.142 |
| -0.426 | 9.96E-10 | NA | + | NA | NA | 1.57E-01 | 7 | -150.381 | 314.762 | 1.667 | 0.138 |
| -0.376 | NA | NA | + | NA | -9.26E-03 | 1.52E-01 | 7 | -150.539 | 315.078 | 1.983 | 0.118 |
| -0.394 | NA | NA | + | 1.04E-05 | NA | 1.51E-01 | 7 | -150.547 | 315.094 | 2 | 0.117 |
| -0.366 | NA | NA | + | NA | NA | 1.42E-01 | 6 | -136.307 | 284.614 | 0 | 0.422 |
| -0.456 | NA | NA | + | -3.77E-04 | NA | 1.50E-01 | 7 | -135.943 | 285.887 | 1.273 | 0.223 |
| -0.393 | 9.34E-10 | NA | + | NA | NA | 1.46E-01 | 7 | -136.141 | 286.282 | 1.668 | 0.183 |
| -0.301 | NA | NA | + | NA | -2.96E-02 | 1.43E-01 | 7 | -136.208 | 286.415 | 1.802 | 0.171 |
| -0.532 | NA | NA | + | -7.62E-04 | -1.03E-01 | 2.20E-01 | 8 | -155.531 | 327.063 | 0 | 0.218 |
| -0.738 | NA | NA | + | -6.51E-04 | NA | 2.19E-01 | 7 | -156.835 | 327.669 | 0.607 | 0.161 |
| -0.614 | NA | NA | + | NA | NA | 2.10E-01 | 6 | -157.89 | 327.781 | 0.718 | 0.152 |
| -0.423 | NA | NA | + | NA | -8.64E-02 | 2.09E-01 | 7 | -156.964 | 327.927 | 0.865 | 0.141 |
| -0.599 | 1.57E-09 | NA | + | -8.07E-04 | -9.83E-02 | 2.30E-01 | 9 | -155.028 | 328.056 | 0.993 | 0.133 |
| -0.802 | 1.74E-09 | NA | + | -7.07E-04 | NA | 2.30E-01 | 8 | -156.221 | 328.442 | 1.379 | 0.109 |
| -0.66 | 1.47E-09 | NA | + | NA | NA | 2.19E-01 | 7 | -157.456 | 328.912 | 1.85 | 0.086 |
| -0.451 | NA | NA | + | NA | NA | 1.68E-01 | 6 | -143.431 | 298.862 | 0 | 0.394 |
| -0.555 | NA | NA | + | -4.81E-04 | NA | 1.78E-01 | 7 | -142.83 | 299.659 | 0.797 | 0.264 |
| -0.48 | 9.90E-10 | NA | + | NA | NA | 1.74E-01 | 7 | -143.236 | 300.473 | 1.611 | 0.176 |
| -0.377 | NA | NA | + | NA | -3.45E-02 | 1.69E-01 | 7 | -143.298 | 300.597 | 1.735 | 0.165 |
| -0.585 | NA | NA | + | NA | NA | 2.06E-01 | 6 | -156.978 | 325.957 | 0 | 0.221 |
| -0.702 | NA | NA | + | -5.57E-04 | NA | 2.16E-01 | 7 | -156.209 | 326.418 | 0.461 | 0.175 |
| -0.533 | NA | NA | + | -6.50E-04 | -8.40E-02 | 2.17E-01 | 8 | -155.41 | 326.82 | 0.863 | 0.143 |
| -0.43 | NA | NA | + | NA | -6.85E-02 | 2.06E-01 | 7 | -156.439 | 326.879 | 0.922 | 0.139 |
| -0.63 | 1.51E-09 | NA | + | NA | NA | 2.14E-01 | 7 | -156.552 | 327.104 | 1.147 | 0.124 |
| -0.763 | 1.72E-09 | NA | + | -6.01E-04 | NA | 2.26E-01 | 8 | -155.656 | 327.313 | 1.356 | 0.112 |
| -0.597 | 1.60E-09 | NA | + | -6.87E-04 | -8.00E-02 | 2.26E-01 | 9 | -154.93 | 327.859 | 1.903 | 0.085 |
| -0.643 | NA | NA | + | NA | NA | 2.13E-01 | 6 | -169.337 | 350.675 | 0 | 0.395 |
| -0.693 | 1.88E-09 | NA | + | NA | NA | 2.20E-01 | 7 | -168.948 | 351.896 | 1.222 | 0.215 |
| -0.522 | NA | NA | + | NA | -5.38E-02 | 2.14E-01 | 7 | -169.024 | 352.047 | 1.373 | 0.199 |
| -0.716 | NA | NA | + | -3.58E-04 | NA | 2.19E-01 | 7 | -169.064 | 352.128 | 1.454 | 0.191 |
| -0.519 | NA | NA | + | NA | NA | 1.85E-01 | 6 | -155.191 | 322.382 | 0 | 0.417 |
| -0.604 | NA | NA | + | -3.77E-04 | NA | 1.93E-01 | 7 | -154.863 | 323.726 | 1.343 | 0.213 |
| -0.557 | 1.37E-09 | NA | + | NA | NA | 1.91E-01 | 7 | -154.932 | 323.863 | 1.481 | 0.199 |
| -0.447 | NA | NA | + | NA | -3.16E-02 | 1.85E-01 | 7 | -155.086 | 324.172 | 1.789 | 0.171 |
| -0.801 | NA | NA | + | -7.74E-04 | NA | 2.35E-01 | 7 | -150.279 | 314.558 | 0 | 0.278 |
| -0.625 | NA | NA | + | -8.60E-04 | -8.65E-02 | 2.36E-01 | 8 | -149.374 | 314.749 | 0.19 | 0.252 |
| -0.859 | 1.58E-09 | NA | + | -8.15E-04 | NA | 2.46E-01 | 8 | -149.734 | 315.469 | 0.91 | 0.176 |
| -0.686 | 1.47E-09 | NA | + | -8.95E-04 | -8.29E-02 | 2.46E-01 | 9 | -148.9 | 315.799 | 1.241 | 0.149 |
| -0.66 | NA | NA | + | NA | NA | 2.24E-01 | 6 | -151.931 | 315.862 | 1.304 | 0.145 |
| -0.602 | NA | NA | + | NA | NA | 2.04E-01 | 6 | -162.337 | 336.674 | 0 | 0.265 |
| -0.726 | NA | NA | + | -5.90E-04 | NA | 2.13E-01 | 7 | -161.53 | 337.061 | 0.387 | 0.218 |
| -0.645 | 1.51E-09 | NA | + | NA | NA | 2.12E-01 | 7 | -161.993 | 337.985 | 1.311 | 0.137 |
| -0.785 | 1.75E-09 | NA | + | -6.32E-04 | NA | 2.22E-01 | 8 | -161.071 | 338.141 | 1.468 | 0.127 |
| -0.596 | NA | NA | + | -6.65E-04 | -6.51E-02 | 2.14E-01 | 8 | -161.073 | 338.146 | 1.472 | 0.127 |
| -0.493 | NA | NA | + | NA | -4.89E-02 | 2.04E-01 | 7 | -162.075 | 338.15 | 1.476 | 0.126 |
| -0.406 | NA | NA | + | NA | NA | 1.47E-01 | 6 | -149.436 | 310.872 | 0 | 0.447 |
| -0.438 | 1.03E-09 | NA | + | NA | NA | 1.52E-01 | 7 | -149.242 | 312.485 | 1.612 | 0.2 |
| -0.452 | NA | NA | + | -1.88E-04 | NA | 1.51E-01 | 7 | -149.356 | 312.711 | 1.839 | 0.178 |
| -0.354 | NA | NA | + | NA | -2.40E-02 | 1.48E-01 | 7 | -149.376 | 312.751 | 1.879 | 0.175 |
| -0.399 | NA | NA | + | NA | NA | 1.59E-01 | 6 | -140.786 | 293.572 | 0 | 0.377 |
| -0.428 | 1.02E-09 | NA | + | NA | NA | 1.64E-01 | 7 | -140.568 | 295.136 | 1.564 | 0.172 |
| -0.452 | NA | NA | + | -2.41E-04 | NA | 1.63E-01 | 7 | -140.641 | 295.282 | 1.71 | 0.16 |
| -0.347 | NA | NA | NA | NA | NA | 1.93E-01 | 3 | -144.731 | 295.461 | 1.89 | 0.146 |
| -0.361 | NA | NA | + | NA | -1.88E-02 | 1.61E-01 | 7 | -140.746 | 295.493 | 1.921 | 0.144 |
| -0.256 | NA | NA | NA | NA | NA | 1.62E-01 | 3 | -143.294 | 292.587 | 0 | 0.26 |
| -0.325 | NA | NA | + | NA | NA | 1.37E-01 | 6 | -140.419 | 292.837 | 0.25 | 0.229 |
| -0.177 | NA | NA | NA | 4.38E-04 | NA | 1.42E-01 | 4 | -142.511 | 293.022 | 0.434 | 0.209 |
| -0.279 | 7.24E-10 | NA | NA | NA | NA | 1.66E-01 | 4 | -143.174 | 294.349 | 1.762 | 0.108 |
| -0.224 | NA | NA | NA | NA | -1.70E-02 | 1.63E-01 | 4 | -143.261 | 294.523 | 1.936 | 0.099 |
| -0.348 | 7.52E-10 | NA | + | NA | NA | 1.41E-01 | 7 | -140.286 | 294.571 | 1.984 | 0.096 |
| -0.34 | NA | NA | + | NA | NA | 1.36E-01 | 6 | -149.576 | 311.153 | 0 | 0.245 |
| -0.277 | NA | NA | NA | NA | NA | 1.73E-01 | 3 | -152.739 | 311.477 | 0.324 | 0.208 |
| -0.193 | NA | NA | NA | 4.63E-04 | NA | 1.50E-01 | 4 | -151.964 | 311.927 | 0.774 | 0.166 |
| -0.365 | 8.34E-10 | NA | + | NA | NA | 1.41E-01 | 7 | -149.433 | 312.866 | 1.713 | 0.104 |
| -0.397 | NA | NA | + | NA | 2.69E-02 | 1.34E-01 | 7 | -149.508 | 313.016 | 1.863 | 0.096 |
| -0.307 | 9.42E-10 | NA | NA | NA | NA | 1.78E-01 | 4 | -152.562 | 313.124 | 1.971 | 0.091 |
| -0.336 | NA | NA | + | 1.54E-05 | NA | 1.36E-01 | 7 | -149.576 | 313.152 | 1.999 | 0.09 |
| -0.554 | NA | NA | + | NA | NA | 1.88E-01 | 6 | -147.366 | 306.731 | 0 | 0.208 |
| -0.673 | NA | NA | + | -5.56E-04 | NA | 1.99E-01 | 7 | -146.542 | 307.083 | 0.352 | 0.175 |
| -0.495 | NA | NA | + | -6.34E-04 | -8.54E-02 | 2.00E-01 | 8 | -145.646 | 307.292 | 0.561 | 0.157 |
| -0.388 | NA | NA | + | NA | -7.29E-02 | 1.88E-01 | 7 | -146.709 | 307.419 | 0.687 | 0.148 |
| -0.596 | 1.29E-09 | NA | + | NA | NA | 1.96E-01 | 7 | -146.985 | 307.971 | 1.239 | 0.112 |
| -0.734 | 1.53E-09 | NA | + | -6.08E-04 | NA | 2.09E-01 | 8 | -146.007 | 308.014 | 1.282 | 0.11 |
| -0.559 | 1.41E-09 | NA | + | -6.78E-04 | -8.14E-02 | 2.10E-01 | 9 | -145.192 | 308.384 | 1.653 | 0.091 |
| -0.697 | NA | NA | + | -6.48E-04 | NA | 1.99E-01 | 7 | -154.325 | 322.65 | 0 | 0.236 |
| -0.556 | NA | NA | + | NA | NA | 1.88E-01 | 6 | -155.331 | 322.663 | 0.012 | 0.235 |
| -0.547 | NA | NA | + | -7.16E-04 | -7.21E-02 | 2.00E-01 | 8 | -153.731 | 323.463 | 0.812 | 0.157 |
| -0.746 | 1.58E-09 | NA | + | -6.76E-04 | NA | 2.06E-01 | 8 | -153.939 | 323.878 | 1.227 | 0.128 |
| -0.424 | NA | NA | + | NA | -5.81E-02 | 1.88E-01 | 7 | -154.945 | 323.89 | 1.239 | 0.127 |
| -0.594 | 1.39E-09 | NA | + | NA | NA | 1.94E-01 | 7 | -155.035 | 324.071 | 1.42 | 0.116 |
| -0.438 | NA | NA | + | NA | NA | 1.63E-01 | 6 | -140.847 | 293.695 | 0 | 0.41 |
| -0.54 | NA | NA | + | -4.46E-04 | NA | 1.73E-01 | 7 | -140.349 | 294.698 | 1.003 | 0.249 |
| -0.466 | 8.98E-10 | NA | + | NA | NA | 1.69E-01 | 7 | -140.666 | 295.332 | 1.638 | 0.181 |
| -0.39 | NA | NA | + | NA | -2.29E-02 | 1.65E-01 | 7 | -140.789 | 295.578 | 1.883 | 0.16 |
| -0.478 | NA | NA | + | NA | NA | 1.73E-01 | 6 | -145.373 | 302.746 | 0 | 0.244 |
| -0.608 | NA | NA | + | -5.94E-04 | NA | 1.84E-01 | 7 | -144.444 | 302.888 | 0.142 | 0.227 |
| -0.469 | NA | NA | + | -6.55E-04 | -7.00E-02 | 1.88E-01 | 8 | -143.872 | 303.744 | 0.998 | 0.148 |
| -0.354 | NA | NA | + | NA | -5.73E-02 | 1.75E-01 | 7 | -144.987 | 303.975 | 1.229 | 0.132 |
| -0.659 | 1.38E-09 | NA | + | -6.29E-04 | NA | 1.93E-01 | 8 | -144.027 | 304.054 | 1.309 | 0.127 |
| -0.515 | 1.19E-09 | NA | + | NA | NA | 1.79E-01 | 7 | -145.064 | 304.129 | 1.383 | 0.122 |
| -0.499 | NA | NA | + | NA | NA | 1.74E-01 | 6 | -145.367 | 302.734 | 0 | 0.393 |
| -0.601 | NA | NA | + | -4.51E-04 | NA | 1.83E-01 | 7 | -144.861 | 303.721 | 0.987 | 0.24 |
| -0.533 | 1.13E-09 | NA | + | NA | NA | 1.80E-01 | 7 | -145.116 | 304.232 | 1.498 | 0.186 |
| -0.4 | NA | NA | + | NA | -4.48E-02 | 1.76E-01 | 7 | -145.136 | 304.273 | 1.539 | 0.182 |
| -0.734 | NA | NA | + | -6.45E-04 | NA | 2.17E-01 | 7 | -154.411 | 322.821 | 0 | 0.238 |
| -0.593 | NA | NA | + | NA | NA | 2.05E-01 | 6 | -155.45 | 322.9 | 0.079 | 0.229 |
| -0.588 | NA | NA | + | -7.21E-04 | -7.21E-02 | 2.18E-01 | 8 | -153.829 | 323.659 | 0.838 | 0.157 |
| -0.79 | 1.71E-09 | NA | + | -6.69E-04 | NA | 2.25E-01 | 8 | -153.97 | 323.94 | 1.119 | 0.136 |
| -0.639 | 1.56E-09 | NA | + | NA | NA | 2.12E-01 | 7 | -155.089 | 324.179 | 1.358 | 0.121 |
| -0.468 | NA | NA | + | NA | -5.54E-02 | 2.05E-01 | 7 | -155.103 | 324.206 | 1.385 | 0.119 |
| -0.504 | NA | NA | + | NA | NA | 1.76E-01 | 6 | -152.602 | 317.204 | 0 | 0.325 |
| -0.598 | NA | NA | + | -4.38E-04 | NA | 1.83E-01 | 7 | -152.14 | 318.28 | 1.076 | 0.19 |
| -0.372 | NA | NA | + | NA | -6.13E-02 | 1.79E-01 | 7 | -152.144 | 318.288 | 1.084 | 0.189 |
| -0.543 | 1.34E-09 | NA | + | NA | NA | 1.83E-01 | 7 | -152.271 | 318.542 | 1.337 | 0.166 |
| -0.459 | NA | NA | + | -5.14E-04 | -7.19E-02 | 1.88E-01 | 8 | -151.519 | 319.037 | 1.833 | 0.13 |
| -0.654 | NA | NA | + | NA | NA | 2.10E-01 | 6 | -163.061 | 338.121 | 0 | 0.26 |
| -0.771 | NA | NA | + | -5.85E-04 | NA | 2.19E-01 | 7 | -162.274 | 338.547 | 0.426 | 0.21 |
| -0.701 | 1.49E-09 | NA | + | NA | NA | 2.19E-01 | 7 | -162.682 | 339.364 | 1.243 | 0.14 |
| -0.526 | NA | NA | + | NA | -5.54E-02 | 2.10E-01 | 7 | -162.723 | 339.447 | 1.325 | 0.134 |
| -0.625 | NA | NA | + | -6.58E-04 | -6.96E-02 | 2.20E-01 | 8 | -161.746 | 339.492 | 1.37 | 0.131 |
| -0.832 | 1.69E-09 | NA | + | -6.25E-04 | NA | 2.30E-01 | 8 | -161.786 | 339.571 | 1.45 | 0.126 |
| -0.566 | NA | NA | + | NA | NA | 1.94E-01 | 6 | -156.37 | 324.741 | 0 | 0.409 |
| -0.641 | NA | NA | + | -3.63E-04 | NA | 2.01E-01 | 7 | -156.07 | 326.139 | 1.398 | 0.203 |
| -0.604 | 1.22E-09 | NA | + | NA | NA | 2.01E-01 | 7 | -156.089 | 326.178 | 1.437 | 0.199 |
| -0.463 | NA | NA | + | NA | -4.57E-02 | 1.94E-01 | 7 | -156.145 | 326.29 | 1.549 | 0.188 |
| -0.526 | NA | NA | + | NA | NA | 1.80E-01 | 6 | -162.628 | 337.256 | 0 | 0.415 |
| -0.569 | 1.52E-09 | NA | + | NA | NA | 1.86E-01 | 7 | -162.315 | 338.63 | 1.374 | 0.209 |
| -0.597 | NA | NA | + | -3.26E-04 | NA | 1.85E-01 | 7 | -162.411 | 338.821 | 1.565 | 0.19 |
| -0.428 | NA | NA | + | NA | -4.48E-02 | 1.81E-01 | 7 | -162.426 | 338.851 | 1.595 | 0.187 |
| -0.572 | NA | NA | + | NA | NA | 1.95E-01 | 6 | -156.108 | 324.216 | 0 | 0.385 |
| -0.671 | NA | NA | + | -4.50E-04 | NA | 2.03E-01 | 7 | -155.628 | 325.256 | 1.04 | 0.229 |
| -0.612 | 1.37E-09 | NA | + | NA | NA | 2.02E-01 | 7 | -155.788 | 325.577 | 1.361 | 0.195 |
| -0.457 | NA | NA | + | NA | -5.14E-02 | 1.96E-01 | 7 | -155.814 | 325.629 | 1.413 | 0.19 |
| -0.465 | NA | NA | + | NA | NA | 1.64E-01 | 6 | -146.665 | 305.331 | 0 | 0.401 |
| -0.551 | NA | NA | + | -3.97E-04 | NA | 1.72E-01 | 7 | -146.276 | 306.553 | 1.222 | 0.218 |
| -0.502 | 1.10E-09 | NA | + | NA | NA | 1.72E-01 | 7 | -146.373 | 306.746 | 1.415 | 0.198 |
| -0.363 | NA | NA | + | NA | -4.49E-02 | 1.65E-01 | 7 | -146.443 | 306.886 | 1.555 | 0.184 |
| -0.143 | NA | NA | NA | 7.41E-04 | NA | 1.35E-01 | 4 | -179.56 | 367.119 | 0 | 0.288 |
| -0.282 | NA | NA | NA | NA | NA | 1.72E-01 | 3 | -181.088 | 368.176 | 1.057 | 0.17 |
| -0.367 | NA | NA | + | NA | NA | 1.31E-01 | 6 | -178.102 | 368.205 | 1.086 | 0.167 |
| 0.233 | NA | NA | NA | 9.70E-04 | NA | NA | 3 | -181.28 | 368.56 | 1.441 | 0.14 |
| -0.226 | NA | NA | NA | 8.04E-04 | 4.54E-02 | 1.33E-01 | 5 | -179.406 | 368.813 | 1.694 | 0.124 |
| -0.163 | 4.48E-10 | NA | NA | 7.26E-04 | NA | 1.40E-01 | 5 | -179.516 | 369.032 | 1.913 | 0.111 |
| -0.747 | NA | NA | + | -7.38E-04 | NA | 2.12E-01 | 7 | -149.234 | 312.468 | 0 | 0.241 |
| -0.574 | NA | NA | + | -8.24E-04 | -8.55E-02 | 2.14E-01 | 8 | -148.376 | 312.752 | 0.284 | 0.209 |
| -0.81 | 1.51E-09 | NA | + | -7.88E-04 | NA | 2.23E-01 | 8 | -148.647 | 313.294 | 0.826 | 0.159 |
| -0.595 | NA | NA | + | NA | NA | 2.00E-01 | 6 | -150.65 | 313.3 | 0.832 | 0.159 |
| -0.639 | 1.44E-09 | NA | + | -8.68E-04 | -8.27E-02 | 2.25E-01 | 9 | -147.84 | 313.68 | 1.211 | 0.132 |
| -0.444 | NA | NA | + | NA | -6.74E-02 | 2.01E-01 | 7 | -150.116 | 314.232 | 1.763 | 0.1 |
| -0.429 | NA | NA | + | NA | NA | 1.63E-01 | 6 | -152.762 | 317.523 | 0 | 0.381 |
| -0.462 | 1.02E-09 | NA | + | NA | NA | 1.69E-01 | 7 | -152.55 | 319.1 | 1.577 | 0.173 |
| -0.365 | NA | NA | NA | NA | NA | 2.05E-01 | 3 | -156.683 | 319.366 | 1.842 | 0.152 |
| -0.463 | NA | NA | + | -1.47E-04 | NA | 1.67E-01 | 7 | -152.712 | 319.424 | 1.901 | 0.147 |
| -0.381 | NA | NA | + | NA | -2.19E-02 | 1.63E-01 | 7 | -152.712 | 319.425 | 1.901 | 0.147 |
| -0.41 | NA | NA | + | NA | NA | 1.54E-01 | 6 | -144.172 | 300.344 | 0 | 0.429 |
| -0.482 | NA | NA | + | -3.09E-04 | NA | 1.61E-01 | 7 | -143.935 | 301.87 | 1.526 | 0.2 |
| -0.44 | 9.58E-10 | NA | + | NA | NA | 1.60E-01 | 7 | -143.959 | 301.919 | 1.575 | 0.195 |
| -0.345 | NA | NA | + | NA | -3.04E-02 | 1.56E-01 | 7 | -144.07 | 302.139 | 1.795 | 0.175 |
| -0.471 | NA | NA | + | NA | NA | 1.68E-01 | 6 | -150.788 | 313.576 | 0 | 0.398 |
| -0.577 | NA | NA | + | -4.62E-04 | NA | 1.77E-01 | 7 | -150.266 | 314.532 | 0.957 | 0.247 |
| -0.506 | 1.18E-09 | NA | + | NA | NA | 1.73E-01 | 7 | -150.572 | 315.143 | 1.567 | 0.182 |
| -0.386 | NA | NA | + | NA | -3.90E-02 | 1.69E-01 | 7 | -150.623 | 315.246 | 1.671 | 0.173 |
| -0.428 | NA | NA | + | NA | NA | 1.61E-01 | 6 | -158.001 | 328.002 | 0 | 0.452 |
| -0.457 | 1.14E-09 | NA | + | NA | NA | 1.65E-01 | 7 | -157.845 | 329.69 | 1.688 | 0.195 |
| -0.48 | NA | NA | + | -2.31E-04 | NA | 1.65E-01 | 7 | -157.887 | 329.774 | 1.772 | 0.187 |
| -0.432 | NA | NA | + | NA | 2.05E-03 | 1.60E-01 | 7 | -158.001 | 330.001 | 1.999 | 0.167 |
| -0.496 | NA | NA | + | NA | NA | 1.76E-01 | 6 | -170.046 | 352.092 | 0 | 0.449 |
| -0.535 | 1.48E-09 | NA | + | NA | NA | 1.81E-01 | 7 | -169.816 | 353.631 | 1.539 | 0.208 |
| -0.441 | NA | NA | + | NA | -2.41E-02 | 1.76E-01 | 7 | -169.991 | 353.983 | 1.89 | 0.175 |
| -0.515 | NA | NA | + | -8.32E-05 | NA | 1.77E-01 | 7 | -170.033 | 354.065 | 1.973 | 0.168 |
| -0.531 | NA | NA | + | NA | NA | 1.86E-01 | 6 | -153.813 | 319.627 | 0 | 0.414 |
| -0.613 | NA | NA | + | -3.92E-04 | NA | 1.93E-01 | 7 | -153.455 | 320.91 | 1.283 | 0.218 |
| -0.567 | 1.23E-09 | NA | + | NA | NA | 1.91E-01 | 7 | -153.565 | 321.129 | 1.503 | 0.195 |
| -0.455 | NA | NA | + | NA | -3.44E-02 | 1.86E-01 | 7 | -153.689 | 321.378 | 1.752 | 0.172 |
| -0.685 | NA | NA | + | -6.35E-04 | NA | 2.00E-01 | 7 | -146.919 | 307.838 | 0 | 0.193 |
| -0.548 | NA | NA | + | NA | NA | 1.89E-01 | 6 | -147.949 | 307.899 | 0.061 | 0.188 |
| -0.524 | NA | NA | + | -7.26E-04 | -8.34E-02 | 2.06E-01 | 8 | -146.053 | 308.107 | 0.269 | 0.169 |
| -0.741 | 1.87E-09 | NA | + | -6.54E-04 | NA | 2.08E-01 | 8 | -146.378 | 308.757 | 0.919 | 0.122 |
| -0.402 | NA | NA | + | NA | -6.73E-02 | 1.93E-01 | 7 | -147.381 | 308.762 | 0.925 | 0.122 |
| -0.596 | 1.75E-09 | NA | + | NA | NA | 1.96E-01 | 7 | -147.479 | 308.959 | 1.121 | 0.11 |
| -0.584 | 1.67E-09 | NA | + | -7.38E-04 | -7.81E-02 | 2.13E-01 | 9 | -145.622 | 309.244 | 1.406 | 0.096 |
| -0.484 | NA | NA | + | NA | NA | 1.69E-01 | 6 | -158.441 | 328.881 | 0 | 0.442 |
| -0.555 | NA | NA | + | -3.20E-04 | NA | 1.74E-01 | 7 | -158.226 | 330.451 | 1.57 | 0.202 |
| -0.514 | 1.06E-09 | NA | + | NA | NA | 1.73E-01 | 7 | -158.295 | 330.59 | 1.709 | 0.188 |
| -0.525 | NA | NA | + | NA | 1.88E-02 | 1.67E-01 | 7 | -158.409 | 330.818 | 1.937 | 0.168 |
| -0.607 | NA | NA | + | -8.03E-04 | -1.01E-01 | 2.41E-01 | 8 | -157.786 | 331.571 | 0 | 0.218 |
| -0.814 | NA | NA | + | -6.75E-04 | NA | 2.41E-01 | 7 | -159.01 | 332.02 | 0.449 | 0.174 |
| -0.683 | NA | NA | + | NA | NA | 2.31E-01 | 6 | -160.161 | 332.322 | 0.751 | 0.15 |
| -0.674 | 1.83E-09 | NA | + | -8.23E-04 | -9.52E-02 | 2.50E-01 | 9 | -157.308 | 332.617 | 1.046 | 0.129 |
| -0.499 | NA | NA | + | NA | -7.99E-02 | 2.29E-01 | 7 | -159.38 | 332.76 | 1.189 | 0.12 |
| -0.877 | 2.08E-09 | NA | + | -7.06E-04 | NA | 2.51E-01 | 8 | -158.398 | 332.795 | 1.224 | 0.118 |
| -0.735 | 1.89E-09 | NA | + | NA | NA | 2.39E-01 | 7 | -159.661 | 333.322 | 1.751 | 0.091 |
| -0.416 | NA | NA | + | NA | NA | 1.48E-01 | 6 | -155.146 | 322.292 | 0 | 0.391 |
| -0.444 | 8.26E-10 | NA | + | NA | NA | 1.53E-01 | 7 | -155.001 | 324.001 | 1.709 | 0.166 |
| -0.333 | NA | NA | NA | NA | NA | 1.88E-01 | 3 | -159.085 | 324.17 | 1.878 | 0.153 |
| -0.388 | NA | NA | + | NA | -1.26E-02 | 1.49E-01 | 7 | -155.131 | 324.262 | 1.971 | 0.146 |
| -0.417 | NA | NA | + | -4.79E-06 | NA | 1.48E-01 | 7 | -155.146 | 324.292 | 2 | 0.144 |
| -0.651 | NA | NA | + | -1.03E-03 | -1.05E-01 | 2.44E-01 | 8 | -153.572 | 323.144 | 0 | 0.338 |
| -0.726 | 1.92E-09 | NA | + | -1.07E-03 | -1.00E-01 | 2.55E-01 | 9 | -152.907 | 323.815 | 0.671 | 0.242 |
| -0.861 | NA | NA | + | -8.89E-04 | NA | 2.45E-01 | 7 | -154.947 | 323.895 | 0.751 | 0.232 |
| -0.933 | 2.10E-09 | NA | + | -9.36E-04 | NA | 2.56E-01 | 8 | -154.161 | 324.322 | 1.178 | 0.188 |
| -0.521 | NA | NA | + | NA | NA | 1.80E-01 | 6 | -174.421 | 360.843 | 0 | 0.455 |
| -0.562 | 1.56E-09 | NA | + | NA | NA | 1.86E-01 | 7 | -174.199 | 362.399 | 1.556 | 0.209 |
| -0.506 | NA | NA | + | NA | -7.35E-03 | 1.80E-01 | 7 | -174.417 | 362.833 | 1.99 | 0.168 |
| -0.523 | NA | NA | + | -6.00E-06 | NA | 1.80E-01 | 7 | -174.421 | 362.843 | 2 | 0.167 |
| -0.399 | NA | NA | + | NA | NA | 1.49E-01 | 6 | -153.602 | 319.204 | 0 | 0.378 |
| -0.326 | NA | NA | NA | NA | NA | 1.88E-01 | 3 | -157.34 | 320.679 | 1.475 | 0.181 |
| -0.424 | 8.78E-10 | NA | + | NA | NA | 1.53E-01 | 7 | -153.474 | 320.949 | 1.745 | 0.158 |
| -0.431 | NA | NA | + | NA | 1.47E-02 | 1.48E-01 | 7 | -153.582 | 321.165 | 1.961 | 0.142 |
| -0.419 | NA | NA | + | -8.49E-05 | NA | 1.51E-01 | 7 | -153.586 | 321.172 | 1.968 | 0.141 |
| -0.576 | NA | NA | + | NA | NA | 2.01E-01 | 6 | -162.602 | 337.204 | 0 | 0.394 |
| -0.667 | NA | NA | + | -4.08E-04 | NA | 2.10E-01 | 7 | -162.229 | 338.459 | 1.254 | 0.211 |
| -0.452 | NA | NA | + | NA | -5.46E-02 | 2.01E-01 | 7 | -162.282 | 338.564 | 1.359 | 0.2 |
| -0.616 | 1.46E-09 | NA | + | NA | NA | 2.09E-01 | 7 | -162.304 | 338.608 | 1.404 | 0.195 |
| -0.532 | NA | NA | + | NA | NA | 1.88E-01 | 6 | -153.048 | 318.096 | 0 | 0.41 |
| -0.573 | 1.37E-09 | NA | + | NA | NA | 1.96E-01 | 7 | -152.723 | 319.447 | 1.351 | 0.209 |
| -0.605 | NA | NA | + | -3.43E-04 | NA | 1.95E-01 | 7 | -152.774 | 319.548 | 1.452 | 0.198 |
| -0.44 | NA | NA | + | NA | -4.15E-02 | 1.89E-01 | 7 | -152.856 | 319.712 | 1.616 | 0.183 |
| -0.348 | NA | NA | + | NA | NA | 1.45E-01 | 6 | -144.647 | 301.293 | 0 | 0.279 |
| -0.288 | NA | NA | NA | NA | NA | 1.78E-01 | 3 | -147.864 | 301.729 | 0.435 | 0.225 |
| -0.21 | NA | NA | NA | 4.23E-04 | NA | 1.57E-01 | 4 | -147.151 | 302.302 | 1.008 | 0.169 |
| -0.375 | 9.00E-10 | NA | + | NA | NA | 1.50E-01 | 7 | -144.48 | 302.961 | 1.667 | 0.121 |
| -0.332 | NA | NA | + | NA | -7.52E-03 | 1.45E-01 | 7 | -144.641 | 303.282 | 1.989 | 0.103 |
| -0.349 | NA | NA | + | -3.86E-06 | NA | 1.45E-01 | 7 | -144.647 | 303.293 | 2 | 0.103 |
| -0.545 | NA | NA | + | NA | NA | 1.92E-01 | 6 | -149.038 | 310.077 | 0 | 0.249 |
| -0.661 | NA | NA | + | -5.76E-04 | NA | 2.00E-01 | 7 | -148.176 | 310.353 | 0.276 | 0.217 |
| -0.517 | NA | NA | + | -6.37E-04 | -7.03E-02 | 2.03E-01 | 8 | -147.605 | 311.211 | 1.134 | 0.141 |
| -0.417 | NA | NA | + | NA | -5.79E-02 | 1.94E-01 | 7 | -148.65 | 311.299 | 1.223 | 0.135 |
| -0.717 | 1.38E-09 | NA | + | -6.32E-04 | NA | 2.11E-01 | 8 | -147.686 | 311.372 | 1.296 | 0.13 |
| -0.582 | 1.12E-09 | NA | + | NA | NA | 2.00E-01 | 7 | -148.713 | 311.425 | 1.348 | 0.127 |
| -0.47 | NA | NA | + | NA | NA | 1.68E-01 | 6 | -143.772 | 299.543 | 0 | 0.33 |
| -0.586 | NA | NA | + | -5.12E-04 | NA | 1.79E-01 | 7 | -143.095 | 300.19 | 0.646 | 0.239 |
| -0.504 | 1.22E-09 | NA | + | NA | NA | 1.74E-01 | 7 | -143.524 | 301.048 | 1.505 | 0.156 |
| -0.368 | NA | NA | + | NA | -4.52E-02 | 1.69E-01 | 7 | -143.543 | 301.087 | 1.544 | 0.153 |
| -0.472 | NA | NA | + | -5.53E-04 | -5.49E-02 | 1.80E-01 | 8 | -142.76 | 301.519 | 1.976 | 0.123 |
| -0.505 | NA | NA | + | NA | NA | 1.76E-01 | 6 | -147.689 | 307.378 | 0 | 0.281 |
| -0.629 | NA | NA | + | -5.67E-04 | NA | 1.86E-01 | 7 | -146.894 | 307.789 | 0.411 | 0.229 |
| -0.542 | 1.11E-09 | NA | + | NA | NA | 1.83E-01 | 7 | -147.405 | 308.809 | 1.432 | 0.137 |
| -0.678 | 1.27E-09 | NA | + | -5.97E-04 | NA | 1.94E-01 | 8 | -146.524 | 309.049 | 1.671 | 0.122 |
| -0.422 | NA | NA | + | NA | -3.74E-02 | 1.77E-01 | 7 | -147.534 | 309.068 | 1.69 | 0.121 |
| -0.528 | NA | NA | + | -6.12E-04 | -4.98E-02 | 1.87E-01 | 8 | -146.622 | 309.244 | 1.867 | 0.11 |
| -0.503 | NA | NA | + | NA | NA | 1.80E-01 | 6 | -146.076 | 304.153 | 0 | 0.316 |
| -0.605 | NA | NA | + | -4.52E-04 | NA | 1.89E-01 | 7 | -145.544 | 305.088 | 0.935 | 0.198 |
| -0.365 | NA | NA | + | NA | -6.32E-02 | 1.82E-01 | 7 | -145.592 | 305.183 | 1.03 | 0.189 |
| -0.539 | 1.41E-09 | NA | + | NA | NA | 1.86E-01 | 7 | -145.785 | 305.57 | 1.417 | 0.155 |
| -0.459 | NA | NA | + | -5.29E-04 | -7.49E-02 | 1.93E-01 | 8 | -144.874 | 305.748 | 1.595 | 0.142 |
| -0.437 | NA | NA | + | NA | NA | 1.61E-01 | 6 | -150.54 | 313.079 | 0 | 0.439 |
| -0.472 | 1.32E-09 | NA | + | NA | NA | 1.66E-01 | 7 | -150.315 | 314.631 | 1.552 | 0.202 |
| -0.501 | NA | NA | + | -2.85E-04 | NA | 1.66E-01 | 7 | -150.361 | 314.723 | 1.644 | 0.193 |
| -0.4 | NA | NA | + | NA | -1.67E-02 | 1.61E-01 | 7 | -150.51 | 315.02 | 1.941 | 0.166 |
| -0.488 | NA | NA | + | NA | NA | 1.70E-01 | 6 | -151.524 | 315.048 | 0 | 0.417 |
| -0.575 | NA | NA | + | -3.93E-04 | NA | 1.77E-01 | 7 | -151.165 | 316.329 | 1.281 | 0.22 |
| -0.522 | 1.18E-09 | NA | + | NA | NA | 1.75E-01 | 7 | -151.293 | 316.585 | 1.537 | 0.193 |
| -0.418 | NA | NA | + | NA | -3.13E-02 | 1.71E-01 | 7 | -151.424 | 316.849 | 1.801 | 0.17 |
| -0.522 | NA | NA | + | NA | NA | 1.83E-01 | 6 | -147.078 | 306.156 | 0 | 0.285 |
| -0.64 | NA | NA | + | -5.50E-04 | NA | 1.94E-01 | 7 | -146.304 | 306.608 | 0.452 | 0.227 |
| -0.556 | 1.16E-09 | NA | + | NA | NA | 1.90E-01 | 7 | -146.809 | 307.619 | 1.463 | 0.137 |
| -0.439 | NA | NA | + | NA | -3.73E-02 | 1.84E-01 | 7 | -146.92 | 307.84 | 1.684 | 0.123 |
| -0.687 | 1.33E-09 | NA | + | -5.80E-04 | NA | 2.02E-01 | 8 | -145.949 | 307.899 | 1.743 | 0.119 |
| -0.543 | NA | NA | + | -5.87E-04 | -4.75E-02 | 1.96E-01 | 8 | -146.048 | 308.096 | 1.94 | 0.108 |
| -0.444 | NA | NA | + | NA | NA | 1.64E-01 | 6 | -135.202 | 282.403 | 0 | 0.287 |
| -0.553 | NA | NA | + | -4.74E-04 | NA | 1.72E-01 | 7 | -134.576 | 283.153 | 0.749 | 0.197 |
| -0.326 | NA | NA | + | NA | -5.39E-02 | 1.66E-01 | 7 | -134.839 | 283.678 | 1.275 | 0.152 |
| -0.474 | 9.28E-10 | NA | + | NA | NA | 1.70E-01 | 7 | -134.939 | 283.878 | 1.475 | 0.137 |
| -0.426 | NA | NA | + | -5.26E-04 | -6.33E-02 | 1.76E-01 | 8 | -134.078 | 284.156 | 1.753 | 0.119 |
| -0.6 | 1.14E-09 | NA | + | -5.24E-04 | NA | 1.80E-01 | 8 | -134.187 | 284.373 | 1.97 | 0.107 |
| -0.436 | NA | NA | + | NA | NA | 1.60E-01 | 6 | -149.224 | 310.449 | 0 | 0.414 |
| -0.523 | NA | NA | + | -3.78E-04 | NA | 1.67E-01 | 7 | -148.888 | 311.775 | 1.327 | 0.213 |
| -0.47 | 1.16E-09 | NA | + | NA | NA | 1.65E-01 | 7 | -148.996 | 311.992 | 1.544 | 0.191 |
| -0.348 | NA | NA | + | NA | -4.09E-02 | 1.62E-01 | 7 | -149.045 | 312.091 | 1.642 | 0.182 |
| -0.642 | NA | NA | + | -1.04E-03 | -1.14E-01 | 2.49E-01 | 8 | -153.082 | 322.164 | 0 | 0.472 |
| -0.709 | 1.81E-09 | NA | + | -1.07E-03 | -1.08E-01 | 2.58E-01 | 9 | -152.576 | 323.153 | 0.989 | 0.288 |
| -0.878 | NA | NA | + | -9.18E-04 | NA | 2.49E-01 | 7 | -154.759 | 323.518 | 1.354 | 0.24 |
| -0.473 | NA | NA | + | NA | NA | 1.73E-01 | 6 | -142.965 | 297.93 | 0 | 0.33 |
| -0.585 | NA | NA | + | -5.01E-04 | NA | 1.82E-01 | 7 | -142.303 | 298.607 | 0.677 | 0.235 |
| -0.369 | NA | NA | + | NA | -4.83E-02 | 1.75E-01 | 7 | -142.701 | 299.401 | 1.471 | 0.158 |
| -0.502 | 1.01E-09 | NA | + | NA | NA | 1.78E-01 | 7 | -142.75 | 299.5 | 1.57 | 0.151 |
| -0.471 | NA | NA | + | -5.41E-04 | -5.73E-02 | 1.85E-01 | 8 | -141.933 | 299.866 | 1.936 | 0.125 |
| -0.5 | NA | NA | + | NA | NA | 1.77E-01 | 6 | -146.312 | 304.623 | 0 | 0.404 |
| -0.588 | NA | NA | + | -3.90E-04 | NA | 1.85E-01 | 7 | -145.931 | 305.863 | 1.24 | 0.217 |
| -0.392 | NA | NA | + | NA | -4.83E-02 | 1.78E-01 | 7 | -146.041 | 306.081 | 1.458 | 0.195 |
| -0.533 | 1.04E-09 | NA | + | NA | NA | 1.83E-01 | 7 | -146.095 | 306.19 | 1.567 | 0.184 |
| -0.493 | NA | NA | + | -6.77E-04 | -9.98E-02 | 2.10E-01 | 8 | -154.211 | 324.421 | 0 | 0.182 |
| -0.563 | NA | NA | + | NA | NA | 1.95E-01 | 6 | -156.272 | 324.544 | 0.123 | 0.171 |
| -0.379 | NA | NA | + | NA | -8.50E-02 | 1.98E-01 | 7 | -155.367 | 324.734 | 0.313 | 0.156 |
| -0.687 | NA | NA | + | -5.70E-04 | NA | 2.05E-01 | 7 | -155.444 | 324.888 | 0.467 | 0.144 |
| -0.611 | 1.84E-09 | NA | + | NA | NA | 2.02E-01 | 7 | -155.861 | 325.722 | 1.301 | 0.095 |
| -0.547 | 1.61E-09 | NA | + | -6.82E-04 | -9.45E-02 | 2.16E-01 | 9 | -153.891 | 325.782 | 1.361 | 0.092 |
| -0.739 | 1.92E-09 | NA | + | -5.83E-04 | NA | 2.12E-01 | 8 | -154.991 | 325.982 | 1.56 | 0.083 |
| -0.43 | 1.57E-09 | NA | + | NA | -7.97E-02 | 2.03E-01 | 8 | -155.07 | 326.14 | 1.719 | 0.077 |
| -0.482 | NA | NA | + | NA | NA | 1.71E-01 | 6 | -164.326 | 340.651 | 0 | 0.457 |
| -0.514 | 1.24E-09 | NA | + | NA | NA | 1.76E-01 | 7 | -164.156 | 342.311 | 1.66 | 0.199 |
| -0.513 | NA | NA | + | -1.43E-04 | NA | 1.74E-01 | 7 | -164.285 | 342.571 | 1.919 | 0.175 |
| -0.469 | NA | NA | + | NA | -5.81E-03 | 1.71E-01 | 7 | -164.323 | 342.646 | 1.994 | 0.169 |
| -0.424 | NA | NA | + | NA | NA | 1.63E-01 | 6 | -152.688 | 317.376 | 0 | 0.445 |
| -0.46 | 1.26E-09 | NA | + | NA | NA | 1.69E-01 | 7 | -152.442 | 318.884 | 1.508 | 0.209 |
| -0.468 | NA | NA | + | -1.93E-04 | NA | 1.67E-01 | 7 | -152.605 | 319.209 | 1.833 | 0.178 |
| -0.388 | NA | NA | + | NA | -1.62E-02 | 1.63E-01 | 7 | -152.661 | 319.322 | 1.946 | 0.168 |
| -0.36 | NA | NA | + | NA | NA | 1.45E-01 | 6 | -143.578 | 299.155 | 0 | 0.375 |
| -0.311 | NA | NA | NA | NA | NA | 1.79E-01 | 3 | -147.343 | 300.685 | 1.53 | 0.175 |
| -0.384 | 9.19E-10 | NA | + | NA | NA | 1.48E-01 | 7 | -143.453 | 300.906 | 1.75 | 0.156 |
| -0.407 | NA | NA | + | -2.00E-04 | NA | 1.49E-01 | 7 | -143.482 | 300.965 | 1.809 | 0.152 |
| -0.324 | NA | NA | + | NA | -1.71E-02 | 1.46E-01 | 7 | -143.546 | 301.092 | 1.936 | 0.142 |
| -0.362 | NA | NA | + | NA | NA | 1.42E-01 | 6 | -151.344 | 314.688 | 0 | 0.279 |
| -0.29 | NA | NA | NA | NA | NA | 1.76E-01 | 3 | -154.581 | 315.163 | 0.475 | 0.22 |
| -0.204 | NA | NA | NA | 4.54E-04 | NA | 1.54E-01 | 4 | -153.834 | 315.668 | 0.98 | 0.171 |
| -0.391 | 7.71E-10 | NA | + | NA | NA | 1.48E-01 | 7 | -151.167 | 316.333 | 1.645 | 0.123 |
| -0.342 | NA | NA | + | NA | -9.46E-03 | 1.43E-01 | 7 | -151.335 | 316.671 | 1.983 | 0.104 |
| -0.365 | NA | NA | + | -1.33E-05 | NA | 1.42E-01 | 7 | -151.344 | 316.687 | 1.999 | 0.103 |
| -0.503 | NA | NA | + | NA | NA | 1.80E-01 | 6 | -146.336 | 304.671 | 0 | 0.402 |
| -0.593 | NA | NA | + | -3.90E-04 | NA | 1.88E-01 | 7 | -145.958 | 305.916 | 1.245 | 0.216 |
| -0.541 | 1.41E-09 | NA | + | NA | NA | 1.85E-01 | 7 | -146.059 | 306.118 | 1.447 | 0.195 |
| -0.408 | NA | NA | + | NA | -4.44E-02 | 1.83E-01 | 7 | -146.105 | 306.21 | 1.538 | 0.186 |
| -0.406 | NA | NA | + | NA | NA | 1.55E-01 | 6 | -138.233 | 288.465 | 0 | 0.403 |
| -0.507 | NA | NA | + | -4.47E-04 | NA | 1.65E-01 | 7 | -137.706 | 289.412 | 0.946 | 0.251 |
| -0.433 | 8.50E-10 | NA | + | NA | NA | 1.60E-01 | 7 | -138.057 | 290.114 | 1.649 | 0.177 |
| -0.334 | NA | NA | + | NA | -3.36E-02 | 1.56E-01 | 7 | -138.095 | 290.189 | 1.724 | 0.17 |
| -0.581 | NA | NA | + | NA | NA | 1.98E-01 | 6 | -156.032 | 324.064 | 0 | 0.246 |
| -0.713 | NA | NA | + | -6.05E-04 | NA | 2.09E-01 | 7 | -155.162 | 324.324 | 0.26 | 0.216 |
| -0.557 | NA | NA | + | -6.80E-04 | -7.50E-02 | 2.10E-01 | 8 | -154.509 | 325.019 | 0.955 | 0.153 |
| -0.441 | NA | NA | + | NA | -6.11E-02 | 1.98E-01 | 7 | -155.596 | 325.193 | 1.129 | 0.14 |
| -0.623 | 1.62E-09 | NA | + | NA | NA | 2.05E-01 | 7 | -155.7 | 325.4 | 1.336 | 0.126 |
| -0.764 | 1.79E-09 | NA | + | -6.30E-04 | NA | 2.17E-01 | 8 | -154.755 | 325.509 | 1.445 | 0.119 |
| -0.448 | NA | NA | + | NA | NA | 1.67E-01 | 6 | -145.726 | 303.452 | 0 | 0.425 |
| -0.532 | NA | NA | + | -3.61E-04 | NA | 1.76E-01 | 7 | -145.4 | 304.8 | 1.347 | 0.216 |
| -0.477 | 9.93E-10 | NA | + | NA | NA | 1.73E-01 | 7 | -145.551 | 305.102 | 1.65 | 0.186 |
| -0.382 | NA | NA | + | NA | -3.04E-02 | 1.69E-01 | 7 | -145.625 | 305.249 | 1.797 | 0.173 |
| -0.423 | NA | NA | + | NA | NA | 1.56E-01 | 6 | -159.762 | 331.525 | 0 | 0.384 |
| -0.352 | NA | NA | NA | NA | NA | 1.95E-01 | 3 | -163.582 | 333.163 | 1.638 | 0.169 |
| -0.451 | 1.01E-09 | NA | + | NA | NA | 1.60E-01 | 7 | -159.622 | 333.243 | 1.718 | 0.163 |
| -0.434 | NA | NA | + | -4.92E-05 | NA | 1.57E-01 | 7 | -159.757 | 333.515 | 1.99 | 0.142 |
| -0.435 | NA | NA | + | NA | 5.82E-03 | 1.56E-01 | 7 | -159.759 | 333.519 | 1.994 | 0.142 |
| -0.632 | NA | NA | + | -8.85E-04 | -1.02E-01 | 2.40E-01 | 8 | -159.655 | 335.309 | 0 | 0.254 |
| -0.849 | NA | NA | + | -7.57E-04 | NA | 2.42E-01 | 7 | -160.866 | 335.732 | 0.423 | 0.206 |
| -0.701 | 1.85E-09 | NA | + | -9.05E-04 | -9.65E-02 | 2.49E-01 | 9 | -159.142 | 336.283 | 0.974 | 0.156 |
| -0.913 | 2.06E-09 | NA | + | -7.87E-04 | NA | 2.53E-01 | 8 | -160.234 | 336.469 | 1.159 | 0.142 |
| -0.701 | NA | NA | + | NA | NA | 2.30E-01 | 6 | -162.277 | 336.553 | 1.244 | 0.137 |
| -0.514 | NA | NA | + | NA | -7.86E-02 | 2.26E-01 | 7 | -161.544 | 337.088 | 1.778 | 0.105 |
| -0.383 | NA | NA | + | NA | NA | 1.47E-01 | 6 | -144.208 | 300.416 | 0 | 0.378 |
| -0.313 | NA | NA | NA | NA | NA | 1.84E-01 | 3 | -148.014 | 302.028 | 1.611 | 0.169 |
| -0.411 | 9.25E-10 | NA | + | NA | NA | 1.52E-01 | 7 | -144.043 | 302.085 | 1.669 | 0.164 |
| -0.424 | NA | NA | + | -1.78E-04 | NA | 1.51E-01 | 7 | -144.133 | 302.267 | 1.851 | 0.15 |
| -0.39 | NA | NA | + | NA | 3.28E-03 | 1.47E-01 | 7 | -144.207 | 302.414 | 1.998 | 0.139 |
| -0.503 | NA | NA | + | NA | NA | 1.79E-01 | 6 | -152.176 | 316.351 | 0 | 0.283 |
| -0.612 | NA | NA | + | -5.36E-04 | NA | 1.88E-01 | 7 | -151.485 | 316.969 | 0.618 | 0.208 |
| -0.541 | 1.20E-09 | NA | + | NA | NA | 1.87E-01 | 7 | -151.867 | 317.735 | 1.384 | 0.142 |
| -0.39 | NA | NA | + | NA | -5.12E-02 | 1.79E-01 | 7 | -151.895 | 317.79 | 1.439 | 0.138 |
| -0.665 | 1.40E-09 | NA | + | -5.79E-04 | NA | 1.97E-01 | 8 | -151.068 | 318.135 | 1.784 | 0.116 |
| -0.486 | NA | NA | + | -5.86E-04 | -6.17E-02 | 1.89E-01 | 8 | -151.078 | 318.156 | 1.805 | 0.115 |
| -0.461 | NA | NA | + | NA | NA | 1.70E-01 | 6 | -161.184 | 334.368 | 0 | 0.457 |
| -0.494 | 9.24E-10 | NA | + | NA | NA | 1.78E-01 | 7 | -160.979 | 335.959 | 1.591 | 0.206 |
| -0.475 | NA | NA | + | -6.21E-05 | NA | 1.71E-01 | 7 | -161.176 | 336.352 | 1.984 | 0.169 |
| -0.46 | NA | NA | + | NA | -4.95E-04 | 1.70E-01 | 7 | -161.184 | 336.368 | 2 | 0.168 |
| -0.618 | NA | NA | + | -1.07E-03 | -1.26E-01 | 2.43E-01 | 8 | -154.653 | 325.306 | 0 | 0.612 |
| -0.686 | 1.80E-09 | NA | + | -1.09E-03 | -1.20E-01 | 2.52E-01 | 9 | -154.108 | 326.217 | 0.911 | 0.388 |
| -0.506 | NA | NA | + | NA | NA | 1.82E-01 | 6 | -148.507 | 309.014 | 0 | 0.261 |
| -0.632 | NA | NA | + | -5.68E-04 | NA | 1.93E-01 | 7 | -147.687 | 309.373 | 0.359 | 0.218 |
| -0.378 | NA | NA | + | NA | -5.73E-02 | 1.82E-01 | 7 | -148.131 | 310.263 | 1.249 | 0.14 |
| -0.491 | NA | NA | + | -6.25E-04 | -6.87E-02 | 1.95E-01 | 8 | -147.147 | 310.294 | 1.28 | 0.137 |
| -0.541 | 1.06E-09 | NA | + | NA | NA | 1.89E-01 | 7 | -148.236 | 310.473 | 1.459 | 0.126 |
| -0.684 | 1.28E-09 | NA | + | -6.11E-04 | NA | 2.03E-01 | 8 | -147.293 | 310.585 | 1.571 | 0.119 |
| -0.429 | NA | NA | + | NA | NA | 1.57E-01 | 6 | -145.674 | 303.348 | 0 | 0.411 |
| -0.525 | NA | NA | + | -3.96E-04 | NA | 1.66E-01 | 7 | -145.285 | 304.571 | 1.223 | 0.223 |
| -0.334 | NA | NA | + | NA | -4.32E-02 | 1.58E-01 | 7 | -145.467 | 304.934 | 1.586 | 0.186 |
| -0.456 | 9.79E-10 | NA | + | NA | NA | 1.61E-01 | 7 | -145.5 | 304.999 | 1.651 | 0.18 |
| -0.388 | NA | NA | + | NA | NA | 1.50E-01 | 6 | -145.264 | 302.529 | 0 | 0.375 |
| -0.459 | NA | NA | + | -2.93E-04 | NA | 1.57E-01 | 7 | -145.066 | 304.133 | 1.604 | 0.168 |
| -0.417 | 1.06E-09 | NA | + | NA | NA | 1.54E-01 | 7 | -145.106 | 304.212 | 1.684 | 0.161 |
| -0.347 | NA | NA | NA | NA | NA | 1.93E-01 | 3 | -149.152 | 304.305 | 1.776 | 0.154 |
| -0.352 | NA | NA | + | NA | -1.68E-02 | 1.50E-01 | 7 | -145.235 | 304.471 | 1.942 | 0.142 |
| -0.464 | NA | NA | + | NA | NA | 1.70E-01 | 6 | -150.05 | 312.101 | 0 | 0.332 |
| -0.586 | NA | NA | + | -5.08E-04 | NA | 1.82E-01 | 7 | -149.424 | 312.847 | 0.747 | 0.228 |
| -0.351 | NA | NA | + | NA | -5.16E-02 | 1.71E-01 | 7 | -149.766 | 313.531 | 1.43 | 0.162 |
| -0.497 | 1.00E-09 | NA | + | NA | NA | 1.77E-01 | 7 | -149.817 | 313.633 | 1.532 | 0.154 |
| -0.463 | NA | NA | + | -5.50E-04 | -6.04E-02 | 1.83E-01 | 8 | -149.035 | 314.069 | 1.968 | 0.124 |
| -0.578 | NA | NA | + | NA | NA | 2.02E-01 | 6 | -148.181 | 308.361 | 0 | 0.251 |
| -0.69 | NA | NA | + | -5.43E-04 | NA | 2.12E-01 | 7 | -147.417 | 308.834 | 0.473 | 0.198 |
| -0.527 | NA | NA | + | -6.30E-04 | -8.04E-02 | 2.14E-01 | 8 | -146.656 | 309.311 | 0.95 | 0.156 |
| -0.43 | NA | NA | + | NA | -6.55E-02 | 2.02E-01 | 7 | -147.667 | 309.333 | 0.972 | 0.154 |
| -0.618 | 1.38E-09 | NA | + | NA | NA | 2.09E-01 | 7 | -147.838 | 309.676 | 1.315 | 0.13 |
| -0.741 | 1.54E-09 | NA | + | -5.73E-04 | NA | 2.21E-01 | 8 | -146.988 | 309.977 | 1.615 | 0.112 |
| -0.694 | NA | NA | + | -7.40E-04 | -1.02E-01 | 2.59E-01 | 8 | -173.828 | 363.657 | 0 | 0.17 |
| -0.796 | NA | NA | + | NA | NA | 2.52E-01 | 6 | -175.867 | 363.733 | 0.077 | 0.163 |
| -0.598 | NA | NA | + | NA | -8.55E-02 | 2.50E-01 | 7 | -175.007 | 364.014 | 0.357 | 0.142 |
| -0.909 | NA | NA | + | -6.13E-04 | NA | 2.60E-01 | 7 | -175.044 | 364.088 | 0.431 | 0.137 |
| -0.861 | 2.37E-09 | NA | + | NA | NA | 2.62E-01 | 7 | -175.257 | 364.514 | 0.858 | 0.111 |
| -0.769 | 2.06E-09 | NA | + | -7.42E-04 | -9.45E-02 | 2.68E-01 | 9 | -173.366 | 364.731 | 1.075 | 0.099 |
| -0.978 | 2.45E-09 | NA | + | -6.27E-04 | NA | 2.71E-01 | 8 | -174.39 | 364.781 | 1.124 | 0.097 |
| -0.672 | 2.05E-09 | NA | + | NA | -7.75E-02 | 2.59E-01 | 8 | -174.557 | 365.114 | 1.458 | 0.082 |
| -0.479 | NA | NA | + | NA | NA | 1.71E-01 | 6 | -162.049 | 336.098 | 0 | 0.45 |
| -0.51 | 1.14E-09 | NA | + | NA | NA | 1.76E-01 | 7 | -161.878 | 337.756 | 1.658 | 0.196 |
| -0.535 | NA | NA | + | -2.38E-04 | NA | 1.76E-01 | 7 | -161.931 | 337.862 | 1.764 | 0.186 |
| -0.458 | NA | NA | + | NA | -9.44E-03 | 1.71E-01 | 7 | -162.041 | 338.081 | 1.984 | 0.167 |
| -0.133 | NA | NA | NA | 5.33E-04 | NA | 1.27E-01 | 4 | -144.566 | 297.132 | 0 | 0.23 |
| -0.294 | NA | NA | + | NA | NA | 1.21E-01 | 6 | -142.597 | 297.193 | 0.061 | 0.223 |
| -0.238 | NA | NA | NA | NA | NA | 1.54E-01 | 3 | -145.753 | 297.506 | 0.374 | 0.191 |
| -0.317 | 7.05E-10 | NA | + | NA | NA | 1.26E-01 | 7 | -142.473 | 298.946 | 1.814 | 0.093 |
| -0.154 | 5.72E-10 | NA | NA | 5.21E-04 | NA | 1.32E-01 | 5 | -144.487 | 298.974 | 1.842 | 0.092 |
| -0.257 | NA | NA | + | 1.52E-04 | NA | 1.18E-01 | 7 | -142.543 | 299.086 | 1.954 | 0.087 |
| -0.13 | NA | NA | NA | 5.31E-04 | -1.61E-03 | 1.27E-01 | 5 | -144.566 | 299.131 | 1.999 | 0.085 |
| -0.641 | NA | NA | + | NA | NA | 2.14E-01 | 6 | -170.612 | 353.225 | 0 | 0.392 |
| -0.735 | NA | NA | + | -4.66E-04 | NA | 2.22E-01 | 7 | -170.158 | 354.316 | 1.092 | 0.227 |
| -0.689 | 1.70E-09 | NA | + | NA | NA | 2.22E-01 | 7 | -170.267 | 354.534 | 1.309 | 0.204 |
| -0.541 | NA | NA | + | NA | -4.43E-02 | 2.14E-01 | 7 | -170.413 | 354.825 | 1.6 | 0.176 |
| -0.569 | NA | NA | + | NA | NA | 2.02E-01 | 6 | -166.706 | 345.412 | 0 | 0.423 |
| -0.649 | NA | NA | + | -3.90E-04 | NA | 2.10E-01 | 7 | -166.387 | 346.774 | 1.362 | 0.214 |
| -0.605 | 1.29E-09 | NA | + | NA | NA | 2.09E-01 | 7 | -166.464 | 346.927 | 1.515 | 0.198 |
| -0.514 | NA | NA | + | NA | -2.50E-02 | 2.03E-01 | 7 | -166.644 | 347.288 | 1.876 | 0.165 |
| -0.37 | NA | NA | + | NA | NA | 1.42E-01 | 6 | -151.484 | 314.967 | 0 | 0.309 |
| -0.312 | NA | NA | NA | NA | NA | 1.80E-01 | 3 | -154.982 | 315.965 | 0.998 | 0.188 |
| -0.223 | NA | NA | NA | 4.61E-04 | NA | 1.56E-01 | 4 | -154.254 | 316.509 | 1.541 | 0.143 |
| -0.4 | 1.09E-09 | NA | + | NA | NA | 1.47E-01 | 7 | -151.322 | 316.645 | 1.678 | 0.133 |
| -0.375 | NA | NA | + | NA | 2.06E-03 | 1.42E-01 | 7 | -151.483 | 316.966 | 1.999 | 0.114 |
| -0.371 | NA | NA | + | -2.22E-06 | NA | 1.42E-01 | 7 | -151.484 | 316.967 | 2 | 0.114 |
| -0.442 | NA | NA | + | NA | NA | 1.66E-01 | 6 | -138.726 | 289.452 | 0 | 0.321 |
| -0.547 | NA | NA | + | -4.68E-04 | NA | 1.75E-01 | 7 | -138.122 | 290.244 | 0.792 | 0.216 |
| -0.326 | NA | NA | + | NA | -5.45E-02 | 1.67E-01 | 7 | -138.346 | 290.692 | 1.241 | 0.173 |
| -0.478 | 1.15E-09 | NA | + | NA | NA | 1.72E-01 | 7 | -138.449 | 290.899 | 1.447 | 0.156 |
| -0.423 | NA | NA | + | -5.24E-04 | -6.44E-02 | 1.78E-01 | 8 | -137.597 | 291.193 | 1.741 | 0.134 |
| -0.537 | NA | NA | + | -9.51E-04 | -1.33E-01 | 2.37E-01 | 8 | -149.325 | 314.65 | 0 | 0.609 |
| -0.605 | 1.77E-09 | NA | + | -9.69E-04 | -1.28E-01 | 2.46E-01 | 9 | -148.769 | 315.537 | 0.887 | 0.391 |
| -0.493 | NA | NA | + | NA | NA | 1.75E-01 | 6 | -160.648 | 333.297 | 0 | 0.412 |
| -0.59 | NA | NA | + | -4.44E-04 | NA | 1.84E-01 | 7 | -160.215 | 334.43 | 1.133 | 0.234 |
| -0.529 | 1.31E-09 | NA | + | NA | NA | 1.81E-01 | 7 | -160.421 | 334.841 | 1.544 | 0.19 |
| -0.429 | NA | NA | + | NA | -2.83E-02 | 1.76E-01 | 7 | -160.573 | 335.146 | 1.849 | 0.164 |
| -0.48 | NA | NA | + | NA | NA | 1.69E-01 | 6 | -147.093 | 306.187 | 0 | 0.333 |
| -0.587 | NA | NA | + | -4.68E-04 | NA | 1.78E-01 | 7 | -146.566 | 307.132 | 0.945 | 0.207 |
| -0.357 | NA | NA | + | NA | -5.56E-02 | 1.71E-01 | 7 | -146.732 | 307.464 | 1.277 | 0.176 |
| -0.517 | 1.30E-09 | NA | + | NA | NA | 1.75E-01 | 7 | -146.812 | 307.624 | 1.437 | 0.162 |
| -0.457 | NA | NA | + | -5.17E-04 | -6.38E-02 | 1.81E-01 | 8 | -146.092 | 308.184 | 1.997 | 0.122 |
| -0.438 | NA | NA | + | NA | NA | 1.67E-01 | 6 | -145.426 | 302.852 | 0 | 0.439 |
| -0.473 | 1.15E-09 | NA | + | NA | NA | 1.72E-01 | 7 | -145.161 | 304.322 | 1.47 | 0.211 |
| -0.488 | NA | NA | + | -2.28E-04 | NA | 1.70E-01 | 7 | -145.3 | 304.601 | 1.749 | 0.183 |
| -0.401 | NA | NA | + | NA | -1.73E-02 | 1.68E-01 | 7 | -145.393 | 304.787 | 1.935 | 0.167 |
| -0.417 | NA | NA | + | NA | NA | 1.59E-01 | 6 | -145.591 | 303.183 | 0 | 0.443 |
| -0.482 | NA | NA | + | -2.74E-04 | NA | 1.65E-01 | 7 | -145.41 | 304.819 | 1.637 | 0.195 |
| -0.445 | 1.05E-09 | NA | + | NA | NA | 1.63E-01 | 7 | -145.426 | 304.852 | 1.669 | 0.192 |
| -0.376 | NA | NA | + | NA | -1.88E-02 | 1.60E-01 | 7 | -145.554 | 305.107 | 1.925 | 0.169 |
| -0.435 | NA | NA | + | NA | NA | 1.59E-01 | 6 | -148.107 | 308.214 | 0 | 0.419 |
| -0.529 | NA | NA | + | -4.09E-04 | NA | 1.68E-01 | 7 | -147.698 | 309.397 | 1.183 | 0.232 |
| -0.463 | 1.04E-09 | NA | + | NA | NA | 1.64E-01 | 7 | -147.925 | 309.85 | 1.636 | 0.185 |
| -0.38 | NA | NA | + | NA | -2.56E-02 | 1.60E-01 | 7 | -148.04 | 310.08 | 1.866 | 0.165 |
| -0.423 | NA | NA | + | NA | NA | 1.59E-01 | 6 | -141.403 | 294.805 | 0 | 0.415 |
| -0.516 | NA | NA | + | -4.30E-04 | NA | 1.66E-01 | 7 | -140.93 | 295.86 | 1.055 | 0.245 |
| -0.449 | 9.08E-10 | NA | + | NA | NA | 1.63E-01 | 7 | -141.23 | 296.46 | 1.655 | 0.182 |
| -0.386 | NA | NA | + | NA | -1.77E-02 | 1.60E-01 | 7 | -141.369 | 296.738 | 1.932 | 0.158 |
| -0.461 | NA | NA | + | NA | NA | 1.69E-01 | 6 | -153.589 | 319.178 | 0 | 0.425 |
| -0.504 | 1.57E-09 | NA | + | NA | NA | 1.75E-01 | 7 | -153.269 | 320.538 | 1.36 | 0.215 |
| -0.374 | NA | NA | + | NA | -4.00E-02 | 1.71E-01 | 7 | -153.414 | 320.828 | 1.65 | 0.186 |
| -0.511 | NA | NA | + | -2.14E-04 | NA | 1.74E-01 | 7 | -153.486 | 320.972 | 1.794 | 0.173 |
| -0.564 | NA | NA | + | NA | NA | 1.97E-01 | 6 | -153.694 | 319.389 | 0 | 0.208 |
| -0.692 | NA | NA | + | -5.93E-04 | NA | 2.07E-01 | 7 | -152.777 | 319.553 | 0.165 | 0.192 |
| -0.527 | NA | NA | + | -6.82E-04 | -8.17E-02 | 2.09E-01 | 8 | -151.999 | 319.997 | 0.609 | 0.154 |
| -0.417 | NA | NA | + | NA | -6.55E-02 | 1.98E-01 | 7 | -153.188 | 320.376 | 0.988 | 0.127 |
| -0.751 | 1.41E-09 | NA | + | -6.51E-04 | NA | 2.18E-01 | 8 | -152.255 | 320.509 | 1.121 | 0.119 |
| -0.603 | 1.15E-09 | NA | + | NA | NA | 2.05E-01 | 7 | -153.347 | 320.693 | 1.305 | 0.109 |
| -0.589 | 1.35E-09 | NA | + | -7.34E-04 | -7.92E-02 | 2.19E-01 | 9 | -151.519 | 321.038 | 1.649 | 0.091 |
| -0.379 | NA | NA | + | NA | NA | 1.44E-01 | 6 | -143.547 | 299.094 | 0 | 0.441 |
| -0.409 | 1.03E-09 | NA | + | NA | NA | 1.49E-01 | 7 | -143.359 | 300.718 | 1.624 | 0.196 |
| -0.442 | NA | NA | + | -2.74E-04 | NA | 1.50E-01 | 7 | -143.365 | 300.73 | 1.636 | 0.194 |
| -0.335 | NA | NA | + | NA | -2.00E-02 | 1.44E-01 | 7 | -143.503 | 301.006 | 1.912 | 0.169 |
| -0.429 | NA | NA | + | NA | NA | 1.58E-01 | 6 | -143.115 | 298.231 | 0 | 0.421 |
| -0.517 | NA | NA | + | -3.83E-04 | NA | 1.64E-01 | 7 | -142.754 | 299.509 | 1.278 | 0.222 |
| -0.459 | 9.38E-10 | NA | + | NA | NA | 1.64E-01 | 7 | -142.897 | 299.794 | 1.563 | 0.193 |
| -0.377 | NA | NA | + | NA | -2.33E-02 | 1.59E-01 | 7 | -143.057 | 300.113 | 1.883 | 0.164 |
| -0.41 | NA | NA | + | NA | NA | 1.57E-01 | 6 | -136.138 | 284.276 | 0 | 0.418 |
| -0.496 | NA | NA | + | -3.79E-04 | NA | 1.65E-01 | 7 | -135.759 | 285.518 | 1.242 | 0.225 |
| -0.438 | 9.12E-10 | NA | + | NA | NA | 1.62E-01 | 7 | -135.957 | 285.915 | 1.638 | 0.184 |
| -0.34 | NA | NA | + | NA | -3.20E-02 | 1.58E-01 | 7 | -136.018 | 286.036 | 1.76 | 0.173 |
| -0.498 | NA | NA | + | NA | NA | 1.79E-01 | 6 | -155.895 | 323.791 | 0 | 0.418 |
| -0.58 | NA | NA | + | -4.02E-04 | NA | 1.85E-01 | 7 | -155.53 | 325.061 | 1.27 | 0.222 |
| -0.533 | 1.26E-09 | NA | + | NA | NA | 1.85E-01 | 7 | -155.64 | 325.28 | 1.489 | 0.199 |
| -0.452 | NA | NA | + | NA | -2.14E-02 | 1.80E-01 | 7 | -155.85 | 325.7 | 1.909 | 0.161 |
| -0.41 | NA | NA | + | NA | NA | 1.54E-01 | 6 | -159.324 | 330.648 | 0 | 0.307 |
| -0.332 | NA | NA | NA | NA | NA | 1.95E-01 | 3 | -162.772 | 331.545 | 0.897 | 0.196 |
| -0.246 | NA | NA | NA | 4.55E-04 | NA | 1.72E-01 | 4 | -162.11 | 332.219 | 1.571 | 0.14 |
| -0.438 | 8.41E-10 | NA | + | NA | NA | 1.60E-01 | 7 | -159.173 | 332.345 | 1.697 | 0.131 |
| -0.419 | NA | NA | + | -3.75E-05 | NA | 1.55E-01 | 7 | -159.321 | 332.642 | 1.994 | 0.113 |
| -0.409 | NA | NA | + | NA | -3.67E-04 | 1.54E-01 | 7 | -159.324 | 332.648 | 2 | 0.113 |
| -0.491 | NA | NA | + | NA | NA | 1.79E-01 | 6 | -157.004 | 326.008 | 0 | 0.43 |
| -0.529 | 1.41E-09 | NA | + | NA | NA | 1.85E-01 | 7 | -156.743 | 327.486 | 1.478 | 0.205 |
| -0.406 | NA | NA | + | NA | -3.86E-02 | 1.80E-01 | 7 | -156.846 | 327.692 | 1.684 | 0.185 |
| -0.547 | NA | NA | + | -2.39E-04 | NA | 1.84E-01 | 7 | -156.879 | 327.758 | 1.751 | 0.179 |
| -0.486 | NA | NA | + | NA | NA | 1.75E-01 | 6 | -142.6 | 297.2 | 0 | 0.289 |
| -0.609 | NA | NA | + | -5.55E-04 | NA | 1.85E-01 | 7 | -141.794 | 297.589 | 0.389 | 0.238 |
| -0.515 | 1.07E-09 | NA | + | NA | NA | 1.79E-01 | 7 | -142.404 | 298.807 | 1.607 | 0.129 |
| -0.413 | NA | NA | + | NA | -3.39E-02 | 1.76E-01 | 7 | -142.465 | 298.93 | 1.73 | 0.122 |
| -0.646 | 1.21E-09 | NA | + | -5.74E-04 | NA | 1.91E-01 | 8 | -141.542 | 299.083 | 1.884 | 0.113 |
| -0.521 | NA | NA | + | -5.90E-04 | -4.44E-02 | 1.88E-01 | 8 | -141.563 | 299.127 | 1.927 | 0.11 |
| -0.606 | NA | NA | + | NA | NA | 2.07E-01 | 6 | -167.757 | 347.514 | 0 | 0.395 |
| -0.704 | NA | NA | + | -4.88E-04 | NA | 2.15E-01 | 7 | -167.253 | 348.506 | 0.993 | 0.24 |
| -0.65 | 1.50E-09 | NA | + | NA | NA | 2.15E-01 | 7 | -167.413 | 348.826 | 1.312 | 0.205 |
| -0.537 | NA | NA | + | NA | -3.15E-02 | 2.08E-01 | 7 | -167.659 | 349.319 | 1.805 | 0.16 |
| -0.439 | NA | NA | + | NA | NA | 1.62E-01 | 6 | -150.086 | 312.172 | 0 | 0.45 |
| -0.467 | 8.84E-10 | NA | + | NA | NA | 1.68E-01 | 7 | -149.914 | 313.829 | 1.656 | 0.197 |
| -0.493 | NA | NA | + | -2.29E-04 | NA | 1.67E-01 | 7 | -149.965 | 313.929 | 1.757 | 0.187 |
| -0.437 | NA | NA | + | NA | -1.11E-03 | 1.62E-01 | 7 | -150.086 | 314.172 | 2 | 0.166 |
| -0.532 | NA | NA | + | NA | NA | 1.85E-01 | 6 | -149.195 | 310.39 | 0 | 0.391 |
| -0.623 | NA | NA | + | -4.09E-04 | NA | 1.93E-01 | 7 | -148.794 | 311.588 | 1.199 | 0.214 |
| -0.411 | NA | NA | + | NA | -5.51E-02 | 1.87E-01 | 7 | -148.851 | 311.701 | 1.312 | 0.203 |
| -0.57 | 1.38E-09 | NA | + | NA | NA | 1.91E-01 | 7 | -148.903 | 311.806 | 1.416 | 0.192 |
| -0.379 | NA | NA | + | NA | NA | 1.49E-01 | 6 | -144.073 | 300.146 | 0 | 0.37 |
| -0.319 | NA | NA | NA | NA | NA | 1.85E-01 | 3 | -147.76 | 301.52 | 1.374 | 0.186 |
| -0.404 | 9.74E-10 | NA | + | NA | NA | 1.52E-01 | 7 | -143.935 | 301.87 | 1.724 | 0.156 |
| -0.423 | NA | NA | + | -1.86E-04 | NA | 1.53E-01 | 7 | -143.989 | 301.979 | 1.833 | 0.148 |
| -0.342 | NA | NA | + | NA | -1.72E-02 | 1.49E-01 | 7 | -144.043 | 302.086 | 1.94 | 0.14 |
| -0.373 | NA | NA | + | NA | NA | 1.45E-01 | 6 | -144.302 | 300.604 | 0 | 0.319 |
| -0.302 | NA | NA | NA | NA | NA | 1.77E-01 | 3 | -147.905 | 301.811 | 1.206 | 0.174 |
| -0.403 | 7.16E-10 | NA | + | NA | NA | 1.52E-01 | 7 | -144.101 | 302.202 | 1.598 | 0.143 |
| -0.232 | NA | NA | NA | 4.14E-04 | NA | 1.60E-01 | 4 | -147.228 | 302.457 | 1.852 | 0.126 |
| -0.388 | NA | NA | + | -7.08E-05 | NA | 1.46E-01 | 7 | -144.29 | 302.58 | 1.975 | 0.119 |
| -0.392 | NA | NA | + | NA | 8.93E-03 | 1.45E-01 | 7 | -144.294 | 302.588 | 1.984 | 0.118 |
| -0.535 | NA | NA | + | NA | NA | 1.92E-01 | 6 | -161.288 | 334.576 | 0 | 0.415 |
| -0.626 | NA | NA | + | -4.56E-04 | NA | 1.99E-01 | 7 | -160.847 | 335.695 | 1.118 | 0.237 |
| -0.572 | 1.27E-09 | NA | + | NA | NA | 1.98E-01 | 7 | -161.046 | 336.091 | 1.515 | 0.195 |
| -0.533 | NA | NA | + | NA | -8.99E-04 | 1.92E-01 | 7 | -161.288 | 336.576 | 2 | 0.153 |
| -0.505 | NA | NA | + | NA | NA | 1.77E-01 | 6 | -150.675 | 313.35 | 0 | 0.285 |
| -0.628 | NA | NA | + | -5.77E-04 | NA | 1.86E-01 | 7 | -149.873 | 313.746 | 0.396 | 0.234 |
| -0.538 | 1.14E-09 | NA | + | NA | NA | 1.83E-01 | 7 | -150.435 | 314.871 | 1.521 | 0.133 |
| -0.425 | NA | NA | + | NA | -3.55E-02 | 1.77E-01 | 7 | -150.543 | 315.086 | 1.737 | 0.12 |
| -0.675 | 1.34E-09 | NA | + | -6.10E-04 | NA | 1.93E-01 | 8 | -149.544 | 315.088 | 1.739 | 0.12 |
| -0.533 | NA | NA | + | -6.12E-04 | -4.61E-02 | 1.87E-01 | 8 | -149.651 | 315.303 | 1.953 | 0.108 |
| -0.451 | NA | NA | + | NA | NA | 1.63E-01 | 6 | -147.404 | 306.807 | 0 | 0.415 |
| -0.535 | NA | NA | + | -3.68E-04 | NA | 1.70E-01 | 7 | -147.069 | 308.137 | 1.33 | 0.214 |
| -0.483 | 9.47E-10 | NA | + | NA | NA | 1.70E-01 | 7 | -147.162 | 308.325 | 1.518 | 0.194 |
| -0.372 | NA | NA | + | NA | -3.62E-02 | 1.65E-01 | 7 | -147.258 | 308.516 | 1.708 | 0.177 |
| -0.452 | NA | NA | + | NA | NA | 1.68E-01 | 6 | -151.919 | 315.838 | 0 | 0.45 |
| -0.484 | 9.08E-10 | NA | + | NA | NA | 1.75E-01 | 7 | -151.706 | 317.413 | 1.575 | 0.205 |
| -0.491 | NA | NA | + | -1.77E-04 | NA | 1.72E-01 | 7 | -151.846 | 317.692 | 1.854 | 0.178 |
| -0.431 | NA | NA | + | NA | -9.30E-03 | 1.68E-01 | 7 | -151.91 | 317.821 | 1.983 | 0.167 |
| -0.514 | NA | NA | + | NA | NA | 1.81E-01 | 6 | -152.525 | 317.051 | 0 | 0.385 |
| -0.619 | NA | NA | + | -4.97E-04 | NA | 1.88E-01 | 7 | -151.931 | 317.862 | 0.811 | 0.256 |
| -0.549 | 1.31E-09 | NA | + | NA | NA | 1.86E-01 | 7 | -152.237 | 318.474 | 1.424 | 0.189 |
| -0.426 | NA | NA | + | NA | -4.01E-02 | 1.83E-01 | 7 | -152.34 | 318.681 | 1.63 | 0.17 |
| -0.503 | NA | NA | + | NA | NA | 1.80E-01 | 6 | -146.49 | 304.981 | 0 | 0.396 |
| -0.601 | NA | NA | + | -4.50E-04 | NA | 1.88E-01 | 7 | -146.006 | 306.013 | 1.032 | 0.237 |
| -0.538 | 1.39E-09 | NA | + | NA | NA | 1.85E-01 | 7 | -146.234 | 306.468 | 1.487 | 0.188 |
| -0.408 | NA | NA | + | NA | -4.23E-02 | 1.81E-01 | 7 | -146.288 | 306.577 | 1.596 | 0.178 |
| -0.421 | NA | NA | + | NA | NA | 1.56E-01 | 6 | -163.8 | 339.6 | 0 | 0.333 |
| -0.35 | NA | NA | NA | NA | NA | 2.00E-01 | 3 | -167.58 | 341.159 | 1.559 | 0.153 |
| -0.452 | 8.82E-10 | NA | + | NA | NA | 1.63E-01 | 7 | -163.632 | 341.265 | 1.665 | 0.145 |
| -0.251 | NA | NA | NA | 5.19E-04 | NA | 1.73E-01 | 4 | -166.796 | 341.592 | 1.992 | 0.123 |
| -0.413 | NA | NA | + | 3.42E-05 | NA | 1.55E-01 | 7 | -163.798 | 341.595 | 1.995 | 0.123 |
| -0.415 | NA | NA | + | NA | -2.87E-03 | 1.56E-01 | 7 | -163.799 | 341.599 | 1.999 | 0.123 |
| -0.446 | NA | NA | + | NA | NA | 1.58E-01 | 6 | -143.464 | 298.929 | 0 | 0.334 |
| -0.547 | NA | NA | + | -4.40E-04 | NA | 1.68E-01 | 7 | -142.969 | 299.937 | 1.008 | 0.202 |
| -0.319 | NA | NA | + | NA | -5.67E-02 | 1.60E-01 | 7 | -143.067 | 300.133 | 1.205 | 0.183 |
| -0.477 | 1.08E-09 | NA | + | NA | NA | 1.64E-01 | 7 | -143.255 | 300.51 | 1.582 | 0.152 |
| -0.412 | NA | NA | + | -5.06E-04 | -6.71E-02 | 1.70E-01 | 8 | -142.419 | 300.839 | 1.91 | 0.129 |
| -0.465 | NA | NA | + | NA | NA | 1.68E-01 | 6 | -151.048 | 314.096 | 0 | 0.419 |
| -0.559 | NA | NA | + | -4.16E-04 | NA | 1.76E-01 | 7 | -150.639 | 315.279 | 1.183 | 0.232 |
| -0.496 | 1.15E-09 | NA | + | NA | NA | 1.73E-01 | 7 | -150.851 | 315.702 | 1.606 | 0.188 |
| -0.417 | NA | NA | + | NA | -2.18E-02 | 1.69E-01 | 7 | -150.998 | 315.997 | 1.901 | 0.162 |
| -0.445 | NA | NA | + | NA | NA | 1.62E-01 | 6 | -156.01 | 324.019 | 0 | 0.454 |
| -0.476 | 1.07E-09 | NA | + | NA | NA | 1.67E-01 | 7 | -155.827 | 325.655 | 1.635 | 0.2 |
| -0.484 | NA | NA | + | -1.62E-04 | NA | 1.66E-01 | 7 | -155.953 | 325.906 | 1.886 | 0.177 |
| -0.425 | NA | NA | + | NA | -9.28E-03 | 1.62E-01 | 7 | -156.001 | 326.003 | 1.983 | 0.168 |
| -0.495 | NA | NA | + | NA | NA | 1.76E-01 | 6 | -149.104 | 310.208 | 0 | 0.389 |
| -0.369 | NA | NA | + | NA | -5.66E-02 | 1.77E-01 | 7 | -148.725 | 311.45 | 1.241 | 0.209 |
| -0.537 | 1.27E-09 | NA | + | NA | NA | 1.84E-01 | 7 | -148.761 | 311.521 | 1.313 | 0.202 |
| -0.578 | NA | NA | + | -3.72E-04 | NA | 1.83E-01 | 7 | -148.767 | 311.534 | 1.325 | 0.2 |
| -0.451 | NA | NA | + | NA | NA | 1.62E-01 | 6 | -154.227 | 320.454 | 0 | 0.439 |
| -0.485 | 1.02E-09 | NA | + | NA | NA | 1.69E-01 | 7 | -153.998 | 321.996 | 1.542 | 0.203 |
| -0.506 | NA | NA | + | -2.48E-04 | NA | 1.66E-01 | 7 | -154.087 | 322.174 | 1.719 | 0.186 |
| -0.396 | NA | NA | + | NA | -2.49E-02 | 1.62E-01 | 7 | -154.163 | 322.326 | 1.872 | 0.172 |
| -0.513 | NA | NA | + | NA | NA | 1.76E-01 | 6 | -153.047 | 318.094 | 0 | 0.326 |
| -0.635 | NA | NA | + | -5.60E-04 | NA | 1.86E-01 | 7 | -152.304 | 318.608 | 0.514 | 0.252 |
| -0.548 | 1.13E-09 | NA | + | NA | NA | 1.82E-01 | 7 | -152.795 | 319.589 | 1.495 | 0.154 |
| -0.687 | 1.37E-09 | NA | + | -6.07E-04 | NA | 1.95E-01 | 8 | -151.931 | 319.862 | 1.768 | 0.135 |
| -0.446 | NA | NA | + | NA | -3.05E-02 | 1.76E-01 | 7 | -152.95 | 319.901 | 1.807 | 0.132 |
| -0.451 | NA | NA | + | NA | NA | 1.65E-01 | 6 | -148.363 | 308.726 | 0 | 0.33 |
| -0.572 | NA | NA | + | -5.23E-04 | NA | 1.74E-01 | 7 | -147.696 | 309.393 | 0.667 | 0.236 |
| -0.488 | 1.21E-09 | NA | + | NA | NA | 1.71E-01 | 7 | -148.1 | 310.2 | 1.474 | 0.158 |
| -0.352 | NA | NA | + | NA | -4.52E-02 | 1.65E-01 | 7 | -148.134 | 310.267 | 1.541 | 0.153 |
| -0.461 | NA | NA | + | -5.73E-04 | -5.61E-02 | 1.76E-01 | 8 | -147.345 | 310.69 | 1.964 | 0.124 |
| -0.573 | NA | NA | + | NA | NA | 1.94E-01 | 6 | -151.128 | 314.255 | 0 | 0.247 |
| -0.693 | NA | NA | + | -5.84E-04 | NA | 2.04E-01 | 7 | -150.25 | 314.499 | 0.244 | 0.219 |
| -0.534 | NA | NA | + | -6.57E-04 | -7.68E-02 | 2.05E-01 | 8 | -149.564 | 315.127 | 0.872 | 0.16 |
| -0.43 | NA | NA | + | NA | -6.30E-02 | 1.94E-01 | 7 | -150.662 | 315.325 | 1.07 | 0.145 |
| -0.611 | 1.38E-09 | NA | + | NA | NA | 2.00E-01 | 7 | -150.844 | 315.687 | 1.432 | 0.121 |
| -0.734 | 1.43E-09 | NA | + | -5.91E-04 | NA | 2.10E-01 | 8 | -149.94 | 315.88 | 1.624 | 0.11 |
| -0.45 | NA | NA | + | NA | NA | 1.63E-01 | 6 | -148.321 | 308.641 | 0 | 0.403 |
| -0.537 | NA | NA | + | -3.86E-04 | NA | 1.70E-01 | 7 | -147.96 | 309.92 | 1.279 | 0.213 |
| -0.486 | 1.29E-09 | NA | + | NA | NA | 1.69E-01 | 7 | -148.037 | 310.074 | 1.432 | 0.197 |
| -0.351 | NA | NA | + | NA | -4.61E-02 | 1.65E-01 | 7 | -148.088 | 310.177 | 1.535 | 0.187 |
| -0.519 | NA | NA | + | NA | NA | 1.80E-01 | 6 | -162.997 | 337.995 | 0 | 0.419 |
| -0.56 | 1.57E-09 | NA | + | NA | NA | 1.86E-01 | 7 | -162.694 | 339.387 | 1.392 | 0.209 |
| -0.417 | NA | NA | + | NA | -4.62E-02 | 1.81E-01 | 7 | -162.782 | 339.564 | 1.569 | 0.191 |
| -0.578 | NA | NA | + | -2.72E-04 | NA | 1.84E-01 | 7 | -162.84 | 339.68 | 1.686 | 0.18 |
| -0.508 | NA | NA | + | NA | NA | 1.85E-01 | 6 | -154.927 | 321.854 | 0 | 0.393 |
| -0.604 | NA | NA | + | -4.48E-04 | NA | 1.94E-01 | 7 | -154.445 | 322.89 | 1.036 | 0.234 |
| -0.543 | 1.23E-09 | NA | + | NA | NA | 1.91E-01 | 7 | -154.666 | 323.332 | 1.477 | 0.188 |
| -0.405 | NA | NA | + | NA | -4.71E-02 | 1.87E-01 | 7 | -154.675 | 323.35 | 1.496 | 0.186 |
| -0.504 | NA | NA | + | NA | NA | 1.76E-01 | 6 | -153.767 | 319.533 | 0 | 0.419 |
| -0.584 | NA | NA | + | -3.38E-04 | NA | 1.85E-01 | 7 | -153.504 | 321.009 | 1.475 | 0.2 |
| -0.542 | 1.33E-09 | NA | + | NA | NA | 1.82E-01 | 7 | -153.528 | 321.056 | 1.523 | 0.196 |
| -0.411 | NA | NA | + | NA | -4.14E-02 | 1.77E-01 | 7 | -153.588 | 321.175 | 1.642 | 0.184 |
| -0.396 | NA | NA | + | NA | NA | 1.53E-01 | 6 | -135.533 | 283.065 | 0 | 0.413 |
| -0.499 | NA | NA | + | -4.24E-04 | NA | 1.61E-01 | 7 | -135.058 | 284.115 | 1.05 | 0.244 |
| -0.422 | 8.56E-10 | NA | + | NA | NA | 1.57E-01 | 7 | -135.37 | 284.74 | 1.675 | 0.179 |
| -0.342 | NA | NA | + | NA | -2.54E-02 | 1.54E-01 | 7 | -135.457 | 284.914 | 1.849 | 0.164 |
| -0.475 | NA | NA | + | NA | NA | 1.72E-01 | 6 | -151.332 | 314.665 | 0 | 0.42 |
| -0.55 | NA | NA | + | -3.50E-04 | NA | 1.79E-01 | 7 | -151.053 | 316.107 | 1.442 | 0.204 |
| -0.511 | 1.37E-09 | NA | + | NA | NA | 1.78E-01 | 7 | -151.066 | 316.132 | 1.467 | 0.201 |
| -0.402 | NA | NA | + | NA | -3.41E-02 | 1.73E-01 | 7 | -151.207 | 316.413 | 1.748 | 0.175 |
| -0.609 | NA | NA | + | NA | NA | 2.01E-01 | 6 | -157.76 | 327.519 | 0 | 0.258 |
| -0.429 | NA | NA | + | NA | -7.70E-02 | 2.00E-01 | 7 | -157.054 | 328.108 | 0.589 | 0.192 |
| -0.711 | NA | NA | + | -4.79E-04 | NA | 2.09E-01 | 7 | -157.216 | 328.432 | 0.913 | 0.163 |
| -0.522 | NA | NA | + | -5.78E-04 | -8.98E-02 | 2.10E-01 | 8 | -156.272 | 328.545 | 1.026 | 0.154 |
| -0.653 | 1.37E-09 | NA | + | NA | NA | 2.09E-01 | 7 | -157.391 | 328.783 | 1.264 | 0.137 |
| -0.477 | 1.24E-09 | NA | + | NA | -7.35E-02 | 2.07E-01 | 8 | -156.749 | 329.498 | 1.979 | 0.096 |
| -0.69 | NA | NA | + | -9.49E-04 | -9.26E-02 | 2.43E-01 | 8 | -156.877 | 329.754 | 0 | 0.252 |
| -0.88 | NA | NA | + | -8.38E-04 | NA | 2.42E-01 | 7 | -157.892 | 329.783 | 0.029 | 0.248 |
| -0.954 | 1.74E-09 | NA | + | -9.13E-04 | NA | 2.56E-01 | 8 | -157.12 | 330.241 | 0.487 | 0.198 |
| -0.767 | 1.65E-09 | NA | + | -1.02E-03 | -8.91E-02 | 2.56E-01 | 9 | -156.175 | 330.35 | 0.596 | 0.187 |
| -0.722 | NA | NA | + | NA | NA | 2.33E-01 | 6 | -159.662 | 331.324 | 1.57 | 0.115 |
| -0.434 | NA | NA | + | NA | NA | 1.59E-01 | 6 | -160.323 | 332.646 | 0 | 0.324 |
| -0.349 | NA | NA | NA | NA | NA | 1.97E-01 | 3 | -164.002 | 334.003 | 1.358 | 0.164 |
| -0.465 | 1.04E-09 | NA | + | NA | NA | 1.65E-01 | 7 | -160.135 | 334.269 | 1.623 | 0.144 |
| -0.265 | NA | NA | NA | 4.98E-04 | NA | 1.76E-01 | 4 | -163.247 | 334.494 | 1.848 | 0.129 |
| -0.44 | NA | NA | + | NA | 2.43E-03 | 1.59E-01 | 7 | -160.322 | 334.645 | 1.999 | 0.119 |
| -0.432 | NA | NA | + | 1.19E-05 | NA | 1.59E-01 | 7 | -160.323 | 334.645 | 1.999 | 0.119 |
| -0.434 | NA | NA | + | NA | NA | 1.61E-01 | 6 | -157.65 | 327.3 | 0 | 0.388 |
| -0.463 | 1.03E-09 | NA | + | NA | NA | 1.66E-01 | 7 | -157.489 | 328.977 | 1.677 | 0.168 |
| -0.339 | NA | NA | NA | NA | NA | 1.95E-01 | 3 | -161.59 | 329.18 | 1.88 | 0.151 |
| -0.47 | NA | NA | + | -1.58E-04 | NA | 1.64E-01 | 7 | -157.598 | 329.196 | 1.896 | 0.15 |
| -0.442 | NA | NA | + | NA | 3.34E-03 | 1.61E-01 | 7 | -157.649 | 329.298 | 1.998 | 0.143 |
| -0.352 | NA | NA | + | NA | NA | 1.39E-01 | 6 | -140.995 | 293.989 | 0 | 0.253 |
| -0.284 | NA | NA | NA | NA | NA | 1.71E-01 | 3 | -144.162 | 294.324 | 0.335 | 0.214 |
| -0.214 | NA | NA | NA | 3.90E-04 | NA | 1.53E-01 | 4 | -143.581 | 295.162 | 1.172 | 0.141 |
| -0.38 | 8.05E-10 | NA | + | NA | NA | 1.44E-01 | 7 | -140.822 | 295.643 | 1.654 | 0.111 |
| -0.314 | 8.53E-10 | NA | NA | NA | NA | 1.77E-01 | 4 | -143.976 | 295.951 | 1.962 | 0.095 |
| -0.369 | NA | NA | + | -6.77E-05 | NA | 1.40E-01 | 7 | -140.984 | 295.968 | 1.978 | 0.094 |
| -0.37 | NA | NA | + | NA | 8.02E-03 | 1.38E-01 | 7 | -140.988 | 295.976 | 1.987 | 0.094 |
| -0.442 | NA | NA | + | NA | NA | 1.70E-01 | 6 | -134.742 | 281.483 | 0 | 0.26 |
| -0.548 | NA | NA | + | -4.88E-04 | NA | 1.80E-01 | 7 | -134.072 | 282.145 | 0.661 | 0.187 |
| -0.302 | NA | NA | + | NA | -6.48E-02 | 1.71E-01 | 7 | -134.162 | 282.324 | 0.841 | 0.171 |
| -0.401 | NA | NA | + | -5.49E-04 | -7.40E-02 | 1.82E-01 | 8 | -133.319 | 282.637 | 1.154 | 0.146 |
| -0.479 | 1.18E-09 | NA | + | NA | NA | 1.77E-01 | 7 | -134.42 | 282.841 | 1.357 | 0.132 |
| -0.598 | 1.35E-09 | NA | + | -5.26E-04 | NA | 1.89E-01 | 8 | -133.647 | 283.293 | 1.81 | 0.105 |
| -0.563 | NA | NA | + | NA | NA | 1.94E-01 | 6 | -158.254 | 328.508 | 0 | 0.393 |
| -0.655 | NA | NA | + | -4.43E-04 | NA | 2.01E-01 | 7 | -157.805 | 329.61 | 1.102 | 0.227 |
| -0.604 | 1.43E-09 | NA | + | NA | NA | 2.01E-01 | 7 | -157.93 | 329.86 | 1.353 | 0.2 |
| -0.462 | NA | NA | + | NA | -4.48E-02 | 1.94E-01 | 7 | -158.037 | 330.074 | 1.567 | 0.18 |
| -0.434 | NA | NA | + | NA | NA | 1.65E-01 | 6 | -158.764 | 329.529 | 0 | 0.384 |
| -0.467 | 1.17E-09 | NA | + | NA | NA | 1.70E-01 | 7 | -158.558 | 331.116 | 1.587 | 0.173 |
| -0.353 | NA | NA | NA | NA | NA | 2.02E-01 | 3 | -162.684 | 331.369 | 1.84 | 0.153 |
| -0.469 | NA | NA | + | -1.51E-04 | NA | 1.68E-01 | 7 | -158.717 | 331.434 | 1.905 | 0.148 |
| -0.414 | NA | NA | + | NA | -9.34E-03 | 1.65E-01 | 7 | -158.756 | 331.511 | 1.983 | 0.142 |
| -0.533 | NA | NA | + | NA | NA | 1.90E-01 | 6 | -167.263 | 346.525 | 0 | 0.445 |
| -0.57 | 1.32E-09 | NA | + | NA | NA | 1.96E-01 | 7 | -167.029 | 348.059 | 1.533 | 0.207 |
| -0.584 | NA | NA | + | -2.37E-04 | NA | 1.95E-01 | 7 | -167.149 | 348.298 | 1.773 | 0.184 |
| -0.544 | NA | NA | + | NA | 4.92E-03 | 1.90E-01 | 7 | -167.26 | 348.521 | 1.996 | 0.164 |
| -0.616 | NA | NA | + | NA | NA | 2.08E-01 | 6 | -162.546 | 337.092 | 0 | 0.263 |
| -0.722 | NA | NA | + | -5.15E-04 | NA | 2.18E-01 | 7 | -161.929 | 337.859 | 0.767 | 0.18 |
| -0.457 | NA | NA | + | NA | -6.83E-02 | 2.07E-01 | 7 | -162.04 | 338.079 | 0.988 | 0.161 |
| -0.663 | 1.48E-09 | NA | + | NA | NA | 2.18E-01 | 7 | -162.122 | 338.244 | 1.152 | 0.148 |
| -0.55 | NA | NA | + | -6.00E-04 | -8.17E-02 | 2.18E-01 | 8 | -161.216 | 338.432 | 1.34 | 0.135 |
| -0.785 | 1.68E-09 | NA | + | -5.62E-04 | NA | 2.30E-01 | 8 | -161.39 | 338.78 | 1.689 | 0.113 |
| -0.423 | NA | NA | + | NA | NA | 1.56E-01 | 6 | -153.759 | 319.518 | 0 | 0.452 |
| -0.482 | NA | NA | + | -2.53E-04 | NA | 1.61E-01 | 7 | -153.619 | 321.237 | 1.72 | 0.191 |
| -0.448 | 8.82E-10 | NA | + | NA | NA | 1.60E-01 | 7 | -153.629 | 321.258 | 1.74 | 0.189 |
| -0.441 | NA | NA | + | NA | 8.32E-03 | 1.55E-01 | 7 | -153.752 | 321.504 | 1.987 | 0.167 |
| -0.355 | NA | NA | + | NA | NA | 1.41E-01 | 6 | -142.602 | 297.204 | 0 | 0.299 |
| -0.285 | NA | NA | NA | NA | NA | 1.75E-01 | 3 | -145.965 | 297.929 | 0.725 | 0.208 |
| -0.207 | NA | NA | NA | 3.95E-04 | NA | 1.54E-01 | 4 | -145.34 | 298.679 | 1.475 | 0.143 |
| -0.379 | 8.12E-10 | NA | + | NA | NA | 1.45E-01 | 7 | -142.468 | 298.937 | 1.733 | 0.126 |
| -0.381 | NA | NA | + | -1.03E-04 | NA | 1.43E-01 | 7 | -142.577 | 299.153 | 1.949 | 0.113 |
| -0.341 | NA | NA | + | NA | -6.62E-03 | 1.41E-01 | 7 | -142.597 | 299.195 | 1.991 | 0.111 |
| -0.408 | NA | NA | + | NA | NA | 1.56E-01 | 6 | -149.377 | 310.755 | 0 | 0.388 |
| -0.434 | 1.02E-09 | NA | + | NA | NA | 1.60E-01 | 7 | -149.242 | 312.485 | 1.73 | 0.164 |
| -0.452 | NA | NA | + | -1.85E-04 | NA | 1.60E-01 | 7 | -149.299 | 312.599 | 1.844 | 0.155 |
| -0.35 | NA | NA | NA | NA | NA | 1.94E-01 | 3 | -153.327 | 312.655 | 1.9 | 0.15 |
| -0.417 | NA | NA | + | NA | 4.30E-03 | 1.56E-01 | 7 | -149.376 | 312.751 | 1.996 | 0.143 |
| -0.527 | NA | NA | + | NA | NA | 1.81E-01 | 6 | -155.567 | 323.133 | 0 | 0.441 |
| -0.563 | 1.33E-09 | NA | + | NA | NA | 1.87E-01 | 7 | -155.336 | 324.673 | 1.54 | 0.204 |
| -0.582 | NA | NA | + | -2.55E-04 | NA | 1.85E-01 | 7 | -155.423 | 324.845 | 1.712 | 0.188 |
| -0.49 | NA | NA | + | NA | -1.66E-02 | 1.82E-01 | 7 | -155.539 | 325.079 | 1.945 | 0.167 |
| -0.541 | NA | NA | + | NA | NA | 1.86E-01 | 6 | -160.658 | 333.317 | 0 | 0.423 |
| -0.624 | NA | NA | + | -3.73E-04 | NA | 1.93E-01 | 7 | -160.355 | 334.711 | 1.394 | 0.211 |
| -0.578 | 1.10E-09 | NA | + | NA | NA | 1.94E-01 | 7 | -160.417 | 334.834 | 1.517 | 0.198 |
| -0.477 | NA | NA | + | NA | -2.86E-02 | 1.86E-01 | 7 | -160.578 | 335.157 | 1.84 | 0.169 |
| -0.438 | NA | NA | + | NA | NA | 1.64E-01 | 6 | -146.116 | 304.232 | 0 | 0.424 |
| -0.532 | NA | NA | + | -3.89E-04 | NA | 1.73E-01 | 7 | -145.742 | 305.483 | 1.251 | 0.227 |
| -0.465 | 9.33E-10 | NA | + | NA | NA | 1.69E-01 | 7 | -145.956 | 305.911 | 1.679 | 0.183 |
| -0.385 | NA | NA | + | NA | -2.40E-02 | 1.65E-01 | 7 | -146.055 | 306.111 | 1.879 | 0.166 |
| -0.405 | NA | NA | + | NA | NA | 1.52E-01 | 6 | -163.218 | 338.436 | 0 | 0.204 |
| -0.33 | NA | NA | NA | NA | NA | 1.90E-01 | 3 | -166.246 | 338.492 | 0.055 | 0.198 |
| -0.221 | NA | NA | NA | 5.64E-04 | NA | 1.62E-01 | 4 | -165.268 | 338.536 | 0.1 | 0.194 |
| -0.483 | NA | NA | + | NA | 3.58E-02 | 1.50E-01 | 7 | -163.111 | 340.221 | 1.785 | 0.084 |
| -0.429 | 8.81E-10 | NA | + | NA | NA | 1.56E-01 | 7 | -163.122 | 340.243 | 1.807 | 0.083 |
| -0.357 | 9.76E-10 | NA | NA | NA | NA | 1.94E-01 | 4 | -166.131 | 340.263 | 1.826 | 0.082 |
| -0.246 | 8.83E-10 | NA | NA | 5.58E-04 | NA | 1.66E-01 | 5 | -165.173 | 340.346 | 1.91 | 0.078 |
| -0.375 | NA | NA | + | 1.24E-04 | NA | 1.49E-01 | 7 | -163.189 | 340.378 | 1.942 | 0.077 |
| -0.537 | NA | NA | + | NA | NA | 1.84E-01 | 6 | -150.715 | 313.429 | 0 | 0.406 |
| -0.621 | NA | NA | + | -3.72E-04 | NA | 1.93E-01 | 7 | -150.38 | 314.76 | 1.331 | 0.209 |
| -0.578 | 1.51E-09 | NA | + | NA | NA | 1.90E-01 | 7 | -150.418 | 314.837 | 1.408 | 0.201 |
| -0.438 | NA | NA | + | NA | -4.41E-02 | 1.85E-01 | 7 | -150.505 | 315.011 | 1.582 | 0.184 |
| -0.37 | NA | NA | + | NA | NA | 1.44E-01 | 6 | -148.751 | 309.503 | 0 | 0.385 |
| -0.396 | 8.76E-10 | NA | + | NA | NA | 1.49E-01 | 7 | -148.602 | 311.203 | 1.7 | 0.164 |
| -0.422 | NA | NA | + | -2.28E-04 | NA | 1.48E-01 | 7 | -148.633 | 311.265 | 1.762 | 0.159 |
| -0.326 | NA | NA | NA | NA | NA | 1.88E-01 | 3 | -152.698 | 311.395 | 1.892 | 0.149 |
| -0.366 | NA | NA | + | NA | -1.96E-03 | 1.44E-01 | 7 | -148.751 | 311.502 | 1.999 | 0.142 |
| -0.39 | NA | NA | + | NA | NA | 1.51E-01 | 6 | -140.846 | 293.693 | 0 | 0.379 |
| -0.446 | NA | NA | + | -2.48E-04 | NA | 1.56E-01 | 7 | -140.692 | 295.385 | 1.692 | 0.163 |
| -0.416 | 9.46E-10 | NA | + | NA | NA | 1.55E-01 | 7 | -140.7 | 295.4 | 1.707 | 0.162 |
| -0.348 | NA | NA | NA | NA | NA | 1.91E-01 | 3 | -144.731 | 295.463 | 1.77 | 0.157 |
| -0.381 | NA | NA | + | NA | -4.08E-03 | 1.51E-01 | 7 | -140.845 | 295.689 | 1.996 | 0.14 |
| -0.483 | NA | NA | + | NA | NA | 1.73E-01 | 6 | -143.068 | 298.135 | 0 | 0.255 |
| -0.611 | NA | NA | + | -5.78E-04 | NA | 1.85E-01 | 7 | -142.179 | 298.357 | 0.222 | 0.229 |
| -0.474 | NA | NA | + | -6.30E-04 | -6.69E-02 | 1.87E-01 | 8 | -141.632 | 299.264 | 1.129 | 0.145 |
| -0.358 | NA | NA | + | NA | -5.63E-02 | 1.73E-01 | 7 | -142.681 | 299.362 | 1.227 | 0.138 |
| -0.518 | 1.29E-09 | NA | + | NA | NA | 1.78E-01 | 7 | -142.819 | 299.637 | 1.502 | 0.121 |
| -0.651 | 1.37E-09 | NA | + | -5.89E-04 | NA | 1.91E-01 | 8 | -141.894 | 299.788 | 1.652 | 0.112 |
| -0.43 | NA | NA | + | NA | NA | 1.59E-01 | 6 | -164.931 | 341.862 | 0 | 0.39 |
| -0.462 | 9.95E-10 | NA | + | NA | NA | 1.65E-01 | 7 | -164.747 | 343.494 | 1.632 | 0.173 |
| -0.369 | NA | NA | NA | NA | NA | 2.00E-01 | 3 | -168.891 | 343.782 | 1.92 | 0.149 |
| -0.413 | NA | NA | + | NA | -7.65E-03 | 1.59E-01 | 7 | -164.926 | 343.851 | 1.989 | 0.144 |
| -0.43 | NA | NA | + | 6.96E-07 | NA | 1.59E-01 | 7 | -164.931 | 343.862 | 2 | 0.144 |
| -0.57 | NA | NA | + | -8.39E-04 | -9.42E-02 | 2.22E-01 | 8 | -154.564 | 325.127 | 0 | 0.212 |
| -0.755 | NA | NA | + | -7.44E-04 | NA | 2.19E-01 | 7 | -155.574 | 325.148 | 0.021 | 0.21 |
| -0.601 | NA | NA | + | NA | NA | 2.06E-01 | 6 | -156.932 | 325.863 | 0.736 | 0.147 |
| -0.811 | 1.75E-09 | NA | + | -7.74E-04 | NA | 2.28E-01 | 8 | -155.067 | 326.135 | 1.007 | 0.128 |
| -0.629 | 1.58E-09 | NA | + | -8.62E-04 | -8.99E-02 | 2.30E-01 | 9 | -154.145 | 326.29 | 1.163 | 0.119 |
| -0.435 | NA | NA | + | NA | -7.61E-02 | 2.08E-01 | 7 | -156.271 | 326.542 | 1.414 | 0.105 |
| -0.645 | 1.54E-09 | NA | + | NA | NA | 2.14E-01 | 7 | -156.54 | 327.081 | 1.953 | 0.08 |
| -0.515 | NA | NA | + | NA | NA | 1.85E-01 | 6 | -163.927 | 339.854 | 0 | 0.445 |
| -0.552 | 1.16E-09 | NA | + | NA | NA | 1.92E-01 | 7 | -163.683 | 341.366 | 1.512 | 0.209 |
| -0.56 | NA | NA | + | -2.10E-04 | NA | 1.89E-01 | 7 | -163.837 | 341.674 | 1.82 | 0.179 |
| -0.485 | NA | NA | + | NA | -1.40E-02 | 1.86E-01 | 7 | -163.908 | 341.816 | 1.963 | 0.167 |
| -0.472 | NA | NA | + | NA | NA | 1.74E-01 | 6 | -152.083 | 316.166 | 0 | 0.43 |
| -0.541 | NA | NA | + | -3.26E-04 | NA | 1.80E-01 | 7 | -151.832 | 317.664 | 1.499 | 0.203 |
| -0.504 | 9.51E-10 | NA | + | NA | NA | 1.81E-01 | 7 | -151.855 | 317.711 | 1.545 | 0.199 |
| -0.417 | NA | NA | + | NA | -2.48E-02 | 1.74E-01 | 7 | -152.02 | 318.04 | 1.874 | 0.168 |
| -0.66 | NA | NA | + | -8.06E-04 | -9.59E-02 | 2.47E-01 | 8 | -164.909 | 345.819 | 0 | 0.202 |
| -0.86 | NA | NA | + | -6.97E-04 | NA | 2.48E-01 | 7 | -166.022 | 346.045 | 0.226 | 0.18 |
| -0.729 | NA | NA | + | NA | NA | 2.37E-01 | 6 | -167.115 | 346.231 | 0.412 | 0.164 |
| -0.547 | NA | NA | + | NA | -7.91E-02 | 2.35E-01 | 7 | -166.352 | 346.703 | 0.884 | 0.13 |
| -0.926 | 2.28E-09 | NA | + | -7.21E-04 | NA | 2.58E-01 | 8 | -165.461 | 346.922 | 1.103 | 0.116 |
| -0.729 | 1.93E-09 | NA | + | -8.18E-04 | -8.91E-02 | 2.55E-01 | 9 | -164.508 | 347.016 | 1.197 | 0.111 |
| -0.786 | 2.12E-09 | NA | + | NA | NA | 2.45E-01 | 7 | -166.636 | 347.272 | 1.453 | 0.098 |
| -0.114 | NA | NA | NA | 5.23E-04 | NA | 1.22E-01 | 4 | -148.752 | 305.504 | 0 | 0.193 |
| -0.223 | NA | NA | NA | NA | NA | 1.53E-01 | 3 | -149.764 | 305.527 | 0.024 | 0.191 |
| -0.291 | NA | NA | + | NA | NA | 1.16E-01 | 6 | -147.02 | 306.039 | 0.536 | 0.148 |
| 0.224 | NA | NA | NA | 7.50E-04 | NA | NA | 3 | -150.505 | 307.01 | 1.506 | 0.091 |
| -0.247 | 9.07E-10 | NA | NA | NA | NA | 1.57E-01 | 4 | -149.655 | 307.311 | 1.807 | 0.078 |
| -0.137 | 7.80E-10 | NA | NA | 5.17E-04 | NA | 1.26E-01 | 5 | -148.671 | 307.342 | 1.838 | 0.077 |
| -0.022 | NA | NA | + | NA | NA | NA | 5 | -148.692 | 307.384 | 1.88 | 0.076 |
| -0.177 | NA | NA | NA | NA | -2.19E-02 | 1.52E-01 | 4 | -149.714 | 307.428 | 1.925 | 0.074 |
| -0.116 | NA | NA | NA | 5.25E-04 | 1.09E-03 | 1.22E-01 | 5 | -148.752 | 307.504 | 2 | 0.071 |
| -0.488 | NA | NA | + | NA | NA | 1.72E-01 | 6 | -154.195 | 320.389 | 0 | 0.412 |
| -0.57 | NA | NA | + | -3.67E-04 | NA | 1.80E-01 | 7 | -153.887 | 321.774 | 1.384 | 0.206 |
| -0.528 | 1.40E-09 | NA | + | NA | NA | 1.79E-01 | 7 | -153.908 | 321.817 | 1.427 | 0.202 |
| -0.4 | NA | NA | + | NA | -3.93E-02 | 1.72E-01 | 7 | -154.026 | 322.052 | 1.663 | 0.18 |
| -0.471 | NA | NA | + | NA | NA | 1.70E-01 | 6 | -143.54 | 299.08 | 0 | 0.39 |
| -0.571 | NA | NA | + | -4.52E-04 | NA | 1.78E-01 | 7 | -143.024 | 300.048 | 0.968 | 0.24 |
| -0.505 | 1.18E-09 | NA | + | NA | NA | 1.76E-01 | 7 | -143.266 | 300.532 | 1.453 | 0.188 |
| -0.373 | NA | NA | + | NA | -4.48E-02 | 1.72E-01 | 7 | -143.301 | 300.603 | 1.523 | 0.182 |
| -0.415 | NA | NA | + | NA | NA | 1.54E-01 | 6 | -145.05 | 302.099 | 0 | 0.455 |
| -0.438 | 8.65E-10 | NA | + | NA | NA | 1.58E-01 | 7 | -144.925 | 303.85 | 1.751 | 0.19 |
| -0.465 | NA | NA | + | -2.08E-04 | NA | 1.59E-01 | 7 | -144.944 | 303.888 | 1.789 | 0.186 |
| -0.39 | NA | NA | + | NA | -1.15E-02 | 1.55E-01 | 7 | -145.036 | 304.072 | 1.973 | 0.17 |
| -0.438 | NA | NA | + | NA | NA | 1.58E-01 | 6 | -159.929 | 331.858 | 0 | 0.454 |
| -0.472 | 1.16E-09 | NA | + | NA | NA | 1.64E-01 | 7 | -159.718 | 333.436 | 1.578 | 0.206 |
| -0.463 | NA | NA | + | -1.08E-04 | NA | 1.60E-01 | 7 | -159.906 | 333.811 | 1.953 | 0.171 |
| -0.417 | NA | NA | + | NA | -9.81E-03 | 1.58E-01 | 7 | -159.92 | 333.84 | 1.982 | 0.169 |
| -0.441 | NA | NA | + | NA | NA | 1.59E-01 | 6 | -148.186 | 308.373 | 0 | 0.39 |
| -0.565 | NA | NA | + | -5.41E-04 | NA | 1.68E-01 | 7 | -147.5 | 309 | 0.628 | 0.285 |
| -0.472 | 1.06E-09 | NA | + | NA | NA | 1.64E-01 | 7 | -147.982 | 309.964 | 1.591 | 0.176 |
| -0.399 | NA | NA | + | NA | -2.00E-02 | 1.60E-01 | 7 | -148.145 | 310.29 | 1.918 | 0.149 |
| -0.631 | NA | NA | + | NA | NA | 2.10E-01 | 6 | -164.484 | 340.968 | 0 | 0.252 |
| -0.757 | NA | NA | + | -6.10E-04 | NA | 2.22E-01 | 7 | -163.639 | 341.278 | 0.31 | 0.216 |
| -0.612 | NA | NA | + | -6.77E-04 | -7.14E-02 | 2.24E-01 | 8 | -163.101 | 342.202 | 1.233 | 0.136 |
| -0.676 | 1.61E-09 | NA | + | NA | NA | 2.18E-01 | 7 | -164.109 | 342.217 | 1.249 | 0.135 |
| -0.502 | NA | NA | + | NA | -5.79E-02 | 2.11E-01 | 7 | -164.128 | 342.255 | 1.287 | 0.132 |
| -0.816 | 1.81E-09 | NA | + | -6.47E-04 | NA | 2.32E-01 | 8 | -163.159 | 342.318 | 1.35 | 0.128 |
| -0.461 | NA | NA | + | NA | NA | 1.68E-01 | 6 | -151.327 | 314.653 | 0 | 0.419 |
| -0.553 | NA | NA | + | -3.83E-04 | NA | 1.76E-01 | 7 | -150.983 | 315.966 | 1.313 | 0.218 |
| -0.494 | 1.11E-09 | NA | + | NA | NA | 1.73E-01 | 7 | -151.113 | 316.225 | 1.572 | 0.191 |
| -0.389 | NA | NA | + | NA | -3.22E-02 | 1.68E-01 | 7 | -151.218 | 316.436 | 1.782 | 0.172 |
| -0.484 | NA | NA | + | NA | NA | 1.79E-01 | 6 | -159.471 | 330.942 | 0 | 0.44 |
| -0.52 | 1.20E-09 | NA | + | NA | NA | 1.86E-01 | 7 | -159.224 | 332.448 | 1.506 | 0.207 |
| -0.532 | NA | NA | + | -2.20E-04 | NA | 1.83E-01 | 7 | -159.366 | 332.733 | 1.79 | 0.18 |
| -0.429 | NA | NA | + | NA | -2.57E-02 | 1.80E-01 | 7 | -159.406 | 332.812 | 1.87 | 0.173 |
| -0.392 | NA | NA | + | NA | NA | 1.48E-01 | 6 | -136.276 | 284.552 | 0 | 0.411 |
| -0.484 | NA | NA | + | -4.02E-04 | NA | 1.56E-01 | 7 | -135.859 | 285.718 | 1.166 | 0.229 |
| -0.422 | 1.05E-09 | NA | + | NA | NA | 1.53E-01 | 7 | -136.056 | 286.113 | 1.561 | 0.188 |
| -0.319 | NA | NA | + | NA | -3.29E-02 | 1.49E-01 | 7 | -136.145 | 286.29 | 1.739 | 0.172 |
| -0.597 | NA | NA | + | NA | NA | 2.08E-01 | 6 | -160.811 | 333.623 | 0 | 0.271 |
| -0.715 | NA | NA | + | -5.79E-04 | NA | 2.18E-01 | 7 | -160.032 | 334.064 | 0.441 | 0.218 |
| -0.642 | 1.75E-09 | NA | + | NA | NA | 2.14E-01 | 7 | -160.464 | 334.929 | 1.306 | 0.141 |
| -0.489 | NA | NA | + | NA | -5.02E-02 | 2.09E-01 | 7 | -160.539 | 335.077 | 1.455 | 0.131 |
| -0.591 | NA | NA | + | -6.38E-04 | -6.31E-02 | 2.20E-01 | 8 | -159.604 | 335.207 | 1.584 | 0.123 |
| -0.763 | 1.81E-09 | NA | + | -5.87E-04 | NA | 2.24E-01 | 8 | -159.659 | 335.318 | 1.695 | 0.116 |
| -0.529 | NA | NA | + | NA | NA | 1.89E-01 | 6 | -150.905 | 313.81 | 0 | 0.262 |
| -0.652 | NA | NA | + | -5.62E-04 | NA | 2.01E-01 | 7 | -150.122 | 314.244 | 0.434 | 0.21 |
| -0.398 | NA | NA | + | NA | -5.88E-02 | 1.89E-01 | 7 | -150.51 | 315.02 | 1.21 | 0.143 |
| -0.508 | NA | NA | + | -6.26E-04 | -7.08E-02 | 2.02E-01 | 8 | -149.552 | 315.104 | 1.294 | 0.137 |
| -0.568 | 1.27E-09 | NA | + | NA | NA | 1.96E-01 | 7 | -150.596 | 315.192 | 1.382 | 0.131 |
| -0.706 | 1.47E-09 | NA | + | -6.00E-04 | NA | 2.10E-01 | 8 | -149.708 | 315.416 | 1.606 | 0.117 |
| -0.507 | NA | NA | + | NA | NA | 1.77E-01 | 6 | -150.799 | 313.598 | 0 | 0.288 |
| -0.618 | NA | NA | + | -5.48E-04 | NA | 1.86E-01 | 7 | -150.052 | 314.104 | 0.506 | 0.223 |
| -0.541 | 1.12E-09 | NA | + | NA | NA | 1.83E-01 | 7 | -150.559 | 315.117 | 1.519 | 0.135 |
| -0.413 | NA | NA | + | NA | -4.39E-02 | 1.79E-01 | 7 | -150.591 | 315.182 | 1.584 | 0.13 |
| -0.663 | 1.30E-09 | NA | + | -5.79E-04 | NA | 1.93E-01 | 8 | -149.726 | 315.452 | 1.854 | 0.114 |
| -0.513 | NA | NA | + | -5.80E-04 | -5.21E-02 | 1.88E-01 | 8 | -149.759 | 315.518 | 1.919 | 0.11 |
| -0.529 | NA | NA | + | NA | NA | 1.85E-01 | 6 | -155.861 | 323.721 | 0 | 0.403 |
| -0.61 | NA | NA | + | -3.89E-04 | NA | 1.92E-01 | 7 | -155.499 | 324.999 | 1.277 | 0.213 |
| -0.566 | 1.31E-09 | NA | + | NA | NA | 1.91E-01 | 7 | -155.589 | 325.177 | 1.456 | 0.195 |
| -0.421 | NA | NA | + | NA | -4.90E-02 | 1.86E-01 | 7 | -155.613 | 325.225 | 1.504 | 0.19 |
| -0.554 | NA | NA | + | NA | NA | 1.95E-01 | 6 | -149.686 | 311.371 | 0 | 0.264 |
| -0.661 | NA | NA | + | -5.15E-04 | NA | 2.04E-01 | 7 | -148.985 | 311.97 | 0.599 | 0.196 |
| -0.415 | NA | NA | + | NA | -6.30E-02 | 1.96E-01 | 7 | -149.199 | 312.397 | 1.026 | 0.158 |
| -0.511 | NA | NA | + | -5.88E-04 | -7.52E-02 | 2.06E-01 | 8 | -148.297 | 312.594 | 1.223 | 0.143 |
| -0.59 | 1.15E-09 | NA | + | NA | NA | 2.02E-01 | 7 | -149.39 | 312.78 | 1.409 | 0.131 |
| -0.712 | 1.34E-09 | NA | + | -5.54E-04 | NA | 2.12E-01 | 8 | -148.582 | 313.164 | 1.793 | 0.108 |
| -0.435 | NA | NA | + | NA | NA | 1.59E-01 | 6 | -151.828 | 315.655 | 0 | 0.429 |
| -0.511 | NA | NA | + | -3.54E-04 | NA | 1.66E-01 | 7 | -151.55 | 317.1 | 1.445 | 0.208 |
| -0.469 | 1.22E-09 | NA | + | NA | NA | 1.64E-01 | 7 | -151.613 | 317.226 | 1.571 | 0.196 |
| -0.386 | NA | NA | + | NA | -2.27E-02 | 1.61E-01 | 7 | -151.774 | 317.549 | 1.893 | 0.167 |
| -0.538 | NA | NA | + | NA | NA | 1.84E-01 | 6 | -158.843 | 329.687 | 0 | 0.332 |
| -0.651 | NA | NA | + | -5.10E-04 | NA | 1.94E-01 | 7 | -158.242 | 330.484 | 0.798 | 0.223 |
| -0.423 | NA | NA | + | NA | -5.18E-02 | 1.85E-01 | 7 | -158.554 | 331.108 | 1.422 | 0.163 |
| -0.578 | 1.54E-09 | NA | + | NA | NA | 1.90E-01 | 7 | -158.571 | 331.141 | 1.455 | 0.16 |
| -0.525 | NA | NA | + | -5.59E-04 | -6.15E-02 | 1.96E-01 | 8 | -157.836 | 331.673 | 1.986 | 0.123 |
| -0.729 | NA | NA | + | -7.96E-04 | NA | 2.11E-01 | 7 | -145.876 | 305.753 | 0 | 0.319 |
| -0.574 | NA | NA | + | -8.71E-04 | -7.72E-02 | 2.13E-01 | 8 | -145.176 | 306.351 | 0.598 | 0.237 |
| -0.773 | 1.48E-09 | NA | + | -8.11E-04 | NA | 2.17E-01 | 8 | -145.523 | 307.047 | 1.294 | 0.167 |
| -0.555 | NA | NA | + | NA | NA | 1.96E-01 | 6 | -147.576 | 307.152 | 1.399 | 0.159 |
| -0.621 | 1.37E-09 | NA | + | -8.82E-04 | -7.46E-02 | 2.19E-01 | 9 | -144.868 | 307.736 | 1.983 | 0.118 |
| -0.542 | NA | NA | + | NA | NA | 1.85E-01 | 6 | -167.858 | 347.716 | 0 | 0.418 |
| -0.64 | NA | NA | + | -4.51E-04 | NA | 1.93E-01 | 7 | -167.437 | 348.873 | 1.157 | 0.234 |
| -0.578 | 1.33E-09 | NA | + | NA | NA | 1.91E-01 | 7 | -167.63 | 349.26 | 1.544 | 0.193 |
| -0.528 | NA | NA | + | NA | -6.54E-03 | 1.85E-01 | 7 | -167.854 | 349.708 | 1.992 | 0.154 |
| -0.472 | NA | NA | + | NA | NA | 1.73E-01 | 6 | -145.338 | 302.676 | 0 | 0.403 |
| -0.568 | NA | NA | + | -4.07E-04 | NA | 1.82E-01 | 7 | -144.919 | 303.839 | 1.162 | 0.225 |
| -0.368 | NA | NA | + | NA | -4.58E-02 | 1.74E-01 | 7 | -145.098 | 304.196 | 1.519 | 0.188 |
| -0.503 | 9.38E-10 | NA | + | NA | NA | 1.80E-01 | 7 | -145.125 | 304.251 | 1.574 | 0.183 |
| -0.575 | NA | NA | + | NA | NA | 1.96E-01 | 6 | -153.564 | 319.127 | 0 | 0.21 |
| -0.699 | NA | NA | + | -5.97E-04 | NA | 2.06E-01 | 7 | -152.672 | 319.345 | 0.218 | 0.188 |
| -0.526 | NA | NA | + | -6.89E-04 | -8.59E-02 | 2.10E-01 | 8 | -151.838 | 319.677 | 0.549 | 0.16 |
| -0.419 | NA | NA | + | NA | -6.98E-02 | 1.99E-01 | 7 | -153.006 | 320.011 | 0.884 | 0.135 |
| -0.616 | 1.35E-09 | NA | + | NA | NA | 2.04E-01 | 7 | -153.204 | 320.407 | 1.28 | 0.111 |
| -0.75 | 1.50E-09 | NA | + | -6.25E-04 | NA | 2.15E-01 | 8 | -152.226 | 320.453 | 1.326 | 0.108 |
| -0.579 | 1.41E-09 | NA | + | -7.12E-04 | -8.32E-02 | 2.18E-01 | 9 | -151.44 | 320.881 | 1.753 | 0.087 |
| -0.408 | NA | NA | + | NA | NA | 1.55E-01 | 6 | -142.335 | 296.669 | 0 | 0.438 |
| -0.479 | NA | NA | + | -3.25E-04 | NA | 1.61E-01 | 7 | -142.072 | 298.144 | 1.475 | 0.21 |
| -0.433 | 8.82E-10 | NA | + | NA | NA | 1.59E-01 | 7 | -142.185 | 298.371 | 1.702 | 0.187 |
| -0.379 | NA | NA | + | NA | -1.39E-02 | 1.56E-01 | 7 | -142.314 | 298.627 | 1.958 | 0.165 |
| -0.494 | NA | NA | + | NA | NA | 1.76E-01 | 6 | -165.395 | 342.791 | 0 | 0.456 |
| -0.527 | 1.26E-09 | NA | + | NA | NA | 1.81E-01 | 7 | -165.197 | 344.394 | 1.603 | 0.205 |
| -0.511 | NA | NA | + | -7.77E-05 | NA | 1.77E-01 | 7 | -165.383 | 344.766 | 1.975 | 0.17 |
| -0.47 | NA | NA | + | NA | -1.09E-02 | 1.76E-01 | 7 | -165.384 | 344.768 | 1.977 | 0.17 |
| -0.591 | NA | NA | + | -9.45E-04 | -1.25E-01 | 2.45E-01 | 8 | -156.412 | 328.825 | 0 | 0.597 |
| -0.663 | 1.80E-09 | NA | + | -9.76E-04 | -1.19E-01 | 2.56E-01 | 9 | -155.804 | 329.609 | 0.784 | 0.403 |
| -0.479 | NA | NA | + | NA | NA | 1.72E-01 | 6 | -156.944 | 325.887 | 0 | 0.438 |
| -0.514 | 1.33E-09 | NA | + | NA | NA | 1.78E-01 | 7 | -156.704 | 327.408 | 1.52 | 0.205 |
| -0.53 | NA | NA | + | -2.30E-04 | NA | 1.77E-01 | 7 | -156.826 | 327.652 | 1.764 | 0.181 |
| -0.416 | NA | NA | + | NA | -2.85E-02 | 1.73E-01 | 7 | -156.861 | 327.722 | 1.835 | 0.175 |
| -0.144 | NA | NA | NA | 5.83E-04 | NA | 1.37E-01 | 4 | -162.632 | 333.264 | 0 | 0.304 |
| -0.258 | NA | NA | NA | NA | NA | 1.68E-01 | 3 | -163.721 | 333.443 | 0.178 | 0.278 |
| -0.325 | NA | NA | + | NA | NA | 1.34E-01 | 6 | -161.179 | 334.358 | 1.094 | 0.176 |
| -0.202 | NA | NA | NA | 6.25E-04 | 3.38E-02 | 1.34E-01 | 5 | -162.534 | 335.067 | 1.803 | 0.124 |
| -0.161 | 6.07E-10 | NA | NA | 5.75E-04 | NA | 1.40E-01 | 5 | -162.581 | 335.162 | 1.898 | 0.118 |
| -0.498 | NA | NA | + | NA | NA | 1.74E-01 | 6 | -153.069 | 318.138 | 0 | 0.416 |
| -0.585 | NA | NA | + | -3.80E-04 | NA | 1.82E-01 | 7 | -152.737 | 319.473 | 1.335 | 0.214 |
| -0.531 | 1.06E-09 | NA | + | NA | NA | 1.81E-01 | 7 | -152.851 | 319.702 | 1.564 | 0.19 |
| -0.409 | NA | NA | + | NA | -3.90E-02 | 1.74E-01 | 7 | -152.909 | 319.817 | 1.679 | 0.18 |
| -0.5 | NA | NA | + | NA | NA | 1.80E-01 | 6 | -146.957 | 305.914 | 0 | 0.382 |
| -0.614 | NA | NA | + | -5.19E-04 | NA | 1.89E-01 | 7 | -146.284 | 306.567 | 0.653 | 0.276 |
| -0.536 | 1.26E-09 | NA | + | NA | NA | 1.86E-01 | 7 | -146.701 | 307.402 | 1.489 | 0.182 |
| -0.426 | NA | NA | + | NA | -3.45E-02 | 1.81E-01 | 7 | -146.828 | 307.655 | 1.742 | 0.16 |
| -0.492 | NA | NA | + | NA | NA | 1.81E-01 | 6 | -158.341 | 328.683 | 0 | 0.44 |
| -0.528 | 1.08E-09 | NA | + | NA | NA | 1.88E-01 | 7 | -158.075 | 330.15 | 1.467 | 0.211 |
| -0.539 | NA | NA | + | -2.09E-04 | NA | 1.85E-01 | 7 | -158.245 | 330.49 | 1.807 | 0.178 |
| -0.441 | NA | NA | + | NA | -2.27E-02 | 1.81E-01 | 7 | -158.29 | 330.579 | 1.896 | 0.17 |
| -0.542 | NA | NA | + | NA | NA | 1.97E-01 | 6 | -158.548 | 329.095 | 0 | 0.395 |
| -0.622 | NA | NA | + | -3.79E-04 | NA | 2.05E-01 | 7 | -158.204 | 330.408 | 1.312 | 0.205 |
| -0.42 | NA | NA | + | NA | -5.46E-02 | 1.96E-01 | 7 | -158.221 | 330.443 | 1.348 | 0.202 |
| -0.582 | 1.16E-09 | NA | + | NA | NA | 2.06E-01 | 7 | -158.239 | 330.478 | 1.383 | 0.198 |
| -0.587 | NA | NA | + | -9.55E-04 | -9.92E-02 | 2.27E-01 | 8 | -145.139 | 306.278 | 0 | 0.34 |
| -0.785 | NA | NA | + | -8.53E-04 | NA | 2.25E-01 | 7 | -146.382 | 306.764 | 0.486 | 0.266 |
| -0.651 | 1.59E-09 | NA | + | -9.96E-04 | -9.53E-02 | 2.37E-01 | 9 | -144.611 | 307.222 | 0.945 | 0.212 |
| -0.847 | 1.74E-09 | NA | + | -9.03E-04 | NA | 2.35E-01 | 8 | -145.761 | 307.522 | 1.244 | 0.182 |
| -0.45 | NA | NA | + | NA | NA | 1.64E-01 | 6 | -141.697 | 295.395 | 0 | 0.279 |
| -0.574 | NA | NA | + | -5.56E-04 | NA | 1.74E-01 | 7 | -140.877 | 295.754 | 0.359 | 0.233 |
| -0.482 | 8.91E-10 | NA | + | NA | NA | 1.71E-01 | 7 | -141.439 | 296.878 | 1.483 | 0.133 |
| -0.628 | 1.14E-09 | NA | + | -6.15E-04 | NA | 1.84E-01 | 8 | -140.454 | 296.909 | 1.514 | 0.131 |
| -0.38 | NA | NA | + | NA | -3.23E-02 | 1.66E-01 | 7 | -141.574 | 297.148 | 1.753 | 0.116 |
| -0.489 | NA | NA | + | -5.93E-04 | -4.35E-02 | 1.77E-01 | 8 | -140.655 | 297.31 | 1.915 | 0.107 |
| -0.584 | NA | NA | + | -6.16E-04 | NA | 1.74E-01 | 7 | -137.447 | 288.895 | 0 | 0.247 |
| -0.446 | NA | NA | + | NA | NA | 1.62E-01 | 6 | -138.516 | 289.032 | 0.137 | 0.23 |
| -0.452 | NA | NA | + | -6.68E-04 | -6.72E-02 | 1.77E-01 | 8 | -136.898 | 289.797 | 0.902 | 0.157 |
| -0.632 | 1.20E-09 | NA | + | -6.56E-04 | NA | 1.82E-01 | 8 | -137.056 | 290.112 | 1.218 | 0.134 |
| -0.328 | NA | NA | + | NA | -5.52E-02 | 1.64E-01 | 7 | -138.146 | 290.293 | 1.398 | 0.123 |
| -0.477 | 9.68E-10 | NA | + | NA | NA | 1.68E-01 | 7 | -138.261 | 290.522 | 1.627 | 0.109 |
| -0.445 | NA | NA | + | NA | NA | 1.66E-01 | 6 | -141.615 | 295.229 | 0 | 0.399 |
| -0.543 | NA | NA | + | -4.21E-04 | NA | 1.75E-01 | 7 | -141.145 | 296.289 | 1.06 | 0.235 |
| -0.477 | 9.60E-10 | NA | + | NA | NA | 1.73E-01 | 7 | -141.387 | 296.774 | 1.545 | 0.184 |
| -0.351 | NA | NA | + | NA | -4.32E-02 | 1.68E-01 | 7 | -141.402 | 296.803 | 1.574 | 0.182 |
| -0.439 | NA | NA | + | NA | NA | 1.61E-01 | 6 | -142.785 | 297.57 | 0 | 0.419 |
| -0.526 | NA | NA | + | -3.75E-04 | NA | 1.68E-01 | 7 | -142.432 | 298.864 | 1.294 | 0.219 |
| -0.469 | 8.96E-10 | NA | + | NA | NA | 1.66E-01 | 7 | -142.574 | 299.149 | 1.579 | 0.19 |
| -0.371 | NA | NA | + | NA | -3.20E-02 | 1.63E-01 | 7 | -142.673 | 299.345 | 1.776 | 0.172 |
| -0.399 | NA | NA | + | NA | NA | 1.52E-01 | 6 | -139.005 | 290.011 | 0 | 0.395 |
| -0.277 | NA | NA | + | NA | -5.70E-02 | 1.55E-01 | 7 | -138.597 | 291.193 | 1.183 | 0.219 |
| -0.49 | NA | NA | + | -3.77E-04 | NA | 1.61E-01 | 7 | -138.624 | 291.249 | 1.238 | 0.213 |
| -0.426 | 9.83E-10 | NA | + | NA | NA | 1.56E-01 | 7 | -138.832 | 291.663 | 1.653 | 0.173 |
| -0.614 | NA | NA | + | NA | NA | 2.08E-01 | 6 | -158.205 | 328.409 | 0 | 0.211 |
| -0.728 | NA | NA | + | -5.59E-04 | NA | 2.17E-01 | 7 | -157.433 | 328.865 | 0.456 | 0.168 |
| -0.532 | NA | NA | + | -6.64E-04 | -9.21E-02 | 2.15E-01 | 8 | -156.456 | 328.913 | 0.504 | 0.164 |
| -0.434 | NA | NA | + | NA | -7.60E-02 | 2.04E-01 | 7 | -157.528 | 329.057 | 0.648 | 0.153 |
| -0.658 | 1.58E-09 | NA | + | NA | NA | 2.15E-01 | 7 | -157.818 | 329.636 | 1.227 | 0.115 |
| -0.783 | 1.74E-09 | NA | + | -5.90E-04 | NA | 2.25E-01 | 8 | -156.957 | 329.915 | 1.506 | 0.1 |
| -0.591 | 1.56E-09 | NA | + | -6.87E-04 | -8.77E-02 | 2.22E-01 | 9 | -156.073 | 330.146 | 1.737 | 0.089 |
| -0.508 | NA | NA | + | -8.31E-04 | -9.94E-02 | 2.05E-01 | 8 | -143.69 | 303.38 | 0 | 0.271 |
| -0.707 | NA | NA | + | -7.18E-04 | NA | 2.01E-01 | 7 | -144.997 | 303.994 | 0.614 | 0.199 |
| -0.563 | 1.33E-09 | NA | + | -8.63E-04 | -9.70E-02 | 2.13E-01 | 9 | -143.239 | 304.478 | 1.099 | 0.156 |
| -0.557 | NA | NA | + | NA | NA | 1.89E-01 | 6 | -146.385 | 304.769 | 1.389 | 0.135 |
| -0.761 | 1.42E-09 | NA | + | -7.56E-04 | NA | 2.10E-01 | 8 | -144.489 | 304.977 | 1.598 | 0.122 |
| -0.377 | NA | NA | + | NA | -8.03E-02 | 1.91E-01 | 7 | -145.526 | 305.053 | 1.673 | 0.117 |
| -0.408 | NA | NA | + | NA | NA | 1.55E-01 | 6 | -152.7 | 317.399 | 0 | 0.377 |
| -0.332 | NA | NA | NA | NA | NA | 1.94E-01 | 3 | -156.407 | 318.815 | 1.415 | 0.186 |
| -0.434 | 9.12E-10 | NA | + | NA | NA | 1.59E-01 | 7 | -152.563 | 319.127 | 1.727 | 0.159 |
| -0.421 | NA | NA | + | -5.61E-05 | NA | 1.56E-01 | 7 | -152.693 | 319.386 | 1.987 | 0.14 |
| -0.406 | NA | NA | + | NA | -8.19E-04 | 1.55E-01 | 7 | -152.7 | 319.399 | 2 | 0.139 |
| -0.821 | NA | NA | + | -7.47E-04 | NA | 2.44E-01 | 7 | -163.853 | 341.706 | 0 | 0.23 |
| -0.65 | NA | NA | + | -8.37E-04 | -8.29E-02 | 2.44E-01 | 8 | -163.079 | 342.158 | 0.453 | 0.183 |
| -0.67 | NA | NA | + | NA | NA | 2.31E-01 | 6 | -165.181 | 342.362 | 0.656 | 0.166 |
| -0.88 | 2.07E-09 | NA | + | -7.69E-04 | NA | 2.53E-01 | 8 | -163.374 | 342.747 | 1.042 | 0.137 |
| -0.714 | 1.82E-09 | NA | + | -8.51E-04 | -7.70E-02 | 2.52E-01 | 9 | -162.709 | 343.419 | 1.713 | 0.098 |
| -0.524 | NA | NA | + | NA | -6.37E-02 | 2.29E-01 | 7 | -164.722 | 343.443 | 1.737 | 0.096 |
| -0.719 | 1.89E-09 | NA | + | NA | NA | 2.39E-01 | 7 | -164.788 | 343.576 | 1.87 | 0.09 |
| -0.49 | NA | NA | + | NA | NA | 1.78E-01 | 6 | -148.783 | 309.566 | 0 | 0.386 |
| -0.591 | NA | NA | + | -4.69E-04 | NA | 1.86E-01 | 7 | -148.241 | 310.482 | 0.916 | 0.244 |
| -0.528 | 1.37E-09 | NA | + | NA | NA | 1.83E-01 | 7 | -148.477 | 310.954 | 1.388 | 0.193 |
| -0.399 | NA | NA | + | NA | -4.38E-02 | 1.80E-01 | 7 | -148.567 | 311.134 | 1.568 | 0.176 |
| -0.52 | NA | NA | + | NA | NA | 1.78E-01 | 6 | -166.909 | 345.817 | 0 | 0.453 |
| -0.554 | 1.21E-09 | NA | + | NA | NA | 1.84E-01 | 7 | -166.724 | 347.448 | 1.631 | 0.2 |
| -0.553 | NA | NA | + | -1.58E-04 | NA | 1.80E-01 | 7 | -166.86 | 347.721 | 1.903 | 0.175 |
| -0.563 | NA | NA | + | NA | 1.91E-02 | 1.77E-01 | 7 | -166.877 | 347.755 | 1.937 | 0.172 |
| -0.456 | NA | NA | + | NA | NA | 1.70E-01 | 6 | -134.165 | 280.331 | 0 | 0.323 |
| -0.562 | NA | NA | + | -4.69E-04 | NA | 1.80E-01 | 7 | -133.561 | 281.122 | 0.791 | 0.217 |
| -0.345 | NA | NA | + | NA | -5.17E-02 | 1.72E-01 | 7 | -133.823 | 281.645 | 1.314 | 0.167 |
| -0.493 | 1.32E-09 | NA | + | NA | NA | 1.76E-01 | 7 | -133.858 | 281.716 | 1.385 | 0.161 |
| -0.441 | NA | NA | + | -5.28E-04 | -6.24E-02 | 1.83E-01 | 8 | -133.067 | 282.134 | 1.803 | 0.131 |
| -0.54 | NA | NA | + | NA | NA | 1.92E-01 | 6 | -147.819 | 307.638 | 0 | 0.275 |
| -0.656 | NA | NA | + | -5.47E-04 | NA | 2.00E-01 | 7 | -147.052 | 308.104 | 0.466 | 0.218 |
| -0.427 | NA | NA | + | NA | -5.11E-02 | 1.93E-01 | 7 | -147.519 | 309.039 | 1.401 | 0.136 |
| -0.574 | 1.21E-09 | NA | + | NA | NA | 1.97E-01 | 7 | -147.556 | 309.112 | 1.475 | 0.132 |
| -0.529 | NA | NA | + | -6.02E-04 | -6.29E-02 | 2.03E-01 | 8 | -146.602 | 309.204 | 1.566 | 0.126 |
| -0.702 | 1.39E-09 | NA | + | -5.78E-04 | NA | 2.07E-01 | 8 | -146.703 | 309.406 | 1.768 | 0.114 |
| -0.596 | NA | NA | + | NA | NA | 1.98E-01 | 6 | -163.059 | 338.119 | 0 | 0.253 |
| -0.729 | NA | NA | + | -6.55E-04 | NA | 2.08E-01 | 7 | -162.076 | 338.152 | 0.033 | 0.249 |
| -0.779 | 1.56E-09 | NA | + | -6.81E-04 | NA | 2.16E-01 | 8 | -161.707 | 339.413 | 1.295 | 0.132 |
| -0.61 | NA | NA | + | -7.09E-04 | -5.82E-02 | 2.09E-01 | 8 | -161.736 | 339.471 | 1.352 | 0.129 |
| -0.636 | 1.39E-09 | NA | + | NA | NA | 2.05E-01 | 7 | -162.77 | 339.54 | 1.422 | 0.124 |
| -0.499 | NA | NA | + | NA | -4.32E-02 | 1.98E-01 | 7 | -162.87 | 339.741 | 1.622 | 0.112 |
| -0.355 | NA | NA | + | NA | NA | 1.41E-01 | 6 | -145.726 | 303.453 | 0 | 0.37 |
| -0.427 | NA | NA | + | -3.00E-04 | NA | 1.47E-01 | 7 | -145.513 | 305.026 | 1.573 | 0.168 |
| -0.297 | NA | NA | NA | NA | NA | 1.76E-01 | 3 | -149.563 | 305.126 | 1.673 | 0.16 |
| -0.378 | 8.55E-10 | NA | + | NA | NA | 1.45E-01 | 7 | -145.593 | 305.187 | 1.734 | 0.155 |
| -0.302 | NA | NA | + | NA | -2.56E-02 | 1.43E-01 | 7 | -145.658 | 305.316 | 1.864 | 0.146 |
| -0.476 | NA | NA | + | NA | NA | 1.72E-01 | 6 | -153.563 | 319.125 | 0 | 0.418 |
| -0.513 | 1.20E-09 | NA | + | NA | NA | 1.79E-01 | 7 | -153.298 | 320.595 | 1.47 | 0.2 |
| -0.37 | NA | NA | + | NA | -4.75E-02 | 1.73E-01 | 7 | -153.307 | 320.614 | 1.489 | 0.199 |
| -0.537 | NA | NA | + | -2.74E-04 | NA | 1.78E-01 | 7 | -153.389 | 320.778 | 1.653 | 0.183 |
| -0.486 | NA | NA | + | NA | NA | 1.82E-01 | 6 | -157.823 | 327.646 | 0 | 0.445 |
| -0.522 | 1.34E-09 | NA | + | NA | NA | 1.88E-01 | 7 | -157.56 | 329.119 | 1.474 | 0.213 |
| -0.438 | NA | NA | + | NA | -2.15E-02 | 1.83E-01 | 7 | -157.776 | 329.553 | 1.907 | 0.172 |
| -0.515 | NA | NA | + | -1.36E-04 | NA | 1.85E-01 | 7 | -157.783 | 329.565 | 1.919 | 0.17 |
| -0.471 | NA | NA | + | NA | NA | 1.67E-01 | 6 | -148.354 | 308.708 | 0 | 0.41 |
| -0.566 | NA | NA | + | -4.18E-04 | NA | 1.75E-01 | 7 | -147.93 | 309.86 | 1.152 | 0.23 |
| -0.5 | 1.05E-09 | NA | + | NA | NA | 1.72E-01 | 7 | -148.157 | 310.315 | 1.607 | 0.184 |
| -0.391 | NA | NA | + | NA | -3.74E-02 | 1.69E-01 | 7 | -148.2 | 310.4 | 1.692 | 0.176 |
| -0.512 | NA | NA | + | NA | NA | 1.84E-01 | 6 | -147.626 | 307.253 | 0 | 0.265 |
| -0.633 | NA | NA | + | -5.36E-04 | NA | 1.95E-01 | 7 | -146.896 | 307.792 | 0.539 | 0.202 |
| -0.377 | NA | NA | + | NA | -6.07E-02 | 1.85E-01 | 7 | -147.166 | 308.333 | 1.08 | 0.154 |
| -0.489 | NA | NA | + | -6.00E-04 | -7.16E-02 | 1.97E-01 | 8 | -146.259 | 308.519 | 1.266 | 0.141 |
| -0.549 | 1.33E-09 | NA | + | NA | NA | 1.90E-01 | 7 | -147.327 | 308.655 | 1.402 | 0.131 |
| -0.68 | 1.46E-09 | NA | + | -5.59E-04 | NA | 2.02E-01 | 8 | -146.532 | 309.065 | 1.812 | 0.107 |
| -0.521 | NA | NA | + | NA | NA | 1.85E-01 | 6 | -157.767 | 327.534 | 0 | 0.415 |
| -0.609 | NA | NA | + | -3.84E-04 | NA | 1.93E-01 | 7 | -157.43 | 328.86 | 1.326 | 0.214 |
| -0.557 | 1.41E-09 | NA | + | NA | NA | 1.91E-01 | 7 | -157.511 | 329.021 | 1.487 | 0.197 |
| -0.44 | NA | NA | + | NA | -3.67E-02 | 1.86E-01 | 7 | -157.634 | 329.268 | 1.734 | 0.174 |
| -0.366 | NA | NA | + | NA | NA | 1.38E-01 | 6 | -139.33 | 290.661 | 0 | 0.375 |
| -0.297 | NA | NA | NA | NA | NA | 1.78E-01 | 3 | -143.157 | 292.314 | 1.653 | 0.164 |
| -0.392 | 7.14E-10 | NA | + | NA | NA | 1.44E-01 | 7 | -139.174 | 292.348 | 1.688 | 0.161 |
| -0.417 | NA | NA | + | -2.09E-04 | NA | 1.42E-01 | 7 | -139.222 | 292.443 | 1.783 | 0.154 |
| -0.315 | NA | NA | + | NA | -2.37E-02 | 1.39E-01 | 7 | -139.267 | 292.533 | 1.873 | 0.147 |
| -0.587 | NA | NA | + | NA | NA | 1.99E-01 | 6 | -154.388 | 320.775 | 0 | 0.216 |
| -0.707 | NA | NA | + | -5.77E-04 | NA | 2.07E-01 | 7 | -153.55 | 321.099 | 0.324 | 0.183 |
| -0.539 | NA | NA | + | -6.65E-04 | -8.22E-02 | 2.09E-01 | 8 | -152.764 | 321.528 | 0.753 | 0.148 |
| -0.436 | NA | NA | + | NA | -6.71E-02 | 2.00E-01 | 7 | -153.859 | 321.717 | 0.942 | 0.135 |
| -0.629 | 1.24E-09 | NA | + | NA | NA | 2.08E-01 | 7 | -154.011 | 322.023 | 1.247 | 0.116 |
| -0.768 | 1.47E-09 | NA | + | -6.30E-04 | NA | 2.19E-01 | 8 | -153.019 | 322.038 | 1.263 | 0.115 |
| -0.603 | 1.39E-09 | NA | + | -7.12E-04 | -7.92E-02 | 2.20E-01 | 9 | -152.287 | 322.574 | 1.799 | 0.088 |
| -0.605 | NA | NA | + | -8.99E-04 | -1.12E-01 | 2.41E-01 | 8 | -159.773 | 335.545 | 0 | 0.336 |
| -0.834 | NA | NA | + | -7.64E-04 | NA | 2.40E-01 | 7 | -161.271 | 336.541 | 0.996 | 0.204 |
| -0.67 | 1.95E-09 | NA | + | -9.13E-04 | -1.05E-01 | 2.47E-01 | 9 | -159.323 | 336.647 | 1.102 | 0.194 |
| -0.895 | 2.30E-09 | NA | + | -7.89E-04 | NA | 2.48E-01 | 8 | -160.653 | 337.306 | 1.761 | 0.139 |
| -0.676 | NA | NA | + | NA | NA | 2.27E-01 | 6 | -162.757 | 337.514 | 1.969 | 0.126 |
| -0.666 | NA | NA | + | NA | NA | 2.19E-01 | 6 | -170.231 | 352.461 | 0 | 0.274 |
| -0.781 | NA | NA | + | -5.59E-04 | NA | 2.28E-01 | 7 | -169.551 | 353.103 | 0.642 | 0.199 |
| -0.715 | 1.66E-09 | NA | + | NA | NA | 2.28E-01 | 7 | -169.836 | 353.673 | 1.212 | 0.15 |
| -0.535 | NA | NA | + | NA | -5.56E-02 | 2.17E-01 | 7 | -169.914 | 353.829 | 1.367 | 0.138 |
| -0.844 | 1.86E-09 | NA | + | -5.99E-04 | NA | 2.39E-01 | 8 | -169.058 | 354.116 | 1.655 | 0.12 |
| -0.633 | NA | NA | + | -6.33E-04 | -6.98E-02 | 2.28E-01 | 8 | -169.059 | 354.119 | 1.658 | 0.12 |
| -0.524 | NA | NA | + | NA | NA | 1.79E-01 | 6 | -161.039 | 334.078 | 0 | 0.426 |
| -0.565 | 1.44E-09 | NA | + | NA | NA | 1.86E-01 | 7 | -160.763 | 335.526 | 1.448 | 0.207 |
| -0.588 | NA | NA | + | -2.99E-04 | NA | 1.85E-01 | 7 | -160.845 | 335.689 | 1.611 | 0.19 |
| -0.446 | NA | NA | + | NA | -3.46E-02 | 1.80E-01 | 7 | -160.917 | 335.835 | 1.757 | 0.177 |
| -0.387 | NA | NA | + | NA | NA | 1.49E-01 | 6 | -152.323 | 316.647 | 0 | 0.318 |
| -0.324 | NA | NA | NA | NA | NA | 1.87E-01 | 3 | -155.894 | 317.789 | 1.142 | 0.18 |
| -0.236 | NA | NA | NA | 4.53E-04 | NA | 1.64E-01 | 4 | -155.188 | 318.375 | 1.729 | 0.134 |
| -0.413 | 8.24E-10 | NA | + | NA | NA | 1.54E-01 | 7 | -152.192 | 318.383 | 1.737 | 0.134 |
| -0.396 | NA | NA | + | -3.86E-05 | NA | 1.50E-01 | 7 | -152.32 | 318.64 | 1.993 | 0.117 |
| -0.383 | NA | NA | + | NA | -1.65E-03 | 1.49E-01 | 7 | -152.323 | 318.646 | 1.999 | 0.117 |
| -0.517 | NA | NA | + | NA | NA | 1.79E-01 | 6 | -153.761 | 319.523 | 0 | 0.417 |
| -0.608 | NA | NA | + | -4.10E-04 | NA | 1.86E-01 | 7 | -153.374 | 320.748 | 1.225 | 0.226 |
| -0.551 | 1.16E-09 | NA | + | NA | NA | 1.85E-01 | 7 | -153.512 | 321.023 | 1.5 | 0.197 |
| -0.476 | NA | NA | + | NA | -1.88E-02 | 1.80E-01 | 7 | -153.726 | 321.451 | 1.928 | 0.159 |
| -0.504 | NA | NA | + | NA | NA | 1.79E-01 | 6 | -148.433 | 308.866 | 0 | 0.395 |
| -0.613 | NA | NA | + | -4.85E-04 | NA | 1.88E-01 | 7 | -147.855 | 309.71 | 0.843 | 0.259 |
| -0.536 | 1.19E-09 | NA | + | NA | NA | 1.84E-01 | 7 | -148.213 | 310.425 | 1.559 | 0.181 |
| -0.429 | NA | NA | + | NA | -3.45E-02 | 1.80E-01 | 7 | -148.304 | 310.608 | 1.742 | 0.165 |
| -0.576 | NA | NA | + | NA | NA | 1.95E-01 | 6 | -151.908 | 315.816 | 0 | 0.238 |
| -0.699 | NA | NA | + | -6.01E-04 | NA | 2.07E-01 | 7 | -151.01 | 316.02 | 0.204 | 0.215 |
| -0.542 | NA | NA | + | -6.75E-04 | -7.77E-02 | 2.09E-01 | 8 | -150.322 | 316.644 | 0.828 | 0.157 |
| -0.435 | NA | NA | + | NA | -6.38E-02 | 1.96E-01 | 7 | -151.44 | 316.881 | 1.065 | 0.14 |
| -0.619 | 1.51E-09 | NA | + | NA | NA | 2.02E-01 | 7 | -151.55 | 317.099 | 1.284 | 0.125 |
| -0.753 | 1.69E-09 | NA | + | -6.31E-04 | NA | 2.15E-01 | 8 | -150.562 | 317.124 | 1.308 | 0.124 |
| -0.754 | NA | NA | + | -7.41E-04 | NA | 2.22E-01 | 7 | -152.632 | 319.263 | 0 | 0.233 |
| -0.606 | NA | NA | + | -8.20E-04 | -7.62E-02 | 2.26E-01 | 8 | -151.937 | 319.873 | 0.61 | 0.172 |
| -0.606 | NA | NA | + | NA | NA | 2.09E-01 | 6 | -153.983 | 319.966 | 0.703 | 0.164 |
| -0.812 | 1.68E-09 | NA | + | -7.77E-04 | NA | 2.32E-01 | 8 | -152.088 | 320.175 | 0.912 | 0.148 |
| -0.668 | 1.57E-09 | NA | + | -8.50E-04 | -7.24E-02 | 2.35E-01 | 9 | -151.458 | 320.916 | 1.652 | 0.102 |
| -0.48 | NA | NA | + | NA | -5.88E-02 | 2.11E-01 | 7 | -153.568 | 321.136 | 1.872 | 0.091 |
| -0.65 | 1.47E-09 | NA | + | NA | NA | 2.17E-01 | 7 | -153.574 | 321.147 | 1.884 | 0.091 |
| -0.843 | NA | NA | + | -7.47E-04 | NA | 2.35E-01 | 7 | -160.212 | 334.424 | 0 | 0.224 |
| -0.671 | NA | NA | + | -8.43E-04 | -8.51E-02 | 2.38E-01 | 8 | -159.396 | 334.791 | 0.368 | 0.186 |
| -0.696 | NA | NA | + | NA | NA | 2.26E-01 | 6 | -161.571 | 335.141 | 0.717 | 0.156 |
| -0.902 | 1.95E-09 | NA | + | -7.76E-04 | NA | 2.44E-01 | 8 | -159.662 | 335.324 | 0.9 | 0.143 |
| -0.735 | 1.79E-09 | NA | + | -8.65E-04 | -8.04E-02 | 2.45E-01 | 9 | -158.932 | 335.865 | 1.441 | 0.109 |
| -0.55 | NA | NA | + | NA | -6.46E-02 | 2.26E-01 | 7 | -161.096 | 336.193 | 1.769 | 0.092 |
| -0.744 | 1.75E-09 | NA | + | NA | NA | 2.33E-01 | 7 | -161.133 | 336.266 | 1.843 | 0.089 |
| -0.418 | NA | NA | + | NA | NA | 1.54E-01 | 6 | -142.48 | 296.961 | 0 | 0.429 |
| -0.49 | NA | NA | + | -3.13E-04 | NA | 1.59E-01 | 7 | -142.217 | 298.434 | 1.473 | 0.206 |
| -0.447 | 7.65E-10 | NA | + | NA | NA | 1.61E-01 | 7 | -142.27 | 298.539 | 1.578 | 0.195 |
| -0.362 | NA | NA | + | NA | -2.57E-02 | 1.55E-01 | 7 | -142.408 | 298.816 | 1.855 | 0.17 |
| -0.519 | NA | NA | + | NA | NA | 1.79E-01 | 6 | -172.766 | 357.531 | 0 | 0.453 |
| -0.552 | 1.02E-09 | NA | + | NA | NA | 1.85E-01 | 7 | -172.577 | 359.154 | 1.623 | 0.201 |
| -0.56 | NA | NA | + | -1.91E-04 | NA | 1.82E-01 | 7 | -172.698 | 359.396 | 1.865 | 0.178 |
| -0.517 | NA | NA | + | NA | -7.92E-04 | 1.79E-01 | 7 | -172.766 | 359.531 | 2 | 0.167 |
| -0.429 | NA | NA | + | NA | NA | 1.61E-01 | 6 | -148.563 | 309.125 | 0 | 0.444 |
| -0.458 | 9.35E-10 | NA | + | NA | NA | 1.66E-01 | 7 | -148.385 | 310.77 | 1.645 | 0.195 |
| -0.488 | NA | NA | + | -2.66E-04 | NA | 1.66E-01 | 7 | -148.393 | 310.786 | 1.661 | 0.193 |
| -0.393 | NA | NA | + | NA | -1.66E-02 | 1.61E-01 | 7 | -148.533 | 311.067 | 1.942 | 0.168 |
| -0.614 | NA | NA | + | -9.20E-04 | -1.17E-01 | 2.50E-01 | 8 | -158.467 | 332.934 | 0 | 0.498 |
| -0.68 | 1.94E-09 | NA | + | -9.26E-04 | -1.10E-01 | 2.58E-01 | 9 | -158.01 | 334.02 | 1.087 | 0.289 |
| -0.851 | NA | NA | + | -7.48E-04 | NA | 2.51E-01 | 7 | -160.32 | 334.639 | 1.705 | 0.212 |
| -0.499 | NA | NA | + | NA | NA | 1.79E-01 | 6 | -171.418 | 354.835 | 0 | 0.452 |
| -0.537 | 1.33E-09 | NA | + | NA | NA | 1.86E-01 | 7 | -171.174 | 356.348 | 1.512 | 0.212 |
| -0.471 | NA | NA | + | NA | -1.24E-02 | 1.80E-01 | 7 | -171.403 | 356.807 | 1.971 | 0.169 |
| -0.495 | NA | NA | + | 1.54E-05 | NA | 1.79E-01 | 7 | -171.417 | 356.834 | 1.999 | 0.166 |
| -0.407 | NA | NA | + | NA | NA | 1.52E-01 | 6 | -144.6 | 301.2 | 0 | 0.44 |
| -0.471 | NA | NA | + | -2.68E-04 | NA | 1.58E-01 | 7 | -144.424 | 302.847 | 1.647 | 0.193 |
| -0.433 | 8.46E-10 | NA | + | NA | NA | 1.57E-01 | 7 | -144.449 | 302.899 | 1.699 | 0.188 |
| -0.339 | NA | NA | + | NA | -3.10E-02 | 1.54E-01 | 7 | -144.498 | 302.997 | 1.797 | 0.179 |
| -0.37 | NA | NA | + | NA | NA | 1.41E-01 | 6 | -154.722 | 321.445 | 0 | 0.28 |
| -0.288 | NA | NA | NA | NA | NA | 1.75E-01 | 3 | -157.96 | 321.92 | 0.476 | 0.221 |
| -0.195 | NA | NA | NA | 4.78E-04 | NA | 1.49E-01 | 4 | -157.173 | 322.346 | 0.901 | 0.178 |
| -0.394 | 7.83E-10 | NA | + | NA | NA | 1.45E-01 | 7 | -154.618 | 323.236 | 1.791 | 0.114 |
| -0.39 | NA | NA | + | NA | 9.16E-03 | 1.40E-01 | 7 | -154.715 | 323.429 | 1.985 | 0.104 |
| -0.362 | NA | NA | + | 3.21E-05 | NA | 1.40E-01 | 7 | -154.72 | 323.44 | 1.996 | 0.103 |
| -0.397 | NA | NA | + | NA | NA | 1.52E-01 | 6 | -142.212 | 296.425 | 0 | 0.431 |
| -0.473 | NA | NA | + | -3.39E-04 | NA | 1.58E-01 | 7 | -141.933 | 297.865 | 1.44 | 0.21 |
| -0.427 | 1.05E-09 | NA | + | NA | NA | 1.57E-01 | 7 | -141.997 | 297.993 | 1.568 | 0.197 |
| -0.368 | NA | NA | + | NA | -1.37E-02 | 1.53E-01 | 7 | -142.192 | 298.385 | 1.96 | 0.162 |
| -0.595 | NA | NA | + | NA | NA | 2.01E-01 | 6 | -159.58 | 331.159 | 0 | 0.266 |
| -0.708 | NA | NA | + | -5.22E-04 | NA | 2.12E-01 | 7 | -158.953 | 331.905 | 0.746 | 0.183 |
| -0.443 | NA | NA | + | NA | -6.86E-02 | 2.03E-01 | 7 | -159.055 | 332.11 | 0.951 | 0.166 |
| -0.638 | 1.65E-09 | NA | + | NA | NA | 2.08E-01 | 7 | -159.215 | 332.43 | 1.271 | 0.141 |
| -0.546 | NA | NA | + | -6.03E-04 | -8.11E-02 | 2.15E-01 | 8 | -158.228 | 332.456 | 1.296 | 0.139 |
| -0.762 | 1.80E-09 | NA | + | -5.50E-04 | NA | 2.20E-01 | 8 | -158.516 | 333.032 | 1.873 | 0.104 |
| -0.705 | NA | NA | + | -6.70E-04 | NA | 2.10E-01 | 7 | -148.113 | 310.227 | 0 | 0.248 |
| -0.561 | NA | NA | + | NA | NA | 1.96E-01 | 6 | -149.264 | 310.528 | 0.301 | 0.213 |
| -0.551 | NA | NA | + | -7.40E-04 | -7.47E-02 | 2.11E-01 | 8 | -147.455 | 310.91 | 0.683 | 0.176 |
| -0.754 | 1.64E-09 | NA | + | -6.96E-04 | NA | 2.18E-01 | 8 | -147.715 | 311.43 | 1.203 | 0.136 |
| -0.426 | NA | NA | + | NA | -5.98E-02 | 1.96E-01 | 7 | -148.842 | 311.683 | 1.456 | 0.12 |
| -0.6 | 1.45E-09 | NA | + | NA | NA | 2.02E-01 | 7 | -148.956 | 311.913 | 1.686 | 0.107 |
| -0.499 | NA | NA | + | NA | NA | 1.81E-01 | 6 | -146.391 | 304.782 | 0 | 0.236 |
| -0.63 | NA | NA | + | -5.88E-04 | NA | 1.94E-01 | 7 | -145.476 | 304.952 | 0.17 | 0.217 |
| -0.469 | NA | NA | + | -6.55E-04 | -8.05E-02 | 1.97E-01 | 8 | -144.698 | 305.395 | 0.613 | 0.174 |
| -0.348 | NA | NA | + | NA | -6.85E-02 | 1.83E-01 | 7 | -145.826 | 305.652 | 0.87 | 0.153 |
| -0.532 | 1.25E-09 | NA | + | NA | NA | 1.87E-01 | 7 | -146.142 | 306.284 | 1.501 | 0.111 |
| -0.672 | 1.39E-09 | NA | + | -6.06E-04 | NA | 2.00E-01 | 8 | -145.166 | 306.332 | 1.55 | 0.109 |
| -0.452 | NA | NA | + | NA | NA | 1.63E-01 | 6 | -148.5 | 309 | 0 | 0.418 |
| -0.492 | 1.38E-09 | NA | + | NA | NA | 1.69E-01 | 7 | -148.178 | 310.357 | 1.356 | 0.212 |
| -0.36 | NA | NA | + | NA | -4.22E-02 | 1.65E-01 | 7 | -148.296 | 310.592 | 1.591 | 0.188 |
| -0.512 | NA | NA | + | -2.70E-04 | NA | 1.67E-01 | 7 | -148.331 | 310.662 | 1.661 | 0.182 |
| -0.543 | NA | NA | + | NA | NA | 1.90E-01 | 6 | -161.266 | 334.531 | 0 | 0.423 |
| -0.618 | NA | NA | + | -3.53E-04 | NA | 1.95E-01 | 7 | -160.983 | 335.966 | 1.435 | 0.206 |
| -0.581 | 1.10E-09 | NA | + | NA | NA | 1.97E-01 | 7 | -160.984 | 335.968 | 1.436 | 0.206 |
| -0.493 | NA | NA | + | NA | -2.33E-02 | 1.91E-01 | 7 | -161.213 | 336.426 | 1.895 | 0.164 |
| -0.424 | NA | NA | + | NA | NA | 1.53E-01 | 6 | -153.063 | 318.126 | 0 | 0.437 |
| -0.457 | 1.06E-09 | NA | + | NA | NA | 1.59E-01 | 7 | -152.843 | 319.686 | 1.561 | 0.2 |
| -0.49 | NA | NA | + | -2.88E-04 | NA | 1.59E-01 | 7 | -152.879 | 319.758 | 1.632 | 0.193 |
| -0.372 | NA | NA | + | NA | -2.33E-02 | 1.54E-01 | 7 | -153.008 | 320.015 | 1.89 | 0.17 |
| -0.586 | NA | NA | + | -8.04E-04 | -9.71E-02 | 2.26E-01 | 8 | -158.648 | 333.296 | 0 | 0.201 |
| -0.779 | NA | NA | + | -6.79E-04 | NA | 2.23E-01 | 7 | -159.751 | 333.502 | 0.206 | 0.182 |
| -0.648 | NA | NA | + | NA | NA | 2.13E-01 | 6 | -160.884 | 333.768 | 0.472 | 0.159 |
| -0.649 | 1.75E-09 | NA | + | -8.22E-04 | -9.29E-02 | 2.35E-01 | 9 | -158.136 | 334.272 | 0.976 | 0.124 |
| -0.839 | 1.90E-09 | NA | + | -7.05E-04 | NA | 2.32E-01 | 8 | -159.15 | 334.299 | 1.003 | 0.122 |
| -0.478 | NA | NA | + | NA | -7.58E-02 | 2.14E-01 | 7 | -160.199 | 334.399 | 1.102 | 0.116 |
| -0.699 | 1.76E-09 | NA | + | NA | NA | 2.21E-01 | 7 | -160.376 | 334.752 | 1.456 | 0.097 |
| -0.613 | NA | NA | + | NA | NA | 2.15E-01 | 6 | -163.007 | 338.015 | 0 | 0.281 |
| -0.435 | NA | NA | + | NA | -7.70E-02 | 2.13E-01 | 7 | -162.299 | 338.597 | 0.582 | 0.21 |
| -0.668 | 1.93E-09 | NA | + | NA | NA | 2.24E-01 | 7 | -162.528 | 339.056 | 1.041 | 0.167 |
| -0.675 | NA | NA | + | -2.81E-04 | NA | 2.21E-01 | 7 | -162.832 | 339.663 | 1.648 | 0.123 |
| -0.497 | 1.69E-09 | NA | + | NA | -7.08E-02 | 2.21E-01 | 8 | -161.934 | 339.868 | 1.853 | 0.111 |
| -0.499 | NA | NA | + | -3.84E-04 | -8.57E-02 | 2.21E-01 | 8 | -161.975 | 339.951 | 1.936 | 0.107 |
| -0.359 | NA | NA | + | NA | NA | 1.40E-01 | 6 | -152.75 | 317.5 | 0 | 0.289 |
| -0.295 | NA | NA | NA | NA | NA | 1.74E-01 | 3 | -156.104 | 318.208 | 0.708 | 0.203 |
| -0.203 | NA | NA | NA | 4.82E-04 | NA | 1.51E-01 | 4 | -155.296 | 318.592 | 1.093 | 0.167 |
| -0.384 | 8.33E-10 | NA | + | NA | NA | 1.44E-01 | 7 | -152.628 | 319.257 | 1.757 | 0.12 |
| -0.42 | NA | NA | + | NA | 2.82E-02 | 1.39E-01 | 7 | -152.679 | 319.359 | 1.859 | 0.114 |
| -0.357 | NA | NA | + | 9.78E-06 | NA | 1.40E-01 | 7 | -152.75 | 319.499 | 2 | 0.106 |
| -0.563 | NA | NA | + | NA | NA | 1.94E-01 | 6 | -167.57 | 347.14 | 0 | 0.415 |
| -0.61 | 1.66E-09 | NA | + | NA | NA | 2.02E-01 | 7 | -167.22 | 348.439 | 1.299 | 0.217 |
| -0.641 | NA | NA | + | -3.84E-04 | NA | 1.99E-01 | 7 | -167.263 | 348.526 | 1.387 | 0.208 |
| -0.515 | NA | NA | + | NA | -2.23E-02 | 1.95E-01 | 7 | -167.521 | 349.043 | 1.903 | 0.16 |
| -0.592 | NA | NA | + | -6.13E-04 | NA | 1.76E-01 | 7 | -134.435 | 282.869 | 0 | 0.246 |
| -0.456 | NA | NA | + | NA | NA | 1.67E-01 | 6 | -135.512 | 283.024 | 0.155 | 0.227 |
| -0.457 | NA | NA | + | -6.68E-04 | -6.89E-02 | 1.80E-01 | 8 | -133.836 | 283.671 | 0.802 | 0.165 |
| -0.635 | 1.16E-09 | NA | + | -6.44E-04 | NA | 1.83E-01 | 8 | -134.08 | 284.159 | 1.29 | 0.129 |
| -0.335 | NA | NA | + | NA | -5.70E-02 | 1.70E-01 | 7 | -135.103 | 284.206 | 1.337 | 0.126 |
| -0.486 | 9.76E-10 | NA | + | NA | NA | 1.72E-01 | 7 | -135.262 | 284.525 | 1.656 | 0.107 |
| -0.459 | NA | NA | + | NA | NA | 1.64E-01 | 6 | -148.707 | 309.414 | 0 | 0.403 |
| -0.334 | NA | NA | + | NA | -5.46E-02 | 1.64E-01 | 7 | -148.378 | 310.756 | 1.341 | 0.206 |
| -0.497 | 1.00E-09 | NA | + | NA | NA | 1.73E-01 | 7 | -148.408 | 310.815 | 1.401 | 0.2 |
| -0.527 | NA | NA | + | -3.22E-04 | NA | 1.70E-01 | 7 | -148.459 | 310.917 | 1.503 | 0.19 |
| -0.719 | NA | NA | + | -6.56E-04 | NA | 2.12E-01 | 7 | -152.112 | 318.223 | 0 | 0.243 |
| -0.58 | NA | NA | + | NA | NA | 2.00E-01 | 6 | -153.175 | 318.351 | 0.128 | 0.228 |
| -0.571 | NA | NA | + | -7.31E-04 | -7.33E-02 | 2.13E-01 | 8 | -151.509 | 319.018 | 0.795 | 0.164 |
| -0.768 | 1.56E-09 | NA | + | -6.78E-04 | NA | 2.20E-01 | 8 | -151.737 | 319.475 | 1.252 | 0.13 |
| -0.453 | NA | NA | + | NA | -5.70E-02 | 2.00E-01 | 7 | -152.808 | 319.617 | 1.394 | 0.121 |
| -0.62 | 1.40E-09 | NA | + | NA | NA | 2.07E-01 | 7 | -152.875 | 319.751 | 1.527 | 0.113 |
| -0.461 | NA | NA | + | NA | NA | 1.66E-01 | 6 | -147.371 | 306.743 | 0 | 0.301 |
| -0.58 | NA | NA | + | -5.25E-04 | NA | 1.76E-01 | 7 | -146.664 | 307.328 | 0.586 | 0.225 |
| -0.321 | NA | NA | + | NA | -6.42E-02 | 1.68E-01 | 7 | -146.897 | 307.794 | 1.052 | 0.178 |
| -0.431 | NA | NA | + | -5.81E-04 | -7.41E-02 | 1.80E-01 | 8 | -146.035 | 308.071 | 1.328 | 0.155 |
| -0.494 | 1.40E-09 | NA | + | NA | NA | 1.70E-01 | 7 | -147.132 | 308.263 | 1.521 | 0.141 |
| -0.438 | NA | NA | + | NA | NA | 1.60E-01 | 6 | -147.168 | 306.335 | 0 | 0.412 |
| -0.536 | NA | NA | + | -4.34E-04 | NA | 1.69E-01 | 7 | -146.704 | 307.407 | 1.072 | 0.241 |
| -0.469 | 1.10E-09 | NA | + | NA | NA | 1.65E-01 | 7 | -146.962 | 307.924 | 1.589 | 0.186 |
| -0.385 | NA | NA | + | NA | -2.47E-02 | 1.61E-01 | 7 | -147.102 | 308.203 | 1.868 | 0.162 |
| -0.445 | NA | NA | + | NA | NA | 1.63E-01 | 6 | -145.126 | 302.252 | 0 | 0.415 |
| -0.534 | NA | NA | + | -3.82E-04 | NA | 1.71E-01 | 7 | -144.754 | 303.509 | 1.256 | 0.221 |
| -0.361 | NA | NA | + | NA | -3.90E-02 | 1.64E-01 | 7 | -144.949 | 303.899 | 1.646 | 0.182 |
| -0.475 | 9.79E-10 | NA | + | NA | NA | 1.68E-01 | 7 | -144.951 | 303.901 | 1.649 | 0.182 |
| -0.431 | NA | NA | + | NA | NA | 1.57E-01 | 6 | -155.459 | 322.918 | 0 | 0.448 |
| -0.465 | 1.14E-09 | NA | + | NA | NA | 1.61E-01 | 7 | -155.283 | 324.565 | 1.647 | 0.197 |
| -0.492 | NA | NA | + | -2.62E-04 | NA | 1.61E-01 | 7 | -155.313 | 324.626 | 1.709 | 0.191 |
| -0.435 | NA | NA | + | NA | 1.73E-03 | 1.57E-01 | 7 | -155.459 | 324.917 | 1.999 | 0.165 |
| -0.555 | NA | NA | + | NA | NA | 1.95E-01 | 6 | -152.709 | 317.418 | 0 | 0.176 |
| -0.492 | NA | NA | + | -7.12E-04 | -9.57E-02 | 2.08E-01 | 8 | -150.734 | 317.469 | 0.051 | 0.172 |
| -0.686 | NA | NA | + | -6.11E-04 | NA | 2.06E-01 | 7 | -151.781 | 317.562 | 0.144 | 0.164 |
| -0.376 | NA | NA | + | NA | -7.95E-02 | 1.95E-01 | 7 | -151.979 | 317.959 | 0.541 | 0.135 |
| -0.598 | 1.67E-09 | NA | + | NA | NA | 2.01E-01 | 7 | -152.31 | 318.62 | 1.202 | 0.097 |
| -0.737 | 1.79E-09 | NA | + | -6.31E-04 | NA | 2.13E-01 | 8 | -151.318 | 318.636 | 1.218 | 0.096 |
| -0.546 | 1.62E-09 | NA | + | -7.26E-04 | -9.18E-02 | 2.14E-01 | 9 | -150.355 | 318.71 | 1.292 | 0.092 |
| -0.424 | 1.51E-09 | NA | + | NA | -7.55E-02 | 2.01E-01 | 8 | -151.652 | 319.305 | 1.887 | 0.069 |
| -0.342 | NA | NA | + | NA | NA | 1.42E-01 | 6 | -133.609 | 279.217 | 0 | 0.315 |
| -0.283 | NA | NA | NA | NA | NA | 1.74E-01 | 3 | -137.134 | 280.268 | 1.05 | 0.186 |
| -0.365 | 7.88E-10 | NA | + | NA | NA | 1.46E-01 | 7 | -133.46 | 280.919 | 1.702 | 0.134 |
| -0.395 | NA | NA | + | -2.15E-04 | NA | 1.45E-01 | 7 | -133.49 | 280.98 | 1.762 | 0.13 |
| -0.369 | NA | NA | + | NA | 1.27E-02 | 1.41E-01 | 7 | -133.591 | 281.183 | 1.965 | 0.118 |
| -0.218 | NA | NA | NA | 3.39E-04 | NA | 1.58E-01 | 4 | -136.605 | 281.21 | 1.992 | 0.116 |
| -0.496 | NA | NA | + | NA | NA | 1.73E-01 | 6 | -148.813 | 309.626 | 0 | 0.39 |
| -0.608 | NA | NA | + | -5.08E-04 | NA | 1.83E-01 | 7 | -148.173 | 310.347 | 0.72 | 0.272 |
| -0.527 | 1.06E-09 | NA | + | NA | NA | 1.78E-01 | 7 | -148.613 | 311.226 | 1.6 | 0.175 |
| -0.423 | NA | NA | + | NA | -3.29E-02 | 1.74E-01 | 7 | -148.695 | 311.389 | 1.763 | 0.162 |
| -0.675 | NA | NA | + | -1.02E-03 | -1.16E-01 | 2.54E-01 | 8 | -160.275 | 336.55 | 0 | 0.366 |
| -0.752 | 1.99E-09 | NA | + | -1.05E-03 | -1.11E-01 | 2.64E-01 | 9 | -159.577 | 337.155 | 0.605 | 0.27 |
| -0.907 | NA | NA | + | -8.55E-04 | NA | 2.51E-01 | 7 | -161.89 | 337.78 | 1.23 | 0.198 |
| -0.979 | 2.18E-09 | NA | + | -8.92E-04 | NA | 2.62E-01 | 8 | -161.064 | 338.128 | 1.578 | 0.166 |
| -0.474 | NA | NA | + | NA | NA | 1.70E-01 | 6 | -158.061 | 328.122 | 0 | 0.443 |
| -0.514 | 9.31E-10 | NA | + | NA | NA | 1.79E-01 | 7 | -157.777 | 329.554 | 1.432 | 0.216 |
| -0.412 | NA | NA | + | NA | -2.82E-02 | 1.70E-01 | 7 | -157.981 | 329.961 | 1.84 | 0.176 |
| -0.49 | NA | NA | + | -7.14E-05 | NA | 1.71E-01 | 7 | -158.05 | 330.099 | 1.978 | 0.165 |
| -0.519 | NA | NA | + | NA | NA | 1.82E-01 | 6 | -155.141 | 322.283 | 0 | 0.401 |
| -0.607 | NA | NA | + | -3.96E-04 | NA | 1.89E-01 | 7 | -154.779 | 323.557 | 1.275 | 0.212 |
| -0.407 | NA | NA | + | NA | -5.00E-02 | 1.83E-01 | 7 | -154.87 | 323.739 | 1.457 | 0.194 |
| -0.556 | 1.29E-09 | NA | + | NA | NA | 1.88E-01 | 7 | -154.872 | 323.744 | 1.461 | 0.193 |
| -0.585 | NA | NA | + | NA | NA | 2.07E-01 | 6 | -155.447 | 322.893 | 0 | 0.276 |
| -0.696 | NA | NA | + | -5.41E-04 | NA | 2.17E-01 | 7 | -154.747 | 323.494 | 0.601 | 0.205 |
| -0.627 | 1.52E-09 | NA | + | NA | NA | 2.14E-01 | 7 | -155.108 | 324.216 | 1.323 | 0.143 |
| -0.471 | NA | NA | + | NA | -5.25E-02 | 2.08E-01 | 7 | -155.131 | 324.261 | 1.368 | 0.139 |
| -0.566 | NA | NA | + | -6.13E-04 | -6.62E-02 | 2.19E-01 | 8 | -154.251 | 324.502 | 1.609 | 0.124 |
| -0.747 | 1.66E-09 | NA | + | -5.68E-04 | NA | 2.25E-01 | 8 | -154.337 | 324.674 | 1.781 | 0.113 |
| -0.446 | NA | NA | + | NA | NA | 1.62E-01 | 6 | -146.668 | 305.337 | 0 | 0.411 |
| -0.538 | NA | NA | + | -4.13E-04 | NA | 1.70E-01 | 7 | -146.251 | 306.503 | 1.166 | 0.23 |
| -0.477 | 1.07E-09 | NA | + | NA | NA | 1.67E-01 | 7 | -146.443 | 306.887 | 1.55 | 0.19 |
| -0.375 | NA | NA | + | NA | -3.28E-02 | 1.63E-01 | 7 | -146.554 | 307.108 | 1.771 | 0.17 |
| -0.437 | NA | NA | + | NA | NA | 1.60E-01 | 6 | -143.471 | 298.942 | 0 | 0.386 |
| -0.541 | NA | NA | + | -4.75E-04 | NA | 1.69E-01 | 7 | -142.91 | 299.819 | 0.877 | 0.249 |
| -0.471 | 1.35E-09 | NA | + | NA | NA | 1.65E-01 | 7 | -143.211 | 300.423 | 1.48 | 0.184 |
| -0.338 | NA | NA | + | NA | -4.49E-02 | 1.61E-01 | 7 | -143.23 | 300.461 | 1.518 | 0.181 |
| -0.51 | NA | NA | + | NA | NA | 1.79E-01 | 6 | -160.54 | 333.08 | 0 | 0.436 |
| -0.541 | 1.10E-09 | NA | + | NA | NA | 1.85E-01 | 7 | -160.329 | 334.658 | 1.579 | 0.198 |
| -0.576 | NA | NA | + | -3.05E-04 | NA | 1.85E-01 | 7 | -160.339 | 334.677 | 1.597 | 0.196 |
| -0.459 | NA | NA | + | NA | -2.32E-02 | 1.80E-01 | 7 | -160.488 | 334.976 | 1.896 | 0.169 |
| -0.455 | NA | NA | + | NA | NA | 1.69E-01 | 6 | -150.618 | 313.237 | 0 | 0.43 |
| -0.53 | NA | NA | + | -3.23E-04 | NA | 1.75E-01 | 7 | -150.372 | 314.745 | 1.508 | 0.202 |
| -0.487 | 1.15E-09 | NA | + | NA | NA | 1.74E-01 | 7 | -150.397 | 314.794 | 1.557 | 0.198 |
| -0.4 | NA | NA | + | NA | -2.59E-02 | 1.70E-01 | 7 | -150.549 | 315.098 | 1.861 | 0.17 |
| -0.519 | NA | NA | + | NA | NA | 1.84E-01 | 6 | -150.763 | 313.526 | 0 | 0.404 |
| -0.593 | NA | NA | + | -3.56E-04 | NA | 1.90E-01 | 7 | -150.445 | 314.89 | 1.365 | 0.204 |
| -0.555 | 1.13E-09 | NA | + | NA | NA | 1.92E-01 | 7 | -150.466 | 314.932 | 1.406 | 0.2 |
| -0.411 | NA | NA | + | NA | -4.78E-02 | 1.84E-01 | 7 | -150.505 | 315.011 | 1.485 | 0.192 |
| -0.465 | NA | NA | + | NA | NA | 1.73E-01 | 6 | -152.19 | 316.379 | 0 | 0.413 |
| -0.563 | NA | NA | + | -4.26E-04 | NA | 1.81E-01 | 7 | -151.768 | 317.535 | 1.156 | 0.232 |
| -0.494 | 1.15E-09 | NA | + | NA | NA | 1.77E-01 | 7 | -151.993 | 317.987 | 1.608 | 0.185 |
| -0.397 | NA | NA | + | NA | -3.16E-02 | 1.74E-01 | 7 | -152.078 | 318.155 | 1.776 | 0.17 |
| -0.53 | NA | NA | + | NA | NA | 1.87E-01 | 6 | -163.716 | 339.433 | 0 | 0.393 |
| -0.644 | NA | NA | + | -5.06E-04 | NA | 1.96E-01 | 7 | -163.144 | 340.288 | 0.855 | 0.256 |
| -0.566 | 1.46E-09 | NA | + | NA | NA | 1.92E-01 | 7 | -163.468 | 340.935 | 1.503 | 0.185 |
| -0.448 | NA | NA | + | NA | -3.67E-02 | 1.87E-01 | 7 | -163.585 | 341.169 | 1.737 | 0.165 |
| -0.824 | NA | NA | + | -6.94E-04 | NA | 2.39E-01 | 7 | -166.07 | 346.139 | 0 | 0.206 |
| -0.644 | NA | NA | + | -7.90E-04 | -8.83E-02 | 2.42E-01 | 8 | -165.188 | 346.375 | 0.236 | 0.183 |
| -0.692 | NA | NA | + | NA | NA | 2.27E-01 | 6 | -167.201 | 346.403 | 0.263 | 0.18 |
| -0.881 | 2.02E-09 | NA | + | -7.23E-04 | NA | 2.47E-01 | 8 | -165.597 | 347.194 | 1.055 | 0.121 |
| -0.532 | NA | NA | + | NA | -7.08E-02 | 2.28E-01 | 7 | -166.631 | 347.261 | 1.122 | 0.117 |
| -0.738 | 1.82E-09 | NA | + | NA | NA | 2.34E-01 | 7 | -166.825 | 347.65 | 1.511 | 0.097 |
| -0.705 | 1.76E-09 | NA | + | -8.09E-04 | -8.25E-02 | 2.49E-01 | 9 | -164.831 | 347.661 | 1.522 | 0.096 |
| -0.556 | NA | NA | + | NA | NA | 1.93E-01 | 6 | -150.956 | 313.912 | 0 | 0.299 |
| -0.393 | NA | NA | + | NA | -7.20E-02 | 1.93E-01 | 7 | -150.338 | 314.676 | 0.763 | 0.204 |
| -0.658 | NA | NA | + | -4.64E-04 | NA | 2.02E-01 | 7 | -150.417 | 314.835 | 0.923 | 0.188 |
| -0.486 | NA | NA | + | -5.52E-04 | -8.41E-02 | 2.03E-01 | 8 | -149.587 | 315.174 | 1.262 | 0.159 |
| -0.594 | 1.40E-09 | NA | + | NA | NA | 1.99E-01 | 7 | -150.647 | 315.294 | 1.381 | 0.15 |
| -0.498 | NA | NA | + | NA | NA | 1.78E-01 | 6 | -151.234 | 314.467 | 0 | 0.4 |
| -0.539 | 1.05E-09 | NA | + | NA | NA | 1.87E-01 | 7 | -150.911 | 315.821 | 1.354 | 0.203 |
| -0.58 | NA | NA | + | -3.65E-04 | NA | 1.85E-01 | 7 | -150.919 | 315.838 | 1.371 | 0.202 |
| -0.39 | NA | NA | + | NA | -4.95E-02 | 1.79E-01 | 7 | -150.952 | 315.905 | 1.438 | 0.195 |
| -0.468 | NA | NA | + | NA | NA | 1.71E-01 | 6 | -143.827 | 299.653 | 0 | 0.27 |
| -0.596 | NA | NA | + | -5.21E-04 | NA | 1.84E-01 | 7 | -143.111 | 300.221 | 0.568 | 0.203 |
| -0.333 | NA | NA | + | NA | -6.07E-02 | 1.72E-01 | 7 | -143.401 | 300.802 | 1.148 | 0.152 |
| -0.45 | NA | NA | + | -5.87E-04 | -7.28E-02 | 1.87E-01 | 8 | -142.503 | 301.006 | 1.352 | 0.137 |
| -0.505 | 1.25E-09 | NA | + | NA | NA | 1.77E-01 | 7 | -143.546 | 301.092 | 1.439 | 0.131 |
| -0.646 | 1.42E-09 | NA | + | -5.51E-04 | NA | 1.91E-01 | 8 | -142.748 | 301.495 | 1.842 | 0.107 |
| -0.447 | NA | NA | + | NA | NA | 1.60E-01 | 6 | -161.556 | 335.112 | 0 | 0.457 |
| -0.476 | 9.85E-10 | NA | + | NA | NA | 1.66E-01 | 7 | -161.377 | 336.754 | 1.642 | 0.201 |
| -0.475 | NA | NA | + | -1.26E-04 | NA | 1.63E-01 | 7 | -161.523 | 337.046 | 1.934 | 0.174 |
| -0.445 | NA | NA | + | NA | -8.74E-04 | 1.60E-01 | 7 | -161.556 | 337.112 | 2 | 0.168 |
| -0.425 | NA | NA | + | NA | NA | 1.60E-01 | 6 | -140.546 | 293.092 | 0 | 0.421 |
| -0.501 | NA | NA | + | -3.35E-04 | NA | 1.67E-01 | 7 | -140.263 | 294.526 | 1.434 | 0.206 |
| -0.455 | 9.44E-10 | NA | + | NA | NA | 1.66E-01 | 7 | -140.341 | 294.682 | 1.59 | 0.19 |
| -0.344 | NA | NA | + | NA | -3.76E-02 | 1.62E-01 | 7 | -140.383 | 294.766 | 1.674 | 0.182 |
| -0.392 | NA | NA | + | NA | NA | 1.53E-01 | 6 | -143.367 | 298.734 | 0 | 0.379 |
| -0.419 | 1.13E-09 | NA | + | NA | NA | 1.57E-01 | 7 | -143.193 | 300.385 | 1.651 | 0.166 |
| -0.442 | NA | NA | + | -2.18E-04 | NA | 1.57E-01 | 7 | -143.253 | 300.507 | 1.773 | 0.156 |
| -0.335 | NA | NA | + | NA | -2.63E-02 | 1.54E-01 | 7 | -143.291 | 300.582 | 1.848 | 0.15 |
| -0.329 | NA | NA | NA | NA | NA | 1.89E-01 | 3 | -147.307 | 300.614 | 1.879 | 0.148 |
| -0.676 | NA | NA | + | -1.01E-03 | -1.29E-01 | 2.76E-01 | 8 | -163.168 | 342.336 | 0 | 0.577 |
| -0.76 | 2.01E-09 | NA | + | -1.05E-03 | -1.22E-01 | 2.88E-01 | 9 | -162.479 | 342.957 | 0.621 | 0.423 |
| -0.694 | NA | NA | + | -7.08E-04 | NA | 2.01E-01 | 7 | -149.946 | 313.893 | 0 | 0.268 |
| -0.536 | NA | NA | + | NA | NA | 1.86E-01 | 6 | -151.189 | 314.378 | 0.485 | 0.21 |
| -0.548 | NA | NA | + | -7.58E-04 | -7.15E-02 | 2.03E-01 | 8 | -149.393 | 314.785 | 0.892 | 0.171 |
| -0.74 | 1.48E-09 | NA | + | -7.31E-04 | NA | 2.08E-01 | 8 | -149.612 | 315.224 | 1.331 | 0.138 |
| -0.405 | NA | NA | + | NA | -5.94E-02 | 1.87E-01 | 7 | -150.809 | 315.619 | 1.726 | 0.113 |
| -0.572 | 1.29E-09 | NA | + | NA | NA | 1.92E-01 | 7 | -150.936 | 315.872 | 1.979 | 0.1 |
| -0.508 | NA | NA | + | -7.93E-04 | -1.09E-01 | 2.21E-01 | 8 | -151.633 | 319.266 | 0 | 0.281 |
| -0.736 | NA | NA | + | -7.02E-04 | NA | 2.19E-01 | 7 | -153.095 | 320.191 | 0.925 | 0.177 |
| -0.567 | 1.47E-09 | NA | + | -8.22E-04 | -1.05E-01 | 2.30E-01 | 9 | -151.231 | 320.461 | 1.196 | 0.155 |
| -0.37 | NA | NA | + | NA | -9.49E-02 | 2.02E-01 | 7 | -153.285 | 320.571 | 1.305 | 0.146 |
| -0.585 | NA | NA | + | NA | NA | 2.02E-01 | 6 | -154.39 | 320.779 | 1.513 | 0.132 |
| -0.792 | 1.68E-09 | NA | + | -7.40E-04 | NA | 2.29E-01 | 8 | -152.58 | 321.16 | 1.894 | 0.109 |
| -0.469 | NA | NA | + | NA | NA | 1.71E-01 | 6 | -149.301 | 310.602 | 0 | 0.427 |
| -0.504 | 1.10E-09 | NA | + | NA | NA | 1.77E-01 | 7 | -149.057 | 312.114 | 1.512 | 0.2 |
| -0.533 | NA | NA | + | -2.88E-04 | NA | 1.77E-01 | 7 | -149.108 | 312.216 | 1.614 | 0.19 |
| -0.386 | NA | NA | + | NA | -3.74E-02 | 1.72E-01 | 7 | -149.148 | 312.295 | 1.694 | 0.183 |
| -0.605 | NA | NA | + | NA | NA | 2.05E-01 | 6 | -157.289 | 326.578 | 0 | 0.265 |
| -0.723 | NA | NA | + | -5.73E-04 | NA | 2.16E-01 | 7 | -156.522 | 327.044 | 0.466 | 0.21 |
| -0.656 | 1.82E-09 | NA | + | NA | NA | 2.13E-01 | 7 | -156.858 | 327.715 | 1.137 | 0.15 |
| -0.783 | 1.96E-09 | NA | + | -6.00E-04 | NA | 2.25E-01 | 8 | -156.015 | 328.03 | 1.453 | 0.128 |
| -0.492 | NA | NA | + | NA | -4.99E-02 | 2.05E-01 | 7 | -157.017 | 328.033 | 1.456 | 0.128 |
| -0.593 | NA | NA | + | -6.33E-04 | -6.28E-02 | 2.17E-01 | 8 | -156.095 | 328.189 | 1.612 | 0.118 |
| -0.822 | NA | NA | + | -7.59E-04 | NA | 2.39E-01 | 7 | -158.655 | 331.31 | 0 | 0.22 |
| -0.648 | NA | NA | + | -8.58E-04 | -8.52E-02 | 2.40E-01 | 8 | -157.797 | 331.593 | 0.283 | 0.191 |
| -0.668 | NA | NA | + | NA | NA | 2.27E-01 | 6 | -160.045 | 332.089 | 0.78 | 0.149 |
| -0.887 | 1.97E-09 | NA | + | -8.00E-04 | NA | 2.49E-01 | 8 | -158.052 | 332.104 | 0.795 | 0.148 |
| -0.717 | 1.79E-09 | NA | + | -8.90E-04 | -7.99E-02 | 2.49E-01 | 9 | -157.297 | 332.595 | 1.285 | 0.116 |
| -0.52 | NA | NA | + | NA | -6.50E-02 | 2.26E-01 | 7 | -159.541 | 333.083 | 1.773 | 0.091 |
| -0.717 | 1.70E-09 | NA | + | NA | NA | 2.35E-01 | 7 | -159.598 | 333.197 | 1.887 | 0.086 |
| -0.595 | NA | NA | + | NA | NA | 2.07E-01 | 6 | -160.128 | 332.256 | 0 | 0.392 |
| -0.455 | NA | NA | + | NA | -6.08E-02 | 2.05E-01 | 7 | -159.713 | 333.425 | 1.17 | 0.219 |
| -0.637 | 1.66E-09 | NA | + | NA | NA | 2.13E-01 | 7 | -159.817 | 333.634 | 1.379 | 0.197 |
| -0.669 | NA | NA | + | -3.53E-04 | NA | 2.14E-01 | 7 | -159.842 | 333.683 | 1.428 | 0.192 |
| -0.584 | NA | NA | + | NA | NA | 1.98E-01 | 6 | -156.722 | 325.443 | 0 | 0.242 |
| -0.708 | NA | NA | + | -6.22E-04 | NA | 2.07E-01 | 7 | -155.781 | 325.562 | 0.119 | 0.229 |
| -0.567 | NA | NA | + | -6.98E-04 | -6.98E-02 | 2.09E-01 | 8 | -155.233 | 326.467 | 1.024 | 0.145 |
| -0.76 | 1.72E-09 | NA | + | -6.43E-04 | NA | 2.15E-01 | 8 | -155.341 | 326.682 | 1.239 | 0.131 |
| -0.628 | 1.59E-09 | NA | + | NA | NA | 2.05E-01 | 7 | -156.349 | 326.698 | 1.255 | 0.129 |
| -0.464 | NA | NA | + | NA | -5.36E-02 | 1.99E-01 | 7 | -156.395 | 326.79 | 1.347 | 0.124 |
| -0.541 | NA | NA | + | NA | NA | 1.90E-01 | 6 | -157.491 | 326.983 | 0 | 0.399 |
| -0.582 | 1.55E-09 | NA | + | NA | NA | 1.97E-01 | 7 | -157.172 | 328.343 | 1.36 | 0.202 |
| -0.621 | NA | NA | + | -3.68E-04 | NA | 1.98E-01 | 7 | -157.183 | 328.365 | 1.382 | 0.2 |
| -0.425 | NA | NA | + | NA | -5.15E-02 | 1.91E-01 | 7 | -157.19 | 328.381 | 1.398 | 0.198 |
| -0.777 | NA | NA | + | -7.15E-04 | NA | 2.27E-01 | 7 | -155.795 | 325.589 | 0 | 0.212 |
| -0.599 | NA | NA | + | -8.15E-04 | -9.01E-02 | 2.29E-01 | 8 | -154.837 | 325.674 | 0.085 | 0.203 |
| -0.643 | NA | NA | + | NA | NA | 2.18E-01 | 6 | -157.134 | 326.267 | 0.678 | 0.151 |
| -0.836 | 1.79E-09 | NA | + | -7.45E-04 | NA | 2.37E-01 | 8 | -155.248 | 326.496 | 0.907 | 0.134 |
| -0.661 | 1.60E-09 | NA | + | -8.36E-04 | -8.51E-02 | 2.38E-01 | 9 | -154.395 | 326.791 | 1.202 | 0.116 |
| -0.49 | NA | NA | + | NA | -7.02E-02 | 2.18E-01 | 7 | -156.548 | 327.096 | 1.507 | 0.1 |
| -0.69 | 1.59E-09 | NA | + | NA | NA | 2.26E-01 | 7 | -156.705 | 327.409 | 1.82 | 0.085 |
| -0.476 | NA | NA | + | NA | NA | 1.71E-01 | 6 | -145.464 | 302.928 | 0 | 0.395 |
| -0.582 | NA | NA | + | -4.80E-04 | NA | 1.81E-01 | 7 | -144.897 | 303.793 | 0.865 | 0.256 |
| -0.505 | 1.02E-09 | NA | + | NA | NA | 1.77E-01 | 7 | -145.259 | 304.518 | 1.59 | 0.178 |
| -0.394 | NA | NA | + | NA | -3.71E-02 | 1.72E-01 | 7 | -145.308 | 304.617 | 1.688 | 0.17 |
| -0.836 | NA | NA | + | -7.90E-04 | NA | 2.38E-01 | 7 | -156.894 | 327.787 | 0 | 0.361 |
| -0.898 | 1.63E-09 | NA | + | -8.39E-04 | NA | 2.49E-01 | 8 | -156.342 | 328.684 | 0.896 | 0.231 |
| -0.705 | NA | NA | + | -8.55E-04 | -6.33E-02 | 2.39E-01 | 8 | -156.46 | 328.919 | 1.132 | 0.205 |
| -0.681 | NA | NA | + | NA | NA | 2.26E-01 | 6 | -158.465 | 328.93 | 1.142 | 0.204 |
| -0.531 | NA | NA | + | NA | NA | 1.88E-01 | 6 | -142.032 | 296.064 | 0 | 0.171 |
| -0.474 | NA | NA | + | -6.80E-04 | -8.84E-02 | 2.00E-01 | 8 | -140.032 | 296.065 | 0.001 | 0.171 |
| -0.659 | NA | NA | + | -5.94E-04 | NA | 1.99E-01 | 7 | -141.048 | 296.096 | 0.032 | 0.168 |
| -0.36 | NA | NA | + | NA | -7.43E-02 | 1.87E-01 | 7 | -141.311 | 296.621 | 0.558 | 0.129 |
| -0.717 | 1.50E-09 | NA | + | -6.47E-04 | NA | 2.10E-01 | 8 | -140.511 | 297.021 | 0.958 | 0.106 |
| -0.535 | 1.38E-09 | NA | + | -7.26E-04 | -8.50E-02 | 2.10E-01 | 9 | -139.57 | 297.14 | 1.076 | 0.1 |
| -0.569 | 1.23E-09 | NA | + | NA | NA | 1.96E-01 | 7 | -141.669 | 297.339 | 1.275 | 0.09 |
| -0.403 | 1.11E-09 | NA | + | NA | -7.08E-02 | 1.94E-01 | 8 | -141.016 | 298.032 | 1.968 | 0.064 |
| -0.752 | NA | NA | + | -7.54E-04 | NA | 2.20E-01 | 7 | -151.401 | 316.803 | 0 | 0.35 |
| -0.61 | NA | NA | + | -8.25E-04 | -7.04E-02 | 2.21E-01 | 8 | -150.838 | 317.677 | 0.874 | 0.226 |
| -0.601 | NA | NA | + | NA | NA | 2.07E-01 | 6 | -152.87 | 317.74 | 0.937 | 0.219 |
| -0.808 | 1.56E-09 | NA | + | -7.90E-04 | NA | 2.30E-01 | 8 | -150.931 | 317.862 | 1.059 | 0.206 |
| -0.451 | NA | NA | + | NA | NA | 1.64E-01 | 6 | -149.242 | 310.484 | 0 | 0.431 |
| -0.482 | 8.74E-10 | NA | + | NA | NA | 1.71E-01 | 7 | -149.019 | 312.038 | 1.554 | 0.198 |
| -0.514 | NA | NA | + | -2.95E-04 | NA | 1.70E-01 | 7 | -149.027 | 312.053 | 1.569 | 0.197 |
| -0.387 | NA | NA | + | NA | -2.94E-02 | 1.66E-01 | 7 | -149.151 | 312.303 | 1.819 | 0.174 |
| -0.517 | NA | NA | + | NA | NA | 1.83E-01 | 6 | -152.995 | 317.99 | 0 | 0.391 |
| -0.608 | NA | NA | + | -4.19E-04 | NA | 1.91E-01 | 7 | -152.584 | 319.168 | 1.178 | 0.217 |
| -0.557 | 1.36E-09 | NA | + | NA | NA | 1.90E-01 | 7 | -152.676 | 319.352 | 1.361 | 0.198 |
| -0.403 | NA | NA | + | NA | -5.19E-02 | 1.85E-01 | 7 | -152.696 | 319.392 | 1.401 | 0.194 |
| -0.527 | NA | NA | + | NA | NA | 1.81E-01 | 6 | -159.234 | 330.467 | 0 | 0.399 |
| -0.635 | NA | NA | + | -4.75E-04 | NA | 1.92E-01 | 7 | -158.727 | 331.454 | 0.987 | 0.243 |
| -0.568 | 1.53E-09 | NA | + | NA | NA | 1.87E-01 | 7 | -158.969 | 331.937 | 1.47 | 0.191 |
| -0.448 | NA | NA | + | NA | -3.57E-02 | 1.82E-01 | 7 | -159.103 | 332.206 | 1.739 | 0.167 |
| -0.651 | NA | NA | + | -1.13E-03 | -1.50E-01 | 2.69E-01 | 8 | -159.148 | 334.296 | 0 | 0.604 |
| -0.726 | 2.07E-09 | NA | + | -1.14E-03 | -1.43E-01 | 2.78E-01 | 9 | -158.569 | 335.138 | 0.842 | 0.396 |
| -0.402 | NA | NA | + | NA | NA | 1.50E-01 | 6 | -146.642 | 305.284 | 0 | 0.387 |
| -0.43 | 8.10E-10 | NA | + | NA | NA | 1.56E-01 | 7 | -146.47 | 306.941 | 1.657 | 0.169 |
| -0.45 | NA | NA | + | -2.16E-04 | NA | 1.54E-01 | 7 | -146.533 | 307.066 | 1.782 | 0.159 |
| -0.42 | NA | NA | + | NA | 8.42E-03 | 1.50E-01 | 7 | -146.635 | 307.27 | 1.986 | 0.143 |
| -0.32 | NA | NA | NA | NA | NA | 1.86E-01 | 3 | -150.64 | 307.279 | 1.996 | 0.143 |
| -0.569 | NA | NA | + | NA | NA | 1.97E-01 | 6 | -163.028 | 338.055 | 0 | 0.397 |
| -0.667 | NA | NA | + | -4.60E-04 | NA | 2.05E-01 | 7 | -162.568 | 339.136 | 1.081 | 0.232 |
| -0.615 | 1.66E-09 | NA | + | NA | NA | 2.04E-01 | 7 | -162.691 | 339.382 | 1.327 | 0.205 |
| -0.49 | NA | NA | + | NA | -3.58E-02 | 1.97E-01 | 7 | -162.899 | 339.799 | 1.743 | 0.166 |
| -0.527 | NA | NA | + | NA | NA | 1.84E-01 | 6 | -149.839 | 311.678 | 0 | 0.385 |
| -0.622 | NA | NA | + | -4.31E-04 | NA | 1.92E-01 | 7 | -149.373 | 312.746 | 1.068 | 0.226 |
| -0.405 | NA | NA | + | NA | -5.48E-02 | 1.85E-01 | 7 | -149.495 | 312.989 | 1.311 | 0.2 |
| -0.563 | 1.02E-09 | NA | + | NA | NA | 1.92E-01 | 7 | -149.551 | 313.102 | 1.424 | 0.189 |
| -0.549 | NA | NA | + | NA | NA | 1.89E-01 | 6 | -151.408 | 314.816 | 0 | 0.278 |
| -0.677 | NA | NA | + | -6.01E-04 | NA | 1.98E-01 | 7 | -150.527 | 315.054 | 0.238 | 0.246 |
| -0.583 | 1.19E-09 | NA | + | NA | NA | 1.94E-01 | 7 | -151.163 | 316.326 | 1.51 | 0.131 |
| -0.723 | 1.37E-09 | NA | + | -6.29E-04 | NA | 2.05E-01 | 8 | -150.201 | 316.402 | 1.586 | 0.126 |
| -0.485 | NA | NA | + | NA | -2.88E-02 | 1.90E-01 | 7 | -151.32 | 316.64 | 1.824 | 0.112 |
| -0.594 | NA | NA | + | -6.35E-04 | -4.08E-02 | 1.99E-01 | 8 | -150.351 | 316.702 | 1.886 | 0.108 |
| -0.449 | NA | NA | + | NA | NA | 1.65E-01 | 6 | -158.948 | 329.896 | 0 | 0.446 |
| -0.486 | 1.35E-09 | NA | + | NA | NA | 1.71E-01 | 7 | -158.699 | 331.399 | 1.503 | 0.21 |
| -0.483 | NA | NA | + | -1.51E-04 | NA | 1.69E-01 | 7 | -158.899 | 331.799 | 1.903 | 0.172 |
| -0.402 | NA | NA | + | NA | -2.16E-02 | 1.66E-01 | 7 | -158.902 | 331.803 | 1.908 | 0.172 |
| -0.789 | 2.13E-09 | NA | + | -1.31E-03 | -1.68E-01 | 3.18E-01 | 9 | -161.422 | 340.844 | 0 | 0.558 |
| -0.68 | NA | NA | + | -1.23E-03 | -1.76E-01 | 2.98E-01 | 8 | -162.657 | 341.314 | 0.47 | 0.442 |
| -0.582 | NA | NA | + | NA | NA | 1.96E-01 | 6 | -158.035 | 328.069 | 0 | 0.286 |
| -0.694 | NA | NA | + | -5.41E-04 | NA | 2.06E-01 | 7 | -157.347 | 328.693 | 0.624 | 0.209 |
| -0.624 | 1.50E-09 | NA | + | NA | NA | 2.03E-01 | 7 | -157.706 | 329.413 | 1.344 | 0.146 |
| -0.471 | NA | NA | + | NA | -4.91E-02 | 1.96E-01 | 7 | -157.787 | 329.573 | 1.504 | 0.135 |
| -0.745 | 1.63E-09 | NA | + | -5.64E-04 | NA | 2.14E-01 | 8 | -156.957 | 329.915 | 1.846 | 0.113 |
| -0.568 | NA | NA | + | -5.91E-04 | -6.03E-02 | 2.06E-01 | 8 | -156.975 | 329.95 | 1.88 | 0.112 |
| -0.738 | NA | NA | + | -7.23E-04 | NA | 2.10E-01 | 7 | -152.081 | 318.162 | 0 | 0.201 |
| -0.546 | NA | NA | + | -8.15E-04 | -9.18E-02 | 2.11E-01 | 8 | -151.097 | 318.194 | 0.033 | 0.198 |
| -0.587 | NA | NA | + | NA | NA | 1.97E-01 | 6 | -153.409 | 318.818 | 0.656 | 0.145 |
| -0.806 | 1.70E-09 | NA | + | -7.80E-04 | NA | 2.22E-01 | 8 | -151.428 | 318.856 | 0.694 | 0.142 |
| -0.617 | 1.60E-09 | NA | + | -8.65E-04 | -8.82E-02 | 2.22E-01 | 9 | -150.514 | 319.027 | 0.866 | 0.13 |
| -0.416 | NA | NA | + | NA | -7.41E-02 | 1.97E-01 | 7 | -152.766 | 319.531 | 1.37 | 0.101 |
| -0.632 | 1.41E-09 | NA | + | NA | NA | 2.06E-01 | 7 | -152.966 | 319.932 | 1.77 | 0.083 |
| -0.418 | NA | NA | + | NA | NA | 1.58E-01 | 6 | -147.388 | 306.776 | 0 | 0.44 |
| -0.492 | NA | NA | + | -3.33E-04 | NA | 1.65E-01 | 7 | -147.128 | 308.256 | 1.48 | 0.21 |
| -0.443 | 9.06E-10 | NA | + | NA | NA | 1.63E-01 | 7 | -147.243 | 308.487 | 1.711 | 0.187 |
| -0.437 | NA | NA | + | NA | 9.03E-03 | 1.58E-01 | 7 | -147.38 | 308.759 | 1.983 | 0.163 |
| -0.572 | NA | NA | + | -8.60E-04 | -1.16E-01 | 2.33E-01 | 8 | -150.144 | 316.287 | 0 | 0.43 |
| -0.627 | 1.57E-09 | NA | + | -8.72E-04 | -1.11E-01 | 2.40E-01 | 9 | -149.8 | 317.601 | 1.313 | 0.223 |
| -0.802 | NA | NA | + | -7.09E-04 | NA | 2.31E-01 | 7 | -152.01 | 318.02 | 1.733 | 0.181 |
| -0.438 | NA | NA | + | NA | -9.45E-02 | 2.17E-01 | 7 | -152.089 | 318.178 | 1.891 | 0.167 |
| -0.392 | NA | NA | + | NA | NA | 1.49E-01 | 6 | -147.421 | 306.842 | 0 | 0.357 |
| -0.316 | NA | NA | NA | NA | NA | 1.84E-01 | 3 | -150.976 | 307.952 | 1.11 | 0.205 |
| -0.423 | 1.07E-09 | NA | + | NA | NA | 1.53E-01 | 7 | -147.225 | 308.449 | 1.607 | 0.16 |
| -0.44 | NA | NA | + | -1.96E-04 | NA | 1.53E-01 | 7 | -147.331 | 308.663 | 1.821 | 0.144 |
| -0.363 | NA | NA | + | NA | -1.33E-02 | 1.49E-01 | 7 | -147.403 | 308.806 | 1.964 | 0.134 |
| -0.454 | NA | NA | + | NA | NA | 1.63E-01 | 6 | -152.638 | 317.276 | 0 | 0.433 |
| -0.492 | 1.28E-09 | NA | + | NA | NA | 1.69E-01 | 7 | -152.361 | 318.722 | 1.446 | 0.21 |
| -0.51 | NA | NA | + | -2.46E-04 | NA | 1.67E-01 | 7 | -152.5 | 318.999 | 1.723 | 0.183 |
| -0.389 | NA | NA | + | NA | -2.88E-02 | 1.63E-01 | 7 | -152.553 | 319.105 | 1.829 | 0.174 |
| -0.402 | NA | NA | + | NA | NA | 1.50E-01 | 6 | -142.943 | 297.885 | 0 | 0.417 |
| -0.29 | NA | NA | + | NA | -4.96E-02 | 1.50E-01 | 7 | -142.65 | 299.3 | 1.415 | 0.206 |
| -0.436 | 1.28E-09 | NA | + | NA | NA | 1.55E-01 | 7 | -142.701 | 299.401 | 1.516 | 0.196 |
| -0.463 | NA | NA | + | -2.61E-04 | NA | 1.55E-01 | 7 | -142.774 | 299.549 | 1.663 | 0.182 |
| -0.704 | NA | NA | + | -6.69E-04 | NA | 2.10E-01 | 7 | -146.148 | 306.296 | 0 | 0.221 |
| -0.562 | NA | NA | + | NA | NA | 1.97E-01 | 6 | -147.299 | 306.598 | 0.302 | 0.19 |
| -0.554 | NA | NA | + | -7.42E-04 | -7.66E-02 | 2.14E-01 | 8 | -145.442 | 306.885 | 0.589 | 0.165 |
| -0.755 | 1.57E-09 | NA | + | -6.97E-04 | NA | 2.18E-01 | 8 | -145.71 | 307.42 | 1.124 | 0.126 |
| -0.429 | NA | NA | + | NA | -6.15E-02 | 1.99E-01 | 7 | -146.843 | 307.685 | 1.39 | 0.111 |
| -0.602 | 1.39E-09 | NA | + | NA | NA | 2.04E-01 | 7 | -146.961 | 307.922 | 1.626 | 0.098 |
| -0.608 | 1.45E-09 | NA | + | -7.65E-04 | -7.29E-02 | 2.21E-01 | 9 | -145.069 | 308.138 | 1.842 | 0.088 |
| -0.486 | NA | NA | + | NA | NA | 1.79E-01 | 6 | -153.351 | 318.703 | 0 | 0.427 |
| -0.522 | 1.43E-09 | NA | + | NA | NA | 1.84E-01 | 7 | -153.101 | 320.203 | 1.5 | 0.202 |
| -0.389 | NA | NA | + | NA | -4.41E-02 | 1.80E-01 | 7 | -153.141 | 320.281 | 1.579 | 0.194 |
| -0.538 | NA | NA | + | -2.27E-04 | NA | 1.84E-01 | 7 | -153.235 | 320.469 | 1.766 | 0.177 |
| -0.525 | NA | NA | + | NA | NA | 1.84E-01 | 6 | -156.228 | 324.455 | 0 | 0.328 |
| -0.64 | NA | NA | + | -5.29E-04 | NA | 1.93E-01 | 7 | -155.562 | 325.123 | 0.668 | 0.235 |
| -0.559 | 1.28E-09 | NA | + | NA | NA | 1.90E-01 | 7 | -155.973 | 325.947 | 1.491 | 0.156 |
| -0.421 | NA | NA | + | NA | -4.83E-02 | 1.86E-01 | 7 | -155.976 | 325.952 | 1.497 | 0.155 |
| -0.524 | NA | NA | + | -5.77E-04 | -5.86E-02 | 1.96E-01 | 8 | -155.193 | 326.386 | 1.931 | 0.125 |
| -0.546 | NA | NA | + | NA | NA | 1.90E-01 | 6 | -162.01 | 336.02 | 0 | 0.436 |
| -0.584 | 1.15E-09 | NA | + | NA | NA | 1.98E-01 | 7 | -161.734 | 337.468 | 1.449 | 0.211 |
| -0.466 | NA | NA | + | NA | -3.64E-02 | 1.90E-01 | 7 | -161.875 | 337.75 | 1.73 | 0.183 |
| -0.581 | NA | NA | + | -1.65E-04 | NA | 1.93E-01 | 7 | -161.952 | 337.904 | 1.884 | 0.17 |
| -0.655 | NA | NA | + | -1.21E-03 | -1.60E-01 | 2.76E-01 | 8 | -157.197 | 330.395 | 0 | 0.6 |
| -0.728 | 2.06E-09 | NA | + | -1.21E-03 | -1.52E-01 | 2.84E-01 | 9 | -156.601 | 331.202 | 0.807 | 0.4 |
| -0.417 | NA | NA | + | NA | NA | 1.53E-01 | 6 | -146.349 | 304.699 | 0 | 0.428 |
| -0.449 | 1.20E-09 | NA | + | NA | NA | 1.58E-01 | 7 | -146.125 | 306.249 | 1.551 | 0.197 |
| -0.486 | NA | NA | + | -3.04E-04 | NA | 1.58E-01 | 7 | -146.133 | 306.265 | 1.566 | 0.195 |
| -0.342 | NA | NA | + | NA | -3.34E-02 | 1.53E-01 | 7 | -146.216 | 306.432 | 1.733 | 0.18 |
| -0.542 | NA | NA | + | NA | NA | 1.91E-01 | 6 | -160.525 | 333.051 | 0 | 0.411 |
| -0.584 | 1.53E-09 | NA | + | NA | NA | 1.98E-01 | 7 | -160.202 | 334.403 | 1.353 | 0.209 |
| -0.624 | NA | NA | + | -3.75E-04 | NA | 1.98E-01 | 7 | -160.212 | 334.424 | 1.373 | 0.207 |
| -0.464 | NA | NA | + | NA | -3.49E-02 | 1.92E-01 | 7 | -160.397 | 334.793 | 1.743 | 0.172 |
| -0.361 | NA | NA | + | NA | NA | 1.41E-01 | 6 | -141.159 | 294.319 | 0 | 0.31 |
| -0.312 | NA | NA | NA | NA | NA | 1.79E-01 | 3 | -144.672 | 295.344 | 1.025 | 0.186 |
| -0.226 | NA | NA | NA | 4.14E-04 | NA | 1.57E-01 | 4 | -143.954 | 295.908 | 1.589 | 0.14 |
| -0.389 | 9.94E-10 | NA | + | NA | NA | 1.46E-01 | 7 | -140.995 | 295.99 | 1.671 | 0.134 |
| -0.382 | NA | NA | + | -8.70E-05 | NA | 1.43E-01 | 7 | -141.141 | 296.282 | 1.964 | 0.116 |
| -0.345 | NA | NA | + | NA | -7.05E-03 | 1.42E-01 | 7 | -141.154 | 296.308 | 1.989 | 0.115 |
| -0.525 | NA | NA | + | NA | NA | 1.92E-01 | 6 | -145.881 | 303.762 | 0 | 0.211 |
| -0.648 | NA | NA | + | -5.86E-04 | NA | 2.03E-01 | 7 | -144.966 | 303.932 | 0.17 | 0.194 |
| -0.492 | NA | NA | + | -6.67E-04 | -7.97E-02 | 2.06E-01 | 8 | -144.183 | 304.367 | 0.605 | 0.156 |
| -0.383 | NA | NA | + | NA | -6.52E-02 | 1.93E-01 | 7 | -145.351 | 304.702 | 0.941 | 0.132 |
| -0.699 | 1.62E-09 | NA | + | -6.18E-04 | NA | 2.11E-01 | 8 | -144.505 | 305.011 | 1.249 | 0.113 |
| -0.565 | 1.44E-09 | NA | + | NA | NA | 1.98E-01 | 7 | -145.52 | 305.04 | 1.278 | 0.111 |
| -0.546 | 1.48E-09 | NA | + | -6.92E-04 | -7.57E-02 | 2.13E-01 | 9 | -143.798 | 305.597 | 1.835 | 0.084 |
| -0.445 | NA | NA | + | NA | NA | 1.62E-01 | 6 | -144.355 | 300.71 | 0 | 0.403 |
| -0.539 | NA | NA | + | -4.03E-04 | NA | 1.71E-01 | 7 | -143.942 | 301.884 | 1.174 | 0.224 |
| -0.342 | NA | NA | + | NA | -4.64E-02 | 1.63E-01 | 7 | -144.104 | 302.207 | 1.497 | 0.191 |
| -0.476 | 1.22E-09 | NA | + | NA | NA | 1.66E-01 | 7 | -144.151 | 302.302 | 1.593 | 0.182 |
| -0.363 | NA | NA | + | NA | NA | 1.39E-01 | 6 | -146.009 | 304.019 | 0 | 0.385 |
| -0.391 | 9.64E-10 | NA | + | NA | NA | 1.43E-01 | 7 | -145.854 | 305.708 | 1.689 | 0.165 |
| -0.415 | NA | NA | + | -2.15E-04 | NA | 1.43E-01 | 7 | -145.903 | 305.807 | 1.788 | 0.157 |
| -0.3 | NA | NA | NA | NA | NA | 1.79E-01 | 3 | -149.942 | 305.883 | 1.865 | 0.151 |
| -0.364 | NA | NA | + | NA | 2.29E-04 | 1.39E-01 | 7 | -146.009 | 306.019 | 2 | 0.141 |
| -0.449 | NA | NA | + | NA | NA | 1.64E-01 | 6 | -144.955 | 301.909 | 0 | 0.414 |
| -0.534 | NA | NA | + | -3.91E-04 | NA | 1.71E-01 | 7 | -144.573 | 303.146 | 1.237 | 0.223 |
| -0.479 | 9.41E-10 | NA | + | NA | NA | 1.70E-01 | 7 | -144.747 | 303.495 | 1.585 | 0.187 |
| -0.369 | NA | NA | + | NA | -3.61E-02 | 1.64E-01 | 7 | -144.809 | 303.619 | 1.709 | 0.176 |
| -0.511 | NA | NA | + | NA | NA | 1.82E-01 | 6 | -155.548 | 323.096 | 0 | 0.427 |
| -0.584 | NA | NA | + | -3.38E-04 | NA | 1.88E-01 | 7 | -155.29 | 324.581 | 1.485 | 0.203 |
| -0.546 | 1.33E-09 | NA | + | NA | NA | 1.87E-01 | 7 | -155.324 | 324.648 | 1.552 | 0.196 |
| -0.443 | NA | NA | + | NA | -3.10E-02 | 1.82E-01 | 7 | -155.446 | 324.892 | 1.796 | 0.174 |
| -0.524 | NA | NA | + | NA | NA | 1.82E-01 | 6 | -160.561 | 333.121 | 0 | 0.432 |
| -0.599 | NA | NA | + | -3.42E-04 | NA | 1.89E-01 | 7 | -160.302 | 334.605 | 1.484 | 0.206 |
| -0.559 | 1.16E-09 | NA | + | NA | NA | 1.89E-01 | 7 | -160.341 | 334.682 | 1.561 | 0.198 |
| -0.484 | NA | NA | + | NA | -1.73E-02 | 1.82E-01 | 7 | -160.531 | 335.063 | 1.942 | 0.164 |
| -0.312 | NA | NA | NA | NA | NA | 1.81E-01 | 3 | -160.571 | 327.142 | 0 | 0.236 |
| -0.206 | NA | NA | NA | 5.14E-04 | NA | 1.53E-01 | 4 | -159.69 | 327.381 | 0.239 | 0.209 |
| -0.36 | NA | NA | + | NA | NA | 1.43E-01 | 6 | -157.768 | 327.535 | 0.393 | 0.194 |
| -0.338 | 8.82E-10 | NA | NA | NA | NA | 1.85E-01 | 4 | -160.462 | 328.925 | 1.783 | 0.097 |
| -0.471 | NA | NA | + | NA | 5.21E-02 | 1.40E-01 | 7 | -157.542 | 329.083 | 1.941 | 0.089 |
| -0.337 | NA | NA | NA | NA | 1.23E-02 | 1.81E-01 | 4 | -160.557 | 329.115 | 1.973 | 0.088 |
| -0.274 | NA | NA | NA | 5.61E-04 | 3.80E-02 | 1.51E-01 | 5 | -159.568 | 329.136 | 1.994 | 0.087 |
| -0.326 | NA | NA | + | NA | NA | 1.32E-01 | 6 | -137.788 | 287.575 | 0 | 0.309 |
| -0.254 | NA | NA | NA | NA | NA | 1.65E-01 | 3 | -141.187 | 288.374 | 0.799 | 0.207 |
| -0.378 | NA | NA | + | -2.01E-04 | NA | 1.38E-01 | 7 | -137.688 | 289.376 | 1.801 | 0.125 |
| -0.347 | 7.84E-10 | NA | + | NA | NA | 1.36E-01 | 7 | -137.692 | 289.384 | 1.808 | 0.125 |
| -0.189 | NA | NA | NA | 3.30E-04 | NA | 1.47E-01 | 4 | -140.749 | 289.498 | 1.922 | 0.118 |
| -0.299 | NA | NA | + | NA | -1.28E-02 | 1.33E-01 | 7 | -137.77 | 289.54 | 1.965 | 0.116 |
| -0.435 | NA | NA | + | NA | NA | 1.54E-01 | 6 | -163.852 | 339.704 | 0 | 0.457 |
| -0.469 | 1.09E-09 | NA | + | NA | NA | 1.60E-01 | 7 | -163.666 | 341.332 | 1.628 | 0.203 |
| -0.467 | NA | NA | + | NA | 1.46E-02 | 1.53E-01 | 7 | -163.833 | 341.666 | 1.962 | 0.171 |
| -0.44 | NA | NA | + | -2.07E-05 | NA | 1.54E-01 | 7 | -163.851 | 341.703 | 1.998 | 0.168 |
| -0.543 | NA | NA | + | NA | NA | 1.91E-01 | 6 | -152.405 | 316.81 | 0 | 0.304 |
| -0.375 | NA | NA | + | NA | -7.28E-02 | 1.90E-01 | 7 | -151.756 | 317.512 | 0.702 | 0.214 |
| -0.641 | NA | NA | + | -4.32E-04 | NA | 2.00E-01 | 7 | -151.949 | 317.899 | 1.089 | 0.176 |
| -0.583 | 1.29E-09 | NA | + | NA | NA | 1.98E-01 | 7 | -152.088 | 318.177 | 1.367 | 0.153 |
| -0.467 | NA | NA | + | -5.27E-04 | -8.46E-02 | 2.00E-01 | 8 | -151.089 | 318.177 | 1.368 | 0.153 |
| -0.383 | NA | NA | + | NA | NA | 1.53E-01 | 6 | -138.036 | 288.072 | 0 | 0.362 |
| -0.321 | NA | NA | NA | NA | NA | 1.85E-01 | 3 | -141.683 | 289.367 | 1.295 | 0.19 |
| -0.408 | 8.15E-10 | NA | + | NA | NA | 1.58E-01 | 7 | -137.873 | 289.745 | 1.673 | 0.157 |
| -0.441 | NA | NA | + | -2.50E-04 | NA | 1.58E-01 | 7 | -137.875 | 289.75 | 1.679 | 0.156 |
| -0.361 | NA | NA | + | NA | -1.06E-02 | 1.54E-01 | 7 | -138.024 | 290.048 | 1.976 | 0.135 |
| -0.577 | NA | NA | + | NA | NA | 1.97E-01 | 6 | -157.081 | 326.161 | 0 | 0.25 |
| -0.711 | NA | NA | + | -6.19E-04 | NA | 2.08E-01 | 7 | -156.159 | 326.318 | 0.157 | 0.231 |
| -0.579 | NA | NA | + | -6.79E-04 | -6.55E-02 | 2.11E-01 | 8 | -155.696 | 327.393 | 1.231 | 0.135 |
| -0.766 | 1.65E-09 | NA | + | -6.52E-04 | NA | 2.17E-01 | 8 | -155.72 | 327.439 | 1.278 | 0.132 |
| -0.62 | 1.46E-09 | NA | + | NA | NA | 2.04E-01 | 7 | -156.74 | 327.48 | 1.319 | 0.129 |
| -0.463 | NA | NA | + | NA | -5.19E-02 | 1.99E-01 | 7 | -156.789 | 327.577 | 1.416 | 0.123 |
| -0.586 | NA | NA | + | NA | NA | 2.00E-01 | 6 | -159.932 | 331.864 | 0 | 0.198 |
| -0.721 | NA | NA | + | -6.22E-04 | NA | 2.12E-01 | 7 | -159.016 | 332.033 | 0.169 | 0.182 |
| -0.541 | NA | NA | + | -7.19E-04 | -8.93E-02 | 2.13E-01 | 8 | -158.077 | 332.154 | 0.29 | 0.171 |
| -0.42 | NA | NA | + | NA | -7.39E-02 | 1.99E-01 | 7 | -159.283 | 332.567 | 0.703 | 0.14 |
| -0.633 | 1.73E-09 | NA | + | NA | NA | 2.07E-01 | 7 | -159.531 | 333.062 | 1.198 | 0.109 |
| -0.778 | 1.88E-09 | NA | + | -6.46E-04 | NA | 2.20E-01 | 8 | -158.541 | 333.081 | 1.217 | 0.108 |
| -0.601 | 1.66E-09 | NA | + | -7.34E-04 | -8.45E-02 | 2.20E-01 | 9 | -157.701 | 333.402 | 1.538 | 0.092 |
| -0.495 | NA | NA | + | NA | NA | 1.78E-01 | 6 | -163.834 | 339.669 | 0 | 0.456 |
| -0.528 | 1.20E-09 | NA | + | NA | NA | 1.83E-01 | 7 | -163.64 | 341.28 | 1.611 | 0.204 |
| -0.516 | NA | NA | + | -9.16E-05 | NA | 1.80E-01 | 7 | -163.818 | 341.636 | 1.967 | 0.171 |
| -0.469 | NA | NA | + | NA | -1.15E-02 | 1.78E-01 | 7 | -163.822 | 341.645 | 1.976 | 0.17 |
| -0.432 | NA | NA | + | NA | NA | 1.59E-01 | 6 | -140.655 | 293.31 | 0 | 0.417 |
| -0.514 | NA | NA | + | -3.59E-04 | NA | 1.67E-01 | 7 | -140.33 | 294.66 | 1.35 | 0.213 |
| -0.466 | 1.28E-09 | NA | + | NA | NA | 1.64E-01 | 7 | -140.4 | 294.8 | 1.49 | 0.198 |
| -0.362 | NA | NA | + | NA | -3.13E-02 | 1.60E-01 | 7 | -140.542 | 295.084 | 1.775 | 0.172 |
| -0.446 | NA | NA | + | NA | NA | 1.67E-01 | 6 | -144.781 | 301.561 | 0 | 0.412 |
| -0.531 | NA | NA | + | -3.93E-04 | NA | 1.75E-01 | 7 | -144.401 | 302.801 | 1.24 | 0.221 |
| -0.477 | 1.13E-09 | NA | + | NA | NA | 1.72E-01 | 7 | -144.551 | 303.103 | 1.541 | 0.19 |
| -0.365 | NA | NA | + | NA | -3.66E-02 | 1.67E-01 | 7 | -144.628 | 303.257 | 1.695 | 0.176 |
| -0.164 | NA | NA | NA | 6.15E-04 | NA | 1.43E-01 | 4 | -170.158 | 348.316 | 0 | 0.294 |
| -0.283 | NA | NA | NA | NA | NA | 1.74E-01 | 3 | -171.255 | 348.509 | 0.193 | 0.267 |
| -0.356 | NA | NA | + | NA | NA | 1.41E-01 | 6 | -168.623 | 349.245 | 0.929 | 0.185 |
| -0.257 | NA | NA | NA | 6.89E-04 | 5.37E-02 | 1.39E-01 | 5 | -169.932 | 349.864 | 1.547 | 0.136 |
| -0.189 | 9.57E-10 | NA | NA | 6.17E-04 | NA | 1.46E-01 | 5 | -170.065 | 350.13 | 1.813 | 0.119 |
| -0.39 | NA | NA | + | NA | NA | 1.51E-01 | 6 | -157.645 | 327.291 | 0 | 0.28 |
| -0.307 | NA | NA | NA | NA | NA | 1.84E-01 | 3 | -160.911 | 327.822 | 0.532 | 0.215 |
| -0.217 | NA | NA | NA | 5.06E-04 | NA | 1.60E-01 | 4 | -160.114 | 328.228 | 0.938 | 0.175 |
| -0.417 | 9.91E-10 | NA | + | NA | NA | 1.55E-01 | 7 | -157.502 | 329.005 | 1.714 | 0.119 |
| -0.428 | NA | NA | + | NA | 1.74E-02 | 1.51E-01 | 7 | -157.619 | 329.238 | 1.947 | 0.106 |
| -0.37 | NA | NA | + | 8.72E-05 | NA | 1.49E-01 | 7 | -157.63 | 329.26 | 1.969 | 0.105 |
| -0.422 | NA | NA | + | NA | NA | 1.53E-01 | 6 | -150.591 | 313.183 | 0 | 0.445 |
| -0.451 | 9.95E-10 | NA | + | NA | NA | 1.57E-01 | 7 | -150.425 | 314.851 | 1.668 | 0.193 |
| -0.486 | NA | NA | + | -2.63E-04 | NA | 1.58E-01 | 7 | -150.432 | 314.865 | 1.682 | 0.192 |
| -0.378 | NA | NA | + | NA | -2.01E-02 | 1.54E-01 | 7 | -150.551 | 315.101 | 1.918 | 0.17 |
| -0.501 | NA | NA | + | NA | NA | 1.74E-01 | 6 | -155.944 | 323.888 | 0 | 0.43 |
| -0.583 | NA | NA | + | -3.46E-04 | NA | 1.82E-01 | 7 | -155.678 | 325.355 | 1.467 | 0.206 |
| -0.535 | 1.31E-09 | NA | + | NA | NA | 1.79E-01 | 7 | -155.741 | 325.483 | 1.594 | 0.194 |
| -0.438 | NA | NA | + | NA | -2.74E-02 | 1.74E-01 | 7 | -155.868 | 325.737 | 1.848 | 0.17 |
| -0.658 | NA | NA | + | NA | NA | 2.18E-01 | 6 | -174.825 | 361.649 | 0 | 0.41 |
| -0.746 | NA | NA | + | -4.55E-04 | NA | 2.25E-01 | 7 | -174.414 | 362.828 | 1.178 | 0.227 |
| -0.702 | 1.46E-09 | NA | + | NA | NA | 2.26E-01 | 7 | -174.522 | 363.044 | 1.395 | 0.204 |
| -0.604 | NA | NA | + | NA | -2.42E-02 | 2.18E-01 | 7 | -174.771 | 363.542 | 1.892 | 0.159 |
| -0.608 | NA | NA | + | NA | NA | 2.05E-01 | 6 | -165.807 | 343.614 | 0 | 0.392 |
| -0.699 | NA | NA | + | -4.48E-04 | NA | 2.14E-01 | 7 | -165.365 | 344.73 | 1.116 | 0.225 |
| -0.653 | 1.61E-09 | NA | + | NA | NA | 2.13E-01 | 7 | -165.446 | 344.893 | 1.278 | 0.207 |
| -0.509 | NA | NA | + | NA | -4.49E-02 | 2.06E-01 | 7 | -165.61 | 345.22 | 1.605 | 0.176 |
| -0.486 | NA | NA | + | NA | NA | 1.74E-01 | 6 | -163.368 | 338.737 | 0 | 0.455 |
| -0.52 | 9.82E-10 | NA | + | NA | NA | 1.81E-01 | 7 | -163.159 | 340.317 | 1.58 | 0.207 |
| -0.505 | NA | NA | + | -8.41E-05 | NA | 1.75E-01 | 7 | -163.354 | 340.708 | 1.971 | 0.17 |
| -0.504 | NA | NA | + | NA | 8.32E-03 | 1.73E-01 | 7 | -163.362 | 340.724 | 1.987 | 0.168 |
| -0.609 | NA | NA | + | -9.01E-04 | -1.11E-01 | 2.45E-01 | 8 | -150.855 | 317.711 | 0 | 0.48 |
| -0.673 | 1.68E-09 | NA | + | -9.24E-04 | -1.06E-01 | 2.53E-01 | 9 | -150.412 | 318.824 | 1.113 | 0.275 |
| -0.832 | NA | NA | + | -7.65E-04 | NA | 2.44E-01 | 7 | -152.529 | 319.058 | 1.348 | 0.245 |
| -0.632 | NA | NA | + | -1.00E-03 | -1.32E-01 | 2.55E-01 | 8 | -158.164 | 332.328 | 0 | 0.599 |
| -0.711 | 2.16E-09 | NA | + | -1.01E-03 | -1.24E-01 | 2.65E-01 | 9 | -157.567 | 333.133 | 0.805 | 0.401 |
| -0.499 | NA | NA | + | NA | NA | 1.82E-01 | 6 | -156.038 | 324.076 | 0 | 0.426 |
| -0.537 | 1.29E-09 | NA | + | NA | NA | 1.88E-01 | 7 | -155.755 | 325.511 | 1.435 | 0.208 |
| -0.57 | NA | NA | + | -3.16E-04 | NA | 1.89E-01 | 7 | -155.813 | 325.625 | 1.55 | 0.196 |
| -0.439 | NA | NA | + | NA | -2.70E-02 | 1.82E-01 | 7 | -155.962 | 325.925 | 1.849 | 0.169 |
| -0.52 | NA | NA | + | NA | NA | 1.78E-01 | 6 | -149.89 | 311.78 | 0 | 0.27 |
| -0.643 | NA | NA | + | -5.42E-04 | NA | 1.89E-01 | 7 | -149.153 | 312.305 | 0.525 | 0.208 |
| -0.387 | NA | NA | + | NA | -5.93E-02 | 1.79E-01 | 7 | -149.484 | 312.968 | 1.189 | 0.149 |
[truncated: 50,862 more chars]
