# Supplementary material for: Traits and ecological space availability predict avian densities at the country scale of the Czech Republic
Source: Ecol Evol. 2022 Jul 17;12(7):e9119. doi: 10.1002/ece3.9119 (PMC9289119; doi:10.1002/ece3.9119)
Supplement: Supplementary file 3 — Table S2 [file ECE3-12-e9119-s003.docx]

| (Intercept) | Range | Forest dependency | Nest type | sfPC | SSI diet | SSI habitat | df | logLik | AIC | delta | weight |
| --- | --- | --- | --- | --- | --- | --- | --- | --- | --- | --- | --- |
| -0.638 | NA | NA | + | NA | NA | 2.06E-01 | 6 | -156.805 | 325.61 | 0 | 0.204 |
| -0.731 | NA | NA | + | -1.33E-03 | NA | 2.11E-01 | 7 | -156.094 | 326.188 | 0.579 | 0.153 |
| -0.543 | NA | NA | + | -1.62E-03 | -9.26E-02 | 2.14E-01 | 8 | -155.125 | 326.25 | 0.64 | 0.148 |
| -0.47 | NA | NA | + | NA | -7.46E-02 | 2.08E-01 | 7 | -156.16 | 326.32 | 0.711 | 0.143 |
| -0.686 | 1.66E-09 | NA | + | NA | NA | 2.14E-01 | 7 | -156.377 | 326.755 | 1.145 | 0.115 |
| -0.776 | 1.62E-09 | NA | + | -1.31E-03 | NA | 2.19E-01 | 8 | -155.683 | 327.365 | 1.756 | 0.085 |
| -0.591 | 1.45E-09 | NA | + | -1.59E-03 | -8.87E-02 | 2.21E-01 | 9 | -154.794 | 327.588 | 1.978 | 0.076 |
| -0.523 | 1.53E-09 | NA | + | NA | -7.08E-02 | 2.15E-01 | 8 | -155.796 | 327.592 | 1.983 | 0.076 |
| -0.329 | NA | NA | + | NA | NA | 1.32E-01 | 6 | -149.411 | 310.823 | 0 | 0.208 |
| -0.183 | NA | NA | NA | 1.42E-03 | NA | 1.41E-01 | 4 | -151.422 | 310.844 | 0.021 | 0.206 |
| -0.28 | NA | NA | NA | NA | NA | 1.68E-01 | 3 | -152.593 | 311.187 | 0.364 | 0.174 |
| -0.355 | 7.55E-10 | NA | + | NA | NA | 1.38E-01 | 7 | -149.28 | 312.559 | 1.737 | 0.087 |
| -0.209 | 7.14E-10 | NA | NA | 1.40E-03 | NA | 1.47E-01 | 5 | -151.307 | 312.614 | 1.792 | 0.085 |
| -0.384 | NA | NA | + | NA | 2.54E-02 | 1.31E-01 | 7 | -149.351 | 312.702 | 1.879 | 0.081 |
| -0.228 | NA | NA | NA | 1.53E-03 | 2.54E-02 | 1.40E-01 | 5 | -151.363 | 312.726 | 1.903 | 0.08 |
| -0.308 | NA | NA | + | 2.43E-04 | NA | 1.31E-01 | 7 | -149.393 | 312.787 | 1.964 | 0.078 |
| -0.53 | NA | NA | + | NA | NA | 1.86E-01 | 6 | -156.812 | 325.625 | 0 | 0.413 |
| -0.599 | NA | NA | + | -9.38E-04 | NA | 1.89E-01 | 7 | -156.5 | 327.001 | 1.376 | 0.207 |
| -0.57 | 1.40E-09 | NA | + | NA | NA | 1.92E-01 | 7 | -156.522 | 327.044 | I.42 | 0.203 |
| -0.446 | NA | NA | + | NA | -3.83E-02 | 1.86E-01 | 7 | -156.657 | 327.314 | 1.689 | 0.177 |
| -0.479 | NA | NA | + | NA | NA | 1.73E-01 | 6 | -159.511 | 331.022 | 0 | 0.441 |
| -0.515 | 1.25E-09 | NA | + | NA | NA | 1.79E-01 | 7 | -159.264 | 332.527 | 1.506 | 0.208 |
| -0.412 | NA | NA | + | NA | -3.07E-02 | 1.74E-01 | 7 | -159.417 | 332.835 | 1.813 | 0.178 |
| -0.512 | NA | NA | + | -4.18E-04 | NA | 1.75E-01 | 7 | -159.45 | 332.899 | 1.878 | 0.173 |
| -0.602 | NA | NA | + | NA | NA | 1.98E-01 | 6 | -168.814 | 349.628 | 0 | 0.421 |
| -0.675 | NA | NA | + | -1.03E-03 | NA | 2.01E-01 | 7 | -168.472 | 350.945 | 1.317 | 0.218 |
| -0.642 | 1.16E-09 | NA | + | NA | NA | 2.06E-01 | 7 | -168.551 | 351.101 | 1.474 | 0.202 |
| -0.568 | NA | NA | + | NA | -1.59E-02 | 1.98E-01 | 7 | -168.791 | 351.583 | 1.955 | 0.159 |
| -0.503 | NA | NA | + | NA | NA | 1.80E-01 | 6 | -147.089 | 306.177 | 0 | 0.334 |
| -0.591 | NA | NA | + | -1.13E-03 | NA | 1.86E-01 | 7 | -146.593 | 307.185 | 1.008 | 0.202 |
| -0.382 | NA | NA | + | NA | -5.59E-02 | 1.82E-01 | 7 | -146.727 | 307.453 | 1.276 | 0.177 |
| -0.539 | 1.34E-09 | NA | + | NA | NA | 1.86E-01 | 7 | -146.836 | 307.672 | 1.495 | 0.158 |
| -0.457 | NA | NA | + | -1.35E-03 | -7.01E-02 | 1.89E-01 | 8 | -146.038 | 308.077 | 01.IX | 0.129 |
| -0.495 | NA | NA | + | NA | NA | 1.76E-01 | 6 | -147.614 | 307.229 | 0 | 0.4 |
| -0.372 | NA | NA | + | NA | -5.50E-02 | 1.78E-01 | 7 | -147.249 | 308.499 | I.27 | 0.212 |
| -0.532 | 1.40E-09 | NA | + | NA | NA | 1.81E-01 | 7 | -147.333 | 308.665 | 1.436 | 0.195 |
| -0.562 | NA | NA | + | -8.48E-04 | NA | 1.80E-01 | 7 | -147.343 | 308.686 | 1.458 | 0.193 |
| -0.419 | NA | NA | + | NA | NA | 1.57E-01 | 6 | -152.483 | 316.966 | 0 | 0.376 |
| -0.453 | 1.26E-09 | NA | + | NA | NA | 1.62E-01 | 7 | -152.252 | 318.504 | 1.538 | 0.174 |
| -0.335 | NA | NA | NA | NA | NA | 1.95E-01 | 3 | -156.296 | 318.591 | 1.626 | 0.167 |
| -0.448 | NA | NA | + | -3.49E-04 | NA | 1.58E-01 | 7 | -152.443 | 318.886 | 1.921 | 0.144 |
| -0.399 | NA | NA | + | NA | -9.02E-03 | 1.57E-01 | 7 | -152.475 | 318.949 | 1.984 | 0.139 |
| -0.469 | NA | NA | + | NA | NA | 1.67E-01 | 6 | -149.44 | 310.881 | 0 | 0.439 |
| -0.501 | 9.41E-10 | NA | + | NA | NA | 1.74E-01 | 7 | -149.224 | 312.448 | 1.567 | 0.2 |
| -0.514 | NA | NA | + | -5.81E-04 | NA | 1.70E-01 | 7 | -149.326 | 312.651 | I.77 | 0.181 |
| -0.4 | NA | NA | + | NA | -3.12E-02 | 1.68E-01 | 7 | -149.334 | 312.668 | 1.787 | 0.18 |
| -0.584 | NA | NA | + | NA | NA | 2.00E-01 | 6 | -153.382 | 318.764 | 0 | 0.324 |
| -0.67 | NA | NA | + | -1.19E-03 | NA | 2.05E-01 | 7 | -152.824 | 319.648 | 0.883 | 0.208 |
| -0.462 | NA | NA | + | NA | -5.74E-02 | 2.02E-01 | 7 | -153.02 | 320.04 | 1.275 | 0.171 |
| -0.624 | 1.45E-09 | NA | + | NA | NA | 2.07E-01 | 7 | -153.073 | 320.145 | 1.381 | 0.162 |
| -0.531 | NA | NA | + | -1.41E-03 | -7.25E-02 | 2.08E-01 | 8 | -152.259 | 320.518 | 1.753 | 0.135 |
| -0.471 | NA | NA | + | NA | NA | 1.75E-01 | 6 | -143.739 | 299.477 | 0 | 0.396 |
| -0.546 | NA | NA | + | -9.85E-04 | NA | 1.78E-01 | 7 | -143.334 | 300.668 | I.19 | 0.219 |
| -0.368 | NA | NA | + | NA | -4.95E-02 | 1.78E-01 | 7 | -143.438 | 300.876 | 1.399 | 0.197 |
| -0.503 | 1.03E-09 | NA | + | NA | NA | 1.80E-01 | 7 | -143.484 | 300.968 | I.49 | 0.188 |
| -0.601 | NA | NA | + | NA | NA | 2.03E-01 | 6 | -168.225 | 348.45 | 0 | 0.397 |
| -0.688 | NA | NA | + | -1.20E-03 | NA | 2.09E-01 | 7 | -167.765 | 349.529 | 01.VIII | 0.231 |
| -0.643 | 1.57E-09 | NA | + | NA | NA | 2.10E-01 | 7 | -167.916 | 349.832 | 1.382 | 0.199 |
| -0.508 | NA | NA | + | NA | -4.12E-02 | 2.03E-01 | 7 | -168.052 | 350.104 | 1.654 | 0.173 |
| -0.424 | NA | NA | + | NA | NA | 1.59E-01 | 6 | -150.545 | 313.09 | 0 | 0.436 |
| -0.457 | 1.17E-09 | NA | + | NA | NA | 1.64E-01 | 7 | -150.31 | 314.621 | 1.531 | 0.203 |
| -0.473 | NA | NA | + | -6.30E-04 | NA | 1.61E-01 | 7 | -150.403 | 314.806 | 1.716 | 0.185 |
| -0.362 | NA | NA | + | NA | -2.90E-02 | 1.60E-01 | 7 | -150.455 | 314.91 | I.82 | 0.176 |
| -0.498 | NA | NA | + | NA | NA | 1.70E-01 | 6 | -152.537 | 317.074 | 0 | 0.437 |
| -0.53 | 1.19E-09 | NA | + | NA | NA | 1.75E-01 | 7 | -152.322 | 318.643 | 1.569 | 0.199 |
| -0.551 | NA | NA | + | -7.02E-04 | NA | 1.73E-01 | 7 | -152.367 | 318.734 | I.66 | 0.191 |
| -0.439 | NA | NA | + | NA | -2.67E-02 | 1.71E-01 | 7 | -152.465 | 318.93 | 1.856 | 0.173 |
| -0.49 | NA | NA | + | NA | NA | 1.75E-01 | 6 | -151.014 | 314.029 | 0 | 0.407 |
| -0.557 | NA | NA | + | -8.77E-04 | NA | 1.79E-01 | 7 | -150.725 | 315.449 | 1.421 | 0.2 |
| -0.527 | 1.24E-09 | NA | + | NA | NA | 1.81E-01 | 7 | -150.735 | 315.471 | 1.442 | 0.198 |
| -0.381 | NA | NA | + | NA | -4.92E-02 | 1.75E-01 | 7 | -150.746 | 315.493 | 1.464 | 0.196 |
| -0.462 | NA | NA | + | NA | NA | 1.71E-01 | 6 | -152.708 | 317.416 | 0 | 0.438 |
| -0.494 | 1.24E-09 | NA | + | NA | NA | 1.76E-01 | 7 | -152.499 | 318.998 | 1.582 | 0.198 |
| -0.527 | NA | NA | + | -7.85E-04 | NA | 1.76E-01 | 7 | -152.5 | 318.999 | 1.584 | 0.198 |
| -0.427 | NA | NA | + | NA | -1.64E-02 | 1.72E-01 | 7 | -152.68 | 319.36 | 1.944 | 0.166 |
| -0.592 | NA | NA | + | NA | NA | 1.98E-01 | 6 | -159.091 | 330.182 | 0 | 0.306 |
| -0.679 | NA | NA | + | -1.26E-03 | NA | 2.02E-01 | 7 | -158.479 | 330.957 | 0.775 | 0.208 |
| -0.456 | NA | NA | + | NA | -6.32E-02 | 2.01E-01 | 7 | -158.647 | 331.294 | 1.111 | 0.176 |
| -0.633 | 1.47E-09 | NA | + | NA | NA | 2.05E-01 | 7 | -158.735 | 331.469 | 1.287 | 0.161 |
| -0.526 | NA | NA | + | -1.49E-03 | -7.81E-02 | 2.06E-01 | 8 | -157.814 | 331.627 | 1.445 | 0.149 |
| -0.494 | NA | NA | + | NA | NA | 1.83E-01 | 6 | -148.186 | 308.371 | 0 | 0.393 |
| -0.575 | NA | NA | + | -1.06E-03 | NA | 1.88E-01 | 7 | -147.764 | 309.527 | 1.156 | 0.22 |
| -0.384 | NA | NA | + | NA | -5.07E-02 | 1.83E-01 | 7 | -147.886 | 309.772 | 1.401 | 0.195 |
| -0.53 | 1.33E-09 | NA | + | NA | NA | 1.88E-01 | 7 | -147.904 | 309.808 | 1.437 | 0.192 |
| -0.486 | NA | NA | + | NA | NA | 1.73E-01 | 6 | -157.086 | 326.172 | 0 | 0.435 |
| -0.523 | 1.37E-09 | NA | + | NA | NA | 1.78E-01 | 7 | -156.816 | 327.633 | I.46 | 0.21 |
| -0.41 | NA | NA | + | NA | -3.45E-02 | 1.74E-01 | 7 | -156.966 | 327.931 | 1.759 | 0.181 |
| -0.526 | NA | NA | + | -5.07E-04 | NA | 1.75E-01 | 7 | -157.002 | 328.004 | 1.832 | 0.174 |
| -0.41 | NA | NA | + | NA | NA | 1.56E-01 | 6 | -150.137 | 312.274 | 0 | 0.31 |
| -0.253 | NA | NA | NA | 1.34E-03 | NA | 1.69E-01 | 4 | -152.79 | 313.581 | 1.307 | 0.162 |
| -0.332 | NA | NA | NA | NA | NA | 1.91E-01 | 3 | -153.857 | 313.715 | 1.441 | 0.151 |
| -0.44 | 1.16E-09 | NA | + | NA | NA | 1.61E-01 | 7 | -149.935 | 313.87 | 1.596 | 0.14 |
| -0.352 | NA | NA | + | NA | -2.69E-02 | 1.58E-01 | 7 | -150.065 | 314.13 | 1.856 | 0.123 |
| -0.404 | NA | NA | + | 6.59E-05 | NA | 1.56E-01 | 7 | -150.136 | 314.271 | 1.997 | 0.114 |
| -0.589 | NA | NA | + | NA | NA | 1.99E-01 | 6 | -152.698 | 317.397 | 0 | 0.253 |
| -0.422 | NA | NA | + | NA | -7.35E-02 | 1.98E-01 | 7 | -152.055 | 318.11 | 0.713 | 0.177 |
| -0.676 | NA | NA | + | -1.24E-03 | NA | 2.03E-01 | 7 | -152.066 | 318.132 | 0.735 | 0.175 |
| -0.491 | NA | NA | + | -1.51E-03 | -8.95E-02 | 2.03E-01 | 8 | -151.134 | 318.267 | 0.87 | 0.164 |
| -0.632 | 1.62E-09 | NA | + | NA | NA | 2.06E-01 | 7 | -152.32 | 318.64 | 1.243 | 0.136 |
| -0.719 | 1.62E-09 | NA | + | -1.24E-03 | NA | 2.10E-01 | 8 | -151.682 | 319.365 | 1.968 | 0.095 |
| -0.543 | NA | NA | + | NA | NA | 1.84E-01 | 6 | -150.227 | 312.455 | 0 | 0.26 |
| -0.657 | NA | NA | + | -1.39E-03 | NA | 1.92E-01 | 7 | -149.505 | 313.011 | 0.556 | 0.197 |
| -0.393 | NA | NA | + | NA | -6.70E-02 | 1.85E-01 | 7 | -149.688 | 313.375 | 0.92 | 0.164 |
| -0.494 | NA | NA | + | -1.62E-03 | -8.07E-02 | 1.95E-01 | 8 | -148.73 | 313.46 | 1.006 | 0.157 |
| -0.577 | 1.08E-09 | NA | + | NA | NA | 1.91E-01 | 7 | -149.973 | 313.946 | 1.491 | 0.123 |
| -0.696 | 1.15E-09 | NA | + | -1.43E-03 | NA | 2.00E-01 | 8 | -149.211 | 314.422 | 1.967 | 0.097 |
| -0.545 | NA | NA | + | NA | NA | 1.95E-01 | 6 | -153.623 | 319.246 | 0 | 0.391 |
| -0.404 | NA | NA | + | NA | -6.20E-02 | 1.94E-01 | 7 | -153.171 | 320.342 | 1.097 | 0.226 |
| -0.591 | 1.71E-09 | NA | + | NA | NA | 2.02E-01 | 7 | -153.228 | 320.455 | 1.209 | 0.214 |
| -0.594 | NA | NA | + | -6.47E-04 | NA | 1.98E-01 | 7 | -153.461 | 320.921 | 1.675 | 0.169 |
| -0.596 | NA | NA | + | NA | NA | 2.05E-01 | 6 | -151.681 | 315.363 | 0 | 0.204 |
| -0.402 | NA | NA | + | NA | -8.64E-02 | 2.05E-01 | 7 | -150.756 | 315.511 | 0.149 | 0.19 |
| -0.472 | NA | NA | + | -1.47E-03 | -1.02E-01 | 2.12E-01 | 8 | -149.826 | 315.652 | 0.289 | 0.177 |
| -0.679 | NA | NA | + | -1.16E-03 | NA | 2.10E-01 | 7 | -151.09 | 316.179 | 0.816 | 0.136 |
| -0.633 | 1.13E-09 | NA | + | NA | NA | 2.13E-01 | 7 | -151.322 | 316.644 | 1.281 | 0.108 |
| -0.443 | 1.03E-09 | NA | + | NA | -8.37E-02 | 2.13E-01 | 8 | -150.453 | 316.906 | 1.543 | 0.094 |
| -0.516 | 1.09E-09 | NA | + | -1.50E-03 | -9.95E-02 | 2.20E-01 | 9 | -149.486 | 316.972 | 1.609 | 0.091 |
| -0.363 | NA | NA | + | NA | NA | 1.42E-01 | 6 | -139.912 | 291.823 | 0 | 0.319 |
| -0.281 | NA | NA | NA | NA | NA | 1.72E-01 | 3 | -143.579 | 293.157 | 1.334 | 0.164 |
| -0.214 | NA | NA | NA | 1.10E-03 | NA | 1.52E-01 | 4 | -142.718 | 293.437 | 1.614 | 0.142 |
| -0.387 | 7.81E-10 | NA | + | NA | NA | 1.46E-01 | 7 | -139.77 | 293.539 | 1.716 | 0.135 |
| -0.393 | NA | NA | + | -3.48E-04 | NA | 1.44E-01 | 7 | -139.871 | 293.742 | 1.919 | 0.122 |
| -0.381 | NA | NA | + | NA | 8.58E-03 | 1.41E-01 | 7 | -139.904 | 293.808 | 1.984 | 0.118 |
| -0.515 | NA | NA | + | NA | NA | 1.78E-01 | 6 | -159.117 | 330.235 | 0 | 0.42 |
| -0.557 | 1.53E-09 | NA | + | NA | NA | 1.84E-01 | 7 | -158.797 | 331.595 | I.36 | 0.213 |
| -0.42 | NA | NA | + | NA | -4.23E-02 | 1.78E-01 | 7 | -158.936 | 331.872 | 1.637 | 0.185 |
| -0.572 | NA | NA | + | -7.11E-04 | NA | 1.82E-01 | 7 | -158.948 | 331.897 | 1.662 | 0.183 |
| -0.374 | NA | NA | + | NA | NA | 1.52E-01 | 6 | -137.207 | 286.414 | 0 | 0.288 |
| -0.298 | NA | NA | NA | NA | NA | 1.80E-01 | 3 | -140.536 | 287.072 | 0.658 | 0.207 |
| -0.238 | NA | NA | NA | 1.04E-03 | NA | 1.63E-01 | 4 | -139.785 | 287.569 | 1.155 | 0.162 |
| -0.401 | 1.00E-09 | NA | + | NA | NA | 1.56E-01 | 7 | -137.036 | 288.071 | 1.657 | 0.126 |
| -0.334 | NA | NA | + | NA | -1.84E-02 | 1.53E-01 | 7 | -137.169 | 288.338 | 1.923 | 0.11 |
| -0.393 | NA | NA | + | -2.26E-04 | NA | 1.54E-01 | 7 | -137.188 | 288.376 | 1.962 | 0.108 |
| -0.679 | NA | NA | + | NA | NA | 2.16E-01 | 6 | -168.227 | 348.455 | 0 | 0.374 |
| -0.732 | 1.87E-09 | NA | + | NA | NA | 2.24E-01 | 7 | -167.762 | 349.524 | 1.069 | 0.219 |
| -0.753 | NA | NA | + | -1.11E-03 | NA | 2.19E-01 | 7 | -167.809 | 349.618 | 1.163 | 0.209 |
| -0.547 | NA | NA | + | NA | -5.86E-02 | 2.17E-01 | 7 | -167.866 | 349.733 | 1.278 | 0.197 |
| -0.579 | NA | NA | + | NA | NA | 1.95E-01 | 6 | -153.287 | 318.573 | 0 | 0.252 |
| -0.68 | NA | NA | + | -1.45E-03 | NA | 2.00E-01 | 7 | -152.468 | 318.935 | 0.362 | 0.21 |
| -0.529 | NA | NA | + | -1.69E-03 | -7.59E-02 | 2.02E-01 | 8 | -151.817 | 319.634 | 1.061 | 0.148 |
| -0.625 | 1.71E-09 | NA | + | NA | NA | 2.02E-01 | 7 | -152.891 | 319.781 | 1.208 | 0.138 |
| -0.45 | NA | NA | + | NA | -5.80E-02 | 1.96E-01 | 7 | -152.898 | 319.797 | 1.224 | 0.137 |
| -0.725 | 1.68E-09 | NA | + | -1.45E-03 | NA | 2.07E-01 | 8 | -152.079 | 320.157 | 1.584 | 0.114 |
| -0.499 | NA | NA | + | NA | NA | 1.83E-01 | 6 | -159.64 | 331.281 | 0 | 0.433 |
| -0.538 | 1.50E-09 | NA | + | NA | NA | 1.89E-01 | 7 | -159.346 | 332.692 | 1.411 | 0.214 |
| -0.413 | NA | NA | + | NA | -3.82E-02 | 1.83E-01 | 7 | -159.484 | 332.968 | 1.687 | 0.186 |
| -0.528 | NA | NA | + | -3.71E-04 | NA | 1.85E-01 | 7 | -159.593 | 333.187 | 1.906 | 0.167 |
| -0.594 | NA | NA | + | -1.87E-03 | -1.02E-01 | 2.29E-01 | 8 | -153.86 | 323.72 | 0 | 0.216 |
| -0.803 | NA | NA | + | -1.55E-03 | NA | 2.27E-01 | 7 | -155.152 | 324.303 | 0.583 | 0.161 |
| -0.702 | NA | NA | + | NA | NA | 2.23E-01 | 6 | -156.176 | 324.352 | 0.632 | 0.157 |
| -0.517 | NA | NA | + | NA | -8.19E-02 | 2.25E-01 | 7 | -155.329 | 324.658 | 0.938 | 0.135 |
| -0.655 | 1.45E-09 | NA | + | -1.88E-03 | -9.61E-02 | 2.39E-01 | 9 | -153.416 | 324.831 | 1.111 | 0.124 |
| -0.86 | 1.69E-09 | NA | + | -1.58E-03 | NA | 2.38E-01 | 8 | -154.555 | 325.111 | 1.391 | 0.108 |
| -0.754 | 1.62E-09 | NA | + | NA | NA | 2.34E-01 | 7 | -155.632 | 325.264 | 1.544 | 0.1 |
| -0.439 | NA | NA | + | NA | NA | 1.55E-01 | 6 | -150.582 | 313.164 | 0 | 0.438 |
| -0.472 | 1.24E-09 | NA | + | NA | NA | 1.60E-01 | 7 | -150.368 | 314.736 | 1.573 | 0.2 |
| -0.351 | NA | NA | + | NA | -3.89E-02 | 1.57E-01 | 7 | -150.42 | 314.839 | 1.675 | 0.19 |
| -0.475 | NA | NA | + | -4.45E-04 | NA | 1.58E-01 | 7 | -150.513 | 315.027 | 1.863 | 0.173 |
| -0.485 | NA | NA | + | NA | NA | 1.69E-01 | 6 | -145.662 | 303.325 | 0 | 0.39 |
| -0.351 | NA | NA | + | NA | -6.03E-02 | 1.71E-01 | 7 | -145.226 | 304.453 | 1.128 | 0.222 |
| -0.561 | NA | NA | + | -9.72E-04 | NA | 1.74E-01 | 7 | -145.289 | 304.579 | 1.254 | 0.208 |
| -0.516 | 1.09E-09 | NA | + | NA | NA | 1.75E-01 | 7 | -145.435 | 304.87 | 1.545 | 0.18 |
| -0.75 | NA | NA | + | NA | NA | 2.47E-01 | 6 | -171.18 | 354.361 | 0 | 0.224 |
| -0.57 | NA | NA | + | NA | -7.84E-02 | 2.45E-01 | 7 | -170.49 | 354.981 | 0.62 | 0.165 |
| -0.811 | 2.29E-09 | NA | + | NA | NA | 2.56E-01 | 7 | -170.593 | 355.186 | 0.825 | 0.149 |
| -0.819 | NA | NA | + | -1.17E-03 | NA | 2.48E-01 | 7 | -170.646 | 355.293 | 0.932 | 0.141 |
| -0.621 | NA | NA | + | -1.43E-03 | -9.24E-02 | 2.46E-01 | 8 | -169.707 | 355.413 | 1.053 | 0.133 |
| -0.641 | 2.03E-09 | NA | + | NA | -7.10E-02 | 2.53E-01 | 8 | -170.031 | 356.063 | 1.702 | 0.096 |
| -0.879 | 2.28E-09 | NA | + | -1.17E-03 | NA | 2.57E-01 | 8 | -170.06 | 356.12 | 1.759 | 0.093 |
| -0.197 | NA | NA | NA | 1.54E-03 | NA | 1.46E-01 | 4 | -159.544 | 327.088 | 0 | 0.326 |
| -0.309 | NA | NA | NA | NA | NA | 1.78E-01 | 3 | -160.923 | 327.845 | 0.757 | 0.223 |
| -0.365 | NA | NA | + | NA | NA | 1.41E-01 | 6 | -158.108 | 328.215 | 1.127 | 0.186 |
| -0.224 | 1.13E-09 | NA | NA | 1.53E-03 | NA | 1.50E-01 | 5 | -159.405 | 328.811 | 1.723 | 0.138 |
| -0.24 | NA | NA | NA | 1.65E-03 | 2.53E-02 | 1.44E-01 | 5 | -159.488 | 328.977 | 1.889 | 0.127 |
| -0.656 | NA | NA | + | NA | NA | 2.14E-01 | 6 | -161.667 | 335.335 | 0 | 0.33 |
| -0.726 | NA | NA | + | -1.06E-03 | NA | 2.17E-01 | 7 | -161.229 | 336.459 | 1.124 | 0.188 |
| -0.703 | 1.75E-09 | NA | + | NA | NA | 2.22E-01 | 7 | -161.271 | 336.541 | 1.207 | 0.181 |
| -0.523 | NA | NA | + | NA | -5.76E-02 | 2.13E-01 | 7 | -161.282 | 336.564 | I.23 | 0.179 |
| -0.577 | NA | NA | + | -1.28E-03 | -7.07E-02 | 2.16E-01 | 8 | -160.663 | 337.326 | 1.991 | 0.122 |
| -0.565 | NA | NA | + | NA | NA | 1.97E-01 | 6 | -152.813 | 317.626 | 0 | 0.302 |
| -0.662 | NA | NA | + | -1.34E-03 | NA | 2.03E-01 | 7 | -152.103 | 318.206 | 0.58 | 0.226 |
| -0.44 | NA | NA | + | NA | -5.82E-02 | 2.00E-01 | 7 | -152.421 | 318.843 | 1.217 | 0.165 |
| -0.518 | NA | NA | + | -1.57E-03 | -7.51E-02 | 2.07E-01 | 8 | -151.467 | 318.933 | 1.307 | 0.157 |
| -0.603 | 1.42E-09 | NA | + | NA | NA | 2.03E-01 | 7 | -152.518 | 319.035 | 1.409 | 0.149 |
| -0.454 | NA | NA | + | NA | NA | 1.67E-01 | 6 | -145.034 | 302.067 | 0 | 0.424 |
| -0.519 | NA | NA | + | -8.25E-04 | NA | 1.70E-01 | 7 | -144.784 | 303.567 | 01.V | 0.2 |
| -0.486 | 1.18E-09 | NA | + | NA | NA | 1.72E-01 | 7 | -144.804 | 303.608 | I.54 | 0.196 |
| -0.379 | NA | NA | + | NA | -3.51E-02 | 1.69E-01 | 7 | -144.893 | 303.786 | 1.718 | 0.18 |
| -0.427 | NA | NA | + | NA | NA | 1.61E-01 | 6 | -150.937 | 313.874 | 0 | 0.447 |
| -0.457 | 1.05E-09 | NA | + | NA | NA | 1.66E-01 | 7 | -150.752 | 315.505 | 1.631 | 0.198 |
| -0.365 | NA | NA | + | NA | -2.96E-02 | 1.63E-01 | 7 | -150.846 | 315.691 | 1.817 | 0.18 |
| -0.462 | NA | NA | + | -4.19E-04 | NA | 1.64E-01 | 7 | -150.878 | 315.756 | 1.882 | 0.175 |
| -0.517 | NA | NA | + | NA | NA | 1.84E-01 | 6 | -155.584 | 323.167 | 0 | 0.425 |
| -0.554 | 1.22E-09 | NA | + | NA | NA | 1.92E-01 | 7 | -155.31 | 324.619 | 1.452 | 0.206 |
| -0.42 | NA | NA | + | NA | -4.32E-02 | 1.85E-01 | 7 | -155.381 | 324.762 | 1.595 | 0.192 |
| -0.562 | NA | NA | + | -5.89E-04 | NA | 1.88E-01 | 7 | -155.46 | 324.92 | 1.753 | 0.177 |
| -0.459 | NA | NA | + | NA | NA | 1.68E-01 | 6 | -146.183 | 304.365 | 0 | 0.417 |
| -0.493 | 9.63E-10 | NA | + | NA | NA | 1.76E-01 | 7 | -145.89 | 305.779 | 1.414 | 0.206 |
| -0.52 | NA | NA | + | -7.59E-04 | NA | 1.72E-01 | 7 | -145.969 | 305.938 | 1.573 | 0.19 |
| -0.368 | NA | NA | + | NA | -4.26E-02 | 1.71E-01 | 7 | -145.981 | 305.961 | 1.596 | 0.188 |
| -0.498 | NA | NA | + | NA | NA | 1.78E-01 | 6 | -159.36 | 330.719 | 0 | 0.448 |
| -0.534 | 1.20E-09 | NA | + | NA | NA | 1.85E-01 | 7 | -159.12 | 332.239 | I.52 | 0.209 |
| -0.443 | NA | NA | + | NA | -2.44E-02 | 1.79E-01 | 7 | -159.298 | 332.597 | 1.877 | 0.175 |
| -0.518 | NA | NA | + | -2.43E-04 | NA | 1.80E-01 | 7 | -159.341 | 332.681 | 1.962 | 0.168 |
| -0.547 | NA | NA | + | NA | NA | 1.94E-01 | 6 | -161.817 | 335.635 | 0 | 0.44 |
| -0.586 | 1.51E-09 | NA | + | NA | NA | 2.00E-01 | 7 | -161.55 | 337.099 | 1.464 | 0.212 |
| -0.583 | NA | NA | + | -4.76E-04 | NA | 1.97E-01 | 7 | -161.742 | 337.485 | I.85 | 0.174 |
| -0.488 | NA | NA | + | NA | -2.69E-02 | 1.95E-01 | 7 | -161.746 | 337.491 | 1.856 | 0.174 |
| -0.486 | NA | NA | + | NA | NA | 1.76E-01 | 6 | -157.133 | 326.265 | 0 | 0.445 |
| -0.521 | 1.04E-09 | NA | + | NA | NA | 1.84E-01 | 7 | -156.89 | 327.78 | 1.514 | 0.209 |
| -0.519 | NA | NA | + | -4.33E-04 | NA | 1.78E-01 | 7 | -157.071 | 328.142 | 1.877 | 0.174 |
| -0.44 | NA | NA | + | NA | -2.15E-02 | 1.77E-01 | 7 | -157.087 | 328.175 | 1.909 | 0.171 |
| -0.131 | NA | NA | NA | 1.37E-03 | NA | 1.26E-01 | 4 | -147.883 | 303.766 | 0 | 0.306 |
| -0.209 | NA | NA | NA | NA | NA | 1.50E-01 | 3 | -149.022 | 304.044 | 0.278 | 0.266 |
| -0.296 | NA | NA | + | NA | NA | 1.25E-01 | 6 | -146.429 | 304.858 | 1.092 | 0.177 |
| -0.199 | NA | NA | NA | 1.54E-03 | 3.96E-02 | 1.23E-01 | 5 | -147.748 | 305.495 | 1.729 | 0.129 |
| -0.151 | 5.93E-10 | NA | NA | 1.36E-03 | NA | 1.30E-01 | 5 | -147.798 | 305.597 | 1.831 | 0.122 |
| -0.44 | NA | NA | + | NA | NA | 1.62E-01 | 6 | -143.045 | 298.09 | 0 | 0.44 |
| -0.472 | 1.06E-09 | NA | + | NA | NA | 1.67E-01 | 7 | -142.815 | 299.629 | 1.539 | 0.204 |
| -0.488 | NA | NA | + | -5.86E-04 | NA | 1.65E-01 | 7 | -142.921 | 299.842 | 1.752 | 0.183 |
| -0.385 | NA | NA | + | NA | -2.44E-02 | 1.63E-01 | 7 | -142.981 | 299.961 | 1.871 | 0.173 |
| -0.412 | NA | NA | + | NA | NA | 1.52E-01 | 6 | -150.605 | 313.21 | 0 | 0.446 |
| -0.444 | 9.69E-10 | NA | + | NA | NA | 1.58E-01 | 7 | -150.403 | 314.807 | 1.597 | 0.201 |
| -0.462 | NA | NA | + | -6.17E-04 | NA | 1.55E-01 | 7 | -150.475 | 314.95 | 1.741 | 0.187 |
| -0.388 | NA | NA | + | NA | -1.07E-02 | 1.53E-01 | 7 | -150.593 | 315.187 | 1.977 | 0.166 |
| -0.48 | NA | NA | + | NA | NA | 1.70E-01 | 6 | -157.388 | 326.777 | 0 | 0.446 |
| -0.516 | 1.14E-09 | NA | + | NA | NA | 1.76E-01 | 7 | -157.16 | 328.32 | 1.543 | 0.206 |
| -0.423 | NA | NA | + | NA | -2.59E-02 | 1.70E-01 | 7 | -157.319 | 328.639 | 1.862 | 0.176 |
| -0.511 | NA | NA | + | -4.02E-04 | NA | 1.72E-01 | 7 | -157.337 | 328.673 | 1.896 | 0.173 |
| -0.398 | NA | NA | + | NA | NA | 1.52E-01 | 6 | -141.643 | 295.287 | 0 | 0.444 |
| -0.426 | 1.01E-09 | NA | + | NA | NA | 1.57E-01 | 7 | -141.472 | 296.944 | 1.657 | 0.194 |
| -0.446 | NA | NA | + | -5.47E-04 | NA | 1.55E-01 | 7 | -141.537 | 297.074 | 1.787 | 0.182 |
| -0.336 | NA | NA | + | NA | -2.85E-02 | 1.54E-01 | 7 | -141.55 | 297.1 | 1.813 | 0.18 |
| -0.626 | NA | NA | + | -1.87E-03 | -1.15E-01 | 2.53E-01 | 8 | -164.014 | 344.029 | 0 | 0.274 |
| -0.544 | NA | NA | + | NA | -9.65E-02 | 2.47E-01 | 7 | -165.407 | 344.813 | 0.785 | 0.185 |
| -0.767 | NA | NA | + | NA | NA | 2.50E-01 | 6 | -166.616 | 345.231 | 1.203 | 0.15 |
| -0.686 | 1.75E-09 | NA | + | -1.84E-03 | -1.08E-01 | 2.60E-01 | 9 | -163.631 | 345.262 | 1.234 | 0.148 |
| -0.867 | NA | NA | + | -1.51E-03 | NA | 2.55E-01 | 7 | -165.691 | 345.382 | 1.354 | 0.139 |
| -0.61 | 1.85E-09 | NA | + | NA | -9.02E-02 | 2.55E-01 | 8 | -164.984 | 345.969 | I.94 | 0.104 |
| -0.518 | NA | NA | + | -1.70E-03 | -1.08E-01 | 2.23E-01 | 8 | -152.08 | 320.161 | 0 | 0.206 |
| -0.44 | NA | NA | + | NA | -8.96E-02 | 2.17E-01 | 7 | -153.339 | 320.678 | 0.517 | 0.159 |
| -0.645 | NA | NA | + | NA | NA | 2.19E-01 | 6 | -154.357 | 320.714 | 0.554 | 0.156 |
| -0.741 | NA | NA | + | -1.38E-03 | NA | 2.24E-01 | 7 | -153.523 | 321.047 | 0.886 | 0.132 |
| -0.569 | 1.51E-09 | NA | + | -1.69E-03 | -1.02E-01 | 2.28E-01 | 9 | -151.777 | 321.554 | 1.393 | 0.102 |
| -0.693 | 1.83E-09 | NA | + | NA | NA | 2.26E-01 | 7 | -153.92 | 321.84 | 1.679 | 0.089 |
| -0.494 | 1.55E-09 | NA | + | NA | -8.43E-02 | 2.23E-01 | 8 | -153.024 | 322.047 | 1.886 | 0.08 |
| -0.79 | 1.85E-09 | NA | + | -1.38E-03 | NA | 2.30E-01 | 8 | -153.074 | 322.148 | 1.987 | 0.076 |
| -0.468 | NA | NA | + | NA | NA | 1.67E-01 | 6 | -146.311 | 304.622 | 0 | 0.434 |
| -0.499 | 1.00E-09 | NA | + | NA | NA | 1.73E-01 | 7 | -146.092 | 306.184 | 1.562 | 0.199 |
| -0.523 | NA | NA | + | -6.86E-04 | NA | 1.71E-01 | 7 | -146.147 | 306.293 | 1.672 | 0.188 |
| -0.398 | NA | NA | + | NA | -3.18E-02 | 1.68E-01 | 7 | -146.202 | 306.403 | 1.781 | 0.178 |
| -0.409 | NA | NA | + | NA | NA | 1.57E-01 | 6 | -150.237 | 312.474 | 0 | 0.329 |
| -0.242 | NA | NA | NA | 1.44E-03 | NA | 1.67E-01 | 4 | -152.968 | 313.935 | 1.461 | 0.159 |
| -0.438 | 9.99E-10 | NA | + | NA | NA | 1.62E-01 | 7 | -150.055 | 314.11 | 1.636 | 0.145 |
| -0.322 | NA | NA | NA | NA | NA | 1.91E-01 | 3 | -154.216 | 314.432 | 1.958 | 0.124 |
| -0.4 | NA | NA | + | 1.06E-04 | NA | 1.57E-01 | 7 | -150.233 | 314.467 | 1.993 | 0.122 |
| -0.409 | NA | NA | + | NA | 4.27E-04 | 1.57E-01 | 7 | -150.237 | 314.474 | 2 | 0.121 |
| -0.526 | NA | NA | + | NA | NA | 1.80E-01 | 6 | -151.542 | 315.084 | 0 | 0.402 |
| -0.609 | NA | NA | + | -1.13E-03 | NA | 1.86E-01 | 7 | -151.067 | 316.133 | 1.049 | 0.238 |
| -0.563 | 1.26E-09 | NA | + | NA | NA | 1.86E-01 | 7 | -151.295 | 316.591 | 1.507 | 0.189 |
| -0.446 | NA | NA | + | NA | -3.64E-02 | 1.81E-01 | 7 | -151.398 | 316.797 | 1.713 | 0.171 |
| -0.39 | NA | NA | + | NA | NA | 1.52E-01 | 6 | -146.183 | 304.366 | 0 | 0.333 |
| -0.418 | 9.79E-10 | NA | + | NA | NA | 1.57E-01 | 7 | -146.009 | 306.017 | 1.651 | 0.146 |
| -0.323 | NA | NA | NA | NA | NA | 1.89E-01 | 3 | -150.065 | 306.13 | 1.763 | 0.138 |
| -0.333 | NA | NA | + | NA | -2.58E-02 | 1.53E-01 | 7 | -146.113 | 306.226 | I.86 | 0.131 |
| -0.241 | NA | NA | NA | 1.23E-03 | NA | 1.65E-01 | 4 | -149.15 | 306.3 | 1.933 | 0.127 |
| -0.41 | NA | NA | + | -2.21E-04 | NA | 1.53E-01 | 7 | -146.167 | 306.334 | 1.968 | 0.125 |
| -0.699 | NA | NA | + | NA | NA | 2.33E-01 | 6 | -162.056 | 336.112 | 0 | 0.234 |
| -0.53 | NA | NA | + | NA | -7.46E-02 | 2.32E-01 | 7 | -161.379 | 336.759 | 0.646 | 0.17 |
| -0.769 | NA | NA | + | -1.12E-03 | NA | 2.36E-01 | 7 | -161.53 | 337.061 | 0.949 | 0.146 |
| -0.584 | NA | NA | + | -1.40E-03 | -8.94E-02 | 2.35E-01 | 8 | -160.583 | 337.165 | 1.053 | 0.138 |
| -0.747 | 1.89E-09 | NA | + | NA | NA | 2.40E-01 | 7 | -161.598 | 337.196 | 1.083 | 0.136 |
| -0.586 | 1.67E-09 | NA | + | NA | -6.89E-02 | 2.39E-01 | 8 | -161.024 | 338.048 | 1.936 | 0.089 |
| -0.82 | 1.92E-09 | NA | + | -1.14E-03 | NA | 2.43E-01 | 8 | -161.053 | 338.106 | 1.994 | 0.086 |
| -0.516 | NA | NA | + | NA | NA | 1.82E-01 | 6 | -156.802 | 325.605 | 0 | 0.43 |
| -0.548 | 1.12E-09 | NA | + | NA | NA | 1.88E-01 | 7 | -156.574 | 327.148 | 1.543 | 0.199 |
| -0.576 | NA | NA | + | -8.03E-04 | NA | 1.85E-01 | 7 | -156.584 | 327.168 | 1.563 | 0.197 |
| -0.452 | NA | NA | + | NA | -2.94E-02 | 1.83E-01 | 7 | -156.71 | 327.421 | 1.816 | 0.174 |
| -0.538 | NA | NA | + | NA | NA | 1.83E-01 | 6 | -172.9 | 357.799 | 0 | 0.454 |
| -0.577 | 1.41E-09 | NA | + | NA | NA | 1.89E-01 | 7 | -172.674 | 359.348 | 1.549 | 0.209 |
| -0.551 | NA | NA | + | -1.85E-04 | NA | 1.84E-01 | 7 | -172.889 | 359.779 | I.98 | 0.169 |
| -0.522 | NA | NA | + | NA | -7.28E-03 | 1.84E-01 | 7 | -172.895 | 359.79 | 1.991 | 0.168 |
| -0.592 | NA | NA | + | NA | NA | 1.99E-01 | 6 | -156.127 | 324.254 | 0 | 0.281 |
| -0.682 | NA | NA | + | -1.31E-03 | NA | 2.03E-01 | 7 | -155.46 | 324.92 | 0.667 | 0.201 |
| -0.635 | 1.47E-09 | NA | + | NA | NA | 2.07E-01 | 7 | -155.761 | 325.522 | 1.268 | 0.149 |
| -0.475 | NA | NA | + | NA | -5.21E-02 | 2.00E-01 | 7 | -155.821 | 325.642 | 1.388 | 0.14 |
| -0.545 | NA | NA | + | -1.50E-03 | -6.67E-02 | 2.05E-01 | 8 | -154.967 | 325.934 | 1.681 | 0.121 |
| -0.725 | 1.48E-09 | NA | + | -1.31E-03 | NA | 2.10E-01 | 8 | -155.086 | 326.173 | 1.919 | 0.108 |
| -0.493 | NA | NA | + | NA | NA | 1.69E-01 | 6 | -149.04 | 310.08 | 0 | 0.401 |
| -0.359 | NA | NA | + | NA | -5.77E-02 | 1.69E-01 | 7 | -148.644 | 311.288 | 1.208 | 0.219 |
| -0.533 | 1.36E-09 | NA | + | NA | NA | 1.75E-01 | 7 | -148.725 | 311.45 | I.37 | 0.202 |
| -0.552 | NA | NA | + | -7.16E-04 | NA | 1.72E-01 | 7 | -148.856 | 311.711 | 1.631 | 0.177 |
| -0.442 | NA | NA | + | NA | NA | 1.63E-01 | 6 | -146.718 | 305.435 | 0 | 0.454 |
| -0.47 | 9.77E-10 | NA | + | NA | NA | 1.68E-01 | 7 | -146.565 | 307.131 | 1.695 | 0.194 |
| -0.479 | NA | NA | + | -4.64E-04 | NA | 1.66E-01 | 7 | -146.641 | 307.282 | 1.847 | 0.18 |
| -0.405 | NA | NA | + | NA | -1.68E-02 | 1.64E-01 | 7 | -146.687 | 307.375 | I.94 | 0.172 |
| -0.359 | NA | NA | + | NA | NA | 1.40E-01 | 6 | -137.823 | 287.646 | 0 | 0.451 |
| -0.384 | 7.42E-10 | NA | + | NA | NA | 1.45E-01 | 7 | -137.682 | 289.364 | 1.717 | 0.191 |
| -0.413 | NA | NA | + | -6.06E-04 | NA | 1.44E-01 | 7 | -137.69 | 289.379 | 1.733 | 0.19 |
| -0.332 | NA | NA | + | NA | -1.25E-02 | 1.41E-01 | 7 | -137.806 | 289.613 | 1.966 | 0.169 |
| -0.576 | NA | NA | + | NA | NA | 2.01E-01 | 6 | -153.64 | 319.28 | 0 | 0.276 |
| -0.673 | NA | NA | + | -1.30E-03 | NA | 2.08E-01 | 7 | -153.008 | 320.015 | 0.736 | 0.191 |
| -0.445 | NA | NA | + | NA | -5.91E-02 | 2.01E-01 | 7 | -153.245 | 320.491 | 1.211 | 0.15 |
| -0.618 | 1.43E-09 | NA | + | NA | NA | 2.08E-01 | 7 | -153.271 | 320.543 | 1.263 | 0.147 |
| -0.523 | NA | NA | + | -1.55E-03 | -7.59E-02 | 2.10E-01 | 8 | -152.374 | 320.748 | 1.469 | 0.132 |
| -0.718 | 1.47E-09 | NA | + | -1.33E-03 | NA | 2.15E-01 | 8 | -152.611 | 321.223 | 1.943 | 0.104 |
| -0.463 | NA | NA | + | -1.72E-03 | -1.00E-01 | 2.03E-01 | 8 | -141.34 | 298.68 | 0 | 0.219 |
| -0.552 | NA | NA | + | NA | NA | 1.94E-01 | 6 | -143.525 | 299.05 | 0.369 | 0.182 |
| -0.373 | NA | NA | + | NA | -8.29E-02 | 1.95E-01 | 7 | -142.622 | 299.245 | 0.564 | 0.165 |
| -0.658 | NA | NA | + | -1.42E-03 | NA | 2.00E-01 | 7 | -142.636 | 299.272 | 0.591 | 0.163 |
| -0.501 | 1.09E-09 | NA | + | -1.72E-03 | -9.67E-02 | 2.08E-01 | 9 | -141.117 | 300.234 | 1.553 | 0.101 |
| -0.588 | 1.28E-09 | NA | + | NA | NA | 2.00E-01 | 7 | -143.226 | 300.453 | 1.772 | 0.09 |
| -0.695 | 1.30E-09 | NA | + | -1.43E-03 | NA | 2.06E-01 | 8 | -142.322 | 300.645 | 1.964 | 0.082 |
| -0.556 | NA | NA | + | NA | NA | 1.97E-01 | 6 | -164.688 | 341.377 | 0 | 0.435 |
| -0.596 | 1.32E-09 | NA | + | NA | NA | 2.04E-01 | 7 | -164.4 | 342.799 | 1.423 | 0.213 |
| -0.471 | NA | NA | + | NA | -3.72E-02 | 1.96E-01 | 7 | -164.552 | 343.103 | 1.727 | 0.183 |
| -0.589 | NA | NA | + | -4.01E-04 | NA | 1.99E-01 | 7 | -164.637 | 343.273 | 1.896 | 0.168 |
| -0.574 | NA | NA | + | NA | NA | 1.99E-01 | 6 | -162.864 | 337.727 | 0 | 0.4 |
| -0.442 | NA | NA | + | NA | -5.83E-02 | 1.99E-01 | 7 | -162.507 | 339.015 | 1.287 | 0.21 |
| -0.616 | 1.40E-09 | NA | + | NA | NA | 2.08E-01 | 7 | -162.543 | 339.086 | 1.359 | 0.203 |
| -0.628 | NA | NA | + | -8.02E-04 | NA | 2.01E-01 | 7 | -162.62 | 339.239 | 1.512 | 0.188 |
| -0.521 | NA | NA | + | NA | NA | 1.80E-01 | 6 | -166.672 | 345.344 | 0 | 0.458 |
| -0.555 | 1.14E-09 | NA | + | NA | NA | 1.86E-01 | 7 | -166.474 | 346.948 | 1.604 | 0.205 |
| -0.512 | NA | NA | + | NA | -4.12E-03 | 1.80E-01 | 7 | -166.671 | 347.341 | 1.997 | 0.169 |
| -0.522 | NA | NA | + | -1.44E-05 | NA | 1.80E-01 | 7 | -166.672 | 347.344 | 2 | 0.168 |
| -0.505 | NA | NA | + | NA | NA | 1.78E-01 | 6 | -155.365 | 322.731 | 0 | 0.402 |
| -0.384 | NA | NA | + | NA | -5.39E-02 | 1.78E-01 | 7 | -155.043 | 324.086 | 1.355 | 0.204 |
| -0.546 | 1.41E-09 | NA | + | NA | NA | 1.84E-01 | 7 | -155.06 | 324.12 | 1.389 | 0.201 |
| -0.571 | NA | NA | + | -8.51E-04 | NA | 1.82E-01 | 7 | -155.103 | 324.207 | 1.476 | 0.192 |
| -0.535 | NA | NA | + | NA | NA | 1.87E-01 | 6 | -152.632 | 317.265 | 0 | 0.326 |
| -0.621 | NA | NA | + | -1.18E-03 | NA | 1.92E-01 | 7 | -152.111 | 318.222 | 0.958 | 0.202 |
| -0.41 | NA | NA | + | NA | -5.74E-02 | 1.88E-01 | 7 | -152.255 | 318.51 | 1.246 | 0.175 |
| -0.575 | 1.15E-09 | NA | + | NA | NA | 1.96E-01 | 7 | -152.306 | 318.612 | 1.348 | 0.166 |
| -0.481 | NA | NA | + | -1.40E-03 | -7.20E-02 | 1.94E-01 | 8 | -151.533 | 319.067 | 1.802 | 0.132 |
| -0.496 | NA | NA | + | NA | NA | 1.73E-01 | 6 | -162.117 | 336.234 | 0 | 0.441 |
| -0.533 | 1.36E-09 | NA | + | NA | NA | 1.79E-01 | 7 | -161.894 | 337.789 | 1.555 | 0.203 |
| -0.541 | NA | NA | + | -5.50E-04 | NA | 1.76E-01 | 7 | -162.021 | 338.042 | 1.808 | 0.179 |
| -0.43 | NA | NA | + | NA | -3.01E-02 | 1.74E-01 | 7 | -162.025 | 338.049 | 1.815 | 0.178 |
| -0.468 | NA | NA | + | -2.05E-03 | -1.26E-01 | 2.22E-01 | 8 | -142.252 | 300.503 | 0 | 0.655 |
| -0.516 | 1.28E-09 | NA | + | -2.03E-03 | -1.22E-01 | 2.29E-01 | 9 | -141.892 | 301.784 | 1.281 | 0.345 |
| -0.621 | NA | NA | + | NA | NA | 2.06E-01 | 6 | -159.55 | 331.1 | 0 | 0.269 |
| -0.72 | NA | NA | + | -1.36E-03 | NA | 2.11E-01 | 7 | -158.878 | 331.756 | 0.656 | 0.194 |
| -0.668 | 1.51E-09 | NA | + | NA | NA | 2.15E-01 | 7 | -159.143 | 332.286 | 1.187 | 0.148 |
| -0.486 | NA | NA | + | NA | -5.92E-02 | 2.07E-01 | 7 | -159.147 | 332.295 | 1.195 | 0.148 |
| -0.567 | NA | NA | + | -1.59E-03 | -7.46E-02 | 2.13E-01 | 8 | -158.252 | 332.504 | 1.404 | 0.133 |
| -0.768 | 1.53E-09 | NA | + | -1.37E-03 | NA | 2.20E-01 | 8 | -158.456 | 332.913 | 1.813 | 0.109 |
| -0.406 | NA | NA | + | NA | NA | 1.55E-01 | 6 | -154.092 | 320.183 | 0 | 0.389 |
| -0.436 | 9.66E-10 | NA | + | NA | NA | 1.60E-01 | 7 | -153.907 | 321.815 | 1.632 | 0.172 |
| -0.345 | NA | NA | NA | NA | NA | 1.94E-01 | 3 | -158.058 | 322.116 | 1.933 | 0.148 |
| -0.37 | NA | NA | + | NA | -1.62E-02 | 1.55E-01 | 7 | -154.066 | 322.132 | 1.949 | 0.147 |
| -0.414 | NA | NA | + | -9.65E-05 | NA | 1.56E-01 | 7 | -154.088 | 322.177 | 1.994 | 0.144 |
| -0.46 | NA | NA | + | NA | NA | 1.68E-01 | 6 | -150.375 | 312.75 | 0 | 0.427 |
| -0.497 | 1.18E-09 | NA | + | NA | NA | 1.75E-01 | 7 | -150.1 | 314.2 | I.45 | 0.207 |
| -0.522 | NA | NA | + | -7.87E-04 | NA | 1.72E-01 | 7 | -150.163 | 314.326 | 1.576 | 0.194 |
| -0.396 | NA | NA | + | NA | -2.92E-02 | 1.69E-01 | 7 | -150.283 | 314.566 | 1.816 | 0.172 |
| -0.611 | NA | NA | + | -1.88E-03 | -1.00E-01 | 2.47E-01 | 8 | -157.069 | 330.138 | 0 | 0.217 |
| -0.817 | NA | NA | + | -1.58E-03 | NA | 2.49E-01 | 7 | -158.29 | 330.58 | 0.441 | 0.174 |
| -0.723 | NA | NA | + | NA | NA | 2.47E-01 | 6 | -159.399 | 330.798 | 0.66 | 0.156 |
| -0.545 | NA | NA | + | NA | -7.94E-02 | 2.45E-01 | 7 | -158.617 | 331.233 | 1.095 | 0.125 |
| -0.667 | 1.49E-09 | NA | + | -1.88E-03 | -9.48E-02 | 2.55E-01 | 9 | -156.649 | 331.297 | 1.159 | 0.121 |
| -0.868 | 1.71E-09 | NA | + | -1.60E-03 | NA | 2.58E-01 | 8 | -157.743 | 331.486 | 1.348 | 0.111 |
| -0.771 | 1.67E-09 | NA | + | NA | NA | 2.55E-01 | 7 | -158.887 | 331.773 | 1.635 | 0.096 |
| -0.382 | NA | NA | + | NA | NA | 1.47E-01 | 6 | -146.678 | 305.355 | 0 | 0.332 |
| -0.413 | 7.64E-10 | NA | + | NA | NA | 1.54E-01 | 7 | -146.429 | 306.858 | 1.502 | 0.157 |
| -0.302 | NA | NA | NA | NA | NA | 1.79E-01 | 3 | -150.575 | 307.15 | 1.795 | 0.135 |
| -0.339 | NA | NA | + | NA | -2.01E-02 | 1.48E-01 | 7 | -146.635 | 307.269 | 1.914 | 0.128 |
| -0.406 | NA | NA | + | -2.96E-04 | NA | 1.48E-01 | 7 | -146.648 | 307.297 | 1.941 | 0.126 |
| -0.233 | NA | NA | NA | 1.19E-03 | NA | 1.60E-01 | 4 | -149.674 | 307.349 | 1.993 | 0.123 |
| -0.535 | NA | NA | + | NA | NA | 1.82E-01 | 6 | -152.617 | 317.235 | 0 | 0.317 |
| -0.638 | NA | NA | + | -1.28E-03 | NA | 1.89E-01 | 7 | -152.009 | 318.018 | 0.784 | 0.214 |
| -0.405 | NA | NA | + | NA | -5.81E-02 | 1.83E-01 | 7 | -152.232 | 318.464 | 1.229 | 0.171 |
| -0.573 | 1.51E-09 | NA | + | NA | NA | 1.88E-01 | 7 | -152.335 | 318.671 | 1.436 | 0.154 |
| -0.492 | NA | NA | + | -1.51E-03 | -7.34E-02 | 1.91E-01 | 8 | -151.408 | 318.815 | 1.581 | 0.144 |
| -0.47 | NA | NA | + | NA | NA | 1.67E-01 | 6 | -152.887 | 317.774 | 0 | 0.443 |
| -0.505 | 1.37E-09 | NA | + | NA | NA | 1.71E-01 | 7 | -152.675 | 319.35 | 1.576 | 0.201 |
| -0.517 | NA | NA | + | -5.72E-04 | NA | 1.70E-01 | 7 | -152.78 | 319.56 | 1.785 | 0.181 |
| -0.415 | NA | NA | + | NA | -2.50E-02 | 1.68E-01 | 7 | -152.822 | 319.643 | 1.869 | 0.174 |
| -0.488 | NA | NA | + | NA | -9.66E-02 | 2.31E-01 | 7 | -161.609 | 337.217 | 0 | 0.181 |
| -0.545 | NA | NA | + | -1.52E-03 | -1.12E-01 | 2.33E-01 | 8 | -160.634 | 337.268 | 0.051 | 0.176 |
| -0.71 | NA | NA | + | NA | NA | 2.33E-01 | 6 | -162.759 | 337.518 | 0.3 | 0.156 |
| -0.782 | NA | NA | + | -1.21E-03 | NA | 2.35E-01 | 7 | -162.141 | 338.282 | 1.064 | 0.106 |
| -0.766 | 2.06E-09 | NA | + | NA | NA | 2.42E-01 | 7 | -162.154 | 338.309 | 1.091 | 0.105 |
| -0.552 | 1.77E-09 | NA | + | NA | -8.99E-02 | 2.39E-01 | 8 | -161.163 | 338.326 | 1.109 | 0.104 |
| -0.607 | 1.72E-09 | NA | + | -1.51E-03 | -1.05E-01 | 2.41E-01 | 9 | -160.205 | 338.409 | 1.192 | 0.1 |
| -0.839 | 2.07E-09 | NA | + | -1.21E-03 | NA | 2.44E-01 | 8 | -161.529 | 339.057 | I.84 | 0.072 |
| -0.518 | NA | NA | + | NA | NA | 1.87E-01 | 6 | -150.988 | 313.976 | 0 | 0.406 |
| -0.557 | 1.47E-09 | NA | + | NA | NA | 1.93E-01 | 7 | -150.665 | 315.331 | 1.354 | 0.206 |
| -0.409 | NA | NA | + | NA | -5.15E-02 | 1.90E-01 | 7 | -150.687 | 315.375 | 1.398 | 0.202 |
| -0.578 | NA | NA | + | -7.79E-04 | NA | 1.91E-01 | 7 | -150.764 | 315.527 | 1.551 | 0.187 |
| -0.507 | NA | NA | + | NA | NA | 1.80E-01 | 6 | -154.082 | 320.163 | 0 | 0.413 |
| -0.589 | NA | NA | + | -1.02E-03 | NA | 1.86E-01 | 7 | -153.711 | 321.421 | 1.258 | 0.22 |
| -0.539 | 1.11E-09 | NA | + | NA | NA | 1.86E-01 | 7 | -153.859 | 321.718 | 1.555 | 0.19 |
| -0.425 | NA | NA | + | NA | -3.88E-02 | 1.82E-01 | 7 | -153.926 | 321.852 | 1.689 | 0.177 |
| -0.514 | NA | NA | + | NA | NA | 1.84E-01 | 6 | -161.864 | 335.728 | 0 | 0.441 |
| -0.549 | 1.16E-09 | NA | + | NA | NA | 1.90E-01 | 7 | -161.601 | 337.202 | 1.473 | 0.211 |
| -0.449 | NA | NA | + | NA | -3.04E-02 | 1.85E-01 | 7 | -161.77 | 337.54 | 1.812 | 0.178 |
| -0.543 | NA | NA | + | -3.96E-04 | NA | 1.85E-01 | 7 | -161.813 | 337.626 | 1.897 | 0.171 |
| -0.502 | NA | NA | + | NA | NA | 1.76E-01 | 6 | -153.514 | 319.027 | 0 | 0.408 |
| -0.577 | NA | NA | + | -9.27E-04 | NA | 1.81E-01 | 7 | -153.225 | 320.45 | 1.422 | 0.2 |
| -0.392 | NA | NA | + | NA | -4.93E-02 | 1.77E-01 | 7 | -153.245 | 320.49 | 1.463 | 0.196 |
| -0.541 | 1.34E-09 | NA | + | NA | NA | 1.82E-01 | 7 | -153.251 | 320.502 | 1.474 | 0.195 |
| -0.528 | NA | NA | + | NA | NA | 1.84E-01 | 6 | -163.614 | 339.228 | 0 | 0.439 |
| -0.563 | 1.12E-09 | NA | + | NA | NA | 1.91E-01 | 7 | -163.386 | 340.771 | 1.543 | 0.203 |
| -0.587 | NA | NA | + | -7.66E-04 | NA | 1.87E-01 | 7 | -163.431 | 340.862 | 1.634 | 0.194 |
| -0.5 | NA | NA | + | NA | -1.25E-02 | 1.84E-01 | 7 | -163.599 | 341.199 | I.97 | 0.164 |
| -0.33 | NA | NA | + | NA | NA | 1.36E-01 | 6 | -144.26 | 300.52 | 0 | 0.214 |
| -0.193 | NA | NA | NA | 1.31E-03 | NA | 1.46E-01 | 4 | -146.362 | 300.725 | 0.205 | 0.193 |
| -0.283 | NA | NA | NA | NA | NA | 1.71E-01 | 3 | -147.387 | 300.775 | 0.255 | 0.188 |
| -0.353 | 8.10E-10 | NA | + | NA | NA | 1.40E-01 | 7 | -144.141 | 302.282 | 1.762 | 0.089 |
| -0.216 | 8.06E-10 | NA | NA | 1.30E-03 | NA | 1.50E-01 | 5 | -146.248 | 302.496 | 1.976 | 0.08 |
| -0.308 | 8.71E-10 | NA | NA | NA | NA | 1.75E-01 | 4 | -147.255 | 302.51 | 1.991 | 0.079 |
| -0.32 | NA | NA | + | 1.18E-04 | NA | 1.35E-01 | 7 | -144.256 | 302.511 | 1.991 | 0.079 |
| -0.339 | NA | NA | + | NA | 3.87E-03 | 1.36E-01 | 7 | -144.258 | 302.517 | 1.997 | 0.079 |
| -0.365 | NA | NA | + | NA | NA | 1.43E-01 | 6 | -142.832 | 297.664 | 0 | 0.388 |
| -0.39 | 8.55E-10 | NA | + | NA | NA | 1.48E-01 | 7 | -142.687 | 299.374 | I.71 | 0.165 |
| -0.409 | NA | NA | + | -5.00E-04 | NA | 1.47E-01 | 7 | -142.747 | 299.495 | 1.831 | 0.155 |
| -0.33 | NA | NA | + | NA | -1.62E-02 | 1.44E-01 | 7 | -142.804 | 299.608 | 1.945 | 0.147 |
| -0.303 | NA | NA | NA | NA | NA | 1.81E-01 | 3 | -146.812 | 299.625 | 1.961 | 0.145 |
| -0.433 | NA | NA | + | NA | NA | 1.57E-01 | 6 | -152.421 | 316.842 | 0 | 0.454 |
| -0.465 | 1.08E-09 | NA | + | NA | NA | 1.62E-01 | 7 | -152.24 | 318.479 | 1.637 | 0.2 |
| -0.468 | NA | NA | + | -4.34E-04 | NA | 1.59E-01 | 7 | -152.361 | 318.722 | I.88 | 0.177 |
| -0.414 | NA | NA | + | NA | -8.86E-03 | 1.57E-01 | 7 | -152.413 | 318.827 | 1.984 | 0.168 |
| -0.425 | NA | NA | + | NA | NA | 1.58E-01 | 6 | -137.925 | 287.849 | 0 | 0.386 |
| -0.509 | NA | NA | + | -1.02E-03 | NA | 1.64E-01 | 7 | -137.511 | 289.022 | 1.173 | 0.215 |
| -0.307 | NA | NA | + | NA | -5.50E-02 | 1.60E-01 | 7 | -137.544 | 289.088 | 1.239 | 0.208 |
| -0.459 | 1.20E-09 | NA | + | NA | NA | 1.63E-01 | 7 | -137.631 | 289.263 | 1.413 | 0.191 |
| -0.498 | NA | NA | + | NA | NA | 1.72E-01 | 6 | -150.805 | 313.61 | 0 | 0.401 |
| -0.582 | NA | NA | + | -1.04E-03 | NA | 1.78E-01 | 7 | -150.416 | 314.831 | 1.221 | 0.218 |
| -0.388 | NA | NA | + | NA | -4.91E-02 | 1.73E-01 | 7 | -150.549 | 315.098 | 1.488 | 0.191 |
| -0.533 | 1.22E-09 | NA | + | NA | NA | 1.78E-01 | 7 | -150.55 | 315.101 | I.49 | 0.19 |
| -0.399 | NA | NA | + | NA | NA | 1.51E-01 | 6 | -162.568 | 337.137 | 0 | 0.285 |
| -0.23 | NA | NA | NA | 1.54E-03 | NA | 1.61E-01 | 4 | -164.888 | 337.776 | 0.64 | 0.207 |
| -0.323 | NA | NA | NA | NA | NA | 1.88E-01 | 3 | -166.099 | 338.199 | 1.062 | 0.168 |
| -0.429 | 1.04E-09 | NA | + | NA | NA | 1.56E-01 | 7 | -162.412 | 338.824 | 1.688 | 0.123 |
| -0.364 | NA | NA | + | 4.25E-04 | NA | 1.49E-01 | 7 | -162.516 | 339.031 | 1.895 | 0.111 |
| -0.433 | NA | NA | + | NA | 1.53E-02 | 1.51E-01 | 7 | -162.548 | 339.096 | 1.959 | 0.107 |
| -0.378 | NA | NA | + | NA | NA | 1.47E-01 | 6 | -148.327 | 308.655 | 0 | 0.284 |
| -0.32 | NA | NA | NA | NA | NA | 1.86E-01 | 3 | -151.675 | 309.35 | 0.695 | 0.2 |
| -0.236 | NA | NA | NA | 1.23E-03 | NA | 1.62E-01 | 4 | -150.767 | 309.535 | 0.88 | 0.183 |
| -0.405 | 9.73E-10 | NA | + | NA | NA | 1.52E-01 | 7 | -148.165 | 310.33 | 1.675 | 0.123 |
| -0.396 | NA | NA | + | -2.03E-04 | NA | 1.48E-01 | 7 | -148.315 | 310.63 | 1.975 | 0.106 |
| -0.383 | NA | NA | + | NA | 2.47E-03 | 1.47E-01 | 7 | -148.327 | 310.654 | 1.999 | 0.104 |
| -0.34 | NA | NA | + | NA | NA | 1.35E-01 | 6 | -139.859 | 291.717 | 0 | 0.259 |
| -0.189 | NA | NA | NA | 1.29E-03 | NA | 1.45E-01 | 4 | -142.013 | 292.026 | 0.309 | 0.222 |
| -0.268 | NA | NA | NA | NA | NA | 1.66E-01 | 3 | -143.021 | 292.041 | 0.324 | 0.22 |
| -0.364 | 6.89E-10 | NA | + | NA | NA | 1.40E-01 | 7 | -139.723 | 293.446 | 1.729 | 0.109 |
| -0.328 | NA | NA | + | 1.34E-04 | NA | 1.35E-01 | 7 | -139.853 | 293.706 | 1.988 | 0.096 |
| -0.346 | NA | NA | + | NA | 2.91E-03 | 1.35E-01 | 7 | -139.858 | 293.716 | 1.998 | 0.095 |
| -0.506 | NA | NA | + | NA | NA | 1.78E-01 | 6 | -154.842 | 321.685 | 0 | 0.415 |
| -0.583 | NA | NA | + | -1.03E-03 | NA | 1.81E-01 | 7 | -154.455 | 322.909 | 1.225 | 0.225 |
| -0.54 | 1.11E-09 | NA | + | NA | NA | 1.84E-01 | 7 | -154.625 | 323.249 | 1.565 | 0.19 |
| -0.434 | NA | NA | + | NA | -3.23E-02 | 1.78E-01 | 7 | -154.732 | 323.464 | I.78 | 0.17 |
| -0.497 | NA | NA | + | NA | NA | 1.75E-01 | 6 | -148.466 | 308.932 | 0 | 0.416 |
| -0.565 | NA | NA | + | -8.91E-04 | NA | 1.80E-01 | 7 | -148.18 | 310.36 | 1.428 | 0.204 |
| -0.531 | 9.76E-10 | NA | + | NA | NA | 1.82E-01 | 7 | -148.217 | 310.434 | 1.502 | 0.196 |
| -0.409 | NA | NA | + | NA | -4.18E-02 | 1.78E-01 | 7 | -148.276 | 310.551 | 1.619 | 0.185 |
| -0.622 | NA | NA | + | NA | NA | 2.08E-01 | 6 | -149.732 | 311.464 | 0 | 0.194 |
| -0.725 | NA | NA | + | -1.51E-03 | NA | 2.13E-01 | 7 | -148.778 | 311.557 | 0.093 | 0.185 |
| -0.545 | NA | NA | + | -1.80E-03 | -8.93E-02 | 2.14E-01 | 8 | -147.826 | 311.652 | 0.188 | 0.177 |
| -0.467 | NA | NA | + | NA | -6.92E-02 | 2.08E-01 | 7 | -149.147 | 312.294 | 0.83 | 0.128 |
| -0.669 | 1.57E-09 | NA | + | NA | NA | 2.16E-01 | 7 | -149.277 | 312.554 | 01.IX | 0.113 |
| -0.773 | 1.59E-09 | NA | + | -1.51E-03 | NA | 2.21E-01 | 8 | -148.312 | 312.624 | I.16 | 0.109 |
| -0.597 | 1.42E-09 | NA | + | -1.79E-03 | -8.49E-02 | 2.21E-01 | 9 | -147.452 | 312.903 | 1.439 | 0.095 |
| -0.56 | NA | NA | + | NA | NA | 1.95E-01 | 6 | -160.341 | 332.681 | 0 | 0.404 |
| -0.605 | 1.66E-09 | NA | + | NA | NA | 2.02E-01 | 7 | -159.968 | 333.935 | 1.254 | 0.216 |
| -0.448 | NA | NA | + | NA | -5.10E-02 | 1.96E-01 | 7 | -160.059 | 334.118 | 1.437 | 0.197 |
| -0.617 | NA | NA | + | -7.65E-04 | NA | 1.98E-01 | 7 | -160.138 | 334.276 | 1.595 | 0.182 |
| -0.397 | NA | NA | + | NA | NA | 1.47E-01 | 6 | -156.777 | 325.554 | 0 | 0.291 |
| -0.222 | NA | NA | NA | 1.44E-03 | NA | 1.57E-01 | 4 | -159.2 | 326.399 | 0.845 | 0.191 |
| -0.309 | NA | NA | NA | NA | NA | 1.83E-01 | 3 | -160.274 | 326.548 | 0.993 | 0.177 |
| -0.425 | 9.53E-10 | NA | + | NA | NA | 1.52E-01 | 7 | -156.63 | 327.26 | 1.706 | 0.124 |
| -0.431 | NA | NA | + | NA | 1.51E-02 | 1.46E-01 | 7 | -156.757 | 327.513 | 1.959 | 0.109 |
| -0.378 | NA | NA | + | 2.34E-04 | NA | 1.46E-01 | 7 | -156.761 | 327.522 | 1.968 | 0.109 |
| -0.407 | NA | NA | + | NA | NA | 1.57E-01 | 6 | -141.477 | 294.953 | 0 | 0.416 |
| -0.491 | NA | NA | + | -1.00E-03 | NA | 1.62E-01 | 7 | -141.094 | 296.188 | 1.235 | 0.225 |
| -0.437 | 1.07E-09 | NA | + | NA | NA | 1.61E-01 | 7 | -141.274 | 296.548 | 1.595 | 0.188 |
| -0.339 | NA | NA | + | NA | -3.15E-02 | 1.58E-01 | 7 | -141.365 | 296.729 | 1.776 | 0.171 |
| -0.546 | NA | NA | + | -1.81E-03 | -1.04E-01 | 2.34E-01 | 8 | -154.825 | 325.651 | 0 | 0.196 |
| -0.648 | NA | NA | + | NA | NA | 2.26E-01 | 6 | -157.01 | 326.019 | 0.368 | 0.163 |
| -0.458 | NA | NA | + | NA | -8.32E-02 | 2.24E-01 | 7 | -156.132 | 326.264 | 0.614 | 0.144 |
| -0.757 | NA | NA | + | -1.45E-03 | NA | 2.34E-01 | 7 | -156.153 | 326.306 | 0.655 | 0.141 |
| -0.596 | 1.59E-09 | NA | + | -1.79E-03 | -9.90E-02 | 2.40E-01 | 9 | -154.489 | 326.979 | 1.328 | 0.101 |
| -0.696 | 1.88E-09 | NA | + | NA | NA | 2.33E-01 | 7 | -156.553 | 327.105 | 1.455 | 0.095 |
| -0.804 | 1.87E-09 | NA | + | -1.44E-03 | NA | 2.41E-01 | 8 | -155.695 | 327.39 | 1.739 | 0.082 |
| -0.511 | 1.66E-09 | NA | + | NA | -7.85E-02 | 2.31E-01 | 8 | -155.773 | 327.547 | 1.896 | 0.076 |
| -0.711 | NA | NA | + | NA | NA | 2.32E-01 | 6 | -164.655 | 341.31 | 0 | 0.227 |
| -0.545 | NA | NA | + | NA | -7.44E-02 | 2.33E-01 | 7 | -164.003 | 342.006 | 0.696 | 0.16 |
| -0.791 | NA | NA | + | -1.26E-03 | NA | 2.34E-01 | 7 | -164.036 | 342.073 | 0.763 | 0.155 |
| -0.61 | NA | NA | + | -1.52E-03 | -8.87E-02 | 2.36E-01 | 8 | -163.125 | 342.249 | 0.939 | 0.142 |
| -0.763 | 1.86E-09 | NA | + | NA | NA | 2.41E-01 | 7 | -164.167 | 342.334 | 1.024 | 0.136 |
| -0.845 | 1.87E-09 | NA | + | -1.27E-03 | NA | 2.43E-01 | 8 | -163.537 | 343.074 | 1.764 | 0.094 |
| -0.605 | 1.65E-09 | NA | + | NA | -6.83E-02 | 2.40E-01 | 8 | -163.62 | 343.24 | I.93 | 0.086 |
| -0.562 | NA | NA | + | NA | NA | 1.87E-01 | 6 | -159.35 | 330.699 | 0 | 0.438 |
| -0.599 | 1.12E-09 | NA | + | NA | NA | 1.94E-01 | 7 | -159.096 | 332.192 | 1.492 | 0.208 |
| -0.613 | NA | NA | + | -6.53E-04 | NA | 1.91E-01 | 7 | -159.213 | 332.427 | 1.728 | 0.184 |
| -0.508 | NA | NA | + | NA | -2.41E-02 | 1.88E-01 | 7 | -159.293 | 332.586 | 1.887 | 0.17 |
| -0.43 | NA | NA | + | NA | NA | 1.68E-01 | 6 | -150.159 | 312.318 | 0 | 0.32 |
| -0.374 | NA | NA | NA | NA | NA | 2.02E-01 | 3 | -153.893 | 313.786 | 1.468 | 0.154 |
| -0.463 | 1.22E-09 | NA | + | NA | NA | 1.73E-01 | 7 | -149.933 | 313.866 | 1.548 | 0.148 |
| -0.299 | NA | NA | NA | 1.22E-03 | NA | 1.83E-01 | 4 | -153.015 | 314.031 | 1.713 | 0.136 |
| -0.397 | NA | NA | + | NA | -1.60E-02 | 1.69E-01 | 7 | -150.132 | 314.264 | 1.946 | 0.121 |
| -0.451 | NA | NA | + | -2.70E-04 | NA | 1.69E-01 | 7 | -150.135 | 314.27 | 1.952 | 0.121 |
| -0.579 | NA | NA | + | -2.23E-03 | -1.19E-01 | 2.36E-01 | 8 | -156.695 | 329.391 | 0 | 0.505 |
| -0.629 | 1.34E-09 | NA | + | -2.23E-03 | -1.15E-01 | 2.43E-01 | 9 | -156.319 | 330.637 | 1.246 | 0.271 |
| -0.82 | NA | NA | + | -1.86E-03 | NA | 2.34E-01 | 7 | -158.506 | 331.013 | 1.622 | 0.224 |
| -0.351 | NA | NA | + | NA | NA | 1.45E-01 | 6 | -133.593 | 279.185 | 0 | 0.363 |
| -0.303 | NA | NA | NA | NA | NA | 1.78E-01 | 3 | -137.249 | 280.499 | 1.313 | 0.188 |
| -0.405 | NA | NA | + | -6.03E-04 | NA | 1.49E-01 | 7 | -133.456 | 280.912 | 1.726 | 0.153 |
| -0.372 | 7.50E-10 | NA | + | NA | NA | 1.49E-01 | 7 | -133.469 | 280.938 | 1.752 | 0.151 |
| -0.297 | NA | NA | + | NA | -2.57E-02 | 1.47E-01 | 7 | -133.515 | 281.03 | 1.844 | 0.144 |
| -0.673 | NA | NA | + | NA | NA | 2.17E-01 | 6 | -164.88 | 341.761 | 0 | 0.384 |
| -0.752 | NA | NA | + | -1.18E-03 | NA | 2.20E-01 | 7 | -164.359 | 342.718 | 0.958 | 0.238 |
| -0.719 | 1.52E-09 | NA | + | NA | NA | 2.25E-01 | 7 | -164.535 | 343.07 | I.31 | 0.2 |
| -0.563 | NA | NA | + | NA | -4.83E-02 | 2.17E-01 | 7 | -164.648 | 343.296 | 1.536 | 0.178 |
| -0.643 | NA | NA | + | NA | NA | 2.20E-01 | 6 | -153.152 | 318.305 | 0 | 0.21 |
| -0.745 | NA | NA | + | -1.44E-03 | NA | 2.26E-01 | 7 | -152.303 | 318.605 | 0.301 | 0.181 |
| -0.569 | NA | NA | + | -1.74E-03 | -8.86E-02 | 2.28E-01 | 8 | -151.354 | 318.707 | 0.403 | 0.172 |
| -0.489 | NA | NA | + | NA | -6.96E-02 | 2.20E-01 | 7 | -152.553 | 319.106 | 0.801 | 0.141 |
| -0.687 | 1.40E-09 | NA | + | NA | NA | 2.28E-01 | 7 | -152.776 | 319.553 | 1.248 | 0.112 |
| -0.79 | 1.43E-09 | NA | + | -1.46E-03 | NA | 2.35E-01 | 8 | -151.905 | 319.81 | 1.505 | 0.099 |
| -0.617 | 1.26E-09 | NA | + | -1.74E-03 | -8.45E-02 | 2.35E-01 | 9 | -151.043 | 320.086 | 1.782 | 0.086 |
| -0.544 | NA | NA | + | NA | NA | 1.92E-01 | 6 | -156.87 | 325.739 | 0 | 0.409 |
| -0.423 | NA | NA | + | NA | -5.40E-02 | 1.92E-01 | 7 | -156.551 | 327.101 | 1.362 | 0.207 |
| -0.583 | 1.25E-09 | NA | + | NA | NA | 1.99E-01 | 7 | -156.569 | 327.138 | 1.399 | 0.203 |
| -0.595 | NA | NA | + | -7.10E-04 | NA | 1.95E-01 | 7 | -156.681 | 327.362 | 1.623 | 0.182 |
| -0.727 | NA | NA | + | NA | NA | 2.34E-01 | 6 | -166.115 | 344.23 | 0 | 0.263 |
| -0.549 | NA | NA | + | NA | -7.45E-02 | 2.32E-01 | 7 | -165.486 | 344.972 | 0.742 | 0.181 |
| -0.799 | NA | NA | + | -1.10E-03 | NA | 2.37E-01 | 7 | -165.624 | 345.249 | 1.019 | 0.158 |
| -0.782 | 1.99E-09 | NA | + | NA | NA | 2.43E-01 | 7 | -165.628 | 345.256 | 1.026 | 0.157 |
| -0.603 | NA | NA | + | -1.39E-03 | -9.04E-02 | 2.35E-01 | 8 | -164.725 | 345.451 | 1.221 | 0.143 |
| -0.613 | 1.77E-09 | NA | + | NA | -6.85E-02 | 2.40E-01 | 8 | -165.099 | 346.198 | 1.968 | 0.098 |
| -0.461 | NA | NA | + | NA | NA | 1.66E-01 | 6 | -161.758 | 335.517 | 0 | 0.45 |
| -0.496 | 1.22E-09 | NA | + | NA | NA | 1.71E-01 | 7 | -161.556 | 337.112 | 1.595 | 0.203 |
| -0.399 | NA | NA | + | NA | -2.78E-02 | 1.66E-01 | 7 | -161.687 | 337.374 | 1.857 | 0.178 |
| -0.483 | NA | NA | + | -2.68E-04 | NA | 1.67E-01 | 7 | -161.736 | 337.472 | 1.955 | 0.169 |
| -0.552 | NA | NA | + | NA | NA | 1.86E-01 | 6 | -162.52 | 337.039 | 0 | 0.427 |
| -0.617 | NA | NA | + | -9.25E-04 | NA | 1.89E-01 | 7 | -162.235 | 338.469 | I.43 | 0.209 |
| -0.591 | 1.38E-09 | NA | + | NA | NA | 1.93E-01 | 7 | -162.257 | 338.514 | 1.475 | 0.204 |
| -0.518 | NA | NA | + | NA | -1.51E-02 | 1.86E-01 | 7 | -162.498 | 338.996 | 1.957 | 0.16 |
| -0.364 | NA | NA | + | NA | NA | 1.44E-01 | 6 | -142.444 | 296.889 | 0 | 0.332 |
| -0.291 | NA | NA | NA | NA | NA | 1.76E-01 | 3 | -146.226 | 298.452 | 1.564 | 0.152 |
| -0.387 | 7.66E-10 | NA | + | NA | NA | 1.48E-01 | 7 | -142.31 | 298.62 | 1.732 | 0.14 |
| -0.39 | NA | NA | + | -3.12E-04 | NA | 1.46E-01 | 7 | -142.411 | 298.822 | 1.933 | 0.126 |
| -0.223 | NA | NA | NA | 1.12E-03 | NA | 1.57E-01 | 4 | -145.422 | 298.844 | 1.956 | 0.125 |
| -0.34 | NA | NA | + | NA | -1.12E-02 | 1.45E-01 | 7 | -142.431 | 298.863 | 1.974 | 0.124 |
| -0.376 | NA | NA | + | NA | NA | 1.44E-01 | 6 | -150.657 | 313.314 | 0 | 0.29 |
| -0.203 | NA | NA | NA | 1.41E-03 | NA | 1.51E-01 | 4 | -153.026 | 314.052 | 0.738 | 0.201 |
| -0.294 | NA | NA | NA | NA | NA | 1.78E-01 | 3 | -154.197 | 314.394 | 01.VIII | 0.169 |
| -0.405 | 9.92E-10 | NA | + | NA | NA | 1.49E-01 | 7 | -150.496 | 314.992 | 1.678 | 0.125 |
| -0.358 | NA | NA | + | 2.09E-04 | NA | 1.43E-01 | 7 | -150.643 | 315.287 | 1.973 | 0.108 |
| -0.384 | NA | NA | + | NA | 3.54E-03 | 1.44E-01 | 7 | -150.656 | 315.311 | 1.998 | 0.107 |
| -0.51 | NA | NA | + | NA | NA | 1.79E-01 | 6 | -152.66 | 317.32 | 0 | 0.426 |
| -0.547 | 1.14E-09 | NA | + | NA | NA | 1.86E-01 | 7 | -152.386 | 318.772 | 1.453 | 0.206 |
| -0.571 | NA | NA | + | -7.85E-04 | NA | 1.83E-01 | 7 | -152.446 | 318.891 | 1.572 | 0.194 |
| -0.443 | NA | NA | + | NA | -3.07E-02 | 1.80E-01 | 7 | -152.561 | 319.122 | 1.803 | 0.173 |
| -0.512 | NA | NA | + | NA | NA | 1.79E-01 | 6 | -144.937 | 301.875 | 0 | 0.327 |
| -0.606 | NA | NA | + | -1.23E-03 | NA | 1.84E-01 | 7 | -144.354 | 302.708 | 0.833 | 0.216 |
| -0.551 | 1.51E-09 | NA | + | NA | NA | 1.85E-01 | 7 | -144.619 | 303.237 | 1.362 | 0.166 |
| -0.403 | NA | NA | + | NA | -5.07E-02 | 1.81E-01 | 7 | -144.637 | 303.275 | 01.IV | 0.162 |
| -0.481 | NA | NA | + | -1.43E-03 | -6.54E-02 | 1.88E-01 | 8 | -143.867 | 303.735 | I.86 | 0.129 |
| -0.599 | NA | NA | + | NA | NA | 2.11E-01 | 6 | -160.554 | 333.109 | 0 | 0.317 |
| -0.451 | NA | NA | + | NA | -6.82E-02 | 2.12E-01 | 7 | -159.986 | 333.972 | 0.863 | 0.206 |
| -0.643 | 1.64E-09 | NA | + | NA | NA | 2.18E-01 | 7 | -160.165 | 334.33 | 1.221 | 0.172 |
| -0.669 | NA | NA | + | -9.77E-04 | NA | 2.15E-01 | 7 | -160.183 | 334.366 | 1.257 | 0.169 |
| -0.512 | NA | NA | + | -1.23E-03 | -8.07E-02 | 2.17E-01 | 8 | -159.408 | 334.816 | 1.707 | 0.135 |
| -0.413 | NA | NA | + | NA | NA | 1.57E-01 | 6 | -142.305 | 296.609 | 0 | 0.432 |
| -0.442 | 1.12E-09 | NA | + | NA | NA | 1.61E-01 | 7 | -142.102 | 298.205 | 1.595 | 0.195 |
| -0.472 | NA | NA | + | -6.84E-04 | NA | 1.61E-01 | 7 | -142.139 | 298.278 | 1.669 | 0.188 |
| -0.335 | NA | NA | + | NA | -3.69E-02 | 1.59E-01 | 7 | -142.148 | 298.295 | 1.686 | 0.186 |
| -0.604 | NA | NA | + | NA | NA | 2.04E-01 | 6 | -161.007 | 334.014 | 0 | 0.26 |
| -0.698 | NA | NA | + | -1.32E-03 | NA | 2.09E-01 | 7 | -160.366 | 334.732 | 0.718 | 0.182 |
| -0.448 | NA | NA | + | NA | -7.02E-02 | 2.05E-01 | 7 | -160.46 | 334.92 | 0.906 | 0.165 |
| -0.525 | NA | NA | + | -1.58E-03 | -8.57E-02 | 2.11E-01 | 8 | -159.566 | 335.131 | 1.117 | 0.149 |
| -0.653 | 1.75E-09 | NA | + | NA | NA | 2.12E-01 | 7 | -160.598 | 335.196 | 1.182 | 0.144 |
| -0.747 | 1.75E-09 | NA | + | -1.32E-03 | NA | 2.17E-01 | 8 | -159.954 | 335.908 | 1.895 | 0.101 |
| -0.403 | NA | NA | + | NA | NA | 1.53E-01 | 6 | -156.496 | 324.992 | 0 | 0.298 |
| -0.335 | NA | NA | NA | NA | NA | 1.91E-01 | 3 | -159.972 | 325.944 | 0.951 | 0.185 |
| -0.252 | NA | NA | NA | 1.32E-03 | NA | 1.68E-01 | 4 | -159.069 | 326.138 | 1.145 | 0.168 |
| -0.429 | 9.90E-10 | NA | + | NA | NA | 1.57E-01 | 7 | -156.382 | 326.763 | 1.771 | 0.123 |
| -0.459 | NA | NA | + | NA | 2.66E-02 | 1.52E-01 | 7 | -156.434 | 326.869 | 1.876 | 0.117 |
| -0.4 | NA | NA | + | 2.70E-05 | NA | 1.53E-01 | 7 | -156.496 | 326.992 | 2 | 0.11 |
| -0.402 | NA | NA | + | NA | NA | 1.57E-01 | 6 | -150.333 | 312.666 | 0 | 0.316 |
| -0.336 | NA | NA | NA | NA | NA | 1.95E-01 | 3 | -153.934 | 313.869 | 1.202 | 0.173 |
| -0.434 | 1.25E-09 | NA | + | NA | NA | 1.61E-01 | 7 | -150.144 | 314.288 | 1.622 | 0.14 |
| -0.262 | NA | NA | NA | 1.19E-03 | NA | 1.74E-01 | 4 | -153.164 | 314.329 | 1.662 | 0.138 |
| -0.415 | NA | NA | + | -1.54E-04 | NA | 1.58E-01 | 7 | -150.326 | 314.652 | 1.985 | 0.117 |
| -0.407 | NA | NA | + | NA | 2.29E-03 | 1.57E-01 | 7 | -150.333 | 314.665 | 1.999 | 0.116 |
| -0.45 | NA | NA | + | NA | NA | 1.65E-01 | 6 | -143.998 | 299.996 | 0 | 0.431 |
| -0.518 | NA | NA | + | -8.40E-04 | NA | 1.69E-01 | 7 | -143.743 | 301.485 | 1.489 | 0.205 |
| -0.481 | 9.64E-10 | NA | + | NA | NA | 1.71E-01 | 7 | -143.774 | 301.549 | 1.552 | 0.198 |
| -0.403 | NA | NA | + | NA | -2.13E-02 | 1.66E-01 | 7 | -143.948 | 301.897 | 01.IX | 0.167 |
| -0.477 | NA | NA | + | NA | NA | 1.68E-01 | 6 | -146.327 | 304.654 | 0 | 0.418 |
| -0.551 | NA | NA | + | -9.35E-04 | NA | 1.73E-01 | 7 | -145.992 | 305.983 | 1.329 | 0.215 |
| -0.51 | 1.20E-09 | NA | + | NA | NA | 1.73E-01 | 7 | -146.087 | 306.173 | 1.519 | 0.195 |
| -0.404 | NA | NA | + | NA | -3.27E-02 | 1.69E-01 | 7 | -146.213 | 306.426 | 1.772 | 0.172 |
| -0.436 | NA | NA | + | NA | NA | 1.57E-01 | 6 | -159.716 | 331.431 | 0 | 0.392 |
| -0.465 | 1.07E-09 | NA | + | NA | NA | 1.61E-01 | 7 | -159.565 | 333.13 | 1.698 | 0.168 |
| -0.27 | NA | NA | NA | 1.38E-03 | NA | 1.72E-01 | 4 | -162.673 | 333.345 | 1.914 | 0.151 |
| -0.454 | NA | NA | + | NA | 7.89E-03 | 1.56E-01 | 7 | -159.71 | 333.42 | 1.989 | 0.145 |
| -0.44 | NA | NA | + | -5.01E-05 | NA | 1.57E-01 | 7 | -159.715 | 333.43 | 1.998 | 0.144 |
| -0.458 | NA | NA | + | NA | NA | 1.62E-01 | 6 | -165.359 | 342.718 | 0 | 0.324 |
| -0.276 | NA | NA | NA | 1.59E-03 | NA | 1.76E-01 | 4 | -168.035 | 344.07 | 1.352 | 0.165 |
| -0.488 | 1.12E-09 | NA | + | NA | NA | 1.67E-01 | 7 | -165.227 | 344.455 | 1.737 | 0.136 |
| -0.377 | NA | NA | NA | NA | NA | 2.05E-01 | 3 | -169.231 | 344.462 | 1.744 | 0.135 |
| -0.44 | NA | NA | + | 2.13E-04 | NA | 1.61E-01 | 7 | -165.346 | 344.692 | 1.974 | 0.121 |
| -0.449 | NA | NA | + | NA | -3.97E-03 | 1.63E-01 | 7 | -165.358 | 344.715 | 1.997 | 0.119 |
| -0.392 | NA | NA | + | NA | NA | 1.52E-01 | 6 | -147.31 | 306.62 | 0 | 0.308 |
| -0.331 | NA | NA | NA | NA | NA | 1.89E-01 | 3 | -150.907 | 307.813 | 1.194 | 0.17 |
| -0.252 | NA | NA | NA | 1.26E-03 | NA | 1.66E-01 | 4 | -149.987 | 307.974 | 1.354 | 0.157 |
| -0.422 | 9.61E-10 | NA | + | NA | NA | 1.57E-01 | 7 | -147.128 | 308.257 | 1.637 | 0.136 |
| -0.36 | NA | NA | + | NA | -1.53E-02 | 1.52E-01 | 7 | -147.286 | 308.571 | 1.952 | 0.116 |
| -0.392 | NA | NA | + | -2.96E-06 | NA | 1.52E-01 | 7 | -147.31 | 308.62 | 2 | 0.113 |
| -0.5 | NA | NA | + | NA | NA | 1.75E-01 | 6 | -155.423 | 322.845 | 0 | 0.427 |
| -0.536 | 1.35E-09 | NA | + | NA | NA | 1.80E-01 | 7 | -155.179 | 324.357 | 1.512 | 0.2 |
| -0.404 | NA | NA | + | NA | -4.27E-02 | 1.75E-01 | 7 | -155.227 | 324.453 | 1.608 | 0.191 |
| -0.547 | NA | NA | + | -6.36E-04 | NA | 1.77E-01 | 7 | -155.277 | 324.554 | 1.709 | 0.182 |
| -0.429 | NA | NA | + | NA | NA | 1.56E-01 | 6 | -148.739 | 309.478 | 0 | 0.439 |
| -0.464 | 1.22E-09 | NA | + | NA | NA | 1.61E-01 | 7 | -148.49 | 310.98 | 1.502 | 0.207 |
| -0.359 | NA | NA | + | NA | -3.14E-02 | 1.57E-01 | 7 | -148.632 | 311.264 | 1.786 | 0.18 |
| -0.468 | NA | NA | + | -4.63E-04 | NA | 1.58E-01 | 7 | -148.665 | 311.33 | 1.852 | 0.174 |
| -0.519 | NA | NA | + | -1.78E-03 | -1.13E-01 | 2.30E-01 | 8 | -153.718 | 323.436 | 0 | 0.306 |
| -0.449 | NA | NA | + | NA | -9.49E-02 | 2.28E-01 | 7 | -155.151 | 324.302 | 0.867 | 0.198 |
| -0.575 | 1.60E-09 | NA | + | -1.78E-03 | -1.08E-01 | 2.38E-01 | 9 | -153.292 | 324.584 | 1.149 | 0.172 |
| -0.659 | NA | NA | + | NA | NA | 2.28E-01 | 6 | -156.336 | 324.671 | 1.236 | 0.165 |
| -0.748 | NA | NA | + | -1.45E-03 | NA | 2.30E-01 | 7 | -155.379 | 324.758 | 1.322 | 0.158 |
| -0.417 | NA | NA | + | NA | NA | 1.54E-01 | 6 | -150.46 | 312.92 | 0 | 0.455 |
| -0.447 | 9.30E-10 | NA | + | NA | NA | 1.60E-01 | 7 | -150.268 | 314.536 | 1.616 | 0.203 |
| -0.447 | NA | NA | + | -3.69E-04 | NA | 1.56E-01 | 7 | -150.417 | 314.834 | 1.914 | 0.175 |
| -0.43 | NA | NA | + | NA | 6.03E-03 | 1.53E-01 | 7 | -150.457 | 314.913 | 1.993 | 0.168 |
| -0.479 | NA | NA | + | NA | NA | 1.69E-01 | 6 | -153.125 | 318.25 | 0 | 0.418 |
| -0.552 | NA | NA | + | -9.70E-04 | NA | 1.73E-01 | 7 | -152.797 | 319.593 | 1.343 | 0.214 |
| -0.512 | 1.11E-09 | NA | + | NA | NA | 1.74E-01 | 7 | -152.898 | 319.796 | 1.546 | 0.193 |
| -0.406 | NA | NA | + | NA | -3.49E-02 | 1.71E-01 | 7 | -152.998 | 319.997 | 1.746 | 0.175 |
| -0.634 | NA | NA | + | NA | NA | 2.11E-01 | 6 | -167.772 | 347.545 | 0 | 0.421 |
| -0.68 | 1.55E-09 | NA | + | NA | NA | 2.19E-01 | 7 | -167.444 | 348.887 | 1.342 | 0.215 |
| -0.524 | NA | NA | + | NA | -4.74E-02 | 2.10E-01 | 7 | -167.544 | 349.089 | 1.544 | 0.194 |
| -0.671 | NA | NA | + | -5.17E-04 | NA | 2.13E-01 | 7 | -167.68 | 349.359 | 1.815 | 0.17 |
| -0.487 | NA | NA | + | NA | NA | 1.75E-01 | 6 | -154.16 | 320.319 | 0 | 0.422 |
| -0.523 | 1.19E-09 | NA | + | NA | NA | 1.82E-01 | 7 | -153.889 | 321.778 | 1.459 | 0.203 |
| -0.386 | NA | NA | + | NA | -4.62E-02 | 1.77E-01 | 7 | -153.935 | 321.87 | 1.551 | 0.194 |
| -0.54 | NA | NA | + | -6.55E-04 | NA | 1.79E-01 | 7 | -154.011 | 322.022 | 1.702 | 0.18 |
| -0.434 | NA | NA | + | NA | NA | 1.63E-01 | 6 | -144.843 | 301.687 | 0 | 0.438 |
| -0.464 | 1.11E-09 | NA | + | NA | NA | 1.68E-01 | 7 | -144.648 | 303.296 | 1.609 | 0.196 |
| -0.49 | NA | NA | + | -6.52E-04 | NA | 1.68E-01 | 7 | -144.692 | 303.384 | 1.697 | 0.187 |
| -0.365 | NA | NA | + | NA | -3.12E-02 | 1.64E-01 | 7 | -144.735 | 303.47 | 1.783 | 0.179 |
| -0.416 | NA | NA | + | NA | NA | 1.54E-01 | 6 | -149.89 | 311.779 | 0 | 0.447 |
| -0.445 | 9.99E-10 | NA | + | NA | NA | 1.60E-01 | 7 | -149.699 | 313.397 | 1.618 | 0.199 |
| -0.347 | NA | NA | + | NA | -3.09E-02 | 1.55E-01 | 7 | -149.79 | 313.581 | 1.801 | 0.182 |
| -0.445 | NA | NA | + | -3.49E-04 | NA | 1.56E-01 | 7 | -149.849 | 313.698 | 1.919 | 0.171 |
| -0.477 | NA | NA | + | NA | NA | 1.69E-01 | 6 | -145.099 | 302.198 | 0 | 0.332 |
| -0.558 | NA | NA | + | -1.09E-03 | NA | 1.72E-01 | 7 | -144.623 | 303.245 | 1.048 | 0.197 |
| -0.358 | NA | NA | + | NA | -5.51E-02 | 1.72E-01 | 7 | -144.727 | 303.455 | 1.257 | 0.177 |
| -0.516 | 1.41E-09 | NA | + | NA | NA | 1.75E-01 | 7 | -144.769 | 303.538 | I.34 | 0.17 |
| -0.426 | NA | NA | + | -1.29E-03 | -6.75E-02 | 1.77E-01 | 8 | -144.076 | 304.153 | 1.955 | 0.125 |
| -0.555 | NA | NA | + | NA | NA | 1.95E-01 | 6 | -138.622 | 289.245 | 0 | 0.184 |
| -0.466 | NA | NA | + | -1.64E-03 | -9.26E-02 | 2.01E-01 | 8 | -136.695 | 289.39 | 0.145 | 0.171 |
| -0.653 | NA | NA | + | -1.32E-03 | NA | 2.01E-01 | 7 | -137.792 | 289.584 | 0.339 | 0.155 |
| -0.391 | NA | NA | + | NA | -7.20E-02 | 1.94E-01 | 7 | -137.938 | 289.877 | 0.632 | 0.134 |
| -0.596 | 1.18E-09 | NA | + | NA | NA | 2.04E-01 | 7 | -138.214 | 290.427 | 1.183 | 0.102 |
| -0.515 | 1.14E-09 | NA | + | -1.67E-03 | -8.93E-02 | 2.10E-01 | 9 | -136.304 | 290.607 | 1.363 | 0.093 |
| -0.699 | 1.25E-09 | NA | + | -1.36E-03 | NA | 2.11E-01 | 8 | -137.326 | 290.653 | 1.408 | 0.091 |
| -0.436 | 1.08E-09 | NA | + | NA | -6.85E-02 | 2.02E-01 | 8 | -137.593 | 291.186 | 1.942 | 0.07 |
| -0.7 | NA | NA | + | NA | NA | 2.31E-01 | 6 | -161.37 | 334.741 | 0 | 0.187 |
| -0.573 | NA | NA | + | -1.59E-03 | -1.02E-01 | 2.36E-01 | 8 | -159.497 | 334.994 | 0.253 | 0.165 |
| -0.505 | NA | NA | + | NA | -8.49E-02 | 2.30E-01 | 7 | -160.502 | 335.004 | 0.264 | 0.164 |
| -0.784 | NA | NA | + | -1.26E-03 | NA | 2.35E-01 | 7 | -160.723 | 335.445 | 0.704 | 0.132 |
| -0.748 | 1.79E-09 | NA | + | NA | NA | 2.39E-01 | 7 | -160.929 | 335.859 | 1.118 | 0.107 |
| -0.627 | 1.55E-09 | NA | + | -1.59E-03 | -9.66E-02 | 2.43E-01 | 9 | -159.163 | 336.327 | 1.586 | 0.085 |
| -0.559 | 1.54E-09 | NA | + | NA | -7.94E-02 | 2.37E-01 | 8 | -160.175 | 336.35 | 1.609 | 0.084 |
| -0.835 | 1.84E-09 | NA | + | -1.28E-03 | NA | 2.43E-01 | 8 | -160.254 | 336.508 | 1.768 | 0.077 |
| -0.576 | NA | NA | + | NA | NA | 1.94E-01 | 6 | -162.23 | 336.46 | 0 | 0.428 |
| -0.618 | 1.21E-09 | NA | + | NA | NA | 2.03E-01 | 7 | -161.931 | 337.863 | 1.403 | 0.212 |
| -0.621 | NA | NA | + | -6.57E-04 | NA | 1.96E-01 | 7 | -162.085 | 338.17 | I.71 | 0.182 |
| -0.502 | NA | NA | + | NA | -3.38E-02 | 1.95E-01 | 7 | -162.113 | 338.225 | 1.766 | 0.177 |
| -0.638 | NA | NA | + | NA | NA | 2.16E-01 | 6 | -176.911 | 365.822 | 0 | 0.401 |
| -0.687 | 1.75E-09 | NA | + | NA | NA | 2.24E-01 | 7 | -176.512 | 367.024 | 1.202 | 0.22 |
| -0.507 | NA | NA | + | NA | -5.98E-02 | 2.17E-01 | 7 | -176.532 | 367.063 | 1.241 | 0.215 |
| -0.677 | NA | NA | + | -5.67E-04 | NA | 2.18E-01 | 7 | -176.802 | 367.604 | 1.783 | 0.164 |
| -0.535 | NA | NA | + | NA | NA | 1.88E-01 | 6 | -164.072 | 340.143 | 0 | 0.444 |
| -0.571 | 1.31E-09 | NA | + | NA | NA | 1.94E-01 | 7 | -163.837 | 341.673 | I.53 | 0.206 |
| -0.47 | NA | NA | + | NA | -2.95E-02 | 1.89E-01 | 7 | -163.988 | 341.976 | 1.833 | 0.177 |
| -0.565 | NA | NA | + | -4.10E-04 | NA | 1.90E-01 | 7 | -164.016 | 342.033 | 1.889 | 0.172 |
| -0.599 | NA | NA | + | NA | NA | 2.05E-01 | 6 | -159.661 | 331.322 | 0 | 0.377 |
| -0.646 | 1.72E-09 | NA | + | NA | NA | 2.13E-01 | 7 | -159.245 | 332.49 | 1.168 | 0.21 |
| -0.462 | NA | NA | + | NA | -6.07E-02 | 2.05E-01 | 7 | -159.251 | 332.502 | I.18 | 0.209 |
| -0.675 | NA | NA | + | -1.03E-03 | NA | 2.11E-01 | 7 | -159.277 | 332.553 | 1.231 | 0.204 |
| -0.39 | NA | NA | + | NA | NA | 1.53E-01 | 6 | -152.529 | 317.058 | 0 | 0.337 |
| -0.319 | NA | NA | NA | NA | NA | 1.86E-01 | 3 | -156.367 | 318.735 | 1.677 | 0.146 |
| -0.413 | 8.81E-10 | NA | + | NA | NA | 1.56E-01 | 7 | -152.413 | 318.826 | 1.769 | 0.139 |
| -0.242 | NA | NA | NA | 1.17E-03 | NA | 1.64E-01 | 4 | -155.49 | 318.981 | 1.923 | 0.129 |
| -0.407 | NA | NA | + | -1.94E-04 | NA | 1.54E-01 | 7 | -152.516 | 319.033 | 1.975 | 0.125 |
| -0.398 | NA | NA | + | NA | 3.96E-03 | 1.53E-01 | 7 | -152.527 | 319.055 | 1.997 | 0.124 |
| -0.562 | NA | NA | + | NA | NA | 1.95E-01 | 6 | -146.884 | 305.769 | 0 | 0.25 |
| -0.677 | NA | NA | + | -1.57E-03 | NA | 2.02E-01 | 7 | -145.908 | 305.816 | 0.048 | 0.244 |
| -0.549 | NA | NA | + | -1.78E-03 | -6.80E-02 | 2.06E-01 | 8 | -145.392 | 306.784 | 1.016 | 0.15 |
| -0.459 | NA | NA | + | NA | -4.86E-02 | 1.98E-01 | 7 | -146.615 | 307.23 | 1.461 | 0.12 |
| -0.595 | 1.14E-09 | NA | + | NA | NA | 2.01E-01 | 7 | -146.631 | 307.261 | 1.493 | 0.118 |
| -0.713 | 1.18E-09 | NA | + | -1.59E-03 | NA | 2.08E-01 | 8 | -145.631 | 307.263 | 1.494 | 0.118 |
| -0.417 | NA | NA | + | NA | NA | 1.62E-01 | 6 | -138.278 | 288.557 | 0 | 0.394 |
| -0.508 | NA | NA | + | -1.09E-03 | NA | 1.70E-01 | 7 | -137.814 | 289.628 | 1.072 | 0.23 |
| -0.314 | NA | NA | + | NA | -4.75E-02 | 1.64E-01 | 7 | -138.003 | 290.006 | 1.449 | 0.191 |
| -0.449 | 9.66E-10 | NA | + | NA | NA | 1.69E-01 | 7 | -138.032 | 290.063 | 1.507 | 0.185 |
| -0.398 | NA | NA | + | NA | NA | 1.49E-01 | 6 | -149.598 | 311.195 | 0 | 0.305 |
| -0.334 | NA | NA | NA | NA | NA | 1.90E-01 | 3 | -153.184 | 312.367 | 1.172 | 0.17 |
| -0.249 | NA | NA | NA | 1.26E-03 | NA | 1.64E-01 | 4 | -152.188 | 312.376 | I.18 | 0.169 |
| -0.425 | 9.76E-10 | NA | + | NA | NA | 1.54E-01 | 7 | -149.443 | 312.885 | I.69 | 0.131 |
| -0.407 | NA | NA | + | -1.10E-04 | NA | 1.50E-01 | 7 | -149.594 | 313.187 | 1.992 | 0.113 |
| -0.403 | NA | NA | + | NA | 2.21E-03 | 1.49E-01 | 7 | -149.597 | 313.194 | 1.999 | 0.112 |
| -0.475 | NA | NA | + | NA | NA | 1.68E-01 | 6 | -143.274 | 298.549 | 0 | 0.273 |
| -0.582 | NA | NA | + | -1.39E-03 | NA | 1.73E-01 | 7 | -142.525 | 299.049 | 0.501 | 0.213 |
| -0.363 | NA | NA | + | NA | -5.12E-02 | 1.70E-01 | 7 | -142.958 | 299.915 | 1.367 | 0.138 |
| -0.51 | 1.09E-09 | NA | + | NA | NA | 1.75E-01 | 7 | -142.982 | 299.964 | 1.416 | 0.135 |
| -0.451 | NA | NA | + | -1.61E-03 | -6.75E-02 | 1.76E-01 | 8 | -141.986 | 299.973 | 1.424 | 0.134 |
| -0.62 | 1.12E-09 | NA | + | -1.41E-03 | NA | 1.80E-01 | 8 | -142.211 | 300.421 | 1.872 | 0.107 |
| -0.69 | NA | NA | + | NA | NA | 2.31E-01 | 6 | -166.012 | 344.023 | 0 | 0.386 |
| -0.739 | 1.73E-09 | NA | + | NA | NA | 2.39E-01 | 7 | -165.593 | 345.186 | 1.163 | 0.216 |
| -0.549 | NA | NA | + | NA | -6.05E-02 | 2.29E-01 | 7 | -165.609 | 345.218 | 1.194 | 0.212 |
| -0.745 | NA | NA | + | -8.37E-04 | NA | 2.34E-01 | 7 | -165.743 | 345.486 | 1.463 | 0.186 |
| -0.551 | NA | NA | + | NA | NA | 1.94E-01 | 6 | -153.183 | 318.367 | 0 | 0.407 |
| -0.634 | NA | NA | + | -1.13E-03 | NA | 1.99E-01 | 7 | -152.727 | 319.454 | 1.087 | 0.236 |
| -0.589 | 9.75E-10 | NA | + | NA | NA | 2.03E-01 | 7 | -152.889 | 319.777 | 1.411 | 0.201 |
| -0.511 | NA | NA | + | NA | -1.83E-02 | 1.94E-01 | 7 | -153.147 | 320.295 | 1.928 | 0.155 |
| -0.434 | NA | NA | + | NA | NA | 1.59E-01 | 6 | -157.071 | 326.143 | 0 | 0.459 |
| -0.463 | 1.09E-09 | NA | + | NA | NA | 1.63E-01 | 7 | -156.903 | 327.806 | 1.663 | 0.2 |
| -0.452 | NA | NA | + | -2.10E-04 | NA | 1.60E-01 | 7 | -157.058 | 328.115 | 1.973 | 0.171 |
| -0.433 | NA | NA | + | NA | -8.07E-04 | 1.59E-01 | 7 | -157.071 | 328.143 | 2 | 0.169 |
| -0.499 | NA | NA | + | NA | NA | 1.74E-01 | 6 | -149.353 | 310.705 | 0 | 0.422 |
| -0.532 | 1.03E-09 | NA | + | NA | NA | 1.81E-01 | 7 | -149.108 | 312.217 | 1.511 | 0.198 |
| -0.393 | NA | NA | + | NA | -4.68E-02 | 1.75E-01 | 7 | -149.121 | 312.242 | 1.537 | 0.195 |
| -0.555 | NA | NA | + | -7.02E-04 | NA | 1.79E-01 | 7 | -149.176 | 312.352 | 1.647 | 0.185 |
| -0.49 | NA | NA | + | NA | NA | 1.73E-01 | 6 | -152.288 | 316.576 | 0 | 0.421 |
| -0.526 | 1.27E-09 | NA | + | NA | NA | 1.80E-01 | 7 | -152.002 | 318.003 | 1.428 | 0.206 |
| -0.394 | NA | NA | + | NA | -4.34E-02 | 1.74E-01 | 7 | -152.074 | 318.148 | 1.572 | 0.192 |
| -0.54 | NA | NA | + | -6.62E-04 | NA | 1.76E-01 | 7 | -152.131 | 318.262 | 1.686 | 0.181 |
| -0.48 | NA | NA | + | NA | NA | 1.71E-01 | 6 | -156.736 | 325.473 | 0 | 0.447 |
| -0.518 | 8.84E-10 | NA | + | NA | NA | 1.81E-01 | 7 | -156.457 | 326.913 | I.44 | 0.217 |
| -0.441 | NA | NA | + | NA | -1.72E-02 | 1.72E-01 | 7 | -156.707 | 327.414 | 1.941 | 0.169 |
| -0.496 | NA | NA | + | -2.14E-04 | NA | 1.72E-01 | 7 | -156.722 | 327.443 | I.97 | 0.167 |
| -0.317 | NA | NA | + | NA | NA | 1.30E-01 | 6 | -131.002 | 274.004 | 0 | 0.296 |
| -0.254 | NA | NA | NA | NA | NA | 1.60E-01 | 3 | -134.453 | 274.906 | 0.902 | 0.188 |
| -0.185 | NA | NA | NA | 1.08E-03 | NA | 1.40E-01 | 4 | -133.56 | 275.12 | 1.116 | 0.169 |
| -0.339 | 6.45E-10 | NA | + | NA | NA | 1.34E-01 | 7 | -130.872 | 275.745 | 1.741 | 0.124 |
| -0.347 | NA | NA | + | -3.45E-04 | NA | 1.32E-01 | 7 | -130.959 | 275.919 | 1.915 | 0.113 |
| -0.3 | NA | NA | + | NA | -7.88E-03 | 1.31E-01 | 7 | -130.995 | 275.989 | 1.986 | 0.11 |
| -0.663 | NA | NA | + | NA | NA | 2.22E-01 | 6 | -172.801 | 357.602 | 0 | 0.39 |
| -0.72 | 2.23E-09 | NA | + | NA | NA | 2.29E-01 | 7 | -172.348 | 358.696 | 1.094 | 0.226 |
| -0.732 | NA | NA | + | -9.51E-04 | NA | 2.26E-01 | 7 | -172.492 | 358.984 | 1.381 | 0.195 |
| -0.547 | NA | NA | + | NA | -5.18E-02 | 2.23E-01 | 7 | -172.523 | 359.045 | 1.443 | 0.189 |
| -0.42 | NA | NA | + | NA | NA | 1.55E-01 | 6 | -144.163 | 300.326 | 0 | 0.451 |
| -0.446 | 8.05E-10 | NA | + | NA | NA | 1.61E-01 | 7 | -144 | 301.999 | 1.673 | 0.195 |
| -0.466 | NA | NA | + | -5.57E-04 | NA | 1.58E-01 | 7 | -144.054 | 302.109 | 1.783 | 0.185 |
| -0.389 | NA | NA | + | NA | -1.44E-02 | 1.56E-01 | 7 | -144.141 | 302.282 | 1.956 | 0.169 |
| -0.522 | NA | NA | + | NA | NA | 1.85E-01 | 6 | -154.092 | 320.184 | 0 | 0.412 |
| -0.563 | 1.37E-09 | NA | + | NA | NA | 1.92E-01 | 7 | -153.763 | 321.526 | 1.342 | 0.211 |
| -0.411 | NA | NA | + | NA | -4.91E-02 | 1.85E-01 | 7 | -153.831 | 321.662 | 1.478 | 0.197 |
| -0.572 | NA | NA | + | -6.85E-04 | NA | 1.88E-01 | 7 | -153.916 | 321.832 | 1.648 | 0.181 |
| -0.377 | NA | NA | + | NA | NA | 1.47E-01 | 6 | -145.58 | 303.161 | 0 | 0.296 |
| -0.301 | NA | NA | NA | NA | NA | 1.79E-01 | 3 | -148.987 | 303.974 | 0.813 | 0.197 |
| -0.23 | NA | NA | NA | 1.18E-03 | NA | 1.59E-01 | 4 | -148.161 | 304.322 | 1.161 | 0.165 |
| -0.401 | 8.80E-10 | NA | + | NA | NA | 1.51E-01 | 7 | -145.453 | 304.905 | 1.745 | 0.124 |
| -0.393 | NA | NA | + | -1.90E-04 | NA | 1.48E-01 | 7 | -145.569 | 305.137 | 1.977 | 0.11 |
| -0.378 | NA | NA | + | NA | 2.30E-04 | 1.47E-01 | 7 | -145.58 | 305.161 | 2 | 0.109 |
| -0.416 | NA | NA | + | NA | NA | 1.56E-01 | 6 | -163.363 | 338.727 | 0 | 0.272 |
| -0.249 | NA | NA | NA | 1.47E-03 | NA | 1.65E-01 | 4 | -165.627 | 339.254 | 0.527 | 0.209 |
| -0.333 | NA | NA | NA | NA | NA | 1.87E-01 | 3 | -166.709 | 339.419 | 0.692 | 0.192 |
| -0.448 | 1.24E-09 | NA | + | NA | NA | 1.60E-01 | 7 | -163.185 | 340.371 | 1.644 | 0.119 |
| -0.469 | NA | NA | + | NA | 2.53E-02 | 1.54E-01 | 7 | -163.308 | 340.617 | I.89 | 0.106 |
| -0.395 | NA | NA | + | 2.58E-04 | NA | 1.55E-01 | 7 | -163.345 | 340.689 | 1.963 | 0.102 |
| -0.482 | NA | NA | + | NA | NA | 1.74E-01 | 6 | -154.558 | 321.116 | 0 | 0.426 |
| -0.519 | 1.32E-09 | NA | + | NA | NA | 1.81E-01 | 7 | -154.268 | 322.535 | 1.419 | 0.21 |
| -0.534 | NA | NA | + | -6.72E-04 | NA | 1.78E-01 | 7 | -154.4 | 322.8 | 1.684 | 0.184 |
| -0.401 | NA | NA | + | NA | -3.60E-02 | 1.74E-01 | 7 | -154.418 | 322.837 | I.72 | 0.18 |
| -0.406 | NA | NA | + | NA | NA | 1.55E-01 | 6 | -144.67 | 301.341 | 0 | 0.45 |
| -0.432 | 9.95E-10 | NA | + | NA | NA | 1.60E-01 | 7 | -144.488 | 302.976 | 1.636 | 0.199 |
| -0.449 | NA | NA | + | -5.28E-04 | NA | 1.58E-01 | 7 | -144.57 | 303.139 | 1.798 | 0.183 |
| -0.377 | NA | NA | + | NA | -1.37E-02 | 1.56E-01 | 7 | -144.651 | 303.301 | I.96 | 0.169 |
| -0.499 | NA | NA | + | NA | NA | 1.79E-01 | 6 | -148.279 | 308.557 | 0 | 0.412 |
| -0.385 | NA | NA | + | NA | -5.12E-02 | 1.79E-01 | 7 | -147.981 | 309.962 | 1.405 | 0.204 |
| -0.535 | 1.31E-09 | NA | + | NA | NA | 1.85E-01 | 7 | -148.008 | 310.015 | 1.458 | 0.199 |
| -0.554 | NA | NA | + | -7.19E-04 | NA | 1.82E-01 | 7 | -148.074 | 310.147 | I.59 | 0.186 |
| -0.607 | NA | NA | + | NA | NA | 2.04E-01 | 6 | -157.224 | 326.449 | 0 | 0.305 |
| -0.438 | NA | NA | + | NA | -7.56E-02 | 2.05E-01 | 7 | -156.543 | 327.086 | 0.637 | 0.222 |
| -0.653 | 1.61E-09 | NA | + | NA | NA | 2.12E-01 | 7 | -156.829 | 327.659 | I.21 | 0.166 |
| -0.675 | NA | NA | + | -9.64E-04 | NA | 2.08E-01 | 7 | -156.861 | 327.721 | 1.273 | 0.161 |
| -0.497 | NA | NA | + | -1.24E-03 | -8.81E-02 | 2.10E-01 | 8 | -155.958 | 327.917 | 1.468 | 0.146 |
| -0.396 | NA | NA | + | NA | NA | 1.52E-01 | 6 | -142.312 | 296.624 | 0 | 0.451 |
| -0.423 | 9.78E-10 | NA | + | NA | NA | 1.56E-01 | 7 | -142.157 | 298.314 | I.69 | 0.194 |
| -0.44 | NA | NA | + | -5.26E-04 | NA | 1.55E-01 | 7 | -142.22 | 298.44 | 1.816 | 0.182 |
| -0.354 | NA | NA | + | NA | -2.01E-02 | 1.53E-01 | 7 | -142.267 | 298.535 | 1.911 | 0.173 |
| -0.45 | NA | NA | + | NA | NA | 1.65E-01 | 6 | -139.017 | 290.034 | 0 | 0.389 |
| -0.537 | NA | NA | + | -1.06E-03 | NA | 1.71E-01 | 7 | -138.552 | 291.104 | 01.VII | 0.228 |
| -0.337 | NA | NA | + | NA | -5.22E-02 | 1.68E-01 | 7 | -138.692 | 291.384 | I.35 | 0.198 |
| -0.48 | 9.04E-10 | NA | + | NA | NA | 1.72E-01 | 7 | -138.767 | 291.533 | 1.499 | 0.184 |
| -0.592 | NA | NA | + | NA | NA | 2.03E-01 | 6 | -161.888 | 335.776 | 0 | 0.258 |
| -0.42 | NA | NA | + | NA | -7.67E-02 | 2.04E-01 | 7 | -161.166 | 336.333 | 0.556 | 0.196 |
| -0.64 | 1.65E-09 | NA | + | NA | NA | 2.12E-01 | 7 | -161.419 | 336.837 | 1.061 | 0.152 |
| -0.663 | NA | NA | + | -1.05E-03 | NA | 2.05E-01 | 7 | -161.443 | 336.885 | 1.109 | 0.148 |
| -0.482 | NA | NA | + | -1.30E-03 | -8.87E-02 | 2.06E-01 | 8 | -160.497 | 336.993 | 1.217 | 0.141 |
| -0.475 | 1.48E-09 | NA | + | NA | -7.19E-02 | 2.12E-01 | 8 | -160.786 | 337.572 | 1.796 | 0.105 |
| -0.418 | NA | NA | + | NA | NA | 1.57E-01 | 6 | -146.367 | 304.734 | 0 | 0.451 |
| -0.451 | 1.11E-09 | NA | + | NA | NA | 1.62E-01 | 7 | -146.142 | 306.285 | 1.551 | 0.208 |
| -0.381 | NA | NA | + | NA | -1.71E-02 | 1.58E-01 | 7 | -146.337 | 306.673 | I.94 | 0.171 |
| -0.436 | NA | NA | + | -2.43E-04 | NA | 1.58E-01 | 7 | -146.346 | 306.691 | 1.958 | 0.17 |
| -0.548 | NA | NA | + | NA | NA | 1.87E-01 | 6 | -152.114 | 316.227 | 0 | 0.333 |
| -0.407 | NA | NA | + | NA | -6.45E-02 | 1.89E-01 | 7 | -151.646 | 317.291 | 1.064 | 0.196 |
| -0.625 | NA | NA | + | -1.03E-03 | NA | 1.90E-01 | 7 | -151.706 | 317.412 | 1.185 | 0.184 |
| -0.583 | 1.19E-09 | NA | + | NA | NA | 1.93E-01 | 7 | -151.856 | 317.712 | 1.485 | 0.159 |
| -0.474 | NA | NA | + | -1.23E-03 | -7.56E-02 | 1.94E-01 | 8 | -151.074 | 318.147 | I.92 | 0.128 |
| -0.489 | NA | NA | + | NA | NA | 1.76E-01 | 6 | -147.102 | 306.204 | 0 | 0.299 |
| -0.584 | NA | NA | + | -1.25E-03 | NA | 1.81E-01 | 7 | -146.483 | 306.965 | 0.762 | 0.204 |
| -0.347 | NA | NA | + | NA | -6.55E-02 | 1.77E-01 | 7 | -146.574 | 307.147 | 0.944 | 0.187 |
| -0.429 | NA | NA | + | -1.49E-03 | -8.01E-02 | 1.84E-01 | 8 | -145.707 | 307.415 | 1.211 | 0.163 |
| -0.525 | 1.15E-09 | NA | + | NA | NA | 1.82E-01 | 7 | -146.812 | 307.623 | I.42 | 0.147 |
| -0.456 | NA | NA | + | NA | NA | 1.69E-01 | 6 | -151.928 | 315.857 | 0 | 0.431 |
| -0.489 | 9.76E-10 | NA | + | NA | NA | 1.76E-01 | 7 | -151.68 | 317.36 | 1.503 | 0.203 |
| -0.352 | NA | NA | + | NA | -4.70E-02 | 1.70E-01 | 7 | -151.688 | 317.376 | I.52 | 0.202 |
| -0.481 | NA | NA | + | -3.03E-04 | NA | 1.71E-01 | 7 | -151.896 | 317.792 | 1.936 | 0.164 |
| -0.473 | NA | NA | + | NA | NA | 1.74E-01 | 6 | -147.364 | 306.728 | 0 | 0.414 |
| -0.508 | 9.80E-10 | NA | + | NA | NA | 1.82E-01 | 7 | -147.092 | 308.184 | 1.456 | 0.2 |
| -0.535 | NA | NA | + | -8.22E-04 | NA | 1.78E-01 | 7 | -147.107 | 308.214 | 1.486 | 0.197 |
| -0.378 | NA | NA | + | NA | -4.30E-02 | 1.74E-01 | 7 | -147.153 | 308.306 | 1.577 | 0.188 |
| -0.419 | NA | NA | + | NA | NA | 1.55E-01 | 6 | -148.368 | 308.736 | 0 | 0.429 |
| -0.451 | 1.01E-09 | NA | + | NA | NA | 1.61E-01 | 7 | -148.153 | 310.305 | 1.569 | 0.196 |
| -0.325 | NA | NA | + | NA | -4.27E-02 | 1.57E-01 | 7 | -148.162 | 310.324 | 1.588 | 0.194 |
| -0.475 | NA | NA | + | -6.60E-04 | NA | 1.59E-01 | 7 | -148.227 | 310.453 | 1.717 | 0.182 |
| -0.681 | NA | NA | + | NA | NA | 2.30E-01 | 6 | -167.662 | 347.324 | 0 | 0.32 |
| -0.761 | NA | NA | + | -1.18E-03 | NA | 2.34E-01 | 7 | -167.135 | 348.27 | 0.947 | 0.199 |
| -0.732 | 1.72E-09 | NA | + | NA | NA | 2.38E-01 | 7 | -167.252 | 348.503 | I.18 | 0.177 |
| -0.549 | NA | NA | + | NA | -5.90E-02 | 2.29E-01 | 7 | -167.288 | 348.576 | 1.252 | 0.171 |
| -0.61 | NA | NA | + | -1.43E-03 | -7.57E-02 | 2.34E-01 | 8 | -166.539 | 349.078 | 1.755 | 0.133 |
| -0.359 | NA | NA | + | NA | NA | 1.44E-01 | 6 | -140.361 | 292.721 | 0 | 0.309 |
| -0.3 | NA | NA | NA | NA | NA | 1.83E-01 | 3 | -143.997 | 293.994 | 1.273 | 0.164 |
| -0.218 | NA | NA | NA | 1.19E-03 | NA | 1.59E-01 | 4 | -143.05 | 294.099 | 1.378 | 0.155 |
| -0.384 | 7.90E-10 | NA | + | NA | NA | 1.50E-01 | 7 | -140.206 | 294.412 | I.69 | 0.133 |
| -0.299 | NA | NA | + | NA | -2.79E-02 | 1.46E-01 | 7 | -140.273 | 294.546 | 1.825 | 0.124 |
| -0.371 | NA | NA | + | -1.31E-04 | NA | 1.45E-01 | 7 | -140.354 | 294.709 | 1.988 | 0.115 |
| -0.474 | NA | NA | + | NA | NA | 1.68E-01 | 6 | -159.532 | 331.063 | 0 | 0.457 |
| -0.506 | 1.13E-09 | NA | + | NA | NA | 1.73E-01 | 7 | -159.336 | 332.671 | 1.608 | 0.204 |
| -0.448 | NA | NA | + | NA | -1.20E-02 | 1.68E-01 | 7 | -159.518 | 333.036 | 1.973 | 0.17 |
| -0.481 | NA | NA | + | -8.14E-05 | NA | 1.68E-01 | 7 | -159.53 | 333.059 | 1.996 | 0.168 |
| -0.336 | NA | NA | + | NA | NA | 1.40E-01 | 6 | -137.432 | 286.864 | 0 | 0.217 |
| -0.272 | NA | NA | NA | NA | NA | 1.68E-01 | 3 | -140.531 | 287.062 | 0.198 | 0.196 |
| -0.202 | NA | NA | NA | 1.10E-03 | NA | 1.48E-01 | 4 | -139.658 | 287.315 | 0.451 | 0.173 |
| -0.358 | 7.27E-10 | NA | + | NA | NA | 1.44E-01 | 7 | -137.309 | 288.618 | 1.754 | 0.09 |
| -0.294 | 7.56E-10 | NA | NA | NA | NA | 1.72E-01 | 4 | -140.403 | 288.805 | 1.941 | 0.082 |
| -0.208 | NA | NA | NA | NA | -3.32E-02 | 1.68E-01 | 4 | -140.405 | 288.81 | 1.946 | 0.082 |
| -0.341 | NA | NA | + | -5.61E-05 | NA | 1.41E-01 | 7 | -137.431 | 288.862 | 1.998 | 0.08 |
| -0.34 | NA | NA | + | NA | 1.84E-03 | 1.40E-01 | 7 | -137.432 | 288.863 | 1.999 | 0.08 |
| -0.497 | NA | NA | + | NA | NA | 1.78E-01 | 6 | -149.1 | 310.201 | 0 | 0.423 |
| -0.533 | 1.18E-09 | NA | + | NA | NA | 1.84E-01 | 7 | -148.824 | 311.648 | 1.448 | 0.205 |
| -0.403 | NA | NA | + | NA | -4.29E-02 | 1.80E-01 | 7 | -148.885 | 311.77 | I.57 | 0.193 |
| -0.544 | NA | NA | + | -6.09E-04 | NA | 1.81E-01 | 7 | -148.966 | 311.932 | 1.732 | 0.178 |
| -0.425 | NA | NA | + | NA | NA | 1.56E-01 | 6 | -147.356 | 306.711 | 0 | 0.438 |
| -0.485 | NA | NA | + | -7.59E-04 | NA | 1.60E-01 | 7 | -147.15 | 308.3 | 1.589 | 0.198 |
| -0.455 | 1.04E-09 | NA | + | NA | NA | 1.61E-01 | 7 | -147.176 | 308.352 | 1.641 | 0.193 |
| -0.371 | NA | NA | + | NA | -2.46E-02 | 1.57E-01 | 7 | -147.292 | 308.585 | 1.874 | 0.172 |
| -0.493 | NA | NA | + | NA | NA | 1.81E-01 | 6 | -142.233 | 296.467 | 0 | 0.256 |
| -0.317 | NA | NA | + | NA | -7.79E-02 | 1.82E-01 | 7 | -141.418 | 296.836 | 0.369 | 0.213 |
| -0.385 | NA | NA | + | -1.25E-03 | -9.09E-02 | 1.88E-01 | 8 | -140.74 | 297.48 | 1.013 | 0.154 |
| -0.568 | NA | NA | + | -9.63E-04 | NA | 1.85E-01 | 7 | -141.823 | 297.646 | 1.179 | 0.142 |
| -0.529 | 1.05E-09 | NA | + | NA | NA | 1.90E-01 | 7 | -141.895 | 297.79 | 1.323 | 0.132 |
| -0.356 | 9.45E-10 | NA | + | NA | -7.49E-02 | 1.90E-01 | 8 | -141.141 | 298.283 | 1.816 | 0.103 |
| -0.589 | NA | NA | + | NA | NA | 2.01E-01 | 6 | -157.275 | 326.55 | 0 | 0.311 |
| -0.669 | NA | NA | + | -1.19E-03 | NA | 2.04E-01 | 7 | -156.722 | 327.444 | 0.894 | 0.199 |
| -0.451 | NA | NA | + | NA | -6.28E-02 | 2.02E-01 | 7 | -156.84 | 327.68 | I.13 | 0.177 |
| -0.632 | 1.28E-09 | NA | + | NA | NA | 2.09E-01 | 7 | -156.869 | 327.737 | 1.187 | 0.172 |
| -0.514 | NA | NA | + | -1.42E-03 | -7.76E-02 | 2.07E-01 | 8 | -156.072 | 328.144 | 1.595 | 0.14 |
| -0.449 | NA | NA | + | NA | NA | 1.68E-01 | 6 | -142.246 | 296.491 | 0 | 0.326 |
| -0.54 | NA | NA | + | -1.15E-03 | NA | 1.73E-01 | 7 | -141.69 | 297.381 | 0.89 | 0.209 |
| -0.329 | NA | NA | + | NA | -5.49E-02 | 1.68E-01 | 7 | -141.894 | 297.788 | 1.297 | 0.17 |
| -0.485 | 1.27E-09 | NA | + | NA | NA | 1.73E-01 | 7 | -141.947 | 297.895 | 1.403 | 0.161 |
| -0.404 | NA | NA | + | -1.37E-03 | -7.01E-02 | 1.76E-01 | 8 | -141.132 | 298.264 | 1.773 | 0.134 |
| -0.399 | NA | NA | + | NA | NA | 1.45E-01 | 6 | -142.007 | 296.014 | 0 | 0.441 |
| -0.429 | 7.63E-10 | NA | + | NA | NA | 1.52E-01 | 7 | -141.792 | 297.584 | I.57 | 0.201 |
| -0.442 | NA | NA | + | -5.19E-04 | NA | 1.47E-01 | 7 | -141.907 | 297.814 | 01.VIII | 0.179 |
| -0.332 | NA | NA | + | NA | -2.96E-02 | 1.45E-01 | 7 | -141.912 | 297.824 | 1.809 | 0.178 |
| -0.397 | NA | NA | + | NA | NA | 1.53E-01 | 6 | -144.693 | 301.386 | 0 | 0.453 |
| -0.425 | 9.34E-10 | NA | + | NA | NA | 1.58E-01 | 7 | -144.511 | 303.023 | 1.637 | 0.2 |
| -0.339 | NA | NA | + | NA | -2.66E-02 | 1.53E-01 | 7 | -144.618 | 303.236 | I.85 | 0.18 |
| -0.411 | NA | NA | + | -1.61E-04 | NA | 1.54E-01 | 7 | -144.684 | 303.368 | 1.982 | 0.168 |
| -0.586 | NA | NA | + | NA | NA | 1.97E-01 | 6 | -164.462 | 340.924 | 0 | 0.432 |
| -0.627 | 1.65E-09 | NA | + | NA | NA | 2.03E-01 | 7 | -164.166 | 342.332 | 1.408 | 0.214 |
| -0.638 | NA | NA | + | -7.26E-04 | NA | 1.99E-01 | 7 | -164.292 | 342.584 | I.66 | 0.188 |
| -0.538 | NA | NA | + | NA | -2.16E-02 | 1.98E-01 | 7 | -164.416 | 342.831 | 1.907 | 0.166 |
| -0.5 | NA | NA | + | NA | NA | 1.82E-01 | 6 | -159.393 | 330.786 | 0 | 0.412 |
| -0.543 | 1.58E-09 | NA | + | NA | NA | 1.89E-01 | 7 | -159.046 | 332.093 | 1.307 | 0.215 |
| -0.386 | NA | NA | + | NA | -5.38E-02 | 1.84E-01 | 7 | -159.057 | 332.113 | 1.327 | 0.212 |
| -0.533 | NA | NA | + | -4.15E-04 | NA | 1.84E-01 | 7 | -159.335 | 332.67 | 1.884 | 0.161 |
| -0.757 | NA | NA | + | NA | NA | 2.45E-01 | 6 | -178.053 | 368.107 | 0 | 0.267 |
| -0.571 | NA | NA | + | NA | -7.99E-02 | 2.42E-01 | 7 | -177.334 | 368.667 | 0.561 | 0.201 |
| -0.811 | 1.96E-09 | NA | + | NA | NA | 2.53E-01 | 7 | -177.565 | 369.13 | 1.023 | 0.16 |
| -0.817 | NA | NA | + | -9.08E-04 | NA | 2.48E-01 | 7 | -177.741 | 369.482 | 1.375 | 0.134 |
| -0.62 | NA | NA | + | -1.22E-03 | -9.36E-02 | 2.46E-01 | 8 | -176.784 | 369.568 | 1.462 | 0.128 |
| -0.633 | 1.76E-09 | NA | + | NA | -7.44E-02 | 2.50E-01 | 8 | -176.943 | 369.886 | 1.779 | 0.11 |
| -0.448 | NA | NA | + | NA | NA | 1.66E-01 | 6 | -147.766 | 307.532 | 0 | 0.428 |
| -0.481 | 1.02E-09 | NA | + | NA | NA | 1.72E-01 | 7 | -147.524 | 309.048 | 1.516 | 0.201 |
| -0.359 | NA | NA | + | NA | -4.08E-02 | 1.67E-01 | 7 | -147.575 | 309.149 | 1.617 | 0.191 |
| -0.499 | NA | NA | + | -6.16E-04 | NA | 1.70E-01 | 7 | -147.627 | 309.253 | 1.721 | 0.181 |
| -0.584 | NA | NA | + | NA | NA | 2.08E-01 | 6 | -145.781 | 303.563 | 0 | 0.241 |
| -0.688 | NA | NA | + | -1.47E-03 | NA | 2.15E-01 | 7 | -144.863 | 303.726 | 0.163 | 0.222 |
| -0.536 | NA | NA | + | -1.72E-03 | -7.72E-02 | 2.16E-01 | 8 | -144.14 | 304.279 | 0.717 | 0.168 |
| -0.457 | NA | NA | + | NA | -5.81E-02 | 2.08E-01 | 7 | -145.362 | 304.725 | 1.162 | 0.135 |
| -0.622 | 1.40E-09 | NA | + | NA | NA | 2.15E-01 | 7 | -145.472 | 304.944 | 1.381 | 0.121 |
| -0.728 | 1.45E-09 | NA | + | -1.49E-03 | NA | 2.21E-01 | 8 | -144.526 | 305.052 | I.49 | 0.114 |
| -0.521 | NA | NA | + | NA | NA | 1.78E-01 | 6 | -151.692 | 315.384 | 0 | 0.412 |
| -0.587 | NA | NA | + | -9.24E-04 | NA | 1.81E-01 | 7 | -151.371 | 316.742 | 1.357 | 0.209 |
| -0.557 | 1.24E-09 | NA | + | NA | NA | 1.85E-01 | 7 | -151.436 | 316.872 | 1.487 | 0.196 |
| -0.426 | NA | NA | + | NA | -4.18E-02 | 1.78E-01 | 7 | -151.503 | 317.007 | 1.622 | 0.183 |
| -0.471 | NA | NA | + | NA | NA | 1.66E-01 | 6 | -147.919 | 307.839 | 0 | 0.419 |
| -0.35 | NA | NA | + | NA | -5.37E-02 | 1.67E-01 | 7 | -147.598 | 309.197 | 1.358 | 0.212 |
| -0.507 | 1.34E-09 | NA | + | NA | NA | 1.71E-01 | 7 | -147.643 | 309.286 | 1.447 | 0.203 |
| -0.509 | NA | NA | + | -4.57E-04 | NA | 1.68E-01 | 7 | -147.843 | 309.686 | 1.848 | 0.166 |
| -0.606 | NA | NA | + | -2.27E-03 | -1.19E-01 | 2.46E-01 | 8 | -155.189 | 326.378 | 0 | 0.501 |
| -0.663 | 1.55E-09 | NA | + | -2.27E-03 | -1.13E-01 | 2.54E-01 | 9 | -154.817 | 327.635 | 1.257 | 0.267 |
| -0.856 | NA | NA | + | -1.89E-03 | NA | 2.49E-01 | 7 | -156.958 | 327.917 | 1.539 | 0.232 |
| -0.508 | NA | NA | + | NA | NA | 1.83E-01 | 6 | -151.877 | 315.754 | 0 | 0.416 |
| -0.379 | NA | NA | + | NA | -5.77E-02 | 1.83E-01 | 7 | -151.491 | 316.981 | 1.228 | 0.225 |
| -0.543 | 1.31E-09 | NA | + | NA | NA | 1.88E-01 | 7 | -151.623 | 317.247 | 1.493 | 0.197 |
| -0.537 | NA | NA | + | -3.90E-04 | NA | 1.85E-01 | 7 | -151.817 | 317.635 | 1.881 | 0.162 |
| -0.414 | NA | NA | + | NA | NA | 1.51E-01 | 6 | -145.13 | 302.26 | 0 | 0.433 |
| -0.481 | NA | NA | + | -7.81E-04 | NA | 1.56E-01 | 7 | -144.918 | 303.837 | 1.577 | 0.197 |
| -0.442 | 9.86E-10 | NA | + | NA | NA | 1.56E-01 | 7 | -144.956 | 303.912 | 1.653 | 0.189 |
| -0.339 | NA | NA | + | NA | -3.45E-02 | 1.53E-01 | 7 | -145.002 | 304.003 | 1.744 | 0.181 |
| -0.445 | NA | NA | + | NA | NA | 1.67E-01 | 6 | -139.679 | 291.358 | 0 | 0.306 |
| -0.29 | NA | NA | + | NA | -6.89E-02 | 1.67E-01 | 7 | -139.054 | 292.107 | 0.749 | 0.21 |
| -0.529 | NA | NA | + | -1.02E-03 | NA | 1.73E-01 | 7 | -139.237 | 292.474 | 1.116 | 0.175 |
| -0.482 | 1.18E-09 | NA | + | NA | NA | 1.74E-01 | 7 | -139.362 | 292.724 | 1.367 | 0.154 |
| -0.364 | NA | NA | + | -1.29E-03 | -8.27E-02 | 1.74E-01 | 8 | -138.363 | 292.726 | 1.368 | 0.154 |
| -0.686 | NA | NA | + | NA | NA | 2.28E-01 | 6 | -157.63 | 327.26 | 0 | 0.244 |
| -0.783 | NA | NA | + | -1.41E-03 | NA | 2.34E-01 | 7 | -156.829 | 327.658 | 0.398 | 0.2 |
| -0.607 | NA | NA | + | -1.70E-03 | -8.66E-02 | 2.35E-01 | 8 | -155.983 | 327.966 | 0.706 | 0.171 |
| -0.535 | NA | NA | + | NA | -6.70E-02 | 2.28E-01 | 7 | -157.11 | 328.22 | 0.96 | 0.151 |
| -0.728 | 1.50E-09 | NA | + | NA | NA | 2.36E-01 | 7 | -157.272 | 328.543 | 1.283 | 0.128 |
| -0.827 | 1.53E-09 | NA | + | -1.42E-03 | NA | 2.42E-01 | 8 | -156.453 | 328.905 | 1.646 | 0.107 |
| -0.469 | NA | NA | + | NA | NA | 1.72E-01 | 6 | -157.2 | 326.4 | 0 | 0.408 |
| -0.557 | NA | NA | + | -1.16E-03 | NA | 1.77E-01 | 7 | -156.735 | 327.471 | 1.071 | 0.239 |
| -0.505 | 1.27E-09 | NA | + | NA | NA | 1.78E-01 | 7 | -156.945 | 327.89 | I.49 | 0.194 |
| -0.416 | NA | NA | + | NA | -2.50E-02 | 1.74E-01 | 7 | -157.134 | 328.269 | 1.868 | 0.16 |
| -0.481 | NA | NA | + | -1.87E-03 | -1.18E-01 | 2.18E-01 | 8 | -150.594 | 317.189 | 0 | 0.318 |
| -0.39 | NA | NA | + | NA | -9.64E-02 | 2.09E-01 | 7 | -152.067 | 318.133 | 0.944 | 0.199 |
| -0.611 | NA | NA | + | NA | NA | 2.11E-01 | 6 | -153.247 | 318.494 | 1.305 | 0.166 |
| -0.529 | 1.40E-09 | NA | + | -1.86E-03 | -1.14E-01 | 2.25E-01 | 9 | -150.264 | 318.528 | 1.339 | 0.163 |
| -0.721 | NA | NA | + | -1.47E-03 | NA | 2.19E-01 | 7 | -152.319 | 318.638 | 1.449 | 0.154 |
| -0.422 | NA | NA | + | NA | NA | 1.62E-01 | 6 | -144.16 | 300.319 | 0 | 0.449 |
| -0.454 | 1.16E-09 | NA | + | NA | NA | 1.66E-01 | 7 | -143.951 | 301.902 | 1.583 | 0.203 |
| -0.462 | NA | NA | + | -4.69E-04 | NA | 1.65E-01 | 7 | -144.083 | 302.166 | 1.847 | 0.178 |
| -0.39 | NA | NA | + | NA | -1.52E-02 | 1.63E-01 | 7 | -144.135 | 302.269 | I.95 | 0.169 |
| -0.505 | NA | NA | + | NA | NA | 1.87E-01 | 6 | -158.558 | 329.115 | 0 | 0.453 |
| -0.54 | 1.40E-09 | NA | + | NA | NA | 1.92E-01 | 7 | -158.336 | 330.671 | 1.556 | 0.208 |
| -0.469 | NA | NA | + | NA | -1.67E-02 | 1.87E-01 | 7 | -158.53 | 331.06 | 1.944 | 0.171 |
| -0.517 | NA | NA | + | -1.51E-04 | NA | 1.87E-01 | 7 | -158.55 | 331.1 | 1.985 | 0.168 |
| -0.637 | NA | NA | + | NA | NA | 2.05E-01 | 6 | -158.118 | 328.237 | 0 | 0.296 |
| -0.466 | NA | NA | + | NA | -7.25E-02 | 2.04E-01 | 7 | -157.528 | 329.055 | 0.819 | 0.196 |
| -0.72 | NA | NA | + | -1.18E-03 | NA | 2.09E-01 | 7 | -157.572 | 329.144 | 0.908 | 0.188 |
| -0.531 | NA | NA | + | -1.46E-03 | -8.82E-02 | 2.09E-01 | 8 | -156.72 | 329.441 | 1.204 | 0.162 |
| -0.683 | 1.55E-09 | NA | + | NA | NA | 2.12E-01 | 7 | -157.741 | 329.482 | 1.246 | 0.159 |
| -0.379 | NA | NA | + | NA | NA | 1.42E-01 | 6 | -146.78 | 305.56 | 0 | 0.317 |
| -0.302 | NA | NA | NA | NA | NA | 1.79E-01 | 3 | -150.434 | 306.869 | 1.309 | 0.165 |
| -0.229 | NA | NA | NA | 1.22E-03 | NA | 1.57E-01 | 4 | -149.569 | 307.138 | 1.578 | 0.144 |
| -0.408 | 9.89E-10 | NA | + | NA | NA | 1.47E-01 | 7 | -146.597 | 307.194 | 1.634 | 0.14 |
| -0.386 | NA | NA | + | -9.17E-05 | NA | 1.43E-01 | 7 | -146.777 | 307.555 | 1.995 | 0.117 |
| -0.38 | NA | NA | + | NA | 4.72E-04 | 1.42E-01 | 7 | -146.78 | 307.56 | 2 | 0.117 |
| -0.363 | NA | NA | + | NA | NA | 1.41E-01 | 6 | -149.689 | 311.378 | 0 | 0.264 |
| -0.206 | NA | NA | NA | 1.42E-03 | NA | 1.51E-01 | 4 | -151.861 | 311.722 | 0.343 | 0.222 |
| -0.299 | NA | NA | NA | NA | NA | 1.78E-01 | 3 | -152.996 | 311.991 | 0.613 | 0.194 |
| -0.393 | 9.21E-10 | NA | + | NA | NA | 1.47E-01 | 7 | -149.513 | 313.026 | 1.648 | 0.116 |
| -0.31 | NA | NA | + | NA | -2.48E-02 | 1.42E-01 | 7 | -149.627 | 313.254 | 1.875 | 0.103 |
| -0.336 | NA | NA | + | 3.12E-04 | NA | 1.39E-01 | 7 | -149.658 | 313.316 | 1.938 | 0.1 |
| -0.549 | NA | NA | + | NA | NA | 1.88E-01 | 6 | -156.142 | 324.285 | 0 | 0.405 |
| -0.632 | NA | NA | + | -1.12E-03 | NA | 1.92E-01 | 7 | -155.689 | 325.377 | 1.093 | 0.235 |
| -0.584 | 1.34E-09 | NA | + | NA | NA | 1.93E-01 | 7 | -155.921 | 325.843 | 1.558 | 0.186 |
| -0.461 | NA | NA | + | NA | -3.92E-02 | 1.88E-01 | 7 | -155.985 | 325.971 | 1.686 | 0.174 |
| -0.401 | NA | NA | + | NA | NA | 1.55E-01 | 6 | -158.109 | 328.219 | 0 | 0.276 |
| -0.253 | NA | NA | NA | 1.44E-03 | NA | 1.66E-01 | 4 | -160.373 | 328.746 | 0.527 | 0.212 |
| -0.353 | NA | NA | NA | NA | NA | 1.94E-01 | 3 | -161.514 | 329.027 | 0.809 | 0.184 |
| -0.431 | 9.57E-10 | NA | + | NA | NA | 1.61E-01 | 7 | -157.922 | 329.843 | 1.625 | 0.123 |
| -0.424 | NA | NA | + | NA | 1.13E-02 | 1.54E-01 | 7 | -158.097 | 330.195 | 1.976 | 0.103 |
| -0.387 | NA | NA | + | 1.59E-04 | NA | 1.54E-01 | 7 | -158.102 | 330.204 | 1.985 | 0.102 |
| -0.423 | NA | NA | + | NA | NA | 1.53E-01 | 6 | -149.285 | 310.57 | 0 | 0.453 |
| -0.452 | 8.72E-10 | NA | + | NA | NA | 1.58E-01 | 7 | -149.121 | 312.242 | 1.673 | 0.196 |
| -0.468 | NA | NA | + | -5.47E-04 | NA | 1.56E-01 | 7 | -149.189 | 312.378 | 1.809 | 0.183 |
| -0.445 | NA | NA | + | NA | 9.93E-03 | 1.53E-01 | 7 | -149.275 | 312.551 | 1.981 | 0.168 |
| -0.534 | NA | NA | + | NA | NA | 1.92E-01 | 6 | -143.241 | 298.482 | 0 | 0.277 |
| -0.637 | NA | NA | + | -1.36E-03 | NA | 1.98E-01 | 7 | -142.496 | 298.992 | 0.51 | 0.215 |
| -0.427 | NA | NA | + | NA | -4.93E-02 | 1.93E-01 | 7 | -142.946 | 299.891 | 1.409 | 0.137 |
| -0.57 | 1.40E-09 | NA | + | NA | NA | 1.97E-01 | 7 | -142.968 | 299.935 | 1.453 | 0.134 |
| -0.51 | NA | NA | + | -1.57E-03 | -6.55E-02 | 2.01E-01 | 8 | -141.985 | 299.969 | 1.487 | 0.132 |
| -0.674 | 1.41E-09 | NA | + | -1.37E-03 | NA | 2.04E-01 | 8 | -142.214 | 300.429 | 1.947 | 0.105 |
| -0.278 | NA | NA | + | NA | NA | 1.23E-01 | 6 | -135.447 | 282.894 | 0 | 0.206 |
| -0.249 | NA | NA | NA | NA | NA | 1.59E-01 | 3 | -138.473 | 282.946 | 0.052 | 0.2 |
| -0.16 | NA | NA | NA | 1.08E-03 | NA | 1.33E-01 | 4 | -137.655 | 283.31 | 0.416 | 0.167 |
| -0.149 | NA | NA | NA | NA | -4.74E-02 | 1.56E-01 | 4 | -138.206 | 284.413 | 1.519 | 0.096 |
| -0.303 | 8.68E-10 | NA | + | NA | NA | 1.27E-01 | 7 | -135.299 | 284.598 | 1.704 | 0.088 |
| -0.275 | 9.17E-10 | NA | NA | NA | NA | 1.63E-01 | 4 | -138.314 | 284.628 | 1.734 | 0.086 |
| -0.228 | NA | NA | + | NA | -2.33E-02 | 1.24E-01 | 7 | -135.385 | 284.771 | 1.877 | 0.08 |
| -0.281 | NA | NA | + | -2.77E-05 | NA | 1.23E-01 | 7 | -135.447 | 284.893 | 1.999 | 0.076 |
| -0.452 | NA | NA | + | NA | NA | 1.72E-01 | 6 | -146.854 | 305.708 | 0 | 0.434 |
| -0.485 | 1.12E-09 | NA | + | NA | NA | 1.78E-01 | 7 | -146.585 | 307.169 | 1.462 | 0.209 |
| -0.369 | NA | NA | + | NA | -3.80E-02 | 1.73E-01 | 7 | -146.699 | 307.398 | I.69 | 0.186 |
| -0.487 | NA | NA | + | -4.27E-04 | NA | 1.74E-01 | 7 | -146.786 | 307.571 | 1.864 | 0.171 |
| -0.411 | NA | NA | + | NA | NA | 1.49E-01 | 6 | -151.6 | 315.2 | 0 | 0.456 |
| -0.44 | 1.10E-09 | NA | + | NA | NA | 1.53E-01 | 7 | -151.43 | 316.861 | 1.661 | 0.199 |
| -0.445 | NA | NA | + | -4.02E-04 | NA | 1.51E-01 | 7 | -151.55 | 317.1 | 01.IX | 0.176 |
| -0.395 | NA | NA | + | NA | -7.17E-03 | 1.49E-01 | 7 | -151.595 | 317.19 | I.99 | 0.169 |
| -0.453 | NA | NA | + | NA | NA | 1.68E-01 | 6 | -146.566 | 305.133 | 0 | 0.426 |
| -0.519 | NA | NA | + | -7.91E-04 | NA | 1.72E-01 | 7 | -146.338 | 306.675 | 1.542 | 0.197 |
| -0.483 | 9.74E-10 | NA | + | NA | NA | 1.73E-01 | 7 | -146.36 | 306.72 | 1.587 | 0.193 |
| -0.37 | NA | NA | + | NA | -3.77E-02 | 1.69E-01 | 7 | -146.407 | 306.814 | 1.682 | 0.184 |
| -0.559 | NA | NA | + | NA | NA | 1.91E-01 | 6 | -155.265 | 322.53 | 0 | 0.386 |
| -0.636 | NA | NA | + | -1.07E-03 | NA | 1.95E-01 | 7 | -154.824 | 323.647 | 1.117 | 0.221 |
| -0.598 | 1.23E-09 | NA | + | NA | NA | 1.99E-01 | 7 | -154.918 | 323.835 | 1.305 | 0.201 |
| -0.446 | NA | NA | + | NA | -5.18E-02 | 1.93E-01 | 7 | -154.966 | 323.933 | 1.402 | 0.192 |
| -0.362 | NA | NA | + | NA | NA | 1.41E-01 | 6 | -147.308 | 306.616 | 0 | 0.317 |
| -0.308 | NA | NA | NA | NA | NA | 1.79E-01 | 3 | -150.995 | 307.99 | 1.373 | 0.16 |
| -0.218 | NA | NA | NA | 1.26E-03 | NA | 1.54E-01 | 4 | -150.031 | 308.063 | 1.446 | 0.154 |
| -0.386 | 9.31E-10 | NA | + | NA | NA | 1.45E-01 | 7 | -147.184 | 308.369 | 1.753 | 0.132 |
| -0.395 | NA | NA | + | NA | 1.55E-02 | 1.41E-01 | 7 | -147.285 | 308.571 | 1.955 | 0.119 |
| -0.373 | NA | NA | + | -1.27E-04 | NA | 1.42E-01 | 7 | -147.303 | 308.606 | I.99 | 0.117 |
| -0.558 | NA | NA | + | NA | NA | 1.92E-01 | 6 | -154.648 | 321.297 | 0 | 0.409 |
| -0.634 | NA | NA | + | -1.00E-03 | NA | 1.97E-01 | 7 | -154.294 | 322.589 | 1.292 | 0.214 |
| -0.593 | 1.05E-09 | NA | + | NA | NA | 1.99E-01 | 7 | -154.4 | 322.799 | 1.503 | 0.193 |
| -0.463 | NA | NA | + | NA | -4.34E-02 | 1.93E-01 | 7 | -154.45 | 322.9 | 1.604 | 0.183 |
| -0.679 | NA | NA | + | -1.80E-03 | NA | 1.94E-01 | 7 | -141.358 | 296.716 | 0 | 0.311 |
| -0.544 | NA | NA | + | NA | NA | 1.87E-01 | 6 | -142.647 | 297.293 | 0.577 | 0.233 |
| -0.562 | NA | NA | + | -2.00E-03 | -6.10E-02 | 1.97E-01 | 8 | -140.927 | 297.853 | 1.137 | 0.176 |
| -0.719 | 1.44E-09 | NA | + | -1.82E-03 | NA | 2.00E-01 | 8 | -141.001 | 298.002 | 1.286 | 0.163 |
| -0.58 | 1.37E-09 | NA | + | NA | NA | 1.93E-01 | 7 | -142.328 | 298.656 | 1.939 | 0.118 |
| -0.5 | NA | NA | + | NA | NA | 1.76E-01 | 6 | -149.338 | 310.676 | 0 | 0.391 |
| -0.582 | NA | NA | + | -1.10E-03 | NA | 1.82E-01 | 7 | -148.892 | 311.784 | 1.108 | 0.225 |
| -0.385 | NA | NA | + | NA | -5.31E-02 | 1.78E-01 | 7 | -149.016 | 312.032 | 1.356 | 0.199 |
| -0.537 | 1.24E-09 | NA | + | NA | NA | 1.83E-01 | 7 | -149.083 | 312.167 | 1.491 | 0.186 |
| -0.48 | NA | NA | + | -1.75E-03 | -1.05E-01 | 2.12E-01 | 8 | -147.463 | 310.926 | 0 | 0.203 |
| -0.589 | NA | NA | + | NA | NA | 2.05E-01 | 6 | -149.717 | 311.433 | 0.507 | 0.158 |
| -0.398 | NA | NA | + | NA | -8.70E-02 | 2.05E-01 | 7 | -148.768 | 311.536 | 0.61 | 0.15 |
| -0.689 | NA | NA | + | -1.44E-03 | NA | 2.10E-01 | 7 | -148.827 | 311.654 | 0.728 | 0.141 |
| -0.527 | 1.21E-09 | NA | + | -1.77E-03 | -1.02E-01 | 2.19E-01 | 9 | -147.108 | 312.216 | I.29 | 0.107 |
| -0.63 | 1.29E-09 | NA | + | NA | NA | 2.13E-01 | 7 | -149.325 | 312.65 | 1.724 | 0.086 |
| -0.735 | 1.35E-09 | NA | + | -1.47E-03 | NA | 2.19E-01 | 8 | -148.389 | 312.779 | 1.853 | 0.08 |
| -0.442 | 1.16E-09 | NA | + | NA | -8.37E-02 | 2.13E-01 | 8 | -148.448 | 312.896 | I.97 | 0.076 |
| -0.524 | NA | NA | + | NA | NA | 1.85E-01 | 6 | -144.661 | 301.323 | 0 | 0.2 |
| -0.432 | NA | NA | + | -1.56E-03 | -9.58E-02 | 1.94E-01 | 8 | -142.86 | 301.721 | 0.398 | 0.164 |
| -0.355 | NA | NA | + | NA | -7.68E-02 | 1.87E-01 | 7 | -143.92 | 301.84 | 0.517 | 0.154 |
| -0.618 | NA | NA | + | -1.23E-03 | NA | 1.90E-01 | 7 | -143.978 | 301.955 | 0.632 | 0.145 |
| -0.563 | 1.32E-09 | NA | + | NA | NA | 1.91E-01 | 7 | -144.31 | 302.619 | 1.296 | 0.104 |
| -0.471 | 1.19E-09 | NA | + | -1.54E-03 | -9.31E-02 | 2.00E-01 | 9 | -142.572 | 303.145 | 1.822 | 0.08 |
| -0.396 | 1.23E-09 | NA | + | NA | -7.43E-02 | 1.93E-01 | 8 | -143.615 | 303.23 | 1.907 | 0.077 |
| -0.656 | 1.31E-09 | NA | + | -1.22E-03 | NA | 1.96E-01 | 8 | -143.631 | 303.261 | 1.938 | 0.076 |
| -0.424 | NA | NA | + | NA | NA | 1.56E-01 | 6 | -156.572 | 325.144 | 0 | 0.459 |
| -0.452 | 9.16E-10 | NA | + | NA | NA | 1.60E-01 | 7 | -156.425 | 326.851 | 1.707 | 0.196 |
| -0.385 | NA | NA | + | NA | -1.78E-02 | 1.56E-01 | 7 | -156.542 | 327.083 | I.94 | 0.174 |
| -0.439 | NA | NA | + | -1.81E-04 | NA | 1.57E-01 | 7 | -156.561 | 327.123 | 1.979 | 0.171 |
| -0.539 | NA | NA | + | NA | NA | 1.91E-01 | 6 | -146.192 | 304.385 | 0 | 0.304 |
| -0.381 | NA | NA | + | NA | -6.99E-02 | 1.91E-01 | 7 | -145.592 | 305.184 | 0.799 | 0.204 |
| -0.621 | NA | NA | + | -1.07E-03 | NA | 1.97E-01 | 7 | -145.725 | 305.449 | 1.064 | 0.178 |
| -0.579 | 1.33E-09 | NA | + | NA | NA | 1.99E-01 | 7 | -145.842 | 305.684 | 01.III | 0.159 |
| -0.45 | NA | NA | + | -1.36E-03 | -8.51E-02 | 1.99E-01 | 8 | -144.861 | 305.722 | 1.338 | 0.156 |
| -0.634 | NA | NA | + | NA | NA | 2.16E-01 | 6 | -159.97 | 331.94 | 0 | 0.256 |
| -0.728 | NA | NA | + | -1.37E-03 | NA | 2.21E-01 | 7 | -159.217 | 332.434 | 0.494 | 0.2 |
| -0.565 | NA | NA | + | -1.62E-03 | -7.94E-02 | 2.21E-01 | 8 | -158.509 | 333.018 | 1.078 | 0.149 |
| -0.494 | NA | NA | + | NA | -6.21E-02 | 2.15E-01 | 7 | -159.527 | 333.053 | 1.113 | 0.147 |
| -0.68 | 1.67E-09 | NA | + | NA | NA | 2.24E-01 | 7 | -159.585 | 333.171 | 1.231 | 0.138 |
| -0.774 | 1.68E-09 | NA | + | -1.38E-03 | NA | 2.29E-01 | 8 | -158.826 | 333.652 | 1.712 | 0.109 |
| -0.471 | NA | NA | + | NA | NA | 1.70E-01 | 6 | -162.671 | 337.342 | 0 | 0.449 |
| -0.505 | 1.15E-09 | NA | + | NA | NA | 1.76E-01 | 7 | -162.445 | 338.889 | 1.548 | 0.207 |
| -0.41 | NA | NA | + | NA | -2.74E-02 | 1.71E-01 | 7 | -162.6 | 339.199 | 1.858 | 0.178 |
| -0.478 | NA | NA | + | -8.86E-05 | NA | 1.71E-01 | 7 | -162.668 | 339.336 | 1.995 | 0.166 |
| -0.482 | NA | NA | + | NA | -9.22E-02 | 2.30E-01 | 7 | -163.825 | 341.651 | 0 | 0.18 |
| -0.542 | NA | NA | + | -1.52E-03 | -1.08E-01 | 2.32E-01 | 8 | -162.862 | 341.723 | 0.072 | 0.173 |
| -0.696 | NA | NA | + | NA | NA | 2.32E-01 | 6 | -164.87 | 341.74 | 0.09 | 0.172 |
| -0.772 | NA | NA | + | -1.19E-03 | NA | 2.35E-01 | 7 | -164.269 | 342.538 | 0.887 | 0.115 |
| -0.748 | 1.67E-09 | NA | + | NA | NA | 2.42E-01 | 7 | -164.39 | 342.781 | I.13 | 0.102 |
| -0.539 | 1.46E-09 | NA | + | NA | -8.72E-02 | 2.38E-01 | 8 | -163.46 | 342.919 | 1.268 | 0.095 |
| -0.598 | 1.45E-09 | NA | + | -1.52E-03 | -1.03E-01 | 2.41E-01 | 9 | -162.496 | 342.993 | 1.342 | 0.092 |
| -0.825 | 1.69E-09 | NA | + | -1.21E-03 | NA | 2.45E-01 | 8 | -163.772 | 343.544 | 1.893 | 0.07 |
| -0.478 | NA | NA | + | NA | NA | 1.68E-01 | 6 | -150.973 | 313.946 | 0 | 0.427 |
| -0.518 | 1.35E-09 | NA | + | NA | NA | 1.75E-01 | 7 | -150.682 | 315.363 | 1.417 | 0.21 |
| -0.39 | NA | NA | + | NA | -3.84E-02 | 1.68E-01 | 7 | -150.812 | 315.624 | 1.677 | 0.185 |
| -0.525 | NA | NA | + | -5.90E-04 | NA | 1.71E-01 | 7 | -150.847 | 315.695 | 1.749 | 0.178 |
| -0.481 | NA | NA | + | NA | NA | 1.72E-01 | 6 | -155.489 | 322.978 | 0 | 0.446 |
| -0.514 | 1.09E-09 | NA | + | NA | NA | 1.79E-01 | 7 | -155.256 | 324.511 | 1.533 | 0.207 |
| -0.514 | NA | NA | + | -4.27E-04 | NA | 1.74E-01 | 7 | -155.428 | 324.856 | 1.878 | 0.174 |
| -0.431 | NA | NA | + | NA | -2.23E-02 | 1.72E-01 | 7 | -155.439 | 324.877 | 1.899 | 0.173 |
| -0.367 | NA | NA | + | NA | NA | 1.46E-01 | 6 | -146.195 | 304.39 | 0 | 0.28 |
| -0.225 | NA | NA | NA | 1.27E-03 | NA | 1.54E-01 | 4 | -148.54 | 305.08 | 0.69 | 0.198 |
| -0.311 | NA | NA | NA | NA | NA | 1.76E-01 | 3 | -149.557 | 305.114 | 0.724 | 0.195 |
| -0.394 | 8.96E-10 | NA | + | NA | NA | 1.51E-01 | 7 | -146.024 | 306.048 | 1.658 | 0.122 |
| -0.358 | NA | NA | + | NA | -4.45E-03 | 1.47E-01 | 7 | -146.193 | 306.386 | 1.996 | 0.103 |
| -0.368 | NA | NA | + | -4.50E-06 | NA | 1.46E-01 | 7 | -146.195 | 306.39 | 2 | 0.103 |
| -0.496 | NA | NA | + | NA | NA | 1.83E-01 | 6 | -141.278 | 294.556 | 0 | 0.409 |
| -0.564 | NA | NA | + | -9.02E-04 | NA | 1.87E-01 | 7 | -140.959 | 295.918 | 1.361 | 0.207 |
| -0.529 | 1.11E-09 | NA | + | NA | NA | 1.89E-01 | 7 | -141 | 295.999 | 1.443 | 0.199 |
| -0.406 | NA | NA | + | NA | -4.25E-02 | 1.85E-01 | 7 | -141.067 | 296.133 | 1.577 | 0.186 |
| -0.56 | NA | NA | + | NA | NA | 1.91E-01 | 6 | -156.982 | 325.964 | 0 | 0.394 |
| -0.425 | NA | NA | + | NA | -6.03E-02 | 1.91E-01 | 7 | -156.582 | 327.164 | 01.II | 0.216 |
| -0.601 | 1.31E-09 | NA | + | NA | NA | 1.99E-01 | 7 | -156.652 | 327.304 | 1.341 | 0.201 |
| -0.62 | NA | NA | + | -8.55E-04 | NA | 1.94E-01 | 7 | -156.715 | 327.431 | 1.467 | 0.189 |
| -0.524 | NA | NA | + | NA | NA | 1.82E-01 | 6 | -144.989 | 301.979 | 0 | 0.261 |
| -0.624 | NA | NA | + | -1.35E-03 | NA | 1.88E-01 | 7 | -144.247 | 302.494 | 0.515 | 0.202 |
| -0.47 | NA | NA | + | -1.60E-03 | -7.98E-02 | 1.92E-01 | 8 | -143.517 | 303.035 | 1.056 | 0.154 |
| -0.388 | NA | NA | + | NA | -6.30E-02 | 1.85E-01 | 7 | -144.525 | 303.051 | 1.072 | 0.153 |
| -0.56 | 1.21E-09 | NA | + | NA | NA | 1.89E-01 | 7 | -144.693 | 303.386 | 1.407 | 0.129 |
| -0.662 | 1.23E-09 | NA | + | -1.36E-03 | NA | 1.95E-01 | 8 | -143.938 | 303.876 | 1.897 | 0.101 |
| -0.414 | NA | NA | + | NA | NA | 1.51E-01 | 6 | -141.563 | 295.126 | 0 | 0.432 |
| -0.447 | 1.06E-09 | NA | + | NA | NA | 1.57E-01 | 7 | -141.328 | 296.656 | 1.529 | 0.201 |
| -0.474 | NA | NA | + | -7.16E-04 | NA | 1.55E-01 | 7 | -141.375 | 296.75 | 1.624 | 0.192 |
| -0.349 | NA | NA | + | NA | -3.01E-02 | 1.53E-01 | 7 | -141.462 | 296.924 | 1.797 | 0.176 |
| -0.473 | NA | NA | + | NA | NA | 1.71E-01 | 6 | -144.532 | 301.064 | 0 | 0.419 |
| -0.546 | NA | NA | + | -9.10E-04 | NA | 1.76E-01 | 7 | -144.214 | 302.428 | 1.364 | 0.212 |
| -0.504 | 1.03E-09 | NA | + | NA | NA | 1.77E-01 | 7 | -144.319 | 302.638 | 1.574 | 0.191 |
| -0.392 | NA | NA | + | NA | -3.65E-02 | 1.72E-01 | 7 | -144.385 | 302.769 | 1.705 | 0.179 |
| -0.413 | NA | NA | + | NA | NA | 1.60E-01 | 6 | -146.235 | 304.47 | 0 | 0.427 |
| -0.443 | 9.94E-10 | NA | + | NA | NA | 1.65E-01 | 7 | -146.018 | 306.035 | 1.566 | 0.195 |
| -0.327 | NA | NA | + | NA | -4.06E-02 | 1.62E-01 | 7 | -146.048 | 306.096 | 1.626 | 0.19 |
| -0.47 | NA | NA | + | -6.98E-04 | NA | 1.64E-01 | 7 | -146.059 | 306.118 | 1.648 | 0.188 |
| -0.492 | NA | NA | + | -1.73E-03 | -1.02E-01 | 2.12E-01 | 8 | -149.685 | 315.369 | 0 | 0.189 |
| -0.603 | NA | NA | + | NA | NA | 2.06E-01 | 6 | -151.847 | 315.694 | 0.325 | 0.161 |
| -0.414 | NA | NA | + | NA | -8.39E-02 | 2.06E-01 | 7 | -150.961 | 315.922 | 0.553 | 0.143 |
| -0.7 | NA | NA | + | -1.42E-03 | NA | 2.12E-01 | 7 | -150.965 | 315.93 | 0.56 | 0.143 |
| -0.544 | 1.22E-09 | NA | + | -1.75E-03 | -9.85E-02 | 2.22E-01 | 9 | -149.254 | 316.509 | I.14 | 0.107 |
| -0.647 | 1.27E-09 | NA | + | NA | NA | 2.16E-01 | 7 | -151.393 | 316.786 | 1.417 | 0.093 |
| -0.75 | 1.33E-09 | NA | + | -1.46E-03 | NA | 2.22E-01 | 8 | -150.457 | 316.915 | 1.545 | 0.087 |
| -0.463 | 1.16E-09 | NA | + | NA | -8.06E-02 | 2.15E-01 | 8 | -150.575 | 317.15 | 1.781 | 0.078 |
| -0.476 | NA | NA | + | NA | NA | 1.70E-01 | 6 | -154.4 | 320.799 | 0 | 0.424 |
| -0.557 | NA | NA | + | -1.03E-03 | NA | 1.74E-01 | 7 | -154.03 | 322.059 | I.26 | 0.226 |
| -0.508 | 9.55E-10 | NA | + | NA | NA | 1.76E-01 | 7 | -154.205 | 322.41 | 1.611 | 0.19 |
| -0.443 | NA | NA | + | NA | -1.55E-02 | 1.70E-01 | 7 | -154.375 | 322.751 | 1.951 | 0.16 |
| -0.356 | NA | NA | + | NA | NA | 1.40E-01 | 6 | -143.697 | 299.395 | 0 | 0.229 |
| -0.195 | NA | NA | NA | 1.32E-03 | NA | 1.48E-01 | 4 | -145.749 | 299.498 | 0.103 | 0.217 |
| -0.281 | NA | NA | NA | NA | NA | 1.74E-01 | 3 | -146.834 | 299.669 | 0.274 | 0.199 |
| -0.379 | 7.86E-10 | NA | + | NA | NA | 1.44E-01 | 7 | -143.578 | 301.157 | 1.762 | 0.095 |
| -0.219 | 7.70E-10 | NA | NA | 1.31E-03 | NA | 1.53E-01 | 5 | -145.637 | 301.275 | I.88 | 0.089 |
| -0.386 | NA | NA | + | NA | 1.41E-02 | 1.39E-01 | 7 | -143.677 | 301.355 | I.96 | 0.086 |
| -0.339 | NA | NA | + | 1.79E-04 | NA | 1.39E-01 | 7 | -143.687 | 301.374 | I.98 | 0.085 |
| -0.474 | NA | NA | + | NA | NA | 1.67E-01 | 6 | -156.021 | 324.043 | 0 | 0.445 |
| -0.507 | 1.10E-09 | NA | + | NA | NA | 1.73E-01 | 7 | -155.818 | 325.636 | 1.593 | 0.201 |
| -0.527 | NA | NA | + | -6.79E-04 | NA | 1.70E-01 | 7 | -155.871 | 325.741 | 1.698 | 0.19 |
| -0.459 | NA | NA | + | NA | -6.98E-03 | 1.68E-01 | 7 | -156.017 | 326.034 | 1.991 | 0.164 |
| -0.593 | NA | NA | + | NA | NA | 2.02E-01 | 6 | -159.353 | 330.706 | 0 | 0.264 |
| -0.408 | NA | NA | + | NA | -8.14E-02 | 2.02E-01 | 7 | -158.56 | 331.121 | 0.415 | 0.214 |
| -0.645 | 1.89E-09 | NA | + | NA | NA | 2.09E-01 | 7 | -158.875 | 331.751 | 1.045 | 0.156 |
| -0.651 | NA | NA | + | -8.11E-04 | NA | 2.06E-01 | 7 | -159.094 | 332.189 | 1.483 | 0.126 |
| -0.462 | NA | NA | + | -1.09E-03 | -9.23E-02 | 2.07E-01 | 8 | -158.101 | 332.202 | 1.497 | 0.125 |
| -0.467 | 1.68E-09 | NA | + | NA | -7.60E-02 | 2.09E-01 | 8 | -158.187 | 332.373 | 1.667 | 0.115 |
| -0.426 | NA | NA | + | NA | NA | 1.58E-01 | 6 | -136.555 | 285.11 | 0 | 0.393 |
| -0.531 | NA | NA | + | -1.24E-03 | NA | 1.62E-01 | 7 | -135.967 | 285.934 | 0.824 | 0.26 |
| -0.455 | 1.01E-09 | NA | + | NA | NA | 1.61E-01 | 7 | -136.368 | 286.736 | 1.626 | 0.174 |
| -0.345 | NA | NA | + | NA | -3.82E-02 | 1.62E-01 | 7 | -136.376 | 286.752 | 1.642 | 0.173 |
| -0.517 | NA | NA | + | NA | NA | 1.83E-01 | 6 | -158.616 | 329.232 | 0 | 0.433 |
| -0.552 | 1.22E-09 | NA | + | NA | NA | 1.90E-01 | 7 | -158.376 | 330.752 | 1.521 | 0.203 |
| -0.567 | NA | NA | + | -6.71E-04 | NA | 1.85E-01 | 7 | -158.455 | 330.909 | 1.678 | 0.187 |
| -0.448 | NA | NA | + | NA | -3.22E-02 | 1.85E-01 | 7 | -158.513 | 331.026 | 1.794 | 0.177 |
| -0.489 | NA | NA | + | -1.82E-03 | -1.11E-01 | 2.10E-01 | 8 | -151.186 | 318.372 | 0 | 0.238 |
| -0.401 | NA | NA | + | NA | -9.29E-02 | 2.03E-01 | 7 | -152.581 | 319.161 | 0.789 | 0.161 |
| -0.613 | NA | NA | + | NA | NA | 2.03E-01 | 6 | -153.662 | 319.325 | 0.952 | 0.148 |
| -0.72 | NA | NA | + | -1.49E-03 | NA | 2.08E-01 | 7 | -152.712 | 319.424 | 1.052 | 0.141 |
| -0.542 | 1.21E-09 | NA | + | -1.83E-03 | -1.08E-01 | 2.20E-01 | 9 | -150.75 | 319.5 | 1.128 | 0.136 |
| -0.452 | 1.17E-09 | NA | + | NA | -8.95E-02 | 2.13E-01 | 8 | -152.178 | 320.356 | 1.984 | 0.088 |
| -0.66 | 1.28E-09 | NA | + | NA | NA | 2.13E-01 | 7 | -153.185 | 320.369 | 1.997 | 0.088 |
| -0.595 | NA | NA | + | NA | NA | 2.07E-01 | 6 | -158.348 | 328.697 | 0 | 0.199 |
| -0.686 | NA | NA | + | -1.33E-03 | NA | 2.11E-01 | 7 | -157.624 | 329.247 | 0.551 | 0.151 |
| -0.501 | NA | NA | + | -1.60E-03 | -9.29E-02 | 2.12E-01 | 8 | -156.626 | 329.251 | 0.555 | 0.151 |
| -0.427 | NA | NA | + | NA | -7.70E-02 | 2.08E-01 | 7 | -157.651 | 329.301 | 0.605 | 0.147 |
| -0.64 | 1.61E-09 | NA | + | NA | NA | 2.15E-01 | 7 | -157.929 | 329.858 | 1.161 | 0.112 |
| -0.733 | 1.64E-09 | NA | + | -1.35E-03 | NA | 2.19E-01 | 8 | -157.186 | 330.373 | 1.676 | 0.086 |
| -0.551 | 1.43E-09 | NA | + | -1.60E-03 | -8.81E-02 | 2.19E-01 | 9 | -156.291 | 330.582 | 1.886 | 0.078 |
| -0.477 | 1.43E-09 | NA | + | NA | -7.22E-02 | 2.15E-01 | 8 | -157.317 | 330.635 | 1.938 | 0.076 |
| -0.586 | NA | NA | + | NA | NA | 1.97E-01 | 6 | -166.483 | 344.965 | 0 | 0.423 |
| -0.622 | 1.32E-09 | NA | + | NA | NA | 2.03E-01 | 7 | -166.229 | 346.458 | 1.492 | 0.201 |
| -0.645 | NA | NA | + | -7.76E-04 | NA | 2.01E-01 | 7 | -166.286 | 346.573 | 1.608 | 0.189 |
| -0.491 | NA | NA | + | NA | -4.32E-02 | 1.98E-01 | 7 | -166.3 | 346.6 | 1.635 | 0.187 |
| -0.497 | NA | NA | + | NA | NA | 1.78E-01 | 6 | -156.305 | 324.61 | 0 | 0.447 |
| -0.531 | 1.14E-09 | NA | + | NA | NA | 1.85E-01 | 7 | -156.073 | 326.145 | 1.535 | 0.208 |
| -0.533 | NA | NA | + | -4.37E-04 | NA | 1.81E-01 | 7 | -156.243 | 326.486 | 1.876 | 0.175 |
| -0.458 | NA | NA | + | NA | -1.82E-02 | 1.79E-01 | 7 | -156.272 | 326.544 | 1.934 | 0.17 |
| -0.566 | NA | NA | + | NA | NA | 1.93E-01 | 6 | -157.315 | 326.63 | 0 | 0.392 |
| -0.641 | NA | NA | + | -9.94E-04 | NA | 1.97E-01 | 7 | -156.963 | 327.926 | 1.296 | 0.205 |
| -0.608 | 1.49E-09 | NA | + | NA | NA | 2.00E-01 | 7 | -156.973 | 327.946 | 1.316 | 0.203 |
| -0.446 | NA | NA | + | NA | -5.37E-02 | 1.94E-01 | 7 | -156.989 | 327.978 | 1.348 | 0.2 |
| -0.426 | NA | NA | + | NA | NA | 1.58E-01 | 6 | -153.266 | 318.532 | 0 | 0.452 |
| -0.458 | 1.08E-09 | NA | + | NA | NA | 1.64E-01 | 7 | -153.063 | 320.126 | 1.594 | 0.204 |
| -0.458 | NA | NA | + | -3.74E-04 | NA | 1.61E-01 | 7 | -153.221 | 320.442 | I.91 | 0.174 |
| -0.388 | NA | NA | + | NA | -1.71E-02 | 1.59E-01 | 7 | -153.237 | 320.475 | 1.942 | 0.171 |
| -0.582 | NA | NA | + | NA | NA | 2.04E-01 | 6 | -158.093 | 328.185 | 0 | 0.404 |
| -0.626 | 1.56E-09 | NA | + | NA | NA | 2.11E-01 | 7 | -157.742 | 329.485 | 1.299 | 0.211 |
| -0.647 | NA | NA | + | -8.98E-04 | NA | 2.08E-01 | 7 | -157.794 | 329.587 | 1.402 | 0.201 |
| -0.487 | NA | NA | + | NA | -4.43E-02 | 2.05E-01 | 7 | -157.882 | 329.764 | 1.579 | 0.184 |
| -0.16 | NA | NA | NA | 1.38E-03 | NA | 1.39E-01 | 4 | -153.326 | 314.652 | 0 | 0.255 |
| -0.256 | NA | NA | NA | NA | NA | 1.67E-01 | 3 | -154.4 | 314.8 | 0.147 | 0.237 |
| -0.314 | NA | NA | + | NA | NA | 1.31E-01 | 6 | -151.555 | 315.11 | 0.458 | 0.203 |
| -0.229 | NA | NA | NA | 1.54E-03 | 3.93E-02 | 1.37E-01 | 5 | -153.19 | 316.381 | 1.729 | 0.107 |
| -0.181 | 7.74E-10 | NA | NA | 1.37E-03 | NA | 1.43E-01 | 5 | -153.241 | 316.482 | I.83 | 0.102 |
| -0.278 | 8.24E-10 | NA | NA | NA | NA | 1.70E-01 | 4 | -154.305 | 316.609 | 1.957 | 0.096 |
| -0.363 | NA | NA | + | NA | NA | 1.42E-01 | 6 | -141.368 | 294.736 | 0 | 0.297 |
| -0.288 | NA | NA | NA | NA | NA | 1.71E-01 | 3 | -144.839 | 295.678 | 0.942 | 0.185 |
| -0.212 | NA | NA | NA | 1.23E-03 | NA | 1.51E-01 | 4 | -143.919 | 295.838 | 1.102 | 0.171 |
| -0.389 | 9.03E-10 | NA | + | NA | NA | 1.46E-01 | 7 | -141.221 | 296.443 | 1.707 | 0.126 |
| -0.338 | NA | NA | + | NA | -1.17E-02 | 1.42E-01 | 7 | -141.354 | 296.707 | 1.971 | 0.111 |
| -0.374 | NA | NA | + | -1.28E-04 | NA | 1.42E-01 | 7 | -141.363 | 296.726 | 1.989 | 0.11 |
| -0.436 | NA | NA | + | NA | NA | 1.58E-01 | 6 | -142.886 | 297.772 | 0 | 0.436 |
| -0.471 | 1.33E-09 | NA | + | NA | NA | 1.64E-01 | 7 | -142.633 | 299.266 | 1.494 | 0.207 |
| -0.359 | NA | NA | + | NA | -3.52E-02 | 1.60E-01 | 7 | -142.741 | 299.481 | 1.709 | 0.186 |
| -0.472 | NA | NA | + | -4.31E-04 | NA | 1.61E-01 | 7 | -142.819 | 299.638 | 1.866 | 0.172 |
| -0.379 | NA | NA | + | NA | NA | 1.45E-01 | 6 | -137.615 | 287.229 | 0 | 0.417 |
| -0.265 | NA | NA | + | NA | -5.30E-02 | 1.47E-01 | 7 | -137.279 | 288.558 | 1.329 | 0.215 |
| -0.406 | 8.95E-10 | NA | + | NA | NA | 1.50E-01 | 7 | -137.431 | 288.862 | 1.633 | 0.184 |
| -0.435 | NA | NA | + | -6.78E-04 | NA | 1.49E-01 | 7 | -137.436 | 288.872 | 1.643 | 0.184 |
| -0.585 | NA | NA | + | NA | NA | 2.00E-01 | 6 | -158.998 | 329.996 | 0 | 0.391 |
| -0.659 | NA | NA | + | -1.09E-03 | NA | 2.03E-01 | 7 | -158.542 | 331.084 | 1.088 | 0.227 |
| -0.624 | 1.38E-09 | NA | + | NA | NA | 2.07E-01 | 7 | -158.698 | 331.396 | 01.IV | 0.194 |
| -0.475 | NA | NA | + | NA | -4.91E-02 | 2.01E-01 | 7 | -158.728 | 331.456 | I.46 | 0.188 |
| -0.478 | NA | NA | + | NA | NA | 1.69E-01 | 6 | -151.338 | 314.676 | 0 | 0.421 |
| -0.511 | 8.38E-10 | NA | + | NA | NA | 1.77E-01 | 7 | -151.103 | 316.205 | 1.529 | 0.196 |
| -0.541 | NA | NA | + | -7.99E-04 | NA | 1.73E-01 | 7 | -151.112 | 316.224 | 1.548 | 0.194 |
| -0.385 | NA | NA | + | NA | -4.31E-02 | 1.71E-01 | 7 | -151.138 | 316.276 | 1.599 | 0.189 |
| -0.386 | NA | NA | + | NA | NA | 1.47E-01 | 6 | -152.748 | 317.496 | 0 | 0.283 |
| -0.21 | NA | NA | NA | 1.48E-03 | NA | 1.54E-01 | 4 | -155.03 | 318.06 | 0.564 | 0.213 |
| -0.297 | NA | NA | NA | NA | NA | 1.78E-01 | 3 | -156.27 | 318.541 | 1.044 | 0.168 |
| -0.414 | 8.82E-10 | NA | + | NA | NA | 1.52E-01 | 7 | -152.583 | 319.165 | 1.669 | 0.123 |
| -0.361 | NA | NA | + | 2.98E-04 | NA | 1.45E-01 | 7 | -152.721 | 319.441 | 1.945 | 0.107 |
| -0.356 | NA | NA | + | NA | -1.40E-02 | 1.48E-01 | 7 | -152.729 | 319.458 | 1.962 | 0.106 |
| -0.508 | NA | NA | + | NA | NA | 1.87E-01 | 6 | -169.329 | 350.658 | 0 | 0.452 |
| -0.545 | 1.36E-09 | NA | + | NA | NA | 1.93E-01 | 7 | -169.092 | 352.184 | 1.525 | 0.211 |
| -0.479 | NA | NA | + | NA | -1.37E-02 | 1.88E-01 | 7 | -169.312 | 352.624 | 1.965 | 0.169 |
| -0.523 | NA | NA | + | -1.89E-04 | NA | 1.88E-01 | 7 | -169.318 | 352.636 | 1.977 | 0.168 |
| -0.66 | NA | NA | + | NA | NA | 2.16E-01 | 6 | -161.169 | 334.339 | 0 | 0.193 |
| -0.475 | NA | NA | + | NA | -7.97E-02 | 2.15E-01 | 7 | -160.416 | 334.833 | 0.494 | 0.151 |
| -0.546 | NA | NA | + | -1.57E-03 | -9.49E-02 | 2.20E-01 | 8 | -159.47 | 334.941 | 0.602 | 0.143 |
| -0.747 | NA | NA | + | -1.29E-03 | NA | 2.21E-01 | 7 | -160.519 | 335.037 | 0.699 | 0.136 |
| -0.717 | 2.09E-09 | NA | + | NA | NA | 2.25E-01 | 7 | -160.609 | 335.218 | 0.88 | 0.124 |
| -0.805 | 2.09E-09 | NA | + | -1.29E-03 | NA | 2.29E-01 | 8 | -159.956 | 335.911 | 1.573 | 0.088 |
| -0.541 | 1.85E-09 | NA | + | NA | -7.31E-02 | 2.23E-01 | 8 | -159.979 | 335.958 | I.62 | 0.086 |
| -0.609 | 1.79E-09 | NA | + | -1.55E-03 | -8.83E-02 | 2.28E-01 | 9 | -159.054 | 336.107 | 1.769 | 0.08 |
| -0.438 | NA | NA | + | NA | NA | 1.61E-01 | 6 | -155.168 | 322.336 | 0 | 0.454 |
| -0.471 | 9.57E-10 | NA | + | NA | NA | 1.67E-01 | 7 | -154.956 | 323.913 | 1.576 | 0.206 |
| -0.464 | NA | NA | + | -3.14E-04 | NA | 1.62E-01 | 7 | -155.137 | 324.273 | 1.937 | 0.172 |
| -0.447 | NA | NA | + | NA | 4.20E-03 | 1.61E-01 | 7 | -155.166 | 324.333 | 1.997 | 0.167 |
| -0.56 | NA | NA | + | NA | NA | 1.90E-01 | 6 | -180.003 | 372.005 | 0 | 0.452 |
| -0.602 | 1.59E-09 | NA | + | NA | NA | 1.96E-01 | 7 | -179.754 | 373.508 | 1.502 | 0.213 |
| -0.541 | NA | NA | + | 2.48E-04 | NA | 1.89E-01 | 7 | -179.985 | 373.97 | 1.965 | 0.169 |
| -0.552 | NA | NA | + | NA | -3.29E-03 | 1.90E-01 | 7 | -180.002 | 374.003 | 1.998 | 0.166 |
| -0.519 | NA | NA | + | NA | NA | 1.80E-01 | 6 | -152.994 | 317.988 | 0 | 0.423 |
| -0.411 | NA | NA | + | NA | -4.86E-02 | 1.81E-01 | 7 | -152.742 | 319.485 | 1.497 | 0.2 |
| -0.551 | 1.13E-09 | NA | + | NA | NA | 1.86E-01 | 7 | -152.788 | 319.575 | 1.588 | 0.191 |
| -0.577 | NA | NA | + | -7.13E-04 | NA | 1.85E-01 | 7 | -152.813 | 319.626 | 1.638 | 0.186 |
| -0.58 | NA | NA | + | -1.94E-03 | -1.07E-01 | 2.33E-01 | 8 | -154.089 | 324.178 | 0 | 0.263 |
| -0.803 | NA | NA | + | -1.60E-03 | NA | 2.33E-01 | 7 | -155.508 | 325.016 | 0.838 | 0.173 |
| -0.696 | NA | NA | + | NA | NA | 2.27E-01 | 6 | -156.579 | 325.159 | 0.981 | 0.161 |
| -0.496 | NA | NA | + | NA | -8.72E-02 | 2.26E-01 | 7 | -155.625 | 325.25 | 1.072 | 0.154 |
| -0.634 | 1.35E-09 | NA | + | -1.95E-03 | -1.02E-01 | 2.42E-01 | 9 | -153.705 | 325.409 | 1.231 | 0.142 |
| -0.854 | 1.57E-09 | NA | + | -1.64E-03 | NA | 2.43E-01 | 8 | -154.994 | 325.989 | 1.811 | 0.106 |
| -0.437 | NA | NA | + | NA | NA | 1.61E-01 | 6 | -154.965 | 321.93 | 0 | 0.449 |
| -0.469 | 9.44E-10 | NA | + | NA | NA | 1.67E-01 | 7 | -154.767 | 323.533 | 1.603 | 0.202 |
| -0.483 | NA | NA | + | -5.73E-04 | NA | 1.64E-01 | 7 | -154.864 | 323.727 | 1.797 | 0.183 |
| -0.451 | NA | NA | + | NA | 6.60E-03 | 1.61E-01 | 7 | -154.961 | 323.921 | 1.992 | 0.166 |
| -0.441 | NA | NA | + | -1.85E-03 | -1.02E-01 | 1.95E-01 | 8 | -141.334 | 298.669 | 0 | 0.225 |
| -0.642 | NA | NA | + | -1.53E-03 | NA | 1.93E-01 | 7 | -142.629 | 299.258 | 0.59 | 0.167 |
| -0.53 | NA | NA | + | NA | NA | 1.86E-01 | 6 | -143.634 | 299.268 | 0.599 | 0.166 |
| -0.349 | NA | NA | + | NA | -8.18E-02 | 1.87E-01 | 7 | -142.782 | 299.564 | 0.895 | 0.144 |
| -0.487 | 1.29E-09 | NA | + | -1.84E-03 | -9.79E-02 | 2.01E-01 | 9 | -141.019 | 300.039 | I.37 | 0.113 |
| -0.686 | 1.49E-09 | NA | + | -1.53E-03 | NA | 1.99E-01 | 8 | -142.215 | 300.43 | 1.761 | 0.093 |
| -0.573 | 1.49E-09 | NA | + | NA | NA | 1.93E-01 | 7 | -143.228 | 300.457 | 1.788 | 0.092 |
| -0.627 | NA | NA | + | NA | NA | 2.13E-01 | 6 | -161.563 | 335.126 | 0 | 0.325 |
| -0.705 | NA | NA | + | -1.15E-03 | NA | 2.17E-01 | 7 | -161.06 | 336.121 | 0.995 | 0.197 |
| -0.676 | 1.76E-09 | NA | + | NA | NA | 2.21E-01 | 7 | -161.161 | 336.322 | 1.196 | 0.179 |
| -0.5 | NA | NA | + | NA | -5.74E-02 | 2.13E-01 | 7 | -161.191 | 336.381 | 1.255 | 0.173 |
| -0.562 | NA | NA | + | -1.36E-03 | -7.08E-02 | 2.17E-01 | 8 | -160.506 | 337.012 | 1.886 | 0.126 |
| -0.485 | NA | NA | + | NA | NA | 1.75E-01 | 6 | -148.485 | 308.97 | 0 | 0.423 |
| -0.553 | NA | NA | + | -8.89E-04 | NA | 1.78E-01 | 7 | -148.199 | 310.397 | 1.428 | 0.207 |
| -0.52 | 1.26E-09 | NA | + | NA | NA | 1.80E-01 | 7 | -148.209 | 310.418 | 1.448 | 0.205 |
| -0.433 | NA | NA | + | NA | -2.42E-02 | 1.76E-01 | 7 | -148.423 | 310.845 | 1.876 | 0.165 |
| -0.336 | NA | NA | + | NA | NA | 1.37E-01 | 6 | -147.651 | 307.301 | 0 | 0.212 |
| -0.201 | NA | NA | NA | 1.34E-03 | NA | 1.47E-01 | 4 | -149.773 | 307.545 | 0.244 | 0.188 |
| -0.289 | NA | NA | NA | NA | NA | 1.72E-01 | 3 | -150.823 | 307.646 | 0.345 | 0.178 |
| -0.368 | 6.88E-10 | NA | + | NA | NA | 1.45E-01 | 7 | -147.446 | 308.892 | I.59 | 0.096 |
| -0.326 | 7.96E-10 | NA | NA | NA | NA | 1.81E-01 | 4 | -150.559 | 309.119 | 1.818 | 0.085 |
| -0.235 | 6.58E-10 | NA | NA | 1.29E-03 | NA | 1.56E-01 | 5 | -149.592 | 309.184 | 1.882 | 0.083 |
| -0.317 | NA | NA | + | 2.39E-04 | NA | 1.35E-01 | 7 | -147.632 | 309.264 | 1.963 | 0.079 |
| -0.317 | NA | NA | + | NA | -8.95E-03 | 1.37E-01 | 7 | -147.643 | 309.286 | 1.984 | 0.079 |
| -0.496 | NA | NA | + | NA | NA | 1.77E-01 | 6 | -147.728 | 307.455 | 0 | 0.426 |
| -0.53 | 1.18E-09 | NA | + | NA | NA | 1.83E-01 | 7 | -147.483 | 308.966 | 1.511 | 0.2 |
| -0.556 | NA | NA | + | -7.68E-04 | NA | 1.80E-01 | 7 | -147.506 | 309.012 | 1.556 | 0.196 |
| -0.424 | NA | NA | + | NA | -3.32E-02 | 1.78E-01 | 7 | -147.605 | 309.21 | 1.754 | 0.177 |
| -0.564 | NA | NA | + | NA | NA | 1.98E-01 | 6 | -162.103 | 336.205 | 0 | 0.432 |
| -0.605 | 1.40E-09 | NA | + | NA | NA | 2.05E-01 | 7 | -161.789 | 337.578 | 1.373 | 0.217 |
| -0.487 | NA | NA | + | NA | -3.45E-02 | 1.98E-01 | 7 | -161.979 | 337.958 | 1.752 | 0.18 |
| -0.597 | NA | NA | + | -4.58E-04 | NA | 2.00E-01 | 7 | -162.029 | 338.057 | 1.852 | 0.171 |
| -0.186 | NA | NA | NA | 1.62E-03 | NA | 1.45E-01 | 4 | -164.846 | 337.693 | 0 | 0.314 |
| -0.29 | NA | NA | NA | NA | NA | 1.75E-01 | 3 | -166.194 | 338.387 | 0.694 | 0.222 |
| -0.36 | NA | NA | + | NA | NA | 1.40E-01 | 6 | -163.243 | 338.487 | 0.794 | 0.211 |
| -0.215 | 9.58E-10 | NA | NA | 1.60E-03 | NA | 1.50E-01 | 5 | -164.712 | 339.424 | 1.731 | 0.132 |
| -0.224 | NA | NA | NA | 1.71E-03 | 2.12E-02 | 1.44E-01 | 5 | -164.809 | 339.618 | 1.926 | 0.12 |
| -0.534 | NA | NA | + | NA | NA | 1.85E-01 | 6 | -152.744 | 317.487 | 0 | 0.318 |
| -0.373 | NA | NA | + | NA | -7.22E-02 | 1.87E-01 | 7 | -152.088 | 318.176 | 0.689 | 0.225 |
| -0.573 | 1.26E-09 | NA | + | NA | NA | 1.92E-01 | 7 | -152.426 | 318.852 | 1.364 | 0.161 |
| -0.598 | NA | NA | + | -8.69E-04 | NA | 1.88E-01 | 7 | -152.439 | 318.878 | I.39 | 0.159 |
| -0.431 | NA | NA | + | -1.13E-03 | -8.35E-02 | 1.91E-01 | 8 | -151.585 | 319.171 | 1.683 | 0.137 |
| -0.529 | NA | NA | + | NA | NA | 1.85E-01 | 6 | -160.505 | 333.01 | 0 | 0.442 |
| -0.567 | 1.21E-09 | NA | + | NA | NA | 1.92E-01 | 7 | -160.251 | 334.502 | 1.492 | 0.21 |
| -0.463 | NA | NA | + | NA | -2.92E-02 | 1.85E-01 | 7 | -160.419 | 334.838 | 1.829 | 0.177 |
| -0.561 | NA | NA | + | -4.01E-04 | NA | 1.88E-01 | 7 | -160.454 | 334.908 | 1.898 | 0.171 |
| -0.522 | NA | NA | + | NA | NA | 1.85E-01 | 6 | -156.485 | 324.97 | 0 | 0.426 |
| -0.589 | NA | NA | + | -9.15E-04 | NA | 1.89E-01 | 7 | -156.189 | 326.378 | 1.408 | 0.211 |
| -0.558 | 1.27E-09 | NA | + | NA | NA | 1.91E-01 | 7 | -156.232 | 326.463 | 1.493 | 0.202 |
| -0.483 | NA | NA | + | NA | -1.84E-02 | 1.86E-01 | 7 | -156.45 | 326.901 | 1.931 | 0.162 |
| -0.464 | NA | NA | + | NA | NA | 1.69E-01 | 6 | -149.185 | 310.37 | 0 | 0.428 |
| -0.5 | 1.10E-09 | NA | + | NA | NA | 1.76E-01 | 7 | -148.885 | 311.77 | 1.401 | 0.212 |
| -0.382 | NA | NA | + | NA | -3.75E-02 | 1.70E-01 | 7 | -149.028 | 312.056 | 1.686 | 0.184 |
| -0.509 | NA | NA | + | -5.68E-04 | NA | 1.71E-01 | 7 | -149.073 | 312.146 | 1.776 | 0.176 |
| -0.447 | NA | NA | + | NA | NA | 1.60E-01 | 6 | -147.813 | 307.626 | 0 | 0.434 |
| -0.479 | 9.56E-10 | NA | + | NA | NA | 1.67E-01 | 7 | -147.58 | 309.159 | 1.533 | 0.201 |
| -0.351 | NA | NA | + | NA | -4.34E-02 | 1.62E-01 | 7 | -147.605 | 309.21 | 1.584 | 0.196 |
| -0.48 | NA | NA | + | -3.94E-04 | NA | 1.62E-01 | 7 | -147.757 | 309.514 | 1.888 | 0.169 |
| -0.612 | NA | NA | + | NA | NA | 2.10E-01 | 6 | -156.643 | 325.286 | 0 | 0.246 |
| -0.418 | NA | NA | + | NA | -8.61E-02 | 2.09E-01 | 7 | -155.78 | 325.56 | 0.274 | 0.214 |
| -0.474 | NA | NA | + | -1.29E-03 | -1.01E-01 | 2.15E-01 | 8 | -155.126 | 326.251 | 0.965 | 0.152 |
| -0.658 | 1.47E-09 | NA | + | NA | NA | 2.18E-01 | 7 | -156.188 | 326.376 | 01.IX | 0.142 |
| -0.678 | NA | NA | + | -9.53E-04 | NA | 2.14E-01 | 7 | -156.274 | 326.549 | 1.262 | 0.131 |
| -0.469 | 1.35E-09 | NA | + | NA | -8.24E-02 | 2.17E-01 | 8 | -155.397 | 326.793 | 1.507 | 0.116 |
| -0.529 | NA | NA | + | NA | NA | 1.82E-01 | 6 | -153.03 | 318.06 | 0 | 0.398 |
| -0.612 | NA | NA | + | -1.17E-03 | NA | 1.85E-01 | 7 | -152.534 | 319.069 | 1.009 | 0.24 |
| -0.569 | 1.37E-09 | NA | + | NA | NA | 1.88E-01 | 7 | -152.733 | 319.466 | 1.406 | 0.197 |
| -0.456 | NA | NA | + | NA | -3.31E-02 | 1.82E-01 | 7 | -152.912 | 319.824 | 1.764 | 0.165 |
| -0.552 | NA | NA | + | NA | NA | 1.83E-01 | 6 | -157.651 | 327.301 | 0 | 0.4 |
| -0.642 | NA | NA | + | -1.20E-03 | NA | 1.88E-01 | 7 | -157.142 | 328.285 | 0.983 | 0.244 |
| -0.588 | 1.42E-09 | NA | + | NA | NA | 1.88E-01 | 7 | -157.408 | 328.817 | 1.515 | 0.187 |
| -0.472 | NA | NA | + | NA | -3.67E-02 | 1.85E-01 | 7 | -157.515 | 329.031 | I.73 | 0.168 |
| -0.447 | NA | NA | + | NA | NA | 1.57E-01 | 6 | -160.836 | 333.672 | 0 | 0.451 |
| -0.477 | 9.95E-10 | NA | + | NA | NA | 1.62E-01 | 7 | -160.666 | 335.332 | I.66 | 0.197 |
| -0.492 | NA | NA | + | -5.51E-04 | NA | 1.60E-01 | 7 | -160.741 | 335.482 | I.81 | 0.182 |
| -0.409 | NA | NA | + | NA | -1.71E-02 | 1.57E-01 | 7 | -160.81 | 335.62 | 1.947 | 0.17 |
| -0.547 | NA | NA | + | NA | NA | 1.87E-01 | 6 | -150.329 | 312.658 | 0 | 0.387 |
| -0.414 | NA | NA | + | NA | -5.95E-02 | 1.89E-01 | 7 | -149.915 | 313.83 | 1.172 | 0.215 |
| -0.623 | NA | NA | + | -1.01E-03 | NA | 1.92E-01 | 7 | -149.955 | 313.909 | 1.251 | 0.207 |
| -0.586 | 1.30E-09 | NA | + | NA | NA | 1.94E-01 | 7 | -150.035 | 314.07 | 1.412 | 0.191 |
| -0.507 | NA | NA | + | NA | NA | 1.73E-01 | 6 | -161.402 | 334.804 | 0 | 0.46 |
| -0.538 | 1.09E-09 | NA | + | NA | NA | 1.78E-01 | 7 | -161.241 | 336.481 | 1.677 | 0.199 |
| -0.527 | NA | NA | + | -2.59E-04 | NA | 1.75E-01 | 7 | -161.382 | 336.764 | I.96 | 0.172 |
| -0.515 | NA | NA | + | NA | 3.50E-03 | 1.73E-01 | 7 | -161.401 | 336.802 | 1.998 | 0.169 |
| -0.443 | NA | NA | + | NA | NA | 1.61E-01 | 6 | -136.844 | 285.688 | 0 | 0.401 |
| -0.322 | NA | NA | + | NA | -5.60E-02 | 1.64E-01 | 7 | -136.441 | 286.883 | 1.195 | 0.221 |
| -0.474 | 9.45E-10 | NA | + | NA | NA | 1.68E-01 | 7 | -136.583 | 287.166 | 1.479 | 0.191 |
| -0.505 | NA | NA | + | -7.80E-04 | NA | 1.65E-01 | 7 | -136.608 | 287.216 | 1.528 | 0.187 |
| -0.478 | NA | NA | + | NA | NA | 1.63E-01 | 6 | -153.56 | 319.121 | 0 | 0.445 |
| -0.508 | 9.15E-10 | NA | + | NA | NA | 1.70E-01 | 7 | -153.372 | 320.744 | 1.624 | 0.198 |
| -0.528 | NA | NA | + | -6.37E-04 | NA | 1.66E-01 | 7 | -153.424 | 320.848 | 1.727 | 0.188 |
| -0.438 | NA | NA | + | NA | -1.75E-02 | 1.63E-01 | 7 | -153.529 | 321.059 | 1.938 | 0.169 |
| -0.543 | NA | NA | + | NA | NA | 1.86E-01 | 6 | -158.453 | 328.906 | 0 | 0.439 |
| -0.581 | 1.33E-09 | NA | + | NA | NA | 1.93E-01 | 7 | -158.188 | 330.377 | 1.471 | 0.21 |
| -0.586 | NA | NA | + | -5.84E-04 | NA | 1.88E-01 | 7 | -158.333 | 330.666 | I.76 | 0.182 |
| -0.497 | NA | NA | + | NA | -2.03E-02 | 1.86E-01 | 7 | -158.413 | 330.825 | 1.919 | 0.168 |
| -0.475 | NA | NA | + | NA | NA | 1.71E-01 | 6 | -156.471 | 324.942 | 0 | 0.445 |
| -0.51 | 1.15E-09 | NA | + | NA | NA | 1.77E-01 | 7 | -156.227 | 326.454 | 1.512 | 0.209 |
| -0.521 | NA | NA | + | -5.81E-04 | NA | 1.73E-01 | 7 | -156.363 | 326.725 | 1.783 | 0.182 |
| -0.47 | NA | NA | + | NA | -2.07E-03 | 1.71E-01 | 7 | -156.471 | 326.941 | 1.999 | 0.164 |
| -0.418 | NA | NA | + | NA | NA | 1.61E-01 | 6 | -148.566 | 309.131 | 0 | 0.386 |
| -0.445 | 1.04E-09 | NA | + | NA | NA | 1.65E-01 | 7 | -148.409 | 310.817 | 1.686 | 0.166 |
| -0.353 | NA | NA | NA | NA | NA | 1.99E-01 | 3 | -152.458 | 310.916 | 1.785 | 0.158 |
| -0.447 | NA | NA | + | -3.45E-04 | NA | 1.63E-01 | 7 | -148.527 | 311.055 | 1.924 | 0.148 |
| -0.41 | NA | NA | + | NA | -3.58E-03 | 1.61E-01 | 7 | -148.564 | 311.129 | 1.997 | 0.142 |
| -0.486 | NA | NA | + | NA | NA | 1.70E-01 | 6 | -156.436 | 324.872 | 0 | 0.438 |
| -0.52 | 9.94E-10 | NA | + | NA | NA | 1.76E-01 | 7 | -156.205 | 326.41 | 1.538 | 0.203 |
| -0.551 | NA | NA | + | -7.90E-04 | NA | 1.73E-01 | 7 | -156.239 | 326.479 | 1.607 | 0.196 |
| -0.46 | NA | NA | + | NA | -1.22E-02 | 1.70E-01 | 7 | -156.422 | 326.843 | 1.971 | 0.163 |
| -0.57 | NA | NA | + | NA | NA | 1.98E-01 | 6 | -164.348 | 340.697 | 0 | 0.408 |
| -0.613 | 1.32E-09 | NA | + | NA | NA | 2.06E-01 | 7 | -163.987 | 341.974 | 1.277 | 0.216 |
| -0.443 | NA | NA | + | NA | -5.76E-02 | 1.99E-01 | 7 | -163.987 | 341.974 | 1.277 | 0.216 |
| -0.599 | NA | NA | + | -4.34E-04 | NA | 1.99E-01 | 7 | -164.28 | 342.56 | 1.863 | 0.161 |
| -0.741 | NA | NA | + | NA | NA | 2.46E-01 | 6 | -166.775 | 345.55 | 0 | 0.179 |
| -0.613 | NA | NA | + | -1.68E-03 | -1.01E-01 | 2.47E-01 | 8 | -164.842 | 345.683 | 0.133 | 0.168 |
| -0.545 | NA | NA | + | NA | -8.40E-02 | 2.43E-01 | 7 | -165.936 | 345.873 | 0.322 | 0.153 |
| -0.829 | NA | NA | + | -1.37E-03 | NA | 2.50E-01 | 7 | -166.036 | 346.071 | 0.521 | 0.138 |
| -0.795 | 1.95E-09 | NA | + | NA | NA | 2.56E-01 | 7 | -166.284 | 346.568 | 1.018 | 0.108 |
| -0.672 | 1.66E-09 | NA | + | -1.67E-03 | -9.51E-02 | 2.55E-01 | 9 | -164.48 | 346.959 | 1.409 | 0.089 |
| -0.884 | 1.97E-09 | NA | + | -1.38E-03 | NA | 2.60E-01 | 8 | -165.526 | 347.052 | 1.502 | 0.085 |
| -0.606 | 1.69E-09 | NA | + | NA | -7.78E-02 | 2.52E-01 | 8 | -165.568 | 347.137 | 1.587 | 0.081 |
| -0.424 | NA | NA | + | NA | NA | 1.57E-01 | 6 | -142.622 | 297.245 | 0 | 0.422 |
| -0.502 | NA | NA | + | -8.89E-04 | NA | 1.63E-01 | 7 | -142.335 | 298.669 | 1.425 | 0.207 |
| -0.333 | NA | NA | + | NA | -4.14E-02 | 1.58E-01 | 7 | -142.43 | 298.86 | 1.615 | 0.188 |
| -0.453 | 1.08E-09 | NA | + | NA | NA | 1.62E-01 | 7 | -142.452 | 298.905 | I.66 | 0.184 |
| -0.561 | NA | NA | + | NA | NA | 1.97E-01 | 6 | -148.412 | 308.824 | 0 | 0.39 |
| -0.634 | NA | NA | + | -1.01E-03 | NA | 2.01E-01 | 7 | -147.993 | 309.986 | 1.161 | 0.218 |
| -0.599 | 1.39E-09 | NA | + | NA | NA | 2.03E-01 | 7 | -148.086 | 310.172 | 1.348 | 0.199 |
| -0.449 | NA | NA | + | NA | -5.07E-02 | 1.97E-01 | 7 | -148.118 | 310.235 | 1.411 | 0.193 |
| -0.443 | NA | NA | + | NA | NA | 1.58E-01 | 6 | -136.652 | 285.303 | 0 | 0.305 |
| -0.555 | NA | NA | + | -1.36E-03 | NA | 1.65E-01 | 7 | -135.923 | 285.847 | 0.543 | 0.232 |
| -0.322 | NA | NA | + | NA | -5.59E-02 | 1.60E-01 | 7 | -136.271 | 286.542 | 1.239 | 0.164 |
| -0.417 | NA | NA | + | -1.57E-03 | -7.10E-02 | 1.70E-01 | 8 | -135.32 | 286.64 | 1.336 | 0.156 |
| -0.475 | 1.11E-09 | NA | + | NA | NA | 1.63E-01 | 7 | -136.411 | 286.822 | 1.519 | 0.143 |
| -0.575 | NA | NA | + | NA | NA | 1.94E-01 | 6 | -161.3 | 334.6 | 0 | 0.415 |
| -0.616 | 1.45E-09 | NA | + | NA | NA | 2.01E-01 | 7 | -161.011 | 336.021 | 1.421 | 0.204 |
| -0.466 | NA | NA | + | NA | -4.87E-02 | 1.94E-01 | 7 | -161.05 | 336.1 | 01.V | 0.196 |
| -0.629 | NA | NA | + | -7.43E-04 | NA | 1.97E-01 | 7 | -161.11 | 336.22 | I.62 | 0.185 |
| -0.49 | NA | NA | + | NA | NA | 1.76E-01 | 6 | -156.231 | 324.462 | 0 | 0.443 |
| -0.524 | 1.13E-09 | NA | + | NA | NA | 1.82E-01 | 7 | -156.018 | 326.035 | 1.573 | 0.202 |
| -0.428 | NA | NA | + | NA | -2.94E-02 | 1.77E-01 | 7 | -156.143 | 326.286 | 1.824 | 0.178 |
| -0.532 | NA | NA | + | -5.22E-04 | NA | 1.79E-01 | 7 | -156.143 | 326.286 | 1.824 | 0.178 |
| -0.597 | NA | NA | + | NA | NA | 2.02E-01 | 6 | -153.55 | 319.1 | 0 | 0.302 |
| -0.434 | NA | NA | + | NA | -7.15E-02 | 2.02E-01 | 7 | -152.95 | 319.899 | 0.799 | 0.203 |
| -0.674 | NA | NA | + | -1.06E-03 | NA | 2.07E-01 | 7 | -153.089 | 320.179 | 1.079 | 0.176 |
| -0.644 | 1.64E-09 | NA | + | NA | NA | 2.10E-01 | 7 | -153.156 | 320.311 | 1.211 | 0.165 |
| -0.496 | NA | NA | + | -1.35E-03 | -8.70E-02 | 2.08E-01 | 8 | -152.227 | 320.455 | 1.355 | 0.154 |
| -0.566 | NA | NA | + | -1.88E-03 | -1.33E-01 | 2.52E-01 | 8 | -158.473 | 332.946 | 0 | 0.454 |
| -0.635 | 1.81E-09 | NA | + | -1.86E-03 | -1.25E-01 | 2.61E-01 | 9 | -157.946 | 333.893 | 0.947 | 0.283 |
| -0.49 | NA | NA | + | NA | -1.14E-01 | 2.45E-01 | 7 | -160.018 | 334.035 | 1.089 | 0.263 |
| -0.672 | NA | NA | + | NA | NA | 2.24E-01 | 6 | -160.464 | 332.928 | 0 | 0.304 |
| -0.497 | NA | NA | + | NA | -7.49E-02 | 2.22E-01 | 7 | -159.831 | 333.663 | 0.735 | 0.211 |
| -0.722 | 1.81E-09 | NA | + | NA | NA | 2.32E-01 | 7 | -160.032 | 334.065 | 1.137 | 0.172 |
| -0.74 | NA | NA | + | -1.01E-03 | NA | 2.28E-01 | 7 | -160.065 | 334.129 | 1.201 | 0.167 |
| -0.551 | NA | NA | + | -1.29E-03 | -8.86E-02 | 2.27E-01 | 8 | -159.203 | 334.407 | 1.479 | 0.145 |
| -0.456 | NA | NA | + | NA | NA | 1.60E-01 | 6 | -156.483 | 324.966 | 0 | 0.445 |
| -0.491 | 1.17E-09 | NA | + | NA | NA | 1.66E-01 | 7 | -156.246 | 326.491 | 1.525 | 0.207 |
| -0.5 | NA | NA | + | -5.62E-04 | NA | 1.63E-01 | 7 | -156.38 | 326.759 | 1.793 | 0.181 |
| -0.425 | NA | NA | + | NA | -1.42E-02 | 1.60E-01 | 7 | -156.463 | 326.926 | 1.961 | 0.167 |
| -0.395 | NA | NA | + | NA | NA | 1.50E-01 | 6 | -146.816 | 305.632 | 0 | 0.441 |
| -0.428 | 1.07E-09 | NA | + | NA | NA | 1.56E-01 | 7 | -146.589 | 307.178 | 1.546 | 0.204 |
| -0.313 | NA | NA | + | NA | -3.74E-02 | 1.51E-01 | 7 | -146.663 | 307.325 | 1.693 | 0.189 |
| -0.416 | NA | NA | + | -2.38E-04 | NA | 1.52E-01 | 7 | -146.796 | 307.593 | 1.961 | 0.166 |
| -0.509 | NA | NA | + | -2.12E-03 | -1.32E-01 | 2.35E-01 | 8 | -151.206 | 318.412 | 0 | 0.622 |
| -0.566 | 1.56E-09 | NA | + | -2.09E-03 | -1.27E-01 | 2.44E-01 | 9 | -150.704 | 319.408 | 0.996 | 0.378 |
| -0.514 | NA | NA | + | NA | NA | 1.84E-01 | 6 | -146.19 | 304.379 | 0 | 0.329 |
| -0.373 | NA | NA | + | NA | -6.29E-02 | 1.84E-01 | 7 | -145.726 | 305.452 | 1.073 | 0.192 |
| -0.594 | NA | NA | + | -1.01E-03 | NA | 1.89E-01 | 7 | -145.797 | 305.593 | 1.214 | 0.179 |
| -0.556 | 1.54E-09 | NA | + | NA | NA | 1.90E-01 | 7 | -145.83 | 305.659 | I.28 | 0.173 |
| -0.444 | NA | NA | + | -1.25E-03 | -7.59E-02 | 1.91E-01 | 8 | -145.14 | 306.279 | 01.IX | 0.127 |
| -0.501 | NA | NA | + | NA | NA | 1.77E-01 | 6 | -147.706 | 307.412 | 0 | 0.394 |
| -0.366 | NA | NA | + | NA | -6.12E-02 | 1.79E-01 | 7 | -147.254 | 308.509 | 1.097 | 0.228 |
| -0.539 | 1.49E-09 | NA | + | NA | NA | 1.83E-01 | 7 | -147.41 | 308.82 | 1.408 | 0.195 |
| -0.565 | NA | NA | + | -7.89E-04 | NA | 1.82E-01 | 7 | -147.476 | 308.952 | I.54 | 0.183 |
| -0.563 | NA | NA | + | NA | NA | 1.98E-01 | 6 | -149.76 | 311.519 | 0 | 0.271 |
| -0.653 | NA | NA | + | -1.29E-03 | NA | 2.02E-01 | 7 | -149.06 | 312.12 | 0.601 | 0.201 |
| -0.441 | NA | NA | + | NA | -5.56E-02 | 1.99E-01 | 7 | -149.391 | 312.782 | 1.263 | 0.144 |
| -0.603 | 1.39E-09 | NA | + | NA | NA | 2.05E-01 | 7 | -149.41 | 312.82 | 01.III | 0.142 |
| -0.51 | NA | NA | + | -1.52E-03 | -7.27E-02 | 2.03E-01 | 8 | -148.447 | 312.894 | 1.374 | 0.136 |
| -0.694 | 1.40E-09 | NA | + | -1.29E-03 | NA | 2.09E-01 | 8 | -148.703 | 313.405 | 1.886 | 0.106 |
| -0.518 | NA | NA | + | NA | NA | 1.78E-01 | 6 | -157.009 | 326.017 | 0 | 0.423 |
| -0.556 | 1.36E-09 | NA | + | NA | NA | 1.84E-01 | 7 | -156.707 | 327.413 | 1.396 | 0.211 |
| -0.58 | NA | NA | + | -8.38E-04 | NA | 1.81E-01 | 7 | -156.77 | 327.54 | 1.522 | 0.198 |
| -0.458 | NA | NA | + | NA | -2.76E-02 | 1.79E-01 | 7 | -156.93 | 327.861 | 1.843 | 0.168 |
| -0.459 | NA | NA | + | NA | NA | 1.68E-01 | 6 | -148.974 | 309.948 | 0 | 0.418 |
| -0.535 | NA | NA | + | -9.56E-04 | NA | 1.71E-01 | 7 | -148.648 | 311.296 | 1.348 | 0.213 |
| -0.493 | 1.33E-09 | NA | + | NA | NA | 1.72E-01 | 7 | -148.731 | 311.463 | 1.515 | 0.196 |
| -0.393 | NA | NA | + | NA | -3.16E-02 | 1.70E-01 | 7 | -148.863 | 311.725 | 1.777 | 0.172 |
| -0.336 | NA | NA | + | NA | NA | 1.41E-01 | 6 | -136.651 | 285.302 | 0 | 0.31 |
| -0.314 | NA | NA | NA | NA | NA | 1.80E-01 | 3 | -140.24 | 286.481 | 1.179 | 0.172 |
| -0.225 | NA | NA | NA | 1.06E-03 | NA | 1.56E-01 | 4 | -139.407 | 286.813 | 1.511 | 0.145 |
| -0.36 | 9.67E-10 | NA | + | NA | NA | 1.45E-01 | 7 | -136.498 | 286.997 | 1.695 | 0.133 |
| -0.375 | NA | NA | + | -4.23E-04 | NA | 1.44E-01 | 7 | -136.588 | 287.175 | 1.873 | 0.121 |
| -0.293 | NA | NA | + | NA | -2.03E-02 | 1.42E-01 | 7 | -136.604 | 287.207 | 1.905 | 0.119 |
| -0.504 | NA | NA | + | NA | NA | 1.73E-01 | 6 | -156.741 | 325.481 | 0 | 0.437 |
| -0.541 | 1.14E-09 | NA | + | NA | NA | 1.80E-01 | 7 | -156.466 | 326.932 | 1.451 | 0.211 |
| -0.546 | NA | NA | + | -5.46E-04 | NA | 1.75E-01 | 7 | -156.64 | 327.28 | 1.799 | 0.178 |
| -0.441 | NA | NA | + | NA | -2.86E-02 | 1.74E-01 | 7 | -156.659 | 327.318 | 1.837 | 0.174 |
| -0.502 | NA | NA | + | NA | NA | 1.75E-01 | 6 | -159.624 | 331.247 | 0 | 0.442 |
| -0.542 | 1.37E-09 | NA | + | NA | NA | 1.81E-01 | 7 | -159.37 | 332.74 | 1.492 | 0.209 |
| -0.426 | NA | NA | + | NA | -3.43E-02 | 1.76E-01 | 7 | -159.501 | 333.003 | 1.755 | 0.184 |
| -0.52 | NA | NA | + | -2.30E-04 | NA | 1.76E-01 | 7 | -159.606 | 333.212 | 1.965 | 0.165 |
| -0.441 | NA | NA | + | NA | NA | 1.62E-01 | 6 | -139.915 | 291.831 | 0 | 0.405 |
| -0.317 | NA | NA | + | NA | -5.51E-02 | 1.64E-01 | 7 | -139.545 | 293.091 | I.26 | 0.216 |
| -0.472 | 1.00E-09 | NA | + | NA | NA | 1.68E-01 | 7 | -139.671 | 293.342 | 1.512 | 0.19 |
| -0.5 | NA | NA | + | -7.52E-04 | NA | 1.65E-01 | 7 | -139.682 | 293.365 | 1.534 | 0.188 |
| -0.587 | NA | NA | + | NA | NA | 1.97E-01 | 6 | -154.18 | 320.359 | 0 | 0.318 |
| -0.675 | NA | NA | + | -1.23E-03 | NA | 2.02E-01 | 7 | -153.613 | 321.227 | 0.867 | 0.206 |
| -0.453 | NA | NA | + | NA | -6.10E-02 | 1.99E-01 | 7 | -153.762 | 321.525 | 1.165 | 0.178 |
| -0.622 | 1.23E-09 | NA | + | NA | NA | 2.03E-01 | 7 | -153.898 | 321.795 | 1.436 | 0.155 |
| -0.526 | NA | NA | + | -1.47E-03 | -7.63E-02 | 2.05E-01 | 8 | -152.976 | 321.952 | 1.593 | 0.143 |
| -0.658 | NA | NA | + | NA | NA | 2.18E-01 | 6 | -154.09 | 320.18 | 0 | 0.181 |
| -0.46 | NA | NA | + | NA | -8.75E-02 | 2.18E-01 | 7 | -153.139 | 320.278 | 0.097 | 0.173 |
| -0.516 | NA | NA | + | -1.48E-03 | -1.04E-01 | 2.22E-01 | 8 | -152.165 | 320.33 | 0.149 | 0.168 |
| -0.731 | NA | NA | + | -1.17E-03 | NA | 2.20E-01 | 7 | -153.471 | 320.942 | 0.761 | 0.124 |
| -0.702 | 1.42E-09 | NA | + | NA | NA | 2.27E-01 | 7 | -153.651 | 321.303 | 1.123 | 0.103 |
| -0.509 | 1.25E-09 | NA | + | NA | -8.32E-02 | 2.26E-01 | 8 | -152.793 | 321.586 | 1.406 | 0.09 |
| -0.565 | 1.27E-09 | NA | + | -1.49E-03 | -9.95E-02 | 2.29E-01 | 9 | -151.805 | 321.61 | I.43 | 0.089 |
| -0.777 | 1.45E-09 | NA | + | -1.19E-03 | NA | 2.29E-01 | 8 | -153.004 | 322.008 | 1.828 | 0.073 |
| -0.386 | NA | NA | + | NA | NA | 1.51E-01 | 6 | -146.513 | 305.027 | 0 | 0.293 |
| -0.325 | NA | NA | NA | NA | NA | 1.87E-01 | 3 | -149.889 | 305.777 | 0.751 | 0.201 |
| -0.248 | NA | NA | NA | 1.21E-03 | NA | 1.66E-01 | 4 | -149.059 | 306.118 | 1.091 | 0.17 |
| -0.41 | 8.49E-10 | NA | + | NA | NA | 1.54E-01 | 7 | -146.401 | 306.801 | 1.775 | 0.121 |
| -0.378 | NA | NA | + | NA | -3.68E-03 | 1.51E-01 | 7 | -146.512 | 307.024 | 1.997 | 0.108 |
| -0.391 | NA | NA | + | -4.98E-05 | NA | 1.51E-01 | 7 | -146.512 | 307.025 | 1.998 | 0.108 |
| -0.412 | NA | NA | + | NA | NA | 1.53E-01 | 6 | -167.246 | 346.492 | 0 | 0.28 |
| -0.229 | NA | NA | NA | 1.62E-03 | NA | 1.64E-01 | 4 | -169.479 | 346.957 | 0.465 | 0.222 |
| -0.34 | NA | NA | NA | NA | NA | 1.98E-01 | 3 | -170.756 | 347.512 | 01.II | 0.168 |
| -0.441 | 1.07E-09 | NA | + | NA | NA | 1.58E-01 | 7 | -167.105 | 348.21 | 1.718 | 0.118 |
| -0.378 | NA | NA | + | 3.78E-04 | NA | 1.50E-01 | 7 | -167.208 | 348.417 | 1.925 | 0.107 |
| -0.454 | NA | NA | + | NA | 1.86E-02 | 1.52E-01 | 7 | -167.217 | 348.434 | 1.942 | 0.106 |
| -0.657 | NA | NA | + | NA | NA | 2.21E-01 | 6 | -159.362 | 330.724 | 0 | 0.314 |
| -0.501 | NA | NA | + | NA | -6.73E-02 | 2.20E-01 | 7 | -158.835 | 331.67 | 0.947 | 0.196 |
| -0.705 | 1.64E-09 | NA | + | NA | NA | 2.30E-01 | 7 | -158.933 | 331.865 | 1.141 | 0.178 |
| -0.729 | NA | NA | + | -1.03E-03 | NA | 2.26E-01 | 7 | -158.945 | 331.891 | 1.167 | 0.175 |
| -0.559 | NA | NA | + | -1.30E-03 | -8.18E-02 | 2.26E-01 | 8 | -158.191 | 332.381 | 1.658 | 0.137 |
| -0.56 | NA | NA | + | NA | NA | 1.93E-01 | 6 | -160.76 | 333.52 | 0 | 0.416 |
| -0.6 | 1.30E-09 | NA | + | NA | NA | 2.00E-01 | 7 | -160.454 | 334.907 | 1.387 | 0.208 |
| -0.442 | NA | NA | + | NA | -5.19E-02 | 1.92E-01 | 7 | -160.47 | 334.94 | I.42 | 0.205 |
| -0.603 | NA | NA | + | -5.58E-04 | NA | 1.96E-01 | 7 | -160.645 | 335.291 | 1.771 | 0.172 |
| -0.646 | NA | NA | + | NA | NA | 2.20E-01 | 6 | -155.864 | 323.728 | 0 | 0.326 |
| -0.73 | NA | NA | + | -1.22E-03 | NA | 2.25E-01 | 7 | -155.282 | 324.564 | 0.837 | 0.214 |
| -0.69 | 1.45E-09 | NA | + | NA | NA | 2.28E-01 | 7 | -155.502 | 325.004 | 1.276 | 0.172 |
| -0.53 | NA | NA | + | NA | -5.09E-02 | 2.19E-01 | 7 | -155.576 | 325.152 | 1.424 | 0.16 |
| -0.592 | NA | NA | + | -1.44E-03 | -6.72E-02 | 2.25E-01 | 8 | -154.794 | 325.587 | 1.859 | 0.128 |
| -0.617 | NA | NA | + | NA | NA | 2.10E-01 | 6 | -156.784 | 325.568 | 0 | 0.394 |
| -0.483 | NA | NA | + | NA | -5.94E-02 | 2.11E-01 | 7 | -156.364 | 326.727 | 1.159 | 0.221 |
| -0.66 | 1.61E-09 | NA | + | NA | NA | 2.17E-01 | 7 | -156.439 | 326.878 | I.31 | 0.205 |
| -0.668 | NA | NA | + | -7.45E-04 | NA | 2.13E-01 | 7 | -156.569 | 327.138 | 1.569 | 0.18 |
| -0.159 | NA | NA | NA | 1.20E-03 | NA | 1.33E-01 | 4 | -138.999 | 285.999 | 0 | 0.204 |
| -0.237 | NA | NA | NA | NA | NA | 1.57E-01 | 3 | -140.003 | 286.007 | 0.008 | 0.203 |
| -0.306 | NA | NA | + | NA | NA | 1.27E-01 | 6 | -137.088 | 286.177 | 0.178 | 0.187 |
| -0.163 | NA | NA | NA | NA | -3.56E-02 | 1.56E-01 | 4 | -139.853 | 287.706 | 1.707 | 0.087 |
| -0.26 | 8.27E-10 | NA | NA | NA | NA | 1.60E-01 | 4 | -139.887 | 287.774 | 1.775 | 0.084 |
| -0.181 | 7.86E-10 | NA | NA | 1.20E-03 | NA | 1.37E-01 | 5 | -138.893 | 287.786 | 1.787 | 0.084 |
| -0.327 | 7.61E-10 | NA | + | NA | NA | 1.30E-01 | 7 | -136.986 | 287.973 | 1.974 | 0.076 |
| -0.152 | NA | NA | NA | 1.19E-03 | -3.87E-03 | 1.33E-01 | 5 | -138.998 | 287.996 | 1.997 | 0.075 |
| -0.489 | NA | NA | + | NA | NA | 1.73E-01 | 6 | -163.451 | 338.903 | 0 | 0.447 |
| -0.526 | 1.34E-09 | NA | + | NA | NA | 1.79E-01 | 7 | -163.223 | 340.447 | 1.544 | 0.206 |
| -0.52 | NA | NA | + | -4.33E-04 | NA | 1.75E-01 | 7 | -163.391 | 340.782 | 1.879 | 0.175 |
| -0.441 | NA | NA | + | NA | -2.19E-02 | 1.73E-01 | 7 | -163.406 | 340.811 | 1.908 | 0.172 |
| -0.501 | NA | NA | + | NA | NA | 1.78E-01 | 6 | -148.774 | 309.547 | 0 | 0.394 |
| -0.59 | NA | NA | + | -1.16E-03 | NA | 1.83E-01 | 7 | -148.262 | 310.525 | 0.978 | 0.242 |
| -0.538 | 9.93E-10 | NA | + | NA | NA | 1.86E-01 | 7 | -148.489 | 310.979 | 1.432 | 0.193 |
| -0.416 | NA | NA | + | NA | -3.89E-02 | 1.78E-01 | 7 | -148.611 | 311.222 | 1.675 | 0.171 |
| -0.491 | NA | NA | + | NA | NA | 1.80E-01 | 6 | -155.747 | 323.494 | 0 | 0.437 |
| -0.529 | 1.20E-09 | NA | + | NA | NA | 1.87E-01 | 7 | -155.477 | 324.954 | I.46 | 0.21 |
| -0.534 | NA | NA | + | -5.68E-04 | NA | 1.82E-01 | 7 | -155.634 | 325.268 | 1.774 | 0.18 |
| -0.432 | NA | NA | + | NA | -2.68E-02 | 1.80E-01 | 7 | -155.673 | 325.346 | 1.852 | 0.173 |
| -0.458 | NA | NA | + | NA | NA | 1.72E-01 | 6 | -146.581 | 305.163 | 0 | 0.398 |
| -0.533 | NA | NA | + | -9.45E-04 | NA | 1.77E-01 | 7 | -146.243 | 306.487 | 1.324 | 0.205 |
| -0.342 | NA | NA | + | NA | -5.44E-02 | 1.74E-01 | 7 | -146.245 | 306.49 | 1.327 | 0.205 |
| -0.492 | 9.84E-10 | NA | + | NA | NA | 1.79E-01 | 7 | -146.313 | 306.625 | 1.462 | 0.192 |
| -0.436 | NA | NA | + | NA | NA | 1.64E-01 | 6 | -150.045 | 312.091 | 0 | 0.412 |
| -0.516 | NA | NA | + | -9.47E-04 | NA | 1.69E-01 | 7 | -149.727 | 313.453 | 1.362 | 0.209 |
| -0.337 | NA | NA | + | NA | -4.52E-02 | 1.64E-01 | 7 | -149.822 | 313.643 | 1.552 | 0.19 |
| -0.467 | 1.24E-09 | NA | + | NA | NA | 1.68E-01 | 7 | -149.826 | 313.652 | 1.561 | 0.189 |
| -0.463 | NA | NA | + | NA | NA | 1.62E-01 | 6 | -151.994 | 315.988 | 0 | 0.43 |
| -0.496 | 1.26E-09 | NA | + | NA | NA | 1.67E-01 | 7 | -151.766 | 317.533 | 1.545 | 0.198 |
| -0.53 | NA | NA | + | -8.12E-04 | NA | 1.67E-01 | 7 | -151.77 | 317.541 | 1.552 | 0.198 |
| -0.394 | NA | NA | + | NA | -3.09E-02 | 1.63E-01 | 7 | -151.896 | 317.792 | 1.804 | 0.174 |
| -0.618 | NA | NA | + | -2.33E-03 | -1.14E-01 | 2.32E-01 | 8 | -161.438 | 338.876 | 0 | 0.471 |
| -0.673 | 1.39E-09 | NA | + | -2.32E-03 | -1.10E-01 | 2.41E-01 | 9 | -160.986 | 339.972 | 1.095 | 0.272 |
| -0.856 | NA | NA | + | -1.99E-03 | NA | 2.31E-01 | 7 | -163.045 | 340.09 | 1.214 | 0.257 |
| -0.69 | NA | NA | + | NA | NA | 2.23E-01 | 6 | -165.935 | 343.869 | 0 | 0.265 |
| -0.773 | NA | NA | + | -1.25E-03 | NA | 2.27E-01 | 7 | -165.335 | 344.669 | 0.8 | 0.178 |
| -0.744 | 1.94E-09 | NA | + | NA | NA | 2.32E-01 | 7 | -165.445 | 344.89 | 1.021 | 0.159 |
| -0.536 | NA | NA | + | NA | -6.76E-02 | 2.24E-01 | 7 | -165.45 | 344.9 | 1.031 | 0.158 |
| -0.599 | NA | NA | + | -1.51E-03 | -8.41E-02 | 2.29E-01 | 8 | -164.603 | 345.206 | 1.337 | 0.136 |
| -0.824 | 1.88E-09 | NA | + | -1.22E-03 | NA | 2.35E-01 | 8 | -164.87 | 345.739 | I.87 | 0.104 |
| -0.437 | NA | NA | + | NA | NA | 1.64E-01 | 6 | -149.214 | 310.428 | 0 | 0.442 |
| -0.467 | 1.11E-09 | NA | + | NA | NA | 1.69E-01 | 7 | -149.017 | 312.034 | 1.606 | 0.198 |
| -0.497 | NA | NA | + | -7.65E-04 | NA | 1.68E-01 | 7 | -149.019 | 312.038 | I.61 | 0.198 |
| -0.43 | NA | NA | + | NA | -3.42E-03 | 1.64E-01 | 7 | -149.213 | 312.426 | 1.998 | 0.163 |
| -0.671 | NA | NA | + | NA | NA | 2.19E-01 | 6 | -163.526 | 339.052 | 0 | 0.269 |
| -0.755 | NA | NA | + | -1.36E-03 | NA | 2.21E-01 | 7 | -162.802 | 339.604 | 0.553 | 0.204 |
| -0.72 | 1.87E-09 | NA | + | NA | NA | 2.26E-01 | 7 | -163.134 | 340.268 | 1.216 | 0.146 |
| -0.543 | NA | NA | + | NA | -5.67E-02 | 2.19E-01 | 7 | -163.182 | 340.365 | 1.313 | 0.139 |
| -0.605 | NA | NA | + | -1.56E-03 | -7.23E-02 | 2.21E-01 | 8 | -162.252 | 340.505 | 1.453 | 0.13 |
| -0.804 | 1.87E-09 | NA | + | -1.36E-03 | NA | 2.28E-01 | 8 | -162.406 | 340.812 | I.76 | 0.111 |
| -0.488 | NA | NA | + | NA | NA | 1.73E-01 | 6 | -148.044 | 308.089 | 0 | 0.392 |
| -0.567 | NA | NA | + | -9.92E-04 | NA | 1.78E-01 | 7 | -147.67 | 309.34 | 1.251 | 0.209 |
| -0.362 | NA | NA | + | NA | -5.68E-02 | 1.74E-01 | 7 | -147.678 | 309.356 | 1.268 | 0.208 |
| -0.525 | 1.34E-09 | NA | + | NA | NA | 1.78E-01 | 7 | -147.761 | 309.523 | 1.434 | 0.191 |
| -0.458 | NA | NA | + | NA | NA | 1.65E-01 | 6 | -146.815 | 305.629 | 0 | 0.438 |
| -0.489 | 1.24E-09 | NA | + | NA | NA | 1.70E-01 | 7 | -146.616 | 307.232 | 1.602 | 0.197 |
| -0.516 | NA | NA | + | -7.00E-04 | NA | 1.69E-01 | 7 | -146.644 | 307.289 | I.66 | 0.191 |
| -0.401 | NA | NA | + | NA | -2.65E-02 | 1.67E-01 | 7 | -146.739 | 307.479 | 1.849 | 0.174 |
| -0.658 | NA | NA | + | -2.17E-03 | -1.45E-01 | 2.83E-01 | 8 | -163.13 | 342.259 | 0 | 0.615 |
| -0.737 | 2.25E-09 | NA | + | -2.13E-03 | -1.35E-01 | 2.92E-01 | 9 | -162.598 | 343.195 | 0.936 | 0.385 |
| -0.597 | NA | NA | + | NA | NA | 1.98E-01 | 6 | -152.96 | 317.919 | 0 | 0.259 |
| -0.697 | NA | NA | + | -1.35E-03 | NA | 2.05E-01 | 7 | -152.272 | 318.543 | 0.624 | 0.189 |
| -0.444 | NA | NA | + | NA | -6.61E-02 | 1.97E-01 | 7 | -152.44 | 318.881 | 0.961 | 0.16 |
| -0.527 | NA | NA | + | -1.60E-03 | -8.20E-02 | 2.05E-01 | 8 | -151.488 | 318.976 | 1.057 | 0.152 |
| -0.641 | 1.55E-09 | NA | + | NA | NA | 2.05E-01 | 7 | -152.589 | 319.178 | 1.258 | 0.138 |
| -0.742 | 1.57E-09 | NA | + | -1.35E-03 | NA | 2.12E-01 | 8 | -151.891 | 319.783 | 1.863 | 0.102 |
| -0.355 | NA | NA | + | NA | NA | 1.36E-01 | 6 | -146.027 | 304.054 | 0 | 0.336 |
| -0.379 | 7.49E-10 | NA | + | NA | NA | 1.40E-01 | 7 | -145.898 | 305.796 | 1.742 | 0.141 |
| -0.277 | NA | NA | NA | NA | NA | 1.70E-01 | 3 | -149.908 | 305.816 | 1.761 | 0.139 |
| -0.197 | NA | NA | NA | 1.25E-03 | NA | 1.47E-01 | 4 | -148.948 | 305.895 | 1.841 | 0.134 |
| -0.328 | NA | NA | + | NA | -1.24E-02 | 1.37E-01 | 7 | -146.011 | 306.023 | 1.968 | 0.125 |
| -0.373 | NA | NA | + | -2.06E-04 | NA | 1.37E-01 | 7 | -146.013 | 306.026 | 1.972 | 0.125 |
| -0.474 | NA | NA | + | NA | NA | 1.68E-01 | 6 | -162.734 | 337.469 | 0 | 0.457 |
| -0.508 | 1.25E-09 | NA | + | NA | NA | 1.74E-01 | 7 | -162.549 | 339.097 | 1.628 | 0.203 |
| -0.492 | NA | NA | + | -2.21E-04 | NA | 1.70E-01 | 7 | -162.72 | 339.439 | I.97 | 0.171 |
| -0.454 | NA | NA | + | NA | -9.19E-03 | 1.69E-01 | 7 | -162.726 | 339.453 | 1.984 | 0.17 |
| -0.541 | NA | NA | + | NA | NA | 1.86E-01 | 6 | -148.741 | 309.482 | 0 | 0.268 |
| -0.644 | NA | NA | + | -1.42E-03 | NA | 1.91E-01 | 7 | -147.962 | 309.924 | 0.442 | 0.215 |
| -0.58 | 9.91E-10 | NA | + | NA | NA | 1.95E-01 | 7 | -148.367 | 310.733 | 1.252 | 0.143 |
| -0.44 | NA | NA | + | NA | -4.71E-02 | 1.88E-01 | 7 | -148.484 | 310.968 | 1.486 | 0.127 |
| -0.523 | NA | NA | + | -1.60E-03 | -6.26E-02 | 1.95E-01 | 8 | -147.516 | 311.032 | 1.551 | 0.123 |
| -0.691 | 1.07E-09 | NA | + | -1.48E-03 | NA | 2.02E-01 | 8 | -147.519 | 311.039 | 1.557 | 0.123 |
| -0.662 | NA | NA | + | NA | NA | 2.19E-01 | 6 | -163.402 | 338.805 | 0 | 0.315 |
| -0.511 | NA | NA | + | NA | -6.54E-02 | 2.18E-01 | 7 | -162.914 | 339.827 | 1.022 | 0.189 |
| -0.74 | NA | NA | + | -1.14E-03 | NA | 2.23E-01 | 7 | -162.919 | 339.838 | 1.033 | 0.188 |
| -0.713 | 1.89E-09 | NA | + | NA | NA | 2.28E-01 | 7 | -163.017 | 340.034 | 1.229 | 0.17 |
| -0.575 | NA | NA | + | -1.38E-03 | -7.85E-02 | 2.23E-01 | 8 | -162.229 | 340.457 | 1.652 | 0.138 |
| -0.559 | NA | NA | + | NA | NA | 1.97E-01 | 6 | -153.52 | 319.039 | 0 | 0.27 |
| -0.659 | NA | NA | + | -1.31E-03 | NA | 2.03E-01 | 7 | -152.81 | 319.62 | 0.581 | 0.202 |
| -0.602 | 1.43E-09 | NA | + | NA | NA | 2.05E-01 | 7 | -153.158 | 320.316 | 1.277 | 0.143 |
| -0.437 | NA | NA | + | NA | -5.50E-02 | 1.98E-01 | 7 | -153.173 | 320.347 | 1.308 | 0.141 |
| -0.514 | NA | NA | + | -1.55E-03 | -7.31E-02 | 2.04E-01 | 8 | -152.214 | 320.428 | 1.389 | 0.135 |
| -0.704 | 1.46E-09 | NA | + | -1.33E-03 | NA | 2.10E-01 | 8 | -152.429 | 320.859 | I.82 | 0.109 |
| -0.199 | NA | NA | NA | 1.38E-03 | NA | 1.51E-01 | 4 | -153.208 | 314.417 | 0 | 0.225 |
| -0.288 | NA | NA | NA | NA | NA | 1.74E-01 | 3 | -154.263 | 314.527 | 0.11 | 0.213 |
| -0.359 | NA | NA | + | NA | NA | 1.46E-01 | 6 | -151.288 | 314.575 | 0.159 | 0.208 |
| -0.223 | 8.38E-10 | NA | NA | 1.38E-03 | NA | 1.55E-01 | 5 | -153.076 | 316.151 | 1.735 | 0.094 |
| -0.312 | 8.56E-10 | NA | NA | NA | NA | 1.78E-01 | 4 | -154.127 | 316.253 | 1.837 | 0.09 |
| -0.382 | 8.26E-10 | NA | + | NA | NA | 1.50E-01 | 7 | -151.156 | 316.312 | 1.896 | 0.087 |
| -0.222 | NA | NA | NA | 1.44E-03 | 1.31E-02 | 1.50E-01 | 5 | -153.193 | 316.385 | 1.969 | 0.084 |
| -0.185 | NA | NA | NA | 1.57E-03 | NA | 1.44E-01 | 4 | -160.863 | 329.726 | 0 | 0.314 |
| -0.279 | NA | NA | NA | NA | NA | 1.73E-01 | 3 | -162.114 | 330.228 | 0.502 | 0.245 |
| -0.357 | NA | NA | + | NA | NA | 1.39E-01 | 6 | -159.443 | 330.887 | 1.161 | 0.176 |
| -0.268 | NA | NA | NA | 1.77E-03 | 4.71E-02 | 1.41E-01 | 5 | -160.68 | 331.36 | 1.634 | 0.139 |
| -0.207 | 7.96E-10 | NA | NA | 1.57E-03 | NA | 1.48E-01 | 5 | -160.778 | 331.556 | I.83 | 0.126 |
| -0.384 | NA | NA | + | NA | NA | 1.50E-01 | 6 | -158.509 | 329.017 | 0 | 0.295 |
| -0.324 | NA | NA | NA | NA | NA | 1.89E-01 | 3 | -161.973 | 329.946 | 0.928 | 0.185 |
| -0.232 | NA | NA | NA | 1.36E-03 | NA | 1.62E-01 | 4 | -161.004 | 330.008 | 0.991 | 0.18 |
| -0.409 | 9.33E-10 | NA | + | NA | NA | 1.54E-01 | 7 | -158.393 | 330.785 | 1.768 | 0.122 |
| -0.375 | NA | NA | + | 1.06E-04 | NA | 1.49E-01 | 7 | -158.505 | 331.011 | 1.993 | 0.109 |
| -0.397 | NA | NA | + | NA | 6.00E-03 | 1.49E-01 | 7 | -158.505 | 331.011 | 1.993 | 0.109 |
| -0.585 | NA | NA | + | NA | NA | 2.01E-01 | 6 | -160.671 | 333.342 | 0 | 0.411 |
| -0.655 | NA | NA | + | -9.81E-04 | NA | 2.04E-01 | 7 | -160.318 | 334.635 | 1.293 | 0.215 |
| -0.625 | 1.41E-09 | NA | + | NA | NA | 2.07E-01 | 7 | -160.371 | 334.743 | 01.IV | 0.204 |
| -0.504 | NA | NA | + | NA | -3.54E-02 | 2.01E-01 | 7 | -160.549 | 335.099 | 1.756 | 0.171 |
| -0.435 | NA | NA | + | NA | NA | 1.60E-01 | 6 | -152.022 | 316.044 | 0 | 0.455 |
| -0.465 | 1.07E-09 | NA | + | NA | NA | 1.65E-01 | 7 | -151.841 | 317.682 | 1.638 | 0.2 |
| -0.392 | NA | NA | + | NA | -1.93E-02 | 1.61E-01 | 7 | -151.984 | 317.968 | 1.924 | 0.174 |
| -0.458 | NA | NA | + | -2.67E-04 | NA | 1.62E-01 | 7 | -151.998 | 317.996 | 1.952 | 0.171 |
| -0.424 | NA | NA | + | NA | NA | 1.59E-01 | 6 | -158.353 | 328.706 | 0 | 0.336 |
| -0.264 | NA | NA | NA | 1.43E-03 | NA | 1.73E-01 | 4 | -161.208 | 330.417 | I.71 | 0.143 |
| -0.451 | 9.75E-10 | NA | + | NA | NA | 1.63E-01 | 7 | -158.211 | 330.421 | 1.715 | 0.142 |
| -0.353 | NA | NA | NA | NA | NA | 1.96E-01 | 3 | -162.305 | 330.61 | 1.903 | 0.13 |
| -0.451 | NA | NA | + | NA | 1.24E-02 | 1.58E-01 | 7 | -158.339 | 330.679 | 1.972 | 0.125 |
| -0.415 | NA | NA | + | 1.04E-04 | NA | 1.58E-01 | 7 | -158.35 | 330.7 | 1.993 | 0.124 |
| -0.493 | NA | NA | + | -1.85E-03 | -1.06E-01 | 2.13E-01 | 8 | -150.747 | 317.494 | 0 | 0.231 |
| -0.6 | NA | NA | + | NA | NA | 2.05E-01 | 6 | -153.135 | 318.27 | 0.776 | 0.157 |
| -0.404 | NA | NA | + | NA | -8.74E-02 | 2.06E-01 | 7 | -152.156 | 318.313 | 0.819 | 0.154 |
| -0.708 | NA | NA | + | -1.52E-03 | NA | 2.11E-01 | 7 | -152.169 | 318.338 | 0.844 | 0.152 |
| -0.544 | 1.46E-09 | NA | + | -1.83E-03 | -1.01E-01 | 2.20E-01 | 9 | -150.391 | 318.782 | 1.288 | 0.122 |
| -0.649 | 1.73E-09 | NA | + | NA | NA | 2.14E-01 | 7 | -152.65 | 319.3 | 1.806 | 0.094 |
| -0.757 | 1.72E-09 | NA | + | -1.51E-03 | NA | 2.19E-01 | 8 | -151.683 | 319.365 | 1.871 | 0.091 |
| -0.472 | NA | NA | + | NA | NA | 1.76E-01 | 6 | -156.365 | 324.731 | 0 | 0.45 |
| -0.511 | 1.50E-09 | NA | + | NA | NA | 1.82E-01 | 7 | -156.09 | 326.179 | 1.448 | 0.218 |
| -0.483 | NA | NA | + | -1.40E-04 | NA | 1.77E-01 | 7 | -156.359 | 326.719 | 1.988 | 0.167 |
| -0.475 | NA | NA | + | NA | 1.21E-03 | 1.76E-01 | 7 | -156.365 | 326.731 | 2 | 0.166 |
| -0.443 | NA | NA | + | NA | NA | 1.71E-01 | 6 | -142.921 | 297.841 | 0 | 0.42 |
| -0.343 | NA | NA | + | NA | -4.68E-02 | 1.73E-01 | 7 | -142.656 | 299.312 | 1.471 | 0.201 |
| -0.473 | 9.79E-10 | NA | + | NA | NA | 1.78E-01 | 7 | -142.705 | 299.409 | 1.568 | 0.192 |
| -0.502 | NA | NA | + | -7.19E-04 | NA | 1.76E-01 | 7 | -142.732 | 299.465 | 1.624 | 0.187 |
| -0.482 | NA | NA | + | NA | NA | 1.70E-01 | 6 | -140.18 | 292.36 | 0 | 0.3 |
| -0.574 | NA | NA | + | -1.21E-03 | NA | 1.73E-01 | 7 | -139.576 | 293.152 | 0.791 | 0.202 |
| -0.341 | NA | NA | + | NA | -6.33E-02 | 1.71E-01 | 7 | -139.655 | 293.31 | 0.949 | 0.187 |
| -0.421 | NA | NA | + | -1.44E-03 | -7.74E-02 | 1.77E-01 | 8 | -138.807 | 293.614 | 1.254 | 0.16 |
| -0.52 | 1.25E-09 | NA | + | NA | NA | 1.76E-01 | 7 | -139.867 | 293.734 | 1.374 | 0.151 |
| -0.407 | NA | NA | + | NA | NA | 1.55E-01 | 6 | -152.186 | 316.372 | 0 | 0.32 |
| -0.341 | NA | NA | NA | NA | NA | 1.91E-01 | 3 | -155.913 | 317.826 | 1.453 | 0.155 |
| -0.26 | NA | NA | NA | 1.27E-03 | NA | 1.69E-01 | 4 | -154.984 | 317.968 | 1.596 | 0.144 |
| -0.437 | 1.07E-09 | NA | + | NA | NA | 1.60E-01 | 7 | -152.006 | 318.013 | I.64 | 0.141 |
| -0.368 | NA | NA | + | NA | -1.83E-02 | 1.56E-01 | 7 | -152.153 | 318.305 | 1.933 | 0.122 |
| -0.406 | NA | NA | + | 1.13E-05 | NA | 1.55E-01 | 7 | -152.186 | 318.372 | 2 | 0.118 |
| -0.369 | NA | NA | + | NA | NA | 1.50E-01 | 6 | -137.482 | 286.964 | 0 | 0.37 |
| -0.329 | NA | NA | NA | NA | NA | 1.86E-01 | 3 | -141.213 | 288.425 | 1.462 | 0.178 |
| -0.397 | 1.07E-09 | NA | + | NA | NA | 1.54E-01 | 7 | -137.306 | 288.612 | 1.648 | 0.162 |
| -0.319 | NA | NA | + | NA | -2.43E-02 | 1.51E-01 | 7 | -137.411 | 288.823 | 1.859 | 0.146 |
| -0.399 | NA | NA | + | -3.66E-04 | NA | 1.52E-01 | 7 | -137.433 | 288.865 | 1.901 | 0.143 |
| -0.584 | NA | NA | + | NA | NA | 2.02E-01 | 6 | -160.239 | 332.478 | 0 | 0.397 |
| -0.444 | NA | NA | + | NA | -6.23E-02 | 2.02E-01 | 7 | -159.819 | 333.638 | I.16 | 0.222 |
| -0.629 | 1.32E-09 | NA | + | NA | NA | 2.12E-01 | 7 | -159.857 | 333.713 | 1.235 | 0.214 |
| -0.627 | NA | NA | + | -5.80E-04 | NA | 2.05E-01 | 7 | -160.112 | 334.224 | 1.746 | 0.166 |
| -0.378 | NA | NA | + | NA | NA | 1.45E-01 | 6 | -148.577 | 309.154 | 0 | 0.286 |
| -0.213 | NA | NA | NA | 1.49E-03 | NA | 1.53E-01 | 4 | -150.822 | 309.643 | 0.489 | 0.224 |
| -0.319 | NA | NA | NA | NA | NA | 1.83E-01 | 3 | -152.237 | 310.474 | I.32 | 0.148 |
| -0.406 | 9.36E-10 | NA | + | NA | NA | 1.50E-01 | 7 | -148.413 | 310.827 | 1.672 | 0.124 |
| -0.347 | NA | NA | + | 3.44E-04 | NA | 1.43E-01 | 7 | -148.537 | 311.075 | I.92 | 0.11 |
| -0.343 | NA | NA | + | NA | -1.60E-02 | 1.46E-01 | 7 | -148.551 | 311.102 | 1.947 | 0.108 |
| -0.489 | NA | NA | + | NA | NA | 1.74E-01 | 6 | -145.376 | 302.752 | 0 | 0.409 |
| -0.565 | NA | NA | + | -9.81E-04 | NA | 1.78E-01 | 7 | -145.005 | 304.01 | 1.258 | 0.218 |
| -0.521 | 1.20E-09 | NA | + | NA | NA | 1.79E-01 | 7 | -145.121 | 304.243 | 1.491 | 0.194 |
| -0.407 | NA | NA | + | NA | -3.90E-02 | 1.76E-01 | 7 | -145.204 | 304.408 | 1.656 | 0.179 |
| -0.424 | NA | NA | + | NA | NA | 1.58E-01 | 6 | -141.147 | 294.294 | 0 | 0.416 |
| -0.506 | NA | NA | + | -9.88E-04 | NA | 1.64E-01 | 7 | -140.776 | 295.552 | 1.258 | 0.222 |
| -0.453 | 1.13E-09 | NA | + | NA | NA | 1.63E-01 | 7 | -140.943 | 295.885 | 1.591 | 0.188 |
| -0.35 | NA | NA | + | NA | -3.46E-02 | 1.60E-01 | 7 | -141.01 | 296.02 | 1.726 | 0.175 |
| -0.408 | NA | NA | + | NA | NA | 1.51E-01 | 6 | -160.49 | 332.98 | 0 | 0.231 |
| -0.236 | NA | NA | NA | 1.58E-03 | NA | 1.63E-01 | 4 | -162.517 | 333.034 | 0.053 | 0.225 |
| -0.333 | NA | NA | NA | NA | NA | 1.90E-01 | 3 | -163.761 | 333.523 | 0.542 | 0.176 |
| -0.437 | 1.03E-09 | NA | + | NA | NA | 1.56E-01 | 7 | -160.349 | 334.697 | 1.717 | 0.098 |
| -0.266 | 1.02E-09 | NA | NA | 1.58E-03 | NA | 1.67E-01 | 5 | -162.381 | 334.762 | 1.782 | 0.095 |
| -0.371 | NA | NA | + | 4.38E-04 | NA | 1.49E-01 | 7 | -160.436 | 334.871 | 1.891 | 0.09 |
| -0.444 | NA | NA | + | NA | 1.62E-02 | 1.50E-01 | 7 | -160.467 | 334.934 | 1.953 | 0.087 |
| -0.439 | NA | NA | + | NA | NA | 1.60E-01 | 6 | -148.934 | 309.868 | 0 | 0.445 |
| -0.468 | 1.04E-09 | NA | + | NA | NA | 1.64E-01 | 7 | -148.767 | 311.533 | 1.665 | 0.194 |
| -0.492 | NA | NA | + | -6.41E-04 | NA | 1.63E-01 | 7 | -148.793 | 311.587 | 1.719 | 0.189 |
| -0.39 | NA | NA | + | NA | -2.23E-02 | 1.61E-01 | 7 | -148.883 | 311.766 | 1.898 | 0.172 |
| -0.641 | NA | NA | + | NA | NA | 2.18E-01 | 6 | -165.214 | 342.428 | 0 | 0.402 |
| -0.688 | 1.67E-09 | NA | + | NA | NA | 2.27E-01 | 7 | -164.819 | 343.637 | 1.209 | 0.22 |
| -0.516 | NA | NA | + | NA | -5.48E-02 | 2.18E-01 | 7 | -164.889 | 343.778 | I.35 | 0.205 |
| -0.688 | NA | NA | + | -6.62E-04 | NA | 2.21E-01 | 7 | -165.057 | 344.114 | 1.685 | 0.173 |
| -0.495 | NA | NA | + | NA | NA | 1.75E-01 | 6 | -153.347 | 318.694 | 0 | 0.442 |
| -0.529 | 1.22E-09 | NA | + | NA | NA | 1.80E-01 | 7 | -153.104 | 320.208 | 1.514 | 0.207 |
| -0.533 | NA | NA | + | -4.89E-04 | NA | 1.77E-01 | 7 | -153.266 | 320.532 | 1.838 | 0.176 |
| -0.439 | NA | NA | + | NA | -2.56E-02 | 1.76E-01 | 7 | -153.281 | 320.562 | 1.868 | 0.174 |
| -0.431 | NA | NA | + | NA | NA | 1.62E-01 | 6 | -143.965 | 299.93 | 0 | 0.441 |
| -0.46 | 9.89E-10 | NA | + | NA | NA | 1.67E-01 | 7 | -143.762 | 301.523 | 1.593 | 0.199 |
| -0.363 | NA | NA | + | NA | -3.24E-02 | 1.64E-01 | 7 | -143.846 | 301.692 | 1.763 | 0.183 |
| -0.476 | NA | NA | + | -5.28E-04 | NA | 1.65E-01 | 7 | -143.87 | 301.739 | 1.809 | 0.178 |
| -0.476 | NA | NA | + | NA | NA | 1.71E-01 | 6 | -155.731 | 323.463 | 0 | 0.442 |
| -0.509 | 1.10E-09 | NA | + | NA | NA | 1.76E-01 | 7 | -155.507 | 325.014 | 1.552 | 0.203 |
| -0.414 | NA | NA | + | NA | -2.96E-02 | 1.72E-01 | 7 | -155.64 | 325.281 | 1.818 | 0.178 |
| -0.514 | NA | NA | + | -4.95E-04 | NA | 1.72E-01 | 7 | -155.644 | 325.289 | 1.826 | 0.177 |
| -0.151 | NA | NA | NA | 1.48E-03 | NA | 1.31E-01 | 4 | -154.561 | 317.123 | 0 | 0.312 |
| -0.247 | NA | NA | NA | NA | NA | 1.60E-01 | 3 | -155.807 | 317.614 | 0.491 | 0.244 |
| -0.322 | NA | NA | + | NA | NA | 1.27E-01 | 6 | -153.028 | 318.055 | 0.932 | 0.195 |
| -0.174 | 6.76E-10 | NA | NA | 1.47E-03 | NA | 1.36E-01 | 5 | -154.467 | 318.933 | I.81 | 0.126 |
| -0.202 | NA | NA | NA | 1.61E-03 | 2.86E-02 | 1.30E-01 | 5 | -154.49 | 318.981 | 1.858 | 0.123 |
| -0.384 | NA | NA | + | NA | NA | 1.44E-01 | 6 | -154.468 | 320.937 | 0 | 0.313 |
| -0.313 | NA | NA | NA | NA | NA | 1.86E-01 | 3 | -158.128 | 322.256 | 1.319 | 0.162 |
| -0.232 | NA | NA | NA | 1.32E-03 | NA | 1.61E-01 | 4 | -157.142 | 322.283 | 1.347 | 0.16 |
| -0.41 | 8.15E-10 | NA | + | NA | NA | 1.50E-01 | 7 | -154.332 | 322.663 | 1.726 | 0.132 |
| -0.346 | NA | NA | + | NA | -1.75E-02 | 1.45E-01 | 7 | -154.438 | 322.877 | I.94 | 0.119 |
| -0.383 | NA | NA | + | 1.49E-05 | NA | 1.44E-01 | 7 | -154.468 | 322.937 | 2 | 0.115 |
| -0.426 | NA | NA | + | NA | NA | 1.51E-01 | 6 | -142.425 | 296.85 | 0 | 0.414 |
| -0.468 | 7.47E-10 | NA | + | NA | NA | 1.64E-01 | 7 | -142.072 | 298.144 | 1.294 | 0.217 |
| -0.327 | NA | NA | + | NA | -4.45E-02 | 1.53E-01 | 7 | -142.207 | 298.413 | 1.564 | 0.189 |
| -0.483 | NA | NA | + | -6.69E-04 | NA | 1.55E-01 | 7 | -142.258 | 298.516 | 1.666 | 0.18 |
| -0.429 | NA | NA | + | NA | NA | 1.58E-01 | 6 | -149.472 | 310.944 | 0 | 0.451 |
| -0.459 | 1.13E-09 | NA | + | NA | NA | 1.62E-01 | 7 | -149.301 | 312.603 | 1.659 | 0.197 |
| -0.466 | NA | NA | + | -4.40E-04 | NA | 1.60E-01 | 7 | -149.404 | 312.808 | 1.864 | 0.177 |
| -0.378 | NA | NA | + | NA | -2.34E-02 | 1.59E-01 | 7 | -149.415 | 312.829 | 1.885 | 0.176 |
| -0.649 | NA | NA | + | NA | NA | 2.21E-01 | 6 | -158.552 | 329.104 | 0 | 0.175 |
| -0.528 | NA | NA | + | -1.64E-03 | -1.04E-01 | 2.27E-01 | 8 | -156.585 | 329.171 | 0.066 | 0.169 |
| -0.451 | NA | NA | + | NA | -8.65E-02 | 2.19E-01 | 7 | -157.643 | 329.287 | 0.183 | 0.16 |
| -0.743 | NA | NA | + | -1.30E-03 | NA | 2.27E-01 | 7 | -157.87 | 329.74 | 0.636 | 0.127 |
| -0.698 | 1.55E-09 | NA | + | NA | NA | 2.31E-01 | 7 | -158.051 | 330.102 | 0.998 | 0.106 |
| -0.584 | 1.41E-09 | NA | + | -1.65E-03 | -9.93E-02 | 2.36E-01 | 9 | -156.163 | 330.326 | 1.222 | 0.095 |
| -0.505 | 1.39E-09 | NA | + | NA | -8.16E-02 | 2.28E-01 | 8 | -157.243 | 330.486 | 1.382 | 0.088 |
| -0.795 | 1.60E-09 | NA | + | -1.33E-03 | NA | 2.37E-01 | 8 | -157.331 | 330.662 | 1.558 | 0.08 |
| -0.421 | NA | NA | + | NA | NA | 1.52E-01 | 6 | -145.408 | 302.816 | 0 | 0.433 |
| -0.499 | NA | NA | + | -9.15E-04 | NA | 1.58E-01 | 7 | -145.12 | 304.24 | 1.424 | 0.213 |
| -0.445 | 7.42E-10 | NA | + | NA | NA | 1.57E-01 | 7 | -145.277 | 304.553 | 1.737 | 0.182 |
| -0.363 | NA | NA | + | NA | -2.68E-02 | 1.54E-01 | 7 | -145.332 | 304.664 | 1.848 | 0.172 |
| -0.377 | NA | NA | + | NA | NA | 1.55E-01 | 6 | -141.302 | 294.604 | 0 | 0.44 |
| -0.404 | 1.08E-09 | NA | + | NA | NA | 1.59E-01 | 7 | -141.112 | 296.225 | I.62 | 0.196 |
| -0.299 | NA | NA | + | NA | -3.63E-02 | 1.56E-01 | 7 | -141.153 | 296.307 | 1.702 | 0.188 |
| -0.417 | NA | NA | + | -4.79E-04 | NA | 1.58E-01 | 7 | -141.214 | 296.427 | 1.823 | 0.177 |
| -0.404 | NA | NA | + | NA | NA | 1.47E-01 | 6 | -159.673 | 331.345 | 0 | 0.299 |
| -0.231 | NA | NA | NA | 1.50E-03 | NA | 1.58E-01 | 4 | -162.124 | 332.249 | 0.903 | 0.19 |
| -0.328 | NA | NA | NA | NA | NA | 1.86E-01 | 3 | -163.289 | 332.578 | 1.232 | 0.161 |
| -0.432 | 8.91E-10 | NA | + | NA | NA | 1.52E-01 | 7 | -159.532 | 333.064 | 1.719 | 0.126 |
| -0.375 | NA | NA | + | 3.31E-04 | NA | 1.44E-01 | 7 | -159.64 | 333.281 | 1.935 | 0.113 |
| -0.418 | NA | NA | + | NA | 6.55E-03 | 1.46E-01 | 7 | -159.669 | 333.338 | 1.993 | 0.11 |
| -0.464 | NA | NA | + | NA | NA | 1.65E-01 | 6 | -153.583 | 319.167 | 0 | 0.457 |
| -0.492 | 1.03E-09 | NA | + | NA | NA | 1.69E-01 | 7 | -153.436 | 320.873 | 1.706 | 0.195 |
| -0.503 | NA | NA | + | -4.48E-04 | NA | 1.68E-01 | 7 | -153.52 | 321.041 | 1.874 | 0.179 |
| -0.44 | NA | NA | + | NA | -1.08E-02 | 1.65E-01 | 7 | -153.572 | 321.144 | 1.977 | 0.17 |
| -0.511 | NA | NA | + | NA | NA | 1.79E-01 | 6 | -139.393 | 290.787 | 0 | 0.244 |
| -0.345 | NA | NA | + | NA | -7.53E-02 | 1.82E-01 | 7 | -138.656 | 291.311 | 0.524 | 0.188 |
| -0.421 | NA | NA | + | -1.52E-03 | -9.14E-02 | 1.88E-01 | 8 | -137.689 | 291.378 | 0.591 | 0.182 |
| -0.601 | NA | NA | + | -1.23E-03 | NA | 1.84E-01 | 7 | -138.751 | 291.501 | 0.715 | 0.171 |
| -0.546 | 1.06E-09 | NA | + | NA | NA | 1.86E-01 | 7 | -139.073 | 292.146 | 1.359 | 0.124 |
| -0.383 | 9.74E-10 | NA | + | NA | -7.28E-02 | 1.88E-01 | 8 | -138.382 | 292.764 | 1.978 | 0.091 |
| -0.695 | NA | NA | + | NA | NA | 2.17E-01 | 6 | -166.376 | 344.751 | 0 | 0.245 |
| -0.788 | NA | NA | + | -1.43E-03 | NA | 2.21E-01 | 7 | -165.61 | 345.22 | 0.469 | 0.194 |
| -0.607 | NA | NA | + | -1.68E-03 | -8.74E-02 | 2.24E-01 | 8 | -164.804 | 345.608 | 0.856 | 0.16 |
| -0.535 | NA | NA | + | NA | -7.04E-02 | 2.19E-01 | 7 | -165.844 | 345.688 | 0.936 | 0.154 |
| -0.742 | 1.49E-09 | NA | + | NA | NA | 2.27E-01 | 7 | -165.955 | 345.91 | 1.159 | 0.137 |
| -0.837 | 1.51E-09 | NA | + | -1.44E-03 | NA | 2.31E-01 | 8 | -165.174 | 346.347 | 1.596 | 0.11 |
| -0.477 | NA | NA | + | NA | NA | 1.65E-01 | 6 | -160.436 | 332.872 | 0 | 0.453 |
| -0.511 | 1.26E-09 | NA | + | NA | NA | 1.70E-01 | 7 | -160.237 | 334.475 | 1.603 | 0.203 |
| -0.511 | NA | NA | + | -4.24E-04 | NA | 1.67E-01 | 7 | -160.38 | 334.76 | 1.889 | 0.176 |
| -0.468 | NA | NA | + | NA | -3.98E-03 | 1.65E-01 | 7 | -160.434 | 334.869 | 1.997 | 0.167 |
| -0.599 | NA | NA | + | NA | NA | 2.03E-01 | 6 | -156.92 | 325.839 | 0 | 0.395 |
| -0.473 | NA | NA | + | NA | -5.54E-02 | 2.04E-01 | 7 | -156.568 | 327.136 | 1.297 | 0.206 |
| -0.641 | 1.58E-09 | NA | + | NA | NA | 2.10E-01 | 7 | -156.594 | 327.188 | 1.349 | 0.201 |
| -0.668 | NA | NA | + | -9.30E-04 | NA | 2.07E-01 | 7 | -156.608 | 327.217 | 1.378 | 0.198 |
| -0.411 | NA | NA | + | NA | NA | 1.54E-01 | 6 | -156.475 | 324.949 | 0 | 0.327 |
| -0.356 | NA | NA | NA | NA | NA | 1.96E-01 | 3 | -160.271 | 326.542 | 1.593 | 0.148 |
| -0.438 | 9.00E-10 | NA | + | NA | NA | 1.59E-01 | 7 | -156.319 | 326.638 | 1.688 | 0.141 |
| -0.478 | NA | NA | + | NA | 3.15E-02 | 1.52E-01 | 7 | -156.387 | 326.773 | 1.824 | 0.132 |
| -0.267 | NA | NA | NA | 1.26E-03 | NA | 1.71E-01 | 4 | -159.4 | 326.8 | 1.851 | 0.13 |
| -0.433 | NA | NA | + | -2.61E-04 | NA | 1.55E-01 | 7 | -156.455 | 326.91 | 1.961 | 0.123 |
| -0.426 | NA | NA | + | NA | NA | 1.57E-01 | 6 | -151.217 | 314.433 | 0 | 0.458 |
| -0.454 | 1.02E-09 | NA | + | NA | NA | 1.61E-01 | 7 | -151.067 | 316.135 | 1.702 | 0.195 |
| -0.463 | NA | NA | + | -4.34E-04 | NA | 1.60E-01 | 7 | -151.156 | 316.313 | I.88 | 0.179 |
| -0.424 | NA | NA | + | NA | -1.26E-03 | 1.57E-01 | 7 | -151.216 | 316.433 | 2 | 0.168 |
| -0.458 | NA | NA | + | NA | NA | 1.65E-01 | 6 | -149.31 | 310.62 | 0 | 0.446 |
| -0.488 | 1.06E-09 | NA | + | NA | NA | 1.70E-01 | 7 | -149.125 | 312.25 | I.63 | 0.197 |
| -0.382 | NA | NA | + | NA | -3.38E-02 | 1.65E-01 | 7 | -149.193 | 312.386 | 1.766 | 0.184 |
| -0.488 | NA | NA | + | -3.74E-04 | NA | 1.67E-01 | 7 | -149.26 | 312.52 | 1.901 | 0.172 |
| -0.571 | NA | NA | + | -1.95E-03 | -9.52E-02 | 2.28E-01 | 8 | -154.662 | 325.325 | 0 | 0.197 |
| -0.765 | NA | NA | + | -1.63E-03 | NA | 2.28E-01 | 7 | -155.697 | 325.394 | 0.069 | 0.19 |
| -0.653 | NA | NA | + | NA | NA | 2.22E-01 | 6 | -156.778 | 325.555 | 0.231 | 0.175 |
| -0.489 | NA | NA | + | NA | -7.30E-02 | 2.21E-01 | 7 | -156.156 | 326.312 | 0.987 | 0.12 |
| -0.815 | 1.93E-09 | NA | + | -1.61E-03 | NA | 2.36E-01 | 8 | -155.234 | 326.467 | 1.142 | 0.111 |
| -0.707 | 1.98E-09 | NA | + | NA | NA | 2.30E-01 | 7 | -156.296 | 326.593 | 1.268 | 0.104 |
| -0.626 | 1.64E-09 | NA | + | -1.91E-03 | -8.95E-02 | 2.34E-01 | 9 | -154.324 | 326.647 | 1.323 | 0.102 |
| -0.339 | NA | NA | + | NA | NA | 1.34E-01 | 6 | -146.852 | 305.704 | 0 | 0.314 |
| -0.293 | NA | NA | NA | NA | NA | 1.75E-01 | 3 | -150.471 | 306.943 | 1.239 | 0.169 |
| -0.2 | NA | NA | NA | 1.21E-03 | NA | 1.49E-01 | 4 | -149.542 | 307.083 | 1.379 | 0.157 |
| -0.361 | 8.59E-10 | NA | + | NA | NA | 1.38E-01 | 7 | -146.744 | 307.487 | 1.783 | 0.129 |
| -0.354 | NA | NA | + | -1.73E-04 | NA | 1.35E-01 | 7 | -146.842 | 307.685 | 1.981 | 0.116 |
| -0.337 | NA | NA | + | NA | -1.07E-03 | 1.34E-01 | 7 | -146.852 | 307.704 | 2 | 0.115 |
| -0.364 | NA | NA | + | NA | NA | 1.42E-01 | 6 | -148.179 | 308.357 | 0 | 0.305 |
| -0.309 | NA | NA | NA | NA | NA | 1.84E-01 | 3 | -151.761 | 309.522 | 1.165 | 0.171 |
| -0.219 | NA | NA | NA | 1.24E-03 | NA | 1.57E-01 | 4 | -150.799 | 309.597 | I.24 | 0.164 |
| -0.392 | 1.04E-09 | NA | + | NA | NA | 1.47E-01 | 7 | -148.014 | 310.028 | 1.671 | 0.132 |
| -0.332 | NA | NA | + | NA | -1.48E-02 | 1.43E-01 | 7 | -148.156 | 310.313 | 1.956 | 0.115 |
| -0.366 | NA | NA | + | -2.48E-05 | NA | 1.42E-01 | 7 | -148.178 | 310.357 | 2 | 0.112 |
| -0.766 | NA | NA | + | NA | NA | 2.49E-01 | 6 | -171.428 | 354.855 | 0 | 0.255 |
| -0.592 | NA | NA | + | NA | -7.46E-02 | 2.46E-01 | 7 | -170.809 | 355.618 | 0.762 | 0.174 |
| -0.845 | NA | NA | + | -1.25E-03 | NA | 2.53E-01 | 7 | -170.832 | 355.665 | 0.809 | 0.17 |
| -0.652 | NA | NA | + | -1.52E-03 | -9.03E-02 | 2.50E-01 | 8 | -169.944 | 355.889 | 1.034 | 0.152 |
| -0.819 | 1.92E-09 | NA | + | NA | NA | 2.58E-01 | 7 | -170.969 | 355.939 | 1.084 | 0.148 |
| -0.9 | 1.95E-09 | NA | + | -1.26E-03 | NA | 2.62E-01 | 8 | -170.357 | 356.713 | 1.858 | 0.101 |
| -0.632 | NA | NA | + | NA | NA | 2.12E-01 | 6 | -162.109 | 336.219 | 0 | 0.397 |
| -0.716 | NA | NA | + | -1.17E-03 | NA | 2.18E-01 | 7 | -161.64 | 337.279 | 1.061 | 0.233 |
| -0.677 | 1.68E-09 | NA | + | NA | NA | 2.19E-01 | 7 | -161.778 | 337.555 | 1.337 | 0.203 |
| -0.55 | NA | NA | + | NA | -3.71E-02 | 2.13E-01 | 7 | -161.974 | 337.949 | I.73 | 0.167 |
| -0.371 | NA | NA | + | NA | NA | 1.46E-01 | 6 | -133.762 | 279.523 | 0 | 0.423 |
| -0.269 | NA | NA | + | NA | -4.71E-02 | 1.48E-01 | 7 | -133.489 | 280.978 | 1.455 | 0.204 |
| -0.398 | 7.65E-10 | NA | + | NA | NA | 1.52E-01 | 7 | -133.566 | 281.132 | 1.609 | 0.189 |
| -0.427 | NA | NA | + | -6.42E-04 | NA | 1.51E-01 | 7 | -133.598 | 281.195 | 1.672 | 0.183 |
| -0.473 | NA | NA | + | NA | NA | 1.65E-01 | 6 | -147.591 | 307.181 | 0 | 0.415 |
| -0.545 | NA | NA | + | -9.09E-04 | NA | 1.70E-01 | 7 | -147.295 | 308.589 | 1.408 | 0.205 |
| -0.507 | 9.08E-10 | NA | + | NA | NA | 1.73E-01 | 7 | -147.336 | 308.673 | 1.492 | 0.197 |
| -0.384 | NA | NA | + | NA | -4.07E-02 | 1.67E-01 | 7 | -147.406 | 308.813 | 1.632 | 0.183 |
| -0.701 | NA | NA | + | -1.61E-03 | NA | 2.02E-01 | 7 | -150.978 | 315.957 | 0 | 0.235 |
| -0.582 | NA | NA | + | NA | NA | 1.96E-01 | 6 | -151.989 | 315.978 | 0.021 | 0.233 |
| -0.552 | NA | NA | + | -1.86E-03 | -7.63E-02 | 2.07E-01 | 8 | -150.35 | 316.701 | 0.744 | 0.162 |
| -0.744 | 1.47E-09 | NA | + | -1.62E-03 | NA | 2.09E-01 | 8 | -150.599 | 317.198 | 1.242 | 0.126 |
| -0.623 | 1.46E-09 | NA | + | NA | NA | 2.03E-01 | 7 | -151.623 | 317.246 | 1.289 | 0.124 |
| -0.461 | NA | NA | + | NA | -5.51E-02 | 1.98E-01 | 7 | -151.654 | 317.308 | 1.351 | 0.12 |
| -0.692 | NA | NA | + | NA | NA | 2.31E-01 | 6 | -166.061 | 344.122 | 0 | 0.196 |
| -0.499 | NA | NA | + | NA | -8.38E-02 | 2.29E-01 | 7 | -165.257 | 344.514 | 0.391 | 0.161 |
| -0.572 | NA | NA | + | -1.57E-03 | -9.93E-02 | 2.34E-01 | 8 | -164.322 | 344.643 | 0.521 | 0.151 |
| -0.78 | NA | NA | + | -1.28E-03 | NA | 2.35E-01 | 7 | -165.43 | 344.86 | 0.738 | 0.136 |
| -0.745 | 1.99E-09 | NA | + | NA | NA | 2.40E-01 | 7 | -165.597 | 345.194 | 1.071 | 0.115 |
| -0.56 | 1.71E-09 | NA | + | NA | -7.74E-02 | 2.37E-01 | 8 | -164.916 | 345.831 | 1.709 | 0.084 |
| -0.834 | 2.00E-09 | NA | + | -1.28E-03 | NA | 2.44E-01 | 8 | -164.96 | 345.921 | 1.798 | 0.08 |
| -0.63 | 1.66E-09 | NA | + | -1.55E-03 | -9.29E-02 | 2.42E-01 | 9 | -163.997 | 345.995 | 1.872 | 0.077 |
| -0.525 | NA | NA | + | NA | NA | 1.84E-01 | 6 | -159.368 | 330.736 | 0 | 0.397 |
| -0.601 | NA | NA | + | -9.93E-04 | NA | 1.89E-01 | 7 | -159.03 | 332.06 | 1.324 | 0.205 |
| -0.404 | NA | NA | + | NA | -5.45E-02 | 1.84E-01 | 7 | -159.046 | 332.092 | 1.356 | 0.202 |
| -0.563 | 1.37E-09 | NA | + | NA | NA | 1.91E-01 | 7 | -159.069 | 332.138 | 1.402 | 0.197 |
| -0.493 | NA | NA | + | NA | NA | 1.73E-01 | 6 | -162.492 | 336.985 | 0 | 0.445 |
| -0.529 | 1.30E-09 | NA | + | NA | NA | 1.78E-01 | 7 | -162.272 | 338.545 | I.56 | 0.204 |
| -0.534 | NA | NA | + | -5.29E-04 | NA | 1.75E-01 | 7 | -162.403 | 338.806 | 1.821 | 0.179 |
| -0.444 | NA | NA | + | NA | -2.32E-02 | 1.74E-01 | 7 | -162.439 | 338.878 | 1.894 | 0.173 |
| -0.586 | NA | NA | + | NA | NA | 2.05E-01 | 6 | -151.828 | 315.657 | 0 | 0.225 |
| -0.415 | NA | NA | + | NA | -7.68E-02 | 2.05E-01 | 7 | -151.099 | 316.197 | 0.541 | 0.172 |
| -0.487 | NA | NA | + | -1.48E-03 | -9.29E-02 | 2.11E-01 | 8 | -150.204 | 316.407 | 0.751 | 0.155 |
| -0.672 | NA | NA | + | -1.18E-03 | NA | 2.09E-01 | 7 | -151.244 | 316.488 | 0.832 | 0.148 |
| -0.634 | 1.73E-09 | NA | + | NA | NA | 2.12E-01 | 7 | -151.397 | 316.794 | 1.137 | 0.127 |
| -0.469 | 1.53E-09 | NA | + | NA | -7.19E-02 | 2.11E-01 | 8 | -150.759 | 317.519 | 1.862 | 0.089 |
| -0.72 | 1.73E-09 | NA | + | -1.18E-03 | NA | 2.17E-01 | 8 | -150.81 | 317.62 | 1.964 | 0.084 |
| -0.514 | NA | NA | + | -1.80E-03 | -9.62E-02 | 2.13E-01 | 8 | -151.996 | 319.991 | 0 | 0.175 |
| -0.598 | NA | NA | + | NA | NA | 2.05E-01 | 6 | -154.006 | 320.011 | 0.02 | 0.174 |
| -0.709 | NA | NA | + | -1.52E-03 | NA | 2.12E-01 | 7 | -153.087 | 320.174 | 0.183 | 0.16 |
| -0.422 | NA | NA | + | NA | -7.83E-02 | 2.05E-01 | 7 | -153.271 | 320.543 | 0.551 | 0.133 |
| -0.645 | 1.42E-09 | NA | + | NA | NA | 2.14E-01 | 7 | -153.572 | 321.144 | 1.153 | 0.099 |
| -0.566 | 1.32E-09 | NA | + | -1.81E-03 | -9.22E-02 | 2.21E-01 | 9 | -151.614 | 321.227 | 1.236 | 0.095 |
| -0.759 | 1.47E-09 | NA | + | -1.54E-03 | NA | 2.21E-01 | 8 | -152.617 | 321.233 | 1.242 | 0.094 |
| -0.473 | 1.30E-09 | NA | + | NA | -7.42E-02 | 2.13E-01 | 8 | -152.911 | 321.823 | 1.832 | 0.07 |
| -0.616 | NA | NA | + | NA | NA | 2.04E-01 | 6 | -156.185 | 324.369 | 0 | 0.264 |
| -0.71 | NA | NA | + | -1.30E-03 | NA | 2.10E-01 | 7 | -155.529 | 325.059 | 0.689 | 0.187 |
| -0.464 | NA | NA | + | NA | -6.64E-02 | 2.04E-01 | 7 | -155.666 | 325.332 | 0.963 | 0.163 |
| -0.542 | NA | NA | + | -1.53E-03 | -8.10E-02 | 2.11E-01 | 8 | -154.772 | 325.544 | 1.175 | 0.147 |
| -0.659 | 1.58E-09 | NA | + | NA | NA | 2.11E-01 | 7 | -155.83 | 325.66 | 1.291 | 0.139 |
| -0.754 | 1.59E-09 | NA | + | -1.30E-03 | NA | 2.16E-01 | 8 | -155.166 | 326.332 | 1.962 | 0.099 |
| -0.517 | NA | NA | + | NA | NA | 1.78E-01 | 6 | -160.597 | 333.194 | 0 | 0.446 |
| -0.553 | 1.15E-09 | NA | + | NA | NA | 1.85E-01 | 7 | -160.347 | 334.694 | 01.V | 0.211 |
| -0.462 | NA | NA | + | NA | -2.46E-02 | 1.79E-01 | 7 | -160.535 | 335.069 | 1.875 | 0.175 |
| -0.54 | NA | NA | + | -3.06E-04 | NA | 1.80E-01 | 7 | -160.566 | 335.132 | 1.938 | 0.169 |
| -0.597 | NA | NA | + | NA | NA | 2.02E-01 | 6 | -162.85 | 337.7 | 0 | 0.405 |
| -0.676 | NA | NA | + | -1.08E-03 | NA | 2.07E-01 | 7 | -162.445 | 338.891 | 1.191 | 0.223 |
| -0.64 | 1.38E-09 | NA | + | NA | NA | 2.10E-01 | 7 | -162.535 | 339.071 | 1.371 | 0.204 |
| -0.521 | NA | NA | + | NA | -3.40E-02 | 2.02E-01 | 7 | -162.733 | 339.467 | 1.767 | 0.167 |
| -0.442 | NA | NA | + | NA | NA | 1.58E-01 | 6 | -144.89 | 301.779 | 0 | 0.431 |
| -0.502 | NA | NA | + | -7.47E-04 | NA | 1.62E-01 | 7 | -144.687 | 303.373 | 1.594 | 0.194 |
| -0.47 | 1.01E-09 | NA | + | NA | NA | 1.62E-01 | 7 | -144.711 | 303.422 | 1.643 | 0.19 |
| -0.359 | NA | NA | + | NA | -3.81E-02 | 1.60E-01 | 7 | -144.733 | 303.465 | 1.686 | 0.185 |
| -0.417 | NA | NA | + | NA | NA | 1.52E-01 | 6 | -142.924 | 297.848 | 0 | 0.424 |
| -0.489 | NA | NA | + | -8.72E-04 | NA | 1.56E-01 | 7 | -142.651 | 299.302 | 1.454 | 0.205 |
| -0.446 | 1.02E-09 | NA | + | NA | NA | 1.57E-01 | 7 | -142.722 | 299.445 | 1.597 | 0.191 |
| -0.337 | NA | NA | + | NA | -3.68E-02 | 1.54E-01 | 7 | -142.775 | 299.549 | 1.702 | 0.181 |
| -0.425 | NA | NA | + | NA | NA | 1.60E-01 | 6 | -138.886 | 289.772 | 0 | 0.429 |
| -0.491 | NA | NA | + | -7.66E-04 | NA | 1.65E-01 | 7 | -138.663 | 291.327 | 1.555 | 0.197 |
| -0.454 | 8.82E-10 | NA | + | NA | NA | 1.65E-01 | 7 | -138.689 | 291.378 | 1.606 | 0.192 |
| -0.345 | NA | NA | + | NA | -3.57E-02 | 1.61E-01 | 7 | -138.742 | 291.483 | 1.712 | 0.182 |
| -0.475 | NA | NA | + | NA | NA | 1.68E-01 | 6 | -143.404 | 298.808 | 0 | 0.389 |
| -0.336 | NA | NA | + | NA | -6.17E-02 | 1.69E-01 | 7 | -142.943 | 299.886 | 1.078 | 0.227 |
| -0.55 | NA | NA | + | -9.20E-04 | NA | 1.73E-01 | 7 | -143.072 | 300.145 | 1.337 | 0.199 |
| -0.51 | 1.16E-09 | NA | + | NA | NA | 1.74E-01 | 7 | -143.148 | 300.295 | 1.487 | 0.185 |
| -0.536 | NA | NA | + | NA | NA | 1.83E-01 | 6 | -153.081 | 318.162 | 0 | 0.325 |
| -0.636 | NA | NA | + | -1.31E-03 | NA | 1.88E-01 | 7 | -152.445 | 318.89 | 0.729 | 0.226 |
| -0.575 | 1.27E-09 | NA | + | NA | NA | 1.90E-01 | 7 | -152.785 | 319.571 | 1.409 | 0.161 |
| -0.426 | NA | NA | + | NA | -5.04E-02 | 1.85E-01 | 7 | -152.806 | 319.611 | I.45 | 0.157 |
| -0.508 | NA | NA | + | -1.51E-03 | -6.61E-02 | 1.91E-01 | 8 | -151.983 | 319.966 | 1.805 | 0.132 |
| -0.519 | NA | NA | + | -1.57E-03 | -1.18E-01 | 2.34E-01 | 8 | -160.882 | 337.764 | 0 | 0.22 |
| -0.452 | NA | NA | + | NA | -1.03E-01 | 2.30E-01 | 7 | -161.929 | 337.858 | 0.094 | 0.21 |
| -0.688 | NA | NA | + | NA | NA | 2.34E-01 | 6 | -163.308 | 338.616 | 0.852 | 0.144 |
| -0.578 | 1.69E-09 | NA | + | -1.55E-03 | -1.12E-01 | 2.42E-01 | 9 | -160.508 | 339.016 | 1.252 | 0.118 |
| -0.515 | 1.76E-09 | NA | + | NA | -9.63E-02 | 2.38E-01 | 8 | -161.527 | 339.054 | I.29 | 0.115 |
| -0.767 | NA | NA | + | -1.22E-03 | NA | 2.38E-01 | 7 | -162.672 | 339.344 | I.58 | 0.1 |
| -0.746 | 2.11E-09 | NA | + | NA | NA | 2.43E-01 | 7 | -162.729 | 339.457 | 1.693 | 0.094 |
| -0.462 | NA | NA | + | NA | NA | 1.66E-01 | 6 | -156.354 | 324.707 | 0 | 0.447 |
| -0.496 | 1.15E-09 | NA | + | NA | NA | 1.71E-01 | 7 | -156.149 | 326.298 | 1.591 | 0.202 |
| -0.385 | NA | NA | + | NA | -3.47E-02 | 1.66E-01 | 7 | -156.232 | 326.463 | 1.756 | 0.186 |
| -0.478 | NA | NA | + | -1.94E-04 | NA | 1.67E-01 | 7 | -156.341 | 326.682 | 1.975 | 0.166 |
| -0.453 | NA | NA | + | NA | NA | 1.63E-01 | 6 | -148.621 | 309.241 | 0 | 0.44 |
| -0.485 | 1.09E-09 | NA | + | NA | NA | 1.68E-01 | 7 | -148.412 | 310.824 | 1.583 | 0.2 |
| -0.503 | NA | NA | + | -6.21E-04 | NA | 1.66E-01 | 7 | -148.484 | 310.969 | 1.727 | 0.186 |
| -0.397 | NA | NA | + | NA | -2.62E-02 | 1.64E-01 | 7 | -148.547 | 311.094 | 1.853 | 0.174 |
| -0.43 | NA | NA | + | NA | NA | 1.64E-01 | 6 | -147.187 | 306.373 | 0 | 0.437 |
| -0.493 | NA | NA | + | -7.59E-04 | NA | 1.68E-01 | 7 | -146.987 | 307.974 | 01.VI | 0.196 |
| -0.459 | 1.14E-09 | NA | + | NA | NA | 1.68E-01 | 7 | -147.004 | 308.009 | 1.636 | 0.193 |
| -0.372 | NA | NA | + | NA | -2.65E-02 | 1.64E-01 | 7 | -147.111 | 308.222 | 1.849 | 0.173 |
| -0.56 | NA | NA | + | NA | NA | 2.05E-01 | 6 | -155.881 | 323.762 | 0 | 0.39 |
| -0.415 | NA | NA | + | NA | -6.50E-02 | 2.05E-01 | 7 | -155.396 | 324.792 | 01.III | 0.233 |
| -0.602 | 1.54E-09 | NA | + | NA | NA | 2.11E-01 | 7 | -155.542 | 325.083 | 1.321 | 0.202 |
| -0.616 | NA | NA | + | -7.28E-04 | NA | 2.09E-01 | 7 | -155.682 | 325.363 | 1.601 | 0.175 |
| -0.484 | NA | NA | + | NA | NA | 1.73E-01 | 6 | -161.706 | 335.412 | 0 | 0.452 |
| -0.516 | 1.04E-09 | NA | + | NA | NA | 1.79E-01 | 7 | -161.521 | 337.042 | 1.629 | 0.2 |
| -0.423 | NA | NA | + | NA | -2.82E-02 | 1.74E-01 | 7 | -161.628 | 337.256 | 1.844 | 0.18 |
| -0.498 | NA | NA | + | -1.73E-04 | NA | 1.74E-01 | 7 | -161.697 | 337.393 | 1.981 | 0.168 |
| -0.413 | NA | NA | + | NA | NA | 1.56E-01 | 6 | -168.381 | 348.762 | 0 | 0.212 |
| -0.24 | NA | NA | NA | 1.68E-03 | NA | 1.65E-01 | 4 | -170.408 | 348.817 | 0.055 | 0.206 |
| -0.348 | NA | NA | NA | NA | NA | 1.94E-01 | 3 | -171.681 | 349.363 | 0.6 | 0.157 |
| -0.441 | 8.73E-10 | NA | + | NA | NA | 1.62E-01 | 7 | -168.24 | 350.48 | 1.718 | 0.09 |
| -0.358 | NA | NA | + | 6.55E-04 | NA | 1.52E-01 | 7 | -168.261 | 350.522 | 1.759 | 0.088 |
| -0.27 | 8.53E-10 | NA | NA | 1.66E-03 | NA | 1.71E-01 | 5 | -170.277 | 350.554 | 1.792 | 0.087 |
| -0.459 | NA | NA | + | NA | 2.17E-02 | 1.55E-01 | 7 | -168.343 | 350.685 | 1.923 | 0.081 |
| -0.278 | NA | NA | NA | 1.76E-03 | 2.11E-02 | 1.64E-01 | 5 | -170.373 | 350.746 | 1.983 | 0.079 |
| -0.376 | NA | NA | + | NA | NA | 1.47E-01 | 6 | -148.497 | 308.995 | 0 | 0.337 |
| -0.404 | 1.06E-09 | NA | + | NA | NA | 1.52E-01 | 7 | -148.32 | 310.64 | 1.645 | 0.148 |
| -0.219 | NA | NA | NA | 1.32E-03 | NA | 1.61E-01 | 4 | -151.414 | 310.827 | 1.833 | 0.135 |
| -0.306 | NA | NA | NA | NA | NA | 1.86E-01 | 3 | -152.464 | 310.928 | 1.934 | 0.128 |
| -0.341 | NA | NA | + | NA | -1.58E-02 | 1.48E-01 | 7 | -148.473 | 310.946 | 1.951 | 0.127 |
| -0.387 | NA | NA | + | -1.26E-04 | NA | 1.48E-01 | 7 | -148.492 | 310.984 | I.99 | 0.125 |
| -0.625 | NA | NA | + | NA | NA | 2.14E-01 | 6 | -158.194 | 328.388 | 0 | 0.249 |
| -0.732 | NA | NA | + | -1.47E-03 | NA | 2.20E-01 | 7 | -157.378 | 328.757 | 0.369 | 0.207 |
| -0.572 | NA | NA | + | -1.73E-03 | -8.02E-02 | 2.21E-01 | 8 | -156.663 | 329.325 | 0.937 | 0.156 |
| -0.487 | NA | NA | + | NA | -6.21E-02 | 2.14E-01 | 7 | -157.756 | 329.512 | 1.124 | 0.142 |
| -0.675 | 1.92E-09 | NA | + | NA | NA | 2.21E-01 | 7 | -157.806 | 329.611 | 1.224 | 0.135 |
| -0.781 | 1.90E-09 | NA | + | -1.47E-03 | NA | 2.27E-01 | 8 | -156.994 | 329.988 | 01.VI | 0.112 |
| -0.588 | NA | NA | + | NA | NA | 2.00E-01 | 6 | -169.134 | 350.269 | 0 | 0.431 |
| -0.633 | 1.63E-09 | NA | + | NA | NA | 2.07E-01 | 7 | -168.808 | 351.615 | 1.346 | 0.22 |
| -0.636 | NA | NA | + | -6.57E-04 | NA | 2.02E-01 | 7 | -168.998 | 351.997 | 1.728 | 0.182 |
| -0.533 | NA | NA | + | NA | -2.54E-02 | 2.00E-01 | 7 | -169.075 | 352.151 | 1.882 | 0.168 |
| -0.47 | NA | NA | + | NA | NA | 1.70E-01 | 6 | -146.154 | 304.308 | 0 | 0.415 |
| -0.507 | 1.25E-09 | NA | + | NA | NA | 1.76E-01 | 7 | -145.87 | 305.74 | 1.432 | 0.203 |
| -0.537 | NA | NA | + | -8.81E-04 | NA | 1.75E-01 | 7 | -145.875 | 305.75 | 1.443 | 0.202 |
| -0.385 | NA | NA | + | NA | -3.82E-02 | 1.71E-01 | 7 | -145.985 | 305.971 | 1.663 | 0.181 |
| -0.581 | NA | NA | + | NA | NA | 1.95E-01 | 6 | -159.357 | 330.713 | 0 | 0.402 |
| -0.659 | NA | NA | + | -1.07E-03 | NA | 1.99E-01 | 7 | -158.924 | 331.848 | 1.135 | 0.228 |
| -0.618 | 1.21E-09 | NA | + | NA | NA | 2.02E-01 | 7 | -159.078 | 332.157 | 1.444 | 0.195 |
| -0.489 | NA | NA | + | NA | -4.02E-02 | 1.95E-01 | 7 | -159.186 | 332.372 | 1.659 | 0.175 |
| -0.489 | NA | NA | + | NA | NA | 1.74E-01 | 6 | -149.103 | 310.206 | 0 | 0.412 |
| -0.385 | NA | NA | + | NA | -4.84E-02 | 1.77E-01 | 7 | -148.831 | 311.661 | 1.455 | 0.199 |
| -0.52 | 9.98E-10 | NA | + | NA | NA | 1.80E-01 | 7 | -148.856 | 311.712 | 1.506 | 0.194 |
| -0.55 | NA | NA | + | -7.95E-04 | NA | 1.77E-01 | 7 | -148.857 | 311.714 | 1.508 | 0.194 |
| -0.633 | NA | NA | + | NA | NA | 2.05E-01 | 6 | -161.118 | 334.237 | 0 | 0.38 |
| -0.478 | NA | NA | + | NA | -6.70E-02 | 2.04E-01 | 7 | -160.624 | 335.248 | 1.011 | 0.229 |
| -0.678 | 1.50E-09 | NA | + | NA | NA | 2.13E-01 | 7 | -160.751 | 335.501 | 1.264 | 0.202 |
| -0.688 | NA | NA | + | -8.70E-04 | NA | 2.07E-01 | 7 | -160.819 | 335.638 | 1.401 | 0.189 |
| -0.495 | NA | NA | + | NA | NA | 1.75E-01 | 6 | -157.792 | 327.585 | 0 | 0.434 |
| -0.535 | 1.43E-09 | NA | + | NA | NA | 1.81E-01 | 7 | -157.506 | 329.013 | 1.428 | 0.212 |
| -0.409 | NA | NA | + | NA | -3.78E-02 | 1.75E-01 | 7 | -157.646 | 329.292 | 1.707 | 0.185 |
| -0.529 | NA | NA | + | -4.20E-04 | NA | 1.77E-01 | 7 | -157.734 | 329.468 | 1.884 | 0.169 |
| -0.429 | NA | NA | + | NA | NA | 1.62E-01 | 6 | -158.281 | 328.562 | 0 | 0.332 |
| -0.367 | NA | NA | NA | NA | NA | 1.99E-01 | 3 | -162.072 | 330.144 | 1.582 | 0.151 |
| -0.459 | 1.10E-09 | NA | + | NA | NA | 1.67E-01 | 7 | -158.136 | 330.272 | I.71 | 0.141 |
| -0.287 | NA | NA | NA | 1.24E-03 | NA | 1.77E-01 | 4 | -161.231 | 330.463 | 1.901 | 0.128 |
| -0.454 | NA | NA | + | NA | 1.17E-02 | 1.62E-01 | 7 | -158.269 | 330.537 | 1.976 | 0.124 |
| -0.442 | NA | NA | + | -1.54E-04 | NA | 1.63E-01 | 7 | -158.273 | 330.547 | 1.985 | 0.123 |
| -0.576 | NA | NA | + | NA | NA | 1.96E-01 | 6 | -150.586 | 313.172 | 0 | 0.31 |
| -0.424 | NA | NA | + | NA | -6.88E-02 | 1.99E-01 | 7 | -150.01 | 314.021 | 0.849 | 0.203 |
| -0.658 | NA | NA | + | -1.08E-03 | NA | 2.01E-01 | 7 | -150.142 | 314.283 | 1.112 | 0.178 |
| -0.617 | 1.30E-09 | NA | + | NA | NA | 2.04E-01 | 7 | -150.239 | 314.477 | 1.305 | 0.162 |
| -0.496 | NA | NA | + | -1.35E-03 | -8.28E-02 | 2.06E-01 | 8 | -149.332 | 314.663 | 1.491 | 0.147 |
| -0.621 | NA | NA | + | -1.85E-03 | -1.33E-01 | 2.67E-01 | 8 | -159.84 | 335.68 | 0 | 0.469 |
| -0.548 | NA | NA | + | NA | -1.12E-01 | 2.61E-01 | 7 | -161.386 | 336.771 | 1.091 | 0.272 |
| -0.681 | 1.71E-09 | NA | + | -1.83E-03 | -1.27E-01 | 2.75E-01 | 9 | -159.433 | 336.866 | 1.186 | 0.259 |
| -0.594 | NA | NA | + | NA | NA | 2.01E-01 | 6 | -160.356 | 332.713 | 0 | 0.32 |
| -0.438 | NA | NA | + | NA | -6.84E-02 | 2.00E-01 | 7 | -159.84 | 333.681 | 0.968 | 0.197 |
| -0.671 | NA | NA | + | -1.06E-03 | NA | 2.06E-01 | 7 | -159.927 | 333.853 | 1.141 | 0.181 |
| -0.636 | 1.57E-09 | NA | + | NA | NA | 2.08E-01 | 7 | -160.013 | 334.027 | 1.314 | 0.166 |
| -0.503 | NA | NA | + | -1.30E-03 | -8.15E-02 | 2.06E-01 | 8 | -159.212 | 334.424 | 1.711 | 0.136 |
| -0.469 | NA | NA | + | NA | NA | 1.70E-01 | 6 | -156.527 | 325.054 | 0 | 0.449 |
| -0.503 | 9.77E-10 | NA | + | NA | NA | 1.77E-01 | 7 | -156.281 | 326.562 | 1.508 | 0.211 |
| -0.426 | NA | NA | + | NA | -2.02E-02 | 1.71E-01 | 7 | -156.486 | 326.971 | 1.917 | 0.172 |
| -0.487 | NA | NA | + | -2.42E-04 | NA | 1.72E-01 | 7 | -156.508 | 327.015 | 1.961 | 0.168 |
| -0.42 | NA | NA | + | NA | NA | 1.57E-01 | 6 | -164.024 | 340.048 | 0 | 0.314 |
| -0.249 | NA | NA | NA | 1.56E-03 | NA | 1.70E-01 | 4 | -166.604 | 341.208 | I.16 | 0.176 |
| -0.345 | NA | NA | NA | NA | NA | 1.98E-01 | 3 | -167.823 | 341.645 | 1.597 | 0.141 |
| -0.448 | 9.87E-10 | NA | + | NA | NA | 1.62E-01 | 7 | -163.875 | 341.751 | 1.703 | 0.134 |
| -0.394 | NA | NA | + | 3.12E-04 | NA | 1.56E-01 | 7 | -163.996 | 341.991 | 1.944 | 0.119 |
| -0.451 | NA | NA | + | NA | 1.38E-02 | 1.57E-01 | 7 | -164.007 | 342.015 | 1.967 | 0.117 |
| -0.484 | NA | NA | + | NA | NA | 1.68E-01 | 6 | -148.369 | 308.738 | 0 | 0.412 |
| -0.562 | NA | NA | + | -1.06E-03 | NA | 1.73E-01 | 7 | -147.961 | 309.923 | 1.185 | 0.228 |
| -0.518 | 1.19E-09 | NA | + | NA | NA | 1.74E-01 | 7 | -148.111 | 310.223 | 1.485 | 0.196 |
| -0.426 | NA | NA | + | NA | -2.60E-02 | 1.69E-01 | 7 | -148.296 | 310.593 | 1.855 | 0.163 |
| -0.21 | NA | NA | NA | 1.46E-03 | NA | 1.51E-01 | 4 | -151.845 | 311.69 | 0 | 0.231 |
| -0.35 | NA | NA | + | NA | NA | 1.41E-01 | 6 | -149.922 | 311.844 | 0.154 | 0.213 |
| -0.309 | NA | NA | NA | NA | NA | 1.77E-01 | 3 | -153 | 311.999 | 0.309 | 0.198 |
| -0.238 | 8.60E-10 | NA | NA | 1.44E-03 | NA | 1.56E-01 | 5 | -151.709 | 313.419 | 1.729 | 0.097 |
| -0.377 | 8.74E-10 | NA | + | NA | NA | 1.46E-01 | 7 | -149.779 | 313.558 | 1.868 | 0.091 |
| -0.338 | 9.43E-10 | NA | NA | NA | NA | 1.82E-01 | 4 | -152.839 | 313.678 | 1.988 | 0.085 |
| -0.224 | NA | NA | NA | 1.49E-03 | 7.87E-03 | 1.50E-01 | 5 | -151.839 | 313.678 | 1.988 | 0.085 |
| -0.626 | NA | NA | + | NA | NA | 2.06E-01 | 6 | -163.305 | 338.61 | 0 | 0.391 |
| -0.476 | NA | NA | + | NA | -6.70E-02 | 2.07E-01 | 7 | -162.821 | 339.642 | 1.032 | 0.234 |
| -0.666 | 1.30E-09 | NA | + | NA | NA | 2.14E-01 | 7 | -162.978 | 339.955 | 1.345 | 0.2 |
| -0.677 | NA | NA | + | -7.45E-04 | NA | 2.09E-01 | 7 | -163.106 | 340.213 | 1.603 | 0.176 |
| -0.427 | NA | NA | + | NA | NA | 1.64E-01 | 6 | -143.25 | 298.5 | 0 | 0.449 |
| -0.455 | 1.01E-09 | NA | + | NA | NA | 1.68E-01 | 7 | -143.062 | 300.124 | 1.624 | 0.199 |
| -0.36 | NA | NA | + | NA | -3.14E-02 | 1.66E-01 | 7 | -143.141 | 300.283 | 1.783 | 0.184 |
| -0.44 | NA | NA | + | -1.68E-04 | NA | 1.64E-01 | 7 | -143.239 | 300.479 | 1.979 | 0.167 |
| -0.588 | NA | NA | + | NA | NA | 2.00E-01 | 6 | -153.585 | 319.171 | 0 | 0.253 |
| -0.679 | NA | NA | + | -1.26E-03 | NA | 2.05E-01 | 7 | -152.951 | 319.902 | 0.731 | 0.175 |
| -0.426 | NA | NA | + | NA | -7.30E-02 | 2.01E-01 | 7 | -152.966 | 319.933 | 0.762 | 0.173 |
| -0.5 | NA | NA | + | -1.54E-03 | -8.96E-02 | 2.07E-01 | 8 | -152.04 | 320.08 | 0.91 | 0.16 |
| -0.636 | 1.86E-09 | NA | + | NA | NA | 2.06E-01 | 7 | -153.165 | 320.329 | 1.158 | 0.142 |
| -0.725 | 1.84E-09 | NA | + | -1.25E-03 | NA | 2.11E-01 | 8 | -152.536 | 321.072 | 1.901 | 0.098 |
| -0.379 | NA | NA | + | NA | NA | 1.49E-01 | 6 | -157.038 | 326.076 | 0 | 0.236 |
| -0.233 | NA | NA | NA | 1.43E-03 | NA | 1.60E-01 | 4 | -159.162 | 326.325 | 0.249 | 0.209 |
| -0.332 | NA | NA | NA | NA | NA | 1.88E-01 | 3 | -160.272 | 326.545 | 0.469 | 0.187 |
| -0.406 | 9.34E-10 | NA | + | NA | NA | 1.53E-01 | 7 | -156.895 | 327.791 | 1.715 | 0.1 |
| -0.432 | NA | NA | + | NA | 2.46E-02 | 1.48E-01 | 7 | -156.983 | 327.965 | I.89 | 0.092 |
| -0.356 | NA | NA | + | 2.71E-04 | NA | 1.47E-01 | 7 | -157.016 | 328.033 | 1.957 | 0.089 |
| -0.26 | 9.01E-10 | NA | NA | 1.42E-03 | NA | 1.65E-01 | 5 | -159.033 | 328.066 | I.99 | 0.087 |
| -0.563 | NA | NA | + | NA | NA | 1.97E-01 | 6 | -162.724 | 337.449 | 0 | 0.424 |
| -0.602 | 1.41E-09 | NA | + | NA | NA | 2.04E-01 | 7 | -162.449 | 338.897 | 1.448 | 0.206 |
| -0.62 | NA | NA | + | -7.43E-04 | NA | 2.01E-01 | 7 | -162.53 | 339.06 | 1.611 | 0.19 |
| -0.479 | NA | NA | + | NA | -3.73E-02 | 1.97E-01 | 7 | -162.58 | 339.159 | 1.711 | 0.18 |
| -0.407 | NA | NA | + | NA | NA | 1.48E-01 | 6 | -155.935 | 323.87 | 0 | 0.451 |
| -0.435 | 1.02E-09 | NA | + | NA | NA | 1.53E-01 | 7 | -155.785 | 325.569 | 1.699 | 0.193 |
| -0.351 | NA | NA | + | NA | -2.69E-02 | 1.50E-01 | 7 | -155.862 | 325.724 | 1.854 | 0.178 |
| -0.447 | NA | NA | + | -4.71E-04 | NA | 1.51E-01 | 7 | -155.865 | 325.73 | I.86 | 0.178 |
| -0.521 | NA | NA | + | NA | NA | 1.83E-01 | 6 | -159.174 | 330.348 | 0 | 0.427 |
| -0.586 | NA | NA | + | -8.61E-04 | NA | 1.86E-01 | 7 | -158.919 | 331.838 | I.49 | 0.203 |
| -0.559 | 1.37E-09 | NA | + | NA | NA | 1.88E-01 | 7 | -158.933 | 331.865 | 1.517 | 0.2 |
| -0.459 | NA | NA | + | NA | -2.87E-02 | 1.84E-01 | 7 | -159.092 | 332.185 | 1.837 | 0.17 |
| -0.507 | NA | NA | + | NA | NA | 1.76E-01 | 6 | -158.364 | 328.728 | 0 | 0.447 |
| -0.543 | 1.38E-09 | NA | + | NA | NA | 1.81E-01 | 7 | -158.121 | 330.242 | 1.513 | 0.21 |
| -0.46 | NA | NA | + | NA | -2.17E-02 | 1.77E-01 | 7 | -158.317 | 330.633 | 1.905 | 0.173 |
| -0.53 | NA | NA | + | -3.16E-04 | NA | 1.77E-01 | 7 | -158.331 | 330.663 | 1.934 | 0.17 |
| -0.428 | NA | NA | + | NA | NA | 1.64E-01 | 6 | -141.135 | 294.269 | 0 | 0.405 |
| -0.309 | NA | NA | + | NA | -5.46E-02 | 1.65E-01 | 7 | -140.771 | 295.541 | 1.272 | 0.215 |
| -0.461 | 1.05E-09 | NA | + | NA | NA | 1.70E-01 | 7 | -140.845 | 295.689 | I.42 | 0.199 |
| -0.487 | NA | NA | + | -7.14E-04 | NA | 1.68E-01 | 7 | -140.941 | 295.881 | 1.612 | 0.181 |
| -0.21 | NA | NA | NA | 1.50E-03 | NA | 1.55E-01 | 4 | -153.006 | 314.012 | 0 | 0.294 |
| -0.366 | NA | NA | + | NA | NA | 1.49E-01 | 6 | -151.162 | 314.324 | 0.312 | 0.252 |
| -0.309 | NA | NA | NA | NA | NA | 1.82E-01 | 3 | -154.296 | 314.592 | 0.58 | 0.22 |
| -0.238 | 7.82E-10 | NA | NA | 1.48E-03 | NA | 1.60E-01 | 5 | -152.858 | 315.716 | 1.705 | 0.125 |
| -0.22 | NA | NA | NA | 1.53E-03 | 5.98E-03 | 1.54E-01 | 5 | -153.002 | 316.005 | 1.993 | 0.109 |
| -0.518 | NA | NA | + | NA | NA | 1.79E-01 | 6 | -158.524 | 329.048 | 0 | 0.42 |
| -0.588 | NA | NA | + | -9.19E-04 | NA | 1.83E-01 | 7 | -158.235 | 330.47 | 1.422 | 0.206 |
| -0.554 | 1.31E-09 | NA | + | NA | NA | 1.85E-01 | 7 | -158.26 | 330.52 | 1.472 | 0.201 |
| -0.446 | NA | NA | + | NA | -3.31E-02 | 1.81E-01 | 7 | -158.41 | 330.819 | 1.771 | 0.173 |
| -0.415 | NA | NA | + | NA | NA | 1.63E-01 | 6 | -146.633 | 305.267 | 0 | 0.449 |
| -0.443 | 8.77E-10 | NA | + | NA | NA | 1.69E-01 | 7 | -146.424 | 306.848 | 1.581 | 0.204 |
| -0.37 | NA | NA | + | NA | -2.21E-02 | 1.65E-01 | 7 | -146.58 | 307.159 | 1.893 | 0.174 |
| -0.445 | NA | NA | + | -3.83E-04 | NA | 1.65E-01 | 7 | -146.583 | 307.166 | 1.899 | 0.174 |
| -0.476 | NA | NA | + | NA | NA | 1.75E-01 | 6 | -156.116 | 324.232 | 0 | 0.448 |
| -0.508 | 1.22E-09 | NA | + | NA | NA | 1.80E-01 | 7 | -155.898 | 325.797 | 1.565 | 0.205 |
| -0.421 | NA | NA | + | NA | -2.44E-02 | 1.74E-01 | 7 | -156.056 | 326.112 | I.88 | 0.175 |
| -0.507 | NA | NA | + | -3.75E-04 | NA | 1.77E-01 | 7 | -156.068 | 326.137 | 1.905 | 0.173 |
| -0.552 | NA | NA | + | NA | NA | 1.91E-01 | 6 | -157.599 | 327.198 | 0 | 0.313 |
| -0.643 | NA | NA | + | -1.25E-03 | NA | 1.95E-01 | 7 | -157.024 | 328.049 | 0.851 | 0.204 |
| -0.414 | NA | NA | + | NA | -6.29E-02 | 1.92E-01 | 7 | -157.146 | 328.292 | 1.094 | 0.181 |
| -0.591 | 1.14E-09 | NA | + | NA | NA | 2.00E-01 | 7 | -157.282 | 328.564 | 1.367 | 0.158 |
| -0.491 | NA | NA | + | -1.46E-03 | -7.61E-02 | 1.98E-01 | 8 | -156.372 | 328.743 | 1.545 | 0.144 |
| -0.401 | NA | NA | + | NA | NA | 1.55E-01 | 6 | -161.985 | 335.97 | 0 | 0.282 |
| -0.338 | NA | NA | NA | NA | NA | 1.94E-01 | 3 | -165.358 | 336.717 | 0.747 | 0.194 |
| -0.253 | NA | NA | NA | 1.38E-03 | NA | 1.69E-01 | 4 | -164.374 | 336.748 | 0.779 | 0.191 |
| -0.429 | 1.04E-09 | NA | + | NA | NA | 1.59E-01 | 7 | -161.835 | 337.669 | 01.VII | 0.12 |
| -0.454 | NA | NA | + | NA | 2.45E-02 | 1.53E-01 | 7 | -161.934 | 337.868 | 1.898 | 0.109 |
| -0.388 | NA | NA | + | 1.54E-04 | NA | 1.54E-01 | 7 | -161.978 | 337.956 | 1.986 | 0.104 |
| -0.488 | NA | NA | + | NA | NA | 1.77E-01 | 6 | -144.055 | 300.109 | 0 | 0.389 |
| -0.353 | NA | NA | + | NA | -6.25E-02 | 1.79E-01 | 7 | -143.575 | 301.15 | 1.041 | 0.231 |
| -0.523 | 1.30E-09 | NA | + | NA | NA | 1.82E-01 | 7 | -143.761 | 301.522 | 1.413 | 0.192 |
| -0.553 | NA | NA | + | -8.11E-04 | NA | 1.81E-01 | 7 | -143.778 | 301.556 | 1.447 | 0.189 |
| -0.611 | NA | NA | + | NA | NA | 2.06E-01 | 6 | -153.358 | 318.715 | 0 | 0.253 |
| -0.703 | NA | NA | + | -1.28E-03 | NA | 2.11E-01 | 7 | -152.665 | 319.331 | 0.616 | 0.186 |
| -0.458 | NA | NA | + | NA | -6.76E-02 | 2.07E-01 | 7 | -152.8 | 319.6 | 0.885 | 0.163 |
| -0.53 | NA | NA | + | -1.55E-03 | -8.47E-02 | 2.13E-01 | 8 | -151.812 | 319.623 | 0.908 | 0.161 |
| -0.651 | 1.34E-09 | NA | + | NA | NA | 2.14E-01 | 7 | -152.988 | 319.975 | I.26 | 0.135 |
| -0.746 | 1.39E-09 | NA | + | -1.30E-03 | NA | 2.19E-01 | 8 | -152.268 | 320.536 | 1.821 | 0.102 |
| -0.405 | NA | NA | + | NA | NA | 1.57E-01 | 6 | -155.805 | 323.61 | 0 | 0.301 |
| -0.351 | NA | NA | NA | NA | NA | 1.98E-01 | 3 | -159.321 | 324.642 | 1.031 | 0.18 |
| -0.265 | NA | NA | NA | 1.29E-03 | NA | 1.72E-01 | 4 | -158.378 | 324.756 | 1.146 | 0.17 |
| -0.432 | 9.93E-10 | NA | + | NA | NA | 1.60E-01 | 7 | -155.68 | 325.36 | 1.749 | 0.126 |
| -0.43 | NA | NA | + | NA | 1.15E-02 | 1.56E-01 | 7 | -155.793 | 325.586 | 1.975 | 0.112 |
| -0.408 | NA | NA | + | -3.21E-05 | NA | 1.57E-01 | 7 | -155.805 | 325.61 | 1.999 | 0.111 |
| -0.442 | NA | NA | + | NA | NA | 1.63E-01 | 6 | -141.952 | 295.903 | 0 | 0.422 |
| -0.342 | NA | NA | + | NA | -4.66E-02 | 1.66E-01 | 7 | -141.688 | 297.376 | 1.472 | 0.202 |
| -0.473 | 1.18E-09 | NA | + | NA | NA | 1.68E-01 | 7 | -141.749 | 297.497 | 1.594 | 0.19 |
| -0.499 | NA | NA | + | -6.85E-04 | NA | 1.67E-01 | 7 | -141.774 | 297.547 | 1.644 | 0.186 |
| -0.529 | NA | NA | + | NA | NA | 1.85E-01 | 6 | -153.097 | 318.194 | 0 | 0.398 |
| -0.605 | NA | NA | + | -9.66E-04 | NA | 1.90E-01 | 7 | -152.749 | 319.498 | 1.304 | 0.207 |
| -0.569 | 1.43E-09 | NA | + | NA | NA | 1.91E-01 | 7 | -152.794 | 319.587 | 1.393 | 0.198 |
| -0.415 | NA | NA | + | NA | -5.21E-02 | 1.87E-01 | 7 | -152.801 | 319.602 | 1.408 | 0.197 |
| -0.387 | NA | NA | + | NA | NA | 1.48E-01 | 6 | -142.536 | 297.072 | 0 | 0.446 |
| -0.417 | 1.06E-09 | NA | + | NA | NA | 1.53E-01 | 7 | -142.348 | 298.696 | 1.624 | 0.198 |
| -0.431 | NA | NA | + | -5.41E-04 | NA | 1.51E-01 | 7 | -142.433 | 298.866 | 1.794 | 0.182 |
| -0.341 | NA | NA | + | NA | -2.21E-02 | 1.50E-01 | 7 | -142.482 | 298.964 | 1.892 | 0.173 |
| -0.409 | NA | NA | + | NA | NA | 1.54E-01 | 6 | -144.307 | 300.614 | 0 | 0.44 |
| -0.441 | 1.14E-09 | NA | + | NA | NA | 1.59E-01 | 7 | -144.099 | 302.199 | 1.584 | 0.199 |
| -0.461 | NA | NA | + | -6.01E-04 | NA | 1.57E-01 | 7 | -144.179 | 302.357 | 1.743 | 0.184 |
| -0.349 | NA | NA | + | NA | -2.76E-02 | 1.55E-01 | 7 | -144.225 | 302.45 | 1.836 | 0.176 |
| -0.721 | NA | NA | + | -1.68E-03 | NA | 2.08E-01 | 7 | -152.325 | 318.649 | 0 | 0.21 |
| -0.547 | NA | NA | + | -1.94E-03 | -8.68E-02 | 2.10E-01 | 8 | -151.444 | 318.888 | 0.239 | 0.187 |
| -0.613 | NA | NA | + | NA | NA | 2.05E-01 | 6 | -153.503 | 319.006 | 0.357 | 0.176 |
| -0.766 | 1.59E-09 | NA | + | -1.69E-03 | NA | 2.16E-01 | 8 | -151.882 | 319.764 | 1.115 | 0.12 |
| -0.468 | NA | NA | + | NA | -6.65E-02 | 2.07E-01 | 7 | -152.978 | 319.957 | 1.308 | 0.109 |
| -0.659 | 1.58E-09 | NA | + | NA | NA | 2.13E-01 | 7 | -153.073 | 320.145 | 1.496 | 0.1 |
| -0.596 | 1.42E-09 | NA | + | -1.93E-03 | -8.25E-02 | 2.17E-01 | 9 | -151.087 | 320.175 | 1.526 | 0.098 |
| -0.593 | NA | NA | + | -1.97E-03 | -9.94E-02 | 2.32E-01 | 8 | -157.413 | 330.825 | 0 | 0.219 |
| -0.802 | NA | NA | + | -1.67E-03 | NA | 2.33E-01 | 7 | -158.562 | 331.125 | 0.3 | 0.189 |
| -0.686 | NA | NA | + | NA | NA | 2.29E-01 | 6 | -159.722 | 331.445 | 0.619 | 0.161 |
| -0.505 | NA | NA | + | NA | -7.85E-02 | 2.28E-01 | 7 | -158.994 | 331.988 | 1.163 | 0.123 |
| -0.645 | 1.49E-09 | NA | + | -1.96E-03 | -9.44E-02 | 2.39E-01 | 9 | -157.09 | 332.18 | 1.355 | 0.111 |
| -0.851 | 1.75E-09 | NA | + | -1.67E-03 | NA | 2.41E-01 | 8 | -158.123 | 332.246 | 1.421 | 0.108 |
| -0.735 | 1.73E-09 | NA | + | NA | NA | 2.36E-01 | 7 | -159.301 | 332.602 | 1.777 | 0.09 |
| -0.534 | NA | NA | + | NA | NA | 1.83E-01 | 6 | -162.349 | 336.698 | 0 | 0.429 |
| -0.604 | NA | NA | + | -8.67E-04 | NA | 1.88E-01 | 7 | -162.102 | 338.203 | 1.506 | 0.202 |
| -0.568 | 1.27E-09 | NA | + | NA | NA | 1.88E-01 | 7 | -162.137 | 338.274 | 1.576 | 0.195 |
| -0.465 | NA | NA | + | NA | -3.12E-02 | 1.83E-01 | 7 | -162.253 | 338.506 | 1.808 | 0.174 |
| -0.681 | NA | NA | + | NA | NA | 2.18E-01 | 6 | -165.664 | 343.327 | 0 | 0.21 |
| -0.781 | NA | NA | + | -1.48E-03 | NA | 2.22E-01 | 7 | -164.848 | 343.697 | 0.369 | 0.175 |
| -0.593 | NA | NA | + | -1.75E-03 | -9.19E-02 | 2.24E-01 | 8 | -163.929 | 343.858 | 0.531 | 0.161 |
| -0.514 | NA | NA | + | NA | -7.41E-02 | 2.18E-01 | 7 | -165.055 | 344.109 | 0.782 | 0.142 |
| -0.731 | 1.69E-09 | NA | + | NA | NA | 2.26E-01 | 7 | -165.2 | 344.4 | 1.073 | 0.123 |
| -0.831 | 1.69E-09 | NA | + | -1.48E-03 | NA | 2.31E-01 | 8 | -164.38 | 344.759 | 1.432 | 0.103 |
| -0.647 | 1.51E-09 | NA | + | -1.74E-03 | -8.72E-02 | 2.32E-01 | 9 | -163.553 | 345.107 | 1.779 | 0.086 |
| -0.498 | NA | NA | + | NA | NA | 1.77E-01 | 6 | -168.559 | 349.117 | 0 | 0.455 |
| -0.536 | 1.42E-09 | NA | + | NA | NA | 1.83E-01 | 7 | -168.335 | 350.669 | 1.552 | 0.21 |
| -0.495 | NA | NA | + | NA | -1.20E-03 | 1.77E-01 | 7 | -168.559 | 351.117 | 2 | 0.168 |
| -0.498 | NA | NA | + | -2.52E-06 | NA | 1.77E-01 | 7 | -168.559 | 351.117 | 2 | 0.168 |
| -0.453 | NA | NA | + | NA | NA | 1.70E-01 | 6 | -155.775 | 323.551 | 0 | 0.442 |
| -0.489 | 9.02E-10 | NA | + | NA | NA | 1.79E-01 | 7 | -155.512 | 325.023 | 1.473 | 0.212 |
| -0.397 | NA | NA | + | NA | -2.61E-02 | 1.71E-01 | 7 | -155.705 | 325.411 | I.86 | 0.174 |
| -0.483 | NA | NA | + | -3.89E-04 | NA | 1.72E-01 | 7 | -155.723 | 325.446 | 1.895 | 0.171 |
| -0.334 | NA | NA | + | NA | NA | 1.31E-01 | 6 | -143.439 | 298.877 | 0 | 0.325 |
| -0.277 | NA | NA | NA | NA | NA | 1.68E-01 | 3 | -147.149 | 300.299 | 1.421 | 0.16 |
| -0.359 | 7.04E-10 | NA | + | NA | NA | 1.36E-01 | 7 | -143.297 | 300.593 | 1.716 | 0.138 |
| -0.194 | NA | NA | NA | 1.17E-03 | NA | 1.45E-01 | 4 | -146.341 | 300.682 | 1.805 | 0.132 |
| -0.362 | NA | NA | + | -3.22E-04 | NA | 1.33E-01 | 7 | -143.406 | 300.811 | 1.934 | 0.124 |
| -0.369 | NA | NA | + | NA | 1.61E-02 | 1.30E-01 | 7 | -143.413 | 300.826 | 1.949 | 0.123 |
| -0.669 | NA | NA | + | NA | NA | 2.18E-01 | 6 | -169.955 | 351.909 | 0 | 0.404 |
| -0.719 | 1.69E-09 | NA | + | NA | NA | 2.27E-01 | 7 | -169.567 | 353.134 | 1.225 | 0.219 |
| -0.555 | NA | NA | + | NA | -4.92E-02 | 2.18E-01 | 7 | -169.713 | 353.426 | 1.516 | 0.189 |
| -0.725 | NA | NA | + | -8.24E-04 | NA | 2.22E-01 | 7 | -169.722 | 353.445 | 1.536 | 0.188 |
| -0.371 | NA | NA | + | NA | NA | 1.50E-01 | 6 | -136.119 | 284.238 | 0 | 0.368 |
| -0.402 | 9.87E-10 | NA | + | NA | NA | 1.56E-01 | 7 | -135.895 | 285.789 | 1.551 | 0.169 |
| -0.288 | NA | NA | + | NA | -3.83E-02 | 1.51E-01 | 7 | -135.942 | 285.883 | 1.646 | 0.161 |
| -0.325 | NA | NA | NA | NA | NA | 1.87E-01 | 3 | -139.994 | 285.989 | 1.751 | 0.153 |
| -0.415 | NA | NA | + | -5.16E-04 | NA | 1.54E-01 | 7 | -136.024 | 286.047 | I.81 | 0.149 |
| -0.431 | NA | NA | + | NA | NA | 1.63E-01 | 6 | -149.932 | 311.863 | 0 | 0.449 |
| -0.462 | 9.98E-10 | NA | + | NA | NA | 1.68E-01 | 7 | -149.724 | 313.447 | 1.584 | 0.203 |
| -0.475 | NA | NA | + | -5.47E-04 | NA | 1.66E-01 | 7 | -149.835 | 313.669 | 1.806 | 0.182 |
| -0.448 | NA | NA | + | NA | 8.04E-03 | 1.62E-01 | 7 | -149.925 | 313.851 | 1.987 | 0.166 |
| -0.492 | NA | NA | + | NA | NA | 1.75E-01 | 6 | -156.484 | 324.967 | 0 | 0.43 |
| -0.528 | 1.20E-09 | NA | + | NA | NA | 1.81E-01 | 7 | -156.232 | 326.464 | 1.497 | 0.204 |
| -0.546 | NA | NA | + | -7.15E-04 | NA | 1.78E-01 | 7 | -156.31 | 326.619 | 1.652 | 0.188 |
| -0.418 | NA | NA | + | NA | -3.36E-02 | 1.76E-01 | 7 | -156.369 | 326.738 | 1.771 | 0.178 |
| -0.672 | NA | NA | + | NA | NA | 2.29E-01 | 6 | -159.086 | 330.173 | 0 | 0.2 |
| -0.566 | NA | NA | + | -1.62E-03 | -9.58E-02 | 2.34E-01 | 8 | -157.358 | 330.716 | 0.543 | 0.152 |
| -0.76 | NA | NA | + | -1.29E-03 | NA | 2.33E-01 | 7 | -158.389 | 330.778 | 0.605 | 0.148 |
| -0.502 | NA | NA | + | NA | -7.52E-02 | 2.28E-01 | 7 | -158.429 | 330.858 | 0.685 | 0.142 |
| -0.725 | 2.04E-09 | NA | + | NA | NA | 2.36E-01 | 7 | -158.617 | 331.233 | 01.VI | 0.118 |
| -0.812 | 2.03E-09 | NA | + | -1.28E-03 | NA | 2.41E-01 | 8 | -157.92 | 331.84 | 1.667 | 0.087 |
| -0.622 | 1.74E-09 | NA | + | -1.60E-03 | -9.00E-02 | 2.40E-01 | 9 | -157.015 | 332.03 | 1.857 | 0.079 |
| -0.562 | 1.82E-09 | NA | + | NA | -6.95E-02 | 2.35E-01 | 8 | -158.058 | 332.116 | 1.943 | 0.076 |
| -0.473 | NA | NA | + | NA | NA | 1.71E-01 | 6 | -150.623 | 313.245 | 0 | 0.426 |
| -0.511 | 1.38E-09 | NA | + | NA | NA | 1.77E-01 | 7 | -150.337 | 314.674 | 1.429 | 0.208 |
| -0.375 | NA | NA | + | NA | -4.47E-02 | 1.73E-01 | 7 | -150.406 | 314.812 | 1.567 | 0.194 |
| -0.515 | NA | NA | + | -5.17E-04 | NA | 1.74E-01 | 7 | -150.531 | 315.061 | 1.816 | 0.172 |
| -0.585 | NA | NA | + | -1.58E-03 | -1.16E-01 | 2.49E-01 | 8 | -167.228 | 350.455 | 0 | 0.187 |
| -0.53 | NA | NA | + | NA | -9.88E-02 | 2.47E-01 | 7 | -168.306 | 350.613 | 0.157 | 0.173 |
| -0.759 | NA | NA | + | NA | NA | 2.51E-01 | 6 | -169.475 | 350.95 | 0.494 | 0.146 |
| -0.654 | 2.03E-09 | NA | + | -1.55E-03 | -1.09E-01 | 2.57E-01 | 9 | -166.784 | 351.568 | 1.112 | 0.107 |
| -0.823 | 2.50E-09 | NA | + | NA | NA | 2.60E-01 | 7 | -168.812 | 351.623 | 1.168 | 0.104 |
| -0.834 | NA | NA | + | -1.23E-03 | NA | 2.53E-01 | 7 | -168.815 | 351.63 | 1.174 | 0.104 |
| -0.602 | 2.13E-09 | NA | + | NA | -9.11E-02 | 2.55E-01 | 8 | -167.825 | 351.649 | 1.194 | 0.103 |
| -0.898 | 2.47E-09 | NA | + | -1.22E-03 | NA | 2.62E-01 | 8 | -168.157 | 352.315 | 1.859 | 0.074 |
| -0.397 | NA | NA | + | NA | NA | 1.58E-01 | 6 | -145.022 | 302.044 | 0 | 0.311 |
| -0.348 | NA | NA | NA | NA | NA | 1.94E-01 | 3 | -148.617 | 303.234 | 1.191 | 0.171 |
| -0.261 | NA | NA | NA | 1.23E-03 | NA | 1.71E-01 | 4 | -147.697 | 303.395 | 1.351 | 0.158 |
| -0.422 | 8.17E-10 | NA | + | NA | NA | 1.62E-01 | 7 | -144.882 | 303.765 | 1.721 | 0.131 |
| -0.406 | NA | NA | + | -1.01E-04 | NA | 1.58E-01 | 7 | -145.019 | 304.037 | 1.993 | 0.115 |
| -0.396 | NA | NA | + | NA | -6.84E-04 | 1.58E-01 | 7 | -145.022 | 304.044 | 2 | 0.114 |
| -0.533 | NA | NA | + | NA | NA | 1.87E-01 | 6 | -159.113 | 330.226 | 0 | 0.41 |
| -0.61 | NA | NA | + | -1.03E-03 | NA | 1.91E-01 | 7 | -158.733 | 331.466 | 1.239 | 0.22 |
| -0.571 | 1.35E-09 | NA | + | NA | NA | 1.93E-01 | 7 | -158.829 | 331.657 | 1.431 | 0.2 |
| -0.459 | NA | NA | + | NA | -3.35E-02 | 1.88E-01 | 7 | -158.996 | 331.992 | 1.765 | 0.169 |
| -0.521 | NA | NA | + | NA | NA | 1.83E-01 | 6 | -146.225 | 304.45 | 0 | 0.322 |
| -0.615 | NA | NA | + | -1.24E-03 | NA | 1.88E-01 | 7 | -145.615 | 305.23 | 0.78 | 0.218 |
| -0.401 | NA | NA | + | NA | -5.45E-02 | 1.84E-01 | 7 | -145.879 | 305.757 | 1.307 | 0.167 |
| -0.555 | 1.26E-09 | NA | + | NA | NA | 1.89E-01 | 7 | -145.958 | 305.915 | 1.465 | 0.155 |
| -0.479 | NA | NA | + | -1.44E-03 | -6.90E-02 | 1.91E-01 | 8 | -145.072 | 306.144 | 1.694 | 0.138 |
| -0.428 | NA | NA | + | NA | NA | 1.61E-01 | 6 | -143.552 | 299.104 | 0 | 0.418 |
| -0.326 | NA | NA | + | NA | -4.60E-02 | 1.62E-01 | 7 | -143.31 | 300.621 | 1.517 | 0.196 |
| -0.46 | 1.01E-09 | NA | + | NA | NA | 1.67E-01 | 7 | -143.321 | 300.643 | 1.539 | 0.194 |
| -0.495 | NA | NA | + | -7.92E-04 | NA | 1.66E-01 | 7 | -143.328 | 300.655 | 1.551 | 0.192 |
| -0.453 | NA | NA | + | NA | NA | 1.64E-01 | 6 | -153.35 | 318.7 | 0 | 0.43 |
| -0.489 | 1.31E-09 | NA | + | NA | NA | 1.69E-01 | 7 | -153.11 | 320.22 | I.52 | 0.201 |
| -0.352 | NA | NA | + | NA | -4.59E-02 | 1.64E-01 | 7 | -153.12 | 320.239 | 1.539 | 0.199 |
| -0.49 | NA | NA | + | -4.56E-04 | NA | 1.66E-01 | 7 | -153.277 | 320.555 | 1.854 | 0.17 |
| -0.459 | NA | NA | + | NA | NA | 1.67E-01 | 6 | -148.246 | 308.493 | 0 | 0.434 |
| -0.494 | 1.09E-09 | NA | + | NA | NA | 1.74E-01 | 7 | -148.007 | 310.015 | 1.522 | 0.203 |
| -0.371 | NA | NA | + | NA | -3.90E-02 | 1.68E-01 | 7 | -148.079 | 310.157 | 1.665 | 0.189 |
| -0.499 | NA | NA | + | -4.94E-04 | NA | 1.70E-01 | 7 | -148.159 | 310.318 | 1.825 | 0.174 |
| -0.389 | NA | NA | + | NA | NA | 1.52E-01 | 6 | -145.054 | 302.108 | 0 | 0.377 |
| -0.32 | NA | NA | NA | NA | NA | 1.88E-01 | 3 | -148.807 | 303.614 | 1.506 | 0.177 |
| -0.414 | 9.40E-10 | NA | + | NA | NA | 1.56E-01 | 7 | -144.912 | 303.824 | 1.715 | 0.16 |
| -0.425 | NA | NA | + | -4.28E-04 | NA | 1.55E-01 | 7 | -144.992 | 303.985 | 1.877 | 0.147 |
| -0.377 | NA | NA | + | NA | -5.57E-03 | 1.53E-01 | 7 | -145.051 | 304.102 | 1.994 | 0.139 |
| -0.563 | NA | NA | + | NA | NA | 1.94E-01 | 6 | -152.186 | 316.372 | 0 | 0.214 |
| -0.671 | NA | NA | + | -1.39E-03 | NA | 2.00E-01 | 7 | -151.397 | 316.794 | 0.422 | 0.173 |
| -0.488 | NA | NA | + | -1.67E-03 | -8.99E-02 | 2.01E-01 | 8 | -150.412 | 316.823 | 0.451 | 0.171 |
| -0.398 | NA | NA | + | NA | -7.28E-02 | 1.93E-01 | 7 | -151.526 | 317.052 | 0.68 | 0.153 |
| -0.604 | 1.43E-09 | NA | + | NA | NA | 2.01E-01 | 7 | -151.834 | 317.668 | 1.296 | 0.112 |
| -0.714 | 1.47E-09 | NA | + | -1.41E-03 | NA | 2.07E-01 | 8 | -151.019 | 318.038 | 1.666 | 0.093 |
| -0.534 | 1.27E-09 | NA | + | -1.67E-03 | -8.56E-02 | 2.07E-01 | 9 | -150.127 | 318.255 | 1.882 | 0.084 |
| -0.434 | NA | NA | + | NA | NA | 1.61E-01 | 6 | -143.327 | 298.654 | 0 | 0.432 |
| -0.494 | NA | NA | + | -7.08E-04 | NA | 1.66E-01 | 7 | -143.138 | 300.275 | 1.621 | 0.192 |
| -0.463 | 1.12E-09 | NA | + | NA | NA | 1.65E-01 | 7 | -143.156 | 300.312 | 1.659 | 0.188 |
| -0.349 | NA | NA | + | NA | -3.82E-02 | 1.62E-01 | 7 | -143.156 | 300.313 | 1.659 | 0.188 |
| -0.576 | NA | NA | + | NA | NA | 1.95E-01 | 6 | -153.042 | 318.084 | 0 | 0.288 |
| -0.414 | NA | NA | + | NA | -7.48E-02 | 1.97E-01 | 7 | -152.35 | 318.7 | 0.616 | 0.212 |
| -0.656 | NA | NA | + | -1.13E-03 | NA | 1.99E-01 | 7 | -152.509 | 319.018 | 0.934 | 0.181 |
| -0.484 | NA | NA | + | -1.39E-03 | -8.83E-02 | 2.03E-01 | 8 | -151.563 | 319.127 | 1.043 | 0.171 |
| -0.614 | 1.11E-09 | NA | + | NA | NA | 2.04E-01 | 7 | -152.708 | 319.416 | 1.332 | 0.148 |
| -0.393 | NA | NA | + | NA | NA | 1.49E-01 | 6 | -155.825 | 323.649 | 0 | 0.313 |
| -0.24 | NA | NA | NA | 1.42E-03 | NA | 1.63E-01 | 4 | -158.463 | 324.927 | 1.278 | 0.165 |
| -0.352 | NA | NA | NA | NA | NA | 1.94E-01 | 3 | -159.578 | 325.155 | 1.506 | 0.147 |
| -0.424 | 1.11E-09 | NA | + | NA | NA | 1.54E-01 | 7 | -155.645 | 325.29 | 1.641 | 0.138 |
| -0.342 | NA | NA | + | NA | -2.29E-02 | 1.49E-01 | 7 | -155.773 | 325.546 | 1.897 | 0.121 |
| -0.393 | NA | NA | + | -3.50E-06 | NA | 1.49E-01 | 7 | -155.825 | 325.649 | 2 | 0.115 |
| -0.509 | NA | NA | + | NA | NA | 1.69E-01 | 6 | -157.954 | 327.909 | 0 | 0.443 |
| -0.543 | 1.25E-09 | NA | + | NA | NA | 1.75E-01 | 7 | -157.749 | 329.498 | 1.589 | 0.2 |
| -0.551 | NA | NA | + | -5.26E-04 | NA | 1.72E-01 | 7 | -157.861 | 329.721 | 1.812 | 0.179 |
| -0.441 | NA | NA | + | NA | -2.98E-02 | 1.70E-01 | 7 | -157.865 | 329.731 | 1.822 | 0.178 |
| -0.495 | NA | NA | + | NA | NA | 1.77E-01 | 6 | -159.013 | 330.027 | 0 | 0.442 |
| -0.529 | 1.28E-09 | NA | + | NA | NA | 1.82E-01 | 7 | -158.818 | 331.635 | 1.608 | 0.198 |
| -0.552 | NA | NA | + | -7.39E-04 | NA | 1.81E-01 | 7 | -158.834 | 331.667 | I.64 | 0.195 |
| -0.463 | NA | NA | + | NA | -1.45E-02 | 1.77E-01 | 7 | -158.993 | 331.987 | I.96 | 0.166 |
| -0.465 | NA | NA | + | NA | NA | 1.73E-01 | 6 | -146.536 | 305.071 | 0 | 0.43 |
| -0.499 | 1.27E-09 | NA | + | NA | NA | 1.78E-01 | 7 | -146.282 | 306.563 | 1.492 | 0.204 |
| -0.383 | NA | NA | + | NA | -3.78E-02 | 1.74E-01 | 7 | -146.376 | 306.752 | I.68 | 0.186 |
| -0.513 | NA | NA | + | -6.10E-04 | NA | 1.76E-01 | 7 | -146.4 | 306.8 | 1.729 | 0.181 |
| -0.544 | NA | NA | + | NA | NA | 1.89E-01 | 6 | -165.155 | 342.311 | 0 | 0.441 |
| -0.583 | 1.34E-09 | NA | + | NA | NA | 1.96E-01 | 7 | -164.91 | 343.82 | 1.509 | 0.207 |
| -0.476 | NA | NA | + | NA | -3.02E-02 | 1.89E-01 | 7 | -165.069 | 344.137 | 1.827 | 0.177 |
| -0.578 | NA | NA | + | -4.64E-04 | NA | 1.91E-01 | 7 | -165.081 | 344.162 | 1.851 | 0.175 |
| -0.366 | NA | NA | + | NA | NA | 1.46E-01 | 6 | -140.147 | 292.295 | 0 | 0.333 |
| -0.308 | NA | NA | NA | NA | NA | 1.81E-01 | 3 | -143.945 | 293.889 | 1.595 | 0.15 |
| -0.392 | 7.53E-10 | NA | + | NA | NA | 1.51E-01 | 7 | -139.995 | 293.989 | 1.695 | 0.143 |
| -0.395 | NA | NA | + | -3.34E-04 | NA | 1.48E-01 | 7 | -140.109 | 294.218 | 1.923 | 0.127 |
| -0.234 | NA | NA | NA | 1.12E-03 | NA | 1.60E-01 | 4 | -143.133 | 294.267 | 1.972 | 0.124 |
| -0.373 | NA | NA | + | NA | 3.39E-03 | 1.46E-01 | 7 | -140.146 | 294.292 | 1.998 | 0.123 |
| -0.57 | NA | NA | + | NA | NA | 1.98E-01 | 6 | -155.889 | 323.778 | 0 | 0.383 |
| -0.647 | NA | NA | + | -1.03E-03 | NA | 2.03E-01 | 7 | -155.496 | 324.992 | 1.214 | 0.209 |
| -0.612 | 1.24E-09 | NA | + | NA | NA | 2.07E-01 | 7 | -155.498 | 324.996 | 1.218 | 0.208 |
| -0.442 | NA | NA | + | NA | -5.68E-02 | 1.99E-01 | 7 | -155.534 | 325.068 | I.29 | 0.201 |
| -0.359 | NA | NA | + | NA | NA | 1.39E-01 | 6 | -151.348 | 314.697 | 0 | 0.301 |
| -0.207 | NA | NA | NA | 1.41E-03 | NA | 1.54E-01 | 4 | -153.827 | 315.654 | 0.958 | 0.186 |
| -0.299 | NA | NA | NA | NA | NA | 1.80E-01 | 3 | -154.959 | 315.919 | 1.222 | 0.163 |
| -0.387 | 8.92E-10 | NA | + | NA | NA | 1.44E-01 | 7 | -151.208 | 316.416 | 1.719 | 0.127 |
| -0.356 | NA | NA | + | 3.46E-05 | NA | 1.39E-01 | 7 | -151.348 | 316.696 | 1.999 | 0.111 |
| -0.356 | NA | NA | + | NA | -1.34E-03 | 1.39E-01 | 7 | -151.348 | 316.696 | 2 | 0.111 |
| -0.563 | NA | NA | + | NA | NA | 1.84E-01 | 6 | -168.896 | 349.791 | 0 | 0.432 |
| -0.603 | 1.49E-09 | NA | + | NA | NA | 1.90E-01 | 7 | -168.641 | 351.281 | I.49 | 0.205 |
| -0.616 | NA | NA | + | -6.88E-04 | NA | 1.88E-01 | 7 | -168.75 | 351.5 | 1.708 | 0.184 |
| -0.482 | NA | NA | + | NA | -3.56E-02 | 1.84E-01 | 7 | -168.779 | 351.558 | 1.766 | 0.179 |
| -0.491 | NA | NA | + | NA | NA | 1.75E-01 | 6 | -152.896 | 317.793 | 0 | 0.436 |
| -0.528 | 1.33E-09 | NA | + | NA | NA | 1.81E-01 | 7 | -152.626 | 319.251 | 1.458 | 0.21 |
| -0.534 | NA | NA | + | -5.39E-04 | NA | 1.77E-01 | 7 | -152.79 | 319.581 | 1.788 | 0.178 |
| -0.426 | NA | NA | + | NA | -2.91E-02 | 1.76E-01 | 7 | -152.81 | 319.62 | 1.827 | 0.175 |
| -0.451 | NA | NA | + | NA | NA | 1.60E-01 | 6 | -149.959 | 311.919 | 0 | 0.435 |
| -0.482 | 1.09E-09 | NA | + | NA | NA | 1.65E-01 | 7 | -149.742 | 313.484 | 1.565 | 0.199 |
| -0.356 | NA | NA | + | NA | -4.36E-02 | 1.62E-01 | 7 | -149.761 | 313.521 | 1.602 | 0.195 |
| -0.483 | NA | NA | + | -4.01E-04 | NA | 1.61E-01 | 7 | -149.9 | 313.801 | 1.882 | 0.17 |
| -0.535 | NA | NA | + | NA | NA | 1.86E-01 | 6 | -156.111 | 324.223 | 0 | 0.419 |
| -0.601 | NA | NA | + | -8.44E-04 | NA | 1.91E-01 | 7 | -155.858 | 325.716 | 1.493 | 0.198 |
| -0.572 | 1.43E-09 | NA | + | NA | NA | 1.91E-01 | 7 | -155.862 | 325.725 | 1.502 | 0.198 |
| -0.44 | NA | NA | + | NA | -4.17E-02 | 1.86E-01 | 7 | -155.926 | 325.851 | 1.628 | 0.185 |
| -0.391 | NA | NA | + | NA | NA | 1.45E-01 | 6 | -159.172 | 330.343 | 0 | 0.303 |
| -0.323 | NA | NA | NA | NA | NA | 1.84E-01 | 3 | -162.734 | 331.469 | 1.125 | 0.172 |
| -0.23 | NA | NA | NA | 1.40E-03 | NA | 1.58E-01 | 4 | -161.756 | 331.511 | 1.168 | 0.169 |
| -0.42 | 9.39E-10 | NA | + | NA | NA | 1.50E-01 | 7 | -159.016 | 332.033 | 1.689 | 0.13 |
| -0.433 | NA | NA | + | NA | 1.90E-02 | 1.44E-01 | 7 | -159.141 | 332.282 | 1.938 | 0.115 |
| -0.392 | NA | NA | + | -1.02E-05 | NA | 1.45E-01 | 7 | -159.172 | 332.343 | 2 | 0.111 |
| -0.532 | NA | NA | + | NA | NA | 1.85E-01 | 6 | -143.729 | 299.459 | 0 | 0.251 |
| -0.636 | NA | NA | + | -1.42E-03 | NA | 1.91E-01 | 7 | -142.907 | 299.815 | 0.356 | 0.21 |
| -0.485 | NA | NA | + | -1.67E-03 | -7.88E-02 | 1.95E-01 | 8 | -142.174 | 300.348 | 0.889 | 0.161 |
| -0.4 | NA | NA | + | NA | -6.14E-02 | 1.88E-01 | 7 | -143.275 | 300.551 | 1.092 | 0.145 |
| -0.567 | 1.14E-09 | NA | + | NA | NA | 1.91E-01 | 7 | -143.42 | 300.841 | 1.382 | 0.126 |
| -0.673 | 1.16E-09 | NA | + | -1.44E-03 | NA | 1.97E-01 | 8 | -142.582 | 301.164 | 1.705 | 0.107 |
| -0.504 | NA | NA | + | NA | NA | 1.77E-01 | 6 | -148.278 | 308.555 | 0 | 0.418 |
| -0.54 | 1.24E-09 | NA | + | NA | NA | 1.83E-01 | 7 | -148.014 | 310.027 | 1.472 | 0.2 |
| -0.399 | NA | NA | + | NA | -4.73E-02 | 1.77E-01 | 7 | -148.024 | 310.047 | 1.492 | 0.198 |
| -0.554 | NA | NA | + | -6.81E-04 | NA | 1.80E-01 | 7 | -148.104 | 310.207 | 1.652 | 0.183 |
| -0.499 | NA | NA | + | NA | NA | 1.75E-01 | 6 | -148.069 | 308.138 | 0 | 0.389 |
| -0.36 | NA | NA | + | NA | -6.13E-02 | 1.76E-01 | 7 | -147.61 | 309.22 | 1.082 | 0.227 |
| -0.536 | 1.19E-09 | NA | + | NA | NA | 1.83E-01 | 7 | -147.766 | 309.532 | 1.394 | 0.194 |
| -0.564 | NA | NA | + | -8.37E-04 | NA | 1.78E-01 | 7 | -147.786 | 309.572 | 1.434 | 0.19 |
| -0.579 | NA | NA | + | NA | NA | 1.98E-01 | 6 | -153.1 | 318.2 | 0 | 0.326 |
| -0.672 | NA | NA | + | -1.26E-03 | NA | 2.04E-01 | 7 | -152.49 | 318.98 | 0.779 | 0.221 |
| -0.618 | 1.34E-09 | NA | + | NA | NA | 2.05E-01 | 7 | -152.798 | 319.596 | 1.395 | 0.162 |
| -0.467 | NA | NA | + | NA | -5.14E-02 | 1.99E-01 | 7 | -152.814 | 319.628 | 1.428 | 0.16 |
| -0.54 | NA | NA | + | -1.48E-03 | -6.77E-02 | 2.07E-01 | 8 | -152.006 | 320.013 | 1.812 | 0.132 |
| -0.494 | NA | NA | + | NA | NA | 1.84E-01 | 6 | -157.134 | 326.267 | 0 | 0.44 |
| -0.528 | 1.13E-09 | NA | + | NA | NA | 1.90E-01 | 7 | -156.89 | 327.78 | 1.513 | 0.206 |
| -0.41 | NA | NA | + | NA | -3.83E-02 | 1.85E-01 | 7 | -156.978 | 327.957 | I.69 | 0.189 |
| -0.513 | NA | NA | + | -2.41E-04 | NA | 1.86E-01 | 7 | -157.114 | 328.227 | I.96 | 0.165 |
| -0.184 | NA | NA | NA | 1.48E-03 | NA | 1.45E-01 | 4 | -152.63 | 313.259 | 0 | 0.231 |
| -0.341 | NA | NA | + | NA | NA | 1.36E-01 | 6 | -150.634 | 313.268 | 0.009 | 0.23 |
| -0.287 | NA | NA | NA | NA | NA | 1.73E-01 | 3 | -153.951 | 313.902 | 0.643 | 0.167 |
| -0.369 | 8.83E-10 | NA | + | NA | NA | 1.42E-01 | 7 | -150.46 | 314.919 | I.66 | 0.101 |
| -0.211 | 8.11E-10 | NA | NA | 1.47E-03 | NA | 1.50E-01 | 5 | -152.486 | 314.973 | 1.714 | 0.098 |
| -0.31 | NA | NA | + | 3.54E-04 | NA | 1.34E-01 | 7 | -150.595 | 315.19 | 1.931 | 0.088 |
| -0.177 | NA | NA | NA | 1.46E-03 | -3.70E-03 | 1.45E-01 | 5 | -152.628 | 315.256 | 1.997 | 0.085 |
| -0.412 | NA | NA | + | NA | NA | 1.56E-01 | 6 | -142.248 | 296.496 | 0 | 0.376 |
| -0.317 | NA | NA | + | NA | -4.39E-02 | 1.58E-01 | 7 | -142.028 | 298.056 | I.56 | 0.173 |
| -0.441 | 1.01E-09 | NA | + | NA | NA | 1.61E-01 | 7 | -142.064 | 298.128 | 1.633 | 0.166 |
| -0.332 | NA | NA | NA | NA | NA | 1.90E-01 | 3 | -146.211 | 298.422 | 1.926 | 0.144 |
| -0.432 | NA | NA | + | -2.23E-04 | NA | 1.58E-01 | 7 | -142.23 | 298.46 | 1.964 | 0.141 |
| -0.58 | NA | NA | + | NA | NA | 1.98E-01 | 6 | -161.273 | 334.546 | 0 | 0.407 |
| -0.622 | 1.40E-09 | NA | + | NA | NA | 2.06E-01 | 7 | -160.915 | 335.83 | 1.284 | 0.214 |
| -0.466 | NA | NA | + | NA | -5.01E-02 | 1.98E-01 | 7 | -160.988 | 335.977 | 1.431 | 0.199 |
| -0.629 | NA | NA | + | -7.13E-04 | NA | 2.00E-01 | 7 | -161.085 | 336.17 | 1.624 | 0.181 |
| -0.488 | NA | NA | + | NA | NA | 1.69E-01 | 6 | -147.873 | 307.747 | 0 | 0.399 |
| -0.579 | NA | NA | + | -1.10E-03 | NA | 1.76E-01 | 7 | -147.401 | 308.801 | 1.054 | 0.236 |
| -0.38 | NA | NA | + | NA | -4.76E-02 | 1.70E-01 | 7 | -147.629 | 309.257 | I.51 | 0.188 |
| -0.516 | 1.04E-09 | NA | + | NA | NA | 1.74E-01 | 7 | -147.682 | 309.363 | 1.617 | 0.178 |
| -0.557 | NA | NA | + | NA | NA | 1.96E-01 | 6 | -145.944 | 303.888 | 0 | 0.26 |
| -0.389 | NA | NA | + | NA | -7.59E-02 | 1.98E-01 | 7 | -145.2 | 304.399 | 0.511 | 0.201 |
| -0.448 | NA | NA | + | -1.30E-03 | -8.97E-02 | 2.02E-01 | 8 | -144.472 | 304.944 | 1.056 | 0.153 |
| -0.627 | NA | NA | + | -1.02E-03 | NA | 1.99E-01 | 7 | -145.486 | 304.971 | 1.083 | 0.151 |
| -0.595 | 1.30E-09 | NA | + | NA | NA | 2.03E-01 | 7 | -145.595 | 305.189 | 1.301 | 0.136 |
| -0.431 | 1.17E-09 | NA | + | NA | -7.25E-02 | 2.04E-01 | 8 | -144.916 | 305.832 | 1.944 | 0.098 |
| -0.576 | NA | NA | + | NA | NA | 1.97E-01 | 6 | -161.304 | 334.609 | 0 | 0.417 |
| -0.614 | 1.31E-09 | NA | + | NA | NA | 2.04E-01 | 7 | -161.013 | 336.026 | 1.417 | 0.206 |
| -0.465 | NA | NA | + | NA | -4.95E-02 | 1.98E-01 | 7 | -161.048 | 336.097 | 1.488 | 0.198 |
| -0.62 | NA | NA | + | -6.31E-04 | NA | 1.99E-01 | 7 | -161.154 | 336.308 | 1.699 | 0.179 |
| -0.472 | NA | NA | + | NA | NA | 1.66E-01 | 6 | -153.6 | 319.2 | 0 | 0.446 |
| -0.505 | 1.22E-09 | NA | + | NA | NA | 1.71E-01 | 7 | -153.415 | 320.831 | 1.631 | 0.197 |
| -0.517 | NA | NA | + | -5.53E-04 | NA | 1.70E-01 | 7 | -153.498 | 320.997 | 1.797 | 0.182 |
| -0.413 | NA | NA | + | NA | -2.58E-02 | 1.67E-01 | 7 | -153.534 | 321.068 | 1.869 | 0.175 |
| -0.494 | NA | NA | + | NA | NA | 1.76E-01 | 6 | -146.051 | 304.101 | 0 | 0.394 |
| -0.582 | NA | NA | + | -1.16E-03 | NA | 1.82E-01 | 7 | -145.545 | 305.089 | 0.988 | 0.24 |
| -0.53 | 9.95E-10 | NA | + | NA | NA | 1.85E-01 | 7 | -145.727 | 305.454 | 1.353 | 0.2 |
| -0.419 | NA | NA | + | NA | -3.42E-02 | 1.77E-01 | 7 | -145.915 | 305.829 | 1.728 | 0.166 |
| -0.433 | NA | NA | + | NA | NA | 1.63E-01 | 6 | -145.412 | 302.824 | 0 | 0.434 |
| -0.464 | 1.01E-09 | NA | + | NA | NA | 1.69E-01 | 7 | -145.204 | 304.408 | 1.584 | 0.196 |
| -0.343 | NA | NA | + | NA | -3.97E-02 | 1.64E-01 | 7 | -145.239 | 304.478 | 1.654 | 0.19 |
| -0.481 | NA | NA | + | -5.72E-04 | NA | 1.67E-01 | 7 | -145.29 | 304.581 | 1.757 | 0.18 |
| -0.649 | NA | NA | + | NA | NA | 2.21E-01 | 6 | -165.304 | 342.609 | 0 | 0.221 |
| -0.472 | NA | NA | + | NA | -8.09E-02 | 2.21E-01 | 7 | -164.534 | 343.068 | 0.459 | 0.176 |
| -0.705 | 2.10E-09 | NA | + | NA | NA | 2.29E-01 | 7 | -164.742 | 343.485 | 0.876 | 0.143 |
| -0.537 | NA | NA | + | -1.43E-03 | -9.51E-02 | 2.26E-01 | 8 | -163.769 | 343.539 | 0.93 | 0.139 |
| -0.726 | NA | NA | + | -1.14E-03 | NA | 2.24E-01 | 7 | -164.809 | 343.618 | 1.009 | 0.134 |
| -0.535 | 1.88E-09 | NA | + | NA | -7.48E-02 | 2.28E-01 | 8 | -164.087 | 344.174 | 1.565 | 0.101 |
| -0.781 | 2.09E-09 | NA | + | -1.13E-03 | NA | 2.32E-01 | 8 | -164.252 | 344.503 | 1.894 | 0.086 |
| -0.397 | NA | NA | + | NA | NA | 1.40E-01 | 6 | -146.863 | 305.726 | 0 | 0.45 |
| -0.43 | 1.17E-09 | NA | + | NA | NA | 1.45E-01 | 7 | -146.668 | 307.336 | I.61 | 0.201 |
| -0.442 | NA | NA | + | -5.34E-04 | NA | 1.43E-01 | 7 | -146.768 | 307.536 | I.81 | 0.182 |
| -0.371 | NA | NA | + | NA | -1.15E-02 | 1.41E-01 | 7 | -146.849 | 307.699 | 1.973 | 0.168 |
| -0.486 | NA | NA | + | NA | NA | 1.73E-01 | 6 | -168.429 | 348.858 | 0 | 0.387 |
| -0.522 | 1.08E-09 | NA | + | NA | NA | 1.81E-01 | 7 | -168.189 | 350.377 | I.52 | 0.181 |
| -0.312 | NA | NA | NA | 1.54E-03 | NA | 1.91E-01 | 4 | -171.393 | 350.786 | 1.928 | 0.147 |
| -0.475 | NA | NA | + | NA | -4.74E-03 | 1.74E-01 | 7 | -168.427 | 350.854 | 1.996 | 0.143 |
| -0.481 | NA | NA | + | 6.27E-05 | NA | 1.73E-01 | 7 | -168.428 | 350.855 | 1.998 | 0.142 |
| -0.487 | NA | NA | + | NA | NA | 1.77E-01 | 6 | -147.563 | 307.126 | 0 | 0.395 |
| -0.573 | NA | NA | + | -1.08E-03 | NA | 1.82E-01 | 7 | -147.138 | 308.275 | 1.149 | 0.223 |
| -0.524 | 1.41E-09 | NA | + | NA | NA | 1.82E-01 | 7 | -147.289 | 308.579 | 1.453 | 0.191 |
| -0.383 | NA | NA | + | NA | -4.84E-02 | 1.80E-01 | 7 | -147.292 | 308.584 | 1.458 | 0.191 |
| -0.441 | NA | NA | + | NA | NA | 1.63E-01 | 6 | -140.543 | 293.086 | 0 | 0.425 |
| -0.473 | 7.93E-10 | NA | + | NA | NA | 1.71E-01 | 7 | -140.281 | 294.562 | 1.476 | 0.203 |
| -0.505 | NA | NA | + | -7.92E-04 | NA | 1.67E-01 | 7 | -140.309 | 294.619 | 1.533 | 0.198 |
| -0.374 | NA | NA | + | NA | -3.08E-02 | 1.64E-01 | 7 | -140.436 | 294.871 | 1.786 | 0.174 |
| -0.476 | NA | NA | + | NA | NA | 1.70E-01 | 6 | -151.52 | 315.039 | 0 | 0.425 |
| -0.544 | NA | NA | + | -8.69E-04 | NA | 1.74E-01 | 7 | -151.246 | 316.491 | 1.452 | 0.205 |
| -0.51 | 1.11E-09 | NA | + | NA | NA | 1.75E-01 | 7 | -151.257 | 316.514 | 1.475 | 0.203 |
| -0.422 | NA | NA | + | NA | -2.48E-02 | 1.71E-01 | 7 | -151.455 | 316.911 | 1.872 | 0.167 |
| -0.413 | NA | NA | + | NA | NA | 1.55E-01 | 6 | -149.505 | 311.011 | 0 | 0.455 |
| -0.443 | 8.11E-10 | NA | + | NA | NA | 1.62E-01 | 7 | -149.308 | 312.615 | 1.605 | 0.204 |
| -0.377 | NA | NA | + | NA | -1.72E-02 | 1.56E-01 | 7 | -149.475 | 312.95 | 1.939 | 0.172 |
| -0.428 | NA | NA | + | -1.92E-04 | NA | 1.56E-01 | 7 | -149.493 | 312.986 | 1.975 | 0.169 |
| -0.496 | NA | NA | + | NA | NA | 1.75E-01 | 6 | -157.898 | 327.795 | 0 | 0.414 |
| -0.373 | NA | NA | + | NA | -5.63E-02 | 1.77E-01 | 7 | -157.549 | 329.098 | 1.303 | 0.216 |
| -0.534 | 1.45E-09 | NA | + | NA | NA | 1.81E-01 | 7 | -157.626 | 329.253 | 1.458 | 0.2 |
| -0.541 | NA | NA | + | -5.62E-04 | NA | 1.77E-01 | 7 | -157.787 | 329.574 | 1.779 | 0.17 |
| -0.209 | NA | NA | NA | 1.38E-03 | NA | 1.51E-01 | 4 | -148.561 | 305.122 | 0 | 0.266 |
| -0.299 | NA | NA | NA | NA | NA | 1.76E-01 | 3 | -149.722 | 305.444 | 0.322 | 0.227 |
| -0.351 | NA | NA | + | NA | NA | 1.44E-01 | 6 | -146.894 | 305.788 | 0.666 | 0.191 |
| -0.236 | 6.77E-10 | NA | NA | 1.35E-03 | NA | 1.57E-01 | 5 | -148.425 | 306.851 | 1.729 | 0.112 |
| -0.251 | NA | NA | NA | 1.49E-03 | 2.41E-02 | 1.50E-01 | 5 | -148.507 | 307.014 | 1.892 | 0.103 |
| -0.328 | 8.02E-10 | NA | NA | NA | NA | 1.82E-01 | 4 | -149.534 | 307.067 | 1.945 | 0.101 |
| -0.632 | NA | NA | + | -2.07E-03 | -1.21E-01 | 2.63E-01 | 8 | -157.72 | 331.441 | 0 | 0.51 |
| -0.695 | 1.71E-09 | NA | + | -2.06E-03 | -1.14E-01 | 2.72E-01 | 9 | -157.258 | 332.516 | 1.075 | 0.298 |
| -0.891 | NA | NA | + | -1.70E-03 | NA | 2.70E-01 | 7 | -159.698 | 333.395 | 1.955 | 0.192 |
| -0.431 | NA | NA | + | NA | NA | 1.63E-01 | 6 | -142.114 | 296.228 | 0 | 0.414 |
| -0.515 | NA | NA | + | -1.08E-03 | NA | 1.67E-01 | 7 | -141.683 | 297.367 | 1.139 | 0.234 |
| -0.463 | 1.03E-09 | NA | + | NA | NA | 1.68E-01 | 7 | -141.888 | 297.776 | 1.548 | 0.191 |
| -0.388 | NA | NA | + | NA | -2.08E-02 | 1.64E-01 | 7 | -142.065 | 298.131 | 1.903 | 0.16 |
| -0.52 | NA | NA | + | NA | NA | 1.82E-01 | 6 | -151.181 | 314.363 | 0 | 0.324 |
| -0.376 | NA | NA | + | NA | -6.41E-02 | 1.83E-01 | 7 | -150.711 | 315.422 | 1.059 | 0.191 |
| -0.6 | NA | NA | + | -1.05E-03 | NA | 1.86E-01 | 7 | -150.731 | 315.462 | 1.099 | 0.187 |
| -0.558 | 1.46E-09 | NA | + | NA | NA | 1.88E-01 | 7 | -150.872 | 315.744 | 1.381 | 0.162 |
| -0.443 | NA | NA | + | -1.28E-03 | -7.79E-02 | 1.88E-01 | 8 | -150.054 | 316.108 | 1.745 | 0.135 |
| -0.386 | NA | NA | + | NA | NA | 1.47E-01 | 6 | -142.539 | 297.077 | 0 | 0.444 |
| -0.414 | 9.31E-10 | NA | + | NA | NA | 1.52E-01 | 7 | -142.359 | 298.718 | I.64 | 0.196 |
| -0.443 | NA | NA | + | -6.65E-04 | NA | 1.51E-01 | 7 | -142.395 | 298.789 | 1.712 | 0.189 |
| -0.341 | NA | NA | + | NA | -2.07E-02 | 1.48E-01 | 7 | -142.492 | 298.983 | 1.906 | 0.171 |
| -0.33 | NA | NA | + | NA | NA | 1.37E-01 | 6 | -130.467 | 272.933 | 0 | 0.323 |
| -0.279 | NA | NA | NA | NA | NA | 1.72E-01 | 3 | -134.24 | 274.48 | 1.547 | 0.149 |
| -0.354 | 7.85E-10 | NA | + | NA | NA | 1.41E-01 | 7 | -130.304 | 274.607 | 1.674 | 0.14 |
| -0.262 | NA | NA | + | NA | -3.20E-02 | 1.39E-01 | 7 | -130.343 | 274.686 | 1.753 | 0.134 |
| -0.21 | NA | NA | NA | 1.03E-03 | NA | 1.52E-01 | 4 | -133.38 | 274.76 | 1.827 | 0.129 |
| -0.363 | NA | NA | + | -3.76E-04 | NA | 1.39E-01 | 7 | -130.411 | 274.823 | I.89 | 0.125 |
| -0.527 | NA | NA | + | NA | NA | 1.86E-01 | 6 | -156.937 | 325.874 | 0 | 0.432 |
| -0.564 | 1.37E-09 | NA | + | NA | NA | 1.91E-01 | 7 | -156.692 | 327.383 | 1.509 | 0.203 |
| -0.59 | NA | NA | + | -8.07E-04 | NA | 1.90E-01 | 7 | -156.708 | 327.415 | 1.541 | 0.2 |
| -0.484 | NA | NA | + | NA | -1.93E-02 | 1.87E-01 | 7 | -156.9 | 327.8 | 1.926 | 0.165 |
| -0.588 | NA | NA | + | NA | NA | 2.07E-01 | 6 | -153.712 | 319.425 | 0 | 0.385 |
| -0.67 | NA | NA | + | -1.13E-03 | NA | 2.12E-01 | 7 | -153.212 | 320.423 | 0.998 | 0.234 |
| -0.628 | 1.51E-09 | NA | + | NA | NA | 2.13E-01 | 7 | -153.392 | 320.784 | 1.359 | 0.195 |
| -0.476 | NA | NA | + | NA | -4.92E-02 | 2.06E-01 | 7 | -153.439 | 320.878 | 1.453 | 0.186 |
| -0.112 | NA | NA | NA | 1.33E-03 | NA | 1.18E-01 | 4 | -141.718 | 291.437 | 0 | 0.332 |
| -0.213 | NA | NA | NA | NA | NA | 1.46E-01 | 3 | -142.919 | 291.837 | 0.401 | 0.271 |
| -0.173 | NA | NA | NA | 1.48E-03 | 3.54E-02 | 1.15E-01 | 5 | -141.599 | 293.197 | I.76 | 0.138 |
| -0.132 | 5.89E-10 | NA | NA | 1.32E-03 | NA | 1.22E-01 | 5 | -141.626 | 293.251 | 1.814 | 0.134 |
| -0.253 | NA | NA | + | NA | NA | 1.18E-01 | 6 | -140.688 | 293.376 | 1.939 | 0.126 |
| -0.6 | NA | NA | + | NA | NA | 2.06E-01 | 6 | -167.472 | 346.943 | 0 | 0.431 |
| -0.641 | 1.35E-09 | NA | + | NA | NA | 2.14E-01 | 7 | -167.198 | 348.396 | 1.453 | 0.208 |
| -0.509 | NA | NA | + | NA | -3.98E-02 | 2.05E-01 | 7 | -167.314 | 348.628 | 1.684 | 0.185 |
| -0.642 | NA | NA | + | -5.62E-04 | NA | 2.09E-01 | 7 | -167.367 | 348.735 | 1.791 | 0.176 |
| -0.487 | NA | NA | + | NA | NA | 1.74E-01 | 6 | -147.875 | 307.75 | 0 | 0.433 |
| -0.519 | 1.16E-09 | NA | + | NA | NA | 1.79E-01 | 7 | -147.639 | 309.279 | 1.529 | 0.202 |
| -0.403 | NA | NA | + | NA | -3.77E-02 | 1.76E-01 | 7 | -147.715 | 309.43 | I.68 | 0.187 |
| -0.531 | NA | NA | + | -5.63E-04 | NA | 1.77E-01 | 7 | -147.761 | 309.521 | 1.771 | 0.179 |
| -0.438 | NA | NA | + | NA | NA | 1.68E-01 | 6 | -151.757 | 315.513 | 0 | 0.452 |
| -0.468 | 1.22E-09 | NA | + | NA | NA | 1.73E-01 | 7 | -151.553 | 317.106 | 1.593 | 0.204 |
| -0.387 | NA | NA | + | NA | -2.38E-02 | 1.69E-01 | 7 | -151.698 | 317.397 | 1.884 | 0.176 |
| -0.453 | NA | NA | + | -1.94E-04 | NA | 1.69E-01 | 7 | -151.743 | 317.487 | 1.974 | 0.168 |
| -0.503 | NA | NA | + | NA | NA | 1.81E-01 | 6 | -147.276 | 306.551 | 0 | 0.309 |
| -0.349 | NA | NA | + | NA | -7.01E-02 | 1.82E-01 | 7 | -146.667 | 307.333 | 0.782 | 0.209 |
| -0.588 | NA | NA | + | -1.05E-03 | NA | 1.87E-01 | 7 | -146.845 | 307.69 | 1.139 | 0.175 |
| -0.542 | 1.37E-09 | NA | + | NA | NA | 1.88E-01 | 7 | -146.957 | 307.914 | 1.363 | 0.157 |
| -0.426 | NA | NA | + | -1.32E-03 | -8.36E-02 | 1.91E-01 | 8 | -146.002 | 308.003 | 1.452 | 0.15 |
| -0.482 | NA | NA | + | NA | NA | 1.68E-01 | 6 | -162.534 | 337.068 | 0 | 0.458 |
| -0.51 | 9.99E-10 | NA | + | NA | NA | 1.73E-01 | 7 | -162.392 | 338.784 | 1.717 | 0.194 |
| -0.514 | NA | NA | + | -4.35E-04 | NA | 1.70E-01 | 7 | -162.476 | 338.951 | 1.883 | 0.179 |
| -0.481 | NA | NA | + | NA | -2.17E-04 | 1.68E-01 | 7 | -162.534 | 339.068 | 2 | 0.169 |
| -0.64 | NA | NA | + | NA | NA | 2.11E-01 | 6 | -157.134 | 326.268 | 0 | 0.252 |
| -0.726 | NA | NA | + | -1.32E-03 | NA | 2.15E-01 | 7 | -156.429 | 326.859 | 0.591 | 0.188 |
| -0.491 | NA | NA | + | NA | -6.69E-02 | 2.13E-01 | 7 | -156.603 | 327.207 | 0.938 | 0.158 |
| -0.556 | NA | NA | + | -1.59E-03 | -8.42E-02 | 2.19E-01 | 8 | -155.609 | 327.219 | 0.95 | 0.157 |
| -0.683 | 1.39E-09 | NA | + | NA | NA | 2.19E-01 | 7 | -156.718 | 327.437 | 1.168 | 0.141 |
| -0.77 | 1.40E-09 | NA | + | -1.32E-03 | NA | 2.23E-01 | 8 | -156.005 | 328.009 | 1.741 | 0.106 |
| -0.47 | NA | NA | + | NA | NA | 1.67E-01 | 6 | -144.379 | 300.758 | 0 | 0.413 |
| -0.353 | NA | NA | + | NA | -5.29E-02 | 1.69E-01 | 7 | -144.057 | 302.114 | 1.356 | 0.21 |
| -0.505 | 1.21E-09 | NA | + | NA | NA | 1.73E-01 | 7 | -144.146 | 302.291 | 1.533 | 0.192 |
| -0.524 | NA | NA | + | -6.93E-04 | NA | 1.71E-01 | 7 | -144.186 | 302.372 | 1.614 | 0.184 |
| -0.302 | NA | NA | + | NA | NA | 1.26E-01 | 6 | -138.269 | 288.538 | 0 | 0.264 |
| -0.248 | NA | NA | NA | NA | NA | 1.59E-01 | 3 | -141.424 | 288.847 | 0.309 | 0.226 |
| -0.167 | NA | NA | NA | 1.13E-03 | NA | 1.36E-01 | 4 | -140.526 | 289.052 | 0.514 | 0.204 |
| -0.326 | 9.23E-10 | NA | + | NA | NA | 1.30E-01 | 7 | -138.138 | 290.277 | 1.739 | 0.111 |
| -0.318 | NA | NA | + | -1.74E-04 | NA | 1.27E-01 | 7 | -138.259 | 290.518 | I.98 | 0.098 |
| -0.301 | NA | NA | + | NA | -6.18E-04 | 1.26E-01 | 7 | -138.269 | 290.538 | 2 | 0.097 |
| -0.452 | NA | NA | + | NA | NA | 1.59E-01 | 6 | -154.743 | 321.487 | 0 | 0.447 |
| -0.487 | 7.66E-10 | NA | + | NA | NA | 1.68E-01 | 7 | -154.521 | 323.042 | 1.555 | 0.206 |
| -0.491 | NA | NA | + | -4.72E-04 | NA | 1.61E-01 | 7 | -154.671 | 323.341 | 1.854 | 0.177 |
| -0.412 | NA | NA | + | NA | -1.83E-02 | 1.59E-01 | 7 | -154.71 | 323.42 | 1.933 | 0.17 |
| -0.375 | NA | NA | + | NA | NA | 1.50E-01 | 6 | -145.46 | 302.919 | 0 | 0.296 |
| -0.301 | NA | NA | NA | NA | NA | 1.78E-01 | 3 | -148.892 | 303.784 | 0.864 | 0.192 |
| -0.233 | NA | NA | NA | 1.11E-03 | NA | 1.59E-01 | 4 | -148.046 | 304.093 | 1.174 | 0.165 |
| -0.401 | 1.01E-09 | NA | + | NA | NA | 1.54E-01 | 7 | -145.298 | 304.596 | 1.677 | 0.128 |
| -0.392 | NA | NA | + | -1.97E-04 | NA | 1.51E-01 | 7 | -145.447 | 304.894 | 1.974 | 0.11 |
| -0.381 | NA | NA | + | NA | 2.82E-03 | 1.50E-01 | 7 | -145.459 | 304.918 | 1.998 | 0.109 |
| -0.534 | NA | NA | + | NA | NA | 1.88E-01 | 6 | -149.6 | 311.201 | 0 | 0.386 |
| -0.407 | NA | NA | + | NA | -5.80E-02 | 1.90E-01 | 7 | -149.203 | 312.406 | 1.206 | 0.211 |
| -0.602 | NA | NA | + | -9.67E-04 | NA | 1.91E-01 | 7 | -149.214 | 312.429 | 1.228 | 0.209 |
| -0.573 | 1.46E-09 | NA | + | NA | NA | 1.93E-01 | 7 | -149.286 | 312.573 | 1.372 | 0.194 |
| -0.608 | NA | NA | + | NA | NA | 2.02E-01 | 6 | -167.494 | 346.987 | 0 | 0.394 |
| -0.472 | NA | NA | + | NA | -6.17E-02 | 2.04E-01 | 7 | -167.084 | 348.167 | I.18 | 0.218 |
| -0.654 | 1.65E-09 | NA | + | NA | NA | 2.09E-01 | 7 | -167.123 | 348.245 | 1.258 | 0.21 |
| -0.659 | NA | NA | + | -7.45E-04 | NA | 2.04E-01 | 7 | -167.29 | 348.581 | 1.593 | 0.178 |
| -0.64 | NA | NA | + | -2.21E-03 | -1.32E-01 | 2.66E-01 | 8 | -158.385 | 332.771 | 0 | 0.641 |
| -0.701 | 1.68E-09 | NA | + | -2.18E-03 | -1.26E-01 | 2.75E-01 | 9 | -157.965 | 333.931 | I.16 | 0.359 |
| -0.625 | NA | NA | + | NA | NA | 2.08E-01 | 6 | -165.335 | 342.671 | 0 | 0.329 |
| -0.479 | NA | NA | + | NA | -6.46E-02 | 2.09E-01 | 7 | -164.876 | 343.751 | 01.VIII | 0.192 |
| -0.696 | NA | NA | + | -1.05E-03 | NA | 2.11E-01 | 7 | -164.939 | 343.878 | 1.207 | 0.18 |
| -0.669 | 1.58E-09 | NA | + | NA | NA | 2.15E-01 | 7 | -164.967 | 343.933 | 1.262 | 0.175 |
| -0.539 | NA | NA | + | -1.26E-03 | -7.60E-02 | 2.12E-01 | 8 | -164.315 | 344.63 | 1.959 | 0.124 |
| -0.336 | NA | NA | + | NA | NA | 1.28E-01 | 6 | -142.569 | 297.138 | 0 | 0.323 |
| -0.179 | NA | NA | NA | 1.27E-03 | NA | 1.41E-01 | 4 | -145.339 | 298.678 | I.54 | 0.15 |
| -0.265 | NA | NA | NA | NA | NA | 1.67E-01 | 3 | -146.346 | 298.693 | 1.555 | 0.149 |
| -0.358 | 7.06E-10 | NA | + | NA | NA | 1.32E-01 | 7 | -142.457 | 298.914 | 1.777 | 0.133 |
| -0.286 | NA | NA | + | NA | -2.34E-02 | 1.30E-01 | 7 | -142.511 | 299.022 | 1.884 | 0.126 |
| -0.349 | NA | NA | + | -1.41E-04 | NA | 1.29E-01 | 7 | -142.562 | 299.125 | 1.987 | 0.12 |
| -0.532 | NA | NA | + | NA | NA | 1.82E-01 | 6 | -165.414 | 342.827 | 0 | 0.437 |
| -0.574 | 1.41E-09 | NA | + | NA | NA | 1.89E-01 | 7 | -165.134 | 344.269 | 1.442 | 0.213 |
| -0.585 | NA | NA | + | -7.03E-04 | NA | 1.85E-01 | 7 | -165.256 | 344.513 | 1.685 | 0.188 |
| -0.518 | NA | NA | + | NA | -6.48E-03 | 1.82E-01 | 7 | -165.41 | 344.82 | 1.992 | 0.162 |
| -0.509 | NA | NA | + | NA | NA | 1.79E-01 | 6 | -165.889 | 343.779 | 0 | 0.441 |
| -0.556 | 1.65E-09 | NA | + | NA | NA | 1.86E-01 | 7 | -165.574 | 345.148 | I.37 | 0.222 |
| -0.448 | NA | NA | + | NA | -2.68E-02 | 1.79E-01 | 7 | -165.82 | 345.639 | 1.861 | 0.174 |
| -0.516 | NA | NA | + | -9.32E-05 | NA | 1.80E-01 | 7 | -165.887 | 345.773 | 1.995 | 0.163 |
| -0.536 | NA | NA | + | NA | NA | 1.80E-01 | 6 | -156.634 | 325.267 | 0 | 0.445 |
| -0.594 | NA | NA | + | -7.79E-04 | NA | 1.84E-01 | 7 | -156.436 | 326.872 | 1.604 | 0.2 |
| -0.564 | 9.34E-10 | NA | + | NA | NA | 1.86E-01 | 7 | -156.477 | 326.953 | 1.686 | 0.192 |
| -0.541 | NA | NA | + | NA | 2.18E-03 | 1.80E-01 | 7 | -156.633 | 327.267 | 1.999 | 0.164 |
| -0.444 | NA | NA | + | NA | NA | 1.64E-01 | 6 | -150.36 | 312.721 | 0 | 0.381 |
| -0.476 | 1.11E-09 | NA | + | NA | NA | 1.70E-01 | 7 | -150.132 | 314.263 | 1.542 | 0.176 |
| -0.374 | NA | NA | + | NA | -3.19E-02 | 1.65E-01 | 7 | -150.252 | 314.503 | 1.782 | 0.156 |
| -0.368 | NA | NA | NA | NA | NA | 2.04E-01 | 3 | -154.329 | 314.659 | 1.938 | 0.145 |
| -0.454 | NA | NA | + | -1.19E-04 | NA | 1.65E-01 | 7 | -150.355 | 314.711 | I.99 | 0.141 |
| -0.605 | NA | NA | + | NA | NA | 2.09E-01 | 6 | -153.533 | 319.066 | 0 | 0.248 |
| -0.702 | NA | NA | + | -1.32E-03 | NA | 2.15E-01 | 7 | -152.851 | 319.703 | 0.636 | 0.181 |
| -0.446 | NA | NA | + | NA | -7.19E-02 | 2.11E-01 | 7 | -152.916 | 319.832 | 0.766 | 0.169 |
| -0.527 | NA | NA | + | -1.59E-03 | -8.76E-02 | 2.18E-01 | 8 | -151.953 | 319.905 | 0.839 | 0.163 |
| -0.651 | 1.62E-09 | NA | + | NA | NA | 2.17E-01 | 7 | -153.13 | 320.26 | 1.193 | 0.137 |
| -0.75 | 1.66E-09 | NA | + | -1.34E-03 | NA | 2.23E-01 | 8 | -152.427 | 320.854 | 1.787 | 0.102 |
| -0.531 | NA | NA | + | -2.27E-03 | -1.15E-01 | 2.17E-01 | 8 | -143.225 | 302.451 | 0 | 0.492 |
| -0.58 | 1.31E-09 | NA | + | -2.26E-03 | -1.11E-01 | 2.25E-01 | 9 | -142.843 | 303.685 | 1.234 | 0.266 |
| -0.757 | NA | NA | + | -1.91E-03 | NA | 2.15E-01 | 7 | -144.937 | 303.873 | 1.423 | 0.242 |
| -0.446 | NA | NA | + | NA | NA | 1.64E-01 | 6 | -150.813 | 313.626 | 0 | 0.433 |
| -0.479 | 9.53E-10 | NA | + | NA | NA | 1.71E-01 | 7 | -150.592 | 315.183 | 1.558 | 0.199 |
| -0.347 | NA | NA | + | NA | -4.44E-02 | 1.65E-01 | 7 | -150.601 | 315.202 | 1.576 | 0.197 |
| -0.484 | NA | NA | + | -4.66E-04 | NA | 1.66E-01 | 7 | -150.735 | 315.469 | 1.844 | 0.172 |
| -0.419 | NA | NA | + | NA | NA | 1.47E-01 | 6 | -138.71 | 289.42 | 0 | 0.329 |
| -0.518 | NA | NA | + | -1.21E-03 | NA | 1.52E-01 | 7 | -138.137 | 290.274 | 0.855 | 0.215 |
| -0.304 | NA | NA | + | NA | -5.22E-02 | 1.49E-01 | 7 | -138.39 | 290.781 | 1.361 | 0.167 |
| -0.452 | 9.17E-10 | NA | + | NA | NA | 1.54E-01 | 7 | -138.45 | 290.9 | 1.481 | 0.157 |
| -0.387 | NA | NA | + | -1.42E-03 | -6.72E-02 | 1.56E-01 | 8 | -137.621 | 291.241 | 1.822 | 0.132 |
| -0.442 | NA | NA | + | NA | NA | 1.71E-01 | 6 | -143.127 | 298.253 | 0 | 0.442 |
| -0.479 | 1.27E-09 | NA | + | NA | NA | 1.76E-01 | 7 | -142.853 | 299.707 | 1.453 | 0.214 |
| -0.477 | NA | NA | + | -4.04E-04 | NA | 1.73E-01 | 7 | -143.066 | 300.132 | 1.879 | 0.173 |
| -0.393 | NA | NA | + | NA | -2.23E-02 | 1.71E-01 | 7 | -143.07 | 300.141 | 1.888 | 0.172 |
| -0.462 | NA | NA | + | NA | NA | 1.65E-01 | 6 | -153.229 | 318.458 | 0 | 0.447 |
| -0.495 | 1.03E-09 | NA | + | NA | NA | 1.71E-01 | 7 | -153.002 | 320.004 | 1.546 | 0.206 |
| -0.504 | NA | NA | + | -5.23E-04 | NA | 1.67E-01 | 7 | -153.138 | 320.276 | 1.818 | 0.18 |
| -0.436 | NA | NA | + | NA | -1.20E-02 | 1.65E-01 | 7 | -153.214 | 320.429 | 1.971 | 0.167 |
| -0.537 | NA | NA | + | NA | NA | 1.75E-01 | 6 | -164.469 | 340.937 | 0 | 0.444 |
| -0.572 | 9.55E-10 | NA | + | NA | NA | 1.83E-01 | 7 | -164.234 | 342.469 | 1.532 | 0.206 |
| -0.575 | NA | NA | + | -5.05E-04 | NA | 1.77E-01 | 7 | -164.388 | 342.776 | 1.838 | 0.177 |
| -0.482 | NA | NA | + | NA | -2.45E-02 | 1.76E-01 | 7 | -164.413 | 342.825 | 1.888 | 0.173 |
| -0.435 | NA | NA | + | NA | NA | 1.60E-01 | 6 | -150.193 | 312.386 | 0 | 0.451 |
| -0.465 | 1.09E-09 | NA | + | NA | NA | 1.65E-01 | 7 | -150.014 | 314.028 | 1.642 | 0.199 |
| -0.479 | NA | NA | + | -5.44E-04 | NA | 1.62E-01 | 7 | -150.096 | 314.192 | 1.806 | 0.183 |
| -0.421 | NA | NA | + | NA | -6.67E-03 | 1.60E-01 | 7 | -150.189 | 314.377 | 1.991 | 0.167 |
| -0.476 | NA | NA | + | NA | NA | 1.71E-01 | 6 | -144.553 | 301.105 | 0 | 0.398 |
| -0.358 | NA | NA | + | NA | -5.28E-02 | 1.71E-01 | 7 | -144.213 | 302.425 | I.32 | 0.206 |
| -0.516 | 1.38E-09 | NA | + | NA | NA | 1.77E-01 | 7 | -144.244 | 302.489 | 1.383 | 0.199 |
| -0.546 | NA | NA | + | -8.94E-04 | NA | 1.76E-01 | 7 | -144.256 | 302.513 | 1.407 | 0.197 |
| -0.458 | NA | NA | + | NA | NA | 1.71E-01 | 6 | -145.972 | 303.945 | 0 | 0.414 |
| -0.537 | NA | NA | + | -9.95E-04 | NA | 1.76E-01 | 7 | -145.577 | 305.153 | 1.209 | 0.226 |
| -0.491 | 1.23E-09 | NA | + | NA | NA | 1.76E-01 | 7 | -145.759 | 305.517 | 1.573 | 0.189 |
| -0.389 | NA | NA | + | NA | -3.21E-02 | 1.72E-01 | 7 | -145.858 | 305.716 | 1.772 | 0.171 |
| -0.507 | NA | NA | + | NA | NA | 1.77E-01 | 6 | -157.671 | 327.342 | 0 | 0.413 |
| -0.379 | NA | NA | + | NA | -5.71E-02 | 1.79E-01 | 7 | -157.321 | 328.641 | 1.299 | 0.216 |
| -0.547 | 1.44E-09 | NA | + | NA | NA | 1.84E-01 | 7 | -157.389 | 328.777 | 1.436 | 0.202 |
| -0.551 | NA | NA | + | -5.51E-04 | NA | 1.81E-01 | 7 | -157.566 | 329.133 | 1.791 | 0.169 |
| -0.537 | NA | NA | + | NA | NA | 1.84E-01 | 6 | -160.895 | 333.791 | 0 | 0.392 |
| -0.386 | NA | NA | + | NA | -6.73E-02 | 1.85E-01 | 7 | -160.397 | 334.795 | 1.004 | 0.237 |
| -0.578 | 1.26E-09 | NA | + | NA | NA | 1.92E-01 | 7 | -160.555 | 335.109 | 1.318 | 0.203 |
| -0.586 | NA | NA | + | -6.66E-04 | NA | 1.86E-01 | 7 | -160.736 | 335.471 | 1.681 | 0.169 |
| -0.564 | NA | NA | + | NA | NA | 1.90E-01 | 6 | -156.307 | 324.614 | 0 | 0.294 |
| -0.652 | NA | NA | + | -1.24E-03 | NA | 1.95E-01 | 7 | -155.725 | 325.449 | 0.835 | 0.193 |
| -0.404 | NA | NA | + | NA | -7.07E-02 | 1.90E-01 | 7 | -155.725 | 325.449 | 0.835 | 0.193 |
| -0.477 | NA | NA | + | -1.49E-03 | -8.49E-02 | 1.95E-01 | 8 | -154.899 | 325.798 | 1.184 | 0.162 |
| -0.608 | 1.38E-09 | NA | + | NA | NA | 1.99E-01 | 7 | -155.935 | 325.87 | 1.255 | 0.157 |
| -0.515 | NA | NA | + | NA | NA | 1.82E-01 | 6 | -147.559 | 307.119 | 0 | 0.294 |
| -0.346 | NA | NA | + | NA | -7.46E-02 | 1.82E-01 | 7 | -146.891 | 307.782 | 0.663 | 0.211 |
| -0.602 | NA | NA | + | -1.12E-03 | NA | 1.89E-01 | 7 | -147.048 | 308.097 | 0.978 | 0.18 |
| -0.421 | NA | NA | + | -1.38E-03 | -8.89E-02 | 1.90E-01 | 8 | -146.121 | 308.242 | 1.123 | 0.168 |
| -0.553 | 1.42E-09 | NA | + | NA | NA | 1.88E-01 | 7 | -147.25 | 308.5 | 1.382 | 0.147 |
| -0.56 | NA | NA | + | NA | NA | 1.86E-01 | 6 | -158.156 | 328.311 | 0 | 0.403 |
| -0.635 | NA | NA | + | -1.03E-03 | NA | 1.90E-01 | 7 | -157.782 | 329.564 | 1.253 | 0.216 |
| -0.597 | 1.21E-09 | NA | + | NA | NA | 1.92E-01 | 7 | -157.871 | 329.742 | I.43 | 0.197 |
| -0.46 | NA | NA | + | NA | -4.47E-02 | 1.87E-01 | 7 | -157.943 | 329.885 | 1.574 | 0.184 |
| -0.537 | NA | NA | + | NA | NA | 1.90E-01 | 6 | -145.711 | 303.421 | 0 | 0.302 |
| -0.631 | NA | NA | + | -1.26E-03 | NA | 1.96E-01 | 7 | -145.037 | 304.074 | 0.653 | 0.218 |
| -0.408 | NA | NA | + | NA | -6.00E-02 | 1.92E-01 | 7 | -145.288 | 304.576 | 1.155 | 0.169 |
| -0.481 | NA | NA | + | -1.52E-03 | -7.81E-02 | 1.99E-01 | 8 | -144.342 | 304.684 | 1.263 | 0.16 |
| -0.576 | 1.30E-09 | NA | + | NA | NA | 1.97E-01 | 7 | -145.406 | 304.812 | I.39 | 0.151 |
| -0.497 | NA | NA | + | NA | NA | 1.71E-01 | 6 | -153.181 | 318.363 | 0 | 0.384 |
| -0.364 | NA | NA | + | NA | -6.07E-02 | 1.73E-01 | 7 | -152.742 | 319.484 | 1.122 | 0.219 |
| -0.577 | NA | NA | + | -1.02E-03 | NA | 1.76E-01 | 7 | -152.793 | 319.586 | 1.223 | 0.208 |
| -0.534 | 1.14E-09 | NA | + | NA | NA | 1.79E-01 | 7 | -152.887 | 319.775 | 1.412 | 0.189 |
| -0.547 | NA | NA | + | -1.98E-03 | -1.17E-01 | 2.32E-01 | 8 | -154.692 | 325.384 | 0 | 0.341 |
| -0.6 | 1.68E-09 | NA | + | -1.97E-03 | -1.12E-01 | 2.38E-01 | 9 | -154.308 | 326.617 | 1.233 | 0.184 |
| -0.782 | NA | NA | + | -1.63E-03 | NA | 2.30E-01 | 7 | -156.369 | 326.738 | 1.353 | 0.173 |
| -0.457 | NA | NA | + | NA | -9.56E-02 | 2.25E-01 | 7 | -156.445 | 326.891 | 1.506 | 0.161 |
| -0.669 | NA | NA | + | NA | NA | 2.25E-01 | 6 | -157.578 | 327.156 | 1.772 | 0.141 |
| -0.605 | NA | NA | + | NA | NA | 2.01E-01 | 6 | -158.672 | 329.345 | 0 | 0.314 |
| -0.456 | NA | NA | + | NA | -6.65E-02 | 2.01E-01 | 7 | -158.17 | 330.34 | 0.995 | 0.191 |
| -0.681 | NA | NA | + | -1.09E-03 | NA | 2.04E-01 | 7 | -158.197 | 330.394 | 01.V | 0.186 |
| -0.65 | 1.71E-09 | NA | + | NA | NA | 2.08E-01 | 7 | -158.301 | 330.602 | 1.257 | 0.168 |
| -0.516 | NA | NA | + | -1.33E-03 | -8.08E-02 | 2.05E-01 | 8 | -157.475 | 330.949 | 1.605 | 0.141 |
| -0.588 | NA | NA | + | NA | NA | 1.98E-01 | 6 | -156.452 | 324.905 | 0 | 0.383 |
| -0.445 | NA | NA | + | NA | -6.30E-02 | 1.98E-01 | 7 | -156.008 | 326.017 | 1.112 | 0.22 |
| -0.656 | NA | NA | + | -9.72E-04 | NA | 2.01E-01 | 7 | -156.085 | 326.17 | 1.265 | 0.204 |
| -0.627 | 1.37E-09 | NA | + | NA | NA | 2.05E-01 | 7 | -156.136 | 326.271 | 1.366 | 0.194 |
| -0.457 | NA | NA | + | NA | NA | 1.70E-01 | 6 | -152.549 | 317.099 | 0 | 0.441 |
| -0.49 | 1.03E-09 | NA | + | NA | NA | 1.77E-01 | 7 | -152.312 | 318.623 | 1.524 | 0.206 |
| -0.507 | NA | NA | + | -6.19E-04 | NA | 1.74E-01 | 7 | -152.421 | 318.841 | 1.742 | 0.184 |
| -0.412 | NA | NA | + | NA | -2.04E-02 | 1.71E-01 | 7 | -152.508 | 319.016 | 1.917 | 0.169 |
| -0.523 | NA | NA | + | NA | NA | 1.84E-01 | 6 | -151.737 | 315.475 | 0 | 0.396 |
| -0.618 | NA | NA | + | -1.17E-03 | NA | 1.91E-01 | 7 | -151.252 | 316.504 | 1.029 | 0.237 |
| -0.559 | 1.29E-09 | NA | + | NA | NA | 1.90E-01 | 7 | -151.496 | 316.991 | 1.517 | 0.186 |
| -0.422 | NA | NA | + | NA | -4.48E-02 | 1.85E-01 | 7 | -151.521 | 317.043 | 1.568 | 0.181 |
| -0.55 | NA | NA | + | NA | NA | 1.92E-01 | 6 | -155.248 | 322.496 | 0 | 0.396 |
| -0.422 | NA | NA | + | NA | -5.72E-02 | 1.92E-01 | 7 | -154.863 | 323.726 | I.23 | 0.214 |
| -0.591 | 1.23E-09 | NA | + | NA | NA | 2.00E-01 | 7 | -154.921 | 323.842 | 1.346 | 0.202 |
| -0.612 | NA | NA | + | -8.36E-04 | NA | 1.96E-01 | 7 | -154.99 | 323.98 | 1.484 | 0.188 |
| -0.569 | NA | NA | + | NA | NA | 1.93E-01 | 6 | -159.722 | 331.444 | 0 | 0.413 |
| -0.439 | NA | NA | + | NA | -5.59E-02 | 1.92E-01 | 7 | -159.376 | 332.753 | 1.309 | 0.215 |
| -0.608 | 1.42E-09 | NA | + | NA | NA | 2.00E-01 | 7 | -159.453 | 332.905 | 1.461 | 0.199 |
| -0.61 | NA | NA | + | -5.83E-04 | NA | 1.95E-01 | 7 | -159.59 | 333.179 | 1.735 | 0.173 |
| -0.545 | NA | NA | + | NA | NA | 1.88E-01 | 6 | -152.167 | 316.335 | 0 | 0.323 |
| -0.384 | NA | NA | + | NA | -7.10E-02 | 1.88E-01 | 7 | -151.577 | 317.154 | 0.819 | 0.214 |
| -0.62 | NA | NA | + | -9.52E-04 | NA | 1.93E-01 | 7 | -151.823 | 317.647 | 1.312 | 0.168 |
| -0.582 | 1.29E-09 | NA | + | NA | NA | 1.95E-01 | 7 | -151.864 | 317.729 | 1.394 | 0.161 |
| -0.453 | NA | NA | + | -1.20E-03 | -8.27E-02 | 1.95E-01 | 8 | -151.043 | 318.085 | I.75 | 0.135 |
| -0.576 | NA | NA | + | NA | NA | 2.00E-01 | 6 | -151.286 | 314.573 | 0 | 0.255 |
| -0.667 | NA | NA | + | -1.35E-03 | NA | 2.04E-01 | 7 | -150.529 | 315.057 | 0.484 | 0.2 |
| -0.515 | NA | NA | + | -1.59E-03 | -7.87E-02 | 2.07E-01 | 8 | -149.804 | 315.607 | 1.034 | 0.152 |
| -0.443 | NA | NA | + | NA | -6.17E-02 | 2.02E-01 | 7 | -150.831 | 315.662 | 1.089 | 0.148 |
| -0.616 | 1.15E-09 | NA | + | NA | NA | 2.09E-01 | 7 | -150.927 | 315.854 | 1.281 | 0.134 |
| -0.712 | 1.21E-09 | NA | + | -1.39E-03 | NA | 2.14E-01 | 8 | -150.123 | 316.246 | 1.673 | 0.11 |
| -0.64 | NA | NA | + | NA | NA | 2.11E-01 | 6 | -169.309 | 350.618 | 0 | 0.4 |
| -0.687 | 1.78E-09 | NA | + | NA | NA | 2.18E-01 | 7 | -168.963 | 351.926 | 1.307 | 0.208 |
| -0.711 | NA | NA | + | -1.00E-03 | NA | 2.15E-01 | 7 | -168.975 | 351.951 | 1.333 | 0.205 |
| -0.533 | NA | NA | + | NA | -4.80E-02 | 2.11E-01 | 7 | -169.073 | 352.146 | 1.527 | 0.186 |
| -0.437 | NA | NA | + | NA | NA | 1.64E-01 | 6 | -139.95 | 291.901 | 0 | 0.411 |
| -0.517 | NA | NA | + | -9.79E-04 | NA | 1.69E-01 | 7 | -139.576 | 293.153 | 1.252 | 0.22 |
| -0.349 | NA | NA | + | NA | -4.10E-02 | 1.66E-01 | 7 | -139.747 | 293.493 | 1.592 | 0.185 |
| -0.467 | 1.10E-09 | NA | + | NA | NA | 1.68E-01 | 7 | -139.752 | 293.504 | 1.603 | 0.184 |
| -0.58 | NA | NA | + | NA | NA | 1.95E-01 | 6 | -151.67 | 315.339 | 0 | 0.322 |
| -0.671 | NA | NA | + | -1.25E-03 | NA | 1.99E-01 | 7 | -151.064 | 316.128 | 0.789 | 0.217 |
| -0.619 | 1.38E-09 | NA | + | NA | NA | 2.02E-01 | 7 | -151.323 | 316.645 | 1.306 | 0.168 |
| -0.462 | NA | NA | + | NA | -5.26E-02 | 1.97E-01 | 7 | -151.356 | 316.712 | 1.372 | 0.162 |
| -0.535 | NA | NA | + | -1.44E-03 | -6.69E-02 | 2.02E-01 | 8 | -150.566 | 317.132 | 1.793 | 0.131 |
| -0.281 | NA | NA | NA | NA | NA | 1.72E-01 | 3 | -147.069 | 300.139 | 0 | 0.218 |
| -0.35 | NA | NA | + | NA | NA | 1.46E-01 | 6 | -144.083 | 300.167 | 0.028 | 0.215 |
| -0.205 | NA | NA | NA | 1.21E-03 | NA | 1.51E-01 | 4 | -146.157 | 300.314 | 0.175 | 0.2 |
| -0.306 | 9.03E-10 | NA | NA | NA | NA | 1.76E-01 | 4 | -146.909 | 301.818 | I.68 | 0.094 |
| -0.208 | NA | NA | NA | NA | -3.71E-02 | 1.72E-01 | 4 | -146.918 | 301.835 | 1.697 | 0.093 |
| -0.375 | 8.96E-10 | NA | + | NA | NA | 1.50E-01 | 7 | -143.92 | 301.841 | 1.702 | 0.093 |
| -0.231 | 8.90E-10 | NA | NA | 1.21E-03 | NA | 1.56E-01 | 5 | -145.999 | 301.999 | I.86 | 0.086 |
| -0.416 | NA | NA | + | NA | NA | 1.54E-01 | 6 | -147.332 | 306.664 | 0 | 0.454 |
| -0.47 | NA | NA | + | -6.61E-04 | NA | 1.57E-01 | 7 | -147.195 | 308.389 | 1.725 | 0.191 |
| -0.439 | 7.96E-10 | NA | + | NA | NA | 1.58E-01 | 7 | -147.212 | 308.424 | I.76 | 0.188 |
| -0.422 | NA | NA | + | NA | 2.94E-03 | 1.54E-01 | 7 | -147.331 | 308.662 | 1.998 | 0.167 |
| -0.585 | NA | NA | + | NA | NA | 2.01E-01 | 6 | -158.8 | 329.599 | 0 | 0.397 |
| -0.659 | NA | NA | + | -1.02E-03 | NA | 2.05E-01 | 7 | -158.426 | 330.851 | 1.252 | 0.212 |
| -0.627 | 1.55E-09 | NA | + | NA | NA | 2.08E-01 | 7 | -158.462 | 330.924 | 1.324 | 0.205 |
| -0.479 | NA | NA | + | NA | -4.75E-02 | 2.02E-01 | 7 | -158.553 | 331.106 | 1.506 | 0.187 |
| -0.424 | NA | NA | + | NA | NA | 1.56E-01 | 6 | -159.829 | 331.658 | 0 | 0.393 |
| -0.453 | 1.07E-09 | NA | + | NA | NA | 1.61E-01 | 7 | -159.685 | 333.369 | 1.712 | 0.167 |
| -0.351 | NA | NA | NA | NA | NA | 1.95E-01 | 3 | -163.793 | 333.585 | 1.927 | 0.15 |
| -0.441 | NA | NA | + | -2.07E-04 | NA | 1.57E-01 | 7 | -159.816 | 333.632 | 1.974 | 0.146 |
| -0.434 | NA | NA | + | NA | 4.79E-03 | 1.56E-01 | 7 | -159.827 | 333.654 | 1.996 | 0.145 |
| -0.481 | NA | NA | + | NA | NA | 1.73E-01 | 6 | -155.882 | 323.763 | 0 | 0.447 |
| -0.516 | 1.31E-09 | NA | + | NA | NA | 1.79E-01 | 7 | -155.623 | 325.247 | 1.484 | 0.213 |
| -0.506 | NA | NA | + | -3.25E-04 | NA | 1.75E-01 | 7 | -155.847 | 325.693 | I.93 | 0.17 |
| -0.446 | NA | NA | + | NA | -1.62E-02 | 1.74E-01 | 7 | -155.855 | 325.711 | 1.948 | 0.169 |
| -0.102 | NA | NA | NA | 1.72E-03 | NA | 1.20E-01 | 4 | -156.722 | 321.443 | 0 | 0.333 |
| -0.228 | NA | NA | NA | NA | NA | 1.56E-01 | 3 | -158.27 | 322.541 | 1.098 | 0.193 |
| 0.235 | NA | NA | NA | 2.26E-03 | NA | NA | 3 | -158.354 | 322.707 | 1.264 | 0.177 |
| -0.207 | NA | NA | NA | 1.98E-03 | 5.98E-02 | 1.17E-01 | 5 | -156.424 | 322.847 | 1.404 | 0.165 |
| -0.123 | 6.30E-10 | NA | NA | 1.70E-03 | NA | 1.24E-01 | 5 | -156.648 | 323.297 | 1.853 | 0.132 |
| -0.573 | NA | NA | + | NA | NA | 1.87E-01 | 6 | -153.492 | 318.985 | 0 | 0.388 |
| -0.67 | NA | NA | + | -1.30E-03 | NA | 1.93E-01 | 7 | -152.884 | 319.768 | 0.783 | 0.262 |
| -0.607 | 1.11E-09 | NA | + | NA | NA | 1.93E-01 | 7 | -153.268 | 320.536 | 1.551 | 0.179 |
| -0.478 | NA | NA | + | NA | -4.14E-02 | 1.87E-01 | 7 | -153.313 | 320.626 | 1.641 | 0.171 |
| -0.412 | NA | NA | + | NA | NA | 1.58E-01 | 6 | -141.254 | 294.507 | 0 | 0.443 |
| -0.445 | 8.99E-10 | NA | + | NA | NA | 1.65E-01 | 7 | -141.005 | 296.011 | 1.504 | 0.209 |
| -0.355 | NA | NA | + | NA | -2.61E-02 | 1.59E-01 | 7 | -141.174 | 296.347 | I.84 | 0.177 |
| -0.443 | NA | NA | + | -3.70E-04 | NA | 1.60E-01 | 7 | -141.204 | 296.408 | 01.IX | 0.171 |
| -0.214 | NA | NA | NA | 1.41E-03 | NA | 1.52E-01 | 4 | -155.841 | 319.682 | 0 | 0.206 |
| -0.364 | NA | NA | + | NA | NA | 1.44E-01 | 6 | -153.852 | 319.704 | 0.022 | 0.204 |
| -0.316 | NA | NA | NA | NA | NA | 1.79E-01 | 3 | -156.965 | 319.929 | 0.247 | 0.182 |
| -0.391 | 8.25E-10 | NA | + | NA | NA | 1.50E-01 | 7 | -153.696 | 321.392 | I.71 | 0.088 |
| -0.242 | 7.90E-10 | NA | NA | 1.38E-03 | NA | 1.57E-01 | 5 | -155.702 | 321.403 | 1.721 | 0.087 |
| -0.346 | 9.00E-10 | NA | NA | NA | NA | 1.85E-01 | 4 | -156.786 | 321.572 | I.89 | 0.08 |
| -0.34 | NA | NA | + | 2.67E-04 | NA | 1.42E-01 | 7 | -153.831 | 321.662 | I.98 | 0.077 |
| -0.229 | NA | NA | NA | 1.45E-03 | 9.25E-03 | 1.51E-01 | 5 | -155.833 | 321.666 | 1.985 | 0.076 |
| -0.465 | NA | NA | + | NA | NA | 1.74E-01 | 6 | -151.103 | 314.206 | 0 | 0.429 |
| -0.499 | 1.12E-09 | NA | + | NA | NA | 1.80E-01 | 7 | -150.859 | 315.718 | 1.512 | 0.202 |
| -0.375 | NA | NA | + | NA | -4.01E-02 | 1.73E-01 | 7 | -150.927 | 315.853 | 1.647 | 0.188 |
| -0.514 | NA | NA | + | -6.16E-04 | NA | 1.77E-01 | 7 | -150.968 | 315.935 | 1.729 | 0.181 |
| -0.497 | NA | NA | + | NA | NA | 1.72E-01 | 6 | -173.617 | 359.234 | 0 | 0.456 |
| -0.532 | 1.16E-09 | NA | + | NA | NA | 1.79E-01 | 7 | -173.416 | 360.833 | 1.599 | 0.205 |
| -0.527 | NA | NA | + | NA | 1.35E-02 | 1.72E-01 | 7 | -173.602 | 361.203 | I.97 | 0.17 |
| -0.496 | NA | NA | + | 8.68E-06 | NA | 1.72E-01 | 7 | -173.617 | 361.234 | 2 | 0.168 |
| -0.471 | NA | NA | + | NA | NA | 1.67E-01 | 6 | -147.339 | 306.677 | 0 | 0.411 |
| -0.556 | NA | NA | + | -1.06E-03 | NA | 1.73E-01 | 7 | -146.92 | 307.84 | 1.163 | 0.23 |
| -0.5 | 9.59E-10 | NA | + | NA | NA | 1.72E-01 | 7 | -147.16 | 308.321 | 1.643 | 0.181 |
| -0.385 | NA | NA | + | NA | -3.90E-02 | 1.68E-01 | 7 | -147.173 | 308.346 | 1.669 | 0.178 |
| -0.543 | NA | NA | + | -1.78E-03 | -1.19E-01 | 2.38E-01 | 8 | -159.853 | 335.706 | 0 | 0.275 |
| -0.467 | NA | NA | + | NA | -1.02E-01 | 2.32E-01 | 7 | -161.167 | 336.334 | 0.628 | 0.201 |
| -0.605 | 1.46E-09 | NA | + | -1.77E-03 | -1.14E-01 | 2.49E-01 | 9 | -159.337 | 336.674 | 0.968 | 0.169 |
| -0.704 | NA | NA | + | NA | NA | 2.35E-01 | 6 | -162.625 | 337.251 | 1.545 | 0.127 |
| -0.53 | 1.48E-09 | NA | + | NA | -9.69E-02 | 2.43E-01 | 8 | -160.65 | 337.3 | 1.594 | 0.124 |
| -0.794 | NA | NA | + | -1.39E-03 | NA | 2.40E-01 | 7 | -161.815 | 337.63 | 1.924 | 0.105 |
| -0.396 | NA | NA | + | NA | NA | 1.52E-01 | 6 | -150.547 | 313.095 | 0 | 0.297 |
| -0.229 | NA | NA | NA | 1.48E-03 | NA | 1.59E-01 | 4 | -152.977 | 313.955 | 0.86 | 0.194 |
| -0.328 | NA | NA | NA | NA | NA | 1.87E-01 | 3 | -154.184 | 314.368 | 1.273 | 0.157 |
| -0.426 | 9.96E-10 | NA | + | NA | NA | 1.57E-01 | 7 | -150.381 | 314.762 | 1.667 | 0.129 |
| -0.373 | NA | NA | + | 2.72E-04 | NA | 1.50E-01 | 7 | -150.524 | 315.049 | 1.954 | 0.112 |
| -0.376 | NA | NA | + | NA | -9.26E-03 | 1.52E-01 | 7 | -150.539 | 315.078 | 1.983 | 0.11 |
| -0.366 | NA | NA | + | NA | NA | 1.42E-01 | 6 | -136.307 | 284.614 | 0 | 0.435 |
| -0.43 | NA | NA | + | -7.54E-04 | NA | 1.47E-01 | 7 | -136.09 | 286.18 | 1.566 | 0.199 |
| -0.393 | 9.34E-10 | NA | + | NA | NA | 1.46E-01 | 7 | -136.141 | 286.282 | 1.668 | 0.189 |
| -0.301 | NA | NA | + | NA | -2.96E-02 | 1.43E-01 | 7 | -136.208 | 286.415 | 1.802 | 0.177 |
| -0.614 | NA | NA | + | NA | NA | 2.10E-01 | 6 | -157.89 | 327.781 | 0 | 0.184 |
| -0.423 | NA | NA | + | NA | -8.64E-02 | 2.09E-01 | 7 | -156.964 | 327.927 | 0.147 | 0.171 |
| -0.489 | NA | NA | + | -1.52E-03 | -1.01E-01 | 2.13E-01 | 8 | -156.011 | 328.023 | 0.242 | 0.163 |
| -0.693 | NA | NA | + | -1.23E-03 | NA | 2.13E-01 | 7 | -157.257 | 328.515 | 0.734 | 0.128 |
| -0.66 | 1.47E-09 | NA | + | NA | NA | 2.19E-01 | 7 | -157.456 | 328.912 | 1.132 | 0.105 |
| -0.474 | 1.29E-09 | NA | + | NA | -8.19E-02 | 2.17E-01 | 8 | -156.626 | 329.253 | 1.472 | 0.088 |
| -0.541 | 1.32E-09 | NA | + | -1.53E-03 | -9.66E-02 | 2.21E-01 | 9 | -155.657 | 329.313 | 1.533 | 0.086 |
| -0.742 | 1.52E-09 | NA | + | -1.26E-03 | NA | 2.22E-01 | 8 | -156.792 | 329.583 | 1.803 | 0.075 |
| -0.451 | NA | NA | + | NA | NA | 1.68E-01 | 6 | -143.431 | 298.862 | 0 | 0.415 |
| -0.53 | NA | NA | + | -1.01E-03 | NA | 1.74E-01 | 7 | -143.046 | 300.093 | 1.231 | 0.225 |
| -0.48 | 9.90E-10 | NA | + | NA | NA | 1.74E-01 | 7 | -143.236 | 300.473 | 1.611 | 0.186 |
| -0.377 | NA | NA | + | NA | -3.45E-02 | 1.69E-01 | 7 | -143.298 | 300.597 | 1.735 | 0.174 |
| -0.585 | NA | NA | + | NA | NA | 2.06E-01 | 6 | -156.978 | 325.957 | 0 | 0.313 |
| -0.43 | NA | NA | + | NA | -6.85E-02 | 2.06E-01 | 7 | -156.439 | 326.879 | 0.922 | 0.197 |
| -0.63 | 1.51E-09 | NA | + | NA | NA | 2.14E-01 | 7 | -156.552 | 327.104 | 1.147 | 0.176 |
| -0.66 | NA | NA | + | -1.02E-03 | NA | 2.10E-01 | 7 | -156.56 | 327.12 | 1.163 | 0.175 |
| -0.493 | NA | NA | + | -1.28E-03 | -8.27E-02 | 2.11E-01 | 8 | -155.795 | 327.591 | 1.634 | 0.138 |
| -0.643 | NA | NA | + | NA | NA | 2.13E-01 | 6 | -169.337 | 350.675 | 0 | 0.405 |
| -0.693 | 1.88E-09 | NA | + | NA | NA | 2.20E-01 | 7 | -168.948 | 351.896 | 1.222 | 0.22 |
| -0.522 | NA | NA | + | NA | -5.38E-02 | 2.14E-01 | 7 | -169.024 | 352.047 | 1.373 | 0.204 |
| -0.687 | NA | NA | + | -6.29E-04 | NA | 2.16E-01 | 7 | -169.2 | 352.4 | 1.725 | 0.171 |
| -0.519 | NA | NA | + | NA | NA | 1.85E-01 | 6 | -155.191 | 322.382 | 0 | 0.427 |
| -0.557 | 1.37E-09 | NA | + | NA | NA | 1.91E-01 | 7 | -154.932 | 323.863 | 1.481 | 0.204 |
| -0.581 | NA | NA | + | -7.80E-04 | NA | 1.89E-01 | 7 | -154.975 | 323.95 | 1.567 | 0.195 |
| -0.447 | NA | NA | + | NA | -3.16E-02 | 1.85E-01 | 7 | -155.086 | 324.172 | 1.789 | 0.175 |
| -0.66 | NA | NA | + | NA | NA | 2.24E-01 | 6 | -151.931 | 315.862 | 0 | 0.211 |
| -0.743 | NA | NA | + | -1.39E-03 | NA | 2.26E-01 | 7 | -151.046 | 316.093 | 0.23 | 0.188 |
| -0.568 | NA | NA | + | -1.66E-03 | -8.62E-02 | 2.26E-01 | 8 | -150.17 | 316.339 | 0.477 | 0.167 |
| -0.511 | NA | NA | + | NA | -6.70E-02 | 2.24E-01 | 7 | -151.39 | 316.779 | 0.917 | 0.134 |
| -0.702 | 1.31E-09 | NA | + | NA | NA | 2.32E-01 | 7 | -151.562 | 317.124 | 1.262 | 0.113 |
| -0.787 | 1.35E-09 | NA | + | -1.41E-03 | NA | 2.35E-01 | 8 | -150.655 | 317.309 | 1.447 | 0.103 |
| -0.615 | 1.21E-09 | NA | + | -1.66E-03 | -8.26E-02 | 2.34E-01 | 9 | -149.849 | 317.697 | 1.835 | 0.084 |
| -0.602 | NA | NA | + | NA | NA | 2.04E-01 | 6 | -162.337 | 336.674 | 0 | 0.384 |
| -0.686 | NA | NA | + | -1.17E-03 | NA | 2.08E-01 | 7 | -161.833 | 337.667 | 0.993 | 0.234 |
| -0.645 | 1.51E-09 | NA | + | NA | NA | 2.12E-01 | 7 | -161.993 | 337.985 | 1.311 | 0.199 |
| -0.493 | NA | NA | + | NA | -4.89E-02 | 2.04E-01 | 7 | -162.075 | 338.15 | 1.476 | 0.183 |
| -0.406 | NA | NA | + | NA | NA | 1.47E-01 | 6 | -149.436 | 310.872 | 0 | 0.451 |
| -0.438 | 1.03E-09 | NA | + | NA | NA | 1.52E-01 | 7 | -149.242 | 312.485 | 1.612 | 0.202 |
| -0.354 | NA | NA | + | NA | -2.40E-02 | 1.48E-01 | 7 | -149.376 | 312.751 | 1.879 | 0.176 |
| -0.43 | NA | NA | + | -2.84E-04 | NA | 1.49E-01 | 7 | -149.409 | 312.819 | 1.946 | 0.171 |
| -0.399 | NA | NA | + | NA | NA | 1.59E-01 | 6 | -140.786 | 293.572 | 0 | 0.382 |
| -0.428 | 1.02E-09 | NA | + | NA | NA | 1.64E-01 | 7 | -140.568 | 295.136 | 1.564 | 0.175 |
| -0.347 | NA | NA | NA | NA | NA | 1.93E-01 | 3 | -144.731 | 295.461 | I.89 | 0.149 |
| -0.43 | NA | NA | + | -3.70E-04 | NA | 1.61E-01 | 7 | -140.738 | 295.475 | 1.903 | 0.148 |
| -0.361 | NA | NA | + | NA | -1.88E-02 | 1.61E-01 | 7 | -140.746 | 295.493 | 1.921 | 0.146 |
| -0.174 | NA | NA | NA | 1.32E-03 | NA | 1.40E-01 | 4 | -142.181 | 292.362 | 0 | 0.26 |
| -0.256 | NA | NA | NA | NA | NA | 1.62E-01 | 3 | -143.294 | 292.587 | 0.225 | 0.232 |
| -0.325 | NA | NA | + | NA | NA | 1.37E-01 | 6 | -140.419 | 292.837 | 0.475 | 0.205 |
| -0.197 | 7.10E-10 | NA | NA | 1.32E-03 | NA | 1.44E-01 | 5 | -142.065 | 294.129 | 1.767 | 0.107 |
| -0.206 | NA | NA | NA | 1.41E-03 | 1.91E-02 | 1.38E-01 | 5 | -142.145 | 294.289 | 1.927 | 0.099 |
| -0.279 | 7.24E-10 | NA | NA | NA | NA | 1.66E-01 | 4 | -143.174 | 294.349 | 1.986 | 0.096 |
| -0.34 | NA | NA | + | NA | NA | 1.36E-01 | 6 | -149.576 | 311.153 | 0 | 0.211 |
| -0.197 | NA | NA | NA | 1.35E-03 | NA | 1.49E-01 | 4 | -151.65 | 311.301 | 0.148 | 0.196 |
| -0.277 | NA | NA | NA | NA | NA | 1.73E-01 | 3 | -152.739 | 311.477 | 0.324 | 0.18 |
| -0.365 | 8.34E-10 | NA | + | NA | NA | 1.41E-01 | 7 | -149.433 | 312.866 | 1.713 | 0.09 |
| -0.397 | NA | NA | + | NA | 2.69E-02 | 1.34E-01 | 7 | -149.508 | 313.016 | 1.863 | 0.083 |
| -0.224 | 8.11E-10 | NA | NA | 1.32E-03 | NA | 1.54E-01 | 5 | -151.518 | 313.036 | 1.883 | 0.082 |
| -0.321 | NA | NA | + | 2.26E-04 | NA | 1.35E-01 | 7 | -149.56 | 313.12 | 1.967 | 0.079 |
| -0.307 | 9.42E-10 | NA | NA | NA | NA | 1.78E-01 | 4 | -152.562 | 313.124 | 1.971 | 0.079 |
| -0.554 | NA | NA | + | NA | NA | 1.88E-01 | 6 | -147.366 | 306.731 | 0 | 0.3 |
| -0.388 | NA | NA | + | NA | -7.29E-02 | 1.88E-01 | 7 | -146.709 | 307.419 | 0.687 | 0.212 |
| -0.628 | NA | NA | + | -1.03E-03 | NA | 1.93E-01 | 7 | -146.929 | 307.858 | 1.127 | 0.171 |
| -0.596 | 1.29E-09 | NA | + | NA | NA | 1.96E-01 | 7 | -146.985 | 307.971 | 1.239 | 0.161 |
| -0.45 | NA | NA | + | -1.31E-03 | -8.71E-02 | 1.94E-01 | 8 | -146.018 | 308.036 | 1.304 | 0.156 |
| -0.556 | NA | NA | + | NA | NA | 1.88E-01 | 6 | -155.331 | 322.663 | 0 | 0.273 |
| -0.657 | NA | NA | + | -1.37E-03 | NA | 1.93E-01 | 7 | -154.62 | 323.241 | 0.578 | 0.205 |
| -0.424 | NA | NA | + | NA | -5.81E-02 | 1.88E-01 | 7 | -154.945 | 323.89 | 1.227 | 0.148 |
| -0.507 | NA | NA | + | -1.57E-03 | -7.23E-02 | 1.94E-01 | 8 | -154.03 | 324.06 | 1.397 | 0.136 |
| -0.594 | 1.39E-09 | NA | + | NA | NA | 1.94E-01 | 7 | -155.035 | 324.071 | 1.408 | 0.135 |
| -0.697 | 1.42E-09 | NA | + | -1.39E-03 | NA | 1.99E-01 | 8 | -154.309 | 324.617 | 1.955 | 0.103 |
| -0.438 | NA | NA | + | NA | NA | 1.63E-01 | 6 | -140.847 | 293.695 | 0 | 0.428 |
| -0.517 | NA | NA | + | -9.55E-04 | NA | 1.69E-01 | 7 | -140.526 | 295.052 | 1.357 | 0.217 |
| -0.466 | 8.98E-10 | NA | + | NA | NA | 1.69E-01 | 7 | -140.666 | 295.332 | 1.638 | 0.189 |
| -0.39 | NA | NA | + | NA | -2.29E-02 | 1.65E-01 | 7 | -140.789 | 295.578 | 1.883 | 0.167 |
| -0.478 | NA | NA | + | NA | NA | 1.73E-01 | 6 | -145.373 | 302.746 | 0 | 0.334 |
| -0.554 | NA | NA | + | -1.04E-03 | NA | 1.77E-01 | 7 | -144.925 | 303.85 | 1.104 | 0.192 |
| -0.354 | NA | NA | + | NA | -5.73E-02 | 1.75E-01 | 7 | -144.987 | 303.975 | 1.229 | 0.181 |
| -0.515 | 1.19E-09 | NA | + | NA | NA | 1.79E-01 | 7 | -145.064 | 304.129 | 1.383 | 0.167 |
| -0.416 | NA | NA | + | -1.25E-03 | -7.05E-02 | 1.80E-01 | 8 | -144.357 | 304.713 | 1.968 | 0.125 |
| -0.499 | NA | NA | + | NA | NA | 1.74E-01 | 6 | -145.367 | 302.734 | 0 | 0.411 |
| -0.57 | NA | NA | + | -9.03E-04 | NA | 1.79E-01 | 7 | -145.064 | 304.128 | 1.394 | 0.205 |
| -0.533 | 1.13E-09 | NA | + | NA | NA | 1.80E-01 | 7 | -145.116 | 304.232 | 1.498 | 0.194 |
| -0.4 | NA | NA | + | NA | -4.48E-02 | 1.76E-01 | 7 | -145.136 | 304.273 | 1.539 | 0.19 |
| -0.593 | NA | NA | + | NA | NA | 2.05E-01 | 6 | -155.45 | 322.9 | 0 | 0.32 |
| -0.679 | NA | NA | + | -1.19E-03 | NA | 2.09E-01 | 7 | -154.871 | 323.741 | 0.841 | 0.21 |
| -0.639 | 1.56E-09 | NA | + | NA | NA | 2.12E-01 | 7 | -155.089 | 324.179 | 1.279 | 0.169 |
| -0.468 | NA | NA | + | NA | -5.54E-02 | 2.05E-01 | 7 | -155.103 | 324.206 | 1.306 | 0.166 |
| -0.534 | NA | NA | + | -1.42E-03 | -7.14E-02 | 2.10E-01 | 8 | -154.31 | 324.621 | 1.721 | 0.135 |
| -0.504 | NA | NA | + | NA | NA | 1.76E-01 | 6 | -152.602 | 317.204 | 0 | 0.388 |
| -0.372 | NA | NA | + | NA | -6.13E-02 | 1.79E-01 | 7 | -152.144 | 318.288 | 1.084 | 0.226 |
| -0.543 | 1.34E-09 | NA | + | NA | NA | 1.83E-01 | 7 | -152.271 | 318.542 | 1.337 | 0.199 |
| -0.568 | NA | NA | + | -8.53E-04 | NA | 1.79E-01 | 7 | -152.33 | 318.659 | 1.455 | 0.187 |
| -0.654 | NA | NA | + | NA | NA | 2.10E-01 | 6 | -163.061 | 338.121 | 0 | 0.383 |
| -0.725 | NA | NA | + | -1.06E-03 | NA | 2.13E-01 | 7 | -162.64 | 339.279 | 1.158 | 0.214 |
| -0.701 | 1.49E-09 | NA | + | NA | NA | 2.19E-01 | 7 | -162.682 | 339.364 | 1.243 | 0.206 |
| -0.526 | NA | NA | + | NA | -5.54E-02 | 2.10E-01 | 7 | -162.723 | 339.447 | 1.325 | 0.197 |
| -0.566 | NA | NA | + | NA | NA | 1.94E-01 | 6 | -156.37 | 324.741 | 0 | 0.423 |
| -0.604 | 1.22E-09 | NA | + | NA | NA | 2.01E-01 | 7 | -156.089 | 326.178 | 1.437 | 0.206 |
| -0.463 | NA | NA | + | NA | -4.57E-02 | 1.94E-01 | 7 | -156.145 | 326.29 | 1.549 | 0.195 |
| -0.607 | NA | NA | + | -5.77E-04 | NA | 1.96E-01 | 7 | -156.25 | 326.501 | I.76 | 0.176 |
| -0.526 | NA | NA | + | NA | NA | 1.80E-01 | 6 | -162.628 | 337.256 | 0 | 0.423 |
| -0.569 | 1.52E-09 | NA | + | NA | NA | 1.86E-01 | 7 | -162.315 | 338.63 | 1.374 | 0.213 |
| -0.428 | NA | NA | + | NA | -4.48E-02 | 1.81E-01 | 7 | -162.426 | 338.851 | 1.595 | 0.19 |
| -0.571 | NA | NA | + | -5.98E-04 | NA | 1.82E-01 | 7 | -162.512 | 339.025 | 1.769 | 0.175 |
| -0.572 | NA | NA | + | NA | NA | 1.95E-01 | 6 | -156.108 | 324.216 | 0 | 0.405 |
| -0.612 | 1.37E-09 | NA | + | NA | NA | 2.02E-01 | 7 | -155.788 | 325.577 | 1.361 | 0.205 |
| -0.457 | NA | NA | + | NA | -5.14E-02 | 1.96E-01 | 7 | -155.814 | 325.629 | 1.413 | 0.2 |
| -0.636 | NA | NA | + | -8.29E-04 | NA | 1.99E-01 | 7 | -155.859 | 325.718 | 1.503 | 0.191 |
| -0.465 | NA | NA | + | NA | NA | 1.64E-01 | 6 | -146.665 | 305.331 | 0 | 0.414 |
| -0.502 | 1.10E-09 | NA | + | NA | NA | 1.72E-01 | 7 | -146.373 | 306.746 | 1.415 | 0.204 |
| -0.526 | NA | NA | + | -7.91E-04 | NA | 1.69E-01 | 7 | -146.43 | 306.861 | I.53 | 0.192 |
| -0.363 | NA | NA | + | NA | -4.49E-02 | 1.65E-01 | 7 | -146.443 | 306.886 | 1.555 | 0.19 |
| -0.157 | NA | NA | NA | 2.00E-03 | NA | 1.35E-01 | 4 | -179.311 | 366.623 | 0 | 0.306 |
| 0.213 | NA | NA | NA | 2.54E-03 | NA | NA | 3 | -181.055 | 368.11 | 1.487 | 0.146 |
| -0.265 | NA | NA | NA | 2.25E-03 | 5.90E-02 | 1.32E-01 | 5 | -179.061 | 368.121 | 1.499 | 0.145 |
| -0.282 | NA | NA | NA | NA | NA | 1.72E-01 | 3 | -181.088 | 368.176 | 1.554 | 0.141 |
| -0.367 | NA | NA | + | NA | NA | 1.31E-01 | 6 | -178.102 | 368.205 | 1.582 | 0.139 |
| -0.184 | 6.54E-10 | NA | NA | 1.99E-03 | NA | 1.41E-01 | 5 | -179.217 | 368.435 | 1.812 | 0.124 |
| -0.595 | NA | NA | + | NA | NA | 2.00E-01 | 6 | -150.65 | 313.3 | 0 | 0.203 |
| -0.706 | NA | NA | + | -1.53E-03 | NA | 2.06E-01 | 7 | -149.707 | 313.414 | 0.113 | 0.192 |
| -0.531 | NA | NA | + | -1.81E-03 | -8.70E-02 | 2.08E-01 | 8 | -148.835 | 313.67 | 0.37 | 0.169 |
| -0.444 | NA | NA | + | NA | -6.74E-02 | 2.01E-01 | 7 | -150.116 | 314.232 | 0.932 | 0.128 |
| -0.638 | 1.25E-09 | NA | + | NA | NA | 2.09E-01 | 7 | -150.254 | 314.508 | 1.208 | 0.111 |
| -0.752 | 1.28E-09 | NA | + | -1.55E-03 | NA | 2.15E-01 | 8 | -149.284 | 314.568 | 1.268 | 0.108 |
| -0.58 | 1.19E-09 | NA | + | -1.82E-03 | -8.39E-02 | 2.16E-01 | 9 | -148.471 | 314.942 | 1.642 | 0.089 |
| -0.429 | NA | NA | + | NA | NA | 1.63E-01 | 6 | -152.762 | 317.523 | 0 | 0.383 |
| -0.462 | 1.02E-09 | NA | + | NA | NA | 1.69E-01 | 7 | -152.55 | 319.1 | 1.577 | 0.174 |
| -0.365 | NA | NA | NA | NA | NA | 2.05E-01 | 3 | -156.683 | 319.366 | 1.842 | 0.153 |
| -0.381 | NA | NA | + | NA | -2.19E-02 | 1.63E-01 | 7 | -152.712 | 319.425 | 1.901 | 0.148 |
| -0.442 | NA | NA | + | -1.57E-04 | NA | 1.64E-01 | 7 | -152.753 | 319.507 | 1.984 | 0.142 |
| -0.41 | NA | NA | + | NA | NA | 1.54E-01 | 6 | -144.172 | 300.344 | 0 | 0.44 |
| -0.44 | 9.58E-10 | NA | + | NA | NA | 1.60E-01 | 7 | -143.959 | 301.919 | 1.575 | 0.2 |
| -0.455 | NA | NA | + | -5.45E-04 | NA | 1.57E-01 | 7 | -144.066 | 302.131 | 1.787 | 0.18 |
| -0.345 | NA | NA | + | NA | -3.04E-02 | 1.56E-01 | 7 | -144.07 | 302.139 | 1.795 | 0.179 |
| -0.471 | NA | NA | + | NA | NA | 1.68E-01 | 6 | -150.788 | 313.576 | 0 | 0.428 |
| -0.506 | 1.18E-09 | NA | + | NA | NA | 1.73E-01 | 7 | -150.572 | 315.143 | 1.567 | 0.195 |
| -0.525 | NA | NA | + | -7.15E-04 | NA | 1.71E-01 | 7 | -150.59 | 315.18 | 1.605 | 0.192 |
| -0.386 | NA | NA | + | NA | -3.90E-02 | 1.69E-01 | 7 | -150.623 | 315.246 | 1.671 | 0.185 |
| -0.428 | NA | NA | + | NA | NA | 1.61E-01 | 6 | -158.001 | 328.002 | 0 | 0.458 |
| -0.457 | 1.14E-09 | NA | + | NA | NA | 1.65E-01 | 7 | -157.845 | 329.69 | 1.688 | 0.197 |
| -0.46 | NA | NA | + | -3.99E-04 | NA | 1.62E-01 | 7 | -157.95 | 329.901 | 1.899 | 0.177 |
| -0.432 | NA | NA | + | NA | 2.05E-03 | 1.60E-01 | 7 | -158.001 | 330.001 | 1.999 | 0.168 |
| -0.496 | NA | NA | + | NA | NA | 1.76E-01 | 6 | -170.046 | 352.092 | 0 | 0.45 |
| -0.535 | 1.48E-09 | NA | + | NA | NA | 1.81E-01 | 7 | -169.816 | 353.631 | 1.539 | 0.209 |
| -0.441 | NA | NA | + | NA | -2.41E-02 | 1.76E-01 | 7 | -169.991 | 353.983 | I.89 | 0.175 |
| -0.499 | NA | NA | + | -3.12E-05 | NA | 1.76E-01 | 7 | -170.046 | 354.092 | 1.999 | 0.166 |
| -0.531 | NA | NA | + | NA | NA | 1.86E-01 | 6 | -153.813 | 319.627 | 0 | 0.431 |
| -0.567 | 1.23E-09 | NA | + | NA | NA | 1.91E-01 | 7 | -153.565 | 321.129 | 1.503 | 0.203 |
| -0.58 | NA | NA | + | -6.69E-04 | NA | 1.88E-01 | 7 | -153.648 | 321.296 | I.67 | 0.187 |
| -0.455 | NA | NA | + | NA | -3.44E-02 | 1.86E-01 | 7 | -153.689 | 321.378 | 1.752 | 0.179 |
| -0.548 | NA | NA | + | NA | NA | 1.89E-01 | 6 | -147.949 | 307.899 | 0 | 0.249 |
| -0.649 | NA | NA | + | -1.32E-03 | NA | 1.95E-01 | 7 | -147.271 | 308.542 | 0.643 | 0.18 |
| -0.402 | NA | NA | + | NA | -6.73E-02 | 1.93E-01 | 7 | -147.381 | 308.762 | 0.864 | 0.161 |
| -0.488 | NA | NA | + | -1.60E-03 | -8.37E-02 | 2.01E-01 | 8 | -146.413 | 308.825 | 0.927 | 0.156 |
| -0.596 | 1.75E-09 | NA | + | NA | NA | 1.96E-01 | 7 | -147.479 | 308.959 | 01.VI | 0.146 |
| -0.698 | 1.76E-09 | NA | + | -1.32E-03 | NA | 2.02E-01 | 8 | -146.792 | 309.585 | 1.686 | 0.107 |
| -0.484 | NA | NA | + | NA | NA | 1.69E-01 | 6 | -158.441 | 328.881 | 0 | 0.453 |
| -0.514 | 1.06E-09 | NA | + | NA | NA | 1.73E-01 | 7 | -158.295 | 330.59 | 1.709 | 0.193 |
| -0.525 | NA | NA | + | -5.33E-04 | NA | 1.71E-01 | 7 | -158.352 | 330.704 | 1.823 | 0.182 |
| -0.525 | NA | NA | + | NA | 1.88E-02 | 1.67E-01 | 7 | -158.409 | 330.818 | 1.937 | 0.172 |
| -0.683 | NA | NA | + | NA | NA | 2.31E-01 | 6 | -160.161 | 332.322 | 0 | 0.194 |
| -0.499 | NA | NA | + | NA | -7.99E-02 | 2.29E-01 | 7 | -159.38 | 332.76 | 0.437 | 0.156 |
| -0.559 | NA | NA | + | -1.52E-03 | -9.68E-02 | 2.33E-01 | 8 | -158.41 | 332.82 | 0.498 | 0.151 |
| -0.761 | NA | NA | + | -1.21E-03 | NA | 2.34E-01 | 7 | -159.528 | 333.055 | 0.733 | 0.134 |
| -0.735 | 1.89E-09 | NA | + | NA | NA | 2.39E-01 | 7 | -159.661 | 333.322 | 1 | 0.117 |
| -0.558 | 1.67E-09 | NA | + | NA | -7.43E-02 | 2.37E-01 | 8 | -158.989 | 333.979 | 1.657 | 0.085 |
| -0.814 | 1.91E-09 | NA | + | -1.22E-03 | NA | 2.42E-01 | 8 | -159.016 | 334.031 | 1.709 | 0.082 |
| -0.616 | 1.64E-09 | NA | + | -1.51E-03 | -9.11E-02 | 2.40E-01 | 9 | -158.03 | 334.06 | 1.738 | 0.081 |
| -0.416 | NA | NA | + | NA | NA | 1.48E-01 | 6 | -155.146 | 322.292 | 0 | 0.323 |
| -0.24 | NA | NA | NA | 1.50E-03 | NA | 1.60E-01 | 4 | -157.778 | 323.556 | 1.264 | 0.172 |
| -0.444 | 8.26E-10 | NA | + | NA | NA | 1.53E-01 | 7 | -155.001 | 324.001 | 1.709 | 0.137 |
| -0.333 | NA | NA | NA | NA | NA | 1.88E-01 | 3 | -159.085 | 324.17 | 1.878 | 0.126 |
| -0.393 | NA | NA | + | 2.74E-04 | NA | 1.47E-01 | 7 | -155.122 | 324.244 | 1.952 | 0.122 |
| -0.388 | NA | NA | + | NA | -1.26E-02 | 1.49E-01 | 7 | -155.131 | 324.262 | 1.971 | 0.121 |
| -0.611 | NA | NA | + | -2.18E-03 | -1.02E-01 | 2.38E-01 | 8 | -154.297 | 324.595 | 0 | 0.264 |
| -0.817 | NA | NA | + | -1.83E-03 | NA | 2.39E-01 | 7 | -155.576 | 325.152 | 0.558 | 0.2 |
| -0.67 | 1.62E-09 | NA | + | -2.17E-03 | -9.71E-02 | 2.46E-01 | 9 | -153.825 | 325.651 | 1.056 | 0.156 |
| -0.7 | NA | NA | + | NA | NA | 2.33E-01 | 6 | -156.947 | 325.895 | 01.III | 0.138 |
| -0.873 | 1.83E-09 | NA | + | -1.84E-03 | NA | 2.49E-01 | 8 | -154.979 | 325.958 | 1.364 | 0.134 |
| -0.526 | NA | NA | + | NA | -7.77E-02 | 2.31E-01 | 7 | -156.195 | 326.39 | 1.795 | 0.108 |
| -0.521 | NA | NA | + | NA | NA | 1.80E-01 | 6 | -174.421 | 360.843 | 0 | 0.454 |
| -0.562 | 1.56E-09 | NA | + | NA | NA | 1.86E-01 | 7 | -174.199 | 362.399 | 1.556 | 0.209 |
| -0.507 | NA | NA | + | 1.80E-04 | NA | 1.79E-01 | 7 | -174.412 | 362.824 | 1.982 | 0.169 |
| -0.506 | NA | NA | + | NA | -7.35E-03 | 1.80E-01 | 7 | -174.417 | 362.833 | I.99 | 0.168 |
| -0.399 | NA | NA | + | NA | NA | 1.49E-01 | 6 | -153.602 | 319.204 | 0 | 0.319 |
| -0.239 | NA | NA | NA | 1.37E-03 | NA | 1.63E-01 | 4 | -156.296 | 320.592 | 1.388 | 0.159 |
| -0.326 | NA | NA | NA | NA | NA | 1.88E-01 | 3 | -157.34 | 320.679 | 1.475 | 0.152 |
| -0.424 | 8.78E-10 | NA | + | NA | NA | 1.53E-01 | 7 | -153.474 | 320.949 | 1.745 | 0.133 |
| -0.431 | NA | NA | + | NA | 1.47E-02 | 1.48E-01 | 7 | -153.582 | 321.165 | 1.961 | 0.12 |
| -0.396 | NA | NA | + | 3.42E-05 | NA | 1.49E-01 | 7 | -153.602 | 321.203 | 1.999 | 0.117 |
| -0.576 | NA | NA | + | NA | NA | 2.01E-01 | 6 | -162.602 | 337.204 | 0 | 0.405 |
| -0.452 | NA | NA | + | NA | -5.46E-02 | 2.01E-01 | 7 | -162.282 | 338.564 | 1.359 | 0.205 |
| -0.616 | 1.46E-09 | NA | + | NA | NA | 2.09E-01 | 7 | -162.304 | 338.608 | 1.404 | 0.201 |
| -0.642 | NA | NA | + | -8.45E-04 | NA | 2.06E-01 | 7 | -162.361 | 338.721 | 1.517 | 0.19 |
| -0.532 | NA | NA | + | NA | NA | 1.88E-01 | 6 | -153.048 | 318.096 | 0 | 0.419 |
| -0.573 | 1.37E-09 | NA | + | NA | NA | 1.96E-01 | 7 | -152.723 | 319.447 | 1.351 | 0.213 |
| -0.44 | NA | NA | + | NA | -4.15E-02 | 1.89E-01 | 7 | -152.856 | 319.712 | 1.616 | 0.187 |
| -0.583 | NA | NA | + | -6.73E-04 | NA | 1.92E-01 | 7 | -152.888 | 319.776 | I.68 | 0.181 |
| -0.348 | NA | NA | + | NA | NA | 1.45E-01 | 6 | -144.647 | 301.293 | 0 | 0.237 |
| -0.206 | NA | NA | NA | 1.29E-03 | NA | 1.54E-01 | 4 | -146.795 | 301.59 | 0.296 | 0.205 |
| -0.288 | NA | NA | NA | NA | NA | 1.78E-01 | 3 | -147.864 | 301.729 | 0.435 | 0.191 |
| -0.375 | 9.00E-10 | NA | + | NA | NA | 1.50E-01 | 7 | -144.48 | 302.961 | 1.667 | 0.103 |
| -0.327 | NA | NA | + | 2.47E-04 | NA | 1.43E-01 | 7 | -144.626 | 303.252 | 1.958 | 0.089 |
| -0.332 | NA | NA | + | NA | -7.52E-03 | 1.45E-01 | 7 | -144.641 | 303.282 | 1.989 | 0.088 |
| -0.233 | 8.76E-10 | NA | NA | 1.28E-03 | NA | 1.59E-01 | 5 | -146.641 | 303.283 | 1.989 | 0.088 |
| -0.545 | NA | NA | + | NA | NA | 1.92E-01 | 6 | -149.038 | 310.077 | 0 | 0.328 |
| -0.624 | NA | NA | + | -1.09E-03 | NA | 1.96E-01 | 7 | -148.548 | 311.097 | 01.II | 0.197 |
| -0.417 | NA | NA | + | NA | -5.79E-02 | 1.94E-01 | 7 | -148.65 | 311.299 | 1.223 | 0.178 |
| -0.582 | 1.12E-09 | NA | + | NA | NA | 2.00E-01 | 7 | -148.713 | 311.425 | 1.348 | 0.167 |
| -0.48 | NA | NA | + | -1.31E-03 | -7.21E-02 | 1.98E-01 | 8 | -147.962 | 311.924 | 1.847 | 0.13 |
| -0.47 | NA | NA | + | NA | NA | 1.68E-01 | 6 | -143.772 | 299.543 | 0 | 0.4 |
| -0.556 | NA | NA | + | -1.06E-03 | NA | 1.75E-01 | 7 | -143.338 | 300.677 | 1.133 | 0.227 |
| -0.504 | 1.22E-09 | NA | + | NA | NA | 1.74E-01 | 7 | -143.524 | 301.048 | 1.505 | 0.188 |
| -0.368 | NA | NA | + | NA | -4.52E-02 | 1.69E-01 | 7 | -143.543 | 301.087 | 1.544 | 0.185 |
| -0.505 | NA | NA | + | NA | NA | 1.76E-01 | 6 | -147.689 | 307.378 | 0 | 0.397 |
| -0.593 | NA | NA | + | -1.14E-03 | NA | 1.81E-01 | 7 | -147.201 | 308.402 | 1.024 | 0.238 |
| -0.542 | 1.11E-09 | NA | + | NA | NA | 1.83E-01 | 7 | -147.405 | 308.809 | 1.432 | 0.194 |
| -0.422 | NA | NA | + | NA | -3.74E-02 | 1.77E-01 | 7 | -147.534 | 309.068 | I.69 | 0.171 |
| -0.503 | NA | NA | + | NA | NA | 1.80E-01 | 6 | -146.076 | 304.153 | 0 | 0.388 |
| -0.365 | NA | NA | + | NA | -6.32E-02 | 1.82E-01 | 7 | -145.592 | 305.183 | 01.III | 0.232 |
| -0.539 | 1.41E-09 | NA | + | NA | NA | 1.86E-01 | 7 | -145.785 | 305.57 | 1.417 | 0.191 |
| -0.57 | NA | NA | + | -8.33E-04 | NA | 1.85E-01 | 7 | -145.798 | 305.595 | 1.443 | 0.189 |
| -0.437 | NA | NA | + | NA | NA | 1.61E-01 | 6 | -150.54 | 313.079 | 0 | 0.447 |
| -0.472 | 1.32E-09 | NA | + | NA | NA | 1.66E-01 | 7 | -150.315 | 314.631 | 1.552 | 0.206 |
| -0.477 | NA | NA | + | -4.92E-04 | NA | 1.64E-01 | 7 | -150.461 | 314.922 | 1.843 | 0.178 |
| -0.4 | NA | NA | + | NA | -1.67E-02 | 1.61E-01 | 7 | -150.51 | 315.02 | 1.941 | 0.169 |
| -0.488 | NA | NA | + | NA | NA | 1.70E-01 | 6 | -151.524 | 315.048 | 0 | 0.433 |
| -0.522 | 1.18E-09 | NA | + | NA | NA | 1.75E-01 | 7 | -151.293 | 316.585 | 1.537 | 0.201 |
| -0.543 | NA | NA | + | -7.07E-04 | NA | 1.73E-01 | 7 | -151.351 | 316.702 | 1.654 | 0.19 |
| -0.418 | NA | NA | + | NA | -3.13E-02 | 1.71E-01 | 7 | -151.424 | 316.849 | 1.801 | 0.176 |
| -0.522 | NA | NA | + | NA | NA | 1.83E-01 | 6 | -147.078 | 306.156 | 0 | 0.394 |
| -0.614 | NA | NA | + | -1.20E-03 | NA | 1.90E-01 | 7 | -146.545 | 307.089 | 0.933 | 0.247 |
| -0.556 | 1.16E-09 | NA | + | NA | NA | 1.90E-01 | 7 | -146.809 | 307.619 | 1.463 | 0.19 |
| -0.439 | NA | NA | + | NA | -3.73E-02 | 1.84E-01 | 7 | -146.92 | 307.84 | 1.684 | 0.17 |
| -0.444 | NA | NA | + | NA | NA | 1.64E-01 | 6 | -135.202 | 282.403 | 0 | 0.396 |
| -0.326 | NA | NA | + | NA | -5.39E-02 | 1.66E-01 | 7 | -134.839 | 283.678 | 1.275 | 0.209 |
| -0.518 | NA | NA | + | -9.05E-04 | NA | 1.68E-01 | 7 | -134.86 | 283.72 | 1.317 | 0.205 |
| -0.474 | 9.28E-10 | NA | + | NA | NA | 1.70E-01 | 7 | -134.939 | 283.878 | 1.475 | 0.189 |
| -0.436 | NA | NA | + | NA | NA | 1.60E-01 | 6 | -149.224 | 310.449 | 0 | 0.429 |
| -0.47 | 1.16E-09 | NA | + | NA | NA | 1.65E-01 | 7 | -148.996 | 311.992 | 1.544 | 0.198 |
| -0.348 | NA | NA | + | NA | -4.09E-02 | 1.62E-01 | 7 | -149.045 | 312.091 | 1.642 | 0.189 |
| -0.49 | NA | NA | + | -6.73E-04 | NA | 1.63E-01 | 7 | -149.065 | 312.13 | 1.681 | 0.185 |
| -0.585 | NA | NA | + | -2.13E-03 | -1.12E-01 | 2.39E-01 | 8 | -153.973 | 323.945 | 0 | 0.407 |
| -0.82 | NA | NA | + | -1.79E-03 | NA | 2.40E-01 | 7 | -155.58 | 325.161 | 1.215 | 0.222 |
| -0.64 | 1.54E-09 | NA | + | -2.12E-03 | -1.07E-01 | 2.46E-01 | 9 | -153.608 | 325.216 | 1.271 | 0.216 |
| -0.498 | NA | NA | + | NA | -9.03E-02 | 2.33E-01 | 7 | -155.939 | 325.877 | 1.932 | 0.155 |
| -0.473 | NA | NA | + | NA | NA | 1.73E-01 | 6 | -142.965 | 297.93 | 0 | 0.41 |
| -0.541 | NA | NA | + | -8.88E-04 | NA | 1.77E-01 | 7 | -142.649 | 299.299 | 1.369 | 0.207 |
| -0.369 | NA | NA | + | NA | -4.83E-02 | 1.75E-01 | 7 | -142.701 | 299.401 | 1.471 | 0.196 |
| -0.502 | 1.01E-09 | NA | + | NA | NA | 1.78E-01 | 7 | -142.75 | 299.5 | I.57 | 0.187 |
| -0.5 | NA | NA | + | NA | NA | 1.77E-01 | 6 | -146.312 | 304.623 | 0 | 0.418 |
| -0.392 | NA | NA | + | NA | -4.83E-02 | 1.78E-01 | 7 | -146.041 | 306.081 | 1.458 | 0.202 |
| -0.533 | 1.04E-09 | NA | + | NA | NA | 1.83E-01 | 7 | -146.095 | 306.19 | 1.567 | 0.191 |
| -0.558 | NA | NA | + | -7.38E-04 | NA | 1.81E-01 | 7 | -146.107 | 306.214 | 1.591 | 0.189 |
| -0.563 | NA | NA | + | NA | NA | 1.95E-01 | 6 | -156.272 | 324.544 | 0 | 0.243 |
| -0.379 | NA | NA | + | NA | -8.50E-02 | 1.98E-01 | 7 | -155.367 | 324.734 | 0.19 | 0.221 |
| -0.443 | NA | NA | + | -1.26E-03 | -9.73E-02 | 2.02E-01 | 8 | -154.712 | 325.424 | 0.88 | 0.157 |
| -0.611 | 1.84E-09 | NA | + | NA | NA | 2.02E-01 | 7 | -155.861 | 325.722 | 1.178 | 0.135 |
| -0.634 | NA | NA | + | -9.78E-04 | NA | 1.98E-01 | 7 | -155.873 | 325.745 | 1.201 | 0.134 |
| -0.43 | 1.57E-09 | NA | + | NA | -7.97E-02 | 2.03E-01 | 8 | -155.07 | 326.14 | 1.596 | 0.11 |
| -0.482 | NA | NA | + | NA | NA | 1.71E-01 | 6 | -164.326 | 340.651 | 0 | 0.46 |
| -0.514 | 1.24E-09 | NA | + | NA | NA | 1.76E-01 | 7 | -164.156 | 342.311 | I.66 | 0.201 |
| -0.469 | NA | NA | + | NA | -5.81E-03 | 1.71E-01 | 7 | -164.323 | 342.646 | 1.994 | 0.17 |
| -0.486 | NA | NA | + | -5.70E-05 | NA | 1.71E-01 | 7 | -164.325 | 342.649 | 1.998 | 0.169 |
| -0.424 | NA | NA | + | NA | NA | 1.63E-01 | 6 | -152.688 | 317.376 | 0 | 0.448 |
| -0.46 | 1.26E-09 | NA | + | NA | NA | 1.69E-01 | 7 | -152.442 | 318.884 | 1.508 | 0.211 |
| -0.451 | NA | NA | + | -3.28E-04 | NA | 1.65E-01 | 7 | -152.652 | 319.304 | 1.928 | 0.171 |
| -0.388 | NA | NA | + | NA | -1.62E-02 | 1.63E-01 | 7 | -152.661 | 319.322 | 1.946 | 0.17 |
| -0.36 | NA | NA | + | NA | NA | 1.45E-01 | 6 | -143.578 | 299.155 | 0 | 0.33 |
| -0.311 | NA | NA | NA | NA | NA | 1.79E-01 | 3 | -147.343 | 300.685 | I.53 | 0.153 |
| -0.384 | 9.19E-10 | NA | + | NA | NA | 1.48E-01 | 7 | -143.453 | 300.906 | I.75 | 0.137 |
| -0.232 | NA | NA | NA | 1.14E-03 | NA | 1.57E-01 | 4 | -146.503 | 301.007 | 1.851 | 0.131 |
| -0.324 | NA | NA | + | NA | -1.71E-02 | 1.46E-01 | 7 | -143.546 | 301.092 | 1.936 | 0.125 |
| -0.382 | NA | NA | + | -2.64E-04 | NA | 1.46E-01 | 7 | -143.555 | 301.109 | 1.954 | 0.124 |
| -0.362 | NA | NA | + | NA | NA | 1.42E-01 | 6 | -151.344 | 314.688 | 0 | 0.235 |
| -0.201 | NA | NA | NA | 1.40E-03 | NA | 1.51E-01 | 4 | -153.453 | 314.906 | 0.218 | 0.211 |
| -0.29 | NA | NA | NA | NA | NA | 1.76E-01 | 3 | -154.581 | 315.163 | 0.475 | 0.185 |
| -0.391 | 7.71E-10 | NA | + | NA | NA | 1.48E-01 | 7 | -151.167 | 316.333 | 1.645 | 0.103 |
| -0.23 | 7.23E-10 | NA | NA | 1.37E-03 | NA | 1.57E-01 | 5 | -153.301 | 316.603 | 1.915 | 0.09 |
| -0.343 | NA | NA | + | 2.18E-04 | NA | 1.41E-01 | 7 | -151.33 | 316.659 | 1.971 | 0.088 |
| -0.342 | NA | NA | + | NA | -9.46E-03 | 1.43E-01 | 7 | -151.335 | 316.671 | 1.983 | 0.087 |
| -0.503 | NA | NA | + | NA | NA | 1.80E-01 | 6 | -146.336 | 304.671 | 0 | 0.419 |
| -0.541 | 1.41E-09 | NA | + | NA | NA | 1.85E-01 | 7 | -146.059 | 306.118 | 1.447 | 0.203 |
| -0.408 | NA | NA | + | NA | -4.44E-02 | 1.83E-01 | 7 | -146.105 | 306.21 | 1.538 | 0.194 |
| -0.556 | NA | NA | + | -6.78E-04 | NA | 1.83E-01 | 7 | -146.164 | 306.328 | 1.657 | 0.183 |
| -0.406 | NA | NA | + | NA | NA | 1.55E-01 | 6 | -138.233 | 288.465 | 0 | 0.419 |
| -0.484 | NA | NA | + | -9.72E-04 | NA | 1.60E-01 | 7 | -137.872 | 289.743 | 1.278 | 0.221 |
| -0.433 | 8.50E-10 | NA | + | NA | NA | 1.60E-01 | 7 | -138.057 | 290.114 | 1.649 | 0.184 |
| -0.334 | NA | NA | + | NA | -3.36E-02 | 1.56E-01 | 7 | -138.095 | 290.189 | 1.724 | 0.177 |
| -0.581 | NA | NA | + | NA | NA | 1.98E-01 | 6 | -156.032 | 324.064 | 0 | 0.308 |
| -0.681 | NA | NA | + | -1.30E-03 | NA | 2.04E-01 | 7 | -155.421 | 324.842 | 0.778 | 0.209 |
| -0.441 | NA | NA | + | NA | -6.11E-02 | 1.98E-01 | 7 | -155.596 | 325.193 | 1.129 | 0.175 |
| -0.623 | 1.62E-09 | NA | + | NA | NA | 2.05E-01 | 7 | -155.7 | 325.4 | 1.336 | 0.158 |
| -0.525 | NA | NA | + | -1.54E-03 | -7.63E-02 | 2.05E-01 | 8 | -154.756 | 325.513 | 1.449 | 0.149 |
| -0.448 | NA | NA | + | NA | NA | 1.67E-01 | 6 | -145.726 | 303.452 | 0 | 0.437 |
| -0.504 | NA | NA | + | -7.05E-04 | NA | 1.71E-01 | 7 | -145.546 | 305.092 | 1.639 | 0.193 |
| -0.477 | 9.93E-10 | NA | + | NA | NA | 1.73E-01 | 7 | -145.551 | 305.102 | I.65 | 0.192 |
| -0.382 | NA | NA | + | NA | -3.04E-02 | 1.69E-01 | 7 | -145.625 | 305.249 | 1.797 | 0.178 |
| -0.423 | NA | NA | + | NA | NA | 1.56E-01 | 6 | -159.762 | 331.525 | 0 | 0.324 |
| -0.262 | NA | NA | NA | 1.43E-03 | NA | 1.70E-01 | 4 | -162.497 | 332.994 | 1.469 | 0.156 |
| -0.352 | NA | NA | NA | NA | NA | 1.95E-01 | 3 | -163.582 | 333.163 | 1.638 | 0.143 |
| -0.451 | 1.01E-09 | NA | + | NA | NA | 1.60E-01 | 7 | -159.622 | 333.243 | 1.718 | 0.137 |
| -0.413 | NA | NA | + | 1.20E-04 | NA | 1.55E-01 | 7 | -159.758 | 333.516 | 1.991 | 0.12 |
| -0.435 | NA | NA | + | NA | 5.82E-03 | 1.56E-01 | 7 | -159.759 | 333.519 | 1.994 | 0.12 |
| -0.701 | NA | NA | + | NA | NA | 2.30E-01 | 6 | -162.277 | 336.553 | 0 | 0.179 |
| -0.583 | NA | NA | + | -1.74E-03 | -9.84E-02 | 2.31E-01 | 8 | -160.347 | 336.693 | 0.14 | 0.167 |
| -0.795 | NA | NA | + | -1.41E-03 | NA | 2.34E-01 | 7 | -161.466 | 336.933 | 0.38 | 0.148 |
| -0.514 | NA | NA | + | NA | -7.86E-02 | 2.26E-01 | 7 | -161.544 | 337.088 | 0.535 | 0.137 |
| -0.754 | 1.87E-09 | NA | + | NA | NA | 2.39E-01 | 7 | -161.765 | 337.531 | 0.977 | 0.11 |
| -0.641 | 1.64E-09 | NA | + | -1.72E-03 | -9.32E-02 | 2.39E-01 | 9 | -159.948 | 337.896 | 1.343 | 0.091 |
| -0.848 | 1.87E-09 | NA | + | -1.41E-03 | NA | 2.43E-01 | 8 | -160.95 | 337.899 | 1.346 | 0.091 |
| -0.575 | 1.69E-09 | NA | + | NA | -7.34E-02 | 2.34E-01 | 8 | -161.127 | 338.255 | 1.702 | 0.076 |
| -0.383 | NA | NA | + | NA | NA | 1.47E-01 | 6 | -144.208 | 300.416 | 0 | 0.333 |
| -0.313 | NA | NA | NA | NA | NA | 1.84E-01 | 3 | -148.014 | 302.028 | 1.611 | 0.149 |
| -0.411 | 9.25E-10 | NA | + | NA | NA | 1.52E-01 | 7 | -144.043 | 302.085 | 1.669 | 0.145 |
| -0.407 | NA | NA | + | -2.92E-04 | NA | 1.49E-01 | 7 | -144.179 | 302.358 | 1.942 | 0.126 |
| -0.245 | NA | NA | NA | 1.15E-03 | NA | 1.64E-01 | 4 | -147.187 | 302.374 | 1.958 | 0.125 |
| -0.39 | NA | NA | + | NA | 3.28E-03 | 1.47E-01 | 7 | -144.207 | 302.414 | 1.998 | 0.123 |
| -0.503 | NA | NA | + | NA | NA | 1.79E-01 | 6 | -152.176 | 316.351 | 0 | 0.391 |
| -0.583 | NA | NA | + | -1.10E-03 | NA | 1.83E-01 | 7 | -151.736 | 317.472 | 1.121 | 0.223 |
| -0.541 | 1.20E-09 | NA | + | NA | NA | 1.87E-01 | 7 | -151.867 | 317.735 | 1.384 | 0.196 |
| -0.39 | NA | NA | + | NA | -5.12E-02 | 1.79E-01 | 7 | -151.895 | 317.79 | 1.439 | 0.19 |
| -0.461 | NA | NA | + | NA | NA | 1.70E-01 | 6 | -161.184 | 334.368 | 0 | 0.457 |
| -0.494 | 9.24E-10 | NA | + | NA | NA | 1.78E-01 | 7 | -160.979 | 335.959 | 1.591 | 0.206 |
| -0.458 | NA | NA | + | 3.72E-05 | NA | 1.70E-01 | 7 | -161.184 | 336.368 | 1.999 | 0.168 |
| -0.46 | NA | NA | + | NA | -4.95E-04 | 1.70E-01 | 7 | -161.184 | 336.368 | 2 | 0.168 |
| -0.567 | NA | NA | + | -2.16E-03 | -1.21E-01 | 2.34E-01 | 8 | -155.614 | 327.228 | 0 | 0.506 |
| -0.623 | 1.53E-09 | NA | + | -2.14E-03 | -1.16E-01 | 2.41E-01 | 9 | -155.222 | 328.444 | 1.216 | 0.275 |
| -0.819 | NA | NA | + | -1.78E-03 | NA | 2.36E-01 | 7 | -157.452 | 328.904 | 1.676 | 0.219 |
| -0.506 | NA | NA | + | NA | NA | 1.82E-01 | 6 | -148.507 | 309.014 | 0 | 0.336 |
| -0.59 | NA | NA | + | -1.09E-03 | NA | 1.87E-01 | 7 | -148.047 | 310.094 | 01.VIII | 0.196 |
| -0.378 | NA | NA | + | NA | -5.73E-02 | 1.82E-01 | 7 | -148.131 | 310.263 | 1.249 | 0.18 |
| -0.541 | 1.06E-09 | NA | + | NA | NA | 1.89E-01 | 7 | -148.236 | 310.473 | 1.459 | 0.162 |
| -0.449 | NA | NA | + | -1.31E-03 | -7.13E-02 | 1.89E-01 | 8 | -147.481 | 310.961 | 1.947 | 0.127 |
| -0.429 | NA | NA | + | NA | NA | 1.57E-01 | 6 | -145.674 | 303.348 | 0 | 0.428 |
| -0.334 | NA | NA | + | NA | -4.32E-02 | 1.58E-01 | 7 | -145.467 | 304.934 | 1.586 | 0.194 |
| -0.492 | NA | NA | + | -7.30E-04 | NA | 1.62E-01 | 7 | -145.486 | 304.971 | 1.624 | 0.19 |
| -0.456 | 9.79E-10 | NA | + | NA | NA | 1.61E-01 | 7 | -145.5 | 304.999 | 1.651 | 0.188 |
| -0.388 | NA | NA | + | NA | NA | 1.50E-01 | 6 | -145.264 | 302.529 | 0 | 0.38 |
| -0.417 | 1.06E-09 | NA | + | NA | NA | 1.54E-01 | 7 | -145.106 | 304.212 | 1.684 | 0.164 |
| -0.347 | NA | NA | NA | NA | NA | 1.93E-01 | 3 | -149.152 | 304.305 | 1.776 | 0.157 |
| -0.436 | NA | NA | + | -5.58E-04 | NA | 1.54E-01 | 7 | -145.163 | 304.326 | 1.797 | 0.155 |
| -0.352 | NA | NA | + | NA | -1.68E-02 | 1.50E-01 | 7 | -145.235 | 304.471 | 1.942 | 0.144 |
| -0.464 | NA | NA | + | NA | NA | 1.70E-01 | 6 | -150.05 | 312.101 | 0 | 0.406 |
| -0.542 | NA | NA | + | -9.35E-04 | NA | 1.76E-01 | 7 | -149.727 | 313.454 | 1.353 | 0.206 |
| -0.351 | NA | NA | + | NA | -5.16E-02 | 1.71E-01 | 7 | -149.766 | 313.531 | I.43 | 0.199 |
| -0.497 | 1.00E-09 | NA | + | NA | NA | 1.77E-01 | 7 | -149.817 | 313.633 | 1.532 | 0.189 |
| -0.578 | NA | NA | + | NA | NA | 2.02E-01 | 6 | -148.181 | 308.361 | 0 | 0.315 |
| -0.43 | NA | NA | + | NA | -6.55E-02 | 2.02E-01 | 7 | -147.667 | 309.333 | 0.972 | 0.194 |
| -0.653 | NA | NA | + | -1.06E-03 | NA | 2.07E-01 | 7 | -147.722 | 309.444 | 1.082 | 0.183 |
| -0.618 | 1.38E-09 | NA | + | NA | NA | 2.09E-01 | 7 | -147.838 | 309.676 | 1.315 | 0.163 |
| -0.489 | NA | NA | + | -1.33E-03 | -8.10E-02 | 2.08E-01 | 8 | -146.962 | 309.925 | 1.563 | 0.144 |
| -0.796 | NA | NA | + | NA | NA | 2.52E-01 | 6 | -175.867 | 363.733 | 0 | 0.217 |
| -0.598 | NA | NA | + | NA | -8.55E-02 | 2.50E-01 | 7 | -175.007 | 364.014 | 0.281 | 0.189 |
| -0.861 | 2.37E-09 | NA | + | NA | NA | 2.62E-01 | 7 | -175.257 | 364.514 | 0.781 | 0.147 |
| -0.646 | NA | NA | + | -1.32E-03 | -9.75E-02 | 2.51E-01 | 8 | -174.346 | 364.692 | 0.959 | 0.134 |
| -0.857 | NA | NA | + | -1.05E-03 | NA | 2.53E-01 | 7 | -175.443 | 364.886 | 1.153 | 0.122 |
| -0.672 | 2.05E-09 | NA | + | NA | -7.75E-02 | 2.59E-01 | 8 | -174.557 | 365.114 | 1.381 | 0.109 |
| -0.92 | 2.34E-09 | NA | + | -1.03E-03 | NA | 2.63E-01 | 8 | -174.847 | 365.693 | I.96 | 0.082 |
| -0.479 | NA | NA | + | NA | NA | 1.71E-01 | 6 | -162.049 | 336.098 | 0 | 0.456 |
| -0.51 | 1.14E-09 | NA | + | NA | NA | 1.76E-01 | 7 | -161.878 | 337.756 | 1.658 | 0.199 |
| -0.511 | NA | NA | + | -3.87E-04 | NA | 1.73E-01 | 7 | -162.002 | 338.004 | 1.906 | 0.176 |
| -0.458 | NA | NA | + | NA | -9.44E-03 | 1.71E-01 | 7 | -162.041 | 338.081 | 1.984 | 0.169 |
| -0.13 | NA | NA | NA | 1.53E-03 | NA | 1.24E-01 | 4 | -144.218 | 296.436 | 0 | 0.327 |
| -0.294 | NA | NA | + | NA | NA | 1.21E-01 | 6 | -142.597 | 297.193 | 0.757 | 0.224 |
| -0.238 | NA | NA | NA | NA | NA | 1.54E-01 | 3 | -145.753 | 297.506 | 01.VII | 0.192 |
| -0.153 | 6.79E-10 | NA | NA | 1.52E-03 | NA | 1.29E-01 | 5 | -144.106 | 298.211 | 1.775 | 0.135 |
| -0.149 | NA | NA | NA | 1.58E-03 | 1.11E-02 | 1.24E-01 | 5 | -144.205 | 298.411 | 1.975 | 0.122 |
| -0.641 | NA | NA | + | NA | NA | 2.14E-01 | 6 | -170.612 | 353.225 | 0 | 0.408 |
| -0.689 | 1.70E-09 | NA | + | NA | NA | 2.22E-01 | 7 | -170.267 | 354.534 | 1.309 | 0.212 |
| -0.704 | NA | NA | + | -9.03E-04 | NA | 2.17E-01 | 7 | -170.338 | 354.675 | 1.451 | 0.197 |
| -0.541 | NA | NA | + | NA | -4.43E-02 | 2.14E-01 | 7 | -170.413 | 354.825 | 01.VI | 0.183 |
| -0.569 | NA | NA | + | NA | NA | 2.02E-01 | 6 | -166.706 | 345.412 | 0 | 0.433 |
| -0.605 | 1.29E-09 | NA | + | NA | NA | 2.09E-01 | 7 | -166.464 | 346.927 | 1.515 | 0.203 |
| -0.626 | NA | NA | + | -7.80E-04 | NA | 2.06E-01 | 7 | -166.51 | 347.02 | 1.608 | 0.194 |
| -0.514 | NA | NA | + | NA | -2.50E-02 | 2.03E-01 | 7 | -166.644 | 347.288 | 1.876 | 0.17 |
| -0.37 | NA | NA | + | NA | NA | 1.42E-01 | 6 | -151.484 | 314.967 | 0 | 0.289 |
| -0.218 | NA | NA | NA | 1.42E-03 | NA | 1.54E-01 | 4 | -153.876 | 315.753 | 0.785 | 0.195 |
| -0.312 | NA | NA | NA | NA | NA | 1.80E-01 | 3 | -154.982 | 315.965 | 0.998 | 0.176 |
| -0.4 | 1.09E-09 | NA | + | NA | NA | 1.47E-01 | 7 | -151.322 | 316.645 | 1.678 | 0.125 |
| -0.35 | NA | NA | + | 2.44E-04 | NA | 1.41E-01 | 7 | -151.465 | 316.93 | 1.962 | 0.108 |
| -0.375 | NA | NA | + | NA | 2.06E-03 | 1.42E-01 | 7 | -151.483 | 316.966 | 1.999 | 0.106 |
| -0.442 | NA | NA | + | NA | NA | 1.66E-01 | 6 | -138.726 | 289.452 | 0 | 0.394 |
| -0.326 | NA | NA | + | NA | -5.45E-02 | 1.67E-01 | 7 | -138.346 | 290.692 | 1.241 | 0.212 |
| -0.513 | NA | NA | + | -9.12E-04 | NA | 1.70E-01 | 7 | -138.384 | 290.768 | 1.317 | 0.204 |
| -0.478 | 1.15E-09 | NA | + | NA | NA | 1.72E-01 | 7 | -138.449 | 290.899 | 1.447 | 0.191 |
| -0.485 | NA | NA | + | -1.87E-03 | -1.29E-01 | 2.27E-01 | 8 | -150.163 | 316.326 | 0 | 0.468 |
| -0.409 | NA | NA | + | NA | -1.08E-01 | 2.21E-01 | 7 | -151.715 | 317.43 | 1.104 | 0.269 |
| -0.541 | 1.55E-09 | NA | + | -1.84E-03 | -1.23E-01 | 2.35E-01 | 9 | -149.741 | 317.483 | 1.156 | 0.263 |
| -0.493 | NA | NA | + | NA | NA | 1.75E-01 | 6 | -160.648 | 333.297 | 0 | 0.429 |
| -0.56 | NA | NA | + | -8.59E-04 | NA | 1.79E-01 | 7 | -160.397 | 334.794 | 1.497 | 0.203 |
| -0.529 | 1.31E-09 | NA | + | NA | NA | 1.81E-01 | 7 | -160.421 | 334.841 | 1.544 | 0.198 |
| -0.429 | NA | NA | + | NA | -2.83E-02 | 1.76E-01 | 7 | -160.573 | 335.146 | 1.849 | 0.17 |
| -0.48 | NA | NA | + | NA | NA | 1.69E-01 | 6 | -147.093 | 306.187 | 0 | 0.393 |
| -0.56 | NA | NA | + | -9.99E-04 | NA | 1.74E-01 | 7 | -146.727 | 307.454 | 1.267 | 0.208 |
| -0.357 | NA | NA | + | NA | -5.56E-02 | 1.71E-01 | 7 | -146.732 | 307.464 | 1.277 | 0.207 |
| -0.517 | 1.30E-09 | NA | + | NA | NA | 1.75E-01 | 7 | -146.812 | 307.624 | 1.437 | 0.191 |
| -0.438 | NA | NA | + | NA | NA | 1.67E-01 | 6 | -145.426 | 302.852 | 0 | 0.445 |
| -0.473 | 1.15E-09 | NA | + | NA | NA | 1.72E-01 | 7 | -145.161 | 304.322 | I.47 | 0.213 |
| -0.468 | NA | NA | + | -3.85E-04 | NA | 1.68E-01 | 7 | -145.372 | 304.744 | 1.892 | 0.173 |
| -0.401 | NA | NA | + | NA | -1.73E-02 | 1.68E-01 | 7 | -145.393 | 304.787 | 1.935 | 0.169 |
| -0.417 | NA | NA | + | NA | NA | 1.59E-01 | 6 | -145.591 | 303.183 | 0 | 0.452 |
| -0.445 | 1.05E-09 | NA | + | NA | NA | 1.63E-01 | 7 | -145.426 | 304.852 | 1.669 | 0.196 |
| -0.456 | NA | NA | + | -4.61E-04 | NA | 1.62E-01 | 7 | -145.518 | 305.036 | 1.853 | 0.179 |
| -0.376 | NA | NA | + | NA | -1.88E-02 | 1.60E-01 | 7 | -145.554 | 305.107 | 1.925 | 0.173 |
| -0.435 | NA | NA | + | NA | NA | 1.59E-01 | 6 | -148.107 | 308.214 | 0 | 0.438 |
| -0.497 | NA | NA | + | -7.36E-04 | NA | 1.64E-01 | 7 | -147.914 | 309.827 | 1.614 | 0.196 |
| -0.463 | 1.04E-09 | NA | + | NA | NA | 1.64E-01 | 7 | -147.925 | 309.85 | 1.636 | 0.193 |
| -0.38 | NA | NA | + | NA | -2.56E-02 | 1.60E-01 | 7 | -148.04 | 310.08 | 1.866 | 0.172 |
| -0.423 | NA | NA | + | NA | NA | 1.59E-01 | 6 | -141.403 | 294.805 | 0 | 0.434 |
| -0.492 | NA | NA | + | -8.74E-04 | NA | 1.63E-01 | 7 | -141.122 | 296.244 | 1.439 | 0.211 |
| -0.449 | 9.08E-10 | NA | + | NA | NA | 1.63E-01 | 7 | -141.23 | 296.46 | 1.655 | 0.19 |
| -0.386 | NA | NA | + | NA | -1.77E-02 | 1.60E-01 | 7 | -141.369 | 296.738 | 1.932 | 0.165 |
| -0.461 | NA | NA | + | NA | NA | 1.69E-01 | 6 | -153.589 | 319.178 | 0 | 0.431 |
| -0.504 | 1.57E-09 | NA | + | NA | NA | 1.75E-01 | 7 | -153.269 | 320.538 | I.36 | 0.218 |
| -0.374 | NA | NA | + | NA | -4.00E-02 | 1.71E-01 | 7 | -153.414 | 320.828 | I.65 | 0.189 |
| -0.483 | NA | NA | + | -2.66E-04 | NA | 1.71E-01 | 7 | -153.564 | 321.128 | I.95 | 0.162 |
| -0.564 | NA | NA | + | NA | NA | 1.97E-01 | 6 | -153.694 | 319.389 | 0 | 0.301 |
| -0.655 | NA | NA | + | -1.20E-03 | NA | 2.02E-01 | 7 | -153.099 | 320.198 | 0.809 | 0.201 |
| -0.417 | NA | NA | + | NA | -6.55E-02 | 1.98E-01 | 7 | -153.188 | 320.376 | 0.988 | 0.184 |
| -0.603 | 1.15E-09 | NA | + | NA | NA | 2.05E-01 | 7 | -153.347 | 320.693 | 1.305 | 0.157 |
| -0.492 | NA | NA | + | -1.45E-03 | -8.07E-02 | 2.04E-01 | 8 | -152.347 | 320.694 | 1.306 | 0.157 |
| -0.379 | NA | NA | + | NA | NA | 1.44E-01 | 6 | -143.547 | 299.094 | 0 | 0.449 |
| -0.409 | 1.03E-09 | NA | + | NA | NA | 1.49E-01 | 7 | -143.359 | 300.718 | 1.624 | 0.199 |
| -0.419 | NA | NA | + | -4.74E-04 | NA | 1.46E-01 | 7 | -143.468 | 300.937 | 1.843 | 0.179 |
| -0.335 | NA | NA | + | NA | -2.00E-02 | 1.44E-01 | 7 | -143.503 | 301.006 | 1.912 | 0.173 |
| -0.429 | NA | NA | + | NA | NA | 1.58E-01 | 6 | -143.115 | 298.231 | 0 | 0.43 |
| -0.5 | NA | NA | + | -8.61E-04 | NA | 1.62E-01 | 7 | -142.852 | 299.704 | 1.473 | 0.206 |
| -0.459 | 9.38E-10 | NA | + | NA | NA | 1.64E-01 | 7 | -142.897 | 299.794 | 1.563 | 0.197 |
| -0.377 | NA | NA | + | NA | -2.33E-02 | 1.59E-01 | 7 | -143.057 | 300.113 | 1.883 | 0.168 |
| -0.41 | NA | NA | + | NA | NA | 1.57E-01 | 6 | -136.138 | 284.276 | 0 | 0.433 |
| -0.47 | NA | NA | + | -7.39E-04 | NA | 1.61E-01 | 7 | -135.928 | 285.856 | I.58 | 0.197 |
| -0.438 | 9.12E-10 | NA | + | NA | NA | 1.62E-01 | 7 | -135.957 | 285.915 | 1.638 | 0.191 |
| -0.34 | NA | NA | + | NA | -3.20E-02 | 1.58E-01 | 7 | -136.018 | 286.036 | I.76 | 0.18 |
| -0.498 | NA | NA | + | NA | NA | 1.79E-01 | 6 | -155.895 | 323.791 | 0 | 0.431 |
| -0.533 | 1.26E-09 | NA | + | NA | NA | 1.85E-01 | 7 | -155.64 | 325.28 | 1.489 | 0.205 |
| -0.558 | NA | NA | + | -8.08E-04 | NA | 1.82E-01 | 7 | -155.671 | 325.343 | 1.552 | 0.198 |
| -0.452 | NA | NA | + | NA | -2.14E-02 | 1.80E-01 | 7 | -155.85 | 325.7 | 1.909 | 0.166 |
| -0.41 | NA | NA | + | NA | NA | 1.54E-01 | 6 | -159.324 | 330.648 | 0 | 0.292 |
| -0.332 | NA | NA | NA | NA | NA | 1.95E-01 | 3 | -162.772 | 331.545 | 0.897 | 0.187 |
| -0.247 | NA | NA | NA | 1.37E-03 | NA | 1.69E-01 | 4 | -161.801 | 331.602 | 0.954 | 0.181 |
| -0.438 | 8.41E-10 | NA | + | NA | NA | 1.60E-01 | 7 | -159.173 | 332.345 | 1.697 | 0.125 |
| -0.404 | NA | NA | + | 7.50E-05 | NA | 1.54E-01 | 7 | -159.322 | 332.645 | 1.997 | 0.108 |
| -0.409 | NA | NA | + | NA | -3.67E-04 | 1.54E-01 | 7 | -159.324 | 332.648 | 2 | 0.107 |
| -0.491 | NA | NA | + | NA | NA | 1.79E-01 | 6 | -157.004 | 326.008 | 0 | 0.434 |
| -0.529 | 1.41E-09 | NA | + | NA | NA | 1.85E-01 | 7 | -156.743 | 327.486 | 1.478 | 0.207 |
| -0.406 | NA | NA | + | NA | -3.86E-02 | 1.80E-01 | 7 | -156.846 | 327.692 | 1.684 | 0.187 |
| -0.531 | NA | NA | + | -4.77E-04 | NA | 1.82E-01 | 7 | -156.93 | 327.86 | 1.852 | 0.172 |
| -0.486 | NA | NA | + | NA | NA | 1.75E-01 | 6 | -142.6 | 297.2 | 0 | 0.406 |
| -0.575 | NA | NA | + | -1.12E-03 | NA | 1.80E-01 | 7 | -142.115 | 298.231 | 1.031 | 0.242 |
| -0.515 | 1.07E-09 | NA | + | NA | NA | 1.79E-01 | 7 | -142.404 | 298.807 | 1.607 | 0.182 |
| -0.413 | NA | NA | + | NA | -3.39E-02 | 1.76E-01 | 7 | -142.465 | 298.93 | I.73 | 0.171 |
| -0.606 | NA | NA | + | NA | NA | 2.07E-01 | 6 | -167.757 | 347.514 | 0 | 0.412 |
| -0.65 | 1.50E-09 | NA | + | NA | NA | 2.15E-01 | 7 | -167.413 | 348.826 | 1.312 | 0.214 |
| -0.676 | NA | NA | + | -9.73E-04 | NA | 2.11E-01 | 7 | -167.444 | 348.888 | 1.374 | 0.207 |
| -0.537 | NA | NA | + | NA | -3.15E-02 | 2.08E-01 | 7 | -167.659 | 349.319 | 1.805 | 0.167 |
| -0.439 | NA | NA | + | NA | NA | 1.62E-01 | 6 | -150.086 | 312.172 | 0 | 0.456 |
| -0.467 | 8.84E-10 | NA | + | NA | NA | 1.68E-01 | 7 | -149.914 | 313.829 | 1.656 | 0.199 |
| -0.473 | NA | NA | + | -3.96E-04 | NA | 1.65E-01 | 7 | -150.035 | 314.071 | 1.898 | 0.177 |
| -0.437 | NA | NA | + | NA | -1.11E-03 | 1.62E-01 | 7 | -150.086 | 314.172 | 2 | 0.168 |
| -0.532 | NA | NA | + | NA | NA | 1.85E-01 | 6 | -149.195 | 310.39 | 0 | 0.409 |
| -0.411 | NA | NA | + | NA | -5.51E-02 | 1.87E-01 | 7 | -148.851 | 311.701 | 1.312 | 0.212 |
| -0.57 | 1.38E-09 | NA | + | NA | NA | 1.91E-01 | 7 | -148.903 | 311.806 | 1.416 | 0.201 |
| -0.583 | NA | NA | + | -6.80E-04 | NA | 1.88E-01 | 7 | -149.026 | 312.052 | 1.662 | 0.178 |
| -0.379 | NA | NA | + | NA | NA | 1.49E-01 | 6 | -144.073 | 300.146 | 0 | 0.322 |
| -0.319 | NA | NA | NA | NA | NA | 1.85E-01 | 3 | -147.76 | 301.52 | 1.374 | 0.162 |
| -0.238 | NA | NA | NA | 1.18E-03 | NA | 1.62E-01 | 4 | -146.906 | 301.811 | 1.665 | 0.14 |
| -0.404 | 9.74E-10 | NA | + | NA | NA | 1.52E-01 | 7 | -143.935 | 301.87 | 1.724 | 0.136 |
| -0.342 | NA | NA | + | NA | -1.72E-02 | 1.49E-01 | 7 | -144.043 | 302.086 | I.94 | 0.122 |
| -0.392 | NA | NA | + | -1.46E-04 | NA | 1.50E-01 | 7 | -144.066 | 302.131 | 1.985 | 0.119 |
| -0.373 | NA | NA | + | NA | NA | 1.45E-01 | 6 | -144.302 | 300.604 | 0 | 0.306 |
| -0.302 | NA | NA | NA | NA | NA | 1.77E-01 | 3 | -147.905 | 301.811 | 1.206 | 0.167 |
| -0.228 | NA | NA | NA | 1.25E-03 | NA | 1.57E-01 | 4 | -146.931 | 301.862 | 1.258 | 0.163 |
| -0.403 | 7.16E-10 | NA | + | NA | NA | 1.52E-01 | 7 | -144.101 | 302.202 | 1.598 | 0.138 |
| -0.392 | NA | NA | + | NA | 8.93E-03 | 1.45E-01 | 7 | -144.294 | 302.588 | 1.984 | 0.113 |
| -0.377 | NA | NA | + | -5.43E-05 | NA | 1.45E-01 | 7 | -144.301 | 302.602 | 1.998 | 0.113 |
| -0.535 | NA | NA | + | NA | NA | 1.92E-01 | 6 | -161.288 | 334.576 | 0 | 0.429 |
| -0.603 | NA | NA | + | -9.56E-04 | NA | 1.95E-01 | 7 | -160.988 | 335.976 | 01.IV | 0.213 |
| -0.572 | 1.27E-09 | NA | + | NA | NA | 1.98E-01 | 7 | -161.046 | 336.091 | 1.515 | 0.201 |
| -0.533 | NA | NA | + | NA | -8.99E-04 | 1.92E-01 | 7 | -161.288 | 336.576 | 2 | 0.158 |
| -0.505 | NA | NA | + | NA | NA | 1.77E-01 | 6 | -150.675 | 313.35 | 0 | 0.4 |
| -0.593 | NA | NA | + | -1.18E-03 | NA | 1.81E-01 | 7 | -150.162 | 314.325 | 0.975 | 0.246 |
| -0.538 | 1.14E-09 | NA | + | NA | NA | 1.83E-01 | 7 | -150.435 | 314.871 | 1.521 | 0.187 |
| -0.425 | NA | NA | + | NA | -3.55E-02 | 1.77E-01 | 7 | -150.543 | 315.086 | 1.737 | 0.168 |
| -0.451 | NA | NA | + | NA | NA | 1.63E-01 | 6 | -147.404 | 306.807 | 0 | 0.428 |
| -0.483 | 9.47E-10 | NA | + | NA | NA | 1.70E-01 | 7 | -147.162 | 308.325 | 1.518 | 0.2 |
| -0.509 | NA | NA | + | -7.17E-04 | NA | 1.67E-01 | 7 | -147.217 | 308.434 | 1.626 | 0.19 |
| -0.372 | NA | NA | + | NA | -3.62E-02 | 1.65E-01 | 7 | -147.258 | 308.516 | 1.708 | 0.182 |
| -0.452 | NA | NA | + | NA | NA | 1.68E-01 | 6 | -151.919 | 315.838 | 0 | 0.454 |
| -0.484 | 9.08E-10 | NA | + | NA | NA | 1.75E-01 | 7 | -151.706 | 317.413 | 1.575 | 0.207 |
| -0.47 | NA | NA | + | -2.31E-04 | NA | 1.70E-01 | 7 | -151.901 | 317.802 | 1.964 | 0.17 |
| -0.431 | NA | NA | + | NA | -9.30E-03 | 1.68E-01 | 7 | -151.91 | 317.821 | 1.983 | 0.169 |
| -0.514 | NA | NA | + | NA | NA | 1.81E-01 | 6 | -152.525 | 317.051 | 0 | 0.407 |
| -0.587 | NA | NA | + | -9.65E-04 | NA | 1.85E-01 | 7 | -152.173 | 318.346 | 1.296 | 0.213 |
| -0.549 | 1.31E-09 | NA | + | NA | NA | 1.86E-01 | 7 | -152.237 | 318.474 | 1.424 | 0.2 |
| -0.426 | NA | NA | + | NA | -4.01E-02 | 1.83E-01 | 7 | -152.34 | 318.681 | I.63 | 0.18 |
| -0.503 | NA | NA | + | NA | NA | 1.80E-01 | 6 | -146.49 | 304.981 | 0 | 0.411 |
| -0.577 | NA | NA | + | -9.45E-04 | NA | 1.85E-01 | 7 | -146.173 | 306.347 | 1.366 | 0.208 |
| -0.538 | 1.39E-09 | NA | + | NA | NA | 1.85E-01 | 7 | -146.234 | 306.468 | 1.487 | 0.196 |
| -0.408 | NA | NA | + | NA | -4.23E-02 | 1.81E-01 | 7 | -146.288 | 306.577 | 1.596 | 0.185 |
| -0.421 | NA | NA | + | NA | NA | 1.56E-01 | 6 | -163.8 | 339.6 | 0 | 0.313 |
| -0.244 | NA | NA | NA | 1.60E-03 | NA | 1.70E-01 | 4 | -166.388 | 340.775 | 1.175 | 0.174 |
| -0.35 | NA | NA | NA | NA | NA | 2.00E-01 | 3 | -167.58 | 341.159 | 1.559 | 0.143 |
| -0.452 | 8.82E-10 | NA | + | NA | NA | 1.63E-01 | 7 | -163.632 | 341.265 | 1.665 | 0.136 |
| -0.39 | NA | NA | + | 3.67E-04 | NA | 1.54E-01 | 7 | -163.762 | 341.523 | 1.923 | 0.119 |
| -0.415 | NA | NA | + | NA | -2.87E-03 | 1.56E-01 | 7 | -163.799 | 341.599 | 1.999 | 0.115 |
| -0.446 | NA | NA | + | NA | NA | 1.58E-01 | 6 | -143.464 | 298.929 | 0 | 0.402 |
| -0.319 | NA | NA | + | NA | -5.67E-02 | 1.60E-01 | 7 | -143.067 | 300.133 | 1.205 | 0.22 |
| -0.511 | NA | NA | + | -8.30E-04 | NA | 1.62E-01 | 7 | -143.189 | 300.378 | 1.449 | 0.195 |
| -0.477 | 1.08E-09 | NA | + | NA | NA | 1.64E-01 | 7 | -143.255 | 300.51 | 1.582 | 0.182 |
| -0.465 | NA | NA | + | NA | NA | 1.68E-01 | 6 | -151.048 | 314.096 | 0 | 0.432 |
| -0.532 | NA | NA | + | -8.44E-04 | NA | 1.72E-01 | 7 | -150.783 | 315.565 | I.47 | 0.207 |
| -0.496 | 1.15E-09 | NA | + | NA | NA | 1.73E-01 | 7 | -150.851 | 315.702 | 1.606 | 0.194 |
| -0.417 | NA | NA | + | NA | -2.18E-02 | 1.69E-01 | 7 | -150.998 | 315.997 | 1.901 | 0.167 |
| -0.445 | NA | NA | + | NA | NA | 1.62E-01 | 6 | -156.01 | 324.019 | 0 | 0.458 |
| -0.476 | 1.07E-09 | NA | + | NA | NA | 1.67E-01 | 7 | -155.827 | 325.655 | 1.635 | 0.202 |
| -0.425 | NA | NA | + | NA | -9.28E-03 | 1.62E-01 | 7 | -156.001 | 326.003 | 1.983 | 0.17 |
| -0.458 | NA | NA | + | -1.47E-04 | NA | 1.63E-01 | 7 | -156.003 | 326.006 | 1.987 | 0.17 |
| -0.495 | NA | NA | + | NA | NA | 1.76E-01 | 6 | -149.104 | 310.208 | 0 | 0.4 |
| -0.369 | NA | NA | + | NA | -5.66E-02 | 1.77E-01 | 7 | -148.725 | 311.45 | 1.241 | 0.215 |
| -0.537 | 1.27E-09 | NA | + | NA | NA | 1.84E-01 | 7 | -148.761 | 311.521 | 1.313 | 0.208 |
| -0.549 | NA | NA | + | -6.96E-04 | NA | 1.79E-01 | 7 | -148.923 | 311.846 | 1.638 | 0.177 |
| -0.451 | NA | NA | + | NA | NA | 1.62E-01 | 6 | -154.227 | 320.454 | 0 | 0.445 |
| -0.485 | 1.02E-09 | NA | + | NA | NA | 1.69E-01 | 7 | -153.998 | 321.996 | 1.542 | 0.206 |
| -0.485 | NA | NA | + | -4.34E-04 | NA | 1.63E-01 | 7 | -154.16 | 322.319 | 1.865 | 0.175 |
| -0.396 | NA | NA | + | NA | -2.49E-02 | 1.62E-01 | 7 | -154.163 | 322.326 | 1.872 | 0.174 |
| -0.513 | NA | NA | + | NA | NA | 1.76E-01 | 6 | -153.047 | 318.094 | 0 | 0.407 |
| -0.595 | NA | NA | + | -1.11E-03 | NA | 1.80E-01 | 7 | -152.594 | 319.188 | 1.094 | 0.235 |
| -0.548 | 1.13E-09 | NA | + | NA | NA | 1.82E-01 | 7 | -152.795 | 319.589 | 1.495 | 0.193 |
| -0.446 | NA | NA | + | NA | -3.05E-02 | 1.76E-01 | 7 | -152.95 | 319.901 | 1.807 | 0.165 |
| -0.451 | NA | NA | + | NA | NA | 1.65E-01 | 6 | -148.363 | 308.726 | 0 | 0.4 |
| -0.537 | NA | NA | + | -1.06E-03 | NA | 1.70E-01 | 7 | -147.947 | 309.895 | 1.169 | 0.223 |
| -0.488 | 1.21E-09 | NA | + | NA | NA | 1.71E-01 | 7 | -148.1 | 310.2 | 1.474 | 0.192 |
| -0.352 | NA | NA | + | NA | -4.52E-02 | 1.65E-01 | 7 | -148.134 | 310.267 | 1.541 | 0.185 |
| -0.573 | NA | NA | + | NA | NA | 1.94E-01 | 6 | -151.128 | 314.255 | 0 | 0.322 |
| -0.646 | NA | NA | + | -1.08E-03 | NA | 1.97E-01 | 7 | -150.638 | 315.277 | 1.022 | 0.193 |
| -0.43 | NA | NA | + | NA | -6.30E-02 | 1.94E-01 | 7 | -150.662 | 315.325 | 01.VII | 0.188 |
| -0.611 | 1.38E-09 | NA | + | NA | NA | 2.00E-01 | 7 | -150.844 | 315.687 | 1.432 | 0.157 |
| -0.487 | NA | NA | + | -1.32E-03 | -7.71E-02 | 1.98E-01 | 8 | -149.958 | 315.916 | 1.661 | 0.14 |
| -0.45 | NA | NA | + | NA | NA | 1.63E-01 | 6 | -148.321 | 308.641 | 0 | 0.417 |
| -0.486 | 1.29E-09 | NA | + | NA | NA | 1.69E-01 | 7 | -148.037 | 310.074 | 1.432 | 0.204 |
| -0.351 | NA | NA | + | NA | -4.61E-02 | 1.65E-01 | 7 | -148.088 | 310.177 | 1.535 | 0.194 |
| -0.507 | NA | NA | + | -7.19E-04 | NA | 1.66E-01 | 7 | -148.13 | 310.26 | 1.618 | 0.186 |
| -0.519 | NA | NA | + | NA | NA | 1.80E-01 | 6 | -162.997 | 337.995 | 0 | 0.427 |
| -0.56 | 1.57E-09 | NA | + | NA | NA | 1.86E-01 | 7 | -162.694 | 339.387 | 1.392 | 0.213 |
| -0.417 | NA | NA | + | NA | -4.62E-02 | 1.81E-01 | 7 | -162.782 | 339.564 | 1.569 | 0.195 |
| -0.549 | NA | NA | + | -3.93E-04 | NA | 1.82E-01 | 7 | -162.946 | 339.892 | 1.898 | 0.165 |
| -0.508 | NA | NA | + | NA | NA | 1.85E-01 | 6 | -154.927 | 321.854 | 0 | 0.412 |
| -0.543 | 1.23E-09 | NA | + | NA | NA | 1.91E-01 | 7 | -154.666 | 323.332 | 1.477 | 0.197 |
| -0.57 | NA | NA | + | -8.23E-04 | NA | 1.89E-01 | 7 | -154.674 | 323.349 | 1.494 | 0.195 |
| -0.405 | NA | NA | + | NA | -4.71E-02 | 1.87E-01 | 7 | -154.675 | 323.35 | 1.496 | 0.195 |
| -0.504 | NA | NA | + | NA | NA | 1.76E-01 | 6 | -153.767 | 319.533 | 0 | 0.434 |
| -0.542 | 1.33E-09 | NA | + | NA | NA | 1.82E-01 | 7 | -153.528 | 321.056 | 1.523 | 0.203 |
| -0.411 | NA | NA | + | NA | -4.14E-02 | 1.77E-01 | 7 | -153.588 | 321.175 | 1.642 | 0.191 |
| -0.542 | NA | NA | + | -4.59E-04 | NA | 1.79E-01 | 7 | -153.691 | 321.383 | I.85 | 0.172 |
| -0.396 | NA | NA | + | NA | NA | 1.53E-01 | 6 | -135.533 | 283.065 | 0 | 0.43 |
| -0.471 | NA | NA | + | -8.79E-04 | NA | 1.57E-01 | 7 | -135.239 | 284.477 | 1.412 | 0.212 |
| -0.422 | 8.56E-10 | NA | + | NA | NA | 1.57E-01 | 7 | -135.37 | 284.74 | 1.675 | 0.186 |
| -0.342 | NA | NA | + | NA | -2.54E-02 | 1.54E-01 | 7 | -135.457 | 284.914 | 1.849 | 0.171 |
| -0.475 | NA | NA | + | NA | NA | 1.72E-01 | 6 | -151.332 | 314.665 | 0 | 0.428 |
| -0.511 | 1.37E-09 | NA | + | NA | NA | 1.78E-01 | 7 | -151.066 | 316.132 | 1.467 | 0.206 |
| -0.531 | NA | NA | + | -7.05E-04 | NA | 1.76E-01 | 7 | -151.161 | 316.321 | 1.656 | 0.187 |
| -0.402 | NA | NA | + | NA | -3.41E-02 | 1.73E-01 | 7 | -151.207 | 316.413 | 1.748 | 0.179 |
| -0.609 | NA | NA | + | NA | NA | 2.01E-01 | 6 | -157.76 | 327.519 | 0 | 0.276 |
| -0.429 | NA | NA | + | NA | -7.70E-02 | 2.00E-01 | 7 | -157.054 | 328.108 | 0.589 | 0.206 |
| -0.653 | 1.37E-09 | NA | + | NA | NA | 2.09E-01 | 7 | -157.391 | 328.783 | 1.264 | 0.147 |
| -0.678 | NA | NA | + | -9.24E-04 | NA | 2.05E-01 | 7 | -157.441 | 328.883 | 1.363 | 0.14 |
| -0.491 | NA | NA | + | -1.21E-03 | -8.92E-02 | 2.05E-01 | 8 | -156.52 | 329.04 | 1.521 | 0.129 |
| -0.477 | 1.24E-09 | NA | + | NA | -7.35E-02 | 2.07E-01 | 8 | -156.749 | 329.498 | 1.979 | 0.103 |
| -0.833 | NA | NA | + | -1.72E-03 | NA | 2.37E-01 | 7 | -158.442 | 330.884 | 0 | 0.204 |
| -0.646 | NA | NA | + | -2.00E-03 | -9.00E-02 | 2.37E-01 | 8 | -157.493 | 330.986 | 0.103 | 0.194 |
| -0.722 | NA | NA | + | NA | NA | 2.33E-01 | 6 | -159.662 | 331.324 | 0.441 | 0.164 |
| -0.885 | 1.43E-09 | NA | + | -1.76E-03 | NA | 2.47E-01 | 8 | -157.919 | 331.837 | 0.954 | 0.127 |
| -0.701 | 1.31E-09 | NA | + | -2.02E-03 | -8.61E-02 | 2.46E-01 | 9 | -157.049 | 332.097 | 1.214 | 0.111 |
| -0.565 | NA | NA | + | NA | -6.89E-02 | 2.33E-01 | 7 | -159.099 | 332.198 | 1.314 | 0.106 |
| -0.768 | 1.35E-09 | NA | + | NA | NA | 2.43E-01 | 7 | -159.2 | 332.399 | 1.516 | 0.096 |
| -0.434 | NA | NA | + | NA | NA | 1.59E-01 | 6 | -160.323 | 332.646 | 0 | 0.304 |
| -0.26 | NA | NA | NA | 1.55E-03 | NA | 1.72E-01 | 4 | -162.847 | 333.694 | 1.048 | 0.18 |
| -0.349 | NA | NA | NA | NA | NA | 1.97E-01 | 3 | -164.002 | 334.003 | 1.358 | 0.154 |
| -0.465 | 1.04E-09 | NA | + | NA | NA | 1.65E-01 | 7 | -160.135 | 334.269 | 1.623 | 0.135 |
| -0.411 | NA | NA | + | 2.79E-04 | NA | 1.57E-01 | 7 | -160.301 | 334.601 | 1.956 | 0.114 |
| -0.44 | NA | NA | + | NA | 2.43E-03 | 1.59E-01 | 7 | -160.322 | 334.645 | 1.999 | 0.112 |
| -0.434 | NA | NA | + | NA | NA | 1.61E-01 | 6 | -157.65 | 327.3 | 0 | 0.39 |
| -0.463 | 1.03E-09 | NA | + | NA | NA | 1.66E-01 | 7 | -157.489 | 328.977 | 1.677 | 0.169 |
| -0.339 | NA | NA | NA | NA | NA | 1.95E-01 | 3 | -161.59 | 329.18 | I.88 | 0.152 |
| -0.45 | NA | NA | + | -1.87E-04 | NA | 1.62E-01 | 7 | -157.639 | 329.279 | 1.979 | 0.145 |
| -0.442 | NA | NA | + | NA | 3.34E-03 | 1.61E-01 | 7 | -157.649 | 329.298 | 1.998 | 0.144 |
| -0.352 | NA | NA | + | NA | NA | 1.39E-01 | 6 | -140.995 | 293.989 | 0 | 0.239 |
| -0.284 | NA | NA | NA | NA | NA | 1.71E-01 | 3 | -144.162 | 294.324 | 0.335 | 0.202 |
| -0.21 | NA | NA | NA | 1.25E-03 | NA | 1.50E-01 | 4 | -143.238 | 294.476 | 0.486 | 0.188 |
| -0.38 | 8.05E-10 | NA | + | NA | NA | 1.44E-01 | 7 | -140.822 | 295.643 | 1.654 | 0.105 |
| -0.314 | 8.53E-10 | NA | NA | NA | NA | 1.77E-01 | 4 | -143.976 | 295.951 | 1.962 | 0.09 |
| -0.37 | NA | NA | + | NA | 8.02E-03 | 1.38E-01 | 7 | -140.988 | 295.976 | 1.987 | 0.089 |
| -0.349 | NA | NA | + | 4.49E-05 | NA | 1.38E-01 | 7 | -140.994 | 295.988 | 1.999 | 0.088 |
| -0.442 | NA | NA | + | NA | NA | 1.70E-01 | 6 | -134.742 | 281.483 | 0 | 0.311 |
| -0.302 | NA | NA | + | NA | -6.48E-02 | 1.71E-01 | 7 | -134.162 | 282.324 | 0.841 | 0.204 |
| -0.523 | NA | NA | + | -1.04E-03 | NA | 1.76E-01 | 7 | -134.286 | 282.571 | 1.088 | 0.18 |
| -0.479 | 1.18E-09 | NA | + | NA | NA | 1.77E-01 | 7 | -134.42 | 282.841 | 1.357 | 0.158 |
| -0.375 | NA | NA | + | -1.28E-03 | -7.69E-02 | 1.78E-01 | 8 | -133.488 | 282.975 | 1.492 | 0.147 |
| -0.563 | NA | NA | + | NA | NA | 1.94E-01 | 6 | -158.254 | 328.508 | 0 | 0.409 |
| -0.604 | 1.43E-09 | NA | + | NA | NA | 2.01E-01 | 7 | -157.93 | 329.86 | 1.353 | 0.208 |
| -0.625 | NA | NA | + | -8.52E-04 | NA | 1.97E-01 | 7 | -157.993 | 329.987 | 1.479 | 0.195 |
| -0.462 | NA | NA | + | NA | -4.48E-02 | 1.94E-01 | 7 | -158.037 | 330.074 | 1.567 | 0.187 |
| -0.434 | NA | NA | + | NA | NA | 1.65E-01 | 6 | -158.764 | 329.529 | 0 | 0.386 |
| -0.467 | 1.17E-09 | NA | + | NA | NA | 1.70E-01 | 7 | -158.558 | 331.116 | 1.587 | 0.174 |
| -0.353 | NA | NA | NA | NA | NA | 2.02E-01 | 3 | -162.684 | 331.369 | I.84 | 0.154 |
| -0.449 | NA | NA | + | -1.76E-04 | NA | 1.66E-01 | 7 | -158.755 | 331.509 | I.98 | 0.143 |
| -0.414 | NA | NA | + | NA | -9.34E-03 | 1.65E-01 | 7 | -158.756 | 331.511 | 1.983 | 0.143 |
| -0.533 | NA | NA | + | NA | NA | 1.90E-01 | 6 | -167.263 | 346.525 | 0 | 0.451 |
| -0.57 | 1.32E-09 | NA | + | NA | NA | 1.96E-01 | 7 | -167.029 | 348.059 | 1.533 | 0.209 |
| -0.563 | NA | NA | + | -3.90E-04 | NA | 1.92E-01 | 7 | -167.216 | 348.432 | 1.907 | 0.174 |
| -0.544 | NA | NA | + | NA | 4.92E-03 | 1.90E-01 | 7 | -167.26 | 348.521 | 1.996 | 0.166 |
| -0.616 | NA | NA | + | NA | NA | 2.08E-01 | 6 | -162.546 | 337.092 | 0 | 0.327 |
| -0.457 | NA | NA | + | NA | -6.83E-02 | 2.07E-01 | 7 | -162.04 | 338.079 | 0.988 | 0.2 |
| -0.663 | 1.48E-09 | NA | + | NA | NA | 2.18E-01 | 7 | -162.122 | 338.244 | 1.152 | 0.184 |
| -0.681 | NA | NA | + | -9.23E-04 | NA | 2.12E-01 | 7 | -162.222 | 338.444 | 1.352 | 0.166 |
| -0.511 | NA | NA | + | -1.18E-03 | -8.12E-02 | 2.12E-01 | 8 | -161.528 | 339.056 | 1.964 | 0.123 |
| -0.423 | NA | NA | + | NA | NA | 1.56E-01 | 6 | -153.759 | 319.518 | 0 | 0.459 |
| -0.448 | 8.82E-10 | NA | + | NA | NA | 1.60E-01 | 7 | -153.629 | 321.258 | I.74 | 0.192 |
| -0.458 | NA | NA | + | -4.20E-04 | NA | 1.58E-01 | 7 | -153.705 | 321.41 | 1.892 | 0.178 |
| -0.441 | NA | NA | + | NA | 8.32E-03 | 1.55E-01 | 7 | -153.752 | 321.504 | 1.987 | 0.17 |
| -0.355 | NA | NA | + | NA | NA | 1.41E-01 | 6 | -142.602 | 297.204 | 0 | 0.277 |
| -0.198 | NA | NA | NA | 1.30E-03 | NA | 1.49E-01 | 4 | -144.88 | 297.761 | 0.557 | 0.21 |
| -0.285 | NA | NA | NA | NA | NA | 1.75E-01 | 3 | -145.965 | 297.929 | 0.725 | 0.193 |
| -0.379 | 8.12E-10 | NA | + | NA | NA | 1.45E-01 | 7 | -142.468 | 298.937 | 1.733 | 0.116 |
| -0.341 | NA | NA | + | NA | -6.62E-03 | 1.41E-01 | 7 | -142.597 | 299.195 | 1.991 | 0.102 |
| -0.348 | NA | NA | + | 8.44E-05 | NA | 1.40E-01 | 7 | -142.6 | 299.199 | 1.995 | 0.102 |
| -0.408 | NA | NA | + | NA | NA | 1.56E-01 | 6 | -149.377 | 310.755 | 0 | 0.392 |
| -0.434 | 1.02E-09 | NA | + | NA | NA | 1.60E-01 | 7 | -149.242 | 312.485 | I.73 | 0.165 |
| -0.35 | NA | NA | NA | NA | NA | 1.94E-01 | 3 | -153.327 | 312.655 | 01.IX | 0.152 |
| -0.427 | NA | NA | + | -2.27E-04 | NA | 1.58E-01 | 7 | -149.36 | 312.72 | 1.965 | 0.147 |
| -0.417 | NA | NA | + | NA | 4.30E-03 | 1.56E-01 | 7 | -149.376 | 312.751 | 1.996 | 0.144 |
| -0.527 | NA | NA | + | NA | NA | 1.81E-01 | 6 | -155.567 | 323.133 | 0 | 0.449 |
| -0.563 | 1.33E-09 | NA | + | NA | NA | 1.87E-01 | 7 | -155.336 | 324.673 | I.54 | 0.208 |
| -0.555 | NA | NA | + | -3.77E-04 | NA | 1.83E-01 | 7 | -155.519 | 325.038 | 1.904 | 0.173 |
| -0.49 | NA | NA | + | NA | -1.66E-02 | 1.82E-01 | 7 | -155.539 | 325.079 | 1.945 | 0.17 |
| -0.541 | NA | NA | + | NA | NA | 1.86E-01 | 6 | -160.658 | 333.317 | 0 | 0.437 |
| -0.578 | 1.10E-09 | NA | + | NA | NA | 1.94E-01 | 7 | -160.417 | 334.834 | 1.517 | 0.205 |
| -0.59 | NA | NA | + | -6.32E-04 | NA | 1.89E-01 | 7 | -160.526 | 335.052 | 1.735 | 0.184 |
| -0.477 | NA | NA | + | NA | -2.86E-02 | 1.86E-01 | 7 | -160.578 | 335.157 | I.84 | 0.174 |
| -0.438 | NA | NA | + | NA | NA | 1.64E-01 | 6 | -146.116 | 304.232 | 0 | 0.442 |
| -0.499 | NA | NA | + | -7.07E-04 | NA | 1.69E-01 | 7 | -145.936 | 305.872 | I.64 | 0.195 |
| -0.465 | 9.33E-10 | NA | + | NA | NA | 1.69E-01 | 7 | -145.956 | 305.911 | 1.679 | 0.191 |
| -0.385 | NA | NA | + | NA | -2.40E-02 | 1.65E-01 | 7 | -146.055 | 306.111 | 1.879 | 0.173 |
| -0.224 | NA | NA | NA | 1.64E-03 | NA | 1.60E-01 | 4 | -164.919 | 337.838 | 0 | 0.305 |
| -0.405 | NA | NA | + | NA | NA | 1.52E-01 | 6 | -163.218 | 338.436 | 0.598 | 0.226 |
| -0.33 | NA | NA | NA | NA | NA | 1.90E-01 | 3 | -166.246 | 338.492 | 0.654 | 0.22 |
| -0.25 | 9.50E-10 | NA | NA | 1.64E-03 | NA | 1.64E-01 | 5 | -164.808 | 339.617 | 1.779 | 0.125 |
| -0.288 | NA | NA | NA | 1.79E-03 | 3.60E-02 | 1.58E-01 | 5 | -164.813 | 339.627 | 1.789 | 0.125 |
| -0.537 | NA | NA | + | NA | NA | 1.84E-01 | 6 | -150.715 | 313.429 | 0 | 0.422 |
| -0.578 | 1.51E-09 | NA | + | NA | NA | 1.90E-01 | 7 | -150.418 | 314.837 | 1.408 | 0.209 |
| -0.438 | NA | NA | + | NA | -4.41E-02 | 1.85E-01 | 7 | -150.505 | 315.011 | 1.582 | 0.191 |
| -0.585 | NA | NA | + | -6.22E-04 | NA | 1.87E-01 | 7 | -150.574 | 315.147 | 1.718 | 0.179 |
| -0.37 | NA | NA | + | NA | NA | 1.44E-01 | 6 | -148.751 | 309.503 | 0 | 0.388 |
| -0.396 | 8.76E-10 | NA | + | NA | NA | 1.49E-01 | 7 | -148.602 | 311.203 | 01.VII | 0.166 |
| -0.404 | NA | NA | + | -4.21E-04 | NA | 1.46E-01 | 7 | -148.691 | 311.382 | 1.879 | 0.152 |
| -0.326 | NA | NA | NA | NA | NA | 1.88E-01 | 3 | -152.698 | 311.395 | 1.892 | 0.151 |
| -0.366 | NA | NA | + | NA | -1.96E-03 | 1.44E-01 | 7 | -148.751 | 311.502 | 1.999 | 0.143 |
| -0.39 | NA | NA | + | NA | NA | 1.51E-01 | 6 | -140.846 | 293.693 | 0 | 0.385 |
| -0.416 | 9.46E-10 | NA | + | NA | NA | 1.55E-01 | 7 | -140.7 | 295.4 | 1.707 | 0.164 |
| -0.348 | NA | NA | NA | NA | NA | 1.91E-01 | 3 | -144.731 | 295.463 | I.77 | 0.159 |
| -0.423 | NA | NA | + | -4.18E-04 | NA | 1.53E-01 | 7 | -140.783 | 295.566 | 1.874 | 0.151 |
| -0.381 | NA | NA | + | NA | -4.08E-03 | 1.51E-01 | 7 | -140.845 | 295.689 | 1.996 | 0.142 |
| -0.483 | NA | NA | + | NA | NA | 1.73E-01 | 6 | -143.068 | 298.135 | 0 | 0.328 |
| -0.57 | NA | NA | + | -1.14E-03 | NA | 1.79E-01 | 7 | -142.537 | 299.073 | 0.938 | 0.205 |
| -0.358 | NA | NA | + | NA | -5.63E-02 | 1.73E-01 | 7 | -142.681 | 299.362 | 1.227 | 0.178 |
| -0.518 | 1.29E-09 | NA | + | NA | NA | 1.78E-01 | 7 | -142.819 | 299.637 | 1.502 | 0.155 |
| -0.432 | NA | NA | + | -1.35E-03 | -6.96E-02 | 1.80E-01 | 8 | -141.957 | 299.914 | 1.779 | 0.135 |
| -0.43 | NA | NA | + | NA | NA | 1.59E-01 | 6 | -164.931 | 341.862 | 0 | 0.334 |
| -0.462 | 9.95E-10 | NA | + | NA | NA | 1.65E-01 | 7 | -164.747 | 343.494 | 1.632 | 0.148 |
| -0.274 | NA | NA | NA | 1.44E-03 | NA | 1.73E-01 | 4 | -167.783 | 343.567 | 1.705 | 0.143 |
| -0.369 | NA | NA | NA | NA | NA | 2.00E-01 | 3 | -168.891 | 343.782 | I.92 | 0.128 |
| -0.413 | NA | NA | + | NA | -7.65E-03 | 1.59E-01 | 7 | -164.926 | 343.851 | 1.989 | 0.124 |
| -0.422 | NA | NA | + | 9.44E-05 | NA | 1.58E-01 | 7 | -164.928 | 343.856 | 1.995 | 0.123 |
| -0.601 | NA | NA | + | NA | NA | 2.06E-01 | 6 | -156.932 | 325.863 | 0 | 0.2 |
| -0.525 | NA | NA | + | -1.79E-03 | -9.46E-02 | 2.16E-01 | 8 | -155.037 | 326.074 | 0.21 | 0.18 |
| -0.71 | NA | NA | + | -1.51E-03 | NA | 2.13E-01 | 7 | -156.04 | 326.081 | 0.218 | 0.179 |
| -0.435 | NA | NA | + | NA | -7.61E-02 | 2.08E-01 | 7 | -156.271 | 326.542 | 0.679 | 0.142 |
| -0.645 | 1.54E-09 | NA | + | NA | NA | 2.14E-01 | 7 | -156.54 | 327.081 | 1.217 | 0.109 |
| -0.755 | 1.56E-09 | NA | + | -1.51E-03 | NA | 2.20E-01 | 8 | -155.635 | 327.269 | 1.406 | 0.099 |
| -0.573 | 1.38E-09 | NA | + | -1.78E-03 | -9.04E-02 | 2.22E-01 | 9 | -154.72 | 327.44 | 1.576 | 0.091 |
| -0.515 | NA | NA | + | NA | NA | 1.85E-01 | 6 | -163.927 | 339.854 | 0 | 0.449 |
| -0.552 | 1.16E-09 | NA | + | NA | NA | 1.92E-01 | 7 | -163.683 | 341.366 | 1.512 | 0.211 |
| -0.542 | NA | NA | + | -3.38E-04 | NA | 1.87E-01 | 7 | -163.892 | 341.783 | I.93 | 0.171 |
| -0.485 | NA | NA | + | NA | -1.40E-02 | 1.86E-01 | 7 | -163.908 | 341.816 | 1.963 | 0.168 |
| -0.472 | NA | NA | + | NA | NA | 1.74E-01 | 6 | -152.083 | 316.166 | 0 | 0.438 |
| -0.504 | 9.51E-10 | NA | + | NA | NA | 1.81E-01 | 7 | -151.855 | 317.711 | 1.545 | 0.202 |
| -0.522 | NA | NA | + | -6.47E-04 | NA | 1.77E-01 | 7 | -151.933 | 317.865 | 1.699 | 0.187 |
| -0.417 | NA | NA | + | NA | -2.48E-02 | 1.74E-01 | 7 | -152.02 | 318.04 | 1.874 | 0.172 |
| -0.729 | NA | NA | + | NA | NA | 2.37E-01 | 6 | -167.115 | 346.231 | 0 | 0.198 |
| -0.547 | NA | NA | + | NA | -7.91E-02 | 2.35E-01 | 7 | -166.352 | 346.703 | 0.472 | 0.157 |
| -0.611 | NA | NA | + | -1.56E-03 | -9.27E-02 | 2.38E-01 | 8 | -165.425 | 346.85 | 0.619 | 0.146 |
| -0.809 | NA | NA | + | -1.30E-03 | NA | 2.40E-01 | 7 | -166.458 | 346.916 | 0.686 | 0.141 |
| -0.786 | 2.12E-09 | NA | + | NA | NA | 2.45E-01 | 7 | -166.636 | 347.272 | 1.041 | 0.118 |
| -0.866 | 2.12E-09 | NA | + | -1.31E-03 | NA | 2.48E-01 | 8 | -165.972 | 347.943 | 1.713 | 0.084 |
| -0.611 | 1.81E-09 | NA | + | NA | -7.25E-02 | 2.42E-01 | 8 | -166.001 | 348.003 | 1.772 | 0.082 |
| -0.673 | 1.76E-09 | NA | + | -1.54E-03 | -8.61E-02 | 2.45E-01 | 9 | -165.09 | 348.179 | 1.949 | 0.075 |
| -0.119 | NA | NA | NA | 1.55E-03 | NA | 1.20E-01 | 4 | -148.347 | 304.694 | 0 | 0.292 |
| -0.223 | NA | NA | NA | NA | NA | 1.53E-01 | 3 | -149.764 | 305.527 | 0.834 | 0.192 |
| -0.291 | NA | NA | + | NA | NA | 1.16E-01 | 6 | -147.02 | 306.039 | 1.346 | 0.149 |
| 0.207 | NA | NA | NA | 2.07E-03 | NA | NA | 3 | -150.085 | 306.171 | 1.477 | 0.139 |
| -0.142 | 8.08E-10 | NA | NA | 1.54E-03 | NA | 1.24E-01 | 5 | -148.259 | 306.518 | 1.825 | 0.117 |
| -0.148 | NA | NA | NA | 1.62E-03 | 1.61E-02 | 1.19E-01 | 5 | -148.322 | 306.644 | 1.951 | 0.11 |
| -0.488 | NA | NA | + | NA | NA | 1.72E-01 | 6 | -154.195 | 320.389 | 0 | 0.425 |
| -0.528 | 1.40E-09 | NA | + | NA | NA | 1.79E-01 | 7 | -153.908 | 321.817 | 1.427 | 0.208 |
| -0.4 | NA | NA | + | NA | -3.93E-02 | 1.72E-01 | 7 | -154.026 | 322.052 | 1.663 | 0.185 |
| -0.538 | NA | NA | + | -6.45E-04 | NA | 1.75E-01 | 7 | -154.05 | 322.099 | I.71 | 0.181 |
| -0.471 | NA | NA | + | NA | NA | 1.70E-01 | 6 | -143.54 | 299.08 | 0 | 0.407 |
| -0.543 | NA | NA | + | -9.17E-04 | NA | 1.74E-01 | 7 | -143.223 | 300.445 | 1.366 | 0.206 |
| -0.505 | 1.18E-09 | NA | + | NA | NA | 1.76E-01 | 7 | -143.266 | 300.532 | 1.453 | 0.197 |
| -0.373 | NA | NA | + | NA | -4.48E-02 | 1.72E-01 | 7 | -143.301 | 300.603 | 1.523 | 0.19 |
| -0.415 | NA | NA | + | NA | NA | 1.54E-01 | 6 | -145.05 | 302.099 | 0 | 0.461 |
| -0.438 | 8.65E-10 | NA | + | NA | NA | 1.58E-01 | 7 | -144.925 | 303.85 | 1.751 | 0.192 |
| -0.443 | NA | NA | + | -3.26E-04 | NA | 1.56E-01 | 7 | -145.014 | 304.027 | 1.928 | 0.176 |
| -0.39 | NA | NA | + | NA | -1.15E-02 | 1.55E-01 | 7 | -145.036 | 304.072 | 1.973 | 0.172 |
| -0.438 | NA | NA | + | NA | NA | 1.58E-01 | 6 | -159.929 | 331.858 | 0 | 0.456 |
| -0.472 | 1.16E-09 | NA | + | NA | NA | 1.64E-01 | 7 | -159.718 | 333.436 | 1.578 | 0.207 |
| -0.417 | NA | NA | + | NA | -9.81E-03 | 1.58E-01 | 7 | -159.92 | 333.84 | 1.982 | 0.169 |
| -0.446 | NA | NA | + | -9.29E-05 | NA | 1.59E-01 | 7 | -159.927 | 333.853 | 1.995 | 0.168 |
| -0.441 | NA | NA | + | NA | NA | 1.59E-01 | 6 | -148.186 | 308.373 | 0 | 0.416 |
| -0.531 | NA | NA | + | -1.11E-03 | NA | 1.63E-01 | 7 | -147.75 | 309.501 | 1.128 | 0.237 |
| -0.472 | 1.06E-09 | NA | + | NA | NA | 1.64E-01 | 7 | -147.982 | 309.964 | 1.591 | 0.188 |
| -0.399 | NA | NA | + | NA | -2.00E-02 | 1.60E-01 | 7 | -148.145 | 310.29 | 1.918 | 0.159 |
| -0.631 | NA | NA | + | NA | NA | 2.10E-01 | 6 | -164.484 | 340.968 | 0 | 0.38 |
| -0.708 | NA | NA | + | -1.10E-03 | NA | 2.15E-01 | 7 | -164.043 | 342.087 | 1.119 | 0.217 |
| -0.676 | 1.61E-09 | NA | + | NA | NA | 2.18E-01 | 7 | -164.109 | 342.217 | 1.249 | 0.203 |
| -0.502 | NA | NA | + | NA | -5.79E-02 | 2.11E-01 | 7 | -164.128 | 342.255 | 1.287 | 0.2 |
| -0.461 | NA | NA | + | NA | NA | 1.68E-01 | 6 | -151.327 | 314.653 | 0 | 0.432 |
| -0.494 | 1.11E-09 | NA | + | NA | NA | 1.73E-01 | 7 | -151.113 | 316.225 | 1.572 | 0.197 |
| -0.523 | NA | NA | + | -7.53E-04 | NA | 1.72E-01 | 7 | -151.126 | 316.251 | 1.598 | 0.194 |
| -0.389 | NA | NA | + | NA | -3.22E-02 | 1.68E-01 | 7 | -151.218 | 316.436 | 1.782 | 0.177 |
| -0.484 | NA | NA | + | NA | NA | 1.79E-01 | 6 | -159.471 | 330.942 | 0 | 0.444 |
| -0.52 | 1.20E-09 | NA | + | NA | NA | 1.86E-01 | 7 | -159.224 | 332.448 | 1.506 | 0.209 |
| -0.429 | NA | NA | + | NA | -2.57E-02 | 1.80E-01 | 7 | -159.406 | 332.812 | I.87 | 0.174 |
| -0.518 | NA | NA | + | -4.11E-04 | NA | 1.82E-01 | 7 | -159.416 | 332.831 | 1.889 | 0.173 |
| -0.392 | NA | NA | + | NA | NA | 1.48E-01 | 6 | -136.276 | 284.552 | 0 | 0.419 |
| -0.472 | NA | NA | + | -9.46E-04 | NA | 1.54E-01 | 7 | -135.945 | 285.89 | 1.338 | 0.214 |
| -0.422 | 1.05E-09 | NA | + | NA | NA | 1.53E-01 | 7 | -136.056 | 286.113 | 1.561 | 0.192 |
| -0.319 | NA | NA | + | NA | -3.29E-02 | 1.49E-01 | 7 | -136.145 | 286.29 | 1.739 | 0.175 |
| -0.597 | NA | NA | + | NA | NA | 2.08E-01 | 6 | -160.811 | 333.623 | 0 | 0.386 |
| -0.674 | NA | NA | + | -1.12E-03 | NA | 2.12E-01 | 7 | -160.339 | 334.678 | 1.055 | 0.227 |
| -0.642 | 1.75E-09 | NA | + | NA | NA | 2.14E-01 | 7 | -160.464 | 334.929 | 1.306 | 0.201 |
| -0.489 | NA | NA | + | NA | -5.02E-02 | 2.09E-01 | 7 | -160.539 | 335.077 | 1.455 | 0.186 |
| -0.529 | NA | NA | + | NA | NA | 1.89E-01 | 6 | -150.905 | 313.81 | 0 | 0.33 |
| -0.614 | NA | NA | + | -1.11E-03 | NA | 1.95E-01 | 7 | -150.432 | 314.865 | 1.055 | 0.195 |
| -0.398 | NA | NA | + | NA | -5.88E-02 | 1.89E-01 | 7 | -150.51 | 315.02 | I.21 | 0.18 |
| -0.568 | 1.27E-09 | NA | + | NA | NA | 1.96E-01 | 7 | -150.596 | 315.192 | 1.382 | 0.166 |
| -0.47 | NA | NA | + | -1.33E-03 | -7.24E-02 | 1.96E-01 | 8 | -149.847 | 315.695 | 1.885 | 0.129 |
| -0.507 | NA | NA | + | NA | NA | 1.77E-01 | 6 | -150.799 | 313.598 | 0 | 0.401 |
| -0.588 | NA | NA | + | -1.11E-03 | NA | 1.82E-01 | 7 | -150.353 | 314.705 | 1.107 | 0.23 |
| -0.541 | 1.12E-09 | NA | + | NA | NA | 1.83E-01 | 7 | -150.559 | 315.117 | 1.519 | 0.187 |
| -0.413 | NA | NA | + | NA | -4.39E-02 | 1.79E-01 | 7 | -150.591 | 315.182 | 1.584 | 0.181 |
| -0.529 | NA | NA | + | NA | NA | 1.85E-01 | 6 | -155.861 | 323.721 | 0 | 0.42 |
| -0.566 | 1.31E-09 | NA | + | NA | NA | 1.91E-01 | 7 | -155.589 | 325.177 | 1.456 | 0.203 |
| -0.421 | NA | NA | + | NA | -4.90E-02 | 1.86E-01 | 7 | -155.613 | 325.225 | 1.504 | 0.198 |
| -0.571 | NA | NA | + | -6.08E-04 | NA | 1.87E-01 | 7 | -155.718 | 325.437 | 1.715 | 0.178 |
| -0.554 | NA | NA | + | NA | NA | 1.95E-01 | 6 | -149.686 | 311.371 | 0 | 0.333 |
| -0.415 | NA | NA | + | NA | -6.30E-02 | 1.96E-01 | 7 | -149.199 | 312.397 | 1.026 | 0.199 |
| -0.621 | NA | NA | + | -9.24E-04 | NA | 1.99E-01 | 7 | -149.324 | 312.649 | 1.278 | 0.176 |
| -0.59 | 1.15E-09 | NA | + | NA | NA | 2.02E-01 | 7 | -149.39 | 312.78 | 1.409 | 0.165 |
| -0.472 | NA | NA | + | -1.15E-03 | -7.51E-02 | 2.00E-01 | 8 | -148.649 | 313.299 | 1.928 | 0.127 |
| -0.435 | NA | NA | + | NA | NA | 1.59E-01 | 6 | -151.828 | 315.655 | 0 | 0.444 |
| -0.469 | 1.22E-09 | NA | + | NA | NA | 1.64E-01 | 7 | -151.613 | 317.226 | 1.571 | 0.202 |
| -0.478 | NA | NA | + | -5.69E-04 | NA | 1.62E-01 | 7 | -151.72 | 317.44 | 1.785 | 0.182 |
| -0.386 | NA | NA | + | NA | -2.27E-02 | 1.61E-01 | 7 | -151.774 | 317.549 | 1.893 | 0.172 |
| -0.538 | NA | NA | + | NA | NA | 1.84E-01 | 6 | -158.843 | 329.687 | 0 | 0.404 |
| -0.607 | NA | NA | + | -9.13E-04 | NA | 1.88E-01 | 7 | -158.535 | 331.069 | 1.382 | 0.202 |
| -0.423 | NA | NA | + | NA | -5.18E-02 | 1.85E-01 | 7 | -158.554 | 331.108 | 1.422 | 0.198 |
| -0.578 | 1.54E-09 | NA | + | NA | NA | 1.90E-01 | 7 | -158.571 | 331.141 | 1.455 | 0.195 |
| -0.679 | NA | NA | + | -1.63E-03 | NA | 2.03E-01 | 7 | -146.456 | 306.912 | 0 | 0.246 |
| -0.555 | NA | NA | + | NA | NA | 1.96E-01 | 6 | -147.576 | 307.152 | 0.24 | 0.219 |
| -0.523 | NA | NA | + | -1.88E-03 | -7.97E-02 | 2.05E-01 | 8 | -145.726 | 307.452 | 0.54 | 0.188 |
| -0.716 | 1.33E-09 | NA | + | -1.62E-03 | NA | 2.09E-01 | 8 | -146.171 | 308.342 | I.43 | 0.121 |
| -0.427 | NA | NA | + | NA | -5.81E-02 | 1.96E-01 | 7 | -147.18 | 308.359 | 1.447 | 0.12 |
| -0.592 | 1.34E-09 | NA | + | NA | NA | 2.02E-01 | 7 | -147.293 | 308.586 | 1.674 | 0.107 |
| -0.542 | NA | NA | + | NA | NA | 1.85E-01 | 6 | -167.858 | 347.716 | 0 | 0.437 |
| -0.578 | 1.33E-09 | NA | + | NA | NA | 1.91E-01 | 7 | -167.63 | 349.26 | 1.544 | 0.202 |
| -0.603 | NA | NA | + | -8.15E-04 | NA | 1.88E-01 | 7 | -167.646 | 349.292 | 1.576 | 0.199 |
| -0.528 | NA | NA | + | NA | -6.54E-03 | 1.85E-01 | 7 | -167.854 | 349.708 | 1.992 | 0.162 |
| -0.472 | NA | NA | + | NA | NA | 1.73E-01 | 6 | -145.338 | 302.676 | 0 | 0.417 |
| -0.541 | NA | NA | + | -8.33E-04 | NA | 1.79E-01 | 7 | -145.077 | 304.155 | 1.479 | 0.199 |
| -0.368 | NA | NA | + | NA | -4.58E-02 | 1.74E-01 | 7 | -145.098 | 304.196 | 1.519 | 0.195 |
| -0.503 | 9.38E-10 | NA | + | NA | NA | 1.80E-01 | 7 | -145.125 | 304.251 | 1.574 | 0.19 |
| -0.575 | NA | NA | + | NA | NA | 1.96E-01 | 6 | -153.564 | 319.127 | 0 | 0.293 |
| -0.665 | NA | NA | + | -1.22E-03 | NA | 2.02E-01 | 7 | -152.967 | 319.935 | 0.808 | 0.196 |
| -0.419 | NA | NA | + | NA | -6.98E-02 | 1.99E-01 | 7 | -153.006 | 320.011 | 0.884 | 0.189 |
| -0.492 | NA | NA | + | -1.51E-03 | -8.72E-02 | 2.05E-01 | 8 | -152.123 | 320.245 | 1.118 | 0.168 |
| -0.616 | 1.35E-09 | NA | + | NA | NA | 2.04E-01 | 7 | -153.204 | 320.407 | I.28 | 0.155 |
| -0.408 | NA | NA | + | NA | NA | 1.55E-01 | 6 | -142.335 | 296.669 | 0 | 0.449 |
| -0.433 | 8.82E-10 | NA | + | NA | NA | 1.59E-01 | 7 | -142.185 | 298.371 | 1.702 | 0.192 |
| -0.458 | NA | NA | + | -6.32E-04 | NA | 1.58E-01 | 7 | -142.188 | 298.375 | 1.706 | 0.191 |
| -0.379 | NA | NA | + | NA | -1.39E-02 | 1.56E-01 | 7 | -142.314 | 298.627 | 1.958 | 0.169 |
| -0.494 | NA | NA | + | NA | NA | 1.76E-01 | 6 | -165.395 | 342.791 | 0 | 0.457 |
| -0.527 | 1.26E-09 | NA | + | NA | NA | 1.81E-01 | 7 | -165.197 | 344.394 | 1.603 | 0.205 |
| -0.47 | NA | NA | + | NA | -1.09E-02 | 1.76E-01 | 7 | -165.384 | 344.768 | 1.977 | 0.17 |
| -0.497 | NA | NA | + | -3.46E-05 | NA | 1.76E-01 | 7 | -165.395 | 344.79 | 1.999 | 0.168 |
| -0.548 | NA | NA | + | -1.83E-03 | -1.18E-01 | 2.36E-01 | 8 | -157.225 | 330.45 | 0 | 0.329 |
| -0.481 | NA | NA | + | NA | -1.00E-01 | 2.33E-01 | 7 | -158.682 | 331.364 | 0.914 | 0.208 |
| -0.607 | 1.54E-09 | NA | + | -1.82E-03 | -1.13E-01 | 2.44E-01 | 9 | -156.786 | 331.571 | 1.121 | 0.188 |
| -0.714 | NA | NA | + | NA | NA | 2.37E-01 | 6 | -160.07 | 332.14 | 1.689 | 0.141 |
| -0.802 | NA | NA | + | -1.47E-03 | NA | 2.40E-01 | 7 | -159.13 | 332.261 | I.81 | 0.133 |
| -0.479 | NA | NA | + | NA | NA | 1.72E-01 | 6 | -156.944 | 325.887 | 0 | 0.445 |
| -0.514 | 1.33E-09 | NA | + | NA | NA | 1.78E-01 | 7 | -156.704 | 327.408 | I.52 | 0.208 |
| -0.416 | NA | NA | + | NA | -2.85E-02 | 1.73E-01 | 7 | -156.861 | 327.722 | 1.835 | 0.178 |
| -0.503 | NA | NA | + | -3.13E-04 | NA | 1.74E-01 | 7 | -156.91 | 327.821 | 1.933 | 0.169 |
| -0.141 | NA | NA | NA | 1.77E-03 | NA | 1.34E-01 | 4 | -162.148 | 332.295 | 0 | 0.412 |
| -0.258 | NA | NA | NA | NA | NA | 1.68E-01 | 3 | -163.721 | 333.443 | 1.148 | 0.232 |
| -0.232 | NA | NA | NA | 2.01E-03 | 5.43E-02 | 1.30E-01 | 5 | -161.904 | 333.808 | 1.513 | 0.194 |
| -0.159 | 6.77E-10 | NA | NA | 1.76E-03 | NA | 1.37E-01 | 5 | -162.084 | 334.167 | 1.872 | 0.162 |
| -0.498 | NA | NA | + | NA | NA | 1.74E-01 | 6 | -153.069 | 318.138 | 0 | 0.424 |
| -0.566 | NA | NA | + | -8.43E-04 | NA | 1.79E-01 | 7 | -152.831 | 319.662 | 1.524 | 0.198 |
| -0.531 | 1.06E-09 | NA | + | NA | NA | 1.81E-01 | 7 | -152.851 | 319.702 | 1.564 | 0.194 |
| -0.409 | NA | NA | + | NA | -3.90E-02 | 1.74E-01 | 7 | -152.909 | 319.817 | 1.679 | 0.183 |
| -0.5 | NA | NA | + | NA | NA | 1.80E-01 | 6 | -146.957 | 305.914 | 0 | 0.41 |
| -0.576 | NA | NA | + | -1.01E-03 | NA | 1.84E-01 | 7 | -146.564 | 307.128 | 1.214 | 0.223 |
| -0.536 | 1.26E-09 | NA | + | NA | NA | 1.86E-01 | 7 | -146.701 | 307.402 | 1.489 | 0.195 |
| -0.426 | NA | NA | + | NA | -3.45E-02 | 1.81E-01 | 7 | -146.828 | 307.655 | 1.742 | 0.172 |
| -0.492 | NA | NA | + | NA | NA | 1.81E-01 | 6 | -158.341 | 328.683 | 0 | 0.446 |
| -0.528 | 1.08E-09 | NA | + | NA | NA | 1.88E-01 | 7 | -158.075 | 330.15 | 1.467 | 0.214 |
| -0.441 | NA | NA | + | NA | -2.27E-02 | 1.81E-01 | 7 | -158.29 | 330.579 | 1.896 | 0.173 |
| -0.511 | NA | NA | + | -2.43E-04 | NA | 1.82E-01 | 7 | -158.322 | 330.644 | 1.961 | 0.167 |
| -0.542 | NA | NA | + | NA | NA | 1.97E-01 | 6 | -158.548 | 329.095 | 0 | 0.411 |
| -0.42 | NA | NA | + | NA | -5.46E-02 | 1.96E-01 | 7 | -158.221 | 330.443 | 1.348 | 0.209 |
| -0.582 | 1.16E-09 | NA | + | NA | NA | 2.06E-01 | 7 | -158.239 | 330.478 | 1.383 | 0.206 |
| -0.587 | NA | NA | + | -6.09E-04 | NA | 2.00E-01 | 7 | -158.405 | 330.81 | 1.715 | 0.174 |
| -0.539 | NA | NA | + | -2.12E-03 | -1.01E-01 | 2.20E-01 | 8 | -145.693 | 307.386 | 0 | 0.271 |
| -0.739 | NA | NA | + | -1.80E-03 | NA | 2.18E-01 | 7 | -146.954 | 307.908 | 0.522 | 0.209 |
| -0.612 | NA | NA | + | NA | NA | 2.11E-01 | 6 | -148.336 | 308.671 | 1.286 | 0.143 |
| -0.587 | 1.31E-09 | NA | + | -2.14E-03 | -9.70E-02 | 2.28E-01 | 9 | -145.338 | 308.675 | 1.289 | 0.142 |
| -0.785 | 1.48E-09 | NA | + | -1.84E-03 | NA | 2.26E-01 | 8 | -146.504 | 309.009 | 1.623 | 0.121 |
| -0.438 | NA | NA | + | NA | -7.85E-02 | 2.12E-01 | 7 | -147.563 | 309.127 | 1.741 | 0.114 |
| -0.45 | NA | NA | + | NA | NA | 1.64E-01 | 6 | -141.697 | 295.395 | 0 | 0.394 |
| -0.547 | NA | NA | + | -1.21E-03 | NA | 1.70E-01 | 7 | -141.136 | 296.273 | 0.878 | 0.254 |
| -0.482 | 8.91E-10 | NA | + | NA | NA | 1.71E-01 | 7 | -141.439 | 296.878 | 1.483 | 0.188 |
| -0.38 | NA | NA | + | NA | -3.23E-02 | 1.66E-01 | 7 | -141.574 | 297.148 | 1.753 | 0.164 |
| -0.446 | NA | NA | + | NA | NA | 1.62E-01 | 6 | -138.516 | 289.032 | 0 | 0.311 |
| -0.551 | NA | NA | + | -1.30E-03 | NA | 1.69E-01 | 7 | -137.84 | 289.679 | 0.647 | 0.225 |
| -0.328 | NA | NA | + | NA | -5.52E-02 | 1.64E-01 | 7 | -138.146 | 290.293 | 1.261 | 0.165 |
| -0.416 | NA | NA | + | -1.53E-03 | -7.17E-02 | 1.73E-01 | 8 | -137.231 | 290.462 | I.43 | 0.152 |
| -0.477 | 9.68E-10 | NA | + | NA | NA | 1.68E-01 | 7 | -138.261 | 290.522 | I.49 | 0.147 |
| -0.445 | NA | NA | + | NA | NA | 1.66E-01 | 6 | -141.615 | 295.229 | 0 | 0.418 |
| -0.508 | NA | NA | + | -7.88E-04 | NA | 1.70E-01 | 7 | -141.364 | 296.728 | 1.499 | 0.198 |
| -0.477 | 9.60E-10 | NA | + | NA | NA | 1.73E-01 | 7 | -141.387 | 296.774 | 1.545 | 0.193 |
| -0.351 | NA | NA | + | NA | -4.32E-02 | 1.68E-01 | 7 | -141.402 | 296.803 | 1.574 | 0.191 |
| -0.439 | NA | NA | + | NA | NA | 1.61E-01 | 6 | -142.785 | 297.57 | 0 | 0.434 |
| -0.469 | 8.96E-10 | NA | + | NA | NA | 1.66E-01 | 7 | -142.574 | 299.149 | 1.579 | 0.197 |
| -0.499 | NA | NA | + | -7.05E-04 | NA | 1.65E-01 | 7 | -142.607 | 299.215 | 1.645 | 0.191 |
| -0.371 | NA | NA | + | NA | -3.20E-02 | 1.63E-01 | 7 | -142.673 | 299.345 | 1.776 | 0.179 |
| -0.399 | NA | NA | + | NA | NA | 1.52E-01 | 6 | -139.005 | 290.011 | 0 | 0.413 |
| -0.277 | NA | NA | + | NA | -5.70E-02 | 1.55E-01 | 7 | -138.597 | 291.193 | 1.183 | 0.229 |
| -0.426 | 9.83E-10 | NA | + | NA | NA | 1.56E-01 | 7 | -138.832 | 291.663 | 1.653 | 0.181 |
| -0.452 | NA | NA | + | -6.28E-04 | NA | 1.55E-01 | 7 | -138.852 | 291.705 | 1.694 | 0.177 |
| -0.614 | NA | NA | + | NA | NA | 2.08E-01 | 6 | -158.205 | 328.409 | 0 | 0.301 |
| -0.434 | NA | NA | + | NA | -7.60E-02 | 2.04E-01 | 7 | -157.528 | 329.057 | 0.648 | 0.218 |
| -0.683 | NA | NA | + | -9.77E-04 | NA | 2.11E-01 | 7 | -157.809 | 329.619 | I.21 | 0.165 |
| -0.658 | 1.58E-09 | NA | + | NA | NA | 2.15E-01 | 7 | -157.818 | 329.636 | 1.227 | 0.163 |
| -0.49 | NA | NA | + | -1.26E-03 | -9.02E-02 | 2.08E-01 | 8 | -156.884 | 329.769 | I.36 | 0.153 |
| -0.478 | NA | NA | + | -1.91E-03 | -9.94E-02 | 2.00E-01 | 8 | -144.054 | 304.108 | 0 | 0.229 |
| -0.676 | NA | NA | + | -1.59E-03 | NA | 1.96E-01 | 7 | -145.346 | 304.691 | 0.583 | 0.171 |
| -0.557 | NA | NA | + | NA | NA | 1.89E-01 | 6 | -146.385 | 304.769 | 0.661 | 0.164 |
| -0.377 | NA | NA | + | NA | -8.03E-02 | 1.91E-01 | 7 | -145.526 | 305.053 | 0.945 | 0.143 |
| -0.518 | 1.10E-09 | NA | + | -1.90E-03 | -9.67E-02 | 2.06E-01 | 9 | -143.746 | 305.491 | 1.383 | 0.115 |
| -0.716 | 1.22E-09 | NA | + | -1.59E-03 | NA | 2.03E-01 | 8 | -144.971 | 305.942 | 1.834 | 0.092 |
| -0.596 | 1.20E-09 | NA | + | NA | NA | 1.96E-01 | 7 | -146.024 | 306.048 | I.94 | 0.087 |
| -0.408 | NA | NA | + | NA | NA | 1.55E-01 | 6 | -152.7 | 317.399 | 0 | 0.319 |
| -0.332 | NA | NA | NA | NA | NA | 1.94E-01 | 3 | -156.407 | 318.815 | 1.415 | 0.157 |
| -0.248 | NA | NA | NA | 1.34E-03 | NA | 1.68E-01 | 4 | -155.416 | 318.831 | 1.432 | 0.156 |
| -0.434 | 9.12E-10 | NA | + | NA | NA | 1.59E-01 | 7 | -152.563 | 319.127 | 1.727 | 0.134 |
| -0.401 | NA | NA | + | 7.47E-05 | NA | 1.54E-01 | 7 | -152.698 | 319.396 | 1.997 | 0.117 |
| -0.406 | NA | NA | + | NA | -8.19E-04 | 1.55E-01 | 7 | -152.7 | 319.399 | 2 | 0.117 |
| -0.67 | NA | NA | + | NA | NA | 2.31E-01 | 6 | -165.181 | 342.362 | 0 | 0.247 |
| -0.768 | NA | NA | + | -1.43E-03 | NA | 2.35E-01 | 7 | -164.369 | 342.738 | 0.376 | 0.205 |
| -0.598 | NA | NA | + | -1.67E-03 | -8.12E-02 | 2.34E-01 | 8 | -163.637 | 343.273 | 0.911 | 0.157 |
| -0.524 | NA | NA | + | NA | -6.37E-02 | 2.29E-01 | 7 | -164.722 | 343.443 | 1.081 | 0.144 |
| -0.719 | 1.89E-09 | NA | + | NA | NA | 2.39E-01 | 7 | -164.788 | 343.576 | 1.213 | 0.135 |
| -0.819 | 1.91E-09 | NA | + | -1.44E-03 | NA | 2.43E-01 | 8 | -163.961 | 343.922 | I.56 | 0.113 |
| -0.49 | NA | NA | + | NA | NA | 1.78E-01 | 6 | -148.783 | 309.566 | 0 | 0.41 |
| -0.528 | 1.37E-09 | NA | + | NA | NA | 1.83E-01 | 7 | -148.477 | 310.954 | 1.388 | 0.205 |
| -0.553 | NA | NA | + | -8.37E-04 | NA | 1.81E-01 | 7 | -148.51 | 311.02 | 1.454 | 0.198 |
| -0.399 | NA | NA | + | NA | -4.38E-02 | 1.80E-01 | 7 | -148.567 | 311.134 | 1.568 | 0.187 |
| -0.52 | NA | NA | + | NA | NA | 1.78E-01 | 6 | -166.909 | 345.817 | 0 | 0.455 |
| -0.554 | 1.21E-09 | NA | + | NA | NA | 1.84E-01 | 7 | -166.724 | 347.448 | 1.631 | 0.201 |
| -0.563 | NA | NA | + | NA | 1.91E-02 | 1.77E-01 | 7 | -166.877 | 347.755 | 1.937 | 0.173 |
| -0.54 | NA | NA | + | -2.79E-04 | NA | 1.79E-01 | 7 | -166.886 | 347.771 | 1.954 | 0.171 |
| -0.456 | NA | NA | + | NA | NA | 1.70E-01 | 6 | -134.165 | 280.331 | 0 | 0.395 |
| -0.345 | NA | NA | + | NA | -5.17E-02 | 1.72E-01 | 7 | -133.823 | 281.645 | 1.314 | 0.205 |
| -0.528 | NA | NA | + | -8.97E-04 | NA | 1.75E-01 | 7 | -133.835 | 281.67 | 1.339 | 0.202 |
| -0.493 | 1.32E-09 | NA | + | NA | NA | 1.76E-01 | 7 | -133.858 | 281.716 | 1.385 | 0.198 |
| -0.54 | NA | NA | + | NA | NA | 1.92E-01 | 6 | -147.819 | 307.638 | 0 | 0.387 |
| -0.624 | NA | NA | + | -1.12E-03 | NA | 1.96E-01 | 7 | -147.315 | 308.63 | 0.993 | 0.236 |
| -0.427 | NA | NA | + | NA | -5.11E-02 | 1.93E-01 | 7 | -147.519 | 309.039 | 1.401 | 0.192 |
| -0.574 | 1.21E-09 | NA | + | NA | NA | 1.97E-01 | 7 | -147.556 | 309.112 | 1.475 | 0.185 |
| -0.596 | NA | NA | + | NA | NA | 1.98E-01 | 6 | -163.059 | 338.119 | 0 | 0.388 |
| -0.679 | NA | NA | + | -1.21E-03 | NA | 2.01E-01 | 7 | -162.504 | 339.008 | 0.889 | 0.249 |
| -0.636 | 1.39E-09 | NA | + | NA | NA | 2.05E-01 | 7 | -162.77 | 339.54 | 1.422 | 0.191 |
| -0.499 | NA | NA | + | NA | -4.32E-02 | 1.98E-01 | 7 | -162.87 | 339.741 | 1.622 | 0.172 |
| -0.355 | NA | NA | + | NA | NA | 1.41E-01 | 6 | -145.726 | 303.453 | 0 | 0.377 |
| -0.297 | NA | NA | NA | NA | NA | 1.76E-01 | 3 | -149.563 | 305.126 | 1.673 | 0.164 |
| -0.378 | 8.55E-10 | NA | + | NA | NA | 1.45E-01 | 7 | -145.593 | 305.187 | 1.734 | 0.159 |
| -0.401 | NA | NA | + | -5.19E-04 | NA | 1.44E-01 | 7 | -145.638 | 305.277 | 1.824 | 0.152 |
| -0.302 | NA | NA | + | NA | -2.56E-02 | 1.43E-01 | 7 | -145.658 | 305.316 | 1.864 | 0.149 |
| -0.476 | NA | NA | + | NA | NA | 1.72E-01 | 6 | -153.563 | 319.125 | 0 | 0.427 |
| -0.513 | 1.20E-09 | NA | + | NA | NA | 1.79E-01 | 7 | -153.298 | 320.595 | I.47 | 0.205 |
| -0.37 | NA | NA | + | NA | -4.75E-02 | 1.73E-01 | 7 | -153.307 | 320.614 | 1.489 | 0.203 |
| -0.506 | NA | NA | + | -4.00E-04 | NA | 1.74E-01 | 7 | -153.504 | 321.008 | 1.883 | 0.166 |
| -0.486 | NA | NA | + | NA | NA | 1.82E-01 | 6 | -157.823 | 327.646 | 0 | 0.448 |
| -0.522 | 1.34E-09 | NA | + | NA | NA | 1.88E-01 | 7 | -157.56 | 329.119 | 1.474 | 0.214 |
| -0.438 | NA | NA | + | NA | -2.15E-02 | 1.83E-01 | 7 | -157.776 | 329.553 | 1.907 | 0.173 |
| -0.492 | NA | NA | + | -8.51E-05 | NA | 1.83E-01 | 7 | -157.821 | 329.641 | 1.995 | 0.165 |
| -0.471 | NA | NA | + | NA | NA | 1.67E-01 | 6 | -148.354 | 308.708 | 0 | 0.426 |
| -0.539 | NA | NA | + | -8.27E-04 | NA | 1.71E-01 | 7 | -148.111 | 310.222 | 1.514 | 0.2 |
| -0.5 | 1.05E-09 | NA | + | NA | NA | 1.72E-01 | 7 | -148.157 | 310.315 | 1.607 | 0.191 |
| -0.391 | NA | NA | + | NA | -3.74E-02 | 1.69E-01 | 7 | -148.2 | 310.4 | 1.692 | 0.183 |
| -0.512 | NA | NA | + | NA | NA | 1.84E-01 | 6 | -147.626 | 307.253 | 0 | 0.322 |
| -0.601 | NA | NA | + | -1.13E-03 | NA | 1.90E-01 | 7 | -147.135 | 308.271 | 1.018 | 0.193 |
| -0.377 | NA | NA | + | NA | -6.07E-02 | 1.85E-01 | 7 | -147.166 | 308.333 | 01.VIII | 0.188 |
| -0.549 | 1.33E-09 | NA | + | NA | NA | 1.90E-01 | 7 | -147.327 | 308.655 | 1.402 | 0.16 |
| -0.456 | NA | NA | + | -1.35E-03 | -7.33E-02 | 1.92E-01 | 8 | -146.478 | 308.957 | 1.704 | 0.137 |
| -0.521 | NA | NA | + | NA | NA | 1.85E-01 | 6 | -157.767 | 327.534 | 0 | 0.433 |
| -0.557 | 1.41E-09 | NA | + | NA | NA | 1.91E-01 | 7 | -157.511 | 329.021 | 1.487 | 0.206 |
| -0.44 | NA | NA | + | NA | -3.67E-02 | 1.86E-01 | 7 | -157.634 | 329.268 | 1.734 | 0.182 |
| -0.567 | NA | NA | + | -5.87E-04 | NA | 1.88E-01 | 7 | -157.645 | 329.291 | 1.756 | 0.18 |
| -0.366 | NA | NA | + | NA | NA | 1.38E-01 | 6 | -139.33 | 290.661 | 0 | 0.33 |
| -0.297 | NA | NA | NA | NA | NA | 1.78E-01 | 3 | -143.157 | 292.314 | 1.653 | 0.144 |
| -0.392 | 7.14E-10 | NA | + | NA | NA | 1.44E-01 | 7 | -139.174 | 292.348 | 1.688 | 0.142 |
| -0.219 | NA | NA | NA | 1.19E-03 | NA | 1.55E-01 | 4 | -142.251 | 292.503 | 1.842 | 0.131 |
| -0.315 | NA | NA | + | NA | -2.37E-02 | 1.39E-01 | 7 | -139.267 | 292.533 | 1.873 | 0.129 |
| -0.39 | NA | NA | + | -2.70E-04 | NA | 1.39E-01 | 7 | -139.305 | 292.611 | I.95 | 0.124 |
| -0.587 | NA | NA | + | NA | NA | 1.99E-01 | 6 | -154.388 | 320.775 | 0 | 0.316 |
| -0.436 | NA | NA | + | NA | -6.71E-02 | 2.00E-01 | 7 | -153.859 | 321.717 | 0.942 | 0.197 |
| -0.656 | NA | NA | + | -1.01E-03 | NA | 2.00E-01 | 7 | -153.958 | 321.916 | 1.141 | 0.179 |
| -0.629 | 1.24E-09 | NA | + | NA | NA | 2.08E-01 | 7 | -154.011 | 322.023 | 1.247 | 0.169 |
| -0.491 | NA | NA | + | -1.25E-03 | -8.07E-02 | 2.02E-01 | 8 | -153.212 | 322.424 | 1.648 | 0.139 |
| -0.546 | NA | NA | + | -1.70E-03 | -1.08E-01 | 2.31E-01 | 8 | -160.556 | 337.112 | 0 | 0.194 |
| -0.676 | NA | NA | + | NA | NA | 2.27E-01 | 6 | -162.757 | 337.514 | 0.402 | 0.159 |
| -0.471 | NA | NA | + | NA | -8.88E-02 | 2.25E-01 | 7 | -161.802 | 337.605 | 0.492 | 0.152 |
| -0.771 | NA | NA | + | -1.37E-03 | NA | 2.32E-01 | 7 | -161.941 | 337.882 | 0.77 | 0.132 |
| -0.602 | 1.77E-09 | NA | + | -1.69E-03 | -1.02E-01 | 2.37E-01 | 9 | -160.19 | 338.381 | 1.268 | 0.103 |
| -0.728 | 2.11E-09 | NA | + | NA | NA | 2.34E-01 | 7 | -162.245 | 338.49 | 1.378 | 0.097 |
| -0.824 | 2.13E-09 | NA | + | -1.37E-03 | NA | 2.39E-01 | 8 | -161.414 | 338.828 | 1.716 | 0.082 |
| -0.53 | 1.82E-09 | NA | + | NA | -8.26E-02 | 2.32E-01 | 8 | -161.423 | 338.847 | 1.734 | 0.081 |
| -0.666 | NA | NA | + | NA | NA | 2.19E-01 | 6 | -170.231 | 352.461 | 0 | 0.384 |
| -0.745 | NA | NA | + | -1.09E-03 | NA | 2.23E-01 | 7 | -169.817 | 353.633 | 1.172 | 0.213 |
| -0.715 | 1.66E-09 | NA | + | NA | NA | 2.28E-01 | 7 | -169.836 | 353.673 | 1.212 | 0.209 |
| -0.535 | NA | NA | + | NA | -5.56E-02 | 2.17E-01 | 7 | -169.914 | 353.829 | 1.367 | 0.194 |
| -0.524 | NA | NA | + | NA | NA | 1.79E-01 | 6 | -161.039 | 334.078 | 0 | 0.435 |
| -0.565 | 1.44E-09 | NA | + | NA | NA | 1.86E-01 | 7 | -160.763 | 335.526 | 1.448 | 0.211 |
| -0.446 | NA | NA | + | NA | -3.46E-02 | 1.80E-01 | 7 | -160.917 | 335.835 | 1.757 | 0.181 |
| -0.56 | NA | NA | + | -4.80E-04 | NA | 1.81E-01 | 7 | -160.961 | 335.922 | 1.844 | 0.173 |
| -0.387 | NA | NA | + | NA | NA | 1.49E-01 | 6 | -152.323 | 316.647 | 0 | 0.298 |
| -0.236 | NA | NA | NA | 1.39E-03 | NA | 1.61E-01 | 4 | -154.79 | 317.58 | 0.934 | 0.187 |
| -0.324 | NA | NA | NA | NA | NA | 1.87E-01 | 3 | -155.894 | 317.789 | 1.142 | 0.169 |
| -0.413 | 8.24E-10 | NA | + | NA | NA | 1.54E-01 | 7 | -152.192 | 318.383 | 1.737 | 0.125 |
| -0.372 | NA | NA | + | 1.72E-04 | NA | 1.48E-01 | 7 | -152.314 | 318.627 | 1.981 | 0.111 |
| -0.383 | NA | NA | + | NA | -1.65E-03 | 1.49E-01 | 7 | -152.323 | 318.646 | 1.999 | 0.11 |
| -0.517 | NA | NA | + | NA | NA | 1.79E-01 | 6 | -153.761 | 319.523 | 0 | 0.435 |
| -0.551 | 1.16E-09 | NA | + | NA | NA | 1.85E-01 | 7 | -153.512 | 321.023 | 01.V | 0.205 |
| -0.575 | NA | NA | + | -7.50E-04 | NA | 1.82E-01 | 7 | -153.57 | 321.14 | 1.618 | 0.194 |
| -0.476 | NA | NA | + | NA | -1.88E-02 | 1.80E-01 | 7 | -153.726 | 321.451 | 1.928 | 0.166 |
| -0.504 | NA | NA | + | NA | NA | 1.79E-01 | 6 | -148.433 | 308.866 | 0 | 0.418 |
| -0.577 | NA | NA | + | -9.49E-04 | NA | 1.83E-01 | 7 | -148.101 | 310.202 | 1.335 | 0.215 |
| -0.536 | 1.19E-09 | NA | + | NA | NA | 1.84E-01 | 7 | -148.213 | 310.425 | 1.559 | 0.192 |
| -0.429 | NA | NA | + | NA | -3.45E-02 | 1.80E-01 | 7 | -148.304 | 310.608 | 1.742 | 0.175 |
| -0.576 | NA | NA | + | NA | NA | 1.95E-01 | 6 | -151.908 | 315.816 | 0 | 0.322 |
| -0.435 | NA | NA | + | NA | -6.38E-02 | 1.96E-01 | 7 | -151.44 | 316.881 | 1.065 | 0.189 |
| -0.651 | NA | NA | + | -1.06E-03 | NA | 2.00E-01 | 7 | -151.465 | 316.93 | 1.114 | 0.185 |
| -0.619 | 1.51E-09 | NA | + | NA | NA | 2.02E-01 | 7 | -151.55 | 317.099 | 1.284 | 0.17 |
| -0.496 | NA | NA | + | -1.31E-03 | -7.81E-02 | 2.02E-01 | 8 | -150.785 | 317.569 | 1.754 | 0.134 |
| -0.606 | NA | NA | + | NA | NA | 2.09E-01 | 6 | -153.983 | 319.966 | 0 | 0.234 |
| -0.713 | NA | NA | + | -1.55E-03 | NA | 2.15E-01 | 7 | -153.04 | 320.079 | 0.113 | 0.221 |
| -0.565 | NA | NA | + | -1.78E-03 | -7.61E-02 | 2.19E-01 | 8 | -152.355 | 320.709 | 0.743 | 0.161 |
| -0.48 | NA | NA | + | NA | -5.88E-02 | 2.11E-01 | 7 | -153.568 | 321.136 | 1.169 | 0.13 |
| -0.65 | 1.47E-09 | NA | + | NA | NA | 2.17E-01 | 7 | -153.574 | 321.147 | 1.181 | 0.129 |
| -0.759 | 1.49E-09 | NA | + | -1.56E-03 | NA | 2.24E-01 | 8 | -152.611 | 321.222 | 1.256 | 0.125 |
| -0.696 | NA | NA | + | NA | NA | 2.26E-01 | 6 | -161.571 | 335.141 | 0 | 0.259 |
| -0.778 | NA | NA | + | -1.28E-03 | NA | 2.27E-01 | 7 | -160.89 | 335.781 | 0.639 | 0.188 |
| -0.55 | NA | NA | + | NA | -6.46E-02 | 2.26E-01 | 7 | -161.096 | 336.193 | 1.052 | 0.153 |
| -0.744 | 1.75E-09 | NA | + | NA | NA | 2.33E-01 | 7 | -161.133 | 336.266 | 1.125 | 0.148 |
| -0.61 | NA | NA | + | -1.53E-03 | -8.14E-02 | 2.29E-01 | 8 | -160.156 | 336.311 | I.17 | 0.144 |
| -0.826 | 1.76E-09 | NA | + | -1.28E-03 | NA | 2.35E-01 | 8 | -160.447 | 336.894 | 1.753 | 0.108 |
| -0.418 | NA | NA | + | NA | NA | 1.54E-01 | 6 | -142.48 | 296.961 | 0 | 0.443 |
| -0.447 | 7.65E-10 | NA | + | NA | NA | 1.61E-01 | 7 | -142.27 | 298.539 | 1.578 | 0.201 |
| -0.462 | NA | NA | + | -5.28E-04 | NA | 1.56E-01 | 7 | -142.373 | 298.746 | 1.785 | 0.181 |
| -0.362 | NA | NA | + | NA | -2.57E-02 | 1.55E-01 | 7 | -142.408 | 298.816 | 1.855 | 0.175 |
| -0.519 | NA | NA | + | NA | NA | 1.79E-01 | 6 | -172.766 | 357.531 | 0 | 0.457 |
| -0.552 | 1.02E-09 | NA | + | NA | NA | 1.85E-01 | 7 | -172.577 | 359.154 | 1.623 | 0.203 |
| -0.538 | NA | NA | + | -2.49E-04 | NA | 1.80E-01 | 7 | -172.748 | 359.496 | 1.965 | 0.171 |
| -0.517 | NA | NA | + | NA | -7.92E-04 | 1.79E-01 | 7 | -172.766 | 359.531 | 2 | 0.168 |
| -0.429 | NA | NA | + | NA | NA | 1.61E-01 | 6 | -148.563 | 309.125 | 0 | 0.45 |
| -0.458 | 9.35E-10 | NA | + | NA | NA | 1.66E-01 | 7 | -148.385 | 310.77 | 1.645 | 0.198 |
| -0.47 | NA | NA | + | -5.09E-04 | NA | 1.63E-01 | 7 | -148.472 | 310.943 | 1.818 | 0.181 |
| -0.393 | NA | NA | + | NA | -1.66E-02 | 1.61E-01 | 7 | -148.533 | 311.067 | 1.942 | 0.171 |
| -0.574 | NA | NA | + | -1.80E-03 | -1.10E-01 | 2.41E-01 | 8 | -159.176 | 334.351 | 0 | 0.243 |
| -0.503 | NA | NA | + | NA | -9.26E-02 | 2.37E-01 | 7 | -160.545 | 335.09 | 0.738 | 0.168 |
| -0.715 | NA | NA | + | NA | NA | 2.39E-01 | 6 | -161.72 | 335.44 | 1.089 | 0.141 |
| -0.633 | 1.78E-09 | NA | + | -1.77E-03 | -1.03E-01 | 2.48E-01 | 9 | -158.793 | 335.587 | 1.235 | 0.131 |
| -0.805 | NA | NA | + | -1.46E-03 | NA | 2.43E-01 | 7 | -160.815 | 335.629 | 1.278 | 0.128 |
| -0.773 | 2.25E-09 | NA | + | NA | NA | 2.48E-01 | 7 | -161.12 | 336.24 | 1.889 | 0.095 |
| -0.568 | 1.88E-09 | NA | + | NA | -8.56E-02 | 2.45E-01 | 8 | -160.124 | 336.248 | 1.897 | 0.094 |
| -0.499 | NA | NA | + | NA | NA | 1.79E-01 | 6 | -171.418 | 354.835 | 0 | 0.451 |
| -0.537 | 1.33E-09 | NA | + | NA | NA | 1.86E-01 | 7 | -171.174 | 356.348 | 1.512 | 0.212 |
| -0.479 | NA | NA | + | 2.60E-04 | NA | 1.78E-01 | 7 | -171.398 | 356.797 | 1.962 | 0.169 |
| -0.471 | NA | NA | + | NA | -1.24E-02 | 1.80E-01 | 7 | -171.403 | 356.807 | 1.971 | 0.168 |
| -0.407 | NA | NA | + | NA | NA | 1.52E-01 | 6 | -144.6 | 301.2 | 0 | 0.448 |
| -0.433 | 8.46E-10 | NA | + | NA | NA | 1.57E-01 | 7 | -144.449 | 302.899 | 1.699 | 0.192 |
| -0.339 | NA | NA | + | NA | -3.10E-02 | 1.54E-01 | 7 | -144.498 | 302.997 | 1.797 | 0.182 |
| -0.446 | NA | NA | + | -4.62E-04 | NA | 1.55E-01 | 7 | -144.524 | 303.048 | 1.848 | 0.178 |
| -0.37 | NA | NA | + | NA | NA | 1.41E-01 | 6 | -154.722 | 321.445 | 0 | 0.235 |
| -0.193 | NA | NA | NA | 1.45E-03 | NA | 1.46E-01 | 4 | -156.797 | 321.594 | 0.149 | 0.218 |
| -0.288 | NA | NA | NA | NA | NA | 1.75E-01 | 3 | -157.96 | 321.92 | 0.476 | 0.185 |
| -0.394 | 7.83E-10 | NA | + | NA | NA | 1.45E-01 | 7 | -154.618 | 323.236 | 1.791 | 0.096 |
| -0.342 | NA | NA | + | 3.19E-04 | NA | 1.38E-01 | 7 | -154.692 | 323.384 | I.94 | 0.089 |
| -0.217 | 7.87E-10 | NA | NA | 1.45E-03 | NA | 1.50E-01 | 5 | -156.694 | 323.388 | 1.943 | 0.089 |
| -0.39 | NA | NA | + | NA | 9.16E-03 | 1.40E-01 | 7 | -154.715 | 323.429 | 1.985 | 0.087 |
| -0.397 | NA | NA | + | NA | NA | 1.52E-01 | 6 | -142.212 | 296.425 | 0 | 0.443 |
| -0.427 | 1.05E-09 | NA | + | NA | NA | 1.57E-01 | 7 | -141.997 | 297.993 | 1.568 | 0.202 |
| -0.449 | NA | NA | + | -6.49E-04 | NA | 1.55E-01 | 7 | -142.064 | 298.128 | 1.703 | 0.189 |
| -0.368 | NA | NA | + | NA | -1.37E-02 | 1.53E-01 | 7 | -142.192 | 298.385 | I.96 | 0.166 |
| -0.595 | NA | NA | + | NA | NA | 2.01E-01 | 6 | -159.58 | 331.159 | 0 | 0.329 |
| -0.443 | NA | NA | + | NA | -6.86E-02 | 2.03E-01 | 7 | -159.055 | 332.11 | 0.951 | 0.204 |
| -0.638 | 1.65E-09 | NA | + | NA | NA | 2.08E-01 | 7 | -159.215 | 332.43 | 1.271 | 0.174 |
| -0.668 | NA | NA | + | -9.46E-04 | NA | 2.07E-01 | 7 | -159.256 | 332.511 | 1.352 | 0.167 |
| -0.508 | NA | NA | + | -1.21E-03 | -8.12E-02 | 2.10E-01 | 8 | -158.541 | 333.082 | 1.923 | 0.126 |
| -0.561 | NA | NA | + | NA | NA | 1.96E-01 | 6 | -149.264 | 310.528 | 0 | 0.264 |
| -0.665 | NA | NA | + | -1.37E-03 | NA | 2.03E-01 | 7 | -148.519 | 311.038 | 0.51 | 0.204 |
| -0.426 | NA | NA | + | NA | -5.98E-02 | 1.96E-01 | 7 | -148.842 | 311.683 | 1.155 | 0.148 |
| -0.51 | NA | NA | + | -1.61E-03 | -7.62E-02 | 2.04E-01 | 8 | -147.846 | 311.693 | 1.165 | 0.147 |
| -0.6 | 1.45E-09 | NA | + | NA | NA | 2.02E-01 | 7 | -148.956 | 311.913 | 1.385 | 0.132 |
| -0.707 | 1.50E-09 | NA | + | -1.39E-03 | NA | 2.10E-01 | 8 | -148.184 | 312.369 | 1.841 | 0.105 |
| -0.499 | NA | NA | + | NA | NA | 1.81E-01 | 6 | -146.391 | 304.782 | 0 | 0.309 |
| -0.348 | NA | NA | + | NA | -6.85E-02 | 1.83E-01 | 7 | -145.826 | 305.652 | 0.87 | 0.2 |
| -0.586 | NA | NA | + | -1.12E-03 | NA | 1.87E-01 | 7 | -145.886 | 305.773 | 0.99 | 0.189 |
| -0.424 | NA | NA | + | -1.38E-03 | -8.29E-02 | 1.90E-01 | 8 | -145.079 | 306.158 | 1.376 | 0.156 |
| -0.532 | 1.25E-09 | NA | + | NA | NA | 1.87E-01 | 7 | -146.142 | 306.284 | 1.501 | 0.146 |
| -0.452 | NA | NA | + | NA | NA | 1.63E-01 | 6 | -148.5 | 309 | 0 | 0.423 |
| -0.492 | 1.38E-09 | NA | + | NA | NA | 1.69E-01 | 7 | -148.178 | 310.357 | 1.356 | 0.215 |
| -0.36 | NA | NA | + | NA | -4.22E-02 | 1.65E-01 | 7 | -148.296 | 310.592 | 1.591 | 0.191 |
| -0.492 | NA | NA | + | -5.12E-04 | NA | 1.65E-01 | 7 | -148.409 | 310.818 | 1.818 | 0.171 |
| -0.543 | NA | NA | + | NA | NA | 1.90E-01 | 6 | -161.266 | 334.531 | 0 | 0.436 |
| -0.581 | 1.10E-09 | NA | + | NA | NA | 1.97E-01 | 7 | -160.984 | 335.968 | 1.436 | 0.213 |
| -0.589 | NA | NA | + | -6.18E-04 | NA | 1.92E-01 | 7 | -161.135 | 336.27 | 1.739 | 0.183 |
| -0.493 | NA | NA | + | NA | -2.33E-02 | 1.91E-01 | 7 | -161.213 | 336.426 | 1.895 | 0.169 |
| -0.424 | NA | NA | + | NA | NA | 1.53E-01 | 6 | -153.063 | 318.126 | 0 | 0.443 |
| -0.457 | 1.06E-09 | NA | + | NA | NA | 1.59E-01 | 7 | -152.843 | 319.686 | 1.561 | 0.203 |
| -0.473 | NA | NA | + | -5.87E-04 | NA | 1.57E-01 | 7 | -152.953 | 319.906 | 1.781 | 0.182 |
| -0.372 | NA | NA | + | NA | -2.33E-02 | 1.54E-01 | 7 | -153.008 | 320.015 | I.89 | 0.172 |
| -0.648 | NA | NA | + | NA | NA | 2.13E-01 | 6 | -160.884 | 333.768 | 0 | 0.201 |
| -0.478 | NA | NA | + | NA | -7.58E-02 | 2.14E-01 | 7 | -160.199 | 334.399 | 0.631 | 0.147 |
| -0.542 | NA | NA | + | -1.55E-03 | -9.34E-02 | 2.19E-01 | 8 | -159.235 | 334.469 | 0.701 | 0.142 |
| -0.731 | NA | NA | + | -1.24E-03 | NA | 2.16E-01 | 7 | -160.246 | 334.492 | 0.724 | 0.14 |
| -0.699 | 1.76E-09 | NA | + | NA | NA | 2.21E-01 | 7 | -160.376 | 334.752 | 0.984 | 0.123 |
| -0.78 | 1.73E-09 | NA | + | -1.23E-03 | NA | 2.25E-01 | 8 | -159.749 | 335.498 | I.73 | 0.085 |
| -0.534 | 1.62E-09 | NA | + | NA | -7.15E-02 | 2.22E-01 | 8 | -159.767 | 335.533 | 1.765 | 0.083 |
| -0.595 | 1.56E-09 | NA | + | -1.52E-03 | -8.90E-02 | 2.26E-01 | 9 | -158.832 | 335.663 | 1.895 | 0.078 |
| -0.613 | NA | NA | + | NA | NA | 2.15E-01 | 6 | -163.007 | 338.015 | 0 | 0.32 |
| -0.435 | NA | NA | + | NA | -7.70E-02 | 2.13E-01 | 7 | -162.299 | 338.597 | 0.582 | 0.239 |
| -0.668 | 1.93E-09 | NA | + | NA | NA | 2.24E-01 | 7 | -162.528 | 339.056 | 1.041 | 0.19 |
| -0.497 | 1.69E-09 | NA | + | NA | -7.08E-02 | 2.21E-01 | 8 | -161.934 | 339.868 | 1.853 | 0.127 |
| -0.644 | NA | NA | + | -4.00E-04 | NA | 2.17E-01 | 7 | -162.95 | 339.899 | 1.884 | 0.125 |
| -0.359 | NA | NA | + | NA | NA | 1.40E-01 | 6 | -152.75 | 317.5 | 0 | 0.273 |
| -0.202 | NA | NA | NA | 1.41E-03 | NA | 1.48E-01 | 4 | -155.003 | 318.005 | 0.505 | 0.212 |
| -0.295 | NA | NA | NA | NA | NA | 1.74E-01 | 3 | -156.104 | 318.208 | 0.708 | 0.192 |
| -0.384 | 8.33E-10 | NA | + | NA | NA | 1.44E-01 | 7 | -152.628 | 319.257 | 1.757 | 0.113 |
| -0.42 | NA | NA | + | NA | 2.82E-02 | 1.39E-01 | 7 | -152.679 | 319.359 | 1.859 | 0.108 |
| -0.342 | NA | NA | + | 2.04E-04 | NA | 1.39E-01 | 7 | -152.738 | 319.476 | 1.976 | 0.102 |
| -0.563 | NA | NA | + | NA | NA | 1.94E-01 | 6 | -167.57 | 347.14 | 0 | 0.426 |
| -0.61 | 1.66E-09 | NA | + | NA | NA | 2.02E-01 | 7 | -167.22 | 348.439 | 1.299 | 0.222 |
| -0.615 | NA | NA | + | -7.35E-04 | NA | 1.96E-01 | 7 | -167.393 | 348.786 | 1.646 | 0.187 |
| -0.515 | NA | NA | + | NA | -2.23E-02 | 1.95E-01 | 7 | -167.521 | 349.043 | 1.903 | 0.165 |
| -0.456 | NA | NA | + | NA | NA | 1.67E-01 | 6 | -135.512 | 283.024 | 0 | 0.266 |
| -0.563 | NA | NA | + | -1.35E-03 | NA | 1.72E-01 | 7 | -134.743 | 283.487 | 0.463 | 0.211 |
| -0.424 | NA | NA | + | -1.58E-03 | -7.32E-02 | 1.76E-01 | 8 | -134.084 | 284.167 | 1.143 | 0.15 |
| -0.335 | NA | NA | + | NA | -5.70E-02 | 1.70E-01 | 7 | -135.103 | 284.206 | 1.182 | 0.147 |
| -0.486 | 9.76E-10 | NA | + | NA | NA | 1.72E-01 | 7 | -135.262 | 284.525 | 1.501 | 0.125 |
| -0.595 | 1.01E-09 | NA | + | -1.37E-03 | NA | 1.77E-01 | 8 | -134.475 | 284.949 | 1.926 | 0.101 |
| -0.459 | NA | NA | + | NA | NA | 1.64E-01 | 6 | -148.707 | 309.414 | 0 | 0.41 |
| -0.334 | NA | NA | + | NA | -5.46E-02 | 1.64E-01 | 7 | -148.378 | 310.756 | 1.341 | 0.209 |
| -0.497 | 1.00E-09 | NA | + | NA | NA | 1.73E-01 | 7 | -148.408 | 310.815 | 1.401 | 0.203 |
| -0.513 | NA | NA | + | -6.81E-04 | NA | 1.68E-01 | 7 | -148.543 | 311.086 | 1.671 | 0.178 |
| -0.58 | NA | NA | + | NA | NA | 2.00E-01 | 6 | -153.175 | 318.351 | 0 | 0.31 |
| -0.677 | NA | NA | + | -1.31E-03 | NA | 2.06E-01 | 7 | -152.508 | 319.017 | 0.666 | 0.222 |
| -0.453 | NA | NA | + | NA | -5.70E-02 | 2.00E-01 | 7 | -152.808 | 319.617 | 1.266 | 0.165 |
| -0.62 | 1.40E-09 | NA | + | NA | NA | 2.07E-01 | 7 | -152.875 | 319.751 | 01.IV | 0.154 |
| -0.53 | NA | NA | + | -1.54E-03 | -7.37E-02 | 2.07E-01 | 8 | -151.909 | 319.817 | 1.467 | 0.149 |
| -0.461 | NA | NA | + | NA | NA | 1.66E-01 | 6 | -147.371 | 306.743 | 0 | 0.388 |
| -0.321 | NA | NA | + | NA | -6.42E-02 | 1.68E-01 | 7 | -146.897 | 307.794 | 1.052 | 0.23 |
| -0.536 | NA | NA | + | -9.32E-04 | NA | 1.70E-01 | 7 | -147.034 | 308.067 | 1.325 | 0.2 |
| -0.494 | 1.40E-09 | NA | + | NA | NA | 1.70E-01 | 7 | -147.132 | 308.263 | 1.521 | 0.182 |
| -0.438 | NA | NA | + | NA | NA | 1.60E-01 | 6 | -147.168 | 306.335 | 0 | 0.43 |
| -0.508 | NA | NA | + | -8.61E-04 | NA | 1.64E-01 | 7 | -146.902 | 307.804 | 1.469 | 0.206 |
| -0.469 | 1.10E-09 | NA | + | NA | NA | 1.65E-01 | 7 | -146.962 | 307.924 | 1.589 | 0.194 |
| -0.385 | NA | NA | + | NA | -2.47E-02 | 1.61E-01 | 7 | -147.102 | 308.203 | 1.868 | 0.169 |
| -0.445 | NA | NA | + | NA | NA | 1.63E-01 | 6 | -145.126 | 302.252 | 0 | 0.43 |
| -0.504 | NA | NA | + | -7.17E-04 | NA | 1.67E-01 | 7 | -144.934 | 303.869 | 1.616 | 0.192 |
| -0.361 | NA | NA | + | NA | -3.90E-02 | 1.64E-01 | 7 | -144.949 | 303.899 | 1.646 | 0.189 |
| -0.475 | 9.79E-10 | NA | + | NA | NA | 1.68E-01 | 7 | -144.951 | 303.901 | 1.649 | 0.189 |
| -0.431 | NA | NA | + | NA | NA | 1.57E-01 | 6 | -155.459 | 322.918 | 0 | 0.454 |
| -0.465 | 1.14E-09 | NA | + | NA | NA | 1.61E-01 | 7 | -155.283 | 324.565 | 1.647 | 0.199 |
| -0.47 | NA | NA | + | -4.66E-04 | NA | 1.59E-01 | 7 | -155.39 | 324.781 | 1.863 | 0.179 |
| -0.435 | NA | NA | + | NA | 1.73E-03 | 1.57E-01 | 7 | -155.459 | 324.917 | 1.999 | 0.167 |
| -0.555 | NA | NA | + | NA | NA | 1.95E-01 | 6 | -152.709 | 317.418 | 0 | 0.253 |
| -0.376 | NA | NA | + | NA | -7.95E-02 | 1.95E-01 | 7 | -151.979 | 317.959 | 0.541 | 0.193 |
| -0.448 | NA | NA | + | -1.44E-03 | -9.58E-02 | 2.02E-01 | 8 | -151.164 | 318.328 | 0.91 | 0.161 |
| -0.64 | NA | NA | + | -1.13E-03 | NA | 2.00E-01 | 7 | -152.193 | 318.386 | 0.968 | 0.156 |
| -0.598 | 1.67E-09 | NA | + | NA | NA | 2.01E-01 | 7 | -152.31 | 318.62 | 1.202 | 0.139 |
| -0.424 | 1.51E-09 | NA | + | NA | -7.55E-02 | 2.01E-01 | 8 | -151.652 | 319.305 | 1.887 | 0.099 |
| -0.342 | NA | NA | + | NA | NA | 1.42E-01 | 6 | -133.609 | 279.217 | 0 | 0.303 |
| -0.283 | NA | NA | NA | NA | NA | 1.74E-01 | 3 | -137.134 | 280.268 | 01.V | 0.179 |
| -0.212 | NA | NA | NA | 1.10E-03 | NA | 1.54E-01 | 4 | -136.266 | 280.531 | 1.314 | 0.157 |
| -0.365 | 7.88E-10 | NA | + | NA | NA | 1.46E-01 | 7 | -133.46 | 280.919 | 1.702 | 0.129 |
| -0.377 | NA | NA | + | -3.88E-04 | NA | 1.44E-01 | 7 | -133.557 | 281.113 | 1.896 | 0.117 |
| -0.369 | NA | NA | + | NA | 1.27E-02 | 1.41E-01 | 7 | -133.591 | 281.183 | 1.965 | 0.113 |
| -0.496 | NA | NA | + | NA | NA | 1.73E-01 | 6 | -148.813 | 309.626 | 0 | 0.417 |
| -0.572 | NA | NA | + | -9.93E-04 | NA | 1.77E-01 | 7 | -148.442 | 310.883 | 1.257 | 0.223 |
| -0.527 | 1.06E-09 | NA | + | NA | NA | 1.78E-01 | 7 | -148.613 | 311.226 | 01.VI | 0.187 |
| -0.423 | NA | NA | + | NA | -3.29E-02 | 1.74E-01 | 7 | -148.695 | 311.389 | 1.763 | 0.173 |
| -0.63 | NA | NA | + | -2.04E-03 | -1.11E-01 | 2.47E-01 | 8 | -161.091 | 338.181 | 0 | 0.245 |
| -0.856 | NA | NA | + | -1.67E-03 | NA | 2.45E-01 | 7 | -162.545 | 339.091 | 0.91 | 0.155 |
| -0.693 | 1.72E-09 | NA | + | -2.02E-03 | -1.06E-01 | 2.55E-01 | 9 | -160.572 | 339.144 | 0.962 | 0.151 |
| -0.752 | NA | NA | + | NA | NA | 2.41E-01 | 6 | -163.729 | 339.458 | 1.277 | 0.129 |
| -0.557 | NA | NA | + | NA | -8.68E-02 | 2.42E-01 | 7 | -162.82 | 339.64 | 1.459 | 0.118 |
| -0.914 | 1.95E-09 | NA | + | -1.66E-03 | NA | 2.55E-01 | 8 | -161.892 | 339.784 | 1.603 | 0.11 |
| -0.811 | 1.96E-09 | NA | + | NA | NA | 2.50E-01 | 7 | -163.079 | 340.158 | 1.977 | 0.091 |
| -0.474 | NA | NA | + | NA | NA | 1.70E-01 | 6 | -158.061 | 328.122 | 0 | 0.443 |
| -0.514 | 9.31E-10 | NA | + | NA | NA | 1.79E-01 | 7 | -157.777 | 329.554 | 1.432 | 0.217 |
| -0.412 | NA | NA | + | NA | -2.82E-02 | 1.70E-01 | 7 | -157.981 | 329.961 | I.84 | 0.177 |
| -0.472 | NA | NA | + | 2.92E-05 | NA | 1.69E-01 | 7 | -158.061 | 330.121 | 1.999 | 0.163 |
| -0.519 | NA | NA | + | NA | NA | 1.82E-01 | 6 | -155.141 | 322.283 | 0 | 0.413 |
| -0.407 | NA | NA | + | NA | -5.00E-02 | 1.83E-01 | 7 | -154.87 | 323.739 | 1.457 | 0.2 |
| -0.556 | 1.29E-09 | NA | + | NA | NA | 1.88E-01 | 7 | -154.872 | 323.744 | 1.461 | 0.199 |
| -0.58 | NA | NA | + | -7.73E-04 | NA | 1.86E-01 | 7 | -154.929 | 323.858 | 1.576 | 0.188 |
| -0.585 | NA | NA | + | NA | NA | 2.07E-01 | 6 | -155.447 | 322.893 | 0 | 0.387 |
| -0.66 | NA | NA | + | -1.05E-03 | NA | 2.12E-01 | 7 | -155.017 | 324.033 | I.14 | 0.219 |
| -0.627 | 1.52E-09 | NA | + | NA | NA | 2.14E-01 | 7 | -155.108 | 324.216 | 1.323 | 0.2 |
| -0.471 | NA | NA | + | NA | -5.25E-02 | 2.08E-01 | 7 | -155.131 | 324.261 | 1.368 | 0.195 |
| -0.446 | NA | NA | + | NA | NA | 1.62E-01 | 6 | -146.668 | 305.337 | 0 | 0.429 |
| -0.477 | 1.07E-09 | NA | + | NA | NA | 1.67E-01 | 7 | -146.443 | 306.887 | I.55 | 0.198 |
| -0.51 | NA | NA | + | -7.84E-04 | NA | 1.66E-01 | 7 | -146.45 | 306.9 | 1.563 | 0.196 |
| -0.375 | NA | NA | + | NA | -3.28E-02 | 1.63E-01 | 7 | -146.554 | 307.108 | 1.771 | 0.177 |
| -0.437 | NA | NA | + | NA | NA | 1.60E-01 | 6 | -143.471 | 298.942 | 0 | 0.408 |
| -0.51 | NA | NA | + | -9.28E-04 | NA | 1.64E-01 | 7 | -143.148 | 300.295 | 1.353 | 0.207 |
| -0.471 | 1.35E-09 | NA | + | NA | NA | 1.65E-01 | 7 | -143.211 | 300.423 | I.48 | 0.194 |
| -0.338 | NA | NA | + | NA | -4.49E-02 | 1.61E-01 | 7 | -143.23 | 300.461 | 1.518 | 0.191 |
| -0.51 | NA | NA | + | NA | NA | 1.79E-01 | 6 | -160.54 | 333.08 | 0 | 0.446 |
| -0.541 | 1.10E-09 | NA | + | NA | NA | 1.85E-01 | 7 | -160.329 | 334.658 | 1.579 | 0.202 |
| -0.55 | NA | NA | + | -5.19E-04 | NA | 1.82E-01 | 7 | -160.452 | 334.905 | 1.825 | 0.179 |
| -0.459 | NA | NA | + | NA | -2.32E-02 | 1.80E-01 | 7 | -160.488 | 334.976 | 1.896 | 0.173 |
| -0.455 | NA | NA | + | NA | NA | 1.69E-01 | 6 | -150.618 | 313.237 | 0 | 0.441 |
| -0.487 | 1.15E-09 | NA | + | NA | NA | 1.74E-01 | 7 | -150.397 | 314.794 | 1.557 | 0.203 |
| -0.5 | NA | NA | + | -5.72E-04 | NA | 1.72E-01 | 7 | -150.502 | 315.005 | 1.768 | 0.182 |
| -0.4 | NA | NA | + | NA | -2.59E-02 | 1.70E-01 | 7 | -150.549 | 315.098 | 1.861 | 0.174 |
| -0.519 | NA | NA | + | NA | NA | 1.84E-01 | 6 | -150.763 | 313.526 | 0 | 0.417 |
| -0.555 | 1.13E-09 | NA | + | NA | NA | 1.92E-01 | 7 | -150.466 | 314.932 | 1.406 | 0.206 |
| -0.411 | NA | NA | + | NA | -4.78E-02 | 1.84E-01 | 7 | -150.505 | 315.011 | 1.485 | 0.198 |
| -0.565 | NA | NA | + | -6.23E-04 | NA | 1.86E-01 | 7 | -150.61 | 315.22 | 1.694 | 0.179 |
| -0.465 | NA | NA | + | NA | NA | 1.73E-01 | 6 | -152.19 | 316.379 | 0 | 0.428 |
| -0.535 | NA | NA | + | -8.63E-04 | NA | 1.77E-01 | 7 | -151.93 | 317.86 | 1.481 | 0.204 |
| -0.494 | 1.15E-09 | NA | + | NA | NA | 1.77E-01 | 7 | -151.993 | 317.987 | 1.608 | 0.192 |
| -0.397 | NA | NA | + | NA | -3.16E-02 | 1.74E-01 | 7 | -152.078 | 318.155 | 1.776 | 0.176 |
| -0.53 | NA | NA | + | NA | NA | 1.87E-01 | 6 | -163.716 | 339.433 | 0 | 0.418 |
| -0.603 | NA | NA | + | -9.43E-04 | NA | 1.90E-01 | 7 | -163.405 | 340.809 | 1.376 | 0.21 |
| -0.566 | 1.46E-09 | NA | + | NA | NA | 1.92E-01 | 7 | -163.468 | 340.935 | 1.503 | 0.197 |
| -0.448 | NA | NA | + | NA | -3.67E-02 | 1.87E-01 | 7 | -163.585 | 341.169 | 1.737 | 0.175 |
| -0.692 | NA | NA | + | NA | NA | 2.27E-01 | 6 | -167.201 | 346.403 | 0 | 0.291 |
| -0.774 | NA | NA | + | -1.26E-03 | NA | 2.32E-01 | 7 | -166.599 | 347.198 | 0.796 | 0.196 |
| -0.532 | NA | NA | + | NA | -7.08E-02 | 2.28E-01 | 7 | -166.631 | 347.261 | 0.859 | 0.19 |
| -0.597 | NA | NA | + | -1.53E-03 | -8.69E-02 | 2.34E-01 | 8 | -165.759 | 347.519 | 1.116 | 0.167 |
| -0.738 | 1.82E-09 | NA | + | NA | NA | 2.34E-01 | 7 | -166.825 | 347.65 | 1.248 | 0.156 |
| -0.556 | NA | NA | + | NA | NA | 1.93E-01 | 6 | -150.956 | 313.912 | 0 | 0.331 |
| -0.393 | NA | NA | + | NA | -7.20E-02 | 1.93E-01 | 7 | -150.338 | 314.676 | 0.763 | 0.226 |
| -0.594 | 1.40E-09 | NA | + | NA | NA | 1.99E-01 | 7 | -150.647 | 315.294 | 1.381 | 0.166 |
| -0.614 | NA | NA | + | -7.53E-04 | NA | 1.96E-01 | 7 | -150.729 | 315.458 | 1.546 | 0.153 |
| -0.446 | NA | NA | + | -1.03E-03 | -8.35E-02 | 1.97E-01 | 8 | -149.925 | 315.851 | 1.938 | 0.125 |
| -0.498 | NA | NA | + | NA | NA | 1.78E-01 | 6 | -151.234 | 314.467 | 0 | 0.408 |
| -0.539 | 1.05E-09 | NA | + | NA | NA | 1.87E-01 | 7 | -150.911 | 315.821 | 1.354 | 0.207 |
| -0.39 | NA | NA | + | NA | -4.95E-02 | 1.79E-01 | 7 | -150.952 | 315.905 | 1.438 | 0.199 |
| -0.559 | NA | NA | + | -7.81E-04 | NA | 1.82E-01 | 7 | -151.024 | 316.048 | 1.581 | 0.185 |
| -0.468 | NA | NA | + | NA | NA | 1.71E-01 | 6 | -143.827 | 299.653 | 0 | 0.391 |
| -0.333 | NA | NA | + | NA | -6.07E-02 | 1.72E-01 | 7 | -143.401 | 300.802 | 1.148 | 0.22 |
| -0.543 | NA | NA | + | -8.85E-04 | NA | 1.77E-01 | 7 | -143.51 | 301.019 | 1.366 | 0.198 |
| -0.505 | 1.25E-09 | NA | + | NA | NA | 1.77E-01 | 7 | -143.546 | 301.092 | 1.439 | 0.191 |
| -0.447 | NA | NA | + | NA | NA | 1.60E-01 | 6 | -161.556 | 335.112 | 0 | 0.459 |
| -0.476 | 9.85E-10 | NA | + | NA | NA | 1.66E-01 | 7 | -161.377 | 336.754 | 1.642 | 0.202 |
| -0.457 | NA | NA | + | -1.19E-04 | NA | 1.61E-01 | 7 | -161.552 | 337.104 | 1.991 | 0.17 |
| -0.445 | NA | NA | + | NA | -8.74E-04 | 1.60E-01 | 7 | -161.556 | 337.112 | 2 | 0.169 |
| -0.425 | NA | NA | + | NA | NA | 1.60E-01 | 6 | -140.546 | 293.092 | 0 | 0.433 |
| -0.455 | 9.44E-10 | NA | + | NA | NA | 1.66E-01 | 7 | -140.341 | 294.682 | I.59 | 0.196 |
| -0.344 | NA | NA | + | NA | -3.76E-02 | 1.62E-01 | 7 | -140.383 | 294.766 | 1.674 | 0.187 |
| -0.474 | NA | NA | + | -6.24E-04 | NA | 1.63E-01 | 7 | -140.402 | 294.803 | 1.711 | 0.184 |
| -0.392 | NA | NA | + | NA | NA | 1.53E-01 | 6 | -143.367 | 298.734 | 0 | 0.382 |
| -0.419 | 1.13E-09 | NA | + | NA | NA | 1.57E-01 | 7 | -143.193 | 300.385 | 1.651 | 0.168 |
| -0.335 | NA | NA | + | NA | -2.63E-02 | 1.54E-01 | 7 | -143.291 | 300.582 | 1.848 | 0.152 |
| -0.329 | NA | NA | NA | NA | NA | 1.89E-01 | 3 | -147.307 | 300.614 | 1.879 | 0.149 |
| -0.427 | NA | NA | + | -4.10E-04 | NA | 1.55E-01 | 7 | -143.31 | 300.62 | 1.886 | 0.149 |
| -0.622 | NA | NA | + | -1.91E-03 | -1.23E-01 | 2.65E-01 | 8 | -164.102 | 344.205 | 0 | 0.341 |
| -0.688 | 1.69E-09 | NA | + | -1.90E-03 | -1.16E-01 | 2.75E-01 | 9 | -163.62 | 345.24 | 1.036 | 0.203 |
| -0.561 | NA | NA | + | NA | -1.04E-01 | 2.63E-01 | 7 | -165.681 | 345.363 | 1.158 | 0.191 |
| -0.889 | NA | NA | + | -1.55E-03 | NA | 2.71E-01 | 7 | -166.021 | 346.042 | 1.838 | 0.136 |
| -0.803 | NA | NA | + | NA | NA | 2.69E-01 | 6 | -167.069 | 346.138 | 1.933 | 0.13 |
| -0.536 | NA | NA | + | NA | NA | 1.86E-01 | 6 | -151.189 | 314.378 | 0 | 0.266 |
| -0.649 | NA | NA | + | -1.46E-03 | NA | 1.93E-01 | 7 | -150.406 | 314.813 | 0.434 | 0.214 |
| -0.498 | NA | NA | + | -1.69E-03 | -7.62E-02 | 1.96E-01 | 8 | -149.792 | 315.584 | 1.206 | 0.146 |
| -0.405 | NA | NA | + | NA | -5.94E-02 | 1.87E-01 | 7 | -150.809 | 315.619 | I.24 | 0.143 |
| -0.572 | 1.29E-09 | NA | + | NA | NA | 1.92E-01 | 7 | -150.936 | 315.872 | 1.494 | 0.126 |
| -0.687 | 1.34E-09 | NA | + | -1.48E-03 | NA | 2.00E-01 | 8 | -150.131 | 316.263 | 1.885 | 0.104 |
| -0.453 | NA | NA | + | -1.60E-03 | -1.10E-01 | 2.11E-01 | 8 | -152.22 | 320.44 | 0 | 0.208 |
| -0.37 | NA | NA | + | NA | -9.49E-02 | 2.02E-01 | 7 | -153.285 | 320.571 | 0.131 | 0.195 |
| -0.585 | NA | NA | + | NA | NA | 2.02E-01 | 6 | -154.39 | 320.779 | 0.339 | 0.176 |
| -0.68 | NA | NA | + | -1.29E-03 | NA | 2.09E-01 | 7 | -153.688 | 321.376 | 0.936 | 0.13 |
| -0.498 | 1.25E-09 | NA | + | -1.60E-03 | -1.06E-01 | 2.18E-01 | 9 | -151.934 | 321.867 | 1.427 | 0.102 |
| -0.414 | 1.24E-09 | NA | + | NA | -9.09E-02 | 2.09E-01 | 8 | -153.006 | 322.013 | 1.573 | 0.095 |
| -0.626 | 1.44E-09 | NA | + | NA | NA | 2.10E-01 | 7 | -154.015 | 322.03 | I.59 | 0.094 |
| -0.469 | NA | NA | + | NA | NA | 1.71E-01 | 6 | -149.301 | 310.602 | 0 | 0.436 |
| -0.504 | 1.10E-09 | NA | + | NA | NA | 1.77E-01 | 7 | -149.057 | 312.114 | 1.512 | 0.205 |
| -0.386 | NA | NA | + | NA | -3.74E-02 | 1.72E-01 | 7 | -149.148 | 312.295 | 1.694 | 0.187 |
| -0.503 | NA | NA | + | -4.47E-04 | NA | 1.73E-01 | 7 | -149.23 | 312.459 | 1.857 | 0.172 |
| -0.605 | NA | NA | + | NA | NA | 2.05E-01 | 6 | -157.289 | 326.578 | 0 | 0.382 |
| -0.683 | NA | NA | + | -1.08E-03 | NA | 2.10E-01 | 7 | -156.854 | 327.709 | 1.131 | 0.217 |
| -0.656 | 1.82E-09 | NA | + | NA | NA | 2.13E-01 | 7 | -156.858 | 327.715 | 1.137 | 0.216 |
| -0.492 | NA | NA | + | NA | -4.99E-02 | 2.05E-01 | 7 | -157.017 | 328.033 | 1.456 | 0.185 |
| -0.668 | NA | NA | + | NA | NA | 2.27E-01 | 6 | -160.045 | 332.089 | 0 | 0.204 |
| -0.776 | NA | NA | + | -1.56E-03 | NA | 2.32E-01 | 7 | -159.098 | 332.195 | 0.106 | 0.193 |
| -0.603 | NA | NA | + | -1.81E-03 | -8.33E-02 | 2.32E-01 | 8 | -158.286 | 332.572 | 0.483 | 0.16 |
| -0.52 | NA | NA | + | NA | -6.50E-02 | 2.26E-01 | 7 | -159.541 | 333.083 | 0.994 | 0.124 |
| -0.717 | 1.70E-09 | NA | + | NA | NA | 2.35E-01 | 7 | -159.598 | 333.197 | 1.107 | 0.117 |
| -0.827 | 1.75E-09 | NA | + | -1.58E-03 | NA | 2.41E-01 | 8 | -158.618 | 333.236 | 1.147 | 0.115 |
| -0.66 | 1.56E-09 | NA | + | -1.82E-03 | -7.80E-02 | 2.40E-01 | 9 | -157.907 | 333.815 | 1.725 | 0.086 |
| -0.595 | NA | NA | + | NA | NA | 2.07E-01 | 6 | -160.128 | 332.256 | 0 | 0.403 |
| -0.455 | NA | NA | + | NA | -6.08E-02 | 2.05E-01 | 7 | -159.713 | 333.425 | I.17 | 0.225 |
| -0.637 | 1.66E-09 | NA | + | NA | NA | 2.13E-01 | 7 | -159.817 | 333.634 | 1.379 | 0.202 |
| -0.638 | NA | NA | + | -6.01E-04 | NA | 2.10E-01 | 7 | -159.995 | 333.989 | 1.734 | 0.17 |
| -0.584 | NA | NA | + | NA | NA | 1.98E-01 | 6 | -156.722 | 325.443 | 0 | 0.321 |
| -0.669 | NA | NA | + | -1.22E-03 | NA | 2.02E-01 | 7 | -156.138 | 326.276 | 0.833 | 0.212 |
| -0.628 | 1.59E-09 | NA | + | NA | NA | 2.05E-01 | 7 | -156.349 | 326.698 | 1.255 | 0.171 |
| -0.464 | NA | NA | + | NA | -5.36E-02 | 1.99E-01 | 7 | -156.395 | 326.79 | 1.347 | 0.164 |
| -0.53 | NA | NA | + | -1.44E-03 | -6.94E-02 | 2.03E-01 | 8 | -155.605 | 327.211 | 1.768 | 0.133 |
| -0.541 | NA | NA | + | NA | NA | 1.90E-01 | 6 | -157.491 | 326.983 | 0 | 0.411 |
| -0.582 | 1.55E-09 | NA | + | NA | NA | 1.97E-01 | 7 | -157.172 | 328.343 | I.36 | 0.208 |
| -0.425 | NA | NA | + | NA | -5.15E-02 | 1.91E-01 | 7 | -157.19 | 328.381 | 1.398 | 0.204 |
| -0.59 | NA | NA | + | -6.49E-04 | NA | 1.93E-01 | 7 | -157.338 | 328.676 | 1.693 | 0.176 |
| -0.643 | NA | NA | + | NA | NA | 2.18E-01 | 6 | -157.134 | 326.267 | 0 | 0.217 |
| -0.726 | NA | NA | + | -1.33E-03 | NA | 2.20E-01 | 7 | -156.356 | 326.712 | 0.445 | 0.174 |
| -0.55 | NA | NA | + | -1.60E-03 | -8.86E-02 | 2.21E-01 | 8 | -155.445 | 326.89 | 0.622 | 0.159 |
| -0.49 | NA | NA | + | NA | -7.02E-02 | 2.18E-01 | 7 | -156.548 | 327.096 | 0.829 | 0.144 |
| -0.69 | 1.59E-09 | NA | + | NA | NA | 2.26E-01 | 7 | -156.705 | 327.409 | 1.142 | 0.123 |
| -0.775 | 1.62E-09 | NA | + | -1.34E-03 | NA | 2.28E-01 | 8 | -155.911 | 327.822 | 1.555 | 0.1 |
| -0.602 | 1.42E-09 | NA | + | -1.59E-03 | -8.36E-02 | 2.28E-01 | 9 | -155.102 | 328.205 | 1.937 | 0.083 |
| -0.476 | NA | NA | + | NA | NA | 1.71E-01 | 6 | -145.464 | 302.928 | 0 | 0.415 |
| -0.555 | NA | NA | + | -9.97E-04 | NA | 1.77E-01 | 7 | -145.099 | 304.198 | 1.269 | 0.22 |
| -0.505 | 1.02E-09 | NA | + | NA | NA | 1.77E-01 | 7 | -145.259 | 304.518 | I.59 | 0.187 |
| -0.394 | NA | NA | + | NA | -3.71E-02 | 1.72E-01 | 7 | -145.308 | 304.617 | 1.688 | 0.178 |
| -0.795 | NA | NA | + | -1.67E-03 | NA | 2.32E-01 | 7 | -157.381 | 328.763 | 0 | 0.254 |
| -0.681 | NA | NA | + | NA | NA | 2.26E-01 | 6 | -158.465 | 328.93 | 0.167 | 0.233 |
| -0.663 | NA | NA | + | -1.87E-03 | -6.40E-02 | 2.32E-01 | 8 | -156.946 | 329.891 | 1.128 | 0.144 |
| -0.842 | 1.40E-09 | NA | + | -1.70E-03 | NA | 2.41E-01 | 8 | -156.974 | 329.949 | 1.186 | 0.14 |
| -0.724 | 1.33E-09 | NA | + | NA | NA | 2.34E-01 | 7 | -158.104 | 330.207 | 1.444 | 0.123 |
| -0.583 | NA | NA | + | NA | -4.34E-02 | 2.25E-01 | 7 | -158.26 | 330.521 | 1.758 | 0.105 |
| -0.531 | NA | NA | + | NA | NA | 1.88E-01 | 6 | -142.032 | 296.064 | 0 | 0.2 |
| -0.445 | NA | NA | + | -1.58E-03 | -9.02E-02 | 1.96E-01 | 8 | -140.28 | 296.559 | 0.495 | 0.156 |
| -0.36 | NA | NA | + | NA | -7.43E-02 | 1.87E-01 | 7 | -141.311 | 296.621 | 0.558 | 0.151 |
| -0.63 | NA | NA | + | -1.30E-03 | NA | 1.95E-01 | 7 | -141.32 | 296.64 | 0.577 | 0.15 |
| -0.569 | 1.23E-09 | NA | + | NA | NA | 1.96E-01 | 7 | -141.669 | 297.339 | 1.275 | 0.106 |
| -0.675 | 1.30E-09 | NA | + | -1.34E-03 | NA | 2.04E-01 | 8 | -140.909 | 297.818 | 1.754 | 0.083 |
| -0.491 | 1.17E-09 | NA | + | -1.60E-03 | -8.67E-02 | 2.03E-01 | 9 | -139.946 | 297.893 | 1.829 | 0.08 |
| -0.403 | 1.11E-09 | NA | + | NA | -7.08E-02 | 1.94E-01 | 8 | -141.016 | 298.032 | 1.968 | 0.075 |
| -0.601 | NA | NA | + | NA | NA | 2.07E-01 | 6 | -152.87 | 317.74 | 0 | 0.254 |
| -0.699 | NA | NA | + | -1.45E-03 | NA | 2.12E-01 | 7 | -151.993 | 317.986 | 0.247 | 0.225 |
| -0.556 | NA | NA | + | -1.68E-03 | -7.15E-02 | 2.12E-01 | 8 | -151.425 | 318.849 | 01.XI | 0.146 |
| -0.642 | 1.31E-09 | NA | + | NA | NA | 2.15E-01 | 7 | -152.539 | 319.079 | 1.339 | 0.13 |
| -0.486 | NA | NA | + | NA | -5.17E-02 | 2.07E-01 | 7 | -152.566 | 319.131 | 1.391 | 0.127 |
| -0.742 | 1.35E-09 | NA | + | -1.47E-03 | NA | 2.20E-01 | 8 | -151.639 | 319.279 | 1.539 | 0.118 |
| -0.451 | NA | NA | + | NA | NA | 1.64E-01 | 6 | -149.242 | 310.484 | 0 | 0.439 |
| -0.482 | 8.74E-10 | NA | + | NA | NA | 1.71E-01 | 7 | -149.019 | 312.038 | 1.554 | 0.202 |
| -0.497 | NA | NA | + | -5.79E-04 | NA | 1.68E-01 | 7 | -149.123 | 312.246 | 1.761 | 0.182 |
| -0.387 | NA | NA | + | NA | -2.94E-02 | 1.66E-01 | 7 | -149.151 | 312.303 | 1.819 | 0.177 |
| -0.517 | NA | NA | + | NA | NA | 1.83E-01 | 6 | -152.995 | 317.99 | 0 | 0.407 |
| -0.557 | 1.36E-09 | NA | + | NA | NA | 1.90E-01 | 7 | -152.676 | 319.352 | 1.361 | 0.206 |
| -0.403 | NA | NA | + | NA | -5.19E-02 | 1.85E-01 | 7 | -152.696 | 319.392 | 1.401 | 0.202 |
| -0.576 | NA | NA | + | -7.68E-04 | NA | 1.87E-01 | 7 | -152.786 | 319.571 | 1.581 | 0.185 |
| -0.527 | NA | NA | + | NA | NA | 1.81E-01 | 6 | -159.234 | 330.467 | 0 | 0.417 |
| -0.597 | NA | NA | + | -9.27E-04 | NA | 1.85E-01 | 7 | -158.93 | 331.861 | 1.394 | 0.208 |
| -0.568 | 1.53E-09 | NA | + | NA | NA | 1.87E-01 | 7 | -158.969 | 331.937 | I.47 | 0.2 |
| -0.448 | NA | NA | + | NA | -3.57E-02 | 1.82E-01 | 7 | -159.103 | 332.206 | 1.739 | 0.175 |
| -0.581 | NA | NA | + | -1.99E-03 | -1.42E-01 | 2.54E-01 | 8 | -160.632 | 337.265 | 0 | 0.504 |
| -0.645 | 1.85E-09 | NA | + | -1.95E-03 | -1.34E-01 | 2.62E-01 | 9 | -160.183 | 338.366 | 1.101 | 0.291 |
| -0.519 | NA | NA | + | NA | -1.19E-01 | 2.54E-01 | 7 | -162.533 | 339.065 | 1.801 | 0.205 |
| -0.402 | NA | NA | + | NA | NA | 1.50E-01 | 6 | -146.642 | 305.284 | 0 | 0.391 |
| -0.43 | 8.10E-10 | NA | + | NA | NA | 1.56E-01 | 7 | -146.47 | 306.941 | 1.657 | 0.171 |
| -0.427 | NA | NA | + | -3.16E-04 | NA | 1.52E-01 | 7 | -146.608 | 307.217 | 1.933 | 0.149 |
| -0.42 | NA | NA | + | NA | 8.42E-03 | 1.50E-01 | 7 | -146.635 | 307.27 | 1.986 | 0.145 |
| -0.32 | NA | NA | NA | NA | NA | 1.86E-01 | 3 | -150.64 | 307.279 | 1.996 | 0.144 |
| -0.569 | NA | NA | + | NA | NA | 1.97E-01 | 6 | -163.028 | 338.055 | 0 | 0.415 |
| -0.615 | 1.66E-09 | NA | + | NA | NA | 2.04E-01 | 7 | -162.691 | 339.382 | 1.327 | 0.214 |
| -0.632 | NA | NA | + | -8.60E-04 | NA | 2.00E-01 | 7 | -162.772 | 339.545 | I.49 | 0.197 |
| -0.49 | NA | NA | + | NA | -3.58E-02 | 1.97E-01 | 7 | -162.899 | 339.799 | 1.743 | 0.174 |
| -0.527 | NA | NA | + | NA | NA | 1.84E-01 | 6 | -149.839 | 311.678 | 0 | 0.402 |
| -0.405 | NA | NA | + | NA | -5.48E-02 | 1.85E-01 | 7 | -149.495 | 312.989 | 1.311 | 0.209 |
| -0.563 | 1.02E-09 | NA | + | NA | NA | 1.92E-01 | 7 | -149.551 | 313.102 | 1.424 | 0.197 |
| -0.59 | NA | NA | + | -8.31E-04 | NA | 1.88E-01 | 7 | -149.576 | 313.152 | 1.474 | 0.192 |
| -0.549 | NA | NA | + | NA | NA | 1.89E-01 | 6 | -151.408 | 314.816 | 0 | 0.39 |
| -0.646 | NA | NA | + | -1.31E-03 | NA | 1.93E-01 | 7 | -150.773 | 315.546 | 0.729 | 0.271 |
| -0.583 | 1.19E-09 | NA | + | NA | NA | 1.94E-01 | 7 | -151.163 | 316.326 | I.51 | 0.183 |
| -0.485 | NA | NA | + | NA | -2.88E-02 | 1.90E-01 | 7 | -151.32 | 316.64 | 1.824 | 0.157 |
| -0.449 | NA | NA | + | NA | NA | 1.65E-01 | 6 | -158.948 | 329.896 | 0 | 0.449 |
| -0.486 | 1.35E-09 | NA | + | NA | NA | 1.71E-01 | 7 | -158.699 | 331.399 | 1.503 | 0.212 |
| -0.402 | NA | NA | + | NA | -2.16E-02 | 1.66E-01 | 7 | -158.902 | 331.803 | 1.908 | 0.173 |
| -0.461 | NA | NA | + | -1.46E-04 | NA | 1.66E-01 | 7 | -158.941 | 331.882 | 1.986 | 0.166 |
| -0.627 | NA | NA | + | -2.31E-03 | -1.65E-01 | 2.88E-01 | 8 | -164.123 | 344.246 | 0 | 0.561 |
| -0.707 | 1.67E-09 | NA | + | -2.31E-03 | -1.57E-01 | 3.02E-01 | 9 | -163.369 | 344.738 | 0.492 | 0.439 |
| -0.582 | NA | NA | + | NA | NA | 1.96E-01 | 6 | -158.035 | 328.069 | 0 | 0.401 |
| -0.624 | 1.50E-09 | NA | + | NA | NA | 2.03E-01 | 7 | -157.706 | 329.413 | 1.344 | 0.205 |
| -0.646 | NA | NA | + | -9.20E-04 | NA | 1.99E-01 | 7 | -157.709 | 329.419 | 1.349 | 0.204 |
| -0.471 | NA | NA | + | NA | -4.91E-02 | 1.96E-01 | 7 | -157.787 | 329.573 | 1.504 | 0.189 |
| -0.587 | NA | NA | + | NA | NA | 1.97E-01 | 6 | -153.409 | 318.818 | 0 | 0.18 |
| -0.51 | NA | NA | + | -1.84E-03 | -9.35E-02 | 2.06E-01 | 8 | -151.486 | 318.973 | 0.155 | 0.166 |
| -0.703 | NA | NA | + | -1.55E-03 | NA | 2.05E-01 | 7 | -152.489 | 318.978 | 0.16 | 0.166 |
| -0.416 | NA | NA | + | NA | -7.41E-02 | 1.97E-01 | 7 | -152.766 | 319.531 | 0.714 | 0.126 |
| -0.632 | 1.41E-09 | NA | + | NA | NA | 2.06E-01 | 7 | -152.966 | 319.932 | 1.114 | 0.103 |
| -0.753 | 1.46E-09 | NA | + | -1.58E-03 | NA | 2.14E-01 | 8 | -152.004 | 320.007 | I.19 | 0.099 |
| -0.564 | 1.34E-09 | NA | + | -1.86E-03 | -8.98E-02 | 2.14E-01 | 9 | -151.077 | 320.154 | 1.337 | 0.092 |
| -0.467 | 1.30E-09 | NA | + | NA | -7.04E-02 | 2.05E-01 | 8 | -152.386 | 320.772 | 1.954 | 0.068 |
| -0.418 | NA | NA | + | NA | NA | 1.58E-01 | 6 | -147.388 | 306.776 | 0 | 0.45 |
| -0.474 | NA | NA | + | -6.78E-04 | NA | 1.62E-01 | 7 | -147.236 | 308.472 | 1.696 | 0.193 |
| -0.443 | 9.06E-10 | NA | + | NA | NA | 1.63E-01 | 7 | -147.243 | 308.487 | 1.711 | 0.191 |
| -0.437 | NA | NA | + | NA | 9.03E-03 | 1.58E-01 | 7 | -147.38 | 308.759 | 1.983 | 0.167 |
| -0.523 | NA | NA | + | -1.71E-03 | -1.11E-01 | 2.24E-01 | 8 | -150.848 | 317.695 | 0 | 0.267 |
| -0.438 | NA | NA | + | NA | -9.45E-02 | 2.17E-01 | 7 | -152.089 | 318.178 | 0.483 | 0.21 |
| -0.652 | NA | NA | + | NA | NA | 2.17E-01 | 6 | -153.348 | 318.697 | 1.001 | 0.162 |
| -0.75 | NA | NA | + | -1.35E-03 | NA | 2.23E-01 | 7 | -152.564 | 319.129 | 1.433 | 0.131 |
| -0.569 | 1.38E-09 | NA | + | -1.69E-03 | -1.07E-01 | 2.30E-01 | 9 | -150.585 | 319.171 | 1.475 | 0.128 |
| -0.487 | 1.44E-09 | NA | + | NA | -8.99E-02 | 2.23E-01 | 8 | -151.807 | 319.614 | 1.918 | 0.102 |
| -0.392 | NA | NA | + | NA | NA | 1.49E-01 | 6 | -147.421 | 306.842 | 0 | 0.307 |
| -0.316 | NA | NA | NA | NA | NA | 1.84E-01 | 3 | -150.976 | 307.952 | 01.XI | 0.176 |
| -0.24 | NA | NA | NA | 1.16E-03 | NA | 1.61E-01 | 4 | -150.143 | 308.287 | 1.445 | 0.149 |
| -0.423 | 1.07E-09 | NA | + | NA | NA | 1.53E-01 | 7 | -147.225 | 308.449 | 1.607 | 0.137 |
| -0.363 | NA | NA | + | NA | -1.33E-02 | 1.49E-01 | 7 | -147.403 | 308.806 | 1.964 | 0.115 |
| -0.412 | NA | NA | + | -2.29E-04 | NA | 1.50E-01 | 7 | -147.404 | 308.808 | 1.966 | 0.115 |
| -0.454 | NA | NA | + | NA | NA | 1.63E-01 | 6 | -152.638 | 317.276 | 0 | 0.44 |
| -0.492 | 1.28E-09 | NA | + | NA | NA | 1.69E-01 | 7 | -152.361 | 318.722 | 1.446 | 0.214 |
| -0.389 | NA | NA | + | NA | -2.88E-02 | 1.63E-01 | 7 | -152.553 | 319.105 | 1.829 | 0.176 |
| -0.484 | NA | NA | + | -3.63E-04 | NA | 1.65E-01 | 7 | -152.593 | 319.186 | I.91 | 0.169 |
| -0.402 | NA | NA | + | NA | NA | 1.50E-01 | 6 | -142.943 | 297.885 | 0 | 0.424 |
| -0.29 | NA | NA | + | NA | -4.96E-02 | 1.50E-01 | 7 | -142.65 | 299.3 | 1.415 | 0.209 |
| -0.436 | 1.28E-09 | NA | + | NA | NA | 1.55E-01 | 7 | -142.701 | 299.401 | 1.516 | 0.199 |
| -0.439 | NA | NA | + | -4.56E-04 | NA | 1.52E-01 | 7 | -142.865 | 299.729 | 1.844 | 0.169 |
| -0.562 | NA | NA | + | NA | NA | 1.97E-01 | 6 | -147.299 | 306.598 | 0 | 0.249 |
| -0.672 | NA | NA | + | -1.45E-03 | NA | 2.05E-01 | 7 | -146.477 | 306.954 | 0.356 | 0.208 |
| -0.519 | NA | NA | + | -1.70E-03 | -7.89E-02 | 2.08E-01 | 8 | -145.74 | 307.481 | 0.883 | 0.16 |
| -0.429 | NA | NA | + | NA | -6.15E-02 | 1.99E-01 | 7 | -146.843 | 307.685 | 1.088 | 0.145 |
| -0.602 | 1.39E-09 | NA | + | NA | NA | 2.04E-01 | 7 | -146.961 | 307.922 | 1.324 | 0.128 |
| -0.713 | 1.42E-09 | NA | + | -1.46E-03 | NA | 2.11E-01 | 8 | -146.12 | 308.24 | 1.642 | 0.11 |
| -0.486 | NA | NA | + | NA | NA | 1.79E-01 | 6 | -153.351 | 318.703 | 0 | 0.434 |
| -0.522 | 1.43E-09 | NA | + | NA | NA | 1.84E-01 | 7 | -153.101 | 320.203 | 01.V | 0.205 |
| -0.389 | NA | NA | + | NA | -4.41E-02 | 1.80E-01 | 7 | -153.141 | 320.281 | 1.579 | 0.197 |
| -0.511 | NA | NA | + | -3.07E-04 | NA | 1.81E-01 | 7 | -153.319 | 320.638 | 1.935 | 0.165 |
| -0.525 | NA | NA | + | NA | NA | 1.84E-01 | 6 | -156.228 | 324.455 | 0 | 0.404 |
| -0.599 | NA | NA | + | -9.86E-04 | NA | 1.88E-01 | 7 | -155.863 | 325.726 | 1.271 | 0.214 |
| -0.559 | 1.28E-09 | NA | + | NA | NA | 1.90E-01 | 7 | -155.973 | 325.947 | 1.491 | 0.192 |
| -0.421 | NA | NA | + | NA | -4.83E-02 | 1.86E-01 | 7 | -155.976 | 325.952 | 1.497 | 0.191 |
| -0.546 | NA | NA | + | NA | NA | 1.90E-01 | 6 | -162.01 | 336.02 | 0 | 0.439 |
| -0.584 | 1.15E-09 | NA | + | NA | NA | 1.98E-01 | 7 | -161.734 | 337.468 | 1.449 | 0.213 |
| -0.466 | NA | NA | + | NA | -3.64E-02 | 1.90E-01 | 7 | -161.875 | 337.75 | I.73 | 0.185 |
| -0.561 | NA | NA | + | -2.02E-04 | NA | 1.91E-01 | 7 | -161.997 | 337.993 | 1.974 | 0.164 |
| -0.588 | NA | NA | + | -2.04E-03 | -1.47E-01 | 2.62E-01 | 8 | -159.045 | 334.09 | 0 | 0.63 |
| -0.653 | 1.85E-09 | NA | + | -2.00E-03 | -1.39E-01 | 2.70E-01 | 9 | -158.576 | 335.153 | 1.063 | 0.37 |
| -0.417 | NA | NA | + | NA | NA | 1.53E-01 | 6 | -146.349 | 304.699 | 0 | 0.434 |
| -0.449 | 1.20E-09 | NA | + | NA | NA | 1.58E-01 | 7 | -146.125 | 306.249 | 1.551 | 0.2 |
| -0.469 | NA | NA | + | -6.34E-04 | NA | 1.56E-01 | 7 | -146.21 | 306.42 | 1.721 | 0.184 |
| -0.342 | NA | NA | + | NA | -3.34E-02 | 1.53E-01 | 7 | -146.216 | 306.432 | 1.733 | 0.182 |
| -0.542 | NA | NA | + | NA | NA | 1.91E-01 | 6 | -160.525 | 333.051 | 0 | 0.426 |
| -0.584 | 1.53E-09 | NA | + | NA | NA | 1.98E-01 | 7 | -160.202 | 334.403 | 1.353 | 0.217 |
| -0.588 | NA | NA | + | -6.14E-04 | NA | 1.94E-01 | 7 | -160.393 | 334.785 | 1.735 | 0.179 |
| -0.464 | NA | NA | + | NA | -3.49E-02 | 1.92E-01 | 7 | -160.397 | 334.793 | 1.743 | 0.178 |
| -0.361 | NA | NA | + | NA | NA | 1.41E-01 | 6 | -141.159 | 294.319 | 0 | 0.295 |
| -0.224 | NA | NA | NA | 1.23E-03 | NA | 1.54E-01 | 4 | -143.642 | 295.284 | 0.966 | 0.182 |
| -0.312 | NA | NA | NA | NA | NA | 1.79E-01 | 3 | -144.672 | 295.344 | 1.025 | 0.177 |
| -0.389 | 9.94E-10 | NA | + | NA | NA | 1.46E-01 | 7 | -140.995 | 295.99 | 1.671 | 0.128 |
| -0.345 | NA | NA | + | NA | -7.05E-03 | 1.42E-01 | 7 | -141.154 | 296.308 | 1.989 | 0.109 |
| -0.367 | NA | NA | + | -7.05E-05 | NA | 1.42E-01 | 7 | -141.158 | 296.315 | 1.997 | 0.109 |
| -0.525 | NA | NA | + | NA | NA | 1.92E-01 | 6 | -145.881 | 303.762 | 0 | 0.307 |
| -0.383 | NA | NA | + | NA | -6.52E-02 | 1.93E-01 | 7 | -145.351 | 304.702 | 0.941 | 0.192 |
| -0.608 | NA | NA | + | -1.11E-03 | NA | 1.97E-01 | 7 | -145.363 | 304.727 | 0.965 | 0.189 |
| -0.565 | 1.44E-09 | NA | + | NA | NA | 1.98E-01 | 7 | -145.52 | 305.04 | 1.278 | 0.162 |
| -0.453 | NA | NA | + | -1.36E-03 | -7.99E-02 | 2.00E-01 | 8 | -144.59 | 305.179 | 1.418 | 0.151 |
| -0.445 | NA | NA | + | NA | NA | 1.62E-01 | 6 | -144.355 | 300.71 | 0 | 0.422 |
| -0.342 | NA | NA | + | NA | -4.64E-02 | 1.63E-01 | 7 | -144.104 | 302.207 | 1.497 | 0.2 |
| -0.476 | 1.22E-09 | NA | + | NA | NA | 1.66E-01 | 7 | -144.151 | 302.302 | 1.593 | 0.19 |
| -0.504 | NA | NA | + | -7.13E-04 | NA | 1.66E-01 | 7 | -144.161 | 302.322 | 1.612 | 0.188 |
| -0.363 | NA | NA | + | NA | NA | 1.39E-01 | 6 | -146.009 | 304.019 | 0 | 0.337 |
| -0.391 | 9.64E-10 | NA | + | NA | NA | 1.43E-01 | 7 | -145.854 | 305.708 | 1.689 | 0.145 |
| -0.219 | NA | NA | NA | 1.25E-03 | NA | 1.55E-01 | 4 | -148.936 | 305.872 | 1.853 | 0.133 |
| -0.3 | NA | NA | NA | NA | NA | 1.79E-01 | 3 | -149.942 | 305.883 | 1.865 | 0.133 |
| -0.39 | NA | NA | + | -3.17E-04 | NA | 1.40E-01 | 7 | -145.976 | 305.953 | 1.934 | 0.128 |
| -0.364 | NA | NA | + | NA | 2.29E-04 | 1.39E-01 | 7 | -146.009 | 306.019 | 2 | 0.124 |
| -0.449 | NA | NA | + | NA | NA | 1.64E-01 | 6 | -144.955 | 301.909 | 0 | 0.428 |
| -0.508 | NA | NA | + | -7.72E-04 | NA | 1.67E-01 | 7 | -144.729 | 303.458 | 1.549 | 0.197 |
| -0.479 | 9.41E-10 | NA | + | NA | NA | 1.70E-01 | 7 | -144.747 | 303.495 | 1.585 | 0.194 |
| -0.369 | NA | NA | + | NA | -3.61E-02 | 1.64E-01 | 7 | -144.809 | 303.619 | 1.709 | 0.182 |
| -0.511 | NA | NA | + | NA | NA | 1.82E-01 | 6 | -155.548 | 323.096 | 0 | 0.437 |
| -0.546 | 1.33E-09 | NA | + | NA | NA | 1.87E-01 | 7 | -155.324 | 324.648 | 1.552 | 0.201 |
| -0.558 | NA | NA | + | -6.17E-04 | NA | 1.85E-01 | 7 | -155.417 | 324.834 | 1.739 | 0.183 |
| -0.443 | NA | NA | + | NA | -3.10E-02 | 1.82E-01 | 7 | -155.446 | 324.892 | 1.796 | 0.178 |
| -0.524 | NA | NA | + | NA | NA | 1.82E-01 | 6 | -160.561 | 333.121 | 0 | 0.443 |
| -0.559 | 1.16E-09 | NA | + | NA | NA | 1.89E-01 | 7 | -160.341 | 334.682 | 1.561 | 0.203 |
| -0.573 | NA | NA | + | -6.40E-04 | NA | 1.85E-01 | 7 | -160.425 | 334.85 | 1.729 | 0.187 |
| -0.484 | NA | NA | + | NA | -1.73E-02 | 1.82E-01 | 7 | -160.531 | 335.063 | 1.942 | 0.168 |
| -0.211 | NA | NA | NA | 1.45E-03 | NA | 1.51E-01 | 4 | -159.452 | 326.905 | 0 | 0.287 |
| -0.312 | NA | NA | NA | NA | NA | 1.81E-01 | 3 | -160.571 | 327.142 | 0.237 | 0.255 |
| -0.36 | NA | NA | + | NA | NA | 1.43E-01 | 6 | -157.768 | 327.535 | 0.631 | 0.21 |
| -0.301 | NA | NA | NA | 1.65E-03 | 5.13E-02 | 1.48E-01 | 5 | -159.237 | 328.474 | 1.569 | 0.131 |
| -0.236 | 8.30E-10 | NA | NA | 1.44E-03 | NA | 1.55E-01 | 5 | -159.355 | 328.71 | 1.805 | 0.117 |
| -0.326 | NA | NA | + | NA | NA | 1.32E-01 | 6 | -137.788 | 287.575 | 0 | 0.294 |
| -0.254 | NA | NA | NA | NA | NA | 1.65E-01 | 3 | -141.187 | 288.374 | 0.799 | 0.197 |
| -0.18 | NA | NA | NA | 1.15E-03 | NA | 1.42E-01 | 4 | -140.337 | 288.674 | 1.099 | 0.17 |
| -0.347 | 7.84E-10 | NA | + | NA | NA | 1.36E-01 | 7 | -137.692 | 289.384 | 1.808 | 0.119 |
| -0.299 | NA | NA | + | NA | -1.28E-02 | 1.33E-01 | 7 | -137.77 | 289.54 | 1.965 | 0.11 |
| -0.342 | NA | NA | + | -1.79E-04 | NA | 1.34E-01 | 7 | -137.777 | 289.553 | 1.978 | 0.109 |
| -0.435 | NA | NA | + | NA | NA | 1.54E-01 | 6 | -163.852 | 339.704 | 0 | 0.39 |
| -0.469 | 1.09E-09 | NA | + | NA | NA | 1.60E-01 | 7 | -163.666 | 341.332 | 1.628 | 0.173 |
| -0.278 | NA | NA | NA | 1.53E-03 | NA | 1.76E-01 | 4 | -166.821 | 341.642 | 1.938 | 0.148 |
| -0.467 | NA | NA | + | NA | 1.46E-02 | 1.53E-01 | 7 | -163.833 | 341.666 | 1.962 | 0.146 |
| -0.428 | NA | NA | + | 9.31E-05 | NA | 1.53E-01 | 7 | -163.85 | 341.699 | 1.995 | 0.144 |
| -0.543 | NA | NA | + | NA | NA | 1.91E-01 | 6 | -152.405 | 316.81 | 0 | 0.322 |
| -0.375 | NA | NA | + | NA | -7.28E-02 | 1.90E-01 | 7 | -151.756 | 317.512 | 0.702 | 0.227 |
| -0.583 | 1.29E-09 | NA | + | NA | NA | 1.98E-01 | 7 | -152.088 | 318.177 | 1.367 | 0.163 |
| -0.608 | NA | NA | + | -8.38E-04 | NA | 1.95E-01 | 7 | -152.13 | 318.259 | I.45 | 0.156 |
| -0.435 | NA | NA | + | -1.10E-03 | -8.39E-02 | 1.96E-01 | 8 | -151.29 | 318.581 | 1.771 | 0.133 |
| -0.383 | NA | NA | + | NA | NA | 1.53E-01 | 6 | -138.036 | 288.072 | 0 | 0.324 |
| -0.321 | NA | NA | NA | NA | NA | 1.85E-01 | 3 | -141.683 | 289.367 | 1.295 | 0.169 |
| -0.408 | 8.15E-10 | NA | + | NA | NA | 1.58E-01 | 7 | -137.873 | 289.745 | 1.673 | 0.14 |
| -0.413 | NA | NA | + | -3.62E-04 | NA | 1.55E-01 | 7 | -137.987 | 289.975 | 1.903 | 0.125 |
| -0.263 | NA | NA | NA | 9.88E-04 | NA | 1.69E-01 | 4 | -141.022 | 290.045 | 1.973 | 0.121 |
| -0.361 | NA | NA | + | NA | -1.06E-02 | 1.54E-01 | 7 | -138.024 | 290.048 | 1.976 | 0.121 |
| -0.577 | NA | NA | + | NA | NA | 1.97E-01 | 6 | -157.081 | 326.161 | 0 | 0.332 |
| -0.663 | NA | NA | + | -1.20E-03 | NA | 2.01E-01 | 7 | -156.538 | 327.075 | 0.914 | 0.21 |
| -0.62 | 1.46E-09 | NA | + | NA | NA | 2.04E-01 | 7 | -156.74 | 327.48 | 1.319 | 0.172 |
| -0.463 | NA | NA | + | NA | -5.19E-02 | 1.99E-01 | 7 | -156.789 | 327.577 | 1.416 | 0.164 |
| -0.532 | NA | NA | + | -1.39E-03 | -6.59E-02 | 2.04E-01 | 8 | -156.077 | 328.155 | 1.994 | 0.123 |
| -0.586 | NA | NA | + | NA | NA | 2.00E-01 | 6 | -159.932 | 331.864 | 0 | 0.293 |
| -0.42 | NA | NA | + | NA | -7.39E-02 | 1.99E-01 | 7 | -159.283 | 332.567 | 0.703 | 0.206 |
| -0.673 | NA | NA | + | -1.15E-03 | NA | 2.05E-01 | 7 | -159.424 | 332.848 | 0.984 | 0.179 |
| -0.495 | NA | NA | + | -1.42E-03 | -8.78E-02 | 2.05E-01 | 8 | -158.528 | 333.056 | 1.192 | 0.161 |
| -0.633 | 1.73E-09 | NA | + | NA | NA | 2.07E-01 | 7 | -159.531 | 333.062 | 1.198 | 0.161 |
| -0.495 | NA | NA | + | NA | NA | 1.78E-01 | 6 | -163.834 | 339.669 | 0 | 0.457 |
| -0.528 | 1.20E-09 | NA | + | NA | NA | 1.83E-01 | 7 | -163.64 | 341.28 | 1.611 | 0.204 |
| -0.469 | NA | NA | + | NA | -1.15E-02 | 1.78E-01 | 7 | -163.822 | 341.645 | 1.976 | 0.17 |
| -0.5 | NA | NA | + | -7.12E-05 | NA | 1.78E-01 | 7 | -163.833 | 341.666 | 1.997 | 0.168 |
| -0.432 | NA | NA | + | NA | NA | 1.59E-01 | 6 | -140.655 | 293.31 | 0 | 0.427 |
| -0.466 | 1.28E-09 | NA | + | NA | NA | 1.64E-01 | 7 | -140.4 | 294.8 | I.49 | 0.203 |
| -0.497 | NA | NA | + | -7.78E-04 | NA | 1.65E-01 | 7 | -140.437 | 294.874 | 1.564 | 0.195 |
| -0.362 | NA | NA | + | NA | -3.13E-02 | 1.60E-01 | 7 | -140.542 | 295.084 | 1.775 | 0.176 |
| -0.446 | NA | NA | + | NA | NA | 1.67E-01 | 6 | -144.781 | 301.561 | 0 | 0.428 |
| -0.477 | 1.13E-09 | NA | + | NA | NA | 1.72E-01 | 7 | -144.551 | 303.103 | 1.541 | 0.198 |
| -0.5 | NA | NA | + | -7.20E-04 | NA | 1.70E-01 | 7 | -144.584 | 303.169 | 1.607 | 0.191 |
| -0.365 | NA | NA | + | NA | -3.66E-02 | 1.67E-01 | 7 | -144.628 | 303.257 | 1.695 | 0.183 |
| -0.169 | NA | NA | NA | 1.78E-03 | NA | 1.41E-01 | 4 | -169.789 | 347.578 | 0 | 0.331 |
| -0.283 | NA | NA | NA | NA | NA | 1.74E-01 | 3 | -171.255 | 348.509 | 0.931 | 0.208 |
| -0.293 | NA | NA | NA | 2.11E-03 | 7.32E-02 | 1.35E-01 | 5 | -169.386 | 348.773 | 1.194 | 0.182 |
| -0.356 | NA | NA | + | NA | NA | 1.41E-01 | 6 | -168.623 | 349.245 | 1.667 | 0.144 |
| -0.195 | 1.02E-09 | NA | NA | 1.79E-03 | NA | 1.45E-01 | 5 | -169.683 | 349.366 | 1.788 | 0.135 |
| -0.39 | NA | NA | + | NA | NA | 1.51E-01 | 6 | -157.645 | 327.291 | 0 | 0.235 |
| -0.221 | NA | NA | NA | 1.51E-03 | NA | 1.59E-01 | 4 | -159.753 | 327.506 | 0.215 | 0.211 |
| -0.307 | NA | NA | NA | NA | NA | 1.84E-01 | 3 | -160.911 | 327.822 | 0.532 | 0.18 |
| -0.417 | 9.91E-10 | NA | + | NA | NA | 1.55E-01 | 7 | -157.502 | 329.005 | 1.714 | 0.1 |
| -0.351 | NA | NA | + | 4.79E-04 | NA | 1.49E-01 | 7 | -157.576 | 329.152 | 1.862 | 0.093 |
| -0.25 | 1.06E-09 | NA | NA | 1.50E-03 | NA | 1.63E-01 | 5 | -159.594 | 329.189 | 1.898 | 0.091 |
| -0.428 | NA | NA | + | NA | 1.74E-02 | 1.51E-01 | 7 | -157.619 | 329.238 | 1.947 | 0.089 |
| -0.422 | NA | NA | + | NA | NA | 1.53E-01 | 6 | -150.591 | 313.183 | 0 | 0.455 |
| -0.451 | 9.95E-10 | NA | + | NA | NA | 1.57E-01 | 7 | -150.425 | 314.851 | 1.668 | 0.197 |
| -0.378 | NA | NA | + | NA | -2.01E-02 | 1.54E-01 | 7 | -150.551 | 315.101 | 1.918 | 0.174 |
| -0.45 | NA | NA | + | -3.35E-04 | NA | 1.54E-01 | 7 | -150.553 | 315.106 | 1.923 | 0.174 |
| -0.501 | NA | NA | + | NA | NA | 1.74E-01 | 6 | -155.944 | 323.888 | 0 | 0.44 |
| -0.535 | 1.31E-09 | NA | + | NA | NA | 1.79E-01 | 7 | -155.741 | 325.483 | 1.594 | 0.198 |
| -0.556 | NA | NA | + | -6.60E-04 | NA | 1.78E-01 | 7 | -155.8 | 325.6 | 1.711 | 0.187 |
| -0.438 | NA | NA | + | NA | -2.74E-02 | 1.74E-01 | 7 | -155.868 | 325.737 | 1.848 | 0.175 |
| -0.658 | NA | NA | + | NA | NA | 2.18E-01 | 6 | -174.825 | 361.649 | 0 | 0.426 |
| -0.702 | 1.46E-09 | NA | + | NA | NA | 2.26E-01 | 7 | -174.522 | 363.044 | 1.395 | 0.212 |
| -0.713 | NA | NA | + | -8.42E-04 | NA | 2.21E-01 | 7 | -174.594 | 363.189 | 1.539 | 0.197 |
| -0.604 | NA | NA | + | NA | -2.42E-02 | 2.18E-01 | 7 | -174.771 | 363.542 | 1.892 | 0.165 |
| -0.608 | NA | NA | + | NA | NA | 2.05E-01 | 6 | -165.807 | 343.614 | 0 | 0.413 |
| -0.653 | 1.61E-09 | NA | + | NA | NA | 2.13E-01 | 7 | -165.446 | 344.893 | 1.278 | 0.218 |
| -0.659 | NA | NA | + | -7.48E-04 | NA | 2.08E-01 | 7 | -165.609 | 345.218 | 1.604 | 0.185 |
| -0.509 | NA | NA | + | NA | -4.49E-02 | 2.06E-01 | 7 | -165.61 | 345.22 | 1.605 | 0.185 |
| -0.486 | NA | NA | + | NA | NA | 1.74E-01 | 6 | -163.368 | 338.737 | 0 | 0.456 |
| -0.52 | 9.82E-10 | NA | + | NA | NA | 1.81E-01 | 7 | -163.159 | 340.317 | I.58 | 0.207 |
| -0.504 | NA | NA | + | NA | 8.32E-03 | 1.73E-01 | 7 | -163.362 | 340.724 | 1.987 | 0.169 |
| -0.486 | NA | NA | + | 2.28E-06 | NA | 1.74E-01 | 7 | -163.368 | 340.737 | 2 | 0.168 |
| -0.568 | NA | NA | + | -1.91E-03 | -1.09E-01 | 2.37E-01 | 8 | -151.464 | 318.929 | 0 | 0.313 |
| -0.478 | NA | NA | + | NA | -8.94E-02 | 2.30E-01 | 7 | -152.997 | 319.995 | 1.066 | 0.184 |
| -0.788 | NA | NA | + | -1.56E-03 | NA | 2.37E-01 | 7 | -153.05 | 320.101 | 1.172 | 0.174 |
| -0.679 | NA | NA | + | NA | NA | 2.31E-01 | 6 | -154.089 | 320.177 | 1.248 | 0.168 |
| -0.62 | 1.46E-09 | NA | + | -1.91E-03 | -1.03E-01 | 2.44E-01 | 9 | -151.131 | 320.262 | 1.333 | 0.161 |
| -0.577 | NA | NA | + | -1.94E-03 | -1.27E-01 | 2.45E-01 | 8 | -159.08 | 334.16 | 0 | 0.468 |
| -0.646 | 1.96E-09 | NA | + | -1.91E-03 | -1.19E-01 | 2.54E-01 | 9 | -158.592 | 335.184 | 1.025 | 0.28 |
| -0.505 | NA | NA | + | NA | -1.04E-01 | 2.40E-01 | 7 | -160.7 | 335.399 | I.24 | 0.252 |
| -0.499 | NA | NA | + | NA | NA | 1.82E-01 | 6 | -156.038 | 324.076 | 0 | 0.435 |
| -0.537 | 1.29E-09 | NA | + | NA | NA | 1.88E-01 | 7 | -155.755 | 325.511 | 1.435 | 0.212 |
| -0.546 | NA | NA | + | -5.97E-04 | NA | 1.85E-01 | 7 | -155.916 | 325.833 | 1.757 | 0.181 |
| -0.439 | NA | NA | + | NA | -2.70E-02 | 1.82E-01 | 7 | -155.962 | 325.925 | 1.849 | 0.172 |
| -0.52 | NA | NA | + | NA | NA | 1.78E-01 | 6 | -149.89 | 311.78 | 0 | 0.335 |
| -0.61 | NA | NA | + | -1.11E-03 | NA | 1.84E-01 | 7 | -149.443 | 312.885 | 1.106 | 0.193 |
| -0.387 | NA | NA | + | NA | -5.93E-02 | 1.79E-01 | 7 | -149.484 | 312.968 | 1.189 | 0.185 |
| -0.555 | 1.24E-09 | NA | + | NA | NA | 1.84E-01 | 7 | -149.626 | 313.251 | 1.472 | 0.16 |
| -0.466 | NA | NA | + | -1.33E-03 | -7.19E-02 | 1.87E-01 | 8 | -148.859 | 313.718 | 1.938 | 0.127 |
| -0.37 | NA | NA | + | NA | NA | 1.43E-01 | 6 | -143.97 | 299.941 | 0 | 0.326 |
| -0.22 | NA | NA | NA | 1.29E-03 | NA | 1.55E-01 | 4 | -146.747 | 301.493 | 1.552 | 0.15 |
| -0.307 | NA | NA | NA | NA | NA | 1.79E-01 | 3 | -147.763 | 301.525 | 1.584 | 0.148 |
| -0.395 | 7.88E-10 | NA | + | NA | NA | 1.47E-01 | 7 | -143.845 | 301.69 | 1.749 | 0.136 |
| -0.375 | NA | NA | + | -5.63E-05 | NA | 1.43E-01 | 7 | -143.969 | 301.939 | 1.998 | 0.12 |
| -0.372 | NA | NA | + | NA | 8.92E-04 | 1.43E-01 | 7 | -143.97 | 301.941 | 2 | 0.12 |
| -0.468 | NA | NA | + | NA | NA | 1.70E-01 | 6 | -148.992 | 309.984 | 0 | 0.427 |
| -0.502 | 9.67E-10 | NA | + | NA | NA | 1.77E-01 | 7 | -148.741 | 311.481 | 1.497 | 0.202 |
| -0.533 | NA | NA | + | -8.18E-04 | NA | 1.74E-01 | 7 | -148.751 | 311.501 | 1.517 | 0.2 |
| -0.405 | NA | NA | + | NA | -2.84E-02 | 1.70E-01 | 7 | -148.905 | 311.811 | 1.827 | 0.171 |
| -0.45 | NA | NA | + | NA | NA | 1.62E-01 | 6 | -153.642 | 319.284 | 0 | 0.447 |
| -0.484 | 1.10E-09 | NA | + | NA | NA | 1.69E-01 | 7 | -153.405 | 320.81 | 1.526 | 0.209 |
| -0.481 | NA | NA | + | -4.18E-04 | NA | 1.64E-01 | 7 | -153.584 | 321.168 | 1.884 | 0.174 |
| -0.412 | NA | NA | + | NA | -1.76E-02 | 1.63E-01 | 7 | -153.61 | 321.221 | 1.937 | 0.17 |
| -0.538 | NA | NA | + | NA | NA | 1.88E-01 | 6 | -150.623 | 313.246 | 0 | 0.315 |
| -0.384 | NA | NA | + | NA | -6.80E-02 | 1.88E-01 | 7 | -150.07 | 314.14 | 0.894 | 0.201 |
| -0.619 | NA | NA | + | -1.05E-03 | NA | 1.93E-01 | 7 | -150.189 | 314.377 | 1.132 | 0.179 |
| -0.582 | 1.60E-09 | NA | + | NA | NA | 1.95E-01 | 7 | -150.283 | 314.566 | 1.321 | 0.163 |
| -0.453 | NA | NA | + | -1.31E-03 | -8.14E-02 | 1.95E-01 | 8 | -149.416 | 314.831 | 1.586 | 0.142 |
| -0.725 | NA | NA | + | -1.77E-03 | NA | 2.11E-01 | 7 | -147.738 | 309.476 | 0 | 0.218 |
| -0.552 | NA | NA | + | -2.03E-03 | -8.81E-02 | 2.13E-01 | 8 | -146.806 | 309.611 | 0.136 | 0.204 |
| -0.599 | NA | NA | + | NA | NA | 2.05E-01 | 6 | -149.024 | 310.048 | 0.573 | 0.164 |
| -0.769 | 1.41E-09 | NA | + | -1.79E-03 | NA | 2.18E-01 | 8 | -147.34 | 310.68 | 1.204 | 0.12 |
| -0.451 | NA | NA | + | NA | -6.77E-02 | 2.06E-01 | 7 | -148.467 | 310.935 | 1.459 | 0.105 |
| -0.598 | 1.25E-09 | NA | + | -2.04E-03 | -8.43E-02 | 2.20E-01 | 9 | -146.487 | 310.974 | 1.498 | 0.103 |
| -0.638 | 1.33E-09 | NA | + | NA | NA | 2.12E-01 | 7 | -148.675 | 311.35 | 1.874 | 0.086 |
| -0.542 | NA | NA | + | NA | NA | 1.94E-01 | 6 | -147.84 | 307.68 | 0 | 0.193 |
| -0.456 | NA | NA | + | -1.66E-03 | -9.54E-02 | 2.02E-01 | 8 | -145.964 | 307.927 | 0.247 | 0.17 |
| -0.642 | NA | NA | + | -1.37E-03 | NA | 1.99E-01 | 7 | -147.047 | 308.094 | 0.414 | 0.157 |
| -0.372 | NA | NA | + | NA | -7.80E-02 | 1.96E-01 | 7 | -147.101 | 308.201 | 0.521 | 0.148 |
| -0.581 | 1.52E-09 | NA | + | NA | NA | 2.00E-01 | 7 | -147.506 | 309.012 | 1.332 | 0.099 |
| -0.682 | 1.53E-09 | NA | + | -1.37E-03 | NA | 2.05E-01 | 8 | -146.703 | 309.406 | 1.725 | 0.081 |
| -0.498 | 1.31E-09 | NA | + | -1.65E-03 | -9.15E-02 | 2.07E-01 | 9 | -145.708 | 309.417 | 1.736 | 0.081 |
| -0.415 | 1.34E-09 | NA | + | NA | -7.42E-02 | 2.01E-01 | 8 | -146.84 | 309.68 | 2 | 0.071 |
| -0.376 | NA | NA | + | NA | NA | 1.43E-01 | 6 | -142.32 | 296.64 | 0 | 0.448 |
| -0.402 | 8.92E-10 | NA | + | NA | NA | 1.47E-01 | 7 | -142.166 | 298.333 | 1.692 | 0.192 |
| -0.421 | NA | NA | + | -5.27E-04 | NA | 1.45E-01 | 7 | -142.219 | 298.439 | 1.798 | 0.182 |
| -0.318 | NA | NA | + | NA | -2.65E-02 | 1.44E-01 | 7 | -142.246 | 298.491 | 1.851 | 0.178 |
| -0.494 | NA | NA | + | NA | NA | 1.77E-01 | 6 | -151.134 | 314.268 | 0 | 0.415 |
| -0.569 | NA | NA | + | -9.38E-04 | NA | 1.82E-01 | 7 | -150.816 | 315.633 | 1.364 | 0.21 |
| -0.53 | 1.19E-09 | NA | + | NA | NA | 1.83E-01 | 7 | -150.866 | 315.733 | 1.465 | 0.199 |
| -0.414 | NA | NA | + | NA | -3.68E-02 | 1.78E-01 | 7 | -150.988 | 315.976 | 1.708 | 0.177 |
| -0.609 | NA | NA | + | NA | NA | 2.03E-01 | 6 | -162.217 | 336.434 | 0 | 0.309 |
| -0.457 | NA | NA | + | NA | -6.89E-02 | 2.04E-01 | 7 | -161.7 | 337.401 | 0.967 | 0.191 |
| -0.687 | NA | NA | + | -1.14E-03 | NA | 2.07E-01 | 7 | -161.737 | 337.474 | 01.IV | 0.184 |
| -0.658 | 1.88E-09 | NA | + | NA | NA | 2.11E-01 | 7 | -161.78 | 337.56 | 1.126 | 0.176 |
| -0.521 | NA | NA | + | -1.37E-03 | -8.25E-02 | 2.08E-01 | 8 | -161.013 | 338.026 | 1.592 | 0.14 |
| -0.389 | NA | NA | + | NA | NA | 1.50E-01 | 6 | -142.645 | 297.289 | 0 | 0.448 |
| -0.416 | 1.00E-09 | NA | + | NA | NA | 1.54E-01 | 7 | -142.485 | 298.969 | I.68 | 0.193 |
| -0.432 | NA | NA | + | -5.19E-04 | NA | 1.53E-01 | 7 | -142.549 | 299.098 | 1.808 | 0.181 |
| -0.334 | NA | NA | + | NA | -2.63E-02 | 1.51E-01 | 7 | -142.569 | 299.137 | 1.848 | 0.178 |
| -0.247 | NA | NA | NA | 1.71E-03 | NA | 1.67E-01 | 4 | -165.945 | 339.889 | 0 | 0.296 |
| -0.426 | NA | NA | + | NA | NA | 1.60E-01 | 6 | -164.117 | 340.233 | 0.344 | 0.249 |
| -0.362 | NA | NA | NA | NA | NA | 2.00E-01 | 3 | -167.305 | 340.61 | 0.721 | 0.206 |
| -0.322 | NA | NA | NA | 1.89E-03 | 4.18E-02 | 1.65E-01 | 5 | -165.802 | 341.604 | 1.715 | 0.126 |
| -0.275 | 9.02E-10 | NA | NA | 1.70E-03 | NA | 1.72E-01 | 5 | -165.823 | 341.646 | 1.757 | 0.123 |
| -0.388 | NA | NA | + | NA | NA | 1.45E-01 | 6 | -150.494 | 312.987 | 0 | 0.46 |
| -0.416 | 9.61E-10 | NA | + | NA | NA | 1.49E-01 | 7 | -150.344 | 314.688 | 01.VII | 0.197 |
| -0.406 | NA | NA | + | -2.24E-04 | NA | 1.46E-01 | 7 | -150.478 | 314.956 | 1.968 | 0.172 |
| -0.368 | NA | NA | + | NA | -9.14E-03 | 1.45E-01 | 7 | -150.485 | 314.971 | 1.983 | 0.171 |
| -0.51 | NA | NA | + | NA | NA | 1.80E-01 | 6 | -158.018 | 328.035 | 0 | 0.408 |
| -0.553 | 1.56E-09 | NA | + | NA | NA | 1.86E-01 | 7 | -157.675 | 329.349 | 1.314 | 0.211 |
| -0.572 | NA | NA | + | -8.50E-04 | NA | 1.83E-01 | 7 | -157.77 | 329.541 | 1.506 | 0.192 |
| -0.413 | NA | NA | + | NA | -4.56E-02 | 1.82E-01 | 7 | -157.785 | 329.571 | 1.536 | 0.189 |
| -0.393 | NA | NA | + | NA | NA | 1.50E-01 | 6 | -148.731 | 309.463 | 0 | 0.299 |
| -0.321 | NA | NA | NA | NA | NA | 1.84E-01 | 3 | -152.22 | 310.439 | 0.976 | 0.183 |
| -0.24 | NA | NA | NA | 1.28E-03 | NA | 1.61E-01 | 4 | -151.299 | 310.599 | 1.136 | 0.169 |
| -0.419 | 7.61E-10 | NA | + | NA | NA | 1.55E-01 | 7 | -148.588 | 311.177 | 1.714 | 0.127 |
| -0.416 | NA | NA | + | NA | 1.08E-02 | 1.50E-01 | 7 | -148.72 | 311.44 | 1.977 | 0.111 |
| -0.398 | NA | NA | + | -5.50E-05 | NA | 1.51E-01 | 7 | -148.73 | 311.461 | 1.998 | 0.11 |
| -0.486 | NA | NA | + | NA | NA | 1.66E-01 | 6 | -156.676 | 325.352 | 0 | 0.461 |
| -0.514 | 1.05E-09 | NA | + | NA | NA | 1.70E-01 | 7 | -156.541 | 327.081 | 1.729 | 0.194 |
| -0.512 | NA | NA | + | -3.18E-04 | NA | 1.67E-01 | 7 | -156.644 | 327.288 | 1.936 | 0.175 |
| -0.473 | NA | NA | + | NA | -5.79E-03 | 1.66E-01 | 7 | -156.673 | 327.346 | 1.994 | 0.17 |
| -0.626 | NA | NA | + | NA | NA | 2.16E-01 | 6 | -158.951 | 329.902 | 0 | 0.328 |
| -0.484 | NA | NA | + | NA | -6.24E-02 | 2.14E-01 | 7 | -158.528 | 331.056 | 1.155 | 0.184 |
| -0.698 | NA | NA | + | -1.03E-03 | NA | 2.20E-01 | 7 | -158.531 | 331.062 | 1.161 | 0.184 |
| -0.672 | 1.48E-09 | NA | + | NA | NA | 2.24E-01 | 7 | -158.564 | 331.128 | 1.227 | 0.178 |
| -0.541 | NA | NA | + | -1.27E-03 | -7.68E-02 | 2.19E-01 | 8 | -157.91 | 331.821 | 1.919 | 0.126 |
| -0.405 | NA | NA | + | NA | NA | 1.56E-01 | 6 | -136.665 | 285.33 | 0 | 0.432 |
| -0.435 | 8.93E-10 | NA | + | NA | NA | 1.61E-01 | 7 | -136.426 | 286.851 | 1.521 | 0.202 |
| -0.467 | NA | NA | + | -7.71E-04 | NA | 1.59E-01 | 7 | -136.439 | 286.879 | 1.548 | 0.199 |
| -0.36 | NA | NA | + | NA | -2.16E-02 | 1.57E-01 | 7 | -136.611 | 287.221 | 1.891 | 0.168 |
| -0.49 | NA | NA | + | NA | NA | 1.70E-01 | 6 | -141.989 | 295.979 | 0 | 0.385 |
| -0.577 | NA | NA | + | -1.16E-03 | NA | 1.75E-01 | 7 | -141.465 | 296.931 | 0.952 | 0.239 |
| -0.527 | 1.29E-09 | NA | + | NA | NA | 1.76E-01 | 7 | -141.706 | 297.412 | 1.433 | 0.188 |
| -0.381 | NA | NA | + | NA | -4.86E-02 | 1.71E-01 | 7 | -141.706 | 297.413 | 1.434 | 0.188 |
| -0.413 | NA | NA | + | NA | NA | 1.60E-01 | 6 | -143.349 | 298.698 | 0 | 0.452 |
| -0.442 | 9.14E-10 | NA | + | NA | NA | 1.65E-01 | 7 | -143.128 | 300.256 | 1.558 | 0.207 |
| -0.381 | NA | NA | + | NA | -1.49E-02 | 1.61E-01 | 7 | -143.326 | 300.652 | 1.954 | 0.17 |
| -0.434 | NA | NA | + | -2.52E-04 | NA | 1.61E-01 | 7 | -143.327 | 300.653 | 1.955 | 0.17 |
| -0.484 | NA | NA | + | NA | NA | 1.76E-01 | 6 | -163.694 | 339.388 | 0 | 0.337 |
| -0.523 | 1.36E-09 | NA | + | NA | NA | 1.82E-01 | 7 | -163.448 | 340.896 | 1.508 | 0.158 |
| -0.408 | NA | NA | NA | NA | NA | 2.17E-01 | 3 | -167.644 | 341.288 | 01.IX | 0.13 |
| -0.458 | NA | NA | + | NA | -1.18E-02 | 1.76E-01 | 7 | -163.681 | 341.362 | 1.974 | 0.125 |
| -0.331 | NA | NA | NA | 1.40E-03 | NA | 1.94E-01 | 4 | -166.682 | 341.364 | 1.976 | 0.125 |
| -0.478 | NA | NA | + | 7.96E-05 | NA | 1.76E-01 | 7 | -163.692 | 341.384 | 1.996 | 0.124 |
| -0.612 | NA | NA | + | NA | NA | 2.08E-01 | 6 | -161.583 | 335.166 | 0 | 0.421 |
| -0.656 | 1.53E-09 | NA | + | NA | NA | 2.15E-01 | 7 | -161.255 | 336.51 | 1.344 | 0.215 |
| -0.66 | NA | NA | + | -6.85E-04 | NA | 2.10E-01 | 7 | -161.413 | 336.826 | I.66 | 0.183 |
| -0.522 | NA | NA | + | NA | -3.92E-02 | 2.07E-01 | 7 | -161.425 | 336.849 | 1.683 | 0.181 |
| -0.597 | NA | NA | + | NA | NA | 1.99E-01 | 6 | -163.44 | 338.88 | 0 | 0.424 |
| -0.64 | 1.52E-09 | NA | + | NA | NA | 2.06E-01 | 7 | -163.106 | 340.212 | 1.332 | 0.218 |
| -0.507 | NA | NA | + | NA | -3.94E-02 | 1.99E-01 | 7 | -163.281 | 340.561 | 1.681 | 0.183 |
| -0.638 | NA | NA | + | -5.78E-04 | NA | 2.01E-01 | 7 | -163.323 | 340.646 | 1.765 | 0.175 |
| -0.536 | NA | NA | + | NA | NA | 1.91E-01 | 6 | -153.382 | 318.765 | 0 | 0.414 |
| -0.575 | 1.45E-09 | NA | + | NA | NA | 1.97E-01 | 7 | -153.049 | 320.099 | 1.334 | 0.212 |
| -0.433 | NA | NA | + | NA | -4.59E-02 | 1.91E-01 | 7 | -153.157 | 320.313 | 1.549 | 0.191 |
| -0.589 | NA | NA | + | -7.03E-04 | NA | 1.94E-01 | 7 | -153.196 | 320.392 | 1.628 | 0.183 |
| -0.624 | NA | NA | + | NA | NA | 2.14E-01 | 6 | -154.996 | 321.993 | 0 | 0.189 |
| -0.728 | NA | NA | + | -1.45E-03 | NA | 2.19E-01 | 7 | -154.143 | 322.285 | 0.292 | 0.163 |
| -0.54 | NA | NA | + | -1.72E-03 | -9.09E-02 | 2.20E-01 | 8 | -153.159 | 322.317 | 0.325 | 0.16 |
| -0.456 | NA | NA | + | NA | -7.38E-02 | 2.13E-01 | 7 | -154.337 | 322.674 | 0.682 | 0.134 |
| -0.671 | 1.70E-09 | NA | + | NA | NA | 2.21E-01 | 7 | -154.567 | 323.133 | 1.141 | 0.107 |
| -0.777 | 1.73E-09 | NA | + | -1.46E-03 | NA | 2.27E-01 | 8 | -153.692 | 323.383 | 1.391 | 0.094 |
| -0.593 | 1.51E-09 | NA | + | -1.72E-03 | -8.60E-02 | 2.27E-01 | 9 | -152.813 | 323.626 | 1.633 | 0.083 |
| -0.509 | 1.52E-09 | NA | + | NA | -6.89E-02 | 2.20E-01 | 8 | -153.993 | 323.987 | 1.994 | 0.07 |
| -0.38 | NA | NA | + | NA | NA | 1.47E-01 | 6 | -140.781 | 293.562 | 0 | 0.377 |
| -0.322 | NA | NA | NA | NA | NA | 1.85E-01 | 3 | -144.608 | 295.215 | 1.653 | 0.165 |
| -0.407 | 1.03E-09 | NA | + | NA | NA | 1.51E-01 | 7 | -140.631 | 295.261 | 1.699 | 0.161 |
| -0.42 | NA | NA | + | -4.78E-04 | NA | 1.50E-01 | 7 | -140.7 | 295.401 | 1.839 | 0.15 |
| -0.334 | NA | NA | + | NA | -2.11E-02 | 1.48E-01 | 7 | -140.732 | 295.463 | 1.901 | 0.146 |
| -0.459 | NA | NA | + | NA | NA | 1.67E-01 | 6 | -164.465 | 340.93 | 0 | 0.391 |
| -0.494 | 1.23E-09 | NA | + | NA | NA | 1.73E-01 | 7 | -164.259 | 342.518 | 1.588 | 0.177 |
| -0.302 | NA | NA | NA | 1.46E-03 | NA | 1.84E-01 | 4 | -167.458 | 342.916 | 1.987 | 0.145 |
| -0.471 | NA | NA | + | NA | 5.74E-03 | 1.67E-01 | 7 | -164.462 | 342.924 | 1.994 | 0.144 |
| -0.456 | NA | NA | + | 3.96E-05 | NA | 1.67E-01 | 7 | -164.464 | 342.929 | 1.999 | 0.144 |
| -0.487 | NA | NA | + | NA | NA | 1.76E-01 | 6 | -157.668 | 327.336 | 0 | 0.435 |
| -0.522 | 1.35E-09 | NA | + | NA | NA | 1.81E-01 | 7 | -157.438 | 328.877 | 1.541 | 0.201 |
| -0.545 | NA | NA | + | -7.15E-04 | NA | 1.80E-01 | 7 | -157.498 | 328.996 | I.66 | 0.19 |
| -0.424 | NA | NA | + | NA | -2.96E-02 | 1.77E-01 | 7 | -157.582 | 329.164 | 1.828 | 0.174 |
| -0.4 | NA | NA | + | NA | NA | 1.51E-01 | 6 | -167.359 | 346.718 | 0 | 0.206 |
| -0.233 | NA | NA | NA | 1.65E-03 | NA | 1.61E-01 | 4 | -169.372 | 346.744 | 0.026 | 0.203 |
| -0.345 | NA | NA | NA | NA | NA | 1.92E-01 | 3 | -170.678 | 347.357 | 0.638 | 0.149 |
| -0.329 | NA | NA | NA | 1.90E-03 | 5.53E-02 | 1.58E-01 | 5 | -169.134 | 348.269 | 1.551 | 0.095 |
| -0.513 | NA | NA | + | NA | 5.30E-02 | 1.48E-01 | 7 | -167.135 | 348.27 | 1.551 | 0.095 |
| -0.429 | 1.00E-09 | NA | + | NA | NA | 1.56E-01 | 7 | -167.222 | 348.443 | 1.725 | 0.087 |
| -0.261 | 9.59E-10 | NA | NA | 1.65E-03 | NA | 1.66E-01 | 5 | -169.249 | 348.498 | I.78 | 0.084 |
| -0.356 | NA | NA | + | 5.25E-04 | NA | 1.48E-01 | 7 | -167.287 | 348.573 | 1.855 | 0.081 |
| -0.558 | NA | NA | + | NA | NA | 1.86E-01 | 6 | -154.023 | 320.046 | 0 | 0.393 |
| -0.427 | NA | NA | + | NA | -5.80E-02 | 1.87E-01 | 7 | -153.628 | 321.256 | 1.209 | 0.215 |
| -0.599 | 1.32E-09 | NA | + | NA | NA | 1.94E-01 | 7 | -153.665 | 321.33 | 1.283 | 0.207 |
[truncated: 35,561 more chars]
