# Supplementary material for: Traits and ecological space availability predict avian densities at the country scale of the Czech Republic
Source: Ecol Evol. 2022 Jul 17;12(7):e9119. doi: 10.1002/ece3.9119 (PMC9289119; doi:10.1002/ece3.9119)
Supplement: Supplementary file 4 — Table S3 [file ECE3-12-e9119-s007.docx]

| (Intercept) | Range | Forest dependency | Nest type | ssPC | SSI diet | SSI habitat | df | logLik | AIC | delta | weight |
| --- | --- | --- | --- | --- | --- | --- | --- | --- | --- | --- | --- |
| 0.00027 | NA | NA | + | NA | NA | NA | 5 | 125.726 | -241.452 | 0 | 0.357 |
| 0.0233 | NA | NA | + | NA | -1.01E-02 | NA | 6 | 126.196 | -240.392 | 01.VI | 0.21 |
| 0.00746 | NA | NA | + | 4.43E-05 | NA | NA | 6 | 125.926 | -239.851 | 01.VI | 0.16 |
| -0.00615 | NA | NA | + | NA | NA | 2.74E-03 | 6 | 125.771 | -239.541 | I.91 | 0.137 |
| -0.00103 | 7.20E-11 | NA | + | NA | NA | NA | 6 | 125.759 | -239.517 | 1.934 | 0.136 |
| 0.00168 | NA | NA | + | NA | NA | NA | 5 | 121.702 | -233.405 | 0 | 0.243 |
| 0.01939 | NA | NA | + | 9.52E-05 | NA | NA | 6 | 122.406 | -232.812 | 0.593 | 0.181 |
| 0.04539 | NA | NA | NA | 1.59E-04 | NA | NA | 3 | 119.188 | -232.376 | 1.029 | 0.145 |
| 0.02157 | NA | NA | + | NA | NA | -8.14E-03 | 6 | 122.004 | -232.009 | 1.396 | 0.121 |
| 0.01993 | NA | NA | + | NA | -8.10E-03 | NA | 6 | 121.916 | -231.832 | 1.573 | 0.111 |
| 0.04732 | NA | NA | + | 1.08E-04 | NA | -1.04E-02 | 7 | 122.894 | -231.788 | 1.617 | 0.108 |
| 0.00066 | 5.77E-11 | NA | + | NA | NA | NA | 6 | 121.73 | -231.46 | 1.945 | 0.092 |
| 0.00308 | NA | NA | + | NA | NA | NA | 5 | 121.401 | -232.801 | 0 | 0.364 |
| 0.01349 | NA | NA | + | 6.05E-05 | NA | NA | 6 | 121.72 | -231.44 | 1.361 | 0.185 |
| 0.02137 | NA | NA | + | NA | -8.25E-03 | NA | 6 | 121.675 | -231.349 | 1.452 | 0.176 |
| 0.00157 | 8.76E-11 | NA | + | NA | NA | NA | 6 | 121.444 | -230.889 | 1.913 | 0.14 |
| 0.0053 | NA | NA | + | NA | NA | -9.12E-04 | 6 | 121.405 | -230.81 | 1.992 | 0.135 |
| 0.00231 | NA | NA | + | NA | NA | NA | 5 | 123.421 | -236.842 | 0 | 0.327 |
| 0.01762 | NA | NA | + | 8.52E-05 | NA | NA | 6 | 124.086 | -236.172 | 0.67 | 0.234 |
| 0.02613 | NA | NA | + | NA | -1.06E-02 | NA | 6 | 123.875 | -235.75 | 1.092 | 0.19 |
| 0.00076 | 9.01E-11 | NA | + | NA | NA | NA | 6 | 123.474 | -234.948 | 1.894 | 0.127 |
| 0.00596 | NA | NA | + | NA | NA | -1.51E-03 | 6 | 123.433 | -234.866 | 1.976 | 0.122 |
| -0.00086 | NA | NA | + | NA | NA | NA | 5 | 123.303 | -236.605 | 0 | 0.359 |
| 0.01022 | NA | NA | + | 6.56E-05 | NA | NA | 6 | 123.725 | -235.449 | 1.156 | 0.201 |
| 0.0173 | NA | NA | + | NA | -8.04E-03 | NA | 6 | 123.568 | -235.137 | 1.468 | 0.172 |
| -0.00174 | 5.04E-11 | NA | + | NA | NA | NA | 6 | 123.326 | -234.652 | 1.953 | 0.135 |
| -0.00118 | NA | NA | + | NA | NA | 1.37E-04 | 6 | 123.303 | -234.605 | 2 | 0.132 |
| 0.00306 | NA | NA | + | NA | NA | NA | 5 | 124.367 | -238.735 | 0 | 0.352 |
| 0.02614 | NA | NA | + | NA | -1.03E-02 | NA | 6 | 124.793 | -237.586 | 1.149 | 0.198 |
| 0.01366 | NA | NA | + | 6.17E-05 | NA | NA | 6 | 124.716 | -237.433 | 1.302 | 0.183 |
| 0.00136 | 1.03E-10 | NA | + | NA | NA | NA | 6 | 124.421 | -236.842 | 1.893 | 0.137 |
| 0.00557 | NA | NA | + | NA | NA | -1.04E-03 | 6 | 124.373 | -236.746 | 1.989 | 0.13 |
| 0.00148 | NA | NA | + | NA | NA | NA | 5 | 121.245 | -232.49 | 0 | 0.345 |
| 0.0149 | NA | NA | + | 7.28E-05 | NA | NA | 6 | 121.694 | -231.387 | 1.102 | 0.199 |
| 0.02509 | NA | NA | + | NA | -1.01E-02 | NA | 6 | 121.66 | -231.32 | I.17 | 0.192 |
| -0.00042 | 1.09E-10 | NA | + | NA | NA | NA | 6 | 121.303 | -230.605 | 1.884 | 0.135 |
| -0.00218 | NA | NA | + | NA | NA | 1.59E-03 | 6 | 121.258 | -230.516 | 1.973 | 0.129 |
| 0.00478 | NA | NA | + | NA | NA | NA | 5 | 121.505 | -233.011 | 0 | 0.318 |
| 0.0189 | NA | NA | + | 7.38E-05 | NA | NA | 6 | 121.942 | -231.885 | 1.126 | 0.181 |
| 0.01771 | NA | NA | + | NA | -5.64E-03 | NA | 6 | 121.621 | -231.242 | 1.768 | 0.131 |
| 0.04889 | NA | NA | NA | 1.39E-04 | NA | NA | 3 | 118.584 | -231.168 | 1.843 | 0.126 |
| 0.00275 | 1.14E-10 | NA | + | NA | NA | NA | 6 | 121.573 | -231.147 | 1.864 | 0.125 |
| 0.0081 | NA | NA | + | NA | NA | -1.43E-03 | 6 | 121.516 | -231.032 | 1.979 | 0.118 |
| 0.00488 | NA | NA | + | NA | NA | NA | 5 | 127.754 | -245.509 | 0 | 0.362 |
| 0.01597 | NA | NA | + | 6.43E-05 | NA | NA | 6 | 128.129 | -244.259 | I.25 | 0.194 |
| 0.02042 | NA | NA | + | NA | -6.84E-03 | NA | 6 | 127.947 | -243.895 | 1.614 | 0.162 |
| 0.0148 | NA | NA | + | NA | NA | -4.19E-03 | 6 | 127.844 | -243.688 | 1.821 | 0.146 |
| 0.00406 | 4.99E-11 | NA | + | NA | NA | NA | 6 | 127.778 | -243.556 | 1.953 | 0.136 |
| 0.00404 | NA | NA | + | NA | NA | NA | 5 | 128.859 | -247.717 | 0 | 0.356 |
| 0.02535 | NA | NA | + | NA | -9.65E-03 | NA | 6 | 129.27 | -246.54 | 1.177 | 0.198 |
| 0.01258 | NA | NA | + | 5.37E-05 | NA | NA | 6 | 129.148 | -246.296 | 1.422 | 0.175 |
| 0.0025 | 9.18E-11 | NA | + | NA | NA | NA | 6 | 128.909 | -245.819 | 1.898 | 0.138 |
| 0.00025 | NA | NA | + | NA | NA | 1.56E-03 | 6 | 128.872 | -245.745 | 1.972 | 0.133 |
| 0.00272 | NA | NA | + | NA | NA | NA | 5 | 122.825 | -235.649 | 0 | 0.368 |
| 0.0221 | NA | NA | + | NA | -8.63E-03 | NA | 6 | 123.124 | -234.248 | 1.402 | 0.182 |
| 0.01247 | NA | NA | + | 5.35E-05 | NA | NA | 6 | 123.077 | -234.153 | 1.496 | 0.174 |
| -0.00233 | NA | NA | + | NA | NA | 2.12E-03 | 6 | 122.853 | -233.706 | 1.943 | 0.139 |
| 0.00204 | 3.71E-11 | NA | + | NA | NA | NA | 6 | 122.836 | -233.671 | 1.978 | 0.137 |
| 0.0043 | NA | NA | + | NA | NA | NA | 5 | 127.085 | -244.17 | 0 | 0.374 |
| 0.01971 | NA | NA | + | NA | -6.92E-03 | NA | 6 | 127.317 | -242.633 | 1.536 | 0.173 |
| 0.01171 | NA | NA | + | 4.77E-05 | NA | NA | 6 | 127.313 | -242.626 | 1.544 | 0.173 |
| 0.00292 | 8.27E-11 | NA | + | NA | NA | NA | 6 | 127.126 | -242.252 | 1.918 | 0.143 |
| 0.00449 | NA | NA | + | NA | NA | -7.93E-05 | 6 | 127.085 | -242.17 | 2 | 0.137 |
| 0.00401 | NA | NA | + | NA | NA | NA | 5 | 124.626 | -239.251 | 0 | 0.353 |
| 0.01648 | NA | NA | + | 7.14E-05 | NA | NA | 6 | 125.069 | -238.139 | 1.112 | 0.202 |
| 0.02221 | NA | NA | + | NA | -8.13E-03 | NA | 6 | 124.885 | -237.77 | 1.481 | 0.168 |
| 0.00231 | 1.03E-10 | NA | + | NA | NA | NA | 6 | 124.694 | -237.388 | 1.864 | 0.139 |
| 0.01185 | NA | NA | + | NA | NA | -3.26E-03 | 6 | 124.68 | -237.36 | 1.892 | 0.137 |
| -0.00121 | NA | NA | + | NA | NA | NA | 5 | 126.492 | -242.984 | 0 | 0.338 |
| 0.01277 | NA | NA | + | 8.14E-05 | NA | NA | 6 | 127.097 | -242.194 | 0.79 | 0.227 |
| 0.02091 | NA | NA | + | NA | -9.59E-03 | NA | 6 | 126.849 | -241.699 | 1.285 | 0.178 |
| -0.00289 | 9.98E-11 | NA | + | NA | NA | NA | 6 | 126.552 | -241.104 | 1.881 | 0.132 |
| 0.00193 | NA | NA | + | NA | NA | -1.32E-03 | 6 | 126.502 | -241.003 | 1.981 | 0.125 |
| 0.00301 | NA | NA | + | NA | NA | NA | 5 | 124.156 | -238.312 | 0 | 0.343 |
| 0.02664 | NA | NA | + | NA | -1.05E-02 | NA | 6 | 124.604 | -237.209 | 1.103 | 0.198 |
| 0.01457 | NA | NA | + | 6.77E-05 | NA | NA | 6 | 124.57 | -237.14 | 1.172 | 0.191 |
| 0.01185 | NA | NA | + | NA | NA | -3.73E-03 | 6 | 124.227 | -236.454 | 1.858 | 0.136 |
| 0.00164 | 7.95E-11 | NA | + | NA | NA | NA | 6 | 124.199 | -236.398 | 1.914 | 0.132 |
| 0.00739 | NA | NA | + | NA | NA | NA | 5 | 121.928 | -233.856 | 0 | 0.374 |
| 0.01821 | NA | NA | + | 6.04E-05 | NA | NA | 6 | 122.241 | -232.482 | 1.374 | 0.188 |
| 0.01969 | NA | NA | + | NA | -5.47E-03 | NA | 6 | 122.04 | -232.08 | 1.776 | 0.154 |
| 0.00557 | 1.04E-10 | NA | + | NA | NA | NA | 6 | 121.982 | -231.964 | 1.892 | 0.145 |
| 0.00906 | NA | NA | + | NA | NA | -7.05E-04 | 6 | 121.931 | -231.861 | 1.995 | 0.138 |
| 0.0016 | NA | NA | + | NA | NA | NA | 5 | 124.443 | -238.886 | 0 | 0.357 |
| 0.02243 | NA | NA | + | NA | -9.20E-03 | NA | 6 | 124.828 | -237.656 | 1.231 | 0.193 |
| 0.00947 | NA | NA | + | 4.71E-05 | NA | NA | 6 | 124.664 | -237.328 | 1.558 | 0.164 |
| -0.0093 | NA | NA | + | NA | NA | 4.73E-03 | 6 | 124.582 | -237.164 | 1.722 | 0.151 |
| 0.00037 | 7.05E-11 | NA | + | NA | NA | NA | 6 | 124.477 | -236.954 | 1.932 | 0.136 |
| 0.0077 | NA | NA | + | NA | NA | NA | 5 | 128.633 | -247.266 | 0 | 0.363 |
| 0.01739 | NA | NA | + | 5.97E-05 | NA | NA | 6 | 128.966 | -245.931 | 1.334 | 0.186 |
| 0.02517 | NA | NA | + | NA | -7.96E-03 | NA | 6 | 128.909 | -245.817 | 1.448 | 0.176 |
| 0.00618 | 9.43E-11 | NA | + | NA | NA | NA | 6 | 128.687 | -245.373 | 1.893 | 0.141 |
| 0.00902 | NA | NA | + | NA | NA | -5.38E-04 | 6 | 128.634 | -245.269 | 1.997 | 0.134 |
| 0.00293 | NA | NA | + | NA | NA | NA | 5 | 116.102 | -222.204 | 0 | 0.35 |
| 0.01735 | NA | NA | + | 8.32E-05 | NA | NA | 6 | 116.652 | -221.304 | 0.9 | 0.223 |
| 0.02101 | NA | NA | + | NA | -8.00E-03 | NA | 6 | 116.333 | -220.666 | 1.538 | 0.162 |
| 0.00133 | 9.24E-11 | NA | + | NA | NA | NA | 6 | 116.146 | -220.292 | 1.912 | 0.135 |
| 0.00054 | NA | NA | + | NA | NA | 9.99E-04 | 6 | 116.107 | -220.214 | I.99 | 0.129 |
| 0.003 | NA | NA | + | NA | NA | NA | 5 | 126.409 | -242.819 | 0 | 0.262 |
| 0.02054 | NA | NA | + | 9.28E-05 | NA | NA | 6 | 127.16 | -242.319 | 0.499 | 0.204 |
| 0.02515 | NA | NA | + | NA | -9.67E-03 | NA | 6 | 126.759 | -241.518 | 01.III | 0.137 |
| 0.00099 | 1.19E-10 | NA | + | NA | NA | NA | 6 | 126.489 | -240.978 | 1.841 | 0.104 |
| 0.03918 | NA | NA | + | 8.80E-05 | -8.53E-03 | NA | 7 | 127.432 | -240.865 | 1.954 | 0.099 |
| 0.04853 | NA | NA | NA | 1.55E-04 | NA | NA | 3 | 123.419 | -240.837 | 1.981 | 0.097 |
| 0.00588 | NA | NA | + | NA | NA | -1.22E-03 | 6 | 126.417 | -240.834 | 1.984 | 0.097 |
| 0.00197 | NA | NA | + | NA | NA | NA | 5 | 122.879 | -235.758 | 0 | 0.346 |
| 0.02666 | NA | NA | + | NA | -1.09E-02 | NA | 6 | 123.402 | -234.804 | 0.954 | 0.215 |
| 0.01167 | NA | NA | + | 5.97E-05 | NA | NA | 6 | 123.217 | -234.435 | 1.324 | 0.178 |
| 0.0004 | 9.38E-11 | NA | + | NA | NA | NA | 6 | 122.927 | -233.853 | 1.905 | 0.133 |
| 0.00019 | NA | NA | + | NA | NA | 7.46E-04 | 6 | 122.882 | -233.764 | 1.994 | 0.128 |
| 0.00071 | NA | NA | + | NA | NA | NA | 5 | 132.265 | -254.53 | 0 | 0.35 |
| 0.02402 | NA | NA | + | NA | -1.02E-02 | NA | 6 | 132.769 | -253.538 | 0.993 | 0.213 |
| 0.01002 | NA | NA | + | 5.16E-05 | NA | NA | 6 | 132.543 | -253.085 | 1.445 | 0.17 |
| -0.0004 | 6.85E-11 | NA | + | NA | NA | NA | 6 | 132.308 | -252.615 | 1.915 | 0.134 |
| 0.00641 | NA | NA | + | NA | NA | -2.46E-03 | 6 | 132.297 | -252.594 | 1.936 | 0.133 |
| 0.00468 | NA | NA | + | NA | NA | NA | 5 | 120.533 | -231.066 | 0 | 0.327 |
| 0.01876 | NA | NA | + | 8.38E-05 | NA | NA | 6 | 121.156 | -230.313 | 0.753 | 0.224 |
| 0.02843 | NA | NA | + | NA | -1.06E-02 | NA | 6 | 121.007 | -230.014 | 1.053 | 0.193 |
| 0.00198 | 1.59E-10 | NA | + | NA | NA | NA | 6 | 120.657 | -229.315 | 1.751 | 0.136 |
| 0.00511 | NA | NA | + | NA | NA | -1.80E-04 | 6 | 120.533 | -229.066 | 2 | 0.12 |
| 0.00514 | NA | NA | + | NA | NA | NA | 5 | 128.134 | -246.268 | 0 | 0.346 |
| 0.02923 | NA | NA | + | NA | -1.07E-02 | NA | 6 | 128.681 | -245.362 | 0.907 | 0.22 |
| 0.01287 | NA | NA | + | 5.06E-05 | NA | NA | 6 | 128.413 | -244.825 | 1.443 | 0.168 |
| -0.00253 | NA | NA | + | NA | NA | 3.21E-03 | 6 | 128.195 | -244.39 | 1.878 | 0.135 |
| 0.00452 | 3.90E-11 | NA | + | NA | NA | NA | 6 | 128.151 | -244.302 | 1.966 | 0.13 |
| -0.00088 | NA | NA | + | NA | NA | NA | 5 | 115.374 | -220.748 | 0 | 0.302 |
| 0.01695 | NA | NA | + | 8.69E-05 | NA | NA | 6 | 115.897 | -219.793 | 0.955 | 0.188 |
| 0.04699 | NA | NA | NA | 1.54E-04 | NA | NA | 3 | 112.707 | -219.414 | 1.335 | 0.155 |
| 0.01434 | NA | NA | + | NA | -6.62E-03 | NA | 6 | 115.508 | -219.016 | 1.732 | 0.127 |
| 0.00447 | NA | NA | + | NA | NA | -2.28E-03 | 6 | 115.401 | -218.801 | 1.947 | 0.114 |
| -0.00196 | 5.82E-11 | NA | + | NA | NA | NA | 6 | 115.397 | -218.794 | 1.955 | 0.114 |
| 0.00541 | NA | NA | + | NA | NA | NA | 5 | 123.091 | -236.182 | 0 | 0.36 |
| 0.01687 | NA | NA | + | 6.72E-05 | NA | NA | 6 | 123.499 | -234.998 | 1.184 | 0.199 |
| 0.0223 | NA | NA | + | NA | -7.45E-03 | NA | 6 | 123.317 | -234.634 | 1.549 | 0.166 |
| 0.00361 | 1.05E-10 | NA | + | NA | NA | NA | 6 | 123.153 | -234.305 | 1.877 | 0.141 |
| 0.00698 | NA | NA | + | NA | NA | -6.69E-04 | 6 | 123.093 | -234.187 | 1.995 | 0.133 |
| 0.00725 | NA | NA | + | NA | NA | NA | 5 | 124.114 | -238.229 | 0 | 0.31 |
| 0.02262 | NA | NA | + | 8.39E-05 | NA | NA | 6 | 124.681 | -237.363 | 0.866 | 0.201 |
| 0.02371 | NA | NA | + | NA | -7.11E-03 | NA | 6 | 124.29 | -236.58 | 1.649 | 0.136 |
| 0.0479 | NA | NA | NA | 1.37E-04 | NA | NA | 3 | 121.163 | -236.325 | 1.904 | 0.12 |
| 0.00582 | 8.09E-11 | NA | + | NA | NA | NA | 6 | 124.149 | -236.298 | 1.931 | 0.118 |
| 0.00765 | NA | NA | + | NA | NA | -1.69E-04 | 6 | 124.115 | -236.229 | 2 | 0.114 |
| 0.00089 | NA | NA | + | NA | NA | NA | 5 | 123.243 | -236.485 | 0 | 0.364 |
| 0.01948 | NA | NA | + | NA | -8.08E-03 | NA | 6 | 123.552 | -235.105 | I.38 | 0.182 |
| 0.00887 | NA | NA | + | 5.01E-05 | NA | NA | 6 | 123.485 | -234.971 | 1.514 | 0.171 |
| -0.00716 | NA | NA | + | NA | NA | 3.50E-03 | 6 | 123.312 | -234.623 | 1.862 | 0.143 |
| -0.00067 | 8.67E-11 | NA | + | NA | NA | NA | 6 | 123.288 | -234.577 | 1.908 | 0.14 |
| 0.00267 | NA | NA | + | NA | NA | NA | 5 | 122.315 | -234.631 | 0 | 0.365 |
| 0.02212 | NA | NA | + | NA | -8.66E-03 | NA | 6 | 122.633 | -233.265 | 1.365 | 0.185 |
| 0.01107 | NA | NA | + | 5.13E-05 | NA | NA | 6 | 122.552 | -233.104 | 1.527 | 0.17 |
| 0.00058 | 1.22E-10 | NA | + | NA | NA | NA | 6 | 122.39 | -232.78 | I.85 | 0.145 |
| 0.0048 | NA | NA | + | NA | NA | -9.01E-04 | 6 | 122.319 | -232.639 | 1.992 | 0.135 |
| 0.00191 | NA | NA | + | NA | NA | NA | 5 | 122.729 | -235.459 | 0 | 0.278 |
| 0.01829 | NA | NA | + | 9.33E-05 | NA | NA | 6 | 123.52 | -235.04 | 0.418 | 0.225 |
| 0.0253 | NA | NA | + | NA | -1.05E-02 | NA | 6 | 123.204 | -234.407 | 1.051 | 0.164 |
| -0.00055 | 1.51E-10 | NA | + | NA | NA | NA | 6 | 122.852 | -233.704 | 1.755 | 0.115 |
| 0.03616 | NA | NA | + | 8.46E-05 | -8.70E-03 | NA | 7 | 123.843 | -233.686 | 1.773 | 0.114 |
| 0.00601 | NA | NA | + | NA | NA | -1.69E-03 | 6 | 122.743 | -233.486 | 1.972 | 0.104 |
| -0.00519 | NA | NA | + | NA | NA | NA | 5 | 126.67 | -243.339 | 0 | 0.282 |
| 0.02435 | NA | NA | + | NA | -1.29E-02 | NA | 6 | 127.513 | -243.026 | 0.313 | 0.241 |
| 0.00426 | NA | NA | + | 5.71E-05 | NA | NA | 6 | 127.019 | -242.038 | 1.301 | 0.147 |
| -0.00716 | 1.15E-10 | NA | + | NA | NA | NA | 6 | 126.783 | -241.565 | 1.774 | 0.116 |
| 0.02909 | NA | NA | + | 4.34E-05 | -1.18E-02 | NA | 7 | 127.711 | -241.423 | 1.916 | 0.108 |
| -0.00854 | NA | NA | + | NA | NA | 1.45E-03 | 6 | 126.681 | -241.363 | 1.976 | 0.105 |
| 0.00046 | NA | NA | + | NA | NA | NA | 5 | 120.227 | -230.454 | 0 | 0.348 |
| 0.0153 | NA | NA | + | 8.04E-05 | NA | NA | 6 | 120.75 | -229.5 | 0.953 | 0.216 |
| 0.02034 | NA | NA | + | NA | -8.61E-03 | NA | 6 | 120.501 | -229.003 | 1.451 | 0.168 |
| -0.00123 | 9.75E-11 | NA | + | NA | NA | NA | 6 | 120.273 | -228.546 | 1.908 | 0.134 |
| 0.00737 | NA | NA | + | NA | NA | -2.93E-03 | 6 | 120.269 | -228.538 | 1.916 | 0.134 |
| 0.00027 | NA | NA | + | NA | NA | NA | 5 | 127.902 | -245.805 | 0 | 0.327 |
| 0.02901 | NA | NA | + | NA | -1.25E-02 | NA | 6 | 128.578 | -245.157 | 0.648 | 0.236 |
| 0.01153 | NA | NA | + | 6.32E-05 | NA | NA | 6 | 128.289 | -244.579 | 1.226 | 0.177 |
| 0.01045 | NA | NA | + | NA | NA | -4.35E-03 | 6 | 127.999 | -243.998 | 1.807 | 0.132 |
| -0.00119 | 8.96E-11 | NA | + | NA | NA | NA | 6 | 127.958 | -243.916 | 1.889 | 0.127 |
| 0.00128 | NA | NA | + | NA | NA | NA | 5 | 125.32 | -240.64 | 0 | 0.337 |
| 0.02462 | NA | NA | + | NA | -1.04E-02 | NA | 6 | 125.902 | -239.804 | 0.836 | 0.222 |
| 0.00946 | NA | NA | + | 5.38E-05 | NA | NA | 6 | 125.634 | -239.269 | 1.371 | 0.17 |
| -0.00105 | 1.35E-10 | NA | + | NA | NA | NA | 6 | 125.42 | -238.841 | 1.799 | 0.137 |
| -0.00789 | NA | NA | + | NA | NA | 3.88E-03 | 6 | 125.404 | -238.807 | 1.833 | 0.135 |
| 0.04678 | NA | NA | NA | 1.68E-04 | NA | NA | 3 | 116.794 | -227.589 | 0 | 0.451 |
| 0.05893 | NA | NA | NA | 1.59E-04 | -6.36E-03 | NA | 4 | 116.931 | -225.862 | 1.726 | 0.19 |
| 0.05851 | NA | NA | NA | 1.76E-04 | NA | -4.14E-03 | 4 | 116.872 | -225.744 | 1.845 | 0.179 |
| 0.04467 | 1.38E-10 | NA | NA | 1.68E-04 | NA | NA | 4 | 116.871 | -225.743 | 1.846 | 0.179 |
| 0.00098 | NA | NA | + | NA | NA | NA | 5 | 119.64 | -229.281 | 0 | 0.363 |
| 0.01057 | NA | NA | + | 5.85E-05 | NA | NA | 6 | 119.955 | -227.91 | 1.371 | 0.183 |
| 0.01891 | NA | NA | + | NA | -8.00E-03 | NA | 6 | 119.934 | -227.868 | 1.412 | 0.179 |
| -0.00062 | 9.50E-11 | NA | + | NA | NA | NA | 6 | 119.688 | -227.375 | 1.906 | 0.14 |
| -0.00286 | NA | NA | + | NA | NA | 1.63E-03 | 6 | 119.653 | -227.307 | 1.974 | 0.135 |
| 0.00515 | NA | NA | + | NA | NA | NA | 5 | 121.238 | -232.475 | 0 | 0.376 |
| 0.02135 | NA | NA | + | NA | -7.24E-03 | NA | 6 | 121.455 | -230.911 | 1.564 | 0.172 |
| 0.01163 | NA | NA | + | 4.00E-05 | NA | NA | 6 | 121.383 | -230.767 | 1.709 | 0.16 |
| -0.00433 | NA | NA | + | NA | NA | 4.00E-03 | 6 | 121.332 | -230.663 | 1.812 | 0.152 |
| 0.00432 | 4.67E-11 | NA | + | NA | NA | NA | 6 | 121.249 | -230.498 | 1.977 | 0.14 |
| 0.00453 | NA | NA | + | NA | NA | NA | 5 | 124.637 | -239.274 | 0 | 0.37 |
| 0.01591 | NA | NA | + | 6.27E-05 | NA | NA | 6 | 124.969 | -237.937 | 1.336 | 0.19 |
| 0.01908 | NA | NA | + | NA | -6.42E-03 | NA | 6 | 124.797 | -237.594 | 1.679 | 0.16 |
| 0.0031 | 8.13E-11 | NA | + | NA | NA | NA | 6 | 124.675 | -237.349 | 1.925 | 0.141 |
| 0.0003 | NA | NA | + | NA | NA | 1.79E-03 | 6 | 124.655 | -237.31 | 1.964 | 0.139 |
| 0.00567 | NA | NA | + | NA | NA | NA | 5 | 121.934 | -233.869 | 0 | 0.373 |
| 0.01691 | NA | NA | + | 6.18E-05 | NA | NA | 6 | 122.252 | -232.504 | 1.365 | 0.189 |
| 0.02025 | NA | NA | + | NA | -6.53E-03 | NA | 6 | 122.093 | -232.185 | 1.684 | 0.161 |
| 0.00473 | 5.43E-11 | NA | + | NA | NA | NA | 6 | 121.952 | -231.904 | 1.965 | 0.14 |
| 0.00768 | NA | NA | + | NA | NA | -8.34E-04 | 6 | 121.938 | -231.876 | 1.993 | 0.138 |
| 0.00561 | NA | NA | + | NA | NA | NA | 5 | 126.321 | -242.641 | 0 | 0.348 |
| 0.01791 | NA | NA | + | 7.47E-05 | NA | NA | 6 | 126.846 | -241.693 | 0.949 | 0.216 |
| 0.02407 | NA | NA | + | NA | -8.24E-03 | NA | 6 | 126.615 | -241.229 | 1.412 | 0.172 |
| 0.00435 | 7.60E-11 | NA | + | NA | NA | NA | 6 | 126.364 | -240.727 | 1.914 | 0.134 |
| 0.01064 | NA | NA | + | NA | NA | -2.07E-03 | 6 | 126.342 | -240.685 | 1.957 | 0.131 |
| 0.00335 | NA | NA | + | NA | NA | NA | 5 | 122.456 | -234.911 | 0 | 0.344 |
| 0.01696 | NA | NA | + | 7.67E-05 | NA | NA | 6 | 122.974 | -233.948 | 0.963 | 0.212 |
| 0.02644 | NA | NA | + | NA | -1.02E-02 | NA | 6 | 122.845 | -233.69 | 1.221 | 0.187 |
| 0.00246 | 5.34E-11 | NA | + | NA | NA | NA | 6 | 122.487 | -232.974 | 1.938 | 0.13 |
| 0.00172 | NA | NA | + | NA | NA | 6.73E-04 | 6 | 122.458 | -232.916 | 1.995 | 0.127 |
| 0.00228 | NA | NA | + | NA | NA | NA | 5 | 121.231 | -232.463 | 0 | 0.337 |
| 0.01826 | NA | NA | + | 9.17E-05 | NA | NA | 6 | 121.962 | -231.924 | 0.539 | 0.258 |
| 0.01692 | NA | NA | + | NA | -6.46E-03 | NA | 6 | 121.4 | -230.8 | 1.662 | 0.147 |
| 0.00063 | 9.74E-11 | NA | + | NA | NA | NA | 6 | 121.295 | -230.589 | 1.873 | 0.132 |
| 0.00614 | NA | NA | + | NA | NA | -1.61E-03 | 6 | 121.243 | -230.487 | 1.976 | 0.126 |
| 0.00533 | NA | NA | + | NA | NA | NA | 5 | 124.37 | -238.739 | 0 | 0.353 |
| 0.01828 | NA | NA | + | 7.61E-05 | NA | NA | 6 | 124.899 | -237.798 | 0.941 | 0.22 |
| 0.01823 | NA | NA | + | NA | -5.73E-03 | NA | 6 | 124.507 | -237.014 | 1.725 | 0.149 |
| -0.00359 | NA | NA | + | NA | NA | 3.76E-03 | 6 | 124.448 | -236.897 | 1.842 | 0.14 |
| 0.00349 | 1.06E-10 | NA | + | NA | NA | NA | 6 | 124.426 | -236.851 | 1.888 | 0.137 |
| 0.0048 | NA | NA | + | NA | NA | NA | 5 | 123.468 | -236.937 | 0 | 0.355 |
| 0.01815 | NA | NA | + | 7.92E-05 | NA | NA | 6 | 124.015 | -236.029 | 0.908 | 0.225 |
| 0.01982 | NA | NA | + | NA | -6.69E-03 | NA | 6 | 123.641 | -235.283 | 1.654 | 0.155 |
| 0.0039 | 5.56E-11 | NA | + | NA | NA | NA | 6 | 123.496 | -234.993 | 1.944 | 0.134 |
| 0.00376 | NA | NA | + | NA | NA | 4.28E-04 | 6 | 123.469 | -234.939 | 1.998 | 0.131 |
| 0.0023 | NA | NA | + | NA | NA | NA | 5 | 116.996 | -223.992 | 0 | 0.302 |
| 0.01971 | NA | NA | + | 8.32E-05 | NA | NA | 6 | 117.482 | -222.963 | 1.028 | 0.181 |
| 0.04892 | NA | NA | NA | 1.45E-04 | NA | NA | 3 | 114.362 | -222.725 | 1.267 | 0.16 |
| 0.01305 | NA | NA | + | NA | NA | -4.60E-03 | 6 | 117.101 | -222.201 | I.79 | 0.123 |
| 0.01502 | NA | NA | + | NA | -5.41E-03 | NA | 6 | 117.078 | -222.157 | 1.835 | 0.121 |
| 0.0016 | 3.98E-11 | NA | + | NA | NA | NA | 6 | 117.008 | -222.017 | 1.975 | 0.113 |
| 0.00153 | NA | NA | + | NA | NA | NA | 5 | 121.876 | -233.752 | 0 | 0.352 |
| 0.01668 | NA | NA | + | 7.92E-05 | NA | NA | 6 | 122.384 | -232.768 | 0.984 | 0.215 |
| 0.02169 | NA | NA | + | NA | -8.68E-03 | NA | 6 | 122.137 | -232.274 | 1.478 | 0.168 |
| 0.00025 | 6.92E-11 | NA | + | NA | NA | NA | 6 | 121.908 | -231.815 | 1.936 | 0.134 |
| 0.0036 | NA | NA | + | NA | NA | -8.87E-04 | 6 | 121.88 | -231.76 | 1.992 | 0.13 |
| 0.00302 | NA | NA | + | NA | NA | NA | 5 | 119.902 | -229.805 | 0 | 0.301 |
| 0.01813 | NA | NA | + | 8.08E-05 | NA | NA | 6 | 120.428 | -228.857 | 0.948 | 0.187 |
| 0.02527 | NA | NA | + | NA | -9.76E-03 | NA | 6 | 120.233 | -228.466 | 1.339 | 0.154 |
| 0.01533 | NA | NA | + | NA | NA | -5.15E-03 | 6 | 120.025 | -228.051 | 1.754 | 0.125 |
| 0.04741 | NA | NA | NA | 1.46E-04 | NA | NA | 3 | 116.959 | -227.918 | 1.886 | 0.117 |
| 0.00169 | 7.30E-11 | NA | + | NA | NA | NA | 6 | 119.942 | -227.885 | I.92 | 0.115 |
| 0.00429 | NA | NA | + | NA | NA | NA | 5 | 126.527 | -243.053 | 0 | 0.345 |
| 0.01748 | NA | NA | + | 7.78E-05 | NA | NA | 6 | 127.081 | -242.163 | 0.891 | 0.221 |
| 0.02308 | NA | NA | + | NA | -8.25E-03 | NA | 6 | 126.814 | -241.629 | 1.425 | 0.169 |
| 0.01111 | NA | NA | + | NA | NA | -2.86E-03 | 6 | 126.567 | -241.133 | I.92 | 0.132 |
| 0.00315 | 6.81E-11 | NA | + | NA | NA | NA | 6 | 126.561 | -241.122 | 1.932 | 0.132 |
| 0.00383 | NA | NA | + | NA | NA | NA | 5 | 127.492 | -244.985 | 0 | 0.337 |
| 0.01908 | NA | NA | + | 8.07E-05 | NA | NA | 6 | 128.049 | -244.098 | 0.887 | 0.216 |
| 0.0267 | NA | NA | + | NA | -1.00E-02 | NA | 6 | 127.882 | -243.763 | 1.222 | 0.183 |
| 0.00195 | 1.13E-10 | NA | + | NA | NA | NA | 6 | 127.565 | -243.13 | 1.854 | 0.133 |
| 0.01222 | NA | NA | + | NA | NA | -3.54E-03 | 6 | 127.554 | -243.108 | 1.877 | 0.132 |
| 0.00004 | NA | NA | + | NA | NA | NA | 5 | 129.126 | -248.251 | 0 | 0.329 |
| 0.02482 | NA | NA | + | NA | -1.11E-02 | NA | 6 | 129.883 | -247.767 | 0.485 | 0.258 |
| 0.00587 | NA | NA | + | 3.76E-05 | NA | NA | 6 | 129.291 | -246.581 | I.67 | 0.143 |
| -0.01227 | NA | NA | + | NA | NA | 5.21E-03 | 6 | 129.285 | -246.571 | 1.681 | 0.142 |
| -0.00177 | 1.03E-10 | NA | + | NA | NA | NA | 6 | 129.188 | -246.377 | 1.875 | 0.129 |
| 0.00165 | NA | NA | + | NA | NA | NA | 5 | 129.47 | -248.939 | 0 | 0.339 |
| 0.02708 | NA | NA | + | NA | -1.14E-02 | NA | 6 | 130.138 | -248.277 | 0.662 | 0.244 |
| 0.00917 | NA | NA | + | 4.51E-05 | NA | NA | 6 | 129.691 | -247.382 | 1.557 | 0.156 |
| -0.00042 | 1.17E-10 | NA | + | NA | NA | NA | 6 | 129.543 | -247.086 | 1.854 | 0.134 |
| -0.00213 | NA | NA | + | NA | NA | 1.60E-03 | 6 | 129.484 | -246.968 | 1.971 | 0.127 |
| 0.00261 | NA | NA | + | NA | NA | NA | 5 | 128.454 | -246.908 | 0 | 0.361 |
| 0.0152 | NA | NA | + | 7.05E-05 | NA | NA | 6 | 128.912 | -245.824 | 1.084 | 0.21 |
| 0.01811 | NA | NA | + | NA | -6.77E-03 | NA | 6 | 128.634 | -245.268 | I.64 | 0.159 |
| 0.00154 | 6.33E-11 | NA | + | NA | NA | NA | 6 | 128.486 | -244.972 | 1.936 | 0.137 |
| 0.00223 | NA | NA | + | NA | NA | 1.62E-04 | 6 | 128.454 | -244.908 | 2 | 0.133 |
| 0.02262 | NA | NA | + | 1.17E-04 | NA | NA | 6 | 121.298 | -230.595 | 0 | 0.239 |
| 0.00045 | NA | NA | + | NA | NA | NA | 5 | 120.214 | -230.428 | 0.167 | 0.219 |
| 0.05131 | NA | NA | NA | 1.80E-04 | NA | NA | 3 | 117.791 | -229.582 | 1.013 | 0.144 |
| 0.02199 | NA | NA | + | NA | -9.39E-03 | NA | 6 | 120.515 | -229.029 | 1.566 | 0.109 |
| 0.03843 | NA | NA | + | 1.11E-04 | -7.37E-03 | NA | 7 | 121.482 | -228.965 | I.63 | 0.106 |
| 0.02083 | 8.90E-11 | NA | + | 1.16E-04 | NA | NA | 7 | 121.349 | -228.697 | 1.898 | 0.092 |
| 0.02996 | NA | NA | + | 1.20E-04 | NA | -2.80E-03 | 7 | 121.337 | -228.675 | I.92 | 0.091 |
| 0.00303 | NA | NA | + | NA | NA | NA | 5 | 127.748 | -245.496 | 0 | 0.372 |
| 0.02074 | NA | NA | + | NA | -7.83E-03 | NA | 6 | 128.003 | -244.007 | 1.489 | 0.177 |
| 0.01114 | NA | NA | + | 4.79E-05 | NA | NA | 6 | 127.967 | -243.935 | 1.561 | 0.17 |
| 0.00168 | 7.22E-11 | NA | + | NA | NA | NA | 6 | 127.78 | -243.56 | 1.936 | 0.141 |
| 0.0076 | NA | NA | + | NA | NA | -1.96E-03 | 6 | 127.768 | -243.536 | I.96 | 0.14 |
| 0.00238 | NA | NA | + | NA | NA | NA | 5 | 121.938 | -233.875 | 0 | 0.327 |
| 0.02033 | NA | NA | + | 9.46E-05 | NA | NA | 6 | 122.659 | -233.317 | 0.558 | 0.248 |
| 0.02411 | NA | NA | + | NA | -9.39E-03 | NA | 6 | 122.248 | -232.496 | I.38 | 0.164 |
| 0.01291 | NA | NA | + | NA | NA | -4.42E-03 | 6 | 122.028 | -232.057 | 1.819 | 0.132 |
| 0.00059 | 1.08E-10 | NA | + | NA | NA | NA | 6 | 122.01 | -232.019 | 1.856 | 0.129 |
| 0.00393 | NA | NA | + | NA | NA | NA | 5 | 123.35 | -236.7 | 0 | 0.348 |
| 0.02257 | NA | NA | + | NA | -8.34E-03 | NA | 6 | 123.702 | -235.404 | 1.295 | 0.182 |
| 0.01221 | NA | NA | + | 5.60E-05 | NA | NA | 6 | 123.671 | -235.342 | 1.357 | 0.177 |
| -0.00973 | NA | NA | + | NA | NA | 5.71E-03 | 6 | 123.539 | -235.077 | 1.623 | 0.155 |
| 0.00201 | 1.17E-10 | NA | + | NA | NA | NA | 6 | 123.424 | -234.849 | 1.851 | 0.138 |
| 0.00308 | NA | NA | + | NA | NA | NA | 5 | 126.514 | -243.027 | 0 | 0.354 |
| 0.01591 | NA | NA | + | 7.40E-05 | NA | NA | 6 | 127.025 | -242.051 | 0.977 | 0.217 |
| 0.01918 | NA | NA | + | NA | -7.17E-03 | NA | 6 | 126.737 | -241.474 | 1.553 | 0.163 |
| 0.00179 | 7.98E-11 | NA | + | NA | NA | NA | 6 | 126.562 | -241.123 | 1.904 | 0.136 |
| 0.00232 | NA | NA | + | NA | NA | 3.20E-04 | 6 | 126.514 | -241.029 | 1.999 | 0.13 |
| 0.00194 | NA | NA | + | NA | NA | NA | 5 | 126.084 | -242.168 | 0 | 0.35 |
| 0.01577 | NA | NA | + | 7.90E-05 | NA | NA | 6 | 126.684 | -241.368 | 0.8 | 0.234 |
| 0.01578 | NA | NA | + | NA | -6.12E-03 | NA | 6 | 126.246 | -240.491 | 1.677 | 0.151 |
| 0.0006 | 7.61E-11 | NA | + | NA | NA | NA | 6 | 126.117 | -240.234 | 1.934 | 0.133 |
| -0.00297 | NA | NA | + | NA | NA | 2.09E-03 | 6 | 126.109 | -240.218 | I.95 | 0.132 |
| 0.00294 | NA | NA | + | NA | NA | NA | 5 | 125.9 | -241.799 | 0 | 0.365 |
| 0.02297 | NA | NA | + | NA | -8.73E-03 | NA | 6 | 126.243 | -240.485 | 1.314 | 0.189 |
| 0.01107 | NA | NA | + | 4.88E-05 | NA | NA | 6 | 126.13 | -240.261 | 1.538 | 0.169 |
| 0.00173 | 6.87E-11 | NA | + | NA | NA | NA | 6 | 125.932 | -239.864 | 1.935 | 0.139 |
| -0.00226 | NA | NA | + | NA | NA | 2.25E-03 | 6 | 125.928 | -239.856 | 1.943 | 0.138 |
| -0.00139 | NA | NA | + | NA | NA | NA | 5 | 121.088 | -232.176 | 0 | 0.349 |
| 0.01176 | NA | NA | + | 6.94E-05 | NA | NA | 6 | 121.489 | -230.977 | 1.199 | 0.192 |
| 0.02188 | NA | NA | + | NA | -9.91E-03 | NA | 6 | 121.486 | -230.973 | 1.204 | 0.191 |
| 0.00535 | NA | NA | + | NA | NA | -2.92E-03 | 6 | 121.13 | -230.26 | 1.916 | 0.134 |
| -0.0029 | 8.06E-11 | NA | + | NA | NA | NA | 6 | 121.126 | -230.253 | 1.924 | 0.134 |
| 0.00081 | NA | NA | + | NA | NA | NA | 5 | 127.318 | -244.635 | 0 | 0.327 |
| 0.01638 | NA | NA | + | 8.60E-05 | NA | NA | 6 | 127.999 | -243.999 | 0.637 | 0.238 |
| 0.02294 | NA | NA | + | NA | -9.82E-03 | NA | 6 | 127.691 | -243.382 | 1.254 | 0.174 |
| 0.01126 | NA | NA | + | NA | NA | -4.33E-03 | 6 | 127.408 | -242.817 | 1.818 | 0.132 |
| -0.00109 | 1.15E-10 | NA | + | NA | NA | NA | 6 | 127.395 | -242.791 | 1.845 | 0.13 |
| 0.00107 | NA | NA | + | NA | NA | NA | 5 | 120.482 | -230.965 | 0 | 0.337 |
| 0.01688 | NA | NA | + | 7.85E-05 | NA | NA | 6 | 120.96 | -229.921 | 1.044 | 0.2 |
| 0.02734 | NA | NA | + | NA | -1.14E-02 | NA | 6 | 120.897 | -229.793 | 1.171 | 0.187 |
| 0.01589 | NA | NA | + | NA | NA | -6.28E-03 | 6 | 120.672 | -229.345 | I.62 | 0.15 |
| 0.00006 | 5.44E-11 | NA | + | NA | NA | NA | 6 | 120.505 | -229.01 | 1.955 | 0.127 |
| 0.00456 | NA | NA | + | NA | NA | NA | 5 | 122.366 | -234.732 | 0 | 0.37 |
| 0.02354 | NA | NA | + | NA | -8.49E-03 | NA | 6 | 122.666 | -233.332 | 01.IV | 0.184 |
| 0.01222 | NA | NA | + | 4.71E-05 | NA | NA | 6 | 122.564 | -233.129 | 1.603 | 0.166 |
| 0.00318 | 8.00E-11 | NA | + | NA | NA | NA | 6 | 122.409 | -232.819 | 1.913 | 0.142 |
| 0.00211 | NA | NA | + | NA | NA | 1.02E-03 | 6 | 122.371 | -232.742 | 1.989 | 0.137 |
| 0.00243 | NA | NA | + | NA | NA | NA | 5 | 131.126 | -252.253 | 0 | 0.347 |
| 0.02777 | NA | NA | + | NA | -1.14E-02 | NA | 6 | 131.749 | -251.499 | 0.754 | 0.238 |
| 0.00961 | NA | NA | + | 4.22E-05 | NA | NA | 6 | 131.315 | -250.63 | 1.623 | 0.154 |
| 0.00122 | 7.14E-11 | NA | + | NA | NA | NA | 6 | 131.161 | -250.321 | 1.931 | 0.132 |
| 0.00235 | NA | NA | + | NA | NA | 3.39E-05 | 6 | 131.126 | -250.253 | 2 | 0.128 |
| 0.00441 | NA | NA | + | NA | NA | NA | 5 | 120.694 | -231.388 | 0 | 0.259 |
| 0.01957 | NA | NA | + | 9.28E-05 | NA | NA | 6 | 121.481 | -230.963 | 0.425 | 0.21 |
| 0.0255 | NA | NA | + | NA | -9.44E-03 | NA | 6 | 121.061 | -230.122 | 1.265 | 0.138 |
| 0.00297 | 9.28E-11 | NA | + | NA | NA | NA | 6 | 120.754 | -229.509 | 1.879 | 0.101 |
| 0.04893 | NA | NA | NA | 1.63E-04 | NA | NA | 3 | 117.723 | -229.447 | 1.941 | 0.098 |
| 0.03525 | NA | NA | + | 8.53E-05 | -7.57E-03 | NA | 7 | 121.715 | -229.429 | 1.958 | 0.097 |
| 0.00708 | NA | NA | + | NA | NA | -1.07E-03 | 6 | 120.699 | -229.399 | 1.989 | 0.096 |
| 0.00422 | NA | NA | + | NA | NA | NA | 5 | 126.725 | -243.451 | 0 | 0.341 |
| 0.02724 | NA | NA | + | NA | -1.03E-02 | NA | 6 | 127.213 | -242.426 | 1.025 | 0.204 |
| 0.01497 | NA | NA | + | 6.62E-05 | NA | NA | 6 | 127.171 | -242.341 | 01.XI | 0.196 |
| 0.00268 | 9.68E-11 | NA | + | NA | NA | NA | 6 | 126.795 | -241.59 | 1.861 | 0.134 |
| 0.00343 | NA | NA | + | NA | NA | 3.31E-04 | 6 | 126.726 | -241.452 | 1.999 | 0.125 |
| 0.00383 | NA | NA | + | NA | NA | NA | 5 | 125.969 | -241.938 | 0 | 0.352 |
| 0.0172 | NA | NA | + | 7.98E-05 | NA | NA | 6 | 126.574 | -241.148 | 0.79 | 0.237 |
| 0.01612 | NA | NA | + | NA | -5.44E-03 | NA | 6 | 126.091 | -240.182 | 1.756 | 0.146 |
| 0.00263 | 7.01E-11 | NA | + | NA | NA | NA | 6 | 126.004 | -240.009 | I.93 | 0.134 |
| 0.00674 | NA | NA | + | NA | NA | -1.23E-03 | 6 | 125.977 | -239.954 | 1.984 | 0.13 |
| 0.00492 | NA | NA | + | NA | NA | NA | 5 | 122.974 | -235.949 | 0 | 0.358 |
| 0.02681 | NA | NA | + | NA | -9.67E-03 | NA | 6 | 123.369 | -234.738 | 1.211 | 0.195 |
| 0.01522 | NA | NA | + | 5.87E-05 | NA | NA | 6 | 123.286 | -234.572 | 1.377 | 0.18 |
| 0.00375 | 6.61E-11 | NA | + | NA | NA | NA | 6 | 123 | -234.001 | 1.948 | 0.135 |
| 0.0073 | NA | NA | + | NA | NA | -1.02E-03 | 6 | 122.98 | -233.96 | 1.989 | 0.132 |
| 0.00328 | NA | NA | + | NA | NA | NA | 5 | 124.126 | -238.252 | 0 | 0.35 |
| 0.0261 | NA | NA | + | NA | -1.03E-02 | NA | 6 | 124.578 | -237.157 | 1.096 | 0.202 |
| 0.01306 | NA | NA | + | 6.13E-05 | NA | NA | 6 | 124.482 | -236.965 | 1.288 | 0.184 |
| 0.0024 | 5.22E-11 | NA | + | NA | NA | NA | 6 | 124.152 | -236.304 | 1.948 | 0.132 |
| 0.0082 | NA | NA | + | NA | NA | -2.01E-03 | 6 | 124.147 | -236.294 | 1.959 | 0.131 |
| 0.00039 | NA | NA | + | NA | NA | NA | 5 | 120.749 | -231.499 | 0 | 0.359 |
| 0.01272 | NA | NA | + | 6.79E-05 | NA | NA | 6 | 121.155 | -230.31 | 1.189 | 0.198 |
| 0.01868 | NA | NA | + | NA | -8.04E-03 | NA | 6 | 121.016 | -230.032 | 1.466 | 0.172 |
| -0.00131 | 9.77E-11 | NA | + | NA | NA | NA | 6 | 120.797 | -229.593 | 1.906 | 0.138 |
| 0.00198 | NA | NA | + | NA | NA | -6.70E-04 | 6 | 120.752 | -229.503 | 1.996 | 0.132 |
| 0.0047 | NA | NA | + | NA | NA | NA | 5 | 127.769 | -245.538 | 0 | 0.281 |
| 0.0347 | NA | NA | + | NA | -1.35E-02 | NA | 6 | 128.715 | -245.43 | 0.108 | 0.267 |
| 0.01234 | NA | NA | + | 4.83E-05 | NA | NA | 6 | 128.018 | -244.037 | 1.501 | 0.133 |
| 0.03768 | NA | NA | + | 3.12E-05 | -1.26E-02 | NA | 7 | 128.816 | -243.632 | 1.905 | 0.108 |
| 0.00356 | 6.78E-11 | NA | + | NA | NA | NA | 6 | 127.804 | -243.609 | 1.929 | 0.107 |
| 0.00341 | NA | NA | + | NA | NA | 5.33E-04 | 6 | 127.77 | -243.541 | 1.997 | 0.104 |
| -0.00021 | NA | NA | + | NA | NA | NA | 5 | 125.732 | -241.465 | 0 | 0.358 |
| 0.02197 | NA | NA | + | NA | -9.62E-03 | NA | 6 | 126.175 | -240.35 | 1.115 | 0.205 |
| 0.00818 | NA | NA | + | 4.94E-05 | NA | NA | 6 | 125.972 | -239.944 | 1.521 | 0.167 |
| -0.00144 | 6.95E-11 | NA | + | NA | NA | NA | 6 | 125.769 | -239.538 | 1.927 | 0.137 |
| -0.00314 | NA | NA | + | NA | NA | 1.27E-03 | 6 | 125.741 | -239.482 | 1.982 | 0.133 |
| 0.0054 | NA | NA | + | NA | NA | NA | 5 | 120.997 | -231.994 | 0 | 0.3 |
| 0.02156 | NA | NA | + | 8.82E-05 | NA | NA | 6 | 121.641 | -231.282 | 0.712 | 0.21 |
| 0.02445 | NA | NA | + | NA | -8.48E-03 | NA | 6 | 121.252 | -230.503 | 1.491 | 0.143 |
| 0.04656 | NA | NA | NA | 1.43E-04 | NA | NA | 3 | 118.046 | -230.091 | 1.903 | 0.116 |
| 0.01302 | NA | NA | + | NA | NA | -3.16E-03 | 6 | 121.045 | -230.089 | 1.905 | 0.116 |
| 0.00418 | 6.86E-11 | NA | + | NA | NA | NA | 6 | 121.031 | -230.063 | 1.931 | 0.114 |
| 0.00436 | NA | NA | + | NA | NA | NA | 5 | 122.795 | -235.59 | 0 | 0.359 |
| 0.01696 | NA | NA | + | 7.41E-05 | NA | NA | 6 | 123.25 | -234.501 | 1.089 | 0.208 |
| 0.01975 | NA | NA | + | NA | -6.83E-03 | NA | 6 | 122.974 | -233.948 | 1.642 | 0.158 |
| 0.00301 | 8.17E-11 | NA | + | NA | NA | NA | 6 | 122.843 | -233.686 | 1.904 | 0.138 |
| 0.01139 | NA | NA | + | NA | NA | -2.89E-03 | 6 | 122.834 | -233.668 | 1.922 | 0.137 |
| 0.00458 | NA | NA | + | NA | NA | NA | 5 | 127.506 | -245.012 | 0 | 0.35 |
| 0.02595 | NA | NA | + | NA | -9.78E-03 | NA | 6 | 128.01 | -244.019 | 0.993 | 0.213 |
| 0.01083 | NA | NA | + | 4.09E-05 | NA | NA | 6 | 127.693 | -243.386 | 1.626 | 0.155 |
| -0.00698 | NA | NA | + | NA | NA | 4.85E-03 | 6 | 127.646 | -243.292 | 1.721 | 0.148 |
| 0.00332 | 7.47E-11 | NA | + | NA | NA | NA | 6 | 127.551 | -243.101 | 1.911 | 0.134 |
| 0.00439 | NA | NA | + | NA | NA | NA | 5 | 115.245 | -220.49 | 0 | 0.32 |
| 0.01866 | NA | NA | + | 7.71E-05 | NA | NA | 6 | 115.683 | -219.367 | 1.123 | 0.182 |
| 0.02155 | NA | NA | + | NA | -7.50E-03 | NA | 6 | 115.431 | -218.863 | 1.628 | 0.142 |
| 0.04769 | NA | NA | NA | 1.43E-04 | NA | NA | 3 | 112.261 | -218.521 | 1.969 | 0.12 |
| 0.0041 | 1.69E-11 | NA | + | NA | NA | NA | 6 | 115.249 | -218.498 | 1.992 | 0.118 |
| 0.00635 | NA | NA | + | NA | NA | -8.21E-04 | 6 | 115.249 | -218.497 | 1.993 | 0.118 |
| 0.00003 | NA | NA | + | NA | NA | NA | 5 | 124.053 | -238.106 | 0 | 0.338 |
| 0.02716 | NA | NA | + | NA | -1.19E-02 | NA | 6 | 124.657 | -237.314 | 0.792 | 0.227 |
| 0.01014 | NA | NA | + | 5.80E-05 | NA | NA | 6 | 124.371 | -236.741 | 1.365 | 0.171 |
| -0.00214 | 1.38E-10 | NA | + | NA | NA | NA | 6 | 124.142 | -236.284 | 1.822 | 0.136 |
| 0.0058 | NA | NA | + | NA | NA | -2.41E-03 | 6 | 124.081 | -236.163 | 1.944 | 0.128 |
| 0.00143 | NA | NA | + | NA | NA | NA | 5 | 123.875 | -237.751 | 0 | 0.361 |
| 0.01362 | NA | NA | + | 6.52E-05 | NA | NA | 6 | 124.24 | -236.479 | 1.271 | 0.191 |
| 0.02 | NA | NA | + | NA | -8.13E-03 | NA | 6 | 124.134 | -236.267 | 1.484 | 0.172 |
| 0.00816 | NA | NA | + | NA | NA | -2.89E-03 | 6 | 123.918 | -235.837 | 1.914 | 0.138 |
| -0.00034 | 9.97E-11 | NA | + | NA | NA | NA | 6 | 123.918 | -235.836 | 1.915 | 0.138 |
| 0.0042 | NA | NA | + | NA | NA | NA | 5 | 126.555 | -243.11 | 0 | 0.349 |
| 0.02518 | NA | NA | + | NA | -9.34E-03 | NA | 6 | 127.026 | -242.052 | 1.058 | 0.206 |
| 0.01073 | NA | NA | + | 4.41E-05 | NA | NA | 6 | 126.76 | -241.52 | I.59 | 0.158 |
| -0.00807 | NA | NA | + | NA | NA | 5.11E-03 | 6 | 126.708 | -241.417 | 1.693 | 0.15 |
| 0.00241 | 1.09E-10 | NA | + | NA | NA | NA | 6 | 126.63 | -241.26 | I.85 | 0.138 |
| 0.00485 | NA | NA | + | NA | NA | NA | 5 | 125.105 | -240.21 | 0 | 0.352 |
| 0.01637 | NA | NA | + | 6.87E-05 | NA | NA | 6 | 125.536 | -239.072 | 1.138 | 0.199 |
| 0.0205 | NA | NA | + | NA | -6.94E-03 | NA | 6 | 125.308 | -238.616 | 1.594 | 0.159 |
| -0.0078 | NA | NA | + | NA | NA | 5.28E-03 | 6 | 125.278 | -238.556 | 1.654 | 0.154 |
| 0.00316 | 9.57E-11 | NA | + | NA | NA | NA | 6 | 125.156 | -238.312 | 1.898 | 0.136 |
| 0.00222 | NA | NA | + | NA | NA | NA | 5 | 124.192 | -238.385 | 0 | 0.363 |
| 0.02349 | NA | NA | + | NA | -9.47E-03 | NA | 6 | 124.545 | -237.091 | 1.294 | 0.19 |
| 0.01197 | NA | NA | + | 5.37E-05 | NA | NA | 6 | 124.459 | -236.919 | 1.466 | 0.175 |
| 0.00109 | 6.51E-11 | NA | + | NA | NA | NA | 6 | 124.222 | -236.444 | I.94 | 0.138 |
| 0.0016 | NA | NA | + | NA | NA | 2.60E-04 | 6 | 124.193 | -236.385 | 1.999 | 0.134 |
| 0.00492 | NA | NA | + | NA | NA | NA | 5 | 123.53 | -237.059 | 0 | 0.354 |
| 0.01767 | NA | NA | + | 7.39E-05 | NA | NA | 6 | 124.027 | -236.054 | 1.005 | 0.214 |
| -0.00673 | NA | NA | + | NA | NA | 4.86E-03 | 6 | 123.674 | -235.347 | 1.712 | 0.151 |
| 0.01743 | NA | NA | + | NA | -5.54E-03 | NA | 6 | 123.66 | -235.32 | I.74 | 0.148 |
| 0.00417 | 4.20E-11 | NA | + | NA | NA | NA | 6 | 123.544 | -235.088 | 1.971 | 0.132 |
| 0.00238 | NA | NA | + | NA | NA | NA | 5 | 125.167 | -240.334 | 0 | 0.364 |
| 0.02372 | NA | NA | + | NA | -9.32E-03 | NA | 6 | 125.535 | -239.069 | 1.265 | 0.193 |
| 0.01144 | NA | NA | + | 5.09E-05 | NA | NA | 6 | 125.4 | -238.8 | 1.534 | 0.169 |
| 0.0085 | NA | NA | + | NA | NA | -2.57E-03 | 6 | 125.201 | -238.403 | 1.931 | 0.138 |
| 0.00137 | 5.51E-11 | NA | + | NA | NA | NA | 6 | 125.184 | -238.369 | 1.965 | 0.136 |
| -0.0002 | NA | NA | + | NA | NA | NA | 5 | 124.027 | -238.055 | 0 | 0.347 |
| 0.01363 | NA | NA | + | 7.98E-05 | NA | NA | 6 | 124.602 | -237.203 | 0.851 | 0.227 |
| 0.01741 | NA | NA | + | NA | -7.72E-03 | NA | 6 | 124.268 | -236.537 | 1.518 | 0.163 |
| -0.00146 | 7.87E-11 | NA | + | NA | NA | NA | 6 | 124.077 | -236.154 | 1.901 | 0.134 |
| 0.00395 | NA | NA | + | NA | NA | -1.74E-03 | 6 | 124.041 | -236.083 | 1.972 | 0.13 |
| 0.00632 | NA | NA | + | NA | NA | NA | 5 | 123.027 | -236.055 | 0 | 0.288 |
| 0.02146 | NA | NA | + | 8.52E-05 | NA | NA | 6 | 123.591 | -235.182 | 0.873 | 0.186 |
| 0.04658 | NA | NA | NA | 1.49E-04 | NA | NA | 3 | 120.549 | -235.098 | 0.957 | 0.178 |
| 0.02104 | NA | NA | + | NA | -6.51E-03 | NA | 6 | 123.171 | -234.342 | 1.713 | 0.122 |
| 0.01671 | NA | NA | + | NA | NA | -4.33E-03 | 6 | 123.117 | -234.235 | I.82 | 0.116 |
| 0.00509 | 7.53E-11 | NA | + | NA | NA | NA | 6 | 123.062 | -234.124 | 1.931 | 0.11 |
| 0.00412 | NA | NA | + | NA | NA | NA | 5 | 120.135 | -230.271 | 0 | 0.317 |
| 0.01636 | NA | NA | + | 6.41E-05 | NA | NA | 6 | 120.454 | -228.908 | 1.362 | 0.161 |
| 0.02462 | NA | NA | + | NA | -8.98E-03 | NA | 6 | 120.4 | -228.8 | 1.471 | 0.152 |
| 0.01576 | NA | NA | + | NA | NA | -4.87E-03 | 6 | 120.244 | -228.487 | 1.783 | 0.13 |
| 0.00285 | 7.35E-11 | NA | + | NA | NA | NA | 6 | 120.169 | -228.338 | 1.932 | 0.121 |
| 0.04692 | NA | NA | NA | 1.37E-04 | NA | NA | 3 | 117.153 | -228.305 | 1.965 | 0.119 |
| 0.00414 | NA | NA | + | NA | NA | NA | 5 | 122.995 | -235.989 | 0 | 0.358 |
| 0.01709 | NA | NA | + | 7.10E-05 | NA | NA | 6 | 123.411 | -234.821 | 1.168 | 0.2 |
| 0.02196 | NA | NA | + | NA | -7.75E-03 | NA | 6 | 123.213 | -234.425 | 1.564 | 0.164 |
| 0.0135 | NA | NA | + | NA | NA | -3.98E-03 | 6 | 123.072 | -234.143 | 1.846 | 0.142 |
| 0.00292 | 6.63E-11 | NA | + | NA | NA | NA | 6 | 123.02 | -234.04 | 1.949 | 0.135 |
| 0.00394 | NA | NA | + | NA | NA | NA | 5 | 122.495 | -234.989 | 0 | 0.357 |
| 0.01587 | NA | NA | + | 6.57E-05 | NA | NA | 6 | 122.854 | -233.708 | 1.281 | 0.188 |
| 0.0254 | NA | NA | + | NA | -9.53E-03 | NA | 6 | 122.84 | -233.68 | 1.309 | 0.185 |
| 0.00257 | 7.84E-11 | NA | + | NA | NA | NA | 6 | 122.533 | -233.066 | 1.924 | 0.136 |
| 0.00779 | NA | NA | + | NA | NA | -1.61E-03 | 6 | 122.509 | -233.018 | 1.972 | 0.133 |
| 0.00108 | NA | NA | + | NA | NA | NA | 5 | 122.569 | -235.139 | 0 | 0.348 |
| 0.02635 | NA | NA | + | NA | -1.10E-02 | NA | 6 | 123.03 | -234.06 | 1.078 | 0.203 |
| 0.01178 | NA | NA | + | 6.16E-05 | NA | NA | 6 | 122.906 | -233.812 | 1.327 | 0.179 |
| 0.00995 | NA | NA | + | NA | NA | -3.73E-03 | 6 | 122.638 | -233.276 | 1.863 | 0.137 |
| -0.00035 | 8.44E-11 | NA | + | NA | NA | NA | 6 | 122.613 | -233.227 | 1.912 | 0.134 |
| 0.00432 | NA | NA | + | NA | NA | NA | 5 | 121.005 | -232.01 | 0 | 0.269 |
| 0.02244 | NA | NA | + | 1.01E-04 | NA | NA | 6 | 121.869 | -231.739 | 0.271 | 0.235 |
| 0.04824 | NA | NA | NA | 1.57E-04 | NA | NA | 3 | 118.535 | -231.069 | 0.941 | 0.168 |
| 0.02106 | NA | NA | + | NA | -7.36E-03 | NA | 6 | 121.196 | -230.391 | 1.618 | 0.12 |
| 0.0026 | 1.00E-10 | NA | + | NA | NA | NA | 6 | 121.065 | -230.13 | I.88 | 0.105 |
| 0.01069 | NA | NA | + | NA | NA | -2.66E-03 | 6 | 121.041 | -230.082 | 1.928 | 0.103 |
| 0.00382 | NA | NA | + | NA | NA | NA | 5 | 118.614 | -227.228 | 0 | 0.288 |
| 0.02074 | NA | NA | + | 8.83E-05 | NA | NA | 6 | 119.183 | -226.366 | 0.862 | 0.187 |
| 0.04806 | NA | NA | NA | 1.52E-04 | NA | NA | 3 | 116.156 | -226.313 | 0.916 | 0.182 |
| 0.01771 | NA | NA | + | NA | -6.04E-03 | NA | 6 | 118.733 | -225.467 | 1.762 | 0.119 |
| 0.00212 | 1.00E-10 | NA | + | NA | NA | NA | 6 | 118.671 | -225.343 | 1.885 | 0.112 |
| 0.0107 | NA | NA | + | NA | NA | -2.90E-03 | 6 | 118.652 | -225.304 | 1.924 | 0.11 |
| 0.00275 | NA | NA | + | NA | NA | NA | 5 | 123.829 | -237.658 | 0 | 0.296 |
| 0.01867 | NA | NA | + | 8.06E-05 | NA | NA | 6 | 124.338 | -236.675 | 0.982 | 0.181 |
| 0.04608 | NA | NA | NA | 1.40E-04 | NA | NA | 3 | 121.296 | -236.592 | 1.066 | 0.174 |
| 0.01489 | NA | NA | + | NA | -5.31E-03 | NA | 6 | 123.923 | -235.846 | 1.812 | 0.12 |
| 0.01293 | NA | NA | + | NA | NA | -4.27E-03 | 6 | 123.919 | -235.838 | I.82 | 0.119 |
| 0.00202 | 4.15E-11 | NA | + | NA | NA | NA | 6 | 123.845 | -235.69 | 1.968 | 0.111 |
| 0.00351 | NA | NA | + | NA | NA | NA | 5 | 124.563 | -239.126 | 0 | 0.364 |
| 0.01331 | NA | NA | + | 5.65E-05 | NA | NA | 6 | 124.854 | -237.709 | 1.417 | 0.179 |
| 0.0222 | NA | NA | + | NA | -8.32E-03 | NA | 6 | 124.844 | -237.689 | 1.437 | 0.178 |
| 0.01134 | NA | NA | + | NA | NA | -3.27E-03 | 6 | 124.615 | -237.229 | 1.896 | 0.141 |
| 0.00249 | 5.95E-11 | NA | + | NA | NA | NA | 6 | 124.587 | -237.175 | 1.951 | 0.137 |
| 0.00503 | NA | NA | + | NA | NA | NA | 5 | 129.032 | -248.064 | 0 | 0.363 |
| 0.01523 | NA | NA | + | 6.18E-05 | NA | NA | 6 | 129.404 | -246.808 | 1.256 | 0.194 |
| 0.02271 | NA | NA | + | NA | -7.87E-03 | NA | 6 | 129.287 | -246.575 | 1.489 | 0.172 |
| 0.00424 | 4.60E-11 | NA | + | NA | NA | NA | 6 | 129.053 | -246.107 | 1.957 | 0.136 |
| 0.00321 | NA | NA | + | NA | NA | 7.54E-04 | 6 | 129.035 | -246.07 | 1.994 | 0.134 |
| -0.00056 | NA | NA | + | NA | NA | NA | 5 | 123.808 | -237.616 | 0 | 0.356 |
| 0.02165 | NA | NA | + | NA | -9.92E-03 | NA | 6 | 124.237 | -236.474 | 1.142 | 0.201 |
| 0.00794 | NA | NA | + | 5.22E-05 | NA | NA | 6 | 124.064 | -236.127 | 1.489 | 0.169 |
| -0.00223 | 9.08E-11 | NA | + | NA | NA | NA | 6 | 123.863 | -235.726 | I.89 | 0.139 |
| -0.00574 | NA | NA | + | NA | NA | 2.19E-03 | 6 | 123.833 | -235.667 | 1.949 | 0.134 |
| 0.00343 | NA | NA | + | NA | NA | NA | 5 | 123.487 | -236.973 | 0 | 0.344 |
| 0.01538 | NA | NA | + | 7.40E-05 | NA | NA | 6 | 123.992 | -235.985 | 0.989 | 0.21 |
| 0.02378 | NA | NA | + | NA | -9.00E-03 | NA | 6 | 123.845 | -235.69 | 1.283 | 0.181 |
| 0.00177 | 9.81E-11 | NA | + | NA | NA | NA | 6 | 123.541 | -235.081 | 1.892 | 0.134 |
| -0.00318 | NA | NA | + | NA | NA | 2.69E-03 | 6 | 123.528 | -235.056 | 1.917 | 0.132 |
| 0.00335 | NA | NA | + | NA | NA | NA | 5 | 117.045 | -224.09 | 0 | 0.295 |
| 0.01951 | NA | NA | + | 8.82E-05 | NA | NA | 6 | 117.633 | -223.266 | 0.824 | 0.195 |
| 0.04748 | NA | NA | NA | 1.45E-04 | NA | NA | 3 | 114.453 | -222.906 | 1.184 | 0.163 |
| 0.01699 | NA | NA | + | NA | -5.90E-03 | NA | 6 | 117.158 | -222.316 | 1.774 | 0.122 |
| 0.01055 | NA | NA | + | NA | NA | -3.03E-03 | 6 | 117.087 | -222.173 | 1.916 | 0.113 |
| 0.00208 | 7.13E-11 | NA | + | NA | NA | NA | 6 | 117.075 | -222.15 | I.94 | 0.112 |
| 0.00261 | NA | NA | + | NA | NA | NA | 5 | 121.346 | -232.692 | 0 | 0.352 |
| 0.01498 | NA | NA | + | 6.51E-05 | NA | NA | 6 | 121.691 | -231.383 | 1.309 | 0.183 |
| 0.02434 | NA | NA | + | NA | -9.72E-03 | NA | 6 | 121.677 | -231.354 | 1.338 | 0.18 |
| 0.01534 | NA | NA | + | NA | NA | -5.33E-03 | 6 | 121.477 | -230.953 | 1.738 | 0.148 |
| 0.00091 | 9.71E-11 | NA | + | NA | NA | NA | 6 | 121.398 | -230.797 | 1.895 | 0.137 |
| 0.00753 | NA | NA | + | NA | NA | NA | 5 | 127.113 | -244.226 | 0 | 0.356 |
| 0.02526 | NA | NA | + | NA | -7.84E-03 | NA | 6 | 127.432 | -242.863 | 1.363 | 0.18 |
| 0.01484 | NA | NA | + | 4.67E-05 | NA | NA | 6 | 127.344 | -242.688 | 1.538 | 0.165 |
| -0.00641 | NA | NA | + | NA | NA | 5.83E-03 | 6 | 127.312 | -242.624 | 1.602 | 0.16 |
| 0.00584 | 1.06E-10 | NA | + | NA | NA | NA | 6 | 127.173 | -242.346 | I.88 | 0.139 |
| -0.00097 | NA | NA | + | NA | NA | NA | 5 | 127.08 | -244.161 | 0 | 0.351 |
| 0.01672 | NA | NA | + | NA | -7.84E-03 | NA | 6 | 127.408 | -242.816 | 1.345 | 0.179 |
| 0.00688 | NA | NA | + | 4.84E-05 | NA | NA | 6 | 127.326 | -242.652 | 1.509 | 0.165 |
| -0.01613 | NA | NA | + | NA | NA | 6.45E-03 | 6 | 127.324 | -242.647 | 1.514 | 0.165 |
| -0.00285 | 1.09E-10 | NA | + | NA | NA | NA | 6 | 127.157 | -242.314 | 1.847 | 0.14 |
| -0.00062 | NA | NA | + | NA | NA | NA | 5 | 126.165 | -242.33 | 0 | 0.353 |
| 0.01197 | NA | NA | + | 7.32E-05 | NA | NA | 6 | 126.663 | -241.327 | 1.003 | 0.214 |
| 0.01805 | NA | NA | + | NA | -8.09E-03 | NA | 6 | 126.432 | -240.864 | 1.466 | 0.17 |
| -0.00154 | 5.26E-11 | NA | + | NA | NA | NA | 6 | 126.189 | -240.378 | 1.952 | 0.133 |
| -0.00071 | NA | NA | + | NA | NA | 3.98E-05 | 6 | 126.165 | -240.33 | 2 | 0.13 |
| 0.00637 | NA | NA | + | NA | NA | NA | 5 | 124.438 | -238.876 | 0 | 0.358 |
| 0.02013 | NA | NA | + | 7.96E-05 | NA | NA | 6 | 124.96 | -237.921 | 0.955 | 0.222 |
| 0.01824 | NA | NA | + | NA | -5.35E-03 | NA | 6 | 124.548 | -237.096 | I.78 | 0.147 |
| 0.00478 | 9.65E-11 | NA | + | NA | NA | NA | 6 | 124.49 | -236.98 | 1.896 | 0.139 |
| 0.00257 | NA | NA | + | NA | NA | 1.54E-03 | 6 | 124.451 | -236.902 | 1.973 | 0.134 |
| 0.00154 | NA | NA | + | NA | NA | NA | 5 | 127.117 | -244.234 | 0 | 0.34 |
| 0.02646 | NA | NA | + | NA | -1.11E-02 | NA | 6 | 127.791 | -243.582 | 0.652 | 0.245 |
| -0.00989 | NA | NA | + | NA | NA | 4.82E-03 | 6 | 127.264 | -242.527 | 1.707 | 0.145 |
| 0.00646 | NA | NA | + | 3.12E-05 | NA | NA | 6 | 127.23 | -242.46 | 1.775 | 0.14 |
| 0.00045 | 6.83E-11 | NA | + | NA | NA | NA | 6 | 127.158 | -242.317 | 1.918 | 0.13 |
| 0.00555 | NA | NA | + | NA | NA | NA | 5 | 121.964 | -233.928 | 0 | 0.362 |
| 0.01888 | NA | NA | + | 7.02E-05 | NA | NA | 6 | 122.338 | -232.677 | 1.251 | 0.193 |
| 0.02506 | NA | NA | + | NA | -8.65E-03 | NA | 6 | 122.215 | -232.429 | 1.499 | 0.171 |
| 0.01284 | NA | NA | + | NA | NA | -3.02E-03 | 6 | 122.011 | -232.023 | 1.905 | 0.139 |
| 0.00481 | 4.49E-11 | NA | + | NA | NA | NA | 6 | 121.977 | -231.954 | 1.974 | 0.135 |
| -0.00043 | NA | NA | + | NA | NA | NA | 5 | 126.079 | -242.159 | 0 | 0.368 |
| 0.01805 | NA | NA | + | NA | -8.14E-03 | NA | 6 | 126.375 | -240.75 | 1.409 | 0.182 |
| 0.00773 | NA | NA | + | 4.97E-05 | NA | NA | 6 | 126.331 | -240.662 | 1.496 | 0.174 |
| -0.00168 | 7.09E-11 | NA | + | NA | NA | NA | 6 | 126.114 | -240.228 | 1.931 | 0.14 |
| -0.00143 | NA | NA | + | NA | NA | 4.28E-04 | 6 | 126.08 | -240.161 | 1.998 | 0.136 |
| 0.00375 | NA | NA | + | NA | NA | NA | 5 | 128.346 | -246.691 | 0 | 0.352 |
| 0.0259 | NA | NA | + | NA | -9.93E-03 | NA | 6 | 128.829 | -245.659 | 1.032 | 0.21 |
| 0.01177 | NA | NA | + | 5.22E-05 | NA | NA | 6 | 128.634 | -245.268 | 1.423 | 0.173 |
| 0.00268 | 6.48E-11 | NA | + | NA | NA | NA | 6 | 128.378 | -244.756 | 1.935 | 0.134 |
| -0.00084 | NA | NA | + | NA | NA | 1.91E-03 | 6 | 128.365 | -244.73 | 1.961 | 0.132 |
| 0.00656 | NA | NA | + | NA | NA | NA | 5 | 124.482 | -238.963 | 0 | 0.358 |
| 0.01694 | NA | NA | + | 6.42E-05 | NA | NA | 6 | 124.869 | -237.738 | 1.225 | 0.194 |
| 0.02535 | NA | NA | + | NA | -8.43E-03 | NA | 6 | 124.789 | -237.578 | 1.385 | 0.179 |
| 0.00533 | 7.37E-11 | NA | + | NA | NA | NA | 6 | 124.524 | -237.047 | 1.916 | 0.137 |
| 0.00422 | NA | NA | + | NA | NA | 9.69E-04 | 6 | 124.486 | -236.972 | 1.991 | 0.132 |
| -0.00058 | NA | NA | + | NA | NA | NA | 5 | 126.365 | -242.73 | 0 | 0.34 |
| 0.02342 | NA | NA | + | NA | -1.04E-02 | NA | 6 | 126.924 | -241.849 | 0.881 | 0.219 |
| 0.00829 | NA | NA | + | 5.57E-05 | NA | NA | 6 | 126.705 | -241.411 | 1.319 | 0.176 |
| -0.00247 | 1.10E-10 | NA | + | NA | NA | NA | 6 | 126.435 | -240.87 | I.86 | 0.134 |
| -0.00728 | NA | NA | + | NA | NA | 2.87E-03 | 6 | 126.41 | -240.821 | 1.909 | 0.131 |
| 0.00252 | NA | NA | + | NA | NA | NA | 5 | 122.07 | -234.141 | 0 | 0.328 |
| 0.01776 | NA | NA | + | 8.41E-05 | NA | NA | 6 | 122.686 | -233.373 | 0.768 | 0.224 |
| 0.02644 | NA | NA | + | NA | -1.05E-02 | NA | 6 | 122.492 | -232.984 | 1.157 | 0.184 |
| 0.01464 | NA | NA | + | NA | NA | -5.06E-03 | 6 | 122.19 | -232.38 | 1.761 | 0.136 |
| 0.00082 | 9.66E-11 | NA | + | NA | NA | NA | 6 | 122.123 | -232.246 | 1.894 | 0.127 |
| 0.00237 | NA | NA | + | NA | NA | NA | 5 | 121.615 | -233.231 | 0 | 0.371 |
| 0.0125 | NA | NA | + | 6.09E-05 | NA | NA | 6 | 121.943 | -231.887 | 1.344 | 0.19 |
| 0.01734 | NA | NA | + | NA | -6.61E-03 | NA | 6 | 121.786 | -231.573 | 1.658 | 0.162 |
| 0.00117 | 6.97E-11 | NA | + | NA | NA | NA | 6 | 121.643 | -231.287 | 1.944 | 0.14 |
| 0.00419 | NA | NA | + | NA | NA | -7.71E-04 | 6 | 121.618 | -231.237 | 1.994 | 0.137 |
| 0.00574 | NA | NA | + | NA | NA | NA | 5 | 120.155 | -230.31 | 0 | 0.376 |
| 0.0164 | NA | NA | + | 5.71E-05 | NA | NA | 6 | 120.407 | -228.814 | 1.496 | 0.178 |
| 0.02154 | NA | NA | + | NA | -6.99E-03 | NA | 6 | 120.315 | -228.631 | 1.679 | 0.162 |
| 0.01264 | NA | NA | + | NA | NA | -2.90E-03 | 6 | 120.199 | -228.398 | 1.912 | 0.145 |
| 0.00525 | 2.76E-11 | NA | + | NA | NA | NA | 6 | 120.161 | -228.321 | 1.989 | 0.139 |
| 0.00143 | NA | NA | + | NA | NA | NA | 5 | 119.438 | -228.876 | 0 | 0.227 |
| 0.02082 | NA | NA | + | 1.02E-04 | NA | NA | 6 | 120.261 | -228.522 | 0.354 | 0.19 |
| 0.02457 | NA | NA | + | NA | -9.97E-03 | NA | 6 | 119.763 | -227.527 | I.35 | 0.115 |
| 0.04862 | NA | NA | NA | 1.61E-04 | NA | NA | 3 | 116.747 | -227.495 | 1.382 | 0.114 |
| 0.01094 | NA | NA | + | NA | NA | -4.01E-03 | 6 | 119.516 | -227.032 | 1.844 | 0.09 |
| 0.04009 | NA | NA | + | 9.74E-05 | -8.69E-03 | NA | 7 | 120.509 | -227.018 | 1.858 | 0.089 |
| -0.00043 | 1.06E-10 | NA | + | NA | NA | NA | 6 | 119.502 | -227.004 | 1.872 | 0.089 |
| 0.0386 | NA | NA | + | 1.12E-04 | NA | -6.71E-03 | 7 | 120.474 | -226.947 | 1.929 | 0.086 |
| 0.00159 | NA | NA | + | NA | NA | NA | 5 | 127.303 | -244.606 | 0 | 0.348 |
| 0.0152 | NA | NA | + | 7.80E-05 | NA | NA | 6 | 127.868 | -243.735 | 0.87 | 0.225 |
| 0.01964 | NA | NA | + | NA | -7.84E-03 | NA | 6 | 127.553 | -243.107 | 1.499 | 0.164 |
| 0.00024 | 7.99E-11 | NA | + | NA | NA | NA | 6 | 127.356 | -242.712 | 1.893 | 0.135 |
| -0.00071 | NA | NA | + | NA | NA | 9.76E-04 | 6 | 127.308 | -242.616 | I.99 | 0.128 |
| 0.00281 | NA | NA | + | NA | NA | NA | 5 | 127.575 | -245.15 | 0 | 0.362 |
| 0.02268 | NA | NA | + | NA | -8.83E-03 | NA | 6 | 127.896 | -243.792 | 1.358 | 0.184 |
| 0.01257 | NA | NA | + | 5.59E-05 | NA | NA | 6 | 127.861 | -243.722 | 1.428 | 0.177 |
| 0.001 | 1.07E-10 | NA | + | NA | NA | NA | 6 | 127.632 | -243.263 | 1.887 | 0.141 |
| -0.00221 | NA | NA | + | NA | NA | 2.09E-03 | 6 | 127.601 | -243.202 | 1.949 | 0.137 |
| 0.00473 | NA | NA | + | NA | NA | NA | 5 | 125.672 | -241.344 | 0 | 0.347 |
| 0.01462 | NA | NA | + | 6.06E-05 | NA | NA | 6 | 126.047 | -240.094 | I.25 | 0.186 |
| 0.02151 | NA | NA | + | NA | -7.65E-03 | NA | 6 | 125.973 | -239.946 | 1.398 | 0.173 |
| -0.00982 | NA | NA | + | NA | NA | 5.98E-03 | 6 | 125.888 | -239.776 | 1.568 | 0.159 |
| 0.00312 | 9.67E-11 | NA | + | NA | NA | NA | 6 | 125.73 | -239.46 | 1.885 | 0.135 |
| 0.00311 | NA | NA | + | NA | NA | NA | 5 | 121.74 | -233.48 | 0 | 0.305 |
| 0.01683 | NA | NA | + | 7.45E-05 | NA | NA | 6 | 122.187 | -232.374 | 1.106 | 0.175 |
| 0.02491 | NA | NA | + | NA | -9.69E-03 | NA | 6 | 122.085 | -232.169 | 1.311 | 0.158 |
| 0.04577 | NA | NA | NA | 1.48E-04 | NA | NA | 3 | 118.867 | -231.734 | 1.747 | 0.127 |
| 0.00123 | 1.13E-10 | NA | + | NA | NA | NA | 6 | 121.806 | -231.613 | 1.868 | 0.12 |
| 0.00848 | NA | NA | + | NA | NA | -2.24E-03 | 6 | 121.766 | -231.532 | 1.949 | 0.115 |
| 0.00144 | NA | NA | + | NA | NA | NA | 5 | 125.193 | -240.386 | 0 | 0.35 |
| 0.02641 | NA | NA | + | NA | -1.10E-02 | NA | 6 | 125.759 | -239.518 | 0.868 | 0.227 |
| 0.009 | NA | NA | + | 4.54E-05 | NA | NA | 6 | 125.397 | -238.794 | 1.592 | 0.158 |
| -0.00022 | 9.55E-11 | NA | + | NA | NA | NA | 6 | 125.245 | -238.49 | 1.896 | 0.136 |
| 0.00259 | NA | NA | + | NA | NA | -4.91E-04 | 6 | 125.194 | -238.389 | 1.997 | 0.129 |
| 0.00522 | NA | NA | + | NA | NA | NA | 5 | 125.544 | -241.088 | 0 | 0.313 |
| 0.01918 | NA | NA | + | 7.78E-05 | NA | NA | 6 | 126.062 | -240.124 | 0.965 | 0.193 |
| 0.04611 | NA | NA | NA | 1.40E-04 | NA | NA | 3 | 122.669 | -239.338 | I.75 | 0.13 |
| 0.0181 | NA | NA | + | NA | -5.69E-03 | NA | 6 | 125.657 | -239.315 | 1.773 | 0.129 |
| 0.00395 | 7.14E-11 | NA | + | NA | NA | NA | 6 | 125.568 | -239.137 | 1.952 | 0.118 |
| 0.00858 | NA | NA | + | NA | NA | -1.42E-03 | 6 | 125.555 | -239.11 | 1.979 | 0.116 |
| 0.0083 | NA | NA | + | NA | NA | NA | 5 | 122.694 | -235.389 | 0 | 0.305 |
| 0.02415 | NA | NA | + | 8.45E-05 | NA | NA | 6 | 123.259 | -234.518 | 0.871 | 0.197 |
| 0.04833 | NA | NA | NA | 1.36E-04 | NA | NA | 3 | 119.953 | -233.906 | 1.482 | 0.145 |
| 0.01864 | NA | NA | + | NA | -4.58E-03 | NA | 6 | 122.768 | -233.536 | 1.853 | 0.121 |
| 0.00646 | 1.02E-10 | NA | + | NA | NA | NA | 6 | 122.739 | -233.478 | I.91 | 0.117 |
| 0.01223 | NA | NA | + | NA | NA | -1.66E-03 | 6 | 122.707 | -233.415 | 1.974 | 0.114 |
| 0.00223 | NA | NA | + | NA | NA | NA | 5 | 120.016 | -230.033 | 0 | 0.362 |
| 0.01586 | NA | NA | + | 7.47E-05 | NA | NA | 6 | 120.463 | -228.926 | 1.106 | 0.208 |
| 0.01754 | NA | NA | + | NA | -6.75E-03 | NA | 6 | 120.175 | -228.35 | 1.683 | 0.156 |
| 0.00097 | 7.37E-11 | NA | + | NA | NA | NA | 6 | 120.059 | -228.117 | 1.915 | 0.139 |
| 0.00603 | NA | NA | + | NA | NA | -1.57E-03 | 6 | 120.028 | -228.057 | 1.976 | 0.135 |
| 0.0007 | NA | NA | + | NA | NA | NA | 5 | 121.482 | -232.965 | 0 | 0.353 |
| 0.0132 | NA | NA | + | 6.81E-05 | NA | NA | 6 | 121.88 | -231.759 | 1.205 | 0.193 |
| 0.0239 | NA | NA | + | NA | -1.01E-02 | NA | 6 | 121.844 | -231.688 | 1.277 | 0.186 |
| -0.00103 | 9.75E-11 | NA | + | NA | NA | NA | 6 | 121.536 | -231.072 | 1.892 | 0.137 |
| 0.00299 | NA | NA | + | NA | NA | -9.92E-04 | 6 | 121.487 | -230.975 | I.99 | 0.13 |
| -0.00047 | NA | NA | + | NA | NA | NA | 5 | 121.533 | -233.065 | 0 | 0.27 |
| 0.01757 | NA | NA | + | 9.63E-05 | NA | NA | 6 | 122.316 | -232.632 | 0.433 | 0.218 |
| 0.04742 | NA | NA | NA | 1.64E-04 | NA | NA | 3 | 119.041 | -232.082 | 0.983 | 0.165 |
| 0.02047 | NA | NA | + | NA | -9.12E-03 | NA | 6 | 121.826 | -231.653 | 1.412 | 0.133 |
| 0.00922 | NA | NA | + | NA | NA | -4.05E-03 | 6 | 121.611 | -231.223 | 1.842 | 0.108 |
| -0.00221 | 1.03E-10 | NA | + | NA | NA | NA | 6 | 121.588 | -231.176 | 1.889 | 0.105 |
| 0.00242 | NA | NA | + | NA | NA | NA | 5 | 122.927 | -235.854 | 0 | 0.347 |
| 0.0164 | NA | NA | + | 7.93E-05 | NA | NA | 6 | 123.473 | -234.947 | 0.908 | 0.221 |
| 0.02043 | NA | NA | + | NA | -7.80E-03 | NA | 6 | 123.161 | -234.322 | 1.533 | 0.161 |
| 0.0126 | NA | NA | + | NA | NA | -4.26E-03 | 6 | 123.014 | -234.027 | 1.827 | 0.139 |
| 0.001 | 8.19E-11 | NA | + | NA | NA | NA | 6 | 122.958 | -233.916 | 1.939 | 0.132 |
| 0.00583 | NA | NA | + | NA | NA | NA | 5 | 126.137 | -242.274 | 0 | 0.29 |
| 0.02119 | NA | NA | + | 8.57E-05 | NA | NA | 6 | 126.763 | -241.526 | 0.748 | 0.199 |
| 0.04606 | NA | NA | NA | 1.41E-04 | NA | NA | 3 | 123.485 | -240.97 | 1.304 | 0.151 |
| 0.02394 | NA | NA | + | NA | -8.10E-03 | NA | 6 | 126.378 | -240.756 | 1.518 | 0.136 |
| 0.01365 | NA | NA | + | NA | NA | -3.23E-03 | 6 | 126.188 | -240.376 | 1.898 | 0.112 |
| 0.00443 | 7.96E-11 | NA | + | NA | NA | NA | 6 | 126.183 | -240.365 | 1.909 | 0.112 |
| 0.00378 | NA | NA | + | NA | NA | NA | 5 | 122.559 | -235.118 | 0 | 0.355 |
| 0.01563 | NA | NA | + | 6.88E-05 | NA | NA | 6 | 122.977 | -233.955 | 1.163 | 0.198 |
| 0.02348 | NA | NA | + | NA | -8.80E-03 | NA | 6 | 122.875 | -233.75 | 1.368 | 0.179 |
| 0.00238 | 8.34E-11 | NA | + | NA | NA | NA | 6 | 122.595 | -233.19 | 1.928 | 0.135 |
| 0.00841 | NA | NA | + | NA | NA | -1.95E-03 | 6 | 122.577 | -233.155 | 1.963 | 0.133 |
| 0.00215 | NA | NA | + | NA | NA | NA | 5 | 121.353 | -232.707 | 0 | 0.339 |
| 0.0174 | NA | NA | + | 8.29E-05 | NA | NA | 6 | 121.924 | -231.848 | 0.859 | 0.221 |
| 0.02483 | NA | NA | + | NA | -9.86E-03 | NA | 6 | 121.714 | -231.428 | 1.279 | 0.179 |
| 0.00055 | 9.19E-11 | NA | + | NA | NA | NA | 6 | 121.403 | -230.805 | 1.901 | 0.131 |
| 0.00932 | NA | NA | + | NA | NA | -3.01E-03 | 6 | 121.399 | -230.798 | 1.908 | 0.131 |
| 0.00357 | NA | NA | + | NA | NA | NA | 5 | 127.16 | -244.321 | 0 | 0.348 |
| 0.02512 | NA | NA | + | NA | -9.75E-03 | NA | 6 | 127.667 | -243.334 | 0.987 | 0.213 |
| -0.01102 | NA | NA | + | NA | NA | 6.03E-03 | 6 | 127.383 | -242.765 | 1.555 | 0.16 |
| 0.00863 | NA | NA | + | 3.23E-05 | NA | NA | 6 | 127.272 | -242.544 | 1.777 | 0.143 |
| 0.00207 | 9.08E-11 | NA | + | NA | NA | NA | 6 | 127.216 | -242.432 | 1.889 | 0.136 |
| 0.00241 | NA | NA | + | NA | NA | NA | 5 | 121.051 | -232.102 | 0 | 0.3 |
| 0.019 | NA | NA | + | 8.98E-05 | NA | NA | 6 | 121.685 | -231.37 | 0.732 | 0.208 |
| 0.04832 | NA | NA | NA | 1.56E-04 | NA | NA | 3 | 118.232 | -230.463 | 1.639 | 0.132 |
| 0.01744 | NA | NA | + | NA | -6.59E-03 | NA | 6 | 121.194 | -230.389 | 1.713 | 0.127 |
| 0.01145 | NA | NA | + | NA | NA | -3.83E-03 | 6 | 121.118 | -230.235 | 1.867 | 0.118 |
| 0.00114 | 7.61E-11 | NA | + | NA | NA | NA | 6 | 121.097 | -230.194 | 1.908 | 0.115 |
| 0.0003 | NA | NA | + | NA | NA | NA | 5 | 119.428 | -228.857 | 0 | 0.373 |
| 0.01016 | NA | NA | + | 5.47E-05 | NA | NA | 6 | 119.68 | -227.36 | 1.497 | 0.176 |
| 0.01827 | NA | NA | + | NA | -8.00E-03 | NA | 6 | 119.665 | -227.33 | 1.527 | 0.174 |
| -0.00082 | 6.06E-11 | NA | + | NA | NA | NA | 6 | 119.453 | -226.905 | 1.952 | 0.14 |
| 0.00045 | NA | NA | + | NA | NA | -6.73E-05 | 6 | 119.428 | -226.857 | 2 | 0.137 |
| -0.00154 | NA | NA | + | NA | NA | NA | 5 | 125.528 | -241.056 | 0 | 0.326 |
| 0.01241 | NA | NA | + | 8.19E-05 | NA | NA | 6 | 126.204 | -240.408 | 0.647 | 0.236 |
| 0.02094 | NA | NA | + | NA | -9.77E-03 | NA | 6 | 125.977 | -239.955 | 1.101 | 0.188 |
| -0.0036 | 1.16E-10 | NA | + | NA | NA | NA | 6 | 125.616 | -239.231 | 1.825 | 0.131 |
| -0.00342 | NA | NA | + | NA | NA | 7.95E-04 | 6 | 125.531 | -239.062 | 1.994 | 0.12 |
| 0.00266 | NA | NA | + | NA | NA | NA | 5 | 124.193 | -238.385 | 0 | 0.348 |
| 0.0159 | NA | NA | + | 7.44E-05 | NA | NA | 6 | 124.687 | -237.375 | 01.I | 0.21 |
| 0.02338 | NA | NA | + | NA | -9.17E-03 | NA | 6 | 124.53 | -237.059 | 1.326 | 0.179 |
| 0.00141 | 7.34E-11 | NA | + | NA | NA | NA | 6 | 124.232 | -236.465 | 1.921 | 0.133 |
| 0.00628 | NA | NA | + | NA | NA | -1.52E-03 | 6 | 124.205 | -236.409 | 1.976 | 0.13 |
| 0.00459 | NA | NA | + | NA | NA | NA | 5 | 128.857 | -247.714 | 0 | 0.347 |
| 0.01757 | NA | NA | + | 7.12E-05 | NA | NA | 6 | 129.314 | -246.629 | 1.085 | 0.202 |
| 0.02382 | NA | NA | + | NA | -8.56E-03 | NA | 6 | 129.148 | -246.296 | 1.417 | 0.171 |
| 0.01676 | NA | NA | + | NA | NA | -5.09E-03 | 6 | 128.98 | -245.96 | 1.753 | 0.145 |
| 0.00294 | 9.84E-11 | NA | + | NA | NA | NA | 6 | 128.913 | -245.826 | 1.888 | 0.135 |
| 0.00391 | NA | NA | + | NA | NA | NA | 5 | 123.169 | -236.338 | 0 | 0.291 |
| 0.01862 | NA | NA | + | 8.22E-05 | NA | NA | 6 | 123.729 | -235.458 | 0.879 | 0.188 |
| 0.02533 | NA | NA | + | NA | -9.48E-03 | NA | 6 | 123.501 | -235.002 | 1.336 | 0.149 |
| 0.04682 | NA | NA | NA | 1.50E-04 | NA | NA | 3 | 120.45 | -234.9 | 1.438 | 0.142 |
| 0.01512 | NA | NA | + | NA | NA | -4.67E-03 | 6 | 123.273 | -234.546 | 1.792 | 0.119 |
| 0.00269 | 7.41E-11 | NA | + | NA | NA | NA | 6 | 123.207 | -234.413 | 1.924 | 0.111 |
| 0.00177 | NA | NA | + | NA | NA | NA | 5 | 121.893 | -233.787 | 0 | 0.362 |
| 0.02292 | NA | NA | + | NA | -9.23E-03 | NA | 6 | 122.236 | -232.473 | 1.314 | 0.187 |
| 0.01217 | NA | NA | + | 5.87E-05 | NA | NA | 6 | 122.188 | -232.377 | I.41 | 0.179 |
| 0.00027 | 8.40E-11 | NA | + | NA | NA | NA | 6 | 121.933 | -231.865 | 1.922 | 0.138 |
| -0.00062 | NA | NA | + | NA | NA | 1.01E-03 | 6 | 121.9 | -231.799 | 1.988 | 0.134 |
| -0.00097 | NA | NA | + | NA | NA | NA | 5 | 123.489 | -236.978 | 0 | 0.333 |
| 0.02479 | NA | NA | + | NA | -1.15E-02 | NA | 6 | 124.022 | -236.044 | 0.934 | 0.209 |
| 0.01154 | NA | NA | + | 7.39E-05 | NA | NA | 6 | 124.002 | -236.003 | 0.975 | 0.204 |
| -0.0024 | 8.65E-11 | NA | + | NA | NA | NA | 6 | 123.559 | -235.118 | I.86 | 0.131 |
| 0.00177 | NA | NA | + | NA | NA | -1.13E-03 | 6 | 123.496 | -234.991 | 1.987 | 0.123 |
| 0.00014 | NA | NA | + | NA | NA | NA | 5 | 125.778 | -241.557 | 0 | 0.333 |
| 0.02526 | NA | NA | + | NA | -1.11E-02 | NA | 6 | 126.405 | -240.81 | 0.747 | 0.229 |
| 0.00906 | NA | NA | + | 5.68E-05 | NA | NA | 6 | 126.121 | -240.241 | 1.316 | 0.172 |
| -0.00877 | NA | NA | + | NA | NA | 3.80E-03 | 6 | 125.861 | -239.722 | 1.835 | 0.133 |
| -0.00179 | 1.17E-10 | NA | + | NA | NA | NA | 6 | 125.86 | -239.721 | 1.836 | 0.133 |
| 0.00146 | NA | NA | + | NA | NA | NA | 5 | 128.94 | -247.88 | 0 | 0.361 |
| 0.01246 | NA | NA | + | 6.78E-05 | NA | NA | 6 | 129.39 | -246.78 | 01.I | 0.208 |
| 0.01599 | NA | NA | + | NA | -6.49E-03 | NA | 6 | 129.134 | -246.269 | 1.612 | 0.161 |
| 0.00044 | 5.91E-11 | NA | + | NA | NA | NA | 6 | 128.973 | -245.946 | 1.934 | 0.137 |
| 0.00055 | NA | NA | + | NA | NA | 3.82E-04 | 6 | 128.941 | -245.882 | 1.998 | 0.133 |
| 0.00037 | NA | NA | + | NA | NA | NA | 5 | 117.247 | -224.495 | 0 | 0.318 |
| 0.01458 | NA | NA | + | 8.68E-05 | NA | NA | 6 | 117.973 | -223.946 | 0.548 | 0.242 |
| 0.0214 | NA | NA | + | NA | -9.44E-03 | NA | 6 | 117.69 | -223.379 | 1.115 | 0.182 |
| -0.01116 | NA | NA | + | NA | NA | 4.72E-03 | 6 | 117.373 | -222.746 | 1.749 | 0.133 |
| -0.00145 | 1.12E-10 | NA | + | NA | NA | NA | 6 | 117.325 | -222.65 | 1.845 | 0.126 |
| 0.00697 | NA | NA | + | NA | NA | NA | 5 | 127.78 | -245.56 | 0 | 0.356 |
| 0.0186 | NA | NA | + | 7.13E-05 | NA | NA | 6 | 128.277 | -244.555 | 1.005 | 0.215 |
| 0.02195 | NA | NA | + | NA | -6.62E-03 | NA | 6 | 127.971 | -243.942 | 1.618 | 0.159 |
| 0.00558 | 8.29E-11 | NA | + | NA | NA | NA | 6 | 127.823 | -243.647 | 1.913 | 0.137 |
| 0.00305 | NA | NA | + | NA | NA | 1.65E-03 | 6 | 127.795 | -243.589 | I.97 | 0.133 |
| 0.00406 | NA | NA | + | NA | NA | NA | 5 | 120.657 | -231.314 | 0 | 0.35 |
| 0.0158 | NA | NA | + | 7.08E-05 | NA | NA | 6 | 121.115 | -230.23 | 1.083 | 0.203 |
| 0.02213 | NA | NA | + | NA | -8.02E-03 | NA | 6 | 120.936 | -229.873 | 1.441 | 0.17 |
| 0.00179 | 1.31E-10 | NA | + | NA | NA | NA | 6 | 120.753 | -229.506 | 1.808 | 0.142 |
| -0.00328 | NA | NA | + | NA | NA | 3.11E-03 | 6 | 120.705 | -229.41 | 1.903 | 0.135 |
| 0.00307 | NA | NA | + | NA | NA | NA | 5 | 118.107 | -226.215 | 0 | 0.298 |
| 0.0206 | NA | NA | + | 9.12E-05 | NA | NA | 6 | 118.774 | -225.549 | 0.666 | 0.214 |
| 0.02056 | NA | NA | + | NA | -7.71E-03 | NA | 6 | 118.308 | -224.616 | 1.599 | 0.134 |
| 0.04821 | NA | NA | NA | 1.56E-04 | NA | NA | 3 | 115.286 | -224.571 | 1.644 | 0.131 |
| 0.00183 | 7.21E-11 | NA | + | NA | NA | NA | 6 | 118.135 | -224.269 | 1.946 | 0.113 |
| 0.00637 | NA | NA | + | NA | NA | -1.40E-03 | 6 | 118.118 | -224.237 | 1.978 | 0.111 |
| 0.00329 | NA | NA | + | NA | NA | NA | 5 | 127.22 | -244.439 | 0 | 0.369 |
| 0.02333 | NA | NA | + | NA | -9.01E-03 | NA | 6 | 127.554 | -243.107 | 1.332 | 0.19 |
| 0.00999 | NA | NA | + | 4.03E-05 | NA | NA | 6 | 127.376 | -242.751 | 1.688 | 0.159 |
| -0.00385 | NA | NA | + | NA | NA | 2.97E-03 | 6 | 127.271 | -242.541 | 1.898 | 0.143 |
| 0.00216 | 6.34E-11 | NA | + | NA | NA | NA | 6 | 127.249 | -242.497 | 1.942 | 0.14 |
| 0.00723 | NA | NA | + | NA | NA | NA | 5 | 124.236 | -238.473 | 0 | 0.356 |
| 0.01853 | NA | NA | + | 6.52E-05 | NA | NA | 6 | 124.601 | -237.202 | 1.271 | 0.189 |
| 0.02912 | NA | NA | + | NA | -9.69E-03 | NA | 6 | 124.591 | -237.183 | I.29 | 0.187 |
| 0.01327 | NA | NA | + | NA | NA | -2.49E-03 | 6 | 124.268 | -236.535 | 1.937 | 0.135 |
| 0.00637 | 5.14E-11 | NA | + | NA | NA | NA | 6 | 124.259 | -236.517 | 1.956 | 0.134 |
| 0.00088 | NA | NA | + | NA | NA | NA | 5 | 125.076 | -240.152 | 0 | 0.264 |
| 0.04736 | NA | NA | NA | 1.57E-04 | NA | NA | 3 | 122.854 | -239.707 | 0.445 | 0.211 |
| 0.0181 | NA | NA | + | 8.83E-05 | NA | NA | 6 | 125.739 | -239.477 | 0.674 | 0.189 |
| 0.02001 | NA | NA | + | NA | -8.35E-03 | NA | 6 | 125.322 | -238.644 | 1.507 | 0.124 |
| 0.01204 | NA | NA | + | NA | NA | -4.74E-03 | 6 | 125.188 | -238.376 | 1.775 | 0.109 |
| -0.00078 | 9.65E-11 | NA | + | NA | NA | NA | 6 | 125.132 | -238.264 | 1.888 | 0.103 |
| -0.00034 | NA | NA | + | NA | NA | NA | 5 | 120.713 | -231.426 | 0 | 0.354 |
| 0.02421 | NA | NA | + | NA | -1.08E-02 | NA | 6 | 121.16 | -230.32 | 1.106 | 0.204 |
| 0.00965 | NA | NA | + | 5.50E-05 | NA | NA | 6 | 120.963 | -229.927 | 01.V | 0.167 |
| 0.00891 | NA | NA | + | NA | NA | -3.88E-03 | 6 | 120.789 | -229.578 | 1.849 | 0.14 |
| -0.00153 | 6.76E-11 | NA | + | NA | NA | NA | 6 | 120.75 | -229.499 | 1.927 | 0.135 |
| 0.00411 | NA | NA | + | NA | NA | NA | 5 | 124.51 | -239.02 | 0 | 0.348 |
| 0.01408 | NA | NA | + | 6.61E-05 | NA | NA | 6 | 124.961 | -237.923 | 1.097 | 0.201 |
| 0.02266 | NA | NA | + | NA | -8.26E-03 | NA | 6 | 124.845 | -237.69 | I.33 | 0.179 |
| 0.00234 | 1.06E-10 | NA | + | NA | NA | NA | 6 | 124.581 | -237.162 | 1.858 | 0.138 |
| -0.0029 | NA | NA | + | NA | NA | 2.90E-03 | 6 | 124.554 | -237.108 | 1.912 | 0.134 |
| 0.00526 | NA | NA | + | NA | NA | NA | 5 | 124.555 | -239.109 | 0 | 0.368 |
| 0.01593 | NA | NA | + | 6.64E-05 | NA | NA | 6 | 124.96 | -237.921 | 1.188 | 0.203 |
| 0.01847 | NA | NA | + | NA | -5.91E-03 | NA | 6 | 124.696 | -237.392 | 1.717 | 0.156 |
| 0.00463 | 3.84E-11 | NA | + | NA | NA | NA | 6 | 124.573 | -237.145 | 1.964 | 0.138 |
| 0.00377 | NA | NA | + | NA | NA | 6.20E-04 | 6 | 124.557 | -237.113 | 1.996 | 0.136 |
| 0.00245 | NA | NA | + | NA | NA | NA | 5 | 118.095 | -226.189 | 0 | 0.357 |
| 0.01715 | NA | NA | + | 7.88E-05 | NA | NA | 6 | 118.592 | -225.184 | 1.005 | 0.216 |
| 0.01927 | NA | NA | + | NA | -7.34E-03 | NA | 6 | 118.278 | -224.555 | 1.634 | 0.158 |
| 0.00103 | 8.34E-11 | NA | + | NA | NA | NA | 6 | 118.131 | -224.263 | 1.927 | 0.136 |
| 0.00428 | NA | NA | + | NA | NA | -7.79E-04 | 6 | 118.098 | -224.195 | 1.994 | 0.132 |
| 0.0033 | NA | NA | + | NA | NA | NA | 5 | 124.842 | -239.684 | 0 | 0.365 |
| 0.01522 | NA | NA | + | 6.55E-05 | NA | NA | 6 | 125.221 | -238.443 | 1.242 | 0.196 |
| 0.02039 | NA | NA | + | NA | -7.53E-03 | NA | 6 | 125.058 | -238.116 | 1.568 | 0.167 |
| 0.00256 | 4.29E-11 | NA | + | NA | NA | NA | 6 | 124.858 | -237.715 | 1.969 | 0.137 |
| 0.00439 | NA | NA | + | NA | NA | -4.62E-04 | 6 | 124.843 | -237.687 | 1.998 | 0.135 |
| 0.00454 | NA | NA | + | NA | NA | NA | 5 | 121.443 | -232.885 | 0 | 0.321 |
| 0.01665 | NA | NA | + | 6.93E-05 | NA | NA | 6 | 121.854 | -231.708 | 1.178 | 0.178 |
| 0.01771 | NA | NA | + | NA | -5.85E-03 | NA | 6 | 121.582 | -231.164 | 1.722 | 0.136 |
| 0.04835 | NA | NA | NA | 1.42E-04 | NA | NA | 3 | 118.487 | -230.974 | 1.912 | 0.123 |
| 0.00328 | 7.50E-11 | NA | + | NA | NA | NA | 6 | 121.479 | -230.958 | 1.927 | 0.122 |
| -0.00023 | NA | NA | + | NA | NA | 1.97E-03 | 6 | 121.463 | -230.926 | I.96 | 0.12 |
| 0.00238 | NA | NA | + | NA | NA | NA | 5 | 123.353 | -236.706 | 0 | 0.304 |
| 0.01777 | NA | NA | + | 8.82E-05 | NA | NA | 6 | 124.05 | -236.101 | 0.605 | 0.225 |
| 0.01675 | NA | NA | + | NA | -6.20E-03 | NA | 6 | 123.504 | -235.008 | 1.698 | 0.13 |
| 0.04942 | NA | NA | NA | 1.55E-04 | NA | NA | 3 | 120.385 | -234.77 | 1.936 | 0.115 |
| 0.00179 | 3.54E-11 | NA | + | NA | NA | NA | 6 | 123.371 | -234.742 | 1.964 | 0.114 |
| 0.00019 | NA | NA | + | NA | NA | 9.32E-04 | 6 | 123.357 | -234.715 | 1.991 | 0.112 |
| 0.00036 | NA | NA | + | NA | NA | NA | 5 | 119.836 | -229.672 | 0 | 0.311 |
| 0.01736 | NA | NA | + | 8.36E-05 | NA | NA | 6 | 120.324 | -228.648 | 1.024 | 0.187 |
| 0.01936 | NA | NA | + | NA | -8.25E-03 | NA | 6 | 120.048 | -228.095 | 1.576 | 0.142 |
| 0.00917 | NA | NA | + | NA | NA | -3.74E-03 | 6 | 119.906 | -227.812 | I.86 | 0.123 |
| 0.04589 | NA | NA | NA | 1.54E-04 | NA | NA | 3 | 116.897 | -227.793 | 1.878 | 0.122 |
| -0.00034 | 3.76E-11 | NA | + | NA | NA | NA | 6 | 119.848 | -227.696 | 1.976 | 0.116 |
| 0.00154 | NA | NA | + | NA | NA | NA | 5 | 119.986 | -229.972 | 0 | 0.34 |
| 0.01323 | NA | NA | + | 6.98E-05 | NA | NA | 6 | 120.464 | -228.928 | 1.044 | 0.202 |
| 0.02033 | NA | NA | + | NA | -8.26E-03 | NA | 6 | 120.312 | -228.623 | 1.349 | 0.173 |
| -0.01106 | NA | NA | + | NA | NA | 5.43E-03 | 6 | 120.157 | -228.314 | 1.657 | 0.149 |
| -0.00093 | 1.37E-10 | NA | + | NA | NA | NA | 6 | 120.065 | -228.131 | 1.841 | 0.136 |
| 0.00003 | NA | NA | + | NA | NA | NA | 5 | 123.743 | -237.487 | 0 | 0.291 |
| 0.01629 | NA | NA | + | 8.68E-05 | NA | NA | 6 | 124.392 | -236.785 | 0.702 | 0.205 |
| 0.02104 | NA | NA | + | NA | -9.18E-03 | NA | 6 | 124.04 | -236.08 | 1.407 | 0.144 |
| 0.04789 | NA | NA | NA | 1.62E-04 | NA | NA | 3 | 121.001 | -236.003 | 1.484 | 0.139 |
| 0.0059 | NA | NA | + | NA | NA | -2.43E-03 | 6 | 123.774 | -235.548 | 1.939 | 0.111 |
| -0.00091 | 5.75E-11 | NA | + | NA | NA | NA | 6 | 123.772 | -235.543 | 1.943 | 0.11 |
| 0.00564 | NA | NA | + | NA | NA | NA | 5 | 120.242 | -230.483 | 0 | 0.348 |
| 0.01825 | NA | NA | + | 7.49E-05 | NA | NA | 6 | 120.724 | -229.448 | 1.035 | 0.208 |
| 0.02678 | NA | NA | + | NA | -9.35E-03 | NA | 6 | 120.584 | -229.168 | 1.315 | 0.18 |
| 0.00411 | 8.79E-11 | NA | + | NA | NA | NA | 6 | 120.291 | -228.583 | 01.IX | 0.135 |
| 0.0089 | NA | NA | + | NA | NA | -1.35E-03 | 6 | 120.25 | -228.501 | 1.982 | 0.129 |
| 0.0057 | NA | NA | + | NA | NA | NA | 5 | 124.179 | -238.359 | 0 | 0.298 |
| 0.04717 | NA | NA | NA | 1.39E-04 | NA | NA | 3 | 121.73 | -237.461 | 0.898 | 0.19 |
| 0.01939 | NA | NA | + | 7.39E-05 | NA | NA | 6 | 124.623 | -237.246 | 1.112 | 0.171 |
| 0.01752 | NA | NA | + | NA | -5.17E-03 | NA | 6 | 124.271 | -236.542 | 1.817 | 0.12 |
| 0.00468 | 6.04E-11 | NA | + | NA | NA | NA | 6 | 124.2 | -236.4 | 1.958 | 0.112 |
| 0.00695 | NA | NA | + | NA | NA | -5.31E-04 | 6 | 124.181 | -236.362 | 1.997 | 0.11 |
| 0.00535 | NA | NA | + | NA | NA | NA | 5 | 117.069 | -224.137 | 0 | 0.301 |
| 0.02184 | NA | NA | + | 9.40E-05 | NA | NA | 6 | 117.744 | -223.488 | 0.649 | 0.218 |
| 0.04673 | NA | NA | NA | 1.49E-04 | NA | NA | 3 | 114.319 | -222.638 | 1.499 | 0.142 |
| 0.00389 | 8.32E-11 | NA | + | NA | NA | NA | 6 | 117.1 | -222.201 | 1.937 | 0.114 |
| 0.01047 | NA | NA | + | NA | -2.24E-03 | NA | 6 | 117.086 | -222.172 | 1.966 | 0.113 |
| 0.00258 | NA | NA | + | NA | NA | 1.15E-03 | 6 | 117.076 | -222.152 | 1.986 | 0.112 |
| 0.00633 | NA | NA | + | NA | NA | NA | 5 | 123.949 | -237.899 | 0 | 0.352 |
| 0.01824 | NA | NA | + | 7.16E-05 | NA | NA | 6 | 124.407 | -236.815 | 1.084 | 0.205 |
| 0.02563 | NA | NA | + | NA | -8.58E-03 | NA | 6 | 124.251 | -236.503 | 1.396 | 0.175 |
| 0.00475 | 9.54E-11 | NA | + | NA | NA | NA | 6 | 124.008 | -236.017 | 1.882 | 0.137 |
| 0.01 | NA | NA | + | NA | NA | -1.53E-03 | 6 | 123.961 | -235.921 | 1.977 | 0.131 |
| 0.00354 | NA | NA | + | NA | NA | NA | 5 | 122.476 | -234.952 | 0 | 0.351 |
| 0.01872 | NA | NA | + | 8.22E-05 | NA | NA | 6 | 123.03 | -234.059 | 0.892 | 0.225 |
| 0.02102 | NA | NA | + | NA | -7.78E-03 | NA | 6 | 122.687 | -233.374 | 1.578 | 0.16 |
| 0.00218 | 8.16E-11 | NA | + | NA | NA | NA | 6 | 122.517 | -233.033 | 1.918 | 0.135 |
| 0.00392 | NA | NA | + | NA | NA | -1.60E-04 | 6 | 122.476 | -232.952 | 2 | 0.129 |
| 0.00427 | NA | NA | + | NA | NA | NA | 5 | 130.277 | -250.554 | 0 | 0.328 |
| 0.02999 | NA | NA | + | NA | -1.14E-02 | NA | 6 | 130.843 | -249.686 | 0.869 | 0.212 |
| 0.01646 | NA | NA | + | 7.26E-05 | NA | NA | 6 | 130.806 | -249.611 | 0.943 | 0.204 |
| 0.00254 | 1.05E-10 | NA | + | NA | NA | NA | 6 | 130.345 | -248.69 | 1.865 | 0.129 |
| 0.01173 | NA | NA | + | NA | NA | -3.12E-03 | 6 | 130.328 | -248.655 | 1.899 | 0.127 |
| 0.00105 | NA | NA | + | NA | NA | NA | 5 | 127.119 | -244.237 | 0 | 0.344 |
| 0.02396 | NA | NA | + | NA | -1.01E-02 | NA | 6 | 127.616 | -243.231 | 1.006 | 0.208 |
| 0.01113 | NA | NA | + | 6.10E-05 | NA | NA | 6 | 127.495 | -242.991 | 1.247 | 0.184 |
| -0.00065 | 9.87E-11 | NA | + | NA | NA | NA | 6 | 127.181 | -242.361 | 1.876 | 0.135 |
| -0.00345 | NA | NA | + | NA | NA | 1.90E-03 | 6 | 127.139 | -242.279 | 1.959 | 0.129 |
| 0.00458 | NA | NA | + | NA | NA | NA | 5 | 123.192 | -236.383 | 0 | 0.359 |
| 0.01825 | NA | NA | + | 7.55E-05 | NA | NA | 6 | 123.649 | -235.297 | 1.086 | 0.209 |
| 0.02151 | NA | NA | + | NA | -7.42E-03 | NA | 6 | 123.388 | -234.777 | 1.607 | 0.161 |
| 0.00319 | 8.04E-11 | NA | + | NA | NA | NA | 6 | 123.226 | -234.452 | 1.932 | 0.137 |
| 0.00875 | NA | NA | + | NA | NA | -1.73E-03 | 6 | 123.207 | -234.414 | I.97 | 0.134 |
| 0.00196 | NA | NA | + | NA | NA | NA | 5 | 128.353 | -246.705 | 0 | 0.353 |
| 0.02682 | NA | NA | + | NA | -1.09E-02 | NA | 6 | 128.819 | -245.638 | 1.067 | 0.207 |
| 0.01236 | NA | NA | + | 5.69E-05 | NA | NA | 6 | 128.656 | -245.311 | 1.394 | 0.176 |
| 0.00115 | 4.87E-11 | NA | + | NA | NA | NA | 6 | 128.377 | -244.754 | 1.951 | 0.133 |
| 0.00226 | NA | NA | + | NA | NA | -1.28E-04 | 6 | 128.353 | -244.705 | 2 | 0.13 |
| 0.00225 | NA | NA | + | NA | NA | NA | 5 | 122.192 | -234.383 | 0 | 0.348 |
| 0.02324 | NA | NA | + | NA | -9.30E-03 | NA | 6 | 122.626 | -233.252 | 1.132 | 0.198 |
| 0.01243 | NA | NA | + | 5.96E-05 | NA | NA | 6 | 122.537 | -233.073 | I.31 | 0.181 |
| -0.00642 | NA | NA | + | NA | NA | 3.67E-03 | 6 | 122.269 | -232.537 | 1.846 | 0.138 |
| 0.00078 | 8.70E-11 | NA | + | NA | NA | NA | 6 | 122.247 | -232.493 | I.89 | 0.135 |
| 0.00099 | NA | NA | + | NA | NA | NA | 5 | 122.149 | -234.298 | 0 | 0.345 |
| 0.01718 | NA | NA | + | 8.84E-05 | NA | NA | 6 | 122.769 | -233.538 | 0.76 | 0.236 |
| 0.01803 | NA | NA | + | NA | -7.58E-03 | NA | 6 | 122.348 | -232.696 | 1.603 | 0.155 |
| -0.00082 | 9.86E-11 | NA | + | NA | NA | NA | 6 | 122.209 | -232.419 | I.88 | 0.135 |
| 0.00545 | NA | NA | + | NA | NA | -1.87E-03 | 6 | 122.167 | -232.333 | 1.965 | 0.129 |
| 0.00074 | NA | NA | + | NA | NA | NA | 5 | 130.384 | -250.769 | 0 | 0.357 |
| 0.02246 | NA | NA | + | NA | -9.55E-03 | NA | 6 | 130.797 | -249.595 | 1.174 | 0.199 |
| 0.01027 | NA | NA | + | 5.38E-05 | NA | NA | 6 | 130.679 | -249.358 | I.41 | 0.177 |
| -0.00037 | 6.67E-11 | NA | + | NA | NA | NA | 6 | 130.418 | -248.835 | 1.933 | 0.136 |
| 0.00169 | NA | NA | + | NA | NA | -4.04E-04 | 6 | 130.385 | -248.77 | 1.998 | 0.132 |
| 0.00453 | NA | NA | + | NA | NA | NA | 5 | 124.671 | -239.341 | 0 | 0.368 |
| 0.02603 | NA | NA | + | NA | -9.68E-03 | NA | 6 | 125.073 | -238.146 | 1.195 | 0.203 |
| 0.01116 | NA | NA | + | 3.86E-05 | NA | NA | 6 | 124.809 | -237.618 | 1.723 | 0.156 |
| 0.00384 | 3.94E-11 | NA | + | NA | NA | NA | 6 | 124.683 | -237.365 | 1.976 | 0.137 |
| 0.00696 | NA | NA | + | NA | NA | -1.01E-03 | 6 | 124.676 | -237.353 | 1.988 | 0.136 |
| 0.00666 | NA | NA | + | NA | NA | NA | 5 | 122.907 | -235.814 | 0 | 0.348 |
| 0.01968 | NA | NA | + | 7.59E-05 | NA | NA | 6 | 123.415 | -234.83 | 0.984 | 0.213 |
| 0.02749 | NA | NA | + | NA | -9.23E-03 | NA | 6 | 123.245 | -234.49 | 1.324 | 0.18 |
| 0.00597 | 4.08E-11 | NA | + | NA | NA | NA | 6 | 122.923 | -233.847 | 1.968 | 0.13 |
| 0.00955 | NA | NA | + | NA | NA | -1.21E-03 | 6 | 122.915 | -233.829 | 1.985 | 0.129 |
| 0.00729 | NA | NA | + | NA | NA | NA | 5 | 127.344 | -244.689 | 0 | 0.361 |
| 0.01733 | NA | NA | + | 5.96E-05 | NA | NA | 6 | 127.669 | -243.339 | I.35 | 0.184 |
| 0.02672 | NA | NA | + | NA | -8.75E-03 | NA | 6 | 127.661 | -243.323 | 1.366 | 0.182 |
| 0.01371 | NA | NA | + | NA | NA | -2.65E-03 | 6 | 127.38 | -242.76 | 1.929 | 0.138 |
| 0.00665 | 3.83E-11 | NA | + | NA | NA | NA | 6 | 127.36 | -242.72 | 1.969 | 0.135 |
| 0.00739 | NA | NA | + | NA | NA | NA | 5 | 120.923 | -231.846 | 0 | 0.374 |
| 0.01778 | NA | NA | + | 5.70E-05 | NA | NA | 6 | 121.185 | -230.371 | 1.475 | 0.179 |
| 0.02398 | NA | NA | + | NA | -7.23E-03 | NA | 6 | 121.122 | -230.245 | 1.601 | 0.168 |
| 0.0067 | 3.93E-11 | NA | + | NA | NA | NA | 6 | 120.934 | -229.868 | 1.978 | 0.139 |
| 0.01071 | NA | NA | + | NA | NA | -1.39E-03 | 6 | 120.933 | -229.865 | I.98 | 0.139 |
| 0.00167 | NA | NA | + | NA | NA | NA | 5 | 125.838 | -241.676 | 0 | 0.345 |
| 0.02441 | NA | NA | + | NA | -1.03E-02 | NA | 6 | 126.364 | -240.727 | 0.948 | 0.215 |
| 0.00995 | NA | NA | + | 5.26E-05 | NA | NA | 6 | 126.131 | -240.262 | 1.414 | 0.17 |
| -0.00628 | NA | NA | + | NA | NA | 3.31E-03 | 6 | 125.899 | -239.799 | 1.877 | 0.135 |
| 0.00006 | 9.30E-11 | NA | + | NA | NA | NA | 6 | 125.895 | -239.789 | 1.887 | 0.134 |
| 0.00659 | NA | NA | + | NA | NA | NA | 5 | 129.582 | -249.163 | 0 | 0.215 |
| 0.02364 | NA | NA | + | 8.95E-05 | NA | NA | 6 | 130.296 | -248.592 | 0.571 | 0.161 |
| 0.05139 | NA | NA | NA | 1.54E-04 | NA | NA | 3 | 127.264 | -248.527 | 0.636 | 0.156 |
| 0.03099 | NA | NA | + | NA | -1.08E-02 | NA | 6 | 130.026 | -248.052 | 1.111 | 0.123 |
| 0.01898 | NA | NA | + | NA | NA | -5.22E-03 | 6 | 129.72 | -247.44 | 1.723 | 0.091 |
| 0.04502 | NA | NA | + | 8.48E-05 | -9.82E-03 | NA | 7 | 130.668 | -247.335 | 1.828 | 0.086 |
| 0.07066 | NA | NA | NA | 1.42E-04 | -1.00E-02 | NA | 4 | 127.65 | -247.301 | 1.862 | 0.085 |
| 0.00548 | 6.82E-11 | NA | + | NA | NA | NA | 6 | 129.622 | -247.244 | 1.919 | 0.082 |
| -0.00043 | NA | NA | + | NA | NA | NA | 5 | 122.304 | -234.608 | 0 | 0.289 |
| 0.01617 | NA | NA | + | 9.15E-05 | NA | NA | 6 | 123.051 | -234.101 | 0.507 | 0.225 |
| 0.01724 | NA | NA | + | NA | -7.62E-03 | NA | 6 | 122.527 | -233.054 | 1.554 | 0.133 |
| 0.04725 | NA | NA | NA | 1.58E-04 | NA | NA | 3 | 119.506 | -233.012 | 1.596 | 0.13 |
| -0.00241 | 1.14E-10 | NA | + | NA | NA | NA | 6 | 122.385 | -232.77 | 1.838 | 0.115 |
| 0.00204 | NA | NA | + | NA | NA | -1.05E-03 | 6 | 122.31 | -232.619 | 1.989 | 0.107 |
| 0.00248 | NA | NA | + | NA | NA | NA | 5 | 118.001 | -226.003 | 0 | 0.275 |
| 0.04473 | NA | NA | NA | 1.43E-04 | NA | NA | 3 | 115.822 | -225.644 | 0.359 | 0.23 |
| 0.01952 | NA | NA | + | 8.40E-05 | NA | NA | 6 | 118.523 | -225.046 | 0.957 | 0.171 |
| 0.01842 | NA | NA | + | NA | -7.00E-03 | NA | 6 | 118.148 | -224.296 | 1.707 | 0.117 |
| 0.00117 | 7.20E-11 | NA | + | NA | NA | NA | 6 | 118.036 | -224.072 | 1.931 | 0.105 |
| 0.00157 | NA | NA | + | NA | NA | 3.83E-04 | 6 | 118.002 | -224.004 | 1.998 | 0.101 |
| 0.00423 | NA | NA | + | NA | NA | NA | 5 | 126.798 | -243.596 | 0 | 0.36 |
| 0.01601 | NA | NA | + | 6.73E-05 | NA | NA | 6 | 127.21 | -242.42 | 1.176 | 0.2 |
| 0.02179 | NA | NA | + | NA | -7.70E-03 | NA | 6 | 127.054 | -242.108 | 1.489 | 0.171 |
| 0.00331 | 5.07E-11 | NA | + | NA | NA | NA | 6 | 126.817 | -241.634 | 1.962 | 0.135 |
| 0.00026 | NA | NA | + | NA | NA | 1.68E-03 | 6 | 126.814 | -241.628 | 1.968 | 0.134 |
| 0.00585 | NA | NA | + | NA | NA | NA | 5 | 122.457 | -234.913 | 0 | 0.373 |
| 0.02512 | NA | NA | + | NA | -8.51E-03 | NA | 6 | 122.715 | -233.43 | 1.483 | 0.178 |
| 0.01375 | NA | NA | + | 4.55E-05 | NA | NA | 6 | 122.63 | -233.26 | 1.653 | 0.163 |
| 0.01508 | NA | NA | + | NA | NA | -3.93E-03 | 6 | 122.534 | -233.069 | 1.845 | 0.148 |
| 0.00505 | 4.41E-11 | NA | + | NA | NA | NA | 6 | 122.468 | -232.935 | 1.978 | 0.139 |
| 0.00224 | NA | NA | + | NA | NA | NA | 5 | 121.45 | -232.899 | 0 | 0.333 |
| 0.01578 | NA | NA | + | 7.72E-05 | NA | NA | 6 | 121.988 | -231.976 | 0.923 | 0.21 |
| 0.02781 | NA | NA | + | NA | -1.12E-02 | NA | 6 | 121.975 | -231.951 | 0.948 | 0.207 |
| 0.00122 | 6.68E-11 | NA | + | NA | NA | NA | 6 | 121.494 | -230.988 | 1.911 | 0.128 |
| 0.00065 | NA | NA | + | NA | NA | 6.63E-04 | 6 | 121.452 | -230.903 | 1.996 | 0.123 |
| -0.00138 | NA | NA | + | NA | NA | NA | 5 | 125.155 | -240.31 | 0 | 0.347 |
| 0.02367 | NA | NA | + | NA | -1.10E-02 | NA | 6 | 125.697 | -239.394 | 0.916 | 0.22 |
| 0.00737 | NA | NA | + | 5.26E-05 | NA | NA | 6 | 125.431 | -238.862 | 1.449 | 0.168 |
| -0.00247 | 6.05E-11 | NA | + | NA | NA | NA | 6 | 125.192 | -238.385 | 1.926 | 0.133 |
| -0.0072 | NA | NA | + | NA | NA | 2.47E-03 | 6 | 125.192 | -238.384 | 1.927 | 0.132 |
| 0.00449 | NA | NA | + | NA | NA | NA | 5 | 118.379 | -226.759 | 0 | 0.359 |
| 0.02715 | NA | NA | + | NA | -1.02E-02 | NA | 6 | 118.744 | -225.488 | 1.271 | 0.19 |
| 0.01554 | NA | NA | + | 6.01E-05 | NA | NA | 6 | 118.675 | -225.35 | 1.409 | 0.177 |
| 0.00275 | 9.67E-11 | NA | + | NA | NA | NA | 6 | 118.433 | -224.866 | 1.893 | 0.139 |
| 0.00982 | NA | NA | + | NA | NA | -2.25E-03 | 6 | 118.403 | -224.807 | 1.952 | 0.135 |
| 0.00263 | NA | NA | + | NA | NA | NA | 5 | 120.807 | -231.614 | 0 | 0.313 |
| 0.01591 | NA | NA | + | 6.94E-05 | NA | NA | 6 | 121.202 | -230.403 | I.21 | 0.171 |
| 0.02241 | NA | NA | + | NA | -8.61E-03 | NA | 6 | 121.058 | -230.117 | 1.497 | 0.148 |
| 0.04828 | NA | NA | NA | 1.40E-04 | NA | NA | 3 | 117.908 | -229.816 | 1.798 | 0.127 |
| 0.01184 | NA | NA | + | NA | NA | -4.00E-03 | 6 | 120.885 | -229.77 | 1.844 | 0.124 |
| 0.00213 | 2.87E-11 | NA | + | NA | NA | NA | 6 | 120.817 | -229.633 | I.98 | 0.116 |
| 0.0042 | NA | NA | + | NA | NA | NA | 5 | 128.548 | -247.096 | 0 | 0.286 |
| 0.01978 | NA | NA | + | 8.44E-05 | NA | NA | 6 | 129.175 | -246.349 | 0.747 | 0.197 |
| 0.04761 | NA | NA | NA | 1.53E-04 | NA | NA | 3 | 125.922 | -245.844 | 1.252 | 0.153 |
| 0.02361 | NA | NA | + | NA | -8.60E-03 | NA | 6 | 128.829 | -245.657 | 1.439 | 0.139 |
| 0.01463 | NA | NA | + | NA | NA | -4.38E-03 | 6 | 128.64 | -245.281 | 1.815 | 0.115 |
| 0.00281 | 8.45E-11 | NA | + | NA | NA | NA | 6 | 128.602 | -245.204 | 1.892 | 0.111 |
| -0.00027 | NA | NA | + | NA | NA | NA | 5 | 120.892 | -231.785 | 0 | 0.358 |
| 0.01292 | NA | NA | + | 7.70E-05 | NA | NA | 6 | 121.415 | -230.83 | 0.954 | 0.222 |
| -0.00258 | 1.38E-10 | NA | + | NA | NA | NA | 6 | 120.98 | -229.96 | 1.825 | 0.144 |
| 0.00978 | NA | NA | + | NA | -4.38E-03 | NA | 6 | 120.972 | -229.944 | 1.841 | 0.142 |
| -0.00526 | NA | NA | + | NA | NA | 2.13E-03 | 6 | 120.915 | -229.829 | 1.955 | 0.135 |
| 0.00412 | NA | NA | + | NA | NA | NA | 5 | 117.134 | -224.269 | 0 | 0.239 |
| 0.04574 | NA | NA | NA | 1.54E-04 | NA | NA | 3 | 114.988 | -223.976 | 0.293 | 0.206 |
| 0.01935 | NA | NA | + | 8.79E-05 | NA | NA | 6 | 117.769 | -223.539 | 0.73 | 0.166 |
| 0.0202 | NA | NA | + | NA | -7.24E-03 | NA | 6 | 117.361 | -222.722 | 1.547 | 0.11 |
| 0.00193 | 1.33E-10 | NA | + | NA | NA | NA | 6 | 117.227 | -222.455 | 1.814 | 0.096 |
| -0.00388 | NA | NA | + | NA | NA | 3.25E-03 | 6 | 117.187 | -222.375 | 1.894 | 0.093 |
| 0.05723 | NA | NA | NA | 1.44E-04 | -6.21E-03 | NA | 4 | 115.15 | -222.301 | 1.968 | 0.089 |
| -0.00137 | NA | NA | + | NA | NA | NA | 5 | 116.974 | -223.947 | 0 | 0.324 |
| 0.02097 | NA | NA | + | NA | -9.90E-03 | NA | 6 | 117.497 | -222.994 | 0.954 | 0.201 |
| 0.00861 | NA | NA | + | 6.41E-05 | NA | NA | 6 | 117.401 | -222.801 | 1.146 | 0.183 |
| -0.01919 | NA | NA | + | NA | NA | 7.60E-03 | 6 | 117.315 | -222.631 | 1.316 | 0.168 |
| -0.00278 | 8.10E-11 | NA | + | NA | NA | NA | 6 | 117.014 | -222.027 | I.92 | 0.124 |
| 0.00582 | NA | NA | + | NA | NA | NA | 5 | 127.907 | -245.815 | 0 | 0.357 |
| 0.01646 | NA | NA | + | 6.16E-05 | NA | NA | 6 | 128.265 | -244.53 | 1.285 | 0.188 |
| 0.02663 | NA | NA | + | NA | -9.19E-03 | NA | 6 | 128.265 | -244.53 | 1.285 | 0.188 |
| 0.01081 | NA | NA | + | NA | NA | -2.07E-03 | 6 | 127.931 | -243.862 | 1.952 | 0.134 |
| 0.00506 | 4.47E-11 | NA | + | NA | NA | NA | 6 | 127.925 | -243.849 | 1.965 | 0.134 |
| 0.0058 | NA | NA | + | NA | NA | NA | 5 | 126.729 | -243.458 | 0 | 0.357 |
| 0.02573 | NA | NA | + | NA | -9.08E-03 | NA | 6 | 127.09 | -242.18 | 1.277 | 0.188 |
| 0.01465 | NA | NA | + | 5.73E-05 | NA | NA | 6 | 127.05 | -242.101 | 1.357 | 0.181 |
| 0.00418 | 1.00E-10 | NA | + | NA | NA | NA | 6 | 126.786 | -241.571 | 1.886 | 0.139 |
| 0.00082 | NA | NA | + | NA | NA | 2.04E-03 | 6 | 126.751 | -241.501 | 1.956 | 0.134 |
| 0.00222 | NA | NA | + | NA | NA | NA | 5 | 128.198 | -246.397 | 0 | 0.361 |
| 0.01291 | NA | NA | + | 6.19E-05 | NA | NA | 6 | 128.557 | -245.113 | 1.283 | 0.19 |
| 0.01946 | NA | NA | + | NA | -7.58E-03 | NA | 6 | 128.439 | -244.878 | 1.518 | 0.169 |
| 0.01046 | NA | NA | + | NA | NA | -3.52E-03 | 6 | 128.26 | -244.52 | 1.877 | 0.141 |
| 0.001 | 7.44E-11 | NA | + | NA | NA | NA | 6 | 128.235 | -244.469 | 1.927 | 0.138 |
| 0.00094 | NA | NA | + | NA | NA | NA | 5 | 117.667 | -225.334 | 0 | 0.248 |
| 0.04877 | NA | NA | NA | 1.53E-04 | NA | NA | 3 | 115.304 | -224.608 | 0.726 | 0.172 |
| 0.0161 | NA | NA | + | 8.29E-05 | NA | NA | 6 | 118.224 | -224.447 | 0.887 | 0.159 |
| 0.02473 | NA | NA | + | NA | -1.03E-02 | NA | 6 | 118.045 | -224.091 | 1.244 | 0.133 |
| -0.00122 | 1.30E-10 | NA | + | NA | NA | NA | 6 | 117.752 | -223.503 | 1.831 | 0.099 |
| 0.00821 | NA | NA | + | NA | NA | -3.11E-03 | 6 | 117.711 | -223.423 | 1.912 | 0.095 |
| 0.06869 | NA | NA | NA | 1.37E-04 | -1.05E-02 | NA | 4 | 115.695 | -223.39 | 1.945 | 0.094 |
| -0.00038 | NA | NA | + | NA | NA | NA | 5 | 126.185 | -242.37 | 0 | 0.334 |
| 0.02666 | NA | NA | + | NA | -1.22E-02 | NA | 6 | 126.959 | -241.919 | 0.451 | 0.266 |
| 0.00536 | NA | NA | + | 3.70E-05 | NA | NA | 6 | 126.337 | -240.674 | 1.696 | 0.143 |
| -0.00218 | 1.10E-10 | NA | + | NA | NA | NA | 6 | 126.262 | -240.524 | 1.846 | 0.133 |
| -0.00394 | NA | NA | + | NA | NA | 1.48E-03 | 6 | 126.197 | -240.393 | 1.976 | 0.124 |
| 0.00595 | NA | NA | + | NA | NA | NA | 5 | 131.723 | -253.445 | 0 | 0.341 |
| 0.0182 | NA | NA | + | 7.20E-05 | NA | NA | 6 | 132.244 | -252.488 | 0.957 | 0.211 |
| 0.027 | NA | NA | + | NA | -9.32E-03 | NA | 6 | 132.133 | -252.266 | 1.179 | 0.189 |
| 0.00454 | 8.54E-11 | NA | + | NA | NA | NA | 6 | 131.768 | -251.535 | I.91 | 0.131 |
| 0.00082 | NA | NA | + | NA | NA | 2.16E-03 | 6 | 131.75 | -251.499 | 1.946 | 0.129 |
| 0.00542 | NA | NA | + | NA | NA | NA | 5 | 125.062 | -240.123 | 0 | 0.363 |
| 0.01714 | NA | NA | + | 6.29E-05 | NA | NA | 6 | 125.404 | -238.808 | 1.315 | 0.188 |
| 0.02349 | NA | NA | + | NA | -7.83E-03 | NA | 6 | 125.289 | -238.578 | 1.546 | 0.167 |
| 0.01429 | NA | NA | + | NA | NA | -3.87E-03 | 6 | 125.136 | -238.273 | I.85 | 0.144 |
| 0.00417 | 7.36E-11 | NA | + | NA | NA | NA | 6 | 125.095 | -238.19 | 1.933 | 0.138 |
| 0.00709 | NA | NA | + | NA | NA | NA | 5 | 122.181 | -234.361 | 0 | 0.347 |
| 0.03151 | NA | NA | + | NA | -1.09E-02 | NA | 6 | 122.658 | -233.316 | 1.045 | 0.206 |
| 0.01868 | NA | NA | + | 6.37E-05 | NA | NA | 6 | 122.534 | -233.069 | 1.293 | 0.182 |
| 0.00561 | 8.66E-11 | NA | + | NA | NA | NA | 6 | 122.234 | -232.468 | 1.894 | 0.135 |
| 0.01216 | NA | NA | + | NA | NA | -2.14E-03 | 6 | 122.203 | -232.406 | 1.956 | 0.131 |
| 0.00396 | NA | NA | + | NA | NA | NA | 5 | 130.316 | -250.632 | 0 | 0.351 |
| 0.02568 | NA | NA | + | NA | -9.66E-03 | NA | 6 | 130.782 | -249.564 | 1.067 | 0.206 |
| 0.01077 | NA | NA | + | 4.52E-05 | NA | NA | 6 | 130.541 | -249.082 | I.55 | 0.162 |
| -0.00755 | NA | NA | + | NA | NA | 4.86E-03 | 6 | 130.459 | -248.917 | 1.715 | 0.149 |
| 0.00297 | 6.10E-11 | NA | + | NA | NA | NA | 6 | 130.342 | -248.684 | 1.948 | 0.133 |
| 0.00465 | NA | NA | + | NA | NA | NA | 5 | 111.293 | -212.586 | 0 | 0.373 |
| 0.01527 | NA | NA | + | 6.07E-05 | NA | NA | 6 | 111.577 | -211.153 | 1.433 | 0.182 |
| 0.02186 | NA | NA | + | NA | -7.63E-03 | NA | 6 | 111.5 | -211.001 | 1.585 | 0.169 |
| 0.00373 | 5.14E-11 | NA | + | NA | NA | NA | 6 | 111.307 | -210.615 | 1.972 | 0.139 |
| 0.00453 | NA | NA | + | NA | NA | 4.91E-05 | 6 | 111.293 | -210.586 | 2 | 0.137 |
| 0.00195 | NA | NA | + | NA | NA | NA | 5 | 120.767 | -231.535 | 0 | 0.322 |
| 0.03224 | NA | NA | + | NA | -1.35E-02 | NA | 6 | 121.599 | -231.197 | 0.338 | 0.272 |
| 0.01122 | NA | NA | + | 5.65E-05 | NA | NA | 6 | 121.077 | -230.154 | 1.381 | 0.162 |
| 0.00029 | 9.75E-11 | NA | + | NA | NA | NA | 6 | 120.824 | -229.649 | 1.886 | 0.125 |
| 0.00054 | NA | NA | + | NA | NA | 5.83E-04 | 6 | 120.769 | -229.538 | 1.996 | 0.119 |
| 0.0031 | NA | NA | + | NA | NA | NA | 5 | 121.397 | -232.793 | 0 | 0.353 |
| 0.01833 | NA | NA | + | 8.15E-05 | NA | NA | 6 | 121.937 | -231.873 | 0.92 | 0.223 |
| 0.01889 | NA | NA | + | NA | -6.95E-03 | NA | 6 | 121.565 | -231.13 | 1.664 | 0.153 |
| 0.00104 | 1.17E-10 | NA | + | NA | NA | NA | 6 | 121.466 | -230.932 | 1.862 | 0.139 |
| -0.00144 | NA | NA | + | NA | NA | 1.89E-03 | 6 | 121.417 | -230.834 | I.96 | 0.132 |
| 0.00794 | NA | NA | + | NA | NA | NA | 5 | 126.826 | -243.653 | 0 | 0.31 |
| 0.02125 | NA | NA | + | 8.19E-05 | NA | NA | 6 | 127.446 | -242.893 | 0.76 | 0.212 |
| 0.01979 | NA | NA | + | NA | -5.32E-03 | NA | 6 | 126.945 | -241.889 | 1.764 | 0.128 |
| 0.00622 | 1.06E-10 | NA | + | NA | NA | NA | 6 | 126.88 | -241.759 | 1.893 | 0.12 |
| 0.00483 | NA | NA | + | NA | NA | 1.28E-03 | 6 | 126.835 | -241.67 | 1.983 | 0.115 |
| 0.04778 | NA | NA | NA | 1.42E-04 | NA | NA | 3 | 123.832 | -241.663 | 1.989 | 0.115 |
| -0.00075 | NA | NA | + | NA | NA | NA | 5 | 124.122 | -238.244 | 0 | 0.355 |
| 0.02379 | NA | NA | + | NA | -1.04E-02 | NA | 6 | 124.611 | -237.223 | 1.021 | 0.213 |
| 0.00757 | NA | NA | + | 5.00E-05 | NA | NA | 6 | 124.365 | -236.729 | 1.515 | 0.166 |
| -0.00193 | 6.72E-11 | NA | + | NA | NA | NA | 6 | 124.151 | -236.301 | 1.943 | 0.134 |
| 0.00178 | NA | NA | + | NA | NA | -1.10E-03 | 6 | 124.128 | -236.256 | 1.988 | 0.131 |
| 0.04673 | NA | NA | NA | 1.44E-04 | NA | NA | 3 | 112.883 | -219.765 | 0 | 0.223 |
| 0.0045 | NA | NA | + | NA | NA | NA | 5 | 114.859 | -219.717 | 0.048 | 0.217 |
| 0.02095 | NA | NA | + | 8.89E-05 | NA | NA | 6 | 115.414 | -218.829 | 0.936 | 0.139 |
| 0.05511 | NA | NA | NA | 1.38E-04 | -4.40E-03 | NA | 4 | 112.943 | -217.885 | I.88 | 0.087 |
| 0.01459 | NA | NA | + | NA | -4.39E-03 | NA | 6 | 114.918 | -217.836 | 1.929 | 0.085 |
| 0.0458 | 5.85E-11 | NA | NA | 1.44E-04 | NA | NA | 4 | 112.902 | -217.804 | 1.961 | 0.083 |
| 0.05188 | NA | NA | NA | 1.48E-04 | NA | -1.84E-03 | 4 | 112.897 | -217.793 | 1.972 | 0.083 |
| 0.00322 | 7.31E-11 | NA | + | NA | NA | NA | 6 | 114.89 | -217.779 | 1.986 | 0.082 |
| 0.0059 | NA | NA | + | NA | NA | NA | 5 | 126.025 | -242.051 | 0 | 0.234 |
| 0.04695 | NA | NA | NA | 1.40E-04 | NA | NA | 3 | 123.825 | -241.651 | 0.4 | 0.192 |
| 0.02128 | NA | NA | + | 8.37E-05 | NA | NA | 6 | 126.625 | -241.25 | 0.801 | 0.157 |
| 0.02973 | NA | NA | + | NA | -1.05E-02 | NA | 6 | 126.438 | -240.876 | 1.175 | 0.13 |
| 0.06547 | NA | NA | NA | 1.28E-04 | -9.65E-03 | NA | 4 | 124.176 | -240.351 | 01.VII | 0.1 |
| 0.01816 | NA | NA | + | NA | NA | -5.12E-03 | 6 | 126.149 | -240.299 | 1.752 | 0.097 |
| 0.00459 | 7.41E-11 | NA | + | NA | NA | NA | 6 | 126.068 | -240.137 | 1.914 | 0.09 |
| 0.00057 | NA | NA | + | NA | NA | NA | 5 | 126.757 | -243.514 | 0 | 0.355 |
| 0.0228 | NA | NA | + | NA | -9.74E-03 | NA | 6 | 127.149 | -242.298 | 1.216 | 0.193 |
| 0.01065 | NA | NA | + | 5.77E-05 | NA | NA | 6 | 127.07 | -242.139 | 1.375 | 0.178 |
| -0.00155 | 1.22E-10 | NA | + | NA | NA | NA | 6 | 126.831 | -241.663 | 1.851 | 0.14 |
| 0.00572 | NA | NA | + | NA | NA | -2.20E-03 | 6 | 126.781 | -241.561 | 1.953 | 0.134 |
| 0.00171 | NA | NA | + | NA | NA | NA | 5 | 121.655 | -233.309 | 0 | 0.206 |
| 0.02169 | NA | NA | + | 1.07E-04 | NA | NA | 6 | 122.588 | -233.175 | 0.134 | 0.192 |
| 0.04671 | NA | NA | NA | 1.70E-04 | NA | NA | 3 | 119.582 | -233.164 | 0.146 | 0.191 |
| 0.01826 | NA | NA | + | NA | -7.37E-03 | NA | 6 | 121.852 | -231.704 | 1.605 | 0.092 |
| 0.05909 | NA | NA | NA | 1.61E-04 | -6.59E-03 | NA | 4 | 119.741 | -231.481 | 1.828 | 0.082 |
| 0.03469 | NA | NA | + | 1.04E-04 | -6.07E-03 | NA | 7 | 122.722 | -231.445 | 1.865 | 0.081 |
| 0.00037 | 7.98E-11 | NA | + | NA | NA | NA | 6 | 121.706 | -231.413 | 1.897 | 0.08 |
| 0.00316 | NA | NA | + | NA | NA | -6.00E-04 | 6 | 121.656 | -231.313 | 1.996 | 0.076 |
| 0.00225 | NA | NA | + | NA | NA | NA | 5 | 114.608 | -219.215 | 0 | 0.376 |
| 0.01501 | NA | NA | + | 6.81E-05 | NA | NA | 6 | 114.943 | -217.886 | I.33 | 0.193 |
| 0.01425 | NA | NA | + | NA | -5.17E-03 | NA | 6 | 114.689 | -217.377 | 1.838 | 0.15 |
| 0.00762 | NA | NA | + | NA | NA | -2.29E-03 | 6 | 114.633 | -217.265 | I.95 | 0.142 |
| 0.00172 | 2.71E-11 | NA | + | NA | NA | NA | 6 | 114.613 | -217.225 | I.99 | 0.139 |
| 0.00434 | NA | NA | + | NA | NA | NA | 5 | 129.617 | -249.234 | 0 | 0.359 |
| 0.02431 | NA | NA | + | NA | -8.93E-03 | NA | 6 | 129.962 | -247.923 | 1.311 | 0.186 |
| 0.01397 | NA | NA | + | 5.71E-05 | NA | NA | 6 | 129.932 | -247.865 | 1.369 | 0.181 |
| 0.00245 | 1.13E-10 | NA | + | NA | NA | NA | 6 | 129.681 | -247.363 | 1.871 | 0.141 |
| 0.00521 | NA | NA | + | NA | NA | -3.64E-04 | 6 | 129.618 | -247.236 | 1.999 | 0.132 |
| 0.00858 | NA | NA | + | NA | NA | NA | 5 | 124.583 | -239.167 | 0 | 0.256 |
| 0.02331 | NA | NA | + | 7.69E-05 | NA | NA | 6 | 125.049 | -238.097 | 1.069 | 0.15 |
| 0.03026 | NA | NA | + | NA | NA | -9.08E-03 | 6 | 124.958 | -237.917 | I.25 | 0.137 |
| 0.04496 | NA | NA | NA | 1.31E-04 | NA | NA | 3 | 121.907 | -237.815 | 1.352 | 0.13 |
| 0.02958 | NA | NA | + | NA | -9.43E-03 | NA | 6 | 124.884 | -237.768 | 1.398 | 0.127 |
| 0.05495 | NA | NA | + | 9.58E-05 | NA | -1.17E-02 | 7 | 125.652 | -237.305 | 1.862 | 0.101 |
| 0.00709 | 8.67E-11 | NA | + | NA | NA | NA | 6 | 124.628 | -237.256 | I.91 | 0.099 |
| 0.00557 | NA | NA | + | NA | NA | NA | 5 | 122.881 | -235.761 | 0 | 0.34 |
| 0.02129 | NA | NA | + | 8.63E-05 | NA | NA | 6 | 123.523 | -235.047 | 0.715 | 0.238 |
| 0.0252 | NA | NA | + | NA | -8.71E-03 | NA | 6 | 123.158 | -234.316 | 1.445 | 0.165 |
| 0.00408 | 8.76E-11 | NA | + | NA | NA | NA | 6 | 122.938 | -233.876 | 1.886 | 0.132 |
| 0.00264 | NA | NA | + | NA | NA | 1.22E-03 | 6 | 122.888 | -233.777 | 1.984 | 0.126 |
| 0.00199 | NA | NA | + | NA | NA | NA | 5 | 117.821 | -225.643 | 0 | 0.357 |
| 0.0164 | NA | NA | + | 7.75E-05 | NA | NA | 6 | 118.278 | -224.556 | 1.087 | 0.207 |
| 0.01806 | NA | NA | + | NA | -6.93E-03 | NA | 6 | 117.983 | -223.965 | 1.678 | 0.154 |
| 0.01266 | NA | NA | + | NA | NA | -4.55E-03 | 6 | 117.918 | -223.836 | 1.806 | 0.145 |
| 0.00025 | 9.92E-11 | NA | + | NA | NA | NA | 6 | 117.869 | -223.738 | 1.904 | 0.138 |
| 0.00448 | NA | NA | + | NA | NA | NA | 5 | 128.087 | -246.175 | 0 | 0.357 |
| 0.01657 | NA | NA | + | 6.70E-05 | NA | NA | 6 | 128.502 | -245.005 | I.17 | 0.199 |
| 0.02241 | NA | NA | + | NA | -7.93E-03 | NA | 6 | 128.343 | -244.685 | I.49 | 0.17 |
| 0.01222 | NA | NA | + | NA | NA | -3.24E-03 | 6 | 128.141 | -244.283 | 1.892 | 0.139 |
| 0.00342 | 6.49E-11 | NA | + | NA | NA | NA | 6 | 128.121 | -244.243 | 1.932 | 0.136 |
| 0.00195 | NA | NA | + | NA | NA | NA | 5 | 127.021 | -244.042 | 0 | 0.361 |
| 0.01163 | NA | NA | + | 5.82E-05 | NA | NA | 6 | 127.361 | -242.722 | I.32 | 0.186 |
| 0.01992 | NA | NA | + | NA | -7.89E-03 | NA | 6 | 127.299 | -242.599 | 1.443 | 0.175 |
| -0.00467 | NA | NA | + | NA | NA | 2.84E-03 | 6 | 127.07 | -242.14 | 1.902 | 0.139 |
| 0.00086 | 6.40E-11 | NA | + | NA | NA | NA | 6 | 127.06 | -242.12 | 1.922 | 0.138 |
| 0.00306 | NA | NA | + | NA | NA | NA | 5 | 120.044 | -230.088 | 0 | 0.287 |
| 0.01969 | NA | NA | + | 9.03E-05 | NA | NA | 6 | 120.669 | -229.338 | 0.75 | 0.198 |
| 0.04647 | NA | NA | NA | 1.57E-04 | NA | NA | 3 | 117.508 | -229.016 | 1.072 | 0.168 |
| 0.0184 | NA | NA | + | NA | -6.79E-03 | NA | 6 | 120.188 | -228.377 | 1.712 | 0.122 |
| 0.01296 | NA | NA | + | NA | NA | -4.12E-03 | 6 | 120.123 | -228.245 | 1.843 | 0.114 |
| 0.00149 | 9.50E-11 | NA | + | NA | NA | NA | 6 | 120.087 | -228.174 | 1.914 | 0.11 |
| 0.00232 | NA | NA | + | NA | NA | NA | 5 | 132.152 | -254.304 | 0 | 0.353 |
| 0.02533 | NA | NA | + | NA | -1.01E-02 | NA | 6 | 132.613 | -253.225 | 1.079 | 0.206 |
| 0.0118 | NA | NA | + | 5.53E-05 | NA | NA | 6 | 132.47 | -252.939 | 1.365 | 0.178 |
| 0.00142 | 5.34E-11 | NA | + | NA | NA | NA | 6 | 132.18 | -252.36 | 1.944 | 0.133 |
| 0.00424 | NA | NA | + | NA | NA | -8.25E-04 | 6 | 132.156 | -252.312 | 1.993 | 0.13 |
| -0.00069 | NA | NA | + | NA | NA | NA | 5 | 123.808 | -237.617 | 0 | 0.356 |
| 0.00991 | NA | NA | + | 6.16E-05 | NA | NA | 6 | 124.15 | -236.3 | 1.317 | 0.184 |
| 0.02026 | NA | NA | + | NA | -9.18E-03 | NA | 6 | 124.134 | -236.268 | 1.349 | 0.181 |
| -0.00302 | 1.42E-10 | NA | + | NA | NA | NA | 6 | 123.921 | -235.843 | 1.774 | 0.147 |
| 0.00048 | NA | NA | + | NA | NA | -4.93E-04 | 6 | 123.81 | -235.619 | 1.997 | 0.131 |
| 0.00391 | NA | NA | + | NA | NA | NA | 5 | 127.667 | -245.334 | 0 | 0.349 |
| 0.02687 | NA | NA | + | NA | -1.03E-02 | NA | 6 | 128.12 | -244.24 | 1.095 | 0.202 |
| 0.01314 | NA | NA | + | 5.64E-05 | NA | NA | 6 | 127.97 | -243.941 | 1.394 | 0.174 |
| 0.01468 | NA | NA | + | NA | NA | -4.46E-03 | 6 | 127.766 | -243.533 | 1.802 | 0.142 |
| 0.00244 | 8.61E-11 | NA | + | NA | NA | NA | 6 | 127.714 | -243.428 | 1.906 | 0.134 |
| 0.0033 | NA | NA | + | NA | NA | NA | 5 | 127.855 | -245.709 | 0 | 0.344 |
| 0.02862 | NA | NA | + | NA | -1.15E-02 | NA | 6 | 128.475 | -244.95 | 0.759 | 0.236 |
| 0.01114 | NA | NA | + | 4.87E-05 | NA | NA | 6 | 128.098 | -244.196 | 1.513 | 0.162 |
| 0.0023 | 5.93E-11 | NA | + | NA | NA | NA | 6 | 127.887 | -243.773 | 1.936 | 0.131 |
| 0.00038 | NA | NA | + | NA | NA | 1.22E-03 | 6 | 127.863 | -243.726 | 1.984 | 0.128 |
| 0.001 | NA | NA | + | NA | NA | NA | 5 | 121.251 | -232.502 | 0 | 0.35 |
| 0.02718 | NA | NA | + | NA | -1.14E-02 | NA | 6 | 121.783 | -231.567 | 0.936 | 0.219 |
| 0.01064 | NA | NA | + | 5.30E-05 | NA | NA | 6 | 121.507 | -231.014 | 1.489 | 0.166 |
| -0.00449 | NA | NA | + | NA | NA | 2.34E-03 | 6 | 121.284 | -230.567 | 1.935 | 0.133 |
| -0.00009 | 6.06E-11 | NA | + | NA | NA | NA | 6 | 121.275 | -230.551 | 1.951 | 0.132 |
| 0.00507 | NA | NA | + | NA | NA | NA | 5 | 125.133 | -240.265 | 0 | 0.361 |
| 0.01664 | NA | NA | + | 6.45E-05 | NA | NA | 6 | 125.504 | -239.007 | 1.258 | 0.192 |
| 0.02394 | NA | NA | + | NA | -8.29E-03 | NA | 6 | 125.394 | -238.787 | 1.478 | 0.172 |
| 0.01244 | NA | NA | + | NA | NA | -3.05E-03 | 6 | 125.179 | -238.358 | 1.907 | 0.139 |
| 0.00415 | 5.13E-11 | NA | + | NA | NA | NA | 6 | 125.151 | -238.302 | 1.963 | 0.135 |
| 0.00539 | NA | NA | + | NA | NA | NA | 5 | 127.747 | -245.493 | 0 | 0.341 |
| 0.02946 | NA | NA | + | NA | -1.06E-02 | NA | 6 | 128.242 | -244.485 | 1.008 | 0.206 |
| 0.01658 | NA | NA | + | 6.71E-05 | NA | NA | 6 | 128.183 | -244.365 | 1.128 | 0.194 |
| 0.00373 | 9.90E-11 | NA | + | NA | NA | NA | 6 | 127.818 | -243.635 | 1.858 | 0.135 |
| 0.00603 | NA | NA | + | NA | NA | -2.71E-04 | 6 | 127.747 | -243.494 | 1.999 | 0.125 |
| 0.00303 | NA | NA | + | NA | NA | NA | 5 | 123.289 | -236.578 | 0 | 0.364 |
| 0.02281 | NA | NA | + | NA | -8.89E-03 | NA | 6 | 123.657 | -235.315 | 1.264 | 0.194 |
| 0.01037 | NA | NA | + | 4.75E-05 | NA | NA | 6 | 123.511 | -235.023 | 1.555 | 0.167 |
| 0.00162 | 8.60E-11 | NA | + | NA | NA | NA | 6 | 123.331 | -234.662 | 1.916 | 0.14 |
| 0.00086 | NA | NA | + | NA | NA | 9.04E-04 | 6 | 123.294 | -234.587 | 1.991 | 0.135 |
| 0.00431 | NA | NA | + | NA | NA | NA | 5 | 121.052 | -232.105 | 0 | 0.338 |
| 0.01942 | NA | NA | + | 8.47E-05 | NA | NA | 6 | 121.689 | -231.378 | 0.726 | 0.235 |
| 0.02561 | NA | NA | + | NA | -9.29E-03 | NA | 6 | 121.388 | -230.776 | 1.328 | 0.174 |
| 0.00318 | 6.30E-11 | NA | + | NA | NA | NA | 6 | 121.08 | -230.161 | 1.944 | 0.128 |
| 0.00456 | NA | NA | + | NA | NA | -1.07E-04 | 6 | 121.052 | -230.105 | 2 | 0.124 |
| 0.00336 | NA | NA | + | NA | NA | NA | 5 | 129.424 | -248.849 | 0 | 0.334 |
| 0.02991 | NA | NA | + | NA | -1.18E-02 | NA | 6 | 130.223 | -248.447 | 0.402 | 0.273 |
| 0.00835 | NA | NA | + | 3.16E-05 | NA | NA | 6 | 129.539 | -247.078 | 1.771 | 0.138 |
| 0.00213 | 7.09E-11 | NA | + | NA | NA | NA | 6 | 129.466 | -246.932 | 1.917 | 0.128 |
| -0.00151 | NA | NA | + | NA | NA | 2.09E-03 | 6 | 129.45 | -246.899 | I.95 | 0.126 |
| 0.00075 | NA | NA | + | NA | NA | NA | 5 | 121.142 | -232.283 | 0 | 0.333 |
| 0.01601 | NA | NA | + | 8.63E-05 | NA | NA | 6 | 121.774 | -231.549 | 0.735 | 0.23 |
| 0.02257 | NA | NA | + | NA | -9.51E-03 | NA | 6 | 121.49 | -230.98 | 1.303 | 0.173 |
| -0.00128 | 1.15E-10 | NA | + | NA | NA | NA | 6 | 121.217 | -230.434 | 1.849 | 0.132 |
| 0.01003 | NA | NA | + | NA | NA | -3.88E-03 | 6 | 121.211 | -230.421 | 1.862 | 0.131 |
| 0.00448 | NA | NA | + | NA | NA | NA | 5 | 115.8 | -221.6 | 0 | 0.342 |
| 0.01991 | NA | NA | + | 9.08E-05 | NA | NA | 6 | 116.455 | -220.911 | 0.689 | 0.243 |
| 0.02176 | NA | NA | + | NA | -7.64E-03 | NA | 6 | 116.005 | -220.01 | I.59 | 0.155 |
| 0.00318 | 7.88E-11 | NA | + | NA | NA | NA | 6 | 115.843 | -219.686 | 1.914 | 0.132 |
| 0.0097 | NA | NA | + | NA | NA | -2.17E-03 | 6 | 115.822 | -219.643 | 1.957 | 0.129 |
| 0.00308 | NA | NA | + | NA | NA | NA | 5 | 119.599 | -229.198 | 0 | 0.295 |
| 0.01944 | NA | NA | + | 8.94E-05 | NA | NA | 6 | 120.202 | -228.404 | 0.794 | 0.199 |
| 0.04488 | NA | NA | NA | 1.55E-04 | NA | NA | 3 | 116.896 | -227.792 | 1.406 | 0.146 |
| 0.02219 | NA | NA | + | NA | -8.52E-03 | NA | 6 | 119.834 | -227.668 | I.53 | 0.137 |
| 0.00184 | 7.40E-11 | NA | + | NA | NA | NA | 6 | 119.637 | -227.275 | 1.923 | 0.113 |
| 0.00521 | NA | NA | + | NA | NA | -8.63E-04 | 6 | 119.603 | -227.206 | 1.992 | 0.109 |
| 0.00654 | NA | NA | + | NA | NA | NA | 5 | 130.266 | -250.532 | 0 | 0.363 |
| 0.01691 | NA | NA | + | 6.34E-05 | NA | NA | 6 | 130.642 | -249.285 | 1.247 | 0.194 |
| 0.02199 | NA | NA | + | NA | -6.93E-03 | NA | 6 | 130.463 | -248.926 | 1.606 | 0.162 |
| -0.00168 | NA | NA | + | NA | NA | 3.41E-03 | 6 | 130.334 | -248.668 | 1.864 | 0.143 |
| 0.00553 | 6.09E-11 | NA | + | NA | NA | NA | 6 | 130.296 | -248.592 | I.94 | 0.137 |
| 0.00179 | NA | NA | + | NA | NA | NA | 5 | 122.382 | -234.763 | 0 | 0.359 |
| 0.01195 | NA | NA | + | 6.19E-05 | NA | NA | 6 | 122.73 | -233.461 | 1.303 | 0.187 |
| 0.02168 | NA | NA | + | NA | -8.86E-03 | NA | 6 | 122.715 | -233.429 | 1.334 | 0.184 |
| 0.00065 | 6.53E-11 | NA | + | NA | NA | NA | 6 | 122.414 | -232.827 | 1.936 | 0.136 |
| 0.0038 | NA | NA | + | NA | NA | -8.31E-04 | 6 | 122.385 | -232.77 | 1.993 | 0.133 |
| 0.00184 | NA | NA | + | NA | NA | NA | 5 | 126.614 | -243.228 | 0 | 0.363 |
| 0.02599 | NA | NA | + | NA | -1.08E-02 | NA | 6 | 127.086 | -242.171 | 1.056 | 0.214 |
| 0.00804 | NA | NA | + | 3.69E-05 | NA | NA | 6 | 126.743 | -241.486 | 1.742 | 0.152 |
| 0.0008 | 5.88E-11 | NA | + | NA | NA | NA | 6 | 126.639 | -241.277 | 1.951 | 0.137 |
| 0.00086 | NA | NA | + | NA | NA | 4.12E-04 | 6 | 126.615 | -241.23 | 1.998 | 0.134 |
| 0.00027 | NA | NA | + | NA | NA | NA | 5 | 121.708 | -233.417 | 0 | 0.346 |
| 0.01371 | NA | NA | + | 7.31E-05 | NA | NA | 6 | 122.134 | -232.268 | 1.149 | 0.195 |
| 0.02324 | NA | NA | + | NA | -1.00E-02 | NA | 6 | 122.061 | -232.123 | 1.294 | 0.181 |
| 0.01184 | NA | NA | + | NA | NA | -4.89E-03 | 6 | 121.827 | -231.654 | 1.763 | 0.143 |
| -0.00138 | 9.09E-11 | NA | + | NA | NA | NA | 6 | 121.764 | -231.528 | 1.889 | 0.135 |
| 0.0052 | NA | NA | + | NA | NA | NA | 5 | 128.571 | -247.143 | 0 | 0.372 |
| 0.01545 | NA | NA | + | 5.87E-05 | NA | NA | 6 | 128.893 | -245.785 | 1.357 | 0.189 |
| 0.02009 | NA | NA | + | NA | -6.54E-03 | NA | 6 | 128.74 | -245.48 | 1.663 | 0.162 |
| 0.00446 | 4.30E-11 | NA | + | NA | NA | NA | 6 | 128.585 | -245.17 | 1.973 | 0.139 |
| 0.00717 | NA | NA | + | NA | NA | -8.31E-04 | 6 | 128.575 | -245.15 | 1.993 | 0.138 |
| 0.00758 | NA | NA | + | NA | NA | NA | 5 | 128.096 | -246.192 | 0 | 0.372 |
| 0.02537 | NA | NA | + | NA | -7.94E-03 | NA | 6 | 128.356 | -244.711 | I.48 | 0.178 |
| 0.01697 | NA | NA | + | 5.18E-05 | NA | NA | 6 | 128.338 | -244.676 | 1.516 | 0.174 |
| 0.00692 | 3.70E-11 | NA | + | NA | NA | NA | 6 | 128.107 | -244.214 | 1.978 | 0.138 |
| 0.00848 | NA | NA | + | NA | NA | -3.81E-04 | 6 | 128.097 | -244.193 | 1.998 | 0.137 |
| 0.00345 | NA | NA | + | NA | NA | NA | 5 | 123.579 | -237.158 | 0 | 0.334 |
| 0.03172 | NA | NA | + | NA | -1.27E-02 | NA | 6 | 124.321 | -236.643 | 0.516 | 0.258 |
| 0.01135 | NA | NA | + | 5.10E-05 | NA | NA | 6 | 123.84 | -235.681 | 1.478 | 0.159 |
| 0.00253 | 5.36E-11 | NA | + | NA | NA | NA | 6 | 123.61 | -235.219 | 1.939 | 0.126 |
| 0.00181 | NA | NA | + | NA | NA | 6.82E-04 | 6 | 123.582 | -235.163 | 1.995 | 0.123 |
| 0.00118 | NA | NA | + | NA | NA | NA | 5 | 118.392 | -226.785 | 0 | 0.37 |
| 0.02048 | NA | NA | + | NA | -8.63E-03 | NA | 6 | 118.66 | -225.32 | 1.465 | 0.178 |
| 0.01054 | NA | NA | + | 5.21E-05 | NA | NA | 6 | 118.619 | -225.238 | 1.547 | 0.171 |
| 0.00905 | NA | NA | + | NA | NA | -3.29E-03 | 6 | 118.445 | -224.891 | 1.894 | 0.144 |
| 0.00049 | 3.73E-11 | NA | + | NA | NA | NA | 6 | 118.403 | -224.806 | 1.979 | 0.138 |
| 0.05 | NA | NA | NA | 1.53E-04 | NA | NA | 3 | 124.02 | -242.041 | 0 | 0.219 |
| 0.00363 | NA | NA | + | NA | NA | NA | 5 | 125.951 | -241.903 | 0.138 | 0.204 |
| 0.02217 | NA | NA | + | 9.24E-05 | NA | NA | 6 | 126.657 | -241.314 | 0.726 | 0.152 |
| 0.06313 | NA | NA | NA | 1.62E-04 | NA | -4.72E-03 | 4 | 124.125 | -240.249 | 1.791 | 0.089 |
| 0.05983 | NA | NA | NA | 1.47E-04 | -5.06E-03 | NA | 4 | 124.11 | -240.22 | I.82 | 0.088 |
| 0.01368 | NA | NA | + | NA | NA | -4.33E-03 | 6 | 126.044 | -240.087 | 1.953 | 0.082 |
| 0.04908 | 5.78E-11 | NA | NA | 1.53E-04 | NA | NA | 4 | 124.042 | -240.084 | 1.956 | 0.082 |
| 0.01512 | NA | NA | + | NA | -4.95E-03 | NA | 6 | 126.036 | -240.071 | 1.969 | 0.082 |
| 0.00138 | NA | NA | + | NA | NA | NA | 5 | 120.152 | -230.304 | 0 | 0.362 |
| 0.01366 | NA | NA | + | 6.88E-05 | NA | NA | 6 | 120.545 | -229.089 | 1.215 | 0.197 |
| 0.01835 | NA | NA | + | NA | -7.48E-03 | NA | 6 | 120.354 | -228.707 | 1.597 | 0.163 |
| 0.00953 | NA | NA | + | NA | NA | -3.45E-03 | 6 | 120.211 | -228.423 | 1.882 | 0.141 |
| 0.00009 | 7.05E-11 | NA | + | NA | NA | NA | 6 | 120.184 | -228.367 | 1.937 | 0.137 |
| 0.00357 | NA | NA | + | NA | NA | NA | 5 | 119.915 | -229.829 | 0 | 0.347 |
| 0.02603 | NA | NA | + | NA | -9.87E-03 | NA | 6 | 120.362 | -228.725 | 1.104 | 0.2 |
| 0.01413 | NA | NA | + | 6.24E-05 | NA | NA | 6 | 120.279 | -228.557 | 1.272 | 0.184 |
| 0.00142 | 1.17E-10 | NA | + | NA | NA | NA | 6 | 119.986 | -227.971 | 1.858 | 0.137 |
| -0.00328 | NA | NA | + | NA | NA | 2.95E-03 | 6 | 119.961 | -227.921 | 1.908 | 0.134 |
| -0.00397 | NA | NA | + | NA | NA | NA | 5 | 109.537 | -209.075 | 0 | 0.362 |
| 0.0108 | NA | NA | + | 7.14E-05 | NA | NA | 6 | 109.875 | -207.749 | 1.325 | 0.186 |
| 0.01943 | NA | NA | + | NA | -9.89E-03 | NA | 6 | 109.843 | -207.687 | 1.388 | 0.181 |
| -0.00864 | NA | NA | + | NA | NA | 2.02E-03 | 6 | 109.562 | -207.124 | I.95 | 0.136 |
| -0.00506 | 5.31E-11 | NA | + | NA | NA | NA | 6 | 109.55 | -207.101 | 1.974 | 0.135 |
| 0.0014 | NA | NA | + | NA | NA | NA | 5 | 129.423 | -248.845 | 0 | 0.335 |
| 0.01475 | NA | NA | + | 7.65E-05 | NA | NA | 6 | 130.017 | -248.034 | 0.812 | 0.223 |
| 0.02343 | NA | NA | + | NA | -9.83E-03 | NA | 6 | 129.838 | -247.677 | 1.169 | 0.187 |
| -0.00015 | 9.48E-11 | NA | + | NA | NA | NA | 6 | 129.486 | -246.973 | 1.873 | 0.131 |
| 0.00162 | NA | NA | + | NA | NA | -8.75E-05 | 6 | 129.423 | -246.846 | 2 | 0.123 |
| 0.00012 | NA | NA | + | NA | NA | NA | 5 | 128.311 | -246.623 | 0 | 0.332 |
| 0.02836 | NA | NA | + | NA | -1.23E-02 | NA | 6 | 129.066 | -246.132 | 0.49 | 0.26 |
| 0.00871 | NA | NA | + | 5.06E-05 | NA | NA | 6 | 128.585 | -245.17 | 1.453 | 0.16 |
| -0.00074 | 5.00E-11 | NA | + | NA | NA | NA | 6 | 128.341 | -244.683 | I.94 | 0.126 |
| -0.00107 | NA | NA | + | NA | NA | 5.11E-04 | 6 | 128.313 | -244.626 | 1.997 | 0.122 |
| 0.00517 | NA | NA | + | NA | NA | NA | 5 | 124.125 | -238.25 | 0 | 0.357 |
| 0.02516 | NA | NA | + | NA | -9.03E-03 | NA | 6 | 124.509 | -237.019 | 1.231 | 0.193 |
| 0.01385 | NA | NA | + | 5.31E-05 | NA | NA | 6 | 124.399 | -236.799 | 1.451 | 0.173 |
| -0.00262 | NA | NA | + | NA | NA | 3.20E-03 | 6 | 124.184 | -236.367 | 1.883 | 0.139 |
| 0.00368 | 9.15E-11 | NA | + | NA | NA | NA | 6 | 124.18 | -236.36 | 1.889 | 0.139 |
| 0.00311 | NA | NA | + | NA | NA | NA | 5 | 131.625 | -253.251 | 0 | 0.361 |
| 0.01243 | NA | NA | + | 5.64E-05 | NA | NA | 6 | 131.967 | -251.934 | 1.317 | 0.187 |
| 0.02032 | NA | NA | + | NA | -7.55E-03 | NA | 6 | 131.901 | -251.802 | 1.449 | 0.175 |
| -0.00397 | NA | NA | + | NA | NA | 3.03E-03 | 6 | 131.68 | -251.36 | 1.891 | 0.14 |
| 0.00203 | 6.88E-11 | NA | + | NA | NA | NA | 6 | 131.66 | -251.32 | 1.931 | 0.137 |
| 0.00385 | NA | NA | + | NA | NA | NA | 5 | 123.625 | -237.25 | 0 | 0.353 |
| 0.01752 | NA | NA | + | 7.70E-05 | NA | NA | 6 | 124.159 | -236.317 | 0.932 | 0.222 |
| 0.01998 | NA | NA | + | NA | -7.05E-03 | NA | 6 | 123.819 | -235.637 | 1.613 | 0.158 |
| 0.00252 | 7.95E-11 | NA | + | NA | NA | NA | 6 | 123.67 | -235.34 | I.91 | 0.136 |
| 0.00024 | NA | NA | + | NA | NA | 1.53E-03 | 6 | 123.637 | -235.274 | 1.976 | 0.132 |
| 0.00206 | NA | NA | + | NA | NA | NA | 5 | 125.639 | -241.278 | 0 | 0.358 |
| 0.01296 | NA | NA | + | 6.38E-05 | NA | NA | 6 | 126.021 | -240.042 | 1.236 | 0.193 |
| 0.02039 | NA | NA | + | NA | -7.99E-03 | NA | 6 | 125.931 | -239.861 | 1.417 | 0.176 |
| 0.00063 | 8.40E-11 | NA | + | NA | NA | NA | 6 | 125.684 | -239.368 | I.91 | 0.138 |
| -0.00245 | NA | NA | + | NA | NA | 1.90E-03 | 6 | 125.659 | -239.318 | I.96 | 0.134 |
| 0.00274 | NA | NA | + | NA | NA | NA | 5 | 124.214 | -238.429 | 0 | 0.341 |
| 0.01801 | NA | NA | + | 8.28E-05 | NA | NA | 6 | 124.8 | -237.6 | 0.828 | 0.226 |
| 0.02247 | NA | NA | + | NA | -8.68E-03 | NA | 6 | 124.495 | -236.989 | I.44 | 0.166 |
| 0.0121 | NA | NA | + | NA | NA | -3.90E-03 | 6 | 124.289 | -236.578 | I.85 | 0.135 |
| 0.00129 | 8.40E-11 | NA | + | NA | NA | NA | 6 | 124.262 | -236.523 | 1.905 | 0.132 |
| 0.00609 | NA | NA | + | NA | NA | NA | 5 | 125.839 | -241.677 | 0 | 0.363 |
| 0.01541 | NA | NA | + | 5.63E-05 | NA | NA | 6 | 126.145 | -240.289 | 1.388 | 0.181 |
| 0.02087 | NA | NA | + | NA | -6.66E-03 | NA | 6 | 126.034 | -240.067 | I.61 | 0.162 |
| -0.00541 | NA | NA | + | NA | NA | 4.88E-03 | 6 | 125.977 | -239.954 | 1.723 | 0.153 |
| 0.00445 | 9.41E-11 | NA | + | NA | NA | NA | 6 | 125.891 | -239.783 | 1.894 | 0.141 |
| 0.05034 | NA | NA | NA | 1.44E-04 | NA | NA | 3 | 119.106 | -232.212 | 0 | 0.282 |
| 0.00811 | NA | NA | + | NA | NA | NA | 5 | 120.92 | -231.84 | 0.372 | 0.234 |
| 0.02594 | NA | NA | + | 9.06E-05 | NA | NA | 6 | 121.536 | -231.073 | 1.139 | 0.16 |
| 0.0615 | NA | NA | NA | 1.51E-04 | NA | -3.96E-03 | 4 | 119.174 | -230.349 | 1.863 | 0.111 |
| 0.04927 | 7.15E-11 | NA | NA | 1.44E-04 | NA | NA | 4 | 119.132 | -230.264 | 1.948 | 0.107 |
| 0.05513 | NA | NA | NA | 1.41E-04 | -2.51E-03 | NA | 4 | 119.126 | -230.253 | 1.959 | 0.106 |
| 0.00473 | NA | NA | + | NA | NA | NA | 5 | 124.785 | -239.57 | 0 | 0.298 |
| 0.01919 | NA | NA | + | 7.84E-05 | NA | NA | 6 | 125.274 | -238.549 | 1.022 | 0.179 |
| 0.04508 | NA | NA | NA | 1.39E-04 | NA | NA | 3 | 122.225 | -238.45 | 01.XII | 0.17 |
| 0.01958 | NA | NA | + | NA | -6.54E-03 | NA | 6 | 124.933 | -237.866 | 1.704 | 0.127 |
| 0.01215 | NA | NA | + | NA | NA | -3.08E-03 | 6 | 124.836 | -237.672 | 1.898 | 0.115 |
| 0.00385 | 4.84E-11 | NA | + | NA | NA | NA | 6 | 124.799 | -237.598 | 1.972 | 0.111 |
| 0.00136 | NA | NA | + | NA | NA | NA | 5 | 120.119 | -230.239 | 0 | 0.268 |
| 0.01775 | NA | NA | + | 9.24E-05 | NA | NA | 6 | 120.794 | -229.588 | 0.651 | 0.194 |
| 0.04749 | NA | NA | NA | 1.63E-04 | NA | NA | 3 | 117.731 | -229.462 | 0.777 | 0.182 |
| 0.02402 | NA | NA | + | NA | -9.89E-03 | NA | 6 | 120.478 | -228.956 | 1.283 | 0.141 |
| -0.00108 | 1.47E-10 | NA | + | NA | NA | NA | 6 | 120.217 | -228.435 | 1.804 | 0.109 |
| 0.0108 | NA | NA | + | NA | NA | -3.88E-03 | 6 | 120.188 | -228.377 | 1.862 | 0.106 |
| 0.0043 | NA | NA | + | NA | NA | NA | 5 | 125.024 | -240.048 | 0 | 0.34 |
| 0.02977 | NA | NA | + | NA | -1.14E-02 | NA | 6 | 125.504 | -239.008 | 01.IV | 0.202 |
| 0.016 | NA | NA | + | 6.26E-05 | NA | NA | 6 | 125.358 | -238.717 | 1.331 | 0.175 |
| 0.02024 | NA | NA | + | NA | NA | -6.71E-03 | 6 | 125.25 | -238.5 | 1.548 | 0.157 |
| 0.0034 | 5.07E-11 | NA | + | NA | NA | NA | 6 | 125.043 | -238.085 | 1.963 | 0.127 |
| 0.00372 | NA | NA | + | NA | NA | NA | 5 | 128.506 | -247.012 | 0 | 0.368 |
| 0.0129 | NA | NA | + | 5.47E-05 | NA | NA | 6 | 128.81 | -245.619 | 1.393 | 0.183 |
| 0.01941 | NA | NA | + | NA | -6.91E-03 | NA | 6 | 128.735 | -245.471 | 1.541 | 0.17 |
| 0.00256 | 6.91E-11 | NA | + | NA | NA | NA | 6 | 128.539 | -245.078 | 1.935 | 0.14 |
| -0.00052 | NA | NA | + | NA | NA | 1.83E-03 | 6 | 128.525 | -245.051 | 1.962 | 0.138 |
| 0.0027 | NA | NA | + | NA | NA | NA | 5 | 126.074 | -242.147 | 0 | 0.359 |
| 0.02374 | NA | NA | + | NA | -9.38E-03 | NA | 6 | 126.431 | -240.862 | 1.285 | 0.189 |
| 0.01305 | NA | NA | + | 5.95E-05 | NA | NA | 6 | 126.408 | -240.817 | I.33 | 0.185 |
| 0.00723 | NA | NA | + | NA | NA | -1.90E-03 | 6 | 126.093 | -240.187 | 1.961 | 0.135 |
| 0.00231 | 2.24E-11 | NA | + | NA | NA | NA | 6 | 126.08 | -240.16 | 1.987 | 0.133 |
| 0.00318 | NA | NA | + | NA | NA | NA | 5 | 120.481 | -230.962 | 0 | 0.287 |
| 0.02087 | NA | NA | + | 9.33E-05 | NA | NA | 6 | 121.171 | -230.342 | 0.62 | 0.211 |
| 0.04879 | NA | NA | NA | 1.54E-04 | NA | NA | 3 | 117.829 | -229.658 | 1.304 | 0.15 |
| 0.02335 | NA | NA | + | NA | -8.72E-03 | NA | 6 | 120.747 | -229.494 | 1.468 | 0.138 |
| 0.00226 | 5.35E-11 | NA | + | NA | NA | NA | 6 | 120.503 | -229.007 | 1.956 | 0.108 |
| 0.00693 | NA | NA | + | NA | NA | -1.58E-03 | 6 | 120.494 | -228.989 | 1.973 | 0.107 |
| 0.0074 | NA | NA | + | NA | NA | NA | 5 | 123.614 | -237.229 | 0 | 0.309 |
| 0.02043 | NA | NA | + | 7.40E-05 | NA | NA | 6 | 124.123 | -236.247 | 0.982 | 0.189 |
| 0.04543 | NA | NA | NA | 1.33E-04 | NA | NA | 3 | 120.841 | -235.682 | 1.546 | 0.142 |
| -0.00149 | NA | NA | + | NA | NA | 3.71E-03 | 6 | 123.69 | -235.38 | 1.849 | 0.122 |
| 0.01525 | NA | NA | + | NA | -3.55E-03 | NA | 6 | 123.669 | -235.337 | 1.892 | 0.12 |
| 0.00607 | 7.74E-11 | NA | + | NA | NA | NA | 6 | 123.65 | -235.301 | 1.928 | 0.118 |
| 0.00147 | NA | NA | + | NA | NA | NA | 5 | 123.556 | -237.112 | 0 | 0.352 |
| 0.02452 | NA | NA | + | NA | -1.01E-02 | NA | 6 | 124.051 | -236.102 | 01.I | 0.212 |
| 0.0094 | NA | NA | + | 4.94E-05 | NA | NA | 6 | 123.794 | -235.588 | 1.524 | 0.164 |
| -0.00083 | 1.31E-10 | NA | + | NA | NA | NA | 6 | 123.649 | -235.297 | 1.814 | 0.142 |
| 0.00054 | NA | NA | + | NA | NA | 3.93E-04 | 6 | 123.557 | -235.113 | 1.998 | 0.13 |
| 0.00112 | NA | NA | + | NA | NA | NA | 5 | 112.943 | -215.887 | 0 | 0.289 |
| 0.01772 | NA | NA | + | 8.96E-05 | NA | NA | 6 | 113.538 | -215.077 | 0.81 | 0.193 |
| 0.04693 | NA | NA | NA | 1.57E-04 | NA | NA | 3 | 110.477 | -214.955 | 0.932 | 0.181 |
| 0.01597 | NA | NA | + | NA | -6.55E-03 | NA | 6 | 113.077 | -214.154 | 1.732 | 0.121 |
| 0.00014 | 5.53E-11 | NA | + | NA | NA | NA | 6 | 112.967 | -213.935 | 1.952 | 0.109 |
| 0.00344 | NA | NA | + | NA | NA | -9.68E-04 | 6 | 112.948 | -213.895 | 1.991 | 0.107 |
| 0.01792 | NA | NA | + | 1.07E-04 | NA | NA | 6 | 117.684 | -223.369 | 0 | 0.228 |
| -0.00012 | NA | NA | + | NA | NA | NA | 5 | 116.666 | -223.332 | 0.037 | 0.224 |
| 0.01515 | NA | NA | + | NA | -6.65E-03 | NA | 6 | 116.847 | -221.695 | 1.674 | 0.099 |
| 0.02809 | NA | NA | + | 1.03E-04 | -4.74E-03 | NA | 7 | 117.776 | -221.552 | 1.817 | 0.092 |
| -0.00257 | 1.49E-10 | NA | + | NA | NA | NA | 6 | 116.773 | -221.546 | 1.823 | 0.092 |
| 0.01558 | 1.27E-10 | NA | + | 1.05E-04 | NA | NA | 7 | 117.764 | -221.528 | 1.841 | 0.091 |
| 0.04789 | NA | NA | NA | 1.71E-04 | NA | NA | 3 | 113.763 | -221.527 | 1.842 | 0.091 |
| 0.01931 | NA | NA | + | 1.07E-04 | NA | -5.42E-04 | 7 | 117.686 | -221.372 | 1.997 | 0.084 |
| 0.00185 | NA | NA | + | NA | NA | NA | 5 | 128.372 | -246.743 | 0 | 0.351 |
| 0.01249 | NA | NA | + | 6.37E-05 | NA | NA | 6 | 128.768 | -245.536 | 1.207 | 0.192 |
| 0.02349 | NA | NA | + | NA | -9.48E-03 | NA | 6 | 128.75 | -245.501 | 1.242 | 0.189 |
| 0.00847 | NA | NA | + | NA | NA | -2.76E-03 | 6 | 128.411 | -244.821 | 1.922 | 0.134 |
| 0.00075 | 6.81E-11 | NA | + | NA | NA | NA | 6 | 128.402 | -244.804 | 1.939 | 0.133 |
| 0.00061 | NA | NA | + | NA | NA | NA | 5 | 126.75 | -243.5 | 0 | 0.336 |
| 0.02676 | NA | NA | + | NA | -1.16E-02 | NA | 6 | 127.429 | -242.858 | 0.642 | 0.244 |
| 0.00755 | NA | NA | + | 4.58E-05 | NA | NA | 6 | 126.975 | -241.951 | I.55 | 0.155 |
| -0.00817 | NA | NA | + | NA | NA | 3.75E-03 | 6 | 126.829 | -241.658 | 1.842 | 0.134 |
| -0.00072 | 8.04E-11 | NA | + | NA | NA | NA | 6 | 126.805 | -241.611 | 1.889 | 0.131 |
| 0.0022 | NA | NA | + | NA | NA | NA | 5 | 118.203 | -226.406 | 0 | 0.369 |
| 0.01558 | NA | NA | + | 7.27E-05 | NA | NA | 6 | 118.619 | -225.239 | 1.167 | 0.206 |
| 0.01454 | NA | NA | + | NA | -5.46E-03 | NA | 6 | 118.305 | -224.61 | 1.795 | 0.15 |
| 0.00148 | 3.84E-11 | NA | + | NA | NA | NA | 6 | 118.215 | -224.43 | 1.976 | 0.137 |
| 0.00553 | NA | NA | + | NA | NA | -1.39E-03 | 6 | 118.213 | -224.426 | I.98 | 0.137 |
| 0.00404 | NA | NA | + | NA | NA | NA | 5 | 119.787 | -229.574 | 0 | 0.363 |
| 0.02784 | NA | NA | + | NA | -1.07E-02 | NA | 6 | 120.24 | -228.48 | 1.094 | 0.21 |
| 0.01123 | NA | NA | + | 4.29E-05 | NA | NA | 6 | 119.948 | -227.896 | 1.678 | 0.157 |
| 0.00275 | 7.14E-11 | NA | + | NA | NA | NA | 6 | 119.817 | -227.633 | 1.941 | 0.137 |
| 0.00343 | NA | NA | + | NA | NA | 2.53E-04 | 6 | 119.787 | -227.575 | 1.999 | 0.133 |
| 0.00302 | NA | NA | + | NA | NA | NA | 5 | 127.015 | -244.03 | 0 | 0.356 |
| 0.01238 | NA | NA | + | 5.88E-05 | NA | NA | 6 | 127.369 | -242.737 | 1.292 | 0.187 |
| 0.02098 | NA | NA | + | NA | -8.10E-03 | NA | 6 | 127.337 | -242.674 | 1.356 | 0.181 |
| 0.00115 | 1.12E-10 | NA | + | NA | NA | NA | 6 | 127.087 | -242.175 | 1.855 | 0.141 |
| -0.00331 | NA | NA | + | NA | NA | 2.63E-03 | 6 | 127.053 | -242.106 | 1.923 | 0.136 |
| 0.00045 | NA | NA | + | NA | NA | NA | 5 | 121.059 | -232.117 | 0 | 0.34 |
| 0.01568 | NA | NA | + | 8.41E-05 | NA | NA | 6 | 121.648 | -231.297 | 0.821 | 0.226 |
| 0.02116 | NA | NA | + | NA | -9.17E-03 | NA | 6 | 121.363 | -230.726 | 1.391 | 0.17 |
| -0.0014 | 1.04E-10 | NA | + | NA | NA | NA | 6 | 121.124 | -230.248 | I.87 | 0.134 |
| -0.00584 | NA | NA | + | NA | NA | 2.63E-03 | 6 | 121.098 | -230.195 | 1.922 | 0.13 |
| 0.00754 | NA | NA | + | NA | NA | NA | 5 | 125.468 | -240.937 | 0 | 0.297 |
| 0.02084 | NA | NA | + | 7.70E-05 | NA | NA | 6 | 125.966 | -239.932 | 1.005 | 0.18 |
| 0.04526 | NA | NA | NA | 1.39E-04 | NA | NA | 3 | 122.823 | -239.645 | 1.291 | 0.156 |
| 0.02527 | NA | NA | + | NA | -7.92E-03 | NA | 6 | 125.687 | -239.373 | 1.564 | 0.136 |
| 0.01833 | NA | NA | + | NA | NA | -4.45E-03 | 6 | 125.565 | -239.13 | 1.807 | 0.12 |
| 0.00714 | 2.40E-11 | NA | + | NA | NA | NA | 6 | 125.478 | -238.955 | 1.982 | 0.11 |
| -0.00107 | NA | NA | + | NA | NA | NA | 5 | 124.579 | -239.158 | 0 | 0.339 |
| 0.01308 | NA | NA | + | 8.10E-05 | NA | NA | 6 | 125.166 | -238.333 | 0.825 | 0.224 |
| 0.02053 | NA | NA | + | NA | -9.52E-03 | NA | 6 | 124.935 | -237.869 | 1.288 | 0.178 |
| -0.00277 | 1.01E-10 | NA | + | NA | NA | NA | 6 | 124.643 | -237.285 | 1.872 | 0.133 |
| 0.00169 | NA | NA | + | NA | NA | -1.14E-03 | 6 | 124.586 | -237.171 | 1.987 | 0.126 |
| 0.00567 | NA | NA | + | NA | NA | NA | 5 | 126.41 | -242.82 | 0 | 0.349 |
| 0.01846 | NA | NA | + | 7.63E-05 | NA | NA | 6 | 126.978 | -241.956 | 0.865 | 0.226 |
| 0.01924 | NA | NA | + | NA | -6.02E-03 | NA | 6 | 126.574 | -241.147 | 1.673 | 0.151 |
| -0.00378 | NA | NA | + | NA | NA | 3.98E-03 | 6 | 126.5 | -241 | 1.821 | 0.14 |
| 0.00433 | 7.90E-11 | NA | + | NA | NA | NA | 6 | 126.455 | -240.909 | 1.911 | 0.134 |
| 0.02594 | NA | NA | + | 1.18E-04 | NA | NA | 6 | 122.016 | -232.033 | 0 | 0.21 |
| 0.04941 | NA | NA | NA | 1.68E-04 | NA | NA | 3 | 118.863 | -231.727 | 0.306 | 0.18 |
| 0.00417 | NA | NA | + | NA | NA | NA | 5 | 120.863 | -231.726 | 0.307 | 0.18 |
| 0.04414 | NA | NA | + | 1.28E-04 | NA | -6.90E-03 | 7 | 122.237 | -230.474 | 1.558 | 0.096 |
| 0.04105 | NA | NA | + | 1.15E-04 | -6.89E-03 | NA | 7 | 122.184 | -230.368 | 1.665 | 0.091 |
| 0.02294 | NA | NA | + | NA | -8.22E-03 | NA | 6 | 121.099 | -230.199 | 1.834 | 0.084 |
| 0.02429 | 8.17E-11 | NA | + | 1.17E-04 | NA | NA | 7 | 122.058 | -230.117 | 1.916 | 0.08 |
| 0.06284 | NA | NA | NA | 1.59E-04 | -6.93E-03 | NA | 4 | 119.032 | -230.063 | I.97 | 0.078 |
| 0.00095 | NA | NA | + | NA | NA | NA | 5 | 124.515 | -239.03 | 0 | 0.342 |
| 0.02462 | NA | NA | + | NA | -1.03E-02 | NA | 6 | 125.019 | -238.038 | 0.992 | 0.209 |
| 0.01251 | NA | NA | + | 6.62E-05 | NA | NA | 6 | 124.937 | -237.875 | 1.155 | 0.192 |
| -0.00022 | 6.56E-11 | NA | + | NA | NA | NA | 6 | 124.548 | -237.095 | 1.934 | 0.13 |
| -0.00143 | NA | NA | + | NA | NA | 1.01E-03 | 6 | 124.521 | -237.041 | 1.988 | 0.127 |
| 0.00413 | NA | NA | + | NA | NA | NA | 5 | 123.06 | -236.12 | 0 | 0.356 |
| 0.01724 | NA | NA | + | 7.72E-05 | NA | NA | 6 | 123.591 | -235.181 | 0.939 | 0.223 |
| 0.01797 | NA | NA | + | NA | -6.05E-03 | NA | 6 | 123.21 | -234.42 | 01.VII | 0.152 |
| 0.00306 | 6.17E-11 | NA | + | NA | NA | NA | 6 | 123.088 | -234.175 | 1.945 | 0.135 |
| 0.00896 | NA | NA | + | NA | NA | -2.04E-03 | 6 | 123.081 | -234.162 | 1.958 | 0.134 |
| 0.00409 | NA | NA | + | NA | NA | NA | 5 | 122.031 | -234.063 | 0 | 0.376 |
| 0.01435 | NA | NA | + | 6.01E-05 | NA | NA | 6 | 122.35 | -232.7 | 1.363 | 0.19 |
| 0.01351 | NA | NA | + | NA | -4.26E-03 | NA | 6 | 122.102 | -232.204 | 1.859 | 0.148 |
| -0.00191 | NA | NA | + | NA | NA | 2.51E-03 | 6 | 122.066 | -232.133 | I.93 | 0.143 |
| 0.0028 | 7.41E-11 | NA | + | NA | NA | NA | 6 | 122.065 | -232.129 | 1.934 | 0.143 |
| 0.00473 | NA | NA | + | NA | NA | NA | 5 | 121.072 | -232.144 | 0 | 0.304 |
| 0.01798 | NA | NA | + | 7.52E-05 | NA | NA | 6 | 121.545 | -231.091 | 1.054 | 0.18 |
| 0.04599 | NA | NA | NA | 1.39E-04 | NA | NA | 3 | 118.343 | -230.685 | 1.459 | 0.147 |
| 0.0219 | NA | NA | + | NA | -7.57E-03 | NA | 6 | 121.292 | -230.583 | 1.561 | 0.139 |
| 0.00343 | 7.36E-11 | NA | + | NA | NA | NA | 6 | 121.119 | -230.239 | 1.905 | 0.117 |
| 0.00355 | NA | NA | + | NA | NA | 4.97E-04 | 6 | 121.073 | -230.147 | 1.998 | 0.112 |
| 0.00157 | NA | NA | + | NA | NA | NA | 5 | 124.025 | -238.05 | 0 | 0.347 |
| 0.01497 | NA | NA | + | 7.29E-05 | NA | NA | 6 | 124.489 | -236.978 | 1.072 | 0.203 |
| 0.02348 | NA | NA | + | NA | -9.59E-03 | NA | 6 | 124.381 | -236.762 | 1.288 | 0.183 |
| 0.00961 | NA | NA | + | NA | NA | -3.44E-03 | 6 | 124.088 | -236.175 | 1.875 | 0.136 |
| 0.00072 | 4.89E-11 | NA | + | NA | NA | NA | 6 | 124.047 | -236.094 | 1.956 | 0.131 |
| 0.00301 | NA | NA | + | NA | NA | NA | 5 | 127.086 | -244.171 | 0 | 0.34 |
| 0.02921 | NA | NA | + | NA | -1.17E-02 | NA | 6 | 127.735 | -243.471 | 0.7 | 0.24 |
| 0.01077 | NA | NA | + | 4.87E-05 | NA | NA | 6 | 127.329 | -242.658 | 1.513 | 0.16 |
| -0.00399 | NA | NA | + | NA | NA | 2.93E-03 | 6 | 127.135 | -242.271 | 01.IX | 0.131 |
| 0.00189 | 6.28E-11 | NA | + | NA | NA | NA | 6 | 127.12 | -242.24 | 1.931 | 0.129 |
| 0.00326 | NA | NA | + | NA | NA | NA | 5 | 125.894 | -241.787 | 0 | 0.376 |
| 0.01193 | NA | NA | + | 5.11E-05 | NA | NA | 6 | 126.127 | -240.254 | 1.533 | 0.175 |
| 0.01811 | NA | NA | + | NA | -6.53E-03 | NA | 6 | 126.07 | -240.139 | 1.648 | 0.165 |
| 0.00186 | 8.07E-11 | NA | + | NA | NA | NA | 6 | 125.934 | -239.868 | 1.919 | 0.144 |
| 0.00602 | NA | NA | + | NA | NA | -1.19E-03 | 6 | 125.901 | -239.802 | 1.986 | 0.139 |
| 0.00014 | NA | NA | + | NA | NA | NA | 5 | 122.032 | -234.064 | 0 | 0.372 |
| 0.01997 | NA | NA | + | NA | -8.70E-03 | NA | 6 | 122.326 | -232.653 | 1.411 | 0.184 |
| 0.00726 | NA | NA | + | 4.22E-05 | NA | NA | 6 | 122.194 | -232.388 | 1.676 | 0.161 |
| -0.00134 | 8.38E-11 | NA | + | NA | NA | NA | 6 | 122.065 | -232.13 | 1.934 | 0.142 |
| 0.00558 | NA | NA | + | NA | NA | -2.33E-03 | 6 | 122.06 | -232.12 | 1.944 | 0.141 |
| 0.00247 | NA | NA | + | NA | NA | NA | 5 | 119.249 | -228.498 | 0 | 0.351 |
| 0.0144 | NA | NA | + | 6.59E-05 | NA | NA | 6 | 119.615 | -227.229 | 1.269 | 0.186 |
| 0.02468 | NA | NA | + | NA | -9.78E-03 | NA | 6 | 119.583 | -227.167 | 1.331 | 0.18 |
| 0.01576 | NA | NA | + | NA | NA | -5.65E-03 | 6 | 119.393 | -226.786 | 1.711 | 0.149 |
| 0.00137 | 6.49E-11 | NA | + | NA | NA | NA | 6 | 119.278 | -226.555 | 1.943 | 0.133 |
| 0.00412 | NA | NA | + | NA | NA | NA | 5 | 129.767 | -249.535 | 0 | 0.369 |
| 0.02267 | NA | NA | + | NA | -8.10E-03 | NA | 6 | 130.066 | -248.131 | 1.403 | 0.183 |
| 0.01276 | NA | NA | + | 5.03E-05 | NA | NA | 6 | 130.012 | -248.023 | 1.511 | 0.173 |
| 0.00303 | 6.25E-11 | NA | + | NA | NA | NA | 6 | 129.794 | -247.588 | 1.946 | 0.139 |
| 0.00328 | NA | NA | + | NA | NA | 3.61E-04 | 6 | 129.768 | -247.536 | 1.999 | 0.136 |
| 0.00394 | NA | NA | + | NA | NA | NA | 5 | 124.474 | -238.948 | 0 | 0.318 |
| 0.01688 | NA | NA | + | 7.54E-05 | NA | NA | 6 | 124.972 | -237.945 | 1.003 | 0.193 |
| 0.01453 | NA | NA | + | NA | -4.61E-03 | NA | 6 | 124.555 | -237.11 | 1.838 | 0.127 |
| 0.04866 | NA | NA | NA | 1.39E-04 | NA | NA | 3 | 121.521 | -237.041 | 1.907 | 0.123 |
| 0.00281 | 6.66E-11 | NA | + | NA | NA | NA | 6 | 124.5 | -236.999 | 1.949 | 0.12 |
| 0.00772 | NA | NA | + | NA | NA | -1.62E-03 | 6 | 124.487 | -236.974 | 1.975 | 0.119 |
| 0.00145 | NA | NA | + | NA | NA | NA | 5 | 128.057 | -246.114 | 0 | 0.334 |
| 0.02847 | NA | NA | + | NA | -1.18E-02 | NA | 6 | 128.629 | -245.258 | 0.856 | 0.218 |
| 0.01401 | NA | NA | + | 7.09E-05 | NA | NA | 6 | 128.507 | -245.014 | 01.I | 0.193 |
| 0.00762 | NA | NA | + | NA | NA | -2.59E-03 | 6 | 128.094 | -244.188 | 1.926 | 0.128 |
| 0.00048 | 5.70E-11 | NA | + | NA | NA | NA | 6 | 128.088 | -244.176 | 1.938 | 0.127 |
| -0.00087 | NA | NA | + | NA | NA | NA | 5 | 124.211 | -238.422 | 0 | 0.348 |
| 0.01206 | NA | NA | + | 7.29E-05 | NA | NA | 6 | 124.687 | -237.374 | 1.048 | 0.206 |
| 0.02021 | NA | NA | + | NA | -9.21E-03 | NA | 6 | 124.536 | -237.071 | 1.351 | 0.177 |
| 0.00733 | NA | NA | + | NA | NA | -3.47E-03 | 6 | 124.269 | -236.538 | 1.884 | 0.136 |
| -0.00182 | 5.94E-11 | NA | + | NA | NA | NA | 6 | 124.242 | -236.483 | 1.939 | 0.132 |
| 0.0009 | NA | NA | + | NA | NA | NA | 5 | 126.949 | -243.899 | 0 | 0.345 |
| 0.01429 | NA | NA | + | 7.68E-05 | NA | NA | 6 | 127.518 | -243.036 | 0.863 | 0.224 |
| 0.01939 | NA | NA | + | NA | -8.02E-03 | NA | 6 | 127.211 | -242.422 | 1.477 | 0.165 |
| -0.00093 | 1.06E-10 | NA | + | NA | NA | NA | 6 | 127.02 | -242.041 | 1.858 | 0.136 |
| 0.0044 | NA | NA | + | NA | NA | -1.50E-03 | 6 | 126.96 | -241.921 | 1.978 | 0.129 |
| 0.00079 | NA | NA | + | NA | NA | NA | 5 | 124.11 | -238.22 | 0 | 0.339 |
| 0.01753 | NA | NA | + | 9.20E-05 | NA | NA | 6 | 124.842 | -237.683 | 0.537 | 0.259 |
| 0.015 | NA | NA | + | NA | -6.22E-03 | NA | 6 | 124.256 | -236.511 | 1.709 | 0.144 |
| -0.0008 | 9.17E-11 | NA | + | NA | NA | NA | 6 | 124.172 | -236.345 | 1.876 | 0.133 |
| 0.00051 | NA | NA | + | NA | NA | 1.19E-04 | 6 | 124.11 | -236.221 | 2 | 0.125 |
| 0.00711 | NA | NA | + | NA | NA | NA | 5 | 126.819 | -243.638 | 0 | 0.309 |
| 0.01986 | NA | NA | + | 7.52E-05 | NA | NA | 6 | 127.307 | -242.613 | 1.025 | 0.185 |
| 0.04808 | NA | NA | NA | 1.41E-04 | NA | NA | 3 | 124.077 | -242.153 | 1.485 | 0.147 |
| 0.02012 | NA | NA | + | NA | -5.74E-03 | NA | 6 | 126.941 | -241.882 | 1.756 | 0.128 |
| 0.00593 | 7.40E-11 | NA | + | NA | NA | NA | 6 | 126.848 | -241.697 | 1.941 | 0.117 |
| 0.01017 | NA | NA | + | NA | NA | -1.26E-03 | 6 | 126.827 | -241.654 | 1.984 | 0.114 |
| 0.00073 | NA | NA | + | NA | NA | NA | 5 | 119.365 | -228.73 | 0 | 0.363 |
| 0.01334 | NA | NA | + | 6.90E-05 | NA | NA | 6 | 119.759 | -227.518 | 1.212 | 0.198 |
| 0.0184 | NA | NA | + | NA | -7.74E-03 | NA | 6 | 119.579 | -227.157 | 1.572 | 0.165 |
| -0.00026 | 5.95E-11 | NA | + | NA | NA | NA | 6 | 119.396 | -226.792 | 1.938 | 0.138 |
| 0.00531 | NA | NA | + | NA | NA | -1.92E-03 | 6 | 119.383 | -226.766 | 1.964 | 0.136 |
| 0.0063 | NA | NA | + | NA | NA | NA | 5 | 128.918 | -247.836 | 0 | 0.353 |
| 0.01678 | NA | NA | + | 6.53E-05 | NA | NA | 6 | 129.359 | -246.717 | 1.119 | 0.202 |
| 0.0239 | NA | NA | + | NA | -7.86E-03 | NA | 6 | 129.229 | -246.457 | 1.379 | 0.177 |
| 0.00015 | NA | NA | + | NA | NA | 2.58E-03 | 6 | 128.957 | -245.913 | 1.923 | 0.135 |
| 0.00532 | 5.79E-11 | NA | + | NA | NA | NA | 6 | 128.951 | -245.902 | 1.934 | 0.134 |
| 0.00113 | NA | NA | + | NA | NA | NA | 5 | 127.804 | -245.607 | 0 | 0.336 |
| 0.02562 | NA | NA | + | NA | -1.09E-02 | NA | 6 | 128.467 | -244.934 | 0.673 | 0.24 |
| 0.00858 | NA | NA | + | 4.96E-05 | NA | NA | 6 | 128.078 | -244.157 | 1.451 | 0.163 |
| -0.00071 | 1.16E-10 | NA | + | NA | NA | NA | 6 | 127.887 | -243.775 | 1.833 | 0.134 |
| -0.00491 | NA | NA | + | NA | NA | 2.52E-03 | 6 | 127.838 | -243.676 | 1.931 | 0.128 |
| 0.0016 | NA | NA | + | NA | NA | NA | 5 | 128.162 | -246.324 | 0 | 0.331 |
| 0.02824 | NA | NA | + | NA | -1.17E-02 | NA | 6 | 128.694 | -245.389 | 0.935 | 0.208 |
| 0.01492 | NA | NA | + | 7.22E-05 | NA | NA | 6 | 128.633 | -245.267 | 1.057 | 0.195 |
| 0.01305 | NA | NA | + | NA | NA | -4.74E-03 | 6 | 128.278 | -244.556 | 1.768 | 0.137 |
| -0.00018 | 1.08E-10 | NA | + | NA | NA | NA | 6 | 128.221 | -244.441 | 1.882 | 0.129 |
| 0.00228 | NA | NA | + | NA | NA | NA | 5 | 125.195 | -240.39 | 0 | 0.352 |
| 0.0136 | NA | NA | + | 6.67E-05 | NA | NA | 6 | 125.611 | -239.222 | 1.168 | 0.196 |
| 0.02208 | NA | NA | + | NA | -8.85E-03 | NA | 6 | 125.516 | -239.033 | 1.357 | 0.179 |
| 0.00041 | 1.09E-10 | NA | + | NA | NA | NA | 6 | 125.267 | -238.534 | 1.856 | 0.139 |
| -0.00407 | NA | NA | + | NA | NA | 2.63E-03 | 6 | 125.232 | -238.464 | 1.926 | 0.134 |
| -0.00038 | NA | NA | + | NA | NA | NA | 5 | 125.449 | -240.899 | 0 | 0.356 |
| 0.02492 | NA | NA | + | NA | -1.11E-02 | NA | 6 | 125.91 | -239.82 | 1.079 | 0.208 |
| 0.00894 | NA | NA | + | 5.02E-05 | NA | NA | 6 | 125.667 | -239.333 | 1.566 | 0.163 |
| 0.00708 | NA | NA | + | NA | NA | -3.23E-03 | 6 | 125.505 | -239.011 | 1.888 | 0.138 |
| -0.00169 | 7.31E-11 | NA | + | NA | NA | NA | 6 | 125.482 | -238.964 | 1.935 | 0.135 |
| 0.00359 | NA | NA | + | NA | NA | NA | 5 | 128.298 | -246.596 | 0 | 0.365 |
| 0.01342 | NA | NA | + | 5.87E-05 | NA | NA | 6 | 128.638 | -245.277 | 1.319 | 0.189 |
| 0.02019 | NA | NA | + | NA | -7.34E-03 | NA | 6 | 128.548 | -245.096 | 01.V | 0.172 |
| 0.00239 | 6.91E-11 | NA | + | NA | NA | NA | 6 | 128.328 | -244.655 | 1.941 | 0.138 |
| 0.00058 | NA | NA | + | NA | NA | 1.29E-03 | 6 | 128.307 | -244.614 | 1.982 | 0.135 |
| 0.00299 | NA | NA | + | NA | NA | NA | 5 | 122.86 | -235.719 | 0 | 0.355 |
| 0.01533 | NA | NA | + | 7.18E-05 | NA | NA | 6 | 123.305 | -234.609 | 01.XI | 0.204 |
| 0.02201 | NA | NA | + | NA | -8.63E-03 | NA | 6 | 123.152 | -234.305 | 1.414 | 0.175 |
| 0.0018 | 6.83E-11 | NA | + | NA | NA | NA | 6 | 122.89 | -233.78 | 1.939 | 0.135 |
| 0.00541 | NA | NA | + | NA | NA | -9.95E-04 | 6 | 122.865 | -233.729 | I.99 | 0.131 |
| 0.00148 | NA | NA | + | NA | NA | NA | 5 | 124.334 | -238.667 | 0 | 0.339 |
| 0.02791 | NA | NA | + | NA | -1.17E-02 | NA | 6 | 124.938 | -237.875 | 0.792 | 0.229 |
| 0.01122 | NA | NA | + | 5.99E-05 | NA | NA | 6 | 124.678 | -237.357 | I.31 | 0.176 |
| -0.00009 | 8.99E-11 | NA | + | NA | NA | NA | 6 | 124.379 | -236.759 | 1.908 | 0.131 |
| 0.0019 | NA | NA | + | NA | NA | -1.75E-04 | 6 | 124.334 | -236.667 | 2 | 0.125 |
| 0.00413 | NA | NA | + | NA | NA | NA | 5 | 125.915 | -241.831 | 0 | 0.322 |
| 0.03037 | NA | NA | + | NA | -1.16E-02 | NA | 6 | 126.712 | -241.423 | 0.407 | 0.263 |
| 0.01071 | NA | NA | + | 4.43E-05 | NA | NA | 6 | 126.132 | -240.265 | 1.566 | 0.147 |
| -0.00811 | NA | NA | + | NA | NA | 5.20E-03 | 6 | 126.071 | -240.142 | 1.689 | 0.139 |
| 0.00226 | 1.09E-10 | NA | + | NA | NA | NA | 6 | 125.996 | -239.993 | 1.838 | 0.129 |
| 0.00366 | NA | NA | + | NA | NA | NA | 5 | 125.345 | -240.691 | 0 | 0.35 |
| 0.02533 | NA | NA | + | NA | -9.48E-03 | NA | 6 | 125.77 | -239.54 | I.15 | 0.197 |
| 0.01246 | NA | NA | + | 5.69E-05 | NA | NA | 6 | 125.668 | -239.336 | 1.355 | 0.178 |
| -0.00555 | NA | NA | + | NA | NA | 3.89E-03 | 6 | 125.426 | -238.852 | 1.839 | 0.14 |
| 0.00205 | 9.53E-11 | NA | + | NA | NA | NA | 6 | 125.396 | -238.793 | 1.898 | 0.136 |
| 0.00402 | NA | NA | + | NA | NA | NA | 5 | 114.911 | -219.821 | 0 | 0.364 |
| 0.0158 | NA | NA | + | 6.98E-05 | NA | NA | 6 | 115.293 | -218.586 | 1.235 | 0.196 |
| 0.02068 | NA | NA | + | NA | -7.38E-03 | NA | 6 | 115.096 | -218.193 | 1.629 | 0.161 |
| 0.01183 | NA | NA | + | NA | NA | -3.28E-03 | 6 | 114.961 | -217.922 | 01.IX | 0.141 |
| 0.00281 | 6.94E-11 | NA | + | NA | NA | NA | 6 | 114.94 | -217.881 | 1.941 | 0.138 |
| 0.00384 | NA | NA | + | NA | NA | NA | 5 | 128.101 | -246.203 | 0 | 0.252 |
| 0.02024 | NA | NA | + | 8.48E-05 | NA | NA | 6 | 128.755 | -245.509 | 0.693 | 0.178 |
| 0.02899 | NA | NA | + | NA | -1.11E-02 | NA | 6 | 128.592 | -245.185 | 1.018 | 0.152 |
| 0.04867 | NA | NA | NA | 1.49E-04 | NA | NA | 3 | 125.364 | -244.728 | 1.475 | 0.121 |
| 0.00208 | 1.02E-10 | NA | + | NA | NA | NA | 6 | 128.178 | -244.355 | 1.847 | 0.1 |
| 0.01279 | NA | NA | + | NA | NA | -3.79E-03 | 6 | 128.175 | -244.349 | 1.854 | 0.1 |
| 0.04164 | NA | NA | + | 7.86E-05 | -9.97E-03 | NA | 7 | 129.151 | -244.302 | 1.901 | 0.097 |
| 0.00738 | NA | NA | + | NA | NA | NA | 5 | 124.49 | -238.98 | 0 | 0.336 |
| 0.035 | NA | NA | + | NA | -1.23E-02 | NA | 6 | 125.256 | -238.513 | 0.467 | 0.266 |
| 0.01207 | NA | NA | + | 3.13E-05 | NA | NA | 6 | 124.594 | -237.189 | 1.791 | 0.137 |
| -0.00108 | NA | NA | + | NA | NA | 3.54E-03 | 6 | 124.564 | -237.128 | 1.852 | 0.133 |
| 0.00642 | 5.72E-11 | NA | + | NA | NA | NA | 6 | 124.516 | -237.031 | 1.949 | 0.127 |
| 0.00365 | NA | NA | + | NA | NA | NA | 5 | 124.549 | -239.098 | 0 | 0.351 |
| 0.02674 | NA | NA | + | NA | -1.03E-02 | NA | 6 | 124.976 | -237.951 | 1.147 | 0.198 |
| 0.01493 | NA | NA | + | 6.33E-05 | NA | NA | 6 | 124.903 | -237.806 | 1.292 | 0.184 |
| 0.00165 | 1.14E-10 | NA | + | NA | NA | NA | 6 | 124.618 | -237.237 | 1.861 | 0.138 |
| 0.00626 | NA | NA | + | NA | NA | -1.08E-03 | 6 | 124.555 | -237.109 | 1.989 | 0.13 |
| -0.00009 | NA | NA | + | NA | NA | NA | 5 | 125.684 | -241.368 | 0 | 0.332 |
| 0.01372 | NA | NA | + | 7.67E-05 | NA | NA | 6 | 126.215 | -240.43 | 0.938 | 0.207 |
| 0.02409 | NA | NA | + | NA | -1.07E-02 | NA | 6 | 126.175 | -240.35 | 1.018 | 0.199 |
| -0.00265 | 1.51E-10 | NA | + | NA | NA | NA | 6 | 125.794 | -239.589 | 1.779 | 0.136 |
| 0.00557 | NA | NA | + | NA | NA | -2.39E-03 | 6 | 125.713 | -239.425 | 1.943 | 0.126 |
| 0.00323 | NA | NA | + | NA | NA | NA | 5 | 123.309 | -236.618 | 0 | 0.365 |
| 0.01264 | NA | NA | + | 5.58E-05 | NA | NA | 6 | 123.599 | -235.199 | 1.419 | 0.179 |
| 0.0214 | NA | NA | + | NA | -8.18E-03 | NA | 6 | 123.591 | -235.183 | 1.435 | 0.178 |
| 0.00172 | 8.88E-11 | NA | + | NA | NA | NA | 6 | 123.361 | -234.721 | 1.897 | 0.141 |
| -0.00137 | NA | NA | + | NA | NA | 1.91E-03 | 6 | 123.329 | -234.657 | 1.961 | 0.137 |
| 0.00212 | NA | NA | + | NA | NA | NA | 5 | 119.281 | -228.563 | 0 | 0.368 |
| 0.01346 | NA | NA | + | 6.58E-05 | NA | NA | 6 | 119.643 | -227.287 | 1.276 | 0.195 |
| 0.01681 | NA | NA | + | NA | -6.42E-03 | NA | 6 | 119.439 | -226.878 | 1.684 | 0.159 |
| 0.00058 | 8.88E-11 | NA | + | NA | NA | NA | 6 | 119.329 | -226.659 | 1.904 | 0.142 |
| 0.00011 | NA | NA | + | NA | NA | 8.56E-04 | 6 | 119.285 | -226.57 | 1.992 | 0.136 |
| 0.00147 | NA | NA | + | NA | NA | NA | 5 | 118.152 | -226.304 | 0 | 0.37 |
| 0.01258 | NA | NA | + | 6.08E-05 | NA | NA | 6 | 118.45 | -224.9 | 1.405 | 0.183 |
| 0.01884 | NA | NA | + | NA | -7.70E-03 | NA | 6 | 118.371 | -224.742 | 1.562 | 0.169 |
| -0.00012 | 9.09E-11 | NA | + | NA | NA | NA | 6 | 118.19 | -224.38 | 1.924 | 0.141 |
| 0.00069 | NA | NA | + | NA | NA | 3.24E-04 | 6 | 118.153 | -224.306 | 1.999 | 0.136 |
| 0.00433 | NA | NA | + | NA | NA | NA | 5 | 124.032 | -238.063 | 0 | 0.286 |
| 0.02036 | NA | NA | + | 8.43E-05 | NA | NA | 6 | 124.593 | -237.186 | 0.877 | 0.184 |
| 0.04548 | NA | NA | NA | 1.56E-04 | NA | NA | 3 | 121.381 | -236.762 | 1.301 | 0.149 |
| 0.02467 | NA | NA | + | NA | -9.19E-03 | NA | 6 | 124.325 | -236.651 | 1.413 | 0.141 |
| 0.0195 | NA | NA | + | NA | NA | -6.22E-03 | 6 | 124.213 | -236.426 | 1.638 | 0.126 |
| 0.00241 | 1.20E-10 | NA | + | NA | NA | NA | 6 | 124.103 | -236.206 | 1.857 | 0.113 |
| 0.00026 | NA | NA | + | NA | NA | NA | 5 | 124.33 | -238.659 | 0 | 0.367 |
| 0.01178 | NA | NA | + | 6.27E-05 | NA | NA | 6 | 124.683 | -237.367 | 1.293 | 0.193 |
| 0.01656 | NA | NA | + | NA | -7.15E-03 | NA | 6 | 124.532 | -237.063 | 1.596 | 0.165 |
| -0.00082 | 5.92E-11 | NA | + | NA | NA | NA | 6 | 124.359 | -236.718 | 1.941 | 0.139 |
| 0.00068 | NA | NA | + | NA | NA | -1.80E-04 | 6 | 124.33 | -236.659 | 2 | 0.135 |
| 0.00099 | NA | NA | + | NA | NA | NA | 5 | 124.994 | -239.988 | 0 | 0.327 |
| 0.01668 | NA | NA | + | 8.97E-05 | NA | NA | 6 | 125.73 | -239.459 | 0.529 | 0.251 |
| 0.02129 | NA | NA | + | NA | -8.77E-03 | NA | 6 | 125.325 | -238.65 | 1.338 | 0.167 |
| -0.00108 | 1.20E-10 | NA | + | NA | NA | NA | 6 | 125.076 | -238.153 | 1.836 | 0.131 |
| 0.0071 | NA | NA | + | NA | NA | -2.59E-03 | 6 | 125.027 | -238.054 | 1.935 | 0.124 |
| 0.00187 | NA | NA | + | NA | NA | NA | 5 | 123.914 | -237.828 | 0 | 0.345 |
| 0.02806 | NA | NA | + | NA | -1.12E-02 | NA | 6 | 124.396 | -236.792 | 1.037 | 0.205 |
| 0.01457 | NA | NA | + | 6.91E-05 | NA | NA | 6 | 124.328 | -236.657 | 1.172 | 0.192 |
| 0.00082 | 5.90E-11 | NA | + | NA | NA | NA | 6 | 123.941 | -235.883 | 1.946 | 0.13 |
| 0.00427 | NA | NA | + | NA | NA | -1.02E-03 | 6 | 123.92 | -235.84 | 1.989 | 0.128 |
| 0.00007 | NA | NA | + | NA | NA | NA | 5 | 124.897 | -239.794 | 0 | 0.354 |
| 0.02252 | NA | NA | + | NA | -9.94E-03 | NA | 6 | 125.323 | -238.647 | 1.147 | 0.199 |
| 0.00976 | NA | NA | + | 5.82E-05 | NA | NA | 6 | 125.218 | -238.436 | 1.358 | 0.18 |
| -0.00506 | NA | NA | + | NA | NA | 2.14E-03 | 6 | 124.923 | -237.846 | 1.948 | 0.134 |
| -0.00089 | 5.67E-11 | NA | + | NA | NA | NA | 6 | 124.921 | -237.841 | 1.953 | 0.133 |
| 0.00104 | NA | NA | + | NA | NA | NA | 5 | 130.503 | -251.006 | 0 | 0.283 |
| 0.02914 | NA | NA | + | NA | -1.24E-02 | NA | 6 | 131.285 | -250.569 | 0.437 | 0.228 |
| 0.0104 | NA | NA | + | 6.16E-05 | NA | NA | 6 | 130.937 | -249.875 | 1.131 | 0.161 |
| 0.03424 | NA | NA | + | 5.04E-05 | -1.12E-02 | NA | 7 | 131.572 | -249.144 | 1.862 | 0.112 |
| -0.00022 | 7.73E-11 | NA | + | NA | NA | NA | 6 | 130.558 | -249.116 | I.89 | 0.11 |
| -0.00404 | NA | NA | + | NA | NA | 2.15E-03 | 6 | 130.531 | -249.061 | 1.945 | 0.107 |
| 0.00674 | NA | NA | + | NA | NA | NA | 5 | 126.164 | -242.328 | 0 | 0.286 |
| 0.04621 | NA | NA | NA | 1.30E-04 | NA | NA | 3 | 123.907 | -241.814 | 0.514 | 0.222 |
| 0.02044 | NA | NA | + | 7.36E-05 | NA | NA | 6 | 126.617 | -241.233 | 1.095 | 0.166 |
| 0.01552 | NA | NA | + | NA | -3.84E-03 | NA | 6 | 126.216 | -240.433 | 1.895 | 0.111 |
| 0.01169 | NA | NA | + | NA | NA | -2.08E-03 | 6 | 126.185 | -240.37 | 1.958 | 0.108 |
| 0.0057 | 6.09E-11 | NA | + | NA | NA | NA | 6 | 126.185 | -240.37 | 1.958 | 0.108 |
| 0.00319 | NA | NA | + | NA | NA | NA | 5 | 120.786 | -231.573 | 0 | 0.235 |
| 0.02382 | NA | NA | + | 1.03E-04 | NA | NA | 6 | 121.674 | -231.347 | 0.225 | 0.21 |
| 0.05235 | NA | NA | NA | 1.66E-04 | NA | NA | 3 | 118.579 | -231.158 | 0.415 | 0.191 |
| 0.01494 | NA | NA | + | NA | -5.07E-03 | NA | 6 | 120.879 | -229.759 | 1.814 | 0.095 |
| 0.00155 | 1.00E-10 | NA | + | NA | NA | NA | 6 | 120.84 | -229.681 | 1.892 | 0.091 |
| 0.00999 | NA | NA | + | NA | NA | -2.94E-03 | 6 | 120.826 | -229.652 | 1.921 | 0.09 |
| 0.03745 | NA | NA | + | 1.10E-04 | NA | -5.33E-03 | 7 | 121.802 | -229.604 | 1.969 | 0.088 |
| 0.0029 | NA | NA | + | NA | NA | NA | 5 | 125.22 | -240.44 | 0 | 0.346 |
| 0.02499 | NA | NA | + | NA | -9.66E-03 | NA | 6 | 125.667 | -239.335 | 1.105 | 0.199 |
| 0.01279 | NA | NA | + | 6.26E-05 | NA | NA | 6 | 125.618 | -239.235 | 1.204 | 0.189 |
| 0.0013 | 9.63E-11 | NA | + | NA | NA | NA | 6 | 125.282 | -238.564 | 1.875 | 0.135 |
| -0.00231 | NA | NA | + | NA | NA | 2.21E-03 | 6 | 125.246 | -238.491 | 1.948 | 0.131 |
| 0.00184 | NA | NA | + | NA | NA | NA | 5 | 123.506 | -237.011 | 0 | 0.346 |
| 0.01439 | NA | NA | + | 7.32E-05 | NA | NA | 6 | 124.02 | -236.039 | 0.972 | 0.213 |
| 0.022 | NA | NA | + | NA | -8.97E-03 | NA | 6 | 123.862 | -235.724 | 1.287 | 0.182 |
| 0.00065 | 6.72E-11 | NA | + | NA | NA | NA | 6 | 123.54 | -235.081 | 1.931 | 0.132 |
| -0.0015 | NA | NA | + | NA | NA | 1.41E-03 | 6 | 123.516 | -235.032 | 1.979 | 0.128 |
| 0.00083 | NA | NA | + | NA | NA | NA | 5 | 129.073 | -248.146 | 0 | 0.364 |
| 0.00957 | NA | NA | + | 5.37E-05 | NA | NA | 6 | 129.366 | -246.732 | 1.415 | 0.18 |
| 0.01828 | NA | NA | + | NA | -7.83E-03 | NA | 6 | 129.356 | -246.712 | 1.434 | 0.178 |
| -0.00062 | 8.23E-11 | NA | + | NA | NA | NA | 6 | 129.123 | -246.245 | 1.901 | 0.141 |
| -0.00415 | NA | NA | + | NA | NA | 2.11E-03 | 6 | 129.097 | -246.193 | 1.953 | 0.137 |
| 0.00358 | NA | NA | + | NA | NA | NA | 5 | 128.34 | -246.68 | 0 | 0.352 |
| 0.01387 | NA | NA | + | 6.28E-05 | NA | NA | 6 | 128.735 | -245.47 | I.21 | 0.192 |
| 0.02024 | NA | NA | + | NA | -7.37E-03 | NA | 6 | 128.609 | -245.217 | 1.463 | 0.17 |
| -0.00812 | NA | NA | + | NA | NA | 5.01E-03 | 6 | 128.484 | -244.969 | 1.711 | 0.15 |
| 0.00194 | 9.25E-11 | NA | + | NA | NA | NA | 6 | 128.388 | -244.776 | 1.904 | 0.136 |
| 0.04838 | NA | NA | NA | 1.55E-04 | NA | NA | 3 | 115.967 | -225.934 | 0 | 0.212 |
| 0.00252 | NA | NA | + | NA | NA | NA | 5 | 117.917 | -225.834 | 0.1 | 0.201 |
| 0.02177 | NA | NA | + | 9.78E-05 | NA | NA | 6 | 118.594 | -225.187 | 0.747 | 0.146 |
| 0.06667 | NA | NA | NA | 1.68E-04 | NA | -6.54E-03 | 4 | 116.149 | -224.298 | 1.635 | 0.093 |
| 0.06213 | NA | NA | NA | 1.46E-04 | -7.14E-03 | NA | 4 | 116.126 | -224.251 | 1.683 | 0.091 |
| 0.02094 | NA | NA | + | NA | -7.94E-03 | NA | 6 | 118.111 | -224.222 | 1.712 | 0.09 |
| 0.0161 | NA | NA | + | NA | NA | -5.75E-03 | 6 | 118.066 | -224.131 | 1.803 | 0.086 |
| 0.04707 | 8.24E-11 | NA | NA | 1.56E-04 | NA | NA | 4 | 116 | -224.001 | 1.933 | 0.081 |
| 0.00363 | NA | NA | + | NA | NA | NA | 5 | 121.431 | -232.863 | 0 | 0.347 |
| 0.01656 | NA | NA | + | 7.78E-05 | NA | NA | 6 | 121.965 | -231.931 | 0.932 | 0.218 |
| 0.02215 | NA | NA | + | NA | -8.37E-03 | NA | 6 | 121.711 | -231.422 | I.44 | 0.169 |
| 0.01163 | NA | NA | + | NA | NA | -3.27E-03 | 6 | 121.481 | -230.963 | 01.IX | 0.134 |
| 0.00206 | 9.04E-11 | NA | + | NA | NA | NA | 6 | 121.475 | -230.95 | 1.913 | 0.133 |
| 0.00473 | NA | NA | + | NA | NA | NA | 5 | 126.011 | -242.022 | 0 | 0.364 |
| 0.02607 | NA | NA | + | NA | -9.53E-03 | NA | 6 | 126.367 | -240.733 | 1.289 | 0.191 |
| 0.01335 | NA | NA | + | 5.14E-05 | NA | NA | 6 | 126.261 | -240.522 | 01.V | 0.172 |
| 0.01016 | NA | NA | + | NA | NA | -2.26E-03 | 6 | 126.038 | -240.077 | 1.946 | 0.138 |
| 0.00412 | 3.55E-11 | NA | + | NA | NA | NA | 6 | 126.025 | -240.05 | 1.973 | 0.136 |
| 0.00401 | NA | NA | + | NA | NA | NA | 5 | 121.943 | -233.885 | 0 | 0.347 |
| 0.01813 | NA | NA | + | 8.19E-05 | NA | NA | 6 | 122.521 | -233.042 | 0.844 | 0.228 |
| 0.02152 | NA | NA | + | NA | -7.98E-03 | NA | 6 | 122.191 | -232.383 | 1.502 | 0.164 |
| 0.00273 | 7.44E-11 | NA | + | NA | NA | NA | 6 | 121.982 | -231.965 | 1.921 | 0.133 |
| 0.00707 | NA | NA | + | NA | NA | -1.25E-03 | 6 | 121.95 | -231.9 | 1.985 | 0.129 |
| 0.00662 | NA | NA | + | NA | NA | NA | 5 | 128.259 | -246.517 | 0 | 0.354 |
| 0.02953 | NA | NA | + | NA | -1.04E-02 | NA | 6 | 128.703 | -245.407 | 01.XI | 0.203 |
| 0.01643 | NA | NA | + | 5.68E-05 | NA | NA | 6 | 128.569 | -245.137 | I.38 | 0.177 |
| 0.01224 | NA | NA | + | NA | NA | -2.33E-03 | 6 | 128.289 | -244.577 | I.94 | 0.134 |
| 0.00597 | 3.94E-11 | NA | + | NA | NA | NA | 6 | 128.275 | -244.549 | 1.968 | 0.132 |
| 0.00611 | NA | NA | + | NA | NA | NA | 5 | 121.777 | -233.553 | 0 | 0.362 |
| 0.02614 | NA | NA | + | NA | -8.97E-03 | NA | 6 | 122.085 | -232.17 | 1.383 | 0.181 |
| 0.01691 | NA | NA | + | 5.80E-05 | NA | NA | 6 | 122.064 | -232.128 | 1.425 | 0.177 |
| 0.01439 | NA | NA | + | NA | NA | -3.47E-03 | 6 | 121.835 | -231.671 | 1.882 | 0.141 |
| 0.00457 | 9.36E-11 | NA | + | NA | NA | NA | 6 | 121.82 | -231.641 | 1.912 | 0.139 |
| 0.00397 | NA | NA | + | NA | NA | NA | 5 | 120.191 | -230.382 | 0 | 0.372 |
| 0.01368 | NA | NA | + | 5.41E-05 | NA | NA | 6 | 120.434 | -228.868 | 1.515 | 0.174 |
| 0.02121 | NA | NA | + | NA | -7.59E-03 | NA | 6 | 120.399 | -228.797 | 1.585 | 0.168 |
| 0.01153 | NA | NA | + | NA | NA | -3.24E-03 | 6 | 120.242 | -228.483 | 1.899 | 0.144 |
| 0.00245 | 8.91E-11 | NA | + | NA | NA | NA | 6 | 120.232 | -228.463 | 1.919 | 0.142 |
| -0.00269 | NA | NA | + | NA | NA | NA | 5 | 119.586 | -229.171 | 0 | 0.364 |
| 0.01831 | NA | NA | + | NA | -9.25E-03 | NA | 6 | 120.018 | -228.035 | 1.136 | 0.206 |
| 0.00267 | NA | NA | + | 3.38E-05 | NA | NA | 6 | 119.701 | -227.401 | I.77 | 0.15 |
| -0.01002 | NA | NA | + | NA | NA | 3.15E-03 | 6 | 119.643 | -227.286 | 1.885 | 0.142 |
| -0.00354 | 5.01E-11 | NA | + | NA | NA | NA | 6 | 119.609 | -227.219 | 1.952 | 0.137 |
| -0.00032 | NA | NA | + | NA | NA | NA | 5 | 126.25 | -242.501 | 0 | 0.352 |
| 0.0226 | NA | NA | + | NA | -9.98E-03 | NA | 6 | 126.731 | -241.461 | 1.039 | 0.209 |
| 0.007 | NA | NA | + | 4.59E-05 | NA | NA | 6 | 126.476 | -240.952 | 1.548 | 0.162 |
| -0.00894 | NA | NA | + | NA | NA | 3.68E-03 | 6 | 126.334 | -240.667 | 1.833 | 0.141 |
| -0.00188 | 8.86E-11 | NA | + | NA | NA | NA | 6 | 126.298 | -240.596 | 1.905 | 0.136 |
| 0.00316 | NA | NA | + | NA | NA | NA | 5 | 125.721 | -241.442 | 0 | 0.351 |
| 0.01649 | NA | NA | + | 7.76E-05 | NA | NA | 6 | 126.246 | -240.493 | 0.949 | 0.218 |
| 0.01996 | NA | NA | + | NA | -7.57E-03 | NA | 6 | 125.929 | -239.858 | 1.584 | 0.159 |
| 0.00134 | 1.13E-10 | NA | + | NA | NA | NA | 6 | 125.797 | -239.593 | 1.849 | 0.139 |
| 0.0086 | NA | NA | + | NA | NA | -2.23E-03 | 6 | 125.746 | -239.492 | I.95 | 0.132 |
| 0.00074 | NA | NA | + | NA | NA | NA | 5 | 125.087 | -240.174 | 0 | 0.363 |
| 0.02132 | NA | NA | + | NA | -9.14E-03 | NA | 6 | 125.476 | -238.953 | 1.222 | 0.197 |
| 0.00721 | NA | NA | + | 4.14E-05 | NA | NA | 6 | 125.262 | -238.524 | 1.651 | 0.159 |
| -0.00125 | 1.16E-10 | NA | + | NA | NA | NA | 6 | 125.153 | -238.306 | 1.868 | 0.143 |
| -0.00502 | NA | NA | + | NA | NA | 2.44E-03 | 6 | 125.119 | -238.238 | 1.936 | 0.138 |
| 0.00126 | NA | NA | + | NA | NA | NA | 5 | 122.966 | -235.933 | 0 | 0.343 |
| 0.02713 | NA | NA | + | NA | -1.14E-02 | NA | 6 | 123.476 | -234.952 | 0.981 | 0.21 |
| 0.01303 | NA | NA | + | 6.69E-05 | NA | NA | 6 | 123.36 | -234.72 | 1.213 | 0.187 |
| -0.00054 | 1.02E-10 | NA | + | NA | NA | NA | 6 | 123.023 | -234.047 | 1.886 | 0.133 |
| 0.00481 | NA | NA | + | NA | NA | -1.50E-03 | 6 | 122.978 | -233.956 | 1.977 | 0.127 |
| 0.00217 | NA | NA | + | NA | NA | NA | 5 | 124.828 | -239.657 | 0 | 0.308 |
| 0.01645 | NA | NA | + | 7.77E-05 | NA | NA | 6 | 125.354 | -238.707 | 0.949 | 0.191 |
| 0.02171 | NA | NA | + | NA | -8.43E-03 | NA | 6 | 125.097 | -238.194 | 1.462 | 0.148 |
| -0.00009 | 1.38E-10 | NA | + | NA | NA | NA | 6 | 124.915 | -237.83 | 1.826 | 0.123 |
| 0.05042 | NA | NA | NA | 1.51E-04 | NA | NA | 3 | 121.844 | -237.688 | 1.969 | 0.115 |
| -0.00099 | NA | NA | + | NA | NA | 1.35E-03 | 6 | 124.838 | -237.676 | 1.981 | 0.114 |
| 0.03035 | NA | NA | + | NA | -1.32E-02 | NA | 6 | 127.697 | -243.395 | 0 | 0.223 |
| 0.00037 | NA | NA | + | NA | NA | NA | 5 | 126.587 | -243.175 | 0.22 | 0.2 |
| 0.01378 | NA | NA | + | NA | -1.29E-02 | 6.75E-03 | 7 | 127.975 | -241.951 | 1.444 | 0.108 |
| -0.01702 | NA | NA | + | NA | NA | 7.43E-03 | 6 | 126.92 | -241.841 | 1.554 | 0.102 |
| 0.00694 | NA | NA | + | 4.76E-05 | NA | NA | 6 | 126.869 | -241.738 | 1.657 | 0.097 |
| -0.00304 | 2.06E-10 | NA | + | NA | NA | NA | 6 | 126.791 | -241.582 | 1.813 | 0.09 |
| 0.02677 | 1.40E-10 | NA | + | NA | -1.27E-02 | NA | 7 | 127.79 | -241.581 | 1.814 | 0.09 |
| 0.0322 | NA | NA | + | 2.78E-05 | -1.23E-02 | NA | 7 | 127.79 | -241.579 | 1.815 | 0.09 |
| 0.00159 | NA | NA | + | NA | NA | NA | 5 | 122.573 | -235.147 | 0 | 0.359 |
| 0.0242 | NA | NA | + | NA | -9.90E-03 | NA | 6 | 123.001 | -234.002 | 1.145 | 0.202 |
| 0.00998 | NA | NA | + | 4.94E-05 | NA | NA | 6 | 122.797 | -233.593 | 1.553 | 0.165 |
| -0.00013 | 9.79E-11 | NA | + | NA | NA | NA | 6 | 122.628 | -233.256 | I.89 | 0.139 |
| 0.00568 | NA | NA | + | NA | NA | -1.77E-03 | 6 | 122.588 | -233.176 | I.97 | 0.134 |
| 0.00213 | NA | NA | + | NA | NA | NA | 5 | 119.075 | -228.15 | 0 | 0.345 |
| 0.01698 | NA | NA | + | 7.88E-05 | NA | NA | 6 | 119.554 | -227.108 | 1.043 | 0.205 |
| 0.02486 | NA | NA | + | NA | -9.79E-03 | NA | 6 | 119.394 | -226.787 | 1.363 | 0.175 |
| 0.01489 | NA | NA | + | NA | NA | -5.36E-03 | 6 | 119.221 | -226.441 | 1.709 | 0.147 |
| 0.00137 | 4.22E-11 | NA | + | NA | NA | NA | 6 | 119.089 | -226.177 | 1.973 | 0.129 |
| 0.00237 | NA | NA | + | NA | NA | NA | 5 | 125.203 | -240.405 | 0 | 0.311 |
| 0.0164 | NA | NA | + | 7.84E-05 | NA | NA | 6 | 125.766 | -239.532 | 0.873 | 0.201 |
| 0.01595 | NA | NA | + | NA | -5.99E-03 | NA | 6 | 125.346 | -238.692 | 1.713 | 0.132 |
| 0.00063 | 1.02E-10 | NA | + | NA | NA | NA | 6 | 125.257 | -238.513 | 1.892 | 0.121 |
| 0.04672 | NA | NA | NA | 1.47E-04 | NA | NA | 3 | 122.256 | -238.513 | 1.893 | 0.121 |
| 0.00484 | NA | NA | + | NA | NA | -1.03E-03 | 6 | 125.208 | -238.416 | 1.989 | 0.115 |
| 0.00287 | NA | NA | + | NA | NA | NA | 5 | 124.495 | -238.991 | 0 | 0.379 |
| 0.01052 | NA | NA | + | 4.57E-05 | NA | NA | 6 | 124.689 | -237.378 | 1.612 | 0.169 |
| 0.01821 | NA | NA | + | NA | -6.78E-03 | NA | 6 | 124.685 | -237.371 | I.62 | 0.169 |
| -0.00151 | NA | NA | + | NA | NA | 1.86E-03 | 6 | 124.515 | -237.029 | 1.962 | 0.142 |
| 0.00235 | 3.14E-11 | NA | + | NA | NA | NA | 6 | 124.509 | -237.018 | 1.972 | 0.141 |
| -0.0008 | NA | NA | + | NA | NA | NA | 5 | 129.713 | -249.425 | 0 | 0.351 |
| 0.0196 | NA | NA | + | NA | -9.02E-03 | NA | 6 | 130.142 | -248.284 | 1.141 | 0.199 |
| 0.00841 | NA | NA | + | 5.67E-05 | NA | NA | 6 | 130.052 | -248.105 | I.32 | 0.182 |
| -0.00275 | 1.15E-10 | NA | + | NA | NA | NA | 6 | 129.779 | -247.558 | 1.867 | 0.138 |
| -0.00373 | NA | NA | + | NA | NA | 1.24E-03 | 6 | 129.721 | -247.441 | 1.984 | 0.13 |
| 0.0017 | NA | NA | + | NA | NA | NA | 5 | 122.583 | -235.165 | 0 | 0.348 |
| 0.02539 | NA | NA | + | NA | -1.05E-02 | NA | 6 | 123.047 | -234.093 | 1.072 | 0.204 |
| 0.01211 | NA | NA | + | 5.94E-05 | NA | NA | 6 | 122.921 | -233.842 | 1.323 | 0.18 |
| -0.00027 | 1.13E-10 | NA | + | NA | NA | NA | 6 | 122.667 | -233.334 | 1.831 | 0.139 |
| 0.00039 | NA | NA | + | NA | NA | 5.48E-04 | 6 | 122.584 | -233.168 | 1.997 | 0.128 |
| 0.00724 | NA | NA | + | NA | NA | NA | 5 | 120.407 | -230.813 | 0 | 0.311 |
| 0.02208 | NA | NA | + | 8.15E-05 | NA | NA | 6 | 120.921 | -229.842 | 0.971 | 0.191 |
| 0.04661 | NA | NA | NA | 1.38E-04 | NA | NA | 3 | 117.671 | -229.343 | 1.471 | 0.149 |
| 0.01456 | NA | NA | + | NA | -3.23E-03 | NA | 6 | 120.441 | -228.883 | 1.931 | 0.118 |
| 0.00667 | 3.37E-11 | NA | + | NA | NA | NA | 6 | 120.414 | -228.829 | 1.984 | 0.115 |
| 0.00833 | NA | NA | + | NA | NA | -4.49E-04 | 6 | 120.408 | -228.816 | 1.998 | 0.115 |
| 0.00377 | NA | NA | + | NA | NA | NA | 5 | 124.056 | -238.112 | 0 | 0.258 |
| 0.04845 | NA | NA | NA | 1.51E-04 | NA | NA | 3 | 121.954 | -237.908 | 0.204 | 0.233 |
| 0.02286 | NA | NA | + | 9.85E-05 | NA | NA | 6 | 124.836 | -237.672 | 0.44 | 0.207 |
| 0.01248 | NA | NA | + | NA | NA | -3.76E-03 | 6 | 124.126 | -236.251 | 1.861 | 0.102 |
| 0.01379 | NA | NA | + | NA | -4.36E-03 | NA | 6 | 124.122 | -236.243 | 1.869 | 0.101 |
| 0.00234 | 8.22E-11 | NA | + | NA | NA | NA | 6 | 124.094 | -236.188 | 1.924 | 0.099 |
| 0.00704 | NA | NA | + | NA | NA | NA | 5 | 122.382 | -234.764 | 0 | 0.293 |
| 0.02266 | NA | NA | + | 8.70E-05 | NA | NA | 6 | 123.011 | -234.022 | 0.742 | 0.202 |
| 0.04895 | NA | NA | NA | 1.47E-04 | NA | NA | 3 | 119.802 | -233.605 | 1.159 | 0.164 |
| 0.01923 | NA | NA | + | NA | -5.38E-03 | NA | 6 | 122.487 | -232.974 | I.79 | 0.12 |
| 0.00582 | 7.32E-11 | NA | + | NA | NA | NA | 6 | 122.41 | -232.821 | 1.943 | 0.111 |
| 0.01162 | NA | NA | + | NA | NA | -1.91E-03 | 6 | 122.399 | -232.799 | 1.965 | 0.11 |
| 0.00317 | NA | NA | + | NA | NA | NA | 5 | 123.079 | -236.159 | 0 | 0.352 |
| 0.01449 | NA | NA | + | 7.00E-05 | NA | NA | 6 | 123.541 | -235.083 | 1.076 | 0.206 |
| 0.0227 | NA | NA | + | NA | -8.59E-03 | NA | 6 | 123.372 | -234.745 | 1.414 | 0.174 |
| 0.00151 | 9.77E-11 | NA | + | NA | NA | NA | 6 | 123.139 | -234.279 | I.88 | 0.138 |
| 0.00097 | NA | NA | + | NA | NA | 9.28E-04 | 6 | 123.084 | -234.167 | 1.991 | 0.13 |
| -0.00031 | NA | NA | + | NA | NA | NA | 5 | 118.189 | -226.378 | 0 | 0.329 |
| 0.01838 | NA | NA | + | 9.62E-05 | NA | NA | 6 | 118.937 | -225.875 | 0.503 | 0.255 |
| 0.0202 | NA | NA | + | NA | -8.96E-03 | NA | 6 | 118.471 | -224.941 | 1.437 | 0.16 |
| -0.00215 | 1.05E-10 | NA | + | NA | NA | NA | 6 | 118.25 | -224.5 | 1.878 | 0.128 |
| 0.00745 | NA | NA | + | NA | NA | -3.28E-03 | 6 | 118.242 | -224.483 | 1.895 | 0.127 |
| 0.00375 | NA | NA | + | NA | NA | NA | 5 | 117.691 | -225.382 | 0 | 0.283 |
| 0.02055 | NA | NA | + | 9.30E-05 | NA | NA | 6 | 118.371 | -224.743 | 0.639 | 0.206 |
| 0.04787 | NA | NA | NA | 1.54E-04 | NA | NA | 3 | 115.267 | -224.535 | 0.847 | 0.185 |
| 0.01491 | NA | NA | + | NA | -4.91E-03 | NA | 6 | 117.772 | -223.543 | 1.839 | 0.113 |
| 0.00252 | 7.34E-11 | NA | + | NA | NA | NA | 6 | 117.721 | -223.442 | 1.939 | 0.107 |
| 0.00727 | NA | NA | + | NA | NA | -1.45E-03 | 6 | 117.701 | -223.403 | 1.979 | 0.105 |
| 0.00371 | NA | NA | + | NA | NA | NA | 5 | 124.332 | -238.665 | 0 | 0.351 |
| 0.02864 | NA | NA | + | NA | -1.10E-02 | NA | 6 | 124.915 | -237.831 | 0.834 | 0.231 |
| 0.01044 | NA | NA | + | 4.13E-05 | NA | NA | 6 | 124.5 | -237 | 1.665 | 0.153 |
| 0.0025 | 7.17E-11 | NA | + | NA | NA | NA | 6 | 124.364 | -236.729 | 1.936 | 0.133 |
| -0.00131 | NA | NA | + | NA | NA | 2.10E-03 | 6 | 124.358 | -236.715 | I.95 | 0.132 |
| 0.00344 | NA | NA | + | NA | NA | NA | 5 | 116.594 | -223.188 | 0 | 0.214 |
| 0.02309 | NA | NA | + | 1.10E-04 | NA | NA | 6 | 117.545 | -223.09 | 0.098 | 0.204 |
| 0.04701 | NA | NA | NA | 1.62E-04 | NA | NA | 3 | 114.222 | -222.445 | 0.743 | 0.147 |
| 0.02035 | NA | NA | + | NA | -7.49E-03 | NA | 6 | 116.783 | -221.565 | 1.623 | 0.095 |
| 0.00012 | 1.91E-10 | NA | + | NA | NA | NA | 6 | 116.756 | -221.512 | 1.676 | 0.093 |
| 0.01981 | 1.68E-10 | NA | + | 1.08E-04 | NA | NA | 7 | 117.671 | -221.342 | 1.846 | 0.085 |
| 0.0353 | NA | NA | + | 1.06E-04 | -5.73E-03 | NA | 7 | 117.656 | -221.311 | 1.877 | 0.084 |
| 0.00172 | NA | NA | + | NA | NA | 7.14E-04 | 6 | 116.596 | -221.192 | 1.996 | 0.079 |
| 0.00688 | NA | NA | + | NA | NA | NA | 5 | 130.126 | -250.251 | 0 | 0.347 |
| 0.01951 | NA | NA | + | 7.46E-05 | NA | NA | 6 | 130.639 | -249.279 | 0.973 | 0.213 |
| 0.02672 | NA | NA | + | NA | -8.94E-03 | NA | 6 | 130.469 | -248.938 | 1.313 | 0.18 |
| 0.0058 | 6.91E-11 | NA | + | NA | NA | NA | 6 | 130.165 | -248.33 | 1.922 | 0.133 |
| 0.00842 | NA | NA | + | NA | NA | -6.27E-04 | 6 | 130.128 | -248.256 | 1.996 | 0.128 |
| 0 | NA | NA | + | NA | NA | NA | 5 | 119.152 | -228.304 | 0 | 0.366 |
| 0.02068 | NA | NA | + | NA | -9.07E-03 | NA | 6 | 119.471 | -226.942 | 1.362 | 0.185 |
| 0.01067 | NA | NA | + | 5.57E-05 | NA | NA | 6 | 119.397 | -226.794 | I.51 | 0.172 |
| -0.00145 | 8.01E-11 | NA | + | NA | NA | NA | 6 | 119.191 | -226.382 | 1.922 | 0.14 |
| 0.00382 | NA | NA | + | NA | NA | -1.63E-03 | 6 | 119.165 | -226.329 | 1.975 | 0.136 |
| 0.00462 | NA | NA | + | NA | NA | NA | 5 | 124.051 | -238.102 | 0 | 0.292 |
| 0.01986 | NA | NA | + | 8.34E-05 | NA | NA | 6 | 124.646 | -237.292 | 0.81 | 0.195 |
| 0.02673 | NA | NA | + | NA | -9.93E-03 | NA | 6 | 124.416 | -236.832 | I.27 | 0.155 |
| 0.0456 | NA | NA | NA | 1.42E-04 | NA | NA | 3 | 121.29 | -236.579 | 1.523 | 0.136 |
| 0.0032 | 7.97E-11 | NA | + | NA | NA | NA | 6 | 124.088 | -236.177 | 1.926 | 0.111 |
| 0.01098 | NA | NA | + | NA | NA | -2.65E-03 | 6 | 124.087 | -236.174 | 1.928 | 0.111 |
| 0.00826 | NA | NA | + | NA | NA | NA | 5 | 127.193 | -244.386 | 0 | 0.313 |
| 0.02153 | NA | NA | + | 7.31E-05 | NA | NA | 6 | 127.634 | -243.267 | 1.118 | 0.179 |
| 0.02236 | NA | NA | + | NA | -6.43E-03 | NA | 6 | 127.35 | -242.701 | 1.685 | 0.135 |
| 0.04505 | NA | NA | NA | 1.34E-04 | NA | NA | 3 | 124.307 | -242.613 | 1.773 | 0.129 |
| 0.01686 | NA | NA | + | NA | NA | -3.51E-03 | 6 | 127.256 | -242.511 | 1.875 | 0.123 |
| 0.00658 | 9.83E-11 | NA | + | NA | NA | NA | 6 | 127.241 | -242.482 | 1.904 | 0.121 |
| 0.00394 | NA | NA | + | NA | NA | NA | 5 | 123.759 | -237.518 | 0 | 0.346 |
| 0.02682 | NA | NA | + | NA | -1.01E-02 | NA | 6 | 124.212 | -236.424 | 1.094 | 0.2 |
| 0.01472 | NA | NA | + | 6.54E-05 | NA | NA | 6 | 124.176 | -236.352 | 1.166 | 0.193 |
| 0.00304 | 5.24E-11 | NA | + | NA | NA | NA | 6 | 123.784 | -235.569 | 1.949 | 0.131 |
| -0.00067 | NA | NA | + | NA | NA | 1.94E-03 | 6 | 123.78 | -235.559 | 1.959 | 0.13 |
| 0.02117 | NA | NA | + | 1.10E-04 | NA | NA | 6 | 124.901 | -237.802 | 0 | 0.169 |
| -0.00013 | NA | NA | + | NA | NA | NA | 5 | 123.879 | -237.757 | 0.045 | 0.165 |
| 0.04802 | NA | NA | NA | 1.75E-04 | NA | NA | 3 | 121.615 | -237.229 | 0.573 | 0.127 |
| 0.0244 | NA | NA | + | NA | -1.08E-02 | NA | 6 | 124.299 | -236.599 | 1.204 | 0.093 |
| 0.0451 | NA | NA | + | 1.22E-04 | NA | -9.07E-03 | 7 | 125.274 | -236.548 | 1.254 | 0.09 |
| 0.0409 | NA | NA | + | 1.04E-04 | -9.19E-03 | NA | 7 | 125.207 | -236.415 | 1.388 | 0.085 |
| 0.01492 | NA | NA | + | NA | NA | -6.28E-03 | 6 | 124.06 | -236.121 | 1.681 | 0.073 |
| -0.00206 | 1.14E-10 | NA | + | NA | NA | NA | 6 | 123.966 | -235.932 | I.87 | 0.066 |
| 0.06647 | NA | NA | NA | 1.61E-04 | -9.75E-03 | NA | 4 | 121.956 | -235.912 | I.89 | 0.066 |
| 0.01936 | 8.80E-11 | NA | + | 1.09E-04 | NA | NA | 7 | 124.954 | -235.907 | 1.895 | 0.066 |
| 0.00319 | NA | NA | + | NA | NA | NA | 5 | 126.435 | -242.87 | 0 | 0.375 |
| 0.01203 | NA | NA | + | 4.98E-05 | NA | NA | 6 | 126.659 | -241.318 | 1.552 | 0.173 |
| 0.01805 | NA | NA | + | NA | -6.56E-03 | NA | 6 | 126.606 | -241.212 | 1.658 | 0.164 |
| -0.00453 | NA | NA | + | NA | NA | 3.28E-03 | 6 | 126.501 | -241.003 | 1.867 | 0.147 |
| 0.0021 | 6.38E-11 | NA | + | NA | NA | NA | 6 | 126.46 | -240.921 | 1.949 | 0.141 |
| 0.00598 | NA | NA | + | NA | NA | NA | 5 | 126.941 | -243.882 | 0 | 0.366 |
| 0.01608 | NA | NA | + | 5.70E-05 | NA | NA | 6 | 127.229 | -242.457 | 1.424 | 0.18 |
| 0.02426 | NA | NA | + | NA | -8.14E-03 | NA | 6 | 127.193 | -242.387 | 1.495 | 0.173 |
| 0.00431 | 1.01E-10 | NA | + | NA | NA | NA | 6 | 126.996 | -241.992 | 1.889 | 0.142 |
| 0.01154 | NA | NA | + | NA | NA | -2.32E-03 | 6 | 126.969 | -241.937 | 1.944 | 0.138 |
| 0.00287 | NA | NA | + | NA | NA | NA | 5 | 123.667 | -237.335 | 0 | 0.26 |
| 0.0476 | NA | NA | NA | 1.53E-04 | NA | NA | 3 | 121.577 | -237.154 | 0.181 | 0.238 |
| 0.02079 | NA | NA | + | 9.57E-05 | NA | NA | 6 | 124.424 | -236.847 | 0.488 | 0.204 |
| 0.01078 | NA | NA | + | NA | -3.45E-03 | NA | 6 | 123.711 | -235.422 | 1.913 | 0.1 |
| 0.00968 | NA | NA | + | NA | NA | -2.88E-03 | 6 | 123.707 | -235.413 | 1.921 | 0.1 |
| 0.00151 | 7.79E-11 | NA | + | NA | NA | NA | 6 | 123.701 | -235.402 | 1.933 | 0.099 |
| 0.00378 | NA | NA | + | NA | NA | NA | 5 | 125.525 | -241.05 | 0 | 0.36 |
| 0.01605 | NA | NA | + | 6.71E-05 | NA | NA | 6 | 125.912 | -239.825 | 1.225 | 0.195 |
| 0.02269 | NA | NA | + | NA | -8.22E-03 | NA | 6 | 125.775 | -239.549 | 01.V | 0.17 |
| 0.01051 | NA | NA | + | NA | NA | -2.85E-03 | 6 | 125.565 | -239.131 | 1.919 | 0.138 |
| 0.00249 | 7.42E-11 | NA | + | NA | NA | NA | 6 | 125.556 | -239.112 | 1.937 | 0.137 |
| 0.00335 | NA | NA | + | NA | NA | NA | 5 | 128.254 | -246.507 | 0 | 0.345 |
| 0.01418 | NA | NA | + | 6.79E-05 | NA | NA | 6 | 128.735 | -245.47 | 1.037 | 0.205 |
| 0.02148 | NA | NA | + | NA | -7.97E-03 | NA | 6 | 128.572 | -245.144 | 1.363 | 0.174 |
| -0.00683 | NA | NA | + | NA | NA | 4.30E-03 | 6 | 128.355 | -244.71 | 1.797 | 0.14 |
| 0.00172 | 1.00E-10 | NA | + | NA | NA | NA | 6 | 128.321 | -244.641 | 1.866 | 0.136 |
| 0.00012 | NA | NA | + | NA | NA | NA | 5 | 125.3 | -240.6 | 0 | 0.346 |
| 0.0144 | NA | NA | + | 7.92E-05 | NA | NA | 6 | 125.859 | -239.718 | 0.882 | 0.222 |
| 0.01982 | NA | NA | + | NA | -8.63E-03 | NA | 6 | 125.588 | -239.176 | 1.424 | 0.17 |
| -0.00158 | 9.80E-11 | NA | + | NA | NA | NA | 6 | 125.361 | -238.722 | 1.878 | 0.135 |
| 0.00183 | NA | NA | + | NA | NA | -7.21E-04 | 6 | 125.303 | -238.605 | 1.995 | 0.127 |
| 0.00361 | NA | NA | + | NA | NA | NA | 5 | 125.869 | -241.738 | 0 | 0.363 |
| 0.01478 | NA | NA | + | 6.29E-05 | NA | NA | 6 | 126.212 | -240.424 | 1.315 | 0.188 |
| 0.02315 | NA | NA | + | NA | -8.58E-03 | NA | 6 | 126.155 | -240.31 | 1.429 | 0.178 |
| 0.0027 | 5.39E-11 | NA | + | NA | NA | NA | 6 | 125.89 | -239.78 | 1.958 | 0.136 |
| 0.00571 | NA | NA | + | NA | NA | -8.77E-04 | 6 | 125.873 | -239.747 | 1.991 | 0.134 |
| 0.00142 | NA | NA | + | NA | NA | NA | 5 | 122.624 | -235.247 | 0 | 0.36 |
| 0.01483 | NA | NA | + | 7.35E-05 | NA | NA | 6 | 123.098 | -234.197 | 1.051 | 0.213 |
| 0.01604 | NA | NA | + | NA | -6.52E-03 | NA | 6 | 122.792 | -233.584 | 1.664 | 0.157 |
| 0.00056 | 4.82E-11 | NA | + | NA | NA | NA | 6 | 122.64 | -233.281 | 1.967 | 0.135 |
| -0.00237 | NA | NA | + | NA | NA | 1.59E-03 | 6 | 122.638 | -233.277 | 1.971 | 0.135 |
| 0.04876 | NA | NA | NA | 1.56E-04 | NA | NA | 3 | 117.156 | -228.312 | 0 | 0.241 |
| 0.00312 | NA | NA | + | NA | NA | NA | 5 | 119.005 | -228.009 | 0.303 | 0.207 |
| 0.02335 | NA | NA | + | 1.04E-04 | NA | NA | 6 | 119.809 | -227.618 | 0.694 | 0.17 |
| 0.06603 | NA | NA | NA | 1.67E-04 | NA | -6.20E-03 | 4 | 117.329 | -226.659 | 1.653 | 0.105 |
| 0.06004 | NA | NA | NA | 1.49E-04 | -5.84E-03 | NA | 4 | 117.265 | -226.53 | 1.782 | 0.099 |
| 0.01662 | NA | NA | + | NA | NA | -5.75E-03 | 6 | 119.161 | -226.321 | 1.991 | 0.089 |
| 0.04838 | 2.40E-11 | NA | NA | 1.56E-04 | NA | NA | 4 | 117.16 | -226.321 | 1.991 | 0.089 |
| 0.0042 | NA | NA | + | NA | NA | NA | 5 | 127.621 | -245.241 | 0 | 0.297 |
| 0.01841 | NA | NA | + | 7.80E-05 | NA | NA | 6 | 128.173 | -244.346 | 0.895 | 0.19 |
| 0.02466 | NA | NA | + | NA | -8.99E-03 | NA | 6 | 127.939 | -243.878 | 1.363 | 0.15 |
| 0.04774 | NA | NA | NA | 1.44E-04 | NA | NA | 3 | 124.773 | -243.545 | 1.696 | 0.127 |
| 0.01548 | NA | NA | + | NA | NA | -4.82E-03 | 6 | 127.737 | -243.474 | 1.767 | 0.123 |
| 0.00335 | 5.05E-11 | NA | + | NA | NA | NA | 6 | 127.642 | -243.284 | 1.957 | 0.112 |
| -0.00263 | NA | NA | + | NA | NA | NA | 5 | 118.943 | -227.886 | 0 | 0.273 |
| 0.02998 | NA | NA | + | NA | -1.42E-02 | NA | 6 | 119.624 | -227.249 | 0.638 | 0.199 |
| 0.0132 | NA | NA | + | 8.47E-05 | NA | NA | 6 | 119.559 | -227.118 | 0.768 | 0.186 |
| 0.01191 | NA | NA | + | NA | NA | -6.07E-03 | 6 | 119.119 | -226.238 | 1.649 | 0.12 |
| 0.04102 | NA | NA | + | 7.57E-05 | -1.29E-02 | NA | 7 | 120.114 | -226.228 | 1.658 | 0.119 |
| -0.00322 | 3.44E-11 | NA | + | NA | NA | NA | 6 | 118.967 | -225.935 | 1.951 | 0.103 |
| 0.00029 | NA | NA | + | NA | NA | NA | 5 | 121.822 | -233.643 | 0 | 0.3 |
| 0.01612 | NA | NA | + | 8.34E-05 | NA | NA | 6 | 122.4 | -232.799 | 0.844 | 0.197 |
| 0.01917 | NA | NA | + | NA | -8.38E-03 | NA | 6 | 122.077 | -232.154 | 1.489 | 0.143 |
| 0.04612 | NA | NA | NA | 1.54E-04 | NA | NA | 3 | 118.972 | -231.945 | 1.699 | 0.128 |
| -0.00183 | 1.20E-10 | NA | + | NA | NA | NA | 6 | 121.889 | -231.778 | 1.865 | 0.118 |
| 0.00613 | NA | NA | + | NA | NA | -2.51E-03 | 6 | 121.852 | -231.704 | I.94 | 0.114 |
| 0.00497 | NA | NA | + | NA | NA | NA | 5 | 127.391 | -244.782 | 0 | 0.337 |
| 0.03053 | NA | NA | + | NA | -1.14E-02 | NA | 6 | 128.049 | -244.098 | 0.684 | 0.239 |
| 0.01307 | NA | NA | + | 5.18E-05 | NA | NA | 6 | 127.68 | -243.36 | 1.422 | 0.165 |
| 0.00362 | 8.35E-11 | NA | + | NA | NA | NA | 6 | 127.453 | -242.907 | 1.876 | 0.132 |
| 0.00003 | NA | NA | + | NA | NA | 2.06E-03 | 6 | 127.414 | -242.829 | 1.954 | 0.127 |
| 0.00177 | NA | NA | + | NA | NA | NA | 5 | 125.886 | -241.772 | 0 | 0.362 |
| 0.02322 | NA | NA | + | NA | -9.34E-03 | NA | 6 | 126.207 | -240.414 | 1.358 | 0.184 |
| 0.01183 | NA | NA | + | 5.43E-05 | NA | NA | 6 | 126.145 | -240.29 | 1.482 | 0.173 |
| 0.01209 | NA | NA | + | NA | NA | -4.41E-03 | 6 | 125.985 | -239.97 | 1.802 | 0.147 |
| 0.00114 | 3.90E-11 | NA | + | NA | NA | NA | 6 | 125.899 | -239.798 | 1.974 | 0.135 |
| 0.00634 | NA | NA | + | NA | NA | NA | 5 | 122.79 | -235.58 | 0 | 0.255 |
| 0.02275 | NA | NA | + | 9.19E-05 | NA | NA | 6 | 123.493 | -234.987 | 0.593 | 0.19 |
| 0.03054 | NA | NA | + | NA | -1.09E-02 | NA | 6 | 123.218 | -234.436 | 1.144 | 0.144 |
| 0.04789 | NA | NA | NA | 1.60E-04 | NA | NA | 3 | 119.947 | -233.894 | 1.686 | 0.11 |
| 0.00424 | 1.31E-10 | NA | + | NA | NA | NA | 6 | 122.879 | -233.758 | 1.822 | 0.103 |
| 0.01639 | NA | NA | + | NA | NA | -4.08E-03 | 6 | 122.87 | -233.74 | I.84 | 0.102 |
| 0.04284 | NA | NA | + | 8.54E-05 | -9.59E-03 | NA | 7 | 123.822 | -233.645 | 1.935 | 0.097 |
| -0.00043 | NA | NA | + | NA | NA | NA | 5 | 121.491 | -232.982 | 0 | 0.229 |
| 0.01727 | NA | NA | + | 9.40E-05 | NA | NA | 6 | 122.242 | -232.485 | 0.497 | 0.178 |
| 0.02359 | NA | NA | + | NA | -1.05E-02 | NA | 6 | 121.863 | -231.726 | 1.256 | 0.122 |
| 0.04586 | NA | NA | NA | 1.54E-04 | NA | NA | 3 | 118.766 | -231.531 | 1.451 | 0.111 |
| 0.01258 | NA | NA | + | NA | NA | -5.45E-03 | 6 | 121.628 | -231.256 | 1.727 | 0.096 |
| -0.00189 | 8.32E-11 | NA | + | NA | NA | NA | 6 | 121.54 | -231.081 | 1.901 | 0.088 |
| 0.0376 | NA | NA | + | 8.90E-05 | -9.26E-03 | NA | 7 | 122.535 | -231.069 | 1.913 | 0.088 |
| 0.03823 | NA | NA | + | 1.04E-04 | NA | -7.95E-03 | 7 | 122.528 | -231.056 | 1.927 | 0.087 |
| 0.00517 | NA | NA | + | NA | NA | NA | 5 | 125.775 | -241.551 | 0 | 0.323 |
| 0.01738 | NA | NA | + | 6.67E-05 | NA | NA | 6 | 126.175 | -240.349 | 1.201 | 0.177 |
| 0.01865 | NA | NA | + | NA | -5.85E-03 | NA | 6 | 125.906 | -239.812 | 1.739 | 0.135 |
| 0.04918 | NA | NA | NA | 1.32E-04 | NA | NA | 3 | 122.815 | -239.631 | I.92 | 0.124 |
| 0.00406 | 6.54E-11 | NA | + | NA | NA | NA | 6 | 125.799 | -239.598 | 1.953 | 0.122 |
| 0.00743 | NA | NA | + | NA | NA | -9.86E-04 | 6 | 125.78 | -239.56 | I.99 | 0.119 |
| 0.00101 | NA | NA | + | NA | NA | NA | 5 | 126.891 | -243.782 | 0 | 0.346 |
| 0.02797 | NA | NA | + | NA | -1.18E-02 | NA | 6 | 127.479 | -242.958 | 0.823 | 0.229 |
| 0.01001 | NA | NA | + | 5.32E-05 | NA | NA | 6 | 127.154 | -242.308 | 1.473 | 0.166 |
| 0.00015 | 4.76E-11 | NA | + | NA | NA | NA | 6 | 126.912 | -241.825 | 1.957 | 0.13 |
| 0.00351 | NA | NA | + | NA | NA | -1.04E-03 | 6 | 126.897 | -241.794 | 1.988 | 0.128 |
| -0.00236 | NA | NA | + | NA | NA | NA | 5 | 126.244 | -242.487 | 0 | 0.349 |
| 0.02152 | NA | NA | + | NA | -1.03E-02 | NA | 6 | 126.77 | -241.539 | 0.948 | 0.217 |
| 0.00502 | NA | NA | + | 4.70E-05 | NA | NA | 6 | 126.484 | -240.967 | I.52 | 0.163 |
| -0.01008 | NA | NA | + | NA | NA | 3.37E-03 | 6 | 126.31 | -240.621 | 1.867 | 0.137 |
| -0.00339 | 5.94E-11 | NA | + | NA | NA | NA | 6 | 126.275 | -240.55 | 1.937 | 0.133 |
| -0.00029 | NA | NA | + | NA | NA | NA | 5 | 116.537 | -223.073 | 0 | 0.348 |
| 0.01539 | NA | NA | + | 8.59E-05 | NA | NA | 6 | 117.119 | -222.238 | 0.835 | 0.23 |
| 0.017 | NA | NA | + | NA | -7.55E-03 | NA | 6 | 116.733 | -221.466 | 1.607 | 0.156 |
| -0.00226 | 1.11E-10 | NA | + | NA | NA | NA | 6 | 116.595 | -221.19 | 1.883 | 0.136 |
| 0.00403 | NA | NA | + | NA | NA | -1.84E-03 | 6 | 116.552 | -221.104 | 1.969 | 0.13 |
| -0.00244 | NA | NA | + | NA | NA | NA | 5 | 127.336 | -244.672 | 0 | 0.327 |
| 0.01043 | NA | NA | + | 7.50E-05 | NA | NA | 6 | 127.887 | -243.774 | 0.898 | 0.209 |
| 0.02225 | NA | NA | + | NA | -1.08E-02 | NA | 6 | 127.885 | -243.77 | 0.902 | 0.209 |
| -0.00478 | 1.39E-10 | NA | + | NA | NA | NA | 6 | 127.442 | -242.884 | 1.789 | 0.134 |
| 0.00005 | NA | NA | + | NA | NA | -1.06E-03 | 6 | 127.342 | -242.683 | 1.989 | 0.121 |
| 0.00278 | NA | NA | + | NA | NA | NA | 5 | 118.89 | -227.78 | 0 | 0.315 |
| 0.01807 | NA | NA | + | 7.93E-05 | NA | NA | 6 | 119.374 | -226.748 | 1.032 | 0.188 |
| 0.04611 | NA | NA | NA | 1.48E-04 | NA | NA | 3 | 116.001 | -226.003 | 1.778 | 0.13 |
| 0.01178 | NA | NA | + | NA | NA | -3.80E-03 | 6 | 118.959 | -225.918 | 1.862 | 0.124 |
| 0.01227 | NA | NA | + | NA | -4.23E-03 | NA | 6 | 118.948 | -225.897 | 1.883 | 0.123 |
| 0.00168 | 6.33E-11 | NA | + | NA | NA | NA | 6 | 118.919 | -225.838 | 1.942 | 0.119 |
| 0.00633 | NA | NA | + | NA | NA | NA | 5 | 123.245 | -236.49 | 0 | 0.31 |
| 0.01938 | NA | NA | + | 7.38E-05 | NA | NA | 6 | 123.699 | -235.398 | 1.092 | 0.179 |
| 0.04722 | NA | NA | NA | 1.37E-04 | NA | NA | 3 | 120.579 | -235.158 | 1.332 | 0.159 |
| 0.01621 | NA | NA | + | NA | -4.35E-03 | NA | 6 | 123.312 | -234.623 | 1.867 | 0.122 |
| 0.00537 | 5.63E-11 | NA | + | NA | NA | NA | 6 | 123.262 | -234.523 | 1.967 | 0.116 |
| 0.00908 | NA | NA | + | NA | NA | -1.15E-03 | 6 | 123.251 | -234.502 | 1.987 | 0.115 |
| 0.00503 | NA | NA | + | NA | NA | NA | 5 | 132.582 | -255.164 | 0 | 0.355 |
| 0.01672 | NA | NA | + | 6.67E-05 | NA | NA | 6 | 133.01 | -254.02 | 1.144 | 0.2 |
| 0.02452 | NA | NA | + | NA | -8.59E-03 | NA | 6 | 132.882 | -253.765 | 1.399 | 0.176 |
| 0.01175 | NA | NA | + | NA | NA | -2.82E-03 | 6 | 132.623 | -253.247 | 1.917 | 0.136 |
| 0.00408 | 5.69E-11 | NA | + | NA | NA | NA | 6 | 132.603 | -253.207 | 1.957 | 0.133 |
| 0.00209 | NA | NA | + | NA | NA | NA | 5 | 124.596 | -239.193 | 0 | 0.337 |
| 0.02878 | NA | NA | + | NA | -1.20E-02 | NA | 6 | 125.267 | -238.534 | 0.658 | 0.242 |
| 0.01057 | NA | NA | + | 5.22E-05 | NA | NA | 6 | 124.872 | -237.744 | 1.449 | 0.163 |
| -0.00004 | 1.22E-10 | NA | + | NA | NA | NA | 6 | 124.671 | -237.341 | 1.852 | 0.133 |
| 0.00394 | NA | NA | + | NA | NA | -7.76E-04 | 6 | 124.599 | -237.199 | 1.994 | 0.124 |
| 0.0042 | NA | NA | + | NA | NA | NA | 5 | 119.886 | -229.771 | 0 | 0.299 |
| 0.01933 | NA | NA | + | 7.88E-05 | NA | NA | 6 | 120.359 | -228.719 | 1.053 | 0.177 |
| 0.02283 | NA | NA | + | NA | NA | -7.81E-03 | 6 | 120.159 | -228.318 | 1.454 | 0.145 |
| 0.02416 | NA | NA | + | NA | -8.84E-03 | NA | 6 | 120.138 | -228.276 | 1.495 | 0.142 |
| 0.04681 | NA | NA | NA | 1.46E-04 | NA | NA | 3 | 116.987 | -227.975 | 1.796 | 0.122 |
| 0.00254 | 9.73E-11 | NA | + | NA | NA | NA | 6 | 119.932 | -227.863 | 1.908 | 0.115 |
| 0.00515 | NA | NA | + | NA | NA | NA | 5 | 123.174 | -236.348 | 0 | 0.279 |
| 0.0208 | NA | NA | + | 8.19E-05 | NA | NA | 6 | 123.734 | -235.468 | 0.88 | 0.18 |
| 0.04803 | NA | NA | NA | 1.45E-04 | NA | NA | 3 | 120.648 | -235.296 | 1.051 | 0.165 |
| 0.02701 | NA | NA | + | NA | -9.64E-03 | NA | 6 | 123.501 | -235.002 | 1.346 | 0.142 |
| 0.02043 | NA | NA | + | NA | NA | -6.53E-03 | 6 | 123.375 | -234.751 | 1.597 | 0.126 |
| 0.0035 | 9.81E-11 | NA | + | NA | NA | NA | 6 | 123.226 | -234.451 | 1.896 | 0.108 |
| 0.00107 | NA | NA | + | NA | NA | NA | 5 | 126.724 | -243.448 | 0 | 0.336 |
| 0.02497 | NA | NA | + | NA | -1.06E-02 | NA | 6 | 127.346 | -242.692 | 0.756 | 0.231 |
| 0.00819 | NA | NA | + | 4.86E-05 | NA | NA | 6 | 126.992 | -241.984 | 1.463 | 0.162 |
| -0.00943 | NA | NA | + | NA | NA | 4.48E-03 | 6 | 126.836 | -241.672 | 1.775 | 0.138 |
| -0.00065 | 1.05E-10 | NA | + | NA | NA | NA | 6 | 126.793 | -241.586 | 1.862 | 0.133 |
| 0.0016 | NA | NA | + | NA | NA | NA | 5 | 125.237 | -240.474 | 0 | 0.359 |
| 0.01097 | NA | NA | + | 5.95E-05 | NA | NA | 6 | 125.58 | -239.16 | 1.314 | 0.186 |
| 0.02086 | NA | NA | + | NA | -8.54E-03 | NA | 6 | 125.543 | -239.085 | 1.388 | 0.179 |
| -0.00053 | 1.27E-10 | NA | + | NA | NA | NA | 6 | 125.319 | -238.638 | 1.835 | 0.143 |
| -0.00166 | NA | NA | + | NA | NA | 1.35E-03 | 6 | 125.247 | -238.493 | I.98 | 0.133 |
| 0.00808 | NA | NA | + | NA | NA | NA | 5 | 122.093 | -234.186 | 0 | 0.36 |
| 0.01964 | NA | NA | + | 6.48E-05 | NA | NA | 6 | 122.436 | -232.871 | 1.314 | 0.187 |
| 0.03008 | NA | NA | + | NA | -9.71E-03 | NA | 6 | 122.422 | -232.845 | 1.341 | 0.184 |
| 0.01338 | NA | NA | + | NA | NA | -2.21E-03 | 6 | 122.117 | -232.235 | 1.951 | 0.136 |
| 0.00753 | 3.25E-11 | NA | + | NA | NA | NA | 6 | 122.103 | -232.206 | 1.979 | 0.134 |
| 0.00167 | NA | NA | + | NA | NA | NA | 5 | 127.148 | -244.296 | 0 | 0.363 |
| 0.02202 | NA | NA | + | NA | -8.90E-03 | NA | 6 | 127.468 | -242.937 | I.36 | 0.184 |
| 0.01249 | NA | NA | + | 5.91E-05 | NA | NA | 6 | 127.468 | -242.937 | I.36 | 0.184 |
| 0.0051 | NA | NA | + | NA | NA | -1.48E-03 | 6 | 127.161 | -242.321 | 1.975 | 0.135 |
| 0.00109 | 3.19E-11 | NA | + | NA | NA | NA | 6 | 127.16 | -242.32 | 1.977 | 0.135 |
| -0.00176 | NA | NA | + | NA | NA | NA | 5 | 120.465 | -230.93 | 0 | 0.365 |
| 0.01807 | NA | NA | + | NA | -8.65E-03 | NA | 6 | 120.756 | -229.513 | 1.417 | 0.18 |
| 0.00735 | NA | NA | + | 5.23E-05 | NA | NA | 6 | 120.708 | -229.417 | 1.513 | 0.171 |
| -0.00955 | NA | NA | + | NA | NA | 3.35E-03 | 6 | 120.528 | -229.056 | 1.874 | 0.143 |
| -0.00345 | 9.37E-11 | NA | + | NA | NA | NA | 6 | 120.519 | -229.039 | 1.891 | 0.142 |
| 0.00142 | NA | NA | + | NA | NA | NA | 5 | 128.949 | -247.898 | 0 | 0.337 |
| 0.02526 | NA | NA | + | NA | -1.06E-02 | NA | 6 | 129.558 | -247.115 | 0.783 | 0.228 |
| 0.01043 | NA | NA | + | 5.53E-05 | NA | NA | 6 | 129.292 | -246.583 | 1.315 | 0.175 |
| -0.00044 | 1.14E-10 | NA | + | NA | NA | NA | 6 | 129.023 | -246.046 | 1.853 | 0.134 |
| -0.00274 | NA | NA | + | NA | NA | 1.74E-03 | 6 | 128.966 | -245.932 | 1.966 | 0.126 |
| 0.00613 | NA | NA | + | NA | NA | NA | 5 | 123.657 | -237.313 | 0 | 0.354 |
| 0.01693 | NA | NA | + | 6.67E-05 | NA | NA | 6 | 124.068 | -236.135 | 1.178 | 0.197 |
| 0.02527 | NA | NA | + | NA | -8.57E-03 | NA | 6 | 123.979 | -235.958 | 1.355 | 0.18 |
| 0.00479 | 8.65E-11 | NA | + | NA | NA | NA | 6 | 123.706 | -235.412 | 1.901 | 0.137 |
| 0.01028 | NA | NA | + | NA | NA | -1.70E-03 | 6 | 123.671 | -235.343 | 1.971 | 0.132 |
| 0.00115 | NA | NA | + | NA | NA | NA | 5 | 121.805 | -233.611 | 0 | 0.357 |
| 0.0138 | NA | NA | + | 7.06E-05 | NA | NA | 6 | 122.246 | -232.493 | 1.118 | 0.204 |
| 0.01939 | NA | NA | + | NA | -8.14E-03 | NA | 6 | 122.075 | -232.149 | 1.462 | 0.172 |
| -0.00013 | 7.02E-11 | NA | + | NA | NA | NA | 6 | 121.832 | -231.664 | 1.947 | 0.135 |
| 0.00114 | NA | NA | + | NA | NA | 7.19E-06 | 6 | 121.805 | -231.611 | 2 | 0.131 |
| 0.00237 | NA | NA | + | NA | NA | NA | 5 | 124.742 | -239.485 | 0 | 0.352 |
| 0.02545 | NA | NA | + | NA | -1.04E-02 | NA | 6 | 125.237 | -238.474 | 1.011 | 0.212 |
| 0.01096 | NA | NA | + | 5.14E-05 | NA | NA | 6 | 125.001 | -238.002 | 1.483 | 0.168 |
| 0.00043 | 1.11E-10 | NA | + | NA | NA | NA | 6 | 124.81 | -237.62 | 1.864 | 0.139 |
| 0.00137 | NA | NA | + | NA | NA | 4.13E-04 | 6 | 124.743 | -237.487 | 1.998 | 0.13 |
| 0.0038 | NA | NA | + | NA | NA | NA | 5 | 126.392 | -242.784 | 0 | 0.359 |
| 0.02707 | NA | NA | + | NA | -1.04E-02 | NA | 6 | 126.896 | -241.791 | 0.992 | 0.218 |
| 0.01053 | NA | NA | + | 3.99E-05 | NA | NA | 6 | 126.551 | -241.102 | 1.681 | 0.155 |
| 0.00278 | 5.70E-11 | NA | + | NA | NA | NA | 6 | 126.42 | -240.839 | 1.944 | 0.136 |
| 0.00231 | NA | NA | + | NA | NA | 6.37E-04 | 6 | 126.394 | -240.788 | 1.996 | 0.132 |
| 0.00166 | NA | NA | + | NA | NA | NA | 5 | 124.714 | -239.429 | 0 | 0.37 |
| 0.01932 | NA | NA | + | NA | -7.71E-03 | NA | 6 | 124.989 | -237.977 | 1.451 | 0.179 |
| 0.01024 | NA | NA | + | 5.03E-05 | NA | NA | 6 | 124.958 | -237.916 | 1.513 | 0.173 |
| 0.00024 | 7.94E-11 | NA | + | NA | NA | NA | 6 | 124.75 | -237.501 | 1.928 | 0.141 |
| -0.00106 | NA | NA | + | NA | NA | 1.18E-03 | 6 | 124.722 | -237.444 | 1.985 | 0.137 |
| 0.00397 | NA | NA | + | NA | NA | NA | 5 | 124.256 | -238.512 | 0 | 0.369 |
| 0.01559 | NA | NA | + | 6.88E-05 | NA | NA | 6 | 124.685 | -237.37 | 1.142 | 0.209 |
| 0.01414 | NA | NA | + | NA | -4.49E-03 | NA | 6 | 124.342 | -236.683 | 1.829 | 0.148 |
| 0.00315 | 4.69E-11 | NA | + | NA | NA | NA | 6 | 124.274 | -236.547 | 1.965 | 0.138 |
| 0.00293 | NA | NA | + | NA | NA | 4.38E-04 | 6 | 124.257 | -236.514 | 1.998 | 0.136 |
| 0.00225 | NA | NA | + | NA | NA | NA | 5 | 121.172 | -232.344 | 0 | 0.372 |
| 0.01127 | NA | NA | + | 5.47E-05 | NA | NA | 6 | 121.447 | -230.894 | I.45 | 0.18 |
| 0.01854 | NA | NA | + | NA | -7.24E-03 | NA | 6 | 121.388 | -230.776 | 1.568 | 0.17 |
| 0.00102 | 7.01E-11 | NA | + | NA | NA | NA | 6 | 121.206 | -230.412 | 1.932 | 0.142 |
| 0.00275 | NA | NA | + | NA | NA | -2.09E-04 | 6 | 121.172 | -230.344 | 2 | 0.137 |
| 0.00166 | NA | NA | + | NA | NA | NA | 5 | 123.684 | -237.367 | 0 | 0.359 |
| 0.02447 | NA | NA | + | NA | -9.84E-03 | NA | 6 | 124.036 | -236.071 | 1.296 | 0.188 |
| 0.01326 | NA | NA | + | 6.30E-05 | NA | NA | 6 | 124.022 | -236.044 | 1.323 | 0.185 |
| 0.00062 | 5.85E-11 | NA | + | NA | NA | NA | 6 | 123.704 | -235.408 | 1.959 | 0.135 |
| 0.00598 | NA | NA | + | NA | NA | -1.87E-03 | 6 | 123.702 | -235.405 | 1.962 | 0.134 |
| 0.00254 | NA | NA | + | NA | NA | NA | 5 | 123.97 | -237.94 | 0 | 0.357 |
| 0.02598 | NA | NA | + | NA | -1.03E-02 | NA | 6 | 124.351 | -236.702 | 1.238 | 0.192 |
| 0.01355 | NA | NA | + | 5.86E-05 | NA | NA | 6 | 124.256 | -236.512 | 1.428 | 0.175 |
| 0.01161 | NA | NA | + | NA | NA | -3.86E-03 | 6 | 124.045 | -236.089 | I.85 | 0.141 |
| 0.00133 | 6.90E-11 | NA | + | NA | NA | NA | 6 | 124.001 | -236.001 | 1.938 | 0.135 |
| 0.0039 | NA | NA | + | NA | NA | NA | 5 | 127.767 | -245.534 | 0 | 0.349 |
| 0.01685 | NA | NA | + | 6.86E-05 | NA | NA | 6 | 128.189 | -244.378 | 1.156 | 0.196 |
| 0.0268 | NA | NA | + | NA | -9.95E-03 | NA | 6 | 128.133 | -244.265 | 1.268 | 0.185 |
| 0.01197 | NA | NA | + | NA | NA | -3.46E-03 | 6 | 127.825 | -243.651 | 1.883 | 0.136 |
| 0.00282 | 6.27E-11 | NA | + | NA | NA | NA | 6 | 127.8 | -243.6 | 1.934 | 0.133 |
| 0.00195 | NA | NA | + | NA | NA | NA | 5 | 124.057 | -238.115 | 0 | 0.337 |
| 0.02857 | NA | NA | + | NA | -1.15E-02 | NA | 6 | 124.589 | -237.178 | 0.937 | 0.211 |
| 0.01426 | NA | NA | + | 6.82E-05 | NA | NA | 6 | 124.468 | -236.937 | 1.178 | 0.187 |
| 0.01183 | NA | NA | + | NA | NA | -4.22E-03 | 6 | 124.146 | -236.292 | 1.823 | 0.136 |
| 0.00065 | 7.35E-11 | NA | + | NA | NA | NA | 6 | 124.092 | -236.184 | 1.931 | 0.128 |
| 0.00061 | NA | NA | + | NA | NA | NA | 5 | 123.614 | -237.228 | 0 | 0.363 |
| 0.02269 | NA | NA | + | NA | -9.85E-03 | NA | 6 | 124.005 | -236.01 | 1.218 | 0.198 |
| 0.00979 | NA | NA | + | 5.04E-05 | NA | NA | 6 | 123.842 | -235.684 | 1.544 | 0.168 |
| -0.00053 | 6.30E-11 | NA | + | NA | NA | NA | 6 | 123.642 | -235.283 | 1.944 | 0.137 |
| 0.00267 | NA | NA | + | NA | NA | -8.76E-04 | 6 | 123.618 | -235.236 | 1.992 | 0.134 |
| 0.0033 | NA | NA | + | NA | NA | NA | 5 | 127.264 | -244.527 | 0 | 0.341 |
| 0.02678 | NA | NA | + | NA | -1.06E-02 | NA | 6 | 127.92 | -243.84 | 0.687 | 0.242 |
| 0.00891 | NA | NA | + | 3.70E-05 | NA | NA | 6 | 127.416 | -242.833 | 1.695 | 0.146 |
| -0.00608 | NA | NA | + | NA | NA | 3.91E-03 | 6 | 127.354 | -242.708 | 1.819 | 0.137 |
| 0.00157 | 1.02E-10 | NA | + | NA | NA | NA | 6 | 127.324 | -242.649 | 1.879 | 0.133 |
| 0.00132 | NA | NA | + | NA | NA | NA | 5 | 121.083 | -232.167 | 0 | 0.337 |
| 0.01586 | NA | NA | + | 8.21E-05 | NA | NA | 6 | 121.666 | -231.331 | 0.835 | 0.222 |
| 0.02338 | NA | NA | + | NA | -9.73E-03 | NA | 6 | 121.445 | -230.89 | 1.277 | 0.178 |
| 0.0106 | NA | NA | + | NA | NA | -3.86E-03 | 6 | 121.155 | -230.31 | 1.857 | 0.133 |
| -0.00025 | 8.89E-11 | NA | + | NA | NA | NA | 6 | 121.13 | -230.26 | 1.907 | 0.13 |
| 0.00298 | NA | NA | + | NA | NA | NA | 5 | 125.674 | -241.347 | 0 | 0.356 |
| 0.0162 | NA | NA | + | 7.25E-05 | NA | NA | 6 | 126.145 | -240.29 | 1.057 | 0.21 |
| 0.0205 | NA | NA | + | NA | -7.69E-03 | NA | 6 | 125.903 | -239.806 | 1.541 | 0.165 |
| 0.00154 | 8.40E-11 | NA | + | NA | NA | NA | 6 | 125.719 | -239.438 | 1.909 | 0.137 |
| 0.00706 | NA | NA | + | NA | NA | -1.76E-03 | 6 | 125.689 | -239.379 | 1.968 | 0.133 |
| 0.00672 | NA | NA | + | NA | NA | NA | 5 | 123.16 | -236.32 | 0 | 0.361 |
| 0.01886 | NA | NA | + | 6.80E-05 | NA | NA | 6 | 123.545 | -235.09 | I.23 | 0.195 |
| 0.02361 | NA | NA | + | NA | -7.60E-03 | NA | 6 | 123.374 | -234.747 | 1.573 | 0.164 |
| 0.00495 | 1.04E-10 | NA | + | NA | NA | NA | 6 | 123.212 | -234.425 | 1.895 | 0.14 |
| 0.01451 | NA | NA | + | NA | NA | -3.17E-03 | 6 | 123.207 | -234.414 | 1.906 | 0.139 |
| 0.00941 | NA | NA | + | NA | NA | NA | 5 | 123.402 | -236.803 | 0 | 0.351 |
| 0.02057 | NA | NA | + | 6.74E-05 | NA | NA | 6 | 123.826 | -235.651 | 1.152 | 0.197 |
| 0.02848 | NA | NA | + | NA | -8.59E-03 | NA | 6 | 123.727 | -235.455 | 1.348 | 0.179 |
| 0.00761 | 1.09E-10 | NA | + | NA | NA | NA | 6 | 123.468 | -234.935 | 1.868 | 0.138 |
| 0.00154 | NA | NA | + | NA | NA | 3.23E-03 | 6 | 123.453 | -234.905 | 1.898 | 0.136 |
| 0.00476 | NA | NA | + | NA | NA | NA | 5 | 127.382 | -244.765 | 0 | 0.344 |
| 0.01809 | NA | NA | + | 7.74E-05 | NA | NA | 6 | 127.965 | -243.931 | 0.834 | 0.227 |
| 0.0232 | NA | NA | + | NA | -8.23E-03 | NA | 6 | 127.674 | -243.347 | 1.418 | 0.169 |
| 0.01032 | NA | NA | + | NA | NA | -2.31E-03 | 6 | 127.411 | -242.821 | 1.943 | 0.13 |
| 0.0038 | 5.85E-11 | NA | + | NA | NA | NA | 6 | 127.409 | -242.818 | 1.947 | 0.13 |
| 0.00598 | NA | NA | + | NA | NA | NA | 5 | 121.568 | -233.136 | 0 | 0.256 |
| 0.02343 | NA | NA | + | 1.02E-04 | NA | NA | 6 | 122.485 | -232.969 | 0.167 | 0.236 |
| 0.04698 | NA | NA | NA | 1.50E-04 | NA | NA | 3 | 118.784 | -231.568 | 1.568 | 0.117 |
| 0.01783 | NA | NA | + | NA | -5.24E-03 | NA | 6 | 121.668 | -231.337 | 1.799 | 0.104 |
| 0.00504 | 5.60E-11 | NA | + | NA | NA | NA | 6 | 121.594 | -231.189 | 1.947 | 0.097 |
| 0.00991 | NA | NA | + | NA | NA | -1.62E-03 | 6 | 121.581 | -231.162 | 1.974 | 0.096 |
| 0.0346 | NA | NA | + | 1.08E-04 | NA | -4.20E-03 | 7 | 122.572 | -231.143 | 1.993 | 0.095 |
| 0.00492 | NA | NA | + | NA | NA | NA | 5 | 120.688 | -231.375 | 0 | 0.325 |
| 0.02288 | NA | NA | + | 9.34E-05 | NA | NA | 6 | 121.383 | -230.767 | 0.609 | 0.24 |
| 0.02969 | NA | NA | + | NA | -1.07E-02 | NA | 6 | 121.065 | -230.13 | 1.245 | 0.175 |
| 0.01529 | NA | NA | + | NA | NA | -4.38E-03 | 6 | 120.78 | -229.56 | 1.815 | 0.131 |
| 0.00304 | 1.16E-10 | NA | + | NA | NA | NA | 6 | 120.76 | -229.52 | 1.856 | 0.129 |
| 0.00298 | NA | NA | + | NA | NA | NA | 5 | 127.992 | -245.985 | 0 | 0.348 |
| 0.02623 | NA | NA | + | NA | -1.04E-02 | NA | 6 | 128.511 | -245.023 | 0.962 | 0.215 |
| 0.01166 | NA | NA | + | 5.25E-05 | NA | NA | 6 | 128.273 | -244.545 | 1.439 | 0.169 |
| 0.00057 | 1.43E-10 | NA | + | NA | NA | NA | 6 | 128.084 | -244.169 | 1.816 | 0.14 |
| 0.00256 | NA | NA | + | NA | NA | 1.76E-04 | 6 | 127.992 | -243.985 | 2 | 0.128 |
| 0.00179 | NA | NA | + | NA | NA | NA | 5 | 120.517 | -231.035 | 0 | 0.355 |
| 0.01402 | NA | NA | + | 7.33E-05 | NA | NA | 6 | 121.007 | -230.013 | 1.021 | 0.213 |
| 0.01768 | NA | NA | + | NA | -7.10E-03 | NA | 6 | 120.722 | -229.444 | 1.591 | 0.16 |
| -0.00001 | 1.07E-10 | NA | + | NA | NA | NA | 6 | 120.58 | -229.16 | 1.874 | 0.139 |
| -0.00208 | NA | NA | + | NA | NA | 1.60E-03 | 6 | 120.53 | -229.06 | 1.975 | 0.132 |
| 0.00527 | NA | NA | + | NA | NA | NA | 5 | 116.666 | -223.331 | 0 | 0.367 |
| 0.01715 | NA | NA | + | 7.04E-05 | NA | NA | 6 | 117.05 | -222.101 | 1.231 | 0.198 |
| 0.02036 | NA | NA | + | NA | -6.74E-03 | NA | 6 | 116.828 | -221.657 | 1.674 | 0.159 |
| 0.0038 | 8.38E-11 | NA | + | NA | NA | NA | 6 | 116.706 | -221.411 | I.92 | 0.141 |
| 0.00691 | NA | NA | + | NA | NA | -6.75E-04 | 6 | 116.668 | -221.335 | 1.996 | 0.135 |
| 0.00136 | NA | NA | + | NA | NA | NA | 5 | 123.22 | -236.439 | 0 | 0.364 |
| 0.02077 | NA | NA | + | NA | -8.49E-03 | NA | 6 | 123.525 | -235.05 | 1.389 | 0.182 |
| 0.01101 | NA | NA | + | 5.59E-05 | NA | NA | 6 | 123.523 | -235.045 | 1.394 | 0.181 |
| 0.00037 | 5.97E-11 | NA | + | NA | NA | NA | 6 | 123.248 | -234.495 | 1.944 | 0.138 |
| 0.00317 | NA | NA | + | NA | NA | -7.73E-04 | 6 | 123.223 | -234.446 | 1.994 | 0.134 |
| 0.00264 | NA | NA | + | NA | NA | NA | 5 | 125.737 | -241.474 | 0 | 0.35 |
| 0.01442 | NA | NA | + | 6.73E-05 | NA | NA | 6 | 126.166 | -240.332 | 1.142 | 0.198 |
| 0.02412 | NA | NA | + | NA | -9.35E-03 | NA | 6 | 126.108 | -240.217 | 1.257 | 0.187 |
| -0.00289 | NA | NA | + | NA | NA | 2.37E-03 | 6 | 125.772 | -239.544 | I.93 | 0.133 |
| 0.00188 | 4.34E-11 | NA | + | NA | NA | NA | 6 | 125.754 | -239.509 | 1.965 | 0.131 |
| 0.00142 | NA | NA | + | NA | NA | NA | 5 | 122.441 | -234.882 | 0 | 0.352 |
| 0.02357 | NA | NA | + | NA | -9.69E-03 | NA | 6 | 122.863 | -233.726 | 1.157 | 0.198 |
| 0.01064 | NA | NA | + | 6.07E-05 | NA | NA | 6 | 122.799 | -233.599 | 1.284 | 0.185 |
| 0.00031 | 6.57E-11 | NA | + | NA | NA | NA | 6 | 122.471 | -232.941 | 1.941 | 0.134 |
| -0.00185 | NA | NA | + | NA | NA | 1.38E-03 | 6 | 122.452 | -232.903 | 1.979 | 0.131 |
| 0.00174 | NA | NA | + | NA | NA | NA | 5 | 124.342 | -238.685 | 0 | 0.344 |
| 0.01626 | NA | NA | + | 7.96E-05 | NA | NA | 6 | 124.905 | -237.81 | 0.875 | 0.222 |
| 0.02143 | NA | NA | + | NA | -8.49E-03 | NA | 6 | 124.639 | -237.278 | 1.407 | 0.17 |
| -0.00002 | 1.02E-10 | NA | + | NA | NA | NA | 6 | 124.401 | -236.802 | 1.883 | 0.134 |
| 0.00715 | NA | NA | + | NA | NA | -2.31E-03 | 6 | 124.369 | -236.738 | 1.947 | 0.13 |
| 0.00488 | NA | NA | + | NA | NA | NA | 5 | 120.61 | -231.221 | 0 | 0.304 |
| 0.02065 | NA | NA | + | 9.10E-05 | NA | NA | 6 | 121.297 | -230.594 | 0.626 | 0.222 |
| 0.01962 | NA | NA | + | NA | -6.55E-03 | NA | 6 | 120.758 | -229.516 | 1.705 | 0.13 |
| 0.00328 | 8.93E-11 | NA | + | NA | NA | NA | 6 | 120.647 | -229.295 | 1.926 | 0.116 |
| 0.04593 | NA | NA | NA | 1.48E-04 | NA | NA | 3 | 117.645 | -229.29 | 1.931 | 0.116 |
| 0.0065 | NA | NA | + | NA | NA | -6.71E-04 | 6 | 120.613 | -229.225 | 1.995 | 0.112 |
| 0.00107 | NA | NA | + | NA | NA | NA | 5 | 124.421 | -238.841 | 0 | 0.356 |
| 0.02163 | NA | NA | + | NA | -8.88E-03 | NA | 6 | 124.77 | -237.54 | 1.302 | 0.186 |
| 0.01119 | NA | NA | + | 5.85E-05 | NA | NA | 6 | 124.738 | -237.476 | 1.366 | 0.18 |
| -0.00777 | NA | NA | + | NA | NA | 3.78E-03 | 6 | 124.5 | -237 | 1.841 | 0.142 |
| -0.00022 | 7.19E-11 | NA | + | NA | NA | NA | 6 | 124.46 | -236.92 | 1.921 | 0.136 |
| 0.00127 | NA | NA | + | NA | NA | NA | 5 | 121.348 | -232.696 | 0 | 0.318 |
| 0.02656 | NA | NA | + | NA | -1.12E-02 | NA | 6 | 122.118 | -232.236 | 0.46 | 0.253 |
| -0.01539 | NA | NA | + | NA | NA | 7.04E-03 | 6 | 121.614 | -231.228 | 1.468 | 0.153 |
| 0.00885 | NA | NA | + | 4.92E-05 | NA | NA | 6 | 121.611 | -231.221 | 1.474 | 0.152 |
| -0.00054 | 1.09E-10 | NA | + | NA | NA | NA | 6 | 121.416 | -230.831 | 1.865 | 0.125 |
| 0.006 | NA | NA | + | NA | NA | NA | 5 | 127.211 | -244.422 | 0 | 0.354 |
| 0.02926 | NA | NA | + | NA | -1.02E-02 | NA | 6 | 127.706 | -243.412 | 01.I | 0.214 |
| 0.01393 | NA | NA | + | 4.79E-05 | NA | NA | 6 | 127.451 | -242.902 | I.52 | 0.166 |
| 0.00462 | 8.43E-11 | NA | + | NA | NA | NA | 6 | 127.254 | -242.509 | 1.913 | 0.136 |
| 0.00572 | NA | NA | + | NA | NA | 1.23E-04 | 6 | 127.211 | -242.422 | 2 | 0.13 |
| 0.00362 | NA | NA | + | NA | NA | NA | 5 | 123.184 | -236.368 | 0 | 0.362 |
| 0.01596 | NA | NA | + | 7.24E-05 | NA | NA | 6 | 123.65 | -235.3 | 1.068 | 0.212 |
| 0.01779 | NA | NA | + | NA | -6.33E-03 | NA | 6 | 123.342 | -234.685 | 1.684 | 0.156 |
| 0.00285 | 4.35E-11 | NA | + | NA | NA | NA | 6 | 123.204 | -234.407 | 1.961 | 0.136 |
| 0.00017 | NA | NA | + | NA | NA | 1.43E-03 | 6 | 123.196 | -234.391 | 1.977 | 0.135 |
| 0.00272 | NA | NA | + | NA | NA | NA | 5 | 121.104 | -232.207 | 0 | 0.228 |
| 0.02198 | NA | NA | + | 1.03E-04 | NA | NA | 6 | 122.011 | -232.022 | 0.185 | 0.208 |
| 0.02151 | NA | NA | + | NA | -8.22E-03 | NA | 6 | 121.348 | -230.695 | 1.512 | 0.107 |
| 0.05018 | NA | NA | NA | 1.65E-04 | NA | NA | 3 | 118.326 | -230.652 | 1.555 | 0.105 |
| 0.0369 | NA | NA | + | 9.90E-05 | -6.85E-03 | NA | 7 | 122.181 | -230.362 | 1.845 | 0.091 |
| 0.00107 | 9.89E-11 | NA | + | NA | NA | NA | 6 | 121.167 | -230.333 | 1.874 | 0.089 |
| 0.0089 | NA | NA | + | NA | NA | -2.61E-03 | 6 | 121.136 | -230.272 | 1.935 | 0.087 |
| 0.03465 | NA | NA | + | 1.09E-04 | NA | -4.89E-03 | 7 | 122.124 | -230.249 | 1.958 | 0.086 |
| 0.00073 | NA | NA | + | NA | NA | NA | 5 | 117.366 | -224.732 | 0 | 0.367 |
| 0.01227 | NA | NA | + | 6.76E-05 | NA | NA | 6 | 117.723 | -223.445 | 1.287 | 0.193 |
| 0.0177 | NA | NA | + | NA | -7.44E-03 | NA | 6 | 117.558 | -223.116 | 1.616 | 0.164 |
| -0.00066 | 8.09E-11 | NA | + | NA | NA | NA | 6 | 117.405 | -222.811 | 1.921 | 0.14 |
| 0.00298 | NA | NA | + | NA | NA | -9.35E-04 | 6 | 117.37 | -222.741 | 1.991 | 0.136 |
| 0.04472 | NA | NA | NA | 1.47E-04 | NA | NA | 3 | 120.641 | -235.283 | 0 | 0.2 |
| 0.00669 | NA | NA | + | NA | NA | NA | 5 | 122.612 | -235.223 | 0.06 | 0.194 |
| 0.02345 | NA | NA | + | 9.54E-05 | NA | NA | 6 | 123.316 | -234.632 | 0.65 | 0.145 |
| 0.0533 | NA | NA | NA | 1.42E-04 | -4.55E-03 | NA | 4 | 120.715 | -233.43 | 1.853 | 0.079 |
| 0.05552 | NA | NA | NA | 1.54E-04 | NA | -3.78E-03 | 4 | 120.704 | -233.408 | 1.875 | 0.078 |
| 0.01778 | NA | NA | + | NA | -5.01E-03 | NA | 6 | 122.7 | -233.4 | 1.882 | 0.078 |
| 0.0167 | NA | NA | + | NA | NA | -4.09E-03 | 6 | 122.688 | -233.375 | 1.907 | 0.077 |
| 0.04401 | 4.36E-11 | NA | NA | 1.47E-04 | NA | NA | 4 | 120.654 | -233.308 | 1.975 | 0.075 |
| 0.00546 | 7.09E-11 | NA | + | NA | NA | NA | 6 | 122.645 | -233.291 | 1.992 | 0.074 |
| 0.00331 | NA | NA | + | NA | NA | NA | 5 | 129.559 | -249.119 | 0 | 0.349 |
| 0.02455 | NA | NA | + | NA | -9.30E-03 | NA | 6 | 129.988 | -247.976 | 1.143 | 0.197 |
| 0.0125 | NA | NA | + | 5.90E-05 | NA | NA | 6 | 129.932 | -247.864 | 1.255 | 0.186 |
| -0.00443 | NA | NA | + | NA | NA | 3.30E-03 | 6 | 129.624 | -247.248 | 1.871 | 0.137 |
| 0.00242 | 5.37E-11 | NA | + | NA | NA | NA | 6 | 129.586 | -247.171 | 1.948 | 0.132 |
| 0.00353 | NA | NA | + | NA | NA | NA | 5 | 126.392 | -242.784 | 0 | 0.298 |
| 0.01969 | NA | NA | + | 8.95E-05 | NA | NA | 6 | 127.086 | -242.172 | 0.612 | 0.219 |
| 0.02175 | NA | NA | + | NA | -8.04E-03 | NA | 6 | 126.635 | -241.271 | 1.513 | 0.14 |
| 0.04679 | NA | NA | NA | 1.52E-04 | NA | NA | 3 | 123.468 | -240.937 | 1.847 | 0.118 |
| 0.00219 | 7.71E-11 | NA | + | NA | NA | NA | 6 | 126.43 | -240.86 | 1.924 | 0.114 |
| 0.00013 | NA | NA | + | NA | NA | 1.42E-03 | 6 | 126.404 | -240.808 | 1.976 | 0.111 |
| 0.0023 | NA | NA | + | NA | NA | NA | 5 | 124.288 | -238.575 | 0 | 0.35 |
| 0.02466 | NA | NA | + | NA | -9.93E-03 | NA | 6 | 124.72 | -237.441 | 1.135 | 0.198 |
| 0.01243 | NA | NA | + | 6.07E-05 | NA | NA | 6 | 124.642 | -237.284 | 1.291 | 0.184 |
| 0.00005 | 1.31E-10 | NA | + | NA | NA | NA | 6 | 124.367 | -236.734 | 1.841 | 0.139 |
| 0.00237 | NA | NA | + | NA | NA | -3.32E-05 | 6 | 124.288 | -236.575 | 2 | 0.129 |
| 0.00455 | NA | NA | + | NA | NA | NA | 5 | 124.374 | -238.748 | 0 | 0.252 |
| 0.0226 | NA | NA | + | 9.82E-05 | NA | NA | 6 | 125.189 | -238.378 | 0.37 | 0.21 |
| 0.04711 | NA | NA | NA | 1.56E-04 | NA | NA | 3 | 121.755 | -237.511 | 1.237 | 0.136 |
| 0.01899 | NA | NA | + | NA | -6.43E-03 | NA | 6 | 124.523 | -237.047 | 1.701 | 0.108 |
| 0.01496 | NA | NA | + | NA | NA | -4.28E-03 | 6 | 124.464 | -236.928 | I.82 | 0.101 |
| 0.00303 | 9.06E-11 | NA | + | NA | NA | NA | 6 | 124.428 | -236.856 | 1.892 | 0.098 |
| 0.04023 | NA | NA | + | 1.07E-04 | NA | -6.62E-03 | 7 | 125.4 | -236.8 | 1.947 | 0.095 |
| 0.00131 | NA | NA | + | NA | NA | NA | 5 | 124.154 | -238.308 | 0 | 0.337 |
| 0.01447 | NA | NA | + | 7.78E-05 | NA | NA | 6 | 124.745 | -237.491 | 0.817 | 0.224 |
| 0.0222 | NA | NA | + | NA | -9.22E-03 | NA | 6 | 124.532 | -237.064 | 1.245 | 0.181 |
| -0.00059 | 1.16E-10 | NA | + | NA | NA | NA | 6 | 124.235 | -236.469 | 1.839 | 0.134 |
| 0.00027 | NA | NA | + | NA | NA | 4.34E-04 | 6 | 124.155 | -236.31 | 1.998 | 0.124 |
| 0.00256 | NA | NA | + | NA | NA | NA | 5 | 123.406 | -236.812 | 0 | 0.35 |
| 0.02441 | NA | NA | + | NA | -9.79E-03 | NA | 6 | 123.781 | -235.561 | 1.251 | 0.187 |
| 0.01321 | NA | NA | + | 5.72E-05 | NA | NA | 6 | 123.687 | -235.374 | 1.438 | 0.171 |
| 0.01825 | NA | NA | + | NA | NA | -6.54E-03 | 6 | 123.613 | -235.226 | 1.586 | 0.158 |
| 0.00124 | 7.78E-11 | NA | + | NA | NA | NA | 6 | 123.44 | -234.88 | 1.932 | 0.133 |
| 0.00403 | NA | NA | + | NA | NA | NA | 5 | 126.34 | -242.68 | 0 | 0.364 |
| 0.01428 | NA | NA | + | 5.94E-05 | NA | NA | 6 | 126.67 | -241.34 | 1.341 | 0.186 |
| 0.02227 | NA | NA | + | NA | -8.10E-03 | NA | 6 | 126.613 | -241.226 | 1.454 | 0.176 |
| 0.00254 | 8.52E-11 | NA | + | NA | NA | NA | 6 | 126.38 | -240.76 | I.92 | 0.139 |
| 0.00395 | NA | NA | + | NA | NA | 3.06E-05 | 6 | 126.34 | -240.68 | 2 | 0.134 |
| 0.00413 | NA | NA | + | NA | NA | NA | 5 | 119.743 | -229.487 | 0 | 0.355 |
| 0.01702 | NA | NA | + | 7.95E-05 | NA | NA | 6 | 120.276 | -228.553 | 0.934 | 0.222 |
| 0.01861 | NA | NA | + | NA | -6.45E-03 | NA | 6 | 119.902 | -227.804 | 1.682 | 0.153 |
| 0.00218 | 1.16E-10 | NA | + | NA | NA | NA | 6 | 119.81 | -227.62 | 1.866 | 0.139 |
| 0.00596 | NA | NA | + | NA | NA | -7.61E-04 | 6 | 119.746 | -227.492 | 1.994 | 0.131 |
| 0.00577 | NA | NA | + | NA | NA | NA | 5 | 124.405 | -238.811 | 0 | 0.354 |
| 0.01798 | NA | NA | + | 6.74E-05 | NA | NA | 6 | 124.792 | -237.583 | 1.228 | 0.192 |
| 0.02751 | NA | NA | + | NA | -9.73E-03 | NA | 6 | 124.777 | -237.554 | 1.257 | 0.189 |
| 0.00465 | 6.52E-11 | NA | + | NA | NA | NA | 6 | 124.442 | -236.885 | 1.926 | 0.135 |
| 0.00762 | NA | NA | + | NA | NA | -7.71E-04 | 6 | 124.408 | -236.817 | 1.994 | 0.131 |
| 0.00683 | NA | NA | + | NA | NA | NA | 5 | 126.131 | -242.263 | 0 | 0.262 |
| 0.04783 | NA | NA | NA | 1.53E-04 | NA | NA | 3 | 123.935 | -241.87 | 0.393 | 0.215 |
| 0.0245 | NA | NA | + | 9.64E-05 | NA | NA | 6 | 126.905 | -241.809 | 0.454 | 0.209 |
| 0.0226 | NA | NA | + | NA | -7.03E-03 | NA | 6 | 126.314 | -240.628 | 1.635 | 0.116 |
| 0.0132 | NA | NA | + | NA | NA | -2.60E-03 | 6 | 126.166 | -240.332 | 1.931 | 0.1 |
| 0.00595 | 5.19E-11 | NA | + | NA | NA | NA | 6 | 126.157 | -240.314 | 1.949 | 0.099 |
| -0.00017 | NA | NA | + | NA | NA | NA | 5 | 118.691 | -227.381 | 0 | 0.352 |
| 0.01312 | NA | NA | + | 7.47E-05 | NA | NA | 6 | 119.168 | -226.337 | 1.044 | 0.209 |
| 0.0195 | NA | NA | + | NA | -8.58E-03 | NA | 6 | 118.979 | -225.959 | 1.422 | 0.173 |
| -0.0018 | 9.33E-11 | NA | + | NA | NA | NA | 6 | 118.742 | -225.484 | 1.898 | 0.136 |
| -0.00111 | NA | NA | + | NA | NA | 3.96E-04 | 6 | 118.691 | -225.383 | 1.998 | 0.13 |
| 0.0082 | NA | NA | + | NA | NA | NA | 5 | 124.247 | -238.494 | 0 | 0.315 |
| 0.02158 | NA | NA | + | 7.61E-05 | NA | NA | 6 | 124.743 | -237.487 | 1.008 | 0.191 |
| 0.02148 | NA | NA | + | NA | -5.93E-03 | NA | 6 | 124.382 | -236.763 | 1.731 | 0.133 |
| 0.05018 | NA | NA | NA | 1.45E-04 | NA | NA | 3 | 121.31 | -236.619 | 1.875 | 0.123 |
| 0.00278 | NA | NA | + | NA | NA | 2.23E-03 | 6 | 124.276 | -236.553 | 1.941 | 0.119 |
| 0.00743 | 4.76E-11 | NA | + | NA | NA | NA | 6 | 124.269 | -236.538 | 1.956 | 0.119 |
| 0.00748 | NA | NA | + | NA | NA | NA | 5 | 122.595 | -235.19 | 0 | 0.304 |
| 0.02042 | NA | NA | + | 7.52E-05 | NA | NA | 6 | 123.09 | -234.179 | 1.011 | 0.184 |
| 0.04671 | NA | NA | NA | 1.35E-04 | NA | NA | 3 | 119.895 | -233.79 | 01.IV | 0.151 |
| 0.02138 | NA | NA | + | NA | -6.21E-03 | NA | 6 | 122.744 | -233.487 | 1.703 | 0.13 |
| 0.006 | 9.07E-11 | NA | + | NA | NA | NA | 6 | 122.642 | -233.283 | 1.907 | 0.117 |
| 0.01213 | NA | NA | + | NA | NA | -1.93E-03 | 6 | 122.612 | -233.224 | 1.966 | 0.114 |
| 0.00347 | NA | NA | + | NA | NA | NA | 5 | 124.316 | -238.633 | 0 | 0.357 |
| 0.02593 | NA | NA | + | NA | -1.00E-02 | NA | 6 | 124.773 | -237.546 | 1.087 | 0.207 |
| 0.01218 | NA | NA | + | 5.17E-05 | NA | NA | 6 | 124.57 | -237.141 | 1.492 | 0.169 |
| 0.00262 | 5.22E-11 | NA | + | NA | NA | NA | 6 | 124.344 | -236.687 | 1.946 | 0.135 |
| 0.00412 | NA | NA | + | NA | NA | -2.77E-04 | 6 | 124.317 | -236.634 | 1.999 | 0.131 |
| 0.00479 | NA | NA | + | NA | NA | NA | 5 | 117.167 | -224.334 | 0 | 0.289 |
| 0.02191 | NA | NA | + | 9.19E-05 | NA | NA | 6 | 117.835 | -223.671 | 0.664 | 0.208 |
| 0.04842 | NA | NA | NA | 1.50E-04 | NA | NA | 3 | 114.599 | -223.198 | 1.137 | 0.164 |
| 0.01671 | NA | NA | + | NA | -5.21E-03 | NA | 6 | 117.256 | -222.512 | 1.822 | 0.116 |
| 0.00279 | 1.17E-10 | NA | + | NA | NA | NA | 6 | 117.241 | -222.482 | 1.853 | 0.115 |
| 0.00998 | NA | NA | + | NA | NA | -2.17E-03 | 6 | 117.189 | -222.378 | 1.957 | 0.109 |
| 0.0029 | NA | NA | + | NA | NA | NA | 5 | 126.384 | -242.769 | 0 | 0.359 |
| 0.02424 | NA | NA | + | NA | -9.44E-03 | NA | 6 | 126.761 | -241.521 | 1.248 | 0.193 |
| 0.01267 | NA | NA | + | 5.51E-05 | NA | NA | 6 | 126.669 | -241.339 | I.43 | 0.176 |
| 0.00136 | 8.98E-11 | NA | + | NA | NA | NA | 6 | 126.433 | -240.867 | 1.902 | 0.139 |
| 0.00022 | NA | NA | + | NA | NA | 1.14E-03 | 6 | 126.392 | -240.784 | 1.985 | 0.133 |
| 0.00064 | NA | NA | + | NA | NA | NA | 5 | 125.649 | -241.297 | 0 | 0.355 |
| 0.02264 | NA | NA | + | NA | -9.57E-03 | NA | 6 | 126.077 | -240.153 | 1.144 | 0.2 |
| 0.00998 | NA | NA | + | 5.38E-05 | NA | NA | 6 | 125.939 | -239.878 | I.42 | 0.175 |
| -0.00063 | 7.41E-11 | NA | + | NA | NA | NA | 6 | 125.693 | -239.385 | 1.912 | 0.136 |
| -0.004 | NA | NA | + | NA | NA | 2.00E-03 | 6 | 125.671 | -239.343 | 1.955 | 0.134 |
| 0.0064 | NA | NA | + | NA | NA | NA | 5 | 124.646 | -239.292 | 0 | 0.296 |
| 0.0225 | NA | NA | + | 8.69E-05 | NA | NA | 6 | 125.286 | -238.571 | 0.721 | 0.207 |
| 0.04785 | NA | NA | NA | 1.46E-04 | NA | NA | 3 | 121.916 | -237.832 | I.46 | 0.143 |
| 0.02022 | NA | NA | + | NA | -6.14E-03 | NA | 6 | 124.784 | -237.568 | 1.724 | 0.125 |
| 0.01514 | NA | NA | + | NA | NA | -3.71E-03 | 6 | 124.714 | -237.428 | 1.865 | 0.117 |
| 0.00495 | 8.32E-11 | NA | + | NA | NA | NA | 6 | 124.681 | -237.362 | I.93 | 0.113 |
| 0.00301 | NA | NA | + | NA | NA | NA | 5 | 127.645 | -245.291 | 0 | 0.35 |
| 0.01635 | NA | NA | + | 7.29E-05 | NA | NA | 6 | 128.115 | -244.23 | 1.061 | 0.206 |
| 0.02314 | NA | NA | + | NA | -8.80E-03 | NA | 6 | 127.966 | -243.931 | 1.359 | 0.177 |
| 0.00115 | 1.12E-10 | NA | + | NA | NA | NA | 6 | 127.708 | -243.417 | 1.874 | 0.137 |
| 0.00469 | NA | NA | + | NA | NA | -7.16E-04 | 6 | 127.648 | -243.296 | 1.994 | 0.129 |
| 0.00197 | NA | NA | + | NA | NA | NA | 5 | 119.204 | -228.409 | 0 | 0.351 |
| 0.02532 | NA | NA | + | NA | -1.02E-02 | NA | 6 | 119.605 | -227.21 | 1.199 | 0.193 |
| 0.01388 | NA | NA | + | 6.68E-05 | NA | NA | 6 | 119.585 | -227.171 | 1.238 | 0.189 |
| 0.00012 | 1.01E-10 | NA | + | NA | NA | NA | 6 | 119.258 | -226.517 | 1.892 | 0.136 |
| -0.00034 | NA | NA | + | NA | NA | 9.84E-04 | 6 | 119.21 | -226.419 | I.99 | 0.13 |
| 0.00462 | NA | NA | + | NA | NA | NA | 5 | 123.151 | -236.303 | 0 | 0.35 |
| 0.01799 | NA | NA | + | 7.38E-05 | NA | NA | 6 | 123.591 | -235.181 | 1.122 | 0.2 |
| 0.02598 | NA | NA | + | NA | -9.53E-03 | NA | 6 | 123.48 | -234.961 | 1.342 | 0.179 |
| 0.0122 | NA | NA | + | NA | NA | -3.14E-03 | 6 | 123.205 | -234.409 | 1.893 | 0.136 |
| 0.00304 | 8.81E-11 | NA | + | NA | NA | NA | 6 | 123.194 | -234.388 | 1.914 | 0.135 |
| 0.00228 | NA | NA | + | NA | NA | NA | 5 | 124.739 | -239.479 | 0 | 0.319 |
| 0.01786 | NA | NA | + | 8.35E-05 | NA | NA | 6 | 125.336 | -238.672 | 0.807 | 0.213 |
| 0.02808 | NA | NA | + | NA | -1.13E-02 | NA | 6 | 125.211 | -238.422 | 1.057 | 0.188 |
| 0.01942 | NA | NA | + | NA | NA | -7.09E-03 | 6 | 124.975 | -237.95 | 1.529 | 0.149 |
| 0 | 1.39E-10 | NA | + | NA | NA | NA | 6 | 124.844 | -237.688 | 1.791 | 0.13 |
| 0.00261 | NA | NA | + | NA | NA | NA | 5 | 125.305 | -240.611 | 0 | 0.369 |
| 0.02166 | NA | NA | + | NA | -8.52E-03 | NA | 6 | 125.635 | -239.27 | 1.341 | 0.189 |
| 0.0086 | NA | NA | + | 3.85E-05 | NA | NA | 6 | 125.45 | -238.9 | I.71 | 0.157 |
| -0.00497 | NA | NA | + | NA | NA | 3.16E-03 | 6 | 125.365 | -238.729 | 1.881 | 0.144 |
| 0.00136 | 7.26E-11 | NA | + | NA | NA | NA | 6 | 125.341 | -238.681 | 1.929 | 0.141 |
| -0.00259 | NA | NA | + | NA | NA | NA | 5 | 129.531 | -249.062 | 0 | 0.339 |
| 0.02355 | NA | NA | + | NA | -1.15E-02 | NA | 6 | 130.215 | -248.429 | 0.633 | 0.247 |
| 0.00502 | NA | NA | + | 4.32E-05 | NA | NA | 6 | 129.736 | -247.473 | 1.589 | 0.153 |
| -0.00433 | 9.71E-11 | NA | + | NA | NA | NA | 6 | 129.59 | -247.181 | 1.881 | 0.132 |
| -0.00711 | NA | NA | + | NA | NA | 1.96E-03 | 6 | 129.553 | -247.106 | 1.956 | 0.128 |
| 0.00484 | NA | NA | + | NA | NA | NA | 5 | 126.496 | -242.992 | 0 | 0.37 |
| 0.01398 | NA | NA | + | 5.29E-05 | NA | NA | 6 | 126.771 | -241.541 | 1.451 | 0.179 |
| 0.02211 | NA | NA | + | NA | -7.59E-03 | NA | 6 | 126.743 | -241.486 | 1.505 | 0.174 |
| 0.00347 | 8.30E-11 | NA | + | NA | NA | NA | 6 | 126.536 | -241.072 | I.92 | 0.142 |
| 0.00537 | NA | NA | + | NA | NA | -2.30E-04 | 6 | 126.496 | -240.992 | 1.999 | 0.136 |
| 0.00015 | NA | NA | + | NA | NA | NA | 5 | 122.598 | -235.195 | 0 | 0.365 |
| 0.02116 | NA | NA | + | NA | -9.24E-03 | NA | 6 | 123.007 | -234.014 | 1.182 | 0.202 |
| 0.00552 | NA | NA | + | 3.30E-05 | NA | NA | 6 | 122.707 | -233.415 | 1.781 | 0.15 |
| -0.00769 | NA | NA | + | NA | NA | 3.36E-03 | 6 | 122.663 | -233.326 | 1.869 | 0.144 |
| -0.00097 | 6.38E-11 | NA | + | NA | NA | NA | 6 | 122.627 | -233.254 | 1.941 | 0.138 |
| 0.0049 | NA | NA | + | NA | NA | NA | 5 | 119.011 | -228.023 | 0 | 0.307 |
| 0.01908 | NA | NA | + | 8.15E-05 | NA | NA | 6 | 119.572 | -227.145 | 0.878 | 0.198 |
| 0.0475 | NA | NA | NA | 1.42E-04 | NA | NA | 3 | 116.255 | -226.51 | 1.512 | 0.144 |
| 0.0136 | NA | NA | + | NA | -3.82E-03 | NA | 6 | 119.067 | -226.135 | 1.888 | 0.119 |
| 0.00338 | 8.54E-11 | NA | + | NA | NA | NA | 6 | 119.047 | -226.094 | 1.929 | 0.117 |
| -0.00054 | NA | NA | + | NA | NA | 2.31E-03 | 6 | 119.039 | -226.078 | 1.945 | 0.116 |
| 0.00614 | NA | NA | + | NA | NA | NA | 5 | 128.629 | -247.258 | 0 | 0.349 |
| 0.01936 | NA | NA | + | 7.58E-05 | NA | NA | 6 | 129.192 | -246.384 | 0.874 | 0.225 |
| 0.024 | NA | NA | + | NA | -7.96E-03 | NA | 6 | 128.899 | -245.798 | I.46 | 0.168 |
| 0.00562 | 3.10E-11 | NA | + | NA | NA | NA | 6 | 128.642 | -245.284 | 1.973 | 0.13 |
| 0.00615 | NA | NA | + | NA | NA | -6.90E-06 | 6 | 128.629 | -245.258 | 2 | 0.128 |
| 0.00544 | NA | NA | + | NA | NA | NA | 5 | 119.406 | -228.813 | 0 | 0.31 |
| 0.01899 | NA | NA | + | 7.34E-05 | NA | NA | 6 | 119.809 | -227.619 | 1.194 | 0.17 |
| 0.0452 | NA | NA | NA | 1.36E-04 | NA | NA | 3 | 116.641 | -227.281 | 1.532 | 0.144 |
| 0.0214 | NA | NA | + | NA | NA | -6.67E-03 | 6 | 119.606 | -227.211 | 1.602 | 0.139 |
| 0.01593 | NA | NA | + | NA | -4.61E-03 | NA | 6 | 119.472 | -226.943 | I.87 | 0.122 |
| 0.00478 | 3.89E-11 | NA | + | NA | NA | NA | 6 | 119.42 | -226.841 | 1.972 | 0.115 |
| 0.00008 | NA | NA | + | NA | NA | NA | 5 | 124.882 | -239.764 | 0 | 0.342 |
| 0.02303 | NA | NA | + | NA | -9.98E-03 | NA | 6 | 125.353 | -238.706 | 1.058 | 0.202 |
| 0.01057 | NA | NA | + | 6.47E-05 | NA | NA | 6 | 125.311 | -238.623 | 1.141 | 0.193 |
| -0.00171 | 1.01E-10 | NA | + | NA | NA | NA | 6 | 124.949 | -237.898 | 1.866 | 0.135 |
| -0.00432 | NA | NA | + | NA | NA | 1.91E-03 | 6 | 124.901 | -237.802 | 1.961 | 0.128 |
| 0.00561 | NA | NA | + | NA | NA | NA | 5 | 124.817 | -239.633 | 0 | 0.295 |
| 0.02078 | NA | NA | + | 8.28E-05 | NA | NA | 6 | 125.359 | -238.717 | 0.916 | 0.187 |
| 0.0465 | NA | NA | NA | 1.49E-04 | NA | NA | 3 | 122.101 | -238.201 | 1.432 | 0.144 |
| 0.02511 | NA | NA | + | NA | -8.68E-03 | NA | 6 | 125.094 | -238.187 | 1.446 | 0.143 |
| 0.01439 | NA | NA | + | NA | NA | -3.60E-03 | 6 | 124.877 | -237.755 | 1.879 | 0.116 |
| 0.00419 | 8.37E-11 | NA | + | NA | NA | NA | 6 | 124.867 | -237.733 | 01.IX | 0.114 |
| 0.00779 | NA | NA | + | NA | NA | NA | 5 | 117.604 | -225.207 | 0 | 0.368 |
| 0.02102 | NA | NA | + | 7.64E-05 | NA | NA | 6 | 118.06 | -224.12 | 1.087 | 0.214 |
| 0.01807 | NA | NA | + | NA | -4.55E-03 | NA | 6 | 117.671 | -223.342 | 1.866 | 0.145 |
| 0.00673 | 5.93E-11 | NA | + | NA | NA | NA | 6 | 117.628 | -223.257 | I.95 | 0.139 |
| 0.00642 | NA | NA | + | NA | NA | 5.64E-04 | 6 | 117.605 | -223.211 | 1.997 | 0.135 |
| 0.00495 | NA | NA | + | NA | NA | NA | 5 | 123.226 | -236.451 | 0 | 0.362 |
| 0.01581 | NA | NA | + | 6.32E-05 | NA | NA | 6 | 123.581 | -235.162 | 1.289 | 0.19 |
| 0.0245 | NA | NA | + | NA | -8.55E-03 | NA | 6 | 123.514 | -235.028 | 1.423 | 0.178 |
| 0.00403 | 5.31E-11 | NA | + | NA | NA | NA | 6 | 123.245 | -234.49 | 1.961 | 0.136 |
| 0.0046 | NA | NA | + | NA | NA | 1.47E-04 | 6 | 123.226 | -234.451 | 2 | 0.133 |
| 0.00076 | NA | NA | + | NA | NA | NA | 5 | 127.407 | -244.815 | 0 | 0.328 |
| 0.02644 | NA | NA | + | NA | -1.13E-02 | NA | 6 | 128.041 | -244.082 | 0.733 | 0.228 |
| 0.00997 | NA | NA | + | 5.72E-05 | NA | NA | 6 | 127.759 | -243.518 | 1.297 | 0.172 |
| -0.01094 | NA | NA | + | NA | NA | 4.96E-03 | 6 | 127.557 | -243.114 | 1.701 | 0.14 |
| -0.00162 | 1.38E-10 | NA | + | NA | NA | NA | 6 | 127.499 | -242.998 | 1.817 | 0.132 |
| 0.00267 | NA | NA | + | NA | NA | NA | 5 | 123.012 | -236.024 | 0 | 0.335 |
| 0.01774 | NA | NA | + | 8.43E-05 | NA | NA | 6 | 123.625 | -235.25 | 0.774 | 0.227 |
| 0.02568 | NA | NA | + | NA | -1.01E-02 | NA | 6 | 123.405 | -234.81 | 1.213 | 0.183 |
| 0.00074 | 1.08E-10 | NA | + | NA | NA | NA | 6 | 123.076 | -234.152 | 1.871 | 0.131 |
| 0.00395 | NA | NA | + | NA | NA | -5.36E-04 | 6 | 123.013 | -234.027 | 1.997 | 0.123 |
| 0.00027 | NA | NA | + | NA | NA | NA | 5 | 125.535 | -241.071 | 0 | 0.316 |
| 0.02673 | NA | NA | + | NA | -1.18E-02 | NA | 6 | 126.323 | -240.647 | 0.424 | 0.256 |
| 0.00778 | NA | NA | + | 4.86E-05 | NA | NA | 6 | 125.813 | -239.625 | 1.446 | 0.153 |
| -0.0142 | NA | NA | + | NA | NA | 6.09E-03 | 6 | 125.761 | -239.523 | 1.548 | 0.146 |
| -0.00221 | 1.46E-10 | NA | + | NA | NA | NA | 6 | 125.644 | -239.288 | 1.783 | 0.13 |
| 0.0054 | NA | NA | + | NA | NA | NA | 5 | 127.556 | -245.112 | 0 | 0.282 |
| 0.02165 | NA | NA | + | 8.94E-05 | NA | NA | 6 | 128.255 | -244.51 | 0.601 | 0.209 |
| 0.04728 | NA | NA | NA | 1.51E-04 | NA | NA | 3 | 125.021 | -244.042 | 01.VII | 0.165 |
| 0.02335 | NA | NA | + | NA | -8.04E-03 | NA | 6 | 127.799 | -243.598 | 1.514 | 0.132 |
| 0.00426 | 6.92E-11 | NA | + | NA | NA | NA | 6 | 127.592 | -243.184 | 1.928 | 0.107 |
| 0.00892 | NA | NA | + | NA | NA | -1.45E-03 | 6 | 127.567 | -243.134 | 1.978 | 0.105 |
| 0.00355 | NA | NA | + | NA | NA | NA | 5 | 125.105 | -240.21 | 0 | 0.375 |
| 0.01305 | NA | NA | + | 5.30E-05 | NA | NA | 6 | 125.368 | -238.736 | 1.475 | 0.179 |
| 0.01484 | NA | NA | + | NA | -4.98E-03 | NA | 6 | 125.212 | -238.424 | 1.786 | 0.154 |
| -0.00538 | NA | NA | + | NA | NA | 3.85E-03 | 6 | 125.195 | -238.39 | I.82 | 0.151 |
| 0.00255 | 5.51E-11 | NA | + | NA | NA | NA | 6 | 125.125 | -238.25 | I.96 | 0.141 |
| 0.00239 | NA | NA | + | NA | NA | NA | 5 | 127.965 | -245.931 | 0 | 0.351 |
| 0.02528 | NA | NA | + | NA | -1.01E-02 | NA | 6 | 128.396 | -244.791 | 1.139 | 0.199 |
| 0.01314 | NA | NA | + | 6.13E-05 | NA | NA | 6 | 128.32 | -244.64 | 1.291 | 0.184 |
| 0.00078 | 9.72E-11 | NA | + | NA | NA | NA | 6 | 128.024 | -244.047 | 1.884 | 0.137 |
| 0.00356 | NA | NA | + | NA | NA | -4.97E-04 | 6 | 127.967 | -243.933 | 1.997 | 0.129 |
| 0.00594 | NA | NA | + | NA | NA | NA | 5 | 127.663 | -245.326 | 0 | 0.348 |
| 0.02931 | NA | NA | + | NA | -1.03E-02 | NA | 6 | 128.086 | -244.172 | 1.155 | 0.195 |
| 0.01767 | NA | NA | + | 6.62E-05 | NA | NA | 6 | 128.053 | -244.106 | I.22 | 0.189 |
| 0.01483 | NA | NA | + | NA | NA | -3.64E-03 | 6 | 127.729 | -243.458 | 1.868 | 0.137 |
| 0.00491 | 6.30E-11 | NA | + | NA | NA | NA | 6 | 127.695 | -243.39 | 1.936 | 0.132 |
| 0.00248 | NA | NA | + | NA | NA | NA | 5 | 122.401 | -234.802 | 0 | 0.308 |
| 0.01626 | NA | NA | + | 7.36E-05 | NA | NA | 6 | 122.863 | -233.726 | 1.076 | 0.18 |
| 0.01961 | NA | NA | + | NA | -7.67E-03 | NA | 6 | 122.638 | -233.276 | 1.526 | 0.144 |
| 0.04526 | NA | NA | NA | 1.43E-04 | NA | NA | 3 | 119.557 | -233.115 | 1.687 | 0.132 |
| 0.00073 | 9.84E-11 | NA | + | NA | NA | NA | 6 | 122.452 | -232.903 | 1.899 | 0.119 |
| 0.00845 | NA | NA | + | NA | NA | -2.55E-03 | 6 | 122.432 | -232.864 | 1.938 | 0.117 |
| 0.00424 | NA | NA | + | NA | NA | NA | 5 | 126.607 | -243.214 | 0 | 0.355 |
| 0.01797 | NA | NA | + | 7.35E-05 | NA | NA | 6 | 127.083 | -242.165 | 1.049 | 0.21 |
| 0.02181 | NA | NA | + | NA | -7.67E-03 | NA | 6 | 126.843 | -241.686 | 1.528 | 0.165 |
| 0.00292 | 7.48E-11 | NA | + | NA | NA | NA | 6 | 126.649 | -241.298 | 1.916 | 0.136 |
| 0.00911 | NA | NA | + | NA | NA | -2.08E-03 | 6 | 126.628 | -241.256 | 1.958 | 0.133 |
| 0.0067 | NA | NA | + | NA | NA | NA | 5 | 123.044 | -236.088 | 0 | 0.316 |
| 0.01994 | NA | NA | + | 7.20E-05 | NA | NA | 6 | 123.46 | -234.92 | 1.168 | 0.176 |
| 0.04785 | NA | NA | NA | 1.37E-04 | NA | NA | 3 | 120.239 | -234.479 | 1.609 | 0.141 |
| 0.01929 | NA | NA | + | NA | -5.54E-03 | NA | 6 | 123.151 | -234.301 | 1.787 | 0.129 |
| 0.00551 | 6.98E-11 | NA | + | NA | NA | NA | 6 | 123.071 | -234.141 | 1.947 | 0.12 |
| 0.00854 | NA | NA | + | NA | NA | -7.78E-04 | 6 | 123.047 | -234.094 | 1.994 | 0.117 |
| 0.00007 | NA | NA | + | NA | NA | NA | 5 | 122.991 | -235.983 | 0 | 0.333 |
| 0.027 | NA | NA | + | NA | -1.19E-02 | NA | 6 | 123.639 | -235.277 | 0.706 | 0.234 |
| 0.0109 | NA | NA | + | 6.14E-05 | NA | NA | 6 | 123.359 | -234.718 | 1.264 | 0.177 |
| -0.00177 | 1.10E-10 | NA | + | NA | NA | NA | 6 | 123.069 | -234.138 | 1.845 | 0.132 |
| 0.00474 | NA | NA | + | NA | NA | -1.95E-03 | 6 | 123.011 | -234.022 | 1.961 | 0.125 |
| 0.00318 | NA | NA | + | NA | NA | NA | 5 | 127.001 | -244.003 | 0 | 0.35 |
| 0.01585 | NA | NA | + | 6.87E-05 | NA | NA | 6 | 127.425 | -242.85 | 1.153 | 0.197 |
| 0.02404 | NA | NA | + | NA | -9.18E-03 | NA | 6 | 127.339 | -242.678 | 1.325 | 0.18 |
| 0.01177 | NA | NA | + | NA | NA | -3.63E-03 | 6 | 127.068 | -242.135 | 1.868 | 0.138 |
| 0.00142 | 1.04E-10 | NA | + | NA | NA | NA | 6 | 127.052 | -242.105 | 1.898 | 0.135 |
| 0.00278 | NA | NA | + | NA | NA | NA | 5 | 131.118 | -252.235 | 0 | 0.352 |
| 0.02493 | NA | NA | + | NA | -9.94E-03 | NA | 6 | 131.62 | -251.24 | 0.996 | 0.214 |
| 0.01085 | NA | NA | + | 4.93E-05 | NA | NA | 6 | 131.389 | -250.778 | 1.457 | 0.17 |
| -0.00207 | NA | NA | + | NA | NA | 2.02E-03 | 6 | 131.143 | -250.286 | 1.949 | 0.133 |
| 0.00211 | 4.15E-11 | NA | + | NA | NA | NA | 6 | 131.137 | -250.275 | 1.961 | 0.132 |
| 0.00382 | NA | NA | + | NA | NA | NA | 5 | 123.39 | -236.779 | 0 | 0.285 |
| 0.01855 | NA | NA | + | 7.78E-05 | NA | NA | 6 | 123.892 | -235.784 | 0.995 | 0.173 |
| 0.02697 | NA | NA | + | NA | -1.03E-02 | NA | 6 | 123.79 | -235.58 | 1.199 | 0.157 |
| 0.04582 | NA | NA | NA | 1.47E-04 | NA | NA | 3 | 120.679 | -235.359 | I.42 | 0.14 |
| 0.02144 | NA | NA | + | NA | NA | -7.28E-03 | 6 | 123.636 | -235.272 | 1.507 | 0.134 |
| 0.00225 | 9.14E-11 | NA | + | NA | NA | NA | 6 | 123.438 | -234.875 | 1.904 | 0.11 |
| -0.0021 | NA | NA | + | NA | NA | NA | 5 | 121.4 | -232.799 | 0 | 0.35 |
| 0.01079 | NA | NA | + | 7.12E-05 | NA | NA | 6 | 121.85 | -231.701 | 1.099 | 0.202 |
| 0.01983 | NA | NA | + | NA | -9.37E-03 | NA | 6 | 121.74 | -231.48 | 1.319 | 0.181 |
| -0.0039 | 1.04E-10 | NA | + | NA | NA | NA | 6 | 121.455 | -230.91 | 1.889 | 0.136 |
| 0.00243 | NA | NA | + | NA | NA | -1.94E-03 | 6 | 121.419 | -230.837 | 1.962 | 0.131 |
| 0.00387 | NA | NA | + | NA | NA | NA | 5 | 124.844 | -239.689 | 0 | 0.364 |
| 0.01434 | NA | NA | + | 6.04E-05 | NA | NA | 6 | 125.174 | -238.349 | I.34 | 0.186 |
| 0.02191 | NA | NA | + | NA | -7.97E-03 | NA | 6 | 125.091 | -238.183 | 1.506 | 0.172 |
| 0.00233 | 8.97E-11 | NA | + | NA | NA | NA | 6 | 124.884 | -237.768 | I.92 | 0.139 |
| 0.00972 | NA | NA | + | NA | NA | -2.48E-03 | 6 | 124.874 | -237.749 | I.94 | 0.138 |
| 0.00486 | NA | NA | + | NA | NA | NA | 5 | 120.815 | -231.63 | 0 | 0.354 |
| 0.01829 | NA | NA | + | 7.75E-05 | NA | NA | 6 | 121.307 | -230.613 | 1.017 | 0.213 |
| 0.02204 | NA | NA | + | NA | -7.67E-03 | NA | 6 | 121.032 | -230.064 | 1.566 | 0.162 |
| 0.003 | 1.12E-10 | NA | + | NA | NA | NA | 6 | 120.881 | -229.762 | 1.867 | 0.139 |
| 0.00837 | NA | NA | + | NA | NA | -1.43E-03 | 6 | 120.825 | -229.65 | I.98 | 0.132 |
| 0.0029 | NA | NA | + | NA | NA | NA | 5 | 121.274 | -232.547 | 0 | 0.36 |
| 0.01388 | NA | NA | + | 6.51E-05 | NA | NA | 6 | 121.66 | -231.321 | 1.227 | 0.195 |
| 0.02143 | NA | NA | + | NA | -8.22E-03 | NA | 6 | 121.546 | -231.092 | 1.455 | 0.174 |
| 0.00143 | 8.18E-11 | NA | + | NA | NA | NA | 6 | 121.313 | -230.626 | 1.921 | 0.138 |
| 0.00655 | NA | NA | + | NA | NA | -1.53E-03 | 6 | 121.285 | -230.569 | 1.978 | 0.134 |
| 0.00336 | NA | NA | + | NA | NA | NA | 5 | 121.721 | -233.442 | 0 | 0.36 |
| 0.01876 | NA | NA | + | 8.04E-05 | NA | NA | 6 | 122.229 | -232.457 | 0.985 | 0.22 |
| 0.01509 | NA | NA | + | NA | -5.21E-03 | NA | 6 | 121.809 | -231.619 | 1.823 | 0.145 |
| 0.01158 | NA | NA | + | NA | NA | -3.44E-03 | 6 | 121.779 | -231.557 | 1.885 | 0.14 |
| 0.00245 | 5.30E-11 | NA | + | NA | NA | NA | 6 | 121.745 | -231.49 | 1.952 | 0.136 |
| -0.00014 | NA | NA | + | NA | NA | NA | 5 | 125.25 | -240.5 | 0 | 0.343 |
| 0.01186 | NA | NA | + | 6.86E-05 | NA | NA | 6 | 125.709 | -239.417 | 1.082 | 0.2 |
| 0.02253 | NA | NA | + | NA | -9.97E-03 | NA | 6 | 125.682 | -239.364 | 1.135 | 0.195 |
| -0.00118 | 6.08E-11 | NA | + | NA | NA | NA | 6 | 125.288 | -238.576 | 1.924 | 0.131 |
| -0.00621 | NA | NA | + | NA | NA | 2.56E-03 | 6 | 125.287 | -238.573 | 1.927 | 0.131 |
| 0.00471 | NA | NA | + | NA | NA | NA | 5 | 124.984 | -239.968 | 0 | 0.291 |
| 0.02073 | NA | NA | + | 8.66E-05 | NA | NA | 6 | 125.617 | -239.234 | 0.734 | 0.201 |
| 0.02284 | NA | NA | + | NA | NA | -7.60E-03 | 6 | 125.264 | -238.527 | I.44 | 0.141 |
| 0.02414 | NA | NA | + | NA | -8.53E-03 | NA | 6 | 125.256 | -238.512 | 1.456 | 0.14 |
| 0.04595 | NA | NA | + | 9.83E-05 | NA | -9.67E-03 | 7 | 126.062 | -238.124 | 1.843 | 0.116 |
| 0.00343 | 7.42E-11 | NA | + | NA | NA | NA | 6 | 125.021 | -238.041 | 1.926 | 0.111 |
| 0.00095 | NA | NA | + | NA | NA | NA | 5 | 119.745 | -229.491 | 0 | 0.354 |
| 0.01188 | NA | NA | + | 6.51E-05 | NA | NA | 6 | 120.128 | -228.255 | 1.235 | 0.191 |
| 0.02197 | NA | NA | + | NA | -9.19E-03 | NA | 6 | 120.084 | -228.168 | 1.322 | 0.183 |
| -0.00057 | 9.15E-11 | NA | + | NA | NA | NA | 6 | 119.788 | -227.575 | 1.916 | 0.136 |
| 0.00763 | NA | NA | + | NA | NA | -2.86E-03 | 6 | 119.785 | -227.57 | 1.921 | 0.136 |
| -0.00024 | NA | NA | + | NA | NA | NA | 5 | 122.738 | -235.477 | 0 | 0.324 |
| 0.01632 | NA | NA | + | 9.01E-05 | NA | NA | 6 | 123.459 | -234.917 | 0.559 | 0.245 |
| 0.02335 | NA | NA | + | NA | -1.02E-02 | NA | 6 | 123.129 | -234.257 | 1.219 | 0.176 |
| -0.00254 | 1.37E-10 | NA | + | NA | NA | NA | 6 | 122.846 | -233.691 | 1.785 | 0.133 |
| 0.004 | NA | NA | + | NA | NA | -1.82E-03 | 6 | 122.754 | -233.509 | 1.968 | 0.121 |
| 0.00007 | NA | NA | + | NA | NA | NA | 5 | 123.448 | -236.895 | 0 | 0.3 |
| 0.01411 | NA | NA | + | 7.62E-05 | NA | NA | 6 | 123.957 | -235.914 | 0.981 | 0.183 |
| 0.0253 | NA | NA | + | NA | -1.10E-02 | NA | 6 | 123.898 | -235.796 | 1.099 | 0.173 |
| 0.04687 | NA | NA | NA | 1.47E-04 | NA | NA | 3 | 120.495 | -234.99 | 1.905 | 0.116 |
| -0.00131 | 8.24E-11 | NA | + | NA | NA | NA | 6 | 123.493 | -234.986 | 1.909 | 0.115 |
| 0.00542 | NA | NA | + | NA | NA | -2.26E-03 | 6 | 123.474 | -234.949 | 1.946 | 0.113 |
| 0.0036 | NA | NA | + | NA | NA | NA | 5 | 126.232 | -242.463 | 0 | 0.362 |
| 0.01452 | NA | NA | + | 6.45E-05 | NA | NA | 6 | 126.626 | -241.252 | 1.212 | 0.198 |
| 0.02084 | NA | NA | + | NA | -7.54E-03 | NA | 6 | 126.476 | -240.951 | 1.512 | 0.17 |
| 0.00229 | 7.68E-11 | NA | + | NA | NA | NA | 6 | 126.261 | -240.522 | 1.942 | 0.137 |
| 0.00356 | NA | NA | + | NA | NA | 1.81E-05 | 6 | 126.232 | -240.463 | 2 | 0.133 |
| 0.00186 | NA | NA | + | NA | NA | NA | 5 | 113.951 | -217.903 | 0 | 0.308 |
| 0.01864 | NA | NA | + | 8.96E-05 | NA | NA | 6 | 114.518 | -217.037 | 0.866 | 0.199 |
| 0.04662 | NA | NA | NA | 1.53E-04 | NA | NA | 3 | 111.08 | -216.161 | 1.742 | 0.129 |
| 0.01561 | NA | NA | + | NA | -5.94E-03 | NA | 6 | 114.06 | -216.12 | 1.783 | 0.126 |
| 0.01176 | NA | NA | + | NA | NA | -4.19E-03 | 6 | 114.029 | -216.059 | 1.844 | 0.122 |
| 0.0008 | 5.97E-11 | NA | + | NA | NA | NA | 6 | 113.974 | -215.948 | 1.954 | 0.116 |
| 0.00456 | NA | NA | + | NA | NA | NA | 5 | 127.635 | -245.271 | 0 | 0.375 |
| 0.02241 | NA | NA | + | NA | -7.95E-03 | NA | 6 | 127.9 | -243.8 | I.47 | 0.18 |
| 0.01164 | NA | NA | + | 4.14E-05 | NA | NA | 6 | 127.796 | -243.593 | 1.678 | 0.162 |
| -0.00089 | NA | NA | + | NA | NA | 2.34E-03 | 6 | 127.668 | -243.336 | 1.934 | 0.143 |
| 0.00385 | 3.97E-11 | NA | + | NA | NA | NA | 6 | 127.649 | -243.297 | 1.973 | 0.14 |
| 0.00327 | NA | NA | + | NA | NA | NA | 5 | 126.676 | -243.351 | 0 | 0.35 |
| 0.01584 | NA | NA | + | 7.60E-05 | NA | NA | 6 | 127.193 | -242.386 | 0.965 | 0.216 |
| 0.02127 | NA | NA | + | NA | -7.98E-03 | NA | 6 | 126.939 | -241.878 | 1.474 | 0.168 |
| 0.00176 | 8.75E-11 | NA | + | NA | NA | NA | 6 | 126.724 | -241.448 | 1.904 | 0.135 |
| 0.00755 | NA | NA | + | NA | NA | -1.79E-03 | 6 | 126.692 | -241.383 | 1.968 | 0.131 |
| -0.00306 | NA | NA | + | NA | NA | NA | 5 | 123.639 | -237.279 | 0 | 0.23 |
| 0.03058 | NA | NA | + | NA | -1.46E-02 | NA | 6 | 124.553 | -237.106 | 0.173 | 0.211 |
| 0.01257 | NA | NA | + | 8.48E-05 | NA | NA | 6 | 124.299 | -236.598 | 0.681 | 0.164 |
| 0.04047 | NA | NA | + | 7.22E-05 | -1.31E-02 | NA | 7 | 125.027 | -236.054 | 1.225 | 0.125 |
| 0.00758 | NA | NA | + | NA | NA | -4.51E-03 | 6 | 123.738 | -235.476 | 1.803 | 0.093 |
| -0.00481 | 1.08E-10 | NA | + | NA | NA | NA | 6 | 123.729 | -235.458 | 1.821 | 0.093 |
| 0.04072 | NA | NA | + | NA | -1.46E-02 | -4.35E-03 | 7 | 124.645 | -235.291 | 1.988 | 0.085 |
| 0.00264 | NA | NA | + | NA | NA | NA | 5 | 124.627 | -239.254 | 0 | 0.367 |
| 0.02346 | NA | NA | + | NA | -9.32E-03 | NA | 6 | 124.982 | -237.965 | 1.289 | 0.192 |
| 0.01014 | NA | NA | + | 4.70E-05 | NA | NA | 6 | 124.837 | -237.675 | 1.579 | 0.166 |
| 0.00164 | 5.94E-11 | NA | + | NA | NA | NA | 6 | 124.65 | -237.3 | 1.954 | 0.138 |
| -0.00067 | NA | NA | + | NA | NA | 1.36E-03 | 6 | 124.637 | -237.274 | I.98 | 0.136 |
| 0.00627 | NA | NA | + | NA | NA | NA | 5 | 120.908 | -231.815 | 0 | 0.322 |
| 0.01801 | NA | NA | + | 6.61E-05 | NA | NA | 6 | 121.29 | -230.58 | 1.235 | 0.173 |
| 0.02049 | NA | NA | + | NA | -6.39E-03 | NA | 6 | 121.071 | -230.143 | 1.672 | 0.139 |
| 0.04658 | NA | NA | NA | 1.32E-04 | NA | NA | 3 | 117.97 | -229.94 | 1.875 | 0.126 |
| 0.00533 | 5.50E-11 | NA | + | NA | NA | NA | 6 | 120.93 | -229.859 | 1.956 | 0.121 |
| 0.00488 | NA | NA | + | NA | NA | 5.77E-04 | 6 | 120.909 | -229.819 | 1.996 | 0.119 |
| 0.04836 | NA | NA | NA | 1.67E-04 | NA | NA | 3 | 115.944 | -225.888 | 0 | 0.28 |
| 0.00407 | NA | NA | + | NA | NA | NA | 5 | 117.584 | -225.168 | 0.72 | 0.196 |
| 0.02409 | NA | NA | + | 1.10E-04 | NA | NA | 6 | 118.493 | -224.986 | 0.902 | 0.179 |
| 0.06405 | NA | NA | NA | 1.54E-04 | -8.31E-03 | NA | 4 | 116.171 | -224.343 | 1.545 | 0.129 |
| 0.06119 | NA | NA | NA | 1.74E-04 | NA | -4.52E-03 | 4 | 116.032 | -224.065 | 1.823 | 0.113 |
| 0.0481 | 1.66E-11 | NA | NA | 1.67E-04 | NA | NA | 4 | 115.946 | -223.892 | 1.996 | 0.103 |
| 0.00183 | NA | NA | + | NA | NA | NA | 5 | 127.648 | -245.297 | 0 | 0.291 |
| 0.01687 | NA | NA | + | 7.61E-05 | NA | NA | 6 | 128.165 | -244.331 | 0.966 | 0.179 |
| 0.02645 | NA | NA | + | NA | -1.07E-02 | NA | 6 | 128.096 | -244.192 | 1.104 | 0.167 |
| 0.04676 | NA | NA | NA | 1.42E-04 | NA | NA | 3 | 124.942 | -243.884 | 1.412 | 0.144 |
| 0.00044 | 7.80E-11 | NA | + | NA | NA | NA | 6 | 127.686 | -243.373 | 1.924 | 0.111 |
| 0.00459 | NA | NA | + | NA | NA | -1.17E-03 | 6 | 127.656 | -243.312 | 1.985 | 0.108 |
| 0.00261 | NA | NA | + | NA | NA | NA | 5 | 124.596 | -239.193 | 0 | 0.342 |
| 0.01585 | NA | NA | + | 7.74E-05 | NA | NA | 6 | 125.176 | -238.352 | 0.84 | 0.224 |
| 0.0199 | NA | NA | + | NA | -7.65E-03 | NA | 6 | 124.874 | -237.749 | 1.444 | 0.166 |
| -0.00601 | NA | NA | + | NA | NA | 3.66E-03 | 6 | 124.672 | -237.345 | 1.848 | 0.136 |
| 0.00121 | 8.08E-11 | NA | + | NA | NA | NA | 6 | 124.647 | -237.295 | 1.898 | 0.132 |
| 0.00165 | NA | NA | + | NA | NA | NA | 5 | 128.965 | -247.93 | 0 | 0.336 |
| 0.0299 | NA | NA | + | NA | -1.24E-02 | NA | 6 | 129.582 | -247.165 | 0.765 | 0.229 |
| 0.01194 | NA | NA | + | 5.65E-05 | NA | NA | 6 | 129.279 | -246.559 | 1.371 | 0.169 |
| 0.01228 | NA | NA | + | NA | NA | -4.63E-03 | 6 | 129.077 | -246.154 | 1.776 | 0.138 |
| 0.00055 | 6.55E-11 | NA | + | NA | NA | NA | 6 | 128.994 | -245.987 | 1.943 | 0.127 |
| 0.00531 | NA | NA | + | NA | NA | NA | 5 | 129.898 | -249.797 | 0 | 0.352 |
| 0.02636 | NA | NA | + | NA | -9.30E-03 | NA | 6 | 130.309 | -248.619 | 1.178 | 0.195 |
| 0.01471 | NA | NA | + | 5.71E-05 | NA | NA | 6 | 130.228 | -248.455 | 1.341 | 0.18 |
| -0.00297 | NA | NA | + | NA | NA | 3.50E-03 | 6 | 129.969 | -247.938 | 1.859 | 0.139 |
| 0.00409 | 7.40E-11 | NA | + | NA | NA | NA | 6 | 129.941 | -247.881 | 1.915 | 0.135 |
| 0.00241 | NA | NA | + | NA | NA | NA | 5 | 124.756 | -239.512 | 0 | 0.348 |
| 0.01373 | NA | NA | + | 6.67E-05 | NA | NA | 6 | 125.19 | -238.381 | 1.131 | 0.198 |
| 0.02417 | NA | NA | + | NA | -9.61E-03 | NA | 6 | 125.163 | -238.326 | 1.186 | 0.192 |
| 0.00128 | 6.72E-11 | NA | + | NA | NA | NA | 6 | 124.789 | -237.577 | 1.935 | 0.132 |
| -0.00166 | NA | NA | + | NA | NA | 1.72E-03 | 6 | 124.772 | -237.544 | 1.968 | 0.13 |
| 0.00297 | NA | NA | + | NA | NA | NA | 5 | 122.118 | -234.235 | 0 | 0.363 |
| 0.01466 | NA | NA | + | 6.57E-05 | NA | NA | 6 | 122.48 | -232.961 | 1.274 | 0.192 |
| 0.02108 | NA | NA | + | NA | -7.86E-03 | NA | 6 | 122.342 | -232.683 | 1.552 | 0.167 |
| 0.01037 | NA | NA | + | NA | NA | -3.16E-03 | 6 | 122.164 | -232.328 | 1.907 | 0.14 |
| 0.0015 | 8.18E-11 | NA | + | NA | NA | NA | 6 | 122.148 | -232.297 | 1.938 | 0.138 |
| 0.00452 | NA | NA | + | NA | NA | NA | 5 | 118.865 | -227.73 | 0 | 0.376 |
| 0.01531 | NA | NA | + | 6.40E-05 | NA | NA | 6 | 119.206 | -226.411 | 1.319 | 0.194 |
| 0.01429 | NA | NA | + | NA | -4.32E-03 | NA | 6 | 118.934 | -225.868 | 1.862 | 0.148 |
| 0.00377 | 4.55E-11 | NA | + | NA | NA | NA | 6 | 118.887 | -225.774 | 1.956 | 0.141 |
| 0.00103 | NA | NA | + | NA | NA | 1.46E-03 | 6 | 118.876 | -225.751 | 1.979 | 0.14 |
| 0.00394 | NA | NA | + | NA | NA | NA | 5 | 122.568 | -235.137 | 0 | 0.353 |
| 0.01731 | NA | NA | + | 7.16E-05 | NA | NA | 6 | 123 | -234.001 | 1.136 | 0.2 |
| 0.02561 | NA | NA | + | NA | -9.45E-03 | NA | 6 | 122.895 | -233.789 | 1.348 | 0.18 |
| 0.00271 | 6.99E-11 | NA | + | NA | NA | NA | 6 | 122.602 | -233.204 | 1.933 | 0.134 |
| 0.00863 | NA | NA | + | NA | NA | -1.99E-03 | 6 | 122.587 | -233.174 | 1.962 | 0.132 |
| 0.00318 | NA | NA | + | NA | NA | NA | 5 | 122.006 | -234.011 | 0 | 0.338 |
| 0.02289 | NA | NA | + | NA | -8.91E-03 | NA | 6 | 122.404 | -232.809 | 1.203 | 0.185 |
| 0.01281 | NA | NA | + | 5.98E-05 | NA | NA | 6 | 122.364 | -232.728 | 1.284 | 0.178 |
| -0.01351 | NA | NA | + | NA | NA | 6.86E-03 | 6 | 122.299 | -232.598 | 1.413 | 0.167 |
| 0.00129 | 1.08E-10 | NA | + | NA | NA | NA | 6 | 122.07 | -232.141 | 1.871 | 0.133 |
| 0.00049 | NA | NA | + | NA | NA | NA | 5 | 112.054 | -214.109 | 0 | 0.29 |
| 0.01625 | NA | NA | + | 8.27E-05 | NA | NA | 6 | 112.523 | -213.046 | 1.062 | 0.17 |
| 0.0475 | NA | NA | NA | 1.50E-04 | NA | NA | 3 | 109.518 | -213.036 | 1.073 | 0.169 |
| 0.0228 | NA | NA | + | NA | -9.64E-03 | NA | 6 | 112.336 | -212.671 | 1.437 | 0.141 |
| 0.01175 | NA | NA | + | NA | NA | -4.83E-03 | 6 | 112.15 | -212.3 | 1.809 | 0.117 |
| -0.00144 | 1.06E-10 | NA | + | NA | NA | NA | 6 | 112.102 | -212.205 | 1.904 | 0.112 |
| 0 | NA | NA | + | NA | NA | NA | 5 | 121.046 | -232.092 | 0 | 0.326 |
| 0.01866 | NA | NA | + | 1.00E-04 | NA | NA | 6 | 121.9 | -231.801 | 0.291 | 0.282 |
| 0.01588 | NA | NA | + | NA | -6.87E-03 | NA | 6 | 121.228 | -230.457 | 1.635 | 0.144 |
| -0.00119 | 6.57E-11 | NA | + | NA | NA | NA | 6 | 121.086 | -230.173 | 1.919 | 0.125 |
| -0.00488 | NA | NA | + | NA | NA | 2.08E-03 | 6 | 121.069 | -230.137 | 1.955 | 0.123 |
| 0.00206 | NA | NA | + | NA | NA | NA | 5 | 120.476 | -230.951 | 0 | 0.357 |
| 0.01552 | NA | NA | + | 7.49E-05 | NA | NA | 6 | 120.939 | -229.878 | 1.073 | 0.209 |
| 0.01802 | NA | NA | + | NA | -7.00E-03 | NA | 6 | 120.664 | -229.329 | 1.622 | 0.159 |
| 0.00025 | 1.05E-10 | NA | + | NA | NA | NA | 6 | 120.527 | -229.053 | 1.898 | 0.138 |
| -0.00477 | NA | NA | + | NA | NA | 2.88E-03 | 6 | 120.521 | -229.041 | I.91 | 0.137 |
| 0.00538 | NA | NA | + | NA | NA | NA | 5 | 126.591 | -243.182 | 0 | 0.367 |
| 0.01617 | NA | NA | + | 6.12E-05 | NA | NA | 6 | 126.927 | -241.855 | 1.327 | 0.189 |
| 0.02375 | NA | NA | + | NA | -8.10E-03 | NA | 6 | 126.835 | -241.669 | 1.512 | 0.172 |
| 0.00492 | 2.90E-11 | NA | + | NA | NA | NA | 6 | 126.603 | -241.205 | 1.976 | 0.137 |
| 0.00722 | NA | NA | + | NA | NA | -7.71E-04 | 6 | 126.594 | -241.188 | 1.994 | 0.135 |
| 0.00417 | NA | NA | + | NA | NA | NA | 5 | 117.628 | -225.256 | 0 | 0.369 |
| 0.01562 | NA | NA | + | 6.36E-05 | NA | NA | 6 | 117.959 | -223.918 | 1.338 | 0.189 |
| 0.02108 | NA | NA | + | NA | -7.40E-03 | NA | 6 | 117.821 | -223.643 | 1.613 | 0.165 |
| 0.00326 | 4.90E-11 | NA | + | NA | NA | NA | 6 | 117.646 | -223.291 | 1.965 | 0.138 |
| 0.00028 | NA | NA | + | NA | NA | 1.67E-03 | 6 | 117.643 | -223.287 | 1.969 | 0.138 |
| 0.00475 | NA | NA | + | NA | NA | NA | 5 | 126.392 | -242.784 | 0 | 0.295 |
| 0.01887 | NA | NA | + | 8.03E-05 | NA | NA | 6 | 126.959 | -241.919 | 0.865 | 0.192 |
| 0.0453 | NA | NA | NA | 1.43E-04 | NA | NA | 3 | 123.799 | -241.597 | 1.187 | 0.163 |
| 0.01984 | NA | NA | + | NA | -6.80E-03 | NA | 6 | 126.568 | -241.135 | 1.649 | 0.13 |
| 0.00406 | 4.05E-11 | NA | + | NA | NA | NA | 6 | 126.411 | -240.821 | 1.963 | 0.111 |
| 0.00727 | NA | NA | + | NA | NA | -1.05E-03 | 6 | 126.398 | -240.796 | 1.988 | 0.109 |
| 0.00379 | NA | NA | + | NA | NA | NA | 5 | 125.827 | -241.655 | 0 | 0.357 |
| 0.02451 | NA | NA | + | NA | -9.08E-03 | NA | 6 | 126.198 | -240.395 | I.26 | 0.19 |
| 0.01417 | NA | NA | + | 5.88E-05 | NA | NA | 6 | 126.153 | -240.306 | 1.348 | 0.182 |
| 0.00223 | 9.29E-11 | NA | + | NA | NA | NA | 6 | 125.873 | -239.746 | 1.908 | 0.138 |
| 0.00632 | NA | NA | + | NA | NA | -1.07E-03 | 6 | 125.834 | -239.667 | 1.988 | 0.132 |
| 0.04653 | NA | NA | NA | 1.49E-04 | NA | NA | 3 | 121.891 | -237.782 | 0 | 0.199 |
| 0.006 | NA | NA | + | NA | NA | NA | 5 | 123.882 | -237.765 | 0.017 | 0.197 |
| 0.02369 | NA | NA | + | 9.67E-05 | NA | NA | 6 | 124.631 | -237.262 | 0.52 | 0.153 |
| 0.0148 | NA | NA | + | NA | -3.89E-03 | NA | 6 | 123.933 | -235.866 | 1.916 | 0.076 |
| 0.05323 | NA | NA | NA | 1.45E-04 | -3.50E-03 | NA | 4 | 121.932 | -235.865 | 1.917 | 0.076 |
| 0.01257 | NA | NA | + | NA | NA | -2.70E-03 | 6 | 123.918 | -235.837 | 1.945 | 0.075 |
| 0.0532 | NA | NA | NA | 1.53E-04 | NA | -2.35E-03 | 4 | 121.917 | -235.834 | 1.948 | 0.075 |
| 0.00528 | 4.33E-11 | NA | + | NA | NA | NA | 6 | 123.903 | -235.805 | 1.976 | 0.074 |
| 0.04669 | -9.74E-12 | NA | NA | 1.50E-04 | NA | NA | 4 | 121.892 | -235.784 | 1.998 | 0.073 |
| 0.0038 | NA | NA | + | NA | NA | NA | 5 | 129.463 | -248.927 | 0 | 0.332 |
| 0.02791 | NA | NA | + | NA | -1.11E-02 | NA | 6 | 130.221 | -248.443 | 0.484 | 0.26 |
| -0.00877 | NA | NA | + | NA | NA | 5.17E-03 | 6 | 129.624 | -247.248 | 1.679 | 0.143 |
| 0.00764 | NA | NA | + | 2.84E-05 | NA | NA | 6 | 129.559 | -247.118 | 1.809 | 0.134 |
| 0.00215 | 1.00E-10 | NA | + | NA | NA | NA | 6 | 129.533 | -247.067 | I.86 | 0.131 |
| 0.00347 | NA | NA | + | NA | NA | NA | 5 | 114.34 | -218.68 | 0 | 0.38 |
| 0.01375 | NA | NA | + | 5.71E-05 | NA | NA | 6 | 114.577 | -217.153 | 1.527 | 0.177 |
| 0.01758 | NA | NA | + | NA | -6.28E-03 | NA | 6 | 114.468 | -216.935 | 1.745 | 0.159 |
| -0.00079 | NA | NA | + | NA | NA | 1.77E-03 | 6 | 114.356 | -216.712 | 1.968 | 0.142 |
| 0.00275 | 3.95E-11 | NA | + | NA | NA | NA | 6 | 114.35 | -216.699 | I.98 | 0.141 |
| 0.00092 | NA | NA | + | NA | NA | NA | 5 | 126.267 | -242.535 | 0 | 0.34 |
| 0.02716 | NA | NA | + | NA | -1.15E-02 | NA | 6 | 126.84 | -241.68 | 0.855 | 0.222 |
| 0.01175 | NA | NA | + | 6.01E-05 | NA | NA | 6 | 126.617 | -241.233 | 1.301 | 0.177 |
| -0.00106 | 1.16E-10 | NA | + | NA | NA | NA | 6 | 126.342 | -240.684 | I.85 | 0.135 |
| 0.00479 | NA | NA | + | NA | NA | -1.64E-03 | 6 | 126.282 | -240.563 | 1.971 | 0.127 |
| 0.00517 | NA | NA | + | NA | NA | NA | 5 | 123.883 | -237.767 | 0 | 0.363 |
| 0.01824 | NA | NA | + | 6.85E-05 | NA | NA | 6 | 124.252 | -236.503 | 1.264 | 0.193 |
| 0.023 | NA | NA | + | NA | -7.82E-03 | NA | 6 | 124.103 | -236.206 | 1.561 | 0.166 |
| 0.01164 | NA | NA | + | NA | NA | -2.75E-03 | 6 | 123.922 | -235.843 | 1.924 | 0.139 |
| 0.00397 | 6.98E-11 | NA | + | NA | NA | NA | 6 | 123.917 | -235.834 | 1.933 | 0.138 |
| 0.0038 | NA | NA | + | NA | NA | NA | 5 | 121.237 | -232.473 | 0 | 0.296 |
| 0.01952 | NA | NA | + | 8.01E-05 | NA | NA | 6 | 121.728 | -231.457 | 1.016 | 0.178 |
| 0.02564 | NA | NA | + | NA | -9.66E-03 | NA | 6 | 121.542 | -231.084 | 1.389 | 0.148 |
| 0.04641 | NA | NA | NA | 1.47E-04 | NA | NA | 3 | 118.519 | -231.039 | 1.434 | 0.145 |
| 0.01435 | NA | NA | + | NA | NA | -4.45E-03 | 6 | 121.335 | -230.669 | 1.804 | 0.12 |
| 0.00259 | 6.98E-11 | NA | + | NA | NA | NA | 6 | 121.272 | -230.544 | 1.929 | 0.113 |
| 0.00244 | NA | NA | + | NA | NA | NA | 5 | 127.68 | -245.359 | 0 | 0.355 |
| 0.01488 | NA | NA | + | 6.94E-05 | NA | NA | 6 | 128.148 | -244.295 | 1.064 | 0.208 |
| 0.01959 | NA | NA | + | NA | -7.50E-03 | NA | 6 | 127.912 | -243.824 | 1.536 | 0.165 |
| 0.00062 | 1.02E-10 | NA | + | NA | NA | NA | 6 | 127.736 | -243.472 | 1.888 | 0.138 |
| -0.00281 | NA | NA | + | NA | NA | 2.25E-03 | 6 | 127.709 | -243.418 | 1.941 | 0.134 |
| 0.00691 | NA | NA | + | NA | NA | NA | 5 | 127.352 | -244.703 | 0 | 0.371 |
| 0.01535 | NA | NA | + | 5.24E-05 | NA | NA | 6 | 127.621 | -243.243 | 1.461 | 0.179 |
| 0.02187 | NA | NA | + | NA | -6.69E-03 | NA | 6 | 127.551 | -243.102 | 1.602 | 0.167 |
| 0.0004 | NA | NA | + | NA | NA | 2.72E-03 | 6 | 127.392 | -242.784 | 1.919 | 0.142 |
| 0.00551 | 8.34E-11 | NA | + | NA | NA | NA | 6 | 127.391 | -242.782 | 1.921 | 0.142 |
| 0.0473 | NA | NA | NA | 1.44E-04 | NA | NA | 3 | 117.089 | -228.178 | 0 | 0.202 |
| 0.00842 | NA | NA | + | NA | NA | NA | 5 | 118.892 | -227.783 | 0.394 | 0.166 |
| 0.02929 | NA | NA | + | 1.05E-04 | NA | NA | 6 | 119.683 | -227.367 | 0.811 | 0.135 |
| 0.06928 | NA | NA | NA | 1.59E-04 | NA | -7.72E-03 | 4 | 117.35 | -226.7 | 1.477 | 0.097 |
| 0.06302 | NA | NA | NA | 1.35E-04 | -8.20E-03 | NA | 4 | 117.29 | -226.579 | 1.598 | 0.091 |
| 0.03148 | NA | NA | + | NA | -1.03E-02 | NA | 6 | 119.2 | -226.401 | 1.777 | 0.083 |
| 0.02527 | NA | NA | + | NA | NA | -7.01E-03 | 6 | 119.117 | -226.235 | 1.943 | 0.076 |
| 0.05563 | NA | NA | + | 1.19E-04 | NA | -9.76E-03 | 7 | 120.111 | -226.223 | 1.955 | 0.076 |
| 0.04696 | 2.12E-11 | NA | NA | 1.44E-04 | NA | NA | 4 | 117.092 | -226.185 | 1.993 | 0.075 |
| 0.00385 | NA | NA | + | NA | NA | NA | 5 | 126.751 | -243.503 | 0 | 0.354 |
| 0.01508 | NA | NA | + | 6.96E-05 | NA | NA | 6 | 127.237 | -242.474 | 1.029 | 0.212 |
| 0.02018 | NA | NA | + | NA | -7.24E-03 | NA | 6 | 126.997 | -241.994 | 1.509 | 0.167 |
| 0.00282 | 6.20E-11 | NA | + | NA | NA | NA | 6 | 126.779 | -241.558 | 1.945 | 0.134 |
| -0.00084 | NA | NA | + | NA | NA | 1.95E-03 | 6 | 126.771 | -241.542 | 1.961 | 0.133 |
| 0.00377 | NA | NA | + | NA | NA | NA | 5 | 123.658 | -237.316 | 0 | 0.364 |
| 0.01617 | NA | NA | + | 6.95E-05 | NA | NA | 6 | 124.079 | -236.159 | 1.157 | 0.204 |
| 0.01947 | NA | NA | + | NA | -6.78E-03 | NA | 6 | 123.838 | -235.676 | 1.639 | 0.16 |
| 0.00243 | 7.60E-11 | NA | + | NA | NA | NA | 6 | 123.693 | -235.387 | 1.929 | 0.139 |
| 0.00357 | NA | NA | + | NA | NA | 8.87E-05 | 6 | 123.658 | -235.316 | 2 | 0.134 |
| 0.00644 | NA | NA | + | NA | NA | NA | 5 | 126.378 | -242.756 | 0 | 0.29 |
| 0.02216 | NA | NA | + | 9.07E-05 | NA | NA | 6 | 127.118 | -242.236 | 0.52 | 0.224 |
| 0.02396 | NA | NA | + | NA | -7.84E-03 | NA | 6 | 126.618 | -241.235 | I.52 | 0.136 |
| 0.04893 | NA | NA | NA | 1.58E-04 | NA | NA | 3 | 123.556 | -241.111 | 1.644 | 0.127 |
| 0.00436 | 1.33E-10 | NA | + | NA | NA | NA | 6 | 126.47 | -240.94 | 1.816 | 0.117 |
| 0.0065 | NA | NA | + | NA | NA | -2.58E-05 | 6 | 126.378 | -240.756 | 2 | 0.107 |
| 0.00381 | NA | NA | + | NA | NA | NA | 5 | 126.872 | -243.744 | 0 | 0.336 |
| 0.03012 | NA | NA | + | NA | -1.17E-02 | NA | 6 | 127.485 | -242.971 | 0.774 | 0.228 |
| 0.01432 | NA | NA | + | 6.17E-05 | NA | NA | 6 | 127.236 | -242.472 | 1.272 | 0.178 |
| 0.00226 | 9.32E-11 | NA | + | NA | NA | NA | 6 | 126.926 | -241.853 | 1.892 | 0.13 |
| 0.0102 | NA | NA | + | NA | NA | -2.65E-03 | 6 | 126.91 | -241.82 | 1.924 | 0.128 |
| 0.00316 | NA | NA | + | NA | NA | NA | 5 | 127.322 | -244.644 | 0 | 0.361 |
| 0.01479 | NA | NA | + | 6.77E-05 | NA | NA | 6 | 127.753 | -243.506 | 1.138 | 0.204 |
| 0.01767 | NA | NA | + | NA | -6.34E-03 | NA | 6 | 127.479 | -242.959 | 1.685 | 0.155 |
| 0.01251 | NA | NA | + | NA | NA | -3.99E-03 | 6 | 127.404 | -242.808 | 1.836 | 0.144 |
| 0.00222 | 5.62E-11 | NA | + | NA | NA | NA | 6 | 127.342 | -242.685 | I.96 | 0.135 |
| 0.00339 | NA | NA | + | NA | NA | NA | 5 | 121.103 | -232.207 | 0 | 0.369 |
| 0.01937 | NA | NA | + | NA | -6.98E-03 | NA | 6 | 121.322 | -230.645 | 1.562 | 0.169 |
| 0.00993 | NA | NA | + | 4.37E-05 | NA | NA | 6 | 121.286 | -230.573 | 1.634 | 0.163 |
| -0.00954 | NA | NA | + | NA | NA | 5.48E-03 | 6 | 121.284 | -230.567 | 1.639 | 0.162 |
| 0.00267 | 4.10E-11 | NA | + | NA | NA | NA | 6 | 121.117 | -230.234 | 1.972 | 0.137 |
| -0.00121 | NA | NA | + | NA | NA | NA | 5 | 121.08 | -232.16 | 0 | 0.339 |
| 0.02457 | NA | NA | + | NA | -1.13E-02 | NA | 6 | 121.554 | -231.108 | 1.052 | 0.2 |
| 0.01192 | NA | NA | + | 7.34E-05 | NA | NA | 6 | 121.536 | -231.072 | 1.088 | 0.197 |
| 0.00688 | NA | NA | + | NA | NA | -3.34E-03 | 6 | 121.136 | -230.272 | 1.888 | 0.132 |
| -0.00298 | 1.01E-10 | NA | + | NA | NA | NA | 6 | 121.133 | -230.267 | 1.893 | 0.132 |
| 0.00349 | NA | NA | + | NA | NA | NA | 5 | 115.124 | -220.247 | 0 | 0.246 |
| 0.04723 | NA | NA | NA | 1.52E-04 | NA | NA | 3 | 112.931 | -219.862 | 0.385 | 0.203 |
| 0.0207 | NA | NA | + | 8.71E-05 | NA | NA | 6 | 115.64 | -219.281 | 0.966 | 0.152 |
| 0.02309 | NA | NA | + | NA | -8.61E-03 | NA | 6 | 115.345 | -218.689 | 1.558 | 0.113 |
| 0.01519 | NA | NA | + | NA | NA | -4.93E-03 | 6 | 115.227 | -218.454 | 1.793 | 0.1 |
| 0.0018 | 9.67E-11 | NA | + | NA | NA | NA | 6 | 115.163 | -218.327 | I.92 | 0.094 |
| 0.06274 | NA | NA | NA | 1.41E-04 | -8.15E-03 | NA | 4 | 113.129 | -218.258 | 1.989 | 0.091 |
| 0.0004 | NA | NA | + | NA | NA | NA | 5 | 124.521 | -239.042 | 0 | 0.358 |
| 0.01196 | NA | NA | + | 6.17E-05 | NA | NA | 6 | 124.865 | -237.729 | 1.313 | 0.186 |
| 0.0198 | NA | NA | + | NA | -8.53E-03 | NA | 6 | 124.8 | -237.6 | 1.442 | 0.174 |
| 0.0119 | NA | NA | + | NA | NA | -4.86E-03 | 6 | 124.639 | -237.279 | 1.763 | 0.148 |
| 0.00003 | 2.07E-11 | NA | + | NA | NA | NA | 6 | 124.528 | -237.055 | 1.987 | 0.133 |
| 0.00416 | NA | NA | + | NA | NA | NA | 5 | 119.205 | -228.41 | 0 | 0.356 |
| 0.021 | NA | NA | + | 8.66E-05 | NA | NA | 6 | 119.763 | -227.526 | 0.885 | 0.229 |
| 0.01807 | NA | NA | + | NA | -6.11E-03 | NA | 6 | 119.328 | -226.656 | 1.754 | 0.148 |
| 0.0027 | 8.10E-11 | NA | + | NA | NA | NA | 6 | 119.239 | -226.477 | 1.933 | 0.135 |
| 0.00182 | NA | NA | + | NA | NA | 9.94E-04 | 6 | 119.211 | -226.422 | 1.988 | 0.132 |
| 0.0046 | NA | NA | + | NA | NA | NA | 5 | 122.81 | -235.621 | 0 | 0.358 |
| 0.01503 | NA | NA | + | 6.47E-05 | NA | NA | 6 | 123.196 | -234.391 | 1.229 | 0.194 |
| 0.0232 | NA | NA | + | NA | -8.22E-03 | NA | 6 | 123.091 | -234.182 | 1.438 | 0.174 |
| -0.0023 | NA | NA | + | NA | NA | 2.86E-03 | 6 | 122.859 | -233.717 | 1.903 | 0.138 |
| 0.00324 | 7.70E-11 | NA | + | NA | NA | NA | 6 | 122.841 | -233.683 | 1.938 | 0.136 |
| 0.00203 | NA | NA | + | NA | NA | NA | 5 | 124.964 | -239.928 | 0 | 0.35 |
| 0.01244 | NA | NA | + | 6.33E-05 | NA | NA | 6 | 125.381 | -238.761 | 1.167 | 0.195 |
| 0.02119 | NA | NA | + | NA | -8.43E-03 | NA | 6 | 125.315 | -238.63 | 1.299 | 0.183 |
| -0.006 | NA | NA | + | NA | NA | 3.45E-03 | 6 | 125.037 | -238.074 | 1.854 | 0.138 |
| 0.00059 | 8.26E-11 | NA | + | NA | NA | NA | 6 | 125.007 | -238.014 | 1.914 | 0.134 |
| 0.00073 | NA | NA | + | NA | NA | NA | 5 | 126.754 | -243.509 | 0 | 0.284 |
| 0.02773 | NA | NA | + | NA | -1.21E-02 | NA | 6 | 127.634 | -243.269 | 0.24 | 0.252 |
| -0.01359 | NA | NA | + | NA | NA | 6.04E-03 | 6 | 126.964 | -241.929 | I.58 | 0.129 |
| 0.00538 | NA | NA | + | 3.06E-05 | NA | NA | 6 | 126.866 | -241.732 | 1.776 | 0.117 |
| -0.00076 | 9.01E-11 | NA | + | NA | NA | NA | 6 | 126.806 | -241.612 | 1.897 | 0.11 |
| 0.01428 | NA | NA | + | NA | -1.18E-02 | 5.42E-03 | 7 | 127.805 | -241.61 | 1.899 | 0.11 |
| 0.00367 | NA | NA | + | NA | NA | NA | 5 | 125.812 | -241.624 | 0 | 0.368 |
| 0.02189 | NA | NA | + | NA | -7.94E-03 | NA | 6 | 126.124 | -240.247 | 1.377 | 0.185 |
| 0.01146 | NA | NA | + | 4.70E-05 | NA | NA | 6 | 126.032 | -240.064 | I.56 | 0.169 |
| 0.00242 | 7.35E-11 | NA | + | NA | NA | NA | 6 | 125.848 | -239.697 | 1.927 | 0.14 |
| -0.00044 | NA | NA | + | NA | NA | 1.79E-03 | 6 | 125.829 | -239.659 | 1.965 | 0.138 |
| 0.00124 | NA | NA | + | NA | NA | NA | 5 | 121.305 | -232.609 | 0 | 0.332 |
| 0.01626 | NA | NA | + | 7.51E-05 | NA | NA | 6 | 121.751 | -231.502 | 1.107 | 0.191 |
| 0.02711 | NA | NA | + | NA | -1.12E-02 | NA | 6 | 121.725 | -231.45 | 1.159 | 0.186 |
| 0.01969 | NA | NA | + | NA | NA | -7.96E-03 | 6 | 121.607 | -231.213 | 1.396 | 0.165 |
| 0.0001 | 6.54E-11 | NA | + | NA | NA | NA | 6 | 121.335 | -230.671 | 1.938 | 0.126 |
| 0.00003 | NA | NA | + | NA | NA | NA | 5 | 117.027 | -224.054 | 0 | 0.357 |
| 0.01282 | NA | NA | + | 7.37E-05 | NA | NA | 6 | 117.487 | -222.974 | 01.VIII | 0.208 |
| 0.0187 | NA | NA | + | NA | -8.11E-03 | NA | 6 | 117.268 | -222.535 | 1.519 | 0.167 |
| -0.00146 | 8.23E-11 | NA | + | NA | NA | NA | 6 | 117.066 | -222.132 | 1.923 | 0.137 |
| 0.00064 | NA | NA | + | NA | NA | -2.57E-04 | 6 | 117.027 | -222.055 | 1.999 | 0.131 |
| 0.00452 | NA | NA | + | NA | NA | NA | 5 | 123.664 | -237.328 | 0 | 0.336 |
| 0.01992 | NA | NA | + | 8.82E-05 | NA | NA | 6 | 124.353 | -236.707 | 0.621 | 0.246 |
| 0.02174 | NA | NA | + | NA | -7.61E-03 | NA | 6 | 123.912 | -235.824 | 1.503 | 0.158 |
| 0.00223 | 1.27E-10 | NA | + | NA | NA | NA | 6 | 123.748 | -235.496 | 1.832 | 0.134 |
| 0.00769 | NA | NA | + | NA | NA | -1.33E-03 | 6 | 123.672 | -235.345 | 1.983 | 0.125 |
| 0.00162 | NA | NA | + | NA | NA | NA | 5 | 126.006 | -242.012 | 0 | 0.37 |
| 0.01285 | NA | NA | + | 6.54E-05 | NA | NA | 6 | 126.403 | -240.805 | 1.206 | 0.203 |
| 0.01253 | NA | NA | + | NA | -4.72E-03 | NA | 6 | 126.091 | -240.183 | 1.829 | 0.148 |
| 0.00061 | 6.03E-11 | NA | + | NA | NA | NA | 6 | 126.033 | -240.065 | 1.946 | 0.14 |
| 0.0058 | NA | NA | + | NA | NA | -1.82E-03 | 6 | 126.021 | -240.043 | 1.969 | 0.138 |
| 0.00623 | NA | NA | + | NA | NA | NA | 5 | 122.337 | -234.675 | 0 | 0.305 |
| 0.01909 | NA | NA | + | 7.30E-05 | NA | NA | 6 | 122.79 | -233.579 | 1.095 | 0.176 |
| 0.04654 | NA | NA | NA | 1.31E-04 | NA | NA | 3 | 119.678 | -233.355 | 1.319 | 0.158 |
| 0.0212 | NA | NA | + | NA | -6.59E-03 | NA | 6 | 122.502 | -233.004 | 1.671 | 0.132 |
| 0.00489 | 7.95E-11 | NA | + | NA | NA | NA | 6 | 122.38 | -232.759 | 1.915 | 0.117 |
| 0.00846 | NA | NA | + | NA | NA | -9.40E-04 | 6 | 122.342 | -232.683 | 1.991 | 0.113 |
| 0.00466 | NA | NA | + | NA | NA | NA | 5 | 130.173 | -250.346 | 0 | 0.345 |
| 0.02841 | NA | NA | + | NA | -1.04E-02 | NA | 6 | 130.704 | -249.407 | 0.939 | 0.216 |
| 0.01335 | NA | NA | + | 5.28E-05 | NA | NA | 6 | 130.462 | -248.923 | 1.422 | 0.169 |
| 0.00278 | 1.12E-10 | NA | + | NA | NA | NA | 6 | 130.252 | -248.505 | 1.841 | 0.137 |
| -0.00183 | NA | NA | + | NA | NA | 2.78E-03 | 6 | 130.216 | -248.432 | 1.914 | 0.132 |
| 0.00055 | NA | NA | + | NA | NA | NA | 5 | 129.828 | -249.657 | 0 | 0.359 |
| 0.02416 | NA | NA | + | NA | -1.06E-02 | NA | 6 | 130.374 | -248.749 | 0.908 | 0.228 |
| 0.00533 | NA | NA | + | 2.98E-05 | NA | NA | 6 | 129.921 | -247.842 | 1.814 | 0.145 |
| -0.00054 | 6.24E-11 | NA | + | NA | NA | NA | 6 | 129.86 | -247.72 | 1.936 | 0.136 |
| -0.00042 | NA | NA | + | NA | NA | 4.09E-04 | 6 | 129.829 | -247.659 | 1.998 | 0.132 |
| 0.00171 | NA | NA | + | NA | NA | NA | 5 | 129.091 | -248.182 | 0 | 0.263 |
| 0.01735 | NA | NA | + | 8.90E-05 | NA | NA | 6 | 129.856 | -247.712 | 0.471 | 0.208 |
| 0.03032 | NA | NA | + | NA | -1.26E-02 | NA | 6 | 129.753 | -247.505 | 0.677 | 0.188 |
| 0.04164 | NA | NA | + | 8.12E-05 | -1.13E-02 | NA | 7 | 130.387 | -246.774 | 1.409 | 0.13 |
| 0.01359 | NA | NA | + | NA | NA | -4.96E-03 | 6 | 129.212 | -246.425 | 1.757 | 0.109 |
| 0.00054 | 7.29E-11 | NA | + | NA | NA | NA | 6 | 129.143 | -246.285 | 1.897 | 0.102 |
| -0.00039 | NA | NA | + | NA | NA | NA | 5 | 118.357 | -226.715 | 0 | 0.334 |
| 0.03001 | NA | NA | + | NA | -1.31E-02 | NA | 6 | 118.944 | -225.888 | 0.827 | 0.221 |
| 0.01048 | NA | NA | + | 5.77E-05 | NA | NA | 6 | 118.626 | -225.252 | 1.463 | 0.161 |
| 0.01644 | NA | NA | + | NA | NA | -7.12E-03 | 6 | 118.599 | -225.199 | 1.516 | 0.157 |
| -0.00142 | 5.85E-11 | NA | + | NA | NA | NA | 6 | 118.389 | -224.778 | 1.937 | 0.127 |
| 0.00474 | NA | NA | + | NA | NA | NA | 5 | 121.139 | -232.279 | 0 | 0.278 |
| 0.02258 | NA | NA | + | 9.67E-05 | NA | NA | 6 | 121.897 | -231.795 | 0.484 | 0.218 |
| 0.04659 | NA | NA | NA | 1.51E-04 | NA | NA | 3 | 118.619 | -231.238 | 1.041 | 0.165 |
| 0.02032 | NA | NA | + | NA | -6.89E-03 | NA | 6 | 121.309 | -230.619 | I.66 | 0.121 |
| 0.00222 | 1.42E-10 | NA | + | NA | NA | NA | 6 | 121.249 | -230.498 | 1.781 | 0.114 |
| -0.00015 | NA | NA | + | NA | NA | 2.02E-03 | 6 | 121.159 | -230.317 | 1.961 | 0.104 |
| 0.00231 | NA | NA | + | NA | NA | NA | 5 | 122.458 | -234.917 | 0 | 0.365 |
| 0.01573 | NA | NA | + | 7.33E-05 | NA | NA | 6 | 122.91 | -233.82 | 1.097 | 0.211 |
| 0.01569 | NA | NA | + | NA | -5.80E-03 | NA | 6 | 122.584 | -233.168 | 1.749 | 0.152 |
| 0.00126 | 5.92E-11 | NA | + | NA | NA | NA | 6 | 122.487 | -232.973 | 1.944 | 0.138 |
| 0.00259 | NA | NA | + | NA | NA | -1.20E-04 | 6 | 122.458 | -232.917 | 2 | 0.134 |
| 0.00002 | NA | NA | + | NA | NA | NA | 5 | 125.761 | -241.521 | 0 | 0.363 |
| 0.01045 | NA | NA | + | 6.05E-05 | NA | NA | 6 | 126.111 | -240.223 | 1.299 | 0.19 |
| 0.01896 | NA | NA | + | NA | -8.20E-03 | NA | 6 | 126.041 | -240.082 | 1.439 | 0.177 |
| -0.00058 | 3.48E-11 | NA | + | NA | NA | NA | 6 | 125.775 | -239.55 | 1.971 | 0.135 |
| 0.00326 | NA | NA | + | NA | NA | -1.42E-03 | 6 | 125.771 | -239.542 | 1.979 | 0.135 |
| 0.0023 | NA | NA | + | NA | NA | NA | 5 | 123.301 | -236.602 | 0 | 0.353 |
| 0.01682 | NA | NA | + | 7.95E-05 | NA | NA | 6 | 123.827 | -235.654 | 0.948 | 0.22 |
| 0.01901 | NA | NA | + | NA | -7.36E-03 | NA | 6 | 123.491 | -234.982 | I.62 | 0.157 |
| 0.00072 | 9.31E-11 | NA | + | NA | NA | NA | 6 | 123.349 | -234.697 | 1.905 | 0.136 |
| 0.00817 | NA | NA | + | NA | NA | -2.44E-03 | 6 | 123.33 | -234.66 | 1.942 | 0.134 |
| 0.00372 | NA | NA | + | NA | NA | NA | 5 | 120.458 | -230.916 | 0 | 0.358 |
| 0.01574 | NA | NA | + | 6.96E-05 | NA | NA | 6 | 120.838 | -229.675 | 1.241 | 0.192 |
| 0.02348 | NA | NA | + | NA | -8.80E-03 | NA | 6 | 120.76 | -229.52 | 1.396 | 0.178 |
| 0.0021 | 8.91E-11 | NA | + | NA | NA | NA | 6 | 120.499 | -228.999 | 1.917 | 0.137 |
| 0.00905 | NA | NA | + | NA | NA | -2.23E-03 | 6 | 120.481 | -228.963 | 1.953 | 0.135 |
| 0.00278 | NA | NA | + | NA | NA | NA | 5 | 120.385 | -230.771 | 0 | 0.361 |
| 0.01415 | NA | NA | + | 6.29E-05 | NA | NA | 6 | 120.718 | -229.436 | 1.334 | 0.185 |
| 0.02265 | NA | NA | + | NA | -8.89E-03 | NA | 6 | 120.67 | -229.341 | I.43 | 0.177 |
| 0.001 | 1.01E-10 | NA | + | NA | NA | NA | 6 | 120.433 | -228.867 | 1.904 | 0.14 |
| 0.00838 | NA | NA | + | NA | NA | -2.37E-03 | 6 | 120.413 | -228.826 | 1.945 | 0.137 |
| 0.00305 | NA | NA | + | NA | NA | NA | 5 | 123.069 | -236.138 | 0 | 0.346 |
| 0.01557 | NA | NA | + | 7.04E-05 | NA | NA | 6 | 123.525 | -235.05 | 1.088 | 0.201 |
| 0.02557 | NA | NA | + | NA | -9.76E-03 | NA | 6 | 123.472 | -234.944 | 1.194 | 0.191 |
| 0.00131 | 9.98E-11 | NA | + | NA | NA | NA | 6 | 123.123 | -234.247 | 1.891 | 0.135 |
| 0.00184 | NA | NA | + | NA | NA | 5.23E-04 | 6 | 123.07 | -234.141 | 1.997 | 0.128 |
| 0.00361 | NA | NA | + | NA | NA | NA | 5 | 122.839 | -235.678 | 0 | 0.35 |
| 0.02597 | NA | NA | + | NA | -9.84E-03 | NA | 6 | 123.273 | -234.546 | 1.132 | 0.199 |
| 0.01469 | NA | NA | + | 6.49E-05 | NA | NA | 6 | 123.227 | -234.454 | 1.225 | 0.19 |
| 0.00269 | 5.21E-11 | NA | + | NA | NA | NA | 6 | 122.864 | -233.727 | 1.951 | 0.132 |
| 0.00228 | NA | NA | + | NA | NA | 5.68E-04 | 6 | 122.841 | -233.682 | 1.997 | 0.129 |
| 0.00213 | NA | NA | + | NA | NA | NA | 5 | 124.152 | -238.304 | 0 | 0.355 |
| 0.02482 | NA | NA | + | NA | -1.01E-02 | NA | 6 | 124.612 | -237.225 | 1.079 | 0.207 |
| 0.01108 | NA | NA | + | 5.40E-05 | NA | NA | 6 | 124.428 | -236.857 | 1.447 | 0.172 |
| 0.00708 | NA | NA | + | NA | NA | -2.09E-03 | 6 | 124.174 | -236.349 | 1.955 | 0.133 |
| 0.00125 | 4.96E-11 | NA | + | NA | NA | NA | 6 | 124.171 | -236.342 | 1.962 | 0.133 |
| 0.00265 | NA | NA | + | NA | NA | NA | 5 | 125.056 | -240.112 | 0 | 0.346 |
| 0.0282 | NA | NA | + | NA | -1.13E-02 | NA | 6 | 125.602 | -239.204 | 0.908 | 0.22 |
| 0.01199 | NA | NA | + | 5.39E-05 | NA | NA | 6 | 125.329 | -238.658 | 1.455 | 0.167 |
| 0.00108 | 8.93E-11 | NA | + | NA | NA | NA | 6 | 125.1 | -238.2 | 1.912 | 0.133 |
| 0.00958 | NA | NA | + | NA | NA | -2.90E-03 | 6 | 125.1 | -238.2 | 1.912 | 0.133 |
| -0.00016 | NA | NA | + | NA | NA | NA | 5 | 119.912 | -229.823 | 0 | 0.365 |
| 0.0193 | NA | NA | + | NA | -8.43E-03 | NA | 6 | 120.199 | -228.399 | 1.425 | 0.179 |
| 0.00968 | NA | NA | + | 5.61E-05 | NA | NA | 6 | 120.188 | -228.375 | 1.448 | 0.177 |
| -0.0078 | NA | NA | + | NA | NA | 3.31E-03 | 6 | 119.974 | -227.947 | 1.876 | 0.143 |
| -0.0009 | 4.11E-11 | NA | + | NA | NA | NA | 6 | 119.924 | -227.849 | 1.975 | 0.136 |
| 0.00328 | NA | NA | + | NA | NA | NA | 5 | 126.065 | -242.129 | 0 | 0.351 |
| 0.02629 | NA | NA | + | NA | -1.04E-02 | NA | 6 | 126.504 | -241.007 | 1.122 | 0.2 |
| 0.01318 | NA | NA | + | 5.98E-05 | NA | NA | 6 | 126.408 | -240.816 | 1.313 | 0.182 |
| 0.00178 | 8.99E-11 | NA | + | NA | NA | NA | 6 | 126.117 | -240.233 | 1.896 | 0.136 |
| 0.00081 | NA | NA | + | NA | NA | 1.01E-03 | 6 | 126.07 | -240.14 | 1.989 | 0.13 |
| 0.00265 | NA | NA | + | NA | NA | NA | 5 | 125.327 | -240.655 | 0 | 0.352 |
| 0.02698 | NA | NA | + | NA | -1.07E-02 | NA | 6 | 125.85 | -239.699 | 0.955 | 0.218 |
| 0.01124 | NA | NA | + | 4.90E-05 | NA | NA | 6 | 125.558 | -239.116 | 1.539 | 0.163 |
| 0.00165 | 5.89E-11 | NA | + | NA | NA | NA | 6 | 125.358 | -238.716 | 1.939 | 0.133 |
| 0.00808 | NA | NA | + | NA | NA | -2.34E-03 | 6 | 125.356 | -238.711 | 1.943 | 0.133 |
| 0.00112 | NA | NA | + | NA | NA | NA | 5 | 123.68 | -237.36 | 0 | 0.335 |
| 0.02663 | NA | NA | + | NA | -1.14E-02 | NA | 6 | 124.319 | -236.639 | 0.721 | 0.233 |
| 0.00881 | NA | NA | + | 4.67E-05 | NA | NA | 6 | 123.913 | -235.826 | 1.534 | 0.155 |
| -0.00137 | 1.47E-10 | NA | + | NA | NA | NA | 6 | 123.795 | -235.59 | I.77 | 0.138 |
| -0.00926 | NA | NA | + | NA | NA | 4.39E-03 | 6 | 123.794 | -235.589 | 1.771 | 0.138 |
| 0.00266 | NA | NA | + | NA | NA | NA | 5 | 122.557 | -235.114 | 0 | 0.365 |
| 0.02267 | NA | NA | + | NA | -8.93E-03 | NA | 6 | 122.914 | -233.828 | 1.286 | 0.192 |
| 0.00975 | NA | NA | + | 4.26E-05 | NA | NA | 6 | 122.729 | -233.458 | 1.656 | 0.159 |
| -0.00524 | NA | NA | + | NA | NA | 3.34E-03 | 6 | 122.621 | -233.241 | 1.873 | 0.143 |
| 0.00101 | 9.63E-11 | NA | + | NA | NA | NA | 6 | 122.605 | -233.209 | 1.905 | 0.141 |
| 0.0034 | NA | NA | + | NA | NA | NA | 5 | 126.171 | -242.342 | 0 | 0.363 |
| 0.0233 | NA | NA | + | NA | -8.81E-03 | NA | 6 | 126.52 | -241.041 | 1.302 | 0.19 |
| 0.01179 | NA | NA | + | 5.11E-05 | NA | NA | 6 | 126.43 | -240.861 | 1.482 | 0.173 |
| 0.00197 | 8.30E-11 | NA | + | NA | NA | NA | 6 | 126.219 | -240.438 | 1.905 | 0.14 |
| 0.00265 | NA | NA | + | NA | NA | 3.19E-04 | 6 | 126.172 | -240.344 | 1.999 | 0.134 |
| 0.00773 | NA | NA | + | NA | NA | NA | 5 | 127.634 | -245.268 | 0 | 0.364 |
| 0.01894 | NA | NA | + | 6.51E-05 | NA | NA | 6 | 128.024 | -244.048 | I.22 | 0.198 |
| 0.02451 | NA | NA | + | NA | -7.47E-03 | NA | 6 | 127.851 | -243.702 | 1.566 | 0.166 |
| 0.00677 | 5.46E-11 | NA | + | NA | NA | NA | 6 | 127.661 | -243.321 | 1.947 | 0.138 |
| 0.00928 | NA | NA | + | NA | NA | -6.59E-04 | 6 | 127.636 | -243.273 | 1.995 | 0.134 |
| 0.00197 | NA | NA | + | NA | NA | NA | 5 | 127.85 | -245.7 | 0 | 0.354 |
| 0.01362 | NA | NA | + | 6.59E-05 | NA | NA | 6 | 128.261 | -244.523 | 1.177 | 0.196 |
| 0.0225 | NA | NA | + | NA | -9.00E-03 | NA | 6 | 128.188 | -244.376 | 1.324 | 0.182 |
| 0.00045 | 9.08E-11 | NA | + | NA | NA | NA | 6 | 127.897 | -243.795 | 1.905 | 0.136 |
| 0.00471 | NA | NA | + | NA | NA | -1.15E-03 | 6 | 127.857 | -243.713 | 1.987 | 0.131 |
| 0.00454 | NA | NA | + | NA | NA | NA | 5 | 126.248 | -242.495 | 0 | 0.353 |
| 0.01546 | NA | NA | + | 6.67E-05 | NA | NA | 6 | 126.673 | -241.346 | 1.149 | 0.199 |
| 0.02376 | NA | NA | + | NA | -8.56E-03 | NA | 6 | 126.589 | -241.177 | 1.318 | 0.182 |
| 0.00345 | 6.49E-11 | NA | + | NA | NA | NA | 6 | 126.285 | -240.57 | 1.926 | 0.135 |
| 0.00038 | NA | NA | + | NA | NA | 1.73E-03 | 6 | 126.263 | -240.527 | 1.969 | 0.132 |
| 0.00235 | NA | NA | + | NA | NA | NA | 5 | 128.671 | -247.342 | 0 | 0.337 |
| 0.01392 | NA | NA | + | 7.04E-05 | NA | NA | 6 | 129.177 | -246.354 | 0.989 | 0.205 |
| 0.02495 | NA | NA | + | NA | -9.96E-03 | NA | 6 | 129.148 | -246.297 | 1.046 | 0.2 |
| 0.00086 | 9.01E-11 | NA | + | NA | NA | NA | 6 | 128.719 | -245.438 | 1.905 | 0.13 |
| 0.00848 | NA | NA | + | NA | NA | -2.60E-03 | 6 | 128.705 | -245.41 | 1.932 | 0.128 |
| 0.00278 | NA | NA | + | NA | NA | NA | 5 | 125.584 | -241.167 | 0 | 0.347 |
| 0.02516 | NA | NA | + | NA | -9.85E-03 | NA | 6 | 126.012 | -240.023 | 1.144 | 0.196 |
| 0.01436 | NA | NA | + | 6.69E-05 | NA | NA | 6 | 126.007 | -240.014 | 1.154 | 0.195 |
| 0.00137 | 8.58E-11 | NA | + | NA | NA | NA | 6 | 125.636 | -239.271 | 1.896 | 0.134 |
| 0.00531 | NA | NA | + | NA | NA | -1.06E-03 | 6 | 125.589 | -239.179 | 1.989 | 0.128 |
| 0.0033 | NA | NA | + | NA | NA | NA | 5 | 127.125 | -244.25 | 0 | 0.355 |
| 0.02418 | NA | NA | + | NA | -9.39E-03 | NA | 6 | 127.527 | -243.054 | 1.196 | 0.195 |
| 0.01223 | NA | NA | + | 5.67E-05 | NA | NA | 6 | 127.454 | -242.909 | 1.341 | 0.182 |
| 0.00248 | 5.12E-11 | NA | + | NA | NA | NA | 6 | 127.153 | -242.307 | 1.944 | 0.135 |
| -0.00067 | NA | NA | + | NA | NA | 1.66E-03 | 6 | 127.141 | -242.281 | 1.969 | 0.133 |
| 0.00193 | NA | NA | + | NA | NA | NA | 5 | 124.728 | -239.457 | 0 | 0.36 |
| 0.01098 | NA | NA | + | 5.71E-05 | NA | NA | 6 | 125.053 | -238.106 | I.35 | 0.183 |
| 0.01984 | NA | NA | + | NA | -7.96E-03 | NA | 6 | 125.032 | -238.065 | 1.392 | 0.18 |
| 0.00023 | 1.00E-10 | NA | + | NA | NA | NA | 6 | 124.781 | -237.561 | 1.895 | 0.14 |
| -0.00391 | NA | NA | + | NA | NA | 2.49E-03 | 6 | 124.762 | -237.524 | 1.932 | 0.137 |
| -0.0017 | NA | NA | + | NA | NA | NA | 5 | 121.256 | -232.512 | 0 | 0.33 |
| 0.02649 | NA | NA | + | NA | -1.25E-02 | NA | 6 | 121.837 | -231.675 | 0.837 | 0.217 |
| 0.01306 | NA | NA | + | 7.78E-05 | NA | NA | 6 | 121.755 | -231.51 | 1.002 | 0.2 |
| -0.00349 | 9.85E-11 | NA | + | NA | NA | NA | 6 | 121.305 | -230.61 | 1.902 | 0.128 |
| 0.00359 | NA | NA | + | NA | NA | -2.21E-03 | 6 | 121.282 | -230.564 | 1.948 | 0.125 |
| -0.00159 | NA | NA | + | NA | NA | NA | 5 | 123.707 | -237.414 | 0 | 0.347 |
| 0.01041 | NA | NA | + | 6.87E-05 | NA | NA | 6 | 124.144 | -236.289 | 1.125 | 0.198 |
| 0.02055 | NA | NA | + | NA | -9.59E-03 | NA | 6 | 124.09 | -236.18 | 1.234 | 0.187 |
| -0.00314 | 9.45E-11 | NA | + | NA | NA | NA | 6 | 123.767 | -235.535 | 1.879 | 0.136 |
| -0.0069 | NA | NA | + | NA | NA | 2.25E-03 | 6 | 123.734 | -235.468 | 1.946 | 0.131 |
| 0.00971 | NA | NA | + | NA | NA | NA | 5 | 117.855 | -225.711 | 0 | 0.287 |
| 0.02584 | NA | NA | + | 9.07E-05 | NA | NA | 6 | 118.468 | -224.936 | 0.775 | 0.195 |
| 0.04814 | NA | NA | NA | 1.40E-04 | NA | NA | 3 | 115.451 | -224.902 | 0.809 | 0.192 |
| 0.01942 | NA | NA | + | NA | -4.30E-03 | NA | 6 | 117.915 | -223.829 | 1.882 | 0.112 |
| 0.00868 | 6.20E-11 | NA | + | NA | NA | NA | 6 | 117.88 | -223.76 | 1.951 | 0.108 |
| 0.00943 | NA | NA | + | NA | NA | 1.18E-04 | 6 | 117.856 | -223.711 | 2 | 0.106 |
| 0.00521 | NA | NA | + | NA | NA | NA | 5 | 124.356 | -238.713 | 0 | 0.375 |
| 0.01718 | NA | NA | + | 6.63E-05 | NA | NA | 6 | 124.729 | -237.459 | 1.254 | 0.2 |
| 0.01486 | NA | NA | + | NA | -4.27E-03 | NA | 6 | 124.422 | -236.845 | 1.868 | 0.147 |
| 0.0045 | 4.14E-11 | NA | + | NA | NA | NA | 6 | 124.368 | -236.736 | 1.977 | 0.14 |
| 0.00606 | NA | NA | + | NA | NA | -3.57E-04 | 6 | 124.357 | -236.714 | 1.999 | 0.138 |
| 0.00189 | NA | NA | + | NA | NA | NA | 5 | 123.521 | -237.042 | 0 | 0.353 |
| 0.01429 | NA | NA | + | 7.28E-05 | NA | NA | 6 | 124.01 | -236.02 | 1.022 | 0.212 |
| 0.0176 | NA | NA | + | NA | -6.95E-03 | NA | 6 | 123.732 | -235.464 | 1.578 | 0.161 |
| -0.00002 | 1.10E-10 | NA | + | NA | NA | NA | 6 | 123.59 | -235.179 | 1.863 | 0.139 |
| -0.00452 | NA | NA | + | NA | NA | 2.72E-03 | 6 | 123.56 | -235.121 | 1.921 | 0.135 |
| 0.00059 | NA | NA | + | NA | NA | NA | 5 | 120.997 | -231.994 | 0 | 0.353 |
| 0.0152 | NA | NA | + | 7.83E-05 | NA | NA | 6 | 121.501 | -231.003 | 0.991 | 0.215 |
| 0.01965 | NA | NA | + | NA | -8.35E-03 | NA | 6 | 121.241 | -230.482 | 1.512 | 0.166 |
| 0.0067 | NA | NA | + | NA | NA | -2.55E-03 | 6 | 121.029 | -230.059 | 1.935 | 0.134 |
| -0.00051 | 6.16E-11 | NA | + | NA | NA | NA | 6 | 121.016 | -230.032 | 1.962 | 0.132 |
| 0.00433 | NA | NA | + | NA | NA | NA | 5 | 118.057 | -226.114 | 0 | 0.285 |
| 0.04712 | NA | NA | NA | 1.44E-04 | NA | NA | 3 | 115.667 | -225.335 | 0.779 | 0.193 |
| 0.01924 | NA | NA | + | 8.43E-05 | NA | NA | 6 | 118.633 | -225.266 | 0.848 | 0.187 |
| 0.01638 | NA | NA | + | NA | -5.30E-03 | NA | 6 | 118.158 | -224.316 | 1.798 | 0.116 |
| 0.00246 | 1.07E-10 | NA | + | NA | NA | NA | 6 | 118.12 | -224.24 | 1.874 | 0.112 |
| 0.00075 | NA | NA | + | NA | NA | 1.51E-03 | 6 | 118.068 | -224.136 | 1.978 | 0.106 |
| 0.05002 | NA | NA | NA | 1.58E-04 | NA | NA | 3 | 115.457 | -224.914 | 0 | 0.246 |
| 0.00649 | NA | NA | + | NA | NA | NA | 5 | 117.179 | -224.359 | 0.555 | 0.186 |
| 0.02818 | NA | NA | + | 1.12E-04 | NA | NA | 6 | 118.093 | -224.186 | 0.728 | 0.171 |
| 0.07156 | NA | NA | NA | 1.72E-04 | NA | -7.58E-03 | 4 | 115.685 | -223.371 | 1.543 | 0.114 |
| 0.05866 | NA | NA | NA | 1.53E-04 | -4.48E-03 | NA | 4 | 115.519 | -223.038 | 1.876 | 0.096 |
| 0.0552 | NA | NA | + | 1.27E-04 | NA | -1.01E-02 | 7 | 118.505 | -223.01 | 1.904 | 0.095 |
| 0.04942 | 3.86E-11 | NA | NA | 1.57E-04 | NA | NA | 4 | 115.467 | -222.934 | I.98 | 0.091 |
| -0.00025 | NA | NA | + | NA | NA | NA | 5 | 127.246 | -244.492 | 0 | 0.361 |
| 0.02321 | NA | NA | + | NA | -1.01E-02 | NA | 6 | 127.668 | -243.335 | 1.157 | 0.203 |
| 0.00767 | NA | NA | + | 4.50E-05 | NA | NA | 6 | 127.441 | -242.882 | 1.611 | 0.162 |
| 0.00628 | NA | NA | + | NA | NA | -2.92E-03 | 6 | 127.289 | -242.577 | 1.915 | 0.139 |
| -0.00119 | 5.29E-11 | NA | + | NA | NA | NA | 6 | 127.266 | -242.533 | I.96 | 0.136 |
| 0.00333 | NA | NA | + | NA | NA | NA | 5 | 128.746 | -247.491 | 0 | 0.352 |
| 0.01812 | NA | NA | + | 7.82E-05 | NA | NA | 6 | 129.282 | -246.565 | 0.927 | 0.221 |
| 0.01937 | NA | NA | + | NA | -7.02E-03 | NA | 6 | 128.943 | -245.887 | 1.605 | 0.158 |
| 0.00968 | NA | NA | + | NA | NA | -2.69E-03 | 6 | 128.784 | -245.567 | 1.924 | 0.135 |
| 0.00233 | 5.73E-11 | NA | + | NA | NA | NA | 6 | 128.781 | -245.562 | 1.929 | 0.134 |
| 0.00387 | NA | NA | + | NA | NA | NA | 5 | 123.05 | -236.101 | 0 | 0.278 |
| 0.02078 | NA | NA | + | 9.10E-05 | NA | NA | 6 | 123.726 | -235.452 | 0.649 | 0.201 |
| 0.04524 | NA | NA | NA | 1.53E-04 | NA | NA | 3 | 120.62 | -235.24 | 0.861 | 0.181 |
| 0.01951 | NA | NA | + | NA | -7.00E-03 | NA | 6 | 123.222 | -234.444 | 1.657 | 0.121 |
| 0.01486 | NA | NA | + | NA | NA | -4.50E-03 | 6 | 123.15 | -234.3 | 1.801 | 0.113 |
| 0.00289 | 5.92E-11 | NA | + | NA | NA | NA | 6 | 123.081 | -234.162 | 1.938 | 0.106 |
| 0.00844 | NA | NA | + | NA | NA | NA | 5 | 125.798 | -241.597 | 0 | 0.356 |
| 0.01987 | NA | NA | + | 6.79E-05 | NA | NA | 6 | 126.221 | -240.442 | 1.155 | 0.2 |
| 0.02689 | NA | NA | + | NA | -8.31E-03 | NA | 6 | 126.081 | -240.162 | 1.435 | 0.174 |
| 0.01593 | NA | NA | + | NA | NA | -3.11E-03 | 6 | 125.846 | -239.692 | 1.905 | 0.137 |
| 0.00749 | 5.58E-11 | NA | + | NA | NA | NA | 6 | 125.821 | -239.643 | 1.954 | 0.134 |
| 0.00189 | NA | NA | + | NA | NA | NA | 5 | 119.005 | -228.01 | 0 | 0.338 |
| 0.01855 | NA | NA | + | 9.48E-05 | NA | NA | 6 | 119.78 | -227.56 | 0.45 | 0.27 |
| 0.01366 | NA | NA | + | NA | -5.09E-03 | NA | 6 | 119.104 | -226.209 | 1.801 | 0.137 |
| 0.00038 | 8.66E-11 | NA | + | NA | NA | NA | 6 | 119.057 | -226.115 | 1.895 | 0.131 |
| 0.00043 | NA | NA | + | NA | NA | 6.27E-04 | 6 | 119.007 | -226.014 | 1.996 | 0.124 |
| 0.00397 | NA | NA | + | NA | NA | NA | 5 | 123.869 | -237.739 | 0 | 0.364 |
| 0.0248 | NA | NA | + | NA | -9.19E-03 | NA | 6 | 124.188 | -236.377 | 1.362 | 0.184 |
| 0.01332 | NA | NA | + | 5.31E-05 | NA | NA | 6 | 124.121 | -236.242 | 1.497 | 0.172 |
| 0.01285 | NA | NA | + | NA | NA | -3.78E-03 | 6 | 123.943 | -235.886 | 1.852 | 0.144 |
| 0.00326 | 3.97E-11 | NA | + | NA | NA | NA | 6 | 123.88 | -235.76 | 1.978 | 0.135 |
| 0.00373 | NA | NA | + | NA | NA | NA | 5 | 126.687 | -243.375 | 0 | 0.332 |
| 0.02892 | NA | NA | + | NA | -1.12E-02 | NA | 6 | 127.453 | -242.906 | 0.469 | 0.262 |
| 0.00958 | NA | NA | + | 3.92E-05 | NA | NA | 6 | 126.86 | -241.719 | 1.656 | 0.145 |
| -0.00598 | NA | NA | + | NA | NA | 4.13E-03 | 6 | 126.789 | -241.577 | 1.798 | 0.135 |
| 0.00273 | 5.62E-11 | NA | + | NA | NA | NA | 6 | 126.721 | -241.442 | 1.933 | 0.126 |
| 0.00269 | NA | NA | + | NA | NA | NA | 5 | 120.674 | -231.348 | 0 | 0.225 |
| 0.02185 | NA | NA | + | 1.05E-04 | NA | NA | 6 | 121.543 | -231.085 | 0.263 | 0.197 |
| 0.0472 | NA | NA | NA | 1.62E-04 | NA | NA | 3 | 118.078 | -230.155 | 1.193 | 0.124 |
| 0.02144 | NA | NA | + | NA | -8.26E-03 | NA | 6 | 120.905 | -229.809 | 1.539 | 0.104 |
| 0.0122 | NA | NA | + | NA | NA | -3.92E-03 | 6 | 120.745 | -229.489 | 1.859 | 0.089 |
| 0.03975 | NA | NA | + | 1.14E-04 | NA | -6.67E-03 | 7 | 121.742 | -229.485 | 1.863 | 0.089 |
| 0.00106 | 9.25E-11 | NA | + | NA | NA | NA | 6 | 120.725 | -229.449 | 1.899 | 0.087 |
| 0.03626 | NA | NA | + | 1.00E-04 | -6.71E-03 | NA | 7 | 121.695 | -229.389 | 1.959 | 0.085 |
| 0.00631 | NA | NA | + | NA | NA | NA | 5 | 116.692 | -223.385 | 0 | 0.369 |
| 0.01827 | NA | NA | + | 6.32E-05 | NA | NA | 6 | 116.98 | -221.96 | 1.425 | 0.181 |
| 0.02473 | NA | NA | + | NA | -8.03E-03 | NA | 6 | 116.892 | -221.785 | 01.VI | 0.166 |
| 0.01555 | NA | NA | + | NA | NA | -3.93E-03 | 6 | 116.767 | -221.534 | 1.851 | 0.146 |
| 0.00545 | 4.52E-11 | NA | + | NA | NA | NA | 6 | 116.703 | -221.406 | 1.978 | 0.137 |
| 0.00254 | NA | NA | + | NA | NA | NA | 5 | 123.69 | -237.381 | 0 | 0.346 |
| 0.0261 | NA | NA | + | NA | -1.08E-02 | NA | 6 | 124.265 | -236.529 | 0.852 | 0.226 |
| 0.01048 | NA | NA | + | 5.05E-05 | NA | NA | 6 | 123.943 | -235.887 | 1.494 | 0.164 |
| 0.0013 | 7.40E-11 | NA | + | NA | NA | NA | 6 | 123.735 | -235.47 | 1.911 | 0.133 |
| -0.00281 | NA | NA | + | NA | NA | 2.23E-03 | 6 | 123.717 | -235.434 | 1.946 | 0.131 |
| 0.00783 | NA | NA | + | NA | NA | NA | 5 | 130.461 | -250.922 | 0 | 0.363 |
| 0.01782 | NA | NA | + | 5.92E-05 | NA | NA | 6 | 130.796 | -249.591 | 1.331 | 0.187 |
| 0.02618 | NA | NA | + | NA | -8.18E-03 | NA | 6 | 130.73 | -249.46 | 1.462 | 0.175 |
| 0.0067 | 6.99E-11 | NA | + | NA | NA | NA | 6 | 130.497 | -248.993 | 1.929 | 0.138 |
| 0.01295 | NA | NA | + | NA | NA | -2.14E-03 | 6 | 130.485 | -248.971 | 1.951 | 0.137 |
| 0.00398 | NA | NA | + | NA | NA | NA | 5 | 122.79 | -235.58 | 0 | 0.337 |
| 0.02873 | NA | NA | + | NA | -1.11E-02 | NA | 6 | 123.336 | -234.671 | 0.909 | 0.214 |
| 0.01527 | NA | NA | + | 6.68E-05 | NA | NA | 6 | 123.226 | -234.453 | 1.128 | 0.192 |
| 0.0023 | 9.95E-11 | NA | + | NA | NA | NA | 6 | 122.863 | -233.726 | 1.854 | 0.133 |
| 0.00109 | NA | NA | + | NA | NA | 1.21E-03 | 6 | 122.797 | -233.595 | 1.985 | 0.125 |
| 0.00175 | NA | NA | + | NA | NA | NA | 5 | 121.898 | -233.796 | 0 | 0.347 |
| 0.01307 | NA | NA | + | 6.92E-05 | NA | NA | 6 | 122.366 | -232.731 | 1.065 | 0.204 |
| 0.01836 | NA | NA | + | NA | -7.24E-03 | NA | 6 | 122.153 | -232.307 | 1.489 | 0.165 |
| -0.01092 | NA | NA | + | NA | NA | 5.48E-03 | 6 | 122.076 | -232.151 | 1.644 | 0.152 |
| 0.00013 | 8.96E-11 | NA | + | NA | NA | NA | 6 | 121.938 | -231.876 | 1.919 | 0.133 |
| 0.00241 | NA | NA | + | NA | NA | NA | 5 | 122.485 | -234.97 | 0 | 0.346 |
| 0.01616 | NA | NA | + | 7.83E-05 | NA | NA | 6 | 123.033 | -234.066 | 0.905 | 0.22 |
| 0.02187 | NA | NA | + | NA | -8.41E-03 | NA | 6 | 122.767 | -233.534 | 1.437 | 0.169 |
| 0.0003 | 1.27E-10 | NA | + | NA | NA | NA | 6 | 122.572 | -233.144 | 1.826 | 0.139 |
| 0.00141 | NA | NA | + | NA | NA | 4.24E-04 | 6 | 122.486 | -232.972 | 1.998 | 0.127 |
| 0.00311 | NA | NA | + | NA | NA | NA | 5 | 127.371 | -244.742 | 0 | 0.358 |
| 0.02452 | NA | NA | + | NA | -9.68E-03 | NA | 6 | 127.806 | -243.613 | 1.129 | 0.204 |
| 0.00982 | NA | NA | + | 4.57E-05 | NA | NA | 6 | 127.593 | -243.186 | 1.556 | 0.165 |
| -0.00367 | NA | NA | + | NA | NA | 2.81E-03 | 6 | 127.415 | -242.829 | 1.913 | 0.138 |
| 0.00222 | 5.42E-11 | NA | + | NA | NA | NA | 6 | 127.396 | -242.792 | I.95 | 0.135 |
| -0.00178 | NA | NA | + | NA | NA | NA | 5 | 118.162 | -226.324 | 0 | 0.326 |
| 0.01218 | NA | NA | + | 7.93E-05 | NA | NA | 6 | 118.738 | -225.476 | 0.848 | 0.213 |
| 0.02352 | NA | NA | + | NA | -1.13E-02 | NA | 6 | 118.71 | -225.421 | 0.903 | 0.207 |
| -0.00411 | 1.37E-10 | NA | + | NA | NA | NA | 6 | 118.274 | -224.548 | 1.776 | 0.134 |
| -0.00127 | NA | NA | + | NA | NA | -2.13E-04 | 6 | 118.162 | -224.325 | 2 | 0.12 |
| -0.00084 | NA | NA | + | NA | NA | NA | 5 | 119.856 | -229.711 | 0 | 0.245 |
| 0.0446 | NA | NA | NA | 1.51E-04 | NA | NA | 3 | 117.65 | -229.301 | 0.41 | 0.2 |
| 0.01546 | NA | NA | + | 8.42E-05 | NA | NA | 6 | 120.418 | -228.836 | 0.875 | 0.158 |
| 0.01803 | NA | NA | + | NA | -8.27E-03 | NA | 6 | 120.098 | -228.195 | 1.516 | 0.115 |
| 0.00807 | NA | NA | + | NA | NA | -3.79E-03 | 6 | 119.924 | -227.848 | 1.864 | 0.096 |
| -0.00234 | 8.34E-11 | NA | + | NA | NA | NA | 6 | 119.899 | -227.798 | 1.913 | 0.094 |
| 0.05958 | NA | NA | NA | 1.39E-04 | -7.94E-03 | NA | 4 | 117.875 | -227.75 | 1.962 | 0.092 |
| 0.00587 | NA | NA | + | NA | NA | NA | 5 | 125.026 | -240.053 | 0 | 0.346 |
| 0.02104 | NA | NA | + | 8.56E-05 | NA | NA | 6 | 125.63 | -239.261 | 0.792 | 0.233 |
| 0.02292 | NA | NA | + | NA | -7.66E-03 | NA | 6 | 125.24 | -238.481 | 1.572 | 0.158 |
| 0.00441 | 8.89E-11 | NA | + | NA | NA | NA | 6 | 125.081 | -238.162 | 1.891 | 0.135 |
| 0.00352 | NA | NA | + | NA | NA | 9.59E-04 | 6 | 125.031 | -238.063 | I.99 | 0.128 |
| 0.00425 | NA | NA | + | NA | NA | NA | 5 | 117.843 | -225.685 | 0 | 0.29 |
| 0.02406 | NA | NA | + | 1.01E-04 | NA | NA | 6 | 118.551 | -225.101 | 0.584 | 0.217 |
| 0.04654 | NA | NA | NA | 1.49E-04 | NA | NA | 3 | 115.235 | -224.47 | 1.215 | 0.158 |
| 0.01668 | NA | NA | + | NA | -5.48E-03 | NA | 6 | 117.934 | -223.868 | 1.817 | 0.117 |
| 0.00933 | NA | NA | + | NA | NA | -2.13E-03 | 6 | 117.864 | -223.729 | 1.956 | 0.109 |
| 0.00326 | 5.34E-11 | NA | + | NA | NA | NA | 6 | 117.863 | -223.725 | I.96 | 0.109 |
| 0.0492 | NA | NA | NA | 1.63E-04 | NA | NA | 3 | 118.939 | -231.877 | 0 | 0.197 |
| 0.00323 | NA | NA | + | NA | NA | NA | 5 | 120.91 | -231.819 | 0.058 | 0.191 |
| 0.02249 | NA | NA | + | 1.01E-04 | NA | NA | 6 | 121.698 | -231.395 | 0.482 | 0.155 |
| 0.01571 | NA | NA | + | NA | -5.51E-03 | NA | 6 | 121.009 | -230.019 | 1.859 | 0.078 |
| 0.05791 | NA | NA | NA | 1.57E-04 | -4.58E-03 | NA | 4 | 119.007 | -230.015 | 1.862 | 0.078 |
| 0.01344 | NA | NA | + | NA | NA | -4.38E-03 | 6 | 120.998 | -229.995 | 1.882 | 0.077 |
| 0.0592 | NA | NA | NA | 1.70E-04 | NA | -3.58E-03 | 4 | 118.996 | -229.993 | 1.885 | 0.077 |
| 0.00166 | 9.60E-11 | NA | + | NA | NA | NA | 6 | 120.976 | -229.953 | 1.924 | 0.075 |
| 0.04844 | 4.62E-11 | NA | NA | 1.63E-04 | NA | NA | 4 | 118.954 | -229.907 | I.97 | 0.073 |
| -0.00046 | NA | NA | + | NA | NA | NA | 5 | 125.965 | -241.93 | 0 | 0.342 |
| 0.02456 | NA | NA | + | NA | -1.09E-02 | NA | 6 | 126.491 | -240.982 | 0.948 | 0.213 |
| 0.01032 | NA | NA | + | 6.35E-05 | NA | NA | 6 | 126.354 | -240.709 | 1.221 | 0.186 |
| -0.00201 | 8.74E-11 | NA | + | NA | NA | NA | 6 | 126.028 | -240.056 | 1.873 | 0.134 |
| -0.0004 | NA | NA | + | NA | NA | -2.64E-05 | 6 | 125.965 | -239.93 | 2 | 0.126 |
| 0.00254 | NA | NA | + | NA | NA | NA | 5 | 125.535 | -241.071 | 0 | 0.367 |
| 0.02266 | NA | NA | + | NA | -8.82E-03 | NA | 6 | 125.886 | -239.773 | 1.298 | 0.192 |
| 0.0108 | NA | NA | + | 4.69E-05 | NA | NA | 6 | 125.745 | -239.491 | I.58 | 0.166 |
| 0.00118 | 7.96E-11 | NA | + | NA | NA | NA | 6 | 125.574 | -239.148 | 1.923 | 0.14 |
| 0.0045 | NA | NA | + | NA | NA | -8.52E-04 | 6 | 125.539 | -239.079 | 1.992 | 0.135 |
| 0.00476 | NA | NA | + | NA | NA | NA | 5 | 123.928 | -237.856 | 0 | 0.378 |
| 0.01551 | NA | NA | + | 5.99E-05 | NA | NA | 6 | 124.223 | -236.445 | I.41 | 0.187 |
| 0.01749 | NA | NA | + | NA | -5.61E-03 | NA | 6 | 124.041 | -236.081 | 1.775 | 0.156 |
| 0.00404 | 4.22E-11 | NA | + | NA | NA | NA | 6 | 123.941 | -235.882 | 1.974 | 0.141 |
| 0.00381 | NA | NA | + | NA | NA | 3.91E-04 | 6 | 123.929 | -235.857 | 1.998 | 0.139 |
| 0.00592 | NA | NA | + | NA | NA | NA | 5 | 123.111 | -236.221 | 0 | 0.368 |
| 0.0267 | NA | NA | + | NA | -9.19E-03 | NA | 6 | 123.442 | -234.885 | 1.336 | 0.189 |
| 0.01431 | NA | NA | + | 4.85E-05 | NA | NA | 6 | 123.322 | -234.643 | 1.578 | 0.167 |
| 0.00483 | 6.15E-11 | NA | + | NA | NA | NA | 6 | 123.139 | -234.277 | 1.944 | 0.139 |
| 0.00981 | NA | NA | + | NA | NA | -1.63E-03 | 6 | 123.125 | -234.25 | 1.971 | 0.137 |
| 0.00166 | NA | NA | + | NA | NA | NA | 5 | 127.865 | -245.73 | 0 | 0.359 |
| 0.01372 | NA | NA | + | 6.75E-05 | NA | NA | 6 | 128.278 | -244.556 | 1.174 | 0.2 |
| 0.02019 | NA | NA | + | NA | -8.05E-03 | NA | 6 | 128.131 | -244.261 | 1.469 | 0.172 |
| 0.00044 | 6.86E-11 | NA | + | NA | NA | NA | 6 | 127.898 | -243.797 | 1.933 | 0.137 |
| 0.00033 | NA | NA | + | NA | NA | 5.64E-04 | 6 | 127.867 | -243.734 | 1.996 | 0.132 |
| 0.00068 | NA | NA | + | NA | NA | NA | 5 | 125.883 | -241.766 | 0 | 0.352 |
| 0.02319 | NA | NA | + | NA | -9.98E-03 | NA | 6 | 126.328 | -240.655 | 01.XI | 0.202 |
| 0.01063 | NA | NA | + | 5.70E-05 | NA | NA | 6 | 126.208 | -240.416 | I.35 | 0.179 |
| -0.00125 | 1.05E-10 | NA | + | NA | NA | NA | 6 | 125.949 | -239.898 | 1.867 | 0.138 |
| -0.00048 | NA | NA | + | NA | NA | 4.92E-04 | 6 | 125.884 | -239.768 | 1.997 | 0.129 |
| 0.00179 | NA | NA | + | NA | NA | NA | 5 | 125.654 | -241.309 | 0 | 0.349 |
| 0.01335 | NA | NA | + | 6.61E-05 | NA | NA | 6 | 126.06 | -240.12 | 1.188 | 0.193 |
| 0.02302 | NA | NA | + | NA | -9.29E-03 | NA | 6 | 126.056 | -240.111 | 1.197 | 0.192 |
| 0.00038 | 8.10E-11 | NA | + | NA | NA | NA | 6 | 125.701 | -239.402 | 1.907 | 0.135 |
| -0.00279 | NA | NA | + | NA | NA | 1.91E-03 | 6 | 125.676 | -239.352 | 1.957 | 0.131 |
| -0.00112 | NA | NA | + | NA | NA | NA | 5 | 126.138 | -242.275 | 0 | 0.352 |
| 0.0212 | NA | NA | + | NA | -9.70E-03 | NA | 6 | 126.592 | -241.183 | 1.092 | 0.204 |
| 0.00812 | NA | NA | + | 5.61E-05 | NA | NA | 6 | 126.458 | -240.915 | I.36 | 0.178 |
| -0.00254 | 8.08E-11 | NA | + | NA | NA | NA | 6 | 126.188 | -240.376 | 01.IX | 0.136 |
| -0.00104 | NA | NA | + | NA | NA | -3.60E-05 | 6 | 126.138 | -240.275 | 2 | 0.13 |
| 0.00321 | NA | NA | + | NA | NA | NA | 5 | 129.645 | -249.29 | 0 | 0.361 |
| 0.01272 | NA | NA | + | 5.96E-05 | NA | NA | 6 | 129.995 | -247.99 | 1.301 | 0.188 |
| 0.02169 | NA | NA | + | NA | -8.18E-03 | NA | 6 | 129.949 | -247.898 | 1.392 | 0.18 |
| 0.00207 | 6.65E-11 | NA | + | NA | NA | NA | 6 | 129.681 | -247.362 | 1.928 | 0.138 |
| 0.00367 | NA | NA | + | NA | NA | -1.92E-04 | 6 | 129.645 | -247.291 | 2 | 0.133 |
| 0.00122 | NA | NA | + | NA | NA | NA | 5 | 117.857 | -225.714 | 0 | 0.353 |
| 0.01514 | NA | NA | + | 7.76E-05 | NA | NA | 6 | 118.347 | -224.695 | 1.019 | 0.212 |
| 0.02007 | NA | NA | + | NA | -8.24E-03 | NA | 6 | 118.125 | -224.25 | 1.464 | 0.17 |
| -0.00054 | 9.49E-11 | NA | + | NA | NA | NA | 6 | 117.905 | -223.81 | 1.904 | 0.136 |
| 0.0002 | NA | NA | + | NA | NA | 4.34E-04 | 6 | 117.858 | -223.716 | 1.998 | 0.13 |
| -0.00099 | NA | NA | + | NA | NA | NA | 5 | 128.527 | -247.053 | 0 | 0.338 |
| 0.01223 | NA | NA | + | 7.46E-05 | NA | NA | 6 | 129.081 | -246.162 | 0.891 | 0.217 |
| 0.02066 | NA | NA | + | NA | -9.49E-03 | NA | 6 | 128.94 | -245.881 | 1.172 | 0.188 |
| -0.0026 | 9.28E-11 | NA | + | NA | NA | NA | 6 | 128.588 | -245.176 | 1.877 | 0.132 |
| -0.00074 | NA | NA | + | NA | NA | -1.05E-04 | 6 | 128.527 | -245.053 | 2 | 0.124 |
| 0.00343 | NA | NA | + | NA | NA | NA | 5 | 121.569 | -233.138 | 0 | 0.359 |
| 0.01466 | NA | NA | + | 6.51E-05 | NA | NA | 6 | 121.924 | -231.849 | 1.289 | 0.189 |
| 0.02401 | NA | NA | + | NA | -9.08E-03 | NA | 6 | 121.871 | -231.743 | 1.395 | 0.179 |
| 0.01137 | NA | NA | + | NA | NA | -3.28E-03 | 6 | 121.622 | -231.245 | 1.893 | 0.139 |
| 0.00269 | 4.14E-11 | NA | + | NA | NA | NA | 6 | 121.583 | -231.166 | 1.972 | 0.134 |
| 0.0224 | NA | NA | + | 1.15E-04 | NA | NA | 6 | 119.021 | -226.042 | 0 | 0.21 |
| -0.00053 | NA | NA | + | NA | NA | NA | 5 | 117.95 | -225.901 | 0.141 | 0.196 |
| 0.04939 | NA | NA | NA | 1.69E-04 | NA | NA | 3 | 115.776 | -225.552 | 0.489 | 0.165 |
| 0.04254 | NA | NA | + | 1.24E-04 | NA | -7.92E-03 | 7 | 119.313 | -224.626 | 1.416 | 0.104 |
| 0.01261 | NA | NA | + | NA | NA | -5.64E-03 | 6 | 118.099 | -224.198 | 1.843 | 0.084 |
| 0.03298 | NA | NA | + | 1.13E-04 | -4.69E-03 | NA | 7 | 119.098 | -224.196 | 1.846 | 0.084 |
| 0.01292 | NA | NA | + | NA | -5.75E-03 | NA | 6 | 118.065 | -224.131 | 1.911 | 0.081 |
| 0.02237 | 1.14E-12 | NA | + | 1.15E-04 | NA | NA | 7 | 119.021 | -224.042 | 2 | 0.077 |
| 0.00158 | NA | NA | + | NA | NA | NA | 5 | 126.99 | -243.979 | 0 | 0.364 |
| 0.02391 | NA | NA | + | NA | -9.85E-03 | NA | 6 | 127.419 | -242.839 | I.14 | 0.206 |
| 0.00861 | NA | NA | + | 4.19E-05 | NA | NA | 6 | 127.163 | -242.325 | 1.654 | 0.159 |
| 0.0007 | 4.85E-11 | NA | + | NA | NA | NA | 6 | 127.013 | -242.025 | 1.954 | 0.137 |
| 0.00051 | NA | NA | + | NA | NA | 4.50E-04 | 6 | 126.991 | -241.981 | 1.998 | 0.134 |
| 0.0085 | NA | NA | + | NA | NA | NA | 5 | 126.975 | -243.951 | 0 | 0.303 |
| 0.0223 | NA | NA | + | 7.98E-05 | NA | NA | 6 | 127.561 | -243.123 | 0.828 | 0.2 |
| 0.02535 | NA | NA | + | NA | -7.42E-03 | NA | 6 | 127.195 | -242.391 | I.56 | 0.139 |
| 0.04883 | NA | NA | NA | 1.37E-04 | NA | NA | 3 | 124.116 | -242.232 | 1.718 | 0.128 |
| 0.00737 | 6.63E-11 | NA | + | NA | NA | NA | 6 | 127.011 | -242.022 | 1.928 | 0.115 |
| 0.01381 | NA | NA | + | NA | NA | -2.22E-03 | 6 | 127 | -241.999 | 1.951 | 0.114 |
| 0.00447 | NA | NA | + | NA | NA | NA | 5 | 125.485 | -240.969 | 0 | 0.3 |
| 0.01758 | NA | NA | + | 7.15E-05 | NA | NA | 6 | 125.926 | -239.852 | 1.117 | 0.172 |
| 0.04863 | NA | NA | NA | 1.43E-04 | NA | NA | 3 | 122.889 | -239.777 | 1.192 | 0.165 |
| 0.02218 | NA | NA | + | NA | -7.76E-03 | NA | 6 | 125.712 | -239.425 | 1.544 | 0.139 |
| 0.00338 | 6.47E-11 | NA | + | NA | NA | NA | 6 | 125.518 | -239.037 | 1.932 | 0.114 |
| 0.0066 | NA | NA | + | NA | NA | -9.03E-04 | 6 | 125.489 | -238.978 | 1.991 | 0.111 |
| 0.00467 | NA | NA | + | NA | NA | NA | 5 | 124.499 | -238.997 | 0 | 0.369 |
| 0.0257 | NA | NA | + | NA | -9.33E-03 | NA | 6 | 124.843 | -237.686 | 1.311 | 0.192 |
| 0.0126 | NA | NA | + | 4.34E-05 | NA | NA | 6 | 124.67 | -237.34 | 1.657 | 0.161 |
| 0.01027 | NA | NA | + | NA | NA | -2.42E-03 | 6 | 124.529 | -237.057 | I.94 | 0.14 |
| 0.00376 | 4.67E-11 | NA | + | NA | NA | NA | 6 | 124.511 | -237.022 | 1.975 | 0.138 |
| 0.00437 | NA | NA | + | NA | NA | NA | 5 | 121.92 | -233.841 | 0 | 0.35 |
| 0.019 | NA | NA | + | 8.09E-05 | NA | NA | 6 | 122.478 | -232.955 | 0.886 | 0.225 |
| 0.02171 | NA | NA | + | NA | -7.65E-03 | NA | 6 | 122.131 | -232.262 | 1.579 | 0.159 |
| 0.00266 | 9.99E-11 | NA | + | NA | NA | NA | 6 | 121.968 | -231.936 | 1.905 | 0.135 |
| 0.00984 | NA | NA | + | NA | NA | -2.31E-03 | 6 | 121.949 | -231.897 | 1.944 | 0.132 |
| 0.00449 | NA | NA | + | NA | NA | NA | 5 | 120.137 | -230.274 | 0 | 0.341 |
| 0.01876 | NA | NA | + | 8.10E-05 | NA | NA | 6 | 120.721 | -229.441 | 0.832 | 0.225 |
| 0.02496 | NA | NA | + | NA | -8.99E-03 | NA | 6 | 120.475 | -228.95 | 1.323 | 0.176 |
| 0.01007 | NA | NA | + | NA | NA | -2.39E-03 | 6 | 120.165 | -228.329 | 1.944 | 0.129 |
| 0.00316 | 7.58E-11 | NA | + | NA | NA | NA | 6 | 120.164 | -228.328 | 1.945 | 0.129 |
| 0.00395 | NA | NA | + | NA | NA | NA | 5 | 126.729 | -243.459 | 0 | 0.359 |
| 0.0151 | NA | NA | + | 6.70E-05 | NA | NA | 6 | 127.147 | -242.294 | 1.165 | 0.201 |
| 0.02155 | NA | NA | + | NA | -7.79E-03 | NA | 6 | 126.978 | -241.957 | 1.502 | 0.169 |
| 0.00269 | 7.20E-11 | NA | + | NA | NA | NA | 6 | 126.763 | -241.527 | 1.932 | 0.137 |
| 0.00012 | NA | NA | + | NA | NA | 1.63E-03 | 6 | 126.744 | -241.488 | 1.971 | 0.134 |
| 0.00253 | NA | NA | + | NA | NA | NA | 5 | 121.246 | -232.492 | 0 | 0.349 |
| 0.0257 | NA | NA | + | NA | -1.01E-02 | NA | 6 | 121.675 | -231.351 | 1.141 | 0.197 |
| 0.01326 | NA | NA | + | 6.04E-05 | NA | NA | 6 | 121.566 | -231.131 | 1.361 | 0.177 |
| 0.00005 | 1.36E-10 | NA | + | NA | NA | NA | 6 | 121.343 | -230.687 | 1.805 | 0.142 |
| -0.0042 | NA | NA | + | NA | NA | 2.90E-03 | 6 | 121.292 | -230.584 | 1.908 | 0.135 |
| -0.00002 | NA | NA | + | NA | NA | NA | 5 | 120.316 | -230.633 | 0 | 0.374 |
| 0.01235 | NA | NA | + | 6.78E-05 | NA | NA | 6 | 120.692 | -229.384 | 1.249 | 0.2 |
| 0.01061 | NA | NA | + | NA | -4.65E-03 | NA | 6 | 120.391 | -228.782 | 1.851 | 0.148 |
| -0.00119 | 6.36E-11 | NA | + | NA | NA | NA | 6 | 120.337 | -228.674 | 1.959 | 0.14 |
| 0.00164 | NA | NA | + | NA | NA | -7.19E-04 | 6 | 120.319 | -228.638 | 1.994 | 0.138 |
| 0.00185 | NA | NA | + | NA | NA | NA | 5 | 126.045 | -242.09 | 0 | 0.328 |
| 0.02772 | NA | NA | + | NA | -1.15E-02 | NA | 6 | 126.723 | -241.445 | 0.645 | 0.238 |
| 0.01089 | NA | NA | + | 5.76E-05 | NA | NA | 6 | 126.401 | -240.801 | 1.289 | 0.172 |
| -0.00745 | NA | NA | + | NA | NA | 3.91E-03 | 6 | 126.131 | -240.262 | 1.828 | 0.132 |
| -0.0001 | 1.14E-10 | NA | + | NA | NA | NA | 6 | 126.123 | -240.246 | 1.845 | 0.13 |
| -0.00181 | NA | NA | + | NA | NA | NA | 5 | 121.693 | -233.386 | 0 | 0.197 |
| 0.01694 | NA | NA | + | 9.87E-05 | NA | NA | 6 | 122.505 | -233.01 | 0.377 | 0.163 |
| 0.04594 | NA | NA | NA | 1.59E-04 | NA | NA | 3 | 119.441 | -232.882 | 0.504 | 0.153 |
| 0.02118 | NA | NA | + | NA | -9.98E-03 | NA | 6 | 122.039 | -232.079 | 1.308 | 0.102 |
| 0.00951 | NA | NA | + | NA | NA | -4.78E-03 | 6 | 121.802 | -231.604 | 1.782 | 0.081 |
| 0.03598 | NA | NA | + | 9.37E-05 | -8.68E-03 | NA | 7 | 122.767 | -231.534 | 1.852 | 0.078 |
| 0.06365 | NA | NA | NA | 1.46E-04 | -9.30E-03 | NA | 4 | 119.743 | -231.487 | 01.IX | 0.076 |
| 0.0354 | NA | NA | + | 1.07E-04 | NA | -7.09E-03 | 7 | 122.741 | -231.482 | 1.904 | 0.076 |
| -0.00295 | 6.25E-11 | NA | + | NA | NA | NA | 6 | 121.725 | -231.45 | 1.936 | 0.075 |
| 0.00217 | NA | NA | + | NA | NA | NA | 5 | 121.442 | -232.885 | 0 | 0.359 |
| 0.02333 | NA | NA | + | NA | -9.68E-03 | NA | 6 | 121.886 | -231.772 | 1.113 | 0.206 |
| 0.00806 | NA | NA | + | 3.95E-05 | NA | NA | 6 | 121.593 | -231.185 | 01.VII | 0.154 |
| -0.00697 | NA | NA | + | NA | NA | 3.76E-03 | 6 | 121.519 | -231.038 | 1.847 | 0.143 |
| 0.00071 | 8.49E-11 | NA | + | NA | NA | NA | 6 | 121.491 | -230.983 | 1.902 | 0.139 |
| 0.00019 | NA | NA | + | NA | NA | NA | 5 | 122.004 | -234.008 | 0 | 0.331 |
| 0.01703 | NA | NA | + | 9.38E-05 | NA | NA | 6 | 122.81 | -233.62 | 0.388 | 0.272 |
| 0.01471 | NA | NA | + | NA | -6.40E-03 | NA | 6 | 122.185 | -232.37 | 1.638 | 0.146 |
| -0.0019 | 1.19E-10 | NA | + | NA | NA | NA | 6 | 122.067 | -232.135 | 1.874 | 0.13 |
| -0.00111 | NA | NA | + | NA | NA | 5.57E-04 | 6 | 122.006 | -232.012 | 1.997 | 0.122 |
| 0.00175 | NA | NA | + | NA | NA | NA | 5 | 123.965 | -237.931 | 0 | 0.292 |
| 0.01848 | NA | NA | + | 9.17E-05 | NA | NA | 6 | 124.685 | -237.369 | 0.562 | 0.221 |
| 0.02016 | NA | NA | + | NA | -7.98E-03 | NA | 6 | 124.183 | -236.367 | 1.564 | 0.134 |
| 0.04814 | NA | NA | NA | 1.58E-04 | NA | NA | 3 | 121.126 | -236.251 | I.68 | 0.126 |
| 0.00961 | NA | NA | + | NA | NA | -3.31E-03 | 6 | 124.019 | -236.038 | 1.892 | 0.114 |
| 0.00025 | 8.96E-11 | NA | + | NA | NA | NA | 6 | 124.016 | -236.033 | 1.898 | 0.113 |
| 0.00379 | NA | NA | + | NA | NA | NA | 5 | 131.523 | -253.047 | 0 | 0.333 |
| 0.01669 | NA | NA | + | 7.44E-05 | NA | NA | 6 | 132.117 | -252.234 | 0.813 | 0.222 |
| 0.02487 | NA | NA | + | NA | -9.30E-03 | NA | 6 | 131.958 | -251.915 | 1.131 | 0.189 |
| 0.00208 | 1.09E-10 | NA | + | NA | NA | NA | 6 | 131.603 | -251.206 | 1.841 | 0.133 |
| 0.00406 | NA | NA | + | NA | NA | -1.13E-04 | 6 | 131.523 | -251.047 | 2 | 0.123 |
| 0.00431 | NA | NA | + | NA | NA | NA | 5 | 126.79 | -243.579 | 0 | 0.347 |
| 0.01704 | NA | NA | + | 7.76E-05 | NA | NA | 6 | 127.347 | -242.695 | 0.885 | 0.223 |
| 0.02202 | NA | NA | + | NA | -7.85E-03 | NA | 6 | 127.057 | -242.114 | 1.465 | 0.167 |
| 0.00277 | 8.78E-11 | NA | + | NA | NA | NA | 6 | 126.842 | -241.685 | 1.895 | 0.135 |
| 0.00222 | NA | NA | + | NA | NA | 8.77E-04 | 6 | 126.794 | -241.587 | 1.992 | 0.128 |
| 0.00895 | NA | NA | + | NA | NA | NA | 5 | 127.908 | -245.816 | 0 | 0.265 |
| 0.02594 | NA | NA | + | 9.26E-05 | NA | NA | 6 | 128.657 | -245.315 | 0.501 | 0.206 |
| 0.04962 | NA | NA | NA | 1.47E-04 | NA | NA | 3 | 125.644 | -245.289 | 0.527 | 0.203 |
| 0.02693 | NA | NA | + | NA | -7.98E-03 | NA | 6 | 128.137 | -244.274 | 1.542 | 0.122 |
| 0.00757 | 8.44E-11 | NA | + | NA | NA | NA | 6 | 127.961 | -243.921 | 1.895 | 0.103 |
| 0.01545 | NA | NA | + | NA | NA | -2.68E-03 | 6 | 127.945 | -243.891 | 1.925 | 0.101 |
| 0.004 | NA | NA | + | NA | NA | NA | 5 | 127.625 | -245.25 | 0 | 0.361 |
| 0.02419 | NA | NA | + | NA | -8.85E-03 | NA | 6 | 127.963 | -243.926 | 1.324 | 0.186 |
| 0.01315 | NA | NA | + | 5.51E-05 | NA | NA | 6 | 127.921 | -243.843 | 1.407 | 0.179 |
| -0.00176 | NA | NA | + | NA | NA | 2.42E-03 | 6 | 127.658 | -243.317 | 1.933 | 0.137 |
| 0.00303 | 5.83E-11 | NA | + | NA | NA | NA | 6 | 127.658 | -243.317 | 1.933 | 0.137 |
| 0.00122 | NA | NA | + | NA | NA | NA | 5 | 118.357 | -226.714 | 0 | 0.349 |
| 0.01364 | NA | NA | + | 7.01E-05 | NA | NA | 6 | 118.757 | -225.513 | 1.201 | 0.191 |
| 0.02438 | NA | NA | + | NA | -1.02E-02 | NA | 6 | 118.717 | -225.434 | I.28 | 0.184 |
| 0.0112 | NA | NA | + | NA | NA | -4.15E-03 | 6 | 118.436 | -224.872 | 1.843 | 0.139 |
| -0.00066 | 1.09E-10 | NA | + | NA | NA | NA | 6 | 118.419 | -224.838 | 1.876 | 0.137 |
| 0.00112 | NA | NA | + | NA | NA | NA | 5 | 120.364 | -230.728 | 0 | 0.37 |
| 0.02225 | NA | NA | + | NA | -9.20E-03 | NA | 6 | 120.676 | -229.352 | 1.376 | 0.186 |
| 0.01033 | NA | NA | + | 5.12E-05 | NA | NA | 6 | 120.582 | -229.164 | 1.564 | 0.169 |
| 0.00495 | NA | NA | + | NA | NA | -1.62E-03 | 6 | 120.378 | -228.755 | 1.973 | 0.138 |
| 0.00038 | 3.97E-11 | NA | + | NA | NA | NA | 6 | 120.376 | -228.752 | 1.975 | 0.138 |
| 0.0017 | NA | NA | + | NA | NA | NA | 5 | 122.521 | -235.042 | 0 | 0.328 |
| 0.02859 | NA | NA | + | NA | -1.19E-02 | NA | 6 | 123.101 | -234.202 | 0.84 | 0.216 |
| 0.01555 | NA | NA | + | 7.68E-05 | NA | NA | 6 | 123.047 | -234.094 | 0.948 | 0.204 |
| -0.00048 | 1.28E-10 | NA | + | NA | NA | NA | 6 | 122.603 | -233.205 | 1.837 | 0.131 |
| 0.0039 | NA | NA | + | NA | NA | -9.20E-04 | 6 | 122.525 | -233.051 | 1.991 | 0.121 |
| 0.00288 | NA | NA | + | NA | NA | NA | 5 | 118.265 | -226.53 | 0 | 0.315 |
| 0.01705 | NA | NA | + | 7.91E-05 | NA | NA | 6 | 118.737 | -225.474 | 1.056 | 0.186 |
| 0.0201 | NA | NA | + | NA | -7.70E-03 | NA | 6 | 118.474 | -224.949 | 1.581 | 0.143 |
| 0.00116 | 9.45E-11 | NA | + | NA | NA | NA | 6 | 118.304 | -224.607 | 1.922 | 0.12 |
| 0.04363 | NA | NA | NA | 1.42E-04 | NA | NA | 3 | 115.294 | -224.588 | 1.942 | 0.119 |
| 0.00552 | NA | NA | + | NA | NA | -1.09E-03 | 6 | 118.27 | -224.541 | 1.989 | 0.117 |
| 0.00353 | NA | NA | + | NA | NA | NA | 5 | 123.216 | -236.432 | 0 | 0.358 |
| 0.0153 | NA | NA | + | 6.68E-05 | NA | NA | 6 | 123.601 | -235.202 | I.23 | 0.194 |
| 0.0233 | NA | NA | + | NA | -8.67E-03 | NA | 6 | 123.494 | -234.989 | 1.443 | 0.174 |
| 0.00208 | 8.05E-11 | NA | + | NA | NA | NA | 6 | 123.256 | -234.512 | I.92 | 0.137 |
| 0.00996 | NA | NA | + | NA | NA | -2.75E-03 | 6 | 123.253 | -234.506 | 1.926 | 0.137 |
| 0.00452 | NA | NA | + | NA | NA | NA | 5 | 128.164 | -246.327 | 0 | 0.371 |
| 0.01426 | NA | NA | + | 5.76E-05 | NA | NA | 6 | 128.479 | -244.959 | 1.368 | 0.187 |
| 0.0197 | NA | NA | + | NA | -6.75E-03 | NA | 6 | 128.353 | -244.706 | 1.621 | 0.165 |
| 0.00345 | 6.35E-11 | NA | + | NA | NA | NA | 6 | 128.194 | -244.387 | I.94 | 0.141 |
| 0.00636 | NA | NA | + | NA | NA | -7.76E-04 | 6 | 128.167 | -244.334 | 1.994 | 0.137 |
| 0.00372 | NA | NA | + | NA | NA | NA | 5 | 123.688 | -237.376 | 0 | 0.363 |
| 0.0249 | NA | NA | + | NA | -9.21E-03 | NA | 6 | 124.002 | -236.003 | 1.373 | 0.183 |
| 0.01523 | NA | NA | + | 6.12E-05 | NA | NA | 6 | 124.001 | -236.002 | 1.374 | 0.183 |
| 0.00291 | 4.71E-11 | NA | + | NA | NA | NA | 6 | 123.708 | -235.417 | 1.959 | 0.136 |
| -0.00029 | NA | NA | + | NA | NA | 1.73E-03 | 6 | 123.705 | -235.409 | 1.967 | 0.136 |
| 0.00494 | NA | NA | + | NA | NA | NA | 5 | 126.048 | -242.096 | 0 | 0.359 |
| 0.02727 | NA | NA | + | NA | -9.75E-03 | NA | 6 | 126.423 | -240.845 | 1.251 | 0.192 |
| 0.01587 | NA | NA | + | 5.97E-05 | NA | NA | 6 | 126.362 | -240.724 | 1.372 | 0.181 |
| 0.00361 | 7.49E-11 | NA | + | NA | NA | NA | 6 | 126.084 | -240.168 | 1.929 | 0.137 |
| 0.00696 | NA | NA | + | NA | NA | -8.65E-04 | 6 | 126.052 | -240.105 | 1.992 | 0.132 |
| 0.00194 | NA | NA | + | NA | NA | NA | 5 | 130.009 | -250.017 | 0 | 0.357 |
| 0.02387 | NA | NA | + | NA | -9.79E-03 | NA | 6 | 130.532 | -249.063 | 0.954 | 0.222 |
| 0.0062 | NA | NA | + | 2.81E-05 | NA | NA | 6 | 130.097 | -248.195 | 1.823 | 0.144 |
| 0.00029 | 9.90E-11 | NA | + | NA | NA | NA | 6 | 130.072 | -248.143 | 1.874 | 0.14 |
| -0.00508 | NA | NA | + | NA | NA | 2.95E-03 | 6 | 130.058 | -248.116 | 1.901 | 0.138 |
| 0.00349 | NA | NA | + | NA | NA | NA | 5 | 125.748 | -241.496 | 0 | 0.36 |
| 0.02629 | NA | NA | + | NA | -1.02E-02 | NA | 6 | 126.144 | -240.289 | 1.207 | 0.197 |
| 0.01327 | NA | NA | + | 5.45E-05 | NA | NA | 6 | 126.016 | -240.033 | 1.464 | 0.173 |
| 0.00234 | 6.72E-11 | NA | + | NA | NA | NA | 6 | 125.781 | -239.562 | 1.934 | 0.137 |
| 0.00528 | NA | NA | + | NA | NA | -7.46E-04 | 6 | 125.751 | -239.502 | 1.994 | 0.133 |
| 0.00002 | NA | NA | + | NA | NA | NA | 5 | 131.919 | -253.838 | 0 | 0.344 |
| 0.01212 | NA | NA | + | 6.80E-05 | NA | NA | 6 | 132.367 | -252.734 | 1.104 | 0.198 |
| 0.02296 | NA | NA | + | NA | -1.01E-02 | NA | 6 | 132.365 | -252.73 | 1.109 | 0.197 |
| 0.00641 | NA | NA | + | NA | NA | -2.76E-03 | 6 | 131.959 | -251.917 | 1.921 | 0.131 |
| -0.00101 | 5.93E-11 | NA | + | NA | NA | NA | 6 | 131.946 | -251.893 | 1.945 | 0.13 |
| 0.00043 | NA | NA | + | NA | NA | NA | 5 | 127.582 | -245.165 | 0 | 0.327 |
| 0.0268 | NA | NA | + | NA | -1.17E-02 | NA | 6 | 128.286 | -244.573 | 0.592 | 0.243 |
| 0.01094 | NA | NA | + | 5.98E-05 | NA | NA | 6 | 127.96 | -243.92 | 1.244 | 0.175 |
| -0.00204 | 1.41E-10 | NA | + | NA | NA | NA | 6 | 127.682 | -243.365 | 01.VIII | 0.133 |
| -0.00274 | NA | NA | + | NA | NA | 1.34E-03 | 6 | 127.594 | -243.187 | 1.978 | 0.122 |
| 0.00485 | NA | NA | + | NA | NA | NA | 5 | 121.317 | -232.635 | 0 | 0.311 |
| 0.0182 | NA | NA | + | 7.83E-05 | NA | NA | 6 | 121.837 | -231.674 | 0.96 | 0.192 |
| 0.04768 | NA | NA | NA | 1.43E-04 | NA | NA | 3 | 118.475 | -230.949 | 1.686 | 0.134 |
| 0.01866 | NA | NA | + | NA | -6.06E-03 | NA | 6 | 121.449 | -230.899 | 1.736 | 0.131 |
| 0.00365 | 7.00E-11 | NA | + | NA | NA | NA | 6 | 121.34 | -230.68 | 1.954 | 0.117 |
| 0.00142 | NA | NA | + | NA | NA | 1.43E-03 | 6 | 121.328 | -230.656 | 1.978 | 0.116 |
| 0.00628 | NA | NA | + | NA | NA | NA | 5 | 116.126 | -222.253 | 0 | 0.303 |
| 0.02175 | NA | NA | + | 9.00E-05 | NA | NA | 6 | 116.751 | -221.501 | 0.751 | 0.208 |
| 0.02107 | NA | NA | + | NA | -6.72E-03 | NA | 6 | 116.282 | -220.565 | 1.688 | 0.13 |
| 0.0466 | NA | NA | NA | 1.52E-04 | NA | NA | 3 | 113.231 | -220.462 | 1.791 | 0.124 |
| 0.00461 | 1.01E-10 | NA | + | NA | NA | NA | 6 | 116.18 | -220.361 | 1.892 | 0.118 |
| 0.01384 | NA | NA | + | NA | NA | -3.06E-03 | 6 | 116.168 | -220.336 | 1.917 | 0.116 |
| 0.00556 | NA | NA | + | NA | NA | NA | 5 | 124.456 | -238.911 | 0 | 0.317 |
| 0.01915 | NA | NA | + | 7.37E-05 | NA | NA | 6 | 124.9 | -237.801 | 01.XI | 0.182 |
| 0.02256 | NA | NA | + | NA | -7.65E-03 | NA | 6 | 124.669 | -237.337 | 1.574 | 0.144 |
| 0.0109 | NA | NA | + | NA | NA | -2.21E-03 | 6 | 124.483 | -236.965 | 1.946 | 0.12 |
| 0.00441 | 6.47E-11 | NA | + | NA | NA | NA | 6 | 124.476 | -236.953 | 1.958 | 0.119 |
| 0.04456 | NA | NA | NA | 1.39E-04 | NA | NA | 3 | 121.457 | -236.914 | 1.997 | 0.117 |
| 0.00528 | NA | NA | + | NA | NA | NA | 5 | 128.618 | -247.237 | 0 | 0.299 |
| 0.0203 | NA | NA | + | 7.74E-05 | NA | NA | 6 | 129.144 | -246.289 | 0.948 | 0.186 |
| 0.04831 | NA | NA | NA | 1.42E-04 | NA | NA | 3 | 125.887 | -245.774 | 1.463 | 0.144 |
| 0.02411 | NA | NA | + | NA | -8.22E-03 | NA | 6 | 128.875 | -245.751 | 1.486 | 0.142 |
| 0.01207 | NA | NA | + | NA | NA | -2.88E-03 | 6 | 128.664 | -245.328 | 1.908 | 0.115 |
| 0.00459 | 4.02E-11 | NA | + | NA | NA | NA | 6 | 128.638 | -245.275 | 1.961 | 0.112 |
| 0.00139 | NA | NA | + | NA | NA | NA | 5 | 124.908 | -239.816 | 0 | 0.349 |
| 0.01582 | NA | NA | + | 7.91E-05 | NA | NA | 6 | 125.461 | -238.921 | 0.895 | 0.223 |
| 0.01738 | NA | NA | + | NA | -6.98E-03 | NA | 6 | 125.107 | -238.214 | 1.603 | 0.157 |
| -0.00088 | 1.30E-10 | NA | + | NA | NA | NA | 6 | 124.992 | -237.984 | 1.832 | 0.14 |
| -0.0034 | NA | NA | + | NA | NA | 2.01E-03 | 6 | 124.93 | -237.86 | 1.957 | 0.131 |
| 0.00684 | NA | NA | + | NA | NA | NA | 5 | 129.125 | -248.25 | 0 | 0.368 |
| 0.01651 | NA | NA | + | 5.53E-05 | NA | NA | 6 | 129.398 | -246.797 | 1.453 | 0.178 |
| 0.02461 | NA | NA | + | NA | -8.04E-03 | NA | 6 | 129.386 | -246.773 | 1.477 | 0.176 |
| 0.01404 | NA | NA | + | NA | NA | -3.00E-03 | 6 | 129.171 | -246.343 | 1.907 | 0.142 |
| 0.00622 | 3.64E-11 | NA | + | NA | NA | NA | 6 | 129.136 | -246.272 | 1.978 | 0.137 |
| 0.00162 | NA | NA | + | NA | NA | NA | 5 | 125.291 | -240.581 | 0 | 0.349 |
| 0.024 | NA | NA | + | NA | -9.78E-03 | NA | 6 | 125.733 | -239.466 | 1.116 | 0.2 |
| 0.01209 | NA | NA | + | 6.00E-05 | NA | NA | 6 | 125.635 | -239.27 | 1.312 | 0.181 |
| -0.00056 | 1.34E-10 | NA | + | NA | NA | NA | 6 | 125.382 | -238.763 | 1.818 | 0.141 |
| 0.00375 | NA | NA | + | NA | NA | -9.12E-04 | 6 | 125.295 | -238.59 | 1.992 | 0.129 |
| 0.00391 | NA | NA | + | NA | NA | NA | 5 | 127.182 | -244.363 | 0 | 0.338 |
| 0.01759 | NA | NA | + | 7.82E-05 | NA | NA | 6 | 127.741 | -243.482 | 0.882 | 0.217 |
| 0.02599 | NA | NA | + | NA | -9.78E-03 | NA | 6 | 127.556 | -243.111 | 1.252 | 0.181 |
| 0.01345 | NA | NA | + | NA | NA | -3.95E-03 | 6 | 127.257 | -242.515 | 1.849 | 0.134 |
| 0.00256 | 8.19E-11 | NA | + | NA | NA | NA | 6 | 127.225 | -242.45 | 1.914 | 0.13 |
| 0.00346 | NA | NA | + | NA | NA | NA | 5 | 123.413 | -236.826 | 0 | 0.278 |
| 0.01973 | NA | NA | + | 9.34E-05 | NA | NA | 6 | 124.17 | -236.34 | 0.486 | 0.218 |
| 0.04758 | NA | NA | NA | 1.57E-04 | NA | NA | 3 | 120.86 | -235.72 | 1.106 | 0.16 |
| 0.02048 | NA | NA | + | NA | -7.52E-03 | NA | 6 | 123.617 | -235.234 | 1.592 | 0.125 |
| 0.0141 | NA | NA | + | NA | NA | -4.36E-03 | 6 | 123.507 | -235.013 | 1.813 | 0.112 |
| 0.00197 | 8.98E-11 | NA | + | NA | NA | NA | 6 | 123.459 | -234.918 | 1.908 | 0.107 |
| -0.00043 | NA | NA | + | NA | NA | NA | 5 | 120.608 | -231.216 | 0 | 0.337 |
| 0.02723 | NA | NA | + | NA | -1.22E-02 | NA | 6 | 121.321 | -230.643 | 0.573 | 0.253 |
| 0.007 | NA | NA | + | 4.78E-05 | NA | NA | 6 | 120.837 | -229.674 | 1.542 | 0.156 |
| -0.00209 | 9.69E-11 | NA | + | NA | NA | NA | 6 | 120.665 | -229.329 | 1.887 | 0.131 |
| -0.00026 | NA | NA | + | NA | NA | -7.56E-05 | 6 | 120.608 | -229.216 | 2 | 0.124 |
| 0.00465 | NA | NA | + | NA | NA | NA | 5 | 123.551 | -237.103 | 0 | 0.304 |
| 0.01997 | NA | NA | + | 8.36E-05 | NA | NA | 6 | 124.109 | -236.218 | 0.885 | 0.195 |
| 0.04726 | NA | NA | NA | 1.45E-04 | NA | NA | 3 | 120.747 | -235.493 | 1.609 | 0.136 |
| 0.02037 | NA | NA | + | NA | -6.89E-03 | NA | 6 | 123.715 | -235.429 | 1.674 | 0.132 |
| 0.01202 | NA | NA | + | NA | NA | -3.10E-03 | 6 | 123.599 | -235.197 | 1.905 | 0.117 |
| 0.00322 | 7.97E-11 | NA | + | NA | NA | NA | 6 | 123.593 | -235.186 | 1.917 | 0.116 |
| 0.00589 | NA | NA | + | NA | NA | NA | 5 | 124.712 | -239.424 | 0 | 0.353 |
| 0.02856 | NA | NA | + | NA | -1.01E-02 | NA | 6 | 125.124 | -238.249 | 1.175 | 0.196 |
| 0.01578 | NA | NA | + | 6.05E-05 | NA | NA | 6 | 125.044 | -238.087 | 1.336 | 0.181 |
| 0.00481 | 6.76E-11 | NA | + | NA | NA | NA | 6 | 124.749 | -237.498 | 1.926 | 0.135 |
| 0.01259 | NA | NA | + | NA | NA | -2.74E-03 | 6 | 124.749 | -237.498 | 1.926 | 0.135 |
| 0.00696 | NA | NA | + | NA | NA | NA | 5 | 127.025 | -244.051 | 0 | 0.291 |
| 0.02264 | NA | NA | + | 9.03E-05 | NA | NA | 6 | 127.785 | -243.57 | 0.481 | 0.229 |
| 0.05027 | NA | NA | NA | 1.54E-04 | NA | NA | 3 | 124.267 | -242.533 | 1.517 | 0.136 |
| 0.02158 | NA | NA | + | NA | -6.42E-03 | NA | 6 | 127.185 | -242.369 | 1.681 | 0.125 |
| 0.01248 | NA | NA | + | NA | NA | -2.30E-03 | 6 | 127.051 | -242.102 | 1.949 | 0.11 |
| 0.00624 | 4.51E-11 | NA | + | NA | NA | NA | 6 | 127.047 | -242.094 | 1.957 | 0.109 |
| -0.00059 | NA | NA | + | NA | NA | NA | 5 | 125.607 | -241.215 | 0 | 0.331 |
| 0.02696 | NA | NA | + | NA | -1.24E-02 | NA | 6 | 126.418 | -240.836 | 0.379 | 0.274 |
| 0.00478 | NA | NA | + | 3.43E-05 | NA | NA | 6 | 125.733 | -239.467 | 1.748 | 0.138 |
| -0.00817 | NA | NA | + | NA | NA | 3.15E-03 | 6 | 125.664 | -239.328 | 1.886 | 0.129 |
| -0.00191 | 7.71E-11 | NA | + | NA | NA | NA | 6 | 125.647 | -239.295 | I.92 | 0.127 |
| 0.00463 | NA | NA | + | NA | NA | NA | 5 | 129.442 | -248.883 | 0 | 0.358 |
| 0.02714 | NA | NA | + | NA | -1.00E-02 | NA | 6 | 129.875 | -247.75 | 1.133 | 0.203 |
| 0.01341 | NA | NA | + | 5.05E-05 | NA | NA | 6 | 129.692 | -247.383 | 01.V | 0.169 |
| 0.00377 | 4.97E-11 | NA | + | NA | NA | NA | 6 | 129.465 | -246.929 | 1.954 | 0.135 |
| 0.00922 | NA | NA | + | NA | NA | -1.95E-03 | 6 | 129.462 | -246.923 | I.96 | 0.134 |
| 0.00306 | NA | NA | + | NA | NA | NA | 5 | 125.115 | -240.23 | 0 | 0.362 |
| 0.02457 | NA | NA | + | NA | -9.52E-03 | NA | 6 | 125.461 | -238.923 | 1.307 | 0.188 |
| 0.01333 | NA | NA | + | 5.57E-05 | NA | NA | 6 | 125.388 | -238.776 | 1.454 | 0.175 |
| 0.00922 | NA | NA | + | NA | NA | -2.63E-03 | 6 | 125.149 | -238.299 | 1.931 | 0.138 |
| 0.00183 | 6.99E-11 | NA | + | NA | NA | NA | 6 | 125.146 | -238.292 | 1.938 | 0.137 |
| 0.00481 | NA | NA | + | NA | NA | NA | 5 | 124.04 | -238.08 | 0 | 0.304 |
| 0.04428 | NA | NA | NA | 1.36E-04 | NA | NA | 3 | 121.439 | -236.878 | 1.201 | 0.167 |
| 0.01728 | NA | NA | + | 6.76E-05 | NA | NA | 6 | 124.408 | -236.815 | 1.264 | 0.162 |
| 0.01953 | NA | NA | + | NA | -6.55E-03 | NA | 6 | 124.189 | -236.378 | 1.702 | 0.13 |
| 0.01455 | NA | NA | + | NA | NA | -4.07E-03 | 6 | 124.114 | -236.228 | 1.852 | 0.12 |
| 0.00309 | 9.93E-11 | NA | + | NA | NA | NA | 6 | 124.088 | -236.175 | 1.904 | 0.117 |
| 0.00383 | NA | NA | + | NA | NA | NA | 5 | 126.326 | -242.651 | 0 | 0.353 |
| 0.02847 | NA | NA | + | NA | -1.11E-02 | NA | 6 | 126.81 | -241.62 | 1.031 | 0.211 |
| 0.01307 | NA | NA | + | 4.90E-05 | NA | NA | 6 | 126.547 | -241.094 | 1.557 | 0.162 |
| 0.01308 | NA | NA | + | NA | NA | -3.92E-03 | 6 | 126.403 | -240.806 | 1.845 | 0.14 |
| 0.00299 | 4.82E-11 | NA | + | NA | NA | NA | 6 | 126.346 | -240.692 | 1.959 | 0.133 |
| 0.00345 | NA | NA | + | NA | NA | NA | 5 | 127.77 | -245.54 | 0 | 0.342 |
| 0.0147 | NA | NA | + | 6.98E-05 | NA | NA | 6 | 128.244 | -244.488 | 1.052 | 0.202 |
| 0.02597 | NA | NA | + | NA | -9.95E-03 | NA | 6 | 128.207 | -244.414 | 1.127 | 0.195 |
| 0.00201 | 8.74E-11 | NA | + | NA | NA | NA | 6 | 127.822 | -243.644 | 1.896 | 0.133 |
| -0.00017 | NA | NA | + | NA | NA | 1.50E-03 | 6 | 127.782 | -243.565 | 1.976 | 0.128 |
| -0.00043 | NA | NA | + | NA | NA | NA | 5 | 126.185 | -242.369 | 0 | 0.348 |
| 0.01787 | NA | NA | + | NA | -8.08E-03 | NA | 6 | 126.582 | -241.164 | 1.205 | 0.191 |
| -0.01517 | NA | NA | + | NA | NA | 6.41E-03 | 6 | 126.431 | -240.863 | 1.506 | 0.164 |
| 0.00626 | NA | NA | + | 4.38E-05 | NA | NA | 6 | 126.403 | -240.806 | 1.563 | 0.159 |
| -0.00238 | 1.11E-10 | NA | + | NA | NA | NA | 6 | 126.255 | -240.509 | I.86 | 0.137 |
| 0.00159 | NA | NA | + | NA | NA | NA | 5 | 124.47 | -238.94 | 0 | 0.341 |
| 0.01608 | NA | NA | + | 7.84E-05 | NA | NA | 6 | 125.025 | -238.05 | 0.89 | 0.218 |
| 0.02126 | NA | NA | + | NA | -8.80E-03 | NA | 6 | 124.773 | -237.545 | 1.395 | 0.17 |
| 0.0132 | NA | NA | + | NA | NA | -4.94E-03 | 6 | 124.584 | -237.167 | 1.773 | 0.14 |
| 0.00004 | 9.13E-11 | NA | + | NA | NA | NA | 6 | 124.517 | -237.034 | 1.906 | 0.131 |
| 0.00109 | NA | NA | + | NA | NA | NA | 5 | 120.57 | -231.139 | 0 | 0.189 |
| 0.02362 | NA | NA | + | 1.15E-04 | NA | NA | 6 | 121.546 | -231.093 | 0.047 | 0.185 |
| 0.04835 | NA | NA | NA | 1.73E-04 | NA | NA | 3 | 118.069 | -230.138 | 1.001 | 0.115 |
| 0.04852 | NA | NA | + | 1.26E-04 | NA | -9.39E-03 | 7 | 121.945 | -229.89 | 1.249 | 0.101 |
| 0.02306 | NA | NA | + | NA | -9.66E-03 | NA | 6 | 120.875 | -229.75 | 1.389 | 0.094 |
| 0.01797 | NA | NA | + | NA | NA | -6.98E-03 | 6 | 120.792 | -229.584 | 1.556 | 0.087 |
| 0.04156 | NA | NA | + | 1.10E-04 | -8.28E-03 | NA | 7 | 121.772 | -229.545 | 1.595 | 0.085 |
| -0.00041 | 8.82E-11 | NA | + | NA | NA | NA | 6 | 120.631 | -229.263 | 1.877 | 0.074 |
| 0.02233 | 5.60E-11 | NA | + | 1.13E-04 | NA | NA | 7 | 121.571 | -229.143 | 1.997 | 0.07 |
| 0.00194 | NA | NA | + | NA | NA | NA | 5 | 126.206 | -242.411 | 0 | 0.354 |
| 0.01234 | NA | NA | + | 6.36E-05 | NA | NA | 6 | 126.621 | -241.243 | 1.169 | 0.197 |
| 0.02031 | NA | NA | + | NA | -8.17E-03 | NA | 6 | 126.535 | -241.07 | 1.341 | 0.181 |
| 0.00043 | 8.74E-11 | NA | + | NA | NA | NA | 6 | 126.251 | -240.501 | I.91 | 0.136 |
| -0.00106 | NA | NA | + | NA | NA | 1.28E-03 | 6 | 126.214 | -240.429 | 1.983 | 0.131 |
| 0.00852 | NA | NA | + | NA | NA | NA | 5 | 126.492 | -242.983 | 0 | 0.368 |
| 0.01877 | NA | NA | + | 6.36E-05 | NA | NA | 6 | 126.892 | -241.783 | 01.II | 0.202 |
| 0.01965 | NA | NA | + | NA | -4.98E-03 | NA | 6 | 126.605 | -241.211 | 1.772 | 0.152 |
| 0.00733 | 7.50E-11 | NA | + | NA | NA | NA | 6 | 126.53 | -241.06 | 1.923 | 0.141 |
| 0.00342 | NA | NA | + | NA | NA | 2.14E-03 | 6 | 126.516 | -241.031 | 1.952 | 0.139 |
| 0.00515 | NA | NA | + | NA | NA | NA | 5 | 118.485 | -226.97 | 0 | 0.277 |
| 0.02201 | NA | NA | + | 9.48E-05 | NA | NA | 6 | 119.182 | -226.365 | 0.605 | 0.205 |
| 0.04542 | NA | NA | NA | 1.47E-04 | NA | NA | 3 | 115.989 | -225.978 | 0.992 | 0.169 |
| 0.02268 | NA | NA | + | NA | -7.88E-03 | NA | 6 | 118.685 | -225.37 | 01.VI | 0.125 |
| 0.0186 | NA | NA | + | NA | NA | -5.56E-03 | 6 | 118.624 | -225.249 | 1.721 | 0.117 |
| 0.00326 | 1.08E-10 | NA | + | NA | NA | NA | 6 | 118.539 | -225.079 | 1.891 | 0.108 |
| 0.00452 | NA | NA | + | NA | NA | NA | 5 | 125.574 | -241.147 | 0 | 0.339 |
| 0.02904 | NA | NA | + | NA | -1.12E-02 | NA | 6 | 126.081 | -240.163 | 0.985 | 0.207 |
| 0.01635 | NA | NA | + | 6.76E-05 | NA | NA | 6 | 125.982 | -239.965 | 1.182 | 0.188 |
| 0.0137 | NA | NA | + | NA | NA | -3.80E-03 | 6 | 125.645 | -239.29 | 1.857 | 0.134 |
| 0.00284 | 9.58E-11 | NA | + | NA | NA | NA | 6 | 125.636 | -239.272 | 1.876 | 0.133 |
| 0.00331 | NA | NA | + | NA | NA | NA | 5 | 123.241 | -236.481 | 0 | 0.327 |
| 0.03161 | NA | NA | + | NA | -1.27E-02 | NA | 6 | 124.047 | -236.093 | 0.388 | 0.269 |
| 0.01067 | NA | NA | + | 4.76E-05 | NA | NA | 6 | 123.467 | -234.934 | 1.547 | 0.151 |
| 0.00165 | 9.65E-11 | NA | + | NA | NA | NA | 6 | 123.3 | -234.601 | I.88 | 0.128 |
| -0.00402 | NA | NA | + | NA | NA | 3.03E-03 | 6 | 123.291 | -234.583 | 1.898 | 0.126 |
| 0.00364 | NA | NA | + | NA | NA | NA | 5 | 121.869 | -233.739 | 0 | 0.345 |
| 0.02802 | NA | NA | + | NA | -1.08E-02 | NA | 6 | 122.31 | -232.62 | 1.118 | 0.197 |
| 0.01467 | NA | NA | + | 6.40E-05 | NA | NA | 6 | 122.238 | -232.475 | 1.263 | 0.184 |
| 0.01394 | NA | NA | + | NA | NA | -4.31E-03 | 6 | 121.96 | -231.919 | 1.819 | 0.139 |
| 0.00191 | 1.04E-10 | NA | + | NA | NA | NA | 6 | 121.928 | -231.856 | 1.882 | 0.135 |
| 0.00268 | NA | NA | + | NA | NA | NA | 5 | 125.899 | -241.798 | 0 | 0.362 |
| 0.01376 | NA | NA | + | 6.06E-05 | NA | NA | 6 | 126.219 | -240.439 | 1.359 | 0.183 |
| 0.02238 | NA | NA | + | NA | -8.62E-03 | NA | 6 | 126.207 | -240.415 | 1.383 | 0.181 |
| 0.00137 | 7.46E-11 | NA | + | NA | NA | NA | 6 | 125.932 | -239.865 | 1.933 | 0.138 |
| 0.00793 | NA | NA | + | NA | NA | -2.25E-03 | 6 | 125.926 | -239.852 | 1.946 | 0.137 |
| 0.00505 | NA | NA | + | NA | NA | NA | 5 | 121.445 | -232.891 | 0 | 0.34 |
| 0.02157 | NA | NA | + | 9.32E-05 | NA | NA | 6 | 122.147 | -232.294 | 0.597 | 0.252 |
| 0.02063 | NA | NA | + | NA | -6.83E-03 | NA | 6 | 121.612 | -231.224 | 1.667 | 0.148 |
| 0.00337 | 9.31E-11 | NA | + | NA | NA | NA | 6 | 121.503 | -231.006 | 1.884 | 0.132 |
| -0.00022 | NA | NA | + | NA | NA | 2.20E-03 | 6 | 121.471 | -230.943 | 1.948 | 0.128 |
| 0.00598 | NA | NA | + | NA | NA | NA | 5 | 125.45 | -240.901 | 0 | 0.301 |
| 0.02055 | NA | NA | + | 7.89E-05 | NA | NA | 6 | 125.99 | -239.98 | 0.921 | 0.19 |
| 0.04987 | NA | NA | NA | 1.44E-04 | NA | NA | 3 | 122.704 | -239.408 | 1.492 | 0.143 |
| 0.02225 | NA | NA | + | NA | -7.13E-03 | NA | 6 | 125.638 | -239.276 | 1.625 | 0.134 |
| 0.00425 | 1.03E-10 | NA | + | NA | NA | NA | 6 | 125.506 | -239.011 | I.89 | 0.117 |
| 0.01206 | NA | NA | + | NA | NA | -2.60E-03 | 6 | 125.483 | -238.966 | 1.934 | 0.115 |
| 0.00267 | NA | NA | + | NA | NA | NA | 5 | 119.809 | -229.617 | 0 | 0.348 |
| 0.02761 | NA | NA | + | NA | -1.10E-02 | NA | 6 | 120.218 | -228.436 | 1.181 | 0.193 |
| 0.01518 | NA | NA | + | 6.88E-05 | NA | NA | 6 | 120.203 | -228.405 | 1.212 | 0.19 |
| 0.01112 | NA | NA | + | NA | NA | -3.54E-03 | 6 | 119.871 | -227.741 | 1.876 | 0.136 |
| 0.00111 | 9.23E-11 | NA | + | NA | NA | NA | 6 | 119.857 | -227.714 | 1.903 | 0.134 |
| 0.00481 | NA | NA | + | NA | NA | NA | 5 | 123.18 | -236.36 | 0 | 0.367 |
| 0.01603 | NA | NA | + | 6.41E-05 | NA | NA | 6 | 123.52 | -235.04 | I.32 | 0.19 |
| 0.02307 | NA | NA | + | NA | -8.02E-03 | NA | 6 | 123.402 | -234.803 | 1.556 | 0.168 |
| 0.00374 | 6.42E-11 | NA | + | NA | NA | NA | 6 | 123.208 | -234.416 | 1.944 | 0.139 |
| 0.00817 | NA | NA | + | NA | NA | -1.43E-03 | 6 | 123.191 | -234.381 | 1.978 | 0.136 |
| 0.00316 | NA | NA | + | NA | NA | NA | 5 | 119.497 | -228.993 | 0 | 0.291 |
| 0.01916 | NA | NA | + | 8.63E-05 | NA | NA | 6 | 120.109 | -228.219 | 0.775 | 0.197 |
| 0.04549 | NA | NA | NA | 1.44E-04 | NA | NA | 3 | 116.82 | -227.641 | 1.353 | 0.148 |
| 0.02153 | NA | NA | + | NA | -8.15E-03 | NA | 6 | 119.756 | -227.512 | 1.482 | 0.139 |
| 0.00043 | 1.48E-10 | NA | + | NA | NA | NA | 6 | 119.599 | -227.198 | 1.795 | 0.118 |
| 0.00293 | NA | NA | + | NA | NA | 9.62E-05 | 6 | 119.497 | -226.993 | 2 | 0.107 |
| 0.00239 | NA | NA | + | NA | NA | NA | 5 | 123.586 | -237.172 | 0 | 0.339 |
| 0.02775 | NA | NA | + | NA | -1.12E-02 | NA | 6 | 124.138 | -236.276 | 0.896 | 0.217 |
| 0.01394 | NA | NA | + | 6.52E-05 | NA | NA | 6 | 124.007 | -236.013 | 1.158 | 0.19 |
| 0.0014 | 5.81E-11 | NA | + | NA | NA | NA | 6 | 123.62 | -235.24 | 1.932 | 0.129 |
| 0.00074 | NA | NA | + | NA | NA | 7.11E-04 | 6 | 123.589 | -235.177 | 1.995 | 0.125 |
| 0.0054 | NA | NA | + | NA | NA | NA | 5 | 121.316 | -232.631 | 0 | 0.297 |
| 0.01846 | NA | NA | + | 7.45E-05 | NA | NA | 6 | 121.758 | -231.516 | 1.115 | 0.17 |
| 0.04519 | NA | NA | NA | 1.41E-04 | NA | NA | 3 | 118.687 | -231.373 | 1.258 | 0.158 |
| 0.02213 | NA | NA | + | NA | -7.52E-03 | NA | 6 | 121.516 | -231.031 | 01.VI | 0.134 |
| 0.0191 | NA | NA | + | NA | NA | -5.63E-03 | 6 | 121.461 | -230.921 | I.71 | 0.126 |
| 0.004 | 8.50E-11 | NA | + | NA | NA | NA | 6 | 121.357 | -230.715 | 1.917 | 0.114 |
| 0.00342 | NA | NA | + | NA | NA | NA | 5 | 125.849 | -241.698 | 0 | 0.316 |
| 0.01686 | NA | NA | + | 7.09E-05 | NA | NA | 6 | 126.272 | -240.544 | 1.154 | 0.178 |
| 0.02016 | NA | NA | + | NA | -7.21E-03 | NA | 6 | 126.038 | -240.076 | 1.622 | 0.141 |
| 0.0485 | NA | NA | NA | 1.43E-04 | NA | NA | 3 | 122.932 | -239.863 | 1.835 | 0.126 |
| 0.00216 | 7.30E-11 | NA | + | NA | NA | NA | 6 | 125.895 | -239.789 | 1.909 | 0.122 |
| 0.00702 | NA | NA | + | NA | NA | -1.55E-03 | 6 | 125.861 | -239.722 | 1.976 | 0.118 |
| 0.00467 | NA | NA | + | NA | NA | NA | 5 | 127.346 | -244.693 | 0 | 0.365 |
| 0.01613 | NA | NA | + | 6.39E-05 | NA | NA | 6 | 127.693 | -243.386 | 1.307 | 0.19 |
| 0.02216 | NA | NA | + | NA | -7.78E-03 | NA | 6 | 127.569 | -243.137 | 1.556 | 0.168 |
| 0.01173 | NA | NA | + | NA | NA | -2.93E-03 | 6 | 127.389 | -242.778 | 1.915 | 0.14 |
| 0.00372 | 5.48E-11 | NA | + | NA | NA | NA | 6 | 127.367 | -242.735 | 1.958 | 0.137 |
| 0.00599 | NA | NA | + | NA | NA | NA | 5 | 120.964 | -231.928 | 0 | 0.366 |
| 0.01718 | NA | NA | + | 6.72E-05 | NA | NA | 6 | 121.349 | -230.699 | I.23 | 0.198 |
| 0.02149 | NA | NA | + | NA | -6.93E-03 | NA | 6 | 121.142 | -230.283 | 1.645 | 0.161 |
| 0.00471 | 7.77E-11 | NA | + | NA | NA | NA | 6 | 121.001 | -230.002 | 1.926 | 0.14 |
| 0.00769 | NA | NA | + | NA | NA | -7.09E-04 | 6 | 120.967 | -229.933 | 1.995 | 0.135 |
| 0.00602 | NA | NA | + | NA | NA | NA | 5 | 125.62 | -241.24 | 0 | 0.289 |
| 0.02166 | NA | NA | + | 8.16E-05 | NA | NA | 6 | 126.206 | -240.413 | 0.827 | 0.191 |
| 0.05078 | NA | NA | NA | 1.43E-04 | NA | NA | 3 | 123.122 | -240.244 | 0.995 | 0.176 |
| 0.02061 | NA | NA | + | NA | -6.35E-03 | NA | 6 | 125.772 | -239.544 | 1.696 | 0.124 |
| 0.0131 | NA | NA | + | NA | NA | -3.03E-03 | 6 | 125.664 | -239.329 | 1.911 | 0.111 |
| 0.00524 | 4.66E-11 | NA | + | NA | NA | NA | 6 | 125.64 | -239.279 | I.96 | 0.109 |
| 0.00391 | NA | NA | + | NA | NA | NA | 5 | 121.37 | -232.741 | 0 | 0.303 |
| 0.01894 | NA | NA | + | 8.21E-05 | NA | NA | 6 | 121.947 | -231.894 | 0.847 | 0.199 |
| 0.02228 | NA | NA | + | NA | -8.22E-03 | NA | 6 | 121.643 | -231.286 | 1.455 | 0.147 |
| 0.00189 | 1.18E-10 | NA | + | NA | NA | NA | 6 | 121.441 | -230.882 | 1.858 | 0.12 |
| 0.04663 | NA | NA | NA | 1.48E-04 | NA | NA | 3 | 118.441 | -230.881 | I.86 | 0.12 |
| 0.00222 | NA | NA | + | NA | NA | 7.11E-04 | 6 | 121.373 | -230.746 | 1.995 | 0.112 |
| 0.00367 | NA | NA | + | NA | NA | NA | 5 | 127.915 | -245.83 | 0 | 0.369 |
| 0.01372 | NA | NA | + | 5.67E-05 | NA | NA | 6 | 128.21 | -244.42 | I.41 | 0.182 |
| 0.02099 | NA | NA | + | NA | -7.70E-03 | NA | 6 | 128.155 | -244.31 | I.52 | 0.173 |
| 0.00255 | 6.62E-11 | NA | + | NA | NA | NA | 6 | 127.941 | -243.883 | 1.947 | 0.139 |
| 0.00233 | NA | NA | + | NA | NA | 5.67E-04 | 6 | 127.917 | -243.834 | 1.996 | 0.136 |
| 0.003 | NA | NA | + | NA | NA | NA | 5 | 122.547 | -235.095 | 0 | 0.361 |
| 0.01354 | NA | NA | + | 6.43E-05 | NA | NA | 6 | 122.941 | -233.881 | 1.213 | 0.197 |
| 0.01784 | NA | NA | + | NA | -6.63E-03 | NA | 6 | 122.739 | -233.478 | 1.616 | 0.161 |
| 0.00136 | 9.61E-11 | NA | + | NA | NA | NA | 6 | 122.611 | -233.222 | 1.873 | 0.141 |
| -0.0045 | NA | NA | + | NA | NA | 3.15E-03 | 6 | 122.6 | -233.2 | 1.894 | 0.14 |
| 0.00494 | NA | NA | + | NA | NA | NA | 5 | 127.163 | -244.325 | 0 | 0.304 |
| 0.01964 | NA | NA | + | 8.06E-05 | NA | NA | 6 | 127.744 | -243.488 | 0.838 | 0.2 |
| 0.04927 | NA | NA | NA | 1.47E-04 | NA | NA | 3 | 124.397 | -242.795 | I.53 | 0.141 |
| 0.01877 | NA | NA | + | NA | -6.02E-03 | NA | 6 | 127.293 | -242.587 | 1.739 | 0.127 |
| 0.00393 | 6.21E-11 | NA | + | NA | NA | NA | 6 | 127.195 | -242.391 | 1.935 | 0.115 |
| 0.00762 | NA | NA | + | NA | NA | -1.14E-03 | 6 | 127.169 | -242.338 | 1.987 | 0.112 |
| 0.00065 | NA | NA | + | NA | NA | NA | 5 | 127.733 | -245.465 | 0 | 0.349 |
| 0.01263 | NA | NA | + | 6.83E-05 | NA | NA | 6 | 128.159 | -244.319 | 1.147 | 0.197 |
| 0.02193 | NA | NA | + | NA | -9.38E-03 | NA | 6 | 128.106 | -244.211 | 1.254 | 0.186 |
| -0.00103 | 9.39E-11 | NA | + | NA | NA | NA | 6 | 127.784 | -243.568 | 1.897 | 0.135 |
| -0.00544 | NA | NA | + | NA | NA | 2.54E-03 | 6 | 127.768 | -243.537 | 1.929 | 0.133 |
| 0.00651 | NA | NA | + | NA | NA | NA | 5 | 127.058 | -244.115 | 0 | 0.29 |
| 0.02053 | NA | NA | + | 7.93E-05 | NA | NA | 6 | 127.603 | -243.206 | 0.909 | 0.184 |
| 0.02938 | NA | NA | + | NA | -1.01E-02 | NA | 6 | 127.418 | -242.837 | 1.279 | 0.153 |
| 0.04759 | NA | NA | NA | 1.42E-04 | NA | NA | 3 | 124.303 | -242.605 | I.51 | 0.136 |
| 0.02004 | NA | NA | + | NA | NA | -5.63E-03 | 6 | 127.21 | -242.421 | 1.695 | 0.124 |
| 0.00483 | 1.03E-10 | NA | + | NA | NA | NA | 6 | 127.111 | -242.223 | 1.892 | 0.113 |
| 0.00349 | NA | NA | + | NA | NA | NA | 5 | 125.474 | -240.947 | 0 | 0.298 |
| 0.01992 | NA | NA | + | 9.28E-05 | NA | NA | 6 | 126.212 | -240.424 | 0.523 | 0.229 |
| 0.01891 | NA | NA | + | NA | -6.76E-03 | NA | 6 | 125.631 | -239.262 | 1.686 | 0.128 |
| 0.04688 | NA | NA | NA | 1.55E-04 | NA | NA | 3 | 122.547 | -239.095 | 1.853 | 0.118 |
| 0.01123 | NA | NA | + | NA | NA | -3.20E-03 | 6 | 125.528 | -239.056 | 1.891 | 0.116 |
| 0.0029 | 3.47E-11 | NA | + | NA | NA | NA | 6 | 125.49 | -238.981 | 1.967 | 0.111 |
| 0.00557 | NA | NA | + | NA | NA | NA | 5 | 120.358 | -230.717 | 0 | 0.37 |
| 0.01645 | NA | NA | + | 6.76E-05 | NA | NA | 6 | 120.748 | -229.497 | I.22 | 0.201 |
| 0.01668 | NA | NA | + | NA | -5.08E-03 | NA | 6 | 120.454 | -228.909 | 1.808 | 0.15 |
| 0.00426 | 7.88E-11 | NA | + | NA | NA | NA | 6 | 120.396 | -228.793 | 1.924 | 0.141 |
| 0.00288 | NA | NA | + | NA | NA | 1.09E-03 | 6 | 120.364 | -228.729 | 1.988 | 0.137 |
| 0.00148 | NA | NA | + | NA | NA | NA | 5 | 124.819 | -239.638 | 0 | 0.355 |
| 0.0239 | NA | NA | + | NA | -9.93E-03 | NA | 6 | 125.198 | -238.395 | 1.243 | 0.191 |
| 0.01243 | NA | NA | + | 6.18E-05 | NA | NA | 6 | 125.159 | -238.319 | 1.319 | 0.184 |
| 0.00025 | 7.34E-11 | NA | + | NA | NA | NA | 6 | 124.856 | -237.712 | 1.926 | 0.136 |
| 0.00765 | NA | NA | + | NA | NA | -2.59E-03 | 6 | 124.852 | -237.703 | 1.935 | 0.135 |
| 0.00415 | NA | NA | + | NA | NA | NA | 5 | 121.29 | -232.58 | 0 | 0.374 |
| 0.01515 | NA | NA | + | 5.93E-05 | NA | NA | 6 | 121.588 | -231.177 | 1.404 | 0.185 |
| 0.01934 | NA | NA | + | NA | -6.71E-03 | NA | 6 | 121.458 | -230.916 | 1.664 | 0.163 |
| 0.00344 | 3.99E-11 | NA | + | NA | NA | NA | 6 | 121.305 | -230.61 | 1.971 | 0.14 |
| 0.00503 | NA | NA | + | NA | NA | -3.77E-04 | 6 | 121.291 | -230.582 | 1.999 | 0.138 |
| 0.00762 | NA | NA | + | NA | NA | NA | 5 | 124.528 | -239.056 | 0 | 0.234 |
| 0.04983 | NA | NA | NA | 1.49E-04 | NA | NA | 3 | 122.503 | -239.005 | 0.051 | 0.228 |
| 0.02255 | NA | NA | + | 8.67E-05 | NA | NA | 6 | 125.194 | -238.388 | 0.669 | 0.168 |
| 0.02146 | NA | NA | + | NA | -6.18E-03 | NA | 6 | 124.671 | -237.341 | 1.715 | 0.099 |
| 0.06125 | NA | NA | NA | 1.40E-04 | -6.03E-03 | NA | 4 | 122.637 | -237.275 | 1.782 | 0.096 |
| 0.00687 | 4.56E-11 | NA | + | NA | NA | NA | 6 | 124.549 | -237.097 | 1.959 | 0.088 |
| 0.0107 | NA | NA | + | NA | NA | -1.29E-03 | 6 | 124.536 | -237.072 | 1.984 | 0.087 |
| 0.00492 | NA | NA | + | NA | NA | NA | 5 | 119.486 | -228.973 | 0 | 0.372 |
| 0.01465 | NA | NA | + | 5.45E-05 | NA | NA | 6 | 119.74 | -227.479 | 1.494 | 0.176 |
| -0.00798 | NA | NA | + | NA | NA | 5.65E-03 | 6 | 119.676 | -227.352 | 1.621 | 0.165 |
| 0.01431 | NA | NA | + | NA | -4.07E-03 | NA | 6 | 119.553 | -227.107 | 1.866 | 0.146 |
| 0.00375 | 6.92E-11 | NA | + | NA | NA | NA | 6 | 119.515 | -227.03 | 1.943 | 0.141 |
| 0.00318 | NA | NA | + | NA | NA | NA | 5 | 127.101 | -244.202 | 0 | 0.365 |
| 0.01439 | NA | NA | + | 6.28E-05 | NA | NA | 6 | 127.444 | -242.888 | 1.314 | 0.189 |
| 0.02058 | NA | NA | + | NA | -7.54E-03 | NA | 6 | 127.331 | -242.663 | 1.539 | 0.169 |
| 0.00151 | 9.85E-11 | NA | + | NA | NA | NA | 6 | 127.148 | -242.296 | 1.907 | 0.141 |
| -0.00088 | NA | NA | + | NA | NA | 1.74E-03 | 6 | 127.117 | -242.234 | 1.968 | 0.136 |
| 0.00664 | NA | NA | + | NA | NA | NA | 5 | 127.778 | -245.556 | 0 | 0.29 |
| 0.02217 | NA | NA | + | 8.92E-05 | NA | NA | 6 | 128.521 | -245.042 | 0.514 | 0.224 |
| 0.0503 | NA | NA | NA | 1.51E-04 | NA | NA | 3 | 125.026 | -244.053 | 1.503 | 0.137 |
| 0.02138 | NA | NA | + | NA | -6.48E-03 | NA | 6 | 127.947 | -243.893 | 1.662 | 0.126 |
| 0.01427 | NA | NA | + | NA | NA | -3.21E-03 | 6 | 127.83 | -243.66 | 1.896 | 0.112 |
| 0.00586 | 4.91E-11 | NA | + | NA | NA | NA | 6 | 127.802 | -243.604 | 1.952 | 0.109 |
| 0.00262 | NA | NA | + | NA | NA | NA | 5 | 123.599 | -237.199 | 0 | 0.343 |
| 0.02744 | NA | NA | + | NA | -1.08E-02 | NA | 6 | 124.077 | -236.153 | 1.046 | 0.203 |
| 0.01421 | NA | NA | + | 6.43E-05 | NA | NA | 6 | 123.956 | -235.912 | 1.287 | 0.18 |
| 0.01516 | NA | NA | + | NA | NA | -5.38E-03 | 6 | 123.743 | -235.486 | 1.712 | 0.146 |
| 0.00162 | 5.77E-11 | NA | + | NA | NA | NA | 6 | 123.619 | -235.239 | I.96 | 0.129 |
| 0.00228 | NA | NA | + | NA | NA | NA | 5 | 123.665 | -237.33 | 0 | 0.355 |
| 0.01513 | NA | NA | + | 7.07E-05 | NA | NA | 6 | 124.103 | -236.205 | 1.125 | 0.202 |
| 0.02092 | NA | NA | + | NA | -8.28E-03 | NA | 6 | 123.926 | -235.852 | 1.478 | 0.17 |
| 0.00069 | 9.07E-11 | NA | + | NA | NA | NA | 6 | 123.71 | -235.421 | I.91 | 0.137 |
| 0.00896 | NA | NA | + | NA | NA | -2.82E-03 | 6 | 123.707 | -235.413 | 1.917 | 0.136 |
| 0.00212 | NA | NA | + | NA | NA | NA | 5 | 125.352 | -240.703 | 0 | 0.312 |
| 0.01718 | NA | NA | + | 8.02E-05 | NA | NA | 6 | 125.917 | -239.835 | 0.868 | 0.202 |
| 0.01797 | NA | NA | + | NA | -6.90E-03 | NA | 6 | 125.533 | -239.066 | 1.637 | 0.137 |
| 0.00063 | 8.36E-11 | NA | + | NA | NA | NA | 6 | 125.397 | -238.793 | I.91 | 0.12 |
| 0.04773 | NA | NA | NA | 1.47E-04 | NA | NA | 3 | 122.352 | -238.704 | 1.999 | 0.115 |
| 0.00228 | NA | NA | + | NA | NA | -6.98E-05 | 6 | 125.352 | -238.703 | 2 | 0.115 |
| 0.00307 | NA | NA | + | NA | NA | NA | 5 | 122.281 | -234.562 | 0 | 0.346 |
| 0.01626 | NA | NA | + | 7.45E-05 | NA | NA | 6 | 122.761 | -233.522 | 01.IV | 0.206 |
| 0.02534 | NA | NA | + | NA | -9.72E-03 | NA | 6 | 122.669 | -233.338 | 1.224 | 0.188 |
| 0.00173 | 7.39E-11 | NA | + | NA | NA | NA | 6 | 122.323 | -232.645 | 1.917 | 0.133 |
| 0.00525 | NA | NA | + | NA | NA | -9.11E-04 | 6 | 122.285 | -232.571 | 1.992 | 0.128 |
| 0.00307 | NA | NA | + | NA | NA | NA | 5 | 127.391 | -244.782 | 0 | 0.296 |
| 0.01753 | NA | NA | + | 7.93E-05 | NA | NA | 6 | 127.972 | -243.944 | 0.838 | 0.195 |
| 0.02155 | NA | NA | + | NA | -8.19E-03 | NA | 6 | 127.667 | -243.334 | 1.449 | 0.144 |
| 0.04681 | NA | NA | NA | 1.45E-04 | NA | NA | 3 | 124.651 | -243.301 | 1.481 | 0.141 |
| 0.00192 | 6.76E-11 | NA | + | NA | NA | NA | 6 | 127.432 | -242.865 | 1.917 | 0.114 |
| 0.00704 | NA | NA | + | NA | NA | -1.70E-03 | 6 | 127.406 | -242.812 | 1.971 | 0.111 |
| 0.00173 | NA | NA | + | NA | NA | NA | 5 | 122.652 | -235.304 | 0 | 0.375 |
| 0.01161 | NA | NA | + | 5.64E-05 | NA | NA | 6 | 122.934 | -233.867 | 1.437 | 0.183 |
| 0.01561 | NA | NA | + | NA | -6.15E-03 | NA | 6 | 122.797 | -233.593 | 1.711 | 0.16 |
| 0.00046 | 7.44E-11 | NA | + | NA | NA | NA | 6 | 122.693 | -233.386 | 1.918 | 0.144 |
| 0.00323 | NA | NA | + | NA | NA | -6.42E-04 | 6 | 122.654 | -233.308 | 1.996 | 0.138 |
| 0.00253 | NA | NA | + | NA | NA | NA | 5 | 120.974 | -231.947 | 0 | 0.353 |
| 0.02586 | NA | NA | + | NA | -1.05E-02 | NA | 6 | 121.393 | -230.786 | 1.162 | 0.198 |
| 0.01295 | NA | NA | + | 5.58E-05 | NA | NA | 6 | 121.234 | -230.468 | 1.479 | 0.169 |
| 0.01466 | NA | NA | + | NA | NA | -5.02E-03 | 6 | 121.099 | -230.198 | I.75 | 0.147 |
| 0.00139 | 6.31E-11 | NA | + | NA | NA | NA | 6 | 120.998 | -229.997 | 1.951 | 0.133 |
| 0.00095 | NA | NA | + | NA | NA | NA | 5 | 127.206 | -244.413 | 0 | 0.348 |
| 0.02567 | NA | NA | + | NA | -1.08E-02 | NA | 6 | 127.731 | -243.463 | 0.95 | 0.217 |
| 0.01008 | NA | NA | + | 5.50E-05 | NA | NA | 6 | 127.506 | -243.012 | 1.401 | 0.173 |
| -0.00032 | 7.26E-11 | NA | + | NA | NA | NA | 6 | 127.237 | -242.474 | 1.939 | 0.132 |
| 0.00493 | NA | NA | + | NA | NA | -1.69E-03 | 6 | 127.222 | -242.443 | I.97 | 0.13 |
| 0.00196 | NA | NA | + | NA | NA | NA | 5 | 118.881 | -227.762 | 0 | 0.349 |
| 0.01623 | NA | NA | + | 7.78E-05 | NA | NA | 6 | 119.374 | -226.749 | 1.014 | 0.21 |
| 0.02314 | NA | NA | + | NA | -9.34E-03 | NA | 6 | 119.196 | -226.393 | 1.369 | 0.176 |
| 0.00025 | 9.49E-11 | NA | + | NA | NA | NA | 6 | 118.933 | -225.865 | 1.897 | 0.135 |
| 0.00473 | NA | NA | + | NA | NA | -1.16E-03 | 6 | 118.888 | -225.776 | 1.986 | 0.129 |
| 0.00182 | NA | NA | + | NA | NA | NA | 5 | 125.904 | -241.808 | 0 | 0.339 |
| 0.01554 | NA | NA | + | 7.78E-05 | NA | NA | 6 | 126.477 | -240.954 | 0.854 | 0.221 |
| 0.02176 | NA | NA | + | NA | -8.74E-03 | NA | 6 | 126.241 | -240.483 | 1.325 | 0.175 |
| -0.00624 | NA | NA | + | NA | NA | 3.41E-03 | 6 | 125.976 | -239.952 | 1.856 | 0.134 |
| 0.0003 | 8.56E-11 | NA | + | NA | NA | NA | 6 | 125.944 | -239.888 | I.92 | 0.13 |
| 0.00832 | NA | NA | + | NA | NA | NA | 5 | 124.603 | -239.206 | 0 | 0.374 |
| 0.01728 | NA | NA | + | 5.28E-05 | NA | NA | 6 | 124.868 | -237.735 | 1.471 | 0.179 |
| 0.02226 | NA | NA | + | NA | -6.18E-03 | NA | 6 | 124.771 | -237.542 | 1.664 | 0.163 |
| 0.00182 | NA | NA | + | NA | NA | 2.75E-03 | 6 | 124.647 | -237.294 | 1.913 | 0.144 |
| 0.00748 | 5.02E-11 | NA | + | NA | NA | NA | 6 | 124.62 | -237.241 | 1.966 | 0.14 |
| 0.00456 | NA | NA | + | NA | NA | NA | 5 | 125.975 | -241.95 | 0 | 0.354 |
| 0.01676 | NA | NA | + | 6.79E-05 | NA | NA | 6 | 126.4 | -240.801 | 1.149 | 0.199 |
| 0.02556 | NA | NA | + | NA | -9.15E-03 | NA | 6 | 126.314 | -240.627 | 1.323 | 0.183 |
| 0.00337 | 6.29E-11 | NA | + | NA | NA | NA | 6 | 125.996 | -239.992 | 1.958 | 0.133 |
| 0.00641 | NA | NA | + | NA | NA | -7.91E-04 | 6 | 125.978 | -239.957 | 1.993 | 0.131 |
| 0.0033 | NA | NA | + | NA | NA | NA | 5 | 126.836 | -243.673 | 0 | 0.319 |
| 0.01491 | NA | NA | + | 6.11E-05 | NA | NA | 6 | 127.148 | -242.295 | 1.378 | 0.16 |
| 0.01957 | NA | NA | + | NA | -7.21E-03 | NA | 6 | 127.025 | -242.051 | 1.622 | 0.142 |
| 0.04606 | NA | NA | NA | 1.40E-04 | NA | NA | 3 | 123.942 | -241.884 | 1.789 | 0.13 |
| 0.01386 | NA | NA | + | NA | NA | -4.36E-03 | 6 | 126.929 | -241.858 | 1.815 | 0.129 |
| 0.00222 | 6.42E-11 | NA | + | NA | NA | NA | 6 | 126.865 | -241.731 | 1.942 | 0.121 |
| 0.00422 | NA | NA | + | NA | NA | NA | 5 | 119.735 | -229.47 | 0 | 0.353 |
| 0.01794 | NA | NA | + | 8.01E-05 | NA | NA | 6 | 120.252 | -228.503 | 0.966 | 0.218 |
| 0.02145 | NA | NA | + | NA | -7.68E-03 | NA | 6 | 119.956 | -227.912 | 1.558 | 0.162 |
| 0.0025 | 1.03E-10 | NA | + | NA | NA | NA | 6 | 119.788 | -227.577 | 1.893 | 0.137 |
| 0.00521 | NA | NA | + | NA | NA | -4.12E-04 | 6 | 119.736 | -227.472 | 1.998 | 0.13 |
| 0.00023 | NA | NA | + | NA | NA | NA | 5 | 122.79 | -235.58 | 0 | 0.344 |
| 0.02518 | NA | NA | + | NA | -1.08E-02 | NA | 6 | 123.333 | -234.666 | 0.914 | 0.218 |
| 0.01073 | NA | NA | + | 6.17E-05 | NA | NA | 6 | 123.15 | -234.3 | 1.281 | 0.181 |
| -0.00089 | 6.23E-11 | NA | + | NA | NA | NA | 6 | 122.821 | -233.642 | 1.939 | 0.13 |
| -0.00067 | NA | NA | + | NA | NA | 3.86E-04 | 6 | 122.791 | -233.582 | 1.998 | 0.127 |
| -0.00017 | NA | NA | + | NA | NA | NA | 5 | 123.724 | -237.448 | 0 | 0.371 |
| 0.01649 | NA | NA | + | NA | -7.36E-03 | NA | 6 | 123.985 | -235.97 | 1.479 | 0.177 |
| 0.00587 | NA | NA | + | 3.70E-05 | NA | NA | 6 | 123.864 | -235.727 | 1.721 | 0.157 |
| -0.01145 | NA | NA | + | NA | NA | 4.93E-03 | 6 | 123.861 | -235.723 | 1.726 | 0.156 |
| -0.00096 | 4.49E-11 | NA | + | NA | NA | NA | 6 | 123.746 | -235.491 | 1.957 | 0.139 |
| 0.0044 | NA | NA | + | NA | NA | NA | 5 | 122.904 | -235.809 | 0 | 0.289 |
| 0.02043 | NA | NA | + | 9.08E-05 | NA | NA | 6 | 123.607 | -235.214 | 0.595 | 0.215 |
| 0.04798 | NA | NA | NA | 1.50E-04 | NA | NA | 3 | 120.329 | -234.659 | I.15 | 0.163 |
| 0.01464 | NA | NA | + | NA | -4.44E-03 | NA | 6 | 122.979 | -233.959 | I.85 | 0.115 |
| 0.00301 | 8.26E-11 | NA | + | NA | NA | NA | 6 | 122.953 | -233.907 | 1.902 | 0.112 |
| 0.0062 | NA | NA | + | NA | NA | -7.60E-04 | 6 | 122.907 | -233.815 | 1.994 | 0.107 |
| 0.00456 | NA | NA | + | NA | NA | NA | 5 | 117.73 | -225.46 | 0 | 0.364 |
| 0.01883 | NA | NA | + | 7.88E-05 | NA | NA | 6 | 118.215 | -224.429 | 1.031 | 0.217 |
| 0.01538 | NA | NA | + | NA | -4.67E-03 | NA | 6 | 117.803 | -223.605 | 1.855 | 0.144 |
| -0.00155 | NA | NA | + | NA | NA | 2.58E-03 | 6 | 117.768 | -223.535 | 1.925 | 0.139 |
| 0.00377 | 4.42E-11 | NA | + | NA | NA | NA | 6 | 117.741 | -223.482 | 1.978 | 0.135 |
| 0.00353 | NA | NA | + | NA | NA | NA | 5 | 122.58 | -235.159 | 0 | 0.291 |
| 0.04587 | NA | NA | NA | 1.41E-04 | NA | NA | 3 | 120.148 | -234.296 | 0.864 | 0.189 |
| 0.01903 | NA | NA | + | 8.10E-05 | NA | NA | 6 | 123.083 | -234.166 | 0.993 | 0.177 |
| 0.01595 | NA | NA | + | NA | -5.40E-03 | NA | 6 | 122.675 | -233.351 | 1.809 | 0.118 |
| 0.0121 | NA | NA | + | NA | NA | -3.61E-03 | 6 | 122.643 | -233.285 | 1.874 | 0.114 |
| 0.00242 | 6.32E-11 | NA | + | NA | NA | NA | 6 | 122.614 | -233.228 | 1.931 | 0.111 |
| 0.00692 | NA | NA | + | NA | NA | NA | 5 | 129.046 | -248.091 | 0 | 0.342 |
| 0.03066 | NA | NA | + | NA | -1.09E-02 | NA | 6 | 129.558 | -247.115 | 0.976 | 0.21 |
| 0.01774 | NA | NA | + | 6.52E-05 | NA | NA | 6 | 129.43 | -246.861 | 1.231 | 0.185 |
| 0.00549 | 8.45E-11 | NA | + | NA | NA | NA | 6 | 129.099 | -246.198 | 1.894 | 0.133 |
| 0.01428 | NA | NA | + | NA | NA | -2.97E-03 | 6 | 129.09 | -246.181 | 1.911 | 0.131 |
| 0.00218 | NA | NA | + | NA | NA | NA | 5 | 123.656 | -237.313 | 0 | 0.351 |
| 0.01463 | NA | NA | + | 7.37E-05 | NA | NA | 6 | 124.158 | -236.317 | 0.996 | 0.213 |
| 0.01994 | NA | NA | + | NA | -7.90E-03 | NA | 6 | 123.924 | -235.849 | 1.464 | 0.169 |
| 0.00048 | 9.93E-11 | NA | + | NA | NA | NA | 6 | 123.719 | -235.439 | 1.874 | 0.138 |
| 0.00182 | NA | NA | + | NA | NA | 1.51E-04 | 6 | 123.656 | -235.313 | 2 | 0.129 |
| 0.00607 | NA | NA | + | NA | NA | NA | 5 | 116.096 | -222.192 | 0 | 0.363 |
| 0.02142 | NA | NA | + | 8.17E-05 | NA | NA | 6 | 116.615 | -221.23 | 0.962 | 0.224 |
| 0.01443 | NA | NA | + | NA | -3.67E-03 | NA | 6 | 116.145 | -220.29 | 1.902 | 0.14 |
| 0.00459 | 8.16E-11 | NA | + | NA | NA | NA | 6 | 116.133 | -220.266 | 1.926 | 0.139 |
| 0.00531 | NA | NA | + | NA | NA | 3.27E-04 | 6 | 116.097 | -220.193 | 1.999 | 0.134 |
| 0.00453 | NA | NA | + | NA | NA | NA | 5 | 121.691 | -233.383 | 0 | 0.3 |
| 0.0187 | NA | NA | + | 8.38E-05 | NA | NA | 6 | 122.327 | -232.654 | 0.728 | 0.209 |
| 0.04821 | NA | NA | NA | 1.50E-04 | NA | NA | 3 | 118.879 | -231.758 | 1.624 | 0.133 |
| 0.01702 | NA | NA | + | NA | -5.53E-03 | NA | 6 | 121.809 | -231.619 | 1.764 | 0.124 |
| -0.00359 | NA | NA | + | NA | NA | 3.38E-03 | 6 | 121.754 | -231.508 | 1.875 | 0.118 |
| 0.00301 | 8.84E-11 | NA | + | NA | NA | NA | 6 | 121.738 | -231.476 | 1.907 | 0.116 |
| 0.00215 | NA | NA | + | NA | NA | NA | 5 | 128.103 | -246.205 | 0 | 0.337 |
| 0.02718 | NA | NA | + | NA | -1.09E-02 | NA | 6 | 128.683 | -245.365 | 0.84 | 0.221 |
| 0.012 | NA | NA | + | 6.08E-05 | NA | NA | 6 | 128.493 | -244.986 | 1.219 | 0.183 |
| 0.00095 | 7.22E-11 | NA | + | NA | NA | NA | 6 | 128.148 | -244.297 | 1.908 | 0.13 |
| -0.00391 | NA | NA | + | NA | NA | 2.58E-03 | 6 | 128.141 | -244.282 | 1.923 | 0.129 |
| 0.00475 | NA | NA | + | NA | NA | NA | 5 | 129.07 | -248.14 | 0 | 0.373 |
| 0.01616 | NA | NA | + | 6.30E-05 | NA | NA | 6 | 129.431 | -246.862 | 1.278 | 0.197 |
| 0.01361 | NA | NA | + | NA | -3.91E-03 | NA | 6 | 129.13 | -246.26 | I.88 | 0.146 |
| 0.01109 | NA | NA | + | NA | NA | -2.71E-03 | 6 | 129.11 | -246.22 | I.92 | 0.143 |
| 0.00377 | 5.88E-11 | NA | + | NA | NA | NA | 6 | 129.094 | -246.187 | 1.952 | 0.141 |
| 0.00322 | NA | NA | + | NA | NA | NA | 5 | 126.476 | -242.952 | 0 | 0.291 |
| 0.01872 | NA | NA | + | 7.97E-05 | NA | NA | 6 | 127.007 | -242.014 | 0.937 | 0.182 |
| 0.04835 | NA | NA | NA | 1.47E-04 | NA | NA | 3 | 123.814 | -241.627 | 1.325 | 0.15 |
| 0.0227 | NA | NA | + | NA | -8.39E-03 | NA | 6 | 126.726 | -241.452 | 01.V | 0.138 |
| 0.01705 | NA | NA | + | NA | NA | -5.81E-03 | 6 | 126.637 | -241.275 | 1.677 | 0.126 |
| 0.00181 | 8.32E-11 | NA | + | NA | NA | NA | 6 | 126.524 | -241.048 | 1.904 | 0.112 |
| 0.00069 | NA | NA | + | NA | NA | NA | 5 | 118.163 | -226.327 | 0 | 0.299 |
| 0.01768 | NA | NA | + | 9.02E-05 | NA | NA | 6 | 118.795 | -225.591 | 0.736 | 0.207 |
| 0.04514 | NA | NA | NA | 1.59E-04 | NA | NA | 3 | 115.462 | -224.925 | 1.402 | 0.148 |
| -0.00174 | 1.44E-10 | NA | + | NA | NA | NA | 6 | 118.254 | -224.508 | 1.818 | 0.12 |
| 0.00996 | NA | NA | + | NA | -4.12E-03 | NA | 6 | 118.221 | -224.442 | 1.884 | 0.116 |
| 0.00217 | NA | NA | + | NA | NA | -6.19E-04 | 6 | 118.165 | -224.33 | 1.996 | 0.11 |
| -0.00191 | NA | NA | + | NA | NA | NA | 5 | 121.497 | -232.993 | 0 | 0.285 |
| 0.01551 | NA | NA | + | 1.01E-04 | NA | NA | 6 | 122.361 | -232.723 | 0.271 | 0.249 |
| 0.018 | NA | NA | + | NA | -8.71E-03 | NA | 6 | 121.778 | -231.555 | 1.438 | 0.139 |
| -0.00421 | 1.33E-10 | NA | + | NA | NA | NA | 6 | 121.585 | -231.17 | 1.823 | 0.114 |
| 0.03031 | NA | NA | + | 9.53E-05 | -6.91E-03 | NA | 7 | 122.537 | -231.074 | 1.919 | 0.109 |
| -0.00099 | NA | NA | + | NA | NA | -3.86E-04 | 6 | 121.497 | -230.995 | 1.999 | 0.105 |
| 0.00098 | NA | NA | + | NA | NA | NA | 5 | 126.786 | -243.571 | 0 | 0.364 |
| 0.0204 | NA | NA | + | NA | -8.57E-03 | NA | 6 | 127.095 | -242.19 | 1.382 | 0.182 |
| 0.01091 | NA | NA | + | 5.61E-05 | NA | NA | 6 | 127.086 | -242.172 | 1.399 | 0.181 |
| 0.00631 | NA | NA | + | NA | NA | -2.28E-03 | 6 | 126.812 | -241.624 | 1.948 | 0.137 |
| 0.00013 | 4.86E-11 | NA | + | NA | NA | NA | 6 | 126.806 | -241.613 | 1.959 | 0.137 |
| 0.00332 | NA | NA | + | NA | NA | NA | 5 | 121.257 | -232.514 | 0 | 0.351 |
| 0.01786 | NA | NA | + | 7.73E-05 | NA | NA | 6 | 121.762 | -231.524 | 0.99 | 0.214 |
| 0.02327 | NA | NA | + | NA | -8.81E-03 | NA | 6 | 121.528 | -231.055 | 1.458 | 0.169 |
| 0.00168 | 9.48E-11 | NA | + | NA | NA | NA | 6 | 121.313 | -230.625 | 1.889 | 0.136 |
| 0.00523 | NA | NA | + | NA | NA | -8.17E-04 | 6 | 121.26 | -230.52 | 1.993 | 0.13 |
| -0.00028 | NA | NA | + | NA | NA | NA | 5 | 122.914 | -235.827 | 0 | 0.237 |
| 0.01949 | NA | NA | + | 1.02E-04 | NA | NA | 6 | 123.774 | -235.548 | 0.279 | 0.206 |
| 0.04881 | NA | NA | NA | 1.66E-04 | NA | NA | 3 | 120.65 | -235.3 | 0.527 | 0.182 |
| 0.01393 | NA | NA | + | NA | -6.13E-03 | NA | 6 | 123.047 | -234.094 | 1.733 | 0.1 |
| 0.00906 | NA | NA | + | NA | NA | -3.95E-03 | 6 | 122.986 | -233.973 | 1.854 | 0.094 |
| 0.03598 | NA | NA | + | 1.10E-04 | NA | -6.33E-03 | 7 | 123.959 | -233.917 | I.91 | 0.091 |
| -0.00183 | 8.98E-11 | NA | + | NA | NA | NA | 6 | 122.957 | -233.913 | 1.914 | 0.091 |
| 0.00236 | NA | NA | + | NA | NA | NA | 5 | 123.933 | -237.866 | 0 | 0.344 |
| 0.01549 | NA | NA | + | 7.71E-05 | NA | NA | 6 | 124.469 | -236.938 | 0.928 | 0.216 |
| 0.02373 | NA | NA | + | NA | -9.28E-03 | NA | 6 | 124.269 | -236.538 | 1.327 | 0.177 |
| 0.00022 | 1.22E-10 | NA | + | NA | NA | NA | 6 | 124.004 | -236.008 | 1.857 | 0.136 |
| 0.00422 | NA | NA | + | NA | NA | -7.94E-04 | 6 | 123.936 | -235.872 | 1.994 | 0.127 |
| 0.00502 | NA | NA | + | NA | NA | NA | 5 | 119.454 | -228.908 | 0 | 0.304 |
| 0.01823 | NA | NA | + | 7.12E-05 | NA | NA | 6 | 119.848 | -227.695 | 1.212 | 0.166 |
| 0.02201 | NA | NA | + | NA | NA | -7.10E-03 | 6 | 119.683 | -227.365 | 1.543 | 0.141 |
| 0.02318 | NA | NA | + | NA | -8.11E-03 | NA | 6 | 119.676 | -227.352 | 1.555 | 0.14 |
| 0.04766 | NA | NA | NA | 1.45E-04 | NA | NA | 3 | 116.646 | -227.292 | 1.616 | 0.136 |
| 0.00405 | 5.93E-11 | NA | + | NA | NA | NA | 6 | 119.477 | -226.954 | 1.953 | 0.114 |
| 0.00358 | NA | NA | + | NA | NA | NA | 5 | 125.953 | -241.906 | 0 | 0.286 |
| 0.01805 | NA | NA | + | 8.18E-05 | NA | NA | 6 | 126.514 | -241.027 | 0.879 | 0.185 |
| 0.04471 | NA | NA | NA | 1.51E-04 | NA | NA | 3 | 123.462 | -240.924 | 0.982 | 0.175 |
| 0.02021 | NA | NA | + | NA | -7.48E-03 | NA | 6 | 126.153 | -240.305 | 1.601 | 0.129 |
| 0.00169 | 1.14E-10 | NA | + | NA | NA | NA | 6 | 126.024 | -240.048 | 1.858 | 0.113 |
| 0.0124 | NA | NA | + | NA | NA | -3.60E-03 | 6 | 126.015 | -240.03 | 1.876 | 0.112 |
| 0.00512 | NA | NA | + | NA | NA | NA | 5 | 129.605 | -249.211 | 0 | 0.357 |
| 0.02712 | NA | NA | + | NA | -9.85E-03 | NA | 6 | 130.024 | -248.048 | 1.163 | 0.2 |
| 0.01367 | NA | NA | + | 5.05E-05 | NA | NA | 6 | 129.85 | -247.7 | 1.511 | 0.168 |
| 0.01292 | NA | NA | + | NA | NA | -3.28E-03 | 6 | 129.663 | -247.327 | 1.884 | 0.139 |
| 0.00382 | 7.54E-11 | NA | + | NA | NA | NA | 6 | 129.636 | -247.272 | 1.939 | 0.136 |
| 0.00267 | NA | NA | + | NA | NA | NA | 5 | 120.362 | -230.724 | 0 | 0.297 |
| 0.01913 | NA | NA | + | 9.31E-05 | NA | NA | 6 | 121.103 | -230.207 | 0.517 | 0.229 |
| 0.02015 | NA | NA | + | NA | -7.74E-03 | NA | 6 | 120.587 | -229.174 | I.55 | 0.137 |
| 0.01198 | NA | NA | + | NA | NA | -3.84E-03 | 6 | 120.433 | -228.867 | 1.857 | 0.117 |
| 0.00186 | 4.77E-11 | NA | + | NA | NA | NA | 6 | 120.38 | -228.76 | 1.964 | 0.111 |
| 0.04594 | NA | NA | NA | 1.57E-04 | NA | NA | 3 | 117.366 | -228.731 | 1.992 | 0.11 |
| 0.00294 | NA | NA | + | NA | NA | NA | 5 | 123.389 | -236.778 | 0 | 0.353 |
| 0.027 | NA | NA | + | NA | -1.08E-02 | NA | 6 | 123.911 | -235.821 | 0.957 | 0.219 |
| 0.01072 | NA | NA | + | 4.67E-05 | NA | NA | 6 | 123.6 | -235.2 | 1.579 | 0.16 |
| 0.00167 | 7.32E-11 | NA | + | NA | NA | NA | 6 | 123.424 | -234.848 | I.93 | 0.135 |
| -0.00211 | NA | NA | + | NA | NA | 2.11E-03 | 6 | 123.414 | -234.828 | I.95 | 0.133 |
| 0.00573 | NA | NA | + | NA | NA | NA | 5 | 128.526 | -247.052 | 0 | 0.348 |
| 0.01912 | NA | NA | + | 7.95E-05 | NA | NA | 6 | 129.128 | -246.257 | 0.795 | 0.234 |
| 0.02005 | NA | NA | + | NA | -6.32E-03 | NA | 6 | 128.701 | -245.403 | 1.649 | 0.153 |
| 0.00459 | 6.66E-11 | NA | + | NA | NA | NA | 6 | 128.563 | -245.127 | 1.925 | 0.133 |
| -0.00022 | NA | NA | + | NA | NA | 2.46E-03 | 6 | 128.559 | -245.119 | 1.933 | 0.132 |
| 0.006 | NA | NA | + | NA | NA | NA | 5 | 125.579 | -241.158 | 0 | 0.361 |
| 0.01793 | NA | NA | + | 7.06E-05 | NA | NA | 6 | 126.032 | -240.064 | 1.094 | 0.209 |
| 0.02154 | NA | NA | + | NA | -6.93E-03 | NA | 6 | 125.763 | -239.527 | 1.631 | 0.16 |
| 0.01193 | NA | NA | + | NA | NA | -2.44E-03 | 6 | 125.609 | -239.218 | 1.939 | 0.137 |
| 0.00541 | 3.60E-11 | NA | + | NA | NA | NA | 6 | 125.592 | -239.183 | 1.975 | 0.134 |
| 0.00245 | NA | NA | + | NA | NA | NA | 5 | 131.576 | -253.153 | 0 | 0.356 |
| 0.02094 | NA | NA | + | NA | -8.24E-03 | NA | 6 | 131.986 | -251.972 | I.18 | 0.198 |
| 0.00871 | NA | NA | + | 4.15E-05 | NA | NA | 6 | 131.77 | -251.539 | 1.613 | 0.159 |
| -0.00806 | NA | NA | + | NA | NA | 4.54E-03 | 6 | 131.697 | -251.393 | 1.759 | 0.148 |
| 0.00067 | 1.04E-10 | NA | + | NA | NA | NA | 6 | 131.635 | -251.27 | 1.882 | 0.139 |
| 0.00197 | NA | NA | + | NA | NA | NA | 5 | 122.432 | -234.865 | 0 | 0.203 |
| 0.02184 | NA | NA | + | 9.67E-05 | NA | NA | 6 | 123.15 | -234.3 | 0.565 | 0.153 |
| 0.05939 | NA | NA | + | 1.18E-04 | NA | -1.43E-02 | 7 | 123.998 | -233.995 | 0.869 | 0.131 |
| 0.04901 | NA | NA | NA | 1.54E-04 | NA | NA | 3 | 119.965 | -233.93 | 0.935 | 0.127 |
| 0.02765 | NA | NA | + | NA | NA | -1.11E-02 | 6 | 122.958 | -233.916 | 0.949 | 0.126 |
| 0.02232 | NA | NA | + | NA | -8.75E-03 | NA | 6 | 122.684 | -233.368 | 1.496 | 0.096 |
| 0.08171 | NA | NA | NA | 1.76E-04 | NA | -1.18E-02 | 4 | 120.523 | -233.046 | 1.818 | 0.082 |
| -0.00025 | 1.29E-10 | NA | + | NA | NA | NA | 6 | 122.51 | -233.021 | 1.844 | 0.081 |
| 0.00389 | NA | NA | + | NA | NA | NA | 5 | 122.751 | -235.502 | 0 | 0.347 |
| 0.01722 | NA | NA | + | 7.77E-05 | NA | NA | 6 | 123.28 | -234.56 | 0.942 | 0.217 |
| 0.02242 | NA | NA | + | NA | -8.18E-03 | NA | 6 | 123.024 | -234.047 | 1.455 | 0.168 |
| 0.00194 | 1.20E-10 | NA | + | NA | NA | NA | 6 | 122.832 | -233.664 | 1.838 | 0.139 |
| 0.00805 | NA | NA | + | NA | NA | -1.71E-03 | 6 | 122.765 | -233.531 | 1.972 | 0.13 |
| 0.00168 | NA | NA | + | NA | NA | NA | 5 | 122.759 | -235.517 | 0 | 0.352 |
| 0.01438 | NA | NA | + | 7.00E-05 | NA | NA | 6 | 123.169 | -234.337 | I.18 | 0.195 |
| 0.02389 | NA | NA | + | NA | -9.77E-03 | NA | 6 | 123.129 | -234.257 | I.26 | 0.188 |
| 0.00038 | 7.54E-11 | NA | + | NA | NA | NA | 6 | 122.796 | -233.592 | 1.926 | 0.135 |
| 0.00308 | NA | NA | + | NA | NA | -5.83E-04 | 6 | 122.761 | -233.521 | 1.996 | 0.13 |
| -0.00025 | NA | NA | + | NA | NA | NA | 5 | 125.804 | -241.607 | 0 | 0.292 |
| 0.01688 | NA | NA | + | 8.84E-05 | NA | NA | 6 | 126.479 | -240.958 | 0.649 | 0.211 |
| 0.04972 | NA | NA | NA | 1.61E-04 | NA | NA | 3 | 123.064 | -240.128 | 1.479 | 0.139 |
| 0.01923 | NA | NA | + | NA | -8.32E-03 | NA | 6 | 126.05 | -240.101 | 1.507 | 0.138 |
| -0.00167 | 8.49E-11 | NA | + | NA | NA | NA | 6 | 125.846 | -239.692 | 1.916 | 0.112 |
| -0.00017 | NA | NA | + | NA | NA | -3.18E-05 | 6 | 125.804 | -239.607 | 2 | 0.107 |
| 0.00544 | NA | NA | + | NA | NA | NA | 5 | 121.19 | -232.381 | 0 | 0.297 |
| 0.0201 | NA | NA | + | 8.25E-05 | NA | NA | 6 | 121.752 | -231.505 | 0.876 | 0.191 |
| 0.047 | NA | NA | NA | 1.40E-04 | NA | NA | 3 | 118.616 | -231.232 | 1.148 | 0.167 |
| 0.01787 | NA | NA | + | NA | -5.47E-03 | NA | 6 | 121.302 | -230.603 | 1.777 | 0.122 |
| 0.00423 | 7.03E-11 | NA | + | NA | NA | NA | 6 | 121.221 | -230.443 | 1.938 | 0.113 |
| 0.00933 | NA | NA | + | NA | NA | -1.62E-03 | 6 | 121.204 | -230.407 | 1.973 | 0.111 |
| 0.00233 | NA | NA | + | NA | NA | NA | 5 | 121.258 | -232.516 | 0 | 0.369 |
| 0.02322 | NA | NA | + | NA | -9.18E-03 | NA | 6 | 121.555 | -231.11 | 1.405 | 0.183 |
| 0.01254 | NA | NA | + | 5.29E-05 | NA | NA | 6 | 121.483 | -230.966 | I.55 | 0.17 |
| 0.00778 | NA | NA | + | NA | NA | -2.36E-03 | 6 | 121.287 | -230.575 | 1.941 | 0.14 |
| 0.00126 | 5.92E-11 | NA | + | NA | NA | NA | 6 | 121.28 | -230.559 | 1.956 | 0.139 |
| 0.00232 | NA | NA | + | NA | NA | NA | 5 | 127.078 | -244.155 | 0 | 0.361 |
| 0.02205 | NA | NA | + | NA | -8.77E-03 | NA | 6 | 127.447 | -242.895 | 1.261 | 0.192 |
| 0.01027 | NA | NA | + | 4.89E-05 | NA | NA | 6 | 127.327 | -242.653 | 1.502 | 0.17 |
| 0.00067 | 9.80E-11 | NA | + | NA | NA | NA | 6 | 127.142 | -242.284 | 1.872 | 0.141 |
| -0.00245 | NA | NA | + | NA | NA | 2.05E-03 | 6 | 127.101 | -242.202 | 1.953 | 0.136 |
| 0.00192 | NA | NA | + | NA | NA | NA | 5 | 126.428 | -242.856 | 0 | 0.346 |
| 0.01507 | NA | NA | + | 7.07E-05 | NA | NA | 6 | 126.882 | -241.764 | 1.093 | 0.2 |
| 0.02347 | NA | NA | + | NA | -9.58E-03 | NA | 6 | 126.791 | -241.582 | 1.274 | 0.183 |
| 0.01089 | NA | NA | + | NA | NA | -3.81E-03 | 6 | 126.497 | -240.994 | 1.862 | 0.136 |
| 0.00042 | 8.72E-11 | NA | + | NA | NA | NA | 6 | 126.479 | -240.958 | 1.898 | 0.134 |
| 0.00613 | NA | NA | + | NA | NA | NA | 5 | 124.277 | -238.554 | 0 | 0.339 |
| 0.02096 | NA | NA | + | 8.49E-05 | NA | NA | 6 | 124.931 | -237.863 | 0.692 | 0.24 |
| 0.02493 | NA | NA | + | NA | -8.40E-03 | NA | 6 | 124.562 | -237.124 | I.43 | 0.166 |
| 0.0047 | 8.57E-11 | NA | + | NA | NA | NA | 6 | 124.33 | -236.659 | 1.895 | 0.131 |
| 0.00586 | NA | NA | + | NA | NA | 1.15E-04 | 6 | 124.277 | -236.554 | 2 | 0.125 |
| 0.0042 | NA | NA | + | NA | NA | NA | 5 | 119.517 | -229.034 | 0 | 0.318 |
| 0.01774 | NA | NA | + | 7.47E-05 | NA | NA | 6 | 119.936 | -227.871 | 1.163 | 0.178 |
| 0.01941 | NA | NA | + | NA | -6.64E-03 | NA | 6 | 119.668 | -227.336 | 1.698 | 0.136 |
| 0.04782 | NA | NA | NA | 1.45E-04 | NA | NA | 3 | 116.603 | -227.205 | 1.829 | 0.128 |
| 0.00271 | 8.91E-11 | NA | + | NA | NA | NA | 6 | 119.563 | -227.125 | 1.908 | 0.123 |
| 0.00646 | NA | NA | + | NA | NA | -9.55E-04 | 6 | 119.521 | -227.042 | 1.991 | 0.118 |
| 0.00641 | NA | NA | + | NA | NA | NA | 5 | 122.166 | -234.332 | 0 | 0.369 |
| 0.01539 | NA | NA | + | 5.56E-05 | NA | NA | 6 | 122.462 | -232.923 | 1.409 | 0.183 |
| 0.01782 | NA | NA | + | NA | -5.17E-03 | NA | 6 | 122.283 | -232.566 | 1.766 | 0.153 |
| -0.0044 | NA | NA | + | NA | NA | 4.49E-03 | 6 | 122.278 | -232.556 | 1.776 | 0.152 |
| 0.00453 | 1.08E-10 | NA | + | NA | NA | NA | 6 | 122.22 | -232.44 | 1.892 | 0.143 |
| 0.00514 | NA | NA | + | NA | NA | NA | 5 | 127.349 | -244.698 | 0 | 0.35 |
| 0.02741 | NA | NA | + | NA | -1.00E-02 | NA | 6 | 127.787 | -243.574 | 1.124 | 0.199 |
| 0.01546 | NA | NA | + | 6.14E-05 | NA | NA | 6 | 127.712 | -243.423 | 1.275 | 0.185 |
| 0.00378 | 8.00E-11 | NA | + | NA | NA | NA | 6 | 127.397 | -242.793 | 1.905 | 0.135 |
| 0.00945 | NA | NA | + | NA | NA | -1.78E-03 | 6 | 127.365 | -242.73 | 1.968 | 0.131 |
| 0.00276 | NA | NA | + | NA | NA | NA | 5 | 126.336 | -242.672 | 0 | 0.368 |
| 0.02361 | NA | NA | + | NA | -9.26E-03 | NA | 6 | 126.683 | -241.367 | 1.306 | 0.192 |
| 0.01043 | NA | NA | + | 4.64E-05 | NA | NA | 6 | 126.539 | -241.078 | 1.594 | 0.166 |
| 0.0019 | 4.91E-11 | NA | + | NA | NA | NA | 6 | 126.354 | -240.708 | 1.965 | 0.138 |
| 0.00341 | NA | NA | + | NA | NA | -2.67E-04 | 6 | 126.337 | -240.673 | 1.999 | 0.136 |
| 0.00324 | NA | NA | + | NA | NA | NA | 5 | 127.272 | -244.545 | 0 | 0.341 |
| 0.01561 | NA | NA | + | 7.39E-05 | NA | NA | 6 | 127.811 | -243.622 | 0.922 | 0.215 |
| 0.02509 | NA | NA | + | NA | -9.57E-03 | NA | 6 | 127.656 | -243.313 | 1.232 | 0.184 |
| 0.00181 | 8.41E-11 | NA | + | NA | NA | NA | 6 | 127.319 | -242.638 | 1.907 | 0.131 |
| -0.0011 | NA | NA | + | NA | NA | 1.85E-03 | 6 | 127.292 | -242.584 | I.96 | 0.128 |
| 0.00403 | NA | NA | + | NA | NA | NA | 5 | 130.567 | -251.134 | 0 | 0.359 |
| 0.02367 | NA | NA | + | NA | -8.81E-03 | NA | 6 | 130.938 | -249.876 | 1.257 | 0.191 |
| 0.01303 | NA | NA | + | 5.38E-05 | NA | NA | 6 | 130.871 | -249.741 | 1.393 | 0.179 |
| -0.00133 | NA | NA | + | NA | NA | 2.28E-03 | 6 | 130.598 | -249.196 | 1.938 | 0.136 |
| 0.00323 | 4.54E-11 | NA | + | NA | NA | NA | 6 | 130.585 | -249.171 | 1.963 | 0.135 |
| 0.00621 | NA | NA | + | NA | NA | NA | 5 | 120.358 | -230.716 | 0 | 0.38 |
| 0.01533 | NA | NA | + | 5.40E-05 | NA | NA | 6 | 120.59 | -229.18 | 1.536 | 0.176 |
| 0.02093 | NA | NA | + | NA | -6.47E-03 | NA | 6 | 120.511 | -229.022 | 1.695 | 0.163 |
| 0.0054 | 4.47E-11 | NA | + | NA | NA | NA | 6 | 120.368 | -228.737 | 1.979 | 0.141 |
| 0.00778 | NA | NA | + | NA | NA | -6.55E-04 | 6 | 120.36 | -228.721 | 1.995 | 0.14 |
| 0.00112 | NA | NA | + | NA | NA | NA | 5 | 122.316 | -234.632 | 0 | 0.355 |
| 0.02308 | NA | NA | + | NA | -9.78E-03 | NA | 6 | 122.723 | -233.446 | 1.186 | 0.196 |
| 0.0109 | NA | NA | + | 5.61E-05 | NA | NA | 6 | 122.608 | -233.217 | 1.415 | 0.175 |
| -0.00072 | 1.06E-10 | NA | + | NA | NA | NA | 6 | 122.368 | -232.736 | 1.895 | 0.138 |
| 0.00779 | NA | NA | + | NA | NA | -2.80E-03 | 6 | 122.355 | -232.709 | 1.922 | 0.136 |
| 0.00329 | NA | NA | + | NA | NA | NA | 5 | 128.157 | -246.314 | 0 | 0.371 |
| 0.02396 | NA | NA | + | NA | -9.27E-03 | NA | 6 | 128.527 | -245.054 | 1.259 | 0.198 |
| 0.00965 | NA | NA | + | 3.66E-05 | NA | NA | 6 | 128.29 | -244.581 | 1.733 | 0.156 |
| 0.00231 | 5.63E-11 | NA | + | NA | NA | NA | 6 | 128.176 | -244.351 | 1.963 | 0.139 |
| 0.00185 | NA | NA | + | NA | NA | 6.05E-04 | 6 | 128.159 | -244.318 | 1.996 | 0.137 |
| 0.00103 | NA | NA | + | NA | NA | NA | 5 | 122.182 | -234.363 | 0 | 0.356 |
| 0.01301 | NA | NA | + | 6.76E-05 | NA | NA | 6 | 122.609 | -233.218 | 1.145 | 0.201 |
| 0.01892 | NA | NA | + | NA | -7.88E-03 | NA | 6 | 122.436 | -232.871 | 1.492 | 0.169 |
| -0.00079 | 1.08E-10 | NA | + | NA | NA | NA | 6 | 122.249 | -232.499 | 1.865 | 0.14 |
| 0.00517 | NA | NA | + | NA | NA | -1.79E-03 | 6 | 122.197 | -232.395 | 1.968 | 0.133 |
| 0.00458 | NA | NA | + | NA | NA | NA | 5 | 127.902 | -245.805 | 0 | 0.364 |
| 0.01626 | NA | NA | + | 6.34E-05 | NA | NA | 6 | 128.274 | -244.548 | 1.257 | 0.194 |
| 0.02128 | NA | NA | + | NA | -7.31E-03 | NA | 6 | 128.121 | -244.241 | 1.563 | 0.167 |
| 0.01064 | NA | NA | + | NA | NA | -2.63E-03 | 6 | 127.936 | -243.872 | 1.933 | 0.139 |
| 0.00382 | 4.48E-11 | NA | + | NA | NA | NA | 6 | 127.92 | -243.84 | 1.965 | 0.136 |
| 0.00094 | NA | NA | + | NA | NA | NA | 5 | 123.394 | -236.788 | 0 | 0.35 |
| 0.02461 | NA | NA | + | NA | -1.03E-02 | NA | 6 | 123.844 | -235.689 | 01.I | 0.202 |
| 0.01073 | NA | NA | + | 5.84E-05 | NA | NA | 6 | 123.717 | -235.434 | 1.354 | 0.178 |
| -0.00677 | NA | NA | + | NA | NA | 3.25E-03 | 6 | 123.457 | -234.915 | 1.874 | 0.137 |
| -0.00007 | 5.76E-11 | NA | + | NA | NA | NA | 6 | 123.419 | -234.838 | I.95 | 0.132 |
| 0.00375 | NA | NA | + | NA | NA | NA | 5 | 118.485 | -226.97 | 0 | 0.365 |
| 0.01722 | NA | NA | + | 7.49E-05 | NA | NA | 6 | 118.915 | -225.83 | I.14 | 0.206 |
| 0.01938 | NA | NA | + | NA | -6.93E-03 | NA | 6 | 118.644 | -225.287 | 1.683 | 0.157 |
| 0.00276 | 5.69E-11 | NA | + | NA | NA | NA | 6 | 118.504 | -225.008 | 1.962 | 0.137 |
| 0.00723 | NA | NA | + | NA | NA | -1.44E-03 | 6 | 118.495 | -224.991 | 1.979 | 0.135 |
| 0.00063 | NA | NA | + | NA | NA | NA | 5 | 124.149 | -238.297 | 0 | 0.292 |
| 0.01817 | NA | NA | + | 9.27E-05 | NA | NA | 6 | 124.932 | -237.865 | 0.433 | 0.235 |
| 0.01467 | NA | NA | + | NA | -6.09E-03 | NA | 6 | 124.309 | -236.617 | I.68 | 0.126 |
| 0.04872 | NA | NA | NA | 1.59E-04 | NA | NA | 3 | 121.263 | -236.527 | 1.771 | 0.121 |
| -0.0011 | 9.71E-11 | NA | + | NA | NA | NA | 6 | 124.201 | -236.402 | 1.895 | 0.113 |
| -0.00598 | NA | NA | + | NA | NA | 2.86E-03 | 6 | 124.197 | -236.393 | 1.904 | 0.113 |
| 0.00177 | NA | NA | + | NA | NA | NA | 5 | 126.577 | -243.154 | 0 | 0.34 |
| 0.02593 | NA | NA | + | NA | -1.08E-02 | NA | 6 | 127.26 | -242.519 | 0.635 | 0.248 |
| 0.00746 | NA | NA | + | 3.98E-05 | NA | NA | 6 | 126.75 | -241.501 | 1.653 | 0.149 |
| 0.00032 | 8.63E-11 | NA | + | NA | NA | NA | 6 | 126.635 | -241.271 | 1.884 | 0.133 |
| -0.00484 | NA | NA | + | NA | NA | 2.71E-03 | 6 | 126.619 | -241.239 | 1.915 | 0.131 |
| 0.00313 | NA | NA | + | NA | NA | NA | 5 | 118.712 | -227.425 | 0 | 0.293 |
| 0.01936 | NA | NA | + | 9.20E-05 | NA | NA | 6 | 119.427 | -226.853 | 0.571 | 0.22 |
| 0.04862 | NA | NA | NA | 1.59E-04 | NA | NA | 3 | 115.941 | -225.882 | 1.542 | 0.136 |
| 0.01776 | NA | NA | + | NA | -6.42E-03 | NA | 6 | 118.867 | -225.734 | 1.691 | 0.126 |
| 0.00095 | 1.30E-10 | NA | + | NA | NA | NA | 6 | 118.798 | -225.596 | 1.828 | 0.117 |
| 0.00411 | NA | NA | + | NA | NA | -4.05E-04 | 6 | 118.713 | -225.426 | 1.998 | 0.108 |
| 0.05012 | NA | NA | NA | 1.53E-04 | NA | NA | 3 | 120.608 | -235.216 | 0 | 0.289 |
| 0.00658 | NA | NA | + | NA | NA | NA | 5 | 122.264 | -234.528 | 0.688 | 0.205 |
| 0.02486 | NA | NA | + | 9.79E-05 | NA | NA | 6 | 123.059 | -234.117 | 1.099 | 0.167 |
| 0.06236 | NA | NA | NA | 1.61E-04 | NA | -4.36E-03 | 4 | 120.699 | -233.397 | 1.819 | 0.116 |
| 0.05931 | NA | NA | NA | 1.47E-04 | -4.76E-03 | NA | 4 | 120.688 | -233.376 | I.84 | 0.115 |
| 0.04927 | 5.45E-11 | NA | NA | 1.53E-04 | NA | NA | 4 | 120.625 | -233.25 | 1.966 | 0.108 |
| 0.00116 | NA | NA | + | NA | NA | NA | 5 | 125.942 | -241.884 | 0 | 0.348 |
| 0.01303 | NA | NA | + | 6.68E-05 | NA | NA | 6 | 126.346 | -240.691 | 1.193 | 0.192 |
| 0.02237 | NA | NA | + | NA | -9.32E-03 | NA | 6 | 126.293 | -240.586 | 1.299 | 0.182 |
| 0.01439 | NA | NA | + | NA | NA | -5.53E-03 | 6 | 126.084 | -240.169 | 1.715 | 0.148 |
| 0.00024 | 5.47E-11 | NA | + | NA | NA | NA | 6 | 125.965 | -239.93 | 1.954 | 0.131 |
| 0.00103 | NA | NA | + | NA | NA | NA | 5 | 122.445 | -234.891 | 0 | 0.356 |
| 0.01263 | NA | NA | + | 6.54E-05 | NA | NA | 6 | 122.814 | -233.628 | 1.263 | 0.19 |
| 0.02242 | NA | NA | + | NA | -9.50E-03 | NA | 6 | 122.778 | -233.555 | 1.335 | 0.183 |
| -0.00085 | 1.04E-10 | NA | + | NA | NA | NA | 6 | 122.506 | -233.012 | 1.878 | 0.139 |
| 0.00358 | NA | NA | + | NA | NA | -1.07E-03 | 6 | 122.451 | -232.902 | 1.988 | 0.132 |
| 0.00594 | NA | NA | + | NA | NA | NA | 5 | 127.276 | -244.553 | 0 | 0.341 |
| 0.02029 | NA | NA | + | 8.01E-05 | NA | NA | 6 | 127.883 | -243.767 | 0.786 | 0.23 |
| 0.02514 | NA | NA | + | NA | -8.47E-03 | NA | 6 | 127.579 | -243.158 | 1.395 | 0.17 |
| 0.00482 | 6.41E-11 | NA | + | NA | NA | NA | 6 | 127.316 | -242.633 | I.92 | 0.131 |
| 0.00205 | NA | NA | + | NA | NA | 1.65E-03 | 6 | 127.291 | -242.583 | I.97 | 0.127 |
| 0.00827 | NA | NA | + | NA | NA | NA | 5 | 127.976 | -245.952 | 0 | 0.351 |
| 0.01882 | NA | NA | + | 6.51E-05 | NA | NA | 6 | 128.416 | -244.832 | 01.XII | 0.201 |
| 0.02803 | NA | NA | + | NA | -8.93E-03 | NA | 6 | 128.345 | -244.691 | 1.261 | 0.187 |
| 0.00754 | 4.48E-11 | NA | + | NA | NA | NA | 6 | 127.996 | -243.991 | I.96 | 0.132 |
| 0.00685 | NA | NA | + | NA | NA | 5.95E-04 | 6 | 127.978 | -243.956 | 1.996 | 0.129 |
| 0.0035 | NA | NA | + | NA | NA | NA | 5 | 127.527 | -245.054 | 0 | 0.364 |
| 0.0229 | NA | NA | + | NA | -8.69E-03 | NA | 6 | 127.875 | -243.75 | 1.303 | 0.19 |
| 0.01019 | NA | NA | + | 4.02E-05 | NA | NA | 6 | 127.687 | -243.375 | 1.679 | 0.157 |
| -0.00687 | NA | NA | + | NA | NA | 4.42E-03 | 6 | 127.637 | -243.274 | 1.779 | 0.15 |
| 0.00236 | 6.67E-11 | NA | + | NA | NA | NA | 6 | 127.561 | -243.121 | 1.933 | 0.139 |
| 0.00328 | NA | NA | + | NA | NA | NA | 5 | 121.651 | -233.303 | 0 | 0.374 |
| 0.02174 | NA | NA | + | NA | -8.13E-03 | NA | 6 | 121.897 | -231.793 | 1.509 | 0.176 |
| 0.01231 | NA | NA | + | 4.99E-05 | NA | NA | 6 | 121.861 | -231.722 | 1.581 | 0.17 |
| 0.00255 | 4.19E-11 | NA | + | NA | NA | NA | 6 | 121.67 | -231.34 | 1.963 | 0.14 |
| 0.00721 | NA | NA | + | NA | NA | -1.65E-03 | 6 | 121.665 | -231.33 | 1.972 | 0.14 |
| 0.00069 | NA | NA | + | NA | NA | NA | 5 | 121.51 | -233.019 | 0 | 0.357 |
| 0.02455 | NA | NA | + | NA | -1.06E-02 | NA | 6 | 121.92 | -231.839 | I.18 | 0.198 |
| 0.0101 | NA | NA | + | 5.25E-05 | NA | NA | 6 | 121.746 | -231.493 | 1.527 | 0.167 |
| 0.01028 | NA | NA | + | NA | NA | -3.97E-03 | 6 | 121.59 | -231.179 | I.84 | 0.142 |
| -0.00028 | 5.81E-11 | NA | + | NA | NA | NA | 6 | 121.538 | -231.076 | 1.944 | 0.135 |
| 0.0007 | NA | NA | + | NA | NA | NA | 5 | 124.803 | -239.605 | 0 | 0.344 |
| 0.01461 | NA | NA | + | 7.49E-05 | NA | NA | 6 | 125.299 | -238.597 | 1.008 | 0.208 |
| 0.02356 | NA | NA | + | NA | -1.02E-02 | NA | 6 | 125.186 | -238.371 | 1.234 | 0.186 |
| -0.00064 | 7.89E-11 | NA | + | NA | NA | NA | 6 | 124.854 | -237.708 | 1.897 | 0.133 |
| 0.00567 | NA | NA | + | NA | NA | -2.08E-03 | 6 | 124.824 | -237.648 | 1.958 | 0.129 |
| 0.00083 | NA | NA | + | NA | NA | NA | 5 | 120.891 | -231.781 | 0 | 0.355 |
| 0.01388 | NA | NA | + | 7.04E-05 | NA | NA | 6 | 121.29 | -230.581 | 01.II | 0.195 |
| 0.02416 | NA | NA | + | NA | -1.02E-02 | NA | 6 | 121.251 | -230.502 | 1.279 | 0.187 |
| -0.00007 | 5.01E-11 | NA | + | NA | NA | NA | 6 | 120.912 | -229.823 | 1.958 | 0.133 |
| 0.00247 | NA | NA | + | NA | NA | -6.88E-04 | 6 | 120.893 | -229.786 | 1.995 | 0.131 |
| 0.00513 | NA | NA | + | NA | NA | NA | 5 | 129.629 | -249.258 | 0 | 0.353 |
| 0.02934 | NA | NA | + | NA | -1.06E-02 | NA | 6 | 130.107 | -248.214 | 1.044 | 0.209 |
| 0.01468 | NA | NA | + | 5.22E-05 | NA | NA | 6 | 129.882 | -247.763 | 1.495 | 0.167 |
| 0.0132 | NA | NA | + | NA | NA | -3.42E-03 | 6 | 129.693 | -247.386 | 1.872 | 0.138 |
| 0.00406 | 6.29E-11 | NA | + | NA | NA | NA | 6 | 129.653 | -247.306 | 1.952 | 0.133 |
| 0.00226 | NA | NA | + | NA | NA | NA | 5 | 121.483 | -232.966 | 0 | 0.328 |
| 0.02995 | NA | NA | + | NA | -1.22E-02 | NA | 6 | 122.163 | -232.327 | 0.64 | 0.239 |
| 0.01313 | NA | NA | + | 6.57E-05 | NA | NA | 6 | 121.901 | -231.802 | 1.164 | 0.184 |
| 0.00055 | 1.01E-10 | NA | + | NA | NA | NA | 6 | 121.546 | -231.091 | 1.875 | 0.129 |
| 0.00167 | NA | NA | + | NA | NA | 2.50E-04 | 6 | 121.484 | -230.967 | 1.999 | 0.121 |
| 0.00219 | NA | NA | + | NA | NA | NA | 5 | 121.943 | -233.886 | 0 | 0.361 |
| 0.02604 | NA | NA | + | NA | -1.04E-02 | NA | 6 | 122.422 | -232.843 | 1.043 | 0.214 |
| 0.0096 | NA | NA | + | 4.32E-05 | NA | NA | 6 | 122.113 | -232.225 | 1.661 | 0.157 |
| 0.00145 | 4.17E-11 | NA | + | NA | NA | NA | 6 | 121.958 | -231.916 | 1.971 | 0.135 |
| 0.00224 | NA | NA | + | NA | NA | -1.90E-05 | 6 | 121.943 | -231.886 | 2 | 0.133 |
| 0.00519 | NA | NA | + | NA | NA | NA | 5 | 125.345 | -240.69 | 0 | 0.269 |
| 0.04926 | NA | NA | NA | 1.48E-04 | NA | NA | 3 | 123.153 | -240.307 | 0.383 | 0.223 |
| 0.02264 | NA | NA | + | 9.09E-05 | NA | NA | 6 | 126.032 | -240.065 | 0.625 | 0.197 |
| 0.01687 | NA | NA | + | NA | -5.07E-03 | NA | 6 | 125.439 | -238.879 | 1.812 | 0.109 |
| 0.00398 | 6.92E-11 | NA | + | NA | NA | NA | 6 | 125.375 | -238.751 | I.94 | 0.102 |
| 0.00778 | NA | NA | + | NA | NA | -1.11E-03 | 6 | 125.351 | -238.702 | 1.988 | 0.1 |
| 0.00219 | NA | NA | + | NA | NA | NA | 5 | 123.487 | -236.974 | 0 | 0.352 |
| 0.02426 | NA | NA | + | NA | -9.85E-03 | NA | 6 | 123.966 | -235.931 | 1.043 | 0.209 |
| 0.00981 | NA | NA | + | 4.79E-05 | NA | NA | 6 | 123.727 | -235.454 | I.52 | 0.165 |
| 0.00006 | 1.31E-10 | NA | + | NA | NA | NA | 6 | 123.571 | -235.142 | 1.832 | 0.141 |
| -0.00409 | NA | NA | + | NA | NA | 2.64E-03 | 6 | 123.524 | -235.047 | 1.927 | 0.134 |
| 0.00189 | NA | NA | + | NA | NA | NA | 5 | 122.547 | -235.094 | 0 | 0.362 |
| 0.01335 | NA | NA | + | 6.60E-05 | NA | NA | 6 | 122.927 | -233.854 | I.24 | 0.195 |
| 0.01833 | NA | NA | + | NA | -7.35E-03 | NA | 6 | 122.759 | -233.518 | 1.577 | 0.165 |
| 0.00019 | 9.89E-11 | NA | + | NA | NA | NA | 6 | 122.603 | -233.206 | 1.888 | 0.141 |
| -0.00355 | NA | NA | + | NA | NA | 2.27E-03 | 6 | 122.578 | -233.155 | 1.939 | 0.137 |
| 0.0014 | NA | NA | + | NA | NA | NA | 5 | 118.884 | -227.768 | 0 | 0.306 |
| 0.01698 | NA | NA | + | 9.05E-05 | NA | NA | 6 | 119.566 | -227.133 | 0.635 | 0.223 |
| 0.0496 | NA | NA | NA | 1.58E-04 | NA | NA | 3 | 115.974 | -225.948 | 1.819 | 0.123 |
| 0.00978 | NA | NA | + | NA | -3.62E-03 | NA | 6 | 118.931 | -225.862 | 1.906 | 0.118 |
| -0.00009 | 8.98E-11 | NA | + | NA | NA | NA | 6 | 118.927 | -225.854 | 1.914 | 0.118 |
| 0.00296 | NA | NA | + | NA | NA | -6.65E-04 | 6 | 118.886 | -225.772 | 1.996 | 0.113 |
| 0.00205 | NA | NA | + | NA | NA | NA | 5 | 123.642 | -237.283 | 0 | 0.348 |
| 0.01542 | NA | NA | + | 7.60E-05 | NA | NA | 6 | 124.116 | -236.233 | 01.V | 0.206 |
| 0.02294 | NA | NA | + | NA | -9.39E-03 | NA | 6 | 123.971 | -235.942 | 1.342 | 0.178 |
| -0.00005 | 1.21E-10 | NA | + | NA | NA | NA | 6 | 123.718 | -235.436 | 1.848 | 0.138 |
| 0.00555 | NA | NA | + | NA | NA | -1.43E-03 | 6 | 123.652 | -235.304 | I.98 | 0.129 |
| 0.00121 | NA | NA | + | NA | NA | NA | 5 | 127.261 | -244.523 | 0 | 0.357 |
| 0.01232 | NA | NA | + | 6.27E-05 | NA | NA | 6 | 127.633 | -243.267 | 1.256 | 0.19 |
| 0.02087 | NA | NA | + | NA | -8.60E-03 | NA | 6 | 127.571 | -243.142 | 1.381 | 0.179 |
| -0.00012 | 8.11E-11 | NA | + | NA | NA | NA | 6 | 127.305 | -242.61 | 1.912 | 0.137 |
| -0.00539 | NA | NA | + | NA | NA | 2.79E-03 | 6 | 127.304 | -242.609 | 1.914 | 0.137 |
| 0.00067 | NA | NA | + | NA | NA | NA | 5 | 117.928 | -225.856 | 0 | 0.374 |
| 0.00908 | NA | NA | + | 5.00E-05 | NA | NA | 6 | 118.155 | -224.311 | 1.545 | 0.173 |
| 0.01755 | NA | NA | + | NA | -7.47E-03 | NA | 6 | 118.151 | -224.301 | 1.555 | 0.172 |
| -0.00065 | 7.74E-11 | NA | + | NA | NA | NA | 6 | 117.964 | -223.928 | 1.928 | 0.143 |
| 0.00167 | NA | NA | + | NA | NA | -4.19E-04 | 6 | 117.929 | -223.858 | 1.998 | 0.138 |
| 0.00421 | NA | NA | + | NA | NA | NA | 5 | 121.027 | -232.055 | 0 | 0.367 |
| 0.01641 | NA | NA | + | 6.36E-05 | NA | NA | 6 | 121.349 | -230.697 | 1.358 | 0.186 |
| 0.0233 | NA | NA | + | NA | -8.47E-03 | NA | 6 | 121.274 | -230.548 | 1.507 | 0.173 |
| 0.00317 | 6.37E-11 | NA | + | NA | NA | NA | 6 | 121.052 | -230.104 | 1.951 | 0.138 |
| 0.00744 | NA | NA | + | NA | NA | -1.35E-03 | 6 | 121.037 | -230.075 | I.98 | 0.136 |
| 0.00559 | NA | NA | + | NA | NA | NA | 5 | 128.39 | -246.78 | 0 | 0.358 |
| 0.01761 | NA | NA | + | 6.93E-05 | NA | NA | 6 | 128.845 | -245.691 | 1.089 | 0.208 |
| 0.02184 | NA | NA | + | NA | -7.17E-03 | NA | 6 | 128.622 | -245.244 | 1.536 | 0.166 |
| 0.00435 | 7.35E-11 | NA | + | NA | NA | NA | 6 | 128.431 | -244.861 | 1.919 | 0.137 |
| 0.00499 | NA | NA | + | NA | NA | 2.53E-04 | 6 | 128.39 | -244.781 | 1.999 | 0.132 |
| 0.01005 | NA | NA | + | NA | NA | NA | 5 | 128.267 | -246.534 | 0 | 0.35 |
| 0.02325 | NA | NA | + | 8.02E-05 | NA | NA | 6 | 128.869 | -245.738 | 0.796 | 0.235 |
| 0.02332 | NA | NA | + | NA | -5.88E-03 | NA | 6 | 128.414 | -244.828 | 1.707 | 0.149 |
| 0.00842 | 9.69E-11 | NA | + | NA | NA | NA | 6 | 128.326 | -244.651 | 1.883 | 0.136 |
| 0.00662 | NA | NA | + | NA | NA | 1.43E-03 | 6 | 128.278 | -244.556 | 1.978 | 0.13 |
| 0.00098 | NA | NA | + | NA | NA | NA | 5 | 122.967 | -235.934 | 0 | 0.364 |
| 0.01248 | NA | NA | + | 6.38E-05 | NA | NA | 6 | 123.317 | -234.633 | 1.301 | 0.19 |
| 0.01944 | NA | NA | + | NA | -8.16E-03 | NA | 6 | 123.222 | -234.444 | 1.491 | 0.173 |
| -0.00018 | 7.11E-11 | NA | + | NA | NA | NA | 6 | 122.999 | -233.998 | 1.936 | 0.138 |
| 0.00092 | NA | NA | + | NA | NA | 2.69E-05 | 6 | 122.967 | -233.934 | 2 | 0.134 |
| 0.00376 | NA | NA | + | NA | NA | NA | 5 | 128.462 | -246.923 | 0 | 0.365 |
| 0.02223 | NA | NA | + | NA | -8.18E-03 | NA | 6 | 128.77 | -245.54 | 1.384 | 0.183 |
| 0.01328 | NA | NA | + | 5.38E-05 | NA | NA | 6 | 128.739 | -245.478 | 1.446 | 0.177 |
| 0.00239 | 8.13E-11 | NA | + | NA | NA | NA | 6 | 128.504 | -245.008 | 1.916 | 0.14 |
| 0.00562 | NA | NA | + | NA | NA | -7.78E-04 | 6 | 128.465 | -244.93 | 1.993 | 0.135 |
| 0.00202 | NA | NA | + | NA | NA | NA | 5 | 126.29 | -242.58 | 0 | 0.352 |
| 0.01433 | NA | NA | + | 6.76E-05 | NA | NA | 6 | 126.729 | -241.458 | 1.122 | 0.201 |
| 0.02207 | NA | NA | + | NA | -8.91E-03 | NA | 6 | 126.612 | -241.224 | 1.356 | 0.179 |
| 0.00011 | 1.14E-10 | NA | + | NA | NA | NA | 6 | 126.36 | -240.72 | I.86 | 0.139 |
| 0.00421 | NA | NA | + | NA | NA | -9.18E-04 | 6 | 126.295 | -240.589 | 1.991 | 0.13 |
| 0.00108 | NA | NA | + | NA | NA | NA | 5 | 124.331 | -238.663 | 0 | 0.295 |
| 0.0149 | NA | NA | + | 7.05E-05 | NA | NA | 6 | 124.731 | -237.463 | 01.II | 0.162 |
| 0.04634 | NA | NA | NA | 1.43E-04 | NA | NA | 3 | 121.689 | -237.378 | 1.285 | 0.155 |
| 0.01858 | NA | NA | + | NA | NA | -7.50E-03 | 6 | 124.588 | -237.175 | 1.488 | 0.14 |
| 0.01979 | NA | NA | + | NA | -8.22E-03 | NA | 6 | 124.573 | -237.146 | 1.517 | 0.138 |
| 0.00032 | 4.38E-11 | NA | + | NA | NA | NA | 6 | 124.351 | -236.701 | 1.962 | 0.11 |
| 0.00057 | NA | NA | + | NA | NA | NA | 5 | 125.071 | -240.142 | 0 | 0.353 |
| 0.02427 | NA | NA | + | NA | -1.04E-02 | NA | 6 | 125.559 | -239.118 | 1.024 | 0.212 |
| 0.00974 | NA | NA | + | 5.30E-05 | NA | NA | 6 | 125.346 | -238.691 | 1.451 | 0.171 |
| -0.00039 | 5.61E-11 | NA | + | NA | NA | NA | 6 | 125.102 | -238.204 | 1.939 | 0.134 |
| -0.00138 | NA | NA | + | NA | NA | 8.37E-04 | 6 | 125.075 | -238.15 | 1.993 | 0.13 |
| 0.00115 | NA | NA | + | NA | NA | NA | 5 | 122.339 | -234.679 | 0 | 0.336 |
| 0.02617 | NA | NA | + | NA | -1.11E-02 | NA | 6 | 122.956 | -233.912 | 0.766 | 0.229 |
| 0.00959 | NA | NA | + | 5.06E-05 | NA | NA | 6 | 122.613 | -233.225 | 1.453 | 0.162 |
| -0.0016 | 1.63E-10 | NA | + | NA | NA | NA | 6 | 122.467 | -232.934 | 1.745 | 0.14 |
| -0.00724 | NA | NA | + | NA | NA | 3.62E-03 | 6 | 122.415 | -232.83 | 1.849 | 0.133 |
| -0.00056 | NA | NA | + | NA | NA | NA | 5 | 120.79 | -231.581 | 0 | 0.339 |
| 0.02456 | NA | NA | + | NA | -1.10E-02 | NA | 6 | 121.349 | -230.697 | 0.884 | 0.218 |
| 0.00993 | NA | NA | + | 6.33E-05 | NA | NA | 6 | 121.186 | -230.371 | I.21 | 0.185 |
| -0.00218 | 9.81E-11 | NA | + | NA | NA | NA | 6 | 120.853 | -229.706 | 1.875 | 0.133 |
| 0.00307 | NA | NA | + | NA | NA | -1.52E-03 | 6 | 120.801 | -229.603 | 1.978 | 0.126 |
| -0.00018 | NA | NA | + | NA | NA | NA | 5 | 124.986 | -239.972 | 0 | 0.331 |
| 0.01397 | NA | NA | + | 8.35E-05 | NA | NA | 6 | 125.638 | -239.276 | 0.696 | 0.234 |
| 0.02181 | NA | NA | + | NA | -9.56E-03 | NA | 6 | 125.377 | -238.754 | 1.218 | 0.18 |
| -0.00223 | 1.18E-10 | NA | + | NA | NA | NA | 6 | 125.065 | -238.131 | 1.841 | 0.132 |
| 0.00301 | NA | NA | + | NA | NA | -1.34E-03 | 6 | 124.995 | -237.99 | 1.982 | 0.123 |
| 0.00472 | NA | NA | + | NA | NA | NA | 5 | 126.956 | -243.912 | 0 | 0.282 |
| 0.02108 | NA | NA | + | 8.91E-05 | NA | NA | 6 | 127.66 | -243.321 | 0.591 | 0.21 |
| 0.04719 | NA | NA | NA | 1.49E-04 | NA | NA | 3 | 124.342 | -242.684 | 1.228 | 0.152 |
| 0.02473 | NA | NA | + | NA | -8.85E-03 | NA | 6 | 127.243 | -242.487 | 1.425 | 0.138 |
| 0.01379 | NA | NA | + | NA | NA | -3.83E-03 | 6 | 127.03 | -242.059 | 1.852 | 0.112 |
| 0.00369 | 5.90E-11 | NA | + | NA | NA | NA | 6 | 126.982 | -241.965 | 1.947 | 0.106 |
| 0.00037 | NA | NA | + | NA | NA | NA | 5 | 124.829 | -239.658 | 0 | 0.365 |
| 0.01275 | NA | NA | + | 6.87E-05 | NA | NA | 6 | 125.25 | -238.5 | 1.157 | 0.205 |
| 0.01409 | NA | NA | + | NA | -5.99E-03 | NA | 6 | 124.967 | -237.935 | 1.723 | 0.154 |
| -0.00092 | 7.47E-11 | NA | + | NA | NA | NA | 6 | 124.869 | -237.738 | 1.919 | 0.14 |
| -0.00292 | NA | NA | + | NA | NA | 1.41E-03 | 6 | 124.839 | -237.678 | 1.979 | 0.136 |
| 0.00347 | NA | NA | + | NA | NA | NA | 5 | 128.5 | -247.001 | 0 | 0.364 |
| 0.01513 | NA | NA | + | 6.55E-05 | NA | NA | 6 | 128.903 | -245.806 | 1.194 | 0.2 |
| 0.01905 | NA | NA | + | NA | -6.83E-03 | NA | 6 | 128.69 | -245.38 | 1.621 | 0.162 |
| 0.00193 | 8.98E-11 | NA | + | NA | NA | NA | 6 | 128.548 | -245.096 | 1.905 | 0.14 |
| 0.00272 | NA | NA | + | NA | NA | 3.22E-04 | 6 | 128.501 | -245.002 | 1.999 | 0.134 |
| 0.00191 | NA | NA | + | NA | NA | NA | 5 | 125.2 | -240.399 | 0 | 0.36 |
| 0.0232 | NA | NA | + | NA | -9.48E-03 | NA | 6 | 125.585 | -239.17 | 1.229 | 0.195 |
| 0.01048 | NA | NA | + | 5.37E-05 | NA | NA | 6 | 125.472 | -238.944 | 1.455 | 0.174 |
| 0.00034 | 9.02E-11 | NA | + | NA | NA | NA | 6 | 125.248 | -238.496 | 1.903 | 0.139 |
| -0.00011 | NA | NA | + | NA | NA | 8.46E-04 | 6 | 125.204 | -238.407 | 1.992 | 0.133 |
| 0.00451 | NA | NA | + | NA | NA | NA | 5 | 120.316 | -230.633 | 0 | 0.379 |
| 0.0114 | NA | NA | + | 4.34E-05 | NA | NA | 6 | 120.485 | -228.97 | 1.662 | 0.165 |
| -0.00734 | NA | NA | + | NA | NA | 4.97E-03 | 6 | 120.45 | -228.9 | 1.732 | 0.159 |
| 0.01486 | NA | NA | + | NA | -4.67E-03 | NA | 6 | 120.411 | -228.822 | 1.811 | 0.153 |
| 0.0035 | 6.07E-11 | NA | + | NA | NA | NA | 6 | 120.342 | -228.684 | 1.948 | 0.143 |
| -0.00129 | NA | NA | + | NA | NA | NA | 5 | 122.576 | -235.153 | 0 | 0.362 |
| 0.01622 | NA | NA | + | NA | -7.70E-03 | NA | 6 | 122.852 | -233.705 | 1.448 | 0.175 |
| 0.00636 | NA | NA | + | 4.56E-05 | NA | NA | 6 | 122.785 | -233.57 | 1.582 | 0.164 |
| -0.01405 | NA | NA | + | NA | NA | 5.50E-03 | 6 | 122.747 | -233.495 | 1.658 | 0.158 |
| -0.00297 | 9.55E-11 | NA | + | NA | NA | NA | 6 | 122.63 | -233.261 | 1.892 | 0.141 |
| -0.00084 | NA | NA | + | NA | NA | NA | 5 | 124.863 | -239.727 | 0 | 0.33 |
| 0.01462 | NA | NA | + | 8.30E-05 | NA | NA | 6 | 125.486 | -238.971 | 0.756 | 0.226 |
| 0.02522 | NA | NA | + | NA | -1.13E-02 | NA | 6 | 125.324 | -238.648 | 1.079 | 0.192 |
| 0.00628 | NA | NA | + | NA | NA | -3.01E-03 | 6 | 124.91 | -237.821 | 1.906 | 0.127 |
| -0.00163 | 4.98E-11 | NA | + | NA | NA | NA | 6 | 124.894 | -237.787 | 1.939 | 0.125 |
| 0.00546 | NA | NA | + | NA | NA | NA | 5 | 123.169 | -236.339 | 0 | 0.374 |
| 0.01659 | NA | NA | + | 6.63E-05 | NA | NA | 6 | 123.57 | -235.139 | 01.II | 0.205 |
| 0.0126 | NA | NA | + | NA | -3.18E-03 | NA | 6 | 123.211 | -234.422 | 1.917 | 0.143 |
| 0.00474 | 4.26E-11 | NA | + | NA | NA | NA | 6 | 123.186 | -234.371 | 1.968 | 0.14 |
| 0.00612 | NA | NA | + | NA | NA | -2.75E-04 | 6 | 123.17 | -234.34 | 1.999 | 0.138 |
| 0.00339 | NA | NA | + | NA | NA | NA | 5 | 125.884 | -241.768 | 0 | 0.338 |
| 0.01825 | NA | NA | + | 8.47E-05 | NA | NA | 6 | 126.52 | -241.039 | 0.729 | 0.235 |
| 0.02247 | NA | NA | + | NA | -8.45E-03 | NA | 6 | 126.159 | -240.317 | 1.451 | 0.164 |
| 0.01272 | NA | NA | + | NA | NA | -3.87E-03 | 6 | 125.959 | -239.918 | 1.849 | 0.134 |
| 0.00227 | 6.69E-11 | NA | + | NA | NA | NA | 6 | 125.917 | -239.835 | 1.933 | 0.129 |
| 0.00529 | NA | NA | + | NA | NA | NA | 5 | 125.762 | -241.524 | 0 | 0.34 |
| 0.02757 | NA | NA | + | NA | -1.00E-02 | NA | 6 | 126.349 | -240.698 | 0.826 | 0.225 |
| 0.01213 | NA | NA | + | 4.74E-05 | NA | NA | 6 | 126.004 | -240.008 | 1.516 | 0.16 |
| 0.00309 | 1.37E-10 | NA | + | NA | NA | NA | 6 | 125.859 | -239.719 | 1.805 | 0.138 |
| -0.00407 | NA | NA | + | NA | NA | 3.90E-03 | 6 | 125.849 | -239.698 | 1.826 | 0.137 |
| 0.00685 | NA | NA | + | NA | NA | NA | 5 | 121.467 | -232.934 | 0 | 0.298 |
| 0.02219 | NA | NA | + | 9.33E-05 | NA | NA | 6 | 122.237 | -232.473 | 0.461 | 0.237 |
| 0.02013 | NA | NA | + | NA | -5.95E-03 | NA | 6 | 121.618 | -231.236 | 1.697 | 0.128 |
| 0.00514 | 1.00E-10 | NA | + | NA | NA | NA | 6 | 121.532 | -231.063 | 1.871 | 0.117 |
| 0.049 | NA | NA | NA | 1.52E-04 | NA | NA | 3 | 118.482 | -230.964 | I.97 | 0.111 |
| 0.00756 | NA | NA | + | NA | NA | -2.94E-04 | 6 | 121.467 | -230.935 | 1.999 | 0.11 |
| 0.00266 | NA | NA | + | NA | NA | NA | 5 | 127.482 | -244.963 | 0 | 0.353 |
| 0.01592 | NA | NA | + | 7.00E-05 | NA | NA | 6 | 127.914 | -243.828 | 1.135 | 0.2 |
| 0.02373 | NA | NA | + | NA | -9.18E-03 | NA | 6 | 127.795 | -243.591 | 1.372 | 0.178 |
| 0.01007 | NA | NA | + | NA | NA | -3.12E-03 | 6 | 127.534 | -243.068 | 1.895 | 0.137 |
| 0.00176 | 5.08E-11 | NA | + | NA | NA | NA | 6 | 127.501 | -243.002 | 1.961 | 0.132 |
| 0.00208 | NA | NA | + | NA | NA | NA | 5 | 121.616 | -233.232 | 0 | 0.297 |
| 0.01902 | NA | NA | + | 8.97E-05 | NA | NA | 6 | 122.269 | -232.537 | 0.694 | 0.21 |
| 0.02243 | NA | NA | + | NA | -8.72E-03 | NA | 6 | 121.874 | -231.749 | 1.483 | 0.142 |
| 0.01561 | NA | NA | + | NA | NA | -5.77E-03 | 6 | 121.779 | -231.557 | 1.674 | 0.129 |
| 0.00105 | 5.75E-11 | NA | + | NA | NA | NA | 6 | 121.637 | -231.274 | 1.957 | 0.112 |
| 0.04779 | NA | NA | NA | 1.49E-04 | NA | NA | 3 | 118.624 | -231.248 | 1.984 | 0.11 |
| 0.00177 | NA | NA | + | NA | NA | NA | 5 | 117.533 | -225.067 | 0 | 0.307 |
| 0.01561 | NA | NA | + | 7.54E-05 | NA | NA | 6 | 117.956 | -223.912 | 1.155 | 0.172 |
| 0.02193 | NA | NA | + | NA | -8.92E-03 | NA | 6 | 117.79 | -223.581 | 1.486 | 0.146 |
| 0.04593 | NA | NA | NA | 1.49E-04 | NA | NA | 3 | 114.731 | -223.462 | 1.605 | 0.138 |
| 0.00021 | 9.19E-11 | NA | + | NA | NA | NA | 6 | 117.584 | -223.167 | 01.IX | 0.119 |
| 0.00881 | NA | NA | + | NA | NA | -2.89E-03 | 6 | 117.574 | -223.148 | 1.919 | 0.118 |
| 0.00238 | NA | NA | + | NA | NA | NA | 5 | 126.04 | -242.079 | 0 | 0.358 |
| 0.01199 | NA | NA | + | 5.66E-05 | NA | NA | 6 | 126.356 | -240.711 | 1.368 | 0.181 |
| 0.02079 | NA | NA | + | NA | -8.16E-03 | NA | 6 | 126.35 | -240.7 | 1.379 | 0.18 |
| -0.00606 | NA | NA | + | NA | NA | 3.64E-03 | 6 | 126.118 | -240.236 | 1.843 | 0.143 |
| 0.00085 | 9.05E-11 | NA | + | NA | NA | NA | 6 | 126.086 | -240.173 | 1.907 | 0.138 |
| 0.00455 | NA | NA | + | NA | NA | NA | 5 | 128.297 | -246.594 | 0 | 0.35 |
| 0.02693 | NA | NA | + | NA | -9.93E-03 | NA | 6 | 128.736 | -245.472 | 1.122 | 0.2 |
| 0.01403 | NA | NA | + | 5.79E-05 | NA | NA | 6 | 128.626 | -245.251 | 1.342 | 0.179 |
| 0.00269 | 1.17E-10 | NA | + | NA | NA | NA | 6 | 128.373 | -244.747 | 1.847 | 0.139 |
| 0.01042 | NA | NA | + | NA | NA | -2.48E-03 | 6 | 128.327 | -244.654 | I.94 | 0.133 |
| 0.00436 | NA | NA | + | NA | NA | NA | 5 | 127.94 | -245.88 | 0 | 0.347 |
| 0.02841 | NA | NA | + | NA | -1.08E-02 | NA | 6 | 128.444 | -244.888 | 0.992 | 0.212 |
| 0.01423 | NA | NA | + | 5.70E-05 | NA | NA | 6 | 128.257 | -244.514 | 1.366 | 0.175 |
| 0.00263 | 1.03E-10 | NA | + | NA | NA | NA | 6 | 128.002 | -244.004 | 1.876 | 0.136 |
| 0.00833 | NA | NA | + | NA | NA | -1.67E-03 | 6 | 127.955 | -243.909 | 1.971 | 0.13 |
| 0.00317 | NA | NA | + | NA | NA | NA | 5 | 121.769 | -233.538 | 0 | 0.295 |
| 0.01875 | NA | NA | + | 8.62E-05 | NA | NA | 6 | 122.374 | -232.749 | 0.789 | 0.199 |
| 0.02234 | NA | NA | + | NA | -8.36E-03 | NA | 6 | 122.045 | -232.09 | 1.447 | 0.143 |
| 0.04787 | NA | NA | NA | 1.50E-04 | NA | NA | 3 | 118.971 | -231.942 | 1.596 | 0.133 |
| 0.00082 | 1.32E-10 | NA | + | NA | NA | NA | 6 | 121.872 | -231.744 | 1.793 | 0.12 |
| 0.00615 | NA | NA | + | NA | NA | -1.26E-03 | 6 | 121.777 | -231.553 | 1.984 | 0.109 |
| 0.00048 | NA | NA | + | NA | NA | NA | 5 | 126.311 | -242.623 | 0 | 0.342 |
| 0.01399 | NA | NA | + | 7.70E-05 | NA | NA | 6 | 126.896 | -241.792 | 0.831 | 0.226 |
| 0.02015 | NA | NA | + | NA | -8.78E-03 | NA | 6 | 126.633 | -241.267 | 1.356 | 0.174 |
| -0.00055 | 5.79E-11 | NA | + | NA | NA | NA | 6 | 126.346 | -240.692 | 1.931 | 0.13 |
| -0.0032 | NA | NA | + | NA | NA | 1.52E-03 | 6 | 126.325 | -240.65 | 1.973 | 0.128 |
| -0.00024 | NA | NA | + | NA | NA | NA | 5 | 119.684 | -229.368 | 0 | 0.35 |
| 0.01363 | NA | NA | + | 7.44E-05 | NA | NA | 6 | 120.13 | -228.259 | 1.109 | 0.201 |
| 0.02089 | NA | NA | + | NA | -9.21E-03 | NA | 6 | 119.989 | -227.978 | I.39 | 0.175 |
| 0.01016 | NA | NA | + | NA | NA | -4.41E-03 | 6 | 119.777 | -227.553 | 1.815 | 0.141 |
| -0.0015 | 7.00E-11 | NA | + | NA | NA | NA | 6 | 119.719 | -227.438 | I.93 | 0.133 |
| 0.00283 | NA | NA | + | NA | NA | NA | 5 | 122.222 | -234.444 | 0 | 0.36 |
| 0.02264 | NA | NA | + | NA | -8.68E-03 | NA | 6 | 122.584 | -233.168 | 1.275 | 0.19 |
| 0.01046 | NA | NA | + | 4.76E-05 | NA | NA | 6 | 122.449 | -232.898 | 1.546 | 0.166 |
| -0.00621 | NA | NA | + | NA | NA | 3.89E-03 | 6 | 122.313 | -232.625 | 1.818 | 0.145 |
| 0.00143 | 8.02E-11 | NA | + | NA | NA | NA | 6 | 122.265 | -232.531 | 1.913 | 0.138 |
| 0.00302 | NA | NA | + | NA | NA | NA | 5 | 126.652 | -243.303 | 0 | 0.26 |
| 0.01797 | NA | NA | + | 9.13E-05 | NA | NA | 6 | 127.495 | -242.99 | 0.313 | 0.223 |
| 0.02784 | NA | NA | + | NA | -1.11E-02 | NA | 6 | 127.301 | -242.601 | 0.702 | 0.183 |
| 0.03682 | NA | NA | + | 8.01E-05 | -9.24E-03 | NA | 7 | 127.937 | -241.874 | 1.429 | 0.127 |
| 0.00044 | 1.53E-10 | NA | + | NA | NA | NA | 6 | 126.787 | -241.573 | I.73 | 0.11 |
| -0.00089 | NA | NA | + | NA | NA | 1.62E-03 | 6 | 126.665 | -241.33 | 1.973 | 0.097 |
| 0.00394 | NA | NA | + | NA | NA | NA | 5 | 120.449 | -230.899 | 0 | 0.305 |
| 0.02054 | NA | NA | + | 8.74E-05 | NA | NA | 6 | 121.02 | -230.039 | 0.859 | 0.198 |
| 0.04645 | NA | NA | NA | 1.49E-04 | NA | NA | 3 | 117.727 | -229.453 | 1.446 | 0.148 |
| 0.01311 | NA | NA | + | NA | -4.05E-03 | NA | 6 | 120.501 | -229.002 | 1.896 | 0.118 |
| 0.01147 | NA | NA | + | NA | NA | -3.12E-03 | 6 | 120.498 | -228.995 | 1.903 | 0.118 |
| 0.00315 | 4.57E-11 | NA | + | NA | NA | NA | 6 | 120.463 | -228.925 | 1.974 | 0.114 |
| 0.00211 | NA | NA | + | NA | NA | NA | 5 | 122.708 | -235.416 | 0 | 0.349 |
| 0.01543 | NA | NA | + | 7.81E-05 | NA | NA | 6 | 123.279 | -234.559 | 0.857 | 0.227 |
| 0.01618 | NA | NA | + | NA | -6.27E-03 | NA | 6 | 122.879 | -233.757 | 1.659 | 0.152 |
| 0.00006 | 1.19E-10 | NA | + | NA | NA | NA | 6 | 122.79 | -233.58 | 1.836 | 0.139 |
| -0.00371 | NA | NA | + | NA | NA | 2.42E-03 | 6 | 122.741 | -233.482 | 1.934 | 0.133 |
| -0.00076 | NA | NA | + | NA | NA | NA | 5 | 126.145 | -242.289 | 0 | 0.357 |
| 0.0221 | NA | NA | + | NA | -1.01E-02 | NA | 6 | 126.538 | -241.076 | 1.213 | 0.195 |
| 0.01051 | NA | NA | + | 6.04E-05 | NA | NA | 6 | 126.467 | -240.934 | 1.355 | 0.182 |
| -0.00166 | 5.25E-11 | NA | + | NA | NA | NA | 6 | 126.167 | -240.334 | 1.955 | 0.134 |
| -0.00066 | NA | NA | + | NA | NA | -4.27E-05 | 6 | 126.145 | -240.289 | 2 | 0.132 |
| 0.00517 | NA | NA | + | NA | NA | NA | 5 | 123.765 | -237.53 | 0 | 0.342 |
| 0.01749 | NA | NA | + | 7.35E-05 | NA | NA | 6 | 124.232 | -236.464 | 1.066 | 0.201 |
| 0.02906 | NA | NA | + | NA | -1.05E-02 | NA | 6 | 124.196 | -236.392 | 1.138 | 0.194 |
| 0.01468 | NA | NA | + | NA | NA | -3.93E-03 | 6 | 123.839 | -235.679 | 1.852 | 0.135 |
| 0.00439 | 4.61E-11 | NA | + | NA | NA | NA | 6 | 123.788 | -235.576 | 1.954 | 0.129 |
| 0.00159 | NA | NA | + | NA | NA | NA | 5 | 124.823 | -239.647 | 0 | 0.359 |
| 0.0245 | NA | NA | + | NA | -1.03E-02 | NA | 6 | 125.275 | -238.55 | 1.097 | 0.207 |
| 0.00982 | NA | NA | + | 4.89E-05 | NA | NA | 6 | 125.05 | -238.1 | 1.547 | 0.166 |
| 0.00049 | 6.36E-11 | NA | + | NA | NA | NA | 6 | 124.847 | -237.694 | 1.952 | 0.135 |
| 0.00343 | NA | NA | + | NA | NA | -7.71E-04 | 6 | 124.826 | -237.653 | 1.994 | 0.133 |
| 0.00401 | NA | NA | + | NA | NA | NA | 5 | 125.541 | -241.082 | 0 | 0.335 |
| 0.03237 | NA | NA | + | NA | -1.26E-02 | NA | 6 | 126.19 | -240.38 | 0.702 | 0.236 |
| 0.01411 | NA | NA | + | 5.70E-05 | NA | NA | 6 | 125.842 | -239.684 | 1.398 | 0.167 |
| 0.00183 | 1.31E-10 | NA | + | NA | NA | NA | 6 | 125.615 | -239.231 | 1.851 | 0.133 |
| 0.01152 | NA | NA | + | NA | NA | -3.16E-03 | 6 | 125.593 | -239.186 | 1.896 | 0.13 |
| 0.00492 | NA | NA | + | NA | NA | NA | 5 | 121.417 | -232.835 | 0 | 0.328 |
| 0.01528 | NA | NA | + | 5.85E-05 | NA | NA | 6 | 121.707 | -231.414 | 1.421 | 0.161 |
| 0.04669 | NA | NA | NA | 1.32E-04 | NA | NA | 3 | 118.543 | -231.087 | 1.748 | 0.137 |
| 0.01494 | NA | NA | + | NA | -4.40E-03 | NA | 6 | 121.488 | -230.975 | I.86 | 0.129 |
| 0.00368 | 7.36E-11 | NA | + | NA | NA | NA | 6 | 121.449 | -230.898 | 1.937 | 0.124 |
| 0.00431 | NA | NA | + | NA | NA | 2.58E-04 | 6 | 121.418 | -230.836 | 1.999 | 0.121 |
| 0.00096 | NA | NA | + | NA | NA | NA | 5 | 122.153 | -234.307 | 0 | 0.347 |
| 0.01442 | NA | NA | + | 7.38E-05 | NA | NA | 6 | 122.622 | -233.243 | 1.063 | 0.204 |
| 0.02378 | NA | NA | + | NA | -1.00E-02 | NA | 6 | 122.538 | -233.077 | I.23 | 0.188 |
| 0.00585 | NA | NA | + | NA | NA | -2.04E-03 | 6 | 122.176 | -232.352 | 1.955 | 0.131 |
| -0.00004 | 5.45E-11 | NA | + | NA | NA | NA | 6 | 122.172 | -232.343 | 1.964 | 0.13 |
| -0.00032 | NA | NA | + | NA | NA | NA | 5 | 110.618 | -211.235 | 0 | 0.358 |
| 0.01499 | NA | NA | + | 8.16E-05 | NA | NA | 6 | 111.087 | -210.173 | 1.062 | 0.21 |
| 0.01916 | NA | NA | + | NA | -8.39E-03 | NA | 6 | 110.83 | -209.66 | 1.575 | 0.163 |
| 0.00586 | NA | NA | + | NA | NA | -2.61E-03 | 6 | 110.651 | -209.302 | 1.933 | 0.136 |
| -0.00124 | 4.50E-11 | NA | + | NA | NA | NA | 6 | 110.627 | -209.253 | 1.982 | 0.133 |
| 0.00473 | NA | NA | + | NA | NA | NA | 5 | 117.521 | -225.043 | 0 | 0.356 |
| 0.02867 | NA | NA | + | NA | -1.06E-02 | NA | 6 | 117.964 | -223.928 | 1.114 | 0.204 |
| 0.01423 | NA | NA | + | 5.55E-05 | NA | NA | 6 | 117.788 | -223.575 | 1.467 | 0.171 |
| 0.00319 | 8.97E-11 | NA | + | NA | NA | NA | 6 | 117.561 | -223.122 | 1.921 | 0.136 |
| 0.00245 | NA | NA | + | NA | NA | 9.55E-04 | 6 | 117.526 | -223.052 | 1.991 | 0.132 |
| 0.00087 | NA | NA | + | NA | NA | NA | 5 | 121.221 | -232.442 | 0 | 0.28 |
| 0.04805 | NA | NA | NA | 1.62E-04 | NA | NA | 3 | 118.877 | -231.754 | 0.688 | 0.199 |
| 0.01889 | NA | NA | + | 8.99E-05 | NA | NA | 6 | 121.819 | -231.638 | 0.804 | 0.187 |
| 0.01688 | NA | NA | + | NA | -7.00E-03 | NA | 6 | 121.369 | -230.738 | 1.704 | 0.12 |
| -0.00044 | 7.89E-11 | NA | + | NA | NA | NA | 6 | 121.264 | -230.527 | 1.915 | 0.108 |
| 0.00716 | NA | NA | + | NA | NA | -2.63E-03 | 6 | 121.255 | -230.511 | 1.931 | 0.107 |
| 0.00168 | NA | NA | + | NA | NA | NA | 5 | 123.67 | -237.34 | 0 | 0.378 |
| 0.01103 | NA | NA | + | 5.36E-05 | NA | NA | 6 | 123.926 | -235.853 | 1.487 | 0.18 |
| 0.01535 | NA | NA | + | NA | -6.03E-03 | NA | 6 | 123.81 | -235.621 | I.72 | 0.16 |
| 0.0006 | 6.16E-11 | NA | + | NA | NA | NA | 6 | 123.695 | -235.389 | 1.951 | 0.143 |
| 0.00168 | NA | NA | + | NA | NA | -1.50E-06 | 6 | 123.67 | -235.34 | 2 | 0.139 |
| -0.00106 | NA | NA | + | NA | NA | NA | 5 | 123.25 | -236.499 | 0 | 0.334 |
| 0.02209 | NA | NA | + | NA | -1.02E-02 | NA | 6 | 123.786 | -235.572 | 0.927 | 0.21 |
| -0.01986 | NA | NA | + | NA | NA | 7.92E-03 | 6 | 123.656 | -235.312 | 1.187 | 0.185 |
| 0.00453 | NA | NA | + | 3.67E-05 | NA | NA | 6 | 123.394 | -234.787 | 1.712 | 0.142 |
| -0.00252 | 7.81E-11 | NA | + | NA | NA | NA | 6 | 123.295 | -234.589 | I.91 | 0.129 |
| 0.00672 | NA | NA | + | NA | NA | NA | 5 | 125.537 | -241.074 | 0 | 0.305 |
| 0.0203 | NA | NA | + | 7.96E-05 | NA | NA | 6 | 126.114 | -240.227 | 0.847 | 0.2 |
| 0.0476 | NA | NA | NA | 1.36E-04 | NA | NA | 3 | 122.726 | -239.451 | 1.623 | 0.136 |
| 0.02131 | NA | NA | + | NA | -6.47E-03 | NA | 6 | 125.709 | -239.418 | 1.656 | 0.133 |
| 0.00616 | 3.27E-11 | NA | + | NA | NA | NA | 6 | 125.552 | -239.104 | I.97 | 0.114 |
| 0.00691 | NA | NA | + | NA | NA | -7.77E-05 | 6 | 125.537 | -239.074 | 2 | 0.112 |
| 0.0015 | NA | NA | + | NA | NA | NA | 5 | 120.579 | -231.158 | 0 | 0.348 |
| 0.01417 | NA | NA | + | 7.08E-05 | NA | NA | 6 | 121.014 | -230.028 | I.13 | 0.198 |
| 0.02474 | NA | NA | + | NA | -1.02E-02 | NA | 6 | 120.997 | -229.994 | 1.164 | 0.194 |
| 0.00026 | 7.17E-11 | NA | + | NA | NA | NA | 6 | 120.61 | -229.22 | 1.938 | 0.132 |
| 0.00246 | NA | NA | + | NA | NA | -4.03E-04 | 6 | 120.58 | -229.16 | 1.998 | 0.128 |
| 0.00535 | NA | NA | + | NA | NA | NA | 5 | 125.403 | -240.806 | 0 | 0.36 |
| 0.01528 | NA | NA | + | 6.14E-05 | NA | NA | 6 | 125.762 | -239.525 | 1.281 | 0.19 |
| 0.02157 | NA | NA | + | NA | -7.36E-03 | NA | 6 | 125.647 | -239.294 | 1.512 | 0.169 |
| -0.00325 | NA | NA | + | NA | NA | 3.51E-03 | 6 | 125.471 | -238.941 | 1.865 | 0.142 |
| 0.00373 | 9.53E-11 | NA | + | NA | NA | NA | 6 | 125.457 | -238.913 | 1.893 | 0.14 |
| 0.00422 | NA | NA | + | NA | NA | NA | 5 | 123.542 | -237.085 | 0 | 0.367 |
| 0.0152 | NA | NA | + | 6.17E-05 | NA | NA | 6 | 123.867 | -235.734 | I.35 | 0.187 |
| 0.02266 | NA | NA | + | NA | -8.12E-03 | NA | 6 | 123.783 | -235.567 | 1.518 | 0.172 |
| 0.00319 | 5.85E-11 | NA | + | NA | NA | NA | 6 | 123.566 | -235.131 | 1.953 | 0.138 |
| 0.00744 | NA | NA | + | NA | NA | -1.37E-03 | 6 | 123.552 | -235.105 | I.98 | 0.136 |
| 0.00525 | NA | NA | + | NA | NA | NA | 5 | 120.894 | -231.788 | 0 | 0.362 |
| 0.01599 | NA | NA | + | 6.08E-05 | NA | NA | 6 | 121.191 | -230.381 | 1.407 | 0.179 |
| 0.02442 | NA | NA | + | NA | -8.47E-03 | NA | 6 | 121.166 | -230.331 | 1.457 | 0.175 |
| 0.01547 | NA | NA | + | NA | NA | -4.23E-03 | 6 | 120.98 | -229.961 | 1.827 | 0.145 |
| 0.00353 | 1.02E-10 | NA | + | NA | NA | NA | 6 | 120.941 | -229.882 | 1.906 | 0.139 |
| 0.00355 | NA | NA | + | NA | NA | NA | 5 | 123.594 | -237.188 | 0 | 0.354 |
| 0.01671 | NA | NA | + | 7.57E-05 | NA | NA | 6 | 124.114 | -236.228 | 0.96 | 0.219 |
| 0.02041 | NA | NA | + | NA | -7.39E-03 | NA | 6 | 123.811 | -235.622 | 1.565 | 0.162 |
| 0.00247 | 6.61E-11 | NA | + | NA | NA | NA | 6 | 123.626 | -235.251 | 1.937 | 0.134 |
| 0.00263 | NA | NA | + | NA | NA | 3.87E-04 | 6 | 123.595 | -235.19 | 1.998 | 0.13 |
| 0.00262 | NA | NA | + | NA | NA | NA | 5 | 121.442 | -232.884 | 0 | 0.309 |
| 0.01714 | NA | NA | + | 7.80E-05 | NA | NA | 6 | 121.956 | -231.913 | 0.971 | 0.19 |
| 0.04667 | NA | NA | NA | 1.46E-04 | NA | NA | 3 | 118.633 | -231.266 | 1.617 | 0.138 |
| 0.01644 | NA | NA | + | NA | -6.18E-03 | NA | 6 | 121.582 | -231.163 | I.72 | 0.131 |
| 0.0013 | 7.58E-11 | NA | + | NA | NA | NA | 6 | 121.476 | -230.952 | 1.931 | 0.118 |
| 0.00028 | NA | NA | + | NA | NA | 9.88E-04 | 6 | 121.447 | -230.894 | I.99 | 0.114 |
| 0.00259 | NA | NA | + | NA | NA | NA | 5 | 122.758 | -235.516 | 0 | 0.331 |
| 0.0171 | NA | NA | + | 8.64E-05 | NA | NA | 6 | 123.438 | -234.875 | 0.641 | 0.24 |
| 0.02417 | NA | NA | + | NA | -9.70E-03 | NA | 6 | 123.138 | -234.276 | I.24 | 0.178 |
| 0.00131 | 8.08E-11 | NA | + | NA | NA | NA | 6 | 122.814 | -233.628 | 1.888 | 0.129 |
| 0.00645 | NA | NA | + | NA | NA | -1.58E-03 | 6 | 122.77 | -233.54 | 1.976 | 0.123 |
| 0.00574 | NA | NA | + | NA | NA | NA | 5 | 123.132 | -236.264 | 0 | 0.37 |
| 0.01747 | NA | NA | + | 6.37E-05 | NA | NA | 6 | 123.484 | -234.969 | 1.295 | 0.193 |
| 0.01923 | NA | NA | + | NA | -5.93E-03 | NA | 6 | 123.277 | -234.554 | I.71 | 0.157 |
| 0.00449 | 7.40E-11 | NA | + | NA | NA | NA | 6 | 123.162 | -234.324 | I.94 | 0.14 |
| 0.00049 | NA | NA | + | NA | NA | 2.24E-03 | 6 | 123.161 | -234.321 | 1.943 | 0.14 |
| 0.00428 | NA | NA | + | NA | NA | NA | 5 | 126.773 | -243.547 | 0 | 0.349 |
| 0.01599 | NA | NA | + | 6.43E-05 | NA | NA | 6 | 127.192 | -242.384 | 1.163 | 0.195 |
| 0.02588 | NA | NA | + | NA | -9.52E-03 | NA | 6 | 127.169 | -242.337 | 1.209 | 0.191 |
| 0.00248 | 1.09E-10 | NA | + | NA | NA | NA | 6 | 126.836 | -241.671 | 1.875 | 0.137 |
| 0.00342 | NA | NA | + | NA | NA | 3.70E-04 | 6 | 126.774 | -241.548 | 1.998 | 0.128 |
| 0.00261 | NA | NA | + | NA | NA | NA | 5 | 125.624 | -241.247 | 0 | 0.352 |
| 0.02212 | NA | NA | + | NA | -8.55E-03 | NA | 6 | 126.006 | -240.012 | 1.235 | 0.19 |
| -0.01227 | NA | NA | + | NA | NA | 6.48E-03 | 6 | 125.877 | -239.755 | 1.493 | 0.167 |
| 0.00861 | NA | NA | + | 3.92E-05 | NA | NA | 6 | 125.791 | -239.582 | 1.665 | 0.153 |
| 0.00077 | 1.12E-10 | NA | + | NA | NA | NA | 6 | 125.69 | -239.38 | 1.868 | 0.138 |
| 0.00175 | NA | NA | + | NA | NA | NA | 5 | 123.963 | -237.926 | 0 | 0.341 |
| 0.02669 | NA | NA | + | NA | -1.10E-02 | NA | 6 | 124.49 | -236.981 | 0.945 | 0.213 |
| 0.01308 | NA | NA | + | 6.57E-05 | NA | NA | 6 | 124.363 | -236.727 | 1.199 | 0.187 |
| 0.00017 | 9.76E-11 | NA | + | NA | NA | NA | 6 | 124.019 | -236.037 | 1.889 | 0.133 |
| 0.0026 | NA | NA | + | NA | NA | -3.53E-04 | 6 | 123.963 | -235.927 | 1.999 | 0.126 |
| 0.00318 | NA | NA | + | NA | NA | NA | 5 | 127.023 | -244.046 | 0 | 0.362 |
| 0.01434 | NA | NA | + | 6.28E-05 | NA | NA | 6 | 127.383 | -242.765 | I.28 | 0.191 |
| 0.02097 | NA | NA | + | NA | -7.93E-03 | NA | 6 | 127.297 | -242.593 | 1.453 | 0.175 |
| 0.00233 | 4.91E-11 | NA | + | NA | NA | NA | 6 | 127.051 | -242.101 | 1.945 | 0.137 |
| 0.0082 | NA | NA | + | NA | NA | -2.08E-03 | 6 | 127.045 | -242.089 | 1.957 | 0.136 |
| 0.00201 | NA | NA | + | NA | NA | NA | 5 | 124.524 | -239.048 | 0 | 0.336 |
| 0.02911 | NA | NA | + | NA | -1.19E-02 | NA | 6 | 125.075 | -238.151 | 0.897 | 0.214 |
| 0.01458 | NA | NA | + | 6.70E-05 | NA | NA | 6 | 124.931 | -237.862 | 1.185 | 0.186 |
| -0.00017 | 1.18E-10 | NA | + | NA | NA | NA | 6 | 124.609 | -237.217 | I.83 | 0.134 |
| 0.00949 | NA | NA | + | NA | NA | -3.13E-03 | 6 | 124.571 | -237.143 | 1.905 | 0.13 |
| 0.00247 | NA | NA | + | NA | NA | NA | 5 | 125.388 | -240.777 | 0 | 0.308 |
| 0.01714 | NA | NA | + | 8.04E-05 | NA | NA | 6 | 125.977 | -239.954 | 0.823 | 0.204 |
| 0.01701 | NA | NA | + | NA | -6.34E-03 | NA | 6 | 125.547 | -239.094 | 1.683 | 0.133 |
| 0.04651 | NA | NA | NA | 1.46E-04 | NA | NA | 3 | 122.475 | -238.951 | 1.826 | 0.124 |
| 0.00156 | 5.31E-11 | NA | + | NA | NA | NA | 6 | 125.411 | -238.822 | 1.955 | 0.116 |
| 0.00533 | NA | NA | + | NA | NA | -1.22E-03 | 6 | 125.397 | -238.793 | 1.984 | 0.114 |
| 0.00415 | NA | NA | + | NA | NA | NA | 5 | 124.599 | -239.198 | 0 | 0.361 |
| 0.01679 | NA | NA | + | 7.05E-05 | NA | NA | 6 | 125.012 | -238.024 | 1.174 | 0.201 |
| 0.02192 | NA | NA | + | NA | -7.89E-03 | NA | 6 | 124.83 | -237.66 | 1.538 | 0.167 |
| 0.00289 | 7.19E-11 | NA | + | NA | NA | NA | 6 | 124.638 | -237.276 | 1.922 | 0.138 |
| 0.00704 | NA | NA | + | NA | NA | -1.20E-03 | 6 | 124.606 | -237.213 | 1.985 | 0.134 |
| 0.00634 | NA | NA | + | NA | NA | NA | 5 | 122.392 | -234.784 | 0 | 0.3 |
| 0.02148 | NA | NA | + | 8.22E-05 | NA | NA | 6 | 122.928 | -233.856 | 0.928 | 0.189 |
| 0.04792 | NA | NA | NA | 1.44E-04 | NA | NA | 3 | 119.695 | -233.391 | 1.393 | 0.15 |
| 0.02242 | NA | NA | + | NA | -7.13E-03 | NA | 6 | 122.572 | -233.145 | 1.639 | 0.132 |
| 0.00449 | 1.12E-10 | NA | + | NA | NA | NA | 6 | 122.448 | -232.897 | 1.887 | 0.117 |
| 0.0107 | NA | NA | + | NA | NA | -1.81E-03 | 6 | 122.409 | -232.817 | 1.967 | 0.112 |
| 0.00401 | NA | NA | + | NA | NA | NA | 5 | 128.869 | -247.737 | 0 | 0.286 |
| 0.0292 | NA | NA | + | NA | -1.14E-02 | NA | 6 | 129.67 | -247.34 | 0.397 | 0.234 |
| -0.01198 | NA | NA | + | NA | NA | 6.63E-03 | 6 | 129.129 | -246.258 | 1.479 | 0.136 |
| 0.0094 | NA | NA | + | 3.87E-05 | NA | NA | 6 | 129.047 | -246.093 | 1.644 | 0.126 |
| 0.00264 | 8.11E-11 | NA | + | NA | NA | NA | 6 | 128.922 | -245.843 | 1.894 | 0.111 |
| 0.01396 | NA | NA | + | NA | -1.11E-02 | 6.03E-03 | 7 | 129.887 | -245.775 | 1.962 | 0.107 |
| 0.00188 | NA | NA | + | NA | NA | NA | 5 | 127.506 | -245.011 | 0 | 0.351 |
| 0.02729 | NA | NA | + | NA | -1.13E-02 | NA | 6 | 128.032 | -244.064 | 0.947 | 0.219 |
| 0.00981 | NA | NA | + | 4.61E-05 | NA | NA | 6 | 127.71 | -243.42 | 1.591 | 0.159 |
| 0.00028 | 9.45E-11 | NA | + | NA | NA | NA | 6 | 127.558 | -243.117 | 1.895 | 0.136 |
| 0.00905 | NA | NA | + | NA | NA | -3.03E-03 | 6 | 127.552 | -243.103 | 1.908 | 0.135 |
| 0.00806 | NA | NA | + | NA | NA | NA | 5 | 132.125 | -254.249 | 0 | 0.344 |
| 0.03451 | NA | NA | + | NA | -1.17E-02 | NA | 6 | 132.834 | -253.667 | 0.582 | 0.257 |
| 0.01307 | NA | NA | + | 3.17E-05 | NA | NA | 6 | 132.239 | -252.478 | 1.772 | 0.142 |
| 0.00717 | 5.65E-11 | NA | + | NA | NA | NA | 6 | 132.15 | -252.299 | I.95 | 0.13 |
| 0.00782 | NA | NA | + | NA | NA | 1.04E-04 | 6 | 132.125 | -252.249 | 2 | 0.127 |
| 0.0037 | NA | NA | + | NA | NA | NA | 5 | 123.725 | -237.451 | 0 | 0.349 |
| 0.0171 | NA | NA | + | 7.73E-05 | NA | NA | 6 | 124.233 | -236.466 | 0.984 | 0.213 |
| 0.02288 | NA | NA | + | NA | -8.60E-03 | NA | 6 | 124.013 | -236.025 | 1.425 | 0.171 |
| 0.01132 | NA | NA | + | NA | NA | -3.11E-03 | 6 | 123.772 | -235.544 | 1.907 | 0.135 |
| 0.00261 | 6.03E-11 | NA | + | NA | NA | NA | 6 | 123.752 | -235.504 | 1.947 | 0.132 |
| 0.00248 | NA | NA | + | NA | NA | NA | 5 | 128.012 | -246.023 | 0 | 0.349 |
| 0.02541 | NA | NA | + | NA | -1.01E-02 | NA | 6 | 128.475 | -244.95 | 1.073 | 0.204 |
| 0.01152 | NA | NA | + | 5.62E-05 | NA | NA | 6 | 128.323 | -244.647 | 1.377 | 0.176 |
| 0.0002 | 1.32E-10 | NA | + | NA | NA | NA | 6 | 128.109 | -244.218 | 1.806 | 0.142 |
| 0.00456 | NA | NA | + | NA | NA | -8.84E-04 | 6 | 128.016 | -244.031 | 1.992 | 0.129 |
| 0.00167 | NA | NA | + | NA | NA | NA | 5 | 120.185 | -230.37 | 0 | 0.37 |
| 0.01822 | NA | NA | + | NA | -7.31E-03 | NA | 6 | 120.433 | -228.865 | 1.505 | 0.174 |
| 0.00945 | NA | NA | + | 4.75E-05 | NA | NA | 6 | 120.399 | -228.799 | 1.572 | 0.169 |
| -0.00023 | 1.14E-10 | NA | + | NA | NA | NA | 6 | 120.264 | -228.528 | 1.843 | 0.147 |
| -0.0039 | NA | NA | + | NA | NA | 2.35E-03 | 6 | 120.212 | -228.425 | 1.945 | 0.14 |
| 0.00468 | NA | NA | + | NA | NA | NA | 5 | 127.541 | -245.082 | 0 | 0.327 |
| 0.01739 | NA | NA | + | 7.98E-05 | NA | NA | 6 | 128.183 | -244.366 | 0.716 | 0.229 |
| 0.02702 | NA | NA | + | NA | -9.98E-03 | NA | 6 | 128.021 | -244.043 | 1.039 | 0.194 |
| 0.00261 | 1.25E-10 | NA | + | NA | NA | NA | 6 | 127.617 | -243.235 | 1.847 | 0.13 |
| 0.00465 | NA | NA | + | NA | NA | 1.31E-05 | 6 | 127.541 | -243.082 | 2 | 0.12 |
| 0.00132 | NA | NA | + | NA | NA | NA | 5 | 123.399 | -236.798 | 0 | 0.359 |
| 0.02167 | NA | NA | + | NA | -9.01E-03 | NA | 6 | 123.758 | -235.517 | 1.282 | 0.189 |
| 0.01068 | NA | NA | + | 5.69E-05 | NA | NA | 6 | 123.709 | -235.417 | 1.381 | 0.18 |
| -0.00032 | 9.28E-11 | NA | + | NA | NA | NA | 6 | 123.449 | -234.899 | 01.IX | 0.139 |
| -0.0018 | NA | NA | + | NA | NA | 1.32E-03 | 6 | 123.409 | -234.818 | 1.981 | 0.133 |
| 0.00488 | NA | NA | + | NA | NA | NA | 5 | 125.093 | -240.186 | 0 | 0.349 |
| 0.01731 | NA | NA | + | 7.25E-05 | NA | NA | 6 | 125.588 | -239.175 | 01.I | 0.211 |
| 0.02303 | NA | NA | + | NA | -8.05E-03 | NA | 6 | 125.389 | -238.778 | 1.408 | 0.173 |
| 0.00282 | 1.23E-10 | NA | + | NA | NA | NA | 6 | 125.175 | -238.351 | 1.835 | 0.139 |
| 0.00453 | NA | NA | + | NA | NA | 1.49E-04 | 6 | 125.093 | -238.186 | 2 | 0.128 |
| -0.00016 | NA | NA | + | NA | NA | NA | 5 | 130.442 | -250.883 | 0 | 0.333 |
| 0.0249 | NA | NA | + | NA | -1.13E-02 | NA | 6 | 131.098 | -250.196 | 0.688 | 0.236 |
| 0.00835 | NA | NA | + | 5.46E-05 | NA | NA | 6 | 130.778 | -249.556 | 1.328 | 0.171 |
| -0.0079 | NA | NA | + | NA | NA | 3.19E-03 | 6 | 130.505 | -249.01 | 1.873 | 0.13 |
| -0.00168 | 8.91E-11 | NA | + | NA | NA | NA | 6 | 130.5 | -249.001 | 1.883 | 0.13 |
| 0.00433 | NA | NA | + | NA | NA | NA | 5 | 124.118 | -238.235 | 0 | 0.377 |
| 0.01453 | NA | NA | + | 5.87E-05 | NA | NA | 6 | 124.411 | -236.823 | 1.412 | 0.186 |
| 0.01705 | NA | NA | + | NA | -5.62E-03 | NA | 6 | 124.239 | -236.479 | 1.756 | 0.156 |
| 0.00344 | 5.42E-11 | NA | + | NA | NA | NA | 6 | 124.138 | -236.276 | I.96 | 0.141 |
| 0.00732 | NA | NA | + | NA | NA | -1.26E-03 | 6 | 124.126 | -236.251 | 1.984 | 0.14 |
| 0.00028 | NA | NA | + | NA | NA | NA | 5 | 129.887 | -249.774 | 0 | 0.359 |
| 0.02099 | NA | NA | + | NA | -9.12E-03 | NA | 6 | 130.279 | -248.558 | 1.216 | 0.196 |
| 0.00794 | NA | NA | + | 4.80E-05 | NA | NA | 6 | 130.14 | -248.279 | 1.495 | 0.17 |
| -0.00092 | 6.94E-11 | NA | + | NA | NA | NA | 6 | 129.931 | -247.862 | 1.912 | 0.138 |
| -0.00534 | NA | NA | + | NA | NA | 2.43E-03 | 6 | 129.92 | -247.84 | 1.934 | 0.137 |
| 0.0017 | NA | NA | + | NA | NA | NA | 5 | 125.768 | -241.536 | 0 | 0.327 |
| 0.02967 | NA | NA | + | NA | -1.23E-02 | NA | 6 | 126.428 | -240.855 | 0.681 | 0.233 |
| 0.01327 | NA | NA | + | 6.79E-05 | NA | NA | 6 | 126.201 | -240.401 | 1.135 | 0.186 |
| 0.00035 | 8.24E-11 | NA | + | NA | NA | NA | 6 | 125.823 | -239.646 | I.89 | 0.127 |
| 0.00957 | NA | NA | + | NA | NA | -3.31E-03 | 6 | 125.82 | -239.64 | 1.896 | 0.127 |
| 0.00318 | NA | NA | + | NA | NA | NA | 5 | 129.079 | -248.158 | 0 | 0.367 |
| 0.02296 | NA | NA | + | NA | -8.96E-03 | NA | 6 | 129.444 | -246.888 | 1.271 | 0.194 |
| 0.01015 | NA | NA | + | 4.36E-05 | NA | NA | 6 | 129.276 | -246.552 | 1.606 | 0.164 |
| 0.00213 | 6.20E-11 | NA | + | NA | NA | NA | 6 | 129.109 | -246.219 | I.94 | 0.139 |
| 0.00066 | NA | NA | + | NA | NA | 1.05E-03 | 6 | 129.085 | -246.171 | 1.988 | 0.136 |
| 0.00335 | NA | NA | + | NA | NA | NA | 5 | 122.541 | -235.082 | 0 | 0.35 |
| 0.01671 | NA | NA | + | 7.81E-05 | NA | NA | 6 | 123.078 | -234.156 | 0.927 | 0.22 |
| 0.02321 | NA | NA | + | NA | -8.74E-03 | NA | 6 | 122.822 | -233.644 | 1.439 | 0.17 |
| 0.00277 | 3.50E-11 | NA | + | NA | NA | NA | 6 | 122.554 | -233.108 | 1.974 | 0.13 |
| 0.00696 | NA | NA | + | NA | NA | -1.51E-03 | 6 | 122.553 | -233.107 | 1.976 | 0.13 |
| 0.00419 | NA | NA | + | NA | NA | NA | 5 | 125.375 | -240.749 | 0 | 0.358 |
| 0.01568 | NA | NA | + | 6.61E-05 | NA | NA | 6 | 125.773 | -239.546 | 1.203 | 0.196 |
| 0.02268 | NA | NA | + | NA | -8.09E-03 | NA | 6 | 125.651 | -239.303 | 1.446 | 0.174 |
| -0.00176 | NA | NA | + | NA | NA | 2.52E-03 | 6 | 125.412 | -238.823 | 1.926 | 0.137 |
| 0.00292 | 6.93E-11 | NA | + | NA | NA | NA | 6 | 125.407 | -238.814 | 1.936 | 0.136 |
| 0.0031 | NA | NA | + | NA | NA | NA | 5 | 125.596 | -241.192 | 0 | 0.367 |
| 0.01281 | NA | NA | + | 5.59E-05 | NA | NA | 6 | 125.895 | -239.789 | 1.403 | 0.182 |
| 0.02017 | NA | NA | + | NA | -7.52E-03 | NA | 6 | 125.836 | -239.672 | I.52 | 0.171 |
| 0.00962 | NA | NA | + | NA | NA | -2.79E-03 | 6 | 125.635 | -239.27 | 1.922 | 0.14 |
| 0.00161 | 8.74E-11 | NA | + | NA | NA | NA | 6 | 125.633 | -239.265 | 1.927 | 0.14 |
| 0.00109 | NA | NA | + | NA | NA | NA | 5 | 124.182 | -238.364 | 0 | 0.319 |
| 0.02914 | NA | NA | + | NA | -1.27E-02 | NA | 6 | 125.11 | -238.221 | 0.143 | 0.297 |
| 0.00567 | NA | NA | + | 3.12E-05 | NA | NA | 6 | 124.294 | -236.588 | 1.775 | 0.131 |
| -0.00775 | NA | NA | + | NA | NA | 3.63E-03 | 6 | 124.257 | -236.515 | 1.849 | 0.127 |
| -0.00077 | 1.13E-10 | NA | + | NA | NA | NA | 6 | 124.254 | -236.508 | 1.856 | 0.126 |
| 0.00226 | NA | NA | + | NA | NA | NA | 5 | 123.008 | -236.015 | 0 | 0.358 |
| 0.01599 | NA | NA | + | 7.54E-05 | NA | NA | 6 | 123.465 | -234.93 | 1.086 | 0.208 |
| 0.01874 | NA | NA | + | NA | -7.12E-03 | NA | 6 | 123.175 | -234.35 | 1.665 | 0.156 |
| 0.01251 | NA | NA | + | NA | NA | -4.28E-03 | 6 | 123.099 | -234.198 | 1.817 | 0.144 |
| 0.00145 | 4.44E-11 | NA | + | NA | NA | NA | 6 | 123.026 | -234.051 | 1.964 | 0.134 |
| 0.00365 | NA | NA | + | NA | NA | NA | 5 | 125.331 | -240.662 | 0 | 0.366 |
| 0.01417 | NA | NA | + | 6.12E-05 | NA | NA | 6 | 125.69 | -239.38 | 1.282 | 0.193 |
| 0.01934 | NA | NA | + | NA | -7.00E-03 | NA | 6 | 125.543 | -239.087 | 1.575 | 0.166 |
| 0.00228 | 7.70E-11 | NA | + | NA | NA | NA | 6 | 125.363 | -238.726 | 1.936 | 0.139 |
| -0.00007 | NA | NA | + | NA | NA | 1.56E-03 | 6 | 125.344 | -238.689 | 1.974 | 0.136 |
| 0.00184 | NA | NA | + | NA | NA | NA | 5 | 128.624 | -247.248 | 0 | 0.352 |
| 0.0135 | NA | NA | + | 6.59E-05 | NA | NA | 6 | 129.046 | -246.092 | 1.156 | 0.198 |
| 0.02274 | NA | NA | + | NA | -9.22E-03 | NA | 6 | 128.996 | -245.991 | 1.257 | 0.188 |
| 0.00108 | 4.53E-11 | NA | + | NA | NA | NA | 6 | 128.646 | -245.292 | 1.956 | 0.133 |
| 0.00165 | NA | NA | + | NA | NA | 8.28E-05 | 6 | 128.624 | -245.248 | 2 | 0.13 |
| -0.00262 | NA | NA | + | NA | NA | NA | 5 | 122.626 | -235.253 | 0 | 0.364 |
| 0.00929 | NA | NA | + | 6.66E-05 | NA | NA | 6 | 123.019 | -234.038 | 1.215 | 0.198 |
| 0.0141 | NA | NA | + | NA | -7.36E-03 | NA | 6 | 122.833 | -233.667 | 1.586 | 0.165 |
| -0.00406 | 8.13E-11 | NA | + | NA | NA | NA | 6 | 122.668 | -233.336 | 1.916 | 0.14 |
| -0.00162 | NA | NA | + | NA | NA | -4.21E-04 | 6 | 122.627 | -233.255 | 1.998 | 0.134 |
| 0.00369 | NA | NA | + | NA | NA | NA | 5 | 118.523 | -227.046 | 0 | 0.343 |
| 0.01897 | NA | NA | + | 8.48E-05 | NA | NA | 6 | 119.114 | -226.228 | 0.819 | 0.228 |
| 0.02224 | NA | NA | + | NA | -8.28E-03 | NA | 6 | 118.78 | -225.561 | 1.486 | 0.163 |
| 0.01157 | NA | NA | + | NA | NA | -3.26E-03 | 6 | 118.576 | -225.152 | 1.894 | 0.133 |
| 0.00195 | 9.60E-11 | NA | + | NA | NA | NA | 6 | 118.571 | -225.143 | 1.904 | 0.132 |
| 0.03263 | NA | NA | + | NA | -1.33E-02 | NA | 6 | 128.351 | -244.701 | 0 | 0.28 |
| 0.00326 | NA | NA | + | NA | NA | NA | 5 | 127.08 | -244.16 | 0.541 | 0.214 |
| 0.0112 | NA | NA | + | NA | -1.26E-02 | 8.24E-03 | 7 | 128.789 | -243.579 | 1.122 | 0.16 |
| -0.01948 | NA | NA | + | NA | NA | 9.40E-03 | 6 | 127.646 | -243.292 | 1.409 | 0.139 |
| 0.03193 | 2.81E-11 | NA | + | NA | -1.32E-02 | NA | 7 | 128.361 | -242.721 | I.98 | 0.104 |
| 0.0329 | NA | NA | + | 3.50E-06 | -1.32E-02 | NA | 7 | 128.352 | -242.704 | 1.997 | 0.103 |
| 0.00079 | NA | NA | + | NA | NA | NA | 5 | 127.231 | -244.463 | 0 | 0.356 |
| 0.02214 | NA | NA | + | NA | -9.44E-03 | NA | 6 | 127.615 | -243.229 | 1.234 | 0.192 |
| 0.01046 | NA | NA | + | 5.80E-05 | NA | NA | 6 | 127.565 | -243.13 | 1.333 | 0.183 |
| -0.00065 | 8.51E-11 | NA | + | NA | NA | NA | 6 | 127.276 | -242.552 | 1.911 | 0.137 |
| 0.00145 | NA | NA | + | NA | NA | -2.78E-04 | 6 | 127.232 | -242.464 | 1.999 | 0.131 |
| 0.0002 | NA | NA | + | NA | NA | NA | 5 | 122.467 | -234.934 | 0 | 0.347 |
| 0.02635 | NA | NA | + | NA | -1.14E-02 | NA | 6 | 123.029 | -234.058 | 0.876 | 0.224 |
| 0.00895 | NA | NA | + | 5.20E-05 | NA | NA | 6 | 122.722 | -233.445 | I.49 | 0.165 |
| -0.00108 | 7.40E-11 | NA | + | NA | NA | NA | 6 | 122.513 | -233.027 | 1.908 | 0.134 |
| 0.00407 | NA | NA | + | NA | NA | -1.62E-03 | 6 | 122.48 | -232.961 | 1.973 | 0.13 |
| 0.00452 | NA | NA | + | NA | NA | NA | 5 | 120.478 | -230.956 | 0 | 0.308 |
| 0.01877 | NA | NA | + | 8.15E-05 | NA | NA | 6 | 121.007 | -230.014 | 0.942 | 0.192 |
| 0.01938 | NA | NA | + | NA | -6.58E-03 | NA | 6 | 120.625 | -229.25 | 1.707 | 0.131 |
| 0.04805 | NA | NA | NA | 1.53E-04 | NA | NA | 3 | 117.618 | -229.236 | 1.721 | 0.13 |
| 0.01332 | NA | NA | + | NA | NA | -3.66E-03 | 6 | 120.539 | -229.078 | 1.879 | 0.12 |
| 0.00307 | 9.01E-11 | NA | + | NA | NA | NA | 6 | 120.526 | -229.053 | 1.903 | 0.119 |
| 0.00251 | NA | NA | + | NA | NA | NA | 5 | 127.086 | -244.173 | 0 | 0.35 |
| 0.02495 | NA | NA | + | NA | -9.96E-03 | NA | 6 | 127.63 | -243.259 | 0.914 | 0.222 |
| 0.00907 | NA | NA | + | 3.91E-05 | NA | NA | 6 | 127.248 | -242.495 | 1.677 | 0.151 |
| -0.00669 | NA | NA | + | NA | NA | 3.96E-03 | 6 | 127.179 | -242.358 | 1.815 | 0.141 |
| 0.00092 | 9.05E-11 | NA | + | NA | NA | NA | 6 | 127.13 | -242.261 | 1.912 | 0.135 |
| 0.00322 | NA | NA | + | NA | NA | NA | 5 | 122.889 | -235.779 | 0 | 0.299 |
| 0.0181 | NA | NA | + | 7.73E-05 | NA | NA | 6 | 123.385 | -234.769 | 01.I | 0.18 |
| 0.02321 | NA | NA | + | NA | -8.68E-03 | NA | 6 | 123.155 | -234.31 | 1.468 | 0.143 |
| 0.04584 | NA | NA | NA | 1.38E-04 | NA | NA | 3 | 120.095 | -234.189 | 1.589 | 0.135 |
| 0.0156 | NA | NA | + | NA | NA | -5.34E-03 | 6 | 123.036 | -234.072 | 1.707 | 0.127 |
| 0.00151 | 9.16E-11 | NA | + | NA | NA | NA | 6 | 122.94 | -233.879 | 1.899 | 0.116 |
| 0.00087 | NA | NA | + | NA | NA | NA | 5 | 116.194 | -222.389 | 0 | 0.266 |
| 0.04652 | NA | NA | NA | 1.53E-04 | NA | NA | 3 | 113.859 | -221.718 | 0.671 | 0.19 |
| 0.01755 | NA | NA | + | 9.02E-05 | NA | NA | 6 | 116.836 | -221.672 | 0.716 | 0.186 |
| 0.02647 | NA | NA | + | NA | -1.12E-02 | NA | 6 | 116.629 | -221.259 | I.13 | 0.151 |
| -0.00104 | 1.05E-10 | NA | + | NA | NA | NA | 6 | 116.258 | -220.517 | 1.872 | 0.104 |
| 0.00936 | NA | NA | + | NA | NA | -3.53E-03 | 6 | 116.249 | -220.498 | 1.891 | 0.103 |
| 0.00313 | NA | NA | + | NA | NA | NA | 5 | 125.046 | -240.092 | 0 | 0.285 |
| 0.01806 | NA | NA | + | 8.17E-05 | NA | NA | 6 | 125.61 | -239.22 | 0.872 | 0.184 |
| 0.02604 | NA | NA | + | NA | -1.01E-02 | NA | 6 | 125.451 | -238.903 | 1.189 | 0.157 |
| 0.04629 | NA | NA | NA | 1.49E-04 | NA | NA | 3 | 122.411 | -238.822 | I.27 | 0.151 |
| 0.00117 | 1.15E-10 | NA | + | NA | NA | NA | 6 | 125.113 | -238.226 | 1.866 | 0.112 |
| 0.01083 | NA | NA | + | NA | NA | -3.25E-03 | 6 | 125.098 | -238.196 | 1.896 | 0.11 |
| 0.00414 | NA | NA | + | NA | NA | NA | 5 | 127.537 | -245.075 | 0 | 0.362 |
| 0.02292 | NA | NA | + | NA | -8.35E-03 | NA | 6 | 127.844 | -243.687 | 1.388 | 0.181 |
| 0.0131 | NA | NA | + | 5.32E-05 | NA | NA | 6 | 127.806 | -243.613 | 1.462 | 0.174 |
| -0.00513 | NA | NA | + | NA | NA | 3.85E-03 | 6 | 127.62 | -243.241 | 1.834 | 0.145 |
| 0.00278 | 7.94E-11 | NA | + | NA | NA | NA | 6 | 127.578 | -243.157 | 1.918 | 0.139 |
| 0.00392 | NA | NA | + | NA | NA | NA | 5 | 122.764 | -235.528 | 0 | 0.289 |
| 0.01983 | NA | NA | + | 9.19E-05 | NA | NA | 6 | 123.498 | -234.997 | 0.531 | 0.222 |
| 0.02527 | NA | NA | + | NA | -9.48E-03 | NA | 6 | 123.124 | -234.248 | I.28 | 0.153 |
| 0.00141 | 1.54E-10 | NA | + | NA | NA | NA | 6 | 122.872 | -233.744 | 1.784 | 0.119 |
| 0.0483 | NA | NA | NA | 1.61E-04 | NA | NA | 3 | 119.801 | -233.602 | 1.925 | 0.11 |
| 0.00692 | NA | NA | + | NA | NA | -1.24E-03 | 6 | 122.771 | -233.542 | 1.985 | 0.107 |
| 0.00357 | NA | NA | + | NA | NA | NA | 5 | 124.475 | -238.949 | 0 | 0.363 |
| 0.02411 | NA | NA | + | NA | -9.20E-03 | NA | 6 | 124.834 | -237.667 | 1.282 | 0.191 |
| 0.01278 | NA | NA | + | 5.24E-05 | NA | NA | 6 | 124.735 | -237.47 | 1.479 | 0.173 |
| 0.00219 | 8.39E-11 | NA | + | NA | NA | NA | 6 | 124.518 | -237.036 | 1.913 | 0.139 |
| 0.00502 | NA | NA | + | NA | NA | -6.10E-04 | 6 | 124.477 | -236.953 | 1.996 | 0.134 |
| 0.00223 | NA | NA | + | NA | NA | NA | 5 | 128.254 | -246.507 | 0 | 0.338 |
| 0.0164 | NA | NA | + | 8.51E-05 | NA | NA | 6 | 128.95 | -245.901 | 0.606 | 0.249 |
| 0.01912 | NA | NA | + | NA | -7.54E-03 | NA | 6 | 128.511 | -245.022 | 1.485 | 0.161 |
| 0.00129 | 5.70E-11 | NA | + | NA | NA | NA | 6 | 128.284 | -244.569 | 1.938 | 0.128 |
| 0.00094 | NA | NA | + | NA | NA | 5.33E-04 | 6 | 128.255 | -244.51 | 1.997 | 0.124 |
| 0.02875 | NA | NA | + | NA | -1.23E-02 | NA | 6 | 131.182 | -250.364 | 0 | 0.25 |
| 0.00113 | NA | NA | + | NA | NA | NA | 5 | 130.107 | -250.215 | 0.15 | 0.232 |
| 0.01331 | NA | NA | + | NA | -1.21E-02 | 6.31E-03 | 7 | 131.449 | -248.898 | 1.466 | 0.12 |
| -0.01496 | NA | NA | + | NA | NA | 6.82E-03 | 6 | 130.415 | -248.831 | 1.533 | 0.116 |
| 0.02691 | 7.29E-11 | NA | + | NA | -1.21E-02 | NA | 7 | 131.215 | -248.43 | 1.934 | 0.095 |
| -0.00096 | 1.23E-10 | NA | + | NA | NA | NA | 6 | 130.201 | -248.401 | 1.963 | 0.094 |
| 0.02903 | NA | NA | + | 3.59E-06 | -1.22E-02 | NA | 7 | 131.184 | -248.368 | 1.997 | 0.092 |
| 0.0035 | NA | NA | + | NA | NA | NA | 5 | 117.714 | -225.428 | 0 | 0.3 |
| 0.0178 | NA | NA | + | 7.85E-05 | NA | NA | 6 | 118.181 | -224.363 | 1.065 | 0.176 |
| 0.04873 | NA | NA | NA | 1.51E-04 | NA | NA | 3 | 115.111 | -224.222 | 1.206 | 0.164 |
| 0.01977 | NA | NA | + | NA | -7.11E-03 | NA | 6 | 117.905 | -223.81 | 1.618 | 0.134 |
| 0.00211 | 8.33E-11 | NA | + | NA | NA | NA | 6 | 117.749 | -223.498 | I.93 | 0.114 |
| 0.00756 | NA | NA | + | NA | NA | -1.72E-03 | 6 | 117.728 | -223.457 | 1.971 | 0.112 |
| 0.00207 | NA | NA | + | NA | NA | NA | 5 | 123.186 | -236.371 | 0 | 0.346 |
| 0.01583 | NA | NA | + | 7.85E-05 | NA | NA | 6 | 123.76 | -235.519 | 0.852 | 0.226 |
| 0.01792 | NA | NA | + | NA | -6.99E-03 | NA | 6 | 123.397 | -234.794 | 1.577 | 0.157 |
| -0.00019 | 1.30E-10 | NA | + | NA | NA | NA | 6 | 123.283 | -234.565 | 1.806 | 0.14 |
| -0.00287 | NA | NA | + | NA | NA | 2.05E-03 | 6 | 123.208 | -234.415 | 1.956 | 0.13 |
| 0.00206 | NA | NA | + | NA | NA | NA | 5 | 124.014 | -238.028 | 0 | 0.173 |
| 0.04663 | NA | NA | NA | 1.59E-04 | NA | NA | 3 | 122.009 | -238.019 | 0.009 | 0.172 |
| 0.02074 | NA | NA | + | 9.87E-05 | NA | NA | 6 | 124.789 | -237.579 | 0.449 | 0.138 |
| 0.02258 | NA | NA | + | NA | -9.02E-03 | NA | 6 | 124.297 | -236.594 | 1.434 | 0.084 |
| 0.06173 | NA | NA | NA | 1.47E-04 | -8.02E-03 | NA | 4 | 122.235 | -236.47 | 1.558 | 0.079 |
| 0.06387 | NA | NA | NA | 1.70E-04 | NA | -6.07E-03 | 4 | 122.17 | -236.339 | 1.688 | 0.074 |
| 0.01623 | NA | NA | + | NA | NA | -5.85E-03 | 6 | 124.169 | -236.339 | 1.689 | 0.074 |
| 0.04259 | NA | NA | + | 1.09E-04 | NA | -8.23E-03 | 7 | 125.092 | -236.183 | 1.844 | 0.069 |
| 0.00004 | 1.16E-10 | NA | + | NA | NA | NA | 6 | 124.087 | -236.174 | 1.853 | 0.068 |
| 0.04494 | 1.09E-10 | NA | NA | 1.60E-04 | NA | NA | 4 | 122.073 | -236.146 | 1.881 | 0.067 |
| 0.00761 | NA | NA | + | NA | NA | NA | 5 | 126.557 | -243.115 | 0 | 0.358 |
| 0.02701 | NA | NA | + | NA | -8.68E-03 | NA | 6 | 126.887 | -241.774 | 1.341 | 0.183 |
| 0.01694 | NA | NA | + | 5.71E-05 | NA | NA | 6 | 126.868 | -241.736 | 1.379 | 0.18 |
| 0.00575 | 1.11E-10 | NA | + | NA | NA | NA | 6 | 126.635 | -241.27 | 1.845 | 0.142 |
| 0.00057 | NA | NA | + | NA | NA | 2.90E-03 | 6 | 126.604 | -241.207 | 1.908 | 0.138 |
| 0.0032 | NA | NA | + | NA | NA | NA | 5 | 126.388 | -242.777 | 0 | 0.347 |
| 0.01518 | NA | NA | + | 6.65E-05 | NA | NA | 6 | 126.787 | -241.574 | 1.203 | 0.19 |
| 0.02467 | NA | NA | + | NA | -9.42E-03 | NA | 6 | 126.745 | -241.491 | 1.286 | 0.182 |
| 0.01501 | NA | NA | + | NA | NA | -4.96E-03 | 6 | 126.509 | -241.018 | 1.759 | 0.144 |
| 0.00116 | 1.22E-10 | NA | + | NA | NA | NA | 6 | 126.459 | -240.919 | 1.858 | 0.137 |
| -0.00138 | NA | NA | + | NA | NA | NA | 5 | 122.607 | -235.214 | 0 | 0.297 |
| 0.01478 | NA | NA | + | 8.44E-05 | NA | NA | 6 | 123.167 | -234.334 | 0.88 | 0.192 |
| 0.01999 | NA | NA | + | NA | -9.23E-03 | NA | 6 | 122.895 | -233.79 | 1.423 | 0.146 |
| 0.01255 | NA | NA | + | NA | NA | -5.85E-03 | 6 | 122.77 | -233.54 | 1.673 | 0.129 |
| 0.04683 | NA | NA | NA | 1.64E-04 | NA | NA | 3 | 119.691 | -233.383 | 1.831 | 0.119 |
| -0.00327 | 1.11E-10 | NA | + | NA | NA | NA | 6 | 122.677 | -233.354 | I.86 | 0.117 |
| 0.00565 | NA | NA | + | NA | NA | NA | 5 | 126.774 | -243.549 | 0 | 0.345 |
| 0.02911 | NA | NA | + | NA | -1.03E-02 | NA | 6 | 127.19 | -242.38 | 1.169 | 0.193 |
| 0.01759 | NA | NA | + | 6.84E-05 | NA | NA | 6 | 127.19 | -242.379 | 1.169 | 0.192 |
| 0.0159 | NA | NA | + | NA | NA | -4.38E-03 | 6 | 126.87 | -241.74 | 1.809 | 0.14 |
| 0.00479 | 4.95E-11 | NA | + | NA | NA | NA | 6 | 126.795 | -241.59 | 1.959 | 0.13 |
| 0.00468 | NA | NA | + | NA | NA | NA | 5 | 127.768 | -245.535 | 0 | 0.351 |
| 0.01748 | NA | NA | + | 7.52E-05 | NA | NA | 6 | 128.299 | -244.599 | 0.936 | 0.22 |
| 0.02118 | NA | NA | + | NA | -7.32E-03 | NA | 6 | 127.999 | -243.997 | 1.538 | 0.163 |
| 0.00284 | 1.09E-10 | NA | + | NA | NA | NA | 6 | 127.83 | -243.66 | 1.875 | 0.137 |
| 0.00287 | NA | NA | + | NA | NA | 7.60E-04 | 6 | 127.771 | -243.541 | 1.994 | 0.129 |
| 0.00228 | NA | NA | + | NA | NA | NA | 5 | 127.669 | -245.338 | 0 | 0.346 |
| 0.01446 | NA | NA | + | 7.24E-05 | NA | NA | 6 | 128.182 | -244.364 | 0.974 | 0.213 |
| 0.02172 | NA | NA | + | NA | -8.56E-03 | NA | 6 | 127.979 | -243.957 | 1.381 | 0.174 |
| 0.01094 | NA | NA | + | NA | NA | -3.67E-03 | 6 | 127.735 | -243.469 | 1.869 | 0.136 |
| 0.00121 | 6.67E-11 | NA | + | NA | NA | NA | 6 | 127.701 | -243.403 | 1.936 | 0.132 |
| 0.04682 | NA | NA | NA | 1.52E-04 | NA | NA | 3 | 120.579 | -235.158 | 0 | 0.265 |
| 0.00462 | NA | NA | + | NA | NA | NA | 5 | 122.506 | -235.013 | 0.145 | 0.246 |
| 0.02328 | NA | NA | + | 9.66E-05 | NA | NA | 6 | 123.241 | -234.483 | 0.675 | 0.189 |
| 0.05616 | NA | NA | NA | 1.58E-04 | NA | -3.30E-03 | 4 | 120.628 | -233.256 | 1.902 | 0.102 |
| 0.0459 | 5.57E-11 | NA | NA | 1.52E-04 | NA | NA | 4 | 120.596 | -233.193 | 1.965 | 0.099 |
| 0.05094 | NA | NA | NA | 1.50E-04 | -2.15E-03 | NA | 4 | 120.594 | -233.188 | 1.969 | 0.099 |
| 0.00453 | NA | NA | + | NA | NA | NA | 5 | 125.504 | -241.008 | 0 | 0.302 |
| 0.04688 | NA | NA | NA | 1.36E-04 | NA | NA | 3 | 122.863 | -239.727 | 1.282 | 0.159 |
| 0.01717 | NA | NA | + | 6.58E-05 | NA | NA | 6 | 125.853 | -239.706 | 1.303 | 0.157 |
| 0.02229 | NA | NA | + | NA | -7.79E-03 | NA | 6 | 125.71 | -239.419 | 1.589 | 0.136 |
| 0.01867 | NA | NA | + | NA | NA | -5.98E-03 | 6 | 125.681 | -239.362 | 1.647 | 0.132 |
| 0.00344 | 6.19E-11 | NA | + | NA | NA | NA | 6 | 125.523 | -239.046 | 1.962 | 0.113 |
| 0.00122 | NA | NA | + | NA | NA | NA | 5 | 118.347 | -226.693 | 0 | 0.283 |
| 0.01949 | NA | NA | + | 9.93E-05 | NA | NA | 6 | 119.138 | -226.277 | 0.416 | 0.23 |
| 0.048 | NA | NA | NA | 1.64E-04 | NA | NA | 3 | 115.734 | -225.468 | 1.225 | 0.153 |
[truncated: 59,691 more chars]
